# Molecular evolutionary patterns of NAD^+^/Sirtuin aging signaling pathway across taxa

**DOI:** 10.1371/journal.pone.0182306

**Published:** 2017-08-02

**Authors:** Uma Gaur, Jianbo Tu, Diyan Li, Yue Gao, Ting Lian, Boyuan Sun, Deying Yang, Xiaolan Fan, Mingyao Yang

**Affiliations:** Institute of Animal Genetics and Breeding, Sichuan Agricultural University, Chengdu, Sichuan, P. R. China; University of South Alabama Mitchell Cancer Institute, UNITED STATES

## Abstract

A deeper understanding of the conserved molecular mechanisms in different taxa have been made possible only because of the evolutionary conservation of crucial signaling pathways. In the present study, we explored the molecular evolutionary pattern of selection signatures in 51 species for 10 genes which are important components of NAD^+^/Sirtuin pathway and have already been directly linked to lifespan extension in worms and mice. Selection pressure analysis using PAML program revealed that *MRPS5* and *PPARGC1A* were under significant constraints because of their functional significance. *FOXO3a* also displayed strong purifying selection. All three sirtuins, which were *SIRT1*, *SIRT2* and *SIRT6*, displayed a great degree of conservation between taxa, which is consistent with the previous report. A significant evolutionary constraint is seen on the anti-oxidant gene, *SOD3*. As expected, *TP53* gene was under significant selection pressure in mammals, owing to its major role in tumor progression. Poly-ADP-ribose polymerase (*PARP*) genes displayed the most sites under positive selection. Further 3D structural analysis of *PARP1* and *PARP2* protein revealed that some of these positively selected sites caused a change in the electrostatic potential of the protein structure, which may allow a change in its interaction with other proteins and molecules ultimately leading to difference in the function. Although the functional significance of the positively selected sites could not be established in the variants databases, yet it will be interesting to see if these sites actually affect the function of *PARP1* and *PARP2*.

## Introduction

The comparative sequence analysis has been used to examine the relationship between nucleotide sequences of different species and has produced several evidences that support the hypothesis of common descent [1]. Plenty of examples prove that biochemical and molecular mechanisms are conserved across different taxa. The same biochemical processes operate in all known living organisms, such as the flow of genetic information, which is DNA to RNA to protein takes place through highly conserved ribosomes. In developmental biology, the common morphology is the result of the sharing of same genetic elements [2].

Many workers have reported that the molecular and biochemical mechanisms behind longevity assurance pathways are conserved in vertebrates and also in invertebrates up to certain extent [3–7]. Out of all the molecular pathways responsible for aging, insulin-like growth factor/insulin signaling (IIS) and target of rapamycin (TOR) pathways are the most conserved nutrient signaling pathways which alter growth, reproduction and metabolism in response to energy status, stress and nutrient availability. Genetic alterations that reduces signaling via these signaling networks have been shown to extend lifespan in yeast, worms, flies and mice, revealing a key evolutionarily conserved role in aging [8–10]. There have been an extensive molecular evolutionary analysis of IIS/TOR aging network which has uncovered substantial variation in IIS/TOR network within and among amniotes and provided a critical step to unlock information on vertebrate patterns of genetic regulation of metabolism, modes of reproduction and rates of aging [11].

Recently an interesting candidate longevity pathway which is NAD^+^/Sirtuin is gaining major attention because of its interplay with FOXO and mitochondrial UPR signaling [12]. NAD^+^ pathway have been shown to be present in a vast array of species and the vertebrate NAD metabolism is constantly undergoing functional diversification [13, 14]. The idea of NAD^+^ mediating the lifespan and health span extension by dietary restriction (DR) was put forward by Lin et al., (2004) [15]. Furthermore, Imai and group [16, 17] did a thorough research on the role of NAD^+^ and sirtuins in aging and diseases and documented that the defect in NAD^+^ level and consequently declined activity of sirtuins may accelerate the normal aging process. Also, they found that the NAD^+^ deficiency related pathologies can be normalized by supplementing with NAD^+^ precursors and intermediates. The role of NAD^+^ and sirtuins in aging and diseases have been reviewed in detail by Imai and Guarente [18] and they have highlighted the regulatory mechanisms by which NAD^+^ and its consuming enzymes sirtuins and PARPs regulate aging and disease states. It is well documented that NAD^+^ regulates metabolism and circadian rhythms through sirtuins and also NAD^+^ gets depleted during aging and diseases affecting the activity of sirtuins.

The NAD^+^/Sirtuin pathway improves life quality during aging and disease states by regulating metabolism, stress resistance, cell survival and proliferation, transcription, apoptosis, and autophagy. Sirtuin enzymes are coming up as competent therapeutic targets in cancer, diabetes, neurodegenerative diseases and inflammatory disorders. Alteration of sirtuin activity has been shown to affect the process of several aggregate-forming neurodegenerative disorders by changing the transcription factor activity and deacetylating the proteotoxic species [19].

Some reports have explicitly documented the regulation of longevity and metabolism through NAD^+^/sirtuin pathway in mice and worm [20, 12]. Modulation of NAD^+^ levels have a powerful metabolic impact because it serves as an obligatory substrate for the deacetylase activity of the sirtuin proteins [21–23]. The best-characterized mammalian sirtuin is *SIRT1*, which controls mitochondrial function through the deacetylation of targets that include PGC-1a and FOXO [24,7]. The administration of NAD^+^ precursors, such as nicotinamide mononucleotide [25] or nicotinamide riboside (NR) [26], has proven to be an efficient method to increase NAD^+^ levels and SIRT1 activity, improving metabolic homeostasis in mice. Poly(ADP-ribosyl)ation is a post-translational modification of proteins which regulates the various cellular pathways. This modification is reported to be majorly employed in eukaryotes and poly(ADP-ribose) polymerase proteins (*PARPs*) are present in major eukaryotic supergroups, with exception of few eukaryotic species which lack PARP genes [27]. Furthermore, the NAD^+^-consuming *PARPs*, with *PARP1* and *PARP2* contributing to the main PARP activities in mammals, were classically described as DNA repair proteins [28, 29], but recent studies have linked these proteins to metabolism [30–33]. Indeed, genetic or pharmacological inactivation of *PARP1* increased tissue NAD^+^ levels and activated mitochondrial metabolism [32]. An association between *PARPs* and lifespan has been postulated [34, 35], but a causal role remained unclear.

Though there have been few reports about the evolutionary analysis of genes involved in NAD^+^ biosynthesis and metabolism, yet the molecular evolutionary analysis of NAD^+^/Sirtuin pathway genes in the context of aging is not well explored. Therefore, the present study was designed to study the evolutionary forces acting on the NAD^+^/Sirtuin pathway regulated aging signaling. The present analysis is the first report which has highlighted the positive selection pressure acting upon all the above mentioned components of NAD^+^ regulated aging signaling pathway at one place. This will be useful in understanding how evolutionary forces have made these genetic components fit in to the bigger picture of aging.

## Materials and methods

### Sequence data collection

To begin with, a set of 10 genes which are documented to play important roles in NAD^+^/Sirtuin signaling pathway is created, which is as follows: *PARP1*(Poly (ADP-ribose) polymerase), *PARP2*, *SIRT1*, *SIRT2*, *SIRT6*, *MRPS5* (mitochondrial ribosomal protein 5), *FOXO3a*, *TP53*, *PPARGC1A* (peroxisome proliferator activated receptor PPARy co-activator 1α), and *SOD3* (Super Oxide Dismutase 3). The CDS sequences were retrieved by extensive searches of NCBI (http://www.ncbi.nlm.nih.gov/) databases and Ensembl genome browser (http://www.ensembl.org/index.html).

In order to test whether the evolutionary trend in the phylogeny was affected by taxonomic sampling used, we sorted the taxonomic samplings into 5 data sets including a total of 51 species, which are model organisms, mammals, birds, reptiles, amphibians and fishes. The following species were used for analysis: model organisms- *Drosophila melanogaster* (Fruitfly), *Caenorhabditis elegans* (Worm), *Saccharomyces cerevisiae* (Yeast), *Mus musculus* (Mouse), *Danio rerio* (Zebra fish); Mammals (15 species)- *Homo sapiens* (Human), *Macaca mulatta* (Macaque), *Pongo abelii* (Orangutan), *Heterocephalus glaber* (Naked mole rat), *Oryctolagus cuniculus* (Rabbit), *Ailuropoda melanoleuca* (Panda), *Canis familiaris* (Dog), *Equus caballus* (Horse), *Bos taurus* (Cow), *Sus scrofa* (Pig), *Tursiops truncatus* (Dolphin), *Loxodonta africana* (Elephant), *Dasypus novemcinctus* (Armadillo), *Monodelphis domestica* (Opossum) and *Ornithorhynchus anatinus* (Platypus); Birds (10 species)- *Gallus gallus* (Chicken), *Meleagris gallopavo* (Turkey), *Anas platyrhynchos* (Duck), *Taeniopygia guttata* (Zebra finch), *Columba livia* (Rock pigeon), *Ficedula albicollis* (Flycatcher), *Parus major* (Great tit), *Struthio camelus* (African ostrich), *Pygoscelis adeliae* (Adelie penguin), *Sturnus vulgaris* (Common starling); Reptiles (10 species)- *Anolis carolinensis* (Anole Lizard), *Pogona vitticeps* (Central bearded dragon), *Gekko japonicas* (Gecko), *Protobothrops mucrosquamatus* (Taiwan habu), *Chrysemys picta bellii* (Western painted turtle), *Pelodiscus sinensis* (Chinese softshell turtle), *Chelonia mydas* (Green sea turtle) *Alligator mississippiensis* (American alligator), *Alligator sinensis* (Chinese alligator), *Gavialis gangeticus* (Gharial); Amphibians (3 species)- *Xenopus tropicalis* (Tropical clawed frog), *Xenopus laevis* (African clawed frog), *Nanorana parkeri* (Tibetan frog); Fishes (8 species)- *Gadus morhua* (Cod), *Oreochromis niloticus* (Tilapia), *Oryzias latipes* (Medaka), *Takifugu rubripes* (Fugu), *Tetraodon nigroviridis* (Tetraodon), *Xiphophorus maculatus* (Southern platyfish), *Lepisosteus oculatus* (Spotted gar), *Poecilia reticulate* (guppy).

### Sequence alignment and editing

The phylogenetic analysis was carried out to infer the relationship among homologous genes, by creating the multiple sequence alignment for CDS sequence of each gene in different species using ClustalX tool of BioEdit (ver. 7.2.5) [36] with appropriate manual adjustments. Some genes did not have homologues in all species, so the CDS sequences were retrieved for all the possible species in which the homologues were available. The species topology was generated by the TimeTree web server (http://www.timetree.org/).

### Selection pressure analyses

The selective pressures acting on the NAD^+^/Sirtuin pathway coding genes were calculated by CODEML program in PAML package version 4.7, using a phylogenetic-based Maximum Likelihood (ML) analysis [37]. The ML estimates of the branch lengths and the ratio of the nonsynonymous (dN) to synonymous substitution rates (dS), ω = dN/dS, were obtained using the codeml program. The ω parameter was used as a measure of the protein selective constraints [38]. These analyses were conducted under different competing evolutionary hypothesis. Alignment was visualized with GeneDoc software [39].

Branch-site models were used to detect positive selection sites among different species lineages. The branch #1 is mammal branch, #2-#5 are leading to birds (#2), birds and reptiles (#3), amphibians (#4) and fishes (#5) were marked as the foreground lineage to analyze whether positive selection occurred along these branches. Only foreground lineages may have experienced positive selection. The model assumes four classes of sites. Site class 0 includes codons that are conserved throughout the tree, with 0 < ω_0_ < 1 estimated. Site class 1 includes codons that are evolving neutrally throughout the tree with ω_1_ = 1. Site classes 2a and 2b include codons that are conserved or neutral on the background branches, but came under positive selection on the foreground branches with ω_2_ > 1, estimated from the data. Likelihood ratio tests were carried out to compare branch site null model and branch site model. The bayes empirical bayes (BEB) approach was used to calculate the posterior probabilities that each site belongs to the site class of positive selection on the foreground lineages with a significance threshold of *p*>95%. Bonferroni correction was used to correct for multiple testing [40].

### Protein structural analysis

The protein structural analysis for two genes PARP1 and PARP2 was further carried out, since PARPs are important candidate genes in NAD^+^/Sirtuin pathway. We predicted the folding pattern using SWISS-MODEL web server (http://swissmodel.expasy.org) [41]. Positive selection sites identified in the branch-site model were marked within the three dimensional structure map of PARP1 and PARP2. Electrostatic potential were calculated using the PBEQ-Solver web server (http://www.charmm-gui.org/?doc=input/pbeqsolver). All figures were displayed and generated with PyMOL software (DeLano Scientific; http://pymol.org).

## Results

### Gene homologues present in different clades and species topology

**Fig 1:**
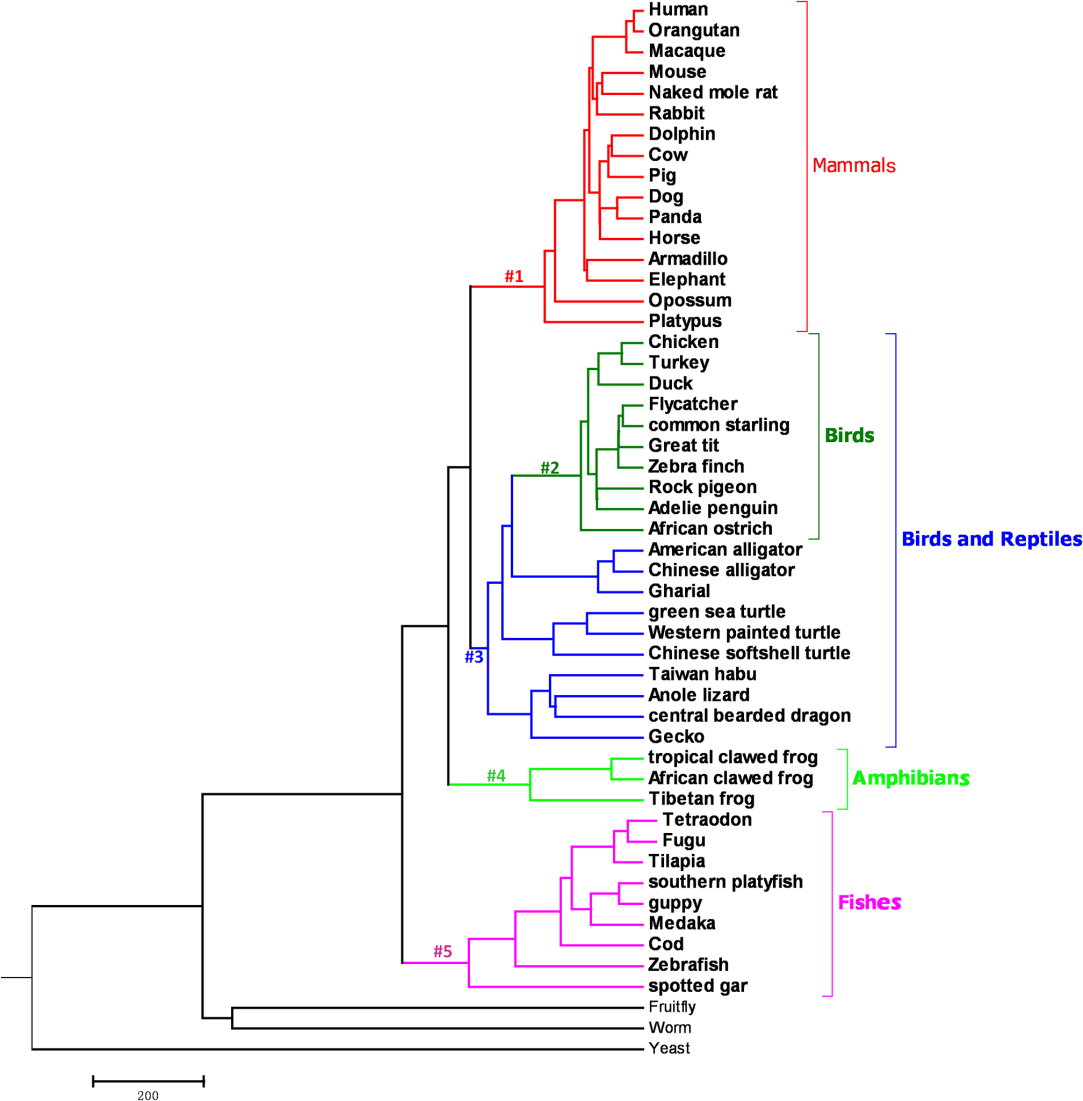
A rooted cladogram showing the phylogenetic relationships among the species included in present study. The branch leading to the mammal (#1), birds (#2), birds and reptiles (#3), amphibians (#4) and fishes (#5), were marked as the foreground lineage to analyze selection sites.

The gene homologues for ten NAD^+^/Sirtuin pathway genes were searched against NCBI and Ensembl databases in different species (Table 1). Sequences and accession numbers of 10 genes in different species were shown in S1 and S2 Tables. Gene *PARP2* was completely absent in birds. The species topology for all the 51 species was constructed and shown in Fig 1.

**Table 1:** Gene homologues information for 10 genes in different species.

Taxon	Species		*PARP1*	*PARP2*	*SIRT1*	*SIRT2*	*SIRT6*	*MRPS5*	*FOXO3a*	*TP53*	*PPARGC1A*	*SOD3*
Model species	Fruitfly	*Drosophila melanogaster*	√	×	√	√	√	√	√	√	√	√
	Worm	*Caenorhabditis elegans*	√	√	×	√	×	√	√	×	×	√
	Yeast	*Saccharomyces cerevisiae*	×	×	×	√	×	√	×	×	×	×
	Mouse	*Mus musculus*	√	√	√	√	√	√	√	√	√	√
	Zebrafish	*Danio rerio*	√	√	√	√	√	√	√	√	√	√
Mammal	Human	*Homo sapiens*	√	√	√	√	√	√	√	√	√	√
	Macaque	*Macaca mulatta*	√	√	√	√	√	√	√	√	√	√
	Orangutan	*Pongo abelii*	√	√	√	√	√	√	√	√	√	√
	Naked mole rat	*Heterocephalus glaber*	√	√	√	√	√	√	√	√	√	√
	Rabbit	*Oryctolagus cuniculus*	√	√	√	√	×	√	√	√	√	√
	Panda	*Ailuropoda melanoleuca*	√	√	√	√	√	√	√	√	√	√
	Dog	*Canis lupus familiaris*	√	√	√	√	√	√	√	√	√	√
	Horse	*Equus caballus*	√	√	√	√	√	√	√	√	√	√
	Cow	*Bos taurus*	√	√	√	√	√	√	√	√	√	√
	Pig	*Sus scrofa*	√	√	√	√	√	√	√	√	√	√
	Dolphin	*Tursiops truncatus*	√	√	√	√	√	√	√	√	√	√
	Elephant	*Loxodonta africana*	√	√	√	√	√	√	√	√	√	√
	Armadillo	*Dasypus novemcinctus*	√	√	√	√	√	√	√	√	√	√
	Opossum	*Monodelphis domestica*	√	√	√	√	√	√	√	√	√	√
	Platypus	*Ornithorhynchus anatinus*	√	√	√	×	√	√	√	√	√	√
Aves	Chicken	*Gallus gallus*	√	×	√	√	√	√	√	√	√	√
	Turkey	*Meleagris gallopavo*	√	×	√	×	√	√	√	×	√	√
	Duck	*Anas platyrhynchos*	√	×	√	×	√	√	√	×	√	√
	Zebra finch	*Taeniopygia guttata*	√	×	√	×	√	√	√	×	√	√
	Rock pigeon	*Columba livia*	√	×	√	×	√	√	√	×	√	√
	Flycatcher	*Ficedula albicollis*	√	×	√	×	√	√	√	×	√	√
	Great tit	*Parus major*	√	×	√	√	√	√	√	×	√	√
	African ostrich	*Struthio camelus*	√	×	√	×	√	√	√	×	√	√
	Adelie penguin	*Pygoscelis adeliae*	√	×	√	×	√	√	√	×	√	√
	Common starling	*Sturnus vulgaris*	√	×	√	√	√	√	√	√	√	√
Reptile	Anole lizard	*Anolis carolinensis*	√	√	√	√	√	√	√	√	√	√
	Central bearded dragon	*Pogona vitticeps*	√	√	√	√	√	√	√	√	√	√
	Gecko	*Gekko japonicus*	√	√	√	√	√	√	√	√	√	√
	Taiwan habu	*Protobothrops mucrosquamatus*	√	√	√	√	√	√	√	×	√	√
	Western painted turtle	*Chrysemys picta bellii*	√	√	√	√	√	√	√	√	√	√
	Chinese softshell turtle	*Pelodiscus sinensis*	√	×	√	√	√	√	√	√	√	√
	Green sea turtle	*Chelonia mydas*	√	√	√	√	√	√	√	×	√	√
	American alligator	*Alligator mississippiensis*	√	√	√	√	√	√	√	√	√	√
	Chinese alligator	*Alligator sinensis*	√	√	√	√	√	√	√	√	√	√
	Gharial	*Gavialis gangeticus*	√	√	√	×	√	√	√	×	√	√
Amphibian	Tropical clawed frog	*Xenopus tropicalis*	√	√	√	√	√	√	√	√	√	√
	African clawed frog	*Xenopus laevis*	√	√	√	√	√	×	√	√	√	×
	Tibetan frog	*Nanorana parkeri*	√	√	√	√	√	√	√	√	√	×
Fish	Cod	*Gadus morhua*	√	√	√	√	√	√	√	√	√	×
	Tilapia	*Oreochromis niloticus*	√	√	√	√	√	√	√	√	√	√
	Medaka	*Oryzias latipes*	√	√	√	√	√	√	√	√	√	√
	Fugu	*Takifugu rubripes*	√	√	√	√	√	√	√	√	√	√
	Tetraodon	*Tetraodon nigroviridis*	√	√	√	√	√	√	√	√	√	√
	Southern platyfish	*Xiphophorus maculatus*	√	√	√	√	√	√	√	√	√	√
	Spotted gar	*Lepisosteus oculatus*	√	√	√	√	×	√	√	√	√	√
	Guppy	*Poecilia reticulata*	√	√	√	√	×	√	√	√	√	√

### Selection pressure analysis for NAD^+^/Sirtuin pathway genes

Selection pressure on each branch was calculated using branch-site model in PAML package (S3 Table). The posterior probabilities of each site belonging to the site class of the positive selection on the foreground lineages were calculated using BEB approach. The sites under positive selection for each gene are shown in Table 2.

**Table 2:** Positively selected sites under branch-site model for foreground lineages Prob(w>1).

Gene	Lineages				
	Mammals	Birds	Birds/Reptiles	Amphibian	Fish
*MRPS5*	-	-	-	-	-
*FOXO3a*	-	-	-	435 F 0.983*	442 S 0.983*
				436 G 0.987*	591 S 0.956*
				437 P 0.979*	
				463 S 0.984*	
				655 N 0.982*	
*PARP1*	-	-	-	146 P 0.991**	72 D 0.966*
				563 V 0.967*	269 K 0.995**
				1011 T 0.987*	293 L 0.978*
				1012 S 0.997**	392 N 0.970*
					606 S 0.965*
					807 D 0.965*
					830 D 0.996**
*PARP2*	-	-	-(Reptiles)	69 S 0.967*	241 M 0.963*
					319 Q 0.977*
*PPARGC1A*	-	-	-	-	-
*SIRT1*	-	-	-	-	-
*SIRT2*	-	-	216 E 0.997**	-	162 L 0.994**
			246 C 0.976*		249 S 0.958*
					253 K 0.960*
*SOD3*	-	-	-	-	197 E 0.979*
*TP53*	-	-	-	-	101 K 0.969*
*SIRT6*	-	-	-	-	-

Lineages corresponded to branches depicted in Fig 1; “-” indicates no positively selected sites were detected in that branch;

*: *P* < 0.05;

**: *P* < 0.001.

Numbers of positively selected sites were amino acid residues at the position of each gene in Human.

The likelihood ratio tests of positive selection based on ML method of [33], was applied. The values of likelihood ratio tests of positive selection operating on the genes in present study have been provided as S4 Table. The Bonferroni correction for multiple testing was applied following LRT tests. The correction *p* value used 0.001 as the significance level (S4 Table). *PARP2* gene is missing in birds that’s why the lineage has been defined exclusively for reptiles in the Table 2 for the birds/reptile lineage.

### Protein structural analysis

**Fig 2:**
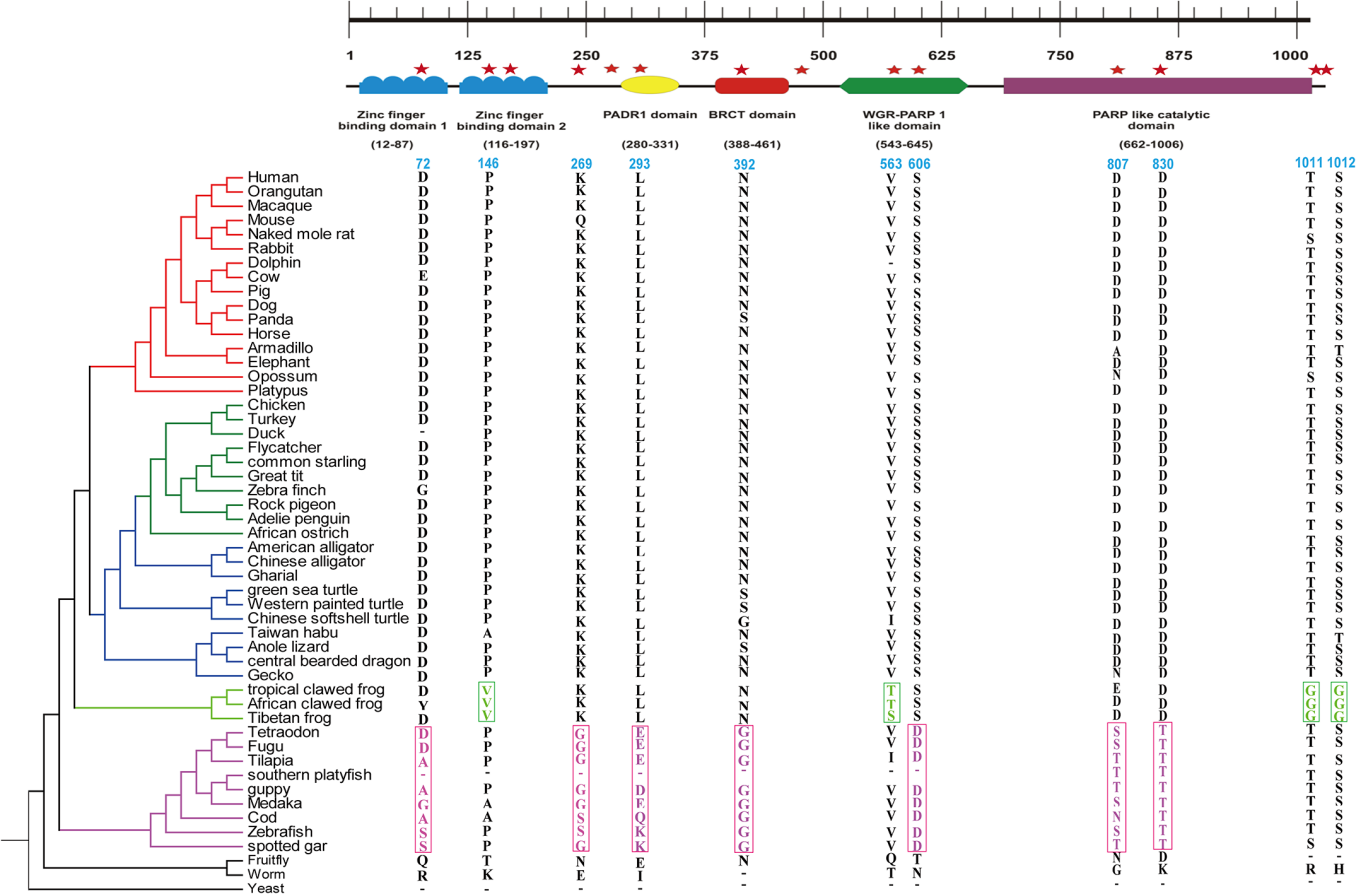
Functional conserved domains of poly ADP- ribosomal protein 1. Red star represents positively selected sites in different branches. Highlighted nucleotide alignments represent the positively selected amino acid. Numbers corresponded to amino acid residues at the position of Human PARP1 gene.

The functional protein of *PARP1* consists of five conserved domain: one PAPR-like domain, one WGR *PARP1*-like domain, two zf-PARP domains, one PADR1 domain and one BRCT1 domain (Fig 2). Poly(ADP-ribose) polymerase (parp)-like catalytic domain catalyzes the covalent attachment of ADP-ribose units from NAD^+^ to itself and to a limited number of other DNA binding proteins, which decreases their affinity for DNA. For gene *PARP1*, Two sites (P146 and V563) were found under positive selection in Amphibian. Seven positively selected sites were found in fish branch. Six potentially functional sites (D72, L293, N392, S606, D807 and D830) were found to be present in five conserved domains in fishes (Fig 2).

**Fig 3:**
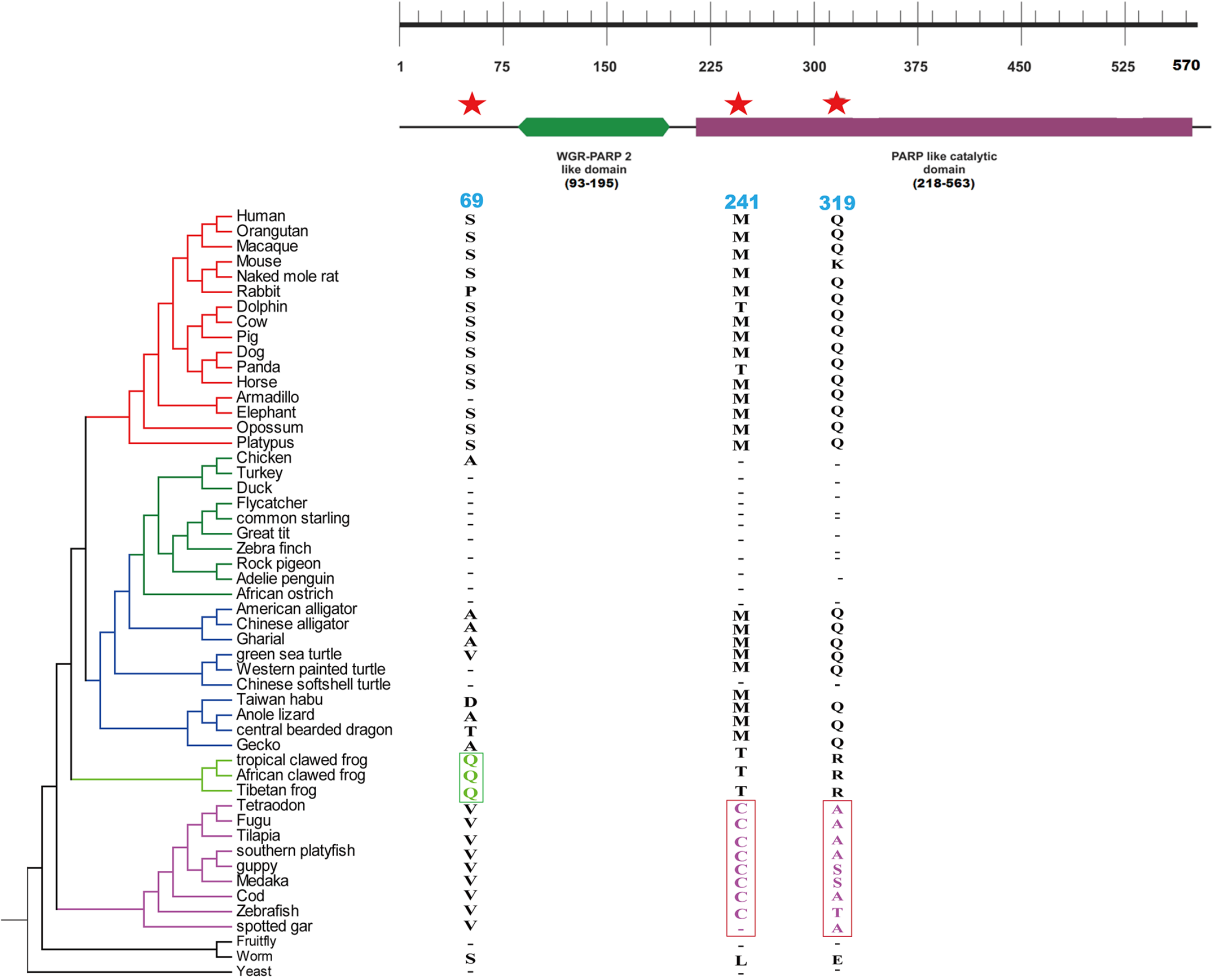
Functional conserved domains of poly ADP- ribosomal protein 2. Red star represents positively selected sites in different branches. Highlighted nucleotide alignments represent the positively selected amino acid. Numbers corresponded to amino acid residues at the position of Human *PARP2* gene.

*PARP2* gene has two functional domains which are WGR-PARP2-like domain and PARP-like catalytic domain (Fig 3). For the gene *PARP2*, two sites (M241 and Q319) in PARP-like catalytic domain were found to be positively selected in fishes (Fig 3).

**Fig 4:**
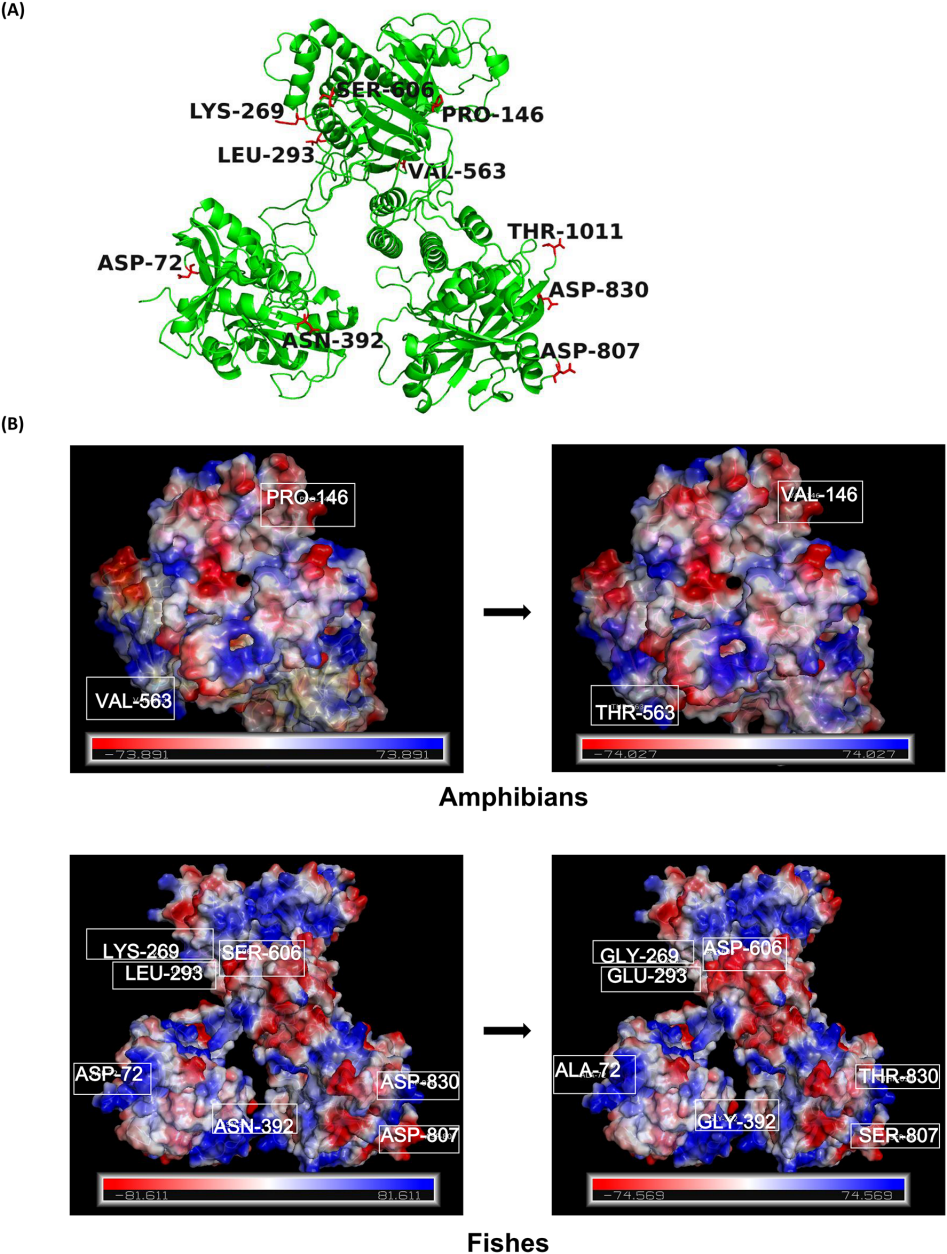
3D structural analysis for poly ADP- ribosomal protein 1. (A). Eleven positively selected sites in PARP1. (B). The folding pattern were predicted using SWISS-MODEL and electrostatic potential were calculated using the PBEQ-Solver web server. The figures were generated in PyMOL software. Numbers corresponded to positively selected amino acid sites at the position of Human *PARP1* gene.

*PARP* genes, which have been predicted to have the most sites under positive selection in the present study, were further used to infer structural changes in the 3D structure of the protein. For gene *PARP1*, 11 positively selected sites, which are D72, P146, K269, L293, N392, V563, S606, D807, D830, T1011, S1012 were present in different lineages, with fishes having the most sites under positive selection (Table 2, Fig 4A). Some of the positively selected sites caused a change in electrostatic potential of the protein structure in mammals, birds, reptiles and fishes (Fig 4B).

**Fig 5:**
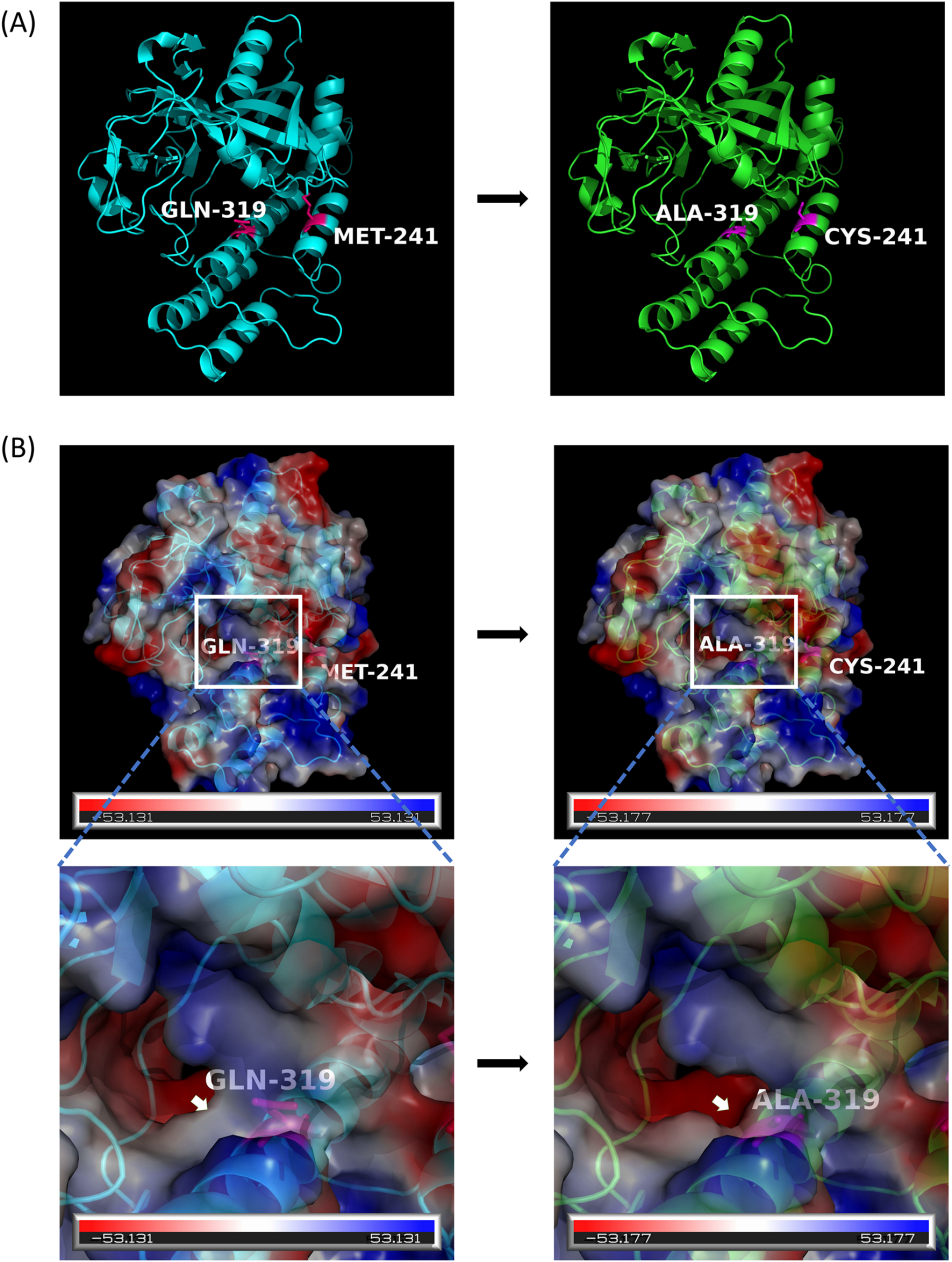
3D structural analysis for poly ADP- ribosomal protein 2. (A). Two positively selected sites in fishes for *PARP2*. (B). The folding pattern were predicted using SWISS-MODEL and electrostatic potential were calculated using the PBEQ-Solver web server. The figures were generated in PyMOL software. Numbers corresponded to positively selected amino acid sites at the position of Human *PARP2* gene.

For the gene *PARP2*, three positively selected sites which are S69, M241, Q319 are seen and two positively selected sites M241 and Q319 in fishes caused a change in the electrostatic potential when mapped on to the 3D protein structure of *PARP2* in fishes (Fig 5).

## Discussion

The present study analyzed the molecular evolution of ten genes involved in NAD^+^/Sirtuin pathway in 51 species that represented all of the major animal eukaryotic taxa. Most of the components of NAD^+^/Sirtuin pathway were present in all the taxa except *PARP2*, which was absent from birds. *SIRT2* and *TP53* were also under-represented in this clade. This is particularly noteworthy since birds represent an independent evolutionary origin [11]. In this present study, the branch leading to the mammals, birds, birds and reptiles, amphibians and fishes were designed as the foreground branches in branch-site models to examine positive selection. We found that *PARP1* and *PARP2* had the most sites under positive selection. These positively selected sites in *PARP1* and *PARP2* are worthy of further investigation.

NAD^+^/Sirtuin pathway lies at the intersection of important aging pathways, such as FOXO and mitochondrial UPR, which makes it difficult to understand its independent role [12]. However, a couple of reports have clearly established NAD^+^/Sirtuin pathway as an important aging regulator [20,42,43,44]. It is very important to understand a genetic mechanism from evolutionary point of view, to gain a clear picture of the natural selection acting on each component of the involved pathways. There have been an exhaustive molecular evolutionary analysis of IIS/TOR aging network which uncovered substantial variation in IIS/TOR network within and among amniotes and provided a critical step to unlock information on vertebrate patterns of genetic regulation of metabolism, modes of reproduction and rates of aging [11]. Also, the evolutionary relationships of sirtuins have been searched for all seven sirtuins present in 77 representative species of animals, plants, bacteria and archaea [45]. Molecular evolutionary patterns of Insulin/FOXO signaling pathway have also been explored in metazoan genomes. It has been found that most of the enzymes associated with vertebrate NAD metabolism are subjected to different levels of purifying selection [14]. No significant evidence of positive selection was found for *MRPS5*, *PPARGC1A*, *SIRT1* and *SIRT6* in any of the clades, indicating significant constraints on these genes, which was probably because of the basic requirement for their function in all clades. In contrast, a series of positively selected sites in *PARPs*, pointed out the regulatory role of DNA repair mechanisms. *FOXO3a* displayed no positive selection among mammals, birds and reptiles, yet multiple positively selected sites were present in amphibians and fishes, which is consistent with the previous reports where most members of *FOXO* gene family are found to be under strong purifying selection [46].

All three sirtuins (*SIRT1*, *SIRT2* and *SIRT6)*, displayed great degree of conservation between taxa, which is consistent with a previous comprehensive evolutionary analysis [45]. A significant evolutionary constraint is seen on the anti-oxidant gene, *SOD3*, which is a member of the superoxide dismutase protein family. *SODs* are antioxidant enzymes that catalyze the dismutation of two superoxide radicals into hydrogen peroxide and oxygen. The product of this gene is thought to protect the brain, lungs, and other tissues from oxidative stress. A previous report has also revealed great deal of evolutionary conservation among the members of *SOD* gene family [47], which is pertinent to the functional significance of this gene family. *TP53*, which is a transcription factor involved in maintaining genomic integrity by regulating genes involved in cell cycle arrest, DNA repair, and programmed cell death, has got a celebrity status in mammals, owing to its function in promoting adaptive responses to stress [48]. The branches representing bird, reptile and amphibian, and mammals did not display any sign of positive selection, whereas one site was positively selected in fishes. The reason for the greater conservation of this gene could be its importance in maintaining the cell cycle, DNA repair and cell death.

What attracted our particular attention was the *PARPs* gene family, which has already been suggested to be evolving under strong recurrent selection pressure [49] and displayed multiple positively selected amino acid sites in the functional protein domains in the present study. *PARPs* are the NAD^+^ consuming poly ADP-ribose polymerase proteins which exerts a direct powerful metabolic impact by altering the NAD^+^ levels, since it competes with sirtuins proteins which also uses NAD^+^ as a mandatory substrate for deacetylase activity [12]. *PARP1* is the most abundant of all the parp’s and contributes to neuronal survival and death under different stress conditions [50]. Ex-vivo supplementation of human peripheral blood mononuclear cells with NAD^+^ precursor nicotinic acid resulted in enhanced levels of cellular NAD^+^ levels which boosted the cellular poly(ADP-ribosyl)ation response to genotoxic treatment, and protected from DNA-damage-induced cell death [51]. Inhibition of PARP has resulted in protection against mitochondrial dysfunction and neurodegeneration in Parkinson fruit fly model [52]. Recent reports suggest that *PARP1* and *PARP2* stand at the crossroad of metabolic stress and inflammation and play important role in longevity and aging [31,32,43,53,54].

In present study, the branch leading to fish displayed greater adaptive evolutionary pressure acting on *PARP1*, since this had the most sites under positive selection (Table 2). In contrast, *PARP1* seems to be more conserved in bird, reptile, amphibian, and mammalian branches. As the basic function of *PARP1* is the maintenance of nucleolus structure and function, it comes as no surprise that this gene has been under greater constraints during evolution [55]. The positively selected sites in *PARP1* caused a change in the electrostatic potential of the protein (Fig 4).

Interestingly, *PARP2* gene also displayed the similar evolutionary trend where the branch leading to fishes had two positively selected sites which is further supported by the fact that the evolution of fish proteins is faster than mammalian orthologs [56]. Furthermore, a change in electrostatic potential in 3D protein structure for *PARP2* protein was found for positively selected sites M241 and Q319. Whether these amino acid changes had some adaptive advantage need further experimental evidences. The change in electrostatic potential of *PARP1* and *PARP2* may allow a change in their interaction with other proteins and molecules, which might ultimately lead to difference in the function.

Looking at the importance of *PARP1* and *PARP2* genes in hematopoiesis, embryonic development, spermatogenesis, T-cell maturation and adipogenesis in mammals, it becomes evident that evolutionary forces have maintained the greater constraints on these genes for all the good reasons [57,58].

## Conclusion

This is the first comprehensive analysis of the molecular evolution of 10 NAD^+^/Sirtuin pathway related genes in mammals, birds, reptiles, amphibians and fishes. All of these genes have been under strong constraints in mammals. *FOXO3a*, *PARP1*, *PARP2*, *SIRT2*, *TP53*, and *SOD3* genes had been under stronger evolutionary pressures in fishes. However, birds and reptiles have not been under much evolutionary selection forces for these genes, owing to their own independent evolutionary origin. *PARP1* and *PARP2* had the most sites under positive selection. Looking at the 3D structure of these proteins in different taxa, a clear change in electrostatic potential is observed which could be one of the ways by which the protein interaction with other proteins and molecules might have evolved. The current evolutionary analysis poses *PARPs* as an important component of the NAD+/Sirtuin pathway, which should be explored further experimentally.

## Supporting information

S1 Table Nucleotide sequences of ten NAD+/Sirtuin pathway genes in different species in this study. (PDF)

S2 Table List of accession numbers of sequences used for 10 genes in 51 species. (PDF)

S3 Table Lineages test for positive selection by branch-site model using PAML. (PDF)

S4 Table Likelihood ratio tests for positive selection using branch-site models. (PDF)
