# Supplementary material for: Molecular evolutionary patterns of NAD+/Sirtuin aging signaling pathway across taxa
Source: PLoS One. 2017 Aug 2;12(8):e0182306. doi: 10.1371/journal.pone.0182306 (PMC5540417; doi:10.1371/journal.pone.0182306)
Supplement: S1 Table — (PDF) [file pone.0182306.s001.pdf]

>platypus_PARP1
ATGTTCGATGGGAAGGTCCCACACTGGCACCATTTTACTTGTTTCTGGAAGCGGGCACGG
GTCATCTCCCATGCCGAGGTGGATGGTTTCCCCGAATTGCGGTGGGATGACCAGGAGAAG
ATCAAGAAAGCCATAGAGACTGGAGGAGCCGCCACAGGTAAAGGTGGAGACCAGGATGGC
GGAGGAGGCAAGGGGGAGAAGACCCTGAATGACTTTGCAGCTGAATATGCCAAGTCTAAC
AGGAGCACGTGCAAGGGATGTGACCAGAAAATCGAAAAGGGCCAGGTACGGCTCTCCAAG
AAGATGGTGGATCCAGTGAAGCCACAGTTGGGGATGATTGACCGCTGGTACCATCCGGAC
TGCTTCGTCAGCCGCCGAGTTGAGCTTGGCTTTCTCCCGCAGTATAACGCCAGCCAGCTC
AAAGGGTTTGGTATCCTGAAGCCAGAGGACAAGGAAACCCTGAAGAAGCAGCTCCCTGCC
GTCAAGAGCGAAGGGAAGAGGAAAGGAGATGAGGTGGATGGAGGTGGCGTGGTGGCCAAG
AAGAAACAGAAAAAGGAAAAAGACAAGGAAACCAAGCAGGAGAAGCTGCTCAAGGAGCAG
ACAGAGTTGATCTGGAACATCAAAGATGAGCTGAAGAAAGCCTGCTCCACGAACGACTTG
AAAGAGCTGCTTATAGCCAACAAGCAAGCCGTGCCTTCTGGGGAGACGGCGATTTTGGAT
AGAGTGGCCGATGGAATGGCCTTTGGAGCACTTCTTCCCTGCGAGGAGTGCAAAGGGCAG
TTCGTCTTCAAGAGCGATGCCTACTATTGCTCCGGGGACATCACGGCCTGGACCAAATGT
GTGGCCAAGACCCAGACGCCCAACAGGAAGGAATGGACCATCCCGAAGGAGTTCAAGGAA
ATCCCGTACCTAAAGAAATTTAAGTGCAAAAAGCAGGACCGAGCCTTCACCCCAGAGGCC
AGCACCCCTGTGATCGTGCCTTTGCCCTCAACGACCCCTGCTCCCCCCACGCTGAACTCG
ACTGTGCCGGCAGATAAGCCGTTGTCCAACATGAAGATACTGACTCTTGGGAAGCTTTCC
AGAAACAAAGAAGAGATGAAGGCCACGATTGAGGAGCTCGGGGGGAAATTGACGGGTTCC
GCCAACAAGGCCTCCCTGTGCATCAGTACTCAAAAGGAAGTGGAGAAGATGAACAAGAAA
ATGGAGGAGGTGAAGGAGGCTGGAGTGCGGGTGGTTTCTGAGGAGTTCCTCAAGGATGTT
GCCGCCTCGGGCAAGAGGCTCCAGGAGCTGCTGACCCTGCACGCCTTGTCACCCTGGGGG
GCTGAGGTGAAGCAAGAGCACCCCGAGGTGCCACTGAGCGGGAAGTCAGGCGGCCCTGTC
TCCACCAAGAGTGCGGGCAAGATCAAGGAGGAGGAAGGAGCCAGCAAGTCCGAAAAGAAG
ATGAAGTTAACCGTAAAAGGAGGCGCAGCTGTCGATCCGGATTCTGGCTTGGAGGACTCG
GCTCACGTCTTTGAAAAGGGCGGGAAAATCTTCAGCGCCACCCTCGGCCTGGTGGACATC
GTCCGAGGAACCAATTCATATTATAAACTGCAGCTCCTGGAGGATGACCGAGAAATCAGC
AGGTACTGGGTGTTTAGATCCTGGGGCCGCGTGGGCACTGAGATTGGCAGTAACAAGCTG
GAGCAGATGCCATCCAAAGAAGATGCCATTGAACACTTTCTGAACTTGTATGAAGAGAAA
ACCGGCAACTCCTGGCATTCCAAGAACTTTACTAAATATCCCAAAAAATTCTATCCTCTA
GAAATCGATTATGGGCAGGATGAGGAGGCAGTGAAGAAACTGACTGTCAGCGCCGGCACC
AAGTCCAAGCTCCCTAAGCCCGTTCAGAACCTCATCAAGATGATCTTTGACGTGGAGAGC
ATGAAGAAGGCCATGGTGGAATTCGAGATTGACCTCCAGAAGATGCCACTGGGAAAGCTA
AGTAAGAGGCAGATCCAGAACGCTTACTCTATCCTGAACGAGGTCCAGCAGGCCGTGTCT
GAGGGAGGCAGCGACTCTCAGATCCTGGACCTGTCCAACCGCTTCTACACACTGATCCCC
CACGACTTTGGGATGAAAAAGCCTCCGCTTCTGAACAACATGGACTGTGTGCAGGCCAAG
GTAGAGATGTTGGACAACCTGCTGGACATCGAGGTGGCCTATAGCCTGCTCCGGGGTGGC
TCTGAAGATGGTGACAAGGACCCCATCGATGTCAACTATGAGAAGCTCAAAACGGACATC
AAGGTGGTTGACAAAGACTCAGAAGAAGCCAGAATCATTAGGGAGTACGTCAAGAACACT
CATGCCACAACACACAATGCTTATGACCTGCAAGTTGTTGATATCTTCAAAATTGAGCGG
GAAGGAGAGAGTCAGCGCTATAAGCCATTCAAGCAGCTTCACAACCGGCAGCTGCTCTGG
CACGGTTCCCGGGCTACAAACTTTGCTGGTATCCTGTCCCAAGGCCTGCGAATCGCCCCC
CCTGAGGCTCCCGTGACCGGCTACATGTTTGGAAAAGGCATCTACTTCGCGGACATGGTC

TCCAAGAGTGCCAACTACTGCCACACATCTCAGGGAGATCCTATCGGCTTGATCCTGCTG
GGAGAAGTGGCCCTCGGAAACATGTATGAGCTGAAGAACGCATCCCATATCAGCAAGCTG
CCCAAAGGGAAGCACAGCGTCAAAGGTTTGGGCAAAACTGCCCCTGATCCCAGTGCCAGC
ATCACCCTCGATGGGGTGGAGGTTCCCCTGGGGACTGGGATTTCGTCGGGGGTCAGCGAC
ACTTGCCTACTGTATAACGAATACATCGTCTATGATATTGCTCAGGTAAATCTGAAGTAT
CTGCTGAAACTGAAGTTCAACTTTAAGACGTCGCTGTGG
>rabbit_PARP1
ATGGCGGAGTCTTCGGACAAGCTCTACCGAGTGGAATACGCCAAGAGCGGGCGCGCCTCC
TGCAAGAAATGCAGCGAGAGCATCCCCAAGGACTCGCTCCGGATGGCCATCATGGTGCAG
TCGCCCATGTTCGATGGGAAGGTCCCACACTGGTACCACTTCTCCTGCTTCTGGAAGGTG
GGCCACTCCATCCGGCAGCCCGACAGCGAGGTGGATGGCTTCTCTGAGCTGCGGTGGGAT
GACCAGCAGAAAGTCAAGAAGACGGCGGAGGCCGGCGGGGTGACTGGCAAAGGCCAGGAT
GGAACCGGCAGCAAAACAGAGAAGACGCTGGGTGACTTTGCAGCCGAGTACGCCAAGTCC
AACAGAAGCACGTGCAAGGGGTGCATGGAGAAGATAGAAAAGGGTCAGGTGCGCCTTTCC
AAGAAGATGCTGGACCCAGAGAAGCCACAGCTGGGCATGATCGACCGCTGGTACCACCCG
AACTGCTTTGTCAAGAACAGGGAGGAGCTGGGTTTCCGGCCCGAGTACAGTGCAAGTCAG
CTCAAGGGCTTCAGCCTTCTCTCTGCAGAGGATAAAGAGGCCCTGAAGAAGCAGCTCCCG
GGGGTCAAGAGTGAAGGAAAAAGGAAAGGGGATGAGGTGGATGGAATGGATGAAGTGGCC
AAGAAAAAATCTAAAAAAGAAAAAGACAAGGAGAGTAAGCTTGAAAAGGCCCTCAAGGCC
CAGAACGACCTGATCTGGAACATCAAGGATGAACTAAAGAAAGTATGTTCGACCAATGAC
CTGAAAGAGCTGCTCATCTTCAACAAGCAGCAAGTGCCTTCCGGGGAGTCGGCGATCTTG
GACCGGGTAGCTGATGGCATGGTGTTCGGTGCCCTCCTTCCCTGCGAGGAGTGCTCGGGC
CAGCTGGTCTTCAAGAGCGATGCTTACTACTGTACCGGGGACGTCACTGCCTGGACCAAG
TGTATGGTCAAGACACAGACACCCAACCGGAAGGAATGGGTGACCCCAAAGGAATTCCGG
GAAATCTCTTACCTCAAGAAATTGAAGATCAAAAAGCAGGACCGCATATTCCCCCCAGAG
AGCAGCGCCCCGGCAGCAGCGGCACCCCCTCCCTCCACAGCCTCGGCCCCCGCTTCTGTG
ACTCCCTCCGCTCCAGCAGATAAGCCACTGTCCAACATGAAGATCCTGACCCTTGGGAAA
CTGTCCCGGAACAAGGACGAAGTGAAGGCCGCCGTCGAGAAACTCGGGGGGAAGGTGACG
GGGACAGCCAGCAAGGCCTCGCTGTGCATCAGCACCCAGAAGGAAGTGGAAAAGATGAAT
AAGAAAATGGAGGAAGTCAGAGAAGCCAACGTCCGCGTCGTGGCTGAGGACTTCCTCCAG
GATGTCTCCAGCTCCGCCAAGAGCCTGCAGGAGTTGCTCTCAGCGCACATCTTATCCCCC
TGGGGGGCCGAGGTGAAGGCAGAGCCTGTTGAAGCAGTGGCCCCAAAACCAAAGTCAGGG
GCTGCTGTCGCCAAGAAGAGCAAGGGACCAGTGGAGGAAGTCCAAGGTATGAACAAATCT
GAAAAGAGGATGAAATTAACTCTCAAAGGAGGTGCAGCCGTCGATCCTGATTCTGGTCTG
GAGCACTCGGCACACGTCCTGGAGAAAGGCGGGAAGGTGTTCAGCGCCACCCTTGGCCTG
GTGGACATCGTTAAGGGAACCAACTCCTATTACAAGCTGCAGCTGCTCGAGGATGACAAG
GAAAGCAGGTACTGGATATTCAGGTCATGGGGTCGCGTGGGAACCGTGATTGGTAGTAAC
AAACTGGAGCAGATGCCATCTAAGGAGGATGCCATTGAGCACTTTATGAAATTATATGAA
GAAAAGACTGGGAACGCCTGGCACTCCAAAAACTTCACAAAGTATCCCAAAAAGTTCTAT
CCTCTGGAAGACTATGGCCAGGATGAAGAGGCGGTGAAGAAGCTGACGGTGAGCCCTGGC
ACCAAGTCGAAGCTCCCCAAGCCCGTTCAGGAACTTATTAAGATGATCTTTGACGTGGAA
AGTATGAAGAAGGCCATGGTGGAGTATGAGATTGACCTTCAGAAGATGCCTCTGGGGAAG
CTGAGCAAAAGGCAGATCCAGGCCGCGTACTCTATTCTCAACGAGGTCCAGCAGGCGGTG
TCCCAGGGCAGCAGTGAATCTCAGATCCTGGACCTCTCAAACCGCTTCTACACCCTGATC

CCCCACGACTTCGGGATGAAGAAGCCCCCGCTCCTGAACAACACAGACAGCGTGCAGGCC
AAGGTGGAAATGCTGGACAACCTCCTGGACATTGAGGTGGCCTACAGTCTGCTCAGGGGT
GGATCTGATGATAGCAGCAAGGACCCCATCGATGTCAACTATGAGAAGCTCAAAACTGAC
ATTAAGGTGGTTGACAAAGATTCCGAAGAAGCTGAGGTCATCAGGAAATACGTTAAGAAC
ACACATGCGACCACGCACAATGCATATGACTTGGAAGTTGTTGATATCTTTAAGATAGAG
CGTGACGGAGAAAGCCAACGCTATAAGCCCTTCAAGCAGCTTCATAACCGACGGTTGCTG
TGGCATGGGTCCAGGACCACCAACTTTGCTGGGATCCTGTCCCAGGGGCTGCGGATAGCC
CCACCTGAAGCGCCTGTGACGGGCTACATGTTTGGTAAAGGGATCTATTTCGCCGACATG
GTCTCCAAGAGTGCCAACTACTGCCACACGTCTCAGGGAGACCCCATCGGTTTGATCCTG
TTGGGAGAAGTTGCCCTTGGAAACATGTATGAACTGAAGCAAGCTTCGCATATCAGCAAG
TTACCCAAGGGCAAGCACAGTGTGAAAGGTTTGGGCAAAACCACTCCTGACCCTTCTGCT
AGTATTACTCTGGATGGTGTGGAGGTTCCTCTGGGGACCGGTATCTCGTCTGGTGTTAAC
GACACCTGTCTGCTGTATAACGAGTACATTGTCTACGATATTGCTCAGGTAAATCTGAAG
TATCTGCTGAAACTGAAGTTTAACTTCAAGACATCGCTGTGG
>tilapia_PARP1
ATGGCAGACTCACAAGAGGACAAGCTGTACAGGGTGGAATACGCCAAAAGTGGCCGCGCG
TCGTGCAAAAAAATGCAAAGAAAACATAGCTAAAGACTCGCTGAGGATGGCTATCATGGTG
CAGTCTCCCATGTTTGATGGGAAGGTCCCCCACTGGCACCACTTCTCCTGCTTCTGGCAG
CGAGCATCAGCTCAGTCCACCGCTGATATCGCTGGGTTTTCTGATCTCCGCTGGGATGAC
CAACAGAAGGTGAAAAAGGCCATTGAAAGCGGTGGCGCCGCAGGAGGGAAAGGTGACCAG
AAGGGTGGCGCTAAAGGCGAGAAGACGCTGAATGACTTTGCTGTGGAATATGCCAAGTCA
AACCGCAGCACATGCAAAGGCTGTGAGCAGAAAATAGAAAAGGATCAGATTCGTGTGTCC
AAGAAAACTGTGGACCCCGAGAAGCCACAGCTGGGTCTTATTGACCGCTGGTACCACACG
GCGTGTTTCGTGAGCCGCAGGGAGGAGCTGGTTTTCAAGCCCGAGTACAGCGCCGCTCAG
CTGAAGGGATTCAATGCACTGCGGGCAGAGGACAAAGAAGAGCTCAAGAAGAGGCTCCCT
GCTGTCAAATCTGAGGGGAAGCGGAAAACTGATGATGTTGATGGAGCGTCAAAGAAACAG
AAGACAGAAGAAGACGACAAGAAGAAAAAGCTAGAAGAACAGTTAAAGAATCAAAGTCAG
CTTATTTGGGGAATTAAGGACAAGCTGCGAAAATATTGTTCAGCCAATGATATGAAGGAA
CTGCTGATTGCAAATGGCCAGGAAGTTCCCTCTGGAGAGACCAATCTGGTTGACAGCCTG
GCCGATGGTATGGCCTTTGGTGCTCTCGAGGCCTGTAAGGAGTGCCAGGGCCAGCTGGTG
TTTAAGGGTGACGCCTATTACTGCACAGGAAACATTTCTGCCTGGACAAAGTGTGTGTTC
AAAACCACAACACCCCTACGCAAAGACTGGGTCATTCCCAAGGAATTCCACGAAGTTTCT
TTTCTGAAAAAATTCAAATTCAAGCGGCAGGACAGGGTTTACCCCAAAGAGGCTCCCACG
AAAACCCTGGCAGCGGCCAAATCAGAACCACTACCGAGTGCATCCAGCGCTCCGACCGAG
ACCCTGCCACAGGGAGCACCTTCAGACAAGCCTCTTACTGGCATGAAGCTGCTGACTGTG
GGCAAGCTGACTAAGAACAAGGATGATCTGAAAACTGCTGTGGAAGAGCTTGGTGGAAAG
ATTACCGGCACAGTCAACAAGGCCTCTTTGTGTATAAGCACCAAGAAGGAGGTGGAGAAG
ATGAGTAAGAAGATGGAGGAAGTAAGGGACGCTGGCGTGCGTGTGGTCTCTGAGGATTTC
CTCACTGACATCAAGTCATCGGGCAAAGCCCTCCAGGAGCTGGTCTCCCTGCACGCCATC
TCCCCCTGGGGGGCAGAGGTTAAAGTCGAAGCTCAGGCTCCATCTGTGGCCTCCAAGTCT
GGAACACAGGCTACTAAGAGCACAGGGAGGGTGAAAGAGGAGGAAGGTGGTAGCAAATCC
AAGAAGATGAAACTCACAGTTAAAGGTGGAGCTGCTGTGGATCCAGACTCAGGGCTGGAG
AACAGTGCCCATGTCTTGGAGCAGAATGGGAAGATGTACAGTGCTACACTCGGTCTTGTG
GATATTATCAGAGGAACTAACTCTTACTATAAACTGCAGCTGCTTGAGGATGATGTACAG

AAACGGTACTGGGTGTTCAGGTCATGGGGCAGAGTGGGTACCACTATCGGAGGAAACAAG
TTGGACAAATTCCATGACAAGAACTCTGCTGTGGATAATTTCCTGGGTGTGTACAAGGAG
AAGACTGGCAATGATTGGGGCTCGTCCAACTTCACCAAATATCCCAATAAGTTCTACCCG
CTGGAGATTGACTACGGACAGGATGAGGAGGCAGTGAAGAGGCTGACAGCCACGGCTGGC
ACCAAGTCAAAGCTGGCCAAACCTGTCCAGGAGCTAATCAAGATGATCTTTGATGTAGAA
AGCATGAAGAAGGCCATGGTCGAGTTTGAGATTGACCTCCAGAAGATGCCACTTGGTAAG
CTGAGCAAGAGGCAAATCCAGAGTGCCTATGCTCTCCTCACTGAAGTACAGCAGGCTGTG
TCAGAGTGTGTGTCTGAGGCGCAGATACTGGATCTCTCCAATCGCTTTTATACTCTAATA
CCTCATGACTTTGGCATGAAGAAGCCTCCACTGCTCAACAACCTGGACTACATTCAAGCT
AAAGTTCAGATGTTGGACAACCTGTTGGATATTGAAGTGGCTTACAGCTTACTGAGAGGA
GGAGCCCAGGACAATGAGAAAGACCCCATCGACATAAACTATGAGAACCTCAAGACAAAG
ATTGAGGTTGTTGACAAGACCACAAGTGAAGCTGAGATCATCTTGCAATACGTTAAGAAC
ACCCACGCTGCTACACACAACACGTACACACTGGAAGTACAAGAGATATTTAAAATTGTC
AGAGAGGGAGAGCACCAGCGATATCGTCCATTCGAGGAGCTTCACAATCGCCAGCTATTG
TGGCACGGCTCTCGCACCACCAACTACGCCGGTATTCTGTCTCAGGGTCTCCGCATTGCC
CCTCCAGAGGCCCCAGTGACTGGTTACATGTTTGGCAAAGGTGTGTACTTTGCTGACATG
GTGTCCAAGAGTGCAAACTACTGCCACACCTCCCAGTCAGATCCTGTAGGCCTCATTCTG
CTCGGCGAGGTTGCTCTAGGAAACATGCATGAACTGAAGAAGGCTTCTCATATTACAAAA
TTACCTAAGGGCAAGCACAGTGTTAAAGGTTTGGGTAGAACTGCTCCCGATCCAAATGCC
ACTGTCACTTTAAATGGAGTGCAAGTGCCTCTGGGAAAAGGAGTCCACACTAATATTGAT
GACACAAGTCTGCTGTACAACGAGTACATCGTGTATGATGTAGCACAGGTAAATCTGAAG
TATCTTCTGAAGATCAAGTTTAACTACCAGACATCCCTGTGG
>worm_PARP1
ATGATTCATTCCAACGAGCCACTGCCCTACGCTATCGAATATGCAAAGTCTGGCCGGTCG
AATTGTAAAACATGCAAAAAGAATATCGCGTTGGATCAATTGAGAATGTCAATGAATCGC
CCGTCAACATTCTTCGATGGAAACATGGATTCGTGGTTTCACTACAATTGCTTCTGGATA
AAAATGATTCGCGGCCGAGATGACATCAATATAAGCTCAATCCGAGGAGTCGATTGGCTT
CGCTGGGAAGATCAGGAAAAGCTTCGCCAGGAAATTCAACACTTTAAAACCGCATCGCCA
CCGACTCTGACACCCCTGTGCTCTACCACTACCGTCATTTTGTCCACAATAAAAACCGAA
AAATCGTTGTCAAATCGTGGGAAATGTGGAAAATGCGGCCAAAATTTCGAGCGCGGCGAA
ATCAAGGCTCATAACAAGGGAAAAGCCAACCATTTCAAGTGTTTTCTGCAGGAATTTGAT
AAAATTTCCGGCACCGTTGAGGATATTCCTGGCTGGGCGGATTATGAGGAGAACTTTAAA
ATTAAGGCGGTTGGGGAGTATGTGGAGGCTTTAGCTGCGAAAAGGCGATCCACGGAGCCA
GCTACCCCGGCTTCTGCCTCTCCAACACCACCAGAAGCTGAAACTCCAGTTTTATCTGCA
GAAGGATCCCCGGAAAGCTCCAATAAACGTCCGGCAAGCTCTGAAATCATTGAAATCGAC
GGTGAAGGGAATCCAGATGAGAATGATTTTGCGAAGAAAAGACGAATGAAGAAGGAAGCA
AGATTGATGGAGGTTCAGAAGAAGCGAATGAAGAAACAATCCGATCTTCTCTGGGAATAC
CGCCAGATCTTCGAAAGAATGCCGTACACCGACAAAATCTCTATATTACGAGAAAACGAG
CAAGACATTCCAGAAGGACACGATCCGACTGCACAAGTAATCGAACGCCTAGTGGATAAT
GCCCTATTCGGATGCCCAATCATTTGCCAAACATGTTCAAATGGAAAAATTGTCTATAAT
TCATCATGCCGCACATATGTCTGCACTGGATATGCTACAGAATACTCGAAATGTACTTAT
GAATCAAAGAATCCAATTCGAACTCCATTCGAAGTTTCTCACCGATTAACTGAAAAGCAT
AAGCTTCAAGATATTGTATTCAATCAAATGAGTGAACGACTTTATATAGGAGAAGAGGAT
GGAGAATCTGTAGTGAAAATTGATAAAAGAAAGTCGAAAGGTGGTACTCGTGGTGAACAA

TTCATTTATGCGGCTGAAGCATTCGATTCGACTAACAATGTTCCGATAAAAGTCGGCGAT
CTCACATCAACCAATACTCATATTATTAAAAAAGGAACAGTTGTTGATGCGAAATTCGCA
CTGGCCGATCGTTGTCATGTATTCAAAAATGAGATTGATGGAAGTCTCTATCAGGCGACA
CTCTCGTTTACTGATCTTACACAGAATAAGAATTCGTATTATAAGATTCAACTGTTGAAG
GATGATCAGAGAGAAAACTACTACGTCTTCCGTTCTTGGGGTCGAGTTGGCACAGAAGTC
GGCGGAAATAAGCATGAATCATACAGTAATTCAAATGAAGCGATTCTCAAATTCCAGGAT
GTTTTCCACGAAAAAACGAAGAACGATTGGATTTATAGAAAACATTTCCGCAAAATGCCC
GGAATGTTCAGCTACGTGGAGACGGATTACTCGGAATTTGCACAAATAACGGACACAGAA
ATCACTCCAGGATCAAAAACTCTACTTCCAAAATCTGTAAAAGAAGTTGTAATGTCAATT
TTCGACGTTGAAAACATGAAATCTGCATTAAAATCGTTTGAAATGGATGTGAATAAAATG
CCGCTTGGAAGATTATCACATAATCAAATCAATTTGGCTTTTGAAGTTCTCAATGATATT
AGTGATTTGCTTGTCAAGCTGCCCATTGATGCTTCGAGAATTTTGGATTTTAGTAACAAG
TTCTACACGATTATTCCGCATAATTTTGGAATGCGGGTGCCCGAGCCGATTGATAGTTTT
CATAAAATTAAGGAGAAAAACAACATGCTCAACGCCCTTCTCGACATCAAATTCGCGTAT
GATCAAATCAGCGGTGGAGATGTTCCAGCATCAACGTCATTGGGTATTGATCCAGTTGAT
ATTAATTATCAAAAATTGAAATGTATTATGGAACCACTACAACAAGGCTGTGATGATTGG
AATATGATTCATCAATATTTGAAGAATACTCACGGAGCCACTCATGATTTGAAAGTCGAG
CTGATTGATATTCTAAAACTGAACCGAGACAATGAATCGTCGAAATTCAAACGACACATC
GGAAATCGACGTCTTTTGTGGCACGGATCAGGAAAAATGAATTTCGCCGGAATTTTGGGT
CAAGGACTTCGAATTGCGCCACCGGAGGCTCCAGTTTCTGGATATATGTTCGGAAAGGGC
GTCTATTTTGCTGATATGTTCAGCAAAAGTTTCTTCTATTGCAGGGCCAACGCCAAAGAA
GAAGCATATCTTCTGCTCTGTGACGTGGCACTCGGCAACGTGCAACAGTTGATGGCTTCG
AAGAATGTTTCGAGACAAACTCTGCCAGCAGGCTTCCAGTCTGTGCAGGGACTAGGCCGC
CAATGTCCACGAGAAATTGGAAGTTACAATCATCCGGATGGTTATACCATTCCACTGGGC
CTCACTTACATGCAACTTCAAGGAAAACAGGATGTCGATTATCACTTGCTTTATAATGAA
TTCATTGTGTATGATGTCGATCAAATTCAGCTCAAGTATCTCGTCCGTGTCAAAATGCAC
CATGCTCGTCATCTT
>zebra fish_PARP1
ATGGCCGACTCACAGGACGACAAGCTGTACAAAGCCGAATATGCAAAAAGCGGACGCGCC
TCATGCAAGAAATGCAAAGACAACATTGCTAAGGACTCGCTGAGGATGGCCATCATGGTG
CAGTCTCCCATGTTTGATGGCAAAGTCCCTCACTGGCACCATTTTTCTTGCTTTTGGCTC
CGGGCCGCAGTTCAGTCTCCTTCTGATATATCTGGATTTACTGACCTCCGCTGGGATGAC
CAAGAGAAAGTGAAGACAGCCATTGAGAGTGGAGGTGCTACAGGAGGGAAAGGAGGACAG
AAGGGAGCGGCTAAAGGAGAGAAGACGCTGAATGACTTTGCAGTGGAGTATGCTAAATCC
AACAGAAGCACCTGCAAGGGCTGCGATCAGAAAATTGAAAAGGACCAGATCCGTGTGTCG
AAGAAGACTGTGGACCCAGAGAAACCTCAGCTTGGTCTAATCGATCGCTGGTACCACACC
GGCTGCTTTGTGAGTCGTCGGGAGGAACTGATATTTAAACCAGAGTACAGCGCTGCCCAG
CTCAAAGGATTCGCAGTTCTACGAGATGAAGACAAAGAGGAGCTGAAGAAACGGCTTCCT
GCTGTGAAGAGCGAAGGAAAGAGAAAAGCTGATGAAGTAGATGGAGGAGTCTCTAAAAAA
CAGAAGAAAGAAGATGAAAAACTAGAGCAGAATCTGAAGGATCAAAGCCAGCTGATCTGG
GGAATTAAGGACAAGCTGAAGAAGTTCTGCTCCATCAATGACATGAAGGAACTGCTAATT
GCTAACAGTCAGGAAGTTCCTTCTGGGGAGTCAAATATTGTGGATCGACTATCAGACTGC
ATGGCATTTGGGTCCCTGAAGCCTTGTGAGACTTGCAAAGGGCAGCTTGTGTTTAAAAGT
GATGCGTATTACTGTACTGGAGACATCTCTGCGTGGACTAAATGTGTATTCAAGACTCAG

ACGCCTGATCGCAAAGACTGGGTCACCCCTAAGGAGTTCAGTGAGATCCCGTTCCTGAAG
AAGTTTAAGTTCAAGCGACAGGACCGTGTGTTTCCTAAAGACGCTCCTCCCGCTGCTGCC
ACTCCATCATCTGGTTCTACCACTTCAGCTGCCACAAGTGTGTCCAGCGCCAGTAAAAAC
CTCACTGAGGCACCTGCAGATAAGCCTCTGACTGGAATGAAGCTCCTGGCTGTGGGAAAG
CTCAGTAAGAACAAAGATGATCTGAAGAAATTTGTGGAAGATCTGGGAGGAAAAATCACA
GGCACGGCCAGCAAAGCTGCTCTCTGCATCAGCAGCAAGAAGGAGATTGAGAAGATGAGT
AAGAAAATGGAGGAGGTGAGAGACGCAGGTGTCCGCGTGGTGGCTGATGATTTCCTTACT
GACATCAAGGAGTCCGGCAAGGCCCTTCAGGAGCTCATCTCTCTGCATGCCATTTCCCCC
TGGGGCGCTGAAGTTAAAGTCGAGGCCCCGGCTGCAGCCGCGGCCACCAAATCCACCGGA
GCTCATTCCTCAAAGAGCACCGGCAAAGTCAAAGAGGAGGAAGGTGGAAGCAAATCAAAG
AAAATGAAGCTGACAGTGAAAGGAGGAGCTGCAGTAGATCCAGATTCAGGTCTGGAAAAC
TGTGCTCATGTTCTGGAACAGAATGGAAAGATTTACAGCGCCACACTGGGTCTCGTGGAC
ATCGTCAGAGGGACAAACTCATACTACAAACTACAGCTGCTGGAGGATGACGTTCAAAAG
CGGTACTGGGTGTTTAGATCGTGGGGTCGAGTCGGGACCACCATTGGAGGAAACAAATTG
GACAAGTTTTACGATAAGAACTCTGCTATGGACAATTTCTGTGGTGTGTATGAGGAAAAG
ACAGGGAATGCTTGGGCCTCCAGCAACTTCACAAAGTACCCCAATAAGTTTTACCCTCTG
GAGATTGACTACGGACAGGATGAGGAGGCTGTGAAGAAGTTGACTCAAAGTGCAGGTGCC
AAATCTCAGCTGGAAAAACCTGTGCAAGACCTCATCCGAATGATCTTCGATGTGGAGAGC
ATGAAGAAAGCCATGGTGGAGTTTGAGATTGACTTGCAGAAGATGCCTTTGGGAAAACTA
AGCAAGCGACAGATCCAGAGTGCTTATTCCCTTCTAAGCGAAGTTCAGCAGGCTGTGGCA
GATAGCTCCTCAGAATCACTGATCCTAGATCTGTCTAATCGCTTCTACACACTGATACCG
CACGACTTCGGCATGAAGAAACCACCACTATTGAGTAATGTGGATTACATCCAGCAAAAG
GTGCAGATGCTGGACAACCTTTTGGACATCGAGGTGGCCTACAGCCTGCTTCGGGGAGGA
GTGGAGAACAACGAAAAAGATCCCATTGACATCAACTATGAGAAACTCAAAACCAAAATT
GAGGTCGTCGACAAGTCTTCACATGAGGCTCAGCTTATCCTTCAATATGTGAAGAACACA
CATGCTGCTACTCACAACACCTACACACTGGATGTTGAGGAGATCTTTAAGATTGAGAGG
GAGGGCGAGTATCAGCGTTATCGCCCCTTCAAAGAGTTACCCAACCGCCAGCTGCTGTGG
CACGGCTCGCGCACCACCAACTATGCTGGTATTCTGTCTCAGGGTCTCCGCATCGCTCCT
CCGGAGGCCCCAGTGACGGGTTACATGTTTGGCAAAGGTGTTTACTTTGCTGATATGGTG
TCGAAGAGTGCCAACTACTGCCACACGTCTCAGGCTGATCCTGTGGGCCTCATTCTGTTA
GGAGAGGTTGCATTAGGGAACATGCATGAACTGAAGAAAGCCTCACACATTACAAAGCTA
CCAAAGGGTAAACACAGTGTAAAAGGTTTGGGAAGAAGTGCTCCAGACCCAAGAGCTACA
GTATCTCTCAATGGAGTGGACATTCCTCTGGGCAAAGGCATGAACACAAATATTGATGAC
ACAAGTCTGCTCTACAACGAGTACATCGTTTATGATGTTTCTCAGGTTAACCTGAAATAC
CTGCTGAAGATCCGCTTCAACTATCAGACGTCTCTGTGG
>panda_PARP1
ATGGCAGGGCAGGATCTGCGCATGCGCCTGTCGCCCATGTTTGATGGAAAAGTCCCACAC
TGGTACCACTTCTCCTGCTTCTGGAAGGTCGGCCACTGCATCCGACACCCCGACGTAGAG
GTGGACGGCTTCTCTGAGCTCAGGTGGGATGACCAGCAGAAAGTCAAGAAGACCGCGGAG
GCTGGAGGCGTCACAGGCAAAGGCCAGGATGGAGGCGGCGGCAAGACGGACAAGACGCTG
GCGGACTTCGCGGCCGAGTATGCCAAGTCCAACAGAAGCACCTGCAAGGGCTGCATGGAG
AAGATAGAGAAGGGCCAGATACGCCTGTCCAAGAAGATGCTGGACCCAGAGAAGCCCCAG
CTGGGCATGATCGACCGCTGGTACCACCCGAACTGCTTTGTGAAGAACAGGGAGGAGCTG
GGCTTCCGGCCCGAGTACAGCGCCAGCCAGCTCAAGGGCTTTGGCCTCCTCACCGCCGAA

GATAAGGAAACCCTGAAGAAGCAGCTCCCGGGAGTCAAGAGTGAAGGAAAGAGGAAGGGC
GACGAGGTGGATGGGATGGATGAAGTGGCCAAGAAGAAATCTAAAAAAGAAAAGGACAAA
GATAGTAAGCTTGAGAGGGCCCTCAAGGCCCAGAACGAGCTGATCTGGAACATCAAGGAC
GAGCTAAAGAAAGCGTGTTCAACAAACGACCTGAAAGAGCTGCTCATCTTCAACAAGCAG
CAAGTGCCCTCCGGGGAGTCGGCGATCTTGGACCGCGTGGCGGACGGCATGGTGTTCGGT
GCCCTCCTTCCCTGTGAGGAGTGCTCGGGCCAGCTGGTCTTCAAGAGCGACGCCTACTAC
TGTACGGGCGACGTCACTGCCTGGACCAAGTGTATGATCAAGACCCAGACCCCCAGCCGG
AAGGACTGGGTGACCCCAAAGGAGTTCCGAGAAATTTCTTACCTCAAAAAATTGAAGATC
AAAAGGCAGGACCGAATATTTCCCCCCGAGACCAGTGCCCCAGTGGCCGCGGTGGCCCTG
CCGTCCACAGCCTCGGCGCCCGCTGCTGTGAACAGCTCCACTCCGCCAGATAAGCCGTTG
TCCAGCATGAAGATCCTGACTCTCGGGAAACTCTCCCGGAACAAGGATGAGGTGAAGGCC
ATGATCGAGAAACTCGGGGGGAAGTTGACGGGCACAGCCAACAAGGCCTCCCTGTGCATC
AGTACCAAAAAGGAGGTGGAGAAGATGAGCAAGAAGATGGAGGAGGTGCGAGAGGCTGGC
ATCCGCGTGGTGTCCGAGGACTTCCTCCGGGACTTGTCCGACTCCACCGGGAGCCTCCAG
GACTTGCTTGCGGCCCACGTCCTGGCCCCCTGGGGCGCTGAGGTGAAGGCCGAGCCGGTG
GAGCCCACGGAGCCCGCGGCCCCGAAAGCAAAGTCGGGGGCTGCGCTTTCCAAGAAGAGC
AAGGGCCCCGCCAAGGAGGAAGGCATCAACAAATCTGAGAAGAGAATGAAATTAACTCTC
AAAGGTGGAGCTGCTGTTGATCCGGACTCGGGTCTGGAACACTCCGCGCACGTCCTGGAG
AAAGGTGGGAAGGTGTTCAGCGCCACCCTCGGCCTGGTGGACATCGTGAAGGGAACCAAC
TCCTATTACAAGCTGCAGCTCCTGGAGGATGACAAAGAAAGCCGGTACTGGATCTTCAGG
TCGTGGGGCCGCGTGGGCACAGTGATCGGCAGTAACAAGCTGGAACGGGTGCCGTCCAAG
GAGGATGCCATCGAGCACTTCATGAAACTGTATGAAGAGAAAACCGGGAACGCCTGGCAC
TCCAAGAACTTCACGAAGTATCCCAAAAAGTTCTACCCCCTGGAGATTGACTACGGTCAG
GACGAGGAGGCGGTAAAGAAGCTGACGGTGAACCCCGGCACCAAGTCCAAGCTCCCCAAG
CCAGTGCAGGAGCTCATTAAGATGATCTTCGACGTGGAGAGTATGAAGAAAGCCATGGTG
GAATACGAGATTGACCTTCAGAAGATGCCCTTGGGAAAGCTGAGCAAGAGGCAGATCCAG
GCGGCGTACTCCATCCTTAGTGAGGTGCAGCAGGCGGTGTCCCAGGGCAGCAGTGACTCC
CAGATCCTGGATCTCTCAAATCGCTTCTACACCCTGATCCCCCACGACTTTGGGATGAGG
AAGCCCCCACTCCTGAATAACGCGGACTGTGTGCAGGCCAAGGTGGAGATGCTGGACAAC
CTGCTGGACATCGAGGTGGCCTACAGTCTGCTCAGGGGAGGCTCTGATGACAGCAGCAAG
GACCCCATCGACGTCAACTATGAGAAGCTGAAGACTGACATTAAGGTGGTGGACAGGGAT
TCGGAGGAAGCGGAGACCATCAGGAAGTACGTTAAGAACACTCACGCGACCACACACAAC
GCGTACGACCTGGAAGTCGTTGACATTTTTAAGATCGAGCGCGAAGGCGAGAGCCAGCGC
TACAAGCCGTTCCGGCAGCTGCATAACCGCAGGCTGCTGTGGCACGGGTCCAGGACCACC
AACTTCGCCGGGATCCTGTCCCAGGGCCTCCGGATAGCCCCGCCTGAGGCGCCCGTGACA
GGCTACATGTTTGGGAAAGGGATCTATTTCGCCGACATGGTCTCCAAGAGCGCCAACTAC
TGCCACACATCCCAGGCCGACCCCATCGGCCTGATCCTGCTGGGAGAAGTTGCCCTCGGA
AACATGTACGAACTAAAGCACGCTTCACACATCAGCAAGCTACCCAAGGGCAAGCACAGC
GTCAAAGGTTTGGGCAAGACGACCCCTGACCCTTCAGCCAGCATCACTATGGACGGCGTG
GAAGTCCCCCTGGGGACCGGGATTTCCTCCGGCGTTAACGACACCTGCCTGCTCTATAAC
GAGTACATCGTCTACGACATCGCTCAGGTAAATCTGAGATACCTGCTGAAGCTCAGGTTC
AACTTCAAGACGTCCCTGTGG
>Elephant_PARP1
ATGAGAGGGGAAACCACCTTTTGTTCCTACGTGGTGGGCTTCCAAGTACCTCCACCTGGG

AAGTGGTGCTTCATCGAGTCACCCATATTCGACGGGAGAGTCCCACACTGGTACCACTTC
TCCTGCTTCTGGAAGGTTGGCCATACCCTCCCGCACCCTGACGTGGATGTGGACGGGTTT
TCCGAGCTGCGTTGGGATGACCAGCAGAAAGTCAAGAAGACTGCAGAAGCTGGCGGAGTG
GCAGGCAAAGGCCAGGATGGAAGTGACAGCAAGCTAGACAAGACACTGGGTGACTTCACC
GCCGAGTACGCCAAGTCCAACAGAAGCACATGCAAGGGGTGTATGGAGAAGATAGAAAAG
GGTCACATGCGCCTGTCCAAGAAGATGCTGGACCCGGAGAAGCCACAGATGGGTGTGATC
GACCGCTGGTACCACCCAAACTGCTTCGTCCAGAACAGGGAGAAGCTGGGTTTCCGGCCT
GAGTACAGCGCAAGCCAGATCGCGGGCTTCTCCATCCTCACTCCAGAGGACAGAGAATTC
CTGAAGAAGCTGTTCCCAAAAGTCAAGAGTGAAGGAAAGAGAAGAGGTGATGAAGTGGAT
GGCAGAGATGAAGGGGGCAAGAAGAAATTTAAAAAAGAAAAAGACAAGGATAGTAAGCTT
GAAAAGGCCCTCAAGGCTCAGAACGACCTAATCTGGAATATCAAGGACGAGCTAAAGAAG
GTGTGTTCGACGAATGACCTGAAGGAGCTCCTCATCTTCAACAAGCAGCAAGTGCCTTCC
GGGGAGTCGGCGATCTTGGACCGAGTAGCTGATGGGATAGTGTTTGGTGCCCTCCTTCCC
TGTGAGGAATGCTCAGGCCAGCTGGTCTTCAAGAGCGATGCCTATTACTGTACTGGGGAC
GTCACTGCCTGGACCAAGTGTATGGTCAGGACACAGACACCCAACCGCAAAGAATGGGTG
ACCCCAAAGGAATTCCGGGAAATCTCTTACCTCAAGAAACTGAAAATCAAAAAGCAGGAC
CGCCTGTTCCCCCCTGAGGCCAGCGCCCCAGTGGTGGCAGCACCCCCACCGTCCAAAGCC
ACACCGCCCCCCACTGAGAACTCTTCTGCTCCAGCAGATAAACCATTGTCCAACATGAAG
ATCCTCACTCTGGGGAAGCTGTCCCGGAACAAGGAGGAAGTGAAGGCCATGATCGAGAAG
CTTGGGGGCAAGCTGACAGGAACAGCCAACAAGGCTTCCGTGTGCATCAGCACCAAGAAG
GAGGTGGAGAAGATGACGAAGAAGATGGAGGAAGTAAAAGAAGCCAACGTTCGAGTTGTG
TCTGAGGACTTCCTCCAGGATGTCTCAGGCTCCACCAAGAGCCTTCAGGAGCTGCTGTCA
GTGCACGTCTTGTCATCCTGGGGGGCTGCGGTGAAGGTGGAGCCTGCCGAAGTAGTGGCC
CCAAAAGCAAAGTCTGGGGCTGCGGTCCCCAAGAAGAGCAAGGGCCCCGTCAAGGAGGAA
GGTGTCAACAAATCTGAAAAGAGAATGAAGTTGACTCTCAAAGGAGGAGCAGCTGTGGAT
CCTGAGTCTGGTCTGGAACATTCTGCACATGTCCTGGAGAAAAGCGGGAAGGTCTTCAGT
GCCACCCTGGGCCTGGTGGACATCGTCAAAGGAACCAACTCCTATTACAAGCTACAACTT
CTGGAGGACGATAAAGAAAGCAGGTACTGGATATTCAGGTCCTGGGGCCGCGTGGGCACA
GTGATCGGGAGTAACAAACTGGAGCAGATGCCGTCCAAGGAGGATGCCATTGAGCACTTC
ATGAAATTATATGAAGAAAAAACCGGGAATGCCTGGAATTCCAAGAACTTCACGAAGTAT
CCCAAAAAATTCTACCCTCTGGAGATTGACTACGGCCAGGATGAAGAGGCAGTGAAGAAG
CTGACAGTAAACCCTGGCACCAAGTCCAAGCTTCCCAAGCCAGTTCAGGAACTCATTAAG
ATGATCTTCGATGTGGAAAGTATGAAGAAGGCCATGGTGGAGTACGAGATTGACCTTCAG
AAGATGCCCTTGGGGAAGCTGAGCAAACGGCAGATCCAGGCAGCATACTCCATCCTCAGT
GAGGTCCAGCAGGCGTTGTCCCAGGGCAGCAGCGACTCCCAGATCCTGGACCTCTCAAAT
CGCTTCTACACCCTGATCCCCCATGACTTTGGGATGAAGAAGCCTCCACTCCTGAACAAT
GCAGACAGCGTGCAGGCCAAGGTGGAAATGCTGGACAACCTGCTAGACATCGAGGTGGCC
TACAGTCTGCTCAGGGGTGGCTCTGAGGACAGCAGCAAGGACCCCATCGATGTCAACTAT
GAGAAGCTCAAAACTGAGATTAAGGTGGTTGACAGAGATTCTGAAGAAGCCGAGATCATC
AGGAAGTACGTTAAGAACACTCACGCCACCACACACAATGCCTATGACTTGGAAGTCGTT
GATATCTTTAAGATAGAGCGTGAAGGGGAGAACCAGCGTTACAAGCCCTTCAGGCAGCTT
CACAACCGGCAGCTGCTGTGGCACGGCTCCAGGACCACCAACTTTGCTGGAATCCTGTCC
CAGGGTCTCCGGATTGCGCCCCCGGAAGCGCCTGTGACAGGCTACATGTTTGGTAAAGGA
ATCTATTTCGCCGACATGGTCTCCAAGAGTGCCAACTACTGCCACACATCTCAGGGAGAC

CCAATAGGATTGATCCTCTTGGGAGAAGTTGCTCTTGGAAATATGTATGAACTGAAGCAT
GCGTCACATATCAGCAAGTTACCCAAGGGCAAGCACAGTGTCAAAGGTTTGGGCAAAACG
ACCCCTGACCCTTCAGCTAGTATTACTCTGGATGGTGTGGAGGTTCCTCTTGGGACCGGC
ATTTCATCTGGTGTTAATGACACTAGTCTACTGTATAATGAGTACATCGTCTACGATATT
GCTCAGGTAAATCTGAAGTATCTGCTAAAACTGAAATTCAATTTTAAGACGTCCCTCTGG
>turkey_PARP1
ATGTTTGATGGCAAAGTCCCTCACTGGCACCACTACAGCTGCTTCTGGAAGCGGGCGCGG
ATCGTGTCCCACACGGACATCGATGGCTTCCCTGAGCTCCGCTGGGAGGACCAGGAGAAA
ATCAAGAAGGCCATTGAAACTGGAGGCCCTGGAGGAGGAAAAGGAGGGGACCAGGAAGGA
GGTGGCAAGGCTGAGAAGAGCCTAACTGACTTTGCTGCAGAGTATGCCAAGTCTAACAGG
AGTACTTGCAAAGGTTGTGAGCAGAAAATAGAAAAGGGCCAGATTCGGATTTCCAAGAAG
ATGGTGCATCCCGAAAAACCACAGCTGGGGATGATCGATAACTGGTACCACCCGGACTGC
TTTGTGAGCCGCCGGGCAGAGCTGGGCTTCCTCCCAGCATATGGGGCCACCCAGCTCCTG
GGTTTCAGCATCTTGAAAGCTGAAGATAAAGAAACTCTGAAGAAGCAACTACCGGCTGCC
AAGACTGAAGGAAAGAGAAAAGGAGAAGAGGTAGATGGAAATGTGATTGCAAAAAAGAAA
TCAAAAAAAGAAAAAGAAAAAGAATCAAAGCAGGAAAAACAGCTGAAGGAGCAGACAGAG
CTGATCTGGGGCATCAAGGATGAGCTGAGGAAGGTCTGCTCCACCAATGACCTGAAAGAG
CTGCTGATTGCCAACAAGCAGGAGGTGCCTTCAGGGGAGAATGCAATTTTGGACCGAGTG
GCAGATGGGATGGCGTTTGGAGCTCTGCTTCCCTGCGAGGAATGTAAGGGGCAGTTTGTG
TTCAAGAGCGATGCATACTACTGTTCAGGGGACATTACTGCCTGGACTAAGTGTGTGGCT
AAAACACAGACTCCCAACAGGAAAGACTGGGTAATCCCAAAGGAATTTCGTGAAATTCCT
TATCTCAAAAAGTTTAAGTGTAAGAAGCAGGACAGGATATTCCCTCCTGAGGCTGCAACT
GTGAACTCTGTGCCACCTCCATCTGTATCTGCTCCTTTGACAGAGACTGTCTCTACACCC
CAAGACAAACCACTGACCAACATGAAGATCCTGACCCTTGGAAAGCTGTCCAAGAACAAG
GAGGAAGTGAAGAATATTGTGGAGGAGTTGGGAGGAAAAATGACAACAACAGCTAACAAG
GCCACCCTGTGCATCAGCACACAGAAGGAGGTGGAGAAAATGAGCAAGAAGATGGAAGAA
GTGAAGGATGCCAAAGTTCGTGTGGTCTCAGAGGAGTTTCTTAAGGATGTGAAATCTTGC
AGCAAGAGCTTTCAGGAGCTTCTCTCTCTCCATGCAATTTCACCTTGGGGTGCAGAGGTG
AAAACGGAGCACCAGGAGGTGGCGGTGGATGGGAAGTGCAGCAAGCCCCCAAATATGAAG
AGTGCTGGGAAGGTCAAAGAAGAACAAGGACCTAGCAAGTCTGAAAAGAAAATGAAGCTA
ACAGTTAAAGGTGGAGCAGCAGTAGATCCTGACTCTGGCTTGGAGGATTCTGCTCACGTC
TTTGAAAAAGGTGGAAAGATTTTCAGTGCAACTCTTGGCCTAGTAGATATTGTGAAAGGA
ACAAATTCCTATTACAAACTGCAGCTGCTAGAGGATGACAGAGAGAGCAGGTACTGGGTG
TTCAGATCCTGGGGTCGTGTGGGCACTGTCATTGGGAGTAACAAGCTGGAGCAGATGCCA
TCAAAAGAAGATGCTGTTGAACACTTCCTAAATTTGTATGAAGAGAAAACTGGCAATTCT
TGGCATTCAAAGAACTTCACTAAATATCCAAAAAAATTCTACCCACTGGAAATAGATTAT
GGACAGGATGAAGAAGCTGTCAAGAAACTGACAGTGGGTGCTGGGACTAAATCAAAGCTT
GCTAAGCCAATCCAAGACCTTATTAAGATGATTTTTGATGTGGAGAGCATGAAGAAAGCT
ATGGTGGAATTTGAGATCGACCTGCAGAAGATGCCACTGGGGAAACTGAGCAAGCGACAG
ATCCAAAGTGCATACTCCATCCTTAATGAAGTTCAGCAGGCAGTTTCTGATGGTGGTTCT
GAATCCCAGATCTTGGACCTCTCAAACCGTTTCTATACTCTGATCCCTCATGACTTTGGG
ATGAAGAAACCACCTCTTCTCAGTAACTTAGAATATATCCAGGCTAAAGTGCAGATGTTG
GACAACTTGCTTGATATTGAGGTTGCCTACAGTCTTCTCAGAGGTGGAAATGAAGATGGA
GATAAAGACCCAATTGATATCAATTATGAAAAGCTTCGAACTGATATTAAGGTAGTTGAC

AAAGATTCAGAAGAAGCCAAGATTATTAAACAATATGTGAAAAATACTCATGCTGCTACT
CACAATGCATATGACCTCAAAGTTGTGGAAATCTTCAGGATTGAACGTGAAGGAGAGAGT
CAGCGTTACAAGCCATTTAAGCAGCTTCATAATCGCCAGCTGCTGTGGCACGGTTCCCGC
ACCACCAACTTCGCTGGTATCCTCTCGCAGGGTCTCCGGATAGCTCCCCCTGAAGCTCCT
GTGACTGGCTACATGTTTGGGAAAGGCATCTACTTTGCAGACATGGTGTCCAAGAGTGCC
AACTACTGTCACACATCTCAAGCTGATCCAATTGGGTTAATACTACTGGGAGAAGTTGCC
CTTGGAAATATGTATGAGCTAAAGAATGCTTCTCACATAACAAAATTGCCCAAAGGAAAG
CATAGTGTGAAAGGCTTGGGTAAAACTGCACCTGATCCCACAGCCACTACAACCCTTGAT
GGTGTAGAAGTTCCCTTAGGGAATGGGATCTCAACAGGAATTAATGATACCTGTCTTCTG
TACAATGAATATATTGTGTATGATGTTGCTCAGGTAAATCTGAAGTACCTGCTGAAACTG
AAATTCAACTATAAGACATCACTCTGG
>zebra finch_PARP1
ATGGCGGCGCGCAGAGGGCCGTTCTTCTCCCGGACCTGCACCACCACCCCGCTGGACGGG
GCCGTGCCGCTCCGGCCGGGCTCGGCGGCCGCCGCGCTCTCGCCGTCTTTGGGCGAGGGC
TCCATGGCCGTGCCCCGGCGGGACCGCAGCTCCCGCCGCCGAGCGCGCTCCCGCCGCCGG
GCGCGGCCCCGCCTGCGCGCCCCCCGCGCCGACCCCTCCCCGCCCTCCGCCCGCCCCTCG
CGGCGCCGCATCCCGCCCGCATCCCGCCCGAGCCCGCCCGCATCCCGCCGGAGCCCGCCC
GCATCCCGCCGGGAGCCGGAGCCCCGAGCCCCCCGCCTCGTCCCGCCTGGGCAGCCAGAG
CCAGGGCGAGGGTGGGACCAAACTGATCTGTCTCTGAACCACTGGTGTCTGGGCCAGGAA
CTCCTGGAAGGAGTGATCAGCAAGAGCGAAGATTCTGCTGTGTGGAGCACCCGGCTGTCT
GTGTGCTCGCAGCACCATCTATCTAGTGTTTACGGCCATCGCTCCTCGTCCTGTCGCTGG
GCACCACCGGAACGCCCGCCTGGAAATGGCATCACGAGGCCAGTATTTGTCTGCGGGAGA
GCTGCTGAGTTCAGGCTCTCCCAAGAGGCGGGAGCCCGCTCTGTGTCGCTCGGGGGCGGG
ACAGGGGCGCGGGGTGGCGGCGGGACCCGAACCGCGGCCCTGAGGGCGCAGCACGGGACG
CGCCGCCGCGCCCGCCTCCCATTGATACACAAGAGGAACCTCCAGACATGTGCAATCGAT
GCCCGAGCGGACGATCCGCCCGGCCGGCGAGGCATAGTGCGGGCTCGGCGTTCGGCTGGC
GCTGCCGCGGGCGGGGTCGCGGCGGGCGCGCGGGGCGGGGTCGCGGCGGGCGCGCGGCGC
GGCGCGGCGCGGGGCGCGGCGCGGCAGCGGGAGCGGGAGAGGCGGCGATGGGAGCCGGCG
GAGAAGCTGTACCGGGCCGAGTATGCCAAGAGCGGCCGCGCCTCCTGCAAGAAGTGCGGC
GAGAGCATCGCCAAGGACTCGCTGCGCCTGGCGCTCATGGTGCAGGGCCAGATTCGGATT
TCCAAGAAGATGGTGCATCCTGAAAAGCCTCAGCTGGGAATGATAGATAACTGGTACCAC
CCGGACTGCTTTGTGAGCCGCCGAGCCGAGCTGGGCTTCCTCCCGGCCTACGGGGCCACC
CAGCTCCTGGGCTTCAGCATCCTGAAAGCTGAAGATAAAGAAACTCTGAAAAAGCAGCTC
CCAGCTACCAAGAGTGAAGGAAAGAGAAAAGGAGAAGAGGTAGATGGAAATGTGACTGCA
AAAAAGAAGCAGAAAAAAGAAAAAGAGAAAGAAACAAAGCAGCAGAAACAGCTGAAGGAG
CAGACAGAGCTGATCTGGGGCATCAAGGATGAGCTGAGGAAGGTCTGCTCCACCAATGAC
CTGAAAGAGCTGCTGATTGCCAACAAGCAGGAGGTCCCCTCAGGGGAGAATGCTATCTTG
GACCGAGTAGCAGATGGGATGGCCTTCGGCGCTCTGCTGCCGTGTGAGGAGTGCAAGGGG
CAGTTTGTGTTCAAGAGTGACGCTTATTACTGTTCAGGGGATATCACTGCCTGGACCAAG
TGCGTGGCTAAAACACAGACTCCCAACAGGAAAGACTGGGTAATCCCAAAGGAGTTCTGG
GAAATTCCTTACCTGAAGAAATTTAAATGTAAGAAGCAGGACAGGGTGTTCCCTCCAGAT
GCTGCGACTGTGAACTCGGCACCTCCTCCCTCTGCATCTGCTCCTTTGTCAGAGACTGTG
TCTGCACCCCGAGACAAACCACTGACCAACATGAAGATCTTGGTTGTTGGGAAGCTGTCA
AAGAACAAGGAGGAGGTGAAAAGCATCGTGGAGGACCTAGGAGGAAAGATGACCACAACA

GCTAACAAGGCAACCCTGTGCATCAGCACCCAGAAGGATGTGGAGAAAATGAGCAAGAAG
ATGGAAGAAGTGAAGGAGGCCAAAGTCCGTGTGGTCTCAGAGGCATTTCTTCAGGATGTG
AAGTCTTCCAGCAAGGACTTCCAGGAGCTTGTGTCTCTCCATGCCCTTTCACCTTGGGGT
GCAGAGGTGAAAATGGAGCACGAGGAAATGGCTGTGGATGGGAAGAGCAGCAAGCCCCCA
AGTACAAAGAGTGCTGGGAAGGTCAAAGAAGAACAAGGACCTAGCAAGTCTGAAAAGAAA
ATGAAGTTAACAGTGAAGGGTGGAGCAGCAGTAGATCCTGATTCTGGTTTGGAGGATTCT
GCTCATGTCTTTGAAAAAGGTGGGAAAATTTTTAGTGCGACCCTGGGACTGGTAGATATT
GTGAAAGGAACAAATTCCTATTATAAGCTGCAGCTGCTAGAGGATGACAGGGAGAACAGA
TACTGGGTGTTCCGATCCTGGGGCCGTGTGGGCACTGTAATCGGCAGTAACAAGCTGGAG
AAGATGCCATCAAAAGAAGATGCCATTGAGCACTTCCTGAATTTGTATGAAGAGAAAACT
GGCAATTCTTGGCATTCGAAGAACTTCACTAAATATCCCAAAAAATTCTACCCACTGGAA
ATAGATTACGGACAGGATGAAGAAGCTGTCAGGAAACTGACAGTAGGTGCCGGGACAAAA
TCAAAACTCGCTAAGCCAATCCAGGATCTTATTAAGATGATCTTTGATGTGGAGAGCATG
AAGAAAGCAATGGTGGAATTTGAGATTGACTTGCAGAAGATGCCGTTGGGAAAACTGAGC
AAGCGACAGATCCAGAGTGCATACTCCATCCTGAATGAGGTCCAACAGGCAGTTTCTGAC
AATGGTTCAGAATCCCAGATTTTGGATCTCTCCAACCGCTTCTATACACTGATTCCTCAT
GACTTTGGGATGAAGAAGCCACCTCTTCTAAATAACTTGGAATACATTCAGGCTAAAGTG
CAGATGTTGGACAACTTGCTTGATATTGAGGTTGCTTACAGCCTTCTCAGAGGTGGAAAT
GAAGATGGAGATAAAGACCCAATTGACATCAACTATGAAAAACTTAAAACAGATATTAAG
GTTGTTGACAAAGATTCAGAAGAAGCCAAGATTATCAAACAATATGTGAAAAACACTCAT
GCGGCTACGCACAACGCATATGACCTCAAAGTTGTGGATATCTTCAGGATTGAGCGTGAG
GGGGAGAGCCAGCGTTACAAGCCCTTCAGGCAGCTCCACAACCGCCAGCTGCTGTGGCAC
GGCTCCCGCACCACCAACTTCGCTGGGATACTCTCGCAGGGTCTGCGGATAGCTCCTCCT
GAAGCTCCTGTGACCGGCTACATGTTTGGGAAGGGCATCTATTTTGCAGACATGGTATCC
AAGAGTGCAAACTACTGTCACACATCTCAAGCTGATCCCATAGGTTTAGTACTACTGGGA
GAAGTCGCCCTTGGAAATATGTATGAACTAAAGAATGCTTCCCACATCACAAAATTGCCC
AAGGGAAAACACAGTGTCAAAGGCTTGGGCAAAACTGCGCCTGATCCCACAGCCACGACC
ACCCTTGGTGGTGTGGAGGTTCCCTTAGGGAATGGGATCTCCACAGGAATTAATGATACC
TGTCTTCTGTATAATGAATATATTGTGTATGATGTTGCTCAGGTGAATCTGAAGTACCTG
TTGAAACTGAAATTCAACTATAAGACATCACTCTGG
>American_alligator_PARP1
ATGGCGGAGCCGGCGGACAAGCTGTATCGGGCCGAGTACGCCAAGAGCGGGCGGGCCTCG
TGCAAGAAATGCGGGGAGAGCATCGCCAAGGACTCGCTGCGCCTGGCTATCATGGTGCAG
TCACCCATGTTCGATGGCAAAGTCCCTCACTGGCATCATTACACTTGTTTTTGGAAGCGG
GCTCGGATCACGTCTCATGCAGATATTGATGGCTTCCCTGAGCTGCGATGGGAAGATCAA
GAGAAAATAAAAAAAACCATTGAAGCAGGGGGACCTGCCACAGGTAAAGGTGGTGACCAG
GAAGGAGGTGGCAAGGCTGAGAAAAGTTTACATGACTTTGCAGTAGAATATGCCAAGTCT
AACAGAAGTACCTGCAAAGGCTGTGAACTGAAAATAGAAAAGAGTCAGATCAGAATTTCT
AAGAAGATGGTGCATCCAGAAAAGCCCCAGCTGGGAATGATAGATAACTGGTACCATCCA
GACTGCTTTGTGAGCCGCCGAGCAGAGCTGGGCTTTCTCCCTGCGTTTGGTGCCAGTCAG
CTCCAGGGCTTTGGCATTTTGGTAGCTGAGGATAAAGAATCCCTGAAGAAGCAGCTGCCT
GCCGTCAAGAGTGAAGGAAAAAGAAAAGCAGATGAGATGGATGGGACTGTGACTACAAAA
AAGAAACAAAAAAAAGAAAAGGATAAAGAATCCAAACAGGAGAAGCTGCTGAAGGAACAG
ACGGAATTGATTTGGAACATCAAAGACGAGCTGAGGAAAGCCTGCTCCACCAACGACCTG

AAAGAGCTGCTGATGGCCAACAAACAGGAAGTGCCTTCTGGGGAATCTGCTATCTTGGAC
AGAGTGGCAGATGGGATGGCATTTGGAGCTCTGCTTCCCTGTGAAGAGTGCAAGGGGCAG
TTTATGTTCAAGAGTGACGCGTACTACTGCTCAGGAGACATTACTGCCTGGACTAAATGT
GTTGCCAAAACACAGTCTCCCAACAGGAAAGAATGGGTTATCCCAAAGGAATTCCGGGAA
ATCCCTTACCTTAAGAAATTCAAGTGTAAAAAGCAGGACAGAGTATTCCCTCAAGAGGCT
GCTGCTGTGAACACTGTGCTTCCAAGTGCAGCTTCTGCTCCTTTGACAGAGGAGGCATCT
GCACCCACAGATAAGCCATTATCCAACATGAAGATTTTGATACTTGGAAAATTATCCAGG
AACAAAGAAGAAATGAAGACCAGAGTTGAGGAGCTTGGAGGAAAAGTGACAGGAACTGCC
AATAAGGCCAACCTGTGCATCAGCACACAAAAGGAAGTTGAGAAAATGAACAAGAAGATG
GAAGAAGTGAAGGAGGCAAAAGTCCGAGTGGTTTCAGAAGAATTTCTCAAGGATGTGAAA
TCTTCCAGCAAAGGCTTTCGGGAACTGCTGTCAGTACATGAGCTCTCATCCTGGGGTGCA
GAGGTGAAGCAGGAGAACATGGAGACAACTGTGGGAGGAAAGTCCAGTGGGCCCCCAAAT
ATGAAGAGTGCTGGAAAAGTCAAGGAAGAGCAAGGGACCTGCAAGTCTGAAAAGAAAATG
AAATTGACAGTTAAAGGAGGAGCGGCCGTAGATCCTGATTCTGGTTTGGAGGACTCTGCT
CACGTCTTTGAAAAAGGTGGTAAAATCTACAGCGCCACTCTTGGCCTGGTAGATATTGTC
AAAGGAACCAATTCCTATTACAAACTGCAGCTGCTGGAGGATGACCGAGAAATCAGGTAC
TGGGTGTTTAGGTCATGGGGTCGCGTTGGCACTGTGATTGGGAGTAACAAGCTGGAGCAG
ATGCCATCTAAAGAAGATGCCATTGAGCACTTTTTAAATCTGTATGAAGAGAAAACGGGC
AATTCCTGGCATTCCAAGAACTTCACGAAATATCCAAAGAAATTCTACCCTCTAGAAATA
GATTATGGACAGGATGAAGAAGCTGTGAAGAAACTGACAGTAGGTGCCGGCACAAAGTCA
AAGCTTCCTAAATCAGTCCAGGACCTTATTAAGATGATCTTTGATGTGGAGAGCATGAAG
AAAGCCATGGTGGAATTTGAGATTGACCTCCAGAAGATGCCATTGGGAAAGCTGAGCAAG
AGACAGATCCAGAGTGCCTATTCCATCCTTAATGATGTTCAGCAGGCAGTTTCTGGTGGC
GGCACTGATTCTCAGATACTGGACCTCTCCAATCGCTTCTACACGTTGATACCTCATGAC
TTTGGGATGAAAAAGCCACCTCTTTTAAATAACCTAGAATATATTCAGGCCAAAGTGCAG
ATGTTGGACAACCTGCTTGATATTGAGGTTGCTTACAGCCTTCTCAGAGGTGGAAACGAG
GATGGGGATAAAGACCCAATTGATGTCAACTATGAAAAACTCAAAACTGAAATTAAGGTG
GTTGATAAAGATTCAGAAGAAGCCAAGATCATAAAGCAATATGTGAAGAATACCCATGCT
GCTACCCACAATGCATATGATCTGAAAGTTGTGGATATCTTCAAAATTGAGCGTGAAGGG
GAGAGTCAGCGTTACAAGCCATTCAGACAGCTTCACAATCGCCAGCTGCTCTGGCATGGC
TCACGTACTACCAACTTTGCTGGTATTCTCTCACAGGGTCTCCGAATAGCTCCGCCTGAA
GCTCCTGTGACTGGTTACATGTTCGGAAAAGGTGTCTATTTTGCAGACATGGTTTCCAAG
AGCGCCAACTACTGTCACACATCTCAGGCTGATCCAGTAGGCTTAATCTTATTGGGAGAG
GTTGCCCTTGGAAACATGTATGAGCTAAAGAATGCTTCTCACATAACTAAGCTGCCGAAG
GGGAAACATAGCGTCAAAGGTTTAGGCAAAACTGCGCCTGATCCCACAGCCACCATCAGT
TATGAGGGTGTAGAAGTTCCTTTGGGGAATGGAATGTCAACAGGAATTAATGATACTTGT
CTTCTGTATAATGAATATATTGTCTACGATGTTGCTCAGGTAAACTTGAAGTACCTGCTG
AAACTGAAATTCAACTACAAGACATCACTCTGG
>Armadillo_PARP1
ATGGCTCTGGCGGGTGAGGCGCGCGGGCAGCTCCGCCAGGCGACTCCTGTCTCCTCTCGC
CGGACATTGGGGTGCTGGCGACGGCACGGCGGAGACCCCGACTCAGCCAGCGGGGCGCCC
CACCCCGGGCTTCGCGTCTCGTCACTGGCGTTCAGCGGCGTTGCTTTGGGCCCGCGCCCG
GCGACGGCCCCTCCCGGGGAGCCGGGCGCGCCAAGCCGCCAGGTGCTCTACCTTCTGGAG
GGTCCAGGTGGGGACAAAGGCTGGTTTCCATGGTTTCACGAGGTGGGGGCCAACAGCCCC

ATCGAAGAGGGAACGTCCCTCCATCAAGCAAACGGCGGGGACTGTGGCTTCCTTAACGTT
GGGCGCCTGGAGCCGCAGCCGCCCGCTGAGCCTCCAGCCAACTGGGAACGGGTGGGCCCC
TGGGTGCAGGTCAGCGGCCCCCGGCTGAGGTCCCCCATGTTTGATGGGAAGGTCCCACAC
TGGTACCACTTTTCCTGCTTCTGGAAGGTCGGCCACTCCATCCGGCACCCTGACGTCGAA
GTGGATGGGTTCTCCGAGATTCGGTGGGATGACCAGCAGAAAATCAAGAAGACGGCAGAG
GCCGGGGGCGTGACGGGCAAAGGCCAGGATGGAGGTGGCAGCAAGACGGAGAAGACACTG
GGTGACTTTGCAGCAGAGTATGCCAAGTCCAACAGAAGCACCTGCAAGGGCTGTATGGAG
AAGATAGAAAAGGGCCAGGTACGCCTGTCCAAGAAGATGCTAGACCCAGAGAAGCCACAG
CTGGGCATGATCGACCGCTGGTACCATCCACACTGCTTCGTCAGCAGCAGGGCGGAGCTG
GGCTTCCGGCCCGAGTACAGCGCCAGCCAGCTCAAGGGCTTCAGCCTCCTCACTCCAGAG
GACAAAGAAGCCCTGAAGAAGCAGCTCCCAGGAGTCAAGAGTGAAGGAAAGAGAAAAGGT
GATGAGGTAGATGGAATTGATGAAGTGGCCAAGAAGAAATCCAAAAAAGAAAAAGACAAG
GATAGTAAGCTAGAAAAGGCCCTCAAGGCCCAGAATGACCTGATCTGGAACATCAAGGAT
GAGCTGAAGAAAGTGTGTTCAACCAACGACCTGAAGGAGCTCCTCATCTTCAACAAGCAG
CAAGTGCCCTCCGGGGAGTCAGCGATCTTGGACCGCGTTGCTGACGGGATGGCGTTTGGG
GCCCTCCTTCCCTGTGGGGAGTGCTCGGGCCAGCTGGTCTTCAAGAGCGACGCTTACTAC
TGTACCGGCGATGTCACTGCCTGGACCAAGTGTACAGTGAAGACACAGACACCCGGTCGG
AAGGAATGGGTGACCCCAAAGGAATTCCGGGAAATCTCTTACCTCAAGAAATTGAAAATC
AAGAAGCAGGACCGTTTATTTGCCCCAGAGACCAGTGTCCCAGCAGCAGCACCCCCGCTG
CCCGCTGCCTCAGCGCCCGCTGCCCTGAACTCCTCCGCTCCAGCAGATAAACCACTCTCC
AACATGAAGATCCTGACTCTCGGGAAGCTCTCGCGGAGCAGGGATGAGGTGAAGGCCACC
GTGGAGAAGCTCGGCGGGAAGCTGACGGGGACGGCCGGCAAGGCCTCGCTGTGCGTCAGC
ACTCGGAAGGAGGTGGAGAAGATGAATAAGAAGATGGAGGAAGTAAAAGAAGCCAATATT
CGCGTTGTGTCTGAGGATTTCCTGCAGGACGTCTCTACCTCAACTAAGAGCCTTCAGGAG
CTGTTCTCAGCACACGTCTTGTCCCCCTGGGGGGCCGAGGTGAAGACGGAGCCTGGAGAA
GCAGTGGCCCCGAGAGGGAAGTCGGCTGCTCCCTCCAAGAAGAGCAAGGGCCCCGTCAAG
GAGGAAGGTATCAACAAATCTGAAAAGAGAATGAAGCTAACTCTTAAGGGAGGAGCAGCA
GTCGATCCTGATTCTGGCCTGGAGCACTCTGCGCACGTCCTGGAGAAGGGCGGGAAGGTC
TTCAGTGCCACCCTCGGCCTGGTGGACATCGTGAAAGGAACCAACTCCTACTACAAGCTG
CAGCTTCTGGAGGACGACAAGGAAAGCAGGTACTGGATATTCAGGTCCTGGGGCCGTGTG
GGCACAGTGATTGGTAGCAACAAGCTGGAGCAGATGTCATCCAGGGAAGATGCCGTTGAG
CACTTCATGAAATTATATGAAGAAAAAACCGGGAATGCCTGGGACTCCAAAAATTTCACT
AAGTATCCCAAAAAGTTCTACCCGCTGGAGATTGACTATGGCCAGGATGAAGAGGCAGTG
AAGAAGCTGACGGTAAACCCTGGCACCAAGTCCAAGCTCCCCAAGCCGGTTCAGGATCTC
ATCAAGATGATCTTTGATGTGGAAAGTATGAAGAAAGCCATGGTGGAGTACGAGATTGAC
CTTCAGAAGATGCCCTTGGGGAAGCTGAGCAGAAGGCAGATCCAGGCCGCCTACTCCATC
CTCAGCGAGGTCCAGCAGGCAGTGTCGCAGGGCAGCAGCGACTCGCAGGTCCTGGACCTC
TCGAACCGCTTCTACACCCTGATCCCCCACGACTTTGGGATGAAGAAGCCTCCGCTGCTC
AACAGTGCCGACAGCGTGCAGGCCAAGGTGGAGATGCTGGACAACCTGCTGGACATCGAG
GTGGCCTACAGCCTGCTCAGGGGGGGCTCTGACGATGGCAGCAGAGACCCCCTCGACGTC
AACTACGACAAGCTCAAAACCAACATTCAGGTGGTGGACAGGGCTTCTGAAGAGGCCGAG
ATCATCAGGAAGTACGTCCGGAACACGCACGCGGCCACGCACAACGCCTACGACCTGGAG
GTGGAGGATATCTTTAGGATAGAGCGTGAAGGGGAGGGCCAGCGCTACAAGCCCTTCAGG
CAGCTGCACAACCGAAGGCTGCTGTGGCACGGGTCCCGGGCCACCAACTTTGCCGGCATC

CTGTCCCAGGGTCTCCGGATCGCTCCACCTGAGGCACCCGTGACGGGCTACATGTTTGGG
AAAGGAATCTATTTCGCCGACATGGTCTCCAAGAGCGCCAACTACTGCCACACGTCCCAG
GGAGACCCCACGGGCTTGGTCCTGCTGGGCGAGGTCGCTCTTGGGAACATGTACGAGCTG
AAGCACGCGTCACATATCAGCAAGTTACCCAAGGGCAAGCACAGTGTCAAAGGTTTGGGC
AAAACCACCCCTGACCCTTCAGCAAGCATTACTCTGGATGGGGTCGAGGTGCCTCTCGGG
CCGGGGGTGTCGTCTGGTGTGAATGACACCTGTCTGCTGTATAACGAGTACATTGTCTAT
GATGTTGCTCAGGTAAATCTGAAGTATCTGCTGAAACTGAAATTCAATTTCAAGACGACC
TTGTGG
>Chicken_PARP1
ATGGCGGAGACGGGGGACAAGCCGTACCGCGCGGAGTACGCCAAGAGCGGGCGGGCGTCG
TGCAAGAAGTGCGGCGAGAGCATCGCCAAGGACTCGCTGCGCCTGGCCCTCATGGTGCAG
TCGCCCATGTTCGATGGCAAAGTCCCTCACTGGCACCACTACAGCTGCTTCTGGAAGCGG
GCGCGGATCGTGTCCCACACAGACATCGATGGCTTCCCTGAGCTCCGCTGGGAGGACCAG
GAGAAAATCAAGAAGGCTATTGAAACCGGAGGCCCTGCAGGAGGAAAAGGGGGGGACCAG
GAAGGAGGTGGCAAGGCTGAGAAGAGCCTAACTGACTTTGCTGCAGAGTATGCCAAGTCT
AACAGGAGTACTTGCAAAGGCTGTGAGCAGAAAATAGAAAAGGGCCAGATTCGGATTTCC
AAGAAGATGGTGCATCCTGAGAAACCACAGCTGGGGATGATCGATAACTGGTACCACCCG
GACTGCTTCGTCAGCCGCCGGGCAGAGCTGGGCTTCCTCCCAGCATATGGGGCCACACAG
CTCCTGGGTTTCAGCATCTTGAAAGCTGAAGATAAAGAAACTCTGAAGAAACAGCTACCA
GCTACTAAGACTGAAGGAAAGAGAAAAGGAGAAGAGGTAGATGGAAATGTGGTTGCAAAA
AAGAAATCAAGAAAAGAAAAAGAAAAAGAATCAAAGCAGGAAAAACAGCTGAAGGAGCAG
ACAGAGCTGATCTGGGGCATCAAGGATGAGCTGAGGAAGGTCTGCTCCACGAATGACCTG
AAAGAGCTGCTGATCGCCAACAAGCAGGAGGTGCCTTCAGGGGAGAATGCAATCTTGGAC
AGAGTGGCAGACGGGATGGCGTTTGGAGCTCTGCTTCCTTGTGAGGAGTGTAAAGGGCAG
TTTGTGTTCAAGAGCGATGCATACTACTGCTCAGGGGACATTACTGCCTGGACTAAGTGT
GTGGCTAAAACACAGACTCCCAATAGGAAAGACTGGGTAATCCCGAAGGAATTTCGTGAG
ATTCCTTATCTCAAAAAATTTAAGTGTAAGAAGCAGGATAGGATATTCCCTCCTGAGGCT
GCAACTGTGAACTCTGCACCGCCTCCATCTGCATCCGCTCCATTGACAGAGACTGTCACT
GCACCCCAAGAGAAACCACTGACCAACATGAAGATCCTGACTCTTGGAAAGCTGTCCAAG
AACAAGGAGGAAGTGAAGAATATTGTGGAGGAGCTGGGAGGAAAGATGACAACAACAGCT
AACAAGGCCACCCTGTGCATCAGCACACAAAAGGAGGTGGAGAAAATGAGCAAGAAGATG
GAAGAAGTGAAGGATGCCAAAGTTCGTGTGGTCTCAGAGGAGTTTCTTAAGGATGTGAAA
TCTTCCAGCAAGGGCTTTCAGGAGCTTCTCTCTCTCCATGCAATTTCACCTTGGGGTGCA
GAGGTGAAAACGGAGCACCAGGAGGTGGCGGTGGATGGGAAGTGCAGCAAGCCTGCAAAT
ATGAAGAGTGCTGGGAAGGTCAAAGAAGAACAAGGACCTAGCAAGTCTGAGAAGAAAATG
AAGCTAACAGTTAAAGGTGGAGCAGCAGTAGATCCTGATTCCGGCTTGGAGGATTCTGCT
CATGTCTTTGAAAAAGGTGGGAAGATTTTCAGTGCAACTCTTGGCCTAGTGGATATTGTG
AAAGGAACAAATTCCTATTACAAACTGCAGCTGCTGGAGGATGACAGAGAGAGCAGGTAC
TGGGTGTTCAGATCCTGGGGTCGTGTGGGCACTGTCATTGGGAGTAACAAGCTGGAGCAG
ATGCCATCAAAAGAAGATGCTGTTGAACACTTCCTAAATTTGTACGAAGAGAAAACTGGC
AATTCTTGGCATTCAAAGAACTTCACTAAATATCCCAAAAAATTCTACCCACTGGAAATA
GATTATGGACAGGATGAAGAAGCTGTCAGGAAACTGACAGTGAGTGCTGGGACTAAATCG
AAGCTTGCTAAGCCAATCCAAGACCTTATTAAGATGATTTTTGATGTGGAGAGCATGAAG
AAAGCCATGGTGGAATTTGAGATTGACCTGCAGAAGATGCCACTGGGGAAACTGAGCAAG

CGACAGATCCAAAGTGCATACTCCATCCTTAACGAGGTTCAGCAGGCGGTTTCTGACGGT
GGTTCTGAATCCCAGATCTTGGACCTCTCAAACCGCTTCTATACTCTGATCCCTCATGAC
TTTGGGATGAAGAAACCACCTCTTCTCAGTAACTTGGAATACATTCAGGCTAAAGTGCAG
ATGTTGGACAACTTGCTCGATATTGAGGTTGCTTACAGTCTTCTCAGAGGTGGAAATGAA
GATGGAGACAAAGACCCAATTGATATCAATTATGAAAAGCTCCGAACTGATATTAAGGTG
GTTGACAAAGATTCAGAAGAAGCCAAGATTATTAAACAATACGTGAAAAATACTCATGCT
GCTACTCACAATGCATATGACCTCAAAGTTGTGGAAATCTTCAGGATTGAACGTGAAGGA
GAGAGTCAGCGTTACAAGCCATTTAAGCAGCTTCATAATCGCCAGCTGCTGTGGCACGGT
TCCCGCACCACCAACTTCGCTGGTATCCTCTCACAGGGTCTCCGGATAGCTCCCCCTGAA
GCTCCTGTGACCGGCTACATGTTTGGGAAGGGCATCTACTTTGCAGATATGGTTTCCAAG
AGTGCCAACTACTGTCACACATCTCAAGCTGATCCAATTGGGTTAATACTACTGGGAGAA
GTTGCCCTCGGAAATATGTATGAGCTAAAGAATGCTTCTCACATAACAAAATTGCCCAAG
GGAAAACATAGTGTGAAAGGCTTGGGCAAAACTGCACCTGATCCCACAGCCACTACAACC
CTTGATGGTGTAGAAGTTCCCTTAGGGAATGGGATCTCAACAGGAATTAATGATACTTGT
CTGCTGTACAATGAATATATTGTGTATGATGTTGCTCAGGTAAATCTGAAGTACCTGCTG
AAACTGAAATTCAACTATAAGACATCACTCTGG
>Chinese softshell turtle_PARP1
ATGGAGGAGGAGGATGGTGATGACAATGAGGAGGCAGAGGAGAAGGCCAGTCTGGACACC
ATGCCCACCAGCTGGGAGCTTGCTATCTGCATGGAGCCGGTATCCTCCTTCCAGGATGTC
TCCCAGGGCTTGGACAATTCCAGGGAAGGCACGTCAGACTCTGGGCTGGAGACACCTGTC
ATCAGCCTCCCGGAGGGTGTAGTTGAGGAGGAGGAAGTGCAGGAGGGGGTGGAGGTCAGC
ATCGACACCATGCCCGCCAGTCAGGAGTTCATCATGTCCCTGGAGCCATCCCCCTCCCAG
GATGTCACCCAAGGGTCAGACGAAGCAGGGGAAGGCCTATCAGAGGCTGGAGTTGTGGAA
GACACGGGCGTCATGTGGCTTCCCCAACCACCTGACATTGATGTCGGTGAACTGGCCTTG
GTGGTGCAAGATGGCTTATGGGACCATGGAAAAGTACCAGATGGTCCAGTTGATACAATC
AGAAGCTTGGTGGTCTGGGGCCCAGATGGGGATGTGGGTGCTGTCAATGGCCTCCCTGCA
TTTGGGGAAGCCTATGGCGGCAAAGCCAAAAACAATGGGGGCCATCTCGCCCCGCCCGAC
GAGCCCTCACCCATGTTTGATGGTAAAGTCCCCCACTGGCATCATTATGCTTGTTTCTGG
AAGCGGGCCCGACTTGTGTCTCATGCAGATGTTGATGGCTTCCCCGAGCTCAGATGGGAA
GACCAGGAGAAAATCAAAAAAGCAATTGAAACTGGAGGAGCTGGAGCAGGTAAAGGCGGT
GACCAGGATGGAGGTGGCAAGGCTGAGAAGAGCTTACATGATTTTGCTGCAGAATATGCC
AAGTCTAACAGAAGTACCTGCAAAGGTTGTGAACAGAAAATAGAAAAGGGCCAGATCAGA
ATTTCCAAGAAGATGGTGCATCCTGAAAAGCCCCAGCTGGGAATGATAGATAATTGGTAC
CATCTGGACTGTTTTGTGAGCCACCGAGCAGAATTGGGCTTTCTCCCTGCATTTGGTGCC
AGTCAGCTCCAGGGCTTTGGGATGTTGAAAGCAGAAGATAAAGAAGCTCTGAAGAAGCAA
CTGCCTGCTGTGAAGAGTGAAGGAAAGAGAAAAGGTGATGAAATGGATGGAAATGTGATC
TCAAAAAAGAAACAAAAAAAAGAAAAAGAGAAAGAATCCAAGCAGGAGAAGCTATTGAAG
GAGCAGACAGAGCTGATCTGGAGCATCAAAGATGAGCTGAAGAAAGTCTGTTCCACTAAC
GACCTGAAAGAGCTGCTGATAGCCAACAAACAAGAAGTGCCTTCTGGGGAATCTGCTATC
TTGGACCGAGTAGCAGATGGGATGGCATTTGGAGCTCTGCTTCCCTGTGAAGAGTGCAAG
GGACAGTTTGTGTTCAAGAGTGATGCATACTACTGCACAGGGGACATCACTGCTTGGACT
AAATGTGTTGCCAAAACGCAGATCCCCAACAGGAAGGAATGGATAATCCCAAAGGAATTC
CGGGAAATCTCCTATCTGAAGAAATTTAAGTGTAAAAAGCAGGACAGAGCATTCCCTCCA
GAGGCTGTGGCGGGGAACACTGCACTTCCAGCAACAGCTTCTGCTCCTTTGACAGAGAAA

GCATCTGTGCCATCAGATAAACCGTTATCGGGCATGAAGATTTTGACCCTTGGAAAATTA
TCCAGGAACAAAGAAGAAGTGAAGGCAATAATTGAGGGCCTGGGTGGAAAGGTGACAGGA
ACAGCAAACAAGGCTACCCTGTGCATCAGCACACAAAAGGAAGTTGAGAAAATGACCAAG
AAGATGGAAGAAGTGAAAGAAGCCAAAGTCCGAGTGGTCTCTGAGGAGTTCTTGCAGGAT
GTGAAATCCTCCAGCAAGGGCTTCCAGGAGCTCCTGTCTTTGCATGCAGTCTCCCCTTGG
GGTGCAGAGGTGAAGCATGAGCATACAGAGATGTCCCTGGGAGGAAAGTCCAGCAGGCTC
CCAAATATGAAGAGTGCTGGAAAGATCAAGGAAGAACAAGGAACTAGCAAGTCTGAAAAG
AAAATGAAGTTAACGGTTAAAGGAGGAGCAGCAGTGGATCCTGATTCAGGTTTGGAGGAC
TCTGCTCATGTCTTTGAAAAGAGTGGTAAAATCTTCAGTGCTACACTTGGCCTGGTAGAT
ATCATCAAAGGAACCAATTCCTATTACAAACTGCAGCTACTGGAGGATGACAGAGAAATC
AGGTACTGGGTTTTCAGATCTTGGGGTCGTGTTGGCACCGTAATTGGGAGTAACAAACTG
GAGCAGATGCCATCTAAAGATGATGCCATTGAGCATTTCTTGAATTTGTATGAAGAGAAA
ACAGGCAACTCCTGGCATTCCAAGAACTTCACTAAATATCCCAAAAAATTCTACCCTCTG
GAAATAGACTATGGACAGGATGAAGAAGCTGTGAGGAAATTGACAGTGAGTGCAGGGACC
AAGTCAAAGCTTCCAAAACCAGTCCAGGACCTTATTAAGATGATATTTGATGTGGAGAGC
ATGAAGAAAGCCATGGTGGAATTTGAGATTGACCTCCAGAAGATGCCCCTGGGAAAGCTG
AGCAAGAGGCAGATCCAGAGTGCATACNNNNNNNNNNNNNNNNNNNNAGGCAGTTTCT
GATGGTGGCAGTGATTCTCAGATACTGGATCTCTCCAATCGCTTCTATACACTGATACCC
CATGATTTTGGAATGAAGAAACCACCTCTCCTAAATAACCTAGAACATATCCAGGCCAAA
GTGCAGATGTTAGACAACCTGCTTGATATCGAGGTTGCTTACAGCCTGCTCAGAGGTGGA
AATGAAGATGGGGATAAAGACCCCATTGATGTCAACTACGAAAAGCTCAAAACTAACATT
AAGGTTCTTGATAAAGATTCCGAAGAAGCCAAGATCATAAAGCAGTATGTGAAGAATACA
CATGCCTCTACCCACAATGCATATGATCTGAAAGTTGTGGATATCTTCAAGATTGAACGT
GAAGGGGAAAGTCAGCGTTACAAACCATTCAAACAGCTTCATAATCGCCAACTGCTCTGG
CATGGCTCCCGCACTACCAACTTTGCTGGCATCTTCTCACAGGGTCTCCGAATAGCTCCA
CCTGAAGCACCTGTGACTGGTTACATGTTTGGTAAAGGTGTCTATTTTGCCGACATGGTG
TCCAAGAGTGCCAACTACTGTCACACATCTCAGACTGACCCAATAGGCTTAATCTTACTG
GGAGAGGTTGCCCTTGGAAACATGCATGAACTAAAGAATGCTTCTCACATATCTAAGTTG
CCCAAGGGAAAACACAGTGTCAAAGGTTTAGGCAAGACTGCACCTGATCCTTCAGCCACT
ATCACTCTAGATGGTGTGGATATTCCTTTAGGGAATGGAATTCCATCTGGAATTAGTGAT
ACCTGTCTTCTATATAATGAATACATTGTCTATGATGTTGCTCAGGTAAATCTGAAGTAT
CTACTGAAACTGAAGTTCAACTACAAGACATCACTCTGG
>Cod_PARP1
GGAGACTCAAACGAAGACAAGCTTTACAAAGCCGAATATGCTAAAAGTGGCCGTGCGTCG
TGCAAGAAATGCAAGGAGAACATTGCTAAAGATTCGCTCCGAATGGCCATCATGGTGCAG
TCGCCCATGTTTGACGGGAAGGTCCCCCACTGGCACCACTTCTCCTGCTTCTGGCAGCGG
GCAGCGGCCCAGTCCACCGCGGACATCGCTGGCTTCTCGGACCTCCGCTGGGAGGACCAG
GAGAAAGTCAAAAAGGCCATCGAGACCGGAGGAGTGACCGGAGGCAAAGCAGACCAAAAA
GGCGGAGCCAAAGGAGAGAAGACGCTCAATGACTTTGCTGTGGAATATGCCAAGTCTAAC
CGCAGCACCTGCAAAGGCTGCGAGCAGAAAATAGAAAAGGATCAGATCCGCGTGTCCAAG
AAGTCAGTGGATGCGGAGAAGCCCCAGCTGGGTCTGATCGACCGCTGGTACCACACAGCC
TGCTTCGTCAGCCGCCGCGAGGAGCTGGTGTTCAAGTCCGAGTACAGCGGCGCCCAGCTC
AAGGGCTTCAACGGACTGCGGGCTGAGGACAAGGAGGACCTGAAGAAGAGGCTGCCCGCG
GTGAAGTCTGAAGGAAAGAGAAACGGTGACGAGTTGGACGGGGTGTCAAAGAAAGTGAAG

AAGGAGAAAGAAGACGAAGATAAAAAGCTTGAGGACCAACTAAAGGTGCAGAGTCAGCTG
ATATGGGGCATCAAGGACAAGCTGAAGAAGAACTGCTCCTCCAACGACATGAAGGAGCTG
CTGATTGCAAACTCCCAGGAGGTGCCATCTGGAGAGTCCAACCTGGTGGACTGCCTGGCG
GACGGCATGGCGTTCGGCTCCCTCCAGCCCTGTGCGGAGTGCAAGGGGCAGCTGGTGTTC
AAGGGCAACGCCTACTACTGCAGCGGGGACATCTCTGCCTGGACCAAGTGTGTGTTCACC
ACCAAGTCGCCCGTCCGCACAGACTGGGTCATTCCAAAGGAATTCCACGAGGTTCCCTTC
CTCAAGAAGTTCAAGTGTAAGAAACAGGACCGGATCTTCCCCAAGGTTGAGCAACGCCAC
CCTGTCGTCGCCACCGCTGCCTCCAGTGGGTCGACTAAACCTTTCCCAGAAGGGGCCCCA
GCTGGCAAGCCTCTGACGGGCATGAAGCTGCTTGCTGTGGGTAAACTGAAGAAGAACAAG
GATGAGATCAAGGCTGTCGTGGAGGAGATGGGCGGGAAGATAACCCCCTCAGCCAATAAG
GCGGACCTCTGCCTCAGCAACGCAAAGGAGCTGGAGAAGATGACCAAGAAGATGGAGGAG
GTGAAGGAGGCCGGGGTGCGAGTGGTCGCCGAGGAGTTCCTCACGGACGTCAAGGCGTCG
GGCAAGTCTCTCCAGGAGCTGGTCTCCGTGCACGCCATCTCTCCCTGGGGGGCGGAGGTC
AAGGTGGAGGTCAAAGTGGAGCCCAAGGCTGCTGCCGTGCCCTCAAAGTCCGGGGCCATG
GCCGCCAAGAGCACCGGCCGCGTGAAGGAGGAGGAAGGGGGCAGCAAGTCTAAGAAGATG
AAGCTAACTGTGAAAGGAGGAGCTGCCGTGGACCCTGATTCGGGCCTGGAGAACAGCGCC
CATGTCCTGGAGCAGAGTGGGAAGATGTACAGCGCCACCCTGGGTCTGGTGGACATCGTC
CGCGGAACCAACTCCTACTACAAGCTGCAGCTCCTGGAGGACGACGTCCAGAAGCGGTAC
TGGGTGTTCAGGTCCTGGGGCCGCGTGGGCACCACCATCGGGGGCCACAAGCTGGACAAG
TTTAGTGACAAGCTCGCAGCCATGGACAACTTCCTGGGCGTCTACACGGATAAGACTGGC
AACACCTGGAACTGCACCAACTTCACCAAGTATCCCAATAAGTTCTACCCGCTGGAGATC
GACTACGGACAGGACGAGGAGGCTGTGAAGAGGCTCACGGAGAGTGCTGGCACCAAGTCT
GAGCTGGCCAAGCCCGTCCAGGAGCTCATCAGGATGATCTTTGACGTGGAGAGCATGAAG
AAGGCCATGGTGGAGTTTGAGATTGACCTCCAGAAGATGCCCCTGGGAAAGCTGAGTAAG
AGGCAGATCCAGAGCGCCTACGCCCTCCTCAGTGAGGTGCAGCAGGCTGTGACAGACAGT
TCGGCCGAGTCCCAGATCCTAGACCTGTCCAACCGCTTCTACACGCTCATCCCCCACGAC
TTCGGCATGAAGAAACCCCCTCTGCTGAGCAACCTGGACTACGTTCAGTCCAAGGTCCAG
ATGTTGGACAACCTGTTGGACATTGAGGTGGCCTACAGCCTGCTGAGAGGAGGGGCAGAG
GACAACGGGAAGGATCCCATCGATATCAACTATGAGAAACTCAAAACCAAGATTGAAGTG
GTTGACAAGAACAGCGAGGAGGCCGAGATCATTATGCAATATGTCAAGAACACACACGCT
GCTACACACAACACCTACACACTGGAAGTAGACGAGATCTTCAAAATAGTTCGCGAGGGC
GAGTACCAAAGGTTCCGGCCCTTCAAGGACCTGCACAACCGGCAGCTGCTGTGGCACGGC
TCGCGCACCACCAACTACGCCGGCATCCTGTCTCAGGGTCTGCGCATCGCCCCCCCAGAG
GCCCCTGTGACTGGTTACATGTTCGGCAAGGGTGTGTACTTTGCCGACATGGTGTCCAAG
AGTGCAAACTACTGCCACGTGTCACAGTTGGATCCCGTAGGCCTTTTACTTCTGGGAGAG
GTCGCCCTTGGCAACGTGCATGAACTGAAGAAGGCTGCGCACATTACCAAACTACCTAAG
GGAAAACACAGTGTTAAAGGTGTGGGCAGAACCGCCCCTGATCCCGGTTCCACTGCCACT
CTAGATGGGGTGCAGGTGCCGCTAGGAAAGGGCTGCAATACCAACATCGACGACACCAGT
CTTCTCTACAACGAGTACATCGTTTACGACGTTGCACAGGTGAACCTGAAGTACCTGCTG
AAGACCAAGTTCAACTACCAGACGTCTCTTTGG
>Cow_PARP1
ATGGCGGAGTCTTCAGACAAGCTCTACCGGGTCGAGTACGCCAAGAGCGGGCGCGCGTCT
TGCAAGAAATGCAAAGAGAGCATCCCCAAGGACTCGATCCGGATGGCCTTCATGGTGCAG
TCGCCCATGTTCGATGGGAAAATCCCGCACTGGTACCACCTCTCCTGCTTCTGGAAGGTC

GGCTTCTCCATCTGGCACCCTGATGTCGAGGTGGAGGGGTTCTCTGAGCTCCGCTGGGAT
GACCAGCAGACGATCAAGAAGATGGCCGAGACTGGCGGAGCGACAGATGTTTCAGGCAAA
GGCCAAGATGGAGTTGGCAGCAAGACCGAGAAGACGCTGATTGACTTCGGGGCAGGGTAC
GCCAAGTCCAACAGAAGCACGTGCAAGAGCTGCATGGAGAAGATAGACAAGGGCCAGGTG
CGCCTGTCTAAGAAGGTGGTGTACCCCGATAAGCCCCAGCTGGGCATGGTTGACTGCTGG
TACCACCCAAAGTGTTTTGTTCAGAAACGGGAGGAGCTGGGCTTCCGTCCCGAGTTCAGC
GCAAGCCAGCTCATGGGCTTCAGCGTCCTCACCGCAGAGGACCAAGAAACCCTCAAGAAG
CAGCTCCCGGCCATCAAGGGTGAAAGAAAGAGAAAAGGTGATGAGGTGGATGGAATAGAT
GAAGTGACCAAGAAGAAATCTAAAAAAGAAAAAGACAAGGAGATTAAACTTGAAAAGGCC
CTTAAGGCCCAGAACGACCTGATCTGGAATGTCAAGGACGAGCTAAAGAAAGCGTGCTCT
ACGAACGACCTGAAAGAACTGCTCATCTTCAACAAGCAGGAAGTGCCCTCCGGGGAGTCG
GCGATCTTGGACCGCGTGGCCGACGGTATGGTGTTTGGCGCCCTCCTTCCCTGCGAGGAA
TGCTCGGGCCAGCTGGTCTTCAAGGGCGACGCCTATTACTGTACCGGGGATGTGACTGCC
TGGACCAAGTGTATGGTCAAGACACAGACGCCCAACCGGAAGGAGTGGGTGACCCCAAAG
GAATTCCGAGAAATCTCTTACTTCAAGAAACTGAAGATCAAAAAGCAGGACCGTATATTC
CCCCCAGAGAGCAGCACCCCGGTGGGGGCAGCAGCCCCACCCTCTGCAGCTTCAGCGCCT
GCCGCTGTGCACTCTGGCCCCCCAGACAAGCCATTATCCAACATGAAGATCCTGACTCTC
GGGAAACTCTCCCAGAACAAGGATGAAGTGAAGGCCACGATTGAGAAACTCGGGGGGAAA
TTGACAGGGACGGCCAACAAGGCCTCCCTGTGTATCAGCACCAAAAAGGAGGTGGACAAG
ATGAATAAAAAGATGGAGGAAGTAAAAGAAGCCAACATCCGTGTCGTGTCTGAGGACTTC
CTCCAAGACATCTCCGCCTCCACCAAGAGCCTTCAGGAGTTGCTCTCCACCCACCTCTTG
TCCCCCTGGGGAGCCGAGGTGAAGGTGGAGCCTGTTGAAGCAGTGGCCCCAAAGGGGAAG
TCGGGGGCCGCGCCCTCCAAGAAGAGCAAGGGTCCCGTCAAGGAGGAAGGTACCAACAAA
TCTGAAAAGAGGATGAAATTAACTCTTAAAGGAGGAGCAGCTGTCGACCCTGATTCAGGT
CTGGAACACAATGCACACGTCCTCGAGAAAGGCGGGAAGGTCTTCAGCGCCACCCTCGGG
CTTGTGGACATCGTCAAAGGGACCAACTCCTATTACAAGCTGCAGCTCCTGGAGGATGAC
AAAGAGAGCAGGTACTGGATATTCAGGTCCTGGGGCCGTGTGGGCACGGTGATTGGTAGT
AACAAACTGGAGCAGATGCCATCCAAGGAGGATGCCATTGAGCATTTTATGAAATTATAT
GAAGAGAAAACCGGAAACGCCTGGCACTCTAAAAACTTTACGAAGCATCCCAAAAAGTTC
TACCCTCTGGAGATTGACTATGGCCAGGATGAAGAGGCGGTGAAGAAGTTGACAGTAAAC
CCTGGCACCAAGTCCAAGCTCCCCAAGCCAGTGCAGAACCTCATTAAGATGATCTTTGAT
GTAGAGAGTATGAAGAAAGCCATGGTGGAGTATGAGATTGACCTTCAGAAGATGCCCTTG
GGGAAGCTGAGCAAAAGGCAGATCCAGGCTGCATACTCCATCCTCAGTGAGGTCCAGCAG
GCACTGTCCCAGGGCAGCAGTGACTCTCACATCCTGGATCTCTCCAACCGCTTCTACACC
CTGATCCCCCACGACTTCGGGATGAAGAAGCCCCCGCTGCTGAACAACGCAAACAGCGTG
CAGGCCAAGGTGGAAATGCTAGACAACCTGCTGGATATTGAGGTGGCCTACAGTCTACTC
AGGGGTGGTTCTGATGACAGCAGCAAGGACCCCATTGATGTCAACTATGAGAAGCTCAAA
ACTGACATTAAGGTTGTGGACAAAGATTCCGAAGAAGCCGAGATCATTAGGAAGTATGTG
AAGAACACTCACGCGACCACACACAACGCGTACGACTTAGAAGTCGTCGACATCTTCAAG
ATAGAGCGCGAAGGGGAAAGCCAGCGTTACAAGCCGTTTAAGCAGCTGCATAACCGGAGG
CTGCTGTGGCACGGGTCCAGGACCACCAACTTCGCGGGCATCCTGTCCCAGGGTCTCCGG
ATAGCCCCACCTGAAGCACCTGTGACGGGCTACATGTTTGGTAAGGGAATCTATTTCGCC
GACATGGTCTCCAAGAGTGCCAACTACTGCCACACGTCCCAGGGAGACCCAATAGGCTTG
ATCCTGTTGGGAGAAGTTGCCCTTGGAAACATGTATGAACTGAAACATGCTTCGCATATC

AGCAAGTTACCCAAGGGCAAGCACAGTGTCAAAGGTTTAGGCAAAACTACCCCTGACCCA
TCGGCTAGTATTACTGTGGATGGTGTGGAGGTGCCTCTCGGGACAGGGATTTCATCTGGT
GTTAATGACACCTGTCTGCTGTATAACGAGTACATCGTCTACGACATTGCTCAGGTCCAC
CTGAAGTACCTGCTGAAGCTGAAGTTCAACTTTAAGACATCCCTGTGG
>Dog_PARP1
ATGCTCAAGGCCAGTCCAACTTTCTTCTCTATGCTGGCGTGCTTCAGCAGCTTCATTGAT
CCTCCCGCCCGCGAGAGCCGGGGAGGGCGGCCCGGAGCGGTGCTGCGGGGTGGGGGGCTC
CTCCTGCCTGTCGCCGGCCGAAGTCTGAGGACGAGGCGAGCCTGGGCTTCTGAAGCCCGC
CGCTTCTGCCCTCCCTCGTGCTGGAAGTCACCCATGTTCGATGGAAAAGTCCCACACTGG
TACCACTTTTCCTGCTTCTGGAAGGTCGGCCACTCCATCCGGCACCCTGACGTGGAGGTG
GACGGGTTCTCTGAACTCAGGTGGGATGACCAGCAGAAAGTCAAAAAGACTGCGGAGGCT
GGAGGCGTGACAGGCAAAGGCCAGGATGGAGGAGGTGGCAAGACAGACAAGACACTGGCT
GACTTCGCAGCTGAATATGCCAAGTCCAACAGGAGCACGTGCAAGGGCTGCCTGGAGAAG
ATAGAAAAGGGCCAAATACGCCTGTCCAAGAAGATGCTGGATCCAGAGAAGCCCCAACTG
GGCATGATTGACCGCTGGTACCACCCGAACTGCTTTGTTAAGAACAGGGAGGAGCTGGGG
TTCCGGCCCGAGTACAGCGCCAGTCAGTTCAAGGGCTTTGGCCTCCTCACTCCAGAAGAT
AAAGAAGCCCTGAAGAAGCAGCTCCCAGGAGTCAAGAGTGAAGGGAAGAGGAAAGGAGAT
GAGGTGGATGGGATGGATGAAGTGGCCAAGAAGAAATCTAAAAAAGAAAAGGATAAGGAC
AGTAGGCTTGAGAAGGCCCTGAAGGCCCAGAACGAGCTGATCTGGAACATCAAGGACGAG
CTAAAGAAAGTGTGTTCTACAAATGACCTGAAAGAGCTGCTCATCTTCAACAAGCAGCAA
GTGCCATCTGGGGAGTCAGCGATCTTGGACCGAGTGGCTGATGGCATGGTGTTCGGTGCC
CTCCTTCCCTGTGAGGAATGCTCAGGCCAGCTGGTCTTCAAGAGTGATGCCTACTACTGT
ACCGGGGATGTCACTGCCTGGACCAAGTGTATGGTCAAGACACAGACACCCAGCCGGAAG
GAGTGGGTGACCCCGAAGGAATTCCGAGAAATCTCTTATCTCAAGAAATTGAAGATCAAA
AAGCAGGACCGGATATTTCCCCCTGAGACCAGTGCCCCAGTGGCAGCGGCACCTCCGCCC
TCCACTGCCTTGGCGCCTGCTAGTGTAAACAGCTCAACTCCGCCAGGTAAGCCATTGTCC
AACATGAAGATCCTGACTCTTGGGAAACTCTCGCGGAACAAGGATGAAGTGAAGGCCATG
ATTGAGAAGCTCGGGGGAAAGTTGACAGGCACAGCCAATAAGGCCTCCCTGTGCATCAGT
ACCAAAAAGGAGGTGGAGAAGATGAACAAGAAGATGGAGGAGGTGAGAGAGGCCAGCATC
CGAGTGGTATCTGAGGATTTTCTCCGGGACGTGTCTGATTCCACCAGGAGCCTTCAGGAT
TTGCTCTCCGCCCATGTGCTGGCTCCCTGGGGGGCTGAGGTGAAGGCAGAGCCAGCAGAG
CCTGCAGCCCCTAAAGCCAAATCAGGAGCTGCGCTCTCCAAGAAGAGCAAGGGCCCGGTC
AAGGAGGAAGGCGTCAACAAATCTGAGAAGAGAATGAAATTAACTCTGAAAGGTGGTGCT
GCTGTTGATCCAGATTCAGGTCTGGAACACTCTGCACATGTTCTGGAGAAAGGTGGTAAA
GTGTTCAGCGCCACCCTAGGCCTGGTAGACATCGTGAAGGGAACCAACTCCTACTACAAG
CTGCAGCTCCTGGAGGATGACAAAGAAAGCAGGTACTGGATCTTTCGGTCCTGGGGCCGT
GTGGGCACCGTGATTGGCAGCAACAAACTGGAGCAGGTGCCGTCCAAGGAGGAAGCCATC
GAGCACTTTATGAAATTGTATGAAGAGAAAACTGGGAATGCCTGGCACGCCAAGAACTTC
ACGAAGCATCCCAAAAAATTCTACCCTCTGGAGATTGACTATGGCCAGGACGAAGAGGCG
GTGAAGAAGCTGACAGTGAAGCCTGGCACCAAGTCCAAGCTCCCCAAGCCCGTACAGGAC
CTCATTAAGATGATCTTTGACGTGGAAAGTATGAAGAAAGCCATGGTGGAATATGAGATT
GACCTTCAGAAGATGCCCTTGGGGAAGCTGAGCAAACGGCAGATTCAGGCCGCGTACTCC
ATCCTCAGTGAAGTGCAGCAGGCAGTGTCCCAGGGCAGCAGTGACTCCCAGATCCTGGAT
CTCTCGAATCGCTTCTACACCCTGATCCCCCACGACTTTGGGATGAAGAAGCCCCCACTC

CTGAACAACGCGGATTGTGTGCAGGCCAAGGTGGAAATGCTGGACAACCTACTGGACATC
GAGGTGGCCTACAGTTTGCTCAGGGGTGGCTCTGATGACAGCAGCAAGGACCCCATCGAC
GTCAACTATGAGAAGCTCAAGACTGACATTAAGGTGGTGGACAGAGATTCTGAGGAAGCC
GAGACCATCAGGAAGTACGTTAAGAACACTCACGCGACCACGCACAACGCATATGACCTG
GAGGTTGTTGATATCTTTAAGATTGAGCGTGAAGGCGAGAGCCAACGCTACAAGCCCTTC
AAGCAGCTGCATAACCGGAGGTTGCTGTGGCACGGGTCCAGGACCACCAACTTCGCGGGG
ATCCTGTCCCAGGGCCTCCGGATAGCCCCACCTGAGGCCCCTGTGACAGGCTACATGTTT
GGTAAAGGGATCTATTTCGCCGACATGGTCTCCAAGAGTGCAAACTACTGCCACACATCC
CAGGGAGACCCCATAGGCCTGATCCTGCTGGGAGAAGTTGCCCTTGGGAACATGTACGAA
CTAAAGCACGCTTCACATATCAGCAAGCTACCCAAGGGCAAGCACAGTGTCAAAGGTTTG
GGCAAGACGACTCCTGACCCTTCCGCTAGTATTACTCTGGATGGCGTAGAAGTCCCTCTT
GGAACTGGGATTTCCTCTGGCGTCAACGACACCTGCCTGCTGTATAACGAGTACATTGTC
TATGACATCGCTCAGGTCAACCTGAAATACCTGCTGAAGCTCAAGTTCAACTTCAAGACG
TCCCTGTGG
>Dolphin_PARP1
ATGGCGGAGTCATCGGACAAGCTCTACCGGGTCGAGTACGCCAAGAGCGGGCGCGCCTCT
TGCAAGAAATGCAGTGAGAGCATCCCCAAGGACTCGCTCCGGATGGCCATCATGGTGCAG
TCGCCCATGTTCGACGGAAAAGTCCCGCACTGGTACCACTTCTCCTGCTTCTGGAAGGTC
GGCCACTCCATACGGCACCCTGACGTCGAGGTGGATGGGTTCTCCGAGCTCCGCTGGGAT
GACCAGCAGAAGGTCAAGAAGACGGCTGAGGCTGGAGGAGTGACGGGCAAAGGCCAAGAT
GGAGTTGGCAGCAAGACGGAGAAGACACTGGGCGACTTTGCGGCAGAGTACGCCAAGTCC
AACAGAAGCACATGCAAGGGCTGCATGGAGAAGATAGAAAAGGGCCAGGTGCGCCTGTCC
AAGAAGATGCTGGACCCCGAGAAGCCCCAGCTAGGCATGATCGACCGCTGGTACCACCCG
AACTGCTTTGTTGAGAACAGAGAGGAGCTGGGCTTCCGGCCTGAGTACAGCGCGAGCCAG
CTCAAGGGCTTCAGCCTCCTCGCCGCAGAGGACAAGGAAGCCCTGAAGAGGCAGCTCCCG
GCCATCAAGAGTGAAGGAAAGCGAAAAGGCGATGAGGTGGATGGAATGGAGGAAGTGGCC
AAGAAGAAATCTAGAAAAGAAAAAGACAAGGAGAGTAAGCTTGAAAAGGCCCTCAAGGCC
CAGAACAACCTGATCTGGAACATCAAGGACGAGCTAAAGAAAGCATGTTCGACGAATGAC
CTGAAAGAGCTGCTCATATTCAACAAGCAACAAGTGCCCTCCGGGGAGTCTGCGATCTTG
GACCGAGTGGCCGATGGCATGGTGTTCGGTGCCCTCCTTCCCTGCGAGGAATGCTCAGGC
CAGCTGGTCTTCAAGAGCGATGCCTATTACTGTACCGGGGATGTCACTGCCTGGACCAAG
TGTATGGTCAAGACACAGACGCCCAACCGGAAGGAGTGGGTGACCCCAAAGGAATTCCGA
GAAATCTCTTACCTCAAGAAATTGAAGATCCGAAAGCAGGACCGTATATTCCCCCCAGAG
ACCAGCGCCCCGCTGGCGGCAGCACCCCTGCCCTCTGCAGCCTCAGCGCCCGCCACTGTG
AACTCCGCTCCCCCAGACAAGCCGTTATCCAACATGAAGATCCTGACTCTTGGGAAACTC
TCCCGGAACAAGGATGAAGTGAAGGCCACAATCGAGAAACTCGGGGGGAAGTTGACAGGG
ACCGTCAACAAGGCCTCCCTGTGCATCAGTAGCAAAAAGGAGGTCGACAAGATGAATAAA
AAGATGGAGGAAGTAAAAGAAGCTAACGTCCGCGTCGTGTCTGAGGATTTCCTCCAGGAC
ATCTCCACCTCGACCAAGAGCCTTCAGGAGTTGCTCTCAACCCACCTCTTGTCCCCCTGG
GGGGCTGAGGTGAAGGCAGAGCCTGTTGAAGCTGTGGCCCCAAAGGGGAAGTCGGGGGCT
GTGCTCTCCAAGAAGAGCAAGGGCCCCGTCAAGGAGGAAGGTACCAACAAATCTGAAAAG
AGAATGAAATTAACTCTTAAAGGAGGAGCAGCNNNNNNNNNNNNNNNNNNNNNNNNNNNN
NNNNNNNNNNNNNNNNNNNNNNNNNNNNNNNNNNNNNNNNNNNNNNNNNNNNNNNNNNNN
NNNNNNNNNNNNNNNNNNNNNNNNNNNNNNNNNNNNNNNNNNNNNNNNNNNNNNNNNNNN

NNGTATTGGATATTCAGGTCCTGGGGCCGCGTGGGCACGGTAATTGGTAGTAACAAACTG
GAGCAGATGCCATCCAAGGAGGAGGCCATTGAGCATTTTACGAAATTATATGAAGAGAAA
ACCGGGAATGCCTGGCACTCCAAAAACTTCACAAAACACCCCAAAAAGTTCTACCCTCTG
GAGATTGACTATGGCCAGGATGAAGAGGCAGTAAAGAAGCTGACGGTAAACCCTGGCACC
AAGTCCAAGCTCCCCAAGCCAGTGCAGGACCTCATTAAGATGATCTTTGATGTGGAAAGT
ATGAAGAAAGCCATGGTGGAGTATGAGATTGACCTTCAGAAGATGCCCTTGGGGAAGCTG
AGCAAAAGGCAGATCCAGGCTGCGTACTCCATCCTTAGTGAGGTCCAGCAGGTACTGTCC
CAGGGCAGCAGCGACTCCCAGATCCTGGACCTCTCAAATCGCTTCTACACCCTGATCCCC
CACGACTTCGGGATGAAGAAGCCTCCGCTCCTGAGCAACTCAGACAGCGTGCAGGCCAAG
GTGGAAATGCTCGACAACCTGCTGGACATCGAGGTGGCCTACAGTCTGCTCAGGGGTGGT
TCTGATGACAGCAGCAAGGACCCCATCGATGTCAACTATGAGAAGCTCAGAACTGACATT
ATGGTGGTGGACAAAGATTCTGAAGAGGCTGAGATCATTAGGAAGTATGTTAAGAACACT
CACGCGACCACACACAACGCATATGACTTGGAAGTCGTGGATATTCTTAGAATAGAGCGT
GAAGAGAGCCAGCGTTACAAGCCGTTCAAGCAGCTGCATAACCGAAGGTTGCTGTGGCAC
GGGTCCAGGACCACCAACTTCGCCGGGATCCTGTCCCAGAGCCTCCGGATAGCCCCAGAA
GCACCGGTGACGGGCTACATGTTTGGTAAGGGGATCTATTTCGCCGACATGGTCTCTAAG
AGTGCCAACTACTGCCACACATCCCAGGGAGACCCAATAGGCTTAATCCTGTTGGGAGAA
GTTGCCCTTGGAAACATGTATGAACTGAAGCATGCTTCGCATATCAGCAAGTTACCCAAG
GGCAAGCCCAGTGTCAAAGGTTTAGGCAAAACTACCCCTGACCCTTCAGCTAGTATTACT
ATGGATGGTGTAGAGGTTCCTCTTGGGACCGGGATTTCATCTGGTGTTAACGACACCTGT
CTACTGTATAACGAGTACATTGTCTATGATATTGCTCAGGTCAGTCTGAAGTATCTGCTG
AAGCTGAAGTTCAACTTCAAGACCTCCCTGTGG
>Duck_PARP1
ATGGTACATCCTGAAAAACCCCAGCTGGGAATGATTGATAACTGGTACCACCCAGACTGC
TTTGTGAGCCGCCGAGCGGAGCTGGGTTTCCTCCCAGCGTACGGGGCCGCCCAGCTCCTT
GGCTTCAGCATCCTGAAAGCTGAAGATAAAGAAACTCTGAAGAAGCAGCTCCCAGCTACC
AAAAGCGAAGGGAAGAGAAAAGGAGAGGAGGTAGATGGAAATGCGACTGTGAAAAAGAAG
CAGAAAAAAGAGAAAGAGAAGGAATCAAAGCAGGAAAAACAGCTGAAGGAACAGACAGAG
CTGATCTGGGGCATCAAGGACGAGCTGAGGAAGGTCTGCTCCACTAATGACCTGAAAGAG
CTGCTGATTGCCAACAAGCAGGAAGTGCCTTCAGGGGAGAACGCCATCTTGGACCGAGTG
GCAGATGGGATGGCATTTGGAGCTCTGCTTCCCTGTGAGGAGTGTAAAGGGCAGTTTGTG
TTCAAGAGCGATGCATATTACTGTTCAGGGGACATCACTGCCTGGACTAAGTGCGTTGCT
AAAACACAGACTCCCAACAGGAAAGACTGGGTAATCCCGAAGGAGTTTCGTGAAATTCCT
TACCTCAAGAAATTTAAGTGTAAGAAACAGGATAGGATATTCCCTCCAGAGGCTGCAACT
GTGAACTCTGCGCTTCCTCCACATGCATCTGCTCCTTTGACAGAGACCGTTTCTGCACCC
CAAGACAAACCACTGGCCAACATGAAGATCCTGGCTCTTGGAAAGCTGTCCAAGAACAAG
GAGGAAGTGAAGAACATCGTGGAGGAGCTGGGAGGAAAGATGACGACGACAGCTAACAAG
GCCACGCTGTGCATCAGCACACAGAAGGATTTGGAGAAAATGAGCAAGAAGATGGAAGAA
GTGAAGGAGGCCAAAGTCCGTGTGGTCTCAGAGGCATTTCTTCAGNNGCTGAAATCCTCC
AGCAAGAGCTTTCAGGAGCTTCTTTCTGTCCATGCAATTTCACCTTGGGGTGCAGAGATA
AAAATGGAGCACCATGAGGTGGCCGTGGATGGGAAATGCAGCAAGCCCCAGCATACGAAG
AGTGCTGGGAAGGTCAAAGAAGAACAAGGACCTAGCAAGTCTGAAAAGAAAATGAAGCTA
ACAGTTAAAGGTGGAGCAGCAGTAGATCCTGATTCTGGTTTGGAGGATTCTGCTCATGTG
TTTGAAAAAGGTGGAAAGATCTTCAGTGCAACTCTTGGTCTAGTAGATATTGTGAAAGGA

ACGAATTCCTATTACAAACTGCAGCTCCTAGAGGATGATAGAGAGAGCAGGTACTGGGTG
TTTAGATCCTGGGGTCGTGTGGGCACTGTAATTGGGAGTAACAAGCTGGAGCAGATGCCA
TCAAAAGAAGATGCTGTTGAACACTTCCTTAACTTGTATGAAGAGAAAACCGGCAATTCG
TGGCATTCGAAGAACTTCACTAAATATCCCAAAAAGTTCTACCCACTGGAAATAGATTAT
GGACAGGATGAAGAAGCTGTCAAGAAACTCACAGTGGGTGCTGGGACTAAATCAAAACTT
GCTAAGCCAATCCAAGACCTTATTAAGATGATCTTTGATGTGGAGAGCATGAAGAAAGCA
ATGGTGGAATTTGAGATTGACCTGCAGAAGATGCCACTGGGAAAACTGAGCAAGCGACAG
ATCCAGAGTGCATACTCCATCCTTAATGAGGTTCAGCAGGCAGTTTCTGACAATGGTTCT
GAATCTCAGATCTTGGATCTCTCCAACCGCTTCTATACTCTGATTCCTCATGACTTCGGA
ATGAAGAAACCACCTCTCCTAAATAACTTAGAATACATTCAGGCTAAAGTGCAGATGTTG
GACAACTTGCTTGATATTGAGGTTGCTTACAGCCTTCTAAGAGGTGGGAATGAAGATGGA
GATAAAGACCCAATTGACATCAACTACGAAAAGCTCCGAACTGATATTAAGGTTGTTGAC
AAAGATTCAGAGGAAGCCAAGATTATTAAACAATATGTGAAAAATACCCATGCTGCTACT
CACAATGCATATGACCTCAAAGTTGTGGATATCTTCAGGATTGAGCGTGAAGGAGAGAGT
CAGCGCTACAAGCCCTTTAAGCAGCTTCATAACCGCCAGCTGCTGTGGCATGGCTCCCGC
ACCACCAACTTTGCTGGTATCCTCTCGCAGGGTCTCCGGATAGCTCCCCCTGAAGCTCCT
GTGACTGGCTACATGTTTGGGAAAGGCATCTATTTTGCAGACATGGTGTCCAAGAGTGCT
AACTACTGTCACACATCTCAAGCTGATCCCATCGGGTTAATACTACTGGGAGAAGTTGCA
CTTGGAAACATGTATGAGCTAAAGAATGCTTCTCACATAACAAAATTGCCCAAGGGAAAA
CACAGTGTGAAAGGCTTGGGCAAAACTGCACCTGATCCCACAGCTACTACCAGCCTTGAT
GGTGTGGAGGTTCCCTTAGGGAATGGAATCTCAACAGGAATTAATGATACCTGTCTTCTA
TATAACGAATACATTGTGTATGATGTTGCTCAGGTAAATCTGAAGTACCTGCTGAAACTG
AAATTCAACTATAAGACATCACTCTGG
>Flycatcher_PARP1
ATGAAATCACCCATGTTTGATGGCAAAGTCCCTCACTGGCACCACTACAGCTGCTTCTGG
AAGCGGGCTCGAATTGTGTCCCACACAGACATTGATGGCTTCTCTGAGCTTCGGTGGGAA
GATCAGGAGAAAATCAAGAAAGCCATTGAAACTGGAGGCCCTGGAGGAGGAAAAGGAGGG
GAGCAGGAAGGAGGTGGTAAGGCTGAGAAGAGCCTAAATGACTTTGCTGCAGAATATGCC
AAGTCTAACAGAAGTACTTGCAAAGGCTGTGAGCAGAAAATAGAAAAGGGCCAGATCCGG
ATTTCCAAGAAGATGGTGCATCCTGAGAAGCCACAGCTGGGAATGATAGATAACTGGTAC
CACCCGGAGTGCTTTGTGAGCCGCCGAGCAGAGCTGGGCTTCCTCCCGGCCTACGGGGCC
ACCCAGCTCCTGGGCTTCAGCATCCTGAAAGCTGAGGATAAAGAAACTCTGAAAAAGCAG
CTCCCAGCTACCAAAAGTGAAGGAAAGAGAAAAGGAGAGGAGGTAGATGGAAATGCGACA
GCGAAAAAGAAGCCGAAAAAAGAAAAAGAGAAAGAATCAAAGCATCAAAAACAGCTGAAG
GAGCAGACAGAGCTGATCTGGGGCATCAAGGATGAGCTGAGGAAGGTCTGCTCCACCAAT
GACCTGAAAGAGCTGCTGATTGCCAACAAGCAGGAGGTCCCCTCAGGGGAGAATGCCATC
TTGGACCGAGTAGCAGATGGGATGGCATTTGGAGCTCTGCTGCCCTGCCAGGAGTGCAAG
GGGCAGTTTGTGTTCAAGAGTGACGCTTATTACTGTTCAGGGGATATCACTGCCTGGACC
AAGTGCGTGGCTAAAACACAGACTCCCAACAGGAAAGACTGGATAATCCCAAAGGAGTTC
CGGGAAATTCCTTACCTGAAGAAATTCAAATGTAAGAAGCAGGACAGAGTGTTCCCTCCA
GATGCTGCAACTGTGAACTCGGCCCCTCCTCCTTCTGCATCTGCTCCTTTGTCAGAGACT
GTGTCTGCACCCAGAGACAAACCACTGACCAACATGAAGATCCTGGTTGTCGGGAAGCTG
TCAAAGAACAAAGAGGAGGTGAAAAACATTGTGGAGGACCTGGGAGGAAAGATGACAACA
ACAGCTAACAAGGCCACCCTGTGCATCAGCACCCAGAAGGATGTGGAGAAAATGAGCAAG

AAGATGGAAGAAGTGAAGGAGGCCAAAGTCCGTGTGGTCTCAGAGGCATTTCTTCAGGAT
GTGAAATCTTCCAGCAAGGACTTCCAGGAGCTTGTGTCTCTCCATGCCCTGTCACCTTGG
GGTGCAGAGGTGAAAATGGAGATTGAGGAAATGGCTGTGGATGGAAAGAGCAGCAAGCCC
CCAAGCACAAAAAGCACTGGGAAGGTCAAAGAAGAACAAGGACCTAGCAAGTCTGAAAAG
AAAATGAAGTTAACAGTGAAGGGTGGAGCAGCAGTAGACCCTGATTCTGGTTTGGAGGAT
TCTGCTCATGTCTTTGAAAAAGGTGGGAAAATTTTCAGTGCAACCCTGGGACTAGTAGAT
ATTGTGAAAGGAACAAATTCCTATTATAAACTGCAGCTGCTAGAGGATGACAGAGAGAAC
AGATACTGGGTGTTCCGATCCTGGGGCCGTGTGGGCACTGTGATCGGCAGTAACAAGCTG
GAGCAGATGCCATCAAAAGAAGATGCCATTGAACACTTCCTGAATTTGTATGAAGAGAAA
ACTGGCAATTCTTGGCATTCAAAGAACTTCACTAAATATCCAAAAAAATTTTACCCACTG
GAAATAGATTATGGACAGGATGAAGAAGCTGTCAGGAAACTGACAGTAGGTGCTGGGACA
AAATCAAAACTCGCTAAGCCAATCCAAGATCTTATTAAGATGATCTTTGATGTGGAGAGC
ATGAAGAAAGCAATGGTGGAATTTGAGATTGACTTGCAGAAGATGCCATTGGGAAAACTG
AGCAAGAGACAGATCCAGAGTGCATACTCCATCCTGAATGAGGTTCAGCAGGCAGTTTCT
GACAGTGGTTCAGAATCTCAGATTTTGGACCTCTCCAACCGCTTTTATACACTGATTCCT
CATGACTTTGGGATGAAGAAGCCACCTCTCCTGAATAACTTGGAATACATTCAGGCTAAA
GTGCAGATGTTGGACAACTTGCTTGATATTGAGGTTGCTTACAGCCTTCTCAGAGGTGGA
AATGAAGATGGAGATAAAGACCCAATTGACATCAACTATGAAAAACTTAAAACAGATATT
AAGGTTGTTGACAAAGATTCAGAAGAAGCCAAGATTATCAAACAATATGTGAAGAACACT
CATGCTGCTACACACAATGCATATGACCTCAAAGTCGTGGAAATCTTCAGGATTGAGCGT
GAGGGGGAGAGCCAGCGTTACAAGCCCTTCAAGCAGCTCCACAACCGCCAGCTGCTGTGG
CACGGCTCCCGCACCACCAACTTCGCCGGGATCCTCTCGCAGGGTCTGCGGATAGCTCCG
CCTGAAGCTCCTGTGACCGGCTACATGTTTGGGAAGGGCATCTATTTTGCAGACATGGTA
TCCAAGAGTGCGAACTACTGTCACACATCTCAAGCTGATCCCATAGGTTTAGTTCTTCTG
GGAGAAGTTGCCCTTGGAAATATGTATGAATTAAAGAATGCTTCCCACATCACAAAATTG
CCCAAGGGAAAACACAGTGTAAAAGGCTTGGGCAAAACCGCACCTGATCCCACAGCCACT
ACCACCCTTGGTGATGTAGAGGTTCCCTTAGGGAATGGGATCTCCACAGGAATCAATGAT
ACCTGTCTTCTGTATAATGAATATATTGTGTATGACGTTGCTCAGGTAAATCTGAAGTAC
CTGTTGAAACTGAAATTCAACTATAAGACGTCACTCTGG
>Fruitfly_PARP1
ATGGATATTGAATTACCTTATCTTGCTGAGTATGCAAGAACTGGACGAGCCACTTGCAAA
GGATGTAAAAGTACTATATCTAAAGATACTCTTCGGATTGCTGTCATGGTTCAATCTGCA
TTTCATGATGCCAAAGTTCCGAATTGGTTTCATAAAACCTGCTTTTTTAAAAACCAGCGT
CCCAGCTCAGTAGGAGACATACAAAACATTGGAAATCTCCGATTTGCCGATCAAAAGGAA
TTAACGGATCTTGTGGAAAATATACAAGAAGTTATAAGCGCACAATTAGGAAAAAAGCGA
TCGAAGGCTTTTAACTTAGCATTAAAAGACTTTGGGATTGAATATGCAAAATCTAGTCGA
TCGACGTGTCGTGGATGTGAACAAAAAATAAACAAGGATCTAGTTCGCTTACGTAAAACT
GTTTATGATACTGAAGTTGGTATGAAGTACGGAGGCCAACCTTTGTGGCATCATTTGGAA
TGCTTCGCCCAATTGCGCTCTGAGCTTGGCTGGTTTGCGTCAGGTGAAGATATGCCAGGA
TTTCAGAGCTTAGCAGATGATGATCAAGCGAAAGTTAAAAACGCCATACCACCAATAAAA
TCTGAAGAACTACCAGATACAAAAAGAGCTAAGATGGAATTATCAGATACAAATGAAGAA
GGAGAAAAGAAACAACGCTTAAAAGATCAAAATGATGCCTACTTCAGGTTTCGCGATGAC
ATTAAAAATAAAATGAAGAAGAAAGACATTGATATACTTCTAAAGTTTAATAATCAACAA
CCTGTAACTGGTGACACAGAAAAGTTATTTGATCAAACTGCCGATTTACTGACATTCGGA

GCTATTGAATCATGTTCTGAATGCAACAGCTGTCAGTTTATTGTTAATAAATCTGGATAT
ATATGTAATGGAAATCATTCTGAGTGGACCAAATGTAACAAGCTGCTAAAAGAGCCAACA
AGATCGGCATGCATAGTGCCAAAAGAACTTAAAGCATTATATAATTTTTTGAATACCGTG
AAAGAAATTCCATCTACACGGATCTTTAATAACTTTCCTCCCAATAAAAGTACCTTTTCT
AGAAGTCTTTTGAAAACGAATAAAAACAATGATGTTTTGGTTAGGCCAACAATACCTCGT
ATAAGTCCGCCATTATACAATTTAAAGTTTTCAATTATAGGCTTAAAGAACCAGCATAAA
GAGCTAAGAAAGCGAATAGAAAATTTGGGCGGTAAATTTGAAGTTAAAATATCGGAAAAC
ACGATAGCAATAATATCAACAGAATTAGAAATACAAAAAAAATCCACCCGTATGAAGTTT
GCAGAAGAGCTCGGAATTCATATTGTGCCCATTGAATTTTTAGATTTTGTTGAAGCCGAT
ACAGAAGGAGCTATTAAATATATAAATAGCACATGTATTTGTAGTTGGGGAACAGATCCA
AAATCCAGAATTCCAAAGGAAACAACAAAAAGTTTAAATTCGAACAGTATATATACAAAA
TCCATGCCAGTATCACGGACATTTAAAGTAAAAGATGGCCTAGCTGTTGATCCGGACAGT
GGGCTCGAGGACATCGCCCATGTTTACGTGGACAGTAACAATAAATACAGTGTTGTTCTT
GGCTTAACTGACATTCAGAGAAATAAGAACTCCTACTACAAAGTTCAGCTTTTAAAAGCG
GATAAAAAGGAGAAATATTGGATTTTTCGTTCATGGGGTCGAATTGGAACAAATATTGGA
AACTCAAAACTTGAAGAGTTCGACACGAGCGAGTCTGCAAAAAGAAATTTTAAAGAAATA
TATGCAGATAAAACTGGAAATGAATACGAGCAACGAGATAACTTTGTTAAAAGAACAGGT
CGAATGTACCCAATCGAAATTCAATATGATGATGACCAAAAGTTGGTAAAACACGAAAGC
CATTTCTTTACTTCCAAATTAGAGATTTCTGTGCAAAATTTAATAAAGCTGATTTTTGAT
ATTGACTCAATGAATAAAACATTGATGGAATTCCATATCGACATGGATAAAATGCCGCTG
GGCAAGCTCAGTGCTCATCAAATCCAATCTGCTTACAGAGTAGTGAAGGAAATTTATAAT
GTACTAGAATGTGGTTCCAATACTGCAAAACTTATTGATGCAACAAATAGGTTTTATACG
TTAATTCCTCATAATTTTGGAGTTCAATTACCAACATTAATTGAAACACATCAACAAATT
GAAGATTTGCGACAAATGCTTGATTCATTAGCTGAGATAGAGGTTGCGTACAGTATAATC
AAAAGCGAAGATGTATCTGATGCTTGTAATCCTTTAGATAATCATTACGCACAGATTAAA
ACTCAGTTGGTGGCATTAGACAAAAATAGTGAAGAATTTTCGATTCTTAGCCAGTACGTA
AAAAACACTCATGCATCTACCCACAAATCTTATGATTTAAAAATTGTTGATGTATTTAAA
GTATCTCGCCAAGGAGAAGCAAGGCGCTTTAAACCATTTAAGAAGCTACATAACAGAAAA
TTATTATGGCACGGATCACGTTTAACTAATTTTGTTGGTATATTATCGCATGGTTTAAGA
ATTGCTCCCCCAGAAGCGCCACCAACAGGTTATATGTTCGGAAAAGGCATTTATTTTGCG
GATATGGTTTCAAAATCCGCAAATTATTGTTGCACAAGTCAACAAAACTCTACTGGATTA
ATGCTTCTATCTGAAGTTGCTTTGGGCGATATGATGGAATGCACTTCAGCGAAATACATT
AATAAACTATCAAATAATAAACATAGTTGTTTCGGTCGTGGTCGCACCATGCCAGATCCT
ACTAAGAGCTATATAAGAAGTGATGGGGTTGAAATTCCTTACGGAGAAACCATTACTGAC
GAACATTTAAAGTCATCGTTATTATATAACGAGTATATAGTATATGATGTTGCGCAGGTC
AATATTCAATATTTGTTTCGTATGGAATTCAAGTATTCTTAT
>Fugu_PARP1
ATGTCGGAATCTCAGAACGACAAGCTCTACAAAGCGGAATATGCCAAGAGCGGCCGCGCC
TCGTGTAAAAAATGCAAAGAAAACATAGCCAAAGACTCGCTGAGGATGGCCATCATGGTG
CAGTCCCCCATGTTTGATGGGAAAGTCCCGCACTGGCACCACTTCTCGTGCTTCTGGCAG
CGAGCAGCAGCTCAATCGACTTCCGACATTGATGGGTTTTCCGGCCTGCGCTGGGAGGAC
CAGGAGAAGATCAAGAAGGCCGTCGAAAGCGGGGGCGCGACGGGAGCAGGAGGAACGGAC
TCAAAGAGTGGAGCGAAAGAAGAGAAGACATTAAACGAATTTGCGGTCGAATATGCCAAA
TCGAACCGAAGCACATGCAAAGGTTGTGAGCAGAAAATAGAAAAGGATCAGATTCGGGTG

TCAAAGAAATCCGTTGACCCGGAGAAACCGCAGCTCGGCCTGATTGACCGCTGGTACCAC
ACAGCGTGCTTCGTGAGCTGGAGGGAGGAACTGGTCTTCAAACCCGAGTACAACGCCTCC
CAGCTGAAGGGATTCAGCAATCTACGGGCAGAAGACAAGGAGGAACTTAAGAGGAAGCTC
CCATCAATCAAAACGGAAGGGAAGCGCAAATCCGATGAGGTGGACGGCGTCTCAAAGAAA
AAGAAGAAGGAGGAGGAGGAAGAGAGGAAGAAGCTGGAGGAGCAGCTGAAGACTCAAAGT
CAGCTCATTTGGGGAATTAAGGACAAGTTGAACAGATTCTGTTCCATTAATGACATGAAA
GAGCTGCTGATTGCAAATGGCCAGGATGTTCCTTCAGGAGAGTCCAATGTGTTGGACTGC
CTGGCTGATGGCATGTCCTTTGGTGCCCTTGAGCCCTGCAGTGAGTGTACTGGCCAGTTG
GTGTTCAAGGGCGATGCTTATTACTGTACAGGCAACATCTCAGCCTGGACAAAGTGCGTT
AACAAGACCACCACACCCAAACGCAAAGACTGGGTGACCCCCAAGGAATTCCATGAAATT
TCCTTCCTGAAGAAATTCAAGTTCAAGAGACAGAACAGGATTTACCCCAAAGAAGCTCCG
CCCCAAACAGTCAGTGCGGTCAAAACTGAATCCTTAGCCAGTGCATCCAGCGTCCCGATG
AGTCTGCCAGAGGGAGCGCCTCCAGACAAGCCTCTCACTGGCATGAAGCTGGTGGCTGTG
GGCAAGCTGAGCAAGAACAAGGATGACATTAAGGCTGCTGTGGAGGAGCTGGGTGGGAAG
ATCACTGGAACAGCCAACAAGGCCTCGCTCTGCCTCAGCTCTAAGAAGGAGCTGGAGAAG
ATGACAAAGAAAATGGAAGAAGTGAAAGATGCAGGGGTGCGCGTGGTCTCCGAAGACTTC
CTCACAGATATCAAGTCGTCGGGTAAAGACCTTCAGGAGCTGGTGTCCCTGCACACCATC
TCTCCCTGGGGGGCAGAGGTCAAAGTGGAGAGTCAGGCACAGCCCGTGGCTTCAAAGTCA
GGGGCAATGGCCGCCAAGAGCACGGGCAAGGTCAAAGAAGAAGAAGGTGGAAGCAAAAGC
AAGAAGATGAAACTAACTGTGAAAGGTGGAGCTGCTGTGGATCCAGATTCAGGTCTGGAA
AACAGTGCACATGTCCTTGAGCAGGGCGGAAAGATGTACAGCGCCACACTGGGTTTGGTG
GACATCGTCAGAGGAACCAACTCCTACTACAAACTACAGCTGCTGGAGGACGATGTTCAG
AAGAGGTACTGGGTGTTCAGGTCGTGGGGCAGAGTGGGCACCACCATCGGAGGCAACAAG
CTGGACAAGTTCAGTGACAAGAACTCTGCCATGAACAACTTCCTGACCGTCTACAAAGAG
AAGACTGGTAACGAGTGGAGCTGCTCCAATTTCACCAAATATCCCAACAAGTTCTACCCC
CTGGAGATCGACTATGGACAGGATGAGGAAGCAGTGAAGAGGTTGACGGCCACAGCAGGG
ACCAAGTCCAAGCTGGCCAAACCCATACAGGAGCTGATCAAGATGATCTTCGATGTGGAG
AGCATGAAGAAGGCCATGGTGGAGTTTGAGATTGACCTCCAGAAGATGCCGCTGGGGAAG
CTGAGTAAGAGGCAGATCCAGAGCGCGTATGCTCTCCTCACTGAAGTCCATCAGGCTGTG
TCAGACTCACTGCCCGAGGCCCAGATATTGGATCTGTCCAATCGTTTCTACACCTTGATC
CCTCACGACTTCGGTATGAAGAAACCACCGCTGCTCAACAATCTGAACTACATTCAGGCG
AAGGTTCAGATGCTGGACAACCTGCTGGACATCGAGGTGGCGTACAGCCTACTGAGAGGA
GGGGCCCAGGACAACGAGAATGACCCCATCGACATCAACTATGAGAAACTCAAAACCAAA
ATCGAGGTCGTTGACAAGTCTGCCAAGGAGGCTGATATTATCCTGCAGTATGTCAAGAAC
ACCCACGCTGCCACACACAACACCTACACACTGGAAGTGCAAGAAATCTTCAAAATACGG
CGAGAGGGGGAGCACCAGCGTTACCGTCCATTCGAAGAGTTGCACAACCGGCAGCTCCTG
TGGCACGGCTCTCGCGCCACCAACTACGCAGGTATAATGTCTCAGGGTCTCCGCATCGCC
CCGCCAGAGGCTCCAGTGACGGGTTACATGTTCGGCAAAGGTGTGTACTTTGCCGACATG
GTGTCCAAGAGTGCAAACTACTGTCACACTTCCCAGTCAGAACCCGTAGGTTTCCTGCTG
CTGGCTGAGGTCGCCCTCGGCAACATGCACGAACTGAAAAAGGCCTCACACATTACAAAA
CTACCCAAAGGAAAACACAGCGTTAAAGGTTTGGGAAGAACCGCCCCTGATCCAAGTGCC
ACTGTCACTCTGGACGGAGTGCAAGTGCCTCTGGGAAATGGAGTCAACACAAACATAGAT
GACACCAGTCTGTTGTACAACGAGTATATCGTGTATGATGTCGCACAGATAAACTTAAAG
TATCTCCTGAAGGTCAGATTTAATTATCAGACGTCCCTGTGG

>Horse_PARP1
ATGTTCGATGGAAAAGTCCCACACTGGTACCACTTCTCCTGCTTCTGGAAGGTCGGCCAC
TCCATCCGGCACCCTGACGTTGAGGTGGATGGGTTCTCGGAGCTGAGGTGGGACGACCAG
CAGAAGGTGAAGAAAACTGCAGAGGCCGGAGGCGTGGCAGGCAAAGGCCAAGGTGGGTCT
GGCAGCAAGGCGGAGAAGACGCTGGGCGACTTTGCGGCTGAGTACGCCAAGTCCAACAGG
AGCACGTGCAAGGGCTGCATGGAGAAGATAGAAAAGGGCCAGATGCGCCTGTCCAAGAAG
ATGCTGGACCCAGAGAAGCCCCAGCTGGGCATGATCGACCGCTGGTACCACCCAAACTGC
TTCGTGAAGAACCGGGAGGAGCTGGGGTTCCGGCCCGAGTACAGCGCGAGCCAGCTCAAG
GGCTTCAGCCTCCTGTCTCCGGAGGATAAAGAAGCCCTGAAGAAGCAGCTCCCGGGAGTC
AAGACTGAAGGAAAGAGAAAAGGCGATGAGGTGGATGGAGTAGACGAAGTGGCCAAGAAG
AAATCTAAGAAAGAGAAGGACAAGGACAGTAAGCTGGAAAAGGCCCTCAAGGCCCAGAAT
GACCTGATCTGGAACATCAAGGACGAGCTAAAGAAAGCGTGTTCAACCAACGACCTGAAA
GAGCTGCTCATCTTCAACAAGCAGCAAGTGCCATCCGGGGAGTCAGCGATCTTGGACCGC
GTGGCTGATGGCATGGTGTTTGGTGCCCTCCTTCCTTGCGAGGAATGCTCAGGCCAGCTG
GTCTTCAAGAGCGATGCGTATTACTGCACTGGGGACGTCACTGCCTGGACCAAGTGTATG
GTCAAGACACAGACACCCAACCGGAAGGAATGGGTCACCCCAAAGGAATTCCGAGAAATC
TCTTACCTCAAGAAACTGAAGATCAAAAAGCAGGACCGGATATTCCCCCCAGAGACCAGC
GCCCCAGCGGCAGCAGCACCCCCGCCCTCCGCGGTCTCAGCACCCGCCACTGTGAACGCC
TCTGCTCTGCCAGACAAGCCGTTATCCAACATGAAGATCCTGACTCTTGGGAAGCTCTCC
CGGAACAAGGATGAAGTGAAGGCCATGATCGAGAAACTGGGGGGAAAGTTGACAGGCACA
ACCAGCAAGGCCTCCCTGTGCATCAGTACCCAAAAGGAGGTGGAAAAGATGAATAAAAAG
ATGGAGGAAGTGAAAGAGGCCAACCTCCGCGTTGTGTCTGAGGATTTCCTGCAGGACGTC
TCCACCTCCACCAAGAGCCTTCAGGAGTTGCTCTCAGCCCACATCTTGTCCCCCTGGGGG
GCCGAGGTGAAAGCGGAGCCTGTGGAAGTAGCGGCCCCAAGAGGGAAGTCAGGGGCAGCG
CTCCCCAAGAAGAGCAAGGGCCCTGTCAAGGAGGAAGGAAGCAACAAATCTGAAAAGAGA
ATGAAATTAACCCTTAAAGGAGGAGCAGCTGTCGATCCTGATTCTGGTCTGGAACATTCT
GCACACGTCCTGGAGAAGGGTGGGAAGGTCTTCAGTGCCACCCTCGGCCTGGTGGACATC
GTGAAAGGAACCAACTCCTATTACAAGCTGCAGCTGCTGGAGGATGACAAAGAAAGCAGG
TACTGGATATTCAGGTCCTGGGGCCGTGTGGGCACAGTGATTGGTAGTAACAAGCGGGAG
CAGATGCCGTCCAAGGAGGATGCCATTGAGCACTTTATGAAATTATACGAAGAGAAAACT
GGGAATGCCTGGCACTCCAAAAACTTCACAAAGTATCCCAAAAAGTTCTACCCTCTGGAG
ATTGACTACGGCCAGGATGAAGAGGCGGTGAAGAAGCTGACGCTAAACCCTGGCACCAAG
TCCAAGCTCCCCAAGCCGGTGCAGGAGCTCATTAAGATGATCTTTGATGTGGAAAGTATG
AAGAAAGCCATGGTGGAGTATGAGATTGACCTTCAGAAGATGCCCTTGGGGAAGCTGAGC
AAGAGGCAGATCCAGGCCGCCTACTCCATCCTCAGTGAGGTCCAGCAGGCGCTGTCCCAG
GGCAGCGGCGACTCCCAGATCCTGGATCTCTCAAACCGCTTCTACACCCTGATCCCCCAC
GACTTTGGGATGAAGAAGCCTCCGCTCCTGAACAACACAGACAGTGTGCAGGCCAAGGTG
GAAATGCTGGACAACCTGCTGGACATCGAAGTGGCCTACAGCCTGCTCAGGGGTGGGTCT
GATGATAGCAGCAAGGACCCAATTGACGTCAACTATGAGAAGCTCAAAACTGACATTAAG
GTGGTGGACAAAGATTCCGAGGAAGCCGAGACCATCAGGAAGTACGTAAAGAACACGCAC
GCAACCACACACAATGCGTATGACCTGGAAGTCGTTGATATCTTTAAGATAGAGCGCGAA
GGGGAGAGCCAGCGTTACAAGCCCTTTAAGCAGCTGCATAACCGGAGGCTGCTGTGGCAC
GGCTCCAGGACCACCAACTTTGCTGGGATCCTGTCCCAGGGTCTCCGGATAGCCCCACCT
GAAGCGCCTGTGACAGGCTACATGTTTGGTAAAGGGATCTATTTCGCTGACATGGTCTCC

AAGAGCGCCAACTACTGCCACACGTCTCAGGGAGACCCGATAGGCTTAATCTTGCTGGGG
GAAGTTGCCCTTGGGAACATGTATGAACTGAAGCATGCGTCGCACATCAGCAAGTTACCC
AAGGGCAAGCACAGCGTCAAAGGTTTGGGCAAAACCACCCCTGACCCCTCAGCCAGTGTT
ACTATGGATGGCGTGGAGGTGCCCCTGGGGACTGGGATTTCGTCGGGGGTGAGCGACACC
TGCCTGCTGTATAATGAGTACATTGTCTATGATATTGCTCAGGTAAATCTGAAGTATCTG
CTGAAGCTGAAGTTCAATTTTAAGACGTCCCTCTGG
>Human_PARP1
ATGGCGGAGTCTTCGGATAAGCTCTATCGAGTCGAGTACGCCAAGAGCGGGCGCGCCTCT
TGCAAGAAATGCAGCGAGAGCATCCCCAAGGACTCGCTCCGGATGGCCATCATGGTGCAG
TCGCCCATGTTTGATGGAAAAGTCCCACACTGGTACCACTTCTCCTGCTTCTGGAAGGTG
GGCCACTCCATCCGGCACCCTGACGTTGAGGTGGATGGGTTCTCTGAGCTTCGGTGGGAT
GACCAGCAGAAAGTCAAGAAGACAGCGGAAGCTGGAGGAGTGACAGGCAAAGGCCAGGAT
GGAATTGGTAGCAAGGCAGAGAAGACTCTGGGTGACTTTGCAGCAGAGTATGCCAAGTCC
AACAGAAGTACGTGCAAGGGGTGTATGGAGAAGATAGAAAAGGGCCAGGTGCGCCTGTCC
AAGAAGATGGTGGACCCGGAGAAGCCACAGCTAGGCATGATTGACCGCTGGTACCATCCA
GGCTGCTTTGTCAAGAACAGGGAGGAGCTGGGTTTCCGGCCCGAGTACAGTGCGAGTCAG
CTCAAGGGCTTCAGCCTCCTTGCTACAGAGGATAAAGAAGCCCTGAAGAAGCAGCTCCCA
GGAGTCAAGAGTGAAGGAAAGAGAAAAGGCGATGAGGTGGATGGAGTGGATGAAGTGGCG
AAGAAGAAATCTAAAAAAGAAAAAGACAAGGATAGTAAGCTTGAAAAAGCCCTAAAGGCT
CAGAACGACCTGATCTGGAACATCAAGGACGAGCTAAAGAAAGTGTGTTCAACTAATGAC
CTGAAGGAGCTACTCATCTTCAACAAGCAGCAAGTGCCTTCTGGGGAGTCGGCGATCTTG
GACCGAGTAGCTGATGGCATGGTGTTCGGTGCCCTCCTTCCCTGCGAGGAATGCTCGGGT
CAGCTGGTCTTCAAGAGCGATGCCTATTACTGCACTGGGGACGTCACTGCCTGGACCAAG
TGTATGGTCAAGACACAGACACCCAACCGGAAGGAGTGGGTAACCCCAAAGGAATTCCGA
GAAATCTCTTACCTCAAGAAATTGAAGGTTAAAAAACAGGACCGTATATTCCCCCCAGAA
ACCAGCGCCTCCGTGGCGGCCACGCCTCCGCCCTCCACAGCCTCGGCTCCTGCTGCTGTG
AACTCCTCTGCTTCAGCAGATAAGCCATTATCCAACATGAAGATCCTGACTCTCGGGAAG
CTGTCCCGGAACAAGGATGAAGTGAAGGCCATGATTGAGAAACTCGGGGGGAAGTTGACG
GGGACGGCCAACAAGGCTTCCCTGTGCATCAGCACCAAAAAGGAGGTGGAAAAGATGAAT
AAGAAGATGGAGGAAGTAAAGGAAGCCAACATCCGAGTTGTGTCTGAGGACTTCCTCCAG
GACGTCTCCGCCTCCACCAAGAGCCTTCAGGAGTTGTTCTTAGCGCACATCTTGTCCCCT
TGGGGGGCAGAGGTGAAGGCAGAGCCTGTTGAAGTTGTGGCCCCAAGAGGGAAGTCAGGG
GCTGCGCTCTCCAAAAAAAGCAAGGGCCAGGTCAAGGAGGAAGGTATCAACAAATCTGAA
AAGAGAATGAAATTAACTCTTAAAGGAGGAGCAGCTGTGGATCCTGATTCTGGACTGGAA
CACTCTGCGCATGTCCTGGAGAAAGGTGGGAAGGTCTTCAGTGCCACCCTTGGCCTGGTG
GACATCGTTAAAGGAACCAACTCCTACTACAAGCTGCAGCTTCTGGAGGACGACAAGGAA
AACAGGTATTGGATATTCAGGTCCTGGGGCCGTGTGGGTACGGTGATCGGTAGCAACAAA
CTGGAACAGATGCCGTCCAAGGAGGATGCCATTGAGCACTTCATGAAATTATATGAAGAA
AAAACCGGGAACGCTTGGCACTCCAAAAATTTCACGAAGTATCCCAAAAAGTTCTACCCC
CTGGAGATTGACTATGGCCAGGATGAAGAGGCAGTGAAGAAGCTGACAGTAAATCCTGGC
ACCAAGTCCAAGCTCCCCAAGCCAGTTCAGGACCTCATCAAGATGATCTTTGATGTGGAA
AGTATGAAGAAAGCCATGGTGGAGTATGAGATCGACCTTCAGAAGATGCCCTTGGGGAAG
CTGAGCAAAAGGCAGATCCAGGCCGCATACTCCATCCTCAGTGAGGTCCAGCAGGCGGTG
TCTCAGGGCAGCAGCGACTCTCAGATCCTGGATCTCTCAAATCGCTTTTACACCCTGATC

CCCCACGACTTTGGGATGAAGAAGCCTCCGCTCCTGAACAATGCAGACAGTGTGCAGGCC
AAGGTGGAAATGCTTGACAACCTGCTGGACATCGAGGTGGCCTACAGTCTGCTCAGGGGA
GGGTCTGATGATAGCAGCAAGGATCCCATCGATGTCAACTATGAGAAGCTCAAAACTGAC
ATTAAGGTGGTTGACAGAGATTCTGAAGAAGCCGAGATCATCAGGAAGTATGTTAAGAAC
ACTCATGCAACCACACACAATGCGTATGACTTGGAAGTCATCGATATCTTTAAGATAGAG
CGTGAAGGCGAATGCCAGCGTTACAAGCCCTTTAAGCAGCTTCATAACCGAAGATTGCTG
TGGCACGGGTCCAGGACCACCAACTTTGCTGGGATCCTGTCCCAGGGTCTTCGGATAGCC
CCGCCTGAAGCGCCCGTGACAGGCTACATGTTTGGTAAAGGGATCTATTTCGCTGACATG
GTCTCCAAGAGTGCCAACTACTGCCATACGTCTCAGGGAGACCCAATAGGCTTAATCCTG
TTGGGAGAAGTTGCCCTTGGAAACATGTATGAACTGAAGCACGCTTCACATATCAGCAAG
TTACCCAAGGGCAAGCACAGTGTCAAAGGTTTGGGCAAAACTACCCCTGATCCTTCAGCT
AACATTAGTCTGGATGGTGTAGACGTTCCTCTTGGGACCGGGATTTCATCTGGTGTGAAT
GACACCTCTCTACTATATAACGAGTACATTGTCTATGATATTGCTCAGGTAAATCTGAAG
TATCTGCTGAAACTGAAATTCAATTTTAAGACCTCCCTGTGG
>Macaque_PARP1
ATGGCGGAGTCTTCGGATAAGCTCTACCGAGTCGAGTACGCCAAGAGCGGGCGCGCCTCT
TGCAAGAAATGCAGCGAGAGCATCCCCAAGGACTCGCTCCGGATGGCCATCATGGTGCAG
TCGCCCATGTTTGATGGAAAAGTCCCACACTGGTACCACTTCTCCTGCTTCTGGAAGGTG
GGCCATTCCATCCGGCACCCTGACGTGGAGGTGGATGGGTTCTCTGAGCTTCGGTGGGAT
GACCAGCAGAAAGTCAAGAAGACAGCGGAAGCTGGAGGAGTGACAGGCAAAGGCCAGGCT
GGAATTGGTAGCAAGGCAGAGAAGACGCTGGGTGACTTTGCAGCAGAGTATGCCAAGTCC
AACAGAAGCACATGCAAGGGGTGTATGGAGAAGATAGAAAAGGGCCAGGTGCGCCTGTCC
AAGAAGATGCTGGACCCGGAGAAGCCACAGCTAGGCATGATTGACCGCTGGTACCACCCA
CACTGCTTTGTCAAGAACAGGGAGGAGCTGGGTTTCCGGCCCGAGTACAGTGCGAGTCAG
CTCAAGGGCTTCAGCCTCCTTGCTGCAGAGGATAAAGAAGCCCTGAAGAAGCAGCTCCCA
GGAGTCAAGAGCGAAGGAAAGAGAAAAGGCGATGAGGTGGATGGAGTAGATGAAGTGGCC
AAGAAGAAATCTAAAAAAGAAAAAGACAAAGATAGTAAGCTTGAAAAAGCTCTAAAGGCT
CAGAACGACCTGATCTGGAACATCAAGGATGAGCTAAAGAAAGTGTGTTCGACTAATGAC
CTGAAGGAGCTACTCATCTTCAACAAGCAGCAAGTGCCTTCTGGGGAGTCGGCGATCTTG
GACCGAGTAGCCGATGGCATGGCGTTCGGTGCCCTCCTTCCCTGCAAGGAATGCTCGGGT
CAGCTGGTCTTCAAGAGTGATGCTTATTACTGCACTGGGGACGTCACTGCCTGGACCAAG
TGTATGGTCAAGACACAGACACCCAACCGGAAGGAGTGGGTAACCCCAAAGGAATTCCGA
GAAATCTCTTACCTCAAGAAATTGAAGGTTAAAAAGCAGGACCGTATATTCCCCCCAGAA
ACCAGCGCCCCAGTGGTGGCCACGCCCCCACCCTCCACAGCCTCGGCGCCTGCTGCTGGG
AACTCCTCTGCTTCAGCAGATAAGCCATTATCCAACATGAAGATCCTGACTCTTGGGAAG
CTCTCCCGGAACAAGGATGAAGTGAAGGCTATGATTGAGAAACTCGGGGGGAAGTTGACA
GGGACAGCCAATAAGGCTTCCCTGTGCATCAGCACCAAAAAGGAGGTGGAAAAGATGAAT
AAGAAGATGGAGGAAGTAAAGGAAGCCAACATCCGAGTTGTGTCTGAGGACTTCCTCCAG
GACGTCTCCGCCTCCACCAAGAGCCTTCAGGAGTTGTTCTTAGCACACATCTTGTCCCCT
TGGGGGGCAGAGGTGAAGGCAGAGCCTGTCGAAGTTGTGGCCCCAAAAGGGAAGTCAGGG
GCTGCACTCTCCAAAAAAAGCAAGGGCCAGGTCAAGGAGGAAGGTATCAACAAATCTGAA
AAGAGAATGAAATTAACTCTTAAAGGAGGAGCAGCTGTGGATCCTGATTCTGGACTGGAA
CACTCTGCGCATGTCCTGGAGAAAGGTGGGAAGGTCTTCAGTGCCACCCTCGGCCTGGTG
GACATCGTTAAAGGAACCAACTCCTATTACAAGCTGCAGCTTCTGGAGGACGACAAGGAA

AGCAGGTATTGGATATTCAGGTCCTGGGGCCGTGTGGGGACGGTGATCGGTAGCAACAAA
CTGGAACAGATGCCGTCCAAGGAGGATGCCATTGAGCACTTCATGAAATTATATGAAGAA
AAAACCGGGAACGCTTGGCACTCCAAAAATTTCACAAAGTATCCCAAAAAGTTCTACCCC
CTGGAGATTGACTACGGCCAGGATGAAGAGGCAGTGAAGAAGCTGACAGTAAATCCTGGC
ACCAAGTCCAAGCTCCCCAAGCCAGTTCAGGACCTCATCAAGCTGATCTTTGATGTGGAA
AGTATGAAGAAAGCCATGGTGGAATATGAGATCGACCTTCAGAAGATGCCCTTGGGGAAG
CTGAGCAAAAGGCAGATCCAGGCCGCGTATTCCATCCTCAGTGAGGTACAGCAGGCAGTG
TCTCAGGGCAGCAGCGACTCTCAGATCCTGGATCTCTCAAATCGCTTTTACACCCTGATC
CCCCATGACTTTGGGATGAAGAAGCCTCCGCTCCTGAACAGTGCAGACAGTGTGCAGGCC
AAGGTGGAAATGCTTGACAACCTGCTGGACATCGAGGTGGCCTACAGTCTGCTCAGGGGA
GGGTCTGATGATAGCAGCAAGGACCCCATCGATGTCAACTATGAGAAGCTCAAAACTGAC
ATTAAGGTGGTTGGCAGAGATTCTGAAGAAGCCGAGATCATCAGGAAGTATGTTAAGAAC
ACTCATGCAACCACACACAATGCGTATGACTTGGAAGTCATCGATATCTTTAAGATAGAG
CGTGAAGGCGAATGCCAGCGCTACAAGCCCTTTAAGCAGCTTCATAACCGAAGATTGCTG
TGGCACGGGTCCAGGACCACCAACTTTGCCGGGATCCTGTCCCAGGGTCTTCGGATAGCC
CCGCCTGAAGCACCTGTGACAGGCTACATGTTTGGTAAAGGGATCTATTTCGCTGACATG
GTCTCTAAGAGTGCCAACTACTGCCATACGTCTCAGGGAGACCCAATAGGCTTAATCGTG
TTGGGAGAAGTTGCCCTTGGAAACATGTATGAACTGAAGCACGCTTCACATATCAGCAAG
TTACCCAAGGGGAAGCACAGTGTCAAAGGTTTGGGCAAAACTACCCCTGACCCTTCAGCT
AGTATTAGTCTGGATGGTGTAGAGGTTCCTCTTGGGACCGGGATTTCATCTGGTGTGAAT
GACACCTGTCTACTATATAACGAGTACATTGTCTACGATATTGCTCAGGTAAATCTGAAG
TATCTGCTGAAACTGAAATTCAATTTTAAGACCTCCCTGTGG
>Medaka_PARP1
ATGGCCGACTCCCAGAATGACAAGCTGTTCCGAGCAGAGTACGCCAAAAGCGGCCAGGCT
TCGTGCAAGAAATGCAAGGAGAAAATCGCGAAGGACTCGCTGAGGATGGCCATCGTGGTG
CAGTCCCCCATGTTCGACGGGAAGGTCCCTCACTGGCATCACTTCTCCTGCTTCTGGCAG
CGAGCCTCGGTGCAGTCCACGGCCGATGTCGGCGGGTTTTCGGATCTCCGCTGGGCGGAC
CAGGAAGCTGTCAAAAAAGCCATAGAAAGTGGTGGAGTGGCCGGAACAGGAAAGGGAAAC
TCTGGTGGTGCGGCTAAAGGAGAAAAGACGCTGAACGACTTTGCGGTGGAGTACGCCAAA
TCAAACCGCAGCACGTGCAAAGGATGTCAGCAGAAAATAGAAAAGGATCAGATTCGTGTG
TCTAAGAAAGTGGTGGATGCAGAGAAGCCTCAGCTGGGCCTGATCGACCGCTGGTACCAC
ACTGCATGTTTTGTGAGCCGCAGAGAGGAGCTGGCTTTCAAACCTGATTACAGCGCTGCT
CAGCTGAAGGGATTCAATGTGCTGCGGGCAGAAGACAAGGAGGAGCTGAAGAAGAGGCTT
CCTGCTGTAAAAACTGAGGGAAAACGTCCAGGCGACGAGTTGGACGGAGTTTCAAAGAAG
CTGAAGCAAGAAGAGGAGGAAGAGAATAAACAGTTGGTGGAGAATTTAAAGAATCAAAGT
CAGCTAATTTGGGGGATTAAGGACAAACTCAGGAAATACTGTTCAACCAACGACATGAAG
GAGCTGCTGATTGCAAACGGTCAGGAAGTTCCTTCTGGGGAGTCCAACGTGGTCGACAGA
GTGGCTGACGGTATGGCCTTCGGGGCTCTGGAGGCCTGTAACGAGTGCCAAGGTCAACTG
GTGTTTAAAGGGGATGCGTATTACTGCACAGGAGACATCTCAGCCTGGACTAAGTGCGTG
TTCAAAACTGCAACACCCCTGCGCAAAGACTGGATCATCCCGAAGGAATTCCATGAAGTT
CCATTTTTGAAAAAGTTCAAGTTCAAGCGTCAAGACCGAGTTTATCCCAAAGAAGCTCCG
ACCCAAACATTGACAACAGCTAAAGCTGAACCTCTGGCGAGTGGATCTGGAGCCCTGAAG
GAGCAGCTGCCAGAAGGAGCACCTGCAGATAAACCTCTGACTGGAATGAAGCTGCTGGCT
GTTGGAAAACTGTCCAAGAACAAGGAGGAGCTGAAGGCTGAAGTGGAGGAGATGGGTGGA

AAGATCACCGGCTCAGCCAACAAGGCGTCTCTCTGTCTCAGCACCAAGAAAATGGGAAAA
AAGATGGAGGAAGTTCGGGACGCCGGAGTGCGCGTGGTCTCTGAGGATTTCCTCCTAGAC
ATAAAGTCTTCAGGCAAAGCTCTTCAGGAGCTGGTCTCTCTGCACGCCATCTCAACATGG
GGCGTGGAGGTTAAAGTAGAGCCACAGGCTCCAGCTGCAGCCTCAAAGTCTGGAGCTCTT
GCTGCAAAGAGCAGCGGCAAGGTGAAGGAGGAAGAAGGTGGCAGCAAATCCAAGAAAATG
AAGCTCACGGTCAAAGGTGGAGCTGCCGTGGATCCAGACTCAGGTCTTGAAAACAGCGCC
CATGTCCTGGAACAGAGCGGGAAGATGTACAGTGCCACTCTGGGCCTGGTGGACATCGTG
AGAGGGACCAACTCCTACTACAAACTGCAGCTGCTGGAGGACGATCTGCAGAAAAGGTAC
TGGGTGTTCAGATCATGGGGCAGAGTGGGTACCACCATCGGAGGAAACAAACTGGACAAA
TTCCATGACAAGAACTCGGCTTTGGATAATTTTCTTAGTGTTTATAAAGAAAAGACGGGA
AACGAATGGGGCTCGTCTAACTTTATCAAATATCCCACTAAGTTCTACCCACTGGAGATT
GATTATGGACAGCAGGATGAGGAAGCCGTGAAGAGGCTGACGGCCTCTGCTGGCACCAAG
TCCAAGCTTCCCAAACCCGTTCAGGAGCTGATTAAGATGATTTTTGATGTGGAGAGCATG
AAGAAGGCCATGGTGGAGTTTGAAATCGACCTCCAGAAGATGCCTCTGGGCAAGCTGAGT
AAAAGACAAATTCAGAGCGCCTACGCTCTCCTCACTGAAGTCCAGCAGGCGGTGTCAGAC
TGTGTGCCCGAAGCACAGATACTGGATCTCTCTAACCGCTTCTACACACTCATACCTCAT
GACTTTGGCATGAAAAAGCCTCCACTACTCAACAGCTTGGATTACATTCAAGCTAAAGTT
GAGATGTTGGACAACCTGTTGGACATTGAAGTGGCTTACAGTCTGCTAAGAGGAGGAGCT
CAGGACAATGAACACGATCCCATCGATATCAACTACGAGAAACTCAAAACCAAGATTGAG
GTTGTTGACAAGTCAACCCAGGAGGCTGAGGTCATTACCCAGTATGTGAAGAACACCCAT
GCTGCTACGCACAACACATACACTCTGGAAGTACAAGAAATCTTCAAAATTGGCCGAGAA
GGAGAGCGCCAACGCTTCCGTCCCTTCGAGGAGCTTCACAACAGACAGTTGTTGTGGCAC
GGCTCTCGCGCAACAAACTACGCCGGCATCATGTCTCAGGGTCTCCGCATCGCTCCTCCT
GAAGCCCCAGTGACCGGTTACATGTTTGGTAAAGGTGTATACTTTGCTGACATGGTGTCC
AAGAGTGCAAACTACTGTCATACCTCGCAGTCAGACCCCGTTGGCCTCATACTGCTGGCA
GAGGTGGCGTTAGGAAACATGCATGAACTGAAGAAGGCCAGTCACATCACGAAATTGCCA
AAGGGAAAGCACAGTGTCAAAGGTCTGGGTAGAACCGCTCCTGATCCAAGTGCTACTGTC
ACTTTGGACGGAGTGCAAGTGCCTCTGGGAAAAGGAGTCCAAACAAACATAGACGATACA
AGCCTACTCTACAACGAATACATTGTGTATGATGTAGCCCAGATCAACTTGAAGTATCTT
CTGAAGATCAAGTTTAACTACCAGACATCCTTGTGG
>Naked_mole_rat_PARP1
ATGGCCGAGGCAGCGGACAAGCTCTACCGCGCCGAGTACGCCAAGAGCGGGCGCGCATCG
TGCAAGAAATGCGGCGAGAGCATCCCCAAGGACTCGCTGCGCATGGCGCTCATGGTGCAG
TCACCCATGTTCGATGGCAAAGTCCCACACTGGTACCACTTCTCCTGCTTCTGGAAAATG
GGCCACACCATCCGGCACCCTGACGTGGAGGTGGACGGCTTCTCGGAGCTGCGGTGGGAT
GACCAACAGAAAGTGAAGAAGACTGCGGAGGCCGGAGGAGTGACGGGCAAAGGCCAGGAT
GGAGTTGGCGGCAAGGCGGAGAAAACCTTGGGTGACTTCGCTGCAGAGTACGCCAGGTCC
AACCGAAGCACGTGCAAGGGGTGCATGGAGAAGATCGAGAAGGGCCAGATGCGCCTGTCC
AAGAAGATGCTAGACCCAGAGAAGCCGCAGCTAGGCATGATCGACCGTTGGTACCACCCA
GACTGCTTTGTCAAGAACAGGGAGGAGCTGGGCTTCCAGCCTGAGTACAATGCCAGCCAG
CTCAAGGGCTTCAACCTCCTCTCTGCAGAGGATAAAGAAGCCCTGAAGAAGCAGCTCCCG
GGGGTCAAGAGTGAAGGAAAGAGGAAAGGCGATGAGGTGGATGGGACAGATGAAGTGGCC
AAGAAGAAATCTAAGAAAGAAAAGGACAAGGACAGCAAGCTGGAAAAGGCCCTCAAGGCC
CAGAATGACCTGATCTGGAACATCAAGGACGAGCTGAAGAAAGCATGTTCGACAAATGAC

CTGAAAGAGCTGCTCATCTTCAACAAGCAGCAAGTGCCATCTGGGGAGTCGGCGATCTTG
GACCGAGTCGCCGACGGCGTAGCATTTGGGGCCCTCCTTCCCTGTGAGCAGTGCTCAGGC
CAGCTGGTCTTCAGGAGCGATGCTTATTACTGTACAGGGGACGTCACGGCCTGGACTAAG
TGCATGGTCAAGACACAGACACCCAGTCGAAAGGAATGGGTGACTCCCAAGGAATTCCGA
GAAATCTCTTACCTGAAGAAATTGAAGGTCAAAAAGCAAGACCGAATATTTCCCCCTGAG
GCCATGGCACCCCCGCCCACCCTGGCCTTGACGCCGGCTGCTGTGAACTGCGCTGCCCCG
GCAGATAAGCCCCTGTCCAACATGAAGATCTTGCTGCTTGGAAAGCTCTCCCGGAACAAG
GATGAAGTGAAGGCTGCCGTCGAGAAGCTTGGGGGCAAGCTAACGGGATCAGCCAGCAAG
GCCGGCCTGTGCATCAGCACCAAGAAGGAGGTAGAAAAGATGAATAAGAAGATGGAGGAA
GTAAAAGAAGCCAACATCCGTGTTGTGTCTGAGGATTTCCTCCAGCATGTGTCCACCTCA
ACCAAGGCCCTACAGGAGCTGTTATTAGCGCACATCTTGTCCCCTTGGGGGGCAGAGGTG
AAGGCAGAGCCTGTTGAAGCAGAGCCCCCAAGGGCGAAGTCGGGGGCTCTGGTCCCCAAG
AAGAGCAAGGGCAGTACCAAGGAAGAAGGTATCAAGTCTGAAAAGAGAATGAAGTTAACT
CTTAAAGGAGGAGCAGCTGTGGATCCTGACTCTGGTCTGGAACACTCAGCACATGTCCTG
GAGAAGGGCGGGAAGGTCTTCAGTGCAACCCTTGGCCTGGTGGACATTGTGAAAGGGACC
AACTCCTACTACAAGCTGCAGCTTCTGGAGGATGACAAGGAGAGCAGGTACTGGACATTC
AGGTCCTGGGGCCGTGTGGGCACTGAGATTGGTAGCAACAAGCTGGAGCAGATGCCATCT
AAGGAAGATGCTATTGAGCACTTTATGAAATTATATGAAGACAAAACTGGAAATGCTTGG
CACTCCAAAAACTTTACAAAGTATCCCAAAAAGTTCTACCCTCTGGAGATTGACTATGGC
CAGGATGAGGAGGCAGTGAAGAAACTGACAGTAAACCCTGGCTCCAAATCCACACTCCCC
AAGCCGGTGCAGGAGCTCATTAGGATGATCTTTGATGTGGAAAGTATGAAGAAAGCCATG
GTGGAGTACGAGATCGACCTTCAGAAGATGCCCTTGGGGAAGCTGAGCACAAGGCAGATC
CAGGCTGCATACGCCATCCTCAGCGAGGTCCAGCAGGCCCTGTCCCAAGGCAGCAGTGAC
TCCCAGATCCTGGATCTCTCAAATCGCTTCTATACCCTGATCCCCCATGACTTTGGGATG
AAGAAGCCCCCGCTGCTGAACAGCGCAGACAGTGTGCAGGCCAAGGTGGAAATGCTGGAC
AACCTCCTGGACATCGAGGTGGCCTACAGTCTGCTGAGGGGTGGGTCTGATGACAGCAGC
AAGGACCCCATCGATGTCAACTATGAGAAGCTCAGAACTGACATAAAGGTGGTTGACAAA
GATTCTGAAGAAGCGGAGATCATCAGGAAATATGTGAAGAACACGCACGCAACCACACAC
AATGCATATGACTTGGAGGTCATCGACATCTTCAAGATAGCGCGGGAAGGCGAGAGCCAG
CGTTACAAGCCCTTCAAGCAGCTCCACAACCGGAAGTTGCTGTGGCACGGGTCCCGGACC
ACCAACTTCGCCGGGATCCTGTCCCAGGGCCTGCGGATAGCGCCGCCTGAAGCTCCTGTG
ACAGGCTACATGTTTGGGAAAGGCATCTATTTTGCTGACATGGTCTCCAAGAGTGCCAAC
TACTGCCACACATCTCAGGGAGACCCAATAGGCCTAATCCTGTTGGGAGAAGTCGCCCTT
GGAAACATGTACGAACTGAAGCATGCTTCACACATCAGCAAGTTACCCAAGGGCAAGCAC
AGCGTCAAAGGTTTGGGAAAAACGACCCCTGACCCCTCGGCTAGCATCGCTGTGCAGGGG
GTCGAGGTGCCCCTGGGCCCGGGGATCTCCTCTGGTGTGAACGACACCTGCCTGCTGTAC
AATGAGTACATCGTCTATGACATTGCTCAGGTGAATCTGAAGTATCTGCTGAAACTGAAG
TTCAATTTTAAGTCCTCCCTGTGG
>Opossum_PARP1
ATGGCGGAGTCGGCGGACAAGCCCTACCGGGCTGAGTACGCGAAGAGCGGACGCGCCTCT
TGCAAGAAATGTGGCGAGAGCATAGCCAAGGACTCGCTCCGCCTGGCCATCATGGTGCAG
TCACCCATGTTTGATGGGAAGATCCCAAACTGGCATCACTATGCTTGCTTCTGGAAGCGT
GGGCTCGTCATCTCTCAGGCTGAGCTTGAAGTGGATGGGTTCTTGGAGCTGCGATGGGAC
GACCAGCAGAAAATTAAGAAGACCATCGAAACTGGAGGGCTGACAGGAGGCAAAGGTGGG

AGTCAAGATGGTGGTGGTAAAGGGGAGAAAACCCTAATCAACTTTGCAGCAGAGTATGCC
AAGTCCAATAGGAGCGCTTGCAAAGGGTGTCAGCAGAAAATAGAAAAGGGCCAGGTACGG
TTGTCCAAGAAAATGATTGATCCAGAGAAGCCACAACTGGGGATGATTGACCGCTGGTAC
CACCCAGACTGCTTCGTCAAATGCCGAGATGAGCTGGGATTCCTGCCTCAATACAGTGCT
AGCCAGTTCAAAGGTTTCAGTATTCTGCAGCCAGAGGACAAGGAGACCCTCAAGAAACAG
CTCCCAGCAGTCAAGACTGAAGGGAAAAGAAAAGGAGATGAGGTGGATGGAGATGATTTA
GCCAAGAAGAAACCCAAGAAAGAAAAAGAAAAAGAAACGAAGATAGAGAAACTCTTCAAG
ACACAAACAGAGCTCATCTGGAACATCAAAGACGAACTCAAGAAAGTCTGTTCTACCAAT
GATCTGAAAGAGCTGCTGATAGCCAACAAGCAGGAAGTGCCTTCTGGGGACTCAGCTATA
TTGGACAGAGTAGCAGATGGGATGGCTTTTGGAGCTCTTCTTCCCTGTGAAGAGTGCAAA
GGACAGTTTGTCTTCAAGAGTGATGCATACTACTGTTCTGGAGACATAACTGCTTGGACC
AAGTGTGTGGCCAAAACACAGACTCCCAACAGGAAGGAATGGATCATCCCCAAAGAGTTC
CGTGAAATCTCTTACCTCAAAAAATTTAAGTGCAAAAAACAGGACCGAGCATTCCCCCCA
GAAGCTAATGCCCCACCTTCAAAGCCCCTCCCCTCATCAGTGGCAGCCCCTCCCACCGTG
AACTCCTCTGCCCAGCCAGATAAGCCATTGTCCAACATGAAGATTTTGACTCTAGGGAAG
CTTTCCAGAAACAAGGATGAAATGAAAGCCACAATAGAGGAACTTGGGGGGAAGCTGACA
GGGACAGCTAACAAAGCTTCCCTCTGCATCAGTACAAAAAAGGAGGTTGAGAAGATGAAT
AAGAAGATGGAAGAGGTCAAAGAAGCCAATGTGCGTGTAGTATCAGAGGATTTCCTCAAG
GATATATCTGCTTCTGGCAAGAGCCTCCAAGAGCTGCTGTCTCTGCATATCTTATCTTCC
TGGGGCACTGAAGTGAAGCAGGAACCTGTGGAAGTGAAGGCAGGGGGGAAGTCAGGCCCT
TCTTCCAAGAAGAGCAAGGGCCAAGTCAAGGAGGAGGAAGGGACCAGCAAATCGGAAAAG
AAAATGAAATTAACACTAAAAGGTGGAGCAGCTGTGGATCCTGACTCTGGGCTAGAGGAT
TCAGCTCACGTACTTGAAAAAAATGGAAAAATCTTCAGTGCTACCCTTGGCCTTGTTGAC
ATTGTTAAAGGGACCAACTCATATTATAAGCTGCAGCTCCTAGAAGATGATAGAGAAATT
AGGTACTGGATATTTAGATCTTGGGGCCGAGTGGGCACTGTGATTGGCAGCAATAAACTG
GAACAGATGTCATCCAGGGAAGATGCTATTGATCACTTTTGTAAGTTATATGAGGAGAAA
ACTGGCAACTCTTGGCATTCTACCAACTTCACTAAATATCCCAAGAAATTCTATCCACTG
GAGATCGACTATGGGCAGGATGAAGAAGCTGTGAAGAAGCTGACTGTCAGTGCTGGTACC
AAGTCAAAACTCCCCATACCAGTTCAAAACCTCATCAAGATGATTTTTGATGTGGAGAGC
ATGAAAAAGGCCATGGTGGAATTTGAGATTGACCTTCAGAAGATGCCATTGGGAAAGCTG
AGCAAGAGACAGATCCAGAATGCATACTCAATCCTCAGTGAGGTACAGCAGGTGGTTTCC
CAGGGTGGCAGTGACTCTCAGATTCTGGATCTCTCCAATCGCTTCTATACCCTGATTCCC
CATGACTTTGGAATGAAGAAGCCTCCACTCCTGAACAATATTGATTGTGTGCAGGCCAAA
GTAGAGATGCTTGACAATCTACTGGACATTGAGGTAGCATATAGTCTACTCCGTGGTGGG
TCTGAAGATGGCAGCAAGGATCCCATTGATGTCAACTATGAGAAGCTTAAAACTGATATT
AAGGTGGTTGACCAGAATTCAGAAGAAGCTGACATCATTAGGCAATATGTTAAGAACACA
CATGCTACTACTCACAATGCCTACGACTTGGAAATTATAGATATCTTTAAGATTGAACGT
GATGGAGAAAGCCAACGTTATAAGCCCTTTAAACAGCTTCATAACCGGCGTTTGCTGTGG
CACGGGTCCAGGGCTACCAACTATGCTGGAATCCTTTCTCAGGGTCTGCGAATTGCCCCA
CCTGAAGCCCCTGTGACTGGTTACATGTTTGGTAAAGGAATCTACTTTGCTGACATGGTT
TCCAAGAGTGCCAATTACTGCCACACATCTCAGGGAGACCCAATAGGATTAATCCTCCTG
GGAGAAGTTGCCCTTGGAAACATGTATGAGCTGAAGCACGCCTCCCACATCAGCAAGTTA
CCCAAGGGCAAGCACAGCGTCAAAGGTTTGGGCAAAACTACACCTGATCCAACGGCCAGC
ATCACTCTTGATGGTGTAGAAATTCCCTTGGGGACTGGGGTTTCATCCGGCGTTAGTGAC

ACCTGTCTGCTCTATAACGAATACATTATCTATGACATTGCTCAGGTCAATCTGAAGTAT
CTGCTGAAACTGAAGTTCAACTTCAAGAGCTCTCTGTGG
>Orangutan_PARP1
ATGTTTGATGGAAAAGTCCCACACTGGTACCACTTCTCCTGCTTCTGGAAGGTGGGCCAC
TCCATCCGGCACCCTGACGTTGAGGTGGATGGGTTCTCTGAGCTTCGGTGGGATGACCAG
CAGAAAGTCAAGAAGACAGCGGAAGCCGGAGGAGTGACAGGCAAAGGCCAGGATGGAATT
GGTAGCAAGGCAGAGAAGACTCTGGGTGACTTTGCAGCAGAGTATGCCAAGTCCAACAGA
AGTACGTGCAAGGGCTGTATGGAGAAGATAGAAAAGGGCCAGGTGCGCCTGTCCAAGAAG
ATGCTGGACCCGGAGAAGCCACAGCTAGGCATGATTGACCGCTGGTACCATCCAGGCTGC
TTTGTCAAGAACAGGGAGGAGCTGGGTTTCCGGCCCGAGTACAGTGCGAGTCAGCTCAAG
GGCTTCAGCCTCCTTGCTACAGAGGATAAAGAAGCCCTGAAGAAGCAGCTCCCAGGAGTC
AAGAGTGAAGGAAAGAGAAAAGGCGATGAGGTGGATGGAGTGGATGAAGTGGCCAAGAAG
AAATCTAAAAAAGAAAAAGACAAAGATAGTAAGCTTGAAAAAGCCCTAAAGGCTCAGAAC
GACCTGATCTGGAACATCAAGGACGAGCTAAAGAAAGTGTGTTCGACTAATGACCTGAAG
GAGCTACTCATCTTCAACAAGCAGCAAGTGCCTTCTGGGGAGTCGGCGATCTTGGACCGA
GTAGCTGATGGCATGGTGTTCGGTGCCCTCCTTCCCTGTGAGGAATGCTCGGGTCAGCTG
GTCTTCAAGAGCGATGCTTATTACTGCACTGGGGACGTCACTGCCTGGACCAAGTGTATG
GTCAAGACACAGACACCCAACCGGAAGGAGTGGGTAACCCCAAAGGAATTCCGAGAAATC
TCTTACCTCAAGAAATTGAAGGTTAAAAAGCAGGACCGTATATTCCCCCCAGAAACCAGC
GCCCCCGTGGCAGCCACGCCCCCGCCCTCCACAGCCTCGGCGCCTGCTGCTGTGAACTCC
TCTGCTTCAGCAGATAAGCCATTATCCAACATGAAGATCCTGACTCTCGGGAAGCTGTCC
CGGAACAAGGATGAAGTGAAGGCTATGATTGAGAAACTCGGGGGGAAGTTGACGGGGACG
GCCAACAAGGCTTCCCTGTGCATCAGCACCAAAAAGGAAGTGGAAAAGATGAATAAGAAG
ATGGAGGAAGTAAAGGAAGCCAACATCCGAGTTGTGTCTGAGGACTTCCTCCAGGACGTC
TCCGCCTCCACCAAGAGCCTTCAGGAGTTGTTCTTAGCGCACATCTTGTCCCCTTGGGGG
GCAGAGGTGAAGGCAGAGCCTGTTGAAGTTGTGGCCCCAAGAGGGAAGTCAGGGGCTGCG
CTCTCCAAAAAAAGCAAGGGCCAGGTCAAGGAGGAAGGTATCAACAAATCTGAAAAGAGA
ATGAAATTAACTCTTAAAGGAGGAGCAGCTGTGGATCCTGATTCTGGACTGGAACACTCT
GCGCATGTCCTGGAGAAAGGTGGGAAGGTCTTCAGTGCCACCCTTGGCCTGGTGGACATC
GTTAAAGGAACCAACTCCTATTACAAGCTGCAGCTTCTGGAGGACGACAAGGAAAACAGG
TATTGGATATTCAGGTCCTGGGGCCGTGTGGGTACGGTGATCGGTAGCAACAAACTGGAA
CAGATGCCGTCCAAGGAGGATGCCATTGAGCACTTCATGAAATTATATGAAGAAAAAACC
GGGAATGCTTGGCACTCCAAAAATTTCACGAAGTATCCCAAAAAGTTCTACCCCCTGGAG
ATTGACTATGGCCAGGATGAAGAGGCAGTGAAGAAGCTGACAGTAAATCCTGGCACCAAG
TCCAAGCTCCCCAAGCCAGTTCAGGACCTCATCAAGATGATCTTTGATGTGGAAAGTATG
AAGAAAGCCATGGTGGAGTATGAGATCGACCTTCAGAAGATGCCCTTGGGGAAGCTGAGC
AAAAGGCAGATCCAGGCCGCATACTCCATCCTCAGTGAGGTCCAGCAGGCAGTGTCTCAG
GGCAGCAGCGACTCTCAGATCCTGGATCTCTCAAATCGCTTTTACACCCTGATCCCCCAC
GACTTTGGGATGAGGAAGCCTCCGCTTCTGAACAATGCAGACAGTGTGCAGGCCAAGGTG
GAAATGCTTGACAACCTGCTGGACATCGAGGTGGCCTACAGTCTGCTCAGGGGAGGGTCT
GATGATAGCAGCAAGGACCCCATCGATGTCAACTATGAGAAGCTCAAAACTGACATTAAG
GTGGTTGACAGAGATTCTGAAGAAGCCGAGATCATCAGGAAGTATGTTAAGAACACTCAT
GCAACCACACACAATGCGTATGACTTGGAAGTCATCGATATCTTTAAGATAGAGCGTGAA
GGCGAATGCCAGCGTTACAAGCCCTTTAAGCAGCTTCATAACCGAAGATTGCTGTGGCAC

GGGTCCAGGACCACCAACTTCGCTGGGATCCTGTCCCAGGGTCTTCGGATAGCCCCGCCT
GAAGCGCCCGTGACGGGCTACATGTTTGGTAAAGGGATCTATTTCGCTGACATGGTCTCC
AAGAGTGCCAACTACTGCCATACATCTCAGGGAGACCCAATAGGCTTAATCCTGTTGGGA
GAAGTTGCCCTTGGAAACATGTATGAACTGAAGCACGCTTCACATATCAGCAAGTTACCC
AAGGGCAAGCACAGTGTCAAAGGTTTGGGCAAAACTACCCCTGACCCTTCAGCTAGCATT
AGTCTGGATGGTGTAGAGGTTCCTCTTGGGACCGGGATTTCATCTGGTGTGAATGACACC
TGTCTACTATATAACGAGTACATTGTCTACGATATTGCTCAGGTAAATCTGAAGTATCTG
CTGAAACTGAAATTCAATTTTAAGACCTCCCTGTGG
>western_painted_turtle_PARP1
ATGTTCGATGGTAAAGTCCCCCACTGGCATCATTATACTTGTTTCTGGAAGCGGGCCCGA
CTCGTGTCCCATGCAGATGTTGATGGCTTCCCTGAGCTCCGATGGGAAGATCAGGAGAAA
ATCAAAAAAGCAATTGAAACTGGAGGAGTCGCAGCAGGTAAAGGGACTGACCAGGAGGGA
GGTGGCAAGGCTGAGAAGAGTTTAAATGACTTCGCTGCAGAATATGCCAAGTCTAACAGA
AGTACCTGCAAAGGTTGTGAACAGAAAATAGAAAAGGGCCAGATCAGAATTTCCAAGAAG
ATGGTGCATCCTGAAAAGCCCCAGCTGGGAATGATAGATAATTGGTACCATCCTGACTGC
TTTGTCAGCCACCGAGCAGAACTGGGCTTTCTCCCTGCATTTGGAGCCAGTCAGCTCCAG
GGCTTTGGGATGTTGAAAGCAGAAGATAAAGAAGCTCTGAAAAAGCAGCTGCCTGCCATG
AAGAGTGAAGGAAAGAGAAAAGGAGATGAGGTGGATGGAAATGTGACCTCAAAAAAGAAA
CAGAAAAAAGAAAAAGACAGAGAATCCAAGCAGGAGAAGCTGTTGAAGGAGCAGACAGAA
TTGATTTGGAGCATCAAAGATGAGCTGAGGAAAGTCTGTTCCACTAACGACCTGAAAGAG
CTGCTGATAGCCAACAAACAGGAAGTGCCTTCTGGGGAATCTGCTATCTTGGACCGAGTA
GCAGATGGGATGGCCTTCGGAGCTCTGCTTCCCTGTGAGGAGTGCAAGGGGCAGTTTGTG
TTCAAGAGTGATGCATACTACTGCTCAGGGGACATCACTGCCTGGACTAAATGTGTTGCC
AAAACACAGACCCCCAACAGGAAGGAATGGATAATCCCAAAGGAGTTCCGGGAAATCTCC
TACCTGAAGAAGTTTAAGTGTAAAAAGCAAGACAGAGCATTCCCTCCAGAGGCTGTGGCT
GTGAACGCAGTGCTTCCAGCAGCAGCTTCTGCTCCTTTGACAGAGAAGGCGTCTGTGCCA
GCAGATAAGCCATTATCCAGCATGAAGATTTTGACCCTTGGAAAATTATCCAGGAACAAG
GAAGAAGTGAAGGCTATGATTGAGGACCTGGGCGGAAAGGTGACAGGGACAGCGAACAAG
GCTACCCTGTGCATCAGCACACAAAAGGAAGTTGAGAAAATGACCAAGAAGATGGAAGAA
GTGAAGGAGGCCAAAGTCCGGGTGGTCTCAGAGGAGTTTCTGCAGGATGTGAAATCCTCC
CGCAAGGGCTTCCAGGAGCTCCTGTCGCTGCATGCGCTCTCGCCTTGGGGTGCAGAGGTG
AAGCAGGAGCACACGGAGACGTCCCTGGGAGGAAAGTCCAGCGGGCTCCCAAATATGAAG
AGTGCTGGGAAGGTCAAGGAAGAACAAGGAACCAGCAAGTCTGAAAAGAAAATGAAGTTA
ACAGTTAAAGGAGGAGCAGCAGTGGATCCTGATTCAGGTTTGGAGGACTCTGCTCATGTC
TTTGAAAAGAGTGGTAAAATCTTCAGTGCCACGCTCGGCCTGGTAGATATCGTCAGAGGA
ACCAATTCCTATTACAAACTGCAGCTACTGGAGGATGACAGAGAGATCAGGTACTGGGTT
TTCAGATCTTGGGGTCGTGTTGGCACTGCGATTGGGAGTAACAAACTGGAGCAGATGCCA
TCTAAAGATGATGCCATTGAGCACTTCTTGAATTTGTATGAAGAGAAAACGGGCAACTCC
TGGCATTCCAAGAACTTCACTAAATATCCCAAAAAATTCTACCCCCTGGAAATAGACTAT
GGACAGGATGAAGAAGCTGTGAGGAAACTGACAGTGAGTGCTGGGACCAAGTCAAAGCTT
CCTAAGCCAGTCCAGGACCTTATTAAGATGATCTTTGATGTGGAGAGCATGAAGAAAGCC
ATGGTGGAATTTGAGATTGACCTCCAGAAGATGCCACTGGGAAAGCTGAGCAAGAGGCAG
ATCCAGAGTGCATACTCCATCCTTAACGAAGTTCAGCAGGCAGTTTCTGATGGTGGCAGT
GATTCTCAGATACTGGATCTCTCCAATCGCTTCTATACCCTGATACCTCATGACTTTGGG

ATGAAGAAACCACCTCTCTTAAATAACCTAGATCATATCCAGGCCAAAGTGGAGATGTTA
GACAACCTGCTTGATATTGAGGTCGCTTACAGCCTTCTCAGAGGTGGAAATGAAGATGGG
GATAAAGACCCAATTGACATCAACTACGAAAAGCTCAAAACTAACATTAAGGTTATTGAT
AAAGATTCAGAAGAAGCCAAGATCATAAAGCAGTATGTGAAGAATACACATGCCTCTACC
CACAATGCATATGATCTAAAAGTTGTGGATATCTTCAAGATTGAACGTGAAGGAGAAAAT
CAGCGTTACAAACCGTTCAGACAGCTTCATAATCGCCAGCTGCTCTGGCATGGCTCCCGC
GCTACCAACTTTGCTGGCATCTTCTCACAGGGTCTCCGAATAGCTCCACCTGAAGCTCCT
GTGACTGGTTACATGTTCGGTAAAGGAATCTATTTTGCGGACATGGTATCCAAGAGTGCC
AACTACTGTCACACGTCTCAGACTGACCCAATAGGCTTAATCTTACTGGGAGAGGTTGCC
CTTGGAAACATGCATGAGCTAAAGAATGCTTCCCACATAACTAAGTTGCCCAAGGGAAAA
CACAGTGTCAAAGGTTTGGGCAAAACTGCACCGGATCCTACAGCCACTATCACTCTAGAT
GGTGTAGATATTCCTTTAGGGAATGGGATTCCATCTGGAGTTAGCGATACCTGTCTTCTA
TATAATGAATATATTGTCTATGATGTTGCTCAGGTAAATTTGAAGTACCTGCTGAAACTG
AAGTTCAACTATAAGACATCACTCTGG
>Tetraodon_PARP1
ATGGCGGAATCTCAGAATGACAAGCTCTACAAAGCGGAATATGCCAAGAGCGGCCGCGCT
TCGTGTAAAAAATGCAAAGAAAACATAGCCAAAGACTCTCTAAGAATGGCCATCATGGTG
CAGTCCCCCATGTTTGATGGGAAAGTCCCCCACTGGCACCACTTCTCCTGCTTCTGGCAG
CGGGCAGCGGCTCAGTCCACTTCGGACATTGATGGGTTTTCCAGCCTGCGCTGGGAGGAC
CAGGAGAAGGTCAAGAAGGCCATCGAAAGTGGCGGCGCGACGGGAGGAGGAACACACTCA
AAGGGTGGAGCGAAGGCGGAGAAAACACTGAGCGAATTTGCGGTGGAATACGCCAAATCG
AACCGAAGCACGTGCAAAGGTTGCGAGCAGAAAATAGAAAAGGATCAGATTCGTGTTTCA
AAGAAAACCATCGACCCCGAGAAGCCTCAGCTGGGCCTGATTGACCGCTGGTACCACACA
GCGTGCTTCGTGAGCCGGAGAGAAGAGCTGCTCTTCAAACCGGAGTACAGCGCCTCCCAG
CTGAAGGGCTTCAACACACTGCGGGCAGAAGACAAGGAGGAACTTAAGAAAAGGCTCCCA
GAAGTCAAAACAGAAGGGAAGCGCAAAGCCGACCAGGTGGACGGCGTCTCCTCTTTTGCC
TTTTCAACTCAACTACTCTTTGTTTTTAAGCGTAACATGAAAAATATGGTGTTGTTCTCT
GTGCTTTTGCAGAACCAAAGTCAGCTTATCTGGGGAATAAAAGACAAGTTGAACAAGTTC
TGTTCTGTTAATGACATGAAAGAGTTGCTGATTGCAAATGGCCAGGAGGTTCCTTCAGGA
GAGTCCAACGTGTTGGACTGCCTGGCCGACGGCATGGCTTTTGGAGCACTGGAGCCCTGT
GGCGAGTGTAGGGGCCAGCTGGTGTTCAAGGGCGACGCTTATTACTGCACAGGCGACATC
TCCGCCTGGACGAAGTGTGTGTACAAGACCACCACCCCCAAACGCAAAGACTGGGTCACC
CCCAAGGAGTTCCATGAAATTTCCTTCCTGAAGAAATTCAAGTTCAAGGGACAGAACAGG
ATTTACCCCAAAGAAGCTGCGCCCCAAACTGTGAGTGCAGTCAAAGCCGAACCCTTAGCC
AGCGCGTCCAGTGTCCCCGTGGGTCTGCCGGAGGGAGCTCCTCCAGACAAACCTCTCACT
GGCATGAAACTGGTGGCTGTGGGCAAGCTGAGCAAGAACAAGGATGATCTGAAGGCTGCC
GTGGAGGAGCTGGGTGGAAAGATCACCGGCGCAGCCAACAAGGCCTCGCTCTGCCTCAGC
TCTAAGAAGGAGTTGGAGAAGATGACCAAAAAAATGGAGGAAGTGAAGGAGGCAGGGGTG
CGCGTGGTCTCCGAGGACTTCCTCACGGATATAAAGTCATCGGGAAAAGCCCTTCAGGAG
CTGGTGTCCCTGCATGCCATCTCTCCCTGGGGGGCAGAGGTCAAAGTGGAGACTCAGGCA
CAGCCCATGGCCTCAAAGTCTGGAGCAATGGCTGCCAAGAGCACCGGCAGGGTGAAGGAA
CAAGAGGGTGGGGGCAAAAGCAAGAAGATGAAGCTGACTGTGAAAGGTGGAGCTGCTGTG
GATCCGGATTCAGGTCTGGAAAACAGCGCCCATGTTCTGGAGCAGGGTGGGAAGATGTAC
AGCGCCACACTGGGTTTGGTGGACATCGTCAGGGGGACTAACTCCTACTACAAACTGCAG

CTGCTGGAGGATGATGTTCAAAAGAGGTACTGGGTGTTCAGGTCATGGGGCAGAGTGGGC
ACCACCATCGGAGGCAACAAGCTGGACAAGTTCAGTGACAGGAACTCTGCCATGGACAAC
TTCCTGACTGTCTACAAGGAGAAGACTGGTAATGACTGGAGCTCCTCCAACTTCACCAAG
TATCCAAATAAGTTCTACCCCCTGGAGATCGACTACGGACAGGACGAGGAAGCAGTGAAG
AAGTTGACGGCCACAGCAGGCACCAAGTCCAAGCTGGCCAAACCCGTGCAAGAGCTGATT
AAGATGATCTTCGACGTGGAGAGCATGAAGAAGGCCATGGTGGAGTTTGAGATTGACCTC
CAGAAGATGCCACTGGGGAAGCTGAGTAAGAGGCAGATCCAGAGCGCGTACGCTCTCCTT
ACTGAAGTCCACCAGGCGGTGTCAGACTCTCTGCCCGAGGCCCAGATCTTGGATCTCTCC
AATCGTTTCTACACTCTGATCCCTCACGACTTCGGTATGAAGAAGCCACCGCTGCTCAGC
AACCTGGACTACATTCAGGCCAAGGTCCAGATGCTGGACAACCTGCTGGACATTGAGGTG
GCGTACAGCCTGCTGAGGGGAGGGGCTCAGGACAACGAGAACGACCCCATCGACATCAAC
TACGAAAAACTCAAAACCAAGATTGAGGTCATTGACAAGTCTGCCAAGGAGGCTGACATT
ATTCTGCAGTATGTCAAGAACACCCACGCTGCCACGCACAACACCTACACTCTGGAAGTG
CAAGAGATCTTCAAAATATGCAGAGAGGGGGAGCACCAGCGTTACCGTCCTTTCGAGGAG
CTGCACAACCGCCAGCTGCTGTGGCACGGCTCTCGCACCACCAACTACGCGGGTATTATG
TCTCAGGGTCTCCGCATCGCCCCGCCGGAGGCTCCAGTGACCGGTTACATGTTTGGCAAA
GGCGTGTACTTTGCCGACATGGTGTCCAAGAGTGCAAACTACTGCCACACCTCCCAGTCG
GAACCAGTGGGGCTCCTTCTGCTGGCTGAGGTCGCCCTCGGCAACATGCATGAACTGAAA
AAGGCCTCACACATTACAAAACTACCCAAAGGAAAACACAGCGTTAAAGGTTTGGGCCGA
ACTGCTCCGGATCCAAATGCTACTGTGACTCTGGACGGAGTACAAGTGCCTCTGGGAAGA
GGAGTCAACACAAACATCGACGACACCAGCCTCCTGTACAACGAGTACATCGTGTATGAC
GTGGCGCAGATCAACCTAAAGTATCTCCTGAAGGTCAGATTTAACTATCAGACATCCCTG
TGG
>African_ostrich_PARP1
ATGTTTGATGGCAAAGTCCCTCACTGGCATCACTACAGCTGCTTCTGGAAGCGGGCTCGA
ATTGTGTCCCATACGGACATCGATGGCTTCCCTGAGCTCCGATGGGAAGATCAGGAGAAA
ATCAAGAAAGCCATTGAAACTGGAGGACCTGGAGGAGGAAAAGGAGGGGACCAGGAAGTA
GGTGGCAAGGCTGAGAAAAGCCTAAACGACTTTGCTGCAGAATATGCCAAGTCTAACAGA
AGTACTTGCAAAGGCTGCGAACAGAAAATAGAAAAGGGCCAGATCCGAATTTCCAAGAAG
ATGGTGCATCCTGAAAAGCCGCAGCTGGGAATGATAGATAACTGGTACCACCCGGACTGT
TTTGTGAGCCGCCGAGCAGAGTTGGGCTTCCTCCCGACGTACGGGGCCGCTCAGCTCTTG
GGCTTCGGGATCTTGAAAGCTGAAGATAAAGAAACTCTGAAGAAGCAGCTTCCAGCTACC
AAGAGCGAAGGAAAGAGAAAAGGAGAAGAGGTAGATGGAAATGCAACTGCGAAAAAGAAA
CAGAAAAAAGAAAAAGAGAAAGAATCAAAGCAGGAGAAACTGCTGAAGGAGCAGACGGAG
CTGATCTGGGGCATCAAGGATGAGCTGAGGAAGGTCTGCTCCACTAACGACCTGAAAGAG
CTGCTGATCGCCAACAAGCAGGAAGTGCCGTCTGGGGAGAATGCTATTTTGGACCGAGTA
GCAGATGGGATGGCATTTGGAGCTCTGCTTCCCTGCGAAGAGTGTAAGGGGCAGTTTGTC
TTCAAGAGTGATGCATATTACTGTTCAGGGGATATTACTGCCTGGACTAAGTGTGTTGCT
AAAACACAGACACCCAACAGGAAAGACTGGATAATCCCAAAGGAGTTCCGGGAAATCCCC
TATCTCAAGAAATTTAAGTGTAAAAAGCAGGACAGGGTATTCCCTCCAGAGGCTGCAACT
ATGAACTCTGTGCCTCCTCCGTCTGCGTCTGCTCCTTTGACAGAAACCGTGTCCACACCC
AGAGATAAACCACTGACCAACATGAAGATCTTGGTTCTCGGAAAGCTGTCCAAAAACAAG
GAGGAGGTGAAGAGCATTGTGGAGGACCTGGGAGGAAAGATGACGGGAAGCGCTAACAAG
GCCACCCTGTGCATCAGCACACCAAAAGATATAGAGAAAATGAACAAGAAGATGGAAGAA

GTGAAGGAAGCCAAAGTCCGCGTGGTCTCAGAGGAGTTTCTTCAGGATGTGAAATCTTCC
AGCAAGGGCTTTCAGGAGCTTCTCTCTCTCCATGCGCTTTCGCCCTGGGGTGCAGAGGTG
AAAATGGAGCACCAGGAGGTCTCTGTAGATGGGAGGTGCAGCAAACCCTCAAATACAAAG
AGTGCTGGGAAAGTCAAAGAAGAACAAGGTCCCAGCAAGTCTGAAAAGAAAATGAAGCTA
ACGGTTAAAGGCGGAGCGGCAGTAGATCCTGATTCTGGTTTGGAGGATTCTGCTCATGTC
TTTGAAAAAGGTGGAAAGATTTTCAGTGCAACCCTTGGGCTAGTAGATATTGTGAAAGGA
ACAAATTCCTATTATAAACTTCAACTGCTAGAGGATGACAGGGAGAGCAGATACTGGGTG
TTCAGATCTTGGGGTCGCGTGGGCACTGTGATCGGGAGTAACAAGCTGGAGCAGATGCCA
TCAAAAGAGGACGCTGTTGAGCACTTTCTGAATTTGTACGAAGAGAAAACTGGCAATTCT
TGGCATTCAAAGAACTTCACTAAATATCCAAAAAAATTTTACCCACTGGAAATTGATTAT
GGACAGGACGAAGAAGCTGTGAGGAAACTGACAGTGGGTGCTGGGACTAAATCCAAACTT
GCTAAGCCGATCCAGGAGCTTATTAAGATGATCTTTGATGTGGAGAGCATGAAGAAAGCG
ATGGTGGAATTTGAGATTGACCTCCAAAAGATGCCGCTGGGGAAACTGAGCAAGCGACAG
ATTCAGAGCGCGTACTCCATCCTTAATGAGGTTCAGCAGGCAGTTTCTGACAGTGGTTCT
GAATCCCAGATCTTGGACCTCTCCAACCGCTTCTATACACTGATTCCTCATGACTTTGGG
ATGAAGAAACCACCTCTCCTAAATAATTTAGAATACATTCAGGCTAAAGTGCAGATGCTG
GACAACCTGCTTGATATTGAGGTTGCCTACAGCCTTCTCAGAGGTGGAAATGAAGATGGA
GATAAAGACCCAATTGACATCAACTACGAAAAGCTCCGAACTGATATTAAGGTTGTTGAC
AAAGATTCAGAAGAAGCCAAGATTATAAAACAATATGTGAAAAATACTCATGCTGCTACT
CACAATGCATATGACCTCAAAGTTGTGGATATCTTCAGGATTGAGCGTGAAGGAGAGAGT
CAGCGGTACAAGCCATTTAAGCAGCTTCATAATCGCCAACTGCTGTGGCACGGTTCCCGA
ACTACCAACTTTGCTGGTATCCTCTCGCAGGGTCTCCGGATAGCTCCCCCTGAAGCTCCT
GTGACCGGCTACATGTTTGGAAAGGGCATCTATTTTGCAGACATGGTATCCAAGAGTGCC
AATTACTGTCACACATCTCAAGCTGATCCCATAGGCCTAATACTACTGGGAGAAGTTGCC
CTTGGAAATATGTACGAGCTAAAGAATGCTTCTCACATAACGAAGTTGCCGAAGGGAAAA
CACAGTGTGAAAGGCTTGGGCAAAACTGCACCTGATCCTACAGCCACTACCACCCTTGAT
GGTGTAGACGTTCCCTTAGGGAACGGGATTTCCACTGGAATTAATGATACCTGTCTTCTG
TATAACGAATATATCGTGTATGATGTTGCTCAGGTAAATCTGAAGTACCTGCTGAAACTG
AAATTCAACTATAAGACATCACTCTGG
>Adelie_penguin_PARP1
ATGTTTGATGGCAAAGTCCCTCACTGGCACCACTACAGCTGCTTCTGGAAGAGGGCTCGA
ATCGTGTCCCACACGGACATCGATGGCTTCCCTGAACTCCGGTGGGAAGATCAGGAAAAA
ATCAAGAAAGCCATTGAAACTGGAGGCCCTGGAGGAGGAAAAGGAGGGGAACAGGAAGGA
GGTGGCAAGGCTGAGAAGAGCCTAAATGACTTTGCTGCAGAATACGCCAAGTCTAACAGA
AGTACTTGCAAAGGCTGTGAACAGAAAATAGAAAAGGGCCAGATCCGGATTTCCAAGAAG
ATGGTGCATCCTGAAAAGCCACAGCTGGGAATGATAGATAACTGGTACCACCCGGACTGC
TTTGTGAGCCGCCGAGCAGAGCTGGGCTTCCTCCCAGCGTATGGGGCTGCCCAGCTCCTG
GGCTTCAGCATCTTGAAAGCTGAAGATAAAGAAACTTTGAAGAAGCAGCTCCCAGCTACC
AAAAGTGACGGAAAGAGAAAAGGAGAAGAGGTAGATGGAAATGTGACTGCAAAAAAGAAA
CAGAAAAAAGAAAAAGAGAAAGAATCAAAGCAGGAAAAACAGCTGAAGGAGCAGACAGAG
CTGATCTGGGGCATCAAGGATGAGCTGAGGAAGGTCTGCTCCACTAACGACCTGAAAGAG
CTGCTGATCGCCAACAAGCAGGAAGTGCCTTCAGGGGAGAATGCCATTTTGGACCGAGTA
GCAGATGGGATGGCATTTGGAGCTCTGCTTCCCTGCGAGGAGTGTAAGGGGCAGTTTGTG
TTCAAGAGTGATGCATATTACTGTTCAGGGGATATTACTGCCTGGACTAAGTGTGTTGCT

AAAACGCAGACTCCCAACAGGAAAGACTGGGTAATCCCAAAGGAGTTTCGGGAAATTCCT
TACCTGAAGAAATTTAAATGTAAGAAGCAAGACAGGGTATTCCCTCCAGATGCTGCGACT
GTGAACTCTGTGCCTCCTCCCTCCGCATCTGCTCCTTTGACAGAGACCGTGTCTGCACCC
AGAGACAAACCACTGACCAACATGAAGATCCTGGTTCTTGGAAAGCTGTCAAAGAACAAG
GAGGAGGTGAAGAGCATTGTGGAGGAGCTGGGAGGAAAGATGACAACAACAGCTAACAAG
GCCACCCTGTGCATCAGCACACAGAAGGATGTGGAGAAGATGAGCAAGAAGATGGAAGAA
GTGAAGGAGGCCAAAGTCCGTGTGGTCTCAGAGGAGTTTCTTCAGGATGTGAAATCCTCC
AGCAAGGACTTTCAGGAGCTTGTGTCTCTCCATGCACTTTCGCCTTGGGGTGCGGAGGTG
AAAATGGAGCACGAGGAAATGGCCGTGGATGGGAAATGCAGCAAGCCCCCAAGTATGAAG
AGTGCTGGGAAGGTCAAAGAAGAACAAGGACCTAGCAAGTCTGAAAAGAAAATGAAGCTA
ACAGTTAAAGGTGGAGCAGCAGTAGATCCTGATTCTGGTTTGGAGGATTCTGCTCACGTC
TTTGAAAAAGGTGGAAAGATTTTCAGTGCAACCCTGGGCCTAGTAGATATTGTGAAAGGA
ACAAATTCCTATTACAAACTGCAGCTGCTAGAGGATGACAGAGAGAACAGATACTGGGTG
TTCAGATCTTGGGGTCGCGTGGGCACTGTAATTGGGAGTAACAAGCTGGAGCAGATGCCC
TCAAAAGAAGATGCTGTTGAACACTTCCTGAATTTGTATGAAGAGAAAACTGGCAATTCT
TGGCATTCAAAGAACTTCACTAAATATCCCAAAAAATTCTACCCGCTGGAAATAGATTAT
GGGCAGGATGAAGAAGCTGTCAGGAAACTGACAGTGGGTGCCGGGACTAAGTCAAAACTC
GCTAAGCCAATCCAAGACCTGATTAAGATGATCTTTGATGTGGAGAGCATGAAGAAAGCG
ATGGTGGAATTTGAGATTGACCTGCAGAAGATGCCATTGGGAAAACTGAGCAAGCGACAG
ATCCAGAGCGCATACTNNNNNNNNNNNNNNNNNNNNNAGGCAGTTTCTGACAGTGGTTCA
GAATCCCAGATCTTGGACCTCTCCAACCGCTTCTATACTCTGATTCCTCATGACTTTGGG
ATGAAGAAACCACCTCTCCTAAATAACTTGGAATACATTCAGGCTAAAGTGCAAATGTTG
GACAACTTGCTTGATATTGAGGTTGCTTACAGCCTTCTCAGAGGTGGAAATGAAGATGGA
GATAAAGACCCAATTGACATCAACTATGAAAAACTTCGAACTGATATTAAGGTTGTTGAC
AAAGATTCAGAAGAAGCCAAGATTATTAAACAATATGTGAAAAATACTCATGCCGCTACT
CACAACGCATATGATCTCAAAGTCGTGGATATCTTCAGAATTGAGCGTGAAGGAGAGAGT
CAGCGTTACAAGCCATTTAAGCAGCTTCATAATCGCCAGCTGCTGTGGCACGGTTCCCGC
ACCACCAACTTTGCTGGTATCCTCTCGCAGGGTCTCCGGATAGCTCCCCCTGAAGCTCCT
GTGACCGGCTACATGTTTGGGAAGGGCATCTATTTTGCAGACATGGTATCCAAGAGTGCC
AACTACTGTCACACATCTCAAGCTGATCCCATAGGCTTAATACTACTGGGAGAAGTTGCC
CTTGGAAATATGTATGAGCTAAAGAATGCTTCTCACATAACAAAATTGCCTAAGGGAAAA
CACAGTGTGAAAGGCTTGGGCAAAACTGCACCTGATCCCACAGCCACTACCACCCTTGGT
GGTGTAGAGATTCCCTTAGGGAATGGGATCTCAACAGGAATTAATGATACCTGTCTCCTG
TATAATGAATATATTGTGTATGATGTTGCTCAGGTAAATCTGAAGTACCTGCTGAAACTG
AAATTCAACTATAAGACATCACTCTGG
>Gecko_PARP1
ATGGCGGAGCCTGCTGAGAAGCTGTACCGGGCCGAGTATGCTAAGAGCGGCCGGGCTTCC
TGCAAGAAATGTGGAGACAACATCGCCAAGGATTCGCTGCGCCTGGCCATCATGGTGCAG
TCCCCCATGTTTGATGGCAAAGTACCCCACTGGCACCATTACTCCTGCTTCTGGAAGCGG
GCACGACTTGTGTCTCACACTGATGTAGATGGCTTCTCTGAACTGCGATGGGACGATCAA
GAGAAAATTAAAAAAACCATAGAGACTGGTGGCGCTGCAACAGGTAAAGGTGGTGACCAA
GAGGGAGGCAGCAAAGCTGAAAAAAGTTTAAATGATTTTGCAGCAGAATATGCAAAGTCC
AACAGAAGTACCTGCAAAGGCTGTGAACAGAAAATAGAAAAGGGCCAGATTAGAATTTCA
AAGAAAATGGTGCACCCTGAAAAGCCCCAACTGGGAATGATAGACAATTGGTACCACCCA

GCCTGTTTTGTCAGTCGCAGAGCAGATCTGGGTTTTCTCCCTACTTTTAGCGCTTCCCAG
CTACTAGGCTTTGGGTCGCTGAGTGCTGAAGATAAAGAGACTCTGAAGAAGCAGCTGCCA
GCTGTCAAGAATGATGGAAAGAGAAAAGGAGATGAGGTGGATAGCAATGAGATCTCAAAA
AAGAAGCCAAAAAAGGAAAAAGAAAAACAGTCCAAGCAGGAGAAGCTGCTGAAGGAACAG
ACAGAGTTGATTTGGAACATCAAAGATGAATTGAAGAAAGCCTGCTCCATTAATGACCTG
AAAGAGCTCCTGATAGCAAACAAACAAGAAGTTCCTTCTGGGGAATCAGCCATCCTGGAC
CGTGTTGCGGATGGGATGGCCTTTGGGGCTCTGCTGCCATGTGAGGAATGCAAAGGGCAG
CTTGTGTTCCGGAGTGATGCATACTACTGTACAGGGGATATTACAGCTTGGACTAAATGT
GTTGCCAAGACGCAGACTCCCGACAGGAAAGAATGGATAATCCCAAAGGAGTTCCGGGAA
ATCACATATCTAAAGAAATTTAAATTTAAAAGGCAGGACAGAGTATTTGCCCCAGAGGCT
GCCTCTGCAAACTCAGTACCTCCTCCAAAAGTTTCTGTTCCAGTTACAGAAAACTCTGCT
GCTCCAGCAGATAAACCTTTATGTAACATGAAGGTCGTGACCCTTGGGAAATTGTCGAAG
AATAAAGATGAAATCAAGTCTACTATTGAAGAGCTCGGAGGAAAGGTGACAGCTTCTGTG
AATAAAGCTAACCTGTGCGTCAGCTCACAAAAGGAAGTTGAGAAAATGAGCAAGAAAATG
GAAGAAGTGAAGGAGGCACAAGTGCGTGTGGTGTCAGAGGAGTTTCTCCAGGACATAAAG
TCCTCCAGCAAGAGCCTTGAGGAGCTCCTGTCACTTCATGCTCTTTCCCCCTGGGGCACA
GAAGTAAAACAAGATCACAAAGAAGTGTCCATAGGAGGAAAATCCAGTGGGCATTCAAAC
ACAAAAAGCACTGGGAAAAGCAAGGAAGAGCAAGGAACCAGCAAGTCAGAAAAGAAAATG
AAGCTGACAGTGAAAGGAGGTGCTGCTGTGGACCCTGACTCAGGCTTAGAAGATTCTGCT
CACGTCTTTGAAAAAGGTGGCAAGATCTTCAGCGCCACTCTAGGCCTGGTAGATATTGTT
AAAGGAACCAACTCCTATTACAAACTTCAGCTGTTGGAAGATGACAGGGAAATCAGATAC
TGGGTGTTCCGTTCCTGGGGCCGTGTTGGCACAGTAATTGGAAGCAACAAGCTAGAGCAG
ATGCCATCTAAAGAGGAAGCCATTGAACACTTTCTGAATCTGTATGAAGACAAGACTGGC
AACTCCTGGCATTCTACAAATTTCACCAAATATCCCAAAAAATTCTACCCTCTGGAAATA
GACTATGGACAGGATGAAGAAGCGGTAAAGAAACTGACAGTTAGTGCAGGAACCAAATCA
AAGCTTCCAAAGCCAGTCCAGGATCTCATTAAGATGATCTTTGATGTGGAAAGCATGAAG
AAAGCCATGGTGGAATTTGAGATTGATCTCCAGAAGATGCCTTTGGGGAAACTAAGCAAG
AGGCAGATCCAGAGTGCATACTCCATCCTTAACGATGTGCAGCAGGCAGTTTCTAATGGT
GGAACAGATTCACAGATACTGGATCTCTCCAATCGCTTTTACACACTGATTCCTCATGAC
TTTGGGATGAAGAAGCCTCCTCTTTTAAACAACCTAGAATACATTACGTCCAAAGTGGAG
ATGCTGGACAACCTGCTTGACATTGAAGTTGCCTACAGCCTACTCAGAAGTGGAGGTCAG
GATGGGGATAAAGATCCAATAGATGTGAACTATGAAAAACTTAAAACCGATATCAAGGTA
GTAGACAAAAATTCAGAAGAAGCCAAGATCATAAAGCAATATGTTAAGAATACTCATGCC
AGTACTCACAATGCTTATGACTTGAAAGTTGTGGAGATTTTCAAGATTGAACGTGAAGGA
GAATATCAGCGTTATAAACCATTCAAAGAGCTACACAACCGGCAGCTACTCTGGCATGGT
TCTCGCACTACCAATTTTGCTGGTATCTTGTCACAGGGTCTCCGAATAGCTCCACCTGAG
GCCCCTGTGACTGGCTACATGTTTGGTAAAGGCATCTATTTTGCGGATATGGTGTCCAAG
AGTGCCAACTATTGTCACACCTCTCAAAATGACCCAGTAGGCTTAATCTTGCTGGGAGAA
GTTGCCCTTGGAAATATGTATGAAATGAAGAATGCTTCCCATATCACCAAGGTGCCGAAA
GGAAAACACAGTGTGAAAGGCTTGGGCAAAACTGCACCAGATCCCTCAGCCACTATCTCT
CTCGATGGCATAGACGTTCCTTTAGGCAAAGGAATACCATCAGGAATTAGTGACACCTGT
CTTCTATATAATGAATATATTGTCTATGATATTGCTCAGGTAAACTTGAAGTACCTGCTG
AAGTTGAAATTCAACTATAAGACATCTCTCTGG
>green_sea_turtle_PARP1

ATGTATGGCATCAAGAAGGCGGAGGTGTTTAATACCTATTTTACTTCAGTCTTCACTGAA
AAGTCACCCATGTTTGATGGTAAAGTCCCCCATTGGCATCATTATACTTGTTTCTGGAAG
CGGGCCCGACTCGTGTCCCATGCAGATGTTGATGGCTTCCCTGAGCTCCGATGGGAAGAT
CAGGAGAAAATCAAAAAAGCAATTGAAACTGGGGGAGCCGCAGCAGGTAAAGGCATTGAC
CAGGAGGGAGGTGGCAAGGCTGAGAAGAGTTTAAATGACTTCGCTGCAGAATATGCCAAG
TCTAACAGAAGTACCTGCAAAGGTTGTGCACAGAAAATAGAAAAGGGGCAGATCAGAATT
TCCAAGAAGATGGTGCATCCCGAAAAGCCCCAACTGGGAATGATAGATAATTGGTACCAT
CCAAACTGCTTTGTGAGCCACCGAGCAGAACTGGGCTTTCTCCCTGCATTTGGAGCCAGT
CAGCTCCAGGGCTTTGGGATGTTGAAAGCAGAGGATAAAGAAGATTTGAAAAAGCAGCTG
CCTGCCATGAAGAGTGAAGGAAAGAGAAAAGGAGATGAAGTGGACGGAAATGTGACCTCA
AAAAAGAAACAGAAAAAAGAAAAAGACAGAGAATCCAAGCAGGAGAAGCTGTTAAAGGAG
CAGACAGAGTTGATTTGGAGCATCAAAGACGAGCTGAGAAAAGTCTGTTCCACTAATGAC
CTGAAAGAGCTGCTGATAGCCAACAAACAGGAAGTGCCTTCTGGGGAATCTGCTCTCTTG
GACCGAGTAGCAGATGGGATGGCATTCGGAGCTCTGCTTCCCTGTGAGGAGTGCAAGGGG
CAGTTTGTGTTCAAGAGTGATGCATACTACTGCTCAGGGGATATCACTGCCTGGACGAAA
TGTGTTGCCAAAACACAGACTCCCAACAGGAAGGAATGGATAATCCCAAAGGAGTTCCGG
GAAATCTCCTACCTGAAGAAATTTAAGTGTAAAAAGCAAGACAGAGCATTCCCTCCAGAG
TCTGTGGCTGTGAATGCAGTGCTTCCAGCAACAGCTTCTGCTCCTTTGACAGAGAAGGCG
TCTGTGCCAGCAGATAAGCCATTATCCAGCATGAAGATTTTGACCCTTGGAAAATTATCC
AGGAACAAGGAAGAAGTGAAGGCTATAATTGAGGACCTGGGCGGAAAGGTGACAGGGACA
GCAAACAAGGCTACCCTGTGCATCAGCACACAAAAGGAAATTGAGAAAATGACCAAGAAG
ATGGAAGAAGTGAAGGAGGCCAAAGTCCGAGTGGTCTCAGAACAGTTTCTGCAGGATGTG
AAATCCTCCAGCAAGGGCTTCCAGGAGCTCCTGTCGCTGCATGCGCTCTCGCCTTGGGGT
GCAGAGGTGAAGCAGGAGCACACGGAGACGTCCCTGGGAGGAAAGTCCAGTGGGCTCCCA
AACATGAAGAGTGCTGGGAAGGTCAAGGAAGAGCAAGGAACCAGCAAGTCTGAAAAGAAA
ATGAAGTTAACAGTTAAAGGAGGAGCAGCAGTGGATCCTGATTCAGGTTTGGAGGACTCT
GCTCATGTCTTTGAAAAGAGTGGTAAAATATTCAGTGCCACGCTCGGCTTGGTAGATATT
GTCAGAGGAACCAATTCCTATTATAAACTGCAGCTACTGGAGGATGACAGAGAGATCAGG
TACTGGGTTTTCAGATCTTGGGGTCGTGTTGGCACTGTGATTGGGAGTAACAAACTGGAG
CAGATGCCGTCTAAAGATGATGCCATTGAGCACTTCTTGAATTTGTATGAAGAGAAAACG
GGCAACTCGTGGCATTCCAAGAACTTCACTAAATATCCAAAAAAATTCTACCCTCTGGAA
ATAGACTATGGACAGGATGAAGAAGCTGTGAGGAAACTGACCGTTAGTGCTGGGACCAAG
TCAAAGCTTCCTAAGCCAGTCCAGGACCTTATTAAGATGATCTTTGATGTGGAGAGCATG
AAGAAAGCCATGGTGGAATTTGAGATTGACCTCCAGAAGATGCCACTGGGAAAGCTGAGC
AAGAGGCAAATCCAGAGTGCATACTCCATCCTTCATGACAAGAGACAGGCAGTGTCTGAT
GGTGGCAGTGATTCTCAGATACTGGATCTCTCCAATCGCTTCTATACCCTGATACCTCAT
GACTTTGGGATGAAGAAACCACCTCTCTTAAATAACCTAGAACATATCCAGGCCAAAGTG
CAGATGTTAGACAACCTGCTTGATATTGAGGTCGCTTACAGCCTTCTCAGAGGTGGAAAT
GAAGATGGGGATAAAGACCCAATTGACATCAACTACGAAAAGCTCAAAACTAACATTAAG
GTTATTGATAAAGATTCAGAAGAAGCCAAGATCATAAAGCAGTATGTGAAGAATACACAT
GCCTCTACCCATAATGCATATGATCTGAAAGTTGTGGATATCTTCAAGATTGAACGTGAA
GGAGAAAATCAGCGTTACAAACCGTTCAGGCAGCTTCATAATCGCCAGCTGCTCTGGCAT
GGCTCCCGCGCTACCAACTTTGCTGGCATCTTCTCGCAGGGTCTCCGAATAGCTCCACCT
GAAGCTCCTGTGACTGGTTACATGTTCGGTAAAGGTATCTATTTTGCGGACATGGTGTCC

AAGAGTGCCAACTACTGTCACACATCTCAGACTGATCCAATAGGCTTAATTTTACTGGGA
GAGGTTGCCCTTGGAAACATGCATGAGCTAAAGAATGCTTCCCACATAACTAAGTTGCCC
AAGGGAAAACACAGTGTCAAAGGTTTGGGCAAAACTGCACCTGATCCTTCAGCCACCATC
ACTCTAGATGGTGTAGATATTCCTTTAGGGAATGGGATTCCATCTGGAATTAGTGATACC
TGTCTTCTATATAATGAATATATTGTCTATGATGTTGCTCAGGTAAATCTGAAGTACCTG
CTGAAACTGAAGTTCAACTATAAAACATCACTCTGG
>Chinese alligator_PARP1
ATGGACACTCTCAAAAACATTGGAAACAGCAGCCTCAAGAGCAGCTGTGAGGGGAACACC
AGAGCAGTTTCACTATCGTACTTCTGGTTTGGGACGGAGTGTGAGCGGGGGCGAGGCGGG
TGGGCGGAGCCGGCGGACAAGCTGTACCGGGCCGAGTACGCCAAGAGTGGGCGGGCCTCG
TGCAAGAAATGCGGGGAGAGCATCGCCAAGGACTCGCTGCGTCTGGCCATCATGGTGCAG
TCACCCATGTTCGATGGCAAAGTCCCTCACTGGCATCATTACACTTGTTTTTGGAAGCGG
GCTCGGATCACGTCTCATGCAGATATTGATGGCTTCCCTGAGCTGCGATGGGAAGATCAG
GAGAAAATTAAAAAAACCATTGAAGCAGGGGGACCTGCCACAGGTAAAGGTGGTGACCAG
GAAGGAGGTGGCAAGGCTGAGAAAAGCTTACATGACTTTGCAGTAGAATATGCCAAGTCT
AACAGAAGTACCTGCAAAGGCTGTGAACTGAAAATAGAAAAGAGTCAGATCAGAATTTCT
AAGAAGATGGTGCATCCGGAAAAGCCCCAGCTGGGAATGATAGATAACTGGTACCATCCA
GACTGCTTTGTGAGCCGCCGAGCAGAGCTGGGCTTTCTCCCTGCATTTGGTGCCAGTCAG
CTCCAGGGCTTTGGCATTTTGGTAGCTGAGGATAAAGAATCCCTGAAGAAGCAGCTGCCT
GCCGTCAAGAGTGAAGGAAAAAGAAAAGCCGATGAGATGGATGGGACTGTGACTACAAAA
AAGAAACAAAAAAAAGAAAAGGATAAAGAATCCAAACAGGAGAAGCTGCTGAAGGAACAG
ACAGAATTGATTTGGAAGATCAAAGACGAGCTGAGGAAAGCCTGCTCCACCAACGACCTG
AAAGAGCTGCTGATAGCCAACAAACAGGAAGTGCCTTCTGGGGAATCTGCTATCTTGGAC
AGAGTGGCAGACGGGATGGCATTTGGAGCTCTGCTTCCCTGTGAAGAGTGCAAGGGGCAG
TTTATGTTCAAGAGTGACGCGTACTACTGCTCAGGAGACATTACTGCCTGGACTAAATGT
GTTGCCAAAACACAGTCTCCCAAAAGGAAAGAATGGGTTATCCCAAAGGAATTCCGGGAA
ATCCCTTACCTAAAGAAATTCAAGTGTAAAAAGCAGGACAGAGTATTCCCTCGAGAGGCT
GCTGCTGTGAACACTGTGCTTCCAAGTGCAGCTTCTGCTCCTTTGACAGAGGAGGCATCT
GCACCCACAGATAAGCCATTATCCAACATGAAGATTTTGATACTTGGAAAATTATCCAGG
AACAAAGAAGAAATGAAGACCACAGTTGAGGAGCTTGGAGGAAAAGTGACAGGAACCGCC
AATAAGGCCAACCTGTGCATCAGCACACAAAAGGAGGTTGAGAAAATGAACAAGAAGATG
GAAGAAGTGAAGGAGGCAAAAGTCCGAGTGGTTTCAGAAGAGTTTCTCAAGGATGTGAAA
TCCTCCAGCAAAGGCTTTCGGGAACTGCTGTCAGTACATGAGCTCTCATCCTGGGGTGCA
GAGGTGAAGCAGGAGAACATGGAGACAACTGTGGGAGGAAAGTCCAGTGGGCCCCCAAAT
ATGAAGAGTGCTGGAAAAGTCAAGGAAGAACAAGGGACCTGCAAGTCTGAAAAGAAAATG
AAATTGACAGTTAAAGGAGGAGCAGCCGTAGATCCTGATTCTGGTTTGGAGGACTCCGCT
CACGTCTTTGAAAAAGGTGGTAAAATCTACAGTGCCACTCTTGGCCTGGTAGATATTGTC
AAAGGAACCAATTCCTATTACAAACTGCAGCTGCTGGAGGATGACCGAGAAATCAGGTAC
TGGGTGTTTAGGTCATGGGGTCGCGTTGGCACTGTGATTGGGAGTAACAAACTGGAGCAG
ATGCCATCTAAAGAAGATGCCATTGAGCACTTTTTGAATCTGTATGAAGAGAAAACGGGC
AATTCCTGGCATTCCAAGAACTTCACAAAATATCCAAAGAAATTCTACCCTCTAGAAATA
GATTATGGACAGGATGAAGAAGCTGTGAAGAAACTGACAGTAGGTGCCGGCACAAAGTCA
AAGCTTCCTAAATCAGTCCAGGACCTTATTAAGATGATCTTTGATGTGGAGAGCATGAAG
AAAGCCATGGTGGAATTTGAGATTGACCTCCAGAAGATGCCATTGGGAAAGCTGAGCAAG

AGACAGATCCAGAGTGCCTATTCCATCCTTAATGATGTTCAGCAGGCAGTTTCTGGTGGC
GGCACTGATTCTCAGATACTGGACCTCTCCAATCGCTTCTACACATTGATACCTCATGAC
TTTGGGATGAAAAAGCCACCTCTTTTAAATAACCTAGAATATATTCAGGCCAAAGTGCAG
ATGTTGGACAACCTGCTTGATATTGAGGTTGCTTACAGCCTTCTCAGAGGTGGAAATGAG
GATGGGGATAAAGACCCAATTGATGTCAACTATGAAAAGCTCAAAACTGAAATTAAGGTG
GTTGATAAAGATTCAGAAGAAGCCAAGATCATAAAGCAATATGTGAAGAATACCCATGCT
GCTACCCACAATGCATATGATCTGAAAGTTGTGGATATCTTCAAAATTGAGCGTGAAGGG
GAGAGTCAGCGTTACAAGCCATTCAGACAGCTTCACAATCGCCAGCTGCTCTGGCATGGC
TCACGTACTACCAACTTTGCTGGTATTCTCTCACAGGGTCTCCGAATAGCTCCGCCTGAA
GCTCCTGTGACTGGTTACATGTTCGGAAAAGGTGTCTATTTTGCAGACATGGTTTCCAAG
AGCGCCAACTACTGTCACACATCTCAGGCTGATCCAGTAGGCTTAATCTTATTGGGAGAG
GTTGCCCTTGGAAACATGTATGAGCTAAAGAATGCTTCTCACATAACTAAGCTGCCGAAG
GGGAAACATAGCGTCAAAGGTTTGGGCAAAACTGCACCTGATCCCACAGCCACCATCAGT
TATGAGGGTGTAGAAGTTCCTTTGGGGAATGGAATGTCAACAGGAATTAATGATACTTGT
CTTCTGTATAATGAATATATTGTCTACGATGTTGCTCAGGTAAACCTGAAGTACCTGCTG
AAACTGAAATTCAACTACAAGACATCACTCTGG
>Mouse_PARP1
ATGGCGGAGGCCTCGGAGAGGCTTTATCGAGTGGAGTACGCGAAGAGCGGGCGCGCCTCT
TGCAAGAAATGCAGCGAGAGTATTCCCAAGGACTCCCTCCGCATGGCCATCATGGTGCAG
TCACCCATGTTCGATGGGAAAGTCCCACACTGGTACCACTTCTCCTGCTTCTGGAAGGTG
GGCCACTCCATCCGGCAGCCTGATGTTGAGGTGGATGGCTTCTCTGAGCTGCGCTGGGAT
GATCAGCAGAAGGTCAAGAAGACGGCCGAGGCTGGAGGCGTGGCAGGCAAAGGCCAGGAT
GGAAGTGGCGGCAAGGCGGAGAAGACATTGGGTGACTTTTTAGCGGAGTACGCCAAGTCC
AACAGGAGCATGTGCAAGGGCTGCCTGGAGAAGATAGAGAAGGGCCAGATGCGCCTGTCC
AAGAAGATGGTGGATCCAGAGAAGCCACAGCTGGGTATGATTGACCGCTGGTACCATCCA
ACTTGCTTTGTCAAGAAGCGGGACGAGCTGGGCTTCCGGCCTGAGTACAGTGCCAGTCAG
CTCAAGGGCTTTAGCCTCCTCTCTGCAGAAGACAAAGAAGCTCTGAAGAAGCAGCTCCCG
GCCATCAAGAATGAAGGAAAGAGAAAAGGTGACGAGGTGGATGGAACAGATGAAGTGGCC
AAAAAGAAATCTAAGAAAGGGAAGGACAAGGATAGTAGTAAGCTGGAGAAGGCCCTCAAG
GCTCAGAATGAGCTGATCTGGAATATCAAAGACGAGCTGAAGAAAGCGTGTTCCACCAAC
GACCTGAAGGAGCTGCTCATCTTCAACCAGCAGCAGGTGCCGTCAGGAGAGTCAGCGATC
TTGGACAGAGTTGCTGACGGCATGGCGTTTGGGGCCCTTCTGCCCTGCAAGGAGTGTTCA
GGCCAGCTGGTCTTTAAGAGCGACGCTTATTACTGTACTGGGGATGTCACTGCCTGGACC
AAGTGCATGGTCAAGACACAGAATCCTAGCCGAAAGGAATGGGTAACTCCAAAGGAATTC
CGAGAAATATCCTACCTCAAGAAGTTAAAGGTCAAAAAACAGGACCGAATATTCCCTCCA
GAAAGCAGCGCCCCAGCACCACTGGCACTGCCCCTCTCTGTCACCTCAGCACCCACAGCT
GTGAACTCCTCTGCTCCAGCAGACAAGCCCCTGTCTAACATGAAGATCCTGACTCTTGGG
AAGCTCTCCCAGAACAAGGACGAAGCAAAAGCTGTGATTGAGAAACTCGGAGGCAAGTTG
ACAGGATCTGCCAACAAGGCCTCCTTGTGTATCAGCACTAAAAAGGAGGTGGAGAAGATG
AGTAAGAAGATGGAGGAAGTGAAAGCGGCCAACGTTCGAGTTGTGTGTGAGGACTTCCTC
CAGGACGTGTCTGCCTCCACTAAAAGCCTCCAAGAGCTGCTCTCGGCCCACAGCTTGTCC
TCGTGGGGGGCTGAGGTGAAGGCAGAGCCTGGTGAAGTGGTGGCCCCCAAGGGGAAGTCA
GCTGCACCCTCCAAGAAGAGCAAGGGTGCTGTCAAGGAGGAAGGTGTCAACAAATCTGAA
AAGAGGATGAAATTAACTCTGAAGGGAGGAGCAGCCGTTGATCCTGACTCTGGTCTGGAA

CACTCTGCACACGTCCTGGAGAAAGGTGGGAAGGTGTTCAGCGCCACACTTGGCCTGGTG
GACATTGTGAAAGGGACGAACTCCTATTACAAACTGCAGCTTCTGGAGGACGACAAGGAG
AGCAGGTACTGGATCTTCCGGTCCTGGGGCCGGGTGGGCACAGTTATCGGCAGTAACAAA
CTTGAGCAGATGCCCTCCAAAGAGGACGCTGTTGAGCACTTCATGAAGCTGTATGAAGAG
AAGACTGGGAATGCCTGGCACTCGAAAAACTTCACAAAGTATCCCAAGAAGTTCTACCCT
CTGGAGATTGACTATGGCCAGGACGAAGAGGCAGTAAAGAAGCTGACGGTGAAGCCTGGC
ACCAAGTCGAAGCTGCCGAAGCCAGTGCAGGAGCTCGTGGGGATGATCTTCGACGTGGAG
AGCATGAAAAAGGCCTTGGTGGAGTACGAGATTGACCTCCAGAAGATGCCCTTGGGGAAG
CTGAGCAGAAGGCAGATCCAGGCCGCCTACTCTATCCTCAGCGAGGTCCAGCAGGCAGTG
TCTCAAGGCAGCAGTGAATCCCAGATCCTAGATCTCTCCAATCGCTTCTACACTCTGATC
CCCCATGACTTTGGAATGAAGAAGCCCCCACTCCTGAACAACGCAGACAGCGTGCAGGCC
AAGGTGGAGATGCTAGACAACCTCCTGGACATCGAGGTGGCCTATAGTCTTCTCAGGGGT
GGCTCTGACGACAGCAGCAAGGATCCCATCGACGTCAACTACGAGAAACTCAAAACTGAC
ATTAAGGTGGTTGACAGAGATTCTGAAGAGGCCGAGGTCATCAGGAAGTACGTGAAGAAC
ACTCATGCTACCACGCACAACGCCTATGACCTGGAAGTGATCGATATCTTCAAGATAGAG
CGCGAGGGGGAGAGCCAGCGCTACAAGCCCTTCAGGCAGCTTCACAACCGGAGGCTGCTG
TGGCACGGCTCCAGGACCACCAACTTTGCTGGCATCCTGTCGCAGGGTCTGCGGATAGCC
CCACCTGAAGCGCCTGTGACAGGCTACATGTTTGGGAAAGGGATCTACTTTGCCGACATG
GTGTCCAAAAGTGCAAACTACTGCCACACATCTCAGGGAGACCCGATTGGCTTAATACTG
CTGGGAGAGGTTGCCCTTGGAAACATGTATGAACTCAAGCATGCTTCACATATCAGCAAG
TTACCCAAGGGCAAGCACAGTGTCAAAGGTTTGGGAAAAACCACCCCTGACCCTTCGGCC
AGCATCACCCTGGAGGGTGTAGAGGTTCCACTGGGAACAGGGATCCCATCTGGTGTCAAC
GACACCTGCCTGCTGTATAATGAGTACATTGTCTACGACATTGCTCAGGTGAATCTCAAA
TACCTGCTGAAACTCAAGTTCAATTTTAAGACATCCCTGTGG
>common_starling_PARP1
ATGGCGGAGGAGGCGGAGAAGCTGTACCGGGCCGAGTATGCCAAGAGCGGCCGCGCCTCC
TGCAAGAAATGCGGCGAGAGCATCGCCAAGGACTCGCTGCGCCTGGCGCTCATGGTGCAG
TCACCCATGTTTGATGGCAAAGTCCCTCACTGGCACCACTACAGCTGCTTCTGGAAGCGG
GCTCGAATTTTGTCCCACACAGACATTGATGGCTTCCCGGAGCTTCGGTGGGAAGATCAG
GAGAAGATCAAGAAAGCCATTGAAACTGGAGGCGCTGGAGGAGGAAAAGGAGGGGAGCAG
GAAGGAGGTGGTAAGGCTGAGAAGAGCCTACATGACTTTGCTGCAGAATATGCCAAGTCT
AACAGAAGTACTTGCAAAGGCTGTGAGCAGAAAATAGAAAAGGGCCAGATCCGGATTTCC
AAGAAAATGGTGCATCCTGAAAAGCCACAGCTGGGAATGATTGATAACTGGTACCACCCA
GACTGCTTCGTGAGCCGCCGAGCAGAGCTCGGCTTCCTCCCGGCCTACGGGGCCACTCAG
CTCCTGGGCTTCAGCATCTTGAAAGCTGAAGATAAAGAAACTCTGAAGAAGCAGCTCCCA
GCTACCAAAAGCGAAGGAAAGAGAAAAGGAGAAGAGGTAGATGGAAATGCAACAGCGAAA
AAGAAGCCAAAAAAAGAAAAGGAGAAAGAATCAAAGCAGCAAAAACAGCTGAAGGAGCAG
ACAGAGCTGATCTGGGGCATCAAGGATGAGCTGAGGAAGGTCTGCTCCACCAATGACCTG
AAAGAGCTGCTGATTGCCAACAAGCAGGAGGTCCCCTCAGGGGAGAATGCCATCTTGGAC
CGAGTAGCAGATGGGATGGCATTTGGAGCTCTGCTGCCCTGCGAGGAGTGCAAGGGGCAG
TTTGTGTTCAAGAGTGACGCTTACTACTGTTCAGGGGATATCACTGCCTGGACCAAGTGC
GTGGCTAAAACACAGACTCCCAACAGGAAAGATTGGGTAATCCCAAAGGAGTTCCGGGAA
ATTCCTTACCTGAAGAAATTTAAATGTAAGAAGCAGGACAGAGTGTTCCCTCCAGAGGCT
GCAAGTGTGAACTCGGCACCTCCTCCCTCTGCATCTGCTCCTTTGTCAGAGACTGTGTCT

ACACCCAGAGACAAACCACTGACCAACATGAAGATCCTGGTTGTCGGGAAGTTGTCAAAG
AACAAAGAGGAGGTGAAAAGCATTGTGGAGGACCTGGGAGGAAAGATGACCACCACAGCT
AACAAGGCCACCCTGTGCATCAGCACCCAGAAGGATGTGGAGAAAATGAGCAAGAAGATG
GAAGAAGTGAAGGAGGCCAAAGTCCGTGTGGTCTCAGAGGCATTTCTTCAGGATGTGAAA
TCTTCCAGCAAGGACTTCCAGGAGCTTGTATCTCTCCATGCCCTTTCACCTTGGGGTGCA
GAGGTGAAAATGGAGCATGAGGAAATGGCTGTGGATGGGAAGAGCAGCAAGCCTCCAAGT
ACAAAAAGCACTGGGAAGGTCAAAGAAGAACAAGGACCTAGCAAGTCTGAAAAGAAAATG
AAGTTAACAGTGAAGGGTGGAGCAGCAGTAGACCCTGATTCTGGTTTGGAGGATTCTGCT
CATGTCTTTGAAAAAGGTGGGAAAATTTTCAGTGCAACCCTGGGACTAGTAGATATTGTG
AAAGGAACAAATTCCTATTATAAACTGCAGCTGCTAGAGGATGACAGAGAGAACAGGTAC
TGGGTGTTCCGATCCTGGGGCCGTGTGGGCACGGTAATCGGCAGTAACAAGCTGGAGCAG
ATGCCATCAAAAGAAGATGCCATTGAACACTTCCTGAATTTGTATGAAGAGAAAACTGGC
AATTCTTGGCATTCAAAGAACTTCACTAAATATCCCAAAAAATTTTACCCACTGGAAATA
GATTACGGACAGGATGAAGAAGCTGTCAGGAAACTGACAGTAGGTGCTGGGACAAAATCA
AAACTCGCTAAGCCAATCCAAGATCTTATTAAGATGATCTTTGATGTGGAGAGCATGAAG
AAAGCAATGGTGGAATTTGAGATTGACTTGCAGAAGATGCCATTGGGAAAACTGAGCAAG
CGACAGATCCAGAGTGCATACTCCATCCTGAATGAGGTTCAGCAGGCAGTTTCTGACAGT
GGTTCAGAATCTCAGATTTTGGACCTCTCCAACCGCTTTTACACACTGATTCCTCATGAC
TTTGGGATGAAGAAGCCACCTCTCCTAAATAACTTGGAATACATTCAGGCTAAAGTGCAG
ATGTTGGACAACTTGCTTGATATCGAGGTTGCTTACAGCCTTCTCAGAGGTGGAAATGAA
GATGGAGATAAAGACCCAATTGACATCAACTATGAAAAACTCAAGACGGATATTAAGGTT
GTTGACAAAGATTCAGAAGAAGCCAAGATTATCAAACAATATGTGAAGAACACTCACGCT
GCTACGCACAACGCATATGACCTCAAAGTCGTGGATATCTTTAGGATTGAGCGTGAGGGG
GAGAGCCAGCGTTTCAAGCCCTTCAAGCAGCTCCACAACCGCCAGCTGCTGTGGCACGGC
TCCCGCACCACCAACTTCGCCGGGATCCTCTCGCAGGGTCTGCGGATAGCTCCGCCTGAA
GCTCCTGTGACCGGCTACATGTTCGGGAAGGGCATCTATTTTGCAGACATGGTATCCAAG
AGTGCAAACTACTGTCACACATCTCAAGCTGATCCCATAGGTTTAGTACTACTGGGAGAA
GTTGCCCTTGGAAATATGTATGAACTAAAGAATGCTTCCCACATCACAAAATTGCCCAAG
GGAAAACACAGTGTCAAAGGCTTGGGCAAAACGGCGCCTGATCCCACAGCCACTACCACC
CTTGGTGATGTAGAGGTTCCCTTAGGGAATGGGATCTCCACAGGAATTAATGATACCTGT
CTTCTGTATAATGAATATATTGTGTATGACGTTGCTCAGGTAAATTTGAAGTACCTGTTG
AAACTGAAATTCAACTACAAGACATCACTCTGG
>central_bearded_dragon_PARP1
ATGGCCGAGCCTGCGGAGAAGTTGTACCGGGCCGAGTACGCCAAGAGCGGCCGGGCTTCG
TGCAAGAAATGCGGGGACAGCATCGCCAAGGACTCGCTCCGCCTGGCCATCATGGTGCAG
TCTCCCATGTTTGATGGTAAAGTACCCCACTGGCACCACTTTTCTTGTTTCTGGAAGCGA
GCTCGACTGGTGTCCCACTCTGATATAGATGGCTTCTCGGAACTGCGTTGGGAAGATCAA
GAGAAAATTAAAAAATCCATAGAAAGTGGTGGTGTTAGCACAGGCAAAGGTGGTGACCAA
GAAGGAGGTGGCAAAAATGAGAAGAGTTTAAATGATTTTGCAGCAGAATATGCAAAGTCG
AACAGAAGTACTTGCAAAGGCTGTGAACAGAAGATAGAAAAGGGTCAAATTAGAATTTCA
AAGAAAATGGTGCATCCTGAAAAGCCCCAGCTGGGGATGATAGACAACTGGTATCACCCA
GCATGCTTCATCAGCCGCAGAGCAGCTCTGGGGTTTCTTGCTACCTTCAGTGCTTCTCAG
CTCTTAGGCTTTGGGCTCCTGAGCGCAGAAGATAAGGAGACTCTGAAGAAGCAGCTGCCT
GCTCTTAAAAATGAGGGAAAGAGAAAAGGGGATGAGATGGATGCCAATGTGATTTCAAAA

AAGAAACCGAAAAAAGAGAAAGAAAAACAGTCCAAACAGGAGAAGCTGCTCAAGGAACAG
ACAGAATTGATTTGGCACATCAAAGATGAGTTAAAGAAAGCCTGTTCCACCAATGATCTG
AAAGAGCTCCTCATAGCCAACAAGCAGGAAGTTCCTTCTGGGGAATCTGCTATCGTGGAC
CGTGTTGCAGATGGGATGGCCTTTGGGGCCCTTCTGCCTTGTGAGGAGTGCAAAGGGCAG
TTTGTATTCAAGGGTGATGCTTACTACTGCACTGGGGACATCACGGCTTGGACCAAGTGT
GTTGCCAAGACACAAACTCCCAACAGAAAAGAGTGGGTAATCCCTAAGGCATTCCGAGAA
ATTACATATTTGAAGAAATTCAAGTTTAAGAGGCAGGATAGAGCATTCCCTCCAGAAGTG
GCTTCGACAAACTCAGCACTTCCTCCAAAAGTATCTGCTCCTATTACAGAGAATTCAGCT
GCTCCACCAGATAAACCATTAAATAATATGAAAATTCTGATCCTTGGGAAATTGTCAAAA
ACCAAAGATGAAATCAAGTCTGCCATTGAAGAACTTGGGGGGAAGATGACAACTACTGTG
AATAAAGCTGACCTGTGCATCAGCTCACAAAAGGAAGTTGAGAAAATGAACAAGAAAATG
GAAGAAGTGAAGGAGGCCCAGGTGCGGGTAATCTCGGACACCTTTCTGCAGGATGTGAAG
TCCTCCAGCAAGGGTTTCCAGGAACTTCTGTCCCTTCACGCTCTCTCTCCCTGGGGTGCA
GAAGTCAAGCAGGAGCACAAAGAGGTGTTTGTGGAAGAAAAACCCAGTGGGCATTCAAAC
ATGAAGAGCACTGGGAAGAATAAGGATGAACAAGGCACAAGCAAGTCTGAAAAGAAAATG
AAGCTGACAGTCAAAGGAGGTGCTGCTGTAGACCCCGATTCAGGCTTGGAAGATACTGCT
CATGTCTTTGAAAAAGGTGGCAAAATCTTCAGTGCCACTCTTGGCCTCGTAGATATTGTC
AGAGGAACAAATTCATATTACAAGCTTCAGCTGCTAGAAGATGACAGAGAACTCAGATAC
TGGGTTTTCCGCTCTTGGGGTCGTGTTGGAACAATTATTGGAAGCAACAAATTAGAGCAG
ATGTCCTCTAAAGAGGAAGCTACTGAACACTTCCTGAATTTGTACGAAGAGAAGACAGGC
AATTCCTGGCATTCAAAAAATTTCACTAAATATCCCAAAAAATTCTACCCTCTGGAAATA
GACTATGGACAGGATGAAGAAGCAGTGAAGAAACTGAAAGTGAGTGCAGGCACAAAATCA
AAGCTTCCAAAGCCAGTTCAGGACCTTATTAAGATGATCTTTGATGTGGAAAGCATGAAG
AAAGCGATGGTGGAATTTGAGATTGACCTCCAAAAGATGCCTTTGGGAAAGCTAAGCAAG
AGGCAGATCCAGAGTGCATATTCCATTCTTAATGAGGTGCAGCAGGCAGTTTCCAATGGT
GGCACAGAGTCACAGATACTGGATCTCTCAAATCGCTTTTACACTCTCATCCCTCATGAT
TTTGGGATGAAAAAGCCTCCTCTTTTAAACAACTTGGACTATATTAAGTCCAAAGTGGAG
ATGCTCGACAATCTGCTTGATATTGAAGTTGCCTACAGCCTGCTCAGAAGTGGTGGTCAA
GATGGGGATAAAGATCCAATAGATGTGAACTATGAAAAGCTCAAGACAGACATAAAGGTA
TTAGACAAAGACTCAGAAGAAGCCAGGATCATAAAGCAGTATGTGAAGAATACCCATGCA
AGCACCCACAATGCTTATGATTTGAAAGTCACGGAGATTTTCAAGATTGGGCGTGAAGGA
GAAAGCCAGCGTTACAAGCCATTCCAAGAATTACACAATCGCCAGTTGCTCTGGCATGGC
TCTCGTACTACCAATTTTGCTGGTATTTTGTCCCAGGGTCTCCGAATTGCTCCCCCTGAA
GCTCCCGTGACTGGCTATATGTTTGGTAAGGGTATCTATTTTGCGGATATGGTATCCAAG
AGTGCCAACTACTGTCATACCTCTCAGACTGACCCAGTCGGCTTAATCTTGCTTGGAGAA
GTTGCCCTTGGAAATATGTATGAACTTAAAAATGCTTCCCACATAACCAAGCTCCCGAAA
GGAAAACACAGTGTCAAAGGTTTGGGCAAAACAGCACCAGATCCTTCAGCCACTATATCA
CTTGATGGTGTGGATGTTCCTTTAGGCCCTGGAATTCCCTCTGGAGTTAGTAACACTTGT
CTTCTGTATAATGAATATATTGTTTACGATGTTGCTCAAGTAAATCTGAAGTACCTGCTA
AAACTGGATTTCAATTATAAGACATCACTCTGG
>spotted_gar_PARP1
ATGGCGGATTCACAAGAGGAGAAGCTGTACAAAGCCGAATACGCTAAAAGTGGTCGAGCT
TCTTGCAAGAAATGCAAAGACAATATCGCAAAAGATTCTCTCCGGATGGCTATAATGGTG
CAGTCTCCCATGTTTGATGGCAAAGTCCCCCATTGGCATCACTTCTCCTGCTTCTGGCAA

AGAGTATCAGTCCAGTCCCCAGGGGATATTTCGGGGTTCACAGACCTCAGATGGGAAGAC
CAGGAGAAGGTGAAGAAAGCCATTGAGACAGGAGGTGCAAGTTCTGGAGGAAAGGGTGAC
GCAAAGGGTGGTGCCAAAGGAGAGAAGACATTGAATGATTTTGCAGTGGAATATGCAAAG
TCCAACAGGAGCACCTGCAAGGGATGCAATGAGAAAATAGAGAAGGATCAAATCAGGGTG
TCTAAGAAGACAATTGACCCAGAGAAGCCTCAGTTGGGCTTAATTGACCGCTGGTACCAC
ACAGGCTGCTTTGTAAGCAGAAGGGAGGAACTGGATTTCAAACCTGCCTACTGCGCCTCG
CAGCTTAAAGGATTCGCCGCTTTAAGAGCAGAGGATAAAGAAGAGCTGAAGAAAAGGCTG
CCAGTTGTCAAGACTGAAGGAAAAAGGAAGGGAGATGAAGTAGACGGAGATGCCACGGCC
AAAAAGAAGCAGAAGAAAGAAGAAAAGGAAGAGAAGCAGCGTGAGAAGCAGCTTAAGGAA
CAGAACCAGCTCATCTGGAACATCAAAGATGAGTTGAAGAAGTCCTGCTCCACCAATGAC
ATGAAGGAGCTTCTCATTGCAAATGGTCAGGAAGTGCCTTCAGGAGAATCCAACATCCTG
GACAGCCTCTCTGACTGCATGGCGTTTGGTGCCCTGAAACCCTGTGAAGAATGCCAAGGC
CAGCTGGTCTTCAAGAGTGATGCTTATTACTGTACCGGAAACATCTCGGCCTGGACCAAG
TGTGTCTCCAAAACGCAGACGCCCAACCGGAAGGACTGGGTCACTCCTAAGGAATTCCAT
GAGATCCCCTACTTGAAGAAGTTCAAGTTCAAACGCCAGGACAGAGTGTTCCCCCGAATA
GAGGCCACATCTGCGCCCAGTTCCACCAGTACCAGTGCAGCTGGTGTGACAGAGTCTGCT
CCAGTTCCAGCAGATAAACCCCTGACAGGCATGAAGATCCTGTGCATCGGGAAGCTGGTG
AAGAACAAGGACGAGCTCAAAGTTACTGTGGAGGAACTGGGAGGGAAGATCACTGGGACG
GCCAACAAAGCCAATCTGTGTATCAGCACAAAGAAGGAGGTAGAGAAGCTAAGCAAGAAG
ATGGAGGAAGTGAAGGAGGCAGGTGTCCGGGTGGTGGCAGAGAACTTCCTGTCCGATATC
AAGTCTTCGGACAAGGCCTTCCAGGAGCTGGTGTCCCTTCACGGCCTTTCGCCGTGGGGC
GCGGAGATCAAGCAGGAGAATCCTGCTCCGGCTGCCAAGTCCAGCGGTGCTCCTGCCAGC
AAGAGCTCGGGGAAAGTGAAAGAAGAAGAAGGTTCCAGTAAATCCAAGAAGATGAAATTA
ACAGTAAAAGGAGGAGCTGCAGTGGATCCAGACTCAGGTCTGGAGGACCGTGCGCATGTT
CTTGATCAAAATGGTAAAATCTTCAGTGCCACACTTGGTCTGGTGGATATTGTCAGAGGA
ACAAATTCGTACTACAAGCTGCAGCTGCTGGAAGACGATGTCAAAAAGCGGTACTGGGTG
TTCAGGTCTTGGGGCCGTGTGGGCACAACTATTGGTGGCAATAAATTGGATAAATTTTCT
GACAAGAACTCCGCTATTGAAAATTTCCTAAGTCTTTATGAAGAAAAAACTGGAAATTCC
TGGCACTCCACCAACTTCACAAAATACCCAAACAAATTTTACCCTCTGGAGATCGACTAT
GGACAGGATGAAGAAGCAGTGAAGAAGCTGACAGCCAGTGCAGGCACTAAGTCCAAGCTG
GAGAAACCTGTTCAGGAGCTCATTAGGATGATCTTTGATGTGGAGAGCATGAAGAAGGCC
ATGGTGGAGTTTGAGATTGACCTGCAGAAGATGCCCCTGGGCAAGCTGAGCAAGAGGCAG
ATTCAGAGCGCGTACTCCATCCTCAGTGAGGTGCAGCAGGCTGTGTCTGACAATGCCTCT
GATTCACATATATTGGATCTGTCCAATCGGTTTTATACACTTATACCGCATGACTTTGGG
ATGAAGAAACCACCCCTCTTGAATAATCTGGATTATGTTCAGGCCAAAGTCCAGATGTTG
GATAATCTTCTGGATATTGAGGTGGCATACAGCCTTCTCTGTGGTGGAACAGAAGATGAT
AAAAAAGATCCCATTGACATCAACTATGAGAAGCTCAAAACTGAGATTCAGGTTGTTGAT
CTGACGTCAAAGGAAGCTGAGGTCATTCTGGAGTACGTCAAGAACACTCATGCTGCTACA
CATAATACCTACACGCTTGAGGTGGAGGAGGTTTTCAAGATAGCCCGGGAGGGCGAGTAC
CAGCGCTACAAGCCCTTCAAAGACTTGCCCAACCGTCAGCTGCTGTGGCATGGCTCTCGT
GCCACAAACTACGCTGGTATCCTGTCTCAGGGCCTGCGCATCGCGCCTCCTGAGGCCCCT
GTGACAGGTTACATGTTTGGTAAAGGTATCTACTTTGCTGACATGGTGTCGAAGAGTGCT
AATTACTGCCACACCTCAACAACTGATCCAGTGGGTCTGATTCTGCTGGCAGAGGTTGCT
TTAGGAAATATGCATGAATTGAAGAGGGCTTCGCATATTACAAAATTACCTAAGGGTAAA

CATAGTGTTAAAGGTGTGGGTAAAACTGCTCCAGATCCAAGTAGCACTGTGACTTTGGAT
GGCATGCAAGTTCCACTGGGAAAAGGCATCAACACTAATATTCAGGACACCAGCCTGCTG
TACAATGAATACATTGTGTATGATGTGGCACAGGTGAATCTGAAGTACCTGTTAAAGATC
AAGTTTAACTACCAGAGCTCTCTGTGG
>guppy_PARP1
ATGGCGGACAACCAGGAGGAGAAGCTGTACAAGGCCGAGTACGCTAAAAGCGGCCGCGCC
TCTTGCAAGAAATGCAAAGATAGCATCGCTAAAGACTCGCTGAGGATGGCCATCATGGTG
CAGTCGCCCATGTTTGACGGGAAGGTCCCCCACTGGCACCACTTCTCCTGCTTCTGGCTG
CGAGCGGCGCCTCAGTCCACGGCTGATATCGCTGGGTTTTCCGATCTGCGCTGGGATGAC
CAGGAGAAGGTGAAAAAGGCCATCGAGAGCGGCGGAGCAGTCGGAATAGGAAAGGGTAGC
CAGAAGAGTGCAGCTAAAGGAGAAAAGACTCTGAACGATTTTGCGGTGGAATATGCCAAG
TCGAACCGCAGCACGTGCAAAGGCTGCGAGCAGAAAATAGAAAAGGACCAGATTCGTGTG
TCCAAGAAGACGGTGGATCCGGAGAAGCCTCAGCTGGGCCTGATCGACCGCTGGTACCAC
ACGGCGTGCTTCGTGTCCCGCAGGGAGGAGCTGGTGTTTAGGCCTGAATACGGCGCCGCC
CAGCTGAAGGGATTCAACGCGCTGCGTGCGGAAGACCAGGAGGAGCTGAAGAAGAGGCTG
CCTGCTGTCAAATCTGAAGGGAAGCGTAAATCCGACGAGGTGGACGGAGCATCAAAGAAG
CAAAAGAAAGAGGAAGAGGAGGAGAAGAAGAAGCTCGAGGAGCAATTGAAGAATCAAAGC
CAGCTGATTTGGGGTATTAAAGACAAGCTGAAGAAACATTGTTCAGTCAACGACATGAAG
GAGCTGCTGATTGCAAACGGCCAGGATGTTCCGTCCGGAGAGTCCAACGTGCTGGACAGC
CTGGCTGACGGCATGGCCTTCGGTGCTCTGGACTCCTGTAACGAGTGCAAGGGCCAGCTG
GTGTTTAAAGGAGACTTGTATTACTGTTCTGGAGACATTTCAGCCTGGACAAAGTGTGTG
TTCAAAACCAAAATTCCTTCACGCAAAGACTTTGTCGTCCCTAAGGAATTCCACGAAGTT
TCTTTTCTAAAGAAATTTAAATTCAAGAGGCAGGACAGGGTTTACCCCAAGGAGGCTCCC
GCTCAAACTCTGAAACCAGCAAAAGCCGAAACCCTGGCGAGCGCTTCCAGTGCTCCCAGT
GAGCGACTGCTGGAAGCAGCACCTTCAGACAAACCTCTAACTGGTATGAAGCTGCTGGCT
GTCGGCAAGTTATCTAAAAACAAAGATGATCTGAAGGCAGCCGTGGAGGAACTGGGTGGA
AAAATTACCGGCACTGCCAATAAGGCCTCTCTCTGTCTCAGCTCCAAGAAGGAGGTGGAG
AAGATGGGTAAGAAGATGGAGGAAGTGAGAGACGCAGGCGTGCGAGTCGTCTCGGAGGAT
TTCCTCTCGGACATCAAGTCATCGGGTAAAGCTCTGCAGGAGCTGGTGTCTCTTCACGCC
ATCTCACCCTGGGGCGCAGAAGTTAAGGCTGAACCTCAGGCTCCAGCTGCAACCTCCAAA
TCTGGAGCTCTGTCTTCCAAGAGCACCGGCAGGGTAAAGGAGGAAGAAGGTGGTAGTAAG
GCCAAGAAGATGAAGCTGACAGTCAAAGGCGGAGCTGCTGTGGATCCAGACTCGGGTCTT
GAAAACAGTGCCCATGTCCTGGAGCAGAATGGGAAGATGTACAGTGCTACTTTGGGTCTT
GTGGACATCGTCAGAGGAACAAACTCTTATTATAAGCTGCAGCTGCTGGAGGATGACGTA
CAAAAAAGATACTGGGTTTTCAGGTCTTGGGGCAGAGTGGGCACCACCATTGGAGGCAAC
AAGCTGGACAAATTCCATGACAAGAACTCTGCGCTGGATAATTTCCTTGGTGTTTATAAG
GAAAAGACTGGAAATGACTGGGGTACCTCCAACTTCACTAAATATCCCAACAAGTTCTAC
CCTCTGGAGATTGACTATGGACAGGACGAGGAAGCGGTGAAACGGCTGACGGCCTCTGCT
GGCACCAAGTCTAAGCTGGACAAACCAGTTCAGGATCTGATTAAGATGATCTTTGACGTA
GAGAGCATGAAGAAGGCCATGGTTGAGTTTGAGATTGATCTGCAGAAGATGCCGCTTGGC
AAACTGAGTAAGAGACAAATCCAGAGTGCCTACGCTCTTCTGACTGAAGTACAACAGGCT
GTGTCGGACAGCGTACCTGAGGCACAGATACTGGATCTCTCCAATCGCTTCTACACCCTC
ATTCCTCATGACTTCGGCATGAAGAAACCTCCTCTCCTCAACAACCTGGATTACATTCAG
GCTAAAGTTGAGATGCTGGACAACCTGCTGGACATCGAGGTCGCCTACAGCTTGCTGAGA

GGAGGAGCTCAGGACAACGAGCACGATCCTATTGACATCAACTACGAGAAGCTCAAAACT
AAGATCGAGGTCGTTGACAAGACCACGCAGGAGGCTGAGATCATCATGCAATACGTTAAA
AACACCCACGCGGCTACACACAACACCTACACCCTGGAAGTGCAAGACATCTTCAAAGTC
GCACGAGAAGGAGAGCAACAACGCTACCGTCCGTTTGAGGAGCTACACAATCAACAGCTA
CTGTGGCACGGCTCTCGCGCCACAAACTACGCTGGTATTCTGTCTCAGGGTCTTCGCATT
GCACCTCCAGAGGCCCCTGTGACTGGCTACATGTTCGGCAAAGGTGTGTACTTTGCCGAC
ATGGTGTCCAAGAGTGCAAACTACTGCCACACCTCTCAGTCGGACCCTGTGGGACTCATT
CTGCTGGCAGAGGTCGCTCTAGGCAACATGCATGAACTGAAGAAAGCCTCTCACATTTCA
AAATTACCAAAGGGAAAGCACAGTGTTAAAGGTTTGGGTAGAACCGCTCCTGATCCAAAT
GCATCTGCCACTTTGAACGGGGTGCAAGTGCCTCTGGGAAAAGGAGTCAATACTAACATT
GATGACACAAGTCTACTGTACAACGAGTACATTGTATATGATGTGGCTCAGATAAACTTG
AAGTATCTCCTGAAGATCAGGTTTAACTACCAGACATCTCTGTGG
>Anole_lizard_PARP1
ATGGCGGAGTCTGCGGAGAAGCCGTACCGCGCCGAATACGCCAAGAGCGGCCGGGCTTCG
TGCAAGAAATGCGGCGACAACATCGCCAAGGACTCGCTCCGCCTGGCCATCATGGTGCAG
TCTCCCATGTTTGATGGCAAAGTACCCCATTGGCATCACTTTTCTTGCTTCTGGAAACGC
GCTCGACTGATATCCCATGCTGATGTGGATGGCTTCTCAGAACTGCGTTGGGAGGATCAG
GAGAAGATTAAAAAATCTATTGAAGCTGGTGGTGCTGGAACAGGGAAGGCGGGGAAAACT
GAAAAGAATTTAAATGATTTTGCAGTAGGATATGCAAAGTCCAACAGAAGTACCTGCAAA
GGCTGTGAACAGAAGATAGAAAAGGGTCATATTAGAATTTCAAAGAAAATGGTGAACCCT
GAGAAGCCCCAGTTGGGGATGATAGACAACTGGTACCATACTTCCTGTTTCATCAACTGC
AGAGGAGATCTGGGTTTCCTTACTACCTTTAGTGCCTCTCAGCTCTTAGGATTTGGGATC
CTGAATGAAGAAGATAAAGAGGCTCTGAAAAAGGAGCTGCCTGCTCTTAAGAATGAAGGA
AAGAGAAAAGGAGATGAAGTAGATGGTGCTGTAGTATCTAAAAAGAAACCAAAAAAGGAG
AAAGAAAAACAGTCCAAACAGGAGAAACTGCTAAAGGAGCAGACAGAGCTGATTTGGAAC
ATTAAAGATGAACTAAAGAAAGCATGTTCCACCAATGACCTCAAAGAGCTCCTGATTGCC
AACAAACAGGAAGTTCCTTCTGGGGAATCTGCTATCTTGGACCAAGTTGCAGATGGGATG
GCTTTTGGGGCTTTGCTGCCCTGTGAGGAGTGCAAAGGCCAGTTTGTGTTCAAATGTGAT
GCTTACTACTGCACTGGGGATATTACGGCTTGGACAAAATGTGTTGCCAAGACACAGTCT
CCCAACAGGAAAGAATGGATCATCCCAAAGGCCTTTCGGGAAATCACATATCTGAAGAAA
TTCAAGTTTAAAAAGCAGGAAAGAGTGTTCCCTCCAGAGGCTGCTTGTACAAACCCTGTA
CCACCTCCAGCAGTTTCCTCCACTCCCGTTATAGAAAACTCCACCACCCCAGCAGATAAA
CCATTAAATAGCATGAAGATTTTAGTCCTTGGGAAATTGTCAAAAAACAAGGATGAAATC
AAATCTGCCATTGAAGAACTTGGAGGAAAAGTGACAGCTACTGTGAATAGAGCAAACTTG
TGCATAAGCTCACAAAAGGAAATTGACAAGATGAATAAGAAGATGGAGGAGGTGAAACAG
GGCCAGGTGCGAGTTGTCTCAGAAGAGTTCCTCCAGGACGCAAAGGCCTCCAGCAAGGGC
TTACAGGAGCTTTTGTCACTCCATGCGCTCTCTCCGTGGGGCACAGAGGTCAAGCAGGAA
CACAAAGAGGTGTTTGTGGCAGAAAAATCCAGTGGACACTCGAACATGAAAAGCACTGGA
AAAAACAAGGAAGAACAAGGAACCAGCAAGTCTGAAAAGAAAATGAAGCTGACAGTGAAA
GGAGGTGCTGCTGTAGATCCTGATTCAGGCTTGGAAGATTCTGCTCACGTCTTTGAAAAA
GGTGCTAAAATCTTCAGTGCTACTCTTGGTCTGGTTGATATTGTCAGGGGAACAAATTCA
TATTATAAGCTTCAGTTACTGGAAGATGACAGAGAACTCAGATACTGGGTTTTCCGTTCC
TGGGGTCGTGTTGGCACAAGCATTGGGAGCAATAAACTAGAACAGATGCCATCTAAAGAA
GAAGCCATTGAACATTTCCTGAATCTGTATGAGGAAAAGACAGGGAACTCCTGGCATTCC

AAAAATTTCACTAAGTATCCCAAGAAATTCTACCCTCTGGAAATAGACTATGGACAGGAT
GAAGAAGCGGTGAAGAAACTGACAGTGAGTGCAGGAACCAAATCAAAGCTTCCCAAGCCA
GTCCAAGACCTTATTAAGATGATCTTTGATGTGGAAAGCATGAAGAAGGCCATGGTGGAA
TTTGAGATTGACCTCCAGAAAATGCCTTTGGGGAAGCTAAGTAAGAGGCAGATTCAGAGT
GCGTACTCCATCCTTAATGAGGTTCAGCAGGCTGTTTCTGATGGTGGCACAGAATCACAG
ATACTGGATCTCTCCAATCGCTTTTACACTCTAATCCCTCATGACTTTGGGATGAAAAAA
CCTCCCCTTTTAAACAGTCTGGACTACATTAAGTCCAAAGTGGAGATGCTGGACAATCTG
CTTGACATTGAGGTGGCCTACAGCCTACTCAGGAGCGGTGCTCAGGATGGGGATAAAGAT
CCAATAGATGTGAACTATGAAAAACTGAAGACAGATATTAAGGTAGTAGACAAAGACACA
GACGAAGCCAAGATCATAAAGCAGTATGTTAAGAATACCCATGCAAGTACCCACAATGCA
TATGACTTGGAAGTTGTGGAGATTTTCAAGATTGAACGTGAAGGAGAATATCAGCGTTAT
AAGCCATTCCGAGAATTGCACAATCGTCAGTTACTCTGGCATGGTTCTCGCGCTACCAAC
TTTGCTGGTATTTTGTCACAAGGTCTCCGAATAGCTCCCCCTGAAGCGCCAGTGACTGGC
TACATGTTTGGGAAAGGTATCTACTTTGCAGATATGGTGTCTAAGAGTGCCAACTACTGT
CATACTTCTCAGACTGATTCAGTAGGCTTAATCTTGCTGGGAGAAGTTGCCCTTGGAAAT
ATGTATGAACTGAAAAATGCTTCTCACATAACTAAGCTACCAAAAGGAAAACACAGCGTC
AAAGGTTTGGGCAAAACAGCACCAGATCCTTCAGCCACAGTGTCTCTTAATGGCGTGGAC
GTTCCTTTAGGCTCTGGCATCCCATCTGGAGTTAGCAATACCTGTCTTTTATATAACGAA
TATATCGTCTATGATATTGCTCAGGTAAATCTGAAGTACTTGCTGAAGCTGGATTTCAAG
TATAAAACTTCCCTCTGG
>Rock_pigeon_PARP1
ATGTTTGATGGCAAAGTCCCTCACTGGCACCACTACAGCTGCTTCTGGAAGCGAGCTCGA
ATCTTGTCCCATGCAGACATTGACGGCTTCCCTGAGCTCCGGTGGGAAGACCAGGAGAAG
ATCAAGAAAGCCATTGAAACTGGAGGCCCTGGAGGAGGAAAAGGAGGGGAACAGGAAGGA
GGTGGCAAGGCTGAAAAGAGCCTGAATGACTTTGCTGCAGAATATGCCAAGTCTAACAGA
AGTACTTGCAAAGGCTGCGAACAGAAAATAGAAAAGGGCCAGATCCGGATTTCCAAGAAG
ATGGTGCATCCTGAGAAGCCGCAGCTGGGAATGATAGATAACTGGTACCACCCGGACTGC
TTCGTGAGCCGCCGAGCGGAGCTGGGCTTCCTCCCGGTGTACGGGGCCACCCAGCTCCTG
GGCTTCAGCATCTTGAAAGCTGAAGATAAAGAAACTCTGAAGAAGCAGCTCCCGGCTACC
AAGAGTGAAGGAAAGAGAAAAGGAGAAGAGGTAGACGGAAATGTGACTGTGAAAAAGAAG
CCGAAAAAGGAAAAAGAGAAAGAATCAAAGCAGGAAAAGCAGCTGAAGGAGCAGACACAG
CTGATCTGGAGCATCAAGGATGAGCTGAGGAAGGTCTGTTCCACTAACGACCTGAAAGAG
CTGCTGATCGCCAACAAGCAGGAAGTTCCTTCAGGGGAGAATGCTATTTTGGACCGAGTA
GCAGATGGGATGGCATTTGGAGCCCTGCTCCCGTGCGAGGAGTGTAAGGGGCAGTTTGTG
TTCAAGAGCGACGCATATTACTGTTCGGGGGATATCACTGCCTGGACTAAGTGTGTTGCT
AAAACACAGACTCCCAACAGGAAAGACTGGGTGATCCCAAAGGAGTTTCGGGAAATCCCT
TATCTGAAGAAATTTAAGTGTAAGAAGCAGGACAGGGTGTTCCCTCCAGATGCTGGGACT
GGGAACTCTGTGCCTCCTCCCTCCGCATCTGCTCCTCCGACAGAGACGGTGTCCGCACCC
AGAGACAAACCGCTGACCAATATGAAGATCCTGGTTCTTGGAAAACTGTCAAAGAACAAG
GAGGAGGTGAAGAGCATTGTGGAGGACCTGGGGGGAAAGATGACGACGACAGCTAACAAG
GCCACTCTGTGCATCAGCACACAGAAGGATGTGGAGAAAATGAGCAAGAAGATGGAAGAA
GTGAAGGCGGCCAAAGTCCGTGTAGTCTCAGAGGAGTTTCTTCAGGATGTGAAATCCTCC
AGCAAGGACTTTCAGGAGCTTGTCTCTCTCCATGCACTTTCACCTTGGGGTGCAGAGGTG
AAAATGGAGCACGAGGAAATGGCCGTGGATGGGAAGTGCAGCAAGCCCCCGAGTATGAAG

AGTGCTGGGAAGGTCAAAGAAGAACAAGGACCTAGCAAGTCTGAAAAGAAAATGAAGCTA
ACAGTGAAGGGTGGAGCGGCAGTAGATCCTGATTCGGGTTTGGAGGATTCTGCTCATGTC
TTTGAAAAAGGTGGAAAGATTTTCAGTGCAACCCTGGGCCTAGTAGATATTGTGAAAGGA
ACAAATTCCTATTACAAACTGCAGCTGCTAGAGGATGACAGAGAGAACAGATACTGGGTG
TTCAGATCTTGGGGTCGTGTGGGCACTGTAATTGGGAGTAACAAGCTGGAGCAGATGCCA
TCAAAAGAAGATGCCATTGAACACTTCTTGAATTTGTATGAAGAGAAAACTGGAAATTCA
TGGCATTCAAAGAACTTCACTAAATATCCCAAAAAATTCTACCCGCTGGAAATAGATTAT
GGGCAGGATGAAGAAGCTGTCAAGAAACTGACAGTGGGTGCTGGGACTAAGTCAAAACTT
CCTAAGCCAATCCAAGACCTTATTAAGATGATCTTTGATGTGGAGAGCATGAAGAAAGCG
ATGGTGGAATTTGAGATTGACCTACAGAAGATGCCATTGGGAAAACTGAGCAAGCGACAG
ATCCAGAGTGCCTACTCCNNNNNNNNNNNNNNNNCATGAAAGAAATGCAGTTTCTGACAAT
GGTTCAGAATCCCAGATCTTGGACCTCTCCAACCGCTTCTATACTCTGATTCCTCATGAC
TTCGGGATGAAGAAACCACCTCTCCTAAATAACCTGGAATACATTCAGGCTAAAGTGCAG
ATGTTGGACAACTTGCTTGATATTGAGGTTGCTTACAGCCTTCTCAGAGGTGGAAATGAA
GATGGAGATAAAGACCCAATTGACATCAACTATGAAAAGCTTAAAACTGATATTAAGGTT
GTTGACAAAGATTCCGAAGAAGCCAAGATTATTAAGCAATATGTGAAAAATACTCACGCT
GCTACTCATAACGCATATGATCTCAAAGTCGTGGATATATTCAGGATTGAGCGTGAAGGA
GAGAGCCAGCGTTACAAGCCGTTCAAGCAGCTTCACAATCGCCAGCTGCTGTGGCACGGA
TCCCGCACCACCAACTTTGCCGGGATCCTCTCACAGGGTCTCCGGATAGCTCCCCCTGAA
GCTCCTGTGACTGGCTACATGTTTGGGAAAGGCATCTATTTTGCAGACATGGTATCCAAG
AGTGCCAACTACTGCCACACATCTCAAGCTGATCCCATAGGCTTAGTACTACTGGGAGAA
GTTGCCCTTGGAAATATGTATGAGCTAAAGAACGCTTCTCACATAACAAAATTGCCCAAG
GGAAAACACAGTGTGAAAGGCTTAGGCAAAACTGCACCTGATCCCACAGCCACTACCACT
CTTGGTGGGGTGGAGGTTCCCTTAGGGAATGGGATCTCGACGGGAATTAATGATACCTGT
CTTCTGTATAATGAATATATTGTGTATGACGTTGCTCAGGTAAATCTGAAGTACCTGCTG
AAACTGAAATTCAACTATAAGACATCACTCTGG
>Great_tit_PARP1
ATGGCGGAGCCGGCGGAGAAGCTGTACCGGGCCGAGTATGCCAAGAGCGGCCGCGCCTCC
TGCAAGAAGTGCGGCGAGAGCATCGCCAAGGACTCGCTGCGCCTGGCGCTCATGGTGCAG
TCACCCATGTTTGATGGCAAAGTCCCTCACTGGCACCACTACAGCTGCTTCTGGAAGCGG
GCTCGAATTGTGTCCCATACAGACATTGATGGCTTCCCTGAGCTTCGGTGGGAAGATCAG
GAGAAAATCAAGAAATCCATTGAAACTGGAGGCCCTGGAGGAGGAAAAGGAGGGGAACAG
GAAGGAGGTGGTAAGGCTGAGAAGAGTCTAAATGACTTTGCTGCTGAATATGCCAAGTCT
AACAGAAGTACTTGCAAAGGCTGTGAGCAGAAAATAGAAAAGGGCCAGATCCGAATTTCC
AAGAAGATGGTGCATCCTGAAAAGCCGCAGCTGGGAATGATAGATAACTGGTACCACCCG
GACTGCTTTGTGAGCCGCCGAGCAGAGCTGGGCTTTCTCCCAGCCTACGGGGCCACCCAG
CTCCTGGGTTTCAGCATCTTGAAAGCTGAAGATAAAGAAACTCTGAAGAAGCAGCTCCCA
GCTACCAAGAGTGAAGGAAAGAGAAAAGGAGAAGAGGTAGATGGAAACGTGACTGCAAAA
AAGAAGCAGAAAAAAGAAAAAGAGAAAGAGTCAAAGCAGCAAAAACAGCTGAAGGAGCAG
ACAGAGCTGATCTGGGGCATCAAAGATGAGCTGAGGAAGGTCTGCTCCACCAATGACCTG
AAAGAGCTGCTGATTGCCAACAAGCAGGAGGTCCCCTCAGGGGAGAATGCTATCTTGGAC
CGAGTAGCAGATGGGATGGCGTTTGGAGCTCTGCTGCCCTGCGAGGAGTGCAAGGGACAG
TTTGTGTTCAAGAGTGATGCTTATTACTGTTCAGGAGATATCACTGCCTGGACCAAGTGT
GTTGCTAAAACACAGACTCCCAACAGAAAAGACTGGGTAATCCCAAAGGAGTTCCGGGAA

ATTCCTTACCTGAAGAAATTTAAATGTAAGAAGCAGGACAGGGTGTTCCCTCCGGATGCT
GCAACTGTGAACTCGGCACCTCCTCCCTCTGCATCTGCTCCTTTGTCAGAGACCATGTCT
GCACCCAGAGACAAACCACTGACCAACATGAAGATCCTGGTTGTTGGGAAGCTGTCAAAG
AACAAGGAGGAGGTGAAAGGCATTGTGGAGGACCTGGGAGGAAAGATGACGACAACAGCT
AACAAAGCCACCCTGTGCATCAGCACCCAGAAGGATGTGGAGAAAATGAGCAAGAAGATG
GAAGAAGTGAAGGAGGCCAAAGTCCGTGTGGTCTCAGAGGCATTTCTTCAGGATGTGAAA
TCTTCCAGCAAGGACTTCCAGGATCTGGTGTCTCTCCATGCTCTTTCACCTTGGGGTGCA
GAGGTGAAAATGGAGCACGAGGAAATGGCTGTGGATGGGAAGATCAGCAAGCCCCCAAGT
ACAAAGAGCGCTGGGAAGGTCAAAGAAGAACAAGGACCTAGCAAATCTGAAAAGAAAATG
AAGTTAACAGTGAAGGGTGGAGCAGCAGTAGACCCTGATTCTGGTTTGGAGGATTCTGCT
CATGTTTTTGAAAAAGGTGGGAAAATTTTCAGTGCAACCCTGGGACTAGTAGATATTGTG
AAAGGAACAAATTCCTATTATAAACTGCAGCTGCTAGAGGATGACAGAGAGAACAGATAC
TGGGTGTTCCGATCCTGGGGCCGTGTAGGCACTGTAATTGGCAGTAACAAGCTGGAGCAG
ATGCCATCAAAAGAAGATGCCATTGAACACTTCCTGAATTTGTATGAAGAGAAAACTGGC
AATTCTTGGCATTCAAAGAACTTCACTAAATATCCAAAAAAATTCTACCCACTGGAAATA
GATTACGGACAGGATGAAGAAGCTGTCAGGAAACTGACAGTAGGTGCCGGGACAAAATCA
AAACTTGCTAAGCCAATCCAAGATCTTATTAAGATGATCTTTGATGTGGAGAGCATGAAG
AAAGCAATGGTGGAATTTGAGATTGACTTGCAGAAGATGCCATTGGGAAAACTGAGCAAG
CGACAGATCCAGAGTGCATATTCCATCCTGAATGAGGTTCAGCAGGCAGTTTCTGACAGT
GGTTCAGAATCCCAGATTTTGGACCTCTCCAACCGCTTCTATACACTGATTCCTCATGAC
TTTGGGATGAAGAAACCACCTCTCCTAAATAACTTGGAATACATTCAGGCTAAAGTGCAG
ATGCTGGACAACTTGCTTGATATTGAGGTTGCTTACAGCCTTCTCAGAGGTGGAAATGAA
GATGGAGATAAAGACCCAATTGACATCAACTATGAAAAACTTAAAACAGATATTAAGGTT
GTTGACAAAGATTCAGAAGAAGCTAAGATTATCAAACAATATGTGAAGAACACTCACGCT
GCTACGCACAACGCATATGACCTCAAAGTCGTGGATATCTTCAGGATTGAGCGTGAGGGG
GAGAGCCAGCGTTACAAGCCCTTCAAGCAGCTCCACAACCGCCAGCTGCTGTGGCACGGC
TCCCGCACCACCAACTTCGCCGGGATCCTCTCGCAGGGTCTGCGGATAGCTCCGCCTGAA
GCTCCTGTGACCGGCTACATGTTCGGAAAGGGCATCTATTTTGCAGACATGGTATCCAAG
AGTGCAAACTACTGTCACACATCTCAAGCTGATCCCATAGGTTTAGTACTACTGGGAGAA
GTTGCCCTTGGAAATATGTATGAACTAAAGAATGCTTCCCACATCACAAAATTGCCCAAG
GGAAAACACAGTGTCAAAGGCTTGGGCAAAACTGCGCCTGATCCCACAGCCACTACCACC
CTTGGTGGTGTAGAGGTTCCCTTAGGGAATGGGATCTCCACAGGAATTAATGATACCTGT
CTTCTGTATAATGAATATATTGTGTATGATGTTGCTCAGGTAAATCTGAAGTACCTGTTG
AAACTGAAATTCAACTATAAGACATCACTCTGG
>pig_PARP1
ATGGCGGAGTCATCGGACAAGCTCTATCGGGTCGAGTATGCTAAGAGTGGGCGCGCCTCC
TGCAAGAAATGCAGCGAGAGCATCCCCAAGGACTCGCTCCGGATGGCCATCATGGTGCAG
TCGCCCATGTTCGATGGGAAAGTCCCGCACTGGTACCACTTCTCCTGCTTCTGGAAGGTC
GGCCACGCCATCCGGCACCCTGACATCGAGGTGGACGGGTTCTCCGAGCTCCGGTGGGAC
GACCAGCAGAAAGTCAAGAAGACGGCGGAGGCTGGAGGAGTGACAGGCAAAGGCCAAGAT
GGGGTTGGCAGCAAGGCGGAGAAGACGCTGGGTGACTTCGCGGCCGAGTATGCCAAGTCC
AACAGAAGCACGTGCAAGGGCTGCATGGAGAAGATCGAGAAGGGCCACGTGCGCCTGTCC
AAGAAGATGCTGGACCCCGAGAAGCCCCAGCTGGGCATGATCGACCGCTGGTACCACCCA
AACTGCTTTGTTCAGAACCGGGAGGAGCTGGGCTTCCGGCCCGAGTACAGCGCCAGCCAG

CTTAAGGGCTTCAGCCTGCTCACCGCAGAGGACAAAGAAGCGCTGAAGAAGCAGCTCCCG
GGCATCAAGAGTGAAGGAAAGAGAAAAGGTGACGAGGTAGATGGAGTGGATGGAGTGGCC
AAGAAGAAATCTAAAAAAGAAAAGGACAAGGAGAGTAAGCTCGAAAAGGCCCTCAAGGCC
CAGAACGACCTGATCTGGAATATCAAGGACGAGCTAAAGAAAGTGTGTTCAACCAATGAC
CTGAAAGAGCTGCTCATCTTCAACAAGCAGCAAGTGCCCTCCGGGGAGTCGGCGATCTTG
GACCGAGTGGCCGACGGCATGGTGTTCGGCGCCCTTCTTCCCTGTGAGGAATGCTCAGGC
CAGCTGGTCTTCAAGAGCGATGCCTACTACTGTACTGGGGACGTCACTGCCTGGACCAAG
TGCATGGTCAAGACACAGACGCCTAACCGGAAGGAGTGGGTGACCCCAAAGGAATTCCGA
GAAATCACTTACCTCAAGAAATTGAAGATCAAAAAACAGGACCGTATATTCCCCCCCGAG
GCCAGCACCCCTGTGGCCACAGCACCCCCACCCCCCGCAGCCTCTGCACCCACCGCCGTG
AACTCCTCGGCCCCACCAGACAAGCCGTTATCCAACATGAAGATCCTGACTCTTGGGAAA
CTCTCCCGGAACAAGGATGAAGTGAAGGCCGCGATCGAGAAACTCGGGGGGAAGTTGACA
GGGACGGCCAGCAAGGCCTCCCTGTGCGTCAGCACCAAGAAGGAGGTGGACAAGATGAGT
AAGAAGATGGAGGAAGTGAAAGAAGCGAATGTCCGAGTGGTGTCCGAGGATTTCCTCCAG
GACGTCTCGGCCTCGACCAAGAGCCTTCAGGAGCTGCTCTGCACCCACATCCTGTCCCCC
TGGGGGGCCGAAATGAAGGCTGAGGTGAAGGCCGAACCTGTGGAAGCCGTGGCCCCCAGA
GGGAAGTCAGGGGGTGCACTCCCCAAGAAGAGCAAGGGCCCCATCAGGGAGGAAGGTGCC
AACAAATCTGAAAAAAGAATGAAATTAACTCTTAAAGGAGGAGCAGCCGTGGACCCCGAT
TCTGGTCTGGAACACTCGGCCCACGTCCTGGAGAAAGGCGGGAAGGTCTTCAGTGCCACA
CTCGGCCTGGTGGATATTGTCAAGGGAACCAACTCCTATTATAAGCTGCAGCTCCTGGAG
GATGACAAGGAGAGCAGGTACTGGATATTCAGGTCCTGGGGCCGTGTGGGCACCGTAATT
GGTAGTAACAAACTGGAGCAGATGCCATCCAAGGAGGATGCCATTGATCATTTTATGAAA
TTATATGAAGAGAAAACTGGGAATGCCTGGCACTCCAAAAACTTCACAAAGTATCCCAAG
AAGTTCTACCCTCTGGAGATTGACTATGGCCAGGATGAAGAGGCAGTGAAGAAGCTGACA
GTGAACCCTGGCACCAAATCCAAGCTCCCCAAATCGGTGCAGGAACTCATCAAGATGATC
TTTGATGTGGAAAGTATGAAGAAAGCCATGGTGGAGTACGAGATTGACCTTCAGAAGATG
CCGTTGGGGAAGCTGAGCAAAAGGCAGATCCAGGCCGCGTACGCCATCCTCAGTGAGGTC
CAGCAGGCCGTGTCCCAGGGCAGCAGCGACTCCCAGATCCTGGATCTCTCAAATCGATTC
TACACCTTGATCCCCCATGACTTTGGGATGAAGAAGCCTCCACTGCTGAACAATGCGGAC
AGCGTGCAGGCCAAGGTGGAAATGCTAGACAACCTATTGGACATCGAGGTGGCCTACAGT
CTGCTCAGGGGTGGTTCTGATGACAGCAGCAAGGACCCCATTGATGTCAACTATGAGAAG
CTCAAAACTGATATTAAGGTGGTGGACAGGGATTCTGAAGAAGCCGAGACCATCAGGAAG
TACGTTAAGAACACTCACGCAACCACACACAATGCATATGACTTGGAAGTTATCGATATC
TTTAAGATCGAGCGTGAAGGGGAGAGTCAGCGTTACAAGCCATTTAAACAGCTGCATAAC
CGGAGGTTGCTGTGGCACGGGTCCAGGACCACCAACTTCGCCGGGATCTTGTCCCAGGGT
CTCCGGATAGCCCCACCTGAAGCACCTGTGACGGGCTACATGTTTGGGAAGGGGATCTAT
TTCGCCGACATGGTCTCCAAGAGTGCCAACTACTGCCACACGTCCCAGGGAGACCCCATA
GGCTTGATCCTGTTGGGAGAAGTTGCCCTTGGAAACATGTATGAGCTGAAGCACGCTTCG
CACATCAGCAAGTTACCCAAGGGCAAGCACAGTGTCAAAGGCTTAGGCAAAACGACCCCT
GACCCCGCAGCCAGTATTACTATGGATGGTGTGGAGGTTCCTCTTGGGACCGGGATCCCA
TCTGGTGTGAACGATACCTGTCTGCTGTATAACGAGTACATTGTCTACGATATCGCTCAG
GTCAATCTCAAGTATCTACTGAAGCTGAAATTCAACTTTAAGACGTCCCTGTGG
>Taiwan_habu_PARP1
ATGGCCGAGCCCGCGGAGAAGCTTTACCGGGCCGAGTACGCCAAGAGCGGGCGGGCTTCC

TGCAAGAAATGCAAGGAAAACATCGCCAAGGACTCGCTCCGCCTCGCCATCATGGTGCAG
TCCCCTATGTTTGATGGCAAAGTACCTCACTGGCACCACTATTCTTGTTTCTGGAAACGA
ACTCAGCTGCTGTCCCATGCTGATCTGGATGGCTTCTCAGAACTGCGTTGGGAAGACCAG
GAAAAAATTAAAAAACACATAGAAAGTGGAGGAGCTGCAGCAGGCAAAGGTGGCCAAGAG
GGAGGTGGCAAACGTGAAAAAAGTTTAAATGATTTTGCAGCAGAATATGCAAAGTCCAAT
AGAAGTACTTGCAAAGATTGTGAACGAAAGATAGAAAAGGGTCAGATCAGAATCTCAAAG
AAAATGGTGCTCGCTGAAAAGCCACAGCTGGGGATGATAGACAATTGGTACCATCTTGAC
TGTTTCAGTAGCCGCAGAACAAATCTGGGTTTTCTTCCTACTTTTAATGCCTCTCAGATC
CAGGGCTTTGAGCTCCTAAAGGCAGAAGACAAAGAAATTCTAAAGAAGCAGCTACCATCT
GTCAAGAATGAAGGAAAGAGAAAGGGAGATGAGGTGGATGCAAATGTGATCTCAAAAAAG
AGACCCAAGAAAGAGAAAGAAAAATTATTGAAACAAGAAAAGCTGTTAAAGGATCAGACA
GAACTGATTTGGAATATCAAAGATGAGTTGAAGAGATCCTGTTCTACCAATGACTTAAAG
GAGCTCCTGATAGCCAACAAGCAAGAAGTTCCTTCTGGGGAAACTGCTATCTTGGACCGT
GTTGCAGATGGGATGGCTTTTGGAGCTCTGCTTCCCTGTGAAGAGTGCAAAGGGCAATTT
GTGTTCAAGAGCAATACGTATTACTGCACAGGGGATATCACAGCTTGGACCAAATGTGTT
TCCAAGACACAAACACCCAGCAGGAAAGAATGGACAATCCCAAAGGAATTCCGGGAGATC
CCTTACCTAAAGAAATTCAAATTTAAAAAGCAAAACAGAGCATTCTTGCCAGAGGCTGCT
TCTACAAATTCTGTACCTCCTTCAGCAGTTTCTGATCCTATTACAGAGAATTCGGCTGCA
CCGGCAGATAAACCATTAAACAACATGACGGTATTAGTCGTTGGAAAATTGTCAAAGACC
AAGGATGAAATTAAATCTGTTGTTGAAGAACTCGGGGGAAAGGTGACAGCGACTGTGAAT
AAAGCTGACTTGTGCATCAGCTCTCAAAAGGAAGTTGACAAAATGAACAAAAAAATGGAA
GAAGTAAAGAAAGCCCACTTGTGTGTGGTGTCGGAGAACTTCCTCCAGGATATAAAAAGC
TCCAGCAAGGGTTTTCAGGAGCTTCAGTCACTTCATGCGGTGTCTCCCTGGGGTGCAGAA
GTAAAGCAGGAACACAAAAAAGTGTCTGTGAGTGGAAAATCCAGCGGACACCCAAACGTG
AAAAATTCTGGGAAGAACAAGGAAGAGCAAGGAACAAGTAAATCTGAGAAGAAAATGAAG
TTAACAGTTAAAGGAGGGGCTGCTGTCGACCCTGATTCAGGTCTGGAAGATTCTGCGCAT
GTTTTTGAAAAAAGTGGTAAAATCTTCAGTGCCACTTTAGGTCTAGTTGATATTGTCCAA
GGAACAAATTCTTATTACAAACTTCAGCTGCTGGAGGATGACCGAGAGACCAGGTACTGG
GTTTTTCGCTCATGGGGTCGTGTTGGAACATCAATTGGAAGTAACAAACTAGAGAAGATG
CCTTCTAAAGAAGAAGCCATTGAACATTTCCTAGATCTATATGAAGAGAAAACAGGCAAT
TCCTGGCATTCCAAAAATTTCACCAAATATCCCAAAAAATTTTACCCTTTGGAGATAGAC
TATGGGCAGGATGAAGAAGCGGTCAAGAAACTGACAGTGAGTGCAGGAACCAAGTCAAAG
CTTCCTAAGGCTGTCCAAGATCTTATTAAGATGATTTTTGATGTAGAAAGTATGAAGAAA
GCCATGGTGGAGTTTGAGATTGATCTCCAGAAGATGCCTTTGGGGAAGTTAAGTGAGAGG
CAGATCCAGAGCGCGTACTCCATTCTGAATGAAGTGCAGCAGGCAGTTTCTGATGGTGGC
ACAGATTCCCAGATATTGGATCTCTCCAATCGCTTCTACACTCTAATTCCCCATGACTTT
GGGATGAAGAAGCCTCCACTTCTAAACAACCTTGACTACATTAAGACTAAAGTGGAAATG
CTGGATAATCTGCTTGACATAGAGGTCGCTTATAGCCTTCTAAGAAGTGGCGGTCAGGAT
GGGGATAAGGATCCAATAGATGTGAACTATGAGAAGCTCAAAACCATTATCCAGGTAGTA
GATAAAGATTCAGAAGAAGCCAAGATTATAAAACAATATGTTAAGAACACTCATGCAAGT
ACCCACAATGCATATGATTTGAAAGTTGCGGAGATTTTCAAGATTGAACGTGAAGGGGAA
TATCAACGTTATGAACCATTCCGAGACCTACATAATCGCCAATTGCTCTGGCATGGTTCT
CGCACTACCAATTTTGCTGGTATCTTGTCACAGGGTCTCCGAATAGCTCCACCTGAAGCC
CCAGTGACTGGCTATATGTTTGGCAAAGGTATCTACTTCGCAGACATGGTGTCCAAAAGT

GCAAACTATTGTCATACCTCTCAAATTGATCCTGTTGGCCTAATCTTGCTGGGAGAAGTT
GCTCTTGGAAACATGTATGAATTGAAAAATGCTTCCCATATAACAAAGCTACCAAAAGGC
AAACACAGTGTCAAAGGTTTGGGTAAAACAGCCCCAGATCCCTCAGCCACTGTTTCTCTT
GAAGGTGCAGATGTTCCTTTAGGAAAAGGAATTGCATCTGGAATTAGCAGTACTTCTCTT
ATGTATAATGAATACATTGTCTATGATATTGCTCAGGTAAAACTGAAGTATCTGCTGAAA
TTAAAGTTCAACTATAAGACAACTCTCTGG
>Gharial_PARP1
ATGGCGGAGCCAGCGGACAAGCTGTACCGAGCCGAGTACGCCAAGAGCGGGCGGGCCTCG
TGCAAGAAATGCGGAGAGAGCATCGCCAAGGACTCGCTGCGCCTGGCCATCATGGTGCAG
TCACCCATGTTTGATGGCAAAGTCCCTCACTGGCATCATTACACTTGCTTTTGGAAGCGG
GCTCGGATCACGTCTCATGCGGATATTGATGGCTTCCCTGAGCTGCGATGGGAAGATCAG
GAGAAAATAAAAAAAACCATTGAAGCGGGGGGACCTGCCACAGGTAAAGGTGGTGACCAG
GAAGGAGGTGGCAAGGCTGATAAAAGCTTACATGACTTTGCAGTAGAATATGCCAAGTCT
AACAGAAGTACCTGCAAAGGCTGTGAACTGAAAATAGAAAAGAGTCAGATCAGAATTTCT
AAGAAGATGGTGCATCCGGAAAAGCCCCAGCTGGGAATGATAGATAACTGGTACCATCCA
GACTGCTTTGTGAGCCGCCGAGCAGAGCTGGGCTTTCTTCCTGCATTTGGTGCCAGTCAG
CTCCAGGGCTTTGGCATTTTGGTAGCTGAGGATAAAGAATCTCTGAAGAAGCAGCTGCCT
GCCGTCAAGAGTGAAGGAAAAAGAAAAGCAGATGAGGTGGATGGGACTGTGACTACAAAA
AAGAAACAAAAAAAAGAAAAGGATAAAGAATCCAAACAGGAGAAGCTGCTGAAGGAACAG
ACAGAATTGATTTGGAACATCAAAGATGAACTGAGGAAAGCCTGCTCCACCAATGACCTG
AAAGAGCTGCTGATAGCCAACAAACAGGAAGTGCCTTCCGGGGAGTCTGCTATCTTGGAC
AGAGTGGCAGATGGGATGGCATTTGGAGCTCTGCTTCCCTGTGAAGAGTGCAAGGGGCAG
TTCATGTTCAAGAGTGATGCGTATTACTGTTCAGGAGACATTACTGCCTGGACTAAATGT
GTTGCCAAAACACAGTCTCCCAACAGGAAAGAATGGGTTATCCCAAAGGAATTCCGGGAA
ATCCCTTACCTAAAGAAATTCAAGTGTAAAAAGCAGGACAGAGTATTCCCTCCAGAGGCT
GCTGTTGTGAACACTGTGCTTCCATCTGCAGCTTCTGCTCCTTTGACAGAGGAGGCATCT
GCACCCACAGATAAGCCATTATCCAACATGAAGATTTTGATACTTGGAAAATTATCCAGG
AACAAAGAAGAAATGAAGACCACAGTTGAGGAGCTTGGAGGAAAAGTGACAGGAACTGCC
AATAAGGCCAACCTGTGTATCAGCACACAAAAGGAAGTTGAGAAAATGAACAAGAAGATG
GAAGAAGTGAAAGAGGCAAAAGTCCGAGTGGTTTCAGAAGAGTTTCTCAAGGATGTGAAA
TCCTCTAGCAAAGGCTTTCGGGAACTGCTGTCCGTACATGGGCTCTCATCCTGGGGTGCA
GAGGTGAAGCAGGAGAACATGGAGACAACTGTGGGAGGAAAGTCCAGTGGACCCCCAAAT
ATGAAGAGTGCTGGAAAAGTCAAGGAAGAACAAGGGACCTGCAAGTCTGAAAAGAAAATG
AAATTGACAGTTAAAGGAGGAGCAGCCGTAGATCCTGATTCTGGTTTGGAGGACTCTGCT
CACGTCTTTGAAAAAGGTGGTAAAATCTACAGCGCCACTCTTGGCTTGGTGGATATTGTC
AAAGGAACCAATTCCTATTACAAGCTGCAGCTGCTAGAGGATGACCGAGAAATCAGGTAC
TGGGTGTTTAGGTCATGGGGTCGCGTTGGCACTGTGATTGGGAGTAACAAGCTGGAGCAG
ATGCCATCTAAAGAAGATGCCATTGAGCACTTTTTGAATCTTTATGAAGAGAAAACGGGC
AATTCCTGGCATTCAAAGAACTTCACGAAATATCCAAAGAAATTCTACCCGCTAGAAATA
GATTATGGACAGGATGAAGAAGCTGTGAAGAAACTGACAGTAGGTGCCGGCACAAAGTCA
AAGCTTCCTAAATCAGTCCAGGACCTTATCAAGATGATCTTTGATGTGGAGAGCATGAAG
AAAGCCATGGTGGAATTTGAGATTGACCTCCAGAAGATGCCATTGGGAAAGCTGAGCAAG
AGACAGATCCAGAGTGCATATTCCATCCTTAATGATGTTCAGCAGGCAGTTTCTGGTGGC
GGCACTGATTCTCAGATACTGGATCTCTCCAATCGTTTCTACACATTGATACCTCATGAC

TTCGGGATGAAGAAGCCACCTCTTTTAAATAACCTAGAATATATTCAGGCCAAAGTGCAG
ATGTTGGACAACCTGCTTGATATTGAGGTTGCTTACAGCCTTCTCAGAGGTGGAAACGAG
GATGGGGATAAAGACCCAATTGATGTCAACTATGAAAAGCTCAAAACTGAAATTAAGGTG
GTTGATAAAGATTCAGAAGAAGCCAAGATCATAAAGCAATATGTGAAGAATACCCATGCT
GCTACCCACAATGCATATGATCTGAAAGTTGTGGATATCTTCAAGATTGAGCGCGAAGGG
GAGAGTCAGCGTTACAAGCCATTCAGACAGCTTCACAATCGCCAGCTGCTCTGGCATGGC
TCACGTACTACCAACTTTGCTGGTATTCTCTCGCAAGGTCTCCGAATAGCTCCACCTGAA
GCTCCTGTGACTGGCTACATGTTCGGAAAAGGTGTCTATTTTGCAGACATGGTTTCCAAG
AGCGCCAACTACTGTCACACATCTCAGGCTGATCCAGTAGGCTTAATCTTATTGGGAGAG
GTTGCCCTTGGAAATATGTATGAGCTAAAGAATGCTTCTCACATAACTAAGTTGCCGAAG
GGGAAACATAGCGTCAAAGGTTTGGGCAAAACTGCACCTGATCCCACAGCCACCATCAGT
TATGAGGGTGTAGAAGTTTCTTTGGGGAATGGAATGTCAACAGGAATTAATGATACTTGT
CTTCTGTATAATGAATACATTGTCTACGATGTTGCTCAGGTAAACCTGAAGTACTTGCTG
AAACTGAAATTCAACTACAAGACATCACTCTGG
>tropical_clawed_frog_PARP1
ATGGCCGAGGCCTCGGAGAAACTTTATCGGGCTGAGTACGCCAAGAGCGGCCGCGCCTCC
TGTAAGAAATGCGGAGACAACATCGCCAAGGAGTCCCTGCGGCTTGCCCTCATGGTGCAG
TCCCCAATGTTCGATGGCAAAGTGCCACACTGGCACCACTACTCCTGTTTCTGGAAACGC
GCCCGGGTCTTATCCCATGGGGATATCGATGGTTTCACTGAGCTCCGATGGGACGATCAG
GAAAAAATCAAGAAAGCCGTTGAAACCGGAGGGGCTTCTGCAGGGGCCGGAGGGGATGCC
AAGGGAGGCAAAGGGGAGATGACGCTGAATGATTTTGCAGTAGAATATGCAAAGTCTAAC
AGAAGCACCTGCAAAGGCTGTGAGAAGAAAATAGAAAAGGGGCAGATCAGAATATCCAAG
AAGATGGTGGATGTGGAGCGGCCCCAGCTGGGAATGATCGACCGGTGGTATCACTCAGAC
TGCTTTGTGAGCTGCAGGGAGGAGCTGGGCTTTCTCCCCTCCTACAGCGCCAGCCAGCTC
AAGGCTTTCTCCATTCTAAAAGCTGAGGACAAAGATGCCCTGAAGGCAATGCTTCCCGCT
GTAAAGAATGAAGGGAAGAGGAAGGCAGATGAGGTGGATGCAAGTTCCGCTGGAACCAAA
AAGAAATTAAAGAAAGAGAAGGAAAAAGAGTCAAAGCTGGAAAAGTTGCTAAAGGAGCAG
ACAGAGCTTATTTGGCACATTAAGGATGAGCTGAAAAAAGTTTGCAGCACCAACGATTTG
AAGGAGCTTCTCATCGCCAACAAACAGCAAGTGCCGTCCGGTGAGACCAACATTTTGGAC
CGGGTATCAGATGGCATGGCATTTGGAGCACTGCTTCCGTGCGAGGAGTGCAGCGGGCAG
TTTGTTTTCAAGAGCGACGCTTATTATTGCACCGGGGACCTCAGCGCCTGGACAAAATGT
GTTGCTAAAACACAGACTCCCAACAGAAAGGATTGGGTGACACCTAAGGAGTTCCATGAA
GTCCCTTATTTGAAGAAATTTAAGTTCAAAAGACAAGACAGAGCTTTCCCGCCTTGTGCC
GCTCCAACACCTATCAGCCCCCCGGCTGCCCCTAAACCAGCGCCGACTGTGGAGGAAACT
GTTCCTGAAGGGAAACCTTTAACCAATGCAAAGGTGCTTGTCATTGGGAAGCTGTCCAAA
AACAAGGATGAGGTAAAGGCGTTGATTGAAGGACTGGGCGGCAAAGTCACCGGCTCTGCA
CACAAGGCCACGCTGTGCATCAGCACTAAAAAGGAAGTTGAGAAGATGAGTAAGAAGATG
GAGGATGTGAAAGCAGCCAATGTGCGTGTAGTAAGTGACGACTTCCTGAAGGAAGTGGAG
TCGGGGAAGAGCGTGCAGGAATTGTTGTCCCAATACGGAATATCCTCATGGGGTGCTGAG
GTCAAGCAAGAGGCCGTGCAGCCAACGGAGAAGCAGCCGTCCAGCGCCCCCGCGGCTGGG
AAGAGTTCGGGCAAAGTGAAAGAAGAGAAAGGGAGCAACAAGTCTGAAAAGAAAATGAAA
TTAACTGTTAAAGGGGGAGCAGCCATTGACCCCGATTCTGGGCTGGAAGATTCATGTCAC
GTCTTGGAAAAAGGAGGGAAGATTTTTAGTGCAACTCTGGGCCTGGTGGATATAACCAGA
GGGACAAATTCTTATTATAAGCTGCAACTTATAGAGCACGACAGAAATTCCAGGTACTGG

GTGTTCCGATCCTGGGGTCGAGTGGGCACTGTAATAGGCAGTAACAAGCTAGAAGAGATG
TCTTCCAAGGAAGATGCCATTGACCATTTCCTCAACCTGTATCAGGAGAAAACCGGCAAT
GCATGGCACTCCCCCAATTTCACCAAATATCCCAACAAGTTCTATCCTCTGGAAATAGAC
TATGGGCAGGAAGAAGATGTGGTGAAGAAACTCTCGGTTGGGGCCGGCACAAAATCCAAG
CTTGCCCAGCCTGTCCAGGAACTGATCAAGTTAATATTTGATGTGGAGAGCATGAAGAAG
GCCATGGTGGAGTTTGAGATCGATCTCCAGAAGATGCCTTTGGGGAAGCTCAGCAAGCGG
CAGATTCAGAGTGCATACTCAATACTCAATCAAGTGCAGCAGGCCGTCTCGGAATCCTCA
AGCGAAGCTCGGTTATTGGATCTCTCCAATCAGTTTTACACACTCATACCTCATGATTTT
GGAATGAAGAAGCCCCCGCTGCTAAATAATCTAGAGTACATACAGGCTAAAGTGCAGATG
CTGGATAATCTGCTTGACATTGAAGTTGCATACAGCCTGTTGAGAGGCGGCGCCGATGAT
GGAGAAAAAGATCCCATTGATGTGAAATATGAAAAGATTAAGACTGACATTAAGGTTGTC
GATAAAGAATCGGAAGAATCTAGAATAATACGTGACTATGTGAAGAACACGCACGCCGAG
ACGCACAATGCCTACGATCTTGAGGTCCTCGAGATATTCAAAATCGAGCGTGAAGGTGAA
TATCAGCGGTATAAGCCATTCAAACAGCTACACAACCGCCAGCTGCTTTGGCACGGATCC
CGAACCACAAATTTTGCAGGGATATTGTCTCAGGGTCTCCGAATTGCTCCACCGGAAGCT
CCCGTTACTGGCTATATGTTTGGCAAAGGTATCTATTTTGCCGATATGGTATCCAAAAGT
GCAAACTATTGTAATGCAATGCCGGGTAATCCCATTGGGCTGATCTTACTGGGAGAGGTG
GCCCTTGGTAACATGCATGAGCTGAGAGCAGCATCACACATTACGAAACTACCCAAGGGC
AAACACAGTGTCAAAGGTTTGGGTCGAACCGCGCCTGATCCCTCAGCTACTGTAAAACTG
GATGGAGTGGACGTTCCTCTTGGGAAAGGAAGTGCTACAAATATCACTGACGCCAGTCTG
TTATATAACGAATACATTGTGTATGATGTTGCTCAGGTTAACCTGAAGTACCTGCTGAAG
CTGAAGTTCAACTACAAAGACAAAGGAGGGTTGCTTTGG
>African_clawed_frog_PARP1
ATGGCCGAGGCCTCCGACAAACTTTACCGGGCTGAGTACGCTAAGAGCGGTCGCGCCTCT
TGCAAGAAATGCGGCGACAACATCGCCAAGGAGTCTCTGCGTCTCGCCATCATGGTGCAG
TCCCCAATGTTCGATGGCAAAGTTCCGCACTGGCATCACTACTCGTGTTTCTGGAAGCGT
GCCCGAGTCTTATCCCAAGGGGATATTTATGGTTACACAGAGCTCCGATGGGAAGATCAG
GAAATGATTAAGAAAGCCATTGAAACTGGAGGGGCTGCTGCAGGAGCTGGAGGAGATTCC
AAGGGGGGCAAAGGGGAGATGACTCTGAATGATTTTGCAGCCGAATATGCAAAGTCAAAC
AGAAGCGCCTGCAAAGGCTGTGAGCAGAAAATAGAAAAGGGGCAGATCAGAATATCCAAA
AAGAGTGTGGATGTGGAGCGGCCCCAGCTGGGGATGATTGATCGGTGGTACCACCCAGAC
TGCTTTGTGAGCAGCAGAGAGGAACTGGGCTTTCTTCCCTCCTACAGCGCCAGCCAGCTT
AAGGGCTTCACCATCCTAAGTGCAGAAGACAAAGACTCCTTAAAGAAAAAGCTTCCAGCT
GTAAAGAATGAAGGGAAGAGGAAGGCAGATGAAGTGGACGGACATTCCGCTGCAACCAAA
AAGAAAATAAAGAAAGAGAAGGAGAAAGAGTCAAAGCTGGAAAAGCTGCTAAAGGAGCAA
ACGGAGCTGATTTGGCATATTAAGGATGAACTGAAAAAAGTTTGCAGCACCAATGATTTG
AAGGAGCTTCTCATAGCCAACAAACAGCAGGTGCCGTCCGGTGAGACCAACATTGTGGAC
CGGGTATCGGATGGCATGGCATTTGGAGCGCTGCTTCCATGCGAGGAATGCAGTGGGCAA
TTTGTTTTCAAGGGCGACGCTTATTATTGCACTGGGGACCTCAGTGCCTGGACAAAATGT
GTTGCTAAAACACAAACTCCCAACAGAAAGGATTGGGTGACACCAAAGGAGTTCCATGAA
ATTCCTTATTTGAAGAAATTTAAGTTCAAAAGACATGACCGAGCTTTCCCTCCATGTGCT
GCTCCTACACCTATCAGCCCACCAGCTGCCCCTGAACCGAAACCGACTGTGGAGGAAACT
TTTCCTGAAGGGAAACCTTTAACCAATACGAAGGTTCTTCTTATTGGAAAGCTGTCAAAA
AACAAAGATGAAGTAAAGACGTTGATTGAGGGACTGGGCGGCAAGGTCGCTGGTTCTGCA

CACAAGGCCAATCTGTGCATCAGCACTAACAAGGAAGTTGAGAAGATGAGTAAAAAGATG
GAGGAAGTGAAAGCAGCCAATGTGCGTGTAGTAAGTGACGACTTCCTAAAGGAAGTGGAG
TCGGGGAAGAGCGTGCAGGAATTGTTATCCCAGTTTGGAATATCTTCATGGGGGGCTGAA
ATCAAGCAGGAGGCTGTGCAGCCAACAGAAAAGCAGCCCTCCAGTGGGCCCGTGGCTGGG
AAGAGTTCGGGCAAAGTAAAAGAAGAGAAAGGGAGCAACAAGTCTGAGAAGAAAATGAAA
TTGACTGTTAAAGGGGGAGCAGCCATTGACCCTGATTCTGAGCTGGAAGATTCATGTCAT
GTCTTGGAAACGGGAGGAAAGATTTTCAGTGCAACTCTGGGACTGGTGGATATAACCAGA
GGGACAAACTCCTATTATAAGCTGCAACTTATAGAGCACGACAGAGATTCCAGGTACTGG
GTGTTCCGATCCTGGGGTCGGGTGGGCACAGTAATAGGCAGTAAAAAGCTAGAAGAGATG
TCTTCCAAGGAAGATGCCATTGAACATTTCCTCAACCTGTATCAGGACAAAACTGGCAAT
GCATGGCACTCGCCCAATTTCACCAAATACCCCAAAAAATTCTATCCCCTGGAAATAGAC
TATGGACAGGAAGAAGATGTGGTGAAGAAGCTCTCAGTGGGGGCCGGCACAAAATCCAAG
CTCGCTAAGCCTGTCCAGGAACTGATCAAATTAATTTTTGATGTGGAGAGCATGAAAAAG
GCCATGGTGGAGTTTGAGATCGATCTCCAGAAGATGCCTTTGGGGAAACTCAGCAAGCGG
CAGATTCAGAGTGCGTACTCTATTCTTAGCCAAGTGCAGCAGGCCGTGTCTGAATCCTTA
AGCGAAGCCCGATTACTGGATCTCTCCAATCAGTTTTACACACTCATACCTCATGATTTT
GGAATGAAGAAGCCCCCGCTGCTAAATAACCTAGAGTACATACAGGCTAAAGTGCAGATG
CTGGATAATCTGCTCGACATTGAAGTTGCTTACAGCCTGTTGAGAGGTGGCGCCGATGAT
GGTGAAAAGGATCCCATTGATGTGAAATATGAAAAGATTAAGACTGACATTAAGGTTGTT
GCTAAAGATTCAGAAGAATCCAGAATTATATGCGATTATGTCAAGAACACGCACGCAGAT
ACGCACAATGCATATGATCTTGAGGTCCTCGAGATATTCAAAATCGACCGTGAAGGTGAA
TATCAGCGGTATAAACCATTTAAACAGCTACACAACCGCCAGCTGCTTTGGCACGGCTCC
CGCACCACAAATTTTGCAGGAATATTGTCTCAGGGTCTCCGAATTGCTCCGCCAGAAGCT
CCTGTTACCGGGTATATGTTTGGCAAAGGTATCTATTTTGCCGACATGGTATCCAAAAGT
GCAAACTACTGTCATGCAATGCCGGGTAGCCCCATAGGGCTGATCTTACTGGGGGAGGTG
GCCCTCGGAAACATGCATGAGCTGAAAGCAGCATCACAAATTACAAAACTTCCCAAGGGC
AAACACAGTGTAAAGGGTTTGGGTAGAACTGCACCAGATCCCTCAGCTACTGTACAGCTG
GATGGAGTGGATGTTCCTCTCGGAAAAGGAACTTCTGCAAATATCAGTGACACCAGCCTG
TTGTATAATGAATATATTGTGTATGATATTGCTCAGGTCAACCTGAAGTACCTGCTGAAG
CTCAAGTTCAACTACAAAGGAGGGATGATGTGG
>Tibetan_frog_PARP1
ATGGCCGAGTCCGCCGACAAGCTCTACCGGGCCGAATACGCCAAGAGCGGCCGCGCCTCC
TGCAAGAAGTGCGGCGACAACATCGCCAAGGAGTCGCTGCGGCTGGCCATTATGGTGCAG
TCGCCTATGTTTGATGGGAAAGTACCACACTGGCACCATTATTCCTGTTTCTGGAAGCGT
GCTCGGGTCATATCCCACGGGGATATTGATGGCTTCACAGAGCTTCGATGGGAGGATCAG
GAAAAGATTAAGAAGGCCATCGAAACTGGTGGTGCCTCAGCAGGAGGGGACGCCAAAGGA
GGCAAAGCAGACATGACACTGAATGATTTCGCAGCTGAATATGCAAAGTCCAACAGAAGC
ACATGCAAAGGCTGTGAGCAGAAAATTGAGAAGGGTCAGATAAGATTGTCTAAGAAAAGC
TTGGATGTGGAGCGACCTCAGCTTGGCATGATCGATCGCTGGTACCACCCAGACTGCTTT
GTAAGCCGCCGGGAAGAGCTTGGCTTCCTATCAATGTACAGTGCCAGCCAGATGAAGGGC
TTTAGCATTCTGAAAGCTGAAGACAAGGAGGCTCTGAAAGCCAAGCTCCCAGCAGTGAAG
AATGAGGGGAAGAGAAAAGCGGATGAGATTGATGGATCTGCTGCGTCCAAAAAGAAACTA
AAAAAAGAGAAGGAAAAGGAATCCAAACTTGAGAAGCTGCTGAAGGAACAGACAGAGCTC
ATCTGGAGTATAAAAGATGAACTGAAGAAAGTTTGCAGCACCAACGACCTGAAAGAGCTG

CTTATAGCCAACAAACAGCAGGTGCCTTCCGGAGAAACCAATATCCTGGACAGAGTATCA
GACGGCATGGCTTTTGGGGCTCTCCTTCCTTGCGAGGAATGTCAGGGACAGTTAGTGTTC
AAGACTGATGCTTATTACTGTACAGGGGACATCAGTGCCTGGACCAAATGTGTTGTCTTA
TATTATCTTATTTTTGCTATGTTTTTTTTCTTGTTTTTCATGCATGTGTTAATCCAGGAG
TTCCATGAAGTTCCATATTTGAAAAAGTTCAAGTTTAAGAGGCAGGACAGAGCGTTCCCT
CCAGCTGTAGCTCCAGCACCGGCTACCGCTCCAGTTGCCACTAAGCCTGTGATTAAAGAG
GAAGCAGTTCCTGATGGTAAGCCACTGACCAATATGACCGTTATGACCATTGGCAAACTG
TCGAAGAACAAAGATGAAGTTAAAGCGATGGTTGAAGAACTAGGTGGAAAGACGACTGGG
TCCGTCAGCAAAACCACAGTATGCGTCAGCACTCAAAAGGAGGTGGAGAAGATGAGTAAG
AAAATGGAGGATCTAAAAGCGGCCAATATTCGAGTGGTCAGCGAGGACTTCCTGAATGAG
CTGCAGTCTGGCAAGAGCTTACAGGACCTGCTTACCCAGCATGGCATCTCTGCTTGGGGT
GCAGAAATAAAACAGGAGGCACAACAGGAGCCCCAGGCTAAAACAAAGTCCGGGAAGAGT
ACTGGCAAAGTGAAAGAGGAGCAGGGTAACAAATCTGAGAAAAAAATGAAGTTGACTGTT
AAAGGTGGAGCAGCTGTTGATCCAGACTCTGGGCTTGAAAATTCAGGTCATGTGTTGGAG
AAAAGCAGCCAGATCTTCAGCGCCACCCTGGGCCTGGTAGATATATCTCGAGGGACAAAC
TCCTACTATAAACTGCAGCTGATCGAACATGACAGAGATGCCAGGTACTGGGTCTTCCGG
TCCTGGGGCCGAGTCGGAACAGTTATAGGCAGTAACAAACTGGAGGAAATGTCTTCCAAA
GAGGAAGCCATTGACCATTTCCTTAACCTGTATCAAGAGAAGACTGGCAATGCCTGGCAC
TCCACCAACTTCACCAAATATCCCAATAAGTTCTACCCACTGGAAATAGACTACGGCCAG
GAAGAGGATGTGGTGAAGAAGCTTTCAGCTGGAGCGGGAACAAAGTCCAAACTTGCAAAG
CCAATACAAGAACTCATTAAAATGATATTTGATGTGGAGAGCATGAAAAAAGCCATGGTG
GAGTTTGAGATTGATCTACAGAAGATGCCTCTAGGCAAACTTAGTAAGCGACAGATTCAG
AGTGCATACTCTATCCTGAATGAGGTACAGCAGGCTGTATCTGATAGCTCTCCTGAAGCT
CGCTTGCTGGATCTGTCCAATCGCTTCTACACGTTAATACCTCATGATTTTGGAATGAAG
AAGCCCCGTCTCTTGGATAACGTAGAATACATCCAGGCAAAGGTGCAGATGCTGGATAAT
CTGCTAGATATAGAGGTCGCTTACAGTCTTCTGAGGGGTGGTACAGAAGATTCAGAGAAG
GATCCCATTGATGTCAAGTATGAGAAGATTAAGACTGTTATTAACGTTGTAGATAAGGAC
TCTGAAGAAGCCAAGATAATCCATCAATATGTTAAGAACACACATGCGGATACACACAAT
GCCTATGATCTTGAGATCCTTGATATCTTCAAGATTGAGAGAGAAGGGGAGAAACAGAGG
TATAAGCCATTCAAACAGCTAAACAACCGTCAGCTGCTCTGGCACGGATCCCGAACCACA
AACTTTGCTGGAATCTTGTCTCAAGGTCTGCGGATTGCCCCTCCTGAAGCACCTGTTACC
GGATACATGTTCGGTAAAGGCGTCTATTTTGCCGACATGGTGTCTAAAAGTGCAAACTAC
TGTCACACAACGCCGAGTAACCCCACAGGTTTGCTCCTACTGGGAGAAGTCGCTCTCGGA
AACATGTGTGAGTTGAAAGCTGCTTCTCACATAACAAAATTACCTAAGGGTAAACATAGT
GTGAAAGGTTTGGGCAGAACTGCACCTGATCCTGCTGCTACTGTACAACTTGATGGAGTG
GACGTTCCTCTGGGGAAAGGCGTTAATACCAACATTAGTGACACCGGTCTACTGTATAAT
GAATATATTGTGTATGACATTGCCCAGGTGAACCTGAAGTACCTCTTGAAGCTCAAGTTC
AACTACAAAGGCGGACTCATGTGG
>southern_platyfish_PARP1
GTTGTTGACAAGACCACGCAGGAGGCTGAGATCATAATGCAATACGTTAAGAACACCCAC
GCGGCTACACACAACACCTACACCCTGGAAGTGCAAGACATCTTCAAAGTTGCACGAGAA
GGAGAGCACCAACGCTACCGTCCATTCGAGGAGCTACACAATCGACAGCTACTGTGGCAC
GGCTCTCGCGCCACAAACTATGCTGGTATTATGTCTCAGGGTCTTCGCATTGCACCTCCA
GAGGCCCCAGTGACTGGTTACATGTTCGGCAAAGGTGTGTACTTTGCTGACATGGTGTCC

AAGAGTGCAAACTACTGTCACACCTCTCAGTCAGACCCTGTGGGACTCATTCTGCTGGCA
GAGGTCGCTCTAGGCAACATGCATGAACTGAAGAAAGCCTCTCACATTACAAAATTACCA
AAGGGAAAGCACAGTGTTAAAGGTTTGGGTAGAACCGCTCCTGATCCAAATGCTTCTACC
ACTTTGAATGGGGTGCAAGTGCCTCTGGGAAAAGGAGTCAATACTAACATTGATGACACA
AGTCTACTGTACAACGAGTACATTGTATATGATGTGGCTCAGATAAACTTGAAGTATCTC
CTGAAGATCAGGTTTAACTACCAGACATCTCTGTGG
>Cod_PARP2
ATGGTTCAAATTAACCCCGCCACCAAGTACAAAAGAAAAATACGCTCTCAGATGGTGAAA
TCAGAGGTGGTAAAGACCGTGATCATGAAGGGGAAGGCTCCAGTGGACTCTGAGTGTAAG
GCCAAGCTGGGCAAGGCCCATGTTTACAGTGAAAGGGAGGACGTCTATGACGTCATGCTA
AATCAGACAAACCTGCAGTTCAACAACAACAAGTACTACCTGCTCCAGCTGCTAGAAGAC
GACAGCTCCAAGAGCTATAGCGTGTGGCTGAGATGGGGCAGAGTTGGTAAATCGGGCCAG
AGCAGCTTGACAGCCTGCGGAGGAGACCTGAACAAGGCCAAAGACTTCTTCAAGAAAAAA
TTCGAGGAAAAGACAAAGAATAACTGGGAACACCGGCTTAACTTTGAGAAAGTCCCTGGA
AAATATGACATGGTGTTCATGGACTACAGTACCTCTTCAAAGGAGGAGCCCACGCTGGAG
GTGGACGCCGCGCCCAAGAAGTGGACCTCCAAGCTGGATGAGAAGGTCCAGTCCCTGCTG
GAGCTCATCTGTGACATCAAGGCCATGGAGGAGTGTGTGCTGGAGATGAAGTTTGACACT
CGCAAAGCCCCCCTGGGCAAGCTGACGTCGGAGCAGATCCGAGCGGGCTACGCCGCCCTG
AAGCGGATCGAAGAGTGCCTGAAGAAGAAGGACAGCCGCAAAGACCTGTTGGAGGCCTGC
AACCAGTTCTACACTCGCATCCCCCACGACTTTGGGCTGAAGACACCCCCCATGATCAGC
TCTGAGGACGACCTGAAGGAAAAGATAGCTCTGTTGGAGGCCCTCAGTGACATCCAGATC
GCCGTGAAGATGGTCCAGGCCAGTGCCAACGCGGAGGAGCATCCCCTGGACCGCCAGTAC
CACTCCCTCCAGTGCCAGCTGCAGCCCCTGGACGCCGGCAGCGACGCATACCAGTACCTG
CAGAGCACCCACGCCCCCACCCACAGCGAGTACACCATGAGCGTCCTGGACATCTTCTCC
GTGGACCGCCAGGGCGAGTCGGAGAAGTTCCTCTCCGGGATGCACAACAAGACTCTGCTG
TGGCACGGATCCCGTCTGTCCAACTACGTGGGCATCCTGAGTCAGGGGCTCCGCGTGGCC
CCCCCCGAGGCCCCCGTCACCGGATACATGTTCGGTAAAGGGATCTACTTCGCCGACATG
TCGTCGAAGAGCGCCAACTACTGCTTCACCAACCAGCGCAACAACATAGGCCTGCTGCTG
CTGGTGGCGCTGGGGGACTGCAACGAGCTCCTGGACGCAGACTACGAGGCGCAGAAACTC
CCCGAGGGCAAACACAGCACCAAGGGCATGGGCCAGACCGGCCCGGACCCCAAGAACGCC
GTCACCCTGGACGGCTTCTCGGTGCCCATTGGCCCGCCGGTGAAGACGGGTGTGGGCCGG
GCGGGGTCCTACTCCCTCCTCTACAACGAGTTCATCGTGTACAACCCCGCCCAGGTCCGC
ATGCGCTACCTGCTCAGAGTCCAGTTCAACTACCCCTCCCTGTGG
>African_clawed_frog_PARP2
ATGTCGGGGCGCAGTGCGAATGTCCAAGGGTGGGTGAGAAAGACCCGGGTTTCCCAGAAG
AGAAAAGCAGATTCTGATCCAGAACCAGAACTTGTGGTGAAAATAGAACCAGAAGAGGAG
AATAATGTTGCTACAGATGTTTGTAATGGAGAGGCAAACACTAATATAAAAAGAGAGCTA
AACTATTGCTGGGAATGGGAAGGAGATGGTGGCATTTGGATCCCATTTTCCCCTGAGCTC
AACACGCAGATTAATTTGTCATTCAGTACAGGGAAACGATCTGTGACGGTCTCACCAGCT
GCAGGGGTGTCTCTACAGGTGGATGTACAGAAAATGGTTCAGAAAAACACACAGAGTGGG
TTCCAGAGATCGGTCCGACTGGCAGTGCAAGATCGTGACAAATATTTTGTGTGGCAGTGG
TGGTCTGATGAGGGGGCTTGGATTTCATATGATGCTTGCACATGTGTAGCTCTGGAGGAA
GGTTTTCAATCAGAAGCTAAGGTGGTGCCTGTAAGCTTGGGAGGTCGTCCATACATCATA
GATCTAGAAGCGATGGTGCAAAAGAATAGCCAAACCCAATATGAAAGACAGATTCAACGC

AGCTTATCTGGTTCTAACAGTGCTCCTCAGCAAAAGAGCATCATTAGTGCACCTGCAAAG
AGAGCCCGTGGAAGCAACAGTGTAAGAAATGTAGAAAATGAATCAGAGGAGAATAAAGAA
CAGGTTAGGACTCTGATAATGAAAGGTAAAGCTCCAGTAGATCCTGAATGCTCCATAAAG
CTTGGGAAGGCTCATGTTTTCTGTGAGGGAGATGATGTGTATGATGTCATGTTGAACCAG
ACCAACCTTCAGTTCAATAACAATAAGTACTACCTTATCCAATTGCTGGAAGATGATGGT
GTGAAAAAATTTCTCTGTTTGGATGCGTTGGGGAAGAGTTGGCAAAGTTGGACAGCACTCT
CTGGTATCCTGTGGAAGCGATCTACAAAAATCAAAAGACATTTTCCAAAAAAAGTTTTAT
GAGAAGACTAAAAACTTATGGACTGAACGAGCACAGTTTGAAAAATGTGCTGGAAAATAT
GATATGCTGCAGATGGACTATAATGCAACCGAGGAGGAAAAAGAGACAACAGTGAAGGAA
GAGAGCATGGCTGACGTTCCCAAACCAGAATCCCAATTGGATGACAGTATCCAGGAGTTA
ATTGAGCTGATTTGTAATTTAAAGGCCATGGAGGAAACTGTACTTGAGATGAAATTCGAC
ACAAAGAAAGCTCCCCTAGGGAAACTGACAGTCGAACAGATCCGTGCTGGCTACTGCTCT
CTGCAGCAAATTGAAAACTGTATTAAGAAACAAAAGTTTGGCAGAGATCTCCTTGAGGCA
TGCAATGAGTTTTACACCCGCATTCCCCATGATTTTGGGCTACGAACACCTCCTCTTATT
AGGACGCTAGAAGACCTGACAGTAAAAGTTCGGCTACTAGAGGCATTGGGTGATATTCAG
ATTGCAGTGAAGTTGGCAAGCCTGGAGCTCAGCTCTCTGGAACACCCTTTCGACAGACAG
TATCGGCAGTTAAATTGTTCTATGCAGCCCTTGGACCAAACATCCAGCACCTTCCAGCTT
ATTGAGGGCTATCTGCAAAGCACCCATGCACCTACACACAATGATTATACCATGACACTC
CTGCGGGTCTTTGAGTTACAAAGAGTGGGAGAAGAGTGCAACTTCAGAGAAGACCTTCCT
AACAGGATGCTGCTGTGGCATGGATCTCGGCTAACTAACTGGGTTGGGATCCTTAGCCAG
GGATTAAGGGTGGCCCCTCCAGAGGCTCCAGTAACTGGCTATATGTTTGGAAAAGGAATA
TATTTTGCTGACGTCTCGTCAAAAAGTGCAAATTACTGCTTCTCATCCCGGGACAAAAAT
GTGGGGGTTCTCCTACTGTCTGAGGTGGCTCTTGGTGAATGCAATGAGCTTCTTGCTGCA
GATTGTGATGCCCAAAAGAAGATAAAGTCAAAGCACAGTACAAAAGGATTGGGAAGGAGC
ATCCCAGACCCCAAAAAGAGCATAATTCATGAAGGAGCAGTGGTTCCATTGGGTCCACTA
ATGGACACTGGACTGACAAATGACGGCGGCTACACATTAAATTACAACGAGTACATTGTT
TATGATCCTCATCAGGTTCGCATGAAGTATCTGCTACAGGTCCGCTTTAACTACAGTTCC
TTATGG
>American_alligator_PARP2
ATGGGCCGCAAGCGGGCAGCGCCAGAGGCGGGCGACGCGGACCCGGAGCTGCGCTGGGAG
TGGCAGGATGCTGCGGGCACTTGGCACCAGTACTCCCCGGAGCAGAGCAGGGTGCTCACG
CAGGCGGCCAGGGCAGGGAAGCCCAGCGTGGAGGTCGGGGCCTGCGTGGACCTGAGAAGG
ATGGTGCAAAAGGACACGCGGACGGGGCAGGACAGACCCGTGGCAGCTGCGGTCCGGGAC
CAGGACTCCTACTTCATCTGGCAGTGGCAGGGCGATGGGGAGGAGGACTGGCTCCCCTAC
CCTGCTGACACCTGCTTAGCTCTGGAGCGGGTGCGATGTGGGAAGGGGGGGCCAACCTTG
CAGATGACTGTTGGCCGGACCCGCTACACGCTGGACCCAGCCCAGATGATCCAGACCAAC
TCGAGAACCAGGTATCAACGCAAGATGGACCGCAGGGAGTCAGATGCAGTAGTTGATAGT
AAGGAATCTCAAGCGAGCAACACTCCCAGCTCCACAGCCCTCCAAGAGGCTCAAGCACTG
AAGAGGTCCCGGAGTAGGGGGTCCCTCCTAGACCCAGTGGCTAAAGAAGTCGGTGGGGGA
GAGGGCAAAGAGGCTGTTAAGACCCTAGTAATGAAGGGGAAAGCACCAGTGGATCCCGAG
TGCTCTGCTAAGCTTGGGAAGGCTCATGTGTACTGTGAGGGGGACGATGTCTACGATGTG
ATGCTGAACCAGACGAACCTCCAGTTCAACAACAACAAATTCTACATCATCCAGCTCCTG
GAGGATGATGGGCAGCGGAGCTACAGTGTCTGGATGCGGTGGGGGCGCGTGGGGCGTCCA
GGCCAGCACACGCTGGTATCCTGTGCTGGGGATCTTGCCAAAGCCAAGGATGTCTTCACC

AAGAAGTTTCTGGATAAGACCAAGAACAGCTGGCCCAGTCGGGGCAGCTTCCAGAAGGTG
CCAGGCAAATACGACCTGCTGCATATGGACTACCAGGCCCATGGTCCAGATGAGGAAAGC
CGCCCTGAGAAAGTCATGCCCCAACTCAAACCAGCCTCCCGGCTTGAGCCCCGCGTGCAA
GCACTGGTGGAGCTCATCTGTGACATCCAGACCATGGAGGAGATGGTGATGGAGATGAAG
TATGACACCAAGAAGGCCCCATTGGGGAAGCTGACGGCCGAGCAGATCCGGGCTGGGTAC
TGCTCGCTGCAGAAGGTGGAGGCGTGTCTGCAAGAGGGAAGCGCTGGCCAGGCCCTGCTG
GACGCCTGCAATGAATTCTACACCCGCATCCCGCACGACTTCGGGCTGCGAACACCCCCA
ATGATCCGGACAAAGCAGGAGCTGCAGGAAAAGATGCAGTTGCTGGAGGCCCTGGGCGAG
ATTGAAATCGCTATCAAACTGGTGCGCTCAGAGAGGCAGGACCACGAGCACCCGCTGGAC
CAGCACTACCGCAGACTGGGCTGCGAGTTGTGTGCCCTGGACAGGGACACCCACGATTTC
CAGGTGCTGGAGCAGTACCTGCTGACCACCCATGCACCCACCCACCGCGACTACTCCATG
GAACTGCTGGAGGCCTTTGCCCTGCGCCGCCCGTCTGAGGAGACGGCTTTCCGTGCTGAC
CTTCCCAACAGGGTGTTGCTGTGGCACGGCTCCCGGCTGGGCAACTGGATGGGCATCCTG
AGCCAGGGGCTGCGGATCGCACCCCCGGAGGCTCCTGTGACTGGCTATATGTTCGGGAGA
GGCATCTACTTTGCTGACATGTCCTCCAAGAGTGCCAACTACTGCTTTGCTTCCCGTCAG
AAGGATGTGGGGCTCCTGCTGCTATCAGAGGTGGCCTTGGGTGAGTGCAACGAGCTGTTG
GAGGCTAACGCTGATGCCCAGAAGCTCTTGAACGGGAAGCACAGCACCAAGGGGATGGGG
AAGATGGCTCCTGCTAACCGCACCAAGCTGGATGGCACCGTGGTCCCGCTAGGACCACCT
GTGGAGACCGGAGTGGTGAACCCCCATGGCTACACCCTGAACTACAATGAATTCATCATC
TATGACCCATGCCAGGTGCGCATGCGCTACCTGCTCAAGGTGCGCTTCAACTTCACGCAA
CTGTGG
>armadillo_PARP2
ATGGCTACTCGGCGGAGGAGAGCTGTCAGCAACCGCGCGCCAGCTTCTGGACCATTAAAT
GAAACTGAGGAAGTTAATGTTGGGACCACAGCTGCAGAAGATCCTCCTCCTGCCAGAAAA
AATCGCAGATGCCAGAGGCAGAGGTTGAAAAATGAGCCTGCGGCTGGAATGAAGGCTGAT
AGTAATGACAAGATAGAAGGCAAGCAAGAATCTGTGAAGACATTGCTTTTAAAAGGCAAA
GTTCCTGTGGACCCAGAGTGCACAGTCAAGGTGGGAAAGGCCCATGTGTACTGTGAAGGG
AGTGATGTCTATGATGTCATGCTAAATCAGACTAATCTCCAGTTCAACAACAATAAGTAC
TATTTGATTCAGCTATTAGAAGACGATGCCCAGAGGAACTTCAGTGTTTGGATGAGATGG
GGTCGAGTTGGAAAAACGGGGCAGCACAGCTTGGCAGCTTGTTCAGGGGACCTTAACAAG
GCCAAGGAAATCTTTCAGAAGAAATTCCTTGATAAAACGAAAAACAATTGGGAGGATCGT
GAGAAGTTTAAGAAGGTGCCTGGAAAATATGATATGCTACAGATGGACTATACCACCAAG
ACTCAGGGTAAAGAGGAAACCAAACAAGAGGATTCTCTTAAATCCTTTTTGAAACCAGAG
TCACGACTAGATCTTCGGGTACAAGAGCTGATAAAGTTGATTTGTAATGTCCAGGCCATG
GAAGAGATGGTGGTAGAAATGAAGTATGATCTCAAGAAAGCCCCACTTGGGAAGCTGACA
GTGGCGCAAATCAAGGCAGGTTACCAGTCTCTTAAGAAGATTGAGGATTGTATTCGGGCT
GGCCGGCATGGACGAGCTCTCATGGAAGCATGCAATGAATTCTATACCAGGATCCCACAT
GACTTTGGACTCCATGTCCCTCCATTAATCCGAACAGAGAAAGAACTGTCAGACAAAGTG
CAGCTGCTAGAGGCACTGGGAGACATTGAAATTGCCATTAAGCTGGTGACAACAGAACTG
CAAAGCTCAGAACACCCTTTGGACCAACACTATAGAAATCTACATTGTGCCTTGCACCCT
CTAGACCATGCAAGTCATGAGTTCAAAGTGATTTCACAGTACCTACAGTCTACCCATGCT
CCCACTCACAATGACTATACCATGACCTTGCTGGATGTTTTTGAAGTAGAGAAGGAGGGT
GAGAAAGAAGCCTTCAGAGAGGACCTTCATAACAGGATGCTGCTATGGCATGGTTCCAGG
CTGAGTAACTGGGTGGGAATCCTGAGCCATGGACTTCGAATTGCCCCACCTGAGGCCCCC

ATAACAGGCTACATGTTTGGAAAAGGAATCTACTTTGCTGACATGTCTTCCAAGAGTGCT
AATTACTGCTTTGCCTCTCACTTAAAGAATACTGGACTGCTGCTTTTATCAGAGGTGGCT
CTAGGTCAGTGTAATGAACTACTAGAGGCCAATCCTGAGGCAGAAGGATTACTTCAGGGC
AAACACAGCACCAAGGGGCTGGGCAAGATGGCTCCCAGCTCTGCCTGTTTCATCACCCTG
AATGGGAGTACAGTGCCCTTAGGACCAGCAAGTGACACAGGAGTTCTGAATCCGGAGGGT
TACACCCTCAACTACAATGAATTTATTGTCTACAACCCCAACCAAGTCCGCATGCGATAT
CTTCTAAAGGTTCAATTTAATTTCCTGCAATTGTGG
>Chinese_alligator_PARP2
ATGGGCCGCAAGCGGGCGGCGCCGGAGGCGGGCGACGCGGACCCGGAGCTGCGCTGGGAG
TGGCAGGATGCTGCGGGCACTTGGCACCAGTACTCCCCGGAGCAGAGCAGGGTGCTCAGC
CAGGCAGCCAGGGCAGGGAAGCCCAGCACGGAGGTCGGGGCCTGCGTGGACCTGAGAAGG
ATGGTGCAAAACGACACGCAGACGGGGCAGGACAGACCCGTGGCAGCTGCCGTCCGGGAC
CAGGACTCCTACTTCATCTGGCAGTGGCAGGGCGATGGGGAGGAGGACTGGCTCCCCTAC
CCTGTTGACACCTGCTTAGCTCTGGAGCGGGTGCGACGTGGGGAGGGGGGGCCAACCTTG
CAGATGACTGTTGGCCGGACCCGCTACACGCTGGACCCAGCCCAGATGACCCAGACCAAC
TCGAGAACCAGGTATCAACGCAAGATGGACCGCAGGGAGTCAGATGCAGTGGTTGATAGT
AAGGAATCTCAAGCGAGCTACACTCCCAGCTCCACAGCCCTCCAAGAGGCTCAAGCACTG
AAGAGGTCCCGGAGTGGGGGGTCCCTCCCAGACCTAGTAGCTAAAGAAGTCAGTGGGGGA
GAGGGCAAAGAGGCTGTTAAGACCCTAGTAATGAAGGGGAAAGCACCAGTGGATCCCGAG
TGCTCTGCTAAGCTTGGGAAGGCTCATGTGTACTGTGAGGGGGACGATGTCTATGATGTG
ATGCTGAACCAGACGAACCTCCAGTTCAACAACAACAAGTTCTACATCATCCAGCTCCTG
CAGGATGATGGGCAGCGGAGCTACAGTGTCTGGATGCGGTGGGGGCGCGTGGGGCGTCCA
GGCCAGCACACGCTGGTATCCTGTGCTGGGGATCTTGCCAAAGCCAAGGATGTCTTCACC
AAGAAGTTTCTGGATAAGACCAAGAACAGCTGGCCCAGTCGGGGCAGCTTCCAGAAGGTG
CCAGGCAAATACGACCTGCTGCATATGGACTACCAGGCCCACGGTCCAGATGAGGAAAGC
CGCCCTGAAAAAGTCATGCCCCAACTCAAACCAGCCTCCCGACTTGACCCCCGTGTGCAA
GCACTGGTGGAGCTCATCTGTGACATCCAGACCATGGAGGAGATGGTGATGGAGATGAAG
TATGACACCAAGAAGGCCCCATTGGGGAAGCTGACGGCCGAGCAGATCCGGGCTGGATAC
CTCTCGCTGCAGAAGGTGGAGGCGTGTCTGCAAGAGGGAAGCGCTGGCCAGGCCCTGCTG
GACGCCTGCAATGAATTTTACACCCGCATCCCGCACGACTTTGGGCTGCGAACACCCCCA
ATGATCCGGACAAAGCAGGAGCTGCAGGAAAAGATGCAGCTGCTGGAGGCCTTGGGCGAG
ATTGAAATCGCTATCAAACTGGTGCGCTCAGAGAGGCAGGACCACGAGCACCCGCTGGAC
CAGCACTACCGCAGACTGGGCTGCGAGTTGTGTGCCCTGGACAGGGACACCCACGATTTC
CAGGTGCTGGAGCAGTACCTGCTGACCACCCATGCACCCACCCACCGCGACTACTCCATG
GAGCTGCTGGAGGCCTTTGCCCTGCGCCGCCCGTCTGAGGAGACGGCTTTCCGTGCTGAC
CTTCCCAACAGGGTGTTGCTGTGGCACGGCTCCCGGCTGGGCAACTGGATGGGCATCCTG
AGCCAGGGGCTGCGGATCGCACCCCCGGAGGCTCCTGTGACTGGCTATATGTTCGGGAGA
GGCATCTACTTTGCTGACATGTCCTCCAAGAGTGCCAACTACTGCTTTGCTTCCCGTCAG
AAGGATGTGGGGCTCCTGCTGCTGTCAGAGGTGGCCTTGGGTGAGTGCAACGAGCTGTTG
GAGGCTAATGCTGATGCCCAGAAGCTCTTGAACGGGAAGCACAGCACCAAGGGGATGGGG
AAGATGGCTCCTGCTAACCGCACCAAGCTGGATGGCACCGTGGTCCCGCTAGGACCACCT
GTGGAGACCGGAGTGGTGAACCCCCATGGCTACACCCTGAACTACAATGAATTCATCATC
TATGACCCATGTCAGGTGCGCATGCGCTACCTGCTCAAGGTGCGCTTCAACTTCACGCAA
CTGTGG

>Dog_PARP2
ATGGCGGCGCGTCGGCAGGGGACACGCGGCGGCAGCGGGGCGAGAGCATTCGACAAAGCT
GGAAGAGTTCATAATGGCAACACGGCTGGAGAAGACCCTCCTCCTGCAAAGAAAACTCGG
AGATGCCAGAGGCAGGGGGTGAAAAGGGGGCCTTTGACTGCAGGAGGGGCCGGTAATGGC
AGCACGGAAGACAAGCCAGCAGAGTCTGTGAAGACCCTGCTGTTAAAAGGCAGAGCTCCC
GTGGACCCCGAGTGCGCAGCCAAGGTGGGGAAGGCCCACGTGTACTGTGAAGGGAGCGAT
GTCTATGATGTCATGCTAAACCAGACCAATCTTCAGTTCAACAACAATAAGTACTATTTG
ATTCAGCTGTTGGAAGATGATGCCCAGAGGCACTTCAGTGTCTGGATGAGATGGGGCCGA
GTTGGGAAAATGGGGCAGCACAGCTTGGTGGCTTGCTCGGGGGACCTCAACAAAGCCAAG
GAAATCTTCCAGAAGAAATTCCTTGACAAAACAAAAAACAATTGGGAGGATCGTGAGAAG
TTTGAGAAGGTGCCTGGAAAATATGATATGCTAGAAATGGACTATGCTACCAATACTCAG
AGTGAAGAGGAAACAAAAAAGGAGTCTCTTAAACTCCCCTTCAAACCAGAATCACAGCTA
GATCTTCGAGTGCAGGAGCTGATAGAGTTGATCTGTAATGTCCAGGCCATGGAAGAGACG
ATGGTAGAAATGAAATACGACACCAAGAAAGCCCCACTTGGGAAGCTGACAGTGGCACAA
ATCAAGGCAGGCTACCAGTCTCTCAAGAAGATTGAGGACTGTATTCGGGCTGGCCAGCAT
GGACGAGCTCTTGTGGAAGCTTGCAATGAATTCTACACCAGGATCCCACATGACTTTGGG
CTCCGCACCCCTCCTTTAATCCGGACAGAGAAAGAGCTGTCAGACAAAGTACAGCTACTG
GAGGCTTTAGGAGACATTGAAATCGCCATTAAGCTGGTGAAGACCGCGCTGCAAAGCCCA
GAACACCCACTGGACCAACACTATAGAAAACTACACTGTGCCTTGCATCCTCTAGACCAT
GAAAGTCACGAGTTCAAAGTGATTTCCCAGTACCTACAGTCTACTCATGCTCCCACACAC
AAGGACTACACCATGACCTTGCTGGACGTTTTTGAAGTGGAGAAGGAGGGTGAAAAAGAA
GCCTTCAGAGAGGACCTTCATAACAGGATGCTGCTGTGGCACGGCTCCAGGCTTAGTAAT
TGGGTGGGAATCCTGAGCCACGGCCTTCGAATTGCCCCCCCTGAAGCTCCCGTCACAGGT
TACATGTTTGGGAAAGGAATCTACTTTGCTGACATGTCTTCTAAGAGCGCCAATTACTGC
TTCGCTTCTCGCGTGAAGGACATTGGGCTGCTGCTTCTATCAGAGGTCGCTCTGGGTCAG
TGTAATGAGCTGCTAGAGGCCAATCCTGAGGCGGAAAGATTACTTCAGGGCAAACACAGC
ACCAAGGGACTGGGCAAGACGGCTCCCAGTCCCGCCTCCTTCATCACCCTGAATGGGAGT
ACGGTTCCTTTAGGACCAGCAAGTGACACAGGAATTCTGAATCCAGATGGTTATACTCTC
AACTACAATGAATTTATTGTCTATAGCCCCAACCAAGTCCGTATGCGGTACCTTCTGAAG
GTTCGATTTAATTTCCTGCAGCTGTGG
>Cow_PARP2
ATGGCAGCTCGGCGGCGGGGGACTGCGGGGGGCAGAGCGCGAGCTATTCTGAATGAAGCT
GAAAGAGTTAATAATGGCAAAACAGTTGCAGAAGACCCTCCTCCTGCAAAGAAAATTCGA
AAATGTCACAGGCTGAAAAAGGAGCCTGTGGCTGAAGGAAAGACAGATAGTGACAGGACT
GAAGACAAGCAAGATACACCAAGGAGAATGTGGACCAGCAAGAGGGTCCCTGAGTCTGTG
AAGACCTTGCTGTTAAAGGGCAAAGCTCCAGTGGACCCAGAGTGCACAACCAAGGTGGGG
AAGGCCCATGTGTACTTTGAAGGAAATGATGTCTATGATGTCATGCTAAATCAGACCAAT
CTCCAGTTCAACAACAACAAGTATTACCTGATTCAGCTGTTAGAAGATGATGCCCAGAGA
AACTTCAGTGTTTGGATGAGATGGGGCCGAGTTGGGAAGACAGGGCAGCACAGCTTGGTG
GCTTGTTCCGGGGACCTCAACAAGGCCAAGGAAATCTTTCAAAAGAAATTCCTTGACAAA
ACAAAAAATAATTGGGAGGATCGTGAGAAGTTTGAGAAGGTGCCTGGAAAATATGATATG
CTACAAATGGACTATGCCAGCAATACACAGAATGAAGAGGAAACAAAGAAAGAGGAATCT
CTTAAATCCCGCTTGAAACTAGAGTCACAGCTAGATCTTCGTGTACAGGAGCTGATAAAG
TTGATCTGTAATGTCCAGGCCATGGAAGAAATGATGGTAGAAATGAAATATGATACCAAG

AAAGCTCCACTTGGGAAGCTGACAGTAGCACAAATCAAGGCAGGTTACCAGTCTCTTAAG
AAGATTGAGGATTGTATTCGGGCTGGCCAGCATGGACGAGCTCTCATGGAAGCATGCAAT
GAATTCTACACCAGAATCCCACATGACTTTGGACTCCGTACCCCTCCATTAATCCGGACA
GAGAAAGAACTGTCAGATAAAGTACAACTACTGGAGGCTTTGGGAGACATTGAAATTGCA
ATTAAGCTGGTGAAGACAGAACTGCAAAGCCCGGAACACCCATTGGACCAACAGTATAGA
AAACTACGGTGTGCCTTGCACCCTTTACACCATGAGAGTTATGAGTTCAAAGTGATTTCC
CAGTACCTACAGTCTACCCATGCTCCCACACACAGTGACTATACCATGACCTTGCTGGAT
GTCTTTGAAGTTGAGAAGGAGGGTGAAAAAGAAGCCTTCAGAGAGGACCTTCATAACAGG
ATGCTACTCTGGCATGGCTCCAGGCTGAGTAACTGGGTAGGAATCCTAAGCCATGGGCTT
CGAATCGCCCCACCTGAGGCTCCCATCACAGGTTACATGTTCGGAAAAGGAATCTATTTT
GCTGACATGTCTTCCAAGAGTGCCAATTACTGCTTTGCCACTCGCCTAAAGGATACTGGG
CTGCTGCTCTTATCAGAGGTAGCTCTGGGTCAGTGTAATGAGCTGCTCGGGGCCAATCCA
GAGGCAGAGGGATTACTTCAAGGCAAACACAGCACCAAGGGGCTGGGCAAGATGGCTCCC
AGTCCTGCGTGCGCCATCACCTTGAATGGGAGTACAGTGCCATTAGGACCAGCAAGTGAC
ACAGGAATTCTGAATCCGGAGGGCTATACCCTCAACTACAATGAATTTATCGTCTATAAC
CCTAACCAGGTCCGTATGCGATACCTTCTAAAGGTTCAGTTTAATTTCCTGCAGCTGTGG
>Dolphin_PARP2
ATGGCAGCTCGGCGGCGGCGGGGGACAGGCGGCAGCCGGGCGCGAGCACTGAATGAAGCT
GAAAGAGTTAATAATGGCAAAACAGCTACAGAAGACCCTCCACCTGCAAAGAAAATTCGA
AAATGCCAGAAGATGAAAAAGGAGCCTGTGGCTGGAGGAAAGGCTGATAATGACAGGACG
GAAAACAAGCAAGGTCAGCTCCTGGACATACATAGGCCAGGGAGCTCCTGGGCCAGCGGG
GTCTCTGAGTCTGTAAAGACCTTGCTGTTAAAGGGCAAAGCTCCAGTGGACCCAGAGTGC
ACAGCCAAGGTGGGGAAGGCCCATGTATACTGTGAAGGAAATGATATCTATGATGTCATG
CTAAATCAGACCAATCTCCAGTTCAACAACAACAAGTATTATCTGATCCAGCTGTTAGAA
GATGATGCACAGAGGAACTTCTGTGTTTGGATGAGATGGGGCCGAGTTGGAAAAATGGGG
CAGCACAGCTTGGTGGCTTGTTCTGGGGACCTCAACAAGGCCAAGGAAATCTTTCAAAAG
AAATTCCTTGACAAAACAAAAAATAATTGGGAGGATCGTGAGAAGTTTGAGAAGGTGCCT
GGAAAATATGATATGCTACAAATGGACTATACCACCAATACTCAGAGTGAAGAGGAAACA
AATAAAGGTGAATCTCTCAAATCCCAGTTGAAACCAGAGTCACAGCTAGATCTTCGTGTA
CAGGAGCTGATAAAGTTGATCTGTAATGTCCAGGCCATGGAAGAGATGATGGTAGAAATG
AAATATGATACCAAGAAAGCCCCACTTGGGAAGCTGACAGTGGCACAAATCAAGGCAGGT
TACCAGTCTCTTAAGAAGATTGAGGATTGTATTCGGGCTGGCCAGCATGGACGAGCTCTC
ATGGAAGCATGCAATGAATTCTACACCAGAATACCACATGACTTTGGCCGTACCCCTCCA
TTAATCCGGACAGAGAAAGAGTTGTCAGACAAAGTACAGCTACTAGAGGCTTTGGGAGAC
ATTGAAATTGCCATTAAACTGGTGAGGACAGAACTGCAGAGCCCAGAACACCCACTGGAC
CAACACTATAGAAAACTACATTGTGCTTTGCACCCTTTAGACCATGAGAGTTATGAGTTC
AAAGTGATTTCCCAGTACCTGCAGTCTACGCATGCTCCCACACACAGTGACTATACCATG
ACCTTGCTGGATGTATTTGAAGTAGAGAAGGAGGGTGAGAAAGAAGCCTTCAGAGAGGAC
CTTCATAACAGGATGCTGCTATGGCATGGTTCCAGGCTGAGTAACTGGGTGGGAATCCTG
AGCCACGGGCTTCGAATCGCCCCACCTGAGGCTCCCATCACAGGTTACATGTTTGGAAAA
GGAATCTACTTTGCTGACATGTCTTCCAAGAGTGCCAATTACTGCTTTGCCACTCGCCTA
AAGGATACTGGACTGCTGCTCCTGTCAGAGGTAGCTCTAGGTCAGTGTAATGAGCTACTA
GGGGCCAATCCAGAGGCAGAAGGATTACTTCAGGGCAAACACAGCACCAAGGGGCTAGGC
AAGATGGCTCCCAGTCCTACGTGCGCCATCACCTTGAATGGGAGTACAGTGCCCTTAGGA

CCAGCAAGTGACACAGGAATTCTGAATCCAGAGGGTTATACCCTCAACTACAACGAATTT
ATTGTCTATAACCCCAACCAGGTCCATATGCGATACCTTCTAAAGGTTCGATTTAATTTC
CTGCAGCTGTGG
>Elephant_PARP2
ATGGCGGCTCGGCGGCGGGGGACGGTCCGCGGCCGCGCGAGCATTAAATGAAACTAAG
AGAGTTAATAATGGCAACACAGCTACAGAAGACTCCCCTCCTGCAAAGAAAACTCGAAGA
TGCCATAATCAGGGGGTGAAAAAGGAACCTGTGGCTGAAGGCAAGGCTGAAAATTATGAC
AAGACAGAAGACAAGCAAGAATCTGTGAAGACTTTACTGTTAAAAGGCAAAGCTCCTGTA
GACCCAGAGTGCACAGTCAAAGTGGGGAAGGCCCATGTATATTGTGAAGGGAATGATGTC
TACGATGTTATGTTGAATCAGACCAATCTCCAGTTCAACAACAACAAGTACTATCTGATT
CAGTTGTTAGAAGATGATGCCCAGAGAAACTTCAGTGTTTGGATGAGATGGGGCCGAGTT
GGGAAAACAGGGCAGCACAGCTTGGTGGCTTGTTCAGGGGACCTCACCAAGGCCAAGGAA
ATCTTTCAGAAAAAATTCCTTGACAAAACGAAAAATAATTGGGAGGATCGTGAAAAGTTT
GAGAAGGTACCTGGAAAATATGATATGCTACAGATGGACTACGCTACCAACACACAGGGT
GAAGAGGAAACAAAAGAAGAGGAATCTCTTAAATCCCCCTTGAAACCAGAGTCGAAGCTA
GATCTTCGGGTACAAGAGCTGATAAAGCTGATTTGTAATGTCCAGGCCATGGAAGAGATG
ATGGTTGAGATGAAGTATGACACCAAGAAAGCCCCACTTGGAAAGCTGACAGTAGCACAA
ATCAAGGCAGGTTACCAGTCTCTTAAGAAGATTGAGGATTGTATTCGGGCTGGCCGGCTC
GGACGAGCTCTCACGGAAGCATGCAACGAATTCTATACCAGAATCCCACATGACTTTGGA
CTCCGTACCCCTCCACTAATCCGAACAGAGAAAGAACTGTCGGAAAAAGTCCAACTATTA
GAGGCATTGGGAGACATTGAAATTGCCATTAAGCTGGTGAAAACAGAGCTGCAAAGCCCA
GAACACCCATTGGACCAACACTATAGAAACCTACATTGTGCCTTGCGCCCTCTAGACCAT
GCGAGTTATGAGTTCAAGGTGATTTCCCAGTATCTACAGTCTACCCATGCTCCCACACAC
ACTGACTACACCATGACCCTGCTGGATGTTTTTGAAGTAGAGAAGGAGGGTGAGAAAGCA
GCCTTCAGGGAGGACCTTCCTAACAGGATGCTGCTCTGGCATGGCTCCAGGCTGAGTAAC
TGGGTAGGAATCCTGAGCCATGGGCTTCGAATTGCCCCAGCTGAGGCTCCCATCACAGGT
TACATGTTTGGAAAAGGAATCTACTTTGCCGACATGTCTTCCAAGAGTGCCAATTACTGC
TTTGCCTCCCGCCTAAAGGACACAGGACTGCTGCTCTTATCAGAGGTAGCTCTGGGTCAG
TGTAATGAGCTACTGGGGGCCAATCCTGAGGCAGAAGGATTACTTCAGGGCAAACACAGC
ACCAAGGGGCTGGGCAAGATGGCTCCCAGTCCTTCACACTTCATCACCCTGAATGGAAGT
ACAGTGCCCTTAGGACCAGCTGGTGACACCGGAGTTCTGAATCCAGAGGGTTATACCCTC
AACTACAACGAATTCATCGTCTATAACCCCAACCAGGTCCGTATGCGATACCTTCTGAAG
GTTCAATTTAATTTCCTGCAGCTCTGG
>tropical_clawed_frog_PARP2
ATGTCAGGGCGCAGCGGAAAGGTCCAAGGGCGGGCGAGAAAGACCCGGGTTTCCCAGAAG
AGAAAAGCAGAGTCTGATCCAGAGCCAGACTTTGTGGTGAAAATAGAACCGGATGAGGAG
AATAATGTTGCTGCAGATGTTTGTGATGGAGAGGCAAGGACCAGTATGAAAACAGAGCTA
GACTCCCGCTGGGAATGGGAAGGAGATGGTGGCATTTGGATTCCTTTTTCCCCTGAGCTC
AGCACTCAGATTAATTTGGCATTCAGTTCAGGGAAACGATTTGTGACAGTCTCACCAGCT
GCAGGGGTGTCGATAAAGGTAGATATGCAGAAAATGGTTCAGAAAAACACACAGACTGGG
TACCAGAGATTTGTCCGACTGGCAGTTCAAGATTGTGACAAATATTTTGTGTGGCAGTGG
TGGTCTGATGAGGGAGACTGGATTTCCTATGATGCTTGCACGTGTGTGGCTCTGGAGGCT
GGATTTCAATCAGAAGCTAAGATGGTGTCTGTAAGCTTGGGGGGTCGCCCATACATCATC
GATTTAGAAACAATGGTGCAAAAAAACAGTCAAACTGAACATGAAAGACAGATTCAACGC

TGTTTATCTGGTCCTAGTAGTGCTCCTCAGCAAAAGAGCACCATTAGTGCACCTGCAAAG
AGATCCCGTGGAAGCAAGAGTGTTATAAATGTAGAAGATGAATCAGAGGAGAATAAAGAA
CAGGTTAGGACTCTGGTAATGAAAGGCAAAGCTCCAGTAGATCCAGAATGCTCCACGAAG
CTGGGGAGGGCTCATGTGTTCTGTGAGGGAGATGATGTGTATGATGTCATGTTAAACCAG
ACCAACCTTCAGTTCAATAACAATAAGTACTACCTAATCCAGCTGCTGGAAGATGATGGT
GTGAGAAATTTCTCTGTTTGGATGCGTTGGGGAAGAGTTGGCAAAGTTGGGCAGAACTCT
CTGGTATGTTGTGGAGGAGACCTACAAAAAGCAAAAGATATTTTTCAGAAAAAGTTTCTT
GATAAAACGAAGAACTTATGGACTGAGCGAGCACAGTTTGAAAAATGCCCCGGTAAATAT
GATATGCTGCAGATGGACTATAATGCAACTGAGCAGGAAGAAGAGAGAGCAGTGAAGGAA
GAGAGCCTGGCTGACCTTCCCAAACTAGAATGCCAGTTAGACCTCAGTATCCAAGAGTTG
ATTCAGCTGATTTGCAATTTAAAAGCCATGGAGGAGACTGTACTTGAGATGAAATTTGAT
ACAAAGAAAGCTCCCCTAGGGAAACTGACAGTCGATCAGATACGTGCTGGCTACTGCTCC
CTTCAACGTATTGAAAACTGTATTAAGAAGCAAAAGTTTGGCCGGGATCTTCTTGAGGCA
TGCAATGAGTTTTACACACGCATTCCCCATGACTTTGGGCTACGAACACCTCCTCTTATT
AGGACATTAGAAGACCTGACAGTAAAAGTTCGGCTATTGGAAGCGCTGGGTGATATTCAG
ATTGCAGTGAAGTTAGCAAGCATGGATCTCAACTCTCTGGAGCACCCTATCGACCGACAG
TATCGGCAGTTAAATTGTTCTATTCAGCCCCTAGACAAAACATCCAGTACTTTCCAGCTT
ATTGATGGTTATCTGCAGAGCACCCATGCTCCAACGCACAATGATTACACCATGACACTG
CTGCAGGTCTTTGAGTTACAGCGAATGGGAGAAGAATGCAGCTTTAGAGCAGACCTCCCT
AACAGGATGCTGCTGTGGCATGGCTCTCGGCTAACTAACTGGGTCGGAATACTTAGCCAG
GGACTAAGAGTGGCCCCTCCAGAGGCTCCAGTAACTGGGTACATGTTTGGAAAAGGGATA
TATTTTGCTGATGTCTCATCAAAAAGTGCAAATTACTGCTTCTCATCCCGGGACAAAAAT
GTTGGAGTTCTCCTACTGTCTGAGGTGGCTCTCGGGGAAAGTAATGAGCTTCTTGCTGCA
GATTATGATGCCCAAAAGAAGCTGAAGTCAAAGCACAGCACAAAAGGATTGGGAAGGAGC
ATCCCAGACCCCAAGAATAGCATCACCCATGACGGAGCTGTGGTTCCATTGGGTCCACTA
ATAGACACAGGCATGACGAATGACAGCGACTACACGCTAAATTACAACGAGTACATTGTT
TATGACCCTCATCAGGTTCGCATGAAGTACCTGCTACAGGTCCACTTTAACTATAGTTCC
TTATGG
>Fugu_PARP2
ATGAGGCGCCAAAGAACCGCAAGAAATAAAAATCAAGCTGTCGAAGAAAGTCCGAGTTCC
GACACAGTGTGGCAGTGGCAGGGAGACGAAGGACAGTGGGAACCATACTCGCCCTCTGTC
TGCACCTTACTGAACTCAGCAGTTGCGTCTGGGAACTCTGTCCATCTCTCTTTGGACTCT
GGGCAATCCTATGAAGTTGACCTGAAGAAAATGCTTCAGATTAACCCTGTGACCAAGTAC
AAGAGGAAGATTCGTTGTCAGACAGTAAAACCAGAAACCATGGCCAATGCTATTGAGGAA
AGCCAACAGAGCAGTACTCGAGTTCAAATTAAAGAGGAAGAGGAGGAAGAAGATACAAAC
AAGCAGCCTGCTACTAAGAGGATGAGAGGACAAACTAACTATGAAGGAAGTCCCAAAGAG
AATCCAAAAATTGAAGAGGTGGTAAAGACGGTGGTCCTGAAAGGAAAAGCTCCAGTGGAC
TCTGAGTGTAAAGCAAAGCTCAGAAAGGCTCATGTTTACACTGAAGGAAATAATGTTTAT
GATGTCATGTTAAATCAGACAAATCTTCAGTTTAATAATAACAAATACTACCTGATGCAG
CTGTTGGAGGATGATAAATCCAAATTTTACAGTGTGTGGTTTAGGTGGGGAAGAGTGGGA
AAAGTGGGTCAAAACAATCTGATCGAATGTGGTGCAGACCTGAACCAAGCCAAAGATATC
TTCAAGAAGAAGTTCTTTGACAAGACTAAAAATGAGTGGGAGCACAGAGAAAACTTTATG
AAAGTAGCAGGAAAATATGACATTGTGCTTATGGACTACAGCGCAGATGAAAAGGAGAAG
AAAAACGCCATGGTAGATACCAAAAAACAGGCCTCCATGCTGGATGTGAAAATCCAGGAA

CTATTGGAGCTAATCTGTGATCTTAAAGCCATGGAGGAATGTGTGCTGGAGATGAAGTTT
GATACCCAAAAAGCTCCACTTGGTAAATTGACCTCAGAACAGATCCGTGCAGGCTATGTG
GCTCTAAGGAAAATTGAGGACTGTTTGAAAAAGAAGAGCAGCCGCAATGAGCTGTTGAAA
GCATGCAACCAGTTCTACACTCGCATCCCTCACAACTTTGGGTTGAAAACTCCTCCAATC
ATTCATACAGAACAAGAGCTAAAGGAAAAGATTGCACTTTTAGAGGCACTGAGTGATATT
CAGATTGCTGTGAAAATGGTTAAATCTAATGAAGACGGTGATGAAAATCCACTAGACAGG
CAGTATCGCTCCCTCCATTGCAGCTTGCAGCCTCTGGACTCCAGCTCCCATGAGTACAGG
GTAATTGAAAAGTATCTCCAGTCTACTCATGCTCCCACCCATTCAGACTACACCATGACC
GTCATTGACATTTTCACCATTGACAGAGAAGGAGAGAGTAACAATTTCCTGTCACACTTT
CACAACAGGACTTTGTTGTGGCATGGTTCCCGTCTTTCTAACTGGGTTAGCATCCTGAGT
CAGGGGCTCAGAGTAGCTCCACCTGAGGCTCCAGTCACTGGATACATGTTTGGAAAGGGT
ATCTACTTTGCTGACATGTCATCAAAAAGTGCTAACTACTGTTTCGCCAATCAGAGTAAC
CACATTGGACTGCTACTACTGTGTGAGGTACGTTCACTTATTTTCTTGATGGTTCTGTGT
TGTATCTTCTTTAATTTAAGATCTGCATTCTTTGCTGTTCGATTGAAAAATACTCTGGCA
CTACGA
>Gecko_PARP2
ATGGGTCGGAAACGGAAGGCCTCCAAAGCCCTGGATGGGGAACCCAGCGATGGAGGGGCG
GAGTTGGAGCTACGCTGGGAGTGGGAGGCCTCCGACGGCAGGTGGCAGCAGTTCCCACCG
GAGCAGAGTGAGGCCCTCAGCCAGGCGGCCAGGGCAGGAAAGCTTTCTGTGGACCTCGGC
GAGTCCTCCGTCGACCTATGTGGGATGGTGCAGCGGAAGAAGCAGACGGGGCAGGAGACA
CACGTGGCTGCAGCAGTCCGGGCACAGGACTCTTATTTTGTGTGGCAGTGGCAAGGGGAC
CAAGAAGGGGATTGGCTTCCGTACCCGGCCAATACCTGCCTGGCCTTGCAGGCAGCCAAG
AACGGCCACGGGGAGCCCACGGTGGAAATGATGGCTGGCCGGACCCGCTACAAGCTGGAC
ACGACCCGCATGGCGCAGATCAACACCCGTACCGGATTTGAACGCGAGATGGAATGCCGG
CCGTCTGATGCCGCTGTGCCCGAGGAAGGAAGATCTCAAGGCGGCGACAGCGTCTCCGAT
CCCAGAGGCCCTGCGGCCACAAAGAAGGCCCGGAACGGAGCAGAGGCGGCAGCATCTCCG
GGAGAGGGGGATGGCAAGGGAGAGGCTGTGAAGACGTTGATCGTGAAGGGGAAAGCTCCT
GTGGACCCAGAATGCACTGCAAAGATAGACAAAGCTCACGTTTATTGTGAGGGAGAGGAC
GTTTACGATGTGATGTTAAATCAGACAAATCTTCAGTTCAACAACAACAAGTACTACCTC
ATCCAGTTGTTGGAGGACGATGGAGCACGAAGCTACAGTGTCTGGATGCGCTGGGGGCGC
GTGGGTAAACCGGGTCAGCACACCCTCGTGAGTTGCGCTGGAGACTTGAGTAAGGCCAAA
GCCGTTTTCACCAAGAAATTCTTGGATAAAACTAAGAATGAGTGGGCCAAGCGGGGTAAC
TTCCAGAAGGTTCCGGGCAAATACGACCTTCTCCACATGGACTATGAAGCCCATGATGCA
GGTGAAGAGGAAGCCACTTCGCAGAAGACAATCTCGTCTCCCAAGCCAGCATCACAGCTG
GATCCACGGGTGCAGGCCTTGGTGGAACTGATCTGCAGCATCCGCACCATGGAAGAGATG
GTTGTGGAGATGAAGTATGACACCAAGAAGGCCCCTCTAGGAAAGCTGACAGCTGAACAG
ATCCGGGCCGGTTACCAGTCGCTGCAAAAGGTGGAGGCCTGTCTGAAGAGGAAGCAGACT
GGCCGCGCCCTGCTGGAGGCCTGCAACGAGTTCTACACTCGGATCCCCCACGATTTTGGG
CTGAAGACTCCCCCATTGATAAAGTCAGAAGAGGAGTTGCAAGAGAAAGCGCAGCTCTTA
GAGGCCCTGGGCGAGATCCGCATCGCCATCAAGCTGGTGCAGTCAGAGCAACTGGCCCTG
GAGCACCCGCTGGACCGGAGCTACCGCGGACTGAGCTGCCAACTGCAGCCCTTGGAGAGG
GATACCCCGGACTTCCAGGTATTAGAGCGATACCTGCTGTCCACCCATGCCCCGACCCAC
ATGGATTACACAATGACCCTCCTGGAGGCCTTCGTGCTGGACAAGGGCAGTTCCGCCTTC
CGCTCTGACCTGCCCAACCGGATGCTGCTGTGGCACGGATCCCGCCTGGGAAACTGGGCC

GGGATTTTGAGCCAGGGGTTGCGAGTGGCACCACCCGAGGCTCCCGTCACAGGCTACATG
TTTGGGAAAGGGATCTATTTTGCTGACATGTCCTCTAAGAGCGCCAATTACTGTTTCGCC
ACCCGTGAGAGAGACATTGGCCTACTCTTGCTTTCAGAGGTGGCTCTGGGCGAATGCAAC
GAACTGCTGGAGGCAAATCCCGAAGCCGTGAATTTGCCGCCCCACAAGCACAGCACCAAA
GGGCTCGGCAAGCTGGCCCCGGCCAACAGCACCACTCTGCATGAGGCTGTTGTTCCTATG
GGTCCAGTCGGGGAGACAGGTGTGGTAAACCCCCACGGCTACACCCTGAACTACAATGAA
TTCATCGTCTATGACCCGCAGCAGGTGCGGATGAGGTATCTCCTCAAGGTGCACTTCAAT
TTTACTCAGCTCTGG
>Gharial_PARP2
ATGTCCCTTCCGTCTTGTGCCTGTCCAGGGATGGGCCGCAAGCGGGCGGCACCGGAGGCG
GGTGACCGGGAGCTGGAGCTGCGCTGGGAGTGGCAGGATGCTGCGGGCACTTGGCATTGG
TACTCCCCGGAGCAGAGCAGGGTGCTCACGCAGGCGGCCAGGGCCAGGAAGCCCAGCGTG
GATGTTGGGGCCTGCGTGGACCTGAGAAGGATGGTGCAACAGGACACACAGACGGGGCAG
GACAGACCCGTGGCAGCTGCTGTCCGGGACCAGGACTCGTACTTCATCTGGCAGTGGCAG
GGCGATGAGGAGGGGGACTGGCTCCCCTACCCTGCTGATACCTGCTTAGCTCTGGAGCGG
GTGCGACGTGGGGAGGGGGAGCCAACCTTGCAGATGACTGTTGGCCGGACCCGCTACGCG
CTGGACACAGCCCAGATGACCCAGACCAACTTGAGAACCAGGTATCAACGCAAGATGGAC
CGCAGGGAGTCAGATGCAGTGGTTGATAGTAAGGAATCTCAAGCGAGCTACACTCCCAGC
TCCACAGCCCTCCAAGAGGCTCAAGCACTGAAGAGGTCCCGCGGTGGGGGGTCCATCCCA
GACCCAGTGGCTAAAGAAGTTGATGGGGGAGAGGTCAAAGAGGCCGTTAAGACCCTAGTA
GTGAAGGGGAAAGCACCAGTGGATCCCGAGTGCTCTGCTAAGCTTGGGAAGGCTCATGTG
TACTGTGAGGGGGACGACGTCTATGATGTGATGCTGAACCAGACGAATCTCCAGTTCAAC
AACAACAAGTTCTACATCATCCAGCTCCTGGAGGATGATGGGCAGCGGAGCTACAGTGTC
TGGATGCGGTGGGGGCGCGTGGGGCGTCCAGGCCAGCACACACTGGTATCCTGCGCTGGG
GATCTTGACAAAGCCAAGGAAGTCTTCACCAAGAAGTTTCTGGATAAGACGAAGAACAGC
TGGCCCAGTCGGGGCAGCTTCCAGAAGGTGCCAGGCAAATACGACCTGCTGCATATGGAC
TACCAGGCCCACGGTCCAGATAAGGAAAGCCGCCCTGAGAAGGTAATGTCCCAACCCAAA
CCAGCCTCCCGACTTGACCCCCGCGTGCAGGCACTGGTGGAGCTCATCTGTAACATCCAG
ACCATGGAGGAGATGGTGATTGAGATGAAGTATGACACTAGGAAGGCCCCATTGGGGAAG
TTGACAGCAGAGCAGATCCGGGCTGGGTACCGCTCGCTGCAGAAGGTGGAGGCGTGTCTG
CAAGAGGGAAGCGCTGGCCGGGCCCTGCTGGACGCCTGCAACGAATTCTACACCCGAATC
CCGCACGACTTTGGGCTCCGAACGCCCCCAATGATCCGGACGAAGCAGGAGCTGCAGGAA
AAGATGCAGCTGCTGGAGGCCCTGGGCGAGATTGAAATTGCTATCAAACTAGTGTGCTCA
GAGAGGCAGGACCATGAGCACCCACTGGACCAGCACTACCACCAACTGGGCTGCGAGTTG
TGTGCCCTGGACAGGGACACCCATGATTTCCAGGTGCTGGAGCAGTACCTGCTGACCACC
CATGCACCCACCCACCGCGACTACTCCATGGAGCTGCTGGAGGCCTTTGCCCTGCGCCGC
CCGTCTGAGGAGACGACTTTCCGCACTGACCTTCCCAACAGGGTGTTGCTGTGGCATGGC
TCCCGGCTGGGCAACTGGATGGGCATCCTGAGCCAGGGGCTGCGAATCGCACCCCCAGAG
GCTCCTATGACTGGCTACATGTTCGGGAGAGGCATCTACTTTGCTGACATGTCCTCCAAG
AGTGCCAACTACTGCTTTGCTTCCCGTCAGAAGGATGTGGGGCTCCTGCTGCTGTCAGAG
GTGGCCTTGGGTGAGTGCAATGAGCTGTTGGAGGCTAATGCTGATGCCCAGAAGCTCTTG
AACGGGAAACACAGCACCAAGGGGATGGGGAAGATGGCTCCTGCTAACCTCACCAAGCTG
GATGGCACCGTGGTCCCTCTAGGACCCGCTGTGGACACCGGAGTGGTGAACCCCCATGGC
TACACCCTGAACTACAATGAATTCATCATCTATGACCCGTGCCAGGTGCGCATGCGCTAC

CTGCTCAAGGTGCGCTTCAACTTCACGCAGCTGTGG
>Anole_lizard_PARP2
ATGGGGAGGAAACGAAAGGCCCCGGAGTCTCAGAACGGAGAGCCCAGTTCCTCTGGGGTT
GATCTAGAACTGCGCTGGGAATGGGAGAGCTGTGATAACCGATGGCAGCAGTTTCCACCT
GGAGAGAACGAAGCTTTGAGCCAAGCCGCCAGGGCTGGGAAGCTGTCCTCAGATATTGGG
GAGTCCTGTATTGATTTGCGCAGGATGGTGCAGCAGGATAAACAGACCGGGGAGGAAACC
CGTGTGGCTGCGGCTGTCCGGGATCAGGATTCCTACTTTGTCTGGCAATGGCAAGGAGAC
CCAGAAGGCACCTGGTTTGCGTACCCTGCGGACACTTGCCTGGCTTTGGAGGCTGCCAGG
AAGAGTCATGGGGAGCCCACTGTCAAGGTGACAGTCGGCAGGACACACTACAAGCTTGAC
ACGACTCGCATGGTACAGATCAACACCCGGACTGCGTTTGAGCGCCAGATGGAACGTAGA
GAGTCAGATGTCACCGAGGTTCAGGATGAAGGGTCCCAGCTGAGCAACAGTGCTGTGAAT
ACTACTACCACCCCCGTTCCTGCCAAGAAGGCTCGGAATAGAGGAGGCGCTTCAGCATCT
CAGGGGGAGGGGGATAGCACAGAAGCTGTAAAGACGCTGATAGTGAAGGGAAAAGCCCCT
GTGGATCCAGAATGCACTGCTAAGCTGGGGAAGGCTCACGTTTATTGTGAAGGAGATGAT
GTTTATGATGTGATGTTAAATCAGACGAATGTCCAGTTCAACAATAACAAGTACTACCTC
ATCCAGCTGTTGGAAGATGATGCCTCAAGGAACTACAGTGTTTGGATGCGCTGGGGACGA
GTGGGTAAACCGGGTCAGCATTCACTTGTGAGCTTTGCTGGAGCCTTAGCTAAAGCTAAA
GACCTCTTCACCAAAAAATTCCTGGACAAAACCAAGAACGAGTGGACCAAGCGGCAGAAC
TTCACAAAGGTTCCTGGAAAATATGATCTCCTCCACTTGGACTACGAAGCCAATGACACA
AGTGAGGAAGAAGCTGCCTCTGAGAAAACCAATTCGTGTCCGACACCAGTGTCACGACTG
GAGCCACGAGTTCAGGCCTTGGTGCAGCTGATCTGTAATATCCGCACCATGGAGGAGATG
GTGATGGAGATGAAATACGATACTAAGAAGGCCCCTTTAGGAAAGCTGACAGCTGAGCAG
ATCCGGGCAGGATACCAGTCCCTGCAGAAAGTGGAGGCGTGCCTTAAACGGAAGCAAACC
GGTCGGACTTTGCTGGAGGCTTGCAATGAATTCTACACAAGGATTCCCCACGATTTTGGG
TTGAAAACTCCTCCATTGATTCACACAGAAAAGGAACTGAAAGAGAAGGTGCAGCTGCTG
GAGGCCCTTAGTGAGATTCGGATCGGTATCAAAGCGGTGCGGTCAGAGCAGCTGGACCAG
GAGCACCCGCTTGACCGCAATTACCGTGGATTAAATTGTGACCTCCAGCCCTTGGAGAAG
GAGAGTCCTGACTTCCAGGTCCTTGAGCGCTACCTGTCCTCCACGCATGCCCCGACCCAC
CAGGACTACACCATGACCCTACTGGAGGTCTTTGTCCTGAAGAAGGAGAATGCTGATTCC
GACTCAGTCTTCTGTTCTGACCTTCCCAACCGGATGCTACTTTGGCACGGATCCCGCTTG
GGCAACTGGGCAGGGATCCTGAGCCAGGGGTTGCGGGTGGCACCCCCAGAGGCGCCTGTC
ACTGGCTACATGTTTGGAAAAGGGATCTATTTTGCAGACATGTCTTCTAAGAGTGCCAAT
TACTGCTTTGCAACCCGGGAAAAAGACATTGGCCTGCTGCTGCTTTCAGAGGTGGCTCTG
GGCGAATGCAATGAATTGCTAGAAGCAAATCACGAAGCAGAGAAATTGCTGGCTGGCAAA
CATAGTACTAAAGGCCTCGGAAAGTTAGCTCCATCCCCAGCCAACAGTGTCACACTGCAT
GGAGCTGTGGTTCCCTTGGGGCCTGCGATAGACACTGGAGTAACGAACCCTCACGGCTAT
ACCCTGAACTACAATGAGTTCATTGTCTATGATCCGCGACAGGTGCGCATGAAGTACCTC
CTCAAGGTGCGCTTCAATTTTACCCAGCTCTGG
>green_sea_turtle_PARP2
ATGACCCAGACCAACGTCAGAACCGGATACCAGCGCCGGATGGAACGGAGGGAGTCAGGT
ACGAACTCGTCCCCACAACGGCCCTCAGCACCAAAGAGACCCCGGGATGGTGGGGCCAGC
CCCAACCCCGGAGCCGGGGGAGAGAGCACAGAGGTCATCAAGACCCTGATTGTGAAGGGG
AAGGCGCCGGTGGATCCCGAGTGCTTGGCTAAACTGGGGAAGGCCCATGTTTACTGCGAA
GGGGACGATGTCTATGACGTGATGCTGAACCAGACCAACCTCCAGTTCAACAACAACAAG

TTCTACGTCCTACAGCTGCTGGAGAACGATGGGTCGCGGAGCTACAGTGTCTGGATGCGC
TGGGGGCGTGTGGGGCGGCCAGGCCAGCACATGCTGGTGTCCTGCGCTGGGGACCTCGTC
CAGGCCAAGGAGATCTTCACTAAGAAGTTCCTCGACAAGACCAAAAACCACTGGGCCGAG
CGGGGCAACTTCCAGAAAGTGATGGGCAAGTACGACCTGCTGCACATGGACAGCCAACCC
CCCGTCCAGGCCAAGGAGATCTTCACTAAGAAGTAAGCCACAGAGCTGAGCTGCGCGGGG
GCCCCCCGGCCCCAGCTGGCCTCACAGCTAGACCCCCGAGTGCAGGCGCTGCTGGGGCTG
GTCTGTGACCTGCAGGCCATGGAGGAGATGGTGCTGGAGATGAAGTATGACACCAAGAAG
GCCCCTCTTGGGAAGCTGACGGTGGAGCAGATCCGTGCCGGGTTCCAGTCACTGCAGAAG
GTGGAGGCGGTTCTGCGAGCCGGGGACACCGGGCGGGCCCTGCTGGAGGCCTGCAACGAG
TTCTACACCCGCGTGCCCCATGACTTTGGGCTCCGGACCCCCCCATTGATCCGGACACGG
CAGGAGCTTCAGGAGAAGGTGCAGCTGCTGGAGGCCCTGGGTGAGATCCAGATTGCCATC
AGGCTGGCACACTTGGAGCTGCACGGCCAGGAGCATCCCCTGGACCAAAGCTACCGCAAG
CTGGGCTGCGAGCTCCGCCCCCTGGACCGGGACTCCGCCCACTTCCAGGTGCTGGAGCGG
TACCTGCTCTCCACCCATGCGCCCACCCACCGTGACTACTCCATGGAGCTGCTGGAGGCC
TTCGCCCTGCGCCGGGCTGGCGAGCCCCCCTTCTGCACCAGCCTGCCCAACCGGTTTGGG
GGCTGGGAATGGATGTTGCTGGGGCACGGCTCCAGGCTGGGCAACTGGGTGGGGATCCTG
AGCCAGGGGCTGAGAGTGGCGCCCCCTGAGGCCCCTGTCACCGGCTACATGTTCGGGAAG
GGCATCTACTTCGCAGACATGTCATCCAAGAGCGCCAACTACTGCTTCGCCTCACGCCAG
CGCGACGTGGGCCTGCTGCTGCTGTGCGAGGTGGCCCTGGGCGAGTGCCAGGAGCTGCTG
GAGGCAAATGCTGAGGCCAGGAAGCTGCCACCTGGGAAGCACAGTACCAAGGGGCTGGGG
AAGCTAGCGCCCGCCCCCGCCAACAGCGTCATGCTGGATGGGGCTGCAGTGCCCCTGGGC
CCAGCGGTGGAGACGGGCGTGACGAACCCCCATGGCTACACCCTCAACTACAACGAGTTT
GTCGTCTACGACCCGGGCCAGGTGCGGATGCGCTACCTGCTCCAAGTGCGCTTCAACTTT
GTGCAGCTGTGG
>Horse_PARP2
ATGCTGAATCAGACCAATCTTCAGTTCAACAACAACAAGTACTATGTGATTCAACTGTTA
GAAGATGATGCCCAGAGGAACTTCAGTGTTTGGATGAGATGGGGCCGAGTTGGGAAAGTG
GGGCAGCACAGCTTGGTGGCTTGTTCAGGGGACCTCAACAAGGCCAAGGAAATCTTTCAG
AAGAAATTCCTTGACAAAACAAAAAACAATTGGGAGGGTCGTGAGAAGTTTGAGAAGGTG
CCTGGCAAATATGATATGCTGCAGATGGACTATTCCACCACTACTCAGAGTGAAGAGGAA
ACTAAAAAAGAGGAATCTCTTAAATCCTCCTTGAAACCAGAGTCACAGCTAGATCTTCGT
GTCCAGGAGCTGATAAAGTTGATCTGTAATGTCCAGGCCATGGAAGAGATGATGGTAGAA
ATGAAATATGATACCAAGAAAGCCCCACTTGGAAAGCTGACAGTGGCACAAATCAAGGCA
GGTTACCAGTCGCTTAAGAAGATTGAGGATTGTATTCGAGCTGGCCAGCATGGACGAGCT
CTCACAGAAGCATGCAATGAATTCTATACTAGGATCCCACATGACTTTGGACTCCGTACC
CCTCCTTTAATCCGGACAGAGAAAGAACTATCAGAAAAAGTACAGCTGCTAGAGGCTTTG
GGAGACATTGAAATTGCCATGAAGCTGGTGAAGACAGAGCTGCAAAGCCCAGAACACCCA
TTGGACCAACACTATAGAAGATTACATTGTGCCTTGCGCCCTCTAGACCGTGACAGTTAT
GAGTTCAAAGTGATTTCCCAGTACCTACAGTCTACTCATGCTCCCACACACAGTGACTAT
ACCATGACCTTGCTGGATGTTTTTGAAGTAGAGAAGGAGGGTGAGAAAGAAGCCTTCAGA
GAGGACCTTCATAACAGGATGCTGCTATGGCATGGTTCCAGGTTGAGTAACTGGGTGGGA
ATCCTGAGCCATGGGCTTCGAATTGCCCCACCTGAAGCTCCCATCACAGGTTACATGTTT
GGAAAAGGAATCTACTTTGCTGACATGTCTTCCAAGAGTGCCAATTACTGCTTTGCCTCT
CACCTAAAGGATACTGGGCTGCTGCTCTTATCAGAGGTCGCTCTAGGTCAGTGTAATGAG

CTACTAGAGGCCAATCCTGAGGCCGAAGGGTTACTTCAAGGAAAACACAGCACCAAAGGA
TTGGGCAAGATGGCTCCCAGTCCTGCCTGCTTCACCACCCTGAATGGAAGTACAGTGCCC
TTAGGACCAGCAAGTGAAACAGGAATTCTGAATCCAGAGGGTTATACCCTCAATTACAAT
GAATTTATTGTCTACAGCCCCAACCAGGTCCGTATGCGATACCTTCTAAAGATTCGATTT
AATTTCTTGCAGTTGTGG
>Human_PARP2
ATGGCGGCGCGGCGGCGACGGAGCACCGGCGGCGGCAGGGCGAGAGCATTAAATGAAAGC
AAAAGAGTTAATAATGGCAACACGGCTCCAGAAGACTCTTCCCCTGCCAAGAAAACTCGT
AGATGCCAGAGACAGGAGTCGAAAAAGATGCCTGTGGCTGGAGGAAAAGCTAATAAGGAC
AGGACAGAAGACAAGCAAGATGAATCTGTGAAGGCCTTGCTGTTAAAGGGCAAAGCTCCT
GTGGACCCAGAGTGTACAGCCAAGGTGGGGAAGGCTCATGTGTATTGTGAAGGAAATGAT
GTCTATGATGTCATGCTAAATCAGACCAATCTCCAGTTCAACAACAACAAGTACTATCTG
ATTCAGCTATTAGAAGATGATGCCCAGAGGAACTTCAGTGTTTGGATGAGATGGGGCCGA
GTTGGGAAAATGGGACAGCACAGCCTGGTGGCTTGTTCAGGCAATCTCAACAAGGCCAAG
GAAATCTTTCAGAAGAAATTCCTTGACAAAACGAAAAACAATTGGGAAGATCGAGAAAAG
TTTGAGAAGGTGCCTGGAAAATATGATATGCTACAGATGGACTATGCCACCAATACTCAG
GATGAAGAGGAAACAAAGAAAGAGGAATCTCTTAAATCTCCCTTGAAGCCAGAGTCACAG
CTAGATCTTCGGGTACAGGAGTTAATAAAGTTGATCTGTAATGTTCAGGCCATGGAAGAA
ATGATGATGGAAATGAAGTATAATACCAAGAAAGCCCCACTTGGGAAGCTGACAGTGGCA
CAAATCAAGGCAGGTTACCAGTCTCTTAAGAAGATTGAGGATTGTATTCGGGCTGGCCAG
CATGGACGAGCTCTCATGGAAGCATGCAATGAATTCTACACCAGGATTCCGCATGACTTT
GGACTCCGTACTCCTCCACTAATCCGGACACAGAAGGAACTGTCAGAAAAAATACAATTA
CTAGAGGCTTTGGGAGACATTGAAATTGCTATTAAGCTGGTGAAAACAGAGCTACAAAGC
CCAGAACACCCATTGGACCAACACTATAGAAACCTACATTGTGCCTTGCGCCCCCTTGAC
CATGAAAGTTATGAGTTCAAAGTGATTTCCCAGTACCTACAATCTACCCATGCTCCCACA
CACAGCGACTATACCATGACCTTGCTGGATTTGTTTGAAGTGGAGAAGGATGGTGAGAAA
GAAGCCTTCAGAGAGGACCTTCATAACAGGATGCTTCTATGGCATGGTTCCAGGATGAGT
AACTGGGTGGGAATCTTGAGCCATGGGCTTCGAATTGCCCCACCTGAAGCTCCCATCACA
GGTTACATGTTTGGGAAAGGAATCTACTTTGCTGACATGTCTTCCAAGAGTGCCAATTAC
TGCTTTGCCTCTCGCCTAAAGAATACAGGACTGCTGCTCTTATCAGAGGTAGCTCTAGGT
CAGTGTAATGAACTACTAGAGGCCAATCCTAAGGCCGAAGGATTGCTTCAAGGTAAACAT
AGCACCAAGGGGCTGGGCAAGATGGCTCCCAGTTCTGCCCACTTCGTCACCCTGAATGGG
AGTACAGTGCCATTAGGACCAGCAAGTGACACAGGAATTCTGAATCCAGATGGTTATACC
CTCAACTACAATGAATATATTGTATATAACCCCAACCAGGTCCGTATGCGGTACCTTTTA
AAGGTTCAGTTTAATTTCCTTCAGCTGTGG
>Macaque_PARP2
ATGCCTGTGACTGGAGGAAATGCTAATGAGGACAGGACAGAAGACAAGCAAGATGGTACG
CCAGGGAGGTCATGGGCCAGCAAGAGGGTTTCTGAATCTGTGAAGGCCTTGCTGTTAAAG
GGCAAAGTTCCTGTGGACCCAGAATGTACAGCCAAGGTGGGGAAGGCTCATGTGTATTGT
GAAGGAAGTAATGTCTATGATGTCATGCTAAATCAGACCAATCTCCAGTTCAACAACAAC
AAGTACTATCTGATTCAGCTATTAGAAGATGATGCCCAGAGGAACTTCAGTGTTTGGATG
AGATGGGGCCGAGTTGGGAAAATGGGACAGCACAGCCTGGTGGCTTGTTCAGGCAATCTC
AACAAGGCCAAGGAAATCTTTGAGAAGAAATTCCTTGACAAAACAAAAAACAATTGGGAA
GATCGAGAAAAGTTTGAGAAGGTGCCTGGAAAATATGATATGCTACAGATGGACTATGCC

ACCAATACTCAGGATGAAGAGGAAACAAAGAAAGAGGAATCTCTTAAATCTCCCTTGAAG
CCAGAGTCACAGCTAGATCTTCGGGTACAGGAGTTAATAAAGTTAATCTGTAATGTTCAG
GCCATGGAAGAAATGATGATGGAAATGAAGTATAATACCAAGAAAGCCCCACTTGGGAAG
CTGACAGTGGCACAAATCAAGGCAGGTTACCAGTCTCTTAAGAAGATTGAGGATTGTATT
CGGGCTGGCCAGCATGGACGACCTCTCATGGAAGCATGCAATGAATTCTACACCAGGATT
CCGCATGACTTTGGACTCCGTACTCCTCCACTAATCCGGACACAGAAGGAACTGTCAGAA
AAAATACAATTACTAGAGGCTTTGGGAGACATTGAAATTGCTATTAAGCTGGTGAAAACA
GAGCTACAAAGCCCAGAACACCCATTGGACCAACACTATAGAAACCTACATTGTGCCTTG
CGCCCTCTTGACCATGAAAGTTATGAGTTCAAAGTTATTTCCCAGTACCTACAATCTACC
CATGCTCCCACACACAGCGACTATACCATGACCTTGCTGGATTTGTTTGAAGTAGAGAAG
GAGGGTGAGAAAGAAGCCTTCAGAGAGGACCTTCATAACAGGATGCTTCTATGGCATGGT
TCCAGGCTGAGTAACTGGGTGGGAATCTTGAGCCATGGGCTTCGAATTGCCCCACCTGAA
GCTCCCATCACAGGTTACATGTTTGGGAAAGGAATCTACTTTGCTGACATGTCTTCCAAG
AGTGCCAATTACTGCTTTGCCTCTCACCTAAAGAATATAGGACTGCTGCTCTTATCAGAG
GTAGCTCTAGGTCAGTGTAATGAACTACTAGAGGCCAATCCTAAGGCCGAAGGATTGCTT
CAAGGTAAACACAGCACCAAAGGGCTGGGCAAGATGGCTCCCAGTTCTGCCCACTTCGTC
ACCCTGAATGGGAGTACAGTGCCATTAGGACCAGCAAGTGACACAGGAATTCTGAATCCA
GATGGTTATACCCTCAACTACAATGAATATATTGTCTATAACCCCAACCAGGTCCGTATG
CGGTACCTGTTAAAGGTTCAGTTTAATTTCCTGCAGCTGTGG
>Medaka_PARP2
ATGAGGCGAACTAGGGGTTCAATGAATAAAAACCAAAGTTTGCCAAAAAACGAAGACATG
GAGTCAATAACAGAATGGCAGTGGCAGGGGGACGGAGGCCGGTGGGAGTCATACCCGCCG
GCGGCGTGTGCCCTGCTGGATTCGGCTCTGTCTGCGGGAACACCGGCCGTCACTCTGAAC
GCGGGTTCCGGCACCGCATACGAGGTGGATCTGAAGAACATGGTCCAGATCAATCCTGTC
ACAAAGTACCGAAGGAAAATTCGCAGTCAGACTGTGAAACCAGACAGTTTAAATGAAGCT
GAACCACAACCTGTCCAGGTTAAAGAGGAAGAGGAGGAGACAGAGGAGCAACCTGTGACG
AAGAAAAGAAGAGGACGAAGCAAGAGTCAGGCAAAAAGCCAAGAAGGAAACAGTGAAGTT
GTGAAGACGGTGGTGATGAAGGGCAGAGCTCCAGTGGACCCTGAATGCAACGCCAAACTC
GGAAAGGCTCATGTTTACAGTGAAGGAAGCATCGTCTATGATGTGATGCTGAACCAGACA
AACCTTCAGTTCAACAACAATAAGTTCTACCTGATCCAGCTTCTGCAGGACGACAGCTCC
AAGTCCTTCAGCGTGTGGATGCGATGGGGCAGAGTGGGCAAAGTGGGCCAGAACAGCCTC
ACGGCGTGTGGTGGAGATCTGCTGAAGGCCAAGGACATCTTCAAGAAGAAGTTCTTTGAT
AAAACCAGGAATGAGTGGGAACAGCGGGGGAGTTTTGTGAAAGTGGCTGGGAAGTATGAC
ATGGTGTTTATGGACTACAGCACCAATGAGAAGGAGGAGAACACCACAGTGGTGGATGCT
GCTACCAACAAGAGGAGCTGCAAGTTGGATCCAAAGGTTCAGTCTCTCCTGGAGCTGATC
TGTGACCTCAAAGCCATGGAGGAGTGTGTGCTGGAGATGAAGTTCGACACACGGAAAGCT
CCTCTTGGCAAGCTGACTTCGGAACAGATCCGTGCGGGCTATGCAGCGCTGAAGAAGATC
GAGGAGTGCTTAAAGAGGAAGGGCAGCAGTCGGGACCTGCTGGAAGCATGCAACCAGTTT
TACACACGCATCCCACATGACTTTGGATTGAGACCTCCTCCGGTCATCCATACAGATGCT
GAGCTGAAGGAGAAGATTTCCCTGCTGGAGGCGCTGAGTGACATCCAGATCGCAGTGAAG
ATGGTCCAGTCCAGCGCAGACAGTGAGGAGCATCCTCTGGACCGACATTACCACTCTCTG
AAGTGTAACCTGCAGCCGCTGGACTCCACCAGCTCTGAGTTCAAGGTGATAGAGAAGTAC
CTGCAGTCCACTCATGCCCCCACCCACCGGGACTACGGCATGACGGTGCTGGACATCTTC
TCAGTGGACAGAGACGGGGAGAGTGAGAGCTTCCTCTCAGATCTGCACAACAGGACTCTG

CTGTGGCACGGCTCCCGGCTCTCAAACTGGGTGGGCATCCTCAGCAAGGGCCTCCGAGTG
GCCCCGCCTGAGGCCCCTGTCACAGGGTATATGTTTGGTAAAGGAATCTACTTTGCTGAC
ATGTCATCAAAAAGTGCCAACTACTGCTTCGCCAACCAGAGCAACCATGTGGGCCTGCTG
CTTCTATGTGAGGTTGCTCTGGGAGACTGCAACGAGCTGCTGGATGCAGACTATGAAGCC
AATAATCTCCCTGCAGGAAAACACAGCACCAAGGGCCTGGGACAGACGGCGCCTGACCCC
AAAAACTCTGTCACTCTGGATGGCGTGACGGTGCCGATGGGTCCAGGAGTGAAAACGGGA
GCGGCTAACAGCAGCGGTTACTCCCTCCTCTACAACGAGTTCATCGTTTACAGTCCAGCT
CAGACTCGCATGAGGTACCTGCTGCGCATCCAGTTCAACTATTCGTCTCTGTGG
>Mouse_PARP2
ATGGCGCCGCGGCGGCAGAGATCAGGCTCTGGAAGGCGAGTGCTAAATGAAGCCAAGAAA
GTTGATAATGGCAACAAAGCAACAGAAGACGACTCTCCTCCTGGCAAGAAGATGCGCACG
TGCCAGAGAAAAGGGCCTATGGCTGGAGGGAAGGACGCAGACAGGACAAAAGACAATCGA
GACTCTGTGAAGACCTTGCTGTTAAAGGGCAAAGCCCCTGTGGACCCAGAGTGTGCAGCC
AAGGTGGGAAAGGCTCATGTGTATTGTGAAGGAGATGATGTCTATGATGTCATGCTAAAT
CAAACCAATCTCCAGTTCAACAACAACAAGTACTACCTTATTCAGCTGTTAGAAGATGAT
GCCCAGAGGAACTTCAGTGTTTGGATGAGGTGGGGCCGAGTTGGAAAGACGGGGCAGCAC
AGCTTGGTGACTTGTTCTGGTGACCTCAACAAAGCAAAAGAAATATTTCAGAAAAAATTC
CTTGACAAAACTAAAAACAATTGGGAGGATCGTGAGAACTTTGAAAAAATACCTGGAAAA
TACGACATGTTACAGATGGACTATGCTGCCAGCACGCAGGATGAAAGTAAAACAAAAGAA
GAGGAAACTTTGAAGCCTGAGTCTCAGCTGGATCTTCGAGTCCAGGAGCTGCTAAAGTTG
ATCTGTAACGTGCAGACCATGGAAGAAATGATGATTGAGATGAAGTATGACACCAAGAGA
GCCCCGCTTGGAAAGCTGACAGTGGCGCAAATCAAGGCCGGTTACCAGTCTCTCAAGAAG
ATTGAGGACTGCATCCGCGCTGGCCAGCATGGGCGAGCGCTTGTTGAAGCGTGCAATGAA
TTCTACACCAGGATCCCTCATGACTTTGGACTCTCCATCCCTCCAGTAATCCGGACAGAG
AAGGAACTGTCAGACAAAGTAAAACTGCTAGAGGCATTGGGAGACATTGAAATTGCCCTT
AAACTGGTGAAGTCAGAGCGCCAAGGCCTAGAACACCCACTGGACCAACACTATAGAAAC
CTACACTGTGCTTTGCGTCCTCTGGACCATGAAAGTAATGAGTTTAAGGTGATTTCTCAG
TACCTACAGTCTACGCATGCTCCTACACACAAGGACTATACTATGACCTTGCTGGATGTT
TTCGAAGTAGAGAAGGAAGGGGAGAAAGAGGCCTTCAGGGAGGACCTTCCTAACAGGATG
CTGCTCTGGCATGGATCCAGGCTGAGTAACTGGGTGGGGATCCTGAGCCACGGGCTTAGA
GTTGCCCCACCTGAGGCTCCCATCACAGGTTATATGTTTGGAAAAGGAATCTACTTTGCT
GACATGTCCTCCAAGAGTGCCAATTACTGCTTTGCCTCTCGCCTAAAGAATACAGGATTG
CTTCTTCTGTCAGAGGTAGCTCTAGGTCAGTGTAATGAACTACTGGAGGCCAATCCTAAA
GCACAAGGATTGCTTCAGGGCAAGCATAGCACCAAGGGGATGGGAAAGATGGCTCCCAGC
CCTGCCCACTTCATCACCCTGAATGGGAGTACAGTGCCCTTAGGACCAGCAAGTGACACA
GGAATTCTCAATCCAGAGGGGTACACCCTCAACTACAATGAGTTTATTGTTTATAGCCCC
AACCAGGTCCGTATGCGATACCTTCTAAAGATTCAATTTAACTTCCTGCAGCTATGG
>Naked_mole_rat_PARP2
ATGGCGGCGAGACGGCGTGCCAGGAGCGGCCGCGGGAAGCTGGCTTCTACAGTATTAAAT
GAAACCAAGAGAGATAATAATGGCACTCCAGCTACAGAAGACTCACCTCCTGCCAAGAAA
ACTCGAAAATGCCAGAGACAGAGAGTGAAAAAGGAGCCTGTGGCTGGAGGAAAGGCTGAT
AAGGGCAGGACAGAAGACACACAAGAACCAGTGAAGTCATTGCTGTTAAAGGGCAAAGCT
CCTGTGGACCCAGAGTGTACAGCCAAGGTGGGAAAGGCTCATGTGTATTGCGAAGGAGAT
GATGTCTACGATGTCATGCTAAATCAAACCAATCTCCAGTTCAACAACAACAAGTACTAC

CTTATTCAGCTGTTAGAAGATGATGCCCGGAGAAACTTCAGTGTTTGGATGAGATGGGGT
CGAGTTGGGAAAATGGGCCAGCACAGCTTGGTGACTTGTTCAGCTGACCTCACCAAGGCC
AAGGAAATCTTTCAAAAGAAATTCCTTGACAAGACAAAAAATAATTGGGAGGATCGTGAG
AAGTTTGTAAAGGTCCCTGGAAAATATGATATCCTGCAGATGGACTACGCTACCAACACT
CAGGATGATAATAAAACAAAACAGGAGGACTCCCTTAAGTCCCCCTTGAAACCAGAGTCA
CAGCTAGATCTTCGGGTCCAGGAGCTGATAAAGTTGATCTGTAATGTCCAGACCATGGAG
GAGATGATGATTGAAATGAAGTATGACACCAAGAAGGCCCCACTTGGAAAGCTGACAGCA
GCGCAAATCAAGGCAGGTTACCAGTCTCTTAAGAAGATTGAAGATTGCATTCGGGCCGGC
CAGCATGGACGAGCTCTCCTGGAAGCATGCAACGAGTTCTACACCAGGATCCCACATGAC
TTTGGACTCCGTACCCCTCCATTAATCCACACCGAGAAAGAACTGTCAGACAAAGTACAA
CTGCTAGAGGCTTTGGGAGACATTGAAATTGCCATTAAGCTGGTGAAGACAGAACTCCAA
AGCCCAGAACACCCATTGGACCAATGCTATAGGAACCTGCACTGTACCTTGCACCCTCTA
GACCATGAAAGTCATGAGTTCACAGTGATTTCCCAGTACCTACAGTCTACACATGCTCCT
ACACACCGTGACTACTCCATGACCTTGCTGGAGGCCTTTGAAGTAGAAAAAGAGGGTGAG
AAAGAAGCCTTCAGAGAGGATCTTCCTAACAGGATGCTGCTATGGCACGGTTCCAGGCTG
AGTAACTGGGTAGGAATCCTGAGCCATGGGCTTCGAATTGCCCCGCCAGAGGCTCCCATC
ACAGGTTACATGTTTGGGAAAGGAATCTACTTTGCTGATATGTCTTCTAAGAGTGCCAAT
TACTGCTTTGCCTCCAGAGTAAAGAATACAGGACTGTTGCTTTTATCAGAGGTAGCTCTG
GGTCAGTGTAATGAGCTACTTGAGGCCAATCCTAAGGCAGAAGGATTACTTCAGGGCAAA
CACAGCACAAAGGGCCTGGGCAAGATGGCTCCCAGTCCTGCCCACTTCATCACCCTGAAT
GGGAGTACAGTGCCCTTGGGGCCAGCAAGTGACACAGAAATTCTAAACCCAGGGGGTTAT
ACCCTCAACTACAATGAGTTTATTGTCTATAACCCAAACCAGGTCCGTATGCGATACCTT
CTAAAGATTCAATTTAATTTTCTGCAGCTGTGG
>Opossum_PARP2
ATGGCTTCGCGCAGGAGCGGTAGAAGCACTAGCCATGCCCGAGGTACAAGGATAAAACTT
CAGTCCCCAGGGTCAGAGTCTGAAAAGAGTGAGGCAGCTTCATCTACAGTTGAATTTCAT
TGGCAATGGGAGGACTCAGAGGGTATCTGGCATTGGTATCCTTCTACTCAGGATATGGAG
ATCACAGAGGCTTTCAGGAACGGAAATGCTTTGGTAAATATCTCATCAGATAGTGGAAAC
ATGAAGCTCCAGGTGGACTTCAAGAAAATGGTCCAGAGGACTACAAAGACAAGGACTGAG
AGGAGAATTGCTGTTGCCGTCAAGGACCAGGGCTCCTATTTTATCTGGCAGTGGCAGGGT
GATGAGGAAGATGGCTGGATCCCATATGATGCTAAGACTTGCCTGGCTCTGGAGAGAACA
CTAGCAGAAAGCAATGAGCTGAAGATAGATGTGATGTTTGGCCGAACACGTTATACTCTG
GATGTAGGCAGCATGGTACAAACCAATTGCAAAAGTGGATACCAACGCAGAATAGAGCGC
CAGCCTTCAGCTGCTGTGGAGTTGACTGAAGCTAATGGAGGCAGTACGAGAAACATATCT
AGTGTTTTATCTATGGAATGCCCTTCTAAAGCCAAGAAAGCCCGAGGTGAATTCGTGGTA
GAAGGAGTGAAACAAGAACTTGTGACAGAAGATGCTGAGAGGAATGGCAAGGCCAAAGAT
AATGAAGTTTCTGTGAAGACCTTATTACTGAAGGGCAAAACTCCAGTGGACCCAGAATGT
ACAATTAAAGTGGGAAAGGCTCATGTATACTGTGAAGGGGATGATGTGTATGACGTTATG
TTGAACCAGACCAATCTCCAGTTCAACAATAACAAGTATTATATCATCCAGCTGCTAGAA
GATGATGGACAGAGAACCTTCAGTGTCTGGACAAGATGGGGCCGAGTTGGGAAGGTTGGA
CAGCACAATTTGGTGACCTGTTCTGGGGACCTCAACAAAGCCAAGGAGATATTTCAGAAG
AAATTTTTTGACAAAACAAAAAATAGTTGGGAAGATCGTGGCAGCTTTAAGAAAGTACCA
GGGAAATATGATATGCTACAACTGGATTATACAAGCAATATTGAGGGTGAAGAGTCAAAG
GATGAGGCACTTGTTCCTATTTCACCTCCCAAACTAGAGTCTCAGCTGGAGCGTCCAGTC

CAAGAGCTGATAGAGTTGATTTGTAACATTCAGAACATGGAAGAAATGATGGTGGAGATG
AAGTATGACATCAAAAAAGCCCCATTAGGGAAGCTGACAGTGGCACAAATCAAGGCAGGT
TACGAGTCACTTAAGAAGATAGAAGATTGTATTCGGTCTGGTCAAAGCGGGCGGATCCTG
GTGGAGGCATGTAATGAATTCTATACTAGGATCCCACATGACTTTGGGCTCCGCACGCCA
CCACTAATCCGAACAGAGCAAGAATTGACAGATAAGATTCAGCTCTTAGAGGCACTGGGA
GACATTGAAATCGCTATCAAGCTGGTGAAGACAGAGCTTAGGAACCCAGAACACCCACTG
GACCAGCATTATAGAAACCTACATTGTGTTCTTCGACCCCTGGACCATTCCTGTCATGAA
TTCAAAGTGATCTCCCAGTATCTACATTCTACCCATGCCCCTACTCACAATGATTATACG
ATGACCTTGCTTGATGTCTTTGAAGTGGAGAAAGAGGGTGAGAATGAAGCCTTCAGGAAG
GATCTTCCCAATAGGATGCTGCTGTGGCATGGTTCCCGACTGGGTAATTGGGTGGGGATC
CTAAGCCATGGACTTCGCATTGCCCCGCCTGAAGCTCCAGTCACAGGCTACATGTTTGGA
AAAGGAATATATTTTGCTGACATGTCCTCCAAGAGTGCCAATTACTGCTTTGCCTCACGA
CTGAAGGACACGGGCCTGTTGCTGCTCTCAGAGGTAGCTTTGGGAGAATGTAATGAGTTG
CTGGAGGCCAATCCTGAGGCAGCAGGACTACTTCAGGGCAAACACAGCACCAAGGGAATG
GGCAAGATGGCTCCCAATCCCTCCCATTATGTTTCTTTGAATGGAACCACAGTGCCTCTA
GGACCAGTGAGTGGCACAGGAATTCTGAACCCACAGGGCTATACCCTCAATTATAATGAA
TTCATTGTCTATAATACTAGCCAGGTTCGTATGCGATACCTGCTGAAAATCAGATTCAAT
TTCACCCAGCTCTGG
>Orangutan_PARP2
ATGGCGGCGCGGCGGCGACGGAGCGCCGGCGGCGGTAGGGCGAGAGCATTAAATGAAAGT
AAAAGAGTTAATAATGGCAACACGGCTCCAGAAGACTCTTCCCCTGCCAAGAAAACTCGC
AGATGCCAGAGACAGGAGTCGAAAAAGATGCCTGTGGCTGGAGGAAAATCTAATAAGGAC
AGGACAGAAGACAAGCAAGATGAATCTGTGAAGGCCTTGCTGTTAAAGGGCAAAGCTCCT
GTGGACCCAGAGTGTACAGCCAAGGTGGGGAAGGCTCATGTGTATTGTGAAGGAAATGAT
GTCTATGATGTCATGCTAAATCAGACCAATCTCCAGTTCAACAACAACAAGTACTATCTG
ATTCAGCTGTTAGAAGATGATGCCCAGAGGAACTTCAGTGTTTGGATGAGATGGGGCCGA
GTTGGGAAAATGGGACAGCACAGCCTGGTGGCTTGTTCAGGCAATCTCAACAAGGCCAAG
GAAATCTTTCAGAAGAAATTCCTTGACAAAACGAAAAACAATTGGGAAGATCGAGAAAAG
TTTGAGAAGGTGCCTGGAAAATATGACATGCTACAGATGGACTATGCCACCAATACTCAG
GATGAAGAGGAAACAAAGAAAGAGGAATCTCTTAAATCTCCCTTGAAGCCAGAGTCACAG
CTAGATCTTCGGGTACAGGAGTTAATAAAGTTGATCTGTAATGTTCAGGCCATGGAAGAA
ATGATGATGGAAATGAAGTATAATACCAAGAAAGCCCCACTTGGGAAGCTGACAGTGGCA
CAAATCAAGGCAGGTTACCAGTCTCTTAAGAAGATTGAAGATTGTATTCGGGCTGGCCAG
CATGGACGAGCTCTCACGGAAGCATGCAATGAATTCTACACCAGGATTCCGCATGACTTT
GGGCTCCGTACGCCTCCACTAATCCGGACACAGAAGGAACTGTCAGAAAAAATACAATTA
CTAGAGGCTTTGGGAGACATTGAAATTGCTATTAAGCTGGTGAAAACAGAGCTACAAAGC
CCAGAACACCCATTGGACCAACACTATAGAAACCTACATTGTGCCTTGCACCCTGTTGAC
CATGAAAGTTATGAGTTCAAAGTGATTTCCCAGTACCTACAGTCTACCCATGCTCCCACA
CACAGTGACTATACCATGACCTTGCTGGATTTGTTTGAAGTGGAGAAGGAGGGTGAGAAA
GAAGCCTTCAGAGAGGACCTTCATAACAGGATGCTTCTATGGCATGGTTCCAGGCTGAGT
AACTGGGTGGGAATCTTGAGCCATGGGCTTCGGATTGCCCCACCTGAAGCTCCCATCACA
GGTTACATGTTTGGGAAAGGAATCTACTTTGCTGACATGTCTTCCAAGAGTGCCAATTAC
TGCTTTGCCTCTCGCCTAAAGAATACAGGACTGCTGCTGTTATCAGAGGTAGCTCTAGGT
CAGTGTAATGAACTACTAGAGGCCAATCCTAAGGCCGAAGGATTGCTTCAAGGTAAACAC

AGCACCAAGGGGCTGGGCAAGATGGCTCCCAGTTCTGCCCACTTCGTCACCCTGAATGGG
AGTACAGTGCCATTAGGACCAGCAAGTGACACAGGAATTCTGAATCCAGATGGTTATACC
CTCAACTACAATGAATATATTGTCTATAACCCCAATCAGGTCCGTATGCGGTACCTTTTA
AAGGTTCAGTTTAATTTCCTTCAGCTGTGG
>Western_painted_turtle_PARP2
ATGAGGCGGAAGCAGAGGGCAGCCGAGGCGGGGGGTGCGGGCCTGGCACTGGAGCTGCGC
TGGGAGTGGCAGGACTCCGGCGGCACCTGGCATCGCTTCGTGCCTGAGCAGAGCGAGGTG
CTGACACAGGCAGCCAGGGCAGGGAAGCCCAGCGTGGCTGTGGGTTCCCGCGTGGATCTG
CGGCGGATGGTGCAGCGGGATGGACAGACGGGGCAGGACAGATGTGTGGCAGCCGCTGTC
CAGGACCAGGACTCCTGGGGACGAAGAGGGGCAGTGGCTGCCCTGCCGATACCTGCCTGG
TGCTGGAGCGAGCGCAGCGTGGCGACGGGGGCCGCAGAGCCGAGCTGCGCGGGGGCCTCC
CAGCCCAAACCAGCCTCACGACTGGACCCCCGAGTGCAAGCGCTGCTGGGGCTGATCTGC
GACCTGCAGGCCATGGAGGAGATGGTGCTGGAGATGAAGGATGACACCAAGAAGGCTCCT
CTCGGGAAGCTGACGGTGGAGCAGATCCGTGCTGGATTCCAGTCACTGCAGAAGGTGGAG
GCGGCCCTGGGCGAGATCCAGATCGCCGCCACGCTGGAGCGCTTAGAGCTGCATGGCCAG
GAGCACCCGCTGGACCGGAGCTACCGCACGCTGGGCTGTGAGCTCCACCCCCTGAAGCAG
AATGGGGCTCCAGTGCTGCTGGGCCCAGCCGTGGAGACGGGCGTGACGAACCCCCATGGC
TACACCCTCAACTACAACGAGTTCGTCATCTACGAGCCAGGCCAGGTGCGGATGCACTAC
CTGCTCCAAGTGCGCTTCAGCTTCGCCCCGCTGTGG
>Panda_PARP2
ATGGCTGCCCGACGGCGGGGGACGCGCAGCGGCGGGGCCCGAGCATCAAATGAAGCTGGA
AGAGTTCATAATGGCAACACAGCCATAGAAGACCCTCCTCCTGCAAAGAAAACTCGAAGA
TGCCAGAGGCAGGGGGTGAAAAAGGAGCCTGTGACTGGAGGAGAGGCCAATAATGACAGG
ACAGAAGACAAGCAAGAGTCTGTGAAGACCTTGCTGTTAAAGGGCAAAGCTCCTGTGGAC
CCTGAGTGCACAGCCAAGGTGGGAAAGGCCCATGTGTACTGTGAAGGGAATGATGTCTAT
GATGTCATGCTAAATCAGACCAATCTTCAGTTCAACAACAACAAGTACTACTTGATTCAG
CTGTTAGAAGATGATGCCCAGAGACACTTCAGTGTTTGGATGAGATGGGGCCGAGTTGGG
AAAATGGGGCAGCACAGCTTGGTGGCTTGTTCAGGGGACCTCAACAAAGCCAAGGAAATC
TTTCAGAAGAAATTCCTTGACAAAACAAAAAATAATTGGGAGGATCGTGAGAAGTTCGAG
AAGGTGCCTGGAAAATATGATATGCTAGAAATGGACTATACCACCAATACTCAGAGTGAA
GAGGAATCAAAAAAGGAATCTCTTCAATCCCCTTTGAAACCAGAATCACAGCTAGATCTT
CGTGTGCAGGAGCTGATAGAGTTGATCTGTAATGTCCAGGCCATGGAAGAGATGATGGTA
GAAATGAAATACGACACCAAGAAAGCCCCACTTGGGAAGCTGACAGTGGCACAAATCAAG
GCAGGCTACCAATCTCTTAAGAAGATTGAGGACTGTATTCGGGCGGGTCAGCATGGACGA
GCTCTCATGGAAGCTTGCAATGAGTTCTACACCAGGATCCCACATGACTTTGGACTCCGT
ACCCCTCCATTAATCCGGACCGAGAAAGAACTGTCAGACAAAGTACAACTACTAGAGACT
TTGGGGGACATTGAAATTGCCATCAAGCTGGTGAAGACAGAGCTGCAAAGCCCAGAACAC
CCATTGGACCAACACTATAGAAAACTACATTGTGCCTTGCGCCCTCTAGACTATGAAAGT
TATGAGTTCAAAGTGATTTCCCAGTACCTGCAGTCTACTCATGCTCCCACACACAGGGAC
TATACCATGACCTTGCTGGATGTTTTTGAAGTGGAGAAGGAGGGTGAAAAAGAAGCCTTC
AGAGAGGACCTTCATAACAGGATGCTGCTGTGGCATGGTTCCAGGCTTAGTAACTGGGTG
GGAATCCTGAGCCATGGGCTTCGAATTGCCCCACCTGACGCTCCCATCACAGGTTACATG
TTCGGTAAAGGAATCTACTTTGCCGACATGTCTTCTAAGAGTGCCAATTACTGCTTTGCT
TCTCGAGTAAAGGATACCGGACTATTGCTTTTATCAGAGGTCGCTCTAGGTCAGTGTAAT

GAGCTACTAGAGGCCAATCCCGAGGCAGAAGAATTACTTCGGGGCAAACATAGCACTAAG
GGCCTGGGCAAGATGGCTCCCAGTCCTGCCTCCTTTATCACCCTGAACGGGAGTACGGTA
CCCTTAGGACCAGCAAGTGACACAGGAATTCTGAATCCAGAAGGTTATACCCTCAACTAC
AATGAATTTATTGTCTATAGCCCCAACCAGGTCCGTATGCGGTATCTTCTAAAGGTTCGG
TTTAATTTCCTGCAGCTGTGG
>Pig_PARP2
ATGGCTGCGCGCGGGAGAGGGACCTGCAGCCGCCGGACGCGAGCATTAAATGAAGCTGCA
AAAATTAATGGCAAAACAGCTACAGAAGACACTCCTCCTGCAAAGAAAATTCGAAGATGT
CAGAGGCAGCAGGTGAAAAGGGAGCCTGTGGCTGGAGGAAAGGCTGACAATGACAGGACA
GAAGACAAGCAAGAGTCTGTGAAGACCTTGCTGTTAAAGGGCAAAGCTCCAGTGGACCCA
GAGTGCACAGCCAAGGTGGGAAAGGCCCATGTATACTGTGAGGGGAATGATGTCTATGAT
GTCATGCTAAATCAGACCAATCTCCAGTTCAACAACAACAAGTATTATCTGATTCAGCTG
TTAGAAGATGATGCCCAGAGGAACTTCAGTGTTTGGATGAGATGGGGCCGAGTTGGGAAA
ATGGGGCAGCATAGCTTGGTGGCTTGTTCAGGGGACCTCAACAAGGCCAAGGAAATCTTT
CAAAAAAAATTCCTTGACAAAACAAAAAATAATTGGGAGGATCGTGAGAAGTTTGAAAAG
GTGCCTGGAAAATATGATATGCTACATATGGACTATACCACCAATGAACAGAGTGAAGAG
GAAACAAAAAAAGAAGAATCTCTTAAATCTCCCTTGAAACCAGAGTCACAGCTAGATCTT
CGTGTACAGGAGCTGATAAAGTTGATCTGTAACGTCCAGGCCATGGAAGAGATGATGGTA
GAAATGAAATACGATACCAAGAAAGCCCCACTTGGGAAGCTGACAGTGGCACAAATCAAG
GCAGGTTACCAGTCTCTTAAGAAGATTGAGGATTGTATTCGGGCTGGCCAGCATGGACGA
GCTCTTGTGGACGCGTGCAATGAATTTTACACCAGAATCCCACATGACTTTGGACTCCGT
ACTCCTCCATTAATCCGGACAGAGCAAGAACTCTTAGACAAATTACAGCTACTAGAGGCT
TTGGGAGACATTGAAATTGCCATTAAGCTGGTGAAAACAGAACTGCAAAGCCCAGAACAC
CCATTGGACCAGCACTATAAAAAACTACATTGTGCTTTGCACCCTTTAGACCATGAGAGT
AACGAATTCAAAGTGATTTCCCAGTACCTACAGTCTACCCATGCTCCCACACACAGTGAC
TATACCATGACCTTGCTGGATGTTTTTGAAGTAGAGAAGGAGGGGGAGAAAGAAGCCTTC
AGAGAGGACCTACCTAACAGGATGCTGCTATGGCATGGTTCCAGGCTGAGTAACTGGGTG
GGAATCCTGAGCCACGGGCTTCGAATTGCCCCACCTGAAGCTCCCATAACAGGTTACATG
TTTGGAAAAGGAATCTACTTTGCTGACATGTCTTCCAAGAGTGCTAATTACTGCTTTGCC
ACTCGCCTAAAGGATACTGGACTGCTGCTCTTATCAGAGGTAGCTCTAGGTCAGTGTAAT
GAGCTATTAGGGGCCAATCCAGAGGCAGAAGGATTACTTCAGGACAAGCATAGCACCAAG
GGGCTGGGCAAGATGGCTCCCAGTCCTTCACGTGCCATCACCTTGAATGGGAGTACGGTG
CCCTTGGGACCAGCAAGTGACACAGGAATTCTGAATCCAGAGGGTTATACCCTCAACTAC
AATGAGTTTGTTGTCTATAACCCCAATCAGGTCCGTATGCGATACCTCCTAAAGGTTCAA
TTTAATTTTCTGCAGCTGTGG
>Platypus_PARP2
ATGGCGGCTCGCCGCCGGACCGGGAGGAACGGGGGCCCTCGAAGGGCGGAATTAGAGGTG
TCCCCGTGCCCGCAGGGCGGCCAGGGTCGCACGGACGGGCTGCGCTGGGAATGGCAGGAC
CGAGGGGGCTCCTGGCGCCTCTACGCCCCCGCGCTGGACATGGACATCTCCCGAGCTTTC
AGGGCAGGAGACAGTCTGGTGAGCATCTCACCCGATGGGGGAGACACAGAACTGCAAGTG
GACCTGAAGAGGATGGTCCAAAGAAACGTGCAGACCGGAATGGAGCAGCGGATCGCCGCT
GCAGTAGGAGGGCCCGGCTCCTATTACATCTGGCAGTGGCAGGAAGAGGAGGAGTGGGTC
CCATACCCTCCCGAAACCTGCCTGGCTCTGGAAGCAGCCCGGGAGGGAGGGGGCGGCAGC
AAACCGAAGGTGGACCTAGCAGTCGGCCGCACCCGCTACACCCTGGACCCAGGCAACATG

CTGCAAACCAACCGCAGGAGTGGCTTCCAGCGCCAGATGGAGCGTCGGCCCTCGGTTGCT
GTGGAATTAAGTGGAGCCGGAGGTTCCAGTGACATCTCATTGGCGGACGGGCCCCCTGTG
GTCAAGAAAGCCCGTGGGCGCCAGCAGCCAGGAAGGCCCGCGGATGCCAGCGGTAGGAAA
CAAAAGGCAGTGGCAGGAGCAGATGCAAAAGGAGATGCCAGAGAAGGAGAGAGCAGTGAG
GCTGTGAAGACCTTGCTTTTGAAGGGGAAGGCTCCAGTGGATCCAGAGTGCACAGCCAAA
CTGGGCAGGGCCCATGTGTACTGTGAAGGGGACGACGTGTATGACGTCATGCTGAGCCAG
ATCCAGCTGGCTGGCCGGCCGGCCGGCCTGCCTGCGCCCCACCTCCATCCAGAGAAACCC
TGCCCTTTGCCCTCTCCTCGCATTGTTCTCTGCTGCGGCAATGGGCCGGGGGCCCAGGCC
GCACAGACCAAAGGTGGAGGGAAGAATTGCCTGGCGGTGACCCTGAAGCCTGGGCAGATT
GAC
>Rabbit_PARP2
ATGACGTTAGATCTCCATGGCGGCGCGGCGGCGGCGGGCGGCTTCCAGCCGCGGCGCTCG
AGGGTCTTTATTTGTTCACTCTTGTTCTTAGCTTCTGTAGCAGTAAATGAAACCAATTCA
GTCACCGAAGACTCTCCCCCTGTCAAGAAAACTCGAAGATGCCAGAAACTAGAGATGAAA
AAGGAACCTTCGGCTGGAAAAAAGGCTGATAAGGAGGGGACAGAAGACAAGCAAGGTGAA
TCTGTGAAGGCCTTGCTGATAAAGGGCAAAGCTCCTGTGGACCCGGAGTGCACAGCCAAA
GTGGGAAAGGCTCATGTGTATAGTGAAGGAAATGAGGTCTATGATGTCATGCTGAATCAG
ACCAATCTCCAATTCAACAACAACAAGTACTATCTGATTCAGCTATTAGAAGATGATGCC
CAGAGGAACTTCAGTGTTTGGATGAGATGGGGCCGAGTTGGGAAAATGGGGCAACACAGC
TTGGTGGCTTGTTCAGGTGACCTCAGCAAGGCCAAGGAAATCTTTCAGAAGAAATTCTTT
GACAAAACCAAAAATAATTGGGAGGACCGCGAAAAGTTTGAGAAGGTACCTGGAAAATAT
GATATGCTACAGATGGACTATGCCGCCAATACTCAGGGTGAAGATGAAACAAAAAAAGAG
GAGGCTCTTAAATCTTTGAAACCAGAGTCACGGCTGGATATTCGGGTGCAGGAGCTGATC
CAGCTGATCTGTAACGTCCAGGCCATGGAAGAGACGATGATAGAAATGAAGTATGATACC
AAGAAAGCCCCCCTTGGGAAACTGACAATGGCACAAATCAAGGCAGGTTACCAGTCTCTT
AAGAAGATTGAGGATTGTATTCGGGCTGGCCAGCATGGGCGAGTTCTCTTGGAAGCTTGC
AATGAATTCTATACCAGGATCCCACATGACTTTGGACTCCGTACCCCTCCATTAATCCGG
ACAGAGAAAGAATTATCAGATAAAGTACAGCTACTGGAGACTTTGGGAGACATTGAAATT
GCCATTAAACTGGTGAAGACAGAGCTACAAAGCCCAGAACACCCATTGGACCAACACTAT
AGAAACCTACACTGTTCCTTGCACCCTCTAGACCATGAAAGTCATGAGTTCAAAGTGATT
TCTCAGTACCTACAGTCTACCCATGCTCCTACACACAATGACTATACCATGACCTTGCTG
GATGTTTTTGAAGTAGAGAAGGAGGGTGAGAAAGAAGCCTTCAGAGAGGACCTTCATAAC
AGGATGCTGCTGTGGCATGGATCCAGGCTGAGTAACTGGGTAGGAATCCTGAGCCATGGG
CTTCGAATTGCCCCTCCTGAAGCTCCCATCACAGGTTACATGTTTGGAAAAGGAATCTAC
TTTGCTGACATGTCTTCCAAAAGTGCCAATTACTGCTTTGCCTCTCGCCTAAAGAATATA
GGACTGCTGCTCTTATCAGAGGTAGCTCTCGGGCACTGTAATGAATTACTAGAGGCCAAT
CCTAAAGCAGAAGGATTACTTCAGGGCAAACACAGCACCAAGGGACTAGGCAAGATGGCT
CCCAATCCCACCTCCTCCATCACCCTGAATGGGAGTACAGTGCCATTAGGACCAGCAAGT
GACACAGGAATTCTGAATCCAGAGGGTTATACTCTCAACTACAATGAGTTTATTGTCTAT
AATCCCAACCAGGTCCGTATGCGGTACCTTCTAAAGGTTCAGTTTAATTTCCTACAGCTC
TGG
>southern_platyfish_PARP2
ATGAGGAGAACCAGGGGTTCCAGAAATAAAAGCCAGAGTACCGTAGAAAACGAGGAAGTC
CCGACAAAGACAGTGTGGCAGTGGAAAGGAGATGACGGGCAGTGGGAACCGTACCCAGAT

GAAGCGGGCTCCATGCTGGACTCAGCTTCCTCATCAGGAAAGACATCGGTCACTCTGACT
CTTGGCGCAGGGAAGAAATATGAAGTTGATCTGAAGAAAATGGTCCAGATTAATCCTACG
ACAAAGTACAAGAGAAAGATTCGCTCTCAGATAGTAAAAACTGAGAGTTTAAATGAAGCC
GGTGACGTGGCGGCTGAAAATGGAGATTCAGTTCAAGTCAAAGAGGAAGAGGAAGAGGAG
GAGACAGCGGAGCAGCCGGCAGCTAAGAGGAGAAGAGGACAGAGGAAGAGTGCAGCAAAA
ACCAAAGAAATGCCTAAAGAGGATATAAAAACTGAAGAGGTGGTGAGGACGGTGGTGATG
AAGGGCAAAGCTCCAGTGGACTCTGAATGCAAAGCTAAACTGGGAAAGGCTCATGTTTAC
AGTGAAGGAAATGATGTTTACGATGTGATGCTGAACCAGACCAACCTGCAGTTCAACAAC
AATAAATATTACCTGATCCAGCTGCTGGAGGACGGCGGCGCCAAGGCTTACAGCGTCTGG
ATGAGATGGGGCCGAGTGGGTAAAGTGGGTCAGAACAGCCTCACACCCTGCGGGGCAGAT
CTGCTGAAAGCCAAAGACATCTTCAAGAAAAAGTTCTTGGATAAGACAAAGAACGAGTGG
GATCACAGGGAATGTTTTGAGAAAGTAGCCGGAAAATATGACCTGGTGTTCATGGACTAC
GCCGCTAACGAGAAGGAGGAGAACATCACCACAGTCGACGTCGCGCCCAGAAAACAGCCC
TCCAAGCTGGAAGCGAAGCTCCAGTCGCTCCTCGAACTCATCTGTGACCTCAGAGCCATG
GAGGAGTGTGTGCTGGAGATGAAGTTCGACACCAGAAAAGCTCCTCTTGGAAAGCTGACC
TCGGAGCAGATCCGCGCCGGATACGAAGCCCTGAAGAGAATCGAGTCGTGCTTGAAGAAG
AAGGGAAGCAATAAGGAGCTGCTGGAAGCCTGCAACCAGTTTTACACCCGTATCCCGCAT
GACTTTGGGTTAAAAACGCCTCCAGTGATTCGCTCAGAAGAAGAACTGAAGGAAAAAATT
GCTCTGTTGGAGGCGCTGAGTGACATCCAGATAGCAGTGAAAATGGCCGAGTCCAATGCA
GACAGTGACGAGCATCCTTTGGACAGACAGTATCACGCCCTGCAGTGCCAACTGGAGCCT
TTGGACCAGAGCAGCCATGAGTTCAAGGTGATAGAACGGTACCTGCAGTCCACTCACGCT
CCCACTCACTCCGACTACACCATGAAGGTCCTGGACGTCTTCTCAGTGGACCGGGGCGGC
GAGAGCGACAACTTCCTGTCTCATTTACACAACAGGACTCTGCTGTGGCACGGCTCCCGT
CTGTCTAACTGGGTCGGGATCCTCAGTAAGGGGCTCCGAGTGGCGCCGCCTGAGGCCCCC
GTCACCGGCTACATGTTTGGTAAAGGGATTTACTTCGCAGACATGTCGTCAAAAAGTGCC
AACTACTGTTTCGCCAACCAGAGCCACCATGTTGGATTGCTGCTGCTGAGTGAGGTGGCT
TTAGGGGACAGTAACGAGCTGCTGGACGCCGACTATGAGGCCCATAAGTTGCCTGCAGGA
AAACACAGCACCAAGGGCCTGGGTGTGACCGGACCCGACCCAAAAAACGCCGTCACGCTC
AACGGAGCGACGGTTCCGATGGGTCCCGGCGTTAAAACGGGGGTTTCTAACTCCAACGGC
TACTCTCTGCTCTACAACGAGTTCATCGTTTACAACCCGGCTCAGACCCGGATGAGGTAC
CTGCTGCGGATCCAGTTTAACTACTCCTCTCTCTGG
>Taiwan_habu_PARP2
ATGAAAAGGAAACGAAAGGCTCCCAAAGTCCAGCCTGCCGATGCAGGGACCCAGCCTGCA
CTGCGCTGGGAGCGCGAGATCAGTGACAACAAATGGCAGATCTTCCCACCTGGACAGAGT
GAGGCCCTGAGCCAAGCCACTCGGGTGCGGAAAAGTACAGAAGATTTTGGGGAAGACATT
GTGGACCTGTGCAAGATGGTGGAGTGGAACAAGCAGAGCGGTGAGGAGAACCGTGTCCGT
GTGGCTGTCTGGGATCAGAAATCCTATTTGGTCTGGCAATGGGAAGGAGACCAAGAAAAC
GGCTGGATCCCATACCCCGCTGCTACCTGCTTGGATTTACAGGCAGCCAAGAACGGTCAC
CGGGAGCCCATAGTGGACTTGACAGTTGGTCGGACCCGCTACAAGCTGGACACGGACCGC
ATGGTGCAGAGAAACCAGCGGACTAAATTTGAACGTCAGATGGAATGCAGGGAATCAGAT
GCAACAGAGGCTGCGGGCGGATCCCAGCTGAACAGCCGTGACTTGGACGCCAGTACGGCG
GTTCCTCCTAAGAAGTCCCAGACCGAATTAGATGCCCCAGCATCGCATGGGGAAGGAGAT
GGGCTGGGAGACGACATTTACGACGTGATGTTAAATCAGACAAACCTTCAATTCAACAAC
AATAAATACTACCTCATACAACTGTTGGAAGAAGACAATGCACAGAACTACAGCGTCTGG

ATGCGCTGGGGGCGCGCGAAAGCCAAAGACATCTTCACCAAAAAGTTCCTAGACAAAACC
AAAAACGAATGGGACAAACGGAACAGCTTCCAGAAAGTCTCAGGCAAATACGATCTCCTT
CATCTGGACTACGAAGCCAGAGATGCTAAGAATTCAGCAGAAGCTGCCCCTGAGAAGACA
ATCTCTAAGCCCAAGCCAGTTTCCCAGCTGGAGCCACGGGTGCAGGCTTTGCTAGAGCTG
ATATGTAATATTCGGACGATGGAGGAGATGGTGATCGAGATGAAGTACGACACCCGGAAA
GCTCCACTAGGAAAGCTGACAGCTGAGCAGATCCAGGCAGGGTACCGGTCATTACAGAAA
GTGGAAGCATGTCTGAAGCGGAGTCAGACTGGTGCTGCCCTGCTGGAAGCTTGCAATGAA
TTCTACACACGGATCCCCCATGACTTCGGGCTGAAAACACCACCACTAATAAAATCTTTA
AGGGAACTCCAAGAAAAGGCACAGCTGCTGGAGGCTCTCAGTGAGATCCGTATTGGCATC
AAACATGTGCAGTCAGAACAGTTGGATCTGGAGCATCCTTTGGACCGGAGCTACAGGAGT
CTCGACTGTGAACTCCAGCCCTTGGAAAAGGCCAGTGATGTTTTCCAGGCTCATGTTTAT
TGTGAAGGAGACGACATTTACGACGTGATGTTAAATCAGACAAACCTTCAATTCAACAAC
AATAAGTACTACCTCATACAACTGTTGGAAGAAGACAATGCACAGAACTACAGCATCTGG
ATGCGCTGGGGGCGCGTGGGTAAGCCTGGCCAGCACTCCCTCGTGCCTTGCTCTGGAGAT
TTAGCGAAAGCCAAAGACATCTTCACCAAAAAGTTCCTAGACAAAACCAAAAACGAATGG
GACAAACGGAACAGCTTCCAGAAAGTCTCAGGCAAATACGATCTCCTTCATCTGGACTAC
GAAGCCAGAGATGCTAATTCAGCAGAAGCTGCCCCTGAGAAGACAATCTCTAAGCCCAAG
CCAGTTTCCCAGCTGGAGCCACGGGTGCAGGCTTTGCTAGAGCTGATATGTAATATTCGG
ACGATGGAGGAGATGGTGATCGAGATGAAGTACGACACCCGGAAGGCTCCACTAGGAAAG
CTGACAGCTGAGCAGATCCAGGCAGGGTACCGGTCATTACAGAAAGTGGAAGCATGTCTG
AAGCGGAGTCAGACTGGTGCTGCCCTGCTGGAAGCTTGCAATGAATTCTACACACGGATC
CCCCATGACTTCGGGCTGAAAACACCACCACTAATAAAATCTTTAAGGGAACTCCAAGAA
AAGGCACAGCTGCTGGAGGCTCTCAGTGAGATCCGTATTGGCATCAAACATGTGCAGTCA
GAACAGTTGGATCTGGAGCATCCTTTGGACCGGAGCTACAGGAGTCTCGACTGTGAACTC
CAGCCCTTGGAAAAGGCCAGTGATGTTTTCCAGGTACTGGAGCGCTATTTGCTCTCCACC
CATGCTCCCACCCACAAGGATTACACAATGACTCTGGTCGAGGCCTTTGAAGTGAACAAG
AAAAGCTCAGAAGCCACTTTCCGTTTTGACCTGTCCAACAGGATGCTGCTGTGGCACGGA
TCTCGCCTGGGGAACTGGGCGGGGATTTTGAGCCAGGGGTTGCGTGTGGCACCACCAGAA
GCACCCATCACGGGCTATATGTTTGGAAAAGGCATTTACTTCGCGGACATGTCCTCTAAG
AGTGCCAATTACTGTTTTGCCACTCGCGAGAAAGACATCGGCCTCCTCCTGCTGTCAGAG
GTTGCGCTAGGAGAGTGCAATGAACTGTTGGAGGCCGATCCAGAAGCAGAACGACTGCCA
GCCAGCAAACACAGTACCAAAGGCTTAGGCAAAATTGCTCCAGCCAGTTGTGTTTCTCTG
CATGATGCAACTGTTCCCCTGGGCCCAGCTGTAGAAACTGGGATAGCAAACCCTCGTGGC
TATACCCTGAATTACAATGAATTCATTGTCTATGACCCCTGCCAGGTGCGTATGAAATAC
CTTCTGAAGGTGCGCTTCAATTTTGTACAGCTGTGG
>Tetradon_PARP2
ATGCTTCAGATTAATCCTGTGACCAAGTACAAGAGGAAGATTCGTTCACAAATCAAGGAG
GAAGAGGAGGAAGAAGATACAAATAAGCAACCTGCTGCTAAGAGGAGGAGAGGACAGAGT
AAGAGTCAAACAAAATGTGAAGAAATGCCTAAAGAGGAGATAAAAAGCAAAGAGGTGGTA
AAGACATTGGTCCTGAAGGGAAAAGCTCCAGTGGACTCTGAATGCAAAGCCAAGCTCGGA
AAGGCTCATGTTTACAGTGAAGGAAATGATGTGTATGATGTCATGTTAAACCAGACAAAC
CTTCAGTTTAATAATAACAAATACTACCTGATGCAGCTGCTGGAAGATGATGGATCTAAA
CTTTACAGTGTGTGGTTTAGGTGGGGGAGAGGGCATTTAGTGGGGAAAGTGGGTCAAAAC
AGTCTGACAGAATGTACTACAGACCTGAACAAAGCCAAAGATATCTTCAAGAAAAAGTTC

TTTGACAAGACTAAAAATGAGTGGGAACGTAAAGACCAGTTTGAAAAAGTAGCTGGAAAA
TACGACATGGTGTTTATGGACTACAGCACAGAGGAAAAGGAAAAGAAACACTCTGTGGTG
GAGACTAAAAAACAGGCCTCCAAGCTGGATGTGAAGATACAGTCGCTATTGGAGCTAATC
TGTGACCTTAAAGCCATGGAGGAATGTGTGCTGGAAATGAAGTTTGATACGAGAAAAGCT
CCACTTGGTAAATTGACGCCGGAGCAGATTCGTGCAGGTTACGTGGCTCTGAGGAAAATT
GAGGACTGTTTGAAAAAAAAGGCCAGCCATCGTCAGCTGTTGGAAGCATGCAACCAGTTC
TACACTCGTATCCCTCATGACTTTGGGTTGAAAACTCCTCCAATTATTCAAACGGAACAA
GAGCTAAAGGACAAGATTGCACTTTTGGAGGCGCTGAGTGATATACAGATTGCCGTCAAA
ATGGTAAAAGCCAACGAAGACAGTGATGAAAATCCTCTGGACAGACAGTATCGCGCCCTC
CAGTGCAGACTGCAGCCCCTGGACGCCGGCTGCCACGAGTACGAGGTGATTGAAAAGTAC
CTCCAGTCTACTCATGCTCCCACCCATTCAGACTACACCATGAGCGTGCTGGACATCTTC
GGAGTTGACAGGGAAGGAGAGAGTGACAGCTTCCTCTCAGACTTACCCAACAGCCTACCA
AGGACCTTGTTGTGGCATGGGTCCCGTCTGTCTAACTGGGTTGGCATCCTGAGTCAGGGC
CTCAGAGTAGCTCCACCTGAAGCTCCTGTCACCGGTTACATGTTTGGAAAAGGCATCTAC
TTCGCTGACATGTCATCCAAAAGTGCCAACTACTGTTTTGCCAACCAGAGTAACCACGTC
GGATTGCTGCTGCTGTGTGAGGTTGCCCTGGGTGACAGCAACGAGTTGTTGGATGCAGAC
TATGAGGCCAACAATCTGCCTAATGGGAAACACAGCACCAAGGGCCTTGGACGGACTGGA
CCTGACCCCAAAAACGCTCTCACTCTAGAGGGTGTGACTGTACCAATGGGTCCTGGAGTA
AACACGGGGGTGGGTAAACATAAAAGTTATACCCTTCTTTACAACGAGTTTGTAATTTAC
AACCCTGCCCAAATTCGAATGAGGTATCTGCTGCGGATCAAGTTCAACTACTCCTCACTG
TGG
>Tibetan_frog_PARP2
ATGTCTGGGCGCAGGGGCTCAGGCAGGGAGCGCAGGCCCCGCGCAGTGTCCCAGAAGAGG
AAAGCTGAGGCAGATTCTGAGCCAGAAGTTGTTATAAAGACTGAACTTGAAGATGTGAGT
GTTGCATCTGCCGAATCTGGAAAAGTCATAGCTAAATCTGCTGTAAAGTCTGAGCTAGTG
ACCCGCTGGGAATGGCTGAATGATGGAGACATGTGGATGGTTTATGCTGCTGAGCTTAAC
AGACAAATTAACCAAGCACTCGGCAATGGGAAACAAAGCTTAACACTCTCACCAGCCGTA
GGCGTGTCTCTGCAAGTGGATTTTAGGAAGATGATCCAAAAAAATACAAAGAGTGGTTAC
CAAAGACAAATACGTCTTGCAGTCAAGGAACAGGACCAATATTTTGTATGGCAGTGGAAG
TCAGATGATGATTACTGGGTCTCCTATGATGCCAAAACCTGCGTCTTGCTGGAGTCTGCA
CTGCAGGATGATGAAAAGCATGTGTCCCTAAGTTTGGGTGGCAGACCTTACACAGTTGAT
CTTGGAGCTATGGTGCAGAAAAATACCCAGACTCAGCATGAGCGAGAGATTCAGCGTTGT
TTATCTGTTGCTGTAGATCAAGGAAGTGACGAGCCAAAACAAAGTGTCCCTAATGGACCA
ACTTCTTCAAAACGTCCCCGAAGGAACAAAAGTGTAGAAATCGTGGAAACTGAAGAGGAC
AGCAAAGAACAAGTGAAAACTCTGGTTTTAAAAGGTAAAGCTCCGATTGATCCAGAATGT
TCCAGCAAAATTGGAAAGGCTCATGTGTTCTGTGAAGAAGATGATGTATATGATGTTATG
TTAAACCAGACTAACCTGCAGTTTAATAACAATAAGTACTACCTGATCCAGCTGCTTGAA
GATGACGGCGCTAAACGTTTCTCTGTCTGGATGCGCTGGGGACGAGTGGGAAAGGTGGGT
CAAAATTCTCTGGTTTCATATGGAGGAGACCTTCAAAAAGCAAAGGACGTATTTCAGAAG
AAGTTTTTCGATAAGACCAAGAATGTTTGGTCAGAGCGTGGAAATTTCGAAAAGGTTCCT
GGTAAATATGACATGTTGCATTTGGATTATAACACAACCACGGAGGAAGAGAAAAATGTT
GTGGAAGTTGACAAGCTATCTGACATTCCCAAACCTGAATGCAAACTGGACAACTTGGTT
CAGGCCTTGATCCAACTTATCTGTAACATGCAGAACATGGAAGATACTGTGCTAGAGATG
AAGTATGACACAAAGAAGGCACCTCTGGGAAAGCTCACTGTGGATCAGATCCATGCGGGG

TACAGCTCCCTGCAACGTATTGAGAACTGTATTAAACAACAAAAGTTTGGCAAACATCTT
CTTGAAGCCTGCAATGAATTTTACACCAGAATTCCACATGATTTCGGACTAAAGACACCC
CCATTAATCAGGACAGTAGAGGAGCTGGCTTTGAAAGTGCGACTACTAGAAGCTTTGGGT
GACATTCAAATTGCAGTGAAGCTGGCAAGTATGGATCTCGGCTCCCATGAGCACCCACTG
GACAGGCATTATCGGCAGTTACAATGCTCACTGGAGCCTTTGGGTAAAACATCAGATGAA
TTCCAGCTTATTGAGTGCTACTTGAAAACAACGCATGCCTCTACTCATAATGATTACACT
ATGACCTTAATGAATGTTTTCCGGCTAAAAAAAGAAGGTGAGGAGAGCAACTTCCGAGCA
GAACTCCCCAACAGAATGCTTCTCTGGCATGGATCTCGTCTGTCTAACTGGGTTGGCATC
CTTAGCCAAGGTCTGCGTGTGGCTCCCCCAGAAGCCCCAGTAACAGGGTATATGTTTGGC
AAAGGAATTTATTTTGCTGATGTCTCCTCAAAGAGTGCAAATGACTGCTTCACCTCTCGT
GATAAGAATGTGGGAATTCTTCTACTTTGTGAGGTGGCCCTTGGTAACTGTAATGAGCTT
ATAGCTGCAGATTATGATGCTCAGAAACAATTGAAGGGAAAACACAGCACAAAAGGTGTT
GGCCGTAGTATTCCAGACCCACAGAAGTCTATCAAACATGAAGGAGCAATGGTTCCGATG
GGGCCCCTGATCGACACAGGACTACAGAATAATGATGGCTATACACTAAATTACAATGAG
TACATTGTGTATGACTCTTGTCAGGTACAAATGAAGTATTTGCTGCAAGTAAATTTCAAC
TATGAATCTCTGTGGTAA
>Tilapia_PARP2
ATGAGGCGAGCGAGAAGTTCGCGAAATAAATGTGAGAGTGCACCAGAAAATGGAGAAGCC
CAGTCAAAAACAGTGTGGCAGTGGATGGGAGATAAAAGACAGTGGGAACCATACTCTCCA
TCAGCCTGTGCCGTCTTGGACTCGGCCGTCTCTTCGGGAAAAACATCTGTCACTCTCTCT
CTGGGCTCGGGGACAGCCTATGAAGTTGATCTGAAGAAGATGGTTCAGATCAATCCTGTC
ACAAAGTACAAACGGAAAATTCGCTGTCAGACTGTAAAAACAGAAAGTCAGAATGAAGCT
GGTGACGTTGCTGCTCATAATGAGAAACCAGCTCAAGTTAAAGAGGAAGAGGAGGACACA
GAAGAGCAGCCTGCAGCTAAGAAGAGGAGAGGACAAAGCAAGAGCCAGACAAAAACTGAA
GAAATGCCCAAAAAAGCCAAAGAAGAAGTAAAAAGTGAAGAAGTTGTGAAGACAGTGGTC
ATGAAGGGAAAAGCACCAGTAGACTCTGAATGTAAAGCCAAACTAGGAAAGGCTCATGTT
TATAATGAAGGAAATGATGTTTACGACGTGATGCTAAACCAGACAAATCTTCAGTTTAAC
AACAACAAATATTACCTGATCCAGCTGCTGGAGGACGACAACTCCAAGGTTTACAGTGTG
TGGATGAGATGGGGCAGGGTGGGTAAAGTAGGTCAAAACAGCCTTACGGCCTGTGGTGGA
GACCTGCTGAAGGCCAAGGACGTCTTTAAGAAAAAGTTCTTTGAGAAGACCAAGAACGAG
TGGGAGCATCGAGATAGTTTTGAGAAAGTAGCTGGAAAATATGACATGGTGTTTATGGAC
TACAGCACGAATGAAAAGGAGGAGAATCAGACCACAGTGGATACTAAACCAAAAAAGAAG
ACCTCTAAGCTGGATTTAAGGATTCAGTCTCTCCTGGAGCTCATCTGTGATCTGAAAGCC
ATGGAGGAGTGTGTGCTGGAGATGAAGTTTGACACCAAGAAAGCTCCTCTTGGCAAGCTG
ACCCCAGAGCAGATCCGAGCAGGTTACTCAGCACTGAAGAAGATCGAGGATTGCTTGAAG
AAGAAGAAAGGGAGCAATCGTGACCTTTTAGAAGCATGCAATCAATTTTACACCCGCATC
CCACATGACTTTGGGTTGAAAACTCCACCACTTATCCAAACAGAAGAAGAGCTGAAAGCC
AAAATTGCCCTTTTGGAGGCACTGAGTGACATCCAGATAGCAGTAAAAATGGTCAAGTCC
AGTGAAGACAGCGATGAACATCCTCTGGACAGACAGTATCACTCCCTCCAGTGCAAACTG
CAGCCTCTGGACTCCAGCAGCAACGAGTACAAGGTGATAGAGAAGTATCTGCACACCACT
CACGCCCCCACCCACTGCGACTACACCATGACTGTGCTTGACATTTTCTCAGTGGACAGA
GGCGGGGAGAGCAACAGTTTCCTGTCACAGTTAAGTAACAGGACTCTGCTGTGGCACGGT
TCACGTCTGTCTAACTGGGTCAGCATCCTCAGTCAGGGACTTCGAGTGGCCCCCCCTGAA
GCTCCTGTTACTGGTTACATGTTTGGTAAAGGTATCTACTTTGCTGACATGTCATCCAAA

AGTGCCAACTACTGCTTTGCCAACCAGAACAACAACGTTGGACTGCTGCTACTGTGTGAA
GTCGCTCTGGGAGAATCTAATGAGCTGCTGGATGCGGACTATGAAGCAAACAATCTGCCT
GCTGGAAAGCACAGCACCAAGGGGCTGGGACAGACTGGACCTGACCCCAAAAACTCTGTC
ACACTGGACGGTGTGACTGTGCCGATGGGGCCTGGCGTGAAGACGGGAGTGGGTAAATGT
AACGCTTACTCCCTCCTCTACAACGAGTTCATCGTTTACAATCCTGCACAGACTCGCATG
AGTTTCACCTCCTCCCCAGGATGTAGAGGAAAATTAGGACTACATCTAGGTAGATTC
>Worm_PARP2
ATGTCTATAATCAACGACGAAAATGGAAGAGGCTATAAAGTACATCTTTGCAAGACGAAT
ATTGCACAGAACAACAATAAATTTTACGATATGGAATTGTTGGATGAGGGAGGTGATTTC
ATTGTGAAGCTTATCAATGGACGTATTGGATATCGTGGTGTCACACAATTAAAAGATTTC
GATGACCTCGACAGAGCCAAGAAGTTCTTCGAAAGCAAATTCTACGAGAAAACTCATCTT
CATTGGGAAGAAAGAGACGATGAACCGGTTCCCAATAAATATGCAGTCGTTGAGTTGGCT
ACTAACGCAAGACAGACTGAAAAAGAAGTTAAAAAGGAGGAACCGGAACCTGAGCCGAAA
GTTGATGAGAAAAATACACGTGGAAGGAAGAAAAGAGGAATTGTTAAAGAAAAGAAAGAA
ATCAAGAAAGAAGAGGAACCAGTTGAAGAAGTTAATGAAAAGCTGAAGGAATTGATGAAA
TGCATCTGCGACGAAGATGTTCATCTGGGACTCTTGAAACAACTGAAATTTAACGAAGCT
TTTGGAAGACCAATTGATTGCCTCTCACTTGCCCAACTCACCACTGGATATGAAATTTTG
AGTAAGATTGAGGAATCGATTGGAGGAAAATCAGCTCGAAGATCTACTCGTGGCCGGCCA
CGAGTAGCTGACCGTGTTCTTGCTGTTAAATCCGATGGTCCATCTCTTCACGACATCAAC
AAATATTATTCTCTCATCCCTCATTCATTTGGTTTCTGTGTTCCTCCAAAAATTGATTCT
CATGCTAAAATTCAAGCTGAACGAGAGCTTCTGGATGCACTGAAAGGATCAATTGAAGCA
TCACTGGAACTGAAGGATTTGAAAAAGACAGCATCGTCGAAGGATATTTATCAAAGACTT
TATGAGCGCCTCCCGTGCCATTTGGAGCCAGTTTCGGAAGAAATTGCAGGAAAAATTGGA
GACTGCTTGGCTATGCGTGGACCCACTCATTGTTACAAGCTTTCACTCATTGATGCATTC
GAGTTAAAGGATCCAAATGAAATTCCAACTGAAGCTCCAGTTGAAGTTCAGGAAGTTCCG
AAGAAGAGAGGACGAAAGAGCACAAAGACCGCTGCTCCAACTGTTCCACCACCAACTACA
AAACGCCTTCTTTGGCACGGTACTCGTGTGACAAATGTCTTCTCAATCCTCATGAATGGA
CTTCAGTTCCCAGTTGGAGATCGCTGTGGTCTGATGTTCGGAAATGGAGTTTATTTCGCA
AATGTTCCAACTAAATCCGCGAATTACTGTTGCCCAGAGGCTTCAAAGAGAGTTTTCATG
CTTCTTTGCGAGGTTGAAACTGCGAACCCATTAGTTCTCTACGAGTCAGAAATTGACGCG
GATGAGAAAATGGAAAAAGCGAAAAAGACGTCGGTTTATGCAGCTGGAAAGCACACTCCA
AGAGACACTGTTGAAATCAATGGAATCCCGGCATTCAAGTCAAATCTTGAGACAATCGAA
GAAGAAACTCGTCTTCTCTACGATGAATATGTGATGTTCAACAAGGAACACTTCAAAATA
AAATATGTTGTCGAAGTGAAAGTGGATCGTCTTACCGCTAAGGAAATGATGGCT
>Zebrafish_PARP2
ATGCGACGAACAAGGAGTTGCAGGGACAAGTCTGTGGCAGATTCAGGCAGTGAAGCTTCA
GAGGCGAAGACGGTTTGGCAGTGGAAAGGGGATGAAGATGAATGGGAACCGTATCCTTCA
GAGGTCTGCGAACAGCTGGATGCAGCAAAGCAGGCTGGTGAAAAGTCTGTTTCGCTTGCG
CTCGGACCTGGTTACGTAGTGGATCTTGTGAAAATGACTCAGACTAATACTGTCAGCAAG
TACAAACGAAAAATACGCCTTCAGAAAGTGAAGTCAGATCTGAATGAAGGGAATGCTTTG
AATGGTAATAAAAAAGCAACAGGTGTTGTTCAGATTAAAGAAGAAAAAGAGGAGGAAGAG
GAGCAGCCCGTTTCCAAGAAGAGACGCAATGAAGGGAGGAGTCAGAAAGCGAACAAAGAC
AAACCTGTGGTAAAAGAAGAATGTGAAGTTGTGAAGACACTGGTTATGAAGGGCAAAGCC
CCTGTTGATCCAGAATGCAAGGCTAAATTGGGAAAGGCACATGTCTACAACGAGGGTGCT

GATGTGTATGATGTTATGCTTAATCAGACTAATCTCCAGTTCAACAATAACAAATATTAT
CTGATCCAACTTCTTGAAGATGACAGTGCAAAAGCCTACAGTGTGTGGTTGAGGTGGGGA
CGAGTGGGCAAAGTAGGACAGAACAATCTGGTTAGCTGTGGGGCAAATCTAGCTCAGGCC
AAAGACACCTTCAAAAAGAAATTTTTTGACAAGACCAAGAACGAGTGGGAGCATCGAGCA
AGTTTTGAGAAAGTTGCAGGAAAATATGACATGGTATTTATGGACTACAGCACTGAAGAC
AAGGGAGAGGAGAAAGCTGTGGTTCCCTCTGCTACTCAAAAAAAGCCTTGCCAACTGAAC
AGCAAGGTCCAGTCTCTCCTAGAGCTCATTTGTGATCTCAAAGCCATGGAGGAGTGTGTG
CTGGAGATGAAGTTTGACACCAAAAAAGCCCCACTCGGAAAACTAACAGCAGAGCAAATT
CGTGCTGGCTATGCCTCACTGAAAAGAATTGAGGAGTGTTTAAAGAAGAAAGGGAGTAAC
AAGGAGCTTTTGGATGCATGCAACCAGTTTTACACCCGCATCCCTCATGATTTTGGGTTG
CGGACACCCCCAATAATTCGCTCAGAGGAGGAGCTGAAGGAGAAAATTACTTTGCTCGAG
ACTCTAAGTGACATTCAGATAGCGGTTAAAATGGTTCAGTCTAATGTTAAGAGTGACGAG
CATCCTTTAGACAGACAGTATCACTCTCTTAACTGCCAACTGCAACCACTGGATACTGAC
AGCAATGAATACAAGGTTATAGAGAAATATCTCAAGTCCACCCATGCACCCACCCATACT
GACTACACAATGACTCTTCTGGATGTCTTTGCTGTGGAACGAGAGGTCGAGAAGGACAAC
TTCAACTCTGAGCTGCAAAACAGGATGCTCCTGTGGCACGGCTCTCGTCTGTCAAACTGG
GTGGGGATCCTGAGTCAGGGTCTGCGAGTGGCCCCTGCAGAGGCTCCTGTGACTGGATAC
ATGTTTGGTAAGGGGATCTATTTCGCAGACATGTCATCCAAAAGTGCCAATTACTGCTTT
GCCAGTCAGAAGAACAACCAGGGACTCTTGTTGCTGAGCGAGGTTGCCCTTGGAGATAGT
AATGAACTTTTGGACGCAGATTACAATGCCGACCAACTGCCTTCTGGGAAGCATAGCACA
AAAGGTCTGGGTCAAACTGCACCAGACCCCAAAAAATCAGTTTCATTGAATGGTGTGACT
GTGCCCTTGGGACCCTCTGTGAAGACCGGTGTTGGCCAGAAAGGAGGATACTCTCTTCTT
TATAATGAGTACATTGTTTATAACCCTGCTCAGATTCAGATGAAGTACCTCCTTAGAGTT
CAGTTTAACTTCTCATCACTTTGG
>central_bearded_dragon_PARP2
ATGGGACGGAAACGCAAGGGCTCCGAAGCCCAGACTGGGGAGCCCACTGCTGCGGGGGCT
GATCTGGAACTGCGCTGGGAGTGGGAGACCGGAGGCAACCGGTGGCAGCAGTTTACGCCT
GCGCAGAGTGAGGTCCTGAGCCAGGCAGCTCGAGCGGGGAAGGTTTCGGCAGACCTCGAG
GATGCCTGCGTGGACCTCCGCAGGATGCTGCGCGAGGACAAACAGACTGGGGAGGAAACC
CGCGTGGCCACCGCTGTCCGAGAGCAGGATTCCTACTTTGTCTGGCAGTGGCAAGGGGAT
CAAGAAGGCGACTGGGTTGCCTACCCGGCGGACACGTGCCTGGCCTTGCAGGCCGCCAGG
AATGGTCAGGGGGAGCCTACGATCCACATGACCGTCGGCCGGACACGCTACAAGCTAGAC
ACGACCCGCATGGTGCAGATCAACAGCCGGACTGGCTTCGAGCGCCAGATGGAAATCAGA
GAGTCAGATGCTGCAGAGGCTGAGGAGGAAGGATCCCAGCGGGGTGACAGTGTCTTGAGT
GCGGAAGCTGCTGTTCCTACGAAGAAGATTCGAAGCAGAGTCGATGCCCCAGTGCCTCAG
GGAGACGGGGATAGCACAGAGACTGTGAAGACGCTGATCGTGAAAGGGAAGGTACCTGTG
GACCCAGAATGCACTGCTAAGCTGGGAAAGGCTCATGTTTATTGTGAAGGAGATGACATT
TATGATGTGATGCTGAATCAGACGAACTTGCAGTTCAACAACAACAAGTACTATCTCATC
CAGTTGCTCGAGGATGATGGAGCACCAAACTACAGCGTATGGATGCGCTGGGGGCGCGTG
GGTAAGCCCGGTCAGCATTCACTCGTGGTTTGTGCTGGAGATTTGGCTAAAGCCAAAGAT
GTCTTCACCAAAAAGTTCCTGGATAAAACCAAGAATGAGTGGCTGAAGCGTGCTAACTTC
CAAAAGGTCCCAGGGAAATACGATATCCTTCATCTGGACTACGAAGCCAATGACGCAGGC
AAGGAAGAAGGTGCGTCCCAGAAGGCAGTCTGTTCCAAGCGGGTGTCACTGCTGGAGCCT
CAGGTGCAGGCCTTGGTGGAGCTGATCTGCAGTATTCGCACCATGGAAGAGATGGTGATG

GAGATGAAGTACGATACCAAGAAGGCCCCTTTAGGGAAGCTGACGGCCGAACAGATCCGC
GCCGGCTACCAGTCTCTGCAAAAGGTGGAGGCGTGTCTCAAGCAGAAGCAGACGGGCCGC
CCCTTGGTAGACGCTTGCAATGAATTCTACACTCGGATCCCCCACGATTTTGGGCTGAAA
ACTCCCCCACTGATCAACACGGATCAGGCGTTGCGAGAGAAGACGCAGCTGCTAGAGGCC
CTCAGCGAGATTCGCATTGGGATCAAAGCAGTGCAGTCAGAACAGCTGGACCAGGAACAT
CCACTTGACCGGAGCTACCGTGGGCTGGGCTGCGAGATCCAACCCTTGGAAAAGGACAGT
CCAGACTTCCAGGTCCTAGAACGCTACCTGTCGTCCACTCATGCTCCCACTCACCGGGAT
TACACAATGACCCTGCTGGAAGTCTTCGTGTTGAACAAACAGAGTGCCAGTCCGGGCTCC
GTCTTCCGCTCCGACCTGCCCAACAGGATGCTGCTATGGCACGGGTCCCGTCTGGGCAAC
TGGGCGGGGATTCTGAGTCAGGGGTTGCGGGTGGCGCCGCCGGAGGCCCCCGTCACGGGA
TACATGTTTGGGAAAGGAATCTATTTTGCCGACATGTCCTCCAAGAGCGCCAACTACTGC
TTCGCGACCCGTGAGAAAGACGTCGGCCTGCTGTTGGTCTCGGAGGTGGCTCTGGGGGCG
TGCAATGAACTGCTGGAGGCGACTCCCGAGGCAGAGACTTTGCCGCCTGGCAAAAACAGT
ACCAAGGGGCTCGGCAAATGGGCTCCATCGCCCGCCAACAGCGTCACACTGCATGGAGCA
GTGGTTCCCATGGGCCCTGCTGTGGAAACTGGGGCGGTGAACCCCCATGGTTACACACTG
AACTACAATGAGTTCATCGTCTATGACCCTTGCCAGGTGCGGATGAAATACCTCCTCAAG
GTGCGCTTCAATTTTACACCGCTCTGG
>spotted_gar_PARP2
ATGGACGTCGGGCGTGTGTGCGGGAGGGGGGCGTGTGCTGTTAAGAATCACTCTGCTCAT
TGCCCTGCTGTGTGCTCTGTGCCCCCAGAGGTGGTGAAGAGGGTGGTGTTCAAGGGGAGG
GCTCCCGTCGACGCTGAGTGCAAGGCCAAACTGGGCAAGGCCCATGTGTACTGCGAGGGA
GAGGATGTCTATGACGTCATGCTGAACCAGACAAACGTCCAGTTCAACAACAACAAGTAC
TACCTGATCCAGCTGCTCCAGGATGACGGGCCGCAGGTCTACAGCGTGTGGATGAGATGG
GGCAGAGGTACTAGCCTGTTCTTCGACAAGACGAAGAACGAGTGGGAGGACCGCTCCCGG
TTTGAGAAAGTGGCGGGGAAATACGACATCGTGCACATGGACTACAGCACCGAGGAGAAG
TTTGTGTTGAGAGCAGCAGGAAAAGCTACATCTTGTATCTCTCATCACATCTTGCTCTGC
TCACTGTCCCTCTCCTTGTCTGTGTTTAGGTTACGCACCCCACCCCTCATTCGCTCTGAG
CAGGAGCTGAAGGAGAAGATCGCTCTGCTGGAGGCCCTCAGTGATATCCAGATTGCGGTG
AAGATGGTCCAGTCCAGTGCGAGTGGTGATGAACACCCCCTGGACAGGCAGTACCACACT
CTGTGCTGTGAGCTGGAGCCCATGGAGCCCAGTTCCCAGGAATACCAGCTGCTGGAGCGC
TACCTGCAGTCCACCCACGCCTCCACTCACAGCGACTACACCATGACCGTGCTGGACATC
TTCACCGTGAACAGGAAGGGGGAGGAAGAGGCCTTCTGCTCACACCTGCACAACAGGACT
CTGCTGTGGCATGGCTCCCGTCTCTCAAACTGGGTCGGGATTCTGAATAAGGGGCTGAGA
GTGGCTCCACCAGAGGCACCAGTCACTGGGTACATGTTTGGAAAAGGAATCTACTTTGCG
GACATGTCATCTAAAAGTGCGAACTACTGTTTTGCCAACCAGAAGAACAGCACAGGACTG
CTGCTGCTCAGTGAGGTCGCCCTGGGGGACTGTAACGAGCTGCTCCATGCCGATTACAAC
GCGGACAAGCTGCCGCCCGGAAAGCACAGTACCAAGGGCTTGGGCCAGACCGCGCCGGAC
CCCAGGAACGCCGTGGCGCTCGACGGGGTCGGCGTGCCCCTGGGCCCGGCCGTCCGGACT
GGGGTGGCCAACGAGCAGGGCTACACCCTGCTCTACAACGAGTTCGTCGTCTACGACCCC
AACCAGGTGCGCATGAGGTTCCTCCTCCGGGTGCGCTTCAACTACCCCTCGCCGTGG
>guppy_PARP2
ATGAGGAGAACCAGAGGTTCCAGAAATAAAAGCCAGAGCGCCGTAGAAAACGAGGAAGTC
CCGACAAAGACAGTGTGGCAGTGGCAAGGAGATGACGGGCAGTGGGAACCGTACCCAGAT
GAAGCGGCCTCCATGCTGGATTCAGCTTCCTCATCAGGGAAGACGTCGGTCACTCTGACT

CTTGGCGCAGGGAAGAAATATGAAGTTGATCTGAAGAAAATGGTCCAGGTTAATCCTACG
ACAAAGTACAAGAGAAAGATTCGCTCTCAGATAGTAAAAGAAGAGAGTTTAAATGAAGCC
GGTGATGTGGCGGCTGAAAATGGAGATTCAGTTCAAGTCAAAGAGGAAGAGGAAGAGGAG
GAGACAGCGGAGCAGCCGGCAGCTAAGAGGAGAAGAGGACAGAGCAAGAGTGCAGCAAAA
ACCAAAGAAATGCCTAAAGAGGAAATAAAAACTGAAGAGGTGGTGCGGACGGTGGTGATG
AAGGGCAAAGCTCCAGTGGACTCCGAATGCAGAGCTAAACTGGGAAAGGCTCATGTTTAC
AGTGAAGGGAATGATGTTTACGATGTGATGCTGAACCAGACCAACCTGCAGTTCAACAAC
AATAAATATTACCTGATCCAGCTGCTGGAGGACGACGGCGCCAAGGCTTACAGCGTCTGG
ATGAGATGGGGCCGGGTGGGTAAAGTGGGTCAGAACAGCCTCACAGTCTGCGGGGCAGAT
CTGCTGAAGGCCAAAGACGTCTTCAAGAAAAAGTTCTTGGATAAGACAAAGAACGAGTGG
GATCGCAGGGAAAGTTTTGAGAAAGTAGCCGGAAAATATGACCTGGTGTTCATGGACTAC
GCCGCTAACGAGAAGGAGGAGAACATGACCACGGTGGACGCCGCCCCCAGAAAGCAGCCC
TCCAAGCTGGACGCGAAGCTCCAGTCGCTCCTCGAACTCATCTGTGACCTCAGAGCCATG
GAGGAGTGTGTGCTGGAGATGAAGTTCGACACCAGAAAAGCTCCTCTTGGAAAGCTGACC
TCGGAGCAGATCCGTGCAGGATACGAAGCCCTGAAGAGGATCGAGTCATGCTTGAAGAAA
AAGGGAAGCAGTAAGGAGTTGCTGGACGCCTGCAACCAGTTTTACACCCGGATCCCGCAT
GACTTTGGGTTAAAAACGCCTCCAATGATTCGCACAGAAGAAGAACTGAAGGAAAAAATT
TCTCTGTTGGAGGCGCTGAGCGACATCCAGATTGCGGTGAAAATGGCCGAGTCCAATGCA
GACAGTGACGAGCATCCTTTGGACAGACAGTATCACGCCCTGCAGTGCCAACTGGAGCCT
GTGGACCAGAGCAGCCACGAGTTCAAGGTGATAGAACGGTACCTGCAGTCCACTCACGCT
CCCACTCACTCTGACTACACCATGAAGGTCCTGGACGTCTTCTCAGTGGACCGGAGCGGC
GAGAGCGACAACTTCCTGTCTCATTTACACAACAGGACTCTGCTGTGGCACGGCTCCCGT
CTGTCCAACTGGGTCGGGATCCTCAGTAAGGGGCTCCGAGTGGCGCCGCCTGAGGCCCCC
GTCACCGGCTACATGTTTGGTAAAGGTATTTACTTCGCAGACATGTCGTCAAAAAGTGCC
AACTACTGTTTTGCCAACCAGAGCCACCATGTTGGACTGCTGCTGCTGAGTGAGGTGGCT
TTAGGGGACAGTAACGAGCTGCTGGACGCCGACTATGAGGCCCACAAGCTGCCTGCAGAA
AAACACAGCACCAAGGGCCTGGGCCTGACCGGACCCGACCCAAAAAACGCCGTCACGCTG
AACGGAGCGACGGTTCCGATGGGTCCCGGCGTTAAAACGGGGGTTTCTAACCCCAATGGC
TACTCTCTGCTCTACAACGAGTTCATCGTTTACAACCCGGCTCAGACCCGGATGAGGTAC
CTGCTGCGGATCCAGTTTAACTTCTCCTCTCTCTGG
>common_starling_SIRT1
ATGTTAGGTACAGACCCACGGACGATTCTGAAGGACCTGCTGCCAGAAACCATCCCCCCC
CCTGAACTGGATGACATGACTCTGTGGCAAATCGTGATAAACATCCTTTCAGAGCCACCA
AAAAGGAAGAAAAGGAAAGATATCAACACCATTGATGATGCTGTGAAACTTTTGCAGGAG
TGCAAGAAGATCATGGTGTTGACTGGAGCTGGGGTGTCAGTGTCTTGTGGAATACCTGAC
TTTAGATCCAGAGATGGCATCTATGCACGCCTTGCTGTAGACTTCCCAGACCTTCCAGAT
CCTCAAGCAATGTTTGATATTGAATACTTCAGGAAGGATCCCAGGCCGTTTTTTAAGTTT
GCAAAGGAAATCTACCCGGGACAGTTCCAGCCGTCTCTCTGTCACAAGTTCATCGCTTTG
ATGGATAAAGAGGGCAAACTCCTTCGCAACTACACTCAGAACATAGACACACTGGAGCAG
GTGGCAGGAATCCAAAGGATAATCCAGTGTCATGGTTCCTTTGCAACAGCTTCCTGCCTG
ATCTGTAAATACAAAGTTGATTGTGAAGTTGTTCGAGGAGATATTTTCAATCAGGTGGTG
CCCCGCTGTCCCCGCTGTCCCCCCGAGGAGCCCCTGGCTATCATGAAGCCCGACATTGTG
TTCTTTGGGGAGAACCTGCCCGAGCAGTTCCACCGTGCCATGAAGTACGACAAAAATGAA
GTGGATCTCCTCATTGTCATTGGGTCTTCGCTCAAAGTAAGACCAGTAGCATTGATCCCA

AGTTCCATCCCCCATGAAGTGCCTCAGATCTTAATTAATAGGGAACCTTTGCCTCATCTA
CACTTTGACGTGGAGCTTCTCGGAGACTGTGACGTTATAATTAATGAATTGTGTCAGAGG
TTGGGTAATGAATACACAAAACTTTGCTACAACTCGGTGAAACTTTCGGAAATCACAGAA
AAGCCTCCACGGCCGCACAAGGAGCTCGAAGCGCTCTCGGCTGAGCTCCCACCAACCCCT
CTGAACATTTCAGAAGGCTCCAGTTCACCAGAAAGGATGAGCCCACCTGATCCTGCAGTG
GTGGCACAACACCCACCTGAATGTAAGGTAGAAAACTGTGAGCCTGCCTCGGAAACTAAA
GGGACCTGCTCCGAGGAGACCCTTCAGGACACGCAGGTGTCATCAGAAAACCCTGAAAAT
CCTGCTAGTGAGCTAATGAACTCTGAAACAATGAAGGAAAATGGATCTAATGATGGAGAA
AATAAAGAAAAGAGTGAAATACTGAAGAAGTGTTGGGTAAACAGATCTGCAAAAGAACAG
ATTAGCAAAAGGCTGGATGGTACTCAGTATCTGTTTCTGCCACCCAGTCGCTATATCTTC
CATGGTGCTGAGGTGTACTCGGATTCTGAAGATGACATCATATCTTCCAGCTCTTGTGGC
AGCAGCAGTGAGAGCGGCTCGTGCCGCAGTCAGAGCTTAGATGTGGAGGATGAGAGTGAG
ATGGAGGAGTTCTACAATGGCTTAGAGGATGAGGATGCTCCCGAGAGGGAAGAGGAGGAT
GGAGCCGAACAGGAGGAATTGGCAGCTGAGGAAGGAGCTGACACAAACGAAGCTGCAGGG
ACGGAAGAGCCGAGCAAGGCGCTG
>central_bearded_dragon_SIRT1
ATGGCGGATGAGGAGACGCCACTCCTCCAGTCCCGCAGCGGCGGCCGCGGCAGCAGCAAC
GGCAGTGTGGCGGCCCCGGTCGCAGAGAGCCCAGAGCCAGCTCTCAAGCGGCCGCGCCGG
GGCCCGACTGACAGCCCCGGGCCGGACTGTGAGACCGCGAGAGGAGCCGCCGGAGAGGCA
GAGACGGCGCCTCCCGCAGGAAGCACCGCCGAGGAGGAGGAGGAGGAAGAGGCCCGGAAG
GTGGAGGAGGTTGCTGCCGCCGCCGCCGCTGTTGTTGCCGCCGCCGCTGCGGAGTCGGGA
GAGGTTTCAGCGGCACCCGGGAGACAAAGCGGCGGCGAAGGCTGGGACTGCGCAGAGAGT
GGGATGTATGTTGACTGTGATAATATATTTTTTAGTGATGAAATCATTGCAAACGGCTTC
CATTCTTGTGAGAGTGATGAAGATGACAGAGCTTCTCATGCAAGCTCTAGTGACTGGACT
CCACGGCCACGTATAGGTCCATATACTTTTGTCCAACAGCATCTCATGATAGGCACAGAT
CCCCGTGCAATTCTGAAAGATTTGTTACCAGAAACAGTTCCTCCCCCTGAACTGGATGAT
ATGACATTATGGCAAATCGTTATTAATATTCTGTCAGAACCACCTAAAAGAAAAAAGAGG
AAAGATATTAATACGTTAGAGGATGCTGTGAAACTCCTACATGAATGCAAAAAAATAATT
GTCTTGACTGGAGCTGGGGTGTCTGTGTCTTGTGGAATTCCTGACTTCCGGTCAAGAGAT
GGTATCTATGCACGCCTTGCTGTGGATTTCCCAGATCTTCCTGATCCTCAAGCAATGTTT
GATATTGAATACTTCAGGAAAGATCCAAGGCCATTTTTTAAATTTGCAAAGGAAATATAT
CCTGGACAGTTTCAACCATCTCTCTGTCATAAATGTATGGCTTTGCTGGATAAAGAAAAA
AAACTGCTTCATAATTATACACAGAACATAGATACAATGGAACAAGTTGCAGGAATTCAA
AGGATAATCCAGTGTCATGGTTCATTTGCAACTGCTTCTTGCCTCATTTGTAAATACAAA
GTTGATTGTGAAGTTGTTCGAGCAGATATTTTTAATCAGGTTGTTCCTAGATGTCCCAGA
TGTCCACCTGATGAACCACTTGCCATCATAAAGCCAGAAATAGTGTTCTTTGGTGAAAAT
CTGCCTGAGCAGTTTCATAGGGCCATGAAATATGACAAAGATGAGGTTGATCTTCTTATT
GTTATTGGGTCTTCACTTAAAGTAAGACCGGTAGCATTAATTCCAAGTTCCATCCCACAT
GAAGTGCCTCAGATTTTAATTAATAGGGAACCCTTGCCTCATCTACACTTCGATGTGGAG
CTTCTTGGTGACTGTGATGTTATTATCAATGAGTTGTGTCATAGATTAGGTGGTGCATAT
GCAAAACTTTGTAACAGTTCAGTCAAACTTTCAGAAATTGCAGAGAAACCTCCACGACCA
CTCAAAGAATTTGAAATACATTCAGTTGAGTTACCACCTACTCCGCTAAACGTTTCTGAA
TATTCTAGTTCACCTGAGAGAATTATCCCACAAGATTCTCAGGTAGTACGTTCAGAGCAT
TCTTCCGAATTTAAAGTAGGAGATTCAGAAGTTGCCTTGGAATCTAAAGAGTACTGTATG

GAGGAAAAGTCACATGAGGTACAAAACTGTTTAGAAAACACTGACAGTCTTCCTGGCCAG
TTAGAAGACCCAGAACATGTGAAGGAACATGGATCTAACCAAGGAGAAAATAAAGATAAA
AGTGAAAAAACATCTGTTGAAGCACTGAGAAAATGTTGGGTTAACAAATGTCCAAAAGAG
CAGATCAGCAAGCGTCTTGATGGTACCCAATACTTGTTTGTACCACCAAATCGGTATATA
TTCCATGGTGCTGAAGTATTCTCGGACTCAGAAGATGATGTCATATCCTCCAGCTCTTGT
GGAAGTAGTAGCGATAGTGGGTCCTGTCATAGTCCAAGTTTAGACATAGAAGATGAGAGT
GAAATTGAAGAATTATATAACGGCATAGAAGAAGATGCACCTGATAGGGAAGATGAGAAT
GGATTTGGGGAAGATGGAATTCAAGACTCTGTGGATGTGTCAGTTCCTGTAAATGAGTCT
GCAGGATTTGACCATTCGGCAGACAAACTG
>Gecko_SIRT1
ATGGCGGACGAGGAGACGCCACTCCTCCAGCCCCGGAGCGGGGGCCGCGGCGGCACCAGC
TGCGTCGCGGCCGGCGCCTCCAAGAGCCCTGAGCCGCCTCTCAAGCGCCAGCGGCGGGAC
AACATATTTCTTAGTGATGAAATTATTGCAAATGGTTTCCATTCCTGTGAGAGTGATGAA
GATGACAGAGCCTCTCATGCAAGCTCTAGCGACTGGACTCCAAGGCCACGTATAGGGCCC
TACACTTTTGTTCAACAACATCTCATGATAGGCACAGATCCACGTGCGATTCTGAAAGAC
TTGTTGCCAGAAACTATTCCTCCCCCTGAACTGGATGATATGACACTGTGGCAAATAGTT
ATAAACATTCTGTCTGAACCACCTAAAAGAAAAAAGCGGAAGGATATTAATACTATAGAG
GATGCTGTGAAACTCCTGCAAGAATGCAAAAAAATTATTGTTTTAACTGGAGCTGGGGTA
TCTGTTTCTTGTGGAATTCCTGACTTCCGATCTAGAGATGGTATCTACGCACGCCTTGCA
GTGGATTTCCCAGATCTTCCGGATCCTCAAGCCATGTTTGACATTGAATATTTCCGAAAA
GACCCAAGGCCATTTTTTAAGTTTGCAAAGGAAATATATCCTGGACAGTTTCAACCATCT
CTCTGTCATAAATTTATAGCGCTGATGGACAAAGAAAGAAAACTGCTTCGCAATTATACT
CAGAATATAGATACACTGGAACAAGTTGCAGGAATTCAAAGGATAATCCAATGTCACGGT
TCCTTTGCAACAGCTTCATGCCTAATCTGTAAATACAAGGTTGATTGTGAAGTTGTTCGT
GAAGACATTTTTAATCAGGTTGTTCCTAGGTGCCCCAGGTGTCCACCTGACGAACCCCTT
GCCATCATGAAGCCAGAAATAGTATTCTTTGGTGAAAATCTACCTGAGCAGTTTCATAGG
GCCATGAAATATGACAAAGACGAAGTTGATCTTCTTATTGTTATTGGGTCGTCTCTCAAA
GTAAGACCAGTAGCATTGATTCCAAGTTCCATCCCCCATGAAGTGCCTCAGATTCTAATT
AATAGGGAACCGTTGCCTCATCTACACTTTGATGTGGAGCTTCTTGGTGACTGTGATGTG
ATTATAAATGAATTATGTCATAGGCTGGGTGGCGAATACACAAAACTTTGTAACAGCTTG
GTCAAACTTTCAGAAATAACAGAGAAACCTCCACGAGTGCACAAGGAACTTGAAATACAC
TCTGCTGAGTTACCACCAACTCCTCTAAACATTTCTGAATATTCTAGTTCACCTGACAGA
ATGGTGCCACAAGATTCTCGGGTGGTACACTCAGAGCCTTCTTTTGAATATAAAGCAGGA
AATTCTGATGCTGCCTTGGTGTCTAAAGAGAACTGTCTAGAAGGGAAAGAACATGACATT
CAAAATTCTCTAGAAAATGCAGAAGGTCTTACTGACCAGTTAGAAAACCCAGAACATGTA
AAAGAAAATGGATCTAACCAAGGTGAAAACAAAGAAAGAATTTTATCTGTTGAAACACTG
AGAAAAAGTTGGATCAACCGATGTGCAAAAGAGCAGATTAGCAAGCGACTTGATGGTACT
CAATACTTGTTTTTACCCCCAAATCGCTATATCTTCCATGGTGCTGAGGTATACTCAGAC
TCTGAAGATGATGTCATATCCTCCAGCTCTTGTGGAAGCAGCAGCGATAGTGGGTCCTGT
CATAGTCCAAGCTTAGACATAGAAGATGAAAGTGAAATTGAAGAATTCTATAATGGCATA
GAAGATGATGATGCTCCTGACAGAGAAGAGGAGAATGGGGAAGATGGAACTGACCTTCAA
GAATCTGTTGATGAGTCAGCTTCTGTAAATGAGGCTGTAGGACCTGATCATCAAACAGAC
AAGCTG
>Taiwan_habu_SIRT1

ATGCCACTCCTCCATTCGCGGAGCGGCGGCCGCCGCGGCAACAACAGCAACGGCGCCGTG
GCGGCCGCTGACACCGAGAGCCCCGAGCCCGCTCTCAAGCGCCAGCGCCAGGCTTCGGCT
GAGCAGAGCTGCGCGGCGGCGGCGGCGGCGGGGGGAAGCGGCGAAGCGGCGGACGCGGCG
CCTCTCCGAGGAAGGGCTGTCGAAGAGGCCCTGGAGGCGGACAGGGCGGTCCCCGCCGCC
TGTGTCAGCGGCGAGCCGGGCGAGGTGGCCGCGCCGGCGGGGGCAGGAGAGGGCTGGAAC
TGCGCGGAAAGCGGGGGCCGGTGGTGGGGCCTGGCCCGAGAGGAGCCGCCGCCGCCGTCG
CCGCACCAACAGCAGCAGCTGCCGCCGCGGGGGCAGAGCCAAGAGGAGGGCGCGGAGGCG
GCGCCCGCCGGAGAGCCAGCGGAGACGGCCATTGGCTGCGGGCCGGCGCAGTCTTCGAAC
GGCGCGGCTGAGGCGGAGAGGGCGCCGCAAACCGATAATATATTTTTTAATGATGAAATT
ATTGCAAATGGCTTCCACTCTTGTGACAGTGATGAAGATGATAGAGCCTCTCATGCAAGT
TCCAGTGATTGGACTCCAAGGCCACGGATAGGTCCATACACTTTTGTCCAACAACATCTC
ATGATAGGCACAGATCCACGGGCAATTCTGAAAGACCTATTACCAGAAACCATTCCTCCT
CCTGAACTGGATAATATGACGTTATGGCAAATTGTCGTTAATATTCTCTCAGAACCACCA
AAAAGGAAAAAGAGAAAAGATATTAATACTATAGAGGATGCTGTGAAACTCCTGCAAGAA
TGCAAAAAAATAATTGTGTTGACTGGAGCTGGGGTATCTGTTTCTTGCGGTATTCCTGAC
TTCCGATCAAGAGATGGTATATATGCACGCCTAGCAGTTGATTTCCCAGATCTTCCTGAT
CCTCAAGCAATGTTTGACATAGAATACTTCAGAAAAGATCCAAGGCCATTTTTCAAATTT
GCAAAGGAAATATATCCTGGACAGTTTCAGCCATCTCTTTGTCATAAATTTATTGCTTTA
ATGGATAAAGAAAGAAAACTCCTTCGCAATTATACTCAGAATATAGATACACTGGAGCAA
GTTGCAGGAATTCAAAGAATAATCCAGTGCCATGGTTCCTTTGCAACTGCTTCTTGCCTC
ATCTGTAAATACAAGGTTGATTGTGAAGTTGTACGAGGAGATATTTTTAATCAGGTTGTT
CCTAGATGTCCCAGATGTTCACCTGATGAACCCCTTGCTATCATGAAGCCAGAAATAGTG
TTCTTTGGTGAAAATCTACCGGAGCAGTTTCACAGGGCCATGAAATATGACAAAGATGAA
GTTGATCTTCTTATTGTTATTGGGTCTTCACTTAAAGTAAGACCAGTAGCATTGATTCCA
AGTTCCATCCCACATGATGTGCCTCAGATTTTGATTAATAGGGAACCGTTGCCTCATCTA
CACTTTGATGTGGAGCTTCTTGGTGATTGTGATGTTATTATCAATGAATTATGTCATAGA
TTGGGTGGAGAATATTCAAAACTTTGTAGTAGTTCCGTTCAGCTTTCAGAAATAACAGAG
AAACCTCCACGGCCTCACAGAGAGTTTGAAAAACATTTAACAGAGTTACCACCCACTCCT
TTAAACATTTCTGAATCTTCTAGTTCATCTGACAGAATTCCATCACAAGATTCTCAGATG
ATACATTCAGAGCATTCTGGTCAATATAAAGCAGGAAATTCTGATGTTGCTTTGGAATCT
AAAGAAAACTCTATAGAAGAAAAACCACTAGAAGTTCACAACTCTTTAGAAAATTCAGAA
AGTACTCATGGTCAGTTAGAAACTACAGAACACATGAAGGAACCTGGATCTCATCAAGAA
GAAAACAAAGAACGAAGTGAAAAAACCTCTGTTGAAACAGTGAGAAAATATTGCTGGAAT
AGATGTGCAAAAGAGCAAGTTAGCAAGCGGCTTGATGGTACTCAATATTTGTTTTCCCCA
CCAAATCGCTATATTTTCCATGGGGCTGAAGTTTATTCAGACTCTGACGACGATGTCATC
TCCTCCAGCTCTTGTGGAAGTAGCAGCGGTAGTGGGTCATGTCTGAGCCGAAGCTTAGAC
GTAGAAGAGGAGAGTGAATTGGAAGACTATTACAATGGCATAGAGGAAGAGGAAGAGGAG
GCAGACACCCCTGACAGAGAAGAGGAGAACGGATTTGAGGAAGAAGGAACGGACCTTCCA
GAATCTGTTGATGAATCAATTTCTATAAATCAGACAGTAGAATTTGACAGTTCGTCAGAC
AAGTTG
>green_sea_turtle_SIRT1
ATGATAGGCACAGACCCACGGACAATTCTGAAGGATTTGCTACCAGAAACAATTCCTCCT
CCTGAACTGGATGATATGACTCTGTGGCAGATTGTTATAAACATTCTTTCAGAACCACCA
AAAAGGAAAAAACGAAAAGATATTAATACTATTGAGGATGCTGTGAAACTTTTACAAGAG

TGCAAAAAAATAATTGTCTTGACTGGAGCTGGGGTGTCTGTTTCTTGTGGAATACCTGAC
TTTCGATCAAGAGATGGCATCTATGCACGCCTTGCAGTAGATTTCCCAGACCTTCCAGAT
CCTCAAGCAATGTTTGATATAGAATACTTCAGAAAGGATCCAAGACCATTTTTTAAGTTT
GCAAAGGAAATATATCCTGGACAGTTCCAACCATCTCTCTGTCATAAGTTCATAGCTTTG
ATGGACAAAGAAGGAAAACTACTTCGCAACTATACTCAGAACATAGACACACTGGAACAG
GTTGCAGGAATCCAAAGGATAATTCAGTGTCATGGTTCCTTTGCAACAGCTTCCTGCCTC
ATCTGTAAATACAAAGTTGACTGTGAAGCTGTTCGAGGAGACATTTTTAATCAGGTTGTT
CCAAGATGTCCCAGGTGTCCACCTGATGAACCACTTGCCATCATGAAGCCAGAGATAGTG
TTCTTTGGAGAAAACTTACCTGAGCAGTTCCATAGGGCCATGAAGTATGACAAAGATGAA
GTTGATCTCCTTATTGTTATTGGGTCTTCACTGAAAGTAAGACCAGTAGCACTGATTCCA
AGTTCCATCCCCCACGAAGTGCCTCAGATCTTAATTAATAGGGAACCTTTGCCTCATCTA
CACTTTGATGTGGAGCTTCTTGGAGACTGTGATGTCATTATTAATGAATTATGTCAAAGG
CTAAGTGGTGAATATACAAAACTTTGCTATAACTCAGTAAAACTTTCAGAAATAACAGAA
AAACCTTCACGAATGCACAAGGAACTTGAAATGCATTCAGCTGAGTTACCACCTACCCCT
TTAAATATTTCTGAAGACTCTAGTTCACCGAACAGAATTACACCGCCAGATCCTTTAGTG
GTACCTTCAGTTGGATGCTCTCAGAGTAAGGCAGAAAATTCTGATCCTGCCTCGGAATCT
AAAGGGAATTGCATGGAGGATAAATTGCAAGAAGCACAAGCATGCTCAGAAAACTCTGAA
AGTATTACTGGCCAGTTAATCAACTCAGAACATATGAAGGATAATAGATCTAACAAGGAG
GAAAATAAAGAGAAAAATGAAATAACTTCATCCGTTGAAACATTGAGGAAATGTTTGGCA
AACAGATTTGCAAAAGAACAAATTAGCAAGCGGCTTGATGGTACTCAATACTTATTTTTA
CCACCAAATCGCTACATTTTCCATGGGGCTGAGGTATACTCAGACTCTGAAGACGATGTC
CTATCATCTAGTTCTTGTGGGAGTAGTAGTGATAGTGGTTCTTGTCATAGTCCAAGCTTA
GATGTAGAAGATGAAAGTGAGATTGAAGAATTCTATAATGGCATGGAGGAGGAGGATGCT
CCAGAGAGAGAAGAGGAGACTGGATTTGGGGAAGATGGAGTGGTTCAAGATGCAGTTGGT
GAACCAGCTTATATAAATGAAGCTGCAGGAATTGATCGTCCATCAAACAAATTG
>Chinese_alligator_SIRT1
ATGTTAGGCACAGACCCAAGGACAATTTTGAAAGATCTGTTACCAGAAACTATTCCTCCA
CCTGAACTAGATGATATGACACTGTGGCAAATTGTTATAAACATTCTTTCAGAACCACCA
AAAAGGAAAAAACGAAAAGATATTAATACCATTGAGGATGCTGTGAAACTTTTGCAAGAG
TGCAAAAAGATTATGGTTTTGACTGGAGCAGGGGTGTCTGTGTCTTGTGGAATACCTGAT
TTCCGATCAAGAGATGGCATCTATGCACGTCTTGCAATAGATTTCCCAGACCTTCCAGAT
CCTCAAGCAATGTTTGATATAGAATACTTCAGAAAGGATCCAAGACCATTTTTTAAGTTT
GCAAAGGAAATATATCCTGGGCAATTCCAGCCATCCCTCTGTCATAAATTCATAGCTTTG
ATGGATAAAGAAGGAAAGCTACTTCGCAACTACACTCAAAACATAGATACACTGGAACAG
GTTGCAGGAATCCAAAGGATAATACAGTGTCATGGTTCTTTTGCTACAGCTTCCTGTCTA
ATCTGTAAATACAAAGTTGACTGTGAAGCTGTTCGAGGAGACATTTTTAATCAGGTTGTT
CCTAGATGTCCCAGATGTCCACCTGATGAACCACTCGCTATTATGAAGCCAGAAATAGTA
TTCTTTGGAGAGAACTTACCAGAGCAGTTCCATAGGGCCATGAAGTATGACAAAGATGAA
GTAGATCTCCTTATTGTAATTGGGTCTTCACTAAAAGTAAGACCAGTAGCACTGATTCCA
AGTTCCATCCCCCATGAAGTGCCTCAGATATTAATTAATAGGGAACCTTTGCCTCATCTA
CACTTTGATGTGGAACTTCTTGGAGACTGTGATGTAATCATTAATGAACTATGTCAAAGG
CTGAGTGGTGAATATACAGAACTTTGCTACAACTCCATAAAACTTTCAGAAATAACAGAA
AAACCACCACGAATGCACAAGGAGCTTGAAATGCATTCAGCTGAGTTACCACCTACCCCT
TTAAACATTTCTGAAGACTCTAGTTCACCTGAAAGAGCAACTCCCCCAGACTCTTTGGTT

GTGCTCTCAGAGCATCCAACAAAATGTAGGGCAGAAAATTCTGAACTTGCCTCAGACTCT
AGTGGGAACTGCATGGAGAAATTCCAGGAGGTACAGACATCCTCAGAAAATCCTGAAAGT
ATTACTGGCCAGTTAACGAATTTAGAACATCCAAAAGAAAATGGAGCTAACAATGGAGAA
CAGAAAGAAAAAAATGATATAACTTCATCGGTTGAAACTTTGAGGAAATGTTGGCCAAAC
AGATGTGCAAAAGAACAGATTAGCAAGCGGCTTGATGGTACTCAATACCTATTTTTACCA
CCAAATCGCTATATTTTCCATGGTGCTGAGGTGTACTCAGATTCTGAAGATGATGTCTTA
TCTTCCAGTTCTTGTGGAAGTAGTAGTGATAGTGGTTCTTGTCATAGTCCAAGTTTAGAT
GTAGAAGATGAAAGTGAGATTGAAGAATTCTACAATGGCATGGAGGAAGAGGATGCTCCA
GAAAGAGAAGAGGAAATTGCATTTGGGGAAGATGGTGTTGAAGAAGATGCAGTTGATGAA
TCAGGTTATATAAATGAAGCTGTAGGAACTGACCATCCATCAAACAAACTG
>African_clawed_frog_SIRT1
ATGGCGGACTCAGATCGTGTAGGTTTTCCCATTGCCGGCACTGTACTAGCTGTCTCAAAA
GAAAATGGCGAGCCCCTTTCTAAAAGGCAGCGCCTGGAAGATACCGGCGGCGGTGGTGGG
CAGCTTGTAGGCGCTGAAAGCGAGGGGAAGGCGGCATTGCCGCCCGTAGCTACTTCTTTG
CAGGAGGAAGGCGAGGCCTCTTCGGCAATGGAAAGCAAATCGAGAGCGCACAATGGCTCG
GGCTTTCAGGGCCTTCCCTTGGAGAAGCGGCAGCTGGGAACATATTTGGTGCAAGGCCAG
GAGGAGGGAGGAGCCGAGGAGTTACCCAATGGAGACCTGTCGGATCAGGCTATTGATTAC
GGAGGGAGTATCCATCTGGATGATGATCTTGCTGGCGGCTTTCATTCTTGTGACAGTGAT
GATGATGGCGCATCTCATGCCAGCTCAAGTGATTGGGCCCCCAGGCCTTGCATAGGGCCT
TATACATTTGTACAACGGCACTTAATGATGGGAACGGATCCCCGGACAATTCTTAAGGAT
TTACTCCCTGACACTGTAGCACCATCAGAGTTAGATGATATGACTTTATGGCAAATTGTT
ATAAACATCCTTTCTGACCCACCAAAACGAAAGAAGCGCAAAGACATCAACACTATTGAT
GATGCTGTGAAACTTTTGCAAGAGAGCAAGAAAATAATTGTTTTAACTGGAGCAGGGGTT
TCAGTGTCTTGTGGAATACCAGATTTTAGATCAAGAGATGGCATTTATGCACGTCTTGCA
GTGGATTTTCCAGACCTTCCAAATCCTCAAGCCATGTTTGATATTGAATACTTCAGGAAA
GATCCAAGGCCGTTTTTTAAATTTGCTAAAGAGATCTTTCCTGGCCAGTTCCAGCCATCG
TTGTGTCACAGATTCATAGCTATGTTGGATAAAGAGGGAAAGCTTCTTAGAAACTATACT
CAGAATATAGACACACTTGAACAAGTTGCAGGAATTGAAAAGATTATCCAGTGTCATGGA
TCATTTGCTGAAGCATCTTGTCTTGTATGTAAATACAAGGTTGACTGTGAAGCTGTTAGG
GAGGACATATTTAATCAGATAGTTCCAAGGTGCCCACAGTGTTCACCTGATGAGCCCCTT
GCCATCATGAAACCGGACATTGTATTTTTCGGGGAAAACTTGCCAGAACAGTTTCACAGA
GCCATGAAATATGACAAAAACGAGGTTGATCTTCTTATCGTTATTGGATCTTCCCTGAAA
GTCAGACCAGTAGCATTAATACCAAGTTCTATCCCACATGAAGTGCCTCAGATACTAATT
AATAGGGAACCATTGCCTCATCTACACTTCGATATAGAACTGCTTGGAGATTGTGATGTA
ATTATAAATGAGTTATGTCAAAGACTGGATGGAAAATACTCTCAGCTTTGTACTAATTTC
TTAAAACTTTCACAAATCACGGAAATGCCTCCTCGAATACACAAAGGTTTCCTTACTTCC
CCAGAGACCATCCCATCTACAGACTTAAACACAGGGCAAAGTGTGGAACTAAAAAGGGAG
CTGCATGAAACTGACTTACTAAGTGCTTCGAATACAGCATGCTCATTGGAGAAACCTAAG
GAAGCTTCTAAGCTTCCACACAGTTGTACTGATGAAAATCTAGAAGTGTTTAAGGAAGTT
AACACACAAAGTCTCAAAGGTGCATCCCAGACTGAAACACAGCCCAGCAATGAGAAAGAT
CAAGAAACAACTGATAAAGATACTGACATTGATTCAACCAAGGATTTGGAAAATAAATAT
ACAAAAGAACAGATCAGTAAGCGCCTAGATAGCACACAGTTTTTGTTTTTAGCACCAAAT
CGCTATATTTTTCATGGTGCAGAAGTGTTTTCGGATTCCGAGGAAGAACTGACATCTAGT
TCCTGTGGAACAAATAGTGACAGTGAATCTTTACTTAGTCCAAGCCTGCATGAACCCATT

GAAGAAGAAAGTGAAACTGAAGAATGCTTCCACGCTAAATATGAAAATGAGACTGATACA
GATAATAGGGCAGATTTAGAGAAAGAACCCGAGAGGGTTGTACTGTATGAAAGTGATGAT
CTCCTAGGTAATGGTACTACAATGAATTTA
>Tibetan_frog_SIRT1
ATGGCGGACGCGGATCGGCTGGGATTGAATCATACCGGCGATCTCTTCGCTTTGCCGAAA
GAAAACGGCGAGCCGGCCGCTAAAAAGCCGCGGATAGACTCCGCCCACGGCGGGCAGCCG
CTGTACGGCGAGAAAGAAGGGAGAGCGGGAGCGTCGGCGGAGCCGGGTGTCCCGGAAGAG
GGGGAGGCCTCTGCGGAGGGAGTAGGCAGAGCGCACAATGATAGCTCGCCCCTTTCGGGC
CTGTTAGGGGAACAGCGGCACCCAGCAATGCAGGCCGAGCAGGAGCTGATTAATGGAGAC
CTGGCGGAGCAGGCAGTCGAGTATGGAGAAGATCTCCATCTAGATGATGATCTTGTTGGC
GGCTTTCATTCATGTGACAGTGATGACGAAGATCGAACTTCCCATGCCAGCTCGAGTGAC
TGGACTCCCAAGCCTTGCATAGGTCCTTACACATTTGTTCAGCGACACCTAATGATGGGT
ACTGACCCTAGGACCATTCTAACGGATCTGCTTCCTGATTCTGTTGCACCACCTGATCTG
GATGACATGACTCTCTGGCAAATTGTGATCAACATACTGTCAGATCCACCGAAAAGGAAA
AAGCGCAAAGACATTAATACTATTGAGGACGCTGTCAGACTCCTGCAAGAGAGCAAGAAA
ATAATGGTGTTAACTGGAGCTGGGGTTTCTGTCTCCTGTGGAATCCCAGATTTTCGATCG
AGGGATGGGATATATGCACGTCTGGCAGTGGATTTCCCTGATCTTCCAAATCCTCAAGCC
ATGTTTGATATTGAATACTTTAGAAAGGACCCAAGGCCATTTTTTAAATTCGCAAAGGAA
ATTTTTCCAGGCCAGTTTCAGCCATCTCTCTGCCACAAGTTTATTGCTATGTTGGATAAA
GAGGGGAAATTACTTAGAAATTATACTCAGAATATAGACACACTGGAACAAGAAGCAGGA
ATTCAAAGAATTATCCAGTGTCATGGGTCATTTGCAATGGCCTCTTGTCTAATATGCAAA
CACAAAGTTGACTGTGAAGCTGTCAGAGAGGACATATTTAATCAGGTTGTTCCACGATGT
CCTAGATGTCCACAGGAAGAGCCCCTTGCAATTATGAAACCTGATATTGTCTTTTTCGGG
GAGAATTTGCCTGAACAATTCCACAGGGCAATGAAATATGACAAAGATGAGGTTGACCTT
CTTATTGTTATTGGCTCCTCGTTGAAAGTGAGACCGGTTGCTTTAATACCAAGCTCCATC
CCTCACGAAGTGCCTCAAATTTTGATTAATAGAGAGCCTTTGCCTCATCTTCATTTTGAT
ATTGAACTTCTTGGAGACTGTGACGTCATAATCAATGAGCTAAGCCAGAGACTAGATGGA
AAGTATGCAGAACTCTGTAACGGTTCCTTGACACTTACAGAAATCACAGAGAAACCTCCC
CGACCAAACAAAGTGCTTTCCATTCTGCTAGATGCACAGTCTAAGGCTTTGGGCACTGGA
CATGGTTTAATCTCCCAAGATGGCCTGGCTCACCCAGAATTACCTTTGACTCCTCCACTT
GAAGACTGCCTGTCATCAGAAAGTGACTCTGAGCTTTCTCAAAGCTGTAAAGGAAATATG
ACTGTAGACAAACCTGTAAGTGAGACCCCAACCAGCAATAGCGAAGAGCCGGAAAAGTGT
GACAATGAGAGCAGTATTGATCTGTCCAGGACGAGTTTGTCCAAAATGGCAAAGGAGCAG
ATAAGCAAACGCCTAGATGGCACACAATATTTATTTCTATCCCCAAATCGCTATATTTTT
CATGGTGCTGAAGTGTTTTCTGATTCTGACGACGACCTGTCATCCAGCTCTTGTGGAACA
AACAGCGACAGTGACTCTTTACAGAGCCCAGGTTTACAGGAAGCGGCTGAAGATGACAGT
GAAATTGAAGATTTCTACCCTATTAAATATGATCATGACTCTGATACTGAAAATCGGACA
GGCTTTGAGGAAGAGCCCGAAGCCGCTGTAATCTATGAGAACAGTGAGATTCTAGGTGAT
GACAATACAGTCACAAAGTTA
>southern_platyfish_SIRT1
ATGGCGGACAGAGAGAACGCTCTCGGAACTGCTTATTCAGGAACCTCTGAGATGGACGAA
CCTATTGCAAAAAGGTCAAAAATCAATACGGTGACCAACCACGGATCTAGAGCCATAGAA
ACGGACACAATCTCATGCGTTTCGCCGGCCATTGGGAGCCGGGAAGAGGCGGGGAATTGT
GCCGAGACAGCGGAGAAGAAGGAAGCGAAGCCGGTGATGATGGCGGTGGAGCAGGCCCTG

GCATCGCGAGGCGGGGACAACAATGGACTGAGTCTGCCGGTCTCCGAGCCGCACAAACTA
GCTGGGCAATTAGGCGACAGCACCGCCTTTGGTGCAACTGAGGAAGGTGCCGATTTTCTT
GGACATAACGATCTGCTTCGCAATGGTCTTGCTGTCACACCAGAGCACATTGATGAGGAA
GATGACAGATCTTCACATGCAAGCTCCAGTGACTGGACTCCTCAACCGCAAATAAATTCA
TACAGTTATATACAGCAGCACATCAGAGAGACGGATCCACGGGCCATTCTGAGGGATCTG
CTTCCTGAAACCATCCTCCCTCCAGACCTGGATGATATGACGTTATGGCAGATAATCATC
AACATTTCTGAGCCTCCAAAAAGAAGAAAACGAAAAGATATCAACACCCTGGAAGACGTG
GTGAAGCTGCTTCATGAAAGCAAAAGGATCCTAGTCCTGACTGGCGCTGGTGTGTCCGTT
TCATGTGGAATACCAGATTTTCGCTCCAGAGATGGAATTTATGCACGACTTGCTGTAGAT
TTTCCTGATCTTCCAGATCCTCAATCAATGTTTGATATTGAATATTTTAGAAGAGACCCT
AGGCCCTTTTTCAAGTTTGCTAAGGAGATCTTCCCTGGTCAGTTCCAGCCGTCGCCCTGT
CACAGATTTATATCCATGCTGGATAAACAAGGGAAGCTGCTTCGCAATTACACTCAAAAC
ATTGATACGTTGGAGCAAGTGGCCGGAGTTCAGCGAATCATCCAGTGCCATGGGTCATTT
GCTACTGCTTCGTGTCTTGTCTGTAAATACAAGGTGGATTGTGAGGCGATAAGGGCTGAC
ATCCTCAATCAGGTCGTCCCCCGTTGCCCCCGGTGTTTGGACATTCCTCTGGCCATCATG
AAACCTGACATCGTGTTTTTTGGAGAGAATCTTCCAGAACTTTTCCACAGAGCCATGAAG
CAGGACAAAGACGAGGTCGACCTTCTGATCGTCATCGGGTCGTCTCTAAAAGTCCGGCCG
GTCGCCCTCATCCCGAACTCCATTCCTCATGAAGTGCCTCAGGTTCTGATCAACAGGGAG
CCGCTGCCTCATCTGAACTTCGATGTGGAGCTGCTGGGCGACTGCGATGGCATCATCAAC
GAACTCTGCCATCGGCTGGGCGGAGACTTCGAGCAGCTCTGCTTCAACACTCTAACCCTT
AACGAGATCACAGAGAAACCACCGCGGTTAGCAGAGCCGCCGCCGCCAGATGAGGCTTCC
ACTGACCCGGCGGACAAACCTCCAGAAGAGACGGAGAGCAAGAATGTCAAGGAAACTGTA
ACGCTGTCCGAGCCTTGTTCGAACGCGGCGAGTCGAAGCGCGGACGACGCCGAGTCGCCG
GAGCCTCAGAGAGAGGACGTAGCGAAAGACGAGGCGGGCGAGGCGAAGAGCCAAACCTCA
GTGCTGGACAATCGAAAGCGATACTGGATGAGTCGGGTCAGCAGGAGTCCAATCAGCAAA
CGGCTTGAAGGCGGTCAGTACCTTTTCCAAGCACCGAATCGATATATCTTCCACGGCGCC
GAGGTCTACTCCGACTCAGAGGAGGACGAGACATCGAGTTCCTGCGGGAGCGACAGCGAC
GGCTCCGACTGCAGCCAGGACGGGCCAGAGGACTGCAGCGAGGCCGAGGACGGCGCCCGG
CCAGCGGGCGACGAGGCGACCCACAGAGACACGTTACCACTAAACGAAGAGGCCACGCCC
ACTCTGCAGACGGACTTCACATCAGAACACTCTGAGAGGACCACACATCTC
>spotted_gar_SIRT1
ATGGCGGACGGAGAGAATATCCTCCTAAACTCCAGCGCCAGTGGCTTCGAAACAGATGAA
CCTCTGCCTAAAAAGCCGAAGGTGGGATTGGCGTTTAGTTACGGGGATAAGGCCGCTCAG
ATCGATCAGGAGAGTGGGATCGGGGCCGGCGAGACTGGGGCGGCGGCTGCCGTAGCACCA
GCAGCTGCAGCGGTGTCGGCGGCGGCGGCGACGACACAGCGGCCGGGGTTGGAGGAGGTG
GTGACGGTGGGACAGGCCTCGGCTGCACCAGGAGGAGACAATGGGCTTCTGGGCTTGGAG
CTGCAGGAGGGGCTGGGGGAGGAGGGAGAGAATGATCCGGTCGGCGGTTCTGCTGCAGAG
CGACCAGAGTACATTGATCTCAATGATGAGATTCTGCCCAATGGATTCCATTCCTACGAT
TGCGATGACGATGATGACAGATCTTCGCACGCAAGTTCTAGTGACTGGACACCTCAGCCC
CAGATAGGCTCCTACAGTTTTATTCAGCAACACATTATGAGAGAAACAGACCCTCGAACG
ATTTTAAAAGACCTTCTCCCAGAAACTGTTCTTCCTCCTGATCTGGATGATATGACACTG
TGGCAAATAATAATCAACATATCGGAGCCTCCAAAAAGAAAGAAACGCAAAGACATCAAC
ACCATGGAAGATGTGGTCAGGTTACTGCAAGAATGTAAAAAGATTATAGTTTTGACCGGA
GCTGGGGTCTCGGTGTCCTGTGGAATACCTGACTTTAGATCCCGTGATGGCATTTACGCG

CGGCTTGCGATTGACTTCCCCGATCTTCCTGATCCTCAGGCGATGTTTGACATAGAATAC
TTCAGGAGAGATCCGCGACCCTTCTTTAAGTTTGCCAAGGAGATCTATCCAGGACAGTTC
CAGCCGTCGCCATGTCATAAGTTCATTGCAATGTTGGATAAGCAGGGGAAGCTTCTTCGA
AACTATACTCAGAATATCGACACACTGGAGCAAGTGGCTGGAGTACAAAGGATCATACAA
TGTCATGGGTCTTTTGCAACAGCGTCTTGTTTAATCTGTAAATACAAAGTTGACTGTGAA
GCTATTAGAGAAGACATTTTTAATCAGGTTGTACCTCATTGCCCCAAGTGTCCACCTGAT
TTTCCTCTTGCCATCATGAAACCAGACATAGTTTTCTTTGGAGAGAACTTACCAGAACAG
TTTCACAGAGGCATGAAACATGACAAAGATGAAGTGGACTTGCTGATTGTCATTGGATCT
TCACTGAAAGTGCGACCAGTGGCTCTTATTCCAAGCTCAATTCCTCATGAAGTGCCTCAG
ATTCTGATCAACAGAGAACAGCTGCCCCATCTGAACTTTGATGTGGAATTGCTTGGAGAC
TGTGATGTTATCGTTAATGAACTATGCCATAGGCTAGGTGGAGACTATGAACAACTGTGC
TACAACCCTTTAAGACTTTCAGAGATTACAGAGAAACCCCCAAGGCCACAGAGGGAGTCT
GAACTACAGTTCTGCGACCTGCCACCCACCCCCCTCGAGCTCACTGAAGACTCTGGTTCC
TGGGAACAGACAAGCGAGCCCACCCACAACTCTGTGGGAACTTTGGAAGAATTGACTGGA
GATCGGTTATCGAACAAAGGAGAGCCTACCTCTGAGCTTGCAGTGGACAACGGGATGGAA
AACTCCTCAAAAAGCAGGACTGGGTCTTCTGGGGACAGTGGAAAAACTGAACCGAATGAG
TTGCAGACTTCAAAGACTGAAGATGCCCCTGGATTGGGAGAGAAAAATGCAGATGTAGAA
ATTCGCAAAAGATACTGGATGAGCAGATTTGGCAGAGAGCCAGTCAGCAAACGCCTCGAC
AATTCACAATACCTGTTCCAGGCACCAAATCACTACATTTTCCAAGGAGCAGAAGTCTAC
TCCAATTCCGAAGATGAGTCTTCGAGTTCCTGTGGAAGTCACAGCGAGGAGTCATACTGC
AGCCCAGGAGAGATGGGGGAGGACGACAGCGATGGTGAAGAGCTCTATGGCCGATTAGAA
GAGGCCGAGGATGGAGAAGAGTGTTTGAGGAGCAATGGAGAACAGCTAGAAAAGCAAACC
AATGACCGGGAAATCCTGAAAGACAGTATAGCTGAGGATGTTACAAATATGACACCGATG

>guppy_SIRT1
ATGGCGGACAGAGAGAACGCTCTCGGAACGGCTTGTTCAGGCACCTCTGAGATGGACGAA
CCTATCGCAAAAAGGTCAAAAATCTATCCGGTGACCAACCACGGACCTAGAGCCATAGAA
ACGGACACAATCTCCTGTGTTTCGGCGGCCATTGGGAGCCTGGAAGAGGCGGGGAATTGT
GCCGAGCCAGCGGAGAAGAAGGAAGCGAAGCCGGTGATGATGGCGGTGGAGCAGGCCCCT
GCATCGCGAGGCGGGGACAACAATGGACTGAGTCTGCCGGTCTCCGAGCCGCTTAAATCA
TCTGGGAAATTAGGCGACAGCACCGCCTTTGGTGCAAGTGAGGAAGGTGCCGATTTTCTT
GGACATGACGATCTGCTTCGCAATGGTCTTGCTGTCACACCAGAGCACATTGATGAGGAA
GATGACAGATCTTCACATGCAAGCTCCAGTGACTGGACTCCTCAACCGCAAATAAGTTCG
TACAGTTACATCCAGCAGCACATCAGAGAGACGGATCCACGGGCCATTCTGAGGGATCTG
CTTCCTGAAACCATCCTCCCCCCAGACCTGGATGATATGACGTTATGGCAGATAATCATC
AACATCTCTGAGCCTCCAAAAAGAAGAAAACGAAAGGATATCAACACCCTGGAAGATGTG
GTGAAGCTGCTTCATGAAAGCAAAAGGATCCTAGTCCTGACTGGCGCTGGTGTGTCCGTT
TCATGTGGAATACCAGACTTTCGCTCCAGAGATGGAATTTATGCACGACTTGCTGTAGAT
TTTCCTGATCTTCCAGATCCTCAATCAATGTTTGATATTGAATATTTCAGAAGAGACCCT
AGACCCTTTTTCAAGTTTGCTAAGGAGATCTTCCCCGGTCAGTTCCAGCCGTCGCCCTGT
CACAGATTTATATCCATGCTGGATAAACAAGGGAAGCTGCTTCGCAATTACACTCAAAAC
ATTGATACGTTGGAGCAAGTGGCCGGAGTCCAGCGAATCATCCAGTGCCATGGATCATTT
GCTACTGCATCGTGTCTTATCTGTAAACACAAGGTGGATTGTGAGGCGATAAGGGCTGAC
ATCCTCAATCAGGTCGTCCCCCGTTGCCCCCGGTGTTCGGACATTCCTCTGGCCATCATG

AAACCTGACATCGTGTTTTTTGGAGAGAATCTTCCAGAACTTTTCCACAGAGCCATGAAG
CAGGACAAAGACGAGGTCGACCTTCTGATCGTCATCGGCTCGTCTCTAAAAGTCCGGCCG
GTCGCCCTCATTCCGAACTCCATTCCTCATGAAGTGCCTCAGGTTCTGATCAACAGGGAG
CCGCTGCCTCATCTGAACTTCGACGTGGAGCTGCTGGGCGACTGCGACGGCATCATCAAC
GAACTCTGCCACCGGCTGGGCGGAGACTTCGAGCAGCTGCGCTTCAACACTCTAACCCTC
AACGAAATCACAGAGAAACCGCCGCGGTTAGCAGAGCCGCCGCCGCCCGACGAGGCTTCC
ACTGACCCGGCGGACAAACCTCCAGAAGAGACGGAGAGCGAGAATGTCACGGAAACCGTA
ACGCCGTCGGAGCCTTGTTCGAACGCGGCGAGTCGAAGCGCGGACGCCGCCGAGTCGCCG
GAGCCTCAGAGAGAGGATGCGGCGAAAGACGAGGCAGGCGAGGCGAAGAGCCAAATCTCA
AAGCCGGACAATCGAAAGCGATACTGGATGAGTCGGGTCAGCAGGAGTCCAATCAGCAAA
CGGCTTGAAGGCTGTCAGTACCTTTTCCAAGCACCGAATCGATACATCTTCCACGGCGCC
GAGGTCTACTCCGACTCCGAGGAGGACGAGACGTCGAGCTCCTGCGGGAGCGACAGCGAC
GGCTCCGACTGCAGCCAGGACGGGCCGGAGGACTGCAGCGAGGCCGAGGACGGCGCCCGG
CCCGCGGGCGACGAGGCGATCCACAGAGACACGTTACCGCTAAACGAAGAGGCCACGCCC
ACTCTGCAGACAGACTTCACATCAGAACACTCTGAGAGGACCACACATCTT
>American_alligator_SIRT1
ATGACTGCGGCGGACAACCTCCTTTTTAGTGATGAGGTCATAGCCAATGGTTTCCATTCT
TGTGATAGTGATGAAGATGACAGAGCCTCGCATGCAAGTTCTAGTGACTGGACTCCAAGA
CCACGGATAGGTCCCTACACTTTTGTTCAACAGCATCTTATGTTAGGCACAGACCCAAGG
ACAATTTTGAAAGATCTGTTACCAGAAACTATTCCTCCACCTGAACTAGATGATATGACA
CTGTGGCAAATTGTTATAAACATTCTTTCAGAACCACCAAAAAGGAAAAAACGAAAAGAT
ATTAATACCATTGAGGATGCTGTGAAACTTTTGCAAGAGTGCAAAAAGATTATGGTTTTG
ACTGGAGCAGGGGTGTCTGTGTCTTGTGGAATACCTGATTTCCGATCAAGAGATGGCATC
TATGCACGTCTTGCAATAGATTTCCCAGACCTTCCAGATCCTCAAGCAATGTTTGATATA
GAATACTTCAGAAAGGATCCAAGACCATTTTTTAAGTTTGCAAAGGAAATATATCCTGGG
CAATTCCAGCCATCCCTCTGTCATAAATTCATAGCTTTGATGGATAAAGAAGGAAAGCTA
CTTCGCAACTACACTCAAAACATAGATACACTGGAACAGGTTGCAGGAATCCAAAGGATA
ATACAGTGTCATGGTTCTTTTGCTACAGCTTCCTGTCTAATCTGTAAATACAAAGTTGAC
TGTGAAGCTGTTCGAGGAGACATTTTTAATCAGGTTGTTCCTAGATGTCCCAGATGTCCA
CCCGATGAACCACTCGCTATTATGAAGCCAGAAATAGTATTCTTTGGAGAGAACTTACCA
GAGCAGTTCCATAGGGCCATGAAGTATGACAAAGATGAAGTAGATCTCCTTATTGTAATT
GGGTCTTCACTAAAAGTAAGACCAGTAGCACTGATTCCAAGTTCTATCCCCCATGAAGTG
CCTCAGATATTAATTAATAGGGAACCTTTGCCTCATCTACACTTTGATGTGGAACTTCTT
GGAGACTGTGATGTAATCATTAATGAACTATGTCAAAGGCTGAGTAGTGAATATACAGAA
CTTTGCTACAACTCCATAAAACTTTCAGAAATAACAGAAAAACCACCACGAATGCACAAG
GAGCTTGAAATGAATTCAGCTGAGTTACCACCTACCCCTTTAAACGTTTCTGAAGACTCT
AGTTCACCTGAAAGAGCAACTCCCCCAGACTCTTTGGTTGTGCTCTCAGAGCATCCAACA
AAATGTAGGGCAGAAAATTCTGAACTTGCCTCAGACTCTAGTAGGAACTGCGTGGAGAAA
TTCCAGGAGGTACAGACATCCTCAGAAAATCCTGAAAGTATTACTGGCCAGTTAACGAAT
TTAGAACATCCAAAAGAAAATGGAGCTAACAATGGAGAACAGAAAGAAAAAAATGATATA
ACTTCATCAGTTGAAACTTTGAGGAAGTGTTGGCCAAACAGATGTGCAAAAGAACAGATT
AGCAAGCGGCTTGATGTGCTAGCCTGTGCCATAATGGATACATCCTACAACAATGTC
>Armadillo_SIRT1
ATGGCGGACGAGGCGGCGCTCGCCCTTCAGCCCGGCGGGTCCCCCTCTGTGGCGGCGGCC

GAGAGGGAGGCCGGGTCGCCGCCAGTAGGGGAGCCGCTCCGGAAGAGGCTGAGGAGAGAC
GGCCCTGGCTTCGGGCGGACACCGGGCGAGCCCGGAGGGGCGGCCCCAGAGCGTGAGATG
CCGGCGGTGGCGGCGCTGTGGCGGGAGGTGCCGGCCGCGGGCGGGGAACGGGAGGCCCAG
GAGGCCGGGGCAACAGGAGGAGACAATGGGCCGGGCCTGCAGGCCCTATCCCGGGAGCTG
CCGCCGGTCGACGACTTCTACGAAGACGACGACGACGAAGGCGAGGAAGAGGAAGAGGCG
GCGGCGGCGGCGATTGGGTACCGAGATAACCTTCTGTTCGGTGATGAAATTATCACCAAT
GGCTTTCATTCCTGTGAAAGTGATGAGGATGATAGAGCCTCACATGCAAGCTCTAGTGAC
TGGACTCCAAGGCCACGGATAGGTCCATATACTTTTGTTCAGCAACATCTCATGATTGGC
ACAGACCCTCGAACAATTCTTAAAGATTTACTACCAGAAACAATTCCTCCACCTGACTTG
GATGATATGACATTGTGGCAGATCGTTATTAATATCCTTTCAGAACCACCAAAAAGGAAA
AAAAGAAAAGATATTAATACAATTGAAGATGCTGTGAAATTACTGCAAGAGTGCAAAAAA
ATAATTGTTCTAACAGGAGCTGGGGTGTCTGTTTCCTGTGGAATACCTGACTTCAGGTCG
AGAGATGGTATTTATGCACGCCTTGCAATAGACTTCTCTACCTTCTCAGATCCTCAAGCA
ATGTTTGATATTGAATATTTCAGAAAGGATCTCAGACTCATTTTCAAGTTTGCAAAGGAA
ATATATCCCGGACAGTTCCAACCATCTCTTTGTCACAAATTCATAGCCTTGTCAGATAAG
GAAGGAAAACTACTTCGCAACTATACTCAGAACATAGATACACTGGAACAGGTTGCAGGA
ATCCAAAGGATAATTCAGTGTCACGGTTCTTTTGCAACAGCATCTTGCCTGATTTGTAAA
TATAAAGTTGACTGTGAAGCTGTACGAGGAGATATTTTTGATCAGGTGGTTCCTCGATGT
CCTAGGTGCCCAGCTGATGAACCACTGGCTATTATGAAACCAGAAATTGTCTTTTTTGGT
GAAAATTTACCAGAACAGTTTCACAGAGCCATGAAGTATGACAAAGATGAAGTCGATCTC
CTCATTGTTATTGGGTCTTCCCTGAAAGTACGACCAGTAGCACTAATTCCAAGTTCCATA
CCCCATGAAGTGCCTCAGATATTAATTAATAGGGAACCTTTGCCTCATCTGCATTTTGAT
GTGGAGCTTCTTGGAGACTGTGATGTCATAATTAATGAGTTGTGTCATAGGTTAGGTGGT
GAATACGCCAAACTTTGCTGCAACCCTGTAAAGCTTTCAGAAATTACTGAAAAACCTCCA
CGAACACAAAAAGAGTTGGCTCATTTGTCAGAGTTGCCACCCACACCTCTTAATATTTCA
GAAGACTCAAGTTCACCAGAAAGAACTTCACCTGATTCTTCAGTGATTGTCACACTTTTA
GATCAAGGTACCAAGAATAAAGTTGATGATTTAGATGTGTTGGAATCAAAAGGTTGTAAT
GAAGAAAAATCACAGGTAATACAGACTTCTACTAGGAACACTGAAAATATTCCTGAACAG
TTGGAGAGTCCGGATTTGAAGAACGTTGGCTCCATTACTGGGGAGAAAAATGAAAGAACT
TCAGTTGCCGAAACAGTGAGAAAATGCTGGCCTACTAGACTTGCAAAGGAGCAGATCAGT
AAGCGGCTTGATGGTAATCAGTATCTGTTTTTACCACCGAATCGTTACATTTTCCATGGC
GCTGAGGTATATTCAGACTCAGAAGATGATGTCTTATCCTCTAGTTCTTGTGGCAGTAAT
AGTGATAGTGGAACTTGCCACAGTCCAAGTTTAGAAGAACACATGGAGGATGAAAGTGAG
ATTGAAGAATTCTACAATGGGTTGGAGGATGATGCTGATGTTCCAGAGCGAGGTGGAGTA
ACTGGCTTTGGGGCTGATGGAGGTGACCAAGAGGCAGTTAATGAAGCTACATCTGTGAAA
CAGGAAGCAGTGGACATTAATTATCCATCAAACAAATCA
>Chicken_SIRT1
ATGGCGGACGGGGAGGCTCCGCTCCTCCGGCCCCGCGACGGCGGCCCCGGCGCCGCGGCC
GAGAGCGTCGAGCCCGCGCCCAAGCGCCAGCGCCTGAACTCGGAAGACGGCGTCTGCGGC
CGGGGCGCGCCGCCCGCCCACCGCCCGGACCGGGGCGCGGGGCCGCCTCCCGCCGCCGCC
GCCGCGACGGAACCTCCGGGCGATGCGGCGGCCGTCAGCGCCGACGGCGACGTCCGGGCG
CGGGAGGAGGACGGCGGCGCGACGACGGAGGGCCGGAGCGGCGCGGACAATAGGGCCGCG
CAGCGGGGCCTGGCCCGGGCGGAGCCGCCTCCGCAGCCGCGGCGGCAGGGCCGGGGGGAG
GGGGCGGAGGCGGCGCCCGGCGAAGACGCGGCGGAGGCGGCCATTGGCTGCGAGCGGGCG

CAGCGTTCAAACGGGGCGGCCGGAGCGCCGGCCCCGCAGCCCGATAACTTCCTTTTAAGT
GATGAAATCATAGCCAATGGTTTCCACTCCTGTGATAGTGATGAAGATGACAGAGCTTCA
CATGCAAGTTCTAGTGACTGGACCCCAAGACCACGTATAGGTCCTTATACTTTTGTTCAA
CAGCATCTCATGTTAGGCACAGACCCACGGACAATTCTTAAAGACCTGCTCCCAGAAACG
ATCCCTCCACCTGAACTGGATGATATGACACTGTGGCAAATTGTCATAAACATTCTTTCC
GAACCACCAAAAAGAAAAAAACGAAAAGACGTTAATACTATTGATGATGCAGTGAAACTT
TTACAGGAGTGCAAAAAAATCATGGTCTTGACAGGAGCCGGGGTGTCTGTGTCTTGTGGA
ATACCCGACTTTAGATCAAGAGACGGCATCTATGCACGCCTTGCTGTAGACTTCCCAGAC
CTTCCAGATCCTCAAGCAATGTTTGATATAGAATACTTCAGAAAGGATCCCAGGCCATTT
TTTAAGTTTGCAAAGGAAATCTACCCAGGCCAGTTCCAGCCATCTCTCTGCCACAAGTTC
ATAGCTTTGATGGATAAAGAAGGAAAATTGCTTCGTAACTATACTCAGAACATAGACACA
TTGGAACAGGTTGCAGGAATCCAAAGGATAATTCAGTGTCATGGTTCCTTTGCAACAGCT
TCCTGCCTGATCTGTAAATACAAAGTTGATTGTGAAGTTGTTCGAGGAGATATTTTCAAT
CAGGTTGTTCCTAGATGTCCTCGCTGTCTGCCTGATGAACCACTTGCTATCATGAAGCCT
GACATAGTGTTCTTTGGAGAGAACTTACCTGAGCAGTTCCATCGCGCCATGAAGTATGAC
AAAAATGAAGTTGATCTCCTTATTGTCATTGGGTCTTCACTGAAAGTAAGACCGGTAGCA
TTGATTCCAAGTTCCATCCCCCATGAAGTGCCTCAGATCTTAATTAATAGGGAACCTCTG
CCTCATCTACACTTTGACGTGGAGCTTCTTGGAGACTGTGACGTTATTATTAGTGAATTA
TGTCAAAGGCTAGGTAGTGAATATACAAAACTGTGCTACAACTCAGTAAAACTTTCAGAA
ATAACAGAAAAGCCTCCACGAATGCACAAAGAGCTCGAAATGCACTCATCTGAGCTGCCA
CCTACACCCTTAGACATTTCAGAAGACTCTGGTTCACCAGAACAAATGACTCCACCGGGT
ACTTCGGTGGTGCCCTCAGAACACGCAGCTGAATGTAAGGTAGAAAACTCTGATCCTGCC
TCTGAAACTAAAGGGATCTGCACAGAGGAAAAGCTTCAAGACACACAGGCATCCTCTGAA
AACCCTGAAAATCCTGCTAGTGAATTAATGAACTCTGAAACGATGAAGGAAAATGGAACC
AACAATGGAGAAAGCAAAGAAAAAAATGAAATAGTGAAGAAGTGCTGGGTAAACAGATCT
GCAAAAGAACAGATCAGCAAAAGGCTGGATGGTACTCAGTATCTGTTTTTACCACCAAAT
CGCTATATTTTCCATGGAGCTGAGGTATACTCAGATTCTGAAGATGATATGATATCTTCT
AGCTCTTGTGGGAGTAGTAGCGAAAGCGGCTCGTGTCACAGTCAGAGCTTAGATGTGGAG
GATGAGAGCGAGATCGAAGAGTTTTACAATGGCATAGAAGATGAGGATGCTCCAGAAAGG
GAAGTGGAAGCTGCATTTGAGGAGGATGGAGTTGAACAAGATGCAGCTGATGAATCAGCT
TACACAAATGAAGCTGCAGGGAACGATCATCCAACAAGCAACAAGTTG
>Chinese_softshell_turtle_SIRT1
ATGATAGGCACAGACCCCCGGGCAATTCTGAAAGACTTGCTACCAGAAACAATCCCCCCA
CCTGAATTGGATGATATGACTCTGTGGCAGATTGTTATAAACATCCTTTCAGAACCACCA
AAAAGGAAAAAACGAAAAGATATTAATACTATTGAAGATGCTGTGAAACTTTTACAAGAG
TGCAAAAAGATAATTGTTTTGACTGGAGCTGGGGTGTCTGTTTCTTGTGGAATACCTGAC
TTCCGATCAAGAGATGGCATATACGCCCGCCTTGCAGTAGACTTCCCAGACCTTCCAGAT
CCTCAAGCAATGTTTGATATAGAATACTTCCGAAAGGATCCAAGACCATTTTTTAAGTTT
GCAAAGGAAATATATCCTGGTCAGTTTCAGCCATCTCTCTGTCATAAATTCATAGCTTTA
ATGGACACAGAAGGAAAGCTACTTCGCAACTATACTCAGAACATAGATACGCTGGAACAG
GTTGCAGGAATCCAGAGGATAATTCAATGTCATGGTTCCTTTGCAACAGCTTCCTGCCTA
ATCTGTAAATACAGAGTTGACTGTGAAGCTGTTCGAGGAGACATTTTTAATCAGGTTGTT
CCTAGATGCCCCAGGTGTCCACCTGATGAACCACTTGCCATCATGAAGCCAGAGATAGTG
TTCTTTGGAGAAAATTTACCTGAGCAGTTCCATAGGGCCATGAAGTATGACAAAGATGAA

GTTGATCTCCTTATAGTTATTGGGTCTTCACTGAAAGTAAGACCAGTAGCACTGATCCCA
AGTTCCATCCCCCATGAAGTGCCTCAGATCTTAATTAATAGGGAACCTTTGCCTCATCTA
CACTTTGATGTGGAGCTTCTAGGGGACTGTGATGTCATTATTAATGAGTTATGTCAAAGG
CTAGGTGATGAATATACAAAACTTTGCTGTAACTCAGTAAAACTTTCAGAAATAACAGAA
AAGCCCTCACGAATGTATAGGGAACTTGAAATGAATTCAGCTGAGTTACCACCTACCCCT
TTAAACATTTCTGAAGACTGCAGTTCACCAAACAGAATGACACCACCTGGTCCTTTAATG
GTACTCTCGGAGAATCCAGCTGAATGGAAGGCAGAACATTCTAATCCTGCCTCAGAATTT
AAAGGCAATCATCAGGAGAAAGAATCGCAAGAAGTACAAACATGCTCAGAAAACACAGAA
TGTATTACTGGCCAGTTAATGAACTCAGAACATATGAAGGATAATAGCTCTAACAAGGAA
GAAAATAAAGAGAAAAATGAAATAACTTCATCTGTTGAAACATTGAGGAAATGTGCAAAT
AGATTTGCAAAAGAACAAATTAGCAAGCGGCTTGATGATACTCAGTACTTATTTTTACCA
CCAAATCGCTACATTTTCCATGGTGCTGAGGTATACTCAGACTCCGAAGACGATGTCATA
TCTTCTAGTTCTTGCGGGAGTAGTAGTGATAGTGGTTCTTGTCATAGTCCAAGCTTAGAT
GTAGAAGACGAAAGTGAGATTGAAGAATTCTATAATGGCATGGAGGAGGAGGAGGCTCCA
GAGAGAGAAGAGGAGACTGGATTTGGGGAAGATGGAGTTGAACAAGATACAGTTGATGAA
CCAGATCAGAAAAATGAAGCAACAGGAATTGATTGTCCATCAAATTGTAATCAT
>Cod_SIRT1
atggcggacggagagaacattctcggcagcgcctgcgctagcgccgctgatacggaggag
cccgctgcaaaaaagtccaaaatcaacatgatttccaactatggattcaaagtagccgag
accgagcacatatcctgcgtgttggaggcgacaggcagctgggaggcgatggtgaattcc
gtgcagcagcagccgccgccgccagcagagaaggaggttgagccggtgatggcggtagag
caggccccagtagcactaggcggagacaatggggtcgccctgctgatcctggagccggac
agaacagacgtgaaaccagacgagaaccatgcgctcggaatcgtcgccgagaccccagat
gttcttggtcttccctccaacgggttgggcgactcccccgaagacgatgacaggtcttca
cacgcaagttccagcgactggacacctcaacctcaaataggttcctacagtttcattcag
caacacataatgagagagacggatcctaggaccattctgaaggacctgcttccagagacc
acgctcccgccagacctggatgacatgacgttgtggcagatcatcataaacatctcggag
ccgccgaaaagaaagaagcggaaggatattaacacgctagaggacgtggctaggctcctc
catgaaagtaaaaggatccttgtgttgaccggtgctggggtgtctgtgtcgtgtggaatt
ccagacttccgatcccgcgatggtatttatgcaaggcttgctgtggacttccctgatctt
ccagatcctcaatccatgtttgacattgaatactttcgaagagatccgcgacccttcttt
aagtttgccaaggagatctaccccggtcagttccagccatcaccctgtcacaaattcata
tccatgctggataagaaggagaagcttttgcgcaattacacccagaacattgacacactg
gaacaagtggcaggtgtacaaaggattattcagtgccatggttcatttgcaacggcgtcc
tgtctggtctgcaaacactcggttgactgtgaggctatccgggaagacatcttcaaccag
gtggttcctcggtgtccacattgtgcagatatccccttggcaatcatgaaaccagacatc
gtcttcttcggagagaatcttccagaattgttccacagagccatgaaacaagacaaagac
gaagtggacctgttgatagtcatcggctcctcacttaaagtgagaccggtcgctcttatc
ccaaactccatccctcacgacgtgccacagatcctcatcaaccgggagcagctcccccac
ctgaacttcgacgtggagctgctgggcgactgtgacgtcatcgtcaacgagctctgccac
cggctgggcggcgacttcgagcagctgtgcgaccagaccttaagactcagtgagatcaca
gagaagccgccgcggttaccggacgacgacgatgacgccgccgccgccgtggagaccgtg
acgacgttgacgacctccgaaccggacgagacgccgcaggaggagacggctcggggcgcc
gcacccgcgccgtccacgccttcagaggagacggcagggggaacggtcgccgaagcctca

gtccccgccgctgaccacgtgcctccagagccgtgtccggcggcggcagaacaacccctc
cagaacgccgccgccacgaccgcggagtccccggaggacgagacggaggacacgccaaag
gaagaggaagcagagcccagacgccgcagctcaaacctggatcttcgcaggagatgctgg
atgagtcgtctcggcaggagcccgatcagcaagcgccttgagagtaagcctgggactgtg
acagggccgctcggttatggaagaaatcgtattcacgatcattttggtcaatat
>Cow_SIRT1
ATGGCGGACGAGGCGGCGCTCGCCCTTCAGCCCGGCGGCTCTCCCTCGGTAGTGGCGGCT
GAGAGGGAGGCCCCGTCGCCGCCTGCTGGGGAGCCGCTCCGCAAGAGGCCGCGAAGAGAC
GGCCCCGGCGTCGGGCGGAGCTCGGGCGAGCCCGGTGGGACGGCCCCCGAGCGGGAACTG
CCGGCGGCGGCCGGCAGTTGCCCGGCGGCGGCGGCGGCGCTGTGGCGGGAGGCCCAGGCG
GCGGCGGCGGCGGCGGCCGAAGAAGAAGACAATGGGCCGGGCTTACAGGGCCTATCCAGG
GAGGCGCCACCGGCCGACGACTTCTACGACGACGACGACGAGGGCGAGGAGGAGGAAGAG
GCGGCGGCCGCTATTGGGTACCGAGATAACCTTCTGTTTGGTGATGAAATCATCACCAAC
GGTTTCCATTCGTGTGAAAGTGACGAAGATGATAGAGCCTCGCATGCGAGTTCTAGTGAC
TGGACTCCAAGACCTCGGATAGGTCCATATACTTTTGTTCAGCAACATCTCATGATTGGC
ACAGATCCTCGAACAATTCTTAAAGATTTACTACCAGAAACAATTCCTCCACCTGAATTG
GATGATATGACACTGTGGCAGATTGTTATTAATATCCTTTCAGAACCACCAAAAAGGAAA
AAAAGAAAAGATATTAATACAATTGAAGATGCTGTGAAATTACTGCAAGAGTGCAAAAAA
ATAATAGTTCTAACTGGAGCTGGGGTTTCTGTTTCTTGTGGAATACCTGACTTCAGGTCA
AGAGACGGTATTTATGCTCGCCTTGCAATAGACTTTCCAGACCTTCCAGATCCTCAAGCA
ATGTTTGATATTGAATATTTCAGAAAAGACCCAAGACCATTCTTCAAGTTTGCAAAGGAA
ATATATCCTGGACAATTCCAACCATCTCTTTGTCACAAATTCATAGCCTTGTCTGATAAG
GAAGGAAAACTACTTCGCAACTATACTCAGAACATAGATACACTGGAGCAGGTTGCAGGA
ATCCAAAAGATAATTCAGTGTCATGGTTCCTTTGCAACAGCATCTTGCCTGATTTGTAAA
TATAAAGTTGACTGTGAAGCTGTACGAGGAGATATTTTTAATCAGGTGGTTCCTCGATGT
CCTAGATGCCCAGCTGATGAACCGCTTGCTATCATGAAACCAGAAATTGTCTTTTTTGGT
GAAAATTTACCAGAACAGTTTCATAGAGCCATGAAGTATGACAAAGATGAAGTTGATCTT
CTCATTGTTATTGGATCTTCCCTGAAAGTAAGACCAGTAGCACTAATTCCAAGTTCCATA
CCCCATGAAGTGCCTCAGATATTAATTAATAGGGAACCTTTGCCTCACCTGCATTTTGAT
GTAGAGCTTCTTGGAGACTGTGACGTAATTATTAATGAACTGTGTCATAGGTTAGGTGGT
GAATATGCCAAACTTTGCTGTAACCCTGTGAAGCTTTCAGAAATTACTGAAAAACCTCCT
CGAATACAAAAAGAGTTGGCACATTTGTCAGAATTGCCACCCACACCCCTCAATATTTCA
GAAGGCTCAAGTTCACCAGAAAGAACTTCACCACCAGATTCTTCAGTGATTGTCACTCTT
TTAGACCAAGAAACAAAGAGTAACGTTGATGATCCAGATGTGTCTGAATCAAAAGACCAT
GTCACAGAAAAATCACAGGAAGTACAGACTTCTACTAGGAGCATTGAAAGTGTTAATGAA
CAGTTGGAGAGTCCAGATTTGAAGAATGCTGTCTCCAATTCTGGTGAGAAAAATGAAAGA
ACTTCAGTAGCTGAAACAGTGAGAAAATGCTGGCCAGCTAGACTTGCAAAGGAGCAGATT
AGTAAACGCCTTGATGATAATCAGTATCTGTTTTTACCACCAAACCGTTACATTTTCCAT
GGCGCTGAGGTATATTCAGACTCTGAAGATGATGTCTTATCCTCTAGTTCTTGCGGCAGT
AACAGTGATAGTGGAACGTGCCAGAGTCCAAGTTTAGAAGAACCCATGGAGGATGAAAGT
GAGAATGAAGAATTTTACAATGGTTTGGAAGATGATGCTGATGTTAATGAGAGAGCTGGA
GGAACTGTATTTGAAGCTGATGGAGGTGATCAAGAGGCAATTAATGAAGCTATATCTGTG
AAACAGGAAGCAACATGCATTAACTATCCATCAAACAAATCA
>Dog_SIRT1

ATGGCGGACGAGGCGGCGCTCGCCCTTCAGCCCGGCGGCTCCCCCTCGGCGGTGGCGGCC
GAGAGGGAGGCCGCGTCGCCGCCCGCCGGGGAGCCACTCCGCAAGAGGCCGCGGAGAGAC
TGTCCTGGCCTGGGGCGGAGCCCAGGCGAGCCCGGCGGGGCGGCCCCTGAGCGGGAGGTG
CCGGCGGCGACCGGGGGCTGCTCCGCGGCGGCCGCGGCGCTGTGGCGGGAGGCGGCGGCG
GGCGGGGAGCGGGAGGCCCAGGCGGCGGTGGCGGCGGCCGGAGAAGGAGACAATGGGCCG
GGCCTACAGGGCCTAACGCGCGAGCCGCTGCCGGCTGACGACTTTGTTGACGACGACGAT
GACGACGAGGGCGAAGAGGAGGAAGAGGCGGCGGCGGCGTCGGCGATTGGGTACCGAGAT
AACCTTCTGTTTGATGATGAAATAATCACCAACGGTTTTCATTCCTGTGAAAGTGATGAG
GATGATAGAGCCTCACATGCAAGCTCTAGTGACTGGACTCCAAGGCCCCGGATAGGTCCA
TATACTTTTGTTCAGCAACATCTCATGATTGGCACAGATCCACGAACAATTCTTAAAGAT
TTACTACCAGAAACAATTCCTCCACCTGAATTGGATGATATGACACTGTGGCAGATTGTT
ATTAATATCCTTTCAGAACCACCAAAAAGGAAGAAAAGAAAAGATATTAATACAATTGAA
GATGCTGTGAAATTACTGCAAGAGTGCAAAAAAATAATAGTTCTAACTGGAGCTGGGGTT
TCTGTTTCTTGTGGAATACCTGACTTCAGGTCAAGAGATGGTATTTATGCTCGCCTTGCA
ATAGACTTCCCAGACCTTCCAGATCCTCAAGCAATGTTTGACATTGAATATTTCAGAAAG
GATCCAAGACCATTCTTCAAGTTTGCAAAGGAAATCTATCCCGGACAGTTCCAACCATCT
CTCTGTCACAAATTCATAGCCTTGTCAGATAAGGAAGGAAAACTACTTCGGAACTATACT
CAGAACATAGATACACTGGAACAGGTTGCAGGAATCCAAAGGATAATTCAGTGTCATGGT
TCCTTTGCAACAGCATCTTGCCTGATTTGTAAATACAAAGTAGACTGTGAAGCTGTACGA
GGAGATATTTTTAATCAGGTGGTTCCTCGTTGTCCTAGGTGCCCAGCTGATGAGCCACTT
GCTATCATGAAACCAGAGATTGTCTTTTTTGGTGAAAATTTACCAGAGCAGTTTCATAGA
GCCATGAAGTATGACAAAGACGAAGTTGATCTCCTCATTGTAATTGGGTCTTCCCTGAAA
GTAAGACCAGTAGCACTAATTCCAAGTTCCATACCCCATGAAGTGCCTCAGATATTAATA
AATAGAGAACCTTTGCCTCATCTGCATTTTGATGTAGAGCTTCTTGGAGACTGTGATGTC
ATAATAAATGAATTATGTCATAGGTTAGGTGGTGAATATGCCAAACTTTGCTGCAACCCT
GTAAAGCTTTCAGAAATCACTGAAAAGCCTCCACGAACACAAAAAGAGTTGGCTCATTTG
TCAGAGTTGCCACCCACACCTCTTAATATTTCAGAAGACTCCAGTTCACCGGAAAGAACT
TCACCACCAGATTCTTCAGTGATTGTTACGCTTTTAGACGAAGCAACTAAGAGTAATGTT
GATGATCCCGGTGTGTCCGAATCAAGAGATTGTATGGAAGAAAAATCACAGGAAGGACAG
AATTCTATTAGGAACATTGAAAGTGTCACTGAACATCTGGAAAGTCCGGATTTGAAGAAT
GTTGGCTGTAATACTGGGGAGAAAAATGAAAGAACTTCAGTTGCTGACCCGGTGAGGAAG
TGCTGGCCAGCTAGACTTGCAAAGGAGCAGATTAGCAAACGGCTTGATGGTAACCAGTAT
CTCTTTTTACCACCAAATCGTTACATTTTCCATGGCGCTGAGGTATATTCAGACTCTGAA
GATGACGTCTTATCCTCTAGTTCTTGTGGCAGTAACAGTGATAGTGGAACCTGCCAGAGT
CCAAGTTTAGAAGAACACTTGGAGGATGAAAGTGAGATTGAAGAATTTTACAATGGTTTG
GAAGATGAAGCTGATGTCAATGAGAGAGCTGGAGGAACTGGATTTGGAATTGATGGAGGT
GATCAAGAGGCAGTCAATGAAGCTATATCCATGAAACAGGAAGCAACAGATACTAACTAT
CCATCAAACAAATCA
>Dolphin_SIRT1
ATGATTGGCACAGATCCTCGAACGATTCTTAAAGATTTACTACCAGAAACAATTCCTCCA
CCTGAATTGGATGATATGACACTGTGGCAGATTGTTATTAATATCCTTTCAGAACCACCA
AAAAGGAAAAAAAGAAAAGATATTAATACAATTGAAGATGCTGTGAAATTACTGCAAGAG
TGCAAAAAAATAATAGTTCTAACTGGAGCTGGGGTTTCTGTTTCTTGTGGAATACCTGAC
TTCAGGTCAAGAGATGGTATTTATGCTCGCCTTGCAGTAGACTTTCCAGACCTTCCAGAT

CCTCAAGCAATGTTTGACATTGAATATTTCAGAAAAGATCCAAGACCATTCTTCAAGTTT
GCAAAGGAAATATATCCTGGACAATTCCAACCATCTCTCTGTCACAAATTCATAGCCTTG
TCAGATAAGGAAGGAAAACTACTTCGCAACTATACTCAGAACATAGATACACTGGAACAG
GTTGCAGGAATCCAAAGGATAATTCAGTGTCATGGTTCCTTTGCAACAGCATCTTGCCTG
ATTTGTAAATACAAAGTTGACTGTGAAGCTGTACGAGGAGATATTTTTAATCAGGTGGTT
CCTCGATGTCCTAGGTGCCCAGCTGATGAACCACTTGCTATCATGAAACCAGAGATTGTC
TTTTTTGGTGAAAATTTACCAGAACAGTTTCATAGAGCCATGAAGTATGACAAAGATGAA
GTTGATCTTCTCATTGTTATTGGGTCTTCCCTGAAAGTAAGACCAGTAGCACTAATTCCG
AGTTCCATACCCCATGAAGTGCCTCAGATATTAATTAATAGGGAACCTTTGCCTCACCTG
CATTTTGATGTAGAGCTTCTTGGAGACTGTGATGTCATTATTAATGAATTGTGTCACAGG
TTAGGTGGTGAATACGCCAAACTTTGCTGCAACCCTGTAAAGCTTTCAGAAATTACTGAA
AAACCTCCACGAACACAAAAAGAATTGGCTCACTTGTCAGAGTTGCCACCCACACCCCTC
AATATTTCAGAAGGCTCAAGTTCACCAGAAGGAACCTCACCAGCAGATTCTTCAGTGATT
GTCACTCTTTTAGACCAAGAAACAAAGAGTAATGTTGATGATCCAGATGCGTCTGAATCA
AAAAACTGTGTCGAAGAAAAATTACAGGAAGTACAGACTTCTGCTAGGAGCATTGAAAGT
GTTAATGAACAGTTGGAGAGTCCGGATTTGAAGAATGTTGCCTCCAATACTGGGGAGAAA
AATGAAAGAACTTCAGTTGCTGAAACAGTGAGAAAATGCTGGCCAGCTAGACTCGCAAAG
GAGCAGATTAGTAAACGGCTTGATGGTAATCAATATCTGTTTTTACCACCAAACCGTTAT
ATTTTCCATGGTGCTGAGGTATATTCAGACTCTGAAGATGATGTCTTATCCTCTAGTTCT
TGTGGCAGTAACAGTGATAGTGGAACATGCCAGAGTCCAAGTTTAGAAGAACCTATGGAG
GATGAAAGTGAGATTGAAGAATTTTACAATGGTTTGGAAGATGATGCTGATGTTAATGAG
AGAACCGGAGGAACTGGATTTGGAGCTGATGGAGGTGATCAAGAGGCAGTTAATGAAGCT
ACATCTATGAAACAGGAAGCAACATGCATTAACTATCCATCGAACAAATCA
>Duck_SIRT1
ATGTCATTGTGCTGTCTTGCAGATAACTTTCTTTTTAGCGATGAAGTCATAGCCAATGGT
TTTCACTCCTGTGATAGTGATGAAGATGACAGAGCCTCACATGCAAGTTCTAGTGATTGG
ACCCCAAGACCACGTATAGGTCCTTACACTTTTGTTCAGCAACATCTCATGCTAGGTACA
GACCCACGAACAATTCTGAAAGACCTGCTACCAGAAACAATCCCTCCACCGGAACTGGAT
GATATGACGCTGTGGCAAATTGTCATAAACATTCTTTCAGAACCACCAAAAAGAAAAAAG
CGAAAAGATATCAATACTATTGATGATGCTGTGAAACTTTTACAAGAGTGCAAAAAAAATA
ATGGTCTTGACGGGAGCTGGGGTGTCTGTGTCTTGTGGAATACCTGACTTTAGATCAAGA
GATGGCATCTATGCACGCCTTGCTGTAGACTTCCCAGACCTTCCAGATCCTCAAGCAATG
TTTGATATAGAATACTTCAGAAAGGATCCCAGGCCATTTTTTAAGTTTGCAAAGGAAATC
TATCCAGGACAATTCCAGCCATCTCTCTGTCACAAGTTCATAGCTTTGATGGATAAAGAA
GGAAAACTACTCCGCAACTATACTCAGAACATAGACACATTGGAACAAGTTGCTGGAATC
CAAAGGATAATTCAGTGTCATGGTTCCTTTGCAACAGCTTCCTGCCTGATCTGTAAATAC
AGAGTTGACTGTGAAGTTGTTCGAGGAGATATTTTCAATCAGGTTGTTCCTAGATGTCCC
AGATGTCCACCTGATGAACCGCTTGCCATCATGAAACCAGACATAGTGTTCTTTGGAGAG
AATTTACCTGAGCAGTTCCATCGCGCCATGAAGTATGACAAAAATGAAGTTGATCTCCTC
ATTGTCATTGGGTCTTCACTGAAAGTAAGACCAGTAGCATTGATTCCAAGTTCCATCCCC
CATGAAGTGCCTCAAATTTTAATAAATAGGGAACCCTTGCCTCATCTACACTTTGATGTG
GAGCTTCTGGGAGACTGTGATGTTATTATTAATGAATTATGTCAAAGGCTAGGAAGCGAA
TATACAAAACTTTGCTACAATTCAGTAAAACTTTCAGAAATAACAGAAAAGCCTCCACGA
CCGCACAAGGAGCTTGAAATGCACTCATGTGAGCTACCACCTACCCCTTTAAACATTTCA

GAAGACTCTAGTTCACCAGAACAAATGACTCCACCGGATTCTGTGGTGGTGTCCTCGGAA
CAGCCAGCTGAATGTAAGGTAGAAAACTGTGATCCTGCCTCAGAAATTAAAATGAGCTGC
ACAGAGGAAAAGCTTCAGGACACACAGACATCCTCTGAAAACCCTGAAAATGTTACTAGT
GAATTAATAAACTCTGAAACAATGAAGGAAAATGGAACCACCAACGGAGAAAACAAAGAG
AAAGAAATCTTGAAGAAGTGCTGGGTAAACAGACCTGCAAAAGAACAGATTAGCAAAAGG
CTGGATGGTACTCAGTATCTATTTTTACCACCAAATCGCTATATTTTCCATGGTGCTGAG
GTATACTCAGATTCTGAAGATGATATCATGTCTTCTAGCTCTTGTGGGAGTAGTAGCGAA
AGTGGCTCATGTCGTAGTCAGAGCTTAGATGTGGAAGATGAGAGTGAGATTGAAGAGTTT
TACAATGGCATAGAGGATGAGGATGCTCCAGAAAGGGAAGAAGAAGCTGGATTTGGGGAA
GATGGAGTTGAACAAGATGCAGCTGATGAATCAGCTTATACAAATGAAGCTGCAAAGAAT
GATCCAACAAGCAACAAATTG
>Elephant_SIRT1
ATGATTGGAACAGACCCTCGAACTATTCTTAAAGATTTACTACCGGAAACAATTCCTCCA
CCTGAATTGGATGATATGACACTGTGGCAGATTGTTATTAATATCCTTTCAGAACCACCA
AAAAGGAAAAAAAGAAAAGATATTAATACAATTGAAGATGCTGTGAAATTACTGCAAGAG
TGCAAAAAAATAATAGTTTTAACTGGAGCAGGGGTGTCTGTTTCCTGTGGAATACCTGAC
TTCAGGTCAAGAGATGGTATTTATGCACGCCTTGCAGTGGACTTCCCAGACCTTCCAGAC
CCTCAAGCAATGTTTGATATTGAATATTTCAGAAAGGATCCAAGACCATTCTTCAAGTTT
GCGAAGGAAATATATCCTGGACAATTCCAGCCATCTCTCTGTCACAAATTTATAGCCTTG
TCAGATAAGGAAGGAAAACTACTTCGCAACTATACTCAGAACATAGATACACTGGAGCAG
GTGGCAGGAATCCAAAGGATAATTCAGTGTCATGGTTCCTTTGCAACAGCATCTTGCCTA
ATTTGTAAATACAAAGTTGACTGTGAAGCTGTACGAGGAGATATTTTTAATCAGGTGGTT
CCTCGATGTCCTAGGTGCCCAGCTGATGAACCACTTGCTATCATGAAACCAGAGATTGTC
TTTTTTGGTGAGAATTTACCAGAACAGTTTCATAGAGCCATGAAGTATGACAAAGATGAA
GTTGACCTCCTCATTGTTATTGGGTCTTCCCTGAAAGTAAGACCAGTAGCACTAATTCCA
AGTTCCATACCCCATGAAGTGCCTCAGATAATAATTAATAGGGAACCTNTCCCTCATCTG
CATTTTGATGTAGAGCTTCTTGGAGACTGTGATGTCATAACTAATGAATTGTGTCATAGG
CTAGGTGGTGAATATGCCAAACTTTGCTGCAGCCCCGTAAAGCTTTCAGAAATTACTGAA
AAACCTCCCCGAACACAAAAAGAGTTGGCTCATTTGTCAGAGTTGCCACCCACACCTCTT
AATATTTCAGAAGACTCAAGTTCACCAGAAAGAACTTCACCACCAGATTCTTCAGTGGTT
GTTACACTTTTAGACCAAGCAACTAAGAGTAATATTGATGATTCAGATGTGTCCGAATCA
AAAGGATGTGTGGAAGAAAAATCACAGGAAGTACAGATTTCTACTAGGAACACTGAAAGT
ATTACTGAACAACTGGAGAGTCCGGATTTGAAGAACGTTGGCTCCAGTACTGGGGAGAAA
AATGAAAGAACTTCAGTTGCTGAAACAGTGAGAAAGTGCTGGCCCATTAGACTTGCAAAG
GAACAGATTAGTAAGCGGCTTGATGGTAATCAGTATCTGTTTTTACCACCAAATCGTTAC
ATTTTCCATGGCGCTGAGGTGTATTCAGATTCTGAAGATGACGCCTTATCCTCCAGTTCT
TGTGGCAGTAACAGTGATAGTGGAACATGCCACAGTCCAAGTTTAGAGGAAGCCATGGAG
GATGAAAGTGAGATTGAAGAGTTCTACGACGGACTGGAAGATGCTGCTGACGTCCCAGGG
AGAGCTGGAGCAGCTGGAGCTGGTGGAGGGGGTCAGGAGGCAGTAAATGAAGCTGTGTCT
GTGAACCAGGGAGCCACAGACATGAGCTGTCCATCAAACGAGTCG
>Flycatcher_SIRT1
ATGCTTAGAAACTTTTTCTTTCAGATCGTATTTGAACTGTACTGGTGGGCAGCACCACTG
CGAAAGTACCTCACAGTACAAACAGATCCAGATAACTTCCTCCTCAGCGATGAAATCATA
GCCAATGGCTTTCACTCCTGTGACAGTGATGAGGAAGACAGAGCCTCACATGCAAGTTCC

AGTGACTGGACCCCAAGACCACGTATAGGTCCCTACACTTTTGTTCAGCAGCATCTCATG
TTAGGTACAGACCCCCGGACGATTCTGAAGGACCTGCTGCCAGAAACCATCCCCCCACCT
GAACTGGATGACATGACTCTGTGGCAGATCGTGATAAACATCCTCTCAGAGCCACCAAAA
AGGAAGAAGAGGAAAGATATTAACACCATTGATGATGCTGTGAAACTTCTGCAGGAGTGC
AAGAAGATCATGGTCCTGACTGGAGCTGGGGTGTCAGTGTCTTGTGGAATCCCTGACTTT
AGATCCAGAGATGGCATCTATGCACGCCTTGCTGTAGACTTCCCAGACCTTCCAGATCCT
CAAGCAATGTTTGATATAGAGTACTTCAGAAAGGATCCCAGGCCCTTTTTTAAGTTTTCA
AAGGAAATCTACCCAGGACAGTTCCAGCCCTCTCTCTGCCACAAGTTCATCGCTTTGATG
GATAAGGAAGGAAAACTCCTTCGCAACTATACTCAGAACATAGACACACTGGAGCAGGTG
GCAGGAATCCAAAGGATAATACAGTGTCATGGTTCCTTTGCAACAGCTTCCTGCCTGATC
TGTAAATACAAAGTTGATTGTGAAGTTGTTCGAGGAGATATTTTCAATCAGGTGGTGCCC
CGCTGTCCCCGCTGTCCCCCCGAGGAGCCCCTGGCTATCATGAAGCCGGACATTGTGTTC
TTTGGGGAGAACCTGCCCGAGCAGTTCCACCGTGCCATGAAGTACGACAAAAACGAAGTG
GATCTCCTCATTGTCATTGGGTCTTCGCTCAAAGTAAGACCAGTAGCATTGATCCCAAGT
TCCATCCCCCATGAAGTGCCTCAGATCTTAATTAATAGGGAGCCTTTGCCTCATCTACAC
TTTGACGTGGAGCTCCTCGGAGACTGTGATGTTATAATCAATGAATTGTGTCAGAGGTTA
GGTAGTGAGTACACAAAACTTTGCTACAACTCGGTGAAACTTTCGGAAATCACAGAAAAG
CCTCCACGGCCGCACAAGGAGCTCGAAGCGCTCTCGGCCGAGCTCCCACCAACCCCTCTG
AACATTTCAGAAGGCTCCAGTTCACCAGAAAGGATGAGCCCACCTGATCCTGCAGCAGGG
TCACAACACCCACCTGAATGTAAGGTAGAAAACTGTGAGCCTGCCTCAGAAACTAAAGGG
ACCTGCTCCGAGGAGACCCTTCAGGACACGCAGGTGTCATCAGAAAACCCTGAAAATCCT
GCTAGTGAGCGAATGAACTCTGAAACAATGAAGGAAAATGGATCTAATGATGGAGAAAAT
AAAGAAAAGAGTGAAATACTGAAGAAGTGTTGGGTAAACAGATCTGCAAAAGAACAGATT
AGCAAAAGGCTGGATGGTACTCAGTATCTGTTTCTGCCACCCAACCGCTATATCTTCCAC
GGTGCTGAGGTGTACTCGGATTCTGAGGACGACATCCTATCTTCCAGCTCTTGCGGCAGC
AGCAGCGAGAGCGGCTCGTGCCGCAGCCAGAGCTTGGATGTGGAGGATGAGAGCGAGATG
GAGGAGTTCTACAATGGCATAGAGGACGAGGATGCTCCCGAGAGGGAAGAGGAGGCTGCA
TTCGGGGAGGATGGAGCCGAGCAGGAGGAACTGGCAGCTGAGGAATCAGCCGAGCCGAAC
GGAGCTGCAGGGACGGAACATCCCAGCGACGCACTG
>Frog_SIRT1
ATGGCGGACTCAGATCGTGTAGGTTTTCCCATTGCCGGCGCTGTGTTAGCTGTCTCAAAA
GAAAATGGCGAGCCCCTTTCCAAAAGGCAGCGCTTGGAAGATACCGGCGGGGGTGGGAGC
CAGCTTGTGGGGGCTGAAAGCGAGGGAAAAGCGGCATTGCCGCCTATAGCTGCTTCTCTC
CAGGAGGAAGGCGAGGCCTCTTCGGCAATGGAAAGCAAATCCAGAGCGCACAATGGCTCG
GGCTTTCAGGGCCTTCCTTTGGGAACGTATTTGGTACAAGGCCAGGAGGAGGGCGGAGCC
GAGGAGATGCCCAATGGAGACCTGTCGGATCAGGCTGTTGATTACGGAGGGCCCTATACA
TTTGTTCAGAGGCACTTAATGATGGGAACTGATCCCCGGACAATTCTTAAGGATCTGCTC
CCTGACACTGTAGCACCGTCAGAGTTAGATGATATGACTTTGTGGCAGATAGTTATAAAC
ATTCTTTCTGACCCACCAAAACGAAAGAAGCGTAAAGACATCAACACCATTGATGATGCT
GTGAAACTTTTACAAGAGAGCAAGAAAATAATTGTTTTAACGGGAGCAGGGGTTTCGGTA
TCTTGTGGAATACCAGATTTTAGATCAAGAGATGGCATTTATGCTCGTCTTGCAGTGGAT
TTTCCAGACCTTCCTAATCCTCAAGCCATGTTTGATATTGAATACTTCAGGAAAGATCCA
AGACCATTTTTTAAATTTGCTAAAGAAATCTTTCCTGGCCAGTTTCAGCCTTCGTTGTGC
CACAGATTTATAGCTATGTTGGATAAAGAGGAAAAGCTGCTCAGAAACTATACCCAGAAT

ATAGACACACTTGAACAAGTTGCTGGGATTGAAAAGATTATACAATGTCATGGATCATTT
GCTGAAGCATCTTGTCTTGTATGTAAATACAAAGTTGACTGTGAAGCTGTTAGAGAGGAC
ATATTTAATCAGATAGTTCCAAGGTGCCCAAGGTGTTCATCTGATGAGCCTCTTGCCATC
ATGAAACCGGACATTGTATTTTTCGGTGAAAACTTGCCAGAACAGTTTCACAGAGCTATG
AAATATGACAAAAACGAGGTTGATCTTCTTATTGTTATTGGATCTTCCCTGAAAGTCAGG
CCAGTAGCATTAATACCAAGTTCTATTCCTCATGAAGTGCCTCAGATACTAATTAATAGG
GAACCATTGCCTCATTTACACTTCGATATAGAACTGCTTGGAGATTGTGATGTAATTATA
AACGAGTTATGTCAAAGACTAGATGGGAAATACTCCCAGCTTTGTACCAATTCCTTAAAA
CTTTCACAAATCACAGAAAAGCCCCCTCGAATACACAAAGGTTTCCTTACCTCACCAGAG
ACTGTCCCATCTACAGACTTAAACACAGGGCAAAGTCCAGCACTACAAAGCGACCTGCGT
GGAACAGACTTACAGCTAAGTGCTTCTAATACAATGCGCTCATTGGAAAAACCCGAGGAA
GCCTCCAAGCTTTCACACAACTGTAGTGAGGAAAATCTAGAAGTGTCGAAGGAAGCTAAC
ACACAGCTCAGCAATGAGAAAGATCAAGAAACAGCTGAGAAAGATACTGACATTGATTCA
GCCAAGGATTTGGAAAGTAAATATACAAAAGAACAGATCAGCAAGCGCCTAGACAGCACG
CAGTTTTTATTTTTAGCGCCAAATCGCTATATTTTTCACGGCGCAGAAGTGTTTTCGGAT
TCAGATGAAGATCTGACATCTAGTTCCTGTGGAACAAACAGCGACAGCGAATCTTTACTA
AGCCCAAGCCTGCATGAGCCTATCGAAGAGGATAGTGATACTGAAGAATGCTTCCATGCT
AAATATGAGAATGAGACTGATACAGATAACAGGGCAGACTTAGAAAGAGAACCCGAGAGG
GTTGTACTGTATCAAAGTGATGATCTTCTAGGAATCGATGGTACTACCATGAACTTA
>Fruitfly_SIRT1
ATGATGGAAAATTACGAGGAAATTCGCCTGGGCCACATTAGGTCTAAAGATCTGGGCAAC
CAGGTGCCAGACACTACGCAATTCTATCCGCCAACTAAGTTTGATTTTGGCGCGGAAATT
CTGGCCTCAACGTCAACAGAGGCAGAGGCAGAGGCAGAAGCAACAGCAACAACCACAGAA
CCAGCAACAAGCGAACTTGCTGGCAAAGCAAATGGTGAAATCAAAACAAAAACATTGGCT
GCCAGGGAAGAACAAGAGATTGGCGCCAATTTGGAGCATAAAACCAAAAATCCCACAAAG
TCAATGGGCGAGGATGAAGATGACGAGGAGGAGGAGGAAGAGGACGATGAGGAGGAGGAG
GAGGACGACGAGGAGGGAATCACCGGAACGAGCAACGAGGATGAGGACTCCAGCTCAAAT
TGCTCCTCATCCGTGGAACCCGACTGGAAGCTGCGCTGGTTGCAACGAGAATTTTACACA
GGTCGTGTGCCGCGCCAGGTTATTGCCAGCATTATGCCGCATTTCGCCACCGGCCTGGCG
GGCGACACCGACGACTCCGTGCTGTGGGACTATTTGGCCCACCTGTTGAACGAGCCGAAG
CGGCGCAACAAGCTGGCCTCAGTGAACACCTTCGACGATGTCATCAGTTTGGTCAAGAAA
TCACAGAAGATCATTGTGCTAACGGGAGCCGGAGTATCCGTCTCCTGCGGCATTCCGGAC
TTCCGGTCCACCAATGGCATATATGCGCGATTGGCCCATGATTTTCCCGATCTGCCCGAT
CCGCAGGCCATGTTTGATATCAACTACTTCAAGAGGGATCCACGACCGTTCTACAAGTTT
GCCCGCGAGATATATCCCGGCGAGTTTCAGCCCTCACCCTGCCATCGTTTCATCAAAATG
CTGGAGACCAAGGGCAAACTGTTGCGCAACTACACACAGAACATCGACACCCTCGAGCGG
GTGGCAGGCATTCAGCGAGTAATCGAGTGTCACGGCTCCTTTTCAACGGCCTCGTGCACC
AAGTGTCGTTTCAAGTGCAACGCTGACGCCCTGCGGGCGGACATATTTGCCCAGCGAATT
CCGGTGTGCCCGCAGTGCCAGCCCAATAAGGAGCAGAGCGTGGATGCCTCGGTGGCCGTT
ACTGAGGAGGAGCTGCGCCAACTGGTCGAGAACGGCATCATGAAGCCGGATATCGTCTTT
TTCGGCGAGGGACTGCCGGATGAGTACCACACGGTCATGGCCACCGACAAGGACGTGTGC
GATCTACTGATCGTGATCGGCTCCTCGCTGAAGGTCCGACCTGTGGCCCACATTCCCAGC
AGCATACCGGCCACGGTGCCGCAGATTCTTATCAATCGCGAGCAGCTGCATCACCTTAAG
TTCGATGTGGAGCTGCTGGGCGACTCCGATGTGATCATCAACCAGATTTGCCACCGGTTG

TCGGACAACGATGATTGCTGGCGGCAGCTGTGCTGCGATGAGTCAGTGCTTACCGAAAGC
AAGGAGCTAATGCCTCCGGAGCACTCTAATCACCACCTCCATCATCATCTACTTCACCAC
CGCCACTGCAGTTCAGAGAGCGAGCGACAGTCGCAACTGGACACGGATACGCAGTCTATT
AAATCAAATAGTTCGGCGGACTACATACTAGGATCAGCTGGCACCTGCTCGGATAGTGGA
TTTGAGTCATCTACTTTTAGCTGTGGAAAGCGTTCCACTGCCGCCGAAGCGGCAGCCATC
GAACGTATTAAAACAGACATACTGGTTGAGCTGAACGAGACCACAGCCCTAAGTTGCGAT
CGTCTGGGCCTGGAAGGCCCTCAGACAACGGTGGAGAGCTATCGCCATCTTTCCATTGAT
TCCTCCAAGGATAGCGGCATCGAGCAGTGCGACAACGAAGCCACGCCTAGCTACGTGCGA
CCCAGCAATCTTGTTCAGGAGACCAAGACAGTGGCGCCCAGCCTGACGCCCATTCCACAA
CAGAGGGGAAAGCGACAGACAGCAGCCGAGCGTCTGCAGCCTGGAACATTCTATTCGCAC
ACCAACAACTATTCGTATGTGTTTCCAGGAGCCCAGGTATTCTGGGACAACGATTACAGC
GATGATGATGACGAAGAAGAGGAAAGATCACACAATAGACACAGTGATCTCTTTGGCAAT
GTGGGGCACAATTATAAGGATGATGATGAGGATGCATGTGATCTGAACGCCGTTCCATTG
TCACCATTGCTACCGCCTTCACTGGAGGCTCACATAGTCACCGATATAGTGAATGGATCC
AACGAACCGCTGCCCAACAGCAGTCCCGGCCAGAAAAGAACCGCCTGCATTATAGAACAG
CAGCCAACGCCCGCTATTGAAACGGAAATTCCCCCACTAAAGAAGCGGCGACCAAGTGAG
GAAAATAAGCAGCAGACCCAAATAGAAAGATCTGAGGAGAGTCCGCCTCCAGGACAGTTA
GCAGCAGTG
>Fugu_SIRT1
ATGGCGGATGGAGAGAACAGTCTCGGAATGGCTTTCACTGGCGCCTCCGATATGGACGAG
CCTGTTGTAAAAAGGGCCAAAATGAGTCCGCTGAACCATTACGGATTTCTAGCCACCAAA
GCAGACCAGTTTCCATGCGTCGCCGGTGCTACTGAGAGCTGGGAGACGGCGGTGAATTGT
GCGCACCCAGCAGGGAAGGAAGCGAAGCCGGCGATGGCGGTAGAACAGGCCCCAGTAGCG
CTAGGCGGAGACAACAATGGACTGGGAATGCTGGTCTCTGAGCCACACAAAACAGTTATG
AAACGAGAAGACAGCGATGAACTTGGGGCGATTGAGGAGAGTGCTGCTTTTCTGGAGCAT
GGAGATCTGTCTTGTAACGGCCTGGCCGTCACACCGGAGCACATCCATGAAGACGATGAC
AGATCCTCACATGCGAGCTCCAGTGACTGGACCCCTCAACCCCAGATAGGTTCCTACAGT
TTCATCCAGCAGCACATCAGAGAGACCGATCCCAGGGCGATTCTGAGGGACTTGCTCCCC
GACACCGTGCTCCCACCGGATTTAGACGACATGACCCTGTGGCAGATCATCATCAATATC
TCAGAACCTCCGAAAAGGAAGAAACGAAAAGATGTCAACACTTTAGAAGATGTAGTCAAG
CTGCTAAAGGAAAGCAAGAGGATCCTTGTCCTGACTGGTGCTGGGGTGTCTGTTTCTTGT
GGCATCCCGGATTTTCGTTCTAGAGATGGAATTTATGCACGCCTTGCTGTAGATTTCCCT
GATCTTCCAGACCCTCAAGCCATGTTTGACATCGAATACTTCAGAAGGGACCCAAGACCA
TTCTTCAAGTTCGCCAAGGAAATCTACCCTGGTCAGTTCCAACCTTCGCTCTGTCATAAA
TTCATATCCATGCTGGATAAGCAAGGGAAGCTGCTGCGGAATTACACCCAAAACATCGAC
ACATTAGAACAAGTAGCTGGAGTTCAGCGGATTATCCAGTGTCACGGTTCATTTGCAACT
GCGTCCTGTCTTGTCTGTAAACACAAAGTAGATTGTGAGGTCATAAGAGAAGACATCTTT
AACCAGGTTGTCCCTCATTGTTCGCGCTGTCTGGATATTCCTCTGGCAATCATGAAGCCT
GATATTGTCTTCTTTGGAGAAAATCTTCCAGAAATGTTCCACAGGGCCATGAAGCAGGAT
AAAGACGAGGTGGACCTCTTGATTGTCATCGGTTCTTCACTTAAAGTTCGGCCGGTTGCC
CTCATCCCAAACTCCATTCCTCACGACGTGCCTCAGGTCCTGATAAATAGGGAGCAACTT
CCTCACCTCAACTTCGATGTGGAACTACTCGGAGACTGCGACGTCATTGTCAACGAGCTC
TGTCATCAGTTGGGCGTGGAATACGAGCAGCTTTGCTCCAACACTCTAAGACTCAATGAG
ATCACAGAGAAACCTCCTCGGTTACCAGAACAGTCACCTAATGAGGCCTTGTCTGCTCCC

AAGGATGCAGCTCATGTGGAGCAGAAGCAGCACCGCATAGACTCAGTAACCAAGTCTTCA
CAGGAGGAAGAAACCCCCAGTGTCCCTGAGACCGCCACTAAGATTACTACGCCTCCGCAG
CCTTGCCCAAATGCTCAGCGTCCAAGTGAAGAGACGACCAAGCCAGCAGAGGGACGCACA
GAGGCTTCACCAACAGAAGAGCCCACTGAAGTGAAGAACCACACCTCTAACCTTCACAGA
CGATACTGGATGAGTCGGATCAGCAGAAGTCCAGTCAGCAAACGGCTTGAAACAGGTCAG
TACCTGTTTCAGGCACCAAATCAGTACATCTTCCACGGGGCTGAGGTGTACTCCGACTCT
GAAGATGACACGTCCAGCTCCAGGAGCAGCGACAGCGATGAGTCCGAGAGCAGCGCAGAT
GGCGTGGAGGACGACAGCGACCCAGAGGAGGCGGCAGCACTACCGGAGGACGCAGAGACG
ACCCTCAGAGACACGATAAACAGTGATGCCACGTCCGGTGTGCAGACAGACAGTACATCA
GAGAAAACAGAGAGCACCATGCATCTT
>Anole_lizard_SIRT1
ATGGCGGACGAGGAGGCGCCGCGCCTCCAGCCCCGCGGCGGCCTCAGCCCCGAGCCGGCT
CCGAAGCGCCTTCGACGGGGGTCGGCTGAGGGGGAGGCGGCCGCCGAAGGAGCCGCGGAG
GAGGAGGCCCCCCTGGGCGTGACGGAGGCCGCAGGAGGCGAAGACGGGGGCTGGAGCCTC
TCCGAGAGCGGGCCGAGGCGGTGGGGCCTGGGCCGAAGGGAAGAAGAGCAGGAGGAGGAG
GAGGAGCGCCAACCCGCCGCGGCTGAGCGAGGGGAGGGGGCGGGGGCGGGGGCGGCGCGC
GCCGGAGAGGAGGCCGCGTCGGGGCCGGCCATTGGCTGCGGCGCGGCGCAGTCTGCCAAC
GGAGAGGCCGTGCAAGGAGGAGGAGAGGCCGATAATATATTTTTTAGTGATGAAATCATT
GCAAATGGCTTCCATTCCTGTGACAGTGATGAAGATGATAGAGCCTCTCATGCAAGCTCA
AGTGACTGGACCCCAAGGCCACGTATAGGTCCATACACTTTTGTCCAGCAACATCTCATG
ATAGGAACAGATCCACGTGCAATTCTCAAAGATTTATTGCCAGAAACAATACCTCCCCCT
GAACTGGATGACATGACATTATGGCAAATCGTAATTAATATTCTCTCAGAACCACCTAAA
AGAAAAAAGAGGAAAGATATTAATACTCTAGAAGATGCTGTGAAACTCCTACATGAGTGC
AAAAAAATAATTGTCTTAACTGGAGCTGGGGTATCTGTTTCTTGTGGAATTCCTGATTTC
CGATCAAGAGATGGCATTTATGCTCGTCTTGCAGTAGATTTCCCAGATCTTCCTGATCCT
CAAGCAATGTTTGATATTGAATACTTCAGAAAAGATCCAAGGCCATTTTTTAAATTTGCA
AAGGAAATATATCCTGGACAGTTTCAACCATCCCTTTGTCATAAATTTATAGCTCTGATG
GATAAAGAAAGAAAACTACTGCGCAATTATACTCAGAACATAGATACTCTGGAACAAGTT
GCAGGAATTCAAAGAATAATTCAATGCCATGGTTCCTTTGCAACAGCTTCTTGCCTCATC
TGTAAATACAAAGTTGATTGTGAAGTTGTTCGAGGAGATATTTTTAATCAGGTTGTTCCT
AGATGTCCCAGATGCCCACCTGACGAACCACTTGCCATCATGAAGCCAGAAATAGTGTTC
TTTGGTGAAAATCTCCCTGAGCAATTTCATAGGGCCATGAAGTATGACAAAGATGAGGTC
GACCTTCTTATTGTTATTGGGTCTTCACTTAAAGTAAGACCAGTAGCATTAATTCCAAGT
TCCATCCCTCATGAAGTGCCTCAGATTCTAATTAATAGGGAACCATTGCCTCATCTACAC
TTCGATGTGGAGCTCCTTGGAGACTGTGATGTTATCATCAATGAACTATGTCATAGATTA
GGTGGTGAATATGCAAAACTGTGCAACAGTTCAATCAAACTTTCAGAAATCACAGAGAAG
CCTCCACGACCACAGAAGGAATTTGAAATACATCCATCAGAGTTGCCACCTACACCACTA
AACATCTCTGAATATTCTAGTTCGCCTGAGAGAATTGCACCACATGAGTCTCAAATAGTA
CATTCAGAGCACTCTTCCGAATTTAAAGCAGCAGACTCTGAAGCTACGTTGGAATCTAAA
GAGAACTGTGTAGAGGAAAAATCTCAAGAGCTACAAAACTGTTTAGAAAATATTGAAAGT
CTTCCTGACCAGTTAGAAAACCCAGAGCATGTGAAGGAACACGGATCTAACCAAGGTGAA
AACAAAGAAAGGAGTGAAAAAACATCTGTTGAAACACTGAAAAAGTGCTGGGTTAACAGA
TGTGCAAAAGAGCAAATAAGCAAGCGGCTTGATGGTACCCAATACTTGTTTTTACCACCA
AATCGCTATATTTTTCATGGTGCCGAGGTATATTCAGACTCTGAAGACGATGTCATGTCC

TCCAGTTCTTGTGGAAGTAGCAGTGATAGCGGATCCTGTCATAGTCCGAGTTTAGATGTA
GAAGATGAAAGTGAACTTGAAGAATTCTATAACGGCATTGAAGATGATGATGTTCCTGAC
AGAGAAGAGGAGAATGGTTTTGGAGAAGATGGAACTGACATTCAAGAAACTGGTGACGAA
TCTGTTTCTTTAAATGAGACTGTACGATTTGATCATTCAGCAGACACATTG
>Horse_SIRT1
ATGATTGGCACAGATCCTCGAACAATTCTTAAAGATTTGCTACCAGAAACAATTCCTCCA
CCTGAATTGGATGATATGACACTGTGGCAGATTGTTATTAATATCCTTTCAGAACCACCA
AAAAGGAAAAAAAGAAAAGATATTAATACAATTGAAGATGCTGTGAAATTACTGCAAGAG
TGCAAGAAAATAATAGTTCTAACTGGAGCTGGGGTTTCTGTTTCTTGTGGAATACCTGAC
TTCAGGTCAAGAGATGGTATTTATGCTCGCCTTGCAGTAGACTTCCCAGACCTTCCAGAT
CCTCAAGCAATGTTTGATATTGAATATTTCAGGAAAGATCCAAGACCATTCTTCAAGTTT
GCAAAGGAAATATATCCTGGACAGTTCCAGCCATCTCTCTGTCACAAATTCATAGCTTTG
TCAGATAAGGAAGGAAAACTACTTCGCAACTATACTCAGAACATAGATACACTGGAACAG
GTTGCAGGAATCCAAAGGATAATTCAGTGTCACGGTTCCTTTGCAACAGCGTCTTGCCTG
ATTTGTAAATACAAAGTTGACTGTGAAGCTGTACGAGGAGATATTTTTAATCAGGTGGTT
CCTCGATGTCCCAGGTGCCCAGCTGATGAACCACTTGCTATCATGAAACCAGAGATTGTT
TTTTTTGGTGAAAATTTACCAGAACAGTTTCATAGAGCCATGAAATATGACAAAGATGAA
GTTGATCTCCTCATCGTTATTGGGTCTTCCCTGAAAGTAAGACCAGTAGCACTAATTCCA
AGTTCCATACCCCATGAAGTGCCTCAGATATTAATTAATAGGGAACCTTTGCCTCATCTG
CATTTTGATGTAGAGCTTCTTGGAGACTGTGATGTCATAATTAATGAATTGTGTCATAGG
TTAGGTGGTGAATATGCCAAACTTTGCTGCAACCCTATAAAGCTTTCAGAAATTACTGAA
AAACCTCCACGAACACAAAGAGAGTTGGCTCATTTGTCAGAATTGCCACCCACACCTCTT
AATATTTCAGAAGACTCAAGTTCACCAGAAAGAACTTCACCACCAGATTCGTCCGTGATT
GTCACACTTTTAGACCAAGCAACAAAGAGTAATGTTGATGATCCAGATGTGTCCAAATCA
AAAGATTGTATGGAAGAAAAATCACAGGAAGTACAGACATCTACTAGGAGCTTTCAAAGT
GTTACTGAACGGTTGGGAAGTCCGTATTTGAAGAATGTTGGCTCTGATACTGGGGAGAAA
AATGAAAGAACTTCAGTTGCTGAAGCAGTAAGAAAGTGCTGGCCAGCTAGACTCGCAAAG
GAGCAGATTAGTAAACGGCTTGATGGTAAGAAAGGCTGT
>Human_SIRT1
ATGGCGGACGAGGCGGCCCTCGCCCTTCAGCCCGGCGGCTCCCCCTCGGCGGCGGGGGCC
GACAGGGAGGCCGCGTCGTCCCCCGCCGGGGAGCCGCTCCGCAAGAGGCCGCGGAGAGAT
GGTCCCGGCCTCGAGCGGAGCCCGGGCGAGCCCGGTGGGGCGGCCCCAGAGCGTGAGGTG
CCGGCGGCGGCCAGGGGCTGCCCGGGTGCGGCGGCGGCGGCGCTGTGGCGGGAGGCGGAG
GCAGAGGCGGCGGCGGCAGGCGGGGAGCAAGAGGCCCAGGCGACTGCGGCGGCTGGGGAA
GGAGACAATGGGCCGGGCCTGCAGGGCCCATCTCGGGAGCCACCGCTGGCCGACAACTTG
TACGACGAAGACGACGACGACGAGGGCGAGGAGGAGGAAGAGGCGGCGGCGGCGGCGATT
GGGTACCGAGATAACCTTCTGTTCGGTGATGAAATTATCACTAATGGTTTTCATTCCTGT
GAAAGTGATGAGGAGGATAGAGCCTCACATGCAAGCTCTAGTGACTGGACTCCAAGGCCA
CGGATAGGTCCATATACTTTTGTTCAGCAACATCTTATGATTGGCACAGATCCTCGAACA
ATTCTTAAAGATTTATTGCCGGAAACAATACCTCCACCTGAGTTGGATGATATGACACTG
TGGCAGATTGTTATTAATATCCTTTCAGAACCACCAAAAAGGAAAAAAAGAAAAGATATT
AATACAATTGAAGATGCTGTGAAATTACTGCAAGAGTGCAAAAAAATTATAGTTCTAACT
GGAGCTGGGGTGTCTGTTTCATGTGGAATACCTGACTTCAGGTCAAGGGATGGTATTTAT
GCTCGCCTTGCTGTAGACTTCCCAGATCTTCCAGATCCTCAAGCGATGTTTGATATTGAA

TATTTCAGAAAAGATCCAAGACCATTCTTCAAGTTTGCAAAGGAAATATATCCTGGACAA
TTCCAGCCATCTCTCTGTCACAAATTCATAGCCTTGTCAGATAAGGAAGGAAAACTACTT
CGCAACTATACCCAGAACATAGACACGCTGGAACAGGTTGCGGGAATCCAAAGGATAATT
CAGTGTCATGGTTCCTTTGCAACAGCATCTTGCCTGATTTGTAAATACAAAGTTGACTGT
GAAGCTGTACGAGGAGATATTTTTAATCAGGTAGTTCCTCGATGTCCTAGGTGCCCAGCT
GATGAACCGCTTGCTATCATGAAACCAGAGATTGTGTTTTTTGGTGAAAATTTACCAGAA
CAGTTTCATAGAGCCATGAAGTATGACAAAGATGAAGTTGACCTCCTCATTGTTATTGGG
TCTTCCCTCAAAGTAAGACCAGTAGCACTAATTCCAAGTTCCATACCCCATGAAGTGCCT
CAGATATTAATTAATAGAGAACCTTTGCCTCATCTGCATTTTGATGTAGAGCTTCTTGGA
GACTGTGATGTCATAATTAATGAATTGTGTCATAGGTTAGGTGGTGAATATGCCAAACTT
TGCTGTAACCCTGTAAAGCTTTCAGAAATTACTGAAAAACCTCCACGAACACAAAAAGAA
TTGGCTTATTTGTCAGAGTTGCCACCCACACCTCTTCATGTTTCAGAAGACTCAAGTTCA
CCAGAAAGAACTTCACCACCAGATTCTTCAGTGATTGTCACACTTTTAGACCAAGCAGCT
AAGAGTAATGATGATTTAGATGTGTCTGAATCAAAAGGTTGTATGGAAGAAAAACCACAG
GAAGTACAAACTTCTAGGAATGTTGAAAGTATTGCTGAACAGATGGAAAATCCGGATTTG
AAGAATGTTGGTTCTAGTACTGGGGAGAAAAATGAAAGAACTTCAGTGGCTGGAACAGTG
AGAAAATGCTGGCCTAATAGAGTGGCAAAGGAGCAGATTAGTAGGCGGCTTGATGGTAAT
CAGTATCTGTTTTTGCCACCAAATCGTTACATTTTCCATGGCGCTGAGGTATATTCAGAC
TCTGAAGATGACGTCTTATCCTCTAGTTCTTGTGGCAGTAACAGTGATAGTGGGACATGC
CAGAGTCCAAGTTTAGAAGAACCCATGGAGGATGAAAGTGAAATTGAAGAATTCTACAAT
GGCTTAGAAGATGAGCCTGATGTTCCAGAGAGAGCTGGAGGAGCTGGATTTGGGACTGAT
GGAGATGATCAAGAGGCAATTAATGAAGCTATATCTGTGAAACAGGAAGTAACAGACATG
AACTATCCATCAAACAAATCA
>Macaque_SIRT1
ATGGCGGACGAGGCGGCCCTCGCCCTTCAGCCCGGCGGCTCCCCCTCGGCGGCGGGGGCC
GAGAGGGAGGCCGGGTCGCCCCCCGCCGGGGAGCCGCTCCGCAAGAGGCCGCGGAGAGAT
GGTCCTGGCCTCGAGCGGAGCCCGGGCGAGCCCGGTGGGGCAGCCCCAGAGCGTGAGGTG
CCGGCGGCCGGGGGCTGCCCGGTTGCGGCGGCGGCGCTGTGGCGGGAGGCAGAGGCGGCG
GCGGCGGGCGGGGAGCAAGAGGCCCAGGCGACTGCGGCGGCTGGGGAAGGAGACAATGGG
CCGGGCCTGCAGGGCCCATCTCGGGAGCCACCGCTGGCCGACAGCTTCTACGACGAAGAC
GACGACGACGAGGGCGAGGAGGAGGAAGAGGCGGCGGCGGCGGCGATTGGGTACCGAGAT
AACCTTCTGTTCGGTGATGAAATTATCACTAATGGTTTTCATTCTTGTGAAAGTGATGAG
GAGGATAGAGCCTCACATGCAAGCTCTAGTGACTGGACTCCAAGGCCACGGATAGGTCCA
TATACTTTTGTTCAGCAACATCTTATGATTGGCACAGATCCTCGAACAATTCTTAAAGAT
TTATTGCCGGAAACAATTCCTCCACCTGAGTTGGATGATATGACACTGTGGCAGATTGTT
ATTAATATCCTTTCAGAACCACCAAAAAGGAAAAAAAGAAAAGATATTAATACAATTGAA
GATGCTGTGAAATTACTGCAAGAGTGCAAAAAAATTATAGTTCTAACTGGAGCTGGGGTG
TCTGTTTCATGTGGAATACCTGACTTCAGGTCAAGGGATGGTATTTATGCTCGCCTTGCT
GTAGACTTCCCAGATCTTCCAGATCCTCAAGCGATGTTTGATATTGAATATTTCAGAAAA
GATCCAAGACCATTCTTCAAGTTTGCAAAGGAAATATATCCTGGACAATTCCAACCATCT
CTCTGTCACAAATTCATAGCCTTGTCAGATAAGGAAGGAAAACTACTTCGCAACTATACC
CAGAACATAGACACGCTGGAACAGGTTGCGGGAATCCAGAGGATAATTCAGTGTCATGGT
TCCTTTGCAACAGCATCTTGCCTGATTTGTAAATACAAAGTTGACTGTGAAGCTGTACGA
GGAGATATTTTTAATCAGGTAGTTCCTCGATGTCCTAGGTGCCCAGCTGATGAACCGCTT

GCTATCATGAAACCAGAGATTGTGTTTTTTGGTGAAAATTTACCAGAACAGTTTCATAGA
GCCATGAAGTATGACAAAGATGAAGTTGACCTCCTCATTGTTATTGGGTCTTCCCTCAAA
GTAAGACCGGTAGCACTAATTCCAAGTTCCATACCCCATGAAGTGCCTCAGATATTAATT
AATAGAGAACCTTTGCCTCATCTGCATTTTGATGTAGAGCTTCTTGGAGACTGTGATGTC
ATAATTAATGAATTGTGTCATAGGTTAGGTGGTGAATATGCCAAACTTTGCTGTAACCCT
GTAAAGCTTTCAGAAATTACTGAAAAACCCCCACGAACACAAAAAGAATTGGCTTATTTG
TCAGACTTGCCACCCACACCTCTTCATATTTCAGAAGACTCAAGTTCACCAGAAAGAACT
TCACCACCAGATTCTTCAGTGATTGTCACACTTTTAGACCAAGCAGCTAAGAGTAATGAT
GATTTAGATGTGTCTGAATCAAAAGGTCGTATGGAAGAAAAACCACAGGAAGTACAGACT
TCTAGGAATGTTGAAAGTATTGCTGAACACATGGGAAATCCGGATTTGAAGAATGTTGGT
TCCAGTACTGGGGAGAAAAATGAAAGAACCTCAGTGGCTGGAACAGTGAGAAAATGCTGG
CCTAGTAGAGTGGCAAAGGAGCAGATTAGTAAGCGGCTTGATGGTAATCAGTATCTGTTT
TTGCCACCAAATCGTTACATTTTCCATGGCGCTGAGGTATATTCAGACTCTGAAGATGAC
GTCTTATCCTCTAGTTCTTGTGGCAGTAACAGTGATAGTGGGACATGCCAGAGTCCAAGT
TTAGAAGAACCCATGGAGGATGAAAGTGAAATTGAAGAATTCTACAATGGCTTAGAAGAT
GAGCCTGATATTCCAGAGAGAGCTGGAGGAGCTGGATTTGGGACTGATGGAGATGATCAA
GAGGCAATTAATGAAGCTATATCTATGAAACAGGAAGTAACAGACATGAACTATCCATCA
AACAAATCA
>Medaka_SIRT1
ATGGCGGACGACGAGAGCAGCCACCGAGCGGCCTTCTCTGGAGCCTGCGTTGCGGACGAA
CCCGCCGCTAAGAGATCGAAAATCACTCCACCGGACGACCGCGTCTTCAGATCCATCGAG
GCGGAGCCGTTTTCCTACGTCTCCCCGGCCACGGAGAGCCGGGAGGCGGCGGTGAATTGC
GAGCAGCGGGCCGAGAGGGAGGCGAAGCCGGCGATGGCGGGGGAGCAGGCCGCAGCGGGC
GGAGACAACAATGGGCTGGGACTGCTGGGATGCGAGCCGCCGAACCCAGCCGAGAGGCTG
AGCTTCGGCTTTGGTTCTGCTGAGGGGAGAACGGGCTTCCTCGGGGCTGACGATCTTCCC
TCCAACGGTCTCGCCGTCACACCAGAACACCTTAACGACGACGATGACGGATCCTCTCAT
GCTAGTTCCAGCGACTGGACTCCGCAGCCCCAGATCGGTTCCTACAGTCTGATCCATCAG
CACATCAGAGAGACGGACCCCAGGGCCATTCTGCGGGACCTGCTGCCCGAGACCATACTC
CCACCCGATCTGGACGACATGACCCTGTGGCAGATCATCATCAACATCTCAGAACCTCCC
AAAAGGAAGAAGAGAAAAGACGTGAACACTGTGGAGGACGTGGTCAGACTGCTTCATGAG
AGCAAGAGGATCATGGTTCTGACCGGTGCAGGTGTTTCAGTTTCCTGTGGAATTCCCGAC
TTTCGCTCCAGAGATGGGATTTACGCTCGGCTTGCTGTCGACTTTCCCGACCTTCCCGAT
CCGCAGTCGATGTTCGACATCGAATACTTCCGACGAGACCCCAGACCGTTTTTCAAGTTT
GCTAAGGAGATCTTCCCCGGGCAGTTCCAGCCGTCTCCCTGCCACAGATTTATCGCTATG
CTGGACAAGCAGGAGAAGCTGCTGCGCAATTACACACAAAACATCGACACGCTGGAACAA
GTGGCTGGAGTGCAGAAAATCATCCAGTGTCACGGATCGTTTGCGACGGCTTCCTGTCTC
GTTTGTAAACACAAAGTGGATTGTGAGGCTATAAGGGAGGACATTTTTAATCAGGTTGTT
CCTCGGTGTCCGCGCTGTTCGGACATTCCTCTGGCCATCATGAAACCGGACATTGTGTTT
TTTGGAGAGAACCTCCCAGAGATGTTCCACAGAGCTATGAAGCAGGACAAAGATGAGGTG
GACCTCCTGATCGTCATCGGATCTTCCCTGAAAGTCCGACCCGTGGCCCTCATCCCAAAT
TCCATTCCTCATGAAGTGCCTCAGGTTCTGATCAACCGGGAGCCGCTGCCTCACCTGAAC
TTCGACGTAGAGTTGCTCGGGGACTGTGACGGCATCGTCAACGAGCTTTGTCATCGATTG
GGTGGAGACTTTGAGCCGCTTTGCTTTAACTCTTTAAGACTGGCTGAGATCACAGAGAAG
CCCCCCCGGTTACCCGAAAGGCCTCCAAACGAAACCTCCAGCGATGCCGAGCCGAACGCC

CGTCCTACCGACACCGCAGACCAACCTTCAGAGGAGGCGGAGAGCCCGCAGGACACGGAC
GCCGCTCAGAGAAACGCCACGCCCCCGGAGCCACGCCCTGAAAAGGAGGCGGCAGAGTCT
CCAGAACGACCGGTGGAGGACACGCCCACGGAGGAGGCAGACGCGTTCAAGAGCCAAGTC
TCAACGCTGGAAGTACGGAAACGATGTTGGATGAGTCGGATCAACAGAAGTCCGATCAGC
AAACGGCTGGAAGCGGGCCAGTACCTCTTTCAAGCACCGAACCGGTACATTTTCCACGGA
GCCGAGGTCTACTCCGACTCTGACGACGAGACGTCCAGCTCCTGTGGGAGCGACAGCGAG
GACTCGGAGGGCAGCGTGGATGGCGTGGAGGACGACAGCGATGCAGAGGAGGACGGAGCA
GAAGCGGCGGCAGAGGAAGAGGAGTGCCTCAGAGACACATTACCACACAGTCCAGCCAAT
GAGACGGCAGACGTGCAGATAAACAGAAGCTCTGAACAGACTGAAGGCTTCGCACAGATT

>Mouse_SIRT1
ATGGCGGACGAGGTGGCGCTCGCCCTTCAGGCCGCCGGCTCCCCTTCCGCGGCGGCCGCC
ATGGAGGCCGCGTCGCAGCCGGCGGACGAGCCGCTCCGCAAGAGGCCCCGCCGAGACGGG
CCTGGCCTCGGGCGCAGCCCGGGCGAGCCGAGCGCAGCAGTGGCGCCGGCGGCCGCGGGG
TGTGAGGCGGCGAGCGCCGCGGCCCCGGCGGCGCTGTGGCGGGAGGCGGCAGGGGCGGCG
GCGAGCGCGGAGCGGGAGGCCCCGGCGACGGCCGTGGCCGGGGACGGAGACAATGGGTCC
GGCCTGCGGCGGGAGCCGAGGGCGGCTGACGACTTCGACGACGACGAGGGCGAGGAGGAG
GACGAGGCGGCGGCGGCAGCGGCGGCGGCAGCGATCGGCTACCGAGACAACCTCCTGTTG
ACCGATGGACTCCTCACTAATGGCTTTCATTCCTGTGAAAGTGATGACGATGACAGAACG
TCACACGCCAGCTCTAGTGACTGGACTCCGCGGCCGCGGATAGGTCCATATACTTTTGTT
CAGCAACATCTCATGATTGGCACCGATCCTCGAACAATTCTTAAAGATTTATTACCAGAA
ACAATTCCTCCACCTGAGCTGGATGATATGACGCTGTGGCAGATTGTTATTAATATCCTT
TCAGAACCACCAAAGCGGAAAAAAAGAAAAGATATCAATACAATTGAAGATGCTGTGAAG
TTACTGCAGGAGTGTAAAAAGATAATAGTTCTGACTGGAGCTGGGGTTTCTGTCTCCTGT
GGGATTCCTGACTTCAGATCAAGAGACGGTATCTATGCTCGCCTTGCGGTGGACTTCCCA
GACCTCCCAGACCCTCAAGCCATGTTTGATATTGAGTATTTTAGAAAAGACCCAAGACCA
TTCTTCAAGTTTGCAAAGGAAATATATCCCGGACAGTTCCAGCCGTCTCTGTGTCACAAA
TTCATAGCTTTGTCAGATAAGGAAGGAAAACTACTTCGAAATTATACTCAAAATATAGAT
ACCTTGGAGCAGGTTGCAGGAATCCAAAGGATCCTTCAGTGTCATGGTTCCTTTGCAACA
GCATCTTGCCTGATTTGTAAATACAAAGTTGATTGTGAAGCTGTTCGTGGAGACATTTTT
AATCAGGTAGTTCCTCGGTGCCCTAGGTGCCCAGCTGATGAGCCACTTGCCATCATGAAG
CCAGAGATTGTCTTCTTTGGTGAAAACTTACCAGAACAGTTTCATAGAGCCATGAAGTAT
GACAAAGATGAAGTTGACCTCCTCATTGTTATTGGATCTTCTCTGAAAGTGAGACCAGTA
GCACTAATTCCAAGTTCTATACCCCATGAAGTGCCTCAAATATTAATAAATAGGGAACCT
TTGCCTCATCTACATTTTGATGTAGAGCTCCTTGGAGACTGCGATGTTATAATTAATGAG
TTGTGTCATAGGCTAGGTGGTGAATATGCCAAACTTTGTTGTAACCCTGTAAAGCTTTCA
GAAATTACTGAAAAACCTCCACGCCCACAAAAGGAATTGGTTCATTTATCAGAGTTGCCA
CCAACACCTCTTCATATTTCGGAAGACTCAAGTTCACCTGAAAGAACTGTACCACAAGAC
TCTTCTGTGATTGCTACACTTGTAGACCAAGCAACAAACAACAATGTTAATGATTTAGAA
GTATCTGAATCAAGTTGTGTGGAAGAAAAACCACAAGAAGTACAGACTAGTAGGAATGTT
GAGAACATTAATGTGGAAAATCCAGATTTTAAGGCTGTTGGTTCCAGTACTGCAGACAAA
AATGAAAGAACTTCAGTTGCAGAAACAGTGAGAAAATGCTGGCCTAATAGACTTGCAAAG
GAGCAGATTAGTAAGCGGCTTGAGGGTAATCAATACCTGTTTGTACCACCAAATCGTTAC
ATATTCCACGGTGCTGAGGTATACTCAGACTCTGAAGATGACGTCTTGTCCTCTAGTTCC

TGTGGCAGTAACAGTGACAGTGGCACATGCCAGAGTCCAAGTTTAGAAGAACCCTTGGAA
GATGAAAGTGAAATTGAAGAATTCTACAATGGCTTGGAAGATGATACGGAGAGGCCCGAA
TGTGCTGGAGGATCTGGATTTGGAGCTGATGGAGGGGATCAAGAGGTTGTTAATGAAGCT
ATAGCTACAAGACAGGAATTGACAGATGTAAACTATCCATCAGACAAATCA
>Naked_mole_rat_SIRT1
ATGGCGGACGAGGCGGCGCTCACCCTTCAGCCCGGCTCCCCCTCGGCAGCGGCGGCCGAG
AGGGAGGCCGCGTCGCCGCCTGCCGGGGAGCCGCTCCGCAAGAGGCCGCGGAGGGAAGGG
TCCGTCCCTGGGCGGAGCCCAAGCGAGCCCAGCGGGGCGGCCGCCGGGGGCTGCCCGGCG
GCGGCCGCGGCGCTGTGGCGGGAGGCGGCGGCCGTGGCCGGCGGGGAGCGAGAAGCCCAG
GGGACAGCCGGGGTAGGAGAAGGAGACAATGGCCGGGCCTGCAGGGCCTAGCCTTGGAG
CTGCCGCCGGGCGACGACTTCGACGACGAGGACGACGACGACGACGACGACGACGACGAC
GAGGGTGAGGAGGAGGAAGAGGCGGCGATTGGGTACCGAGATAACCTTCTGTTAAGTGAT
GACATTATCACCAATGGCTTTCATTCCTGTGAAAGTGATGACGATGACAGGGCTTCGCAT
GCCAGCTCTAGTGACTGGACTCCAAGGCCGAGGATAGGTCCATATACTTTTGTTCAGCAA
CATCTCATGATTGGCACAGATCCTCGAACAATTCTTAAAGATTTACTACCAGAAACAATT
CCACCACCTGAACTAGATGACATGACATTGTGGCAGATTGTCATTAATATCCTTTCAGAA
CCACCGAAAAGGAAAAAAAGAAAAGATATTAATACAATTGAAGATGCTGTGAAATTACTG
CAAGAGTGCAAAAAAATAATAGTTCTAACTGGAGCTGGGGTATCTGTTTCATGTGGAATA
CCTGACTTCAGGTCAAGAGATGGTATTTATGCTCGCCTTGCTGTTGACTTCCCAGATCTT
CCAGATCCTCAAGCGATGTTTGATATTGAATATTTCAGAAAAGATCCAAGACCATTCTTC
AAGTTTGCAAAGGAAATATATCCTGGACAGTTCCAACCGTCTCTCTGTCATCAGTTCATA
GCCTTATCAGATAAAGAAGGAAAACTACTTCGCAACTATACCCAGAATATAGATACCTTG
GAACAAGTTGCAGGAATCCAAAGGATAATTCAATGTCATGGTTCCTTTGCAACAGCATCT
TGCTTGATTTGTAGATATAAAGTTGACTGTGAAGCTGTACGAGGAGATATTTTTAATCAG
GTAGTCCCTCGATGTCCTAGGTGCCCACCTGATGAACCACTTGCTATCATGAAGCCAGAG
ATCGTCTTTTTTGGTGAAAATTTACCAGAACAGTTTCATAGAGCCATGAAGTATGACAAA
GATGAAGTTGATCTCCTCATTGTTATTGGGTCTTCCCTGAAAGTGAGACCAGTAGCACTA
ATTCCAAGTTCCATACCCCATGAAGTGCCTCAGATATTAATTAATCGGGAACCTCTGCCT
CATCTGCATTTTGATGTAGAGCTTCTTGGAGACTGTGATGTCATAATTAATGAGTTGTGT
CATAGGTTAGGTGGTGAATATGCCAAACTTTGTTGTAACCCCGTAAAGCTTTCAGAAATT
ACTGAGAAACCTCCACGAACACAGAAAGAGTTGGTTCATTTGTCACAGTTGCCACCAACA
CCTCTTCATATTTCAGAAGACTCAAGCTCACCAGAAAGAACTTCACCTGATTCTTCAGTG
ATTGTTAAACTTTTAGACCAAGCAACCAAGAGCAGTGTGGATGATTTAGATGGAGCTGAA
TCAAAAGGCTATGTAGAAGAAAAACCACAGGAAGTACAGGCTTCTACTAGGAATGCTGAG
AGTATTAATGTGGAAAATCCAGATCTGAAGGACAGTTCCAATACTGCAGAGAAAAGTGGA
AGAACTTCAGTTGCTGAAACAGTGAGAAAGTGCTGGCCCAATAGACTTGCAAAGGAACAG
ATTAGTAAGCGGCTTGATGGTAATCAATATCTGTTTTTACCACCAAATCGTTACATTTTC
CATGGCGCTGAGGTGTATTCAGACTCTGAAGATGACGTATTGTCCTCTAGTTCTTGTGGC
AGTAACAGTGATAGTGGCACATGCCAGAGTCCAAGTTTGGAAGAGCCCTTGGAGGATGAA
AGTGAAATTGAAGAGTTCTACAATGGCTTGGAGGAGGATCCTGATGCTCCAGAGATCACT
GGAGGAACTGGATTTGGGGCCAATGGAGGTGATCAAGAGGCAGTTAATGAAGCTATATCC
ACAAAACAGGAAGTAACAGATAGGAACTATCCATCAAACAAATCA
>Opossum_SIRT1
ATGGCGGATCAGGCGGCGCTCGCCCTGGAGCCCAGCGCAGGCGGCGCAGGTCCCGCGGAG

CCCGGCGGGGAGCCGCTCAGCAAGCGGCAGCGCAGGGACGGACCGGGCCAGGGCCCGGGG
GCGGAGCGGGCTGTCGGGGCGGGGAGCCCGGGCCCGGAGGCGGCGGCCGCCCCACTCGGA
GAGGCCTCGGAGGCAGCCGCCCCCGGCGGGGACAATGGGCCCGGCCCGCGGAGCCAGCGG
GGCCTGCCCCGGGAGCCGCCGCCCGCGCCGCCGGACGACGACGACGACGACGAGGAGGAG
GACGACGACGAGGAGGAGGAGGAAGAGGGGGGCGAGGACGAGGACGAGGCGGCGGCGGCG
ATTGGCTACCGAGAAAATCTCTTCAGTGATGAAACTCTTGCCAATGGCTTTCATTCCTGT
GATAGTGATGAGGATGACCGTGCCTCTCACGCAAGCTCCAGTGACTGGACTCCCAGACCT
CGAACAGGTCCTTACACTTTTGTGCAGCAGCATCTCATGATTGGCACAGATCCTCGGACG
ATCCTGAAGGATCTGCTGCCAGAAACAATCCCACCACCTGAGCTAGATGATATGACTCTC
TGGCAGATTGTTATCAACATCCTTTCAGAACCACCAAAAAGGAAAAAGAGAAAAGATATT
AATACAATTGAGGATGCTGTGAAGTTATTGCAGGAGTGCAAAAAGATCATTGTTCTCACT
GGAGCTGGGGTATCTGTTTCCTGTGGAATACCTGACTTCAGATCAAGAGATGGTATTTAT
GCCCGCCTGGCAGTAGATTTCCCCGACCTCCCTGATCCTCAGGCAATGTTTGACATTGAA
TATTTCAGAAAGGATCCAAGACCATTTTTCAAGTTTGCAAAGGAAATCTATCCTGGGCAA
TTCCAGCCATCTCTCTGCCATAAATTTATAGCCTTGTCCGATAAAGAAGGAAAACTACTT
CGAAACTACACTCAGAACATAGATACACTGGAACAAGTTGCAGGGATTCAGAGGATAATT
CAGTGTCATGGTTCCTTTGCAACAGCTTCTTGCTTGATTTGTAAATACAAGGTGGATTCT
GAAGCTGTACGTGAAGATATTTTTAATCAGGTGGTTCCTCGATGTCCCCGGTGTCCTGCT
GACGAGCCACTTGCCATCATGAAGCCAGAGATTGTGTTCTTTGGGGAAAACTTACCAGAG
CAGTTTCACAGAGCCATGAAGTATGACAAGGATGAAGTCGATCTCCTCATTGTTATTGGG
TCTTCCCTGAAAGTAAGACCAGTAGCGCTGATTCCGAGTTCCATACCCCACGAAGTGCCT
CAAATCCTGATTAACCGGGAACCTCTGCCTCACCTGCACTTTGACGTCGAGCTTCTGGGG
GACTGTGACGTCATCATCAACGAGCTCTGCCAGCGGCTCGGCGGCGAGTACGCCCGGCTC
TGCTCCAACCCGACGAGGCTTTCGGAGATCACGGAAAAGCCTCCACGACCGCAGAGGGAG
CTGGGCGCCCGCTGGGGAGCGCTGCCGCCCACGCCGCTCCACGTTTCCGAAGACTCTAGT
TCCCCCGGCAGAACTTCCCCGCCAGACTCTTGGGCAGGTGAGAGGAGAGCCGGGGAGGCC
GACGATGGGGCCGCATCACGGGGGAGCTGCAGAGCGGACAAGCCGCCACCGGAGGTTCAC
CCTGCGAGCATCACGGAGCAGCTGGAGGATGCCGGAGCCAACGGTGGGGAGAAAAACGAA
AGAACTAACGTCGTGGAAACCCTGAGGAAGTGCTGGCCTAACAGGCTCGCCAAAGAGCAG
ATCAGTAAGCGGCTTGATGGTAACCAGTACCTCTTTTTACCGCCAAATCGCTATATTTTT
CATGGCGCCGAGGTGTACTCAGACTCTGAAGACGACGTCCTGTCCTCCAGCTCTTGTGGC
AGCAACAGTGACAGCGGCACCTGCCGGAGCCCCAGCCTAGAGGAGCCCATGGAGGACGAG
AGCGAGATGGAAGAATTCTACAACGGCCTGGAGGAGGCCGACGGTCCCGAGAGAGGCGGC
CCTGCGTGCGCGTGCGACAAAGAGGACCAGCGCGCCGTCGGCGAAGCCGCGTCCATCACG
GACGAGGCCGCTGCGATCGACCATCCGTCCAGCAGGTTA
>Orangutan_SIRT1
ATGGCGGACGAGGCGGCCCTCGCCCTTCAGCCCGGCGGCTCCCCCTCGGCGGCGGGGGCC
GAGAGGGAGGCCGCGTCGTCCCCCGCCGGGGAGCCGCTCCGCAAGAGGCCGCGGAGAGAC
GGTCCCGGCCTCGAGCGGAGCCCGGGCGAGCCCGGTGGGGCGGCCCCAGAGCGTGAGGTG
CCGGCGGCGGCCGGGGGCTGCCCGGCTGCGGCGGCGCTGTGGCGGGAGGCGGAGGCA
GAGGCGGCGGCGGGCGGGGAGCAAGAGGCCCAGGCGACTGCGGCGGCTGGGNAAGGAGAC
AATGGGCCGGGCCTGCAGGGCCCATCTCGGGAGCCACCGCTGGCCGACAACTTCTACGAC
GAAGACGACGACGACGACGAGGGCGAGGAGGAGGAAGAGGCGGCGGCGGCGGCGGCGATT
GGGTACCGAGATAACCTTCTGTTCGGTGATGAAATTATCACTAATGGTTTTCATTCCTGT

GAAAGTGATGAGGAGGATAGAGCCTCACATGCAAGCTCTAGTGACTGGACTCCGAGGCCA
CGGATAGGTCCATATACTTTTGTTCAGCAACATCTTATGATTGGCACAGATCCTCGAACA
ATTCTTAAAGATTTATTGCCGGAAACAATTCCTCCACCTGAGTTGGATGATATGACACTG
TGGCAGATTGTTATTAATATCCTTTCAGAACCACCAAAAAGGAAAAAAAGAAAAGATATT
AATACAATTGAAGATGCTGTGAAATTACTGCAAGAGTGCAAAAAAATTATAGTTCTAACT
GGAGCTGGGGTGTCTGTTTCATGTGGAATACCTGACTTCAGGTCAAGGGATGGTATTTAT
GCTCGCCTTGCTGTAGACTTCCCAGATCTTCCAGATCCTCAAGCGATGTTTGATATTGAA
TATTTCAGAAAAGATCCAAGACCATTCTTCAAGTTTGCAAAGGAAATATATCCTGGACAA
TTCCAACCATCTCTCTGTCACAAATTCATAGCCTTGTCAGATAAGGAAGGAAAACTACTA
CGCAACTATACCCAGAACATAGACACGCTGGAGCAGGTTGCAGGAATCCAAAGGATAATT
CAGTGTCATGGTTCCTTTGCAACAGCATCTTGCCTGATTTGTAAATACAAAGTTGACTGT
GAAGCTGTACGAGGAGATATTTTTAATCAGGTAGTTCCTCGATGTCCTAGGTGCCCAGCT
GATGAACCGCTTGCTATCATGAAACCAGAGATTGTGTTTTTTGGTGAAAATTTACCAGAA
CAGTTTCATAGAGCCATGAAGTATGACAAAGATGAAGTTGACCTCCTCATTGTTATTGGG
TCTTCCCTCAAAGTAAGACCAGTAGCACTAATTCCAAGTTCCATACCCCATGAAGTGCCT
CAGATATTAATTAATAGAGAACCTTTGCCTCATCTGCATTTTGATGTAGAGCTTCTTGGA
GACTGTGATGTCATAATTAATGAATTGTGTCATAGGTTAGGTGGTGAATATGCCAAACTT
TGCTGTAACCCTGTAAAGCTTTCAGAAATTACTGAAAAACCTCCACGAACACAAAAAGAA
TTGGCTTATTTGTCAGAGTTGCCACCCACACCTCTTCATATTTCAGAAGACTCAAGTTCA
CCAGAAAGAAGTTCACCACCAGATTCTTCAGTGATTGTCACACTTTTAGACCAAGCAGCT
AAGAGTAATGATGATTTAGATGTGTCTGAATCAAAAGGTTGTATGGAAGAAAAACCACAG
GAAGTACAAACTTCTAGGAACGTTGAAAGTATTGCTGAACAGATGGAAAATCCGGATTTG
AAGAATGTTGGTTCCAGTACTGGGGAGAAAAATGAAAGAACTTCAGTGACTGGAACAGTG
AGAAAATGCTGGCCTAATAGAGTGGCAAAGGAGCAGATTAGTAAGCGGCTTGATGGTAAT
CAGTATCTGTTTTTGCCACCAAATCGTTACATTTTCCATGGCGCTGAGGTATATTCAGAC
TCTGAAGATGACGTCTTATCCTCTAGTTCTTGTGGCAGTAACAGTGATAGTGGGACATGC
CAGAGTCCAAGTTTAGAAGAACCCATGGAGGATGAAAGTGAAATTGAAGAATTCTACAAT
GGCTTAGAAGATGAGCCTGATGTTCCAGAGAGAGCTGGAGGAGCTGGATTTGGGACTGAT
GGAGATGATCAAGAGGCAATTGATGAAGCTATATCTATGAAACAGGAAGTAACAGACATG
AACTATCCATCAAACAAATCA
>Western_painted_turtle_SIRT1
ATGGCGGACGAGACGACTCTGCTCCTCCAACCGCGTAGCGGCCGCTGCGCTGCGGCCGAG
GGTGCCGAGCCCCTTCCTAAGCGCCAGCGCCTGGACTCGGAAGACGGCGGCGGGCGGGGC
TCGGGGAAGGGCCTAGGCCCAGAACGAGGGGTTGGGACGGCGCCCACCACGGGCTCCGCG
ACTAGGGCGGCGGCGGTGAGGCTGCAGGGGGAGGTAGCGGCGGGCTGGAACGACGGGGAG
GACGGGGCCGGGCTGCGGGGCCTGCCCCGCGAGGAGCCGCCTCCGCAGCAGCAGCAACAG
GGGNGAGGGGCGGAGACGGCGCCCAGTGGAGACGCGGTGGAGACGGCCATTGGCTACAGA
CGGTCGCAGTGTTCACACGGGTCGGCCGAGGCGGCTGCCCCGCATCCTGATGACATCCTT
TTTAGTGATGAAATCATAGCCAATGGTTTCCATTCCTGTGATAGTGATGAAGATGACAGA
GCCTCACGTGCAAGCTCTAGTGACTGGACTCCAAGACCACGTATAGGTCCCTACACTTTT
GTTCAACAACATCTAATGATAGGCACAGACCCACGGACAATTCTGAAGGATTTGCTACCA
GAAACGATCCCTCCTCCTGAACTGGATGATATGACTCTGTGGCAGATTGTTATAAACATT
CTTTCAGAACCACCAAAAAGGAAAAAACGAAAAGATATTAATACTATTGAGGATGCTGTG
AAACTTTTACAAGAGTGCAAAAAAATAATTGTCTTGACTGGGGCTGGGGTGTCTGTTTCT

TGTGGAATACCTGACTTTCGATCAAGAGATGGCATCTATGCACGCCTTGCAGTAGATTTT
CCAGACCTTCCAGATCCTCAAGCGATGTTTGATATAGAATACTTCAGAAAGGATCCAAGA
CCATTTTTTAAATTTGCAAAGGAAATATATCCTGGACAGTTCCAACCATCTCTCTGTCAT
AAGTTCATAGCTTTGATGGACAAAGAAGGAAAACTACTTCGCAACTATACTCAGAACATA
GACACACTGGAACAGGTTGCAGGAATCCAAAGGATAATTCAGTGTCATGGTTCCTTTGCA
ACAGCTTCCTGCCTCATCTGTAAATACAAAGTTGACTGTGAAGCTGTTCGAGGAGACATT
TTTAATCAGGTTGTTCCAAGATGTCCCAGGTGTCCACCTGATGAACCACTTGCCATCATG
AAGCCAGAGATAGTGTTCTTTGGAGAAAACTTACCTGAGCAGTTCCATAGGGCCATGAAG
TATGACAAAGATGAAGTTGATCTCCTTATTGTTATTGGGTCTTCACTGAAAGTAAGACCA
GTAGCATTGATTCCAAGTTCCATCCCCCATGAAGTGCCTCAGATCTTAATTAATAGGGAA
CCTTTGCCTCATCTACACTTTGATGTGGAGCTTCTTGGAGACTGTGATGTCATTATTAAT
GAATTATGTCAAAGGCTAGGTGGTGAATATACAAAACTTTGCTATAACTCTGTAAAACTT
TCAGAAATAACAGAAAAGCCCTCACGAATGCACAAGGAATTTGAAATGCATTCAGCTGAG
TTACCACCTACCCCTTTAAAAGTTTCTGAAGACTCTAGTTCACCGAACAGAATGACACCA
CCAGATCCTTTAGTGGTACTTTCAGAGCATCCAACTGAAGGTCAGGCAGAAAATGCTGAT
CGTTCCTCAGAATCTAAAGGGAATTGCATGGAGGATAAATTGCAAGAAGTACAAGCATGC
TCAGAAAACTCTGAAAGTATTACTGGCCAGCTAATGAACTCAGAACATATGAAGGATAAT
AGATCTAACAAGGAGGAAAATAAAGAGAAAAATGAAATAACTTCATCAGTTGAAACATTG
AGAAAATGTTTGGCAAACAGATTTGCAAAAGAACAAATTAGCAAGCGGCTTGATGGTACT
CAATACTTATTTTTACCACCAAATCGCTACATTTTCCATGGGGCTGAGGTATACTCAGAC
TCTGAAGACGATGTCCTATCTTCTAGTTCTTGTGGAAGTAGTAGTGATAGTGGTTCTTGT
CATAGTCCAAGCTTAGATGTTGAAGATGAAAGTGAGATTGAAGAATTCTATAATGGCATG
GAGGAGGAGGATGCTCCAGAGAGAGAAGAGGAGACTGGATTTGGGGAAGATGGAATTGTT
CAAGATGCAGTTGGTGAACCAGCTTATATAAATGAAGCTGCAGGAATTGATCGTCCATCA
AACAAATTG
>Panda_SIRT1
ATGGCGGACGAGGCGGCGCTCGCCCTTCAGCCCGGCGGCTCCCCCTCGGCGGGACTGCGC
ATCCCCTCCTCCCTCTCGGGGCCTCTTCTGGTGTGGTGTTGGGGGGGGTTCTCTGAGGCG
GGCTGGGGGCTCAGATCTGGTTCTGCGGCGTCCCTCTCCCCACCGCGGGCCTCAACTGTG
CGCCTTCACGGCCGGTGGCCGGGCTGCGGGGGAGCGGCCGATGTACTCCCTCCCTCGGCT
CCGTCTTTCCCTCCCTCCGCGGCCCGTCTGGGCGGCCTCGCGCTTTGCGCAGCCGCCGGG
CGGGGAAGCTCCCGCATTACTTTGGGTTCTTTTGCAGATGACCTTCTGTTTGATGATGAA
ATAATCACCAATGGTTTTCATTCCTGTGAAAGTGATGAGGATGATAGAGCCTCACATGCA
AGCTCTAGTGACTGGACTCCAAGGCCACGGATAGGTCCATATACTTTTGTTCAGCAACAT
CTCATGATTGGCACAGATCCTCGAACAATTCTTAAAGATTTACTACCAGAAACAATTCCT
CCACCTGAATTGGATGATATGACACTGTGGCAGATTGTTATTAATATCCTTTCAGAACCA
CCAAAAAGGAAAAAAAGAAAAGATATTAATACAATTGAAGATGCTGTGAAATTACTGCAA
GAGTGCAAAAAAATAATAGTTCTAACTGGAGCTGGGGTTTCTGTTTCTTGTGGAATACCT
GACTTCAGGTCAAGAGATGGTATTTATGCTCGCCTTGCAATAGACTTTCCAGACCTTCCA
GATCCTCAAGCAATGTTTGATATTGAATATTTCAGAAAAGATCCAAGACCATTCTTCAAG
TTTGCAAAGGAAATATATCCTGGACAGTTCCAACCATCTCTCTGTCACAAATTCATAGCC
TTGTCAGATAAAGAAGGAAAACTGCTTCGGAACTATACTCAGAACATAGATACACTGGAA
CAGGTTGCAGGAATCCAAAGGATAATTCAATGTCATGGTTCCTTTGCAACAGCATCTTGT
CTGATTTGTAAATACAAAGTAGACTGTGAAGCTGTACGAGGAGATATTTTTAATCAGGTG

GTTCCTCGTTGTCCTAGGTGCCCAGCTGATGAGCCACTTGCTATCATGAAACCAGAGATA
GTCTTTTTTGGTGAAAATTTACCAGAGCAGTTTCATAGAGCCATGAAGTATGACAAAGAT
GAAGTTGATCTCCTCATTGTTATTGGGTCTTCCCTGAAAGTAAGACCAGTAGCACTAATT
CCAAGTTCCATACCCCATGAAGTGCCTCAGATATTAATAAATAGAGAACCTTTGCCTCAT
CTGCATTTTGATGTAGAGCTTCTTGGAGACTGTGATGTTATAATAAATGAATTATGTCAT
AGGTTAGGTGGTGAATATGCCAAACTTTGCTGCAACCCTGTAAAGCTTTCAGAAATTACT
GAAAAACCTCCACGAACACAAAAAGAGTTGGCTCATTTGGCAGAGTTGCCACCGACACCT
CTTAATATTTCAGAAGACTCAAGTTCACCGGAAAGAACTTCACCACCTGATTCTTCAGTG
ATTGTTACACTTTTAGACCAAGCACGTAAGAGTAATGTTGATGATCCTGGTGTGTCCAAA
TCAAAAGATTGTATGGAAGAAAAGTCACAGGAAGGACAGAATTCTAGGAACATTGAAAGT
GTTACTGAACAGTTGGAAAGTCCAGATTTGAAGAATGTTGGCTCTAACACTGGGGAGAAA
AATGAAAGAACTTCAGTTGCTGACACAGTGAGGAAGTGCTGGCCAGCTAGACTTGCAAAG
GAGCAAATTAGCAAACGGCTTGATGGTAACCAGTATCTGTTCTTACCACCAAATCGTTAC
ATTTTCCATGGCGCTGAGGTATATTCAGACTCTGAAGATGACGTCTTATCCTCTAGTTCT
TGTGGTAGTAACAGTGATAGTGGAACGTGCCAGAGTCCAAGTGTAGAAGAACACTTGGAG
GATGAAAGTGAGATTGAAGAATTTTACAATGGTTTGGAAGATGAAGCTGATGTTAATGAG
AGAGCTGGAGGAACTGGATTTGGAATTGCTGGAGGTGATCATGAGGCAGTCAGTGAAGCT
ATATCCATGAAACAGGAAGTAACAGACATTAACTATCCATCAAACAAATCA
>Piegon_SIRT1
ATGATTCCTTATAACTTCCTTTTTAGTGATGAAATCATAGCCAATGGTTTTCACTCCTGT
GACAGTGATGAAGATGACAGAGCCTCACATGCAAGTTCTAGTGACTGGACCCCAAGACCA
CGTATAGGTCCCTACACTTTTGTGCAGCAGCATCTCATGTTAGGTACAGACCCACGGACC
ATTCTGAAAGACCTGCTGCCAGAAACCATCCCTCCACCGGAACTGGATGATATGACGCTG
TGGCAAATTGTTATAAACATTCTTTCAGAACCACCAAAAAGGAAAAAACGGAAAGATGTT
AATACTATCGATGATGCCGTGAAACTTTTACAAGAATGCAAAAAAATAATGGTCCTGACT
GGAGCTGGGGTGTCAGTGTCTTGTGGAATACCTGACTTTAGATCCAGAGACGGCATCTAT
GCACGCCTTGCTGTAGACTTCCCAGACCTGCCAGATCCTCAAGCAATGTTTGATATAGAA
TACTTCAGAAAGGACCCCAGGCCATTTTTTAAGTTTGCAAAGGAAATTTATCCAGGACAG
TTCCAGCCATCTCTCTGTCACAAGTTCATAGCTTTGATGGACAAAGAAGGAAAACTACTT
CGCAACTATACTCAGAACATAGACACATTGGAACAGGTTGCAGGAATCCAAAGGATAATA
CAGTGTCATGGTTCCTTTGCAACAGCTTCCTGCCTGATCTGTAAATACAAAGTTGATTGT
GAAGTTGTCCGAGGAGACATTTTCAATCAGGTTGTCCCTAGATGTCCCAGATGTCCACCT
GATGAGCCCCTCGCTATCATGAAGCCGGATATCGTGTTCTTTGGAGAGAACTTGCCTGAG
CAGTTCCATCGCGCCATGAAGTACGACAAAAATGAAGTTGATCTCCTTATTGTCATTGGG
TCTTCACTGAAAGTAAGACCAGTAGCATTGATTCCAAGTTCCATCCCCCATGAAGTGCCT
CAGATCTTAATTAATAGGGAACCTTTGCCTCATCTACACTTTGACGTGGAGCTTCTTGGA
GACTGTGATGTTATTATTAACGAATTATGTCAACGGCTAGGTAGCGAATATACAAAACTT
TGCTACAACTCAGTAAAACTTTCCGAAATAACAGAAAAGCCTCCTCGAACGCACAAGGAG
CTGGAAATGCACTCGGCCGAGCTCCCACCCACCCCTCTCAACATTTCGGAAGACTCTAGT
TCACCAGAAGGAACGACCCCACCAGACGCTTCGGTTGTGTCTGCAGAACTGCCAGCAGAA
TGCAAGGCAGAAAACTGCGATCCTGCCTCGGAAACTAAAGGGACCTGCACAGAGGAAAAG
CTTCAGGACACACAGACATCGCCAGAAAACCTTGACAATCCCACTAGTGAATTAATGAAC
TCTGAAACAATGAAGGAAAATGGATCTAACAGTGGAGAAAATAAAGAAAAAAATGAAATA
TTGAAGAAGTGCTGGGTAAACAGACCTGCAAAAGAACAGATTAGCAAAAGGCTGGATGGA

AATTTTTTTGTAATTCATAATAGGTTTTATAACCAG
>Pig_SIRT1
ATGGCGGACGAGGCGGCGCTCGCCCTTCAGCCCGGCGGCTCCCCCTCGGCGGTGGCGGCT
GAGAGGGAGGCCCCGTCGCCCCCCGCCGGGGAGCCGCTCCGCAAGAAACCGCGGAGAGAC
GGCCCGGGCGTCGGGCGGAGCCCGGGCGAGCCTGGTGGGGCGGCCTTGGAGCGTGAGCTG
CCGGCGGCGGCGGGCGGCTGCCCGGCGGCGGCGGTGCTGTGGCGGGAGACCGCTGCGGGC
GGGGAGCGGGAGGCCCAGGCGGCTGCGGCGGCAGGAGAAGGAAACAATGGGCCGGGCTTA
CAGGGCCTATCCCGGGAGGCGCCCCCGGCCGACGACTTCTACGACGACGACGACGACGAC
GAGGGCGAGGAGGAGGAAGAGGCGGCGGCAGCGGCGGCTATTGGGTACCGAGATAACCTT
CTGTTTGGTGATGAAATCGTCACCAATGGTTTCCATTCTTGTGAAAGTGATGAGGATGAT
AGAGCCTCACATGCCAGTTCTAGTGACTGGACTCCAAGACCACGGATAGGTCCATATACT
TTTGTTCAGCAACATCTCATGATTGGCACAGATCCTCGAACAATTCTTAAAGATTTGCTA
CCAGAAACAATTCCTCCACCTGAATTGGATGATATGACACTGTGGCAGATTGTTATTAAT
ATCCTTTCAGAACCACCAAAAAGGAAAAAAAGAAAAGATATTAATACAATTGAAGATGCT
GTGAAATTACTACAAGAGTGCAAAAAAATAATAGTTCTAACTGGAGCTGGGGTTTCTGTT
TCTTGTGGAATACCTGACTTCAGGTCAAGAGATGGCATTTATGCACGCCTTGCAGTAGAC
TTTCCAGACCTTCCTGATCCTCAAGCAATGTTTGATATTGAATATTTCAGAAAAGATCCA
AGACCATTCTTCAAGTTTGCAAAGGAAATATATCCTGGACAGTTCCAGCCATCTCTCTGT
CACAAATTCATAGCCTTGTCAGATAAGGAAGGAAAACTGCTTCGCAACTATACTCAGAAC
ATAGATACACTGGAACAGGTTGCAGGAATCCAGAGGATAATCCAGTGTCATGGTTCCTTT
GCAACAGCATCTTGCCTGATTTGTAAATATAAAGTTGACTGTGAAGCTGTACGAGGAGAT
ATTTTTAATCAGGTGGTTCCTCGATGTCCTAGGTGCCCAGCTGATGAACCACTTGCTATC
ATGAAACCAGAGATTGTCTTTTTTGGTGAAAATTTACCAGAGCAGTTTCATAGAGCCATG
AAGTATGACAAAGATGAAGTTGATCTTCTCATTGTTATTGGGTCTTCCCTGAAAGTAAGA
CCAGTAGCACTAATTCCAAGTTCCATACCCCATGAAGTGCCTCAGATATTAATTAATAGG
GAACCTTTGCCTCACCTGCATTTTGATGTAGAGCTTCTTGGAGACTGTGATGTCATTATT
AATGAATTGTGTCATAGGTTAGGAGGTGAATATGCCAAGCTTTGCTGCAACCCTGTAAAG
CTTTCAGAAATTACTGAAAAGCCTCCACGAACACAAAAAGAGTTGGCTCACTTGTCAGAG
TTACCACCCACACCTCTTAATATTTCAGAAGGCTCTAGTTCACCAGAAAGAACTTCACCA
CCAGCTTCTTCAGTGACTCTCCCTCTTTTAGACCAAGCAACAAAGAGTAATGTTGATGAT
CTAGATGTGTCTGAGTCAAAAGACTGTGTTGAAGAGAAATCACAGGAAGTACAGACTTCT
AGGAGCATTGAAAGTGTTAAAGAACCGATGGAGAGTCCAGGTTTGAAGAATGTTGCCTGC
AGTAATGGAGAGAAAAATGAAAGAACTTCAGTTGCCGAAACAGTAAGAAAATGCTGGCCA
GCTAGACTTGCAAAGGAGCAGATTAGTAAACGGCTTGATGGTAATCAGTATCTGTTTTTA
CCACCAAACCGTTATATTTTCCATGGCGCTGAGGTATATTCAGACTCTGAAGATGATGTC
TTATCCTCTAGTTCTTGTGGCAGTAACAGTGAGAGTGGAACTTGCCAGAGTCCAAGTCTA
GAAGAACCCATGGAGGATGAAAGTGAGATTGAAGAATTTTACAATGGTTTGGAAGATGAT
GCTGATGTTAATATGCGAGCTGGAGGAACTGGATTTGGAGCTGATGGAAGTGACCAAGAG
GCAGTTAATGAAGCTATATCCATGAAACAGGAAGCAACAGGCGTTAACTATCCATCAAAC
AAATCA
>Platypus_SIRT1
ATGCCCCCAAAGAGGCACTGGCGACCGATTGGGGACGGGGACGGGGGGACTCTGGGTGCC
ACCCGGCCGAGGCCCCCTGCCCAGGAAATCTATCCTGGGCAGTTTCAACCATCTCTGTGC
CACAAGTTTATCGCCTTGTCGGATAAGGAAGGAAAATTACTTCGCAACTACACTCAGAAT

ATAGATACACTGGAACAAGTTGCAGGGATCCAAAGAATAATTCAGTGTCATGGTTCCTTT
GCGACAGCTTCATGCCTGATTTGTAAATACAAAGTTGACTGTGAAGCTGTACGAGGAGAC
ATTTTTAATCAGGTTGTTCCTAGATGTCCCAGATGCCCAGCTGATGAGCCACTTGCCATC
ATGAAGCCAGAGATTGTGTTTTTTGGGGAAAATTTACCAGAGCAATTTCATAGGGCCATG
AAGTATGACAAAGATGAAGTTGACCTCCTCATTGTTATTGGGTCTTCCCTGAAAGTAAGA
CCAGTAGCGCTGATTCCGAGTTCCATACCCCACGAAGTGCCTCAGATCTTGATTAATAGG
GAACCGTTACCTCATCTGCATTTTGATGTAGAGCTTCTTGGAGACTGCGATGTCATCATT
AATGAATTGTGTCAGAGGCTGGGAGGGGAATACACGCAACTCTGCTCCAACCCCGTAAAG
CTCTCGGAAATCACGGAAAAGCCTCCACGCACACACAAGGAGCTTGAAGCTCGGTGGGCC
GCGCTCCCCCCGACCCCACTCCACGTGTCAGAAGGCTCGAGCTCGCCCGACCGAACCTCG
CCGCCAGATGCCTTGGCGGCTCCCGCCCGCCCCGAGCAGATGACTGACAGCCGAGTGGTG
CAAGTCGAATCGGGGGCGGAGGCAGGAGGGTGCCCCGGGGGGGCGGCCCCGCAGGCAGGG
CAGTCATCTTCCGAAAGCGCCGCCGCTGTGAGCCAGCAGTTGGAGGGTTTGGATCGTACG
AAGGACGCCGCTGCCAGCATTGGGGAAAATAAAGAGAGGAATGAAAGAACTTCAGCTGCG
GAAACACTGAGAAAATGCTGGGGCAGTAGGCTTGCAAAAGAGCAGATTAGTAAGCGGCTT
GATGGGAACCAGTACCTGTTTTTACCACCGAATCGCTACATTTTCCACGGGGCTGAAGTA
TACTCCGACTCTGAAGATGACATCCTGTCCTCCAGCTCTTGTGGAAGTAATAGTGACAGT
GGGACCTGCCATAGTCCAAGCTTAGAAGAACCTCTAGAGGATGAAAGTGAGATTGAAGAA
TTCTATAACGGCTTAGAGGATGACGCTGTCGGTCCTGAGAGAGGAGGAGGAGGAGCAGCA
GCTGGATTTGGGGGTGACGATGATGAACAAGAGGCAGTTACCGAAGCCGTGTCTGTGAAA
GTCGAGGCAGCAGAAATGGACCATTCATCAAATGGCTTA
>Rabbit_SIRT1
ATGGCGGACGAGGCGGCGCTCGCCCTTCAGCCTGGCGGCTCCCCGTCGGCTGCGGCGGCC
GAGAGGGAGGCCGCTTCGCCGCCTGCTGGAGAGCCGCTCCGCAAGAGGCCGCGGAGAGAC
GGCCCTGGCCTCGGGTGGAGCCCGGGCGAGTCCGGCGGGCCGGCCCCCGAGCGTGAGGTG
CCGGCGGCGGCGGCGGCCGGGAGCTGCCCGGCTGTGGCGGCGGCGCTGTGGCGGGAGGCG
GAGGCGGCGACGGCGACGGGCGGGGAGCGAGAGGCCCAGGCTATGGTAGCGGCCGGAGGA
GACAATGGGCCTGGCCTGCAGGGCCTACTCCGGGAGCTGCCGCCGGCCGACGATTTCTGC
GAAGACGAAGACGACGACGACGAGGGCGAAGAGGAGGAAGAGGCGGCGGCGGGGATTGGG
TACAGAGATAATCTCTTGTTCGGTGATGAAATTATCACTAATGGCTTTCACTCATGTGAA
AGCGATGACGACGACAGAGCCTCACATGCAAGCTCTAGTGACTGGACTCCAAGGCCACGG
ATAGGTCCGTATACTTTTGTTCAGCAGCATCTCATGATTGGCACAGATCCTCGAACAATT
CTTAAAGATTTACTACCAGAAACAATTCCTCCGCCTGAACTGGATGATATGACACTGTGG
CAGATTGTTGTTAATATCCTTTCAGAACCACCAAAAAGGAAAAAAAGAAAAGATATTAAT
ACAATTGAAGATGCCGTGAAATTACTGCAAGAGTGCAAAAAAGTAATAGTTCTAACTGGA
GCTGGGGTGTCTGTTTCATGTGGAATACCTGACTTCAGGTCAAGAGATGGTATTTATGCT
CGCCTTGCAGTAGACTTCCCAGATCTTCCAGATCCTCAAGCAATGTTTGATATTGAATAT
TTCAGAAAAGATCCTCGGCCATTCTTCAAGTTTGCAAAGGAAATATATCCTGGACAGTTT
CAACCGTCTCTCTGTCACAAATTCATTGCCTTGTCAGATAAGGAAGGAAAACTTCTTCGC
AACTATACCCAGAACATAGACACTCTTGAACAGGTTGCAGGAATCCAAAGGATAATTCAA
TGTCATGGTTCCTTTGCAACAGCATCTTGCCTGATCTGTAAATACAAAGTTGACTGTGAA
GCTGTACGAGGAGATATTTTTAATCAGGTAGTTCCTCGATGTCCTAGGTGCCCAGCTGAT
GAACCACTGGCTATCATGAAACCAGAGATTGTCTTTTTTGGTGAAAACTTACCCGAACAA
TTTCATAGAGCCATGAAGTATGACAAAGATGAAGTTGATCTCCTCATTGTTATTGGGTCT

TCCCTGAAAGTAAGACCAGTAGCACTAATTCCAAGTTCCATACCCCACGAAGTGCCTCAG
ATATTAATTAATAGGGAACCTTTGCCTCATCTACATTTTGATGTAGAGCTTCTTGGAGAC
TGTGATGTCATAATTAATGAACTGTGTCACAGGCTAGGTGGTGAATATGCCAAACTTTGC
TGCAACCCTATAAAGCTTTCAGAAATTACTGAAAAACCTCCACGAACACAAAAAGAGTTG
GTTCATTTGTCAGAGTTGCCACCAACACCTCTACATATTTCAGAAGATTCAAGTTCACCA
GAAAGAACTTCACCACCAAATTCTTCAATGATTGTTAAGCTTATAGACCAAGCAACTAAG
AGTAATGTGGACGATTCAGATGTCTCTCAATCAAAAGTTTGTATGGAAGAAAAATCACAG
GAAGTACAGACTAATCCTAGGAATACTGAAAGTGTCACTGAACAGGTGGAAAATCCGGAT
ATGAAGAGTGTTGGTTCCAATGCTGGGGAGAAAAATGAACGAACCTCAGTTGCTGACACA
GTGAGAAAGTGCTGGCCCAGCAGAATTGCAAAGGAACAGATTAGTAAGCGGCTCGATGGT
AATCAGTATCTGTTTTTACCACCAAATCGTTATATTTTCCATGGCGCTGAGGTATATTCA
GACTCTGAAGATGATGTCTTATCCTCTAGTTCTTGTGGAAGTAACAGTGATAGTGGCACA
TGCCAGAGTCCAAGTTTAGAAGAACCACTGGAGGATGAAAGTGAGATTGAAGAATTCTAC
AATGGCTTGGAAGATGATGTTGATGTTCCAGAGAGACCTGGAGGAACTGGATTTGGGGCT
GATGGAGGTCTTCAAGAGGCAGTTAATGAAGCTGTATCTGTGAAACAGGAAGCAACAGAT
GTCAACTATCCATCAAACAAATCA
>Tetraodon_SIRT1
atgggtcacgtgccctatgcaaatgagccagctgtccgggaggaaggctctgctcggcgg
ccgctctgcgggctgaagatggcggatggcgagaacagtctcggaatggctttcgcaggc
gcctctgatatggaccagcctgtcgcgaaaaggccaaaaatcagtccgctgaccaactat
gggttctcagtcgccaaagcggaccagtttccgtgcctcgccggttctactggaagctgg
gagacggcggtgaattgtgcgcacccagcggggaaagaagcgaagccggcgatggcggta
gaacaggccacggtcgctgcgctaggcggagacaacaatggactggaaatgctggtctcc
gagccgcataaatcagttatgaaacaagaggacagcgctgtgtttgggacgaccgaggag
agcaccgactttcttgagcacgacgagctttcttgcaacggcctggccgtcactccggat
cacatcaacgaggacgatgacaggtcttcccacgccagctccagcgactgggcccctcag
ccccaaatagaatccatcccttcattgcgtactgtctgcatgtgcatattggttgaaaac
gacgcgaatctgagattttcctcgaaattgtgggcaggttcctacagtttcatccagcag
catatcagagagacggatcccagggctattctgagagatctgctgcccgacactgtgctc
ccaccagatttagacgacatgacattgtggcagatcatcatcaatatctccgagcctccg
aaaagaaagaagcgaaaagacgtcaacactttggacgatgtagtgaagctactgaaggaa
agcaaaaggatccttgtattgaccggtgctggggtgtctgtttcttgcggcataccagac
ttccggtccagagatggaatttatgcacgtcttgctgtcgatttccctgatcttccagac
cctcaagcgatgtttgacattgattactttagacgggacccaaggccgtttttaagttt
gccaaggaaatctacccgggtcagttccagccttccctgtgtcacaaattcatatccatg
ctggataagcaggggaagctgctgcggaattatacacaaaatattgacacattagaacaa
gtggctggagttcagcggattatccagtgtcatggttcttttgcaactgcatcctgtctc
atctgtaaacacaaagtagattgtgaggtcataagagaagacatctttaaccaggttgtt
cctcattgttcacgctgcccagatattcctctggcaattatgaagcctgatattgtcttc
tttggagaaaatctaccagaaatgttccacagagccatgaagcaggataaagatgaggtg
gacctcttgattgtcattggttcttcacttaaagttcggccagttgcccttatcccaaac
tccattcctcatgaagtgcctcaggtcttgataaatagagagcagctgcctcacctcaat
tttgacgtggagctactcggagactgtgacgtcattgtcaacgagctctgtcaccagttg
ggtggggaatacgagcagcttcgctccaacgttctaagactccgcgagatcacagagaaa

cctcctcggttaccggaacagtcacctaacgaggccttgtctgctcccagggatgcagct
cacgaggagcaaaagccgcccagtacagactcggtaaccaggccttcgcggaaggcagaa
acactgaatgtcgctgaggcccctgctcatattgttacacctccacagccttgtccaaac
gctcagtgtccaggtgaagaaccgactgagccggtagcgatatgcacagagggctcacca
gcagaagtggccactgaagtaaagaaccaaccctccagccttgaatttcacagacgatgc
tggatgagtcgaatcagccgaagtccactcagcaaacggcttgagacagaccagtacctg
ttcttggcaccgaatcagtacatcttccacggggctgaggtttactcggactctgaagac
gacacgtcgagctccagcggcagtgacagcgatgagtctgaatgcagcgcagatggcgtg
gacgaagaaagtgaactagaggaggcttcagcactgccaacagatggagaaacacgcttt
agagacataatacccagtgacgccacactaagtgtgcagacacttgcatcagaaaagacg
gagagcaccacacatctt
>Tilapia_SIRT1
ATGCAAATGAGCCAGCTGTCCGAGAGGAAGGCTCCTGCTCAGCGGCTCTGCGGGCTGAAG
ATGGCGGACGGAGAGAACAGTCTGAAAACGGCCTTTTCGGGCGCCTCCGATACGGGGGAA
CCTCACGCTAAGATTTCGAAAATCAGTCCAGGGACTAACTATGGACTGAAAGCCGTCGAA
GCGGACCAGTTCTCGTGCGTCTCCGCGGCAACTGAGAGCTGGGAGGCGGCGTTGAATTGT
GCGCAGCCCGCGGAGAAGGAAGCGAAGCCGGTGATGGCGGTAGAACAGGCCCCGGCAGCG
CTAGGCGGAGACAACAATGGACTGGAAGTATTGCTCTCCGAGACTCACAAGCCAGTGGGG
AAACTTGACGACACCGCTGTCTTTGGCGGAACCGAGGACAGTCCAGATTTTCTTGGGCAC
GATGATCTTCCCTCCAACGGTTTGGCTGTCACTCCAGAGCACATCACTGAAGACGATGAC
AGGTCCTCACATGCAAGCTCCAGCGACTGGACTCCTCAACCGCAAATAGGTTCCTACAGT
TTCATCCAGCAGCACATTAGAGAGACAGATCCAAGGGCCATCCTGAGGGATTTGCTGCCT
GAGACTGTACTCCCACCAGATTTAGATGACATGACATTGTGGCAGATCATCATCAACATC
TCAGAGCCTCCAAAAAGAAAGAAGCGGAAAGATATTAATACCTTAGAAGATGTGGTCAGG
CTACTACATGAAAGTAAAAGGATCCTAGTGCTGACTGGTGCTGGGGTTTCAGTTTCATGT
GGAATACCAGACTTCCGCTCCAGAGATGGAATTTATGCACGGCTTGCTGTAGATTTTCCA
GATCTTCCAGATCCTCAAGCTATGTTTGACATTGAGTACTTCAGACGGGACCCTAGACCG
TTTTTTAAGTTTGCTAAGGAGATTTACCCTGGGCAGTTTGAACCTTCTCCCTGTCATAGA
TTTATATCTATGCTAGATAAGCAAAGAAAGCTGCTGCGCAATTATACACAAAACATCGAT
ACACTGGAACAAGTGGCTGGGGTTCAGCGGATTATCCAGTGTCACGGGTCATTTGCAACT
GCATCCTGTCTCGTCTGTAAGCACAAAGTGGACTGTGAGGCTATAAGGGAAGACATCTTT
AACCAGGTTGTCCCTCATTGTCCACGGTGTCCAGATATCCCCCTGGCAATCATGAAACCA
GACATCGTCTTTTTTGGAGAGAACCTTCCAGAAATGTTTCACAGAGCCATGAAACAGGAT
AAAGATGAGGTGGATCTCTTGATCGTCATTGGCTCTTCACTTAAAGTCCGACCGGTTGCC
CTCATCCCAAACTCCATTCCTCATGAAGTGCCTCAGGTCCTGATCAATCGGGAGCAGCTG
CCTCATCTCAACTTTGACGTGGAGCTACTCGGGGACTGTGACGTCATTATCAATGAGCTC
TGTCATCGATTGGGTGGAGACTTTGAGCAGCTCTGCTACAACACTGTAAGATTAACTGAG
ATCACAGAGAAACCCCCTCGGTTACCAGAACAGCCACCAAGTGAGGCCTTGTCTGCTTCG
AGTGATGCGACTCAGGAGGAGCAGAAGCAGCACACTACAGACTCAGTAAATATGCCTTCG
GAGGAGACAGAAAGTCTGAATGTCACAGAGACTGCTGATAATAATGTTACACCTCTAGAG
CCTTGTCCAAATGCTCAGTGTCCAACGGAGAGCACTGAATCTCCAGAGAAAGGTGCGCCA
AAAGAGGAGGCAGCCGAACGAAAGAGTCAAACCTCCAACCTTGAATTTCGTAGACGTTGC
TGGATGAGTCGAGTTAACAGAACTCCAATCAGCAAACGCCTTGAGGCAGGCCAATACCTG
TTTCAACCACCAAATCACTATATCTTCCACGGGGCAGAGGTTTATTCTGACTCTGAAGAT

GAGACGTCGAGCTCCTGCGGGAGTGACAGCGAGGACTCTGAATGCTGTGCTGAAGGGGAG
GAAGACGACAGCGAGCCGGAGGATGCCGACATAGTACCAGCAGTGGATGGAGAAACATGC
CTCAAAGACATAGTACAGCACACTGTAGCCACTGAGGCCACGTCAAGTGTGCAGACAGAC
AATAATTCTGAAAAGACTGAGAGTACCACCCACCTT
>Turkey_SIRT1
ATGTTAGGTACGGACCCACGGACAATTTTGAAAGACCTGCTCCCAGAAACAATCCCTCCA
CCTGAACTGGATGATATGACGCTGTGGCAAATCGTCATAAACATTCTTTCCGAGCCGCCA
AAAAGAAAAAAACGAAAAGATATTAATACTATTGATGATGCAGTGAAACTTTTACAGGAA
TGCAAAAAAATCATGGTCTTGACAGGAGCTGGGGTGTCTGTGTCTTGTGGAATACCTGAC
TTTAGATCAAGAGATGGCATCTATGCACGCCTTGCTGTAGACTTCCCAGACCTTCCAGAT
CCTCAAGCAATGTTTGATATAGAATACTTCAGAAAGGATCCCAGGCCATTTTTTAAGTTT
GCAAAGGAAATCTATCCAGGCCAGTTCCAGCCATCTCTCTGCCACAAGTTCATAGCTTTG
ATGGATAAAGAAGGAAAATTGCTTCGTAACTATACTCAGAACATAGACACATTGGAACAG
GTTGCAGGAATCCAAAAGATAATTCAGTGTCATGGTTCCTTTGCAACAGCTTCCTGCCTG
ATCTGTAAATACAAAGTTGATTGTGAAGTTGTTCGAGGAGATATTTTCAATCAGGTTGTT
CCTAGATGTCCTCGCTGTCCGCCTGATGAACCACTTGCTATCATGAAGCCTGACATAGTG
TTCTTCGGAGAGAACTTACCTGAGCAGTTCCATCGTGCCATGAAGTATGACAAAAATGAA
GTTGATCTCCTTATTGTCATTGGGTCTTCACTGAAAGTAAGACCAGTAGCATTGATTCCA
AGTTCCATCCCCCATGAAGTGCCTCAGATCTTAATTAATAGGGAACCTCTGCCTCATCTA
CACTTTGATGTGGAGCTTCTTGGAGACTGTGACGTTATTATTAACGAATTATGTCAAAGG
CTAGGTAGTGAATATACAAAACTTTGCTACAACTCAGTAAAACTTTCTGAAATAACAGAA
AAGCCTCCACGAATGCACAAAGAGCTTGAAATGCACTCATCTGAGCTACCACCTACACCC
TTGAACATTTCTGAAGACTCTGGTTCACCACAACAAATGACTCCACCAGATACTTCGGTG
GTGCCCTCAGAACATGCAGCTGAATGTAAGATAGAAAACTGTGATCCTGCTTCTGAAACT
AAAGGGACCTGCACAGAGGAAAAGCTTCAAGATCCACAGGCATCCTCTGAAAACCCTGAA
AATACTACTAGTGAATTAATGAACTCTGAAACAATGAAGGAAAATGGAACCAACAATGGA
GAAAGCAAAGAAAAAAATGAAATAGTGAAGAAGTGCTGGGTAAATAGATCTGCAAAAGAA
CAGATTAGCAAAAGGCTGGATGGTACTCAGTATCTATTTTTACCACCAAATCGCTATATT
TTCCACGGAGCTGAGGTATACTCAGATTCTGAAGACGATATGATATCTTCTAGCTCTTGT
GGGAGTAGTAGCGAAAGCGGCTCGTGTCGCAGTCAGAGCTTAGATGTGGAGGACGAGAGC
GAGATTGAAGAGTTTTACAATGGCATAGAGGATGAGGATGCTCCGGAAAGGGAAGAGGAA
GCTGCATTTGGGGAAGATGGGGTTGAACAAGATGCAGCTGATGAATCAGCTTACACAAAT
GAAGCTGCAGGGAACGATCATCCAACAAGCAACAAGTTG
>Zebra_finch_SIRT1
ATGTTAGGTACAGACCCACGGACGATTCTGAAGGACCTGCTGCCAGAAACGATCCCCCCA
CCTGAACTGGATGACATGACTCTGTGGCAAATCGTGATAAACATCCTTTCAGAGCCACCA
AAAAGGAAGAAGAGGAAAGATATCAACACCATTGATGATGCTGTGAAACTTTTGCAGGAG
TGCAAGAAGATCATGGTCTTGACTGGAGCTGGGGTGTCAGTGTCTTGTGGAATACCTGAC
TTTAGATCCAGAGATGGCATCTATGCACGCCTTGCTGTGGACTTCCCAGACCTTCCAGAT
CCTCAAGCAATGTTTGATATAGAATATTTCAGAAAGGATCCCAGGCCATTTTTTAAGTTT
GCAAAGGAAATCTACCCGGGACAGTTCCAGCCATCTCTCTGTCACAGGTTCATCGCTCTG
ATGGATAAAGAAGGAAAACTCCTCCGCAACTATACTCAGAACATAGACACACTGGAACAG
GTTGCAGGAATCCAAAGGATAATACAGTGTCATGGTTCCTTTGCAACAGCTTCCTGCCTG
ATCTGTAAATACAAAGTTGATTGTGAAGTTGTTCGAGGAGATATTTTCAATCAGGTGGTG

CCCCGCTGTCCCTGCTGCCCCCCCGAGGAGCCGCTGGCCATCATGAAGCCAGACATCGTG
TTCTTTGGGGAGAATCTGCCTGAGCAGTTCCACCGTGCCATGAAGTATGACAAAAATGAA
GTGGATCTCCTCATTGTCATTGGGTCTTCACTCAAAGTAAGACCAGTAGCATTGATCCCA
AGTTCCATCCCCCATGAAGTGCCTCAGATCTTAATTAATAGGGAACCTTTGCCTCATCTA
CACTTCGACGTGGAGCTTCTTGGAGACTGTGATGTCATTATTAATGAATTGTGTCAGAGG
TTGGGTAGTGAATACACAAAACTTTGCTACAACTCGGTAAAACTTTCGGAAATCACAGAA
AAGCCTCCACGGCCGCACAAGGAGCTCGAAGCGCTCTCAGCTGAGCTCCCACCAACCCCT
CTGAACATTTCAGAAGGCTCCAGTTCACCAGAAAGGATGAGCCCAGCCAATTCTGCGCGG
GCGTCAGAACATCCACCGGAATGTAAGGTAGAAAACTGTCAGCCTGCCCCAGAAACTACA
GGGACCTGCTCAGAGGAGACGCTTCAGGACACACAGGTGTCATCAGAAAACCCTGAAAAT
CCTGCTAGTGAGCTAATGAACTCTGAAACAATGAAGGAAAATGGGTCTAATGATGGAGAA
AATAAAGAAAAGAGTGAAATATTGAAGAAGTGTTGGGTAAACAGATCTGCAAAAGAACAG
ATTAGCAAAAGGCTGGATGGTACTCAATATCTGTTTTTGCCACCCAATCGCTATATCTTC
CACGGTGCTGAGGTCTACTCAGATTCTGAAGACGATATCATATCTTCCAGCTCTTGTGGC
AGCAGCAGTGAGAGCGGCTCCTGCCGCAGTCAGAGCTTAGATGTGGAGGATGAGAGTGAG
ATGGAGGAGTTTTACAATGGCATAGAGGATGAGGATGCTCCCGAGCGGGAAGAGGAGCCT
GGATTCGGGGAGGATGGAGCCGAACAGGAGGAGTTGGCAGCTGAGGAATCAGCTGAGACA
AATGAAGCTGCAGGGACTGAACATCCGAGCAACGCGCTG
>zebrafish_SIRT1
ATGGCGGACGGCGAAAATAAACGGGCCGAATCCGCCGAGCCGGACGAGCCGCTACCGAAG
AAACCGAGGCTTCTGGAGCTGTCCGGTGATTCGGAGCACAGCGCGACCGCCGGTGCCGAT
ACATTGGACGAGAAACCGGCGCGGATGGACGAGTCACAGCAGGCCTTATCGATCAACAAC
AACAACAACACTAGACCGACTGAACCCGGGCAGCCAGCCGACCCGGAGCCCGAGATCTCC
GAGTTAACTGATGAAGGTGTTCATCCCAATGGTTTTACATCCCCTGATCTTCTTCGGGAC
GATGATGACTGCTCATCCCGTGCCAGCTCTAGCGACTGGACGCCTCAGCCACAGATTGGG
TCCTATCGGTTCATTCAGCAGCACATCATGAGAGGAACTGATCCCAGAGCCATTCTGAAA
GATTTGCTTCCTGAAACGGTGCTTCCTCCAGACCTGGACGACATGACGCTGTGGCAGATC
ATCATCAACATCTCTGAGCCGCCCAAACGAAAGAAACGCAAAGACATCAACACGTTAGAG
GACGTGGTGCGGCTCCTGAACGAGAGGAAGAAGATTCTGGTGCTCACCGGTGCTGGGGTG
TCTGTTTCCTGTGGGATTCCTGACTTTCGCTCTAGAGATGGTATTTATGCTCGACTCGCT
GTAGATTTCCCAGACCTTCCTGATCCTCAAGCCATGTTTGACATCGACTACTTCAGGAGA
GATCCCAGGCCTTTTTTCAAGTTTGCCAAGGAAATCTACCCCGGACAGTTCCAGCCATCT
CCATGTCACCGATTTATATCAATGCTGGACAAGAAGGGAAGGTTATTGAGAAACTACACG
CAGAATATCGACACACTGGAGCAGGTGGCTGGAATACAGAAGATCATTCAGTGCCACGGG
TCTTTTGCGACTGCCTCCTGTCTTATCTGTAAGCATAAGGTGGACTGTGAAGCCATAAGA
GAAGATATATTCAACCAGGTTGTTCCTCATTGTCCCAGGTGTCCGTCTGATGTCCCGTAC
GCCATCATGAAGCCAGACATCGTCTTTTTTGGAGAAAACCTTCCAGAGTTTTTCCACAGA
GCCATGAAGCAGGATAAAGACGAGGTGGACCTCCTCATCGTGATCGGCTCCTCGCTGAAA
GTGCGGCCAGTGGCTCTTATACCCAGCTCGATTCCTCATGACGTGCCTCAAGTGCTGATC
AACCGTGAGCCGCTGCCGCATCTGAACTTTGACGTGGAGCTCCTGGGCGATTGTGACGTG
ATCGTGAACGAACTCTGCCATCGCCTGAACGGAGACTTCCAGCAGCTCTGCTACAATTCA
TCGCGTCTCAGCGAGATCACAGAGAAACCGGCAGCTCCTGAACACACGGAGAACACGTCT
GCAGATCACTCTCATGCTGATGCTGAACATATCGAGAACACGTCTGCAGATCACTCTCAT
GCTGATGCTGAACATATAGAGAACACGTCTGCAGATCGCGATGATGCTAAACATACAGAA

AATACACCTACAGATCACGCTGATGCTGAACATACGAAGAACACGTCTGCAGATCACGCT
AATGCTGAACATACAGAGAACACATCTGCAGGTCACGTTAATGCTGAACATATAGAGCAC
ATGTCTAAAGATCATGCTAATCCTAAAGACGATCAGAGCTCACTATCAGTTAATGAAGAG
GAATTAGCGTCTCCAGCAGCAGAAACACACGCGCTGGACTCTACAGAGATTTCAGCACAC
ACAGAGAGGAGTAAAGAGGCCGATGCTGTGAACACTGATGATGCAGCGTGTGTAAAAGAT
GAAGAAAACACAGATCGACTGCGCGTGGAAATGCGCAGACGCTGCTGGAGGAGCCGAATC
TGTCAGAGTCCAATCAGCAAACGACTCGGAGCGTCGCAGTATTTATTTCAAGCACCGAAC
CGTTATGTTTTCCACGGGGCGGAGGTTTACTCGAGCTCAGAGGACGAGAGCTCCAGTTCG
TGCGGCAGTGAAAGCGACGGATCTTTCCAGCATGAAGACAGCGAGGTGGAGGAAAACGGT
GCAGCAATGACGGATAAGGAAACGGACACAGAAACAGTACAGGACAGTGAACACAGACGC
CTTCAGACACACTGCACACAACACACACAA
>Great_tit_SIRT1
ATGAGATTCCACCAAGATAACTTCCTCCTCAGTGATGAAATTATAGCCAATGGCTTTCAC
TCCTGTGACAGTGATGAAGAAGACAGAGCCTCACATGCAAGTTCCAGTGACTGGACCCCA
AGACCACGTATAGGTCCCTACACTTTTGTGCAGCAGCACCTGATGTTAGGTACAGACCCA
CGGTCGATTCTGAAGGACCTGCTGCCAGAAACGATCCCCCCACCTGAACTGGACGACATG
ACTCTGTGGCAAATCGTCATCAACATCCTTTCAGAGCCACCCAAAAGGAAGAAGAGGAAA
GACATTAACACCATTGATGATGCTGTGAAACTTCTGCAGGAATGCAAGAAGATCATGGTG
TTGACTGGAGCTGGGGTGTCAGTGTCCTGTGGAATACCTGACTTTAGATCCAGAGATGGC
ATCTACGCACGCCTTGCTGTAGACTTCCCAGACCTTCCAGATCCTCAAGCAATGTTTGAT
ATAGAATACTTCAGAAAGGATCCCAGGCCTTTTTTTAAGTTTGCAAAGGAAATCTACCCA
GGACAATTCCAGCCATCTCTCTGTCACAAGTTCATCGCTTTGATGGATAAAGAGGGAAAA
CTCCTTCGCAACTATACTCAGAACATAGACACACTGGAACAGGTTGCAGGAATCCAAAGG
ATAATACAGTGTCATGGTTCCTTTGCAACAGCTTCCTGCCTGATCTGTAAATACAAAGTT
GATTGTGAAGTTGTTCGAGGAGATATTTTCAATCAGGTGGTGCCCCGCTGTCCCCGCTGT
CCCCCCGAGGAGCCGCTGGCCATCATGAAGCCGGACATTGTGTTCTTTGGGGAGAACCTG
CCCGAGCAGTTCCACCGTGCCATGAAGTACGACAAAAATGAAGTGGATCTCCTCATTGTC
ATTGGGTCTTCACTCAAAGTAAGACCAGTAGCATTGATCCCAAGTTCCATCCCCCATGAA
GTGCCTCAGATCTTAATTAATAGGGAACCTTTGCCTCATCTACACTTTGACGTGGAGCTT
CTCGGAGACTGTGATGTAATTATTAATGAATTATGCCAGAGGTTAGGTAGCGAATACACA
AAACTTTGCTACAACTCCGTAAAACTTTCGGAAATCACGGAAAAGCCTCCACGGCCGCAC
AAGGAGCTGGAAGCGCTCTCAGCTGAGCTCCCACCAACCCCTCTGAACATTTCAGAAGAC
TCCAGTTCACCAGAAAGGATGAGCCCACCTGAGAGCGCGGCCGTGTCCCAGCACCCACCC
GAATGTAAGGTAGAAAACTGTGAGCCTGCCTCAGAAACTAAAGGGACCTGCTCGGAGGAG
ACGCTTCAGGACACGCAGGTGGCGTCAGAAAACCCTGAAAATCCTGCTAGTGAGCTAATG
AACTCTGAAACAATGAAGGAAAATGGATCTAACGATGGAGAAAATAAAGAAAAGAGTGAA
ATATTGAGGAAGTGTTGGGTAAACAGATCTGCAAAAGAACAGATTAGCAAAAGGCTGGAT
GGTACTCAGTATCTGTTTTTGCCACCCAATCGCTATATCTTCCACGGTGCTGAGGTTTAC
TCGGATTCTGAAGACGATATCATATCTTCCAGCTCTTGTGGCAGCAGCAGTGAGAGCGGC
TCGTGCCGCAGTCAGAGCTTAGATGTGGAGGACGAGAGTGAGATGGAGGAGTTTTACAAT
GGCATAGAGGATGAGGATGCTCCCGAGAGGGAAGAGGAGCCCGGATTTGGGGAGGATGGA
GCAGAACAGGAGGAATTGGCAGCTGAGGAATCAGCTGAGACAAACGAAGCCGCAGGGACG
GAACATGCGAGCGACGCGCTG
>African_ostrich_SIRT1

ATGTCCCTGTTGTGCTGTCTTGCAGATAACTTCCTTTTTAGCGATGAAGTCATAGCCAAT
GGTTTCCATTCCTGTGACAGTGATGATGATGACAGAGCCTCACATGCAAGTTCTAGTGAT
TGGACTCCAAGACCACGTATAGGTCCCTACACTTTTGTTCAGCAACATCTCATGTTAGGT
ACAGACCCACGGACGATTCTGAAAGACCTACTACCAGAAACAATCCCTCCACCTGAACTG
GATGACATGACACTGTGGCAAATTGTTATAAACATTCTTTCAGAACCACCGAAAAGAAAA
AAACGAAAAGATATTAATACTATTGATGATGCTGTGAAACTTTTACAAGAATGCAAAAAA
ATAATGGTCTTGACTGGAGCAGGGGTTTCTGTGTCTTGTGGAATACCTGACTTCAGATCA
AGAGATGGCATCTATGCGCGCCTTGCTGTAGACTTTCCAGACCTTCCAGATCCTCAAGCA
ATGTTTGATATAGAATACTTCAGAAAGGATCCCAGGCCGTTTTTTAAGTTTGCAAAGGAA
ATATATCCAGGACAGTTCCAACCATCCCTCTGTCACAAGTTCATAGCTTTGATGGACAAA
GAAGGAAAATTGCTTCGCAACTATACACAAAACATAGACACCCTGGAACAAGTTGCAGGA
ATCCAAAGGATAATTCAGTGTCACGGTTCCTTTGCAACAGCTTCCTGCCTGATCTGTAAA
TACAAAGTTGACTGTGAAGTTGTTCGAGGAGATATTTTCAATCAGGTCGTTCCTAGATGT
CCCAGATGTCCACCTGATGAACCGCTTGCTATCATGAAGCCAGACATAGTGTTCTTTGGA
GAGAACTTACCTGAGCAGTTCCATCGTGCCATGAAGTATGACAAAAACGAAGTTGATCTC
CTTATTGTTATTGGGTCTTCGCTGAAAGTAAGACCAGTAGCGTTGATTCCAAGTTCCATC
CCTCATGAAGTGCCTCAAATCTTAATTAATAGGGAACCTTTGCCACATCTACACTTCGAC
GTGGAGCTTCTTGGAGACTGTGATGTTATTATTAATGAATTATGTCAAAGGCTAGGTAGT
GAATATACAAAACTTTGCTACAACTCAGTAAAACTTTCTGAAATAACAGAAAAGCCTCCA
CGAACACAGAAGGAGCTTGACATGCACTCATCTGAGTTACCACCTACCCCTTTAAACATT
TCAGAAGACTCTAGTTCACCAGAAAGAACGACTCCACCAGATACTTCGGTGGTGCCCTCA
GAACACCCAGCTGAATGCAAGGTAGAAAACTGTGATCCTGCCTCAGAAACTAAAGCGATC
TGCACAGAGGAGAAGCTTCAGGACACGCAGACATCCTCAGAGAGCCCTGGAAATACTACT
AGTGAATTAATGAACTCTGAACCAATGAAGGAAAATGGATCCAACAATGGAGAAAATAGA
GAAAAAAATGAAATATTGAGGAAGTGCTGGGTAAACAGATCTGCAAAAGAACAGATCAGC
AAAAGGCTGGATGGTACTCAGTATCTGTTTTTACCGCCAAATCGCTATATTTTCCATGGT
GCTGAGGTGTACTCGGATTCTGAAGATGATATCATATCTTCTAGCTCTTGTGGCAGTAGT
AGTGAAAGTGGCTCATGTCATAGCCAGAGCTTAGATGTGGAAGATGAGAGCGAGATTGAG
GAATTCTACAATGGCATAGAGGATGAGGATGCTCCAGAAAGGGAAGAGGAACATGGATTT
GGGGAAGATGGAGTTGAACCAGATGCAGCAGATGAATCAGCTTATATAAACGAAGCCACA
GGGACTGATCATCCAACAAGCAACAAGTTG
>Adelie_penguin_SIRT1
TATATCTGTGCCATTTGGCTCAAGTGTAACCCGTCATTGTTGTGCTGTCTTGCAGATAAC
TTCCTTTTTAGTGATGAAATCATAGCCAATGGTTTTCACTCCTGTGATAGCGATGAAGAT
GACAGAGCCTCACATGCAAGTTCTAGTGACTGGACTCCAAGACCACGTATAGGTCCCTAC
ACTTTCGTTCAGCAGCATCTCATGTTAGGTACAGACCCACGGACAATTCTGAAAGACCTG
CTACCAGAAACAATTCCTCCACCTGAACTGGATGATATGACACTGTGGCAAATTGTCATA
AATATTCTTTCAGAACCACCAAAAAGAAAAAAACGAAAAGATATTAATACTATTGATGAT
GCTGTGAAACTTTTACAAGAGTGCAAAAAAATAATGGTCTTGACTGGAGCTGGGGTGTCC
GTGTCTTGTGGAATACCTGACTTTAGATCAAGAGATGGCATCTATGCACGCCTTGCCGTA
GACTTCCCAGACCTTCCAGATCCTCAAGCAATGTTTGATATAGAATACTTCAGAAAGGAT
CCCAGGCCATTTTTTAAGTTTGCAAAGGAAATCTATCCAGGACAATTCCAGCCATCTCTC
TGTCACAAGTTCATAGCTCTGATGGATAAAGAAGGAAAACTACTTCGCAACTATACTCAG
AACATAGACACATTGGAACAGGTTGCAGGAATCCAAAGGATAATACAGTGTCATGGTTCC

TTTGCAACAGCTTCCTGCCTGATCTGTAAATACAAAGTTGATTGTGAAGTTGTTCGAGGA
GATATTTTCAATCAGGTTGTACCTAGATGTCCCAGATGTCCACCTGATGAACTGCTCGCT
ATCATGAAGCCAGACATAGTGTTCTTTGGAGAGAACTTACCTGAGCAGTTCCATCGCGCC
ATGAAGTATGACAAAAATGAAGTTGATCTCCTTATTGTTATTGGGTCTTCACTGAAAGTA
AGACCAGTAGCATTGATTCCAAGTTCCATCCCCCATGAAGTGCCTCAGATCTTAATTAAT
AGGGAACCTTTGCCTCATCTACACTTTGACGTGGAGCTTCTTGGAGACTGTGATGTTATT
ATTAATGAATTATGTCAAAGGCTAGGTAGCGAATATACAAAACTTTGCTACAACTCAGTA
AAACTTTCAGAAATAACAGAAAAGCCTCCACGAACGCACAAGGAGCTTGAAATACACTCA
GCCGAGCTACCGCCTACCCCTTTAAACATTTCAGAAGACTCTAGTTCACCAGAAAGGATG
ACTCCACCAGATACTTCAGTGGTGTCTTCAGAACACCCAGCTGAATGTAAGTTAGAAAAC
TGTGATCCTGCCTCAGAAACTAAAGGGACCTGCACAGAGGAAAAGCTTCAGGACACACAG
ACATCGTCAGAAAACCCTGAAAATCCTACTAATGAATTAATGAACTCTGAAACAATGAAG
GAAAATGGATCTAACAACGGAGAAAATAAAGAAAAAAATGAAATCCTGAAGAAGTGCTGG
GTAAACAGATCTGCAAAAGAACAGATTAGCAAAAGGCTGGATGGTACTCAGTATCTATTT
TTGCCACCAAATCGCTATATTTTCCATGGTGCTGAGGTTTACTCAGATTCTGAAGACGAT
ATCGTATCTTCCAGCTCTTGCGGGAGTAGTAGTGAAAGTGGTTCATGTCGTAGTCAGAGC
TTAGATGTGGAGGATGAGAGTGAGATCGAAGAGTTTTACAATGGCATAGAGGATGAGGAT
GCTCCTGAAAGGGAAGAGGAACCTGGATTCGGGGAAGATGGAGTTGAACAAGATGAATCG
GCAGCTGATGAATCAGCTTATGCAAATGAAGCTGCAGGGACTGATCATCCAAGCAACAAG
TTG
>Gharial_SIRT1
ATGTTAGGCACAGACCCAAGGACAATTTTGAAAGATCTGTTACCAGAAACTATTCCTCCA
CCTGAACTAGATGATATGACACTGTGGCAAATTGTTATAAACATTCTTTCAGAACCACCA
AAAAGGAAAAAACGAAAAGATATTAATACCATTGAGGATGCTGTGAAACTTTTGCAAGAA
TGCAAAAAGATTATGGTCTTGACTGGAGCTGGGGTGTCTGTGTCTTGTGGAATACCTGAT
TTCCGATCAAGAGATGGCATCTATGCACGTCTTGCAGTAGATTTCCCAGACCTTCCAGAT
CCTCAAGCAATGTTTGATATAGAATACTTCAGAAAGGATCCAAGACCATTTTTTAAGTTT
GCAAAGGAAATATATCCTGGGCAATTCCAGCCATCCCTCTGTCATAAATTCATAGCTTTG
ATGGATAAAGGAGGAAAGCTACTTCGCAACTACACTCAAAACATAGATACACTGGAACAG
GTTGCAGGAATCCAAAGGATAATACAGTGTCATGGTTCTTTTGCTACAGCTTCCTGTCTA
ATCTGTAAATACAAAGTTGACTGTGAAGCTGTTCGAGGAGACATTTTTAATCAGGTTGTT
CCTAGATGTCCCAGATGTCCACCTGATGAACCACTTGCTATTATGAAGCCAGAAATAGTA
TTCTTTGGAGAGAACTTACCAGAGCAGTTCCACAGGGCCATGAAGTATGACAAAGATGAA
GTAGATCTCCTTATTGTAATTGGGTCTTCACTAAAAGTAAGACCAGTAGCATTGATTCCA
AGTTCCATCCCCCATGAAGTGCCTCAGATATTAATTAATAGGGAACCTTTGCCCCATCTA
CACTTTGATGTGGAACTCCTTGGAGACTGTGATGTAATCATTAATGAACTATGTCAAAGG
CTGAGTGGTGAATATACAGAACTTTGCTACAACTCCATAAAACTTTCAGAAATAACAGAA
AAACCACCACGAATGCACAAGGAGCTTGAAATGCATTCAGCTGAGTTACCACCTACCCCT
TTAAACATTTCTGAAGATTCTAGTTCACCTGAAAGAGCAACTCCCCCAAACTCTTTGGTT
GTGCTTTCAGAGCATCCAACAAAATGTAGGGCAGAAAATTCTGAACTTGCCTCAGACTCT
AATGGGAACTGCATGGAGAAATTCCAGGAGGTACAGACATCCTCAGAAAATCCTGAAAGT
ATTACTGGCCAGTTAACGAACTTAGAACATCCAAAAGAAAATGGAGCTAACAATGGAGAA
CAGAAAGAAAAAAATGATATAACTCCGTCGGTTGAAACTTTGAGGAAATGCTGGCCAAAC
AGATGTGCAAAAGAACAGATTAGCAAGCGGCTTGATGGTACTCAATACCTATTTTTACCA

CCAAATCGCTATATTTTCCATGGTGCTGAGGTGTACTCAGATTCTGAAGATGATGTCCTA
TCTTCCAGTTCTTGTGGAAGTAGTAGTGATAGTGGTTCTTGTCATAGTCCAAGCTTAGAT
GTGGAAGATGAAAGTGAGATTGAAGAATTCTACAATGGCATGGAGGAAGAGGATGCTCCA
GAAAGAGAAGAGGAAATTGCATTTGGGGAAGATGGTGTTGAAGAAGATGCAGTTGATGAA
TCAGGTTATATAAATGAAGCTGTAGGGACTGACCATCCATCAAACAAACTG
>Great_tit_SIRT2
ATGGTGGGCGCCGGCATCTCCACCTCTGCCGGGATCCCGGATTTCCGCTCCCCCGGCACC
GGGCTCTACTCCAACCTGCAGAGTTACAACCTCCCCTACCCCGAGGCCATCTTTGAGATC
GGTTTCTTCAAGAAACACCCAGAGCCCTTTTTCGCCCTTGCCCGTGAGCTCTACCCAGGG
CAGTTCAAGCCCACCGTGTGTCACTACTTCATGCGGCTGCTGCAGGACAAGGGGCTCTTG
CTGCGCTGCTACACCCAGAACATCGACACTCTGGAGAGGGTGGCAGGGCTGGAGCCGGAG
CTGCTGGTGGAGGCTCACGGCACGTTCTTCACGTCGCACTGCCTGCGCTCCTCGTGCCGC
CAGCGCTACGACCTGGCCTGGATGAGGGAGAGGATTTTCTCCTCCCTGGTCCCGAAATGT
GAGAAGTGCCAGGGGCTGGTGAAGCCTGACATTGTGTTTTTTGGGGAGAACCTCCCCTCG
CGCTTCTTTACCCTCCTGGAGTCGGACTTTGAGAAGGTTGATCTCCTGCTCATCATGGGC
ACCTCACTGCAGGTGCAGCCCTTTGCCTCCCTCATCAGCAGGGTCCCCACCAACACCCCC
AGACTCCTGATCAACAAGGAGAAGACAGGACAG
>common_starling_SIRT2
ATGGAGCTGCTGCGGACGCTCCTGTCCCGGACCCTGGGGCTCGGAGGGGACAAACCGGAG
CCGGTGCTGGACGAGCTGAGCCTGCAGGGCGTGAGCCGCTTCCTGAAGAGTGACCGCTGT
AAGAACGTCGTGTGCATGGTGGGCGCTGGCATCTCCACCTCCGCTGGGATCCCGGACTTC
CGCTCCCCCGGCACCGGGCTCTACTCCAACCTGCAGAGCTACGACCTCCCCTACCCTGAG
GCCATCTTCGAAATCGGTTTCTTCAAGAAACACCCGGAGCCCTTTTTCGCCCTTGCCCGT
GAGCTCTATCCAGGGCAGTTCAAGCCCACAGTTTGTCACTACTTCATGCGGCTGCTGCAG
GACAAGGGGCTTTTGCTGCGCTGCTACACCCAGAACATCGACACTCTGGAGAGGGTGGCA
GGGCTGGACCCGGAACTGCTGGTGGAGGCTCACGGCACATTCTTCACGTCCCACTGCCTG
CGGCCCTCGTGCCGCCAGCGCTACGGCCTGGCCTGGATGAGGGAGCGGATTTTCTCCTCC
CTTGTCCCTAAATGTGAGAAGTGCCAGGGGCTGGTGAAACCTGACATTGTGTTTTTTGGG
GAGAGCCTCCCCTCTCGCTTCTTCGCCCTCCTGGAGTCCGACTTTGAGAAAGTTGACCTG
CTGCTCATCATGGGCACCTCGCTGCAGGTGCAGCCCTTTGCCTCCCTTATCAGCAGGGTC
CCCACCAACACCCCCAGACTCCTGATCAACAAGGAGAAGACGGGGCAGAGTGACCCTCTG
ATGTCACTGATGGGCTTTGGTGGGATGGACTTTGACTCAGACAAGGCCTACAGGGATGTG
GCCTGGCTGGGGGACTGTAACAGCGGGTGCCTGGCTCTGGCTGAACTGCTGGGCTGGAAG
GTACCCCCC
>central_bearded_dragon_SIRT2
ATGCTTCATGGGGGTTGTAGTTTTCCTGTCAGGTCGAGGCGGAGGGAAGCTGGGGAAGAT
TGCTCCTCCTTCTCCTTCGGGGCTCGTTTGAGAACCGGAGTTCGTGGTGCGGAGAAGGAG
GGGGGGGACCCTCGATGGAAAAACGGGAAGGATCCAGATTCTGATGAAGAAGCTGGGGCT
TCTGGTCTGACAGAAATGGAATTTTTGAAGAACCTCTTGTCCCGGACGCTGAACTTGGGC
AGTGAAAAACCTGAAAAGGTGCTGGATGAGCTGACGTTGGAGGGTGTGAGCAGGTTCATG
CTTAGTGAAAAAATGCAAGAGGGTAATATGTATGGTCGGAGCAGGAATCTCAACGGCTGCT
GGGATCCCAGATTTCCGTTCACCGGGTACAGGACTGTACGCCAATCTGCAGCAATACAAC
TTACCATACCCTGAAGCTATCTTTGAGATTGGCTACTTCAAGCAACACCCAGAGCCCTTC
TTTGCCTTGGCCCGGGAGCTGTACCCAGGACAGTTCAAGCCCACAATCTGTCACTACTTC

ATGCGCCTCCTCAAGGAGAAAGGGCTGCTGCTGCGCTGCTACACCCAGAACATCGACACC
TTGGAACGGGTGGCAGGCCTGGGTAACGAAGATTTGGTGGAAGCTCATGGCACCTTTTTT
ACCTCCCACTGCACAGGTTCCTTCTGCAGGGAAATGTACAGCTTGGACTGGATGAAAGAA
AAGATCTTCTCCTCTGCCATTCCCAAGTGTGAAAAATGCCAGAATGTAGTGAAGCCAGAT
ATCGTGTTTTTTGGGGAGAATTTGCCCTCTCGTTTCTTCTCTCTGATGAAGTCAGATTTC
CGTGATGTAGACTTGCTTATCATCATGGGCACCTCCCTTCAGGTCCAGCCCTTTGCCTCA
CTGGTCAGCAGGGTGCCTACAAACACACCACGGCTCCTCATCAATAAGGAAAAGACTGGA
GAGAGTGATCCTTTCATGTCCTTGATGGGCTTTGGCTGCGGGATGGACTTCGACTCAGAA
AAGGCATACCGGGATGTGGCACAACTTGGAGACTGTGACAAGGGCTGCCTAGCTTTAGCA
GAGCTGCTGGGATGGAAGAAAGAGCTGGAAGAACTGGTGAAAAGGGAGCACGCGGCCATA
GATGCTAAGTCAGGGCAAGCTATTGGAGATGGGGCCAGTGCGACCCATCCTTCAGGAAAG
GAGCAGCAGAAGCCCTCCCCAAAGAAGGAAGCCCTTCCTCCCAGCAAAGAAGAA
>Gecko_SIRT2
ATGGACAACCGGGAGGACCCCGGAGCCGCCGCCAAGGTCGACGCCGGGAAGGGCTCGGGG
TCGTCGAGCTCCGATTCTGACGACGAAGCCGGGGCATCAGGCCAGTCTGAAATGGATTTC
CTACGGAACCTCTTCTCCCGGACGTTGGGTCTGGGCAGCGAGAAGCCCGAGAAGGTGCTG
GATGAGCTGACGTTGGAGGGCGTGAGCAAGTTCATGCTGAGTGAGAAATGCAAGAAGGTA
GTGTGCATGGTGGGAGCAGGGATCTCAACCGGTGCAGGGATCCCAGATTTCCGTTCCCCG
GGCACGGGGCTCTACGCCAATCTGCAGCAGTACAACCTACCATACCCTGAAGCCATCTTT
GAGATCAGCTACTTCAAGCAACACCCAGAGCCGTTCTTTGCCCTGGCCCGGGAGCTGTAC
CCAGGGCAGTTTAAGCCCACCGTCTGCCACTACTTCATGCGCCTCCTGAAGGAGAAAGGG
CTGCTGCTGCGTTGCTACACCCAGGTAGGTGGAATCCAGCCCTGCCCCTGGGTCAAGGTA
CACCTGACAGCGCACTACAAAGCCACAGCCAACAGAATCTCCTCAGGATGAGAAGAAACC
ACCTCCTTCTGTTTCGTTTCAGAAAAGATCTTCTCGTCCCTCATCCCCAAGTGTGAAAAG
TGTCAGAGTGTGGTGAAGCCAGATATTGTCTTCTTCGGGGAGAACCTGCCTTCCCGTTTC
TTCTCTCTGATGCAGTCGGATTTCCGCAACGTGGATTTGCTGATCATCATGGGCACCTCC
CTTCAGGTCCAGCCCTTTGCTTCCCTCGTTGGCAGGGTGCCCACAAACGCTCCCCGGCTC
TTGATCAATAAAGAGAAGACTGGGGAGAGCGACCCTCTCATGTCCCTGATGGGCTTCGGA
TGCGGGATGGACTTTGATTCCGAGAAGGCGTACAGGGACGTCGCCTGGCTCGGAGACTGT
GACGAAGGCTGCCTGGCGTTGGCTGAGCTCCTAGGATGGAAGAAAGAGCTGGAGAAACTG
GTGAGGGAGGAGCACGCGGCCATAGACGCCAAATCAGGCCAGGCTGTCGGAGACGGGGCA
AGCGCCTGCAGCGCTCCGGCGAAGGAAGCGAAGCGGCCACCGTCCCCGAAGAAGAAATGC
GAAGAGAAG
>Taiwan_habu_SIRT2
ATGGACAGCCGGGAAGGCGCTGCCGCCAGTCCCCAAGCGGACGCCGAAAAGGCCTCCAGG
TCCTCGAGTTCAGATTCTGATGAGGAAGGGGGAGCTTCAGGCCAGACAGAAATGGAATTT
TTGCGAAACCTCTTTTCCCGGACTCTGGGCTTTGGCAGTGAGAAGCCCGAGAAGGTGCTG
GAGGAACTGACGCTGGAAGGCGTGACCAACTTCATACTGACTGAGAAATGCAAGAATATA
GTCTGTATGGTTGGTGCAGGGATCTCGACCAGTGCGGGCATCCCCGATTTCCGCTCACCT
GGCACGGGGCTGTATGCCAACCTGCAGCAGTACAATCTACCGTATCCCGAAGCCATCTTT
GAGATTGGCTATTTCAAGCAAAACCCAGAACCATTCTTCACCTTGGCTCGGGAGCTTTAC
CCAGGGCAGTTCAAGCCCACTGTCTGTCACTACTTCATTCGTCTCTTGAAGGATAAAGGG
CTGCTACTGCGCTGCTACACTCAGAACATCGACACCTTGGAGAGGGTGGCCGGTCTGGAT
CCCGAACACTTAGTGGAAGCTCACGGCACCTTTTATACCTCCCACTGCATCAGCTCCACT

TGCAAGAAGCCCTACAGCCTGGAGTGGATGAAAGAAAAAATATTCGGATCTCTCACCCCC
CGATGTGAAAAATGTCAGAACATCGTGAAGCCAGACATCGTGTTTTTTGGGGAGAATCTG
CCCCCTCGTTTCTTCACTCTCATGCAGTCGGATTTCCAGAATGCGGACATGCTTATTATC
ATGGGCACCTCACTTCAGGTCCAGCCTTTTGCCTCTCTGGTCAGCAGGGTACCTGCAAAC
ACACCACGGCTTCTGATCAATAAGGAGAAGACTGGAGAGAGTGATCCTTTCATGTCCCTG
ATGGGCTTAGGCTGCGGGATGGATTTTGATTCGGAGAAGGCATACAGGGACGTGGCCTGG
CTCGGAGACTGCGATGAGGGCTGCCTGGCTTTGGCAGAGCTATTGGGATGGAAGAAAGAA
CTGGAGGAACTTGTGAAAAATGAACACGCTGTCATTGAGGCCAAATCAGGGCAGACTGTG
GGTGTCGGGGCAGGTGCTGATCGGCAACCAGCCAAGAAGCAAGAACAGAATCCATCTTTG
GAGAAAGAAAAGCCACCTGCAAACAAAGAAGAA
>green_sea_turtle_SIRT2
ATGCTTCTAACAGTAGCCACTAAAGGTAGAAAACAGTTCTGTGTTCGCACTGGCTTACTT
CACCGACAGATCTTTTTATATTTTGTTCTTCAGGACTCTGATTCTGACTCCGAAAACGGT
GGTGCCTCTGGAGAGTCTGAAATGGATTTCCTGCGAAACCTCCTGTCCCGGACGCTGGGC
CTGGGCAGCGAGAAGCCGGAGAAGGTGCTGGATGAGCTGACGCTGGACGGAGTGAGCCGC
TTCATGCAGAGCGAGAAGTGCAGGAACGTCGTCTGCATAGTGGGCGCTGGGATCTCGACC
TCTGCGGGGATCCCGGACTTCCGGTCGCCCGGCACGGGGCTCTACGCCAATCTGCAGAGC
TACAACCTGCCGTACCCTGAAGCCATCTTCGAAATCAACTACTTCAAGCAACACCCGGAG
CCGTTCTTTGCCCTTGCCAGGGAGCTGTATCCAGGCCAGTTTAAGCCCACCGTTTGCCAC
TATTTCATTCGGCTGCTGAAGGAGAAGGGTTTGCTGCTGCGCTGTTACACGCAGAACATT
GACACGTTGGAGCGGGTGGCCGGCTTGGACCAGGAAGATCTGGTGGAAGCCCACGGCACC
TTCTTCACCTCTCATTGTCTCGGCTCCTCCTGCAAGAAGAAATACACCCTGGACTGGATG
AAAGAAAAGATTTTCTCGACTGTCACCCCCAAGTGCGACAAATGTCAGAGTCTGGTGAAG
CCAGATATTGTGTTCTTTGGGGAGAACTTGCCCCCCCGCTTCTTCACACTCATGCAGTCG
GATTTCCAGAAGGTGGATCTGCTCATCATCATGGGCACGTCGCTGCAGGTGCAGCCCTTC
GCCTCCCTCGTCAGCAGGGTGCCCACAAACACCCCAAGGCTGCTGATTAACAAGGAGAAG
ACGGGGCAGAGCGATCCCTTTATGTCCCTTATGGGCCTCAGCACCGGCATGGACTTCGAC
TCAGAAAAGGCCTACAGGGATGTGGCATGGCTCGGGGAGTGCGACCAAGGCTGCACAGCA
CTGGCCGAGCTGCTGGGATGGAAGAACGAGCTGGAGGCGCTGGTGAAGAAGGAGCACTCT
GCCATCGATGCCAAATCGGGGCAGGCCGGCGAGGCCGGGGCAAGCCCCTCCCTTCCCCCG
GCTGGGGCCACGGCAAGCCCCTCCTCTCCCCCAGCGAAGCCGGGGTCATCGCCCGAGAAG
GAAAGCGACGCCGGAAACAAGGCGGAA
>African_clawed_frog_SIRT2
ATGGAGAGGAATCCTGTGAGGAAAGCGATACCAGAGAGGCAGCTGCAGGAGACTTGTGAC
AGGAGTGAGACAAGCGATGAAGGAAAGAGAAGCCCTTCATCGCATAAAACCGAATCGAGC
GAACCAGCGGATCTTAAGGCACAAGCTGAAGATTCAGAGGACTCGGACTCCAGTGAAGAC
AATAGTGGTGCATCTGAAATGGATTTCCTGCGTAACCTCTTTTCCCGTACACTGGGCATT
GGCACCCCAGAGAAAGTCCTGGATGAGCTGAGCCTGGAGAGTGTGAGCAGGTTCATGCTA
AGTGAGAAATGCAAGAATGTGATCTGTATGGTAGGGGCTGGTATCTCAACATCTGCTGGA
ATTCCGGACTTCCGTTCACCCGGAAGTGGGCTGTACTCCAACCTGCAAAAATACAACTTA
CCATATCCAGAGGCCATCTTTCAAATTGGATACTTTAAGGAAAACCCAGAACCATTTTTT
GCACTGGCTCGAGAACTATTTCCAGGGCAATTCAAGCCAACAATTTGTCATTATTTTATG
CGTCTGTTGAAGGAGAAGGGCCTGCTTCTTCGTTGCTATTCTCAGAACATTGACACCCTG
GAGAGGGTGGCAGGTCTGACTTCCGAGGACCTGGTGGAGGCTCATGGAACATTTTATAGT

TCCCATTGTGTTGGCGCCTTCTGTGGGGCTGAATATTCTCTTTCTTGGATGAAAGAGAAG
ATCTTTTCTGATCTGATCCCAAAGTGTGAAAAATGTAACAATTTGGTTAAACCAGATATT
GTTTTCTTTGGTGAAAGTTTGCCATCCAGATTCTTCTCTGCCGTTAAGTCTGACTTTCCC
AAGGTTGATCTGCTCATAGTGATGGGAACCTCTCTGCAAGTTCAACCATTTGCATCTCTT
GTCAGCAAAGTTTCTAGTAAGACCCCTCGACTTCTAATAAACAAGGAGCTGACAGGTCAG
GGCGACTCATTTTTAAGTGTTCTAGGCTTGGGGGGAGGCATGGACTTTGATTCTGAGAAA
GCATACAGGGACGTTGCATGGCTTGGAGACTGTGATGACGGTTGCCTTGCACTGGCCGAT
TTCTTGGGGTGGAAGGCTGAACTGGAAGAATTGGTAAAGAAGGAACATGCAGAAATAGAT
GCAGCAGCAGAAGCAGCAAAGAAAAAAGAAAGCAAGCCAGACAATTCATCCAAAGAAGAG
AGTTCTGCTGCAAATACTTCAGATGATAAAGCAAACAAC
>Tibetan_frog_SIRT2
ATGGCTGCAGAAGCAAAAGAAAGTGAGGAACTCAAACCCCAGGCGGAAGAATCAGAGGAA
TCAGATTCCAGCGAAGAAAACAGCAGTGCATCAGAAATGGATTTTCTGCGTGATCTTTTT
TCCCGGACGCTGGGCATTGGTACTCCGGAAAAAGTCCTTGATGAACTAACCCTTGAAGGA
GTAAGCAAATTCATGCTTAGCGACAAATGCAAGAATGTTGTGTTTATGGCTGGAGCAGGC
ATCTCAACTTCTGCAGGAATCCCTGACTTTCGTTCACCAGGCAGTGGACTATATTCAAAC
CTTCAAAAGTACAACTTGCCATATCCAGAAGCCATATTTGAAATTGGATTCTTTAAGCAA
AATCCAGAACCATTTTTTGCTCTGGCTCGAGAACTGTATCCTGGTCAGTTCAAGCCAACT
ATTTGCCATTATTTTATGCGTCTGATGAAAGAAAAAGGATTGTTACTGCGGTGCTACACC
CAGAACATAGATACACTTGAGCGGGTAGCCGGCCTCTCGTCTGATGACTTGGTAGAGGCT
CATGGAACTTTCTACACCTCTCACTGTGTTGGAACATTTTGTGGAGAGGAGTATTCTCTT
TCTTGGATGAAAGAAAAGATTTTCTCTGATCTGATCCCTAAGTGTGAAAAGTGTAACAAT
TTGGTCAAGCCAGACATTGTATTTTTTGGGGAAAGCCTTCCATCCCGCTTTTTTTCTGCT
TTGAAATCTGATTTCCCCAAGATTGATCTCCTTATTGTAATGGGAACATCACTACAAGTT
CAGCCATTTGCTTCTCTTGTTGGCAAAGTGCCTAGTAAGACACCGCGTCTTTTAATCAAC
AAAGAAAAAACTGGTCAGGGAGACTCATTTTTTGGACTGCTCGGTTTAGGAGGCGGAATG
GACTTTGATTCAGACAAAGCTTACAGGGATGTAGCCTGGCTTGGAGACTGTGATGATGGT
TGTCTTGCCTTAGCTGAATTCTTAGGATGGAAGGCTGAACTGGAAGAAATGGTGAAGAAA
GAACATGCAGCTATTGACGCAGCATCTAAGAAAACAGACAGCACCCCAGGAAAGCTGGAA
ACAAAAGAATCGCCTGAGAAAAATCAAGAGGAAAAACCATCAGAA
>southern_platyfish_SIRT2
ATGTCTGAACCATCAGAGTTCCCCAAAAAAGAAGAAGAAGGGGAGGAGGCCACCGCTGAA
CCCGAGGATCAATCAGACACCAGCAGTGAGGACGAAGCTGCAGGAGAAGCAGAGATGGAC
GTCTTGCGCGGCCTTTTCGCCAATGCGCTGGGGTTGGGCTCTTCAGAGAAAGTTCTGGAT
GAGTTGTCTCTGGAGAGCGTGGCGCGCTACATAAACAGCGGCAAATGTAAAAACATCATT
TGCATGGTTGGAGCAGGAATATCAACATCTGCTGGGATCCCTGATTTCCGCTCTCCAGAA
ACCGGTCTCTATGCAAACCTGCAAAAGTACAACCTGCCTTACCCGGAGGCCATCTTCCAG
ATCGATTACTTTAAGAATCATCCAGAACCATTCTTTGCTTTGGCCAGGGAGCTGTACCCA
GGACAGTTTAAGCCCACAATCTGCCACTACTTCATAAAGATGTTGAAGGACAAGGGGGTT
CTGAGACGATGCTACACGCAGAACATCGACACCCTGGAGCGAGTCGCAGGGATAAAAGGA
GACGATCTGATCGAAGCACATGGAACGTTCTACACCTCTCACTGTGTCAAATTGCTCTGC
AAAAAGGAGTACGACCTGGACTGGATGAAAGAAAAAATCTTCTCTGACGAGATTCCAAAG
TGTGAAAAAATGCAACAGTTTGGTCAAACCAGATATAGTATTTTTTGGAGAGAGCCTGCCA
ACCCGTTTCTTCACTACCATGAAGATGGATTTTCCTCTATGCGATCTCCTCATAGTCATG

GGCACATCTCTGCAGGTTCAACCGTTTGCAAGTTTAATCGGCAGGGTTTCAAAAAGTTGC
CCCAGGTTGCTCATTAACATGGAGAAAACAGGACAGGCAGATCCTATTTTGGGGTTGCTT
GGCTTTGGAGGAGGGATGGACTTTGACTCAGAAAATGCGTACAGAGACGTAGCTCAGATC
AGTACGTGTGATGATGGCTGCTTGGCTCTCGCTGACTTGCTGGGTTGGAAGGCGGAGCTG
GAAGAGCTTGTGAAGAACGAGCATGCCAGGATCGACAGTGAGGACAAGCAGGAAAATCCT
GGAGGAAGCGAAGGAGCTGCAGCCAAAGGAGCTGCAGCCAAAGGCGCTGCAGCCAAAGGC
GCTGCAGCCAAAGGCGCTGCAGCCAAAGGCGCTGCAGCCAAAGGCGCTGCAGCCAAAGGC
GCTCCAGCTTCAGCTGAACCAAAATCCAAAGCAGAGGAG
>guppy_SIRT2
ATGTCTGAACCATCAGAGTCCCCTAAAAAAGAAGAACAAGAGGAGCAGGCTACAGCTGAA
CCCGAGGACCAATCGGACGACAGCAGTGAAGACGAAGCTACAGGAGAAGCAGATATGGAC
TTCCTGCGCGGCCTCTTCTCCAGTGCGCTGGGATTGGGCTCTTCAGAGAAGGTTCTGGAT
GAGTTGACTCTGGAGGGCGTGGCCCGCTACATAAACAGCGGCAAATGTAAAAACATAATT
TGCATGGTTGGAGCAGGAATATCAACATCTGCTGGGATCCCGGATTTTCGCTCTCCAGAA
ACCGGTCTCTATGCAAACCTGCAGAAGTACAACCTGCCTTACCCGGAAGCCATCTTCCAG
ATCGATTACTTTAAGAAACATCCAGAACCATTCTTCGCTTTGGCCAGGGAGCTGTACCCA
GGACAGTTTAAGCCCACAATCTGCCACTACTTCATAAAGATGCTGAAGGACAAAGGGGTT
CTGAGACGATGCTACACACAGAACATCGACACCCTGGAGCGAGTCGCAGGGCTGGAAGGA
GACGATCTGATCGAAGCACATGGAACGTTCTACACTTCACACTGTGTCAAATTCCTCTGC
AAAAAGGAGTACAACCTGGACTGGATGAAAGAAAAAATCTTTTCTGACGAGATTCCAAAG
TGTGAAAAGTGCAGCAGTTTGGTCAAACCAGATATAGTTTTCTTTGGAGAGAACCTGCCA
ACCCGTTTCTTCACTTCCATGAAAATGGATTTTCCTCGATGCGATCTCCTCATAGTAATG
GGCACGTCTCTGCAGGTTCAACCGTTTGCAAGTTTAGTCAGCAGGGTTTCAAAAAGCTGC
CCTAGGTTGCTCATTAACATGGAGAAAACAGGACAGGCAGATCCGATGTTGGGGTTGCTT
GGCTTCGGAGGAGGGATGGACTTTGACTCAGACAAGGCGTACAGAGACGTAGCTCAGATC
AGTACGTGTGATGACGGCTGCTTGGCTCTCGCTGACTTGCTGGGTTGGAAGGTGGAGCTA
GAAGAGCTTGTGAAGAAGGAGCACGCCAGGATCGACAGCGAGGACAATCAGGAGAGTCCC
GGAGGAAGCAAAGGAGCTGCAGGCAAAGGCGCTTCGGCTTCAGCTTCAGCTGAACCAAAA
TCCAAAGCAGAGGAG
>American_alligator_SIRT2
ATGGCCGAGCAGGAGGCTCCGGGGGCCGCAGACGCGGGGCCAGGCCCGGGCTCCGACTCC
CCGGACTCGGACTCCGACTCGGACAGCGGCGCCGGCGCCTCCGGAGAGTCCGAGATGGAG
TTCCTGCGGACCCTGCTGTCGCGCACGCTGGGCCTGGGCGGCGAGGGGCCGGAGAAGGTG
CTGGAGGAGCTGACGCTGGAGGGCGTGAGCCGGTTCATGCTGAGCGACCAGTGCAAGAAC
GTCGTGTGCATGGTGGGAGCCGGGATCTCCACCTCTGCGGGGATCCCGGATTTCCGGTCG
CCCGGCACGGGGCTCTACGCCAACTTGGCGAGCTACAACCTGCCGTACCCCGAAGCCATC
TTCGACATCCGCTTCTTCAAGAAAAACCCAGAGCCGTTCTTCGCCCTGGCTAAGGAGCTG
TACCCGGGGCAGTTCAAGCCCACCGTGTGCCACTACTTCATGCGCCTGCTGAAGGAGAAG
GGGCTGCTGCTGCGGTGCTACACCCAGAACATCGACACGCTGGAGCGCGTGGCCGGGCTG
GCGGCCGAGGACCTGGTGGAAGCGCATGGCACCTTCTTCACCTCCCACTGCCTTGGCACC
GCCTGCCGCAAGCAATACAACCTGGACTGGATGAAAGAGAAGATCTTTTCCTCTCTGGTC
CCCAAATGTGACCAGTGCAATAACGTGGTGAAGCCTGACATCGTGTTCTTCGGGGAGAGT
CTCCCCTCCCGCTTCTTCAGTCTCATGCAGTCGGACTTCCAGAAGGTGGATCTGCTGCTC
GTCATGGGCACCTCCCTGCAGGTGCAGCCTTTCGCCTCCCTTGTCAGCAGGGTTCCTGCC

AAGACCCCCGGCTCCTCATCAACAAGGAGAAGACAGGGCAGAGCGACTTCTGCCTCTCG
CTGCTGGGCTACGGCCCCGGCCTCGACTTTGACTCCGACAAGGCCTACAGGGATGTGGCC
TGGCTCGGGGACTGTGACGCCGGCTGTGCGGCACTGGCCGAGCTCCTGGGCTGGAAGACG
GAGCTGCAGGAGCTAGTGAAGAGAGAGCACGCCGCCATTGACACCAAGTCCCAGCAGCCC
CCCAGGGACTCCGCAGCCGGCTCCACTCCCCCCCCGCAGCCGGCCCAGACGCCGGGGTCC
TCGTCAGAGAAGAGCAGCGGTGCTGAGGGCAAGGAG
>Armadillo_SIRT2
ATGGCTGAACCGGACCCCTCTGACCCTCTGGAGACCCAGGCAGGGAAGGTGCAGGAGGCT
CAGGACTCAGGTTCTGACACTGAGGAAGGAGCAACTGGAGGAGAAGCAGAGATGGACTTC
CTGCGGAATTTCTTCTCCCAGACACTGGGCCTGGGCACCGAGAAGGAGCGACGACTGGAT
GAACTCACTCTGGAAGGGGTGTCCCGCTACATGCAGAGCGAGCGCTGTCGCAGGGTCATC
TGTTTGGTGGGAGCTGGAGTCTCCACGTCCGCGGGCATTCCTGACTTCCGCTCCCCATCC
ACGGGCCTCTACGCCAACCTGGAGAAGTACCATCTTCCCTACCCGGAGGCCATCTTTGAG
ATTGGCTACTTCAAGAAACACCCAGAGCCCTTCTTCACCCTCGCCAAGGAGCTCTACCCT
GGGCAGTTTAAGCCGACCATCTGTCACTACTTCATCCGCCTGTTGAAGGAGAAAGGGCTG
CTGCTGCGTTGCTACACGCAGAACATAGACACCCTGGAGCGAGTGGCCGGCCTGGAGCCC
GAGGACCTGGTGGAGGCCCACGGCACCTTCTACACGTCGCACTGCGTTAGCGCCGGCTGC
CGGCGGGAGTACCCGCTGAGCTGGATGAAAGGTGAGGCTGCAGGTCAGCCAGCGGGCGCC
CAGCTCGCCGTCCCCGCCTCAGCCCCAGGAGCAGGGTCCTCTTACCCCCTACCCCCCCAC
CCACCCCCGCCCTCCGCACAGACTGACCCCTTCCTGGGAATGATGATGGGCCTGGGAGGC
GGCATGGACTTTGACTCCAAGAAGGCCTACAGGGACGTGGCCTGGGTGGGTGACTGCGAC
CAGGGCTGCCTGGCCCTCGCCGACCTCCTGGGATGGAAGAAGGAGCTGGAGGACCTGGTC
CAGAAGGAGCATGCCACCATCGACGCCCAGTCGGGGCCGGGGGCTCCCAACCCCACCACC
TCAGCTTCCCCCAGGAAGTCTCCACCACCCACCAAGGAGGAGGCCAGAACCACGGAGAAG
GAGAAAGCCCAG
>Chicken_SIRT2
ATGTCCGAAGCGGACGCTCCCCCTCCCGGGGCTGATCCCGACGCTGAGGCTGAGGGCGGC
TCCGAGGAAGACTCCGATATGGAGCTGCTGCGGAACCTCCTGGCACGGACGCTGGGGTTG
GGCACGGAGCCGCCGGAGCGCGTTCTGGATGAGCTGTCGCTGGCGGGAATCGCCCGGTTC
ATGCAAAGCGAGCGCTGCCGCCGTGTGGTGTGCATGGTGGGCGCCGGCATCTCCACGGCC
GCTGGAATCCCCGACTTCCGCTCACCCGGAACCGGGCTGTACGCCAACCTGGGCCGCTAC
GAACTGCCCTACCCCGAGGCCATCTTTGACATCAGCTATTTCAAGCAACACCCAGAGCCT
TTCTTTGCGTTGGCTAAGGAGCTGCTCCCAGGGCAGCTGAAGCCCACCGTTTGCCACTAC
TTCATGCGGCTGCTGAAGGAGAAGGGGCTGCTGCTGCGCTGCTACACACAGAACATCGAC
ACGCTGGAGCGCGTGGCGGGGCTGCAGCCCGAGGAGTTGGTGGAGGCTCACGGCACCTTC
CAAACCGCGCACTGCCTGCGCTCCTCCTGCCGGCATCAGTATGACCTGAGCTGGGTGAAG
GAGAAAATCTTCTCTTCCCTCGTTCCCAAGTGCGACAAATGCCAGAGCGTGGTGAAGCCA
GACATCGTATTTTTTGGTGAGAATCTTCCATCTCGCTTCTTCAGTCTTCTGCAGTCGGAC
TTCCAGAAGGTTGATCTTCTCATCATCATGGGTACTTCACTGCAGGTCCAGCCCTTCGCC
TCCCTTGTTAGCAGGGTCCCTGCCAGCACCCCCAGGCTCCTCATCAACAAGGAGAAAACA
GGGCAGAGCGATGTCTTCATGTCCCTCATGGGCTTCGGCTGCGGGATGGACTTCGACTCG
GACAAAGCCTACAGGGACGTGGCCTGGCTGGGGGACTGCGATGAGGGCTGCCTGGCACTG
GCAGAGCTGCTGGGCTGGAAGAAGGAGCTGCAGGAGCTGGTGAGGAAGGAGCACGCCGCC
ATTGACGCCGTGGCAGCCCCGGAGGACACGAGCAGCGCCTCAGGGGGAGACCCCACGTCC

CGGAGGGGCCGCACCGATGGCTCGGGGGGCCGCGCTGAGAGCGGCGGAGCTTCCAGCGAA
CAGAGGGGGGACGACAAAGAGCCG
>Chinese_softshell_turtle_SIRT2
ATGGATTTCCTGCGAAATCTCCTGTCCCGGACGCTGGGCCTGGGCAGTGAGAAACCGGAG
AAGGTGCTGGATGAGCTGACACTAGATGGTGTGAGCCGCTTCATGCTGAGCGAGAAGTGC
AGGAACATCGTCTGCATGGTGGGCGCTGGGATTTCAACCTCCGCGGGGATCCCAGACTTC
CGTTCGCCCGGCACCGGGCTCTACGCCAACCTGCAGAGCTACAACCTGCCATACCCGGAA
GCCATCTTCGAAATCAGCTACTTCAAGCAACACCCGGAGCCGTTCTTTGCCCTCGCCAGG
GAGCTGTACCCAGGGCAGTTCAAGCCCACCGTGTGCCACTACTTCATCCGGCTGCTGAAG
GAGAAGGGCTTGCTGCTGCGCTGTTACACACAGAACATTGACACGCTGGAGCGGGTGGCC
GGATTGGACCAGGACGATTTGGTGGAGGCTCACGGCACCTTCTTCACCTCTCACTGCCTC
AGCCCCTCCTGCAAGAAGCAGTACAGCCTGGACTGGATGAAAGAAAAGATTTTCTCGTCT
CTGACGCCCAAGTGCGACAAATGTCAGAGTCTGGTGAAGCCGGATATCGTCTTCTTCGGG
GAGAGCCTGCCCCCCCGCTTCTTCGCACTCATGCAGTCGGATTTCCAGAAGGTGGATTTG
CTTATCATCCTGGGCACGTCGCTGCAGGTGCAGCCCTTCGCCTCCCTCGTCAGCAGGGTG
CCCACAAACACCCCCAGGCTCCTGATTAACAAGGAGAAGACAGGGCAGAGCGATCCCTTC
ATGTCGCTGATGGGTCTCGGCAGCGGCATGGACTTCGACTCAGAAAAGGCCTACAGGGAC
GTGGCCTGGCTGGGGGACTGTGCTGGAGGCTGCACAGCACTGGCAGAGCTGCTGGGATGG
AAGAAGGAGCTGGAGGAGCTGGTGAGAAAGGAGCATGCTGCCATTGATGCTGGATCAGGG
CAGGCCAGCGAGGCTGGGGCGAGCCCCTCCCCTCCCCCAGTGAAGCCGGCGGGGCCCTTG
CCCGAGAAGGAGGGCGGCGCTGGAAGCAAAGCAGAA
>Cod_SIRT2
AACCAATCAGAAGACAGCAGCGAGGACGAAGCCTCCGGGGCGACAGAGATGGACTTTCTG
CGTAACCTCTTCTCCAGCACCCTGGGCCTTGGCTCGGTGGAGAAGGTTCTAGATGAGCTC
AACCTGGAAGGGTTGGCGCGTTACATACAGAGTGGAAAATGTAAAAATATAGTCTGCATG
GTGGGAGCAGGGATATCCACCTCGGCTGGGATTCCAGACTTTCGCTCCCCAGGGACTGGT
CTCTATGCCAACCTGCAGAAATATAACCTGCCGTATCCAGAGGCCATCTTTCAGATTGAC
TACTTCAAGAAACATCCTGAGCCATTCTTTGCCCTGGCTAGGGAACTCTACCCAGGACAG
TTTAAGCCTACAGTATGTCACTACTTCATGAAGATGCTGAAGGACAAGGGACTGCTCAGG
CGCTGCTACTCACAGAACATCGACACCCTGGAACGGGTGGCCGGTCTGGAGGGAGATGAC
CTGATCGAGGCTCATGGAACGTTCTTCACCTCCCACTGCGTCAGCTTCTCCTGCCGCAAA
GAGTACAACCTGGAGTGGATGAAAGAGAAGATCTTCTCTGACGACATCCCCAAGTGTGAC
AAGTGTAGCAGTCTGGTCAAGCCAGATATCGTCTTCTTCGGAGAGAGTCTTCCTGTCAGA
TTTTTCACTTCAATGAAGATGGACTTCCCGCAGTGTGACCTCCTCATCATTATGGGAACG
TCCCTGCAGGTCCAGCCTTTCGCCGGTCTAGTGGGCAGAGTTTCCAAGAGCTGCCCCAGA
CTGCTCATTAACATGGAGAAGGCAGGACAGAGGCTGACCCGTTCTTTGGGGTGTACTTCG
GGGGAGGGATGGACTTTGACTCAGAAAAGGCTTACAGTCTTTAGGGATGTAGCTCACATC
AGTACGTGTGACGAGGGCTGTCTGGCTCTTGCTGACCTGCTTGGATGGAAG
>Cow_SIRT2
ATGGCCGATCCGGATCCCTCTGACCCTGAGGAGACCCAGGCAGGGAAGGTGCAGGAGGCT
CAGGACTCAGATTCAGACACTGAGGCAGGAGCCACTGGCGGAGAAGCAGAGATGGACTTC
CTGCGGAATTTCTTCTCCCAGACTCTGGGCCTGGGCACCCAGAAGGAGCGACTCCTGGAC
GAACTAACCCTGGAAGGAGTGAGCCGCTACATGCAGAGCGAGCGCTGTCGCAGGGTCATC
TGTTTGGTGGGAGCTGGAATCTCCACTTCCGCGGGTATCCCTGRCTTTCGGTCCCCAAAC

ACGGGCCTCTATGCCAACCTGGAGAAATACCGTCTTCCCTACCCGGAGGCCATCTTTGAA
ATCAGCTACTTCAAGAAACATCCAGAGCCCTTCTTTGCTCTCGCCAAGGAACTCTATCCT
GGGCAGTTCAAGCCCACCATCTGCCACTACTTCATCCGCCTGCTGAAGGAAAAAGGACTG
CTCCTGCGCTGCTACACACAGAACATAGACACCCTGGAGCGAGTGGCTGGGCTGGAGCCC
GAAGACCTGGTGGAGGCCCACGGCACCTTCTACACGTCCCACTGCATCAGCTCAGGCTGC
CGGCAGGAGTACTCACTAAGCTGGATGAAAGAGAAGATCTTCTCCGAGGTGACTCCCAAG
TGTGAGAAATGTCAGAGCGTGGTGAAGCCTGATATCGTGTTCTTCGGCGAGAACCTCCCA
GCGCGTTTCTTCTCCTGCATGCAGTCAGACTTCCTAAAGGTGGACCTCCTTATCATCATG
GGCACCTCCCTGCAGGTGCAGCCCTTCGCGTCCCTCATCGGCAAGGCGCCCCTGTCGACC
CCGCGCCTGCTCATCAACAAGGAGAAGACTGGCCAGACTGACCCTTTCCTCGGGATGATG
ATGGCCCTCGGAGGAGGCATGGACTTTGACTCCAAGAAGGCCTACAGGGACGTGGCCTGG
CTGGGCGACTGTGACCAGGGCTGCCTGGCCCTCGCCGACCTCCTTGGATGGAAGAAGGAG
CTGGAGGACCTTGTTCGGAAGGAGCATGCCAGCATAGATGCCCAGTCGGGGTCGGGGGCC
TCCAACCCCGCTACCTCAGCTTCCCCCAGGAATTCTCCACCACCTCCCACCAAGGAGGAG
CCCAGGACCACTGAGGGAGAGAAACCCCAG
>Dog_SIRT2
ATGGACTTCCTGCGGAACTTATTCTCCCAGACACTGGGCCTGGGCACCCAGAAGGAGCGT
CTGCTGGACGACCTCACCCTGGAGGGGGTGACCCGCTACATGCAGAGCGAACGCTGTCGC
AGGGTCATCTGTTTGGTGGGAGCTGGGATCTCCACGTCTGCCGGCATCCCTGACTTCCGC
TCCCCATCCACGGGCCTCTACGCCAACCTAGAGAAGTACCACCTTCCCTACCCAGAGGCC
ATCTTTGAGATTGGCTACTTCAAGAAACACCCAGAACCTTTCTTTGCTCTCGCCAAGGAA
CTCTATCCTGGCCAGTTCAAGCCAACTGTGTGTCACTACTTCATCCGGCTGCTGAAGGAG
AAGGGGCTGCTGCTGCGATGCTACACACAGAACATAGACACCCTGGAGCGGGTGGCCGGG
CTGGAACCTGAGGACCTGGTGGAGGCCCACGGCACCTTCTACACCTCGCACTGTATCAGC
CCCCTGTGCCGACGGGAGTACCCGCTCAGCTGGATGAAAGAGAAGATCTTCTCCGAGGTG
ACTCCCAAGTGTGAGAAATGTCACAGCGTGGTGAAGCCTGACATCGTGTTCTTCGGCGAG
AACCTCCCAGCAAGGTTCTTCTCCTGCATGCAGTCAGACTTCCTGAAGGTGGACCTCCTC
ATCATCATGGGTACCTCCCTGCAAGTGCAGCCCTTTGCTTCCCTCATCAGCAAGGCGCCC
CTCTCTACCCCGCGCCTGCTCATCAACAAGGAGAAGACTGGGCAGACTGACCCTTTCCTG
GGGATGATGATGGGCCTTGGAGGAGGCATGGACTTCGACTCCAAGAAGGCCTACAGGGAC
GTGGCCTGGCTGGGTGACTGTGACCAGGGCTGCCTGGCCCTTGCCGACCTCCTCGGATGG
AAGAAAGAACTGGAGGACCTTGTCCGGAAGGAGCATGCCCACATAGACGCCCAGGCAGGG
TCAGGGGCCCCCAACCCCAGCACTTCAACTTCAGCTTCTCCCAGCAAGTCTCCACCTCCT
GCCAAGGAGGAGGCCAGGACCAAGGAGGGAGAGAAACCCCAA
>Dolphin_SIRT2
ATGGCTGAGCCGGATCCCTCTGACCCTGTGGAGACCCAGGCAGGGAAGGTGCAGGAGGCT
CAGGACTCAGATTCAGACACTGAGGGAGGAGCGGCTGGCGGAGAAGGAGAGATGGACTTC
CTGCGGAATTTCTTCTCCCAGACACTGGGCCTGGGCACCCAGAAGGAGCGTCTGCTGGAC
GAATTAACCCTGGAAGGGGTGACCCGCTACATGCAGAGTGAGCGCTGTCGCAGGGTTATC
TGTTTGGTGGGAGCTGGAATCTCCACTTCGGCGGGCATCCCTGACTTCCGCTCCCCAAAC
ACGGGCCTCTACGCCAACCTGGAGAAATACCATCTTCCCTACCCGGAGGCCATCTTTGAG
ATTGGCTACTTCAAGAAACACCCAGAGCCCTTCTTTGCCCTCGCCAAGGAACTCTATCCT
GGGCAGTTCAAGCCCACCGTCTGCCACTACTTCATCCGCCTGCTGAAGGAGAAAGGGCTG
CTCCTGCGCTGCTACACGCAGAACATAGACACCCTGGAGCGAGTGGCGGGGCTGGAGGCC

GAGGACCTGGTGGAGGCCCACGGCACCTTCTACACGTCTCACTGCATCAGCCCCGTCTGC
CGGCAGGAGTACGCGCTAAGCTGGATGAAAGAGAAGATCTTCTCCGAGGTGACTCCCAAG
TGTGAGAAATGTCGGAGTGTGGTGAAGCCTGATATCGTGTTCTTTGGCGAGAACCTCCCG
GCGCGTTTCTTCTCCTGCCTGCAGTCAGACTTCCTGAAGGTGGACCTCCTCATCATCATG
GGCACCTCCCTGCAGGTGCAGCCCTTCGCATCCCTTATCAGCAAGGCGCCCCTCTCCACC
CCGCGCCTGCTCATCAACAAGGAGAAGACTGGCCAGATGGACCCTTTCCTCGGGATGATG
ATGGGCCTCGGAGGAGGCATGGACTTCGACTCCAAGAAGGCCTACAGGGATGTGGCCTGG
CTGGGTGACTGCGACCAGGGCTGCCTGGCCCTTGCCGACCTCCTCGGATGGAAGAAGGAA
CTGGAGGACCTTGTCCGGAAGGAGCATGCCAGCATAGATGCCCAGTCAGGGTCAGGGGCT
CCCAACCCCACCACTTCAGCTTCCTCCAGGAAGTCTCCACCTCCTGCCAAGGAGGAGGCC
AGGACCACGGAGGGAGAGAAACCCCAG
>Elephant_SIRT2
ATGGCCGAGCCGGACCCCTCTGACCCTCTGGAGACCCAGGCAGGGAAGGTGCAGGAGGCT
CAGGACTCAGATTCGGATTCAGACACTGAGGAAGGAGCCACTGGCGGAGAAGCTGAGATG
GACTTCCTGCGGAATTTCTTCTCCCAAACGCTGGGCCTGGGCTCTGAGAGGGAGCGTCTG
CTGGATGAGCTAACCCTGGAAGGGGTGACCCGCTACATGCAGAGCGAGCGCTGTCGCAGG
GTCATCTGCTTGGTGGGAGCTGGAATCTCCACGTCCGCGGGCATCCCTGACTTCCGCTCA
CCGACCACCGGCCTCTATGCCAACCTGGAGAAGTACCGTCTTCCCTACCCAGAGGCCATT
TTCGAGATTGGCTACTTCAAGAAACATCCAGAGCCCTTCTTTGCCCTCGCCAAGGAGCTC
TATCCTGGGCAGTTTAAGCCCACCACCTGCCACTACTTCATCCGCCTGCTGAAGGAGAAG
GGGCTCCTCCTGCGCTGCTACACGCAGAACATAGACACCTTGGAGCGTGTGGCGGGGCTC
GAGCCTGAGGACCTGGTGGAGGCCCATGGCACCTTCTACACGTCACACTGCCTCAGCCCC
CTCTGCCGGCGGGAGTACACACTAAGCTGGATGAAAGAGAAGATCTTCTCCGAGGTGACT
CCCAAGTGTGAGAAATGTCAGAGCCTGGTGAAGCCTGACATCGTGTTCTTCGGCGAGAGC
CTCCCGGCGCGTTTCTTCTCCTGCATGCAGTCAGACTTCCTGAAGGTGGACCTCCTCATC
ATCATGGGCACCTCCCTGCAGGTGCAGCCCTTCGCATCCCTCATCAGCAAGGCACCCCTC
TCCACCCCGCGCCTCCTCATCAACAAGGAGAAGACGGGCCAGACCGACCCCTTCCTGGGC
ATGATGATGGGTCTTGGAGGAGGCATGGACTTCGACTCCAAGAAAGCCTACAGGGATGTG
GCCTGGCTGGGTGATTGTGACCAGGGCTGCCTGGCCCTTGCTGACCTCCTCGGATGGAAG
AAGGAACTGGAGGATCTTGTCCAGAAGGAGCATGCAGCCATAGATGCCCAGTCGGTGTCA
GGGGACCCCAACCCCGGCATTTCTACTTCCCCCAGGAAGTCTCCACCACCCGCCAAGGAG
GAGGCCAAAACCACGGAGGGGGAGAAACCCCAG
>tropical_clawed_frog_SIRT2
ATGTACATTGAATCCATTTGTACCAATGGGCAGACGGCAAAGGCGGATTCAATGTTCTCA
CGTGATCCACTGTTGTTGTTAAACCCGGAAGTGGCCGAAGGTTCAATGGCTGCAGAAGCT
AGCAAACCAGCGGATCTCACGGCACAAGCTGAAGACTCAGAGGACTCAGACTCCAGTGAA
GACATTAGTGGTGCATCTGAAATGGACTACCTACGGAATCTCTTCTCCCGCACACTGGGC
ATAGGAACCCCAGAGAAAGTTCTGGATGAGTTGAGCATAGAAGGCATAAGCAGGTTCATG
CTAAGTGAGAAATGCAAGAATGTGGTTTGTATGGTAGGGGCTGGCATCTCGACATCTGCT
GGCATTCCTGACTTCCGTTCCCCAGGCAGTGGGCTGTACTCCAACCTGCAAAAATACAAC
TTACCATATCCAGAGGCCATTTTTCAAATTGGATACTTTAAGGAAAATCCAGAACCTTTT
TTTGCTTTGGCTCGAGAACTATTTCCAGGGCAATTTAAGCCAACAATTTGTCATTACTTT
ATTCGCCTACTGAAAGAAAAAGGCCTGCTTCTTCGTTGCTATTCTCAGAACATTGACACC
CTGGAGAGAGTGGCCGGTCTGACTTCTGATGACCTGGTGGAGGCTCATGGAACTTTTCAT

AGTTCCCATTGTGTTGACACATTCTGTAGGGCTGAGTATTCTCTCTCCTGGATGAAGGAA
AAGATCTTTTCTGATCTGATCCCAAAGTGTGAAAAATGTAACAATTTGGTTAAACCAGAT
ATTGTTTTCTTTGGTGAAAGTTTGCCATCCAGATTCTTCTCTGCCATTAAGTCTGACTTT
CCCAAGGTTGATCTGCTCATAGTGATGGGAACCTCTCTGCAAGTTCAGCCATTTGCATCT
CTTGTCAGCAAAGTGTCTAGTAAGACCCCTAGACTTCTAATAAACAAGGAGCTGGCAGGA
CAGGGTGACCCATTTTTCAGTGTTTTTGGCTTTGGAGGAGGCATGGACTTTGATTCTGAG
AAAGCATACAGGGACGTTGCATGGCTTGGAGACTGTGATGATGGCTGCCTTGCACTAGCT
GATTTCTTGGGGTGGAAGGCTGAATTGGAAGAATTAGTAAAGAAGGAACATGCAGCAATA
GATGCAGCAGCAGAAGCAGCAAAGAAAAAAGAAAGCAAGCCAGGGGGCTCATCCAGTGAA
GAAAGTTCTGCAGCAAATGCTTCAGATGAGAAAGCAAATAAC
>Fruitfly_SIRT2
ATGGATAAGGTTCGACGCTTCTTTGCAAACACTCTACATCTCGGCGGCTCCTCGGACGCA
AAGGAGGAAGTTAAAGTAGAGAAGGTTATTCCAGATTTGAGTTTTGACGGCTTTGCCGAA
CATTGGCGTGTGCATGGTTTCCGCAAGATTGTGACCATGGTGGGAGCCGGCATATCTACA
TCTGCTGGCATTCCGGATTTCAGATCCCCAGGTTCCGGGTTATACAGCAATCTGAAGAAG
TACGAGCTGCCACATCCTACGGCCATATTCGATCTGGACTACTTCGAAAAGAATCCAGCG
CCGTTTTTTGCACTGGCCAAGGAACTTTACCCTGGATCCTTCATTCCCACGCCGGCCCAC
TACTTTATCCGTTTGCTTAACGACAAGGGACTACTGCAGCGCCACTACACCCAGAACATA
GACACGCTAGACCGGCTTACTGGCCTGCCCGAGGACAAGATCATTGAGGCCCATGGCAGC
TTCCACACCAACCACTGCATTAAGTGCCGCAAGGAGTACGACATGGACTGGATGAAGGCG
GAGATCTTCGCCGATCGTCTGCCCAAATGCCAAAAGTGCCAAGGCGTTGTTAAACCAGAC
ATTGTTTTCTTTGGCGAAAACTTGCCAAAGAGATTTTACTCCAGTCCTGAAGAGGATTTC
CAGGATTGCGATCTGCTGATCATCATGGGCACATCGTTGGAAGTGCAACCGTTTGCTTCA
CTGGTGTGGAGACCAGGACCACGTTGCATTCGCCTCTTGATCAATCGCGATGCAGTGGGT
CAGGCCAGCTGTGTGCTCTTTATGGATCCCAACACGCGATCGCTACTCTTCGATAAGCCC
AATAACACTAGGGATGTGGCCTTTCTGGGCGACTGTGATGCTGGCGTAATGGCTCTGGCC
AAAGCCTTGGGCTGGGACCAAGAGCTGCAGCAGCTAATTACAAGTGAAAGGAAGAAACTG
AGCGGCAGCCAGAATAGTGAGGAGCTGCAACAAGGCAAAGAGAAACCGCAATCGGACCCG
GATAAGATGACTTCGGGCGATAGGGACAAGAAGGATGCTTCGCTT
>Mouse_SIRT2
ATGGACTTCCTGAGGAATTTATTCACCCAGACCCTGGGCCTGGGTTCCCAAAAGGAGCGT
CTTCTAGACGAGCTGACCCTCGAAGGAGTGACACGCTACATGCAGAGCGAGCGCTGCCGC
AAGGTCATCTGTTTGGTGGGAGCCGGAATCTCCACGTCCGCGGGTATCCCTGACTTCCGC
TCCCCGTCCACTGGCCTCTATGCAAACCTGGAGAAGTACCACCTTCCTTACCCAGAGGCC
ATCTTTGAGATCAGCTACTTCAAGAAACATCCGGAACCCTTCTTTGCCCTTGCCAAGGAG
CTCTATCCCGGGCAGTTCAAGCCAACCATCTGCCACTACTTCATCCGCCTGCTGAAGGAG
AAGGGGCTGCTGCTGCGCTGCTACACGCAGAACATAGACACGCTGGAACGAGTGGCGGGG
CTGGAGCCCCAGGACCTGGTGGAGGCCCACGGCACCTTCTACACATCACACTGTGTCAAC
ACCTCCTGCAGAAAAGAATACACGATGGGCTGGATGAAAGAGAAGATCTTCTCAGAAGCA
ACTCCCAGGTGTGAGCAGTGTCAGAGTGTGGTAAAGCCTGATATCGTGTTTTTCGGTGAG
AACCTTCCATCGCGCTTCTTCTCCTGCATGCAGTCAGACTTCTCCAAGGTGGACCTCCTC
ATCATCATGGGCACCTCCCTGCAGGTGCAGCCCTTCGCCTCCCTCATCAGCAAGGCACCA
CTAGCCACCCCACGGCTGCTCATTAACAAGGAAAAGACAGGCCAGACGGACCCCTTCCTG
GGCATGATGATGGGCCTGGGAGGTGGCATGGATTTTGACTCCAAGAAGGCTTACAGGGAC

GTGGCCTGGCTGGGTGACTGTGATCAAGGCTGCCTGGCTCTCGCTGACCTCCTCGGATGG
AAGAAGGAACTGGAAGACCTTGTCCGGAGGGAGCATGCCAACATAGATGCCCAGTCAGGG
TCACAGGCCCCCAACCCCAGCACTACCATCTCCCCTGGAAAGTCCCCACCGCCTGCCAAG
GAGGCGGCCAGGACCAAAGAGAAAGAGGAACAGCAG
>Zebrafish_SIRT2
ATGTCTGAAGAGGTTTCTAAAAGGGTAGAAGAGGAGGCTGACACACCAGGTCTCGAGGGT
CAGTCAGATGATAGCAGTGACGAGGGTGACGCTTCAGGAGATACTGAGATGGATTTTTTG
CGCAGTCTTTTCTCGCGAACACTTGGCCTTAGTCCTGGAGATAAAGTCTTGGATGAGTTA
ACCCTGGACTCAGTTGCTCGCTACATACTGAGTGGCAAATGTAAGAACATTATCTGTATG
GTCGGAGCTGGGATATCTACATCTGCAGGAATCCCAGATTTCCGCTCTCCTGGCACGGGC
TTATATGCAAATCTGCAGAAGTACAACCTGCCTTACCCAGAGGCCATCTTTCAGATAGAC
TATTTCAAGAAACATCCGGAGCCCTTCTTTGCTCTGGCCAGAGAACTGTACCCAGGACAG
TTTAAGCCCACAGTGTACCACTATTTCATAAAGATGCTGAAAGACAAAGGCTTACTGAGG
CGCTGTTATTCTCAAAATATTGATACTCTAGAAAGGGTTGCAGGACTGGAAGGAGAGGAC
CTGATTGAAGCTCATGGTACATTTCACACATCTCACTGTGTGAGCTTCCTCTGTCGTAAA
GAGTACAGCATGGACTGGATGAAAAATCAAATTTTCTCTGAAGAAATTCCTAAGTGCGAT
TCCTGTGGAAGCCTCGTAAAACCCGATATTGTGTTTTTTGGGGAAAGTCTGCCTTCCCGA
TTTTTCACTTCAATGAAAGCGGACTTTCCTCAATGTGACCTTCTGATCATAATGGGGACA
TCTTTGCAGGTTCAGCCATTTGCATCTCTAGTGAGCAGAGTCTCAAACCGCTGTCCACGA
TTGCTGATCAATATGGAGAAAACTGGACAGTCTGAATTTGGCATGGGGCTATTCAGTTTT
GGAGGCGGAATGGATTTTGATTCAGATAAGGCCTACAGAGATGTGGCTCATCTGAGCACC
TGTGACGATGGCTGCATGACTCTCGCTGAACTGCTGGGCTGGAAAAAAGAGCTGGAGGAG
ATGGTGAAGCGTGAACATGCTTTGATTGACAGCAAAGATGCCAAAAAGACTGACAAAGAA
GCCAGTCAGAGCTCCAAAAGTGCTGTGGCGGAGGCAGAGAAGACTGACAAAACAGAA
>Rabbit_SIRT2
ATGGCTGCCACCGCCAGGCCGTCTGGGACTCGTAGTTCCGCCTTCGGGCGCGCACGGGGC
GTTTGCGGGAAAGCCCTGTATGCGGCCGAGATGGGTGCTGGGAGTCGTAGTTCTATGCCC
GACGCCGTCCCCTTCTGTTTCCGGTGCCGTCACGGGACAGAGCAGTCGGTGACAGCCCCG
AGGGCCCCCGCCCCGAGCCCATGGCCGAGCCGGATCGATTCCGATTCTGACTCCGAGGGA
GGAGCGGCTGGCGGAGAAGCAGAGATGGACTTCCTGCGGAATCTGTTCTCCCAGACCCTG
GGCCTGGGTACCCAGAAGGAGCGGCTGCTGGACGAGCTGACCCTGGAAGGCGTGAGCCGC
TTCATTCAGAGCGACCGCTGTCGCAGGATCATCTGCCTGGTGGGCGCTGGCATCTCCACG
TCCGCGGGCATCCCTGACTTCCGGTCTCCGTCCACCGGCCTCTATGCCAACCTGGAGAAG
TACCGGCTGCCCTACCCAGAGGCCATCTTTGAGATCGGCTACTTCAAGAAACATCCGGAA
CCCTTCTTCGCTCTTGCCAAGGAACTCTATCCCGGGCAGTTCAAGCCCACCACCTGCCAC
TACTTCATCCGGCTGCTGAAGGACAAGGGGCTGCTGCTGCGCTGCTACACGCAGAACATA
GACACCCTGGAGCGCGTGGCGGGGCTGGAGCCAGAGGACCTGGTGGAGGCCCACGGTACC
TTCTACACATCACACTGTATCAGCTCCCTGTGCCGGCGTGAGTACTCGCTGGACTGGATG
AAAGAGAAGATCTTCTCGGAGGTGACTCCCAAGTGTGAGAAGTGCCAGAGCGTGGTGAAG
CCTGACATCGTATTTTTCGGAGAGAACCTGCCAGCGCGCTTCTTCTCCTGCATGCAGTCA
GACTTCCTGAAGGTGGACCTGCTCATCGTCATGGGCACCTCCCTGCAGGTGCAGCCCTTC
GCGTCCCTCATCGGCAAGGCACCCCTGTCCACCCCGCGCCTGCTCATCAACAAGGAGAAG
ACCGGCCAGACGGACCCCTTCCTGGGGATGATGATGGGCCTGGGAGGAGGCATGGACTTT
GACTCCAAGAAGGCCTACAGGGACGTGGCCTGGCTGGGGGATTGTGACCAGGGCTGCCTG

GCCCTCGCCGACCTCCTCGGATGGAAAAAGGAGCTGGAGGACCTTGTCCAGAAGGAGCAC
GCCCGCATAGACGCCCAGCTGGGATCGGGGACCCCCAACCCCACCACTGCAGCCTCCCCC
AAGACGGCCCCACTGCCTGCCCAGGAAGAGGCCAGGACCGCAGAGGGAGAGAAACCCCAG

>Chinese_alligator_SIRT2
GGGGCCGCAGACGCGGGGCCAGGCCCGGGCTCCGACTCCCCGGACTCCGACCTGGACTCG
GACAGCGGCGCCGGCGCCTCCGGAGAGTCCGAGATGGAGTTCCTGCGGACCCTGCTGTCG
CGGACGCTGGGCCTGGGCGGCGAGGGGCCGGAGAAGGTGCTGGAGGAGCTGACGCTGGAG
GGCGGGAGCCGGTTCATGCTGAGCGACCAGTGCAAGAACGTCGTGTGCATGGTGGGAGCC
GGGATCTCCACCTCTGCAGGGATCCCGGATTTCCGGTCGCCGGGCACGGGGCTCTACGCC
AACCTGACGAGCTATAACCTGCCGTACCCAGAAGCCATCTTCGACATCCGCTTCTTCAAG
AAAACCCCCGAGCCGTTCTTCGCCTTGGCTAAGGAGCTGTACCCGGGGCAGTTCAAGNCC
GCCGTGGGCCACTACTTCAGGCGCCTGCTGAAGGAGAAGGGGCTGCTGCTGCGGTGCTAC
ACCCAGAACATCGACACGCTGGAGCGCGTGGCCGGGCTGGCGGCCGAGGACCTGGTGGAA
GCGCATGGCACCTTCTTCACCTCCCACTGCCTTGGCACCGCCTGCCGCAAGCAATACGAC
CTGGACTGGATGAAAGAGAAGATCTTTTCCTCCCTGGTCCCCAGATGTGACCAGTGCAAT
AACGTGGTGAAGCCTGACATCGTGTTCTTCGGGGAGAGTCTCCCCTCCCGCTTCTTCAGT
CTCATGCAGTCGCTTTGTCTGTCCTTCTCCCCCCGCAGGGATGTGGCCTGGCTCGGGGAC
TGTGACACCGGCTGTGCGGCACTGGCCGAGCTCCTGGGCTGGAAGACGGAGCTGCAGGAG
CTAGTGAAGAGAGAGCACGCCGCCATTGACGCCAAGTCCCAGCAGCCCCCCGGGGACTCC
GCAGCCGGCTCCGCTCCCCCCCCGCAGCCGGCCCAGACGCCGAAGTCCTCGTCAGAGAAG
AGCAGCGGTGCCGAGGGCAAGAAGGAG
>Yeast_SIRT2
ATGACCATCCCACATATGAAATACGCCGTATCAAAGACTAGCGAAAATAAGGTTTCAAAT
ACAGTAAGCCCCACACAAGATAAAGACGCGATCAGAAAACAACCCGATGACATTATAAAT
AATGATGAACCTTCACATAAGAAGATAAAAGTAGCACAGCCGGATTCCTTGAGGGAAACC
AACACAACAGATCCACTTGGGCACACTAAAGCTGCGCTCGGAGAAGTGGCATCGATGGAG
CTCAAACCAACTAATGACATGGATCCCTTGGCAGTGTCAGCAGCTTCAGTAGTGTCAATG
TCCAATGACGTTTTGAAACCAGAGACGCCCAAGGGGCCAATCATAATCAGTAAAAACCCA
TCAAATGGTATTTTCTATGGTCCCTCCTTCACTAAACGAGAGTCTCTCAATGCTCGAATG
TTTCTGAAATACTATGGTGCACACAAATTTTTAGACACTTACCTCCCCGAGGATTTGAAC
TCGTTATACATTTACTATCTTATCAAGTTGCTAGGCTTTGAAGTTAAAGATCAAGCGCTT
ATCGGCACCATCAACAGTATTGTCCATATCAACTCGCAAGAGCGTGTTCAAGATTTGGGA
AGTGCAATATCTGTCACAAATGTTGAAGACCCATTGGCAAAAAAGCAAACAGTTCGTCTA
ATCAAAGATTTGCAAAGAGCAATTAACAAAGTTCTATGTACAAGATTAAGATTATCCAAT
TTTTTCACTATTGATCATTTTATTCAAAAATTACATACCGCTAGAAAAATTTTGGTCCTG
ACTGGTGCAGGTGTTTCAACTTCATTAGGGATCCCGGACTTCAGATCTTCTGAGGGGTTC
TATTCAAAGATCAAACATTTGGGGCTCGATGATCCCCAAGACGTTTTCAATTACAATATA
TTTATGCACGACCCCTCTGTTTTCTATAATATTGCCAATATGGTTTTACCTCCAGAAAAA
ATTTATTCTCCATTGCATAGTTTCATTAAGATGCTACAAATGAAAGGGAAATTATTGAGA
AATTATACTCAAAACATTGATAATTTGGAATCTTATGCGGGAATAAGCACAGATAAACTG
GTGCAGTGCCATGGCTCTTTTGCTACTGCCACCTGCGTTACCTGCCATTGGAACCTACCC
GGTGAGAGGATATTTAATAAAATTAGAAACCTCGAACTTCCACTATGCCCGTACTGTTAC
AAAAAAAGAAGAGAATATTTCCCAGAGGGATATAATAATAAAGTAGGTGTTGCTGCATCA

CAGGGTTCAATGTCGGAAAGGCCTCCATATATCCTTAACTCATATGGCGTTCTCAAACCA
GATATCACATTCTTTGGCGAAGCACTGCCAAATAAATTTCATAAGAGCATTCGCGAAGAT
ATCTTAGAATGTGATTTGTTGATTTGCATTGGGACAAGTTTAAAAGTAGCGCCAGTGTCT
GAAATCGTAAACATGGTTCCTTCCCACGTTCCCCAAGTCCTGATTAATCGTGATCCCGTC
AAGCACGCAGAATTTGATTTATCTCTTTTGGGGTACTGTGATGACATTGCAGCTATGGTA
GCCCAAAAATGTGGCTGGACGATTCCGCATAAGAAATGGAACGATTTGAAGAACAAGAAC
TTTAAATGCCAAGAGAAGGATAAGGGCGTGTATGTCGTTACATCAGATGAACATCCCAAA
ACCCTC
>Worm_SIRT2
ATGTCACGTGATAGTGGCAACGATTCAGAAGTTGCGGTCACACACGGGGAAGTGCAAGAA
ATAACGGAGGAAAATCCAGAAATCGGTTCTATGCACATTACACAAGAAACAGATATTTCC
GATGCACCCGAAACAAACACAGATTCGAGTAGACAAAGAACAGAAAGTACAACCAGTGTC
AGTTCAGAGTCTTGGCAAAATAATGACGAAATGATGTCGAATCTTCGTCGTGCTCAAAGA
CTTCTTGATGATGGTGCCACTCCATTACAAATTATTCAACAAATTTTTCCTGATTTTAAT
GCATCACGAATTGCAACAATGTCAGAGAACGCGCATTTCGCCATATTAAGCGATCTTCTA
GAACGTGCTCCTGTAAGGCAGAAACTCACAAACTACAACTCACTAGCAGATGCAGTCGAA
CTATTTAAAACAAAGAAACATATTCTTGTGTTGACTGGCGCTGGAGTGTCAGTATCCTGT
GGAATTCCAGATTTTCGAAGTAAAGATGGTATTTATGCACGACTTCGGAGCGAGTTTCCC
GATCTTCCTGATCCGACAGCAATGTTCGATATTCGTTACTTCCGAGAAAATCCTGCTCCG
TTCTACAATTTTGCGAGAGAAATCTTCCCAGGACAGTTCGTACCATCTGTATCACATCGA
TTTATCAAAGAGCTTGAAACTTCTGGTCGTCTTCTTCGAAATTACACTCAAAATATCGAT
ACATTAGAGCATCAGACTGGAATTAAAAGAGTTGTGGAATGTCATGGATCCTTTTCGAAA
TGTACATGTACACGTTGTGGACAAAAATATGATGGGAATGAGATTCGTGAGGAAGTTCTT
GCTATGCGTGTTGCCCATTGCAAACGTTGCGAAGGAGTGATCAAACCGAATATTGTATTC
TTTGGAGAGGATCTCGGAAGAGAATTTCATCAACATGTTACAGAAGACAAGCATAAAGTA
GATCTCATTGTAGTTATTGGATCTTCTCTCAAAGTTCGACCAGTTGCATTAATTCCACAT
TGTGTTGATAAGAATGTTCCACAAATTCTAATCAACAGAGAATCCCTCCCACATTATAAT
GCAGATATTGAATTGCTCGGAAATTGTGATGACATCATCAGAGATATCTGTTTCAGTCTT
GGAGGCTCCTTCACTGAACTGATTACATCTTATGATTCGATTATGGAACAACAAGGAAAA
ACGAAATCACAAAAGCCTTCACAAAACAAACGACAGTTGATTTCACAGGAAGACTTTTTG
AATATTTGTATGAAAGAGAAAAGAAATGATGATTCGAGTGATGAGCCAACATTGAAGAAA
CCAAGAATGTCGGTGGCTGACGATTCGATGGATTCCGAGAAGAATAATTTCCAAGAAATA
CAAAAACATAAGTCAGAAGATGACGATGATACACGGAATTCGGACGATATTCTCAAGAAA
ATAAAGCATCCAAGGCTGCTGAGCATTACTGAGATGCTCCATGACAATAAGTGTGTCGCG
ATTTCTGCTCATCAAACCGTCTTTCCTGGAGCCGAATGCTCATTTGATCTAGAAACATTG
AAACTGGTTCGTGATGTTCATCACGAGACACATTGCGAAAGTTCGTGTGGTTCGTCTTGC
TCATCAAATGCCGATTCAGAAGCAAATCAATTATCTAGAGCACAATCGCTAGATGACTTT
GTACTATCTGACGAGGATCGAAAAAACACGATACATCTGGACTTGCAACGAGCCGATTCA
TGTGATGGAGATTTTCAGTATGAACTATCTGAAACGATTGATCCTGAAACATTCTCGCAT
TTGTGTGAAGAAATGCGTATC
>Tilapia_SIRT2
ATGTCTGATTCGTCAGAACTTCCTAAATCAGAAAAAGAGGAGGAGGTCACTCCCGAACCA
GAGGAACAATCCGACGACAGCAGTGAAGACGAGGCTGCAGGAGACACAGACATGGACTTC
CTGCGCAACCTCTTCTCCAGCGCGCTGGGCCTCGGTTCAGCTGAGAAAGTCCTAGATGAG

CTGACTCTGGATGGAGTGGCACGATACATAAACAGCGGCAAATGTAAAAACATCATCTGC
ATGGTTGGAGCAGGAATATCCACATCTGCTGGAATCCCAGATTTTCGCTCTCCAGGAACT
GGCCTGTACGCAAACCTGCAAAAATACAACTTGCCTTACCCAGAGGCTATTTTCCAGATA
GATTACTTTAAGAAACATCCAGAGCCATTCTTTGCCTTGGCAAGGGAGCTTTACCCAGGA
CAGTTTAAGCCAACAATCTGTCACTATTTCATGAAGATGTTGAAAGACAAAGGCATCCTG
AGACGCTGCTACACACAGAATATCGACACGCTGGAGCGAGTAGCCGGGCTCGAAGGAGAT
GACCTAATCGAAGCTCACGGGACGTTCTACACGTCACACTGTGTCAGCTTCTGCTGCCGG
AAGGAGTACAGCCTGGAGTGGATGAAAGAGAAAATCTTTTCTGACGACATTCCCAAATGT
GACAAGTGCAGCAGTTTGGTTAAACCAGATATTGTCTTCTTTGGAGAGAACCTACCTGCC
AGATTCTTCACTTCAATGAAGATGGATTTTCCTCGTTGCGACCTTCTCATCGTTATGGGG
ACGTCCCTTCAGGTCCAACCATTTGCGAGTCTAGTCAGCAGGGTTTCAAAAAGTTGCCCC
AGACTGCTCATTAACATGGAGAAAGCAGGGCAGGTTAATCCCATGATGGGTTTGTTTGGT
TTCGGAGAAGGGATGGACTTTGACTCAGACAAGGCTTACAGAGATGTCGCTCACATTAGT
ACATGTGATGATGGATGTTTGGCTCTAGCTGATCTTCTGGGATGGAAGGAAGAGCTGGAA
GAGCTGGTGAAGAAGGAGCACACCAAGATCGACAGTCAGGATGCAAAAGAGAAGGCCAGT
GAAAACAAAGGAGCTACAGCCAAGGCGAGCTCTGCATCAGCGCCACCAGAACCCAAAGCT
GAGGAG
>Tetradon_SIRT2
ATGTCTGATGCATCAGAATTACCTAAAAAAGAAGAGGGGGCGTTATCACCGGAATTACAG
GAACAGTCAGACGACAGCAGTGAAGACGAGGCTGCAGAAGACAGTATGTTGGACTTCCTG
CGTAATCTTTTCTCTAGAAACCTGGGCATTGGCACCCCAGACAAGGTTCTCGATGAGCTG
ACTCTGGAGGGGGTGGCGCAATATATAAAAAGTGGAAAATGTAAAAACATCATCTGCATG
GTCGGAGCAGGGATTTCCACCTCGGCTGGTATCCCTGATTTTCGCTCCCCTGGAACTGGC
CTGTATGCAAACTTGCAGAAGTATAACCTGCCTTACCCAGAGGCC
>Spotted_gar_SIRT2
ATGTCTGACCCACCAGCTCCTGACAAAAAGGACGAAGAGGCCACAATCGCAGAGCCTGAG
GAGGACCAATCAGGGGACAGCAGCGACGAGGGCGGGGCCACTGGAGACACTGAGATGGAG
TTCCTGAGGAATCTGTTTTCGCAGACTCTGGGTCTCGGTTCCGGTTCTAGGGAGAAGGTT
CTGGATGAGCTGAGCTTGGAGGGGGTGGCCCGATACATCCAGAGCGGCAACTGTAAAAAC
GTTATCTGCATGGTGGGAGCAGGAATATCGACGTCGGCTGGCATTCCAGATTTCCGCTCT
CCTGATACGGGGCTGTATGCCAACCTGCAGAAGTACAACTTGCCCTACCCTGAGGCCATC
TTTCAGATCGACTACTTCAAGAAACACCCAGAGCCTTTCTTTGCCCTGGCCAGAGAGCTG
TACCCCGGGCAGTTCAAGCCCACAGTGTGTCATTACTTCATCCGGATGCTGAAGGACAAG
GGGCTGCTGAGGCGTTGCTACTCCCAGAACATTGATACACTGGAGCGGGTGGCAGGGCTA
CAGGGAGATGACCTGGTTGAAGCCCATGGCACGTTCTACACCTCCCACTGTGTCAGCTTC
CTCTGCCGGAAAGAGTACACCCTGGACTGGATGAAAGAGAAGATCTTTGCAGATGAGATC
CCAAAGTGTGACAAGTGCAGCAGCCTTGTGAAGCCTGACATTGTGTTCTTCGGGGAGAAC
CTCCCAGCTCGATTCTTCACTTCGTTGCAGATGGATTTCCCTCGCTGTGATCTCCTCATC
ATCATGGGCACCTCCCTGCAGGTCCAGCCATTTGCTTCACTAGTCGGCAAGGTCTCTGCT
AGAACCCCACGGCTTCTGATAAACAAAGAGAAGACAGGACAGTCAGACTCTCTGATGGGA
TTCCTGGGCTTTGGTGGAGGAATGGACTTTGATTCAGAGAAAGCATACAGGGATGTGGCG
TATCTGGGGACCTGCGATGATGGCTGTCTTGCTCTCGCGGACCTGCTGGGCTGGAAGACC
GAGTTGGAGGAGCTGGTGAAGCAGGAGCACACTTTAATTGACAGCAAGGACAAGCGGGAG
AAGGCCAGCGCAGCCAATCAGAACTCTGGGGCGGGGCAAAAGGCAGGCCTGGGGGTGGAG

CCCAGGGAGAGTGACAAACCTGAGCAGCCAATGGGAGAAGGCAGCTCAAAGCAGGAC
>Pig_SIRT2
ATGGCCGAGCCGGATCCCTCAGACCCTGTGGAGACCCAGACAGGGAAGGTGCAAGAGGCT
CAGGACTCCGATTCCGATTCGGACACTGAGGAAGGAGCAGCTGGTGGAGAAGCCGAGATG
GACTTCCTGCGGAATTTCTTCTCCCAGACCCTGGGCCTGGGCACCCAGAAGGAGCGTCTG
CTGGACGAGCTCACCCTGGAAGGGGTGTCCCGCTACATGCAGAGTGAGCGCTGTCGCAGG
GTCATCTGTTTGGTGGGAGCTGGAATCTCCACGTCCGCGGGCATCCCTGACTTCCGCTCC
CCGTCCACGGGCCTCTATGCCAACCTGGAGAAGTACCGTCTGCCCTACCCGGAGGCCATC
TTTGAGATTGGCTACTTCAAGAAACATCCAGAGCCCTTCTTCGCCCTCGCCAAGGAACTC
TATCCTGGGCAGTTCAAGCCGACCATCTGTCACTACTTCATCCGCCTGCTGAAGGAGAAA
GGGCTGCTCCTGCGCTGCTACACGCAGAACATAGACACCCTGGAGCGAGTGGCGGGACTA
GAGCCCGAGGACCTGGTGGAGGCCCATGGCACCTTCTACACGTCACACTGCATCAGCCCC
CTCTGCCGGCAGGAGTACACGCTAGGCTGGATGAAAGAGAAGATCTTCTCCGAGGTGACC
CCCAAGTGTGAGAAATGTCAGAGCGTGGTGAAGCCTGATATCGTGTTCTTCGGTGAGAAC
CTCCCAGCGCGTTTCTTCTCCTGCATGCAGTCAGACTTCCTGAAGGTGGACCTTCTCATC
GTCATGGGCACCTCCCTGCAGGTGCAGCCCTTCGCATCCCTCATCAGCAAGGCGCCCCTC
TCCACCCCACGCCTGCTCATCAACAAGGAGAAGACCGGCCAGACTGACCCGTTCCTCGGG
ATGATGATGGGCCTCGGAGGAGGCATGGACTTCGATTCAAAAAAGGCGTACAGGGACGTG
GCCTGGCTGGGTGACTGCGACCAGGGCTGCTTGGCCCTCGCTGATCTCCTCGGATGGAAG
AAGGAGCTGGAGGACCTTGTCCGGAAAGAGCATGCCAGCATAGATGCCCAGTCGGGATCG
GGCACCCCCAATCCCACCACTTCAGCTTCCCCCAGGAAGTCTCCGCCTCCCGCCAAGGCA
GAGGCCAGGACCTCGGAGGGAGAGAAACCCCAG
>Panda_SIRT2
ATGGCCGAGCCGGACCCCTCTGACCCTCTGGAGACCCAGGCAGGGAAGGTGCAGGAGGCC
CAGGACTCAGATTCAGACACTGAGGAAGGAGCGGCTGGGGGGGGGGGGGGGGAAGCAGAG
ATGGACTTCCTGCGGAATTTCTTCTCCCAGACACTGGGCCTGGGCACCCAGAAGGAGCGT
CTGCTGGACGAACTGACCCTGGAAGGAGTGACCCGCTATATGCAGAGCGAGCGCTGTCGC
AGGGTCATCTGTTTGGTGGGAGCTGGGATCTCCACGTCTGCCGGCATCCCTGACTTCCGC
TCCCCATCCACGGGCCTCTACGCCAACCTAGAGAAGTACCATCTTCCCTACCCGGAGGCC
ATCTTTGAGATTGGTTACTTCAAGAAACACCCAGAGCCCTTCTTTGCTCTGGCCAAGGAA
CTCTATCCTGGGCAGTTCAAGCCGACGGTCTGTCACTACTTCATCCGCCTGCTGAAGGAG
AAGGGACTGCTGCTGCGCTGCTACACGCAGAACATAGACACGCTGGAGCGAGTGGCAGGG
CTGGAGAGCGAGGACCTGGTGGAGGCCCACGGCACCTTCCACACCTCGCACTGCACCAGT
CCCCTCTGCCGACGGGAGTATACGCTCAGCTGGATGAAAGAGAAGATCTTCTCCGAGGTG
ACTCCCAAGTGTGAGAAATGCCACAGCGTGGTGAAGCCTGACATCGTGTTCTTCGGTGAG
AACCTCCCAGCGAGGTTCTTCTCCTGCATGCAGTCAGACTTCCTGAAGGTGGATCTCCTC
ATCATCATGGGCACCTCCCTGCAGGTGCAGCCCTTCGCCTCCCTCATCAGCAAGGCACCC
CTCTCCACCCCGCGTCTGCTCATCAACAAGGAGAAGACTGGGCAGACTGACCCTTTCCTG
GGGATGATGATGGGCCTCGGAGGAGGCATGGACTTCGACTCCAAGAAGGCCTACCGGGAC
GTGGCCTGGCTGGGAGACTGTGACCAGGGCTGCTTGGCCCTTGCTGACCTCCTCGGCTGG
AAGAAGGAGCTGGAGGACCTTGTCCGGAAGGAGCATGCCAACATAGATGCCCAGGCAGGG
TCAGAGGGCCCCAACCCCAACACTTCAGCTTCCCCCAGGAAGTCTCCACCTCCTGCCAAG
GAAGAGGCCAGGAGCACGGAGGGAGAGAAGCCCCAG
>Western_painted_turtle_SIRT2

ATGGCCGAGCGGGACGCGGCGGGTGCCGGAGAAATTGAGGCAGAGAGGGGCTCAGACTCG
CCTGACTCCGATTCTGACTCCGAAAACGGTGGCGCCTCTGGAGAGTCTGAAATGGATTTC
CTGCGACACCTCCTGTCCCGGACGCTGGGCCTGGGCAGCGAGAAGCCGGAGAAGGTGCTG
GATGAGCTGACGTTGGACGGAGTGAGCCGCTTCATGCAAAGCGAGAAGTGCAGGAACGTC
GTCTGCATAGTGGGCGCTGGGATCTCAACCTCCGCGGGGATCCCGGACTTCCGGTCGCCC
GGCACGGGGCTCTACGCCAATCTGCAGAGCTACAACCTGCCGTACCCTGAAGCCATCTTC
GAAATCAACTACTTCAAGCAACACCCGGAGCCGTTCTTTGCCCTTGCCAGGGAGCTGTAT
CCAGGACAGTTTAAGCCCACCGTGTGCCACTACTTCATCCGGCTGCTGAAGGAGAAGGGT
TTGCTGCTGCGCTGTTACACGCAGAATATTGACACGTTGGAGCGGGTGGCCGGACTGGAC
CAGGAAGATCTGGTGGAAGCCCACGGCACCTTCTTCACCTCTCACTGTCTCGGCTCCTCC
TGCAAGAAACAATACACCCTGGACTGGATGAAAGAAAAGATTTTCTCGACTGTCACTCCC
AAGTGCGACAAATGTCAGAGTCTGGTGAAGCCGGATATCGTGTTCTTTGGGGAGAGCCTG
CCCCCCCGCTTCTTCACACTCATGCAGTCGGATTTCCAGAAGGTGGACCTGCTCCTCATC
ATGGGCACATCGCTGCAGGTGCAGCCCTTCGCCTCCCTCGTCAGCAGGGTGCCCACAAAC
ACCCCGAGGCTGCTGATTAACAAGGAGAAGACGGGGCAGAGTGATCCCTTCATGTCCCTG
ATGGGCCTCAGCACCGGCATGGACTTCGACTCGGAAAAGGCCTACAGGGATGTGGCATGG
CTCGGGGAGTGCGACGAAGGCTGCACCGCGCTGGCCGAGCTGCTGGGATGGAAGAACGAG
CTGGAGGAGCTGGTGAAGAAGGAGCACTCTGCCATCGATGCCAAGTCGGGGCAGGCAGGC
GAGGCCGGGAGAAGCCCTTCCCCTCCCCCGGCTGGGGCCACGGCAAGCCCCTCCCCTCCC
CCAGCGAAGCCGGGGTCATCGCCCCAGAAGGAAAGCGACGCCGGAAGCAAAGCGGAA
>Orangutan_SIRT2
ATGGACTTCCTGCGGAACTTATTCTCCCAGACGCTCAGCCTGGGCAGCCAGAAGGAGCGT
CTGCTGGACGAGCTGACCTTGGAAGGGGTGGCCCGGTACATGCAGAGCGAACGCTGTCGC
AGAGTCATCTGTTTGGTGGGAGCTGGAATCTCCACATCCGCAGGCATCCCCGACTTTCGC
TCTCCATCCACCGGCCTCTATGACAACCTAGAGAAGTACCATCTTCCCTACCCAGAGGCC
ATCTTTGAGATCAGCTATTTCAAGAAACATCCGGAACCCTTCTTCGCCCTCGCCAAGGAA
CTCTATCCTGGGCAGTTCAAGCCAACCATCTGTCACTACTTCATGCGCCTGCTGAAGGAC
AAGGGGCTACTCCTGCGCTGCTACACGCAGAACATAGATACCCTGGAGCGAATAGCCGGG
CTGGAACAGGAGGACTTGGTGGAGGCGCACGGCACCTTCTACACATCACACTGCGTCAGC
GCCAGCTGCCGGCACGAATACCCGCTAAGCTGGATGAAAGAGAAGATCTTCTCTGAGGTG
ACGCCCAAGTGTGAAGACTGTCAGAGCCTGGTGAAGCCTGATATCGTCTTTTTTGGTGAG
AGCCTCCCAGCGCGTTTCTTCTCCTGTATGCAGTCAGACTTCCTGAAGGTGGACCTCCTC
CTGGTCATGGGTACCTCCTTGCAGGTGCAGCCCTTTGCCTCCCTCATCAGCAAGGCACCC
CTCTCCACCCCTCGCCTGCTCATCAACAAGGAGAAAGCTGGCCAGTCGGACCCTTTCCTG
GGGATGATTATGGGCCTCGGAGGAGGCATGGACTTTGACTCCAAGAAGGCCTACAGGGAC
GTGGCCTGGCTGGGTGAATGCGACCAGGGCTGCCTGGCCCTTGCTGAGCTCCTTGGATGG
AAGAAGGAGCTGGAGGACCTTGTCCGGAGGGAGCACGCCAGCATAGATGCCCAGTCGGGG
GCGGGGGTCCCCAACCCCAGCACTTCAGCTTCCCCCAAGAAGTCCCCGCCACCTGCCAAG
GACGAGGCCAGGACAACAGAGAGGGAGAAACCCCAG
>Opossum_SIRT2
ATGGCCGAGGCGGACCCCCCCGGTCCCCCCCAGGACGAGGCAGGGAAGGTGCTGGATGCT
CAGGACTCGGATTCAGACATTGAGAGTGGAGCCTCATCTGGAGAGACTGAGATGGATTTT
TTAAGAAATCTCTTCTCCCGGACCCTGGGCCTGGGCAGCGAGAAGCGGGAAAAGCTGCTG
GATGAACTTTCCTTGGAAGGGGTGACAAGGTTCATCCAGAGTGATCGATGTCAGAACATC

ATCTGCATGGTGGGGGCCGGAATCTCTACATCTGCAGGCATCCCTGACTTTCGATCCCCG
ACCACCGGCCTGTATTCCAACTTGGAGAAGTACAACCTTCCATATCCTGAAGCTATCTTT
GAAATCAACTACTTTAAGAAACACCCTGAGCCTTTCTTCGCCCTGGCCCGGGAGCTGTAC
CCAGGGCAATTTAAGCCCACCGTGTGTCATTACTTCATTCGTCTGCTCAAGGAGAAGGGG
CTGCTGCTTCGATGCTATACCCAGGTGAGGACATCTAGGGTCGGGAGGTGGGGAGGAGGA
GATGCCCACACACTGGGCTTTGGGCAGTCAGGCCTA
>Naked_mole_rat_SIRT2
ATGGACTTCCTGAGGAGTCTCTTCTCTCAGACCCTGGGCCTGGGCTCCCAGAAGGAGCGT
CTGCTGGATGAGCTAACTCTGGAAGGGGTGGTCCGCTACATGCAGAGCGAGCGCTGTCAG
AGGGTCATCTGCTTGGTGGGAGCTGGAATCTCCACATCTGCGGGCATCCCCGATTTCCGC
TCTCCAACCACTGGCCTCTATGCCAACCTGGAGAAGTACCATCTTCCCTACCCCGAGGCC
ATCTTTGAGATTGGCTATTTCAAGAAACATCCGGAACCCTTCTTTGCCCTTGCCAAGGAA
CTGTATCCTGGGCAGTTCAAGCCGACCATCTGTCACTACTTCATCCGCCTGCTGAAGGAG
AAGGGGCTGCTCCTGCGCTGCTACACACAGAACATTGACACCCTGGAGCGTGTGGCCGGC
CTGGAGCCCGAGGACCTGGTGGAGGCCCACGGCACCTTCTACACATCACACTGCACCAGC
CAGCTCTGCCGGCACGAGTACACACTGGGCTGGATGAAAGAGAAGCTCTTCTCGGAGGTG
ACCCCCAAGTGCGAGAAATGTCAGAGCGTGGTGAAGCCTGACATCATCTTTTTCGGCGAG
AACCTCCCGTCTCGGTTCTTCTCCTGCTTGCAGTCAGACTTCCGGAAGGTGGACCTGCTC
ATCATCATGGGCACCTCGCTGCAGGTGCAGCCCTTCGCCTCTCTCATTGGCAAAGCACCC
CTGTCCACCCCTCGCCTGCTCATCAACAAGGAGAAGACTGGCCAGACTGACCCCTTCCTG
GGAATGATGATGGGCCTGGGAGGAGGCATGGACTTTGACTCCAAGAAGGCCTACAGGGAC
GTGGCCTGGCTGGGTGACTGTGACCAGGGCTGCCTGGCCCTCGCCGACCTCCTTGGATGG
AAGAAGGAGCTCGAGGACTTGGTCCGGAAGGAGCACGCCCACATAGATGCCCAGTCGGGG
TCCAGCACTCCCAACCCTACCACTGCAGCTTCCCCCAGGACATCCCCACCTCCTGGCAAG
GCAGAGGCCAAGACCACAGATGGGGAGAAGCCCCAG
>Medaka_SIRT2
ATGTCTAATGCTGCAGATCTTCCAAAAGCAGAGGAGGAGGAGGAGGTCACCCCGGATTTA
CAGGATCTGTCGGACGACAGCAGTGACGACGAGGCGGCAGCAGGAGCTGAGATGGACTTC
CTGCGGAACCTCTTCTCCAGCACTTTGGGCCTCGGCTCTGCAGAGAAGGTTCTGGATGCT
CTGACTCTGGAAGGAGTGGCTCAGTATATAAAGAGCGGCAAATGTAAAAACATCATCTGC
ATGGTTGGTGCAGGAATCTCCACATCTGCTGGAATTCCAGATTTCCGTTCTCCAGAAACC
GGCCTGTATGCAAACCTGCAGAAATACAACCTGCCTTACCCAGAAGCCATCTTCCAGATA
GATTACTTTAAGAAACATCCAGAACCGTTCTTTACTTTGGCGAAGGAGCTCTACCCAGGA
CAGTTTAAGCCAACCATCTGTCACTACTTCATAAAGTTGCTGAAGAACAAAGGCCTCCTG
AGACGCTGCTACACTCAGAACATCGACACTCTGGAGCGAGTTGCAGGTCTTGAAAAAGAA
GATCTGATTGAAGCTCATGGGACCTTCTACACCTCCCACTGTGTCAGCTTCTGCTGCCGT
AAAGAGTACAGCCTGGACTGGATGAAAGAGAAAATCTTCTCTGATGCCATTCCCAAATGT
GAGAAGTGCAACAGTTTGGTGAAACCAGGTCAGGTCCTGATGTTTGATCCTGCTGTTTGG
TCGGTCGCTCTCTGCAGGGATGTGGCTCACATCAGTTCCTGTGATGACGGCTGCCTGGCT
CTGGCTGACCTGCTGGGCTGGAAGGCCGAGCTGGAGGCTCTGGTGAAGCAGGAGCACGCT
CAGATCGACAGTAAAGACAAGAAGGAGCAGCCTGGAGAAGCTGGAGGAGCGGCGGCCAAA
GCCAGCTCGTCCTCTGTGAAGCCAGAGGAG
>Macaque_SIRT2
ATGGCAGAGCCGGACCCCTCTCACCCTCTGGAGACCCAGGCAGGGAAGGTGCAGGAGGCT

CAGGACTCAGATTCAGACTCTGAGGGAGGAGCCGCTGGTGGAGAAGCAGACATGGACTTC
CTGCGGAACTTATTCTCCCAGACGCTCAGCCTGGGCAGCCAGAAGGAGCGTCTGCTGGAC
GAACTGACCTTGGAAGGGGTGGCCCGGTACATGCAGAGCGAACGCTGTCGCAGAGTCATC
TGTTTGGTGGGTGCTGGAATCTCCACATCTGCGGGCATTCCCGACTTTCGTTCTCCATCC
ACTGGCCTCTATGACAACCTAGAGAAGTACCATCTTCCCTACCCAGAGGCCATCTTCGAG
ATCAGCTATTTCAAGAAACATCCGGAACCCTTCTTCGCCCTCGCCAAGGAGCTCTATCCT
GGGCAGTTCAAGCCGACCATCTGTCACTACTTTATGCGCCTGCTGAAGGACAAGGGGCTG
CTCCTGCGCTGCTACACGCAGAATATAGATACCCTGGAGCGAATAGCGGGGCTGGAACAA
GAGGACTTGGTGGAGGCCCACGGCACCTTCTACACGTCGCACTGCGTCAGTGCCAGCTGC
CGGCACGAATACCCGCTAAGCTGGATGAAAGAGAAGATCTTCTCTGAGGTGACGCCCAAG
TGTGAGGACTGTCAGAGCCTGGTGAAGCCTGATATCGTCTTTTTTGGTGAGAGCCTCCCA
GCGCGTTTCTTCTCCTGTATGCAGTCAGACTTCCTGAAGGTGGACCTCCTCCTCGTCATG
GGCACCTCCTTACAGGTGCAGCCCTTTGCCTCCCTCATCAGCAAGGCACCCCTCTCCACC
CCTCGCCTACTCATCAACAAGGAGAAAGCTGGCCAGTCGGACCCTTTCCTGGGGATGATC
CTGGGCCTCGGAGGAGGCATGGACTTTGACTCCAAGAAGGCCTACAGGGACGTGGCCTGG
CTGGGTGACTGCGACCAGGGCTGCCTGGCCCTTGCTGAGCTCCTCGGATGGAAGAAGGAG
CTGGAGGACCTTGTCCGGAGGGAGCACGCCAGCATAGATGCCCAGTCGGGGGCGGAGGCC
CCTAACCCCAGCACTTCAGCTTCCCCCAGGAAGTCCCCGCCACCTGCCAAGGACGAGGCC
AGGACAACAGAGAGGGAGAAACCACAG
>Human_SIRT2
ATGGCAGAGCCAGACCCCTCTCACCCTCTGGAGACCCAGGCAGGGAAGGTGCAGGAGGCT
CAGGACTCAGATTCAGACTCTGAGGGAGGAGCCGCTGGTGGAGAAGCAGACATGGACTTC
CTGCGGAACTTATTCTCCCAGACGCTCAGCCTGGGCAGCCAGAAGGAGCGTCTGCTGGAC
GAGCTGACCTTGGAAGGGGTGGCCCGGTACATGCAGAGCGAACGCTGTCGCAGAGTCATC
TGTTTGGTGGGAGCTGGAATCTCCACATCCGCAGGCATCCCCGACTTTCGCTCTCCATCC
ACCGGCCTCTATGACAACCTAGAGAAGTACCATCTTCCCTACCCAGAGGCCATCTTTGAG
ATCAGCTATTTCAAGAAACATCCGGAACCCTTCTTCGCCCTCGCCAAGGAACTCTATCCT
GGGCAGTTCAAGCCAACCATCTGTCACTACTTCATGCGCCTGCTGAAGGACAAGGGGCTA
CTCCTGCGCTGCTACACGCAGAACATAGATACCCTGGAGCGAATAGCCGGGCTGGAACAG
GAGGACTTGGTGGAGGCGCACGGCACCTTCTACACATCACACTGCGTCAGCGCCAGCTGC
CGGCACGAATACCCGCTAAGCTGGATGAAAGAGAAGATCTTCTCTGAGGTGACGCCCAAG
TGTGAAGACTGTCAGAGCCTGGTGAAGCCTGATATCGTCTTTTTTGGTGAGAGCCTCCCA
GCGCGTTTCTTCTCCTGTATGCAGTCAGACTTCCTGAAGGTGGACCTCCTCCTGGTCATG
GGTACCTCCTTGCAGGTGCAGCCCTTTGCCTCCCTCATCAGCAAGGCACCCCTCTCCACC
CCTCGCCTGCTCATCAACAAGGAGAAAGCTGGCCAGTCGGACCCTTTCCTGGGGATGATT
ATGGGCCTCGGAGGAGGCATGGACTTTGACTCCAAGAAGGCCTACAGGGACGTGGCCTGG
CTGGGTGAATGCGACCAGGGCTGCCTGGCCCTTGCTGAGCTCCTTGGATGGAAGAAGGAG
CTGGAGGACCTTGTCCGGAGGGAGCACGCCAGCATAGATGCCCAGTCGGGGGCGGGGGTC
CCCAACCCCAGCACTTCAGCTTCCCCCAAGAAGTCCCCGCCACCTGCCAAGGACGAGGCC
AGGACAACAGAGAGGGAGAAACCCCAG
>Horse_SIRT2
ATGGCCGAGCCGGACCCCTCTGACCCTCTGGAGACCCAGGCAGGGAAGGTGCAGGAGGCT
CAGGACTCAGATTCAGACACTGAGGGAGGAGCGGCTGGCGGAGAAGCAGAAATGGACTTC
CTGCGGAACTTCTTCTCCCAGACGCTGGGCCTGGGCACCCAGAAGGAGCGTCTGCTGGAC

GACCTCACCCTGGAAGGGGTGGCCCGCTATATGCAGAGCGAGCGCTGTCGCAGGGTCATC
TGTTTGGTGGGAGCTGGAATCTCCACGTCCGCGGGCATCCCTGACTTCCGCTCCCCATCC
ACCGGCCTCTACGCCAACCTGGAGAAGTACCACCTTCCTTACCCGGAGGCCATCTTTGAG
ATTGGCTACTTCAAGAAACATCCAGAGCCCTTCTTTGCCCTCGCCAAGGAGCTCTATCCT
GGGCAGTTCAAGCCCACCGTCTGTCACTACTTCCTGCGCCTGCTGAAGGAGAAGGGGCTG
CTCCTGCGCTGCTACACGCAGAACATAGACACCCTGGAGCGCGTGGCGGGGCTGGAGCCT
GAGGACCTGGTGGAGGCCCACGGCACCTTCTACACGTCGCACTGCATCAGCCCCGTCTGC
CGGCGGGAGTACACGCTGGGCTGGATGAAAGAGAAGATCTTCTCCCAGGTGACTCCCAGG
TGCGAAAAGTGTCAGAGCGTGGTGAAGCCTGACATCGTGTTCTTCGGGGAGAGCCTCCCA
GCGCGGTTCTTCTCCTGCATGCAGTCAGACTTCCTGAAAGTGGACCTCCTCATCATCATG
GGCACCTCCCTGCAGGTGCAGCCCTTTGCATCCCTCATCAGCAAGGCGCCCCTGTCCACC
CCGCGCCTGCTCATCAACAAGGAGAAAGCTGGCCAGACGGACCCCTTCCTGGGAATGATG
ATGGGTCTCGGCGGAGGCATGGACTTTGACTCCAAGAAGGCCTACAGGGACGTGGCCTGG
CTGGGGGACTGCGACCAGGGCTGCCTGGCCTTGCTGACCTCCTCGGATGGAAGG
>Anole_lizard_SIRT2
ATGGAGAGTCGGGAAGACCCCGGAGCTGCCGCCACCTCCTCCGCCAGTAACGAAGCCGAT
GCGGCCGCAGAGAGGGAAGCAGCGTCCGGCTCCTCGGGTTCCGATTCTGATGACGAAGGT
GGGGCTTCTGGATTGACCGAAATGGAGTTTTTGCGAAATCTCTTGTCCCGGACATTAAAC
TTGGGCAGCGAGAAGCCTGAAAAAGTGCTGGATGAGCTGACCTTGGAGGGAGTGAGCAGA
TACATGCTGACTGAAAAATGCAAAAATGTGGTGTTTATGGTTGGAGCAGGAATCTCGACT
GCTGCTGGGATTCCAGATTTCCGCTCCCCAGGGACAGGGCTCTATGCCAATCTGCAGCAG
TACAACCTGCCATACCCTGAAGCCATCTTTGAGATCAACTACTTCAAGAAACACCCGGAA
CCATTTTTTGCTTTGGCCCGGGAGCTATACCCAGGACAGTTCAAGCCCACAGTTTGTCAC
TACTTCATGCGCCTCCTAAAAGAGAAAGGGTTGCTGCTCCGTTGCTACACACAGAACATT
GATACCTTGGAGCGGGTAGCTGGTCTGGATCATGAAGATTTGGTAGAAGCTCATGGCACC
TTCTTTACCTCGCACTGCATCAGCCCCACCTGTAAGAAGATGTACAGCTTGGAGTGGATG
AAAGAAAAGATCTTTTCCTCTCTCATCCCCAAATGTGAAAAATGTCAGAGTGTGGTGAAG
CCAGATATCGTGTTCTTTGGGGAAAATTTGCCCTCTCGGTTCTTCTCCCTGATGCAGTCA
GATTTCCAGAATGTGGACTTGCTTATTATCATGGGCACTTCCCTTCAGGTCCAGCCCTTT
GCCTCACTTGTTGCCAGAGTCCCCACAAACACCCCACGGCTTCTTATCAATAAAGAAAAG
ACAGGGGAGAGTGACCCTTTTATGTCCTTGATGGGCTTTGGCTGTGGGATGGACTTTGAC
TCAGAAAAGGCATACAGGGATGTTGCATGGCTTGGAGATTGTGATGAAGGCTGCTATGCT
TTGGCTGAGTTTTTGGGATGGAAGAAGGAGCTTGAAGACCTGGTGAAAAGTGAGCATGCC
ACCATTGATGCCAAGTCAGGGCAGGCGGTTGGTGATGGGGCGAGCGCCTCCCGCCCTCAC
AAGAAGGACCACCAGCAGAAACCGTCCCCAAAGAGAGAAAGGCCTCCTCACCCCCAAGAT
GAA
>Fugu_SIRT2
ATGTCTGATGCATCAGAAATACCTAAAAAAGAAGAGGGGGCTTTAGCACCTGAGCCAGAG
GAAGAGTCCGACGACAGCGGTGAGGACGAGGCTACAGAAGACATGGAGTTCCTGCGTAAT
CTTTTCTCGAGAAACCTGGGCATTGGCACCCCAGACAAGGTTCTCGATGAGCTGACTCTG
GAGGGAGTAGCACAGTATATAAAGAGTGGAAAATGTAAAAACATCGTCTGCATGGTTGGA
GCAGGGATATCCACCTCGGCTGGGATCCCTGATTTTCGCTCACCTGGAACTGGCCTGTAT
GCAAACCTGCAGAAGTATAACCTGCCGTACCCAGAGGCGATCTTCCAGATTGATTACTTC
AAGAAACATCCAGAGCCTTTCTTTGCTTTGGCCAGAGAGCTTTACCCAGGACAGTTTAAG

CCCACAGTTTGTCACTACTTCATGAAGCTGCTGAAGGATAAAGGACTCCTGAGGCGGTGC
TACTCGCAGAATATTGACACTCTGGAGCGTGTGGCTGGCCTCGAGGGGGACGACCTCATT
GAAGCTCATGGAACGTTCTACACATCCCACTGTGTCAGTTTCTGCTGCCGCAAGGAGTAC
TCGCTGGGCTGGATGAAAGAAAAAATCTTTTCCGACGACGTTCCCCGGTGTGAGAAATGC
AGCAGCTTGGTGAAACCCGATATCGTCTTCTTTGGGGAGAATCTTCCTCTTCGTTTCTTC
ACTTCTGTTAAGTCTGACTTCCCTCGTTGTGACCTCCTCATCATCATGGGGACGTCCCTG
CAGGTCCAACCCTTTGCCAGTCTTGTCAGCAGGGTTTCCAAAAGTTGCCCCAGACTGCTC
ATAAACATGGAAAAGGCTGGTCAGGCTAATGCTCTGTTTGGGATTCTCGGGTTTGGCGGA
GGGATGGACTTTGACTCAGACAAGGCATACAGAGATGTCGCTCACATCAGTACCTGTGAC
GATGGCTGTTTGGCTTTGGCTGACCTATTGGGGTGGAAGGCAGAGCTGGAGGATTTGGTG
AAGCAGGAACACGCCAGGATTGACAGCCAGGACCAAAGCAGTGAGAGCAAAGGGGCTCCG
GCCAAGGCGAGCTCCGCCTCCGCGGCGCCGCAGCACAAACGGGGGCAGGAG
>Great_tit_SIRT6
ATGGCGGTGAATTACGCGGCCGGGCTGTCCCCGTACTCGGACAAGGGCAAGTGCGGCCTC
CCCGAGATTTTCGACCCTCCGGAGGAGCTGGAGCGGAAGGTGCAGGAGCTGGCAGACCTG
ATCCGGAGCTCCTCCAATGTGGTGTTTCACACAGGAGCAGGGATCAGCACGGCCTCGGGG
ATCCCTGACTTCAGAGGTCCCAATGGTGTCTGGACTATGGAAGAGAAAGGGCTCTCCCCA
AAATTCGACACCACCTTTGAGAACGCCAGGCCCTCCAAGACTCACATGGCGCTGCTGGGG
CTGCAGAGAGTGGGAATCCTGAAATTCCTGGTCAGCCAGAACGTGGACGGCCTGCACGTG
CGGTCAGGATTCCCACGGGACAAGTTGGCCGAGCTCCACGGGAACATGTTTGTGGAAGAG
TGCGTGAAATGCGGGAAGCAGTACGTGCGCGACGCCGTCGTGGGCAGCATGGGGCTCAAG
CCCACGGGGCGCCTCTGCAGCGTCACCAAGGCCCGGGGGCTGCGGGCCTGCAGAGGGAAG
TTACGAGACACTATTCTGGATTGGGAAGATTCCCTGCCCGACCGTGACCTGACACTGGCA
GATGAAGCCTGCAGGAAAGCCGATCTCTCCGTCACCCTGGGGACCTCTCTGCAGATCAAA
CCCAGCGGGAACCTCCCACTGATCACCAAGAAGAGAGGAGGAAAGTTGGTCATTGTCAAC
CTCCAAGCAACCAAACACGTGGCCTCTCTTAGCAGGCAAACAGGCTGGGAGTCCTGGGTT
GGAGGGTTTGAACCTGCAACTCCTGCTGAACACGCAGAGATTGCAGGACCGCCAGGCCGA
CCTGCGCATCCACGGCTACGTGGACGAGGTGATGACCAAGCTGATGAAGCACCTGGGGCT
GGAGGTGCCCGAGTGGACGGGGCCGGTGGTGGTGGAGAGCGCCGAGCTGGCCAAGGCCGA
GCAGCTGCAGGGGCGGCTGAAGGAGGAGCCGCTGTCCCAGCACAACGGCACGGGAGCGCC
GTGTCCCGGGAACGCGCCGCTGGAGCGCCGCGACGGGCTCAAGCTGGAGTGTCCCAGCCC
GGACACGGGGCCGACACCGGTGAAGAAGATGAAGGTGGAGCCTCTCCTCACCTGACCTGG
ACTGTCCCTGTCTCACCTGGGCTGCTTTTCCTACCAACTTTTTTTTATACCCTTAAATAT
TGTCCCTTTTTTTATGTCAGTATTAAACACACT
>African_ostrich_SIRT6
ATGGCCACCCTCTTCTTCCGCTGTGGCTCGCTGGCCCCGAGCCCTGTCGAGCCCGCGGCT
CTGCTGGGGAACGGTGCCGGGGATCAGTGCACCTGGGCTGGCCCCGGGGTCGCTCCCTTC
CTCCTGCTGCTCCACTGTGCCCTTGATCCAAGATGCTTCTGTGGGTATCAGATTTTTGAT
CCTCCGGAGGAGCTGGAGAGGAAAGTGCGTGAACTGGCAGATTTGATAAGGAGTTCCTCG
AACGTGGTGTTTCACACAGGGGCTGGAATCAGTACTGCCTCAGGGATTCCCGACTTCAGG
GGGCCAAATGGTGTCTGGACTATGGAAGAGAAGGGGCTCTCCCCAAAATTTGACACCACC
TTTGAGAATGCCAGGCCCTCCAAGACTCACATGGCACTGCTGGGGCTGCAGAGAGTCGGC
ATCCTCAAGTTCCTGGTCAGCCAGAACGTGGACGGCCTTCACGTCCGTTCGGGATTCCCA
CGGGACAAGTTGGCCGAGCTGCACGGGAACATGTTTGTGGAAGAGTGCGTGAAATGTGGC

AAGTACGTTGAGTCCCAAGCCCGGGGAGGGCGTCAGCGCCGTGGGGGCTTCGCTGCTCTC
CCCGGCGAAGGCTGGAGGCCCTGCACAGGGAAGTTAAGAGACACCATTTTGGACTGGGAA
GATTCCCTGCCCGACCGCGACCTCACGCTGGCGGATGAAGCCTGCAGGAAAGCTGATCTC
TCTGTCACGCTGGGGACCTCTCTGCAGATCAAACCCAGCGGCAACCTCCCGCTGATCACG
AAGAAGAGGGGAGGGAAGCTGGTCATAGTCAACCTACAGGCAACCAAGCACGACAGACAG
GCTGACCTGCGCATCCACGCCTACGTTGATGACGTCATGACAAAGCTGATGAAGCACCTG
GGGCTGGAGGTGCCGGAGTGGACGGGGCCGGTGGTGGTAGAGAGTGCCGAGCTCATCAAG
CCTGAACAGCTCTTCAAATTTGACCTTGGGGCTCGCCGACCACTCAAAGAAGACCCCATC
TCCCAGCACAATGGCACTACTGGGGTGCACTCTGACCTTGGGACCACGCTCACAGAGCAC
CGTGACAGTCTGAAGCAGGACTGTCCCAGCCCGGACACGGGGCCAACAACGGCAAAGAAG
ATGAAGGTGGAGTCTCTCCTCACC
>Adelie_penguin_SIRT6
ATGAAATCCTGTGCCCGGCGCCAGAAAGCCGGCTGGGCACACGGTCCCATCCAGCCACGG
CGTCCCCGCGTCCCCCACCTCCGTGGCTTTCCGTGCAGCTCGCCGTCCCTCGGTCCTGTC
CCGTCCGCAGAGCTGGTGGCTGTGCTGGTGACAGCTGGAGTTGAGCACAGGTGGGGCGGC
ACCGGGTTCACCCCTGTCCTCCCGCTGCCCGGCGGTGTCCTGGAGCCCAGCCACTTCTCG
GGGTATCAGCTGCCTATTTTTGACCCGCCGGAGGAACTGGAGAGGAAGGTGCGGGAGCTG
GCGGATTTGATCAGGAGTTCCTCCAATGTGGTGTTTCACACGGGGGCTGGAATCAGCACC
GCCTCGGGGATTCCCGACTTCAGGGGGCCCAATGGTGTCTGGACTATGGAAGAGAAGGGG
CTCTCCCCAAAATTTGACACCACCTTTGAGAATGCCAGGCCCTCCAAGACTCACATGGCG
CTGCTGGGGCTGCAGAGAGTCGGCATCCTGAAATTCCTGGTCAGCCAGAACGTGGACGGC
CTTCACGTGCGTTCAGGATTCCCACGGTACTGCCTCTCCCGCCCTGTCTCCGGGTCCCTC
ACGCTTTCTCCAGCCGCTGCCACGTGGCTGCAGTACGTGCGGGACGCAGTCGTGGGCAGC
ATGGGGCTGAAGCCAACGGGCAGGCTGTGCAGCGTCACCAAAGCCCGGGGGCTGCGGGCC
TGCAGAGGGAAGTTAAGAGACACTATTCTGGACTGGGAAGATTCCCTGCCCGACCGCGAC
CTCACGCTGGCGGACGAAGCCTGCAGGAAAGCTGATCTCTCCGTCACCCTGGGGACCTCC
CTGCAGATCAAACCCAGTGGCAACCTCCCGCTGATCACGAAGAAGAGAGGAGGGAAGCTG
GTCATAGTCAACCTACAAGCAACCAAACACGACAGACAGGCCGACCTGCGCATCCACGCC
TACGTCGATGATGTCATGACGAAGCTGATGAAGCACTTGGGGCTGGAGGTCCCGGAGTGG
ACGGGGCCGGTGGTGGTGGAAAGCGCTGAGCTCACCAAGCCCGAACAGCTCTTCAAATTT
GACCCCGGGGCTCGCGGGCTGCTGAAGGAGGAGCCTCTCTCGCGGCACAACGGCACCGGC
GGGCTGTGCCCTGACCTTGGGACCACGCTGGTGGAGCGCCGTGACAGTCTGAGGCAGGAG
GGTCCCAGCCCGGACACGGGGCCAACGACGGTGAAGAAGATGAAGGGGGAGCCTCTCCTC
ACC
>common_starling_SIRT6
ATGGCGGTGAATTACGCGGCCGGGCTGTCGCCGTACTCGGACAAGGGCAAGTGCGGCCTC
CCCGAGATTTTTGACCCTCCGGAGGAGCTGGAGCGGAAGGTGCAGGAGCTGGCAGAGCTG
ATCCGGAGCTCCTCCAATGTGGTGTTTCACACGGGGGCAGGGATCAGCACGGCCTCGGGC
ATCCCTGACTTCAGGGGCCCCAATGGTGTCTGGACCATGGAAGAGAAAGGGCTCTCCCCA
AAATTCGACACCACCTTTGAGAACGCCAGGCCCTCCAAGACTCACATGGCGCTGCTGGGG
CTGCAGAGAGTGGGAATCCTGAAATTCCTGGTCAGCCAGAACGTGGACGGGCTGCACGTG
CGGTCAGGATTCCCCCGGGACAAGTTGGCCGAGCTCCACGGGAACATGTTTGTGGAAGAG
TGCGTGAAATGTGGGAAGCAGTACGTGCGTGACGCCGTCGTGGGCAGCATGGGGCTCAAG
CCCACAGGACGCCTCTGCAGCGTCACCAAGGCCCGGGGGCTGCGGGCCTGCAGGGGGAAG

TTACGAGACACTATTCTGGATTGGGAGGATTCCCTGCCTGACCGTGACCTGACGCTGGCA
GATGAAGCCTGCAGGAAAGCCGATCTCTCTGTCACCCTGGGGACCTCTCTGCAGATCAAA
CCCAGCGGGAACCTCCCACTCATCACCAAGAAGAGAGGAGGGAAATTGGTCATTGTCAAT
CTCCAGGCAACCAAACACGACCGCCAGGCCGACCTGCGCATCCATGGCTACGTGGATGAG
GTGATGACCAAGCTGATGAAGCACCTGGGGCTGGAGGTGCCCGAGTGGACGGGGCCAGTG
GTGGTGGAGAGAGCCGAGGTGGCCAAGGCCGAGCTCAGCCAGGCCGAGGAGCTGCAGGGG
AGGCTGAAGGAGGAGCCCCTGGCCCAGCACAATGGCACCACAGCTCTGTGTCCAGGGAAG
GCACCACGGGAGCGCCGGGATGGGCTCAAGCTGGAGTGTCCCAGCCCAGACATGGGGCCA
ACACCGGTGAAGAAGATGAAGGTGGAGCCTCTCCTCACC
>central_bearded_dragon_SIRT6
ATGTCGGTGAATTACGCGGCCGGGCTGTCCCCTTACTCGGACAAAGGGAAATGCGGCCTT
CCTGAGTTCTTGGACCCTCCCGAGGAGCTGGAGGAGAAAACCCGCCGGCTTGCGGACATG
ATCCGAGAAGCGGCCAACATCGTGTTTCACACGGGGGCTGGCATCAGTACGGCGTCGGGG
ATCCCGGACTTCAGAGGCCCAAACGGCGTCTGGACCATGGAAGAACGGGGCTTGGCGCCC
AAGTTTGACACCACTTTTGAGAATGCTCGGCCGTCCAAAACCCACATGGCCCTTCTGGGG
CTGCAGAGAGCAGGCATCCTGCAGTTCCTGGTCAGTCAGAACGTGGACGGTCTCCATGTG
CGATCCGGCTTCCCCAGAGACAAGCTGGCGGAGTTGCACGGGAACATGTTTGTGGAGGAA
TGCATGAAATGTGGCAAGCAGTATGTGAGGGACACTGTCGTAGGCACAATGGGGTTGAAG
CCGACAGGGCGGCTGTGTGACGCTTCCAAACGCCGAGGGCTGCGCTCCTGCAGGGGAAAG
CTCATGGACACCATCTTAGACTGGGAAGATTCTCTGCCAGATCGGGATCTCAGCCTGGCA
AGTGAAGCCAGCAGGAAAGCAGACCTTTCTGTCACGTTGGGAACCTCCCTTCAGATCAAG
CCCAGTGGAGATCTTCCTTTGCTGACGAAGAAGAAAGGGGGAAAGCTTGTGATAGTGAAT
TTGCAGCCCACGAAACACGACAAGCACGCAGATCTGCGCATCCATGGTTATGTCGACGAC
ATCATGGCAAAGCTCATGAAGCACCTTGGGGTGGAGATCCCTGAGTGGGCAAAGCCTGTG
GTGGTGGAGAAGGCCGAGCCCCTCGACCTCAAGCCTTTGGCAAAACCCGGCACGAACTTT
CAGCTCCCGAGCAAGGAGGAGCCCATCCCGCACTGTAACGGCACAGGGGGGAGCACAGAT
CTCGGGAGGTCTCCGAAGCATGGATCCAGCCATTCGGACACAAACTCCGGAATCGCCAAA
CGGCTAAAGCGGGAATCGCCACTGACC
>Gecko_SIRT6
ATGGCCAAGCATCGTGAGCAGGGGCTCCGGCTGGTTGGCTGCCCCTCGTCCTCTTCCACT
GTCCTGCTTCTGCTGACGCGAGTCAGTTCTTCTTGCCTGACGGGCAGCGAATGCCTTCGG
GAGTCTTTCAGGGCTTGCCAGCAAGCAGTCTTTAAGTCAGTGACTCTCTCTGGTGGACCC
ATCACGGTGCACGGCCCCACGCCAGACGCAGCTCCGGCTCACGTCCTTTCCGGCTCTGCC
ACGTCGTGCCGACACGCCACCACGCCTCCGGCTGTTGGCAACCACAGTCCCAGCTTGTCC
TTCGATGTCGCTGCGGACCCGCGACAATGCGTTGCTCCCCCGGGCCGGCCGGGGCAGGAG
GAGAGCGTAGCGCCTCAGCTGACCGCTAGAGGGCAGGGCCGCGCTGCGGTCCGGCTGCCC
CCGGCTAGGCTGCCTGGAGCCGGCCCTGCGGTGTTGCGTAACGGCGCCTGGCCGGCTTCC
CAGCCCGCGGAGCCCGAGAGGGTGGGCCCCCTCTCCCGGGAAGAGAAGGGAGGCCGAGAG
AGGATTGATGGCCGTCAGGCGGAGGAAAGCTGTTGTTTGGAGCCACCACCCAAGCCCCGG
GGCGGTGCCAAAGGCGCCTCGTCCTCGCCGCGGGCGGGGGGCTTGTTTACTGCGCCGCCG
GCCAGCGTCGCCGCGTTTCTTTTCCCGTGTCTCTTTTGTGCTTTTGACAGACAAGCATTT
GACAGAGGCCCCAACGGCGTTTGGACCATGGAGGAGAGAGGCTTGGCCCCCAAATTCGAC
ACCACCTTCGAGAACGCGCAGCCTTCCAAGACCCACATGGCCCTCCTGGAGCTACAGAGG
AAGGGCATCCTGCGATTCCTAGTCAGCCAAAATGTGGACGGACTTCACGTGCGCTCCGGG

TTCCCGAGAGATAAGCTGGCAGAGTTACATGGGAACATGTTCGTGGAGGAATGTATGAAA
TGTGGCAAGCAGTACATACGAGACACCGTGGTGGGGACGATGGGTCTGAAGCCGACGGGT
CGGCTATGTGACGTTTCCAAACGCAGAGGGCTTCGGTCCTGCAGAGGAAAACTAATGGAC
ACCATTTTGGACTGGGAAGACTCTCTACCTGATCGGGATCTCAGTCTGGCTAGCGAGGCC
TGCAGAAAAGCCGACTTGTCCGTCACCTTGGGAACATCCCTGCAGATCAAACCCAGCGGC
GACCTCCCTCTGCTGACGAAGAAGAAAGGGGGCAAGCTGGTGATCGTCAACCTGCAGCCA
ACCAAGCACGACAAGCACGCTGACCTGCGGATTCATGGTTATGTGGACGAGGTCATGACG
AAGCTGATGAAGCACCTGGAACTGGAGATTCCTGAGTGGACGGGGCCACGTGTGGTAGAG
GAGAGCGCTGAGATCCCAGAGTCCAAACCGCGGGCAAAACCAAGCACAGACTTTAAGTTC
CTGAGTAAAGAGGAGCCCCTTTCATACTGCAACGGCACAGAGGAGAGCCCCAAGCCTGCA
CGCCAGGAGCACAGTGCTTCTCTGAAGCATGAATCCTGCCCACTGGACAGCAACCCGAAC
GCAGCCAAACGCACTAAAACGGAACCACCGCCCACT
>Taiwan_habu_SIRT6
ATGTCGGTCAATTACGCGGCGGGGTTGTCGCCCTACCCGGACAAGGGGAAGTGCGGCCTC
CCGGAGATTTTTGATTCGCCGGACGAGTTGGAGAGGAAGATCCACCAGTTGGCTGACTTG
ATCCAGAATTCTTTCAGTGTGGTGTTTCACACCGGGGCGGGAATCAGCACAGCCTCAGGG
ATTCCGGATTTCAGGGGCCCGAATGGGGTGTGGACCATGGAGGAGCGGGGCCTGGTCCCC
AGATTCGACACGACCTTTGAGAACGCGAGGCCATCGAAGACGCACATGGCCCTGCTGGAG
CTGCACCGCGTGGGCATCCTGCACTTCCTGGTCAGCCAAAACATCGACGGGCTTCACGTG
AGGTCCGGCTTCCCCAGGGACAAGCTGGCAGAACTGCATGGGAACATGTTCGTAGAGGAA
TGCGTGAAATGTGGCAAGCAGTACGTGAGGGACACCGTGGTGGGCACCATGGGGCTGAAG
CCCACTGGCCGGCTCTGCGACGCCTCCAAGCACCGAGGGCTCCGATCCTGCAGAGGCAAG
TTAATGGACACCATCCTAGACTGGGAAGATTCCCTGCCGGACCGTGACCTGAATTGGGCC
TCTGAAGCCAGCAGGAGGGCGTCCTTATCCATCACCCTGGGGACCTCCCTGCAGATCAAG
CCCAGCGGGGACCTTCCGCTGCTGACCAAGAAGAAGGGCGGGAAGCTGGTGATCGTCAAC
CTGCAGCCCACAAAGCACGACAGGCACGCGGACCTGCGCATCCACGGCTACGTGGACGAG
GTGATGACGAAGCTGATGAAACTCCTCGGGCTGGAGATCCCCGAGTGGGCTGGGCCGCTG
GTGGTGGAGCGTGCAGAACAGCGAGAGGCCAAGCCGCTCTCCCAAACCGGCGCAGACTTG
CCCTTCCTGAGCAAGGGAGAGCCGGCTCCCTGCTGGAACGGTGGCCGGGAGGGCCTGGGA
CACACGGCGGTCCTGAAGCAGGAATCCCGCCTGCTGGACGGCGGCTCCGTCCCGGCCAAG
CAGCCGAAGCTGGAGCCGTTGCCCACT
>green_sea_turtle_SIRT6
ATGGAGGAGAGGGGATTGTCCCCCAAGTTTGACACTACCTTTGAGAATGCAAGGCCTTCC
AAGACCCATATGGCACTGCTGGAGCTGCAGAGAGTCGGCATCTTGAAGTTCCTGGTCAGC
CAAAACGTGGATGGCCTGCATGTGCGTTCTGGATTCCCACGGGACAAGCTGGCCGAGCTG
CACGGGAACATGTTTGTGGAAGAATGTGTGAAATGCAGCAAGCAGTACGTGCGGGATGCT
GTCGTGGGCACCATGGGCCTCAAGCCGACCGGCAGGCTGTGCGACGTCTCCAAAGCAAGA
GGGCTTCGTGCCTGCCGAGGAAAGTTAATAGACACTGTTTTAGACTGGGAAGATTCTCTG
CCTGATCGGGACCTGAGTCTAGCAGACGACGCCTGCAGGAAAGCTGACCTGTCAGTCACT
CTGGGGACCTCTCTTCAGATCAAACCCAGTGGCAATCTCCCCCTGCTCACGAAAAGGAAA
GGAGGGAAGCTGGTCATAGTAAATCTGCAAGCAACGAAGCATGACCGGCAAGCCGACCTG
CGCATCCATGGCTACGTGGATGAAGTCATGACAAGGCTGATGAAGCAGCTTGGTCTGGAG
ATCCCCGAGTGGACGGGGCCGATGGTGGTGGAGAGTTCTGAGCTTGTGAAGCCCGAACCA
GCCCTAAAACTCGATCCGGATGCTCAGCGCCCAGCCAAGGAAGAGCCCTGCTCCCACCAC

AATGGCACGGCAGAGGGGGCTGATGGGACATGCCCGGGGCTCGGCACGGCACGCGAGGAG
CACGGTGACCGTCTGAAACAGGAAAGTCCTGGCTCAGGCCGCAGCCCCGTAGCAGCCAAG
AGAGTGAGAGTGGAGTCGCTGCTCACC
>Chinese_alligator_SIRT6
ATGGGAAGCGACTGCCTCAAGGTCACTTGGATGATCTTTGATCCTCCGGAGGAGCTGGAG
AGGAAAGTGCGTGAGCTGGCCGACCTGATCAGGAGCTCTTCCAACGTGGTGTTTCACACC
GGGGCTGGGATCAGCACAGCCGCAGGGATCCCCGACTTCAGGGGGCCCAATGGCGTCTGG
ACCATGGAGGAAAAGGGGTTGTCCCCCAAGTTCGACACCACCTTTGAGAATGCCAAGCCC
TCCAAGACACACATGGCACTGCTGGAGCTGCAGAGAGCTGGCATCCTGAAGTTCCTGGTC
AGCCAGAACGTGGACGGGCTGCATGTCCGCTCTGGATTCCCACGGGACAAGCTGTCCGAG
CTGCACGGGAACATGTTTGTGGAGGAATGTGTGAAATGCGGCAAGCAGTACGTGCGGGAC
ACCGTGGTGGGGAGCATGGGCCTCAAGTCGACAGGCAGGCTGTGCAACGTCACTAAAGCG
CGTGGTCTCCGTGCCTGCAGGGGCAAGCTCATAGACACTATTCTGGACTGGGAGGATTCG
CTACCTGGCCGGGACCTCAGCCTGGCCGATGAAGCCTGCAGGAAAGCAGACCTGTCGATC
ACTCTGGGAACCTCTCTCCAGATCAAACCCAGTGGCAATCTCCCCCTGATCACGAAAAAG
AGAGGTGGGAAGCTGGTGATTGTCAATCTCCAAGCAACCAAACACGACAGACAAGCCGAC
CTGCGCATCCATGGCTATGTGGATGAAGTCATGATGAAGCTGATGAAGCACCTGGGTCTG
GAGATCCCTGAGTGGAGAGGGCCAGTGGTAGTGGAGAGGGCAGAGCTGGTGAAGCCCGAA
GAGCCCCTCAAGCCCGACCCTGATGCTCTTTGTCCAGCCAAAGCTGAGTCCCCTTCCCAC
CACAACGGCATCATGGAGGGGGCCAGCGGGACCTGCCCTGGCCTGGGGCTCGTGCCCAAG
TCTCGCTGTGACAGTGTGAAACAGGAATGTCCCAGCCCTGTAGCAGCCAAGAGGAGGAAA
GCAGAGTCACTGCTCACC
>Gharial_SIRT6
ATGTCGGTGAATTACGCGGCCGGGCTGTCCCCCTACTCGGACAAGGGCAAGTGTTCTTTC
CTTCAGATCTTTGATCCTCCGGAGGAGCTGGAGAGGAAAGTGCATGAGCTAGCCGACCTG
ATAAGGTGTTCTTCCAACGTGGTGTTTCACACGGGGGCTGGGATCAGCACAGCCGCAGGG
ATCCCCGACTTCAGGGGGCCCAATGGCGTCTGGACCATGGAGGAAAAGGGGTTGTCCCCC
AAGTTTGACACCACCTTTGAGAATGCCAAGCCCTCCAAGACCCACATGGCGCTGCTGGAG
CTGCAGAGAGCCGGCATCCTGAAGTTCCTGGTCAGCCAGAATGTGGACGGTCTGCACGTC
CGCTCTGGATTCCCACGGGACAAGCTGGCCGAGCTGCACGGGAACATGTTTGTGGAGGAA
TGTGTGAAATGCGGCAAGCAGTACGTGCGGGACACCGTGGTGGGGAGCATGGGCCTCAAG
TCGACAGGCAGGCTGTGCAACGTCACTAAAGCGCGTGGTCTCCGTGCCTGTAGGGGCAAG
CTCATAGACACTATTCTGGACTGGGAAGATTCGCTACCTGACCGGGACCTCAGCCTGGCC
GATGAAGCCTGCAGGAAAGCGGACCTGTCGATCACTCTGGGAACCTCTCTTCAGATCAAA
CCCAGTGGCAATCTCCCCCTGATCACGAAAAAGAGAGGTGGGAAGCTGGTGATTGTCAAT
CTCCAAGCAACTAAACACGACAGACAAGCCGACCTGCGCATCCATGGTTATGTGGATGAA
GTCATGATGAAGCTGATGAAGCACCTGGGTCTGGAGATCCCTGAGTGGAGAGGGCCAGTG
GTGGTGGAGAGGGCAGAGCTGGTGAAGCCCGAAGAGCCCCTCAAGCCTGACCCTGATGCT
CTTTGTCCAGCCAAGGCTGAATCCCCTTCCCACCACAACGGCATCATGGAGGGGGCCAGC
GGGACCTGCCCTGGCCTGGGGCTCGTGCCCAAGTCTCGCTGTGACAGTGTGAAACAGGAA
TGTCCCAGCCCTGTAGCAGCCAAGAGAAGAAAAGCAGAATCACTGCTCACC
>African_clawed_frog_SIRT6
ATGTCGGTGAACTATGCGGCCGGTTTGTCTCCGTATGCCGATAAAGGTCGCTGCGGGCTC
CCGGAGCAATTTGATTCCCCGGACGAATTGCGTCAGAAAGTAGAGGAACTAGCTGAAATG

ATACGTGAATCCTCCTACGTTGTTTTTCACACGGGAGCCGGGATCAGTACTTCCTGCGGA
ATCCCAGACTTCAGGGGGCCTAATGGAGTGTGGACTCTGGAGGAGAAAGGGCTGGACCCA
AAGTTTGACACCACCTTTGAAACCGCATGTCCTTCCCCTACTCACATGGCGCTGCTGAAG
CTGCAGAGGGTTGGGATCCTGAAGTTTCTGGTTAGTCAAAATGTAGATGGTCTGCACGTA
AGATCCGGCTTCCCCCGGGAGCAGCTGGCGGAACTGCATGGGAACATGTTCGTGGAGGAA
TGTAGCAAATGTGCTAAACAGTATGTGCGGGATCAAGTGGTGGGAACCATGGGGCTCAAA
CCTACCGGCAGGTTTTGTGATGTGCCAAAGGTGCGAGGCCTGCGAGCTTGCAGGGGGAAA
CTGAAAGACACCATCCTGGATTGGGAAGACTCCCTGCCTGACAGAGACCTAAACCTGGCT
GATGAAGCCTGCAGGAAAGCAGACCTGTCCATTACTTTGGGGACCTCACTCCAGATCCGA
CCCAGTGGGAACCTTCCGCTCCTCACCAAGCGCAAGGGAGGGAAACTAGTCATTGTAAAC
CTTCAGCCCACCAAGCATGACAAACATGCGGATTTGAGAATACATGGTTATGTGGATGAG
GTGATGATACAGCTCATGGAGCTTCTGGGTCACAAGATACCAGTTTGGACTGGGATACAT
ACTAAAACAGAGCCAACTTATGAGAACCATAAGGAGGAAGATAACTCCTACCATAATTCA
GTGCTGGATGCTAACCTGGACCAAAAGAGGGAAGGGTGCAAGGAAGAATCTAATCTTGAG
CTAAAGAAGGCAAAAGTTGAACCAGCATGTGTA
>Tibetan_frog_SIRT6
ATGTCGGTGAATTATGCAGCTGGTCTGTCCCAATATCCTGATAAAGGGAAATGCGGACTT
CCTGAGGTTTTTGATCCACCGCGAGAATTGCATCGGAAGGTGCAGAAGCTTGCTGAAATG
ATCCGAAAAGCCTCTTATGTGGTATTTCATACTGGAGCAGGAATCAGCACTTCTTGTGGG
ATCCCTGATTTTAGGGGCCCTAAAGGTGTGTGGACATTGGAGGAGAAAGGTCTAAATCCA
AATTTTGATACAACATTTGAAAATGCTCGTCCGTCTATAACGCATATGTCACTTCTACAA
CTGCAGAGAGTTGGTATCTTGAAGTTTTTGATCAGCCAAAATGTGGATGGTTTGCATTTA
AGGTCTGGATTTCCCAGGGAAGAGCTTGCAGAGCTCCACGGCAACATGTTTGTTGAGGAG
TGCACCAAATGTGGCAAGCAGTATGTTCGAGACTGTGTGGTGGGAACCATGGGATTAAAA
CCTACTGGAAGACTTTGTGATGTTTCCAAAGTTAGAGGTCTACGGTCATGCAGAGGAAAG
CTGATCGATACTATTCTGGATTGGGAGGATTCTTTGCCTGATAGAGACTTGAACTTAGCT
GATGAAGCCTGCAGAAAAGCAGATTTGGCGATAACACTTGGAACATCACTTCAAATTAAA
CCTAGTGGGAATCTTCCTCTTGTAACCAAGCGTAAAGGTGGCAAGTTAGTCATAGTAAAC
CTTCAGCCAACCAAACATGACCGGCATGCTGACTTGTGCATCCATGGTTATGTCGATGAG
GTTATGACACAGCTCATGCAACATCTAGATATTAAAATCCCCATATGGACTGGCATATCT
GTTAAAATAGAATCAAGCGAGCACAATCTTAAATCCGAAGTGGTGTCAAACTGTAATGGA
GACCTGGGTTCTTTAGAGTGCCAAAAGAGCAAGAGGGAATGTTGTGTTGAGGAGGTTTCC
AGTCTGCCGAAGCGAGCAAAAGTTGAAACGCCTTGTGTA
>southern_platyfish_SIRT6
ATGTCAGTGAATTATGCTGCTGGACTCTCTCCGTACGCAGATAAAGGTGTCTGCGGACTG
CCTGAGAGTTTCGACAGTTCTGAGGAACTAAAGGCAAAAGTGGAGACCCTTGCTCAGCTG
ATTAAAGAATCGGAGTACTTGGTCGTCCACTCTGGAGCTGGAATTAGCACCTCATCAGGC
ATTCCTGACTTCAGAGGTCCCAAGGGTGTGTGGACGCTAGAAGAAAAGGGTGAGTCACCT
CACTTTAATACCACATTTGAAGATGCCCGCCCCAGTTTGACTCACCTGGCCCTCCTGGGA
CTGCAAAGGGCAGGATACCTGAAATACCTCATCAGCCAGAATGTCGACGGGCTGCATGTT
CGATCAGGCTTCCCCAGGGATAAACTATCAGAGCTTCATGGAAACATGTTTGTGGAGGAA
TGCGAGAAGTGTGGCAGGCAGTACGTGAGGGAAAAGGTGATCGGTGTGATGGGGCTGAAA
CCGACAGGACGTTACTGCGAGGTGGTGCGGTCCAGAGGGCTGAGGGCCTGCAGAGGAAAG
CTGATCAGCACCATACTGGATTGGGAGGATGCTCTGCCTGACAGAGACCTGAACAGGGCA

GATGATGCAAGCAGACGAGCAGACCTGGCACTGACTCTGGGCACGTCCATGCAGATCAAA
CCCAGTGGGGACCTCCCACTCCTCACAAAGCGCAAAGGAGGGAAAGTGGTTATAGTCAAC
TTGCAACCAACCAAACATGACAAACACGCCCACCTGCGTATCAACGGTTATGTGGACGAT
GTCATGAAGCAGCTGATGGAGCTGCTGGATTTAGAAATCCCAAAATGGGAGGGACCCGTC
ATCTGCGAGAGCTCCACTTTCTCACCCGAGACCACCGCTGATGTCAAGCCTCCTCGCGGC
ATGTCTGCAAAGGACAAAGTGAAAAAGGACAAAATCAAGGAGGAAAGGAAAAGGGAAGCA
ACGCAGCCAACGGAGGACGCCGGCGTGAAAGACGAGACGGTTTCAGTAAAGCGAGAGCGA
GCAGATTTTTCCGCGGAGACAGACGAAAAGGAA
>American_alligator_SIRT6
ATGTCGGTGAATTACGCGGCCGGGCTGTCCCCCTACTCGGACAAGGGCAAGTGCGGCCTC
CCCGAGATCTTTGATCCTCCGGAGGAGCTGGAGAGGAAAGTGCATGAGCTGGCCGACCTG
ATAAGGAGTTCTTCCAACGTGGTGTTTCACACTGGGGCCGGGATCAGCACAGCCGTAGGG
ATCCCCGACTTCAGGGGGCCCAATGGCGTCTGGACCATGGAGGAAAAGGGGTTGTCCCCC
AAGTTCGACACCACCTTTGAGAATGCCAGGCCCTCCAAGACCCACATGGCACTGCTGGAG
CTGCAGAGAGTCGGCATCCTGAAGTTCCTGGTCAGCCAGAACGTGGACGGGCTGCACGTC
CGCTCTGGATTCCCACGGGACAAGCTGTCCGAGCTGCACGGGAACATGTTTGTGGAGGAA
TGTGTGAAATGCGGCAAGCAGTACGTGCGGGACACCGTGGTGGGGAGCATGGGCCTCAAG
TCAACAGGCAGGCTGTGCAACGTCACTAAAGCACGTGGTCTCCGTGCCTGCAGGGGCAAG
CTCATAGACACTATTCTGGACTGGGAGGATTCGCTACCTGGCCGGGACCTCAGCCTGGCC
GATGAAGCCTGCAGGAAAGCAGACCTGTCGATCACTCTGGGAACCTCTCTCCAGATCAAA
CCCAGTGGCAATCTCCCCCTGATCACGAAAAAGAGAGGTGGGAAGCTGGTGATTGTCAAT
CTCCAAGCAACCAAACACGACAGACAAGCCGACCTGCGCATCCATGGCTATGTGGATGAA
GTCATGATGAAGCTGATGAAGCACCTGGGTCTGGAGATCCCTGAGTGGAGAGGGCCAGTG
GTGGTGGAGAGGGCAGAGCTGGTGAAGCCCGACGAGCCCCTCAAGCCTGACCCTGATGCT
CTTTGTCCAGCCAAAGCTGAGTCCTCTTCCCATCACAACGGCATCATGGAGGGGGTCAGC
GGGACCTGCCCTGGCCTGGGGCTCGTGCCCAAGTCTCACTGTGACAGTGTGAAACAGGAA
TGTCCCAGCCCTGTAGCAGCCAAGAGGAGGAAAGCAGAGTCGCTGCTCACC
>Armadillo_SIRT6
ATGCATTTAAGGAGTGCCCAGGTGGCAGGACTCCCTCCCGAAGCCCCACCCTTCCCTGCT
GCTTTCAGTCATCTTGTTCCGCCAGCATTTATTGAGTGCCTGCTGTGTGCCAGGGACTTT
ATCTTTGACCCCCCGGAGGAGCTGGAGCAGAAGGTGCGGGAGCTGGCGCAGCTGGTGTGG
CGGGCGTCATACGTGGTTTTCCACACGGGGGCTGGCATCAGCACCGCCTCGGGCATCCCC
GACTTCAGGGGCCCGCACGGCGTGTGGACCATGGAGGAGCGCGGCCTGGCGCCCAAGTTT
GACACCACGTTCGAGAACGCGCGGCCCACGCGGACGCACATGGCGCTGGTGCAGCTGGAG
CGCGTGGGCCTGCTGCACTTCCTGGTCAGCCAGAACGTGGACGGGCTGCACGTGCGCTCT
GGCTTCCCCAGGGACAAGCTGGCAGAGCTCCATGGAAACATGTTCATAGAAGAATGTGTC
AAGTGTAAGACCCAGTACGTCCGGGACACCGTCGTGGGCAGCATGGGACTCAAGCCCACG
GGACGTCTCTGCACCGTCGCCAAGGCCCGGGGCCTGCGGGCCTGCAGGGGCGAGCTGAGG
GACACCATCCTGGACTGGGAGGACGCTCTGCCCGCCCGGGACCTCGCCCTCGCCGACGAG
GCCAGCAGGTGGGGTGCCCCAGCCCCCGCCCAGCCCCCACCCTGCCGCGCCCAGGGTGGG
GGGGCCAGGGATGCCAGCCTGGACATGCTCGGCGGGCGGGCCTCAGTTTCCCCGTCC
>Chicken_SIRT6
ATGGCGGTGAATTACGCGGCCGGGCTCTCGCCCTACTCGGATAAGGGCAAGTGCGGCCTC
CCCGAGATTTTCGATCCACCTGAAGAGCTGGAGAGGAAGGTGTGTGAGCTGGCAGACTTG

ATAAGGAGCTCTTCCAATGTGGTGTTCCATACAGGGGCTGGCATCAGCACCGCCTCGGGG
ATTCCTGACTTCAGGGGGCCTAATGGTGTCTGGACTATGGAAGAGAAGGGGCTTTCCCCC
AAATTTGACACCACCTTTGAGAACGCCAGGCCCTCCAAGACTCACATGGCACTTCTGGGG
CTGCAGAGAGTTGGCATCCTGAAATTCCTGGTCAGCCAGAACGTGGATGGCCTTCATGTG
CGCTCAGGATTCCCACGGGACAAGTTGGCTGAGCTCCACGGGAACATGTTTGTGGAGGAG
TGCATGAAATGTGGCAAGCAGTACGTGCGGGATGCTGTTGTGGGCAGCATGGGGTTGAAG
CCAACGGGCAGGCTGTGCAGCGTCACCAAAGCACGAGGGCTACGGGCCTGCAGAGGGAAG
CTAAGAGACACTATTCTGGACTGGGAAGATTCCCTGCCTGACCGTGACCTCACACTAGCA
GATGAAGCCTGCAGGAAAGCTGATCTCTCTGTTACACTGGGAACCTCTCTGCAGATCAAA
CCCAGTGGCAACCTCCCACTGATCACAAAGAAGAGAGGAGGGAAGCTGGTCATAGTCAAC
CTACAAGCAACCAAGCACGACAGACAGGCCGACCTGCGCATTCATGCTTACGTCGATGAT
GTCATGACAAAACTGTTGAAGCACCTGGGGCTGGAAGTCCCAGAGTGGACAGGGCCAGTG
GTGGTGGAAAGTGCTGACTCTGCCAAGCCTGAACAGCTCTACACATTTAAGCCCGAGGCT
CACGGGTTGCTCAAGGAGGAGCCCTTTTCCCAGCACAATGGAACAGCTGGGCAGTGCCCC
GACCTTGGGACCACGCTGGTGGAGCACCGTGACAGTCTGAAGCAGGAGTGTCCCAGTCCA
GACACAGGACCACCACTGACAAAGAAGATGAAGGTAGAGCCTCTCCTCACC
>Chinese_softshell_turtle_SIRT6
ATGGCGCCGGGCGGCGCGCCCCTCCGAGTTCTGCGCATGCGGACTGAGGGGCGGCGGCTT
CAGTCGCTTTGTGGCTTCGGTGAAACTGCTTATCCACCATTCCGCGTGCTGGGCTGGCGG
GACGCTTATCCCTGCTTCTCCATTTCAATTTTTCCTTCCCCTCAGATTTTTGACCCGCCA
GAGGAGCTGGAGAGGAAAGTGTGTGCCTTAGCAGACTTGATCAGGGGCTCTTCCAATGTA
GTATTTCACACAGGAGCCGGGATCAGCACAGCCTCAGGAATCCCTGATTTCAGGGGGCCC
AATGGCGTATGGACTATGGAAGAGAGGGGATTGTCCCCCAAGTTTGACACTACCTTTGAG
AATGCGAGGCCCACCAAGACACACATGGCGCTGCTGGAGCTGCAGAGAGTCGGCATCTTG
AAGTTCCTGGTCAGCCAGAACGTGGATGGCCTGCATGTGCGTTCCGGATTCCCACGGGAC
AAGCTGGCCGAGCTGCACGGGAACATGTTTGTGGAGGAATGTGTGAAATGCGGCAAGCAG
TATGTGCGGGACACCATTGTGGGCAGCATGGGCCTCAAGCCAACAGGCAGGCTGTGTGAT
GTCTCCAAAGCTAGAGGGCTTCGTGCCTGCCGAGGAAAGTTAATCGACACTATTTTGGAC
TGGGAAGATTCCCTGCCTGATCGGGACCTCAGCTTGGCAGATGAAGCCTGCAGGAAAGCC
GACCTGTCAGTCACGCTGGGGACCTCTCTTCAGATCAAACCGAGTGGCAACCTCCCCCTG
CTCACGAAAAGGAAAGGAGGGAAGCTGGTGATCGTGAATCTGCAAGCAACGAAGCACGAC
AGGCAAGCCGACCTGCGCATCCATGGCTACGTGGACGAAGTCATGACAAAGCTGATGAAG
CAGCTCGGTCTGGAGATCCCCGAGTGGACGGGGCCGGCGGTGGTGGAGAGTTCTGCGCTT
GTGAAGCCCGAACCACCCCTAAAACTCGACCCAGAGGCTCCGCGCCCGGCCAAGGAAGAG
CCCTGCTCCCACCACAACGGCACGACGGAGGAGGCCGACGGGGCTTGCCCGGAGCGCGGG
CCCTCACGCAAGGAGCACTGGGACAGTCTGAAACAGGAGTGTCCTGGCTCAGACCGCGGT
CCCGTAGCAGCCAAGAGGGTGAAAGTGGAGTCGCTGCTCTCT
>Cod_SIRT6
ATGTCTGTGAACTACGCGGCAGGGCTCTCGCCTTACGCGGACAAAGGTGTCTGCGGACTA
CCCGAGAAGAAATTTGACAGTCCGGAAGAGCTGACAGGCAAGGTTAAAATCCTGACAGAG
ATGATAAAGAAGTGCGAGTTCCTCGTAGTGCACTCTGGAGCAGGCATCAGCACCGCCTCG
GGTATCCCAGACTTCAGAGGGCCAAAGGGCGTGTGGACCATGGAGGAGAAGGGCGAGACG
CCTAAGTTCGATACCACGTTCGAGGACGCGCGGCCCAGCCTGACGCACATGGCCCTCCTT
GGCCTCTACAAGGCCGGCATCCTTAAGTACCTCGTCAGCCAGAACGTGGACGGGCTGCAC

GTGAGATCCGGCTTCCCCAGGGATTCCTTGTCGGAGCTTCACGGAAACATGTTTGTTGAA
GACTGTGAGAAATGTGGCAGGCAGTATGTGAGGGAGAAGGTGATCGGTGTGATGGGCCTG
AAGCCGACCGGGCGGTACTGTGACGTGGTCCGCTCCAGGGGTCTCCGTGCCTGCCGGGGG
AAGCTGATCAGCACCATACTGGACTGGGAAGAGGCCCTTCCCATCAAAGACCTGACCAGA
GCTGAGGCCGCCAGCAGACAAGCGGATCTGGCCTTGACGCTGGGCACCTCCCTGCAGATC
AAGCCCAGCGGAGACCTGCCACTCCTCACCAAGAAGAAGGGCGGCCAGCTGGCGGTGGTC
AACCTGCAGGCCACCAAACACGATAAGCACGCTAACCTCCGTATCCATGGCTACGTCGAC
GAGGTCATGAAACAGCTGATGGAGGCCCTGGGATTGGACATTCCGAAGTGGGAGGGGCCG
ACGGTCTGCGAGAGCTTCGGTGCCAAACCCGAACCACCAGGCCATCTTGCAGCACCAGAG
GTGGAGGAAGAGGTGAAGAAGGAGGGGAAGAAGAAGGGGCAGAGGAAGCGACCCCCGGCG
CCGCCTACGAACGGGGAGGTAGACGAAGAGGCCGCTTTGGTGAAGAAGGAGAGAGCCGAA
TCTCCA
>Cow_SIRT6
ATGCAGACAAGGGCAAGTGCGGTCTACCCGAGGTCTTCGACCCCCCCGAGGAGTTGGAGC
AGAAGGTGTGGGAGCTGGCACAGCTCATCTGGCAGTCCTCCAGTGTGGTGTTCCACACAG
GCGCAGGCATCAGCACTGCCTCAGGCATCCCCGACTTCAGGGGCCCCCATGGCGTCTGGA
CGATGGAGGAGCGGGGCCTGGCCCCCACGTTCGACACCACCTTCGAGAACGCCCAGCCCA
CAAAGACCCACATGGCGCTGGTCCAGCTGGAGCGTGTGGGCCTCCTGCACTTCCTGGTCA
GCCAGAACGTGGACGGACTGCACAGACAAGCTGGCAGAGCTCCACGGAAACATGTTTATA
GAAGAATGTGTCAAGTGTAAGATGCAGTATGTCCGGGACACCGTGGTGGGCAGCATGGGC
CTGAAACCCACGGGCCGGCTCTGCACCGTGGCCAAGTCCAGGGGGCTGCGGGCCTGCAGG
GGGGAGCTGAGAGATACCATCCTGGATTGGGAGGATTCCCTGCCTGACCGGGACCTCACC
TTGGCCGACGAAGCCAGCAGGAATGCAGACCTGTCCATCACACTGGGCACCTCCCTGCAA
ATCCGGCCCAGCGGGAACCTTCCCCTCGCCACCAAGCGACGTGGAGGTCGCCTGGTCATC
GTCAACCTTCAGCCCACCAAGCACGACCGCCACGCAGACCTGCGAATCCACGGTTATGTT
GATGAGGTCATGACGCGGCTCATGAAACACCTGGGCCTGGAGATCCCGGCCTGGGACGGC
CCCCACATGGTGGAGAGGGCACTGCCGCCCCTGCCACGCCCACCTGCCCCCAAGCTGGAG
CCCAAGGAGGAGGCCTCCCCCCAGCTCAACAGCCCAGTGCCTGCCAACCCCAAGCAGGAA
CCCACAGCTGAGCCCTGCACCCAGCACAATGGTTCTGGACCCACCAGCCCCAAAAGGGAG
CGGCCGGACAGCCCTTCCCCTCACAGGCCTCCCAAAAGAGTGAAAACCGAGGTGGTGCCT
AGC
>Dog_SIRT6
ATGGAGGAGCGGGGCCTGGCCCCCAAATTCGACACCACCTTTGAGAGCGCGCGGCCCACG
CAGACCCACATGGCACTGGTGCAGCTGGAGCGCGTGGGCCTCCTCCGCTTCCTGGTCAGC
CAGAATGTGGATGGGCTGCATGTGCGCTCCGGTTTCCCCAGGGACAAGCTGGCGGAGCTC
CACGGAAACATGTTCGTAGAGGAATGTGTCAAGTGTAAGACGCAGTACGTCCGGGACACC
GTAGTGGGCAGCATGGGCCTCAGGGCCACAGGCCGGCTCTGCACTGTGGCCAAAGCGAGG
GGGCTGCGGGCCTGCAGGGGGGGAGCTGAGGGATACCATCCTGGACTGGGAGGACGCCCTG
CCTGACCGGGACCTCACTCTTGCCGACGAGGCCAGCAGGAACGCAGACCTGTCCATCACG
CTGGGCACCTCCCTGCAAATCCGGCCCAGCGGGAACCTGCCACTCGCCACTAAGCGCCGA
GGAGGCCGACTGGTCATTGTCAACCTTCAGCCCACAAAGCACGACCGCCATGCCGACCTG
CGCATCCACGGCTATGTGGATGAGGTCATGACCAGGCTTATGAAGCACCTGGGCCTGGAG
ATCCCTGCCTGGGACGGTCCCCGCGTGCTGGAGAGAGCGCTGCCACCCCTGCCCCGCCCA
CCCGCAAAGCCCCCCGAGCCCGAGCCCAAGGAGGAGGCGCCCGCCCAGCTCAATGGCCCA

GCACCCGCCAGTCCCAAGCAGGAGCCCTCCACAGAGCCCTGCACCCAGCACAATGGCTCT
GGGCCCGGCAGCCCCAAGAGGGAGCGGCTGGACAGTCCTGTACCACACAGGCCCCCCAAA
AGGGTGAAGGCCGAGGTGGCCCCCAGC
>Dolphin_SIRT6
ATGTCGGTGAATTATGCGGCGGGGCTGTCGCCGTACGCGGACAAGGGCAAGTGCGGTCTC
CCCGAGGTCTTTGACCCCCCTGAGGAGTTGGAGCGGAAGGTATGGGAGCTGGCACAGCTG
GTCTGGCAGTCCTCCAACGTGGTGTTCCACACGGGCGCGGGCATCAGCACAGCCTCAGGC
ATCCCCGACTTCAGGTCCGTGCGCATGGAGGGAAGGGCCCCCCATGGCGTCTGGACGATG
GAGGAGCAGGGCCTGGCCCCCAAGTTCGACACCACCTTCGAGAACGCGCGGCCCACGAAG
ACTCACATGGCACTGGTGCAGCTGGAGCGCGTGGGCCTCCTGCGCTTCCTGGTCAGCCAG
AACGTGGACGGGCTGCACGTGCGCTCTGGCTTCCCCAGGGACAAGCTGGCAGAGCTTCAC
GGAAACATGTTTGTAGAAGAATGTGTCAAGTGTAAGATGCAGTACGTCCGGGACACCGTC
GTGGGCAGCATGGGCCTGAAGGCCACCGGCCGGCTCTGCACCGTGGCCAAGTCAAGGGGG
CTGCGGGCCTGCAGGGGGGAGCTGAGAGACACTATCCTGGACTGGGAAGACTCCCTGCCT
GACCGGGACCTCACTCTGGCCGATGAGGCCAGCAGGAACGCGGACCTGTCCATCACGCTG
GGCACCTCCCTGCAANTCCGGCCCAGCGGGAACCTGCCCCTCGCCACCAAACGCCACGGA
GGCGGGCTGGTCATCGTCAACCTTCAGCCCACCAAGCACGACCGTCACGCAGACCTGCGT
ATCCACGGCTATGTAGATGAAGTCATGACCCGTCTCATGAAGCATCTGGGCCTGGAGATC
CCGGCCTGGGACGGCCCCCGCGTGCTGGAGAGGGCGCTGCCACCCCTGCCCCGCCCACCC
ACCCCCAAGCTGGAGCCCAAGGAGGAGGCCCCTGCCCAGCTCCACAGCCCAGCGCCCGCC
AGCCCCAAGCAGGAGCCCGCAGCTGAGCCCGGCGCCCAGCATGACGGCTCCGGGCCCGCC
AGCCCCAAAAGGGAGCGGCTGGACAGTCCTGCTCCCCACAGGCCCCCCAAAAGAGTGAAG
ACCGAGGTGGTTCCCAGC
>Duck_SIRT6
ATGGCCCCAGGACTCCCTCTGCCAGTGCCCACCATCCTGCTGGCGCGCAGCGCGGAGCTC
CTGGCTGTGTCTCTCCATTCCCCTGGTGGTTTCTCTTCTCAGATCTTTGACCCGCCGGAG
GAGGTGGAGAGGAAGGTGCGTGAGCTGGCGGATTTGATCAGGAGCTCCTCCAACGTGGTG
TTCCACACCGGGGCCGGGATCAGCACCGCCTCGGGGATCCCCGACTTCAGGGGGCCCAAT
GGCGTCTGGACTATGGAAGAGAAGGGGCTCTCCCCAAAGTTCGACACCACCTTCGAGAAC
GCCAGGCCCTCCAAGACTCACATGGCGCTGCTGGGGCTGCAGAGGGTCGGCATCCTGAAA
TTCCTGGTCAGCCAAAATGTGGACGGCCTTCACGTGCGGTCGGGATTCCCACGGTACTAC
CCCCTCCTCCCTGCCTCCAGGTCCCTCGTGCCTGTCCCCTCTGCGTGCGGTTCGTGCTTA
GCACAGCCTCACGTGCAAACGTTTGCAGACAGAAAGCAACAGTTTAAGGGTAACCCCGGT
CCCCGTGTGTCTTCTAGAGGGAAGCTACGAGACACGATTCTGGACTGGGAGGATTCCCTT
CCCGACCGTGACCTCACGCTGGCGGACGAAGCCTGCAGGAAAGCCGACCTCTCTGTCACG
CTGGGGACCTCCCTGCAGATCAAACCCAGCGGCAACCTCCCGCTGATCACGAAGAAGAGA
GGAGGGAAGCTCGTCATCGTCAACCTGCAAGCCACCAAGCACGACAAACAGGCCGACCTG
CGCATCCACGCCTACGTCGACGATGTCATGACCAAGCTGATGAAGCACCTGGGGCTGGAG
GTCCCGCTCTTCAAATTTGAGCCCGAGGCACGCGGGCTGCTCAAGGAGGAATCCTTCGCC
CAGTGCAACGGCACGGCCGGGCTGTGCCCCGACCTCGGGACCACGCTGGTGGAGNNNNNN
GACAGCCTGCAGCAGGACACGGGGCCGCCGCCGCCGAAGAAGGTGAAGGTGGAGCCTCTC
CTCACC
>Elephant_SIRT6
ATGTCGGTGAATTACGCGGCGGGGCTGTCGCCGTATGCTGACAAGGGCAAATGCGGCCTC

CCCGAGATCTTCGACCCCCCAGAGGAGCTGGAGCGGAAGGTGTGGGCGCTGGCCCAGCTG
CTCTGGGAGGCCTCCAATGTGGTCTTCCACACAGGCGCCGGCATCAGCACCTCCTCCGGT
ATCCCCGACTTCAGGGGCCCACATGGCGTCTGGACTATGGAGGAAAGGGGCCTGGCCCCC
AAGTTCGATACCACCTTCGAGAGCGCACGGCCCTCGCAGACCCACATGGCGCTGGTGCAG
CTGGAGCGGGTGGGCCTGCTTCACTTCCTGGTCAGTCAGAACGTGGACGGGCTGCACATG
CGCTCTGGCTTCCCCAGGGACAAGCTGGCCGAGCTTCATGGGAACATGTTTGTAGAAGAA
TGCGTCAAGTGTAAGACGCAATACGTCCGGGACACAGTTGTAGGCAGCATGGGCCTCAAG
GCCACCGGCCGGTTCTGCACCGTGGCCAAGGCCAGGGGGCTGCGGGCCTGCAGGGGGTCG
CTGAGAGACACCATCCTGGACTGGGAGGACTCACTGCCTGACCGGGACCTCGCCCTCGCT
GATGAGGCCAGCAGGAACGCCGACCTGTCCATCGCCCTGGGCACCTCCCTGCAAATCCGG
CCCAGCGGCAACTTGCCGCTGGCCACCAAGCGCCGGGGCGGCCGGCTGGTCATCGTCAAC
CTCCAGCCCACCAAACACGACCGCCACGCCGACCTGCGCATCCACGGCTACGTAGATGAC
GTCATGACGCAGCTCATGAAGCACCTGGGGCTGGAGATCCCCGCCTGGGACGGACCCCGC
GTGGTGGAGAGGGCGCTGCCACCCCTGCCCCGCCCGCCCGCCCCCAAGCTGGAGCCCAGG
GAGGAGGACCCTGCCCAGCTCAACGGCTCAGCGCCCCCAAGCCCCAAGCAGGAGTCCACT
GCCCAGCACAACGGCTCCGGGCCCGCCAGCCCCAAGCGGGAGTGGCCAGACACTCCTGCT
CTCTGCAGGCCCCCAAAAAGG
>Flycatcher_SIRT6
ATGCAAAATCCTTCATGGATTTTTGACCCTCCGGAGGAGCTGGAGCGGAAGGTGCAGGAG
CTGGCAGAGCTGATCCGGAGCTCCTCCAATGTGGTGTTTCACACAGGGGCGGGGATCAGC
ACAGCCTCGGGGATCCCTGACTTCAGGGGCCCCAATGGTGTCTGGACCATGGAAGAGAAA
GGGCTCTCCCCAAAATTCGACACCACCTTTGAGAACGCCAGGCCCTCCAAGACTCACATG
GCGCTGCTGGGGCTGCAGAGAGTGGGAATCCTGAAATTCCTGGTCAGCCAGAACGTGGAC
GGGCTGCACGTGCGGTCAGGATTCCCACGGGACAAGCTGGCTGAGCTCCACGGGAACATG
TTTGTGGAAGAGTGTGTGAAATGTGGGAAGCAGTACGTGCGTGATGCCGTCGTGGGCAGC
ATGGGGCTCAAGCCCACAGGACGCCTCTGCAGCGTCACCAAGGCCCGGGGGCTGAGGGCC
TGCAGAGGGAAGTTACGAGACACTATTCTGGATTGGGAGGATTCCCTGCCTGACCGTGAC
CTGATGCTGGCAGATGAAGCCTGCAGGAAAGCCGATCTCTCTGTCACCCTGGGGACCTCT
CTGCAGATCAAACCCAGCGGGAACCTCCCACTCATCACCAAGAAGAGAGGAGGGAAGTTG
GTCATTGTCAATCTCCAAGCAACCAAACACGACCGCCAGGCCGACCTGCGCATCCACGGC
TACGTCGATGAGGTGATGACCAAGCTGATGAAGCACCTGGGGCTGGAGGTGCCCGAGTGG
ACGGGGCCGGTGGTGGTGGAGAGGGCTGAGGTGGCCAAGGCTGAGGTGGCCCAGGCTGAG
GTGGCCCAGGCCCAGCAGCTGCAGTGTCCCCTGAAGGAGGAGCCCCTGGCCCAGCAGCTG
CAGTGTCCCCTGAAGGAGGAGCCCCTGGCCCAGCACAATGGCACGGTGCTGCCGGTGCCG
TGTGCCGGGCAGGCGCCGCGGCAGCGCCGGGAGGGGCTCAGGCTGGAGTGTCCCAGCCCC
GACACGGGGCCAACGCCGGTGAAGAAGATGAAGGTGGAGCCTCTCCTCACC
>tropical_clawed_frog_SIRT6
ATGTCGGTGAATTATGCGGCTGGCCTGTCTCCCTATTCCGATAAGGGACGATGTGGGCTC
CCGGAGGCATTTGATCCACCGGACGAACTGTGTCGCAAGGTAGTGGAACTAGCTGATATG
ATTCGGAAATCCTCCTACGTAGTATTTCACACGGGAGCCGGGATCAGTACTTCCTGCGGA
ATCCCAGATTTCAGGGGGCCTAATGGAGTGTGGACTCTGGAGGAGAAAGGTGTGAACCCA
AAGTTTGACATCACGTTTGAATCTGCATGTCCTTCCCCTACCCACATGGCGCTGCTTCAG
CTGCAGAGGGTTGGCATCCTGAAATTTTTGGTTAGCCAAAACGTAGATGGCTTGCATGTA
AGATCTGGCTTCCCCAGAGAGCAGCTGGCAGAACTACATGGAAACATGTTCGTGGAGGAA

TGCAGCAAATGTAGCAAACAGTACGTGCGAGATCAAGTTGTAGGAACCATGGGGCTCAAA
CCTACTGGGAGACTCTGTGATGTGCCCAAGGTGCGAGGCCTGAGAGCTTGCAGGGGGAAG
CTCAAAGACACCATTTTGGATTGGGAAGACTCACTTCCTGACAGAGACCTAAACCTAGCC
GATGAAGCCTGCAGGAAAGCAGACCTGTCCATTACATTGGGGACATCACTGCAGATTAGA
CCCAGTGGGAACCTACCACTCCTCACCAAGCGCAAGGGTGGGAAACTAGTTATTGTAAAC
CTTCAGCCGACCAAGCATGACAAGCACGCGGATTTGAGAATACACGGTTATGTGGATGAA
GTGATGACACAGCTCATGGAGCTTTTGGGTCACAAGATACCAGTTTGGACTGGTATGCCT
ACTAAAACAGAGCCAACTAATGGAAACTATAAGGAGGAAAATCATTTCTACAATGATTCA
GTGCTGGGTGCTAACCCAAACCAAAAAAGAGAAGGATGCAAGGAAGAACCTAATCTCGAG
CCAAAAAAGGCAAAAGTTGAGCCAGCATGTGTA
>Fruitfly_SIRT6
ATGAGCTGCAACTACGCGGATGGATTGTCAGCCTACGACAACAAGGGAATTTTGGGAGCA
CCAGAGAGTTTCGACAGCGATGAGGTTGTGGCCGAAAAGTGCCAGGAATTGGCTGAATTG
ATCAAGAAATCGGGACACGTTGTCCTCCACACGGGAGCTGGGATCAGTACGTCTGCAGGA
ATTCCGGATTTCCGCGGACCCAAGGGCGTTTGGACCCTGGAGGAGAAGGGCGAGAAGCCG
GACTTCAATGTTTCCTTCGATGAAGCCAGACCAACTAAAACCCACATGGCTATCATAGCC
CTGATTGAAAGTGGCTATGTGCAGTACGTAATCTCACAGAATATTGATGGTCTCCACTTG
AAATCCGGACTGGATCGGAAGTATCTTTCCGAATTGCACGGCAACATTTACATCGAACAG
TGTAAGAAATGCAGACGGCAATTTGTGAGCCCATCTGCCGTGGAAACAGTGGGTCAAAAA
TCCCTGCAACGTGCCTGCAAGTCTTCAATGGATAGCAAAGGTCGTAGCTGTAGATCGGGA
ATCCTATACGATAACGTTCTGGACTGGGAGCACGATCTTCCCGAAAACGACCTCGAAATG
GGTGTGATGCACTCCACCGTCGCTGACCTAAATATTGCGCTTGGAACCACTTTGCAGATC
GTTCCCAGCGGAGACCTTCCTTTAAAGAATCTAAAATGCGGTGGAAAAATTTGTCATTTGT
AATCTGCAGCCCACAAAACATGACAAAAAGGCTAACTTAATCATATCCAGTTATGTGGAT
GTGGTTTTGTCCAAAGTTTGTAAACTATTGGGTGTTGAGATACCCGAATATTCGGAGGCT
TCCGATCCTACAAAGCAGTCCAAGCCAATGGAGTGGACAATACCCACAAGCAATGTAAAT
ACCTTTCACAGACAATATAAAAAGTATGTAAAAGACTCAAAAATTGAATCTAAAGCTAAG
AAAACAAAGTACACG
>Fugu_SIRT6
ATGTCTGTGAATTATGCAGCCGGGCTTTCGCCGTACGCAGATAAAGGTGTCTGTGGTCTT
CCCGAGGAGTTTGATAGTGCTGAGGAAGTTAAGGCAAAGGTGGAGATCCTTGCCCAGCTA
ATAAAAGATTCTCGGCACTTGGTTGTCCACTCTGGAGCAGGAATAAGCACCTCATCGGGA
ATCCCAGACTTCAGAGGGCCAAAGGGTGTGTGGACGTTGGAGGAGAAGGGCGAATCGCCT
CAGTTTGAAACGACGTTTGAGGCTGCCCGACCCAGCTTAACTCACATGGCTCTCCTGGGA
CTGCAGAGGGCCGGCTATCTCAAGTACCTCATCAGCCAGAACGTGGACGGCCTGCATGTC
CGGTCAGGATTTCCCCGAGACATGCTGTCAGAGCTTCATGGAAACATGTTCGTGGAGGAG
TGTGAGAAGTGTGGCAGGCAGTACGTGAGAGATAAAGTGATTGGTGTCATGGGCCTGAAA
CCAACGGGGCGGCTCTGCGATGTGGTGCGATCCAGGGGCCTAAGAGCCTGCAGGGGAAAG
CTGATAAGCACTATACTGGACTGGGAGGATGCTTTGCCCGATAGAGACCTGAACAAGGCA
GAAGAAGCCAGTAGACAAGCGGATCTGGCTCTGACGCTGGGCACATCCATGCAGATCAAA
CCCAGCGGAGACCTGCCCCTCATCACGAAGCGCAAGGGAGGAAAGCTGGCCATTGTCAAC
CTACAGCCCACAAAGCACGACAAGCATTCATACCTGCGTATCCATGGTTATGTAGATGAT
ATAATGAAACATCTGATGGAGCTGCTGGGATTGGACATTCCAAAGTGGGAGGGGCCAACC
ATCTGCCAGAGTTCCACAAGAACGTCGGAGTGCAAAGCTGATGTCAAACCACATCAGTCG

GTTACTGTTGGCGTGAAGGTGAAAAAGGAGGTGAGGAAACGAGAAGCAGAGGCGTTAATG
GACACTGGGGACATTAAGGACGAGGTTTTGTTGAAGAAGGAGAAAGCAGAGTCTCCTGAG
GCAGATGAAGAAAACAAGTCTTTA
>Anole_lizard_SIRT6
ATGTCGGTGGACTACGCGGCGGGCCTGTCGCCCTACTCGGACAAAGGCGTGTGCGGCCTA
CCGGAGTCCTTTGACCCTCCAGAGGAGCTGGAGGAGAAGATCTCTCGCCTGGCGGACCTG
ATCCGGGAGGCTTCCAACGTCGTCTTCCACACCGGAGCCGGGATCAGCACCTCCTCGGGG
ATCCCAGACTTCCGAGGCCCCAACGGGGTCTGGACCCTGGAGGAGCGTGGCCAGCCGGCC
AAGTTCGACACCACCTTTGAGGAGGCGCGTCCTTCCCCGACCCACATGGCGCTGCTGGGC
CTCCACCGGGCCGGCCTGCTCCACTTCCTGGTCAGCCAGAACGTGGACGGCCTTCACGTC
CGCTCCGGCTTCCCCAGGGACAAACTGGCCGAGCTGCACGGGAACATGTTCGTGGAGGAA
TGCATGAAGTGCGGCAAGCAGTACGTGAGGGAGACGGTGGTGGGGACGATGGGCCTGAAG
CCGACGGGGAGGCTGTGCGACGTCTCCAAGCGCCGAGGGCTCCGCGCCTGCAGAGGGAAG
CTGATGGACACCATCTTGGACTGGGAAGATTCCCTGCCCGACCGGGACCTCCTCCTCGCC
AGCAAGGCCAGCAGGAAGGCCGACCTGTCCGTCACCCTGGGGACCTCCTTGCAGATCAAG
CCCAGCGGGGACCTGCCGCTCCTGACCAAGCGCAAGGGTGGCCGGCTGGTCATCGTCAAC
CTCCAGCCCACCAAACACGACAAGCAGGCCGACCTGCGCATCCATGCATATGTGGATGTG
GTCATGACGAAGCTGATGGGGCGGCTGGGCCTGGACATCCCCGAGTGGACCAAGCCCGAC
CCCGACCCCCGGCCGTGCCCCAAAGAGGAGAAGGCTCCACGCTACAACGGCGCCGCCGCT
CTCTGCAAACAGGAGCCGTGCACCCCGGACGCCAAGCGGTTCAAGCTGGAGCCGCTCCCT
TCC
>Horse_SIRT6
ATGTCGGTGAATTACGCGGCGGGGTTGTCGCCGTACGCGGACAAGGGCAAGTGCGGCCTT
CCCGAGATCTTTGACCCCCCAGACGAGTTGGAGCGGAAGGTGTGGGAGCTGGCACAGCTG
GTCTGGCAGTCCTCCAACGTGGTGTTCCACACGGGCGCAGGCATCAGCACCGCCTCGGGC
ATCCCTGACTTCAGGGGTCCCCATGGCGTCTGGACAATGGAGGAGCGGGGCCTGGCCCCC
AGGTTCGACACGACCTTCGAGATGGGCCGGCCCACTCAGACGCACATGGCGGCTGGGTGC
AGCTGGAGCGCGTGGGGCCTCCTGCGCTTCCTGGTCAGCCAGAACGTGGCCGGTCTCCAG
GTGCGGCCCGGGTTTCCCAGGGACAAGCTGGCAGAGCTGCACGGGAACATGTTTGTAGAA
GAATGTGTCAAGTGTAAGACGCAGTACGTCCGGGACACCGTCGTGGGCAGCATGGGCCTC
AAGGCCACGGGCCGGCTGTGCACCATGGCCAAGGCCAGGGGGCTGCGGGCCTGCAGAGGG
GAGCTGAGAGACACCATCCTCGACTGGGAAGATGCTCTGCCTGACAGGGACCTCACTCTC
GCCGACGAGGCCAGCAGGTCTGAGCCCCGGATGAGCCCAGGGTGGGGCGGGCGGGGACCT
GGGCGGAAACAGCCAGCGCCAGCCCAGGGCAGGGGATGCCAGCTCAGAGGGGGAGTCAGA
GGAGGCTTCCTGGAGGAGGCGGCCCTCTCTGGGCCTCACTTGCCCGTAATAACAGCAGCT
CCTGTGTAC
>Human_SIRT6
ATGTCGGTGAATTACGCGGCGGGGCTGTCGCCGTACGCGGACAAGGGCAAGTGCGGCCTC
CCGGAGATCTTCGACCCCCCGGAGGAGCTGGAGCGGAAGGTGTGGGAACTGGCGAGGCTG
GTCTGGCAGTCTTCCAGTGTGGTGTTCCACACGGGTGCCGGCATCAGCACTGCCTCTGGC
ATCCCCGACTTCAGGGGTCCCCACGGAGTCTGGACCATGGAGGAGCGAGGTCTGGCCCCC
AAGTTCGACACCACCTTTGAGAGCGCGCGGCCCACGCAGACCCACATGGCGCTGGTGCAG
CTGGAGCGCGTGGGCCTCCTCCGCTTCCTGGTCAGCCAGAACGTGGACGGGCTCCATGTG
CGCTCAGGCTTCCCCAGGGACAAACTGGCAGAGCTCCACGGGAACATGTTTGTGGAAGAA

TGTGCCAAGTGTAAGACGCAGTACGTCCGAGACACAGTCGTGGGCACCATGGGCCTGAAG
GCCACGGGCCGGCTCTGCACCGTGGCTAAGGCAAGGGGGCTGCGAGCCTGCAGGGGAGAG
CTGAGGGACACCATCCTAGACTGGGAGGACTCCCTGCCCGACCGGGACCTGGCACTCGCC
GATGAGGCCAGCAGGAACGCCGACCTGTCCATCACGCTGGGTACATCGCTGCAGATCCGG
CCCAGCGGGAACCTGCCGCTGGCTACCAAGCGCCGGGGAGGCCGCCTGGTCATCGTCAAC
CTGCAGCCCACCAAGCACGACCGCCATGCTGACCTCCGCATCCATGGCTACGTTGACGAG
GTCATGACCCGGCTCATGAAGCACCTGGGGCTGGAGATCCCCGCCTGGGACGGCCCCCGT
GTGCTGGAGAGGGCGCTGCCACCCCTGCCCCGCCCGCCCACCCCCAAGCTGGAGCCCAAG
GAGGAATCTCCCACCCGGATCAACGGCTCTATCCCCGCCGGCCCCAAGCAGGAGCCCTGC
GCCCAGCACAACGGCTCAGAGCCCGCCAGCCCCAAACGGGAGCGGCCCACCAGCCCTGCC
CCCCACAGACCCCCCAAAAGGGTGAAGGCCAAGGCGGTCCCCAGC
>Macaque_SIRT6
ATGTCGGTGAATTATGCGGCGGGGCTGTCGCCGTACGCGGACAAGGGCAAGTGCGGCCTC
CCTGAGATCTTCGACCCCCCGGAGGAGCTGGAGCGGAAGGTGTGGGAGCTGGCAAGGCTG
GTCTGGCAGTCTTCCCATGTGGTGTTCCATACGGGTGCCGGCATCAGCACTGCCTCTGGC
ATTCCCGACTTCAGGGGTCCCCACGGAGTCTGGACCATGGAGGAGCGAGGCCTGGCCCCC
AAGTTCGACACCACCTTTGAGAGCGCACGGCCCACGCAGACCCACATGGCGCTGGTGCAG
CTGGAACGCGTGGGCCTCCTTCGCTTCCTGGTCAGCCAGAACGTGGACGGGCTCCATGTG
CGCTCAGGCTTCCCTAGGGACAAATTGGCAGAGCTTCATGGGAACATGTTCGTGGAAGAA
TGTGCCAAGTGTAAGACGCAGTATGTCCGGGACACAGTCGTGGGCACCATGGGCCTGAAG
GCCACGGGCCGGCTCTGCACCGTGGCTAAGGCAAGGGGGCTGCGGGCCTGCAGGGGGGAG
CTGAGGGACACCATCCTAGACTGGGAGGACTCCCTGCCCGACCGGGACCTGGCACTCGCC
GATGAGGCCAGCAGGAACGCCGACCTGTCCATCACGCTGGGAACATCACTGCAGATCCGA
CCCAGTGGGAACCTGCCGCTGGCTACCAAGCGCCGGGGGGGCCGCCTGGTCATCGTCAAC
CTGCAGCCCACCAAGCACGACCGCCATGCTGACCTCCGCATCCACGGCTACGTCGACGAG
GTCATGACCCGGCTCATGAAGCACCTGGGGCTGGAGATCCCCGCCTGGGACGGCCCCCAC
GTGCTGGAGAGGGCGCTGCCACCCCTGCCCCGCCCGCCTACCCCCAAGCTGGAGCCCAAG
GAGGAATCACCCACCCGGATCAACGGCTCTATCCCTGCCGGCCCCAAGCAGGAGCCCTGC
GCCCAGCACAACGGCTCAGAGCCCGCCAGCCCCAAACGGGAGCGGCCCACCAGCCCTGCC
CCCAACAGACCCCCCAAAAGGGTGAAGGCCGAGGCGGTCCCCAGC
>Medaka_SIRT6
ATGTCAGTAAATTATGCTGCCGGATTGTCACCTTACGCAGATAAGGGGGTCTGCGGCCTT
CCTGAGAAGTTTGACAGTCCTGAAGAGCTGAAGGAGAAGGTGCAGACTCTTGCTGAGTTG
GTTAAAGAATCTCAGTACCTGGTCGTCCACACTGGAGCTGGCATCAGCACCTCAGCCGGC
ATCCCTGACTTCAGGGGTCCCAAAGGAGTGTGGACTCTAGAGGAAAAGGGTGAGTCGCCT
CACTTTGATACGACGTTTGAAGACGCTCGGCCCAGCTTGACTCACATGGCCCTCCTGGGA
CTGGAGAGGGCCGGCTACCTCAAGTATCTGATCAGCCAGAACGTTGACGGCCTGCATGTT
CGTTCAGGCTTTCCCAGGGACAAGTTATCAGAGCTTCACGGAAACATGTTTGTGGAGGAG
TGTGAGAAGTGTGGCAGACAATATGTCAGAGACAAGGTGATTGGCGTGATGGGACTCAAA
CCAACAGGCCGTTTCTGCAGTGTGGTGCGATCCAGAGGACTGAGGGGCTGCAGAGGGAAG
CTGATCAGCACAATATTGGATTGGGAGGACGCACTTCCTGACAGGGATCTGAACCGAGCG
GATGATGCAAGCAGGAAAGCAGACCTGGCACTGACTCTGGGCACGTCCATGCAGATCAAA
CCCAGCGGAGACCTCCCACTCCTCACAAAGCGCAAAGGAGGGAGGATAGCTATAGTCAAC
CTGCAGCCCACAAAGCATGACAAACAGGCCCACCTCCGTATCCATGGTTACGTGGACGAG

GTCATGAAACAGCTGATGGAGCAGTTAGGCTTAGACATCCCAAAGTGGGAGGGACCTGTT
GTGCATGAGAGCTCTGAAGTCCTGCCCGACATCAAACCACCGCACTGCATTTCTGCTGAC
GGGAAGGTGAAAAAGGAAGAAATTAAAATAGAGAGAAAAAGAGAAGCAACACAACTAACA
GGCGAGGAGGATGTGAAGGAGGAGGCAGGTTCAGTAAAAAAGGAGAGAGCAGATTTGACA
TTGTTAATAAATGAAGAAAAA
>Mouse_SIRT6
ATGTCGGTGAATTATGCAGCAGGGTTGTCGCCTTACGCGGATAAGGGCAAGTGCGGGCTG
CCCGAGATCTTCGACCCACCAGAGGAGCTGGAACGCAAGGTGTGGGAGCTGGCCCGGCTA
ATGTGGCAGTCCTCCAGCGTGGTTTTCCACACGGGCGCCGGCATCAGCACCGCCTCTGGC
ATCCCCGACTTCAGAGGCCCCCATGGCGTGTGGACCATGGAGGAACGCGGCCTGGCCCCC
AAGTTTGACACCACCTTCGAGAATGCTCGGCCCTCGAAGACCCACATGGCCCTGGTTCAG
CTAGAACGCATGGGCTTCCTCAGCTTCCTGGTCAGCCAGAACGTAGACGGGCTGCACGTG
CGCTCGGGCTTCCCCAGGGACAAGCTGGCAGAGCTGCACGGAAACATGTTTGTAGAGGAA
TGTCCCAAGTGTAAGACGCAGTACGTCAGAGACACGGTTGTGGGCACCATGGGCCTCAAG
GCCACAGGCCGGCTCTGCACCGTGGCCAAGACCAGGGGACTTCGGGCCTGTAGAGGGGAG
CTGAGAGACACCATTCTGGACTGGGAGGACTCGTTGCCTGACCGGGACCTGATGCTCGCT
GATGAGGCCAGCAGGACCGCAGACCTGTCTGTCACCCTGGGTACCTCGCTGCAGATCCGC
CCCAGTGGGAACCTGCCCCTTGCCACTAAGCGCCGAGGAGGCCGTCTGGTCATTGTCAAC
CTGCAACCCACAAAACATGACCGCCAGGCTGACCTGCGCATCCACGGCTACGTGGATGAG
GTGATGTGCAGACTCATGAAGCATCTGGGGCTGGAGATTCCAGCCTGGGATGGACCCTGC
GTGCTAGACAAAGCCCTGCCACCTCTGCCTCGCCCAGTAGCACTCAAGGCTGAGCCCCCC
GTGCATCTCAATGGTGCAGTGCATGTTTCGTATAAGTCCAAGCCCAACAGCCCTATACTC
CACAGGCCCCCCAAAAGAGTGAAGACCGAGGCTGCCCCCAGC
>Naked_mole_rat_SIRT6
ATGTCGGTGAACTACGCGGCGGGGCTGTCGCCGTATGCGGATAAGGGCAAGTGTGGCCTC
CCCGAGATTTTTGACCCCCCGAAGGAGTTGGAGCTGAAGGTGTGGGAGCTGGCCAGGCTG
GTGTGGCAGTCCTCCAATGTGGTGTTCCACACAGGCGCTGGCATCAGTACTTCCTCAGGC
ATCCCTGACTTCAGGGGCCCGCACGGTGTGTGGACCATGGAGGAGCGGGGTCTGGCGCCC
AAGTTCGACACGACCTTTGAGAGCGCGCGGCCCACGCGCACACACATGGCGCTGGTGCAG
CTGGAGCGCGTGGGGCTGCTGCACTTCCTCGTCAGCCAGAACGTGGACGGGCTGCATGTG
CGCTCCGGCTTCCCCAGGGACAAACTGGCAGAGCTGCATGGGAACATGTTCGTAGAAGAA
TGTGCCAAGTGTAAGACGCAGTACGTCCGGGACACGGTCGTGGGCACCATGGGCCTCAAG
GCCACCGGGAGGTTCTGCACAGTGGCCAAGGCGCGGGGACTGCGGGCTTGCCGGGGGGAG
CTGAGAGACACCATCCTGGACTGGGAAGATGCCCTGCCTGAGCGGGATCTGGCTCTCGCC
GACGAGGCCAGCAGGAACGCGGACCTGTCCATCACCCTGGGGACCTCCCTGCAGATCCGG
CCCAGCGGGAACCTGCCCCTGGCCACCAAACGCCGGGGCGGCCGCCTGGTCATTGTGAAC
CTGCAGCCCACCAAGCACGACCGCCAGGCTGACTTGCGCATCCACGGCTACGTGGACGAT
GTGATGGCTGGGCTCATGCGACACCTGGGGCTGGACATCCCCGCCTGGCACGGGCCCCGA
GTGCTGGAAAGGGCCCTTCCCCCACTGCCCCGGCCTCCCGCACCCAAGCTGGAGCAGAAG
GATGACAAGGGTGAGGACTCTACAGCCCAGCTCAATGGCTCTGCGCCCACCCAGCCCAAG
CCAGAGCCCAGCAGCCCCAAACTGGAGCGGCCCGAGAGCCTGGCCCCTTGCAGGCCCCCC
AAGAGGCTGAAGGCTGAGGCAGTGCCCAGC
>Opossum_SIRT6
ATGGAAGAAAGGGGTCTGGCACCCAAGTTTGACACAACATTTGAGAGTGCCCAGCCTTCT

AAGACCCACATGGCCCTGATTCAACTGGAGCGAGTGGGCATCTTGAAATTCCTGGTGAGC
CAGAATGTAGATGGTTTGCACGTACGCTCCGGCTTCCCCAGGGACAAACTGGCCGAGCTC
CATGGGAACATGTTTGTGGAAGAATGTGCCAAGTGTAAGACGCAGTATGTACGAGATGTG
GTGGTGGGCAGCATGGGCCTCAAGGCAACAGGTCGGTTGTGCACTGTGGCAAAGGCCCGA
GGTCTCCGGGCCTGCAGGGGGGAACTGAGGGACACAATCCTGGACTGGGAAGATGCTCTA
CCTGATCGAGACCTCAGCCTTGCTGATGAAGCTTGCAGGAATGCAGATCTATCCATCACG
CTGGGTACATCACTGCAGATACGACCCAGTGGCAACCTGCCTCTGCTCACTAAACGCAAA
GGAGGCCGCTTGGTCATAGTCAACCTGCAGGCCACCAAACATGATCGCCAGGCTGACCTT
CGAATTCATGGCTACGTCGATGATGTCATGGCCAAGCTCATGAAGCACCTATGCTTGGAG
ATCCCTGAGTGGCAGGGGCCCCTTGTGGTGGAACAAGCCCCACCCCTCTTACCCCTTCCG
ATACAGGTGAAAGCTGAGCCCCCCACCCTGCAAGCCCTGCCTGCAGCACCCAAGGCAGAG
GCCTCTGTCCAGCGCCACAGACCAAAGCAGGAAGGGGGCAAACGGGAGCAACCCCCTGCT
ACCCAGCTGCCCAAGAGGCTGAAGGTGGAACCTATGCCCAGC
>Orangutan_SIRT6
ATGTCGGTGAATTACGCGGCGGGGCTGTCGCCGTACGCGGACAAGGGCAAGTGCGGCCTT
CCCGAGATCTTCGACCCCCCAGAGGAGCTGGAGCGGAAGGTGTGGGAACTGGCGAGGCTG
GTCTGGCAGTCCTCCAATGTGGTGTTCCACACGGGTGCCGGCATCAGCACTGCCTCTGGC
ATCCCCGACTTCAGGGGTCCCCACGGAGTCTGGACCATGGAGGAGCGAGGTCTGGCCCCC
AAGTTCGACACCACCTTTGAGAGCGCGCGGCCCACGCAGACCCACATGGCGCTGGTGCAG
CTGGAGCGCGTGGGCCTCCTCCACTTCCTGGTCAGCCAGAACGTGGACGGGCTCCATGTG
CGCTCAGGCTTCCCCAGGGACAAACTGGCAGAGCTTCACGGGAACATGTTTGTGGAAGAA
TGTGCCAAGTGTAAGACGCAGTATGTCCGGGACACAGTCGTGGGCACGATGGGCCTGATA
GCCACGGGCCGGCTCTGCACCGTAGCTAAGGCAAGGGGGCTGCGGGCCTGCAGGGGAGAG
CTGAGGGACACCATCCTAGACTGGGAGGACTCCCTGCCCGACCGGGACCTGGCACTCGCC
GATGAGGCCAGCAGGATATGAACGCCGAACCTGTCCATCACGCTGGGCACATCGCTGCAG
ATCCGACCCAGCGGGAACCTGCCGCTGGCTACCAAGCGCCGGGGAGGCCGCCTGGTCATC
GTCAACTTGCAGCCCACCAAGCACGACCGCCATGCTGACCTCCGCATCCATGGCTACGTT
GACGAGGTCATGACCCGGCTCATGAAGCACCTGGGGCTGGAGATCCCCGCCTGGGACGGC
CCCCGTGTGGCTGGAGAAGGCCGCTGACCACCCCTGCCCCGTCCGCCCACCCCCAAGCTG
GAGCCCAAGGAGGAATCACCCACCCGGATCAACGGCTCTATCCCCGCCGGCCCCAAGCAG
GAGCCCTGCGCCCAGCACAACGGTTCAGAGCCCGCCAGCCCCAAACGGGAGCGGCCCACC
AGCCCTGCCCCCCACAGACCCCCCAAAAGGGTGAAGGCCGAGGTGGTCCCGAGC
>Western_painted_turtle_SIRT6
ATGGAGGAGAGGGGATTGTCCCCCAAGTTTGACACTACCTTTGAGAACGCGAGGCCTTCC
AAGACCCACATGGCGCTGCTGGAGCTGCAGAGAGTCGGCATCTTGAAGTTCCTGGTCAGC
CAAAACGTGGATGGCCTGCATGTGCGTTCTGGATTCCCACGGGACAAGCTGTCCGAGCTG
CACGGGAACATGTTTGTGGAGGAATGTGTGAAATGCGGCAAGCAGTACGTGCGGGACGTT
GTCGTGGGCAGCATGGGCCTCAAGCCGACAGGCAGGCTGTGCGATGTCTCCAAAGCAAGA
GGGCTTCGTGCCTGCCGAGGAAAGTTAATAGACACCATTTTAGACTGGGAAGATTCTCTG
CCTGATCGGGACCTCAGCCTAGCAGACGACGCCTGCAGGAAAGCTGACCTATCAGTCACT
CTGGGGACCTCTCTTCAGATCAAACCCAGTGGCAATCTCCCGCTGCTCACTAAAAGGAAA
GGAGGGAAGCTGGTCATAGTAAATCTGCAAGCAACGAAGCACGACCGGCAAGCCGACCTG
CGCATCCACGGCTACGTGGATGAAGTCATGACAAAGCTGATGAAGCAGCTTGGTCTGGAG
ATCCCCGAGTGGACGGGGCCGGCGGTGGTGGAGAGTTCTGAGCTTGTGAAGCCCGAACCA

GCCCTAAAACTCGACCCGGACGCTCAGCGCCCAGCCAAGGAAGAACCCTGCTCCCACCAC
AATGGCACGGCAGAGGGGGCTGACGGGATACGCCCGGGGCTCGGCACGGCACGCGAGGAG
CACTGTGACGGTCTGAAGCAGGAATGTCCTGGCTCAGGCCACAGTCCCGTAGCAGCCAAG
AGAGTAAGAGTGGAGTCGCTGCTCACC
>Panda_SIRT6
ATGTCGGTGAATTACGCGGCGGGACTGTCGCCGTACGCGGACAAGGGCAAGTGTGGCCTC
CCCGAGATCTTCGACCCCCCTGAGGAGTTGGAGCGGAAGGTGTGGGAGCTGGCGCAGCTG
GTCTGGCAGTCCTCCAACGTGGTGTTCCACACGGGTGCTGGCATCAGCACCGCCTCGGGG
ATCCCTGACTTCAGGGGCCCCCATGGTGTCTGGACGATGGAGGAGCGGGGCCTGGCCCCC
AAATTTGACACCACCTTCGAGAGCGCGCGGCCCACGCAGACCCACATGGCGCTGGTGCAG
CTGGAGCGCGTGGGCCTCCTCCGTTTCCTGGTCAGCCAGAACGTGGATGGGCTGCACGTG
CGCTCCGGTTTCCCCAGGGACAAGCTGGCGGAGCTCCACGGAAACATGTTCGTAGAGGAG
TGTGTCAAGTGTAAGACGCAGTACGTCCGGGACACCGTCGTGGGCAGCATGGGCCTCAAG
GCCACGGGCCGGCTCTGCACTGTGGCCAAGGCGAGGGGGCTGCGGGCCTGCAGGGGGGAG
CTGAGAGATACCATCCTGGACTGGGAGGACGCCCTGCCTGACCGGGACCTCACTCTCGCT
GATGAGGCCAGCAGGAACGCAGACCTGTCCATCACACTGGGCACCTCCCTGCAAATCCGG
CCGAGCGGGAACCTGCCGCTTGCCACCAAGCGCCGAGGAGGCCGACTGGTCATTGTCAAC
CTTCAGCCCACGAAGCATGACCGCCACGCTGACCTGCGAATCCATGGCTATGTGGACGAG
GTCATGACCCGGCTCATGAAGCACCTGGGCCTGGAGATTCCCGCCTGGGACGGCCCCCGC
GTGCTGGAGAGGGCGCTGCCCCCCCTGCCCCGCCCGCCCGCGCCCAAGCTGGAGCCCAAG
GAGGAGGCCCCCACCCAGCTCAATGGCCCAGCACCCGCCAGCCCCAAGCCGGAGCCCTCC
TCGGAGCCCTGCACCCAGCACAACGGCTCTGGGCCCGCCAGCCCCAAGAGGGAGCGGCTG
GACAGTCCTGCACTGCACAGGCCCCCCAAAAGGGTGAAGGCCGAGGTGGCCCCCAGC
>Rock_pigeon_SIRT6
ATGTGGGGACCCTTTATTTTTGACCCGCCGGAGGAGCTGGAGAGGAAGGTGCGGGAGCTG
GCGGACCTGATCAGGAGCTCCTCCAATGTGGTGTTTCACACGGGGGCTGGAATCAGCACC
GCCTCGGGGATTCCTGACTTCAGGGGGCCCAATGGTGTGTGGACTATGGAAGAGAAGGGG
CTCTCCCCAAAATTCGACACCACCTTTGAGAACGCCCGCCCCTCCAAGACCCACATGGCG
CTGCTGGGGCTGCAGAGAGTCGGCATCCTGAAGTTCCTGGTCAGCCAGAACGTGGACGGC
CTGCACGTGCGTTCGGGATTCCCACGGGACAAGCTGGCCGAGCTGCACGGGAACATGTTC
GTGGAAGAGTGCGTGAAATGCGGCAAGCAGTACGTGCGCGACGCCGTGGTGGGCAGCATG
GGGCTGAAGCCGACGGGCCGGCTGTGCAGCGTCACCAAAGCGCGGGGGCTGCGCGCCTGC
AGAGGGAAGTTACGAGACACCATCCTGGACTGGGAGGATTCCCTGCCGGACCGTGACCTG
ACGCTGGCAGACGAAGCCTGCAGGAAAGCCGATCTCTCCGTCACGCTGGGGACCTCTCTG
CAGATCAAACCCAGTGGCAACCTCCCGCTGATCACCAAGAAGAGGGGAGGGAAGCTGGTC
ATCGTCAACCTGCAAGCAACCAAACACGACAGACAGGCCGACCTGCGCATCCACGCCTAC
GTCGACGACGTCATGACGAAGCTGATGAAGCATTTGGGGCTGGAGGTCCCGGAGTGGACG
GGGCCGGTGGTGGTGGAAAGCGCCGAGCTCCCCAAACCCGAACAGCTCTTCGAATTTGCC
CCCGGGGCTCACGGGCCGCTGAAGGAGGAGCCCCTGTCGCAGCAGAACGGCACGGGGGGG
CTCGGCCCCGACCGCGGGACCACGCTGGGCGAGCGCCGCGACAGTTTGAAGCAGGAGCGT
CCCAGCCCGGACACGGGGCCAACGACGGTGAAGAAGATGAAGGTGGAGCCTCTCGCCACC

>pig_SIRT6
ATGTCGGTGAATTATGCGGCTGGCCTGTCGCCGTACGCAGACAAGGGCAAGTGCGGTCTC

CCCGAGGTGTTTGACCCCCCTGAGGAATTGGAGCAGAAGGTATGGGAGCTGGCACAGCTG
GTCTGGCAGTCCTCCAACGTGGTGTTCCACACGGGCGCGGGCATCAGCACCGCCTCAGGC
ATCCCTGACTTCAGGGGTCCCCATGGTGTCTGGACAATGGAGGAGCGAGGCCTGGCCCCC
AAGTTCGACACCACCTTCGAGAACGCGAGGCCCACGAAGACCCACATGGCACTGGTGCAG
CTGGAGCGCGTGGGCCTCCTCCGCTTCCTGGTCAGCCAGAACGTGGATGGGCTGCATGTG
CGATCCGGCTTCCCCAGGGACAAGCTGGCAGAGCTTCATGGAAACATGTTCGTAGAAGAA
TGTGTCAAGTGTAAGACGCAATACGTCCGGGACACCGTGGTGGGGAGCATGGGCCTGAAG
GCTACCGGCCGCCTCTGCACCGTGGCCAAGTCAAGGGGGCTACGGGCCTGCAGGGTGGAG
CTGAGAGACACCATCCTAGACTGGGAGGATGCCCTGCCTGACCGAGACCTCACCCTCGCT
GACGAGGCCAGCAGGAACGCCGACCTGTCCATCACGCTGGGCACCTCCCTGCAAATCCGG
CCCAGCGGGAACCTGCCGCTCACCACCAAACGCCGGGGGGGCCGGCTGGTCATTGTCAAC
CTTCAGCCCACCAAGCACGACCGCCATGCAGACCTGCGTATCCACGGCTACGTAGATGAG
GTCATGACCCGGCTCATGAAGCACCTGGGCCTGGAGATCCCGGCCTGGGACGGCCCCCGT
GTCCTAGAGCTCACTTTATTACTCGTCTCTGCCCTTGTGGGGAGACCTCAGGACTCTGAG
AGCTGGGCTCCAGGCCCACATTACACCTCCCTTAGCTTGGGGCCTCTGGAGGGGGCCTGG
CAAGGGGCATCCCTGCCCTGGGCC
>Platypus_SIRT6
ATGGAAGAACGTGGATTGGAGCCCAAATTTGACACAACCTTTGAGAGCGCCAGGCCCTCT
AAGACCCACATGGCCCTGCTGGAACTGGAGCGTGTTGGTATCCTGAAGTTCCTGGTGAGC
CAAAATGTGGATGGCTTGCACGTGCGCTCTGGATTCCCCAGGGACAAACTGGCAGAGCTC
CACGGAAACATGTTTGTGGAGGAATGTGTTAAATGCAAGAAGCAGTACGTGCGTGACACG
GTGGTGGGCAGCATGGGACTCAAGGCGACGGGTCGGCTGTGCAGCGTGGCGAAGGGTCGT
GGCCTCCGGGCCTGCAGAGGAGAGCTCCGGGACACCATCTTAGACTGGGAGGACTCCCTG
CCGGACCGGGACCTCAACCTCGCGGACGAAGCGTGCAGGAACGCCGATCTGTCCATCACC
CTGGGCACCTCGCTGCAGATCAAGCCCAGCGGCAATCTTCCCCTCATCACCAAGCGCAAA
GGTGGCAAGCTGGTCATCGTCAACCTTCAGGCCACCAAGCATGTGAGTGAGCCGCCGGGC
AGAGCCCTCGTCCCCCCGTGGGGACCACTGCCTGTCAGCCCGTCCCCTCCCCGAGCCGGC
CTTAGGCCAGACCAGAAGGAGCCTCTGGCTCGGCGGGCGTTGAGAGGCCAGGCCAGACGG
CTACCGCGAAAGCGTGAGAGGCAGCGGCCGGGAAGCCCGCTCACCTGCTCGGCTGCCGCT
TCCTCTCCAACCCTGCCACGGCGAGGGCCCAGAGGGGCCCCTCGAGACCGCAGCGTTTCA
CTTCTCTTGGTGCGCCGGGGCGGATACGGGTGGGAGAGA
>Tetraodon_SIRT6
ATGTCTGTGAATTATGCAGCCGGGCTCTCGCCGTACGCAGATAAAGGTGTCTGTGGTCTT
CCCGAGGAGTTTGATAGTCCTGATGAACTTAAGGCAAAGGTGGAGATCCTTGCTCAGCTC
ATAAAAGAATCCCAGTACTTGGTTGTCCACTCTGGGGCAGGAATAAGCACCTCGGCAGGC
ATCCCAGACTTCAGGGGCCCAAAGGGCGTGTGGACGTTGGAGGAGAAAGGTGAATCACCT
CAGTTTGAAACGACGTTTGAGGACGCCCGACCCAGCTTAACTCACATGGCCCTCCTTGGA
CTGCAGAGGGCCGGCTACCTCAAGTACCTCATCAGCCAGAATGTGGACGGTCTGCATGTC
CGCTCGGGATTCCCCAGGGACATGCTGTCAGAGCTTCATGGAAACATGTTTGTGGAGGAG
TGCGAGAAGTGTGGCAGGCAGTATGTGCGAGACAAAGTGATCGGCGTCATGGGCCTGAAA
CCAACAGGACGGCTCTGCGATGTGGTGCGATCCATGAGCCTCAGAGCCTGCCGAGCAGGT
GAGACGGCAAAGACTGCATGTGTCTTAGAAGAGTGCAGTTCTGGTATTGCTAAAGTCAAT
CGTGACAACCTCCACAGACGAGCAGACCTGGCGCTGACGCTTGGGACGTCCATGCAGATC
AAACCTAGTGGAGATCTGCCCCTCATCACAAAGCGCAAGGGAGGAAAGCTGGCCATCGTC

AACCTGCAGCCCACTAAGCATGACAAGCATTCATACCTGCGTATCCATGGTTACGTGGAT
GATATCATGAAACAGCTGGTGGAGCTGCTGGGACTTGACGTTCCAAAATGGGAGGGTCCG
ACCGTCTGCGAGAGGTCCACAATAACGCCAGAGTGCACGGCTGATGTCAAACCACCCCAG
TCGGCTCCTGTC
>Tilapia_SIRT6
ATGTCTGTGAATTATGCAGCTGGACTCACGCCGTACGCAAACAAAGGTGTCTGCGGACTT
CCTGAGCACTTTGATAGTCCTGAGGAGCTGAAGGCCAAGGTGGAGACTCTAGCTCAGCTG
ATTAAAGAATCTCAGTACTTGGTTGTCCACTCAGGAGCTGGGATCAGCACCTCGGCAGGT
ATCCCTGACTTCAGGGGTCCCAAGGGTGTGTGGACTCTTGAAGAAAAGGGTGAGTCGCCT
CATTTTGATACCACATTTGAAGATGCTCGCCCAAGCTTGACTCACATGGCTCTCCTGGGA
CTACAGAGGGCAGGGTACCTCAAATATCTCATCAGCCAGAATGTGGACGGCCTGCATGTG
CGATCAGGCTTCCCCAGGGATTTGTTATCAGAGCTTCATGGGAACATGTTTGTGGAGGAG
TGTGAGAAGTGTGGCAGGCAGTACGTCCGAGAAAAGGTGATCGGTGTGATGGGCCTGAAA
CCGACAGGACGCTACTGCGAGGTGGTACGATCCAGGGGGCTCCGAGCGTGCAGAGGGAAG
CTGATCAGCACTATACTTGACTGGGAGGATGCTCTTCCTGACAGAGACCTGAACAAAGCA
GATGACGCAAGCAGACGAGCGGACCTGGCTCTGACGCTGGGCACGTCCCTGCAGATCAAA
CCCAGTGGAGACCTCCCACTCCTCACCAAGCGCAAGGGTGGCAAACTGGTTATTGTCAAC
CTGCAGTCGACCAAACATGACAAGCATGCACACCTGCGTATGCATGGTTATGTGGACGAC
GTCATGAAACAGCTGATGGAGCTGCTGGGACTGGAAATCCCAAAGTGGGATGGACCAACT
GTCTGCGAGAGCTCCACAGCTACCACTGAGACCACCGCTGATGTCAAACCACCGCCTGGT
GTTACTGCGAAGGAAAAGGTGGAAAAGAACTTCGTTAAGGAGGAGAGGAAAAGAGGAGCT
TCACAGCTAACAGACGATGGAAGCGTTAAGGAGGAGACAGTTTCAGTAAAGAGGGAGAGA
GCGGACCTCCCGCTGGAGATAAATGAAGAGAAA
>Turkey_SIRT6
ATGGCGGTGAATTACGCGGCCGGGCTCTCGCCCTACTCGGATAAGGGCAAGTGCGGCCTC
CCCGAGATTTTTGATCCACCAGAAGAGCTGGAGAGGAAGGTGTGTGAGCTGGCAGACTTG
ATAAGGAGTTCTTCCAATGTGGTGTTCCACACAGGAGCTGGCATCAGCACCGCCTCGGGG
ATTCCTGACTTCAGGGGGCCTAATGGTGTCTGGACTATGGAAGAGAAGGGGCTTTCCCCA
AAATTTGACACCACCTTTGAGAATGCCAAGCCCTCCAAAACTCACATGGCACTTCTGGGG
CTGCAGAGAGTTGGCATCCTGAAATTCCTGGTCAGCCAGAACGTGGATGGCCTTCATGTG
CGCTCAGGATTCCCACGGGACAAGTTGGCTGAGCTCCACGGGAACATGTTTGTGGAGGAG
TGCATGAAATGTGGCAAGCAGTACGTGCGGGATGCTGTTGTGGGCAGCATGGGGCTGAAG
CCAACAGGCAGGCTGTGCAGCGTCACCAAAGCACGAGGGCTACGAGCCTGCAGAGGGAAG
TTAAGAGACACTATTCTGGACTGGGAAGATTCCCTGCCTGACCGTGACCTCACACTAGCA
GATGAAGCCTGCAGGAAAGCTGATCTCTCTGTTACACTGGGGACCTCTCTGCAGATCAAA
CCCAGTGGCAACCTCCCACTGATCACAAAGAAGAGAGGAGGGAAGCTGGTCATAGTCAAC
CTGCAAGCAACCAAGCATGACAGACAGGCCGACCTGCGCATTCATGCTTACGTCGATGAT
GTCATGACAAAACTGATGAAGCACCTGGGACTGGAAGTCCCAGAGTGGACAGGGCCAGTG
GTGGTGGAAAGTGCTGACTCTGCCAAGCCTGAACAGCTCTACACATTTAAGCCTGAGGTT
CACGGGTTGCTCAAGGAAGAACCCTTTTCCCAGCACAATGGAACAGCTGGGCAGTGCCCC
GACCTTGGGACCACGCTGGTGGAGCACCATGATAGTCTGAAGCAGGAGTGTCCAAGTCTG
GACACAGGACCACCACTGACAAAGAAGATGAAGGTAGAGCCTCTCCTCACC
>Zebra_finch_SIRT6
ATGGGAAAAGCAATTTTTGACCCTCCGGAGGAGCTGGAGCGGAAGGTGCAGGAGCTGGCA

GAGCTGATCCGGAGCTCCTCCCATGTGGTGTTCCACACGGGGGCAGGGATCAGCACGGCC
TCGGGCATCCCTGACTTCAGGGGCCCCAATGGTGTCTGGACTATGGAAGAAAAGGGGCTC
TCCCCAAAATTCGACACCACCTTCGAGAATGCCAGGCCCTCCAAGACTCACATGGCGCTG
CTGGGGCTGCAGAGAGTGGGAATCCTGAAATTCCTGGAGGGACAATCAGTGGAAGCCCTT
GAGGTTCCTGGGGAGGAGGGGGGGTTCCCCCAGGACTCCCCACCCTTCCCCAGCTCACCT
GTGCCTTTCTCCCACAGGGACAAGTTGGCCGAGCTCCACGGGAACATGTTTGTGGAAGAG
TGCGTGAAATGTGGGAAGCAGTACGTGCGCGACGCCGTCGTGGGCAGCATGGGGCTCAAG
CCCACCGGCCGCCTCTGCAGCGTCACCAAGGCCCGGGGGCTGCGGGCCTGCAGAGGGAAG
TTACGAGACACTATTCTGGATTGGGAAGATTCCCTGCCTGACCGTGACCTGACGCTGGCA
GATGAAGCCTGCAGGAAAGCTGATCTCTCCATCACCCTGGGGACCTCTCTGCAGATCAAA
CCCAGCGGGAACCTCCCACTGATCACCAAGAAGAGAGGAGGGAAGCTGGTCATTGTCAAC
CTCCAAGCAACCAAACACGACCGCCAGGCCGACCTGCGCATCCACGGCTACGTGGACGAG
GTGATGACCAAGCTGATGAAGCACCTGGGGCTGGAGGTGCCCGAGTGGACGGGGCCGGTG
GTGGTGGAGAGCGCCGAGCTGGCCAAGGGCGAGCAGCTGCAGGGGCGGCTGAAGGAGGAG
TCCCTGGGCCAGCACAACGGCACGGGAGCGCCGGGTGCCGGGAAGGCGCCGCTGGAGCGC
CGTGATGGGCTCAAGCAGGAGTGTCCCAGCCCGGACACGGGGCCAACGCCGGTGAAGAAG
ATGAAGGTGGAGCCTCTCCTCACC
>Zebrafish_SIRT6
ATGTCGGTGAATTACGCCGCAGGACTGTCTCCATACGCGGATAAAGGTATCTGCGGTCTG
CCGGAGACATTCGACAGTCCTGAGGAGCTGAAGACTAAAGTGGAGACTCTGGCCCAGTGG
ATCAGAGAGTCTCAGTACATGGTGGTTCATTCAGGAGCTGGAATCAGTACGTCTACAGGC
ATACCCGACTTCAGAGGTCCTAATGGTGTGTGGACGATGGAAGAGAGAGGGGAGACTCCA
CACTTTAACACCACGTTTGAGGACGCTCGACCCAGCCTGACTCACATGGCTCTGCTGCAG
ATGCAGAGGACAGGACACCTCAAATACCTCATCAGCCAAAACGTCGATGGCCTTCACGTA
CGCTCTGGCTTCCCCAGGGATCGATTGTCGGAGCTGCATGGGAACATGTTTGTTGAAGAA
TGTGAGAAGTGTGGCAAGCAATACGTTCGGGACACGGTGGTCGGAGTGATGGGACTGAAG
CCGACTGGAAGATACTGCGACGTCATGCGCTCCAGAGGACTACGATCCTGCAGAGGGAAG
CTGATCAGCTCTATATTGGACTGGGAGGACTCTCTGCCCGACAGAGACCTGAACAGAGCT
GATGAGGCGAGCAGGCGAGCTGATCTGGCTTTGACACTGGGGACGTCTCTTCAGATTAAA
CCCAGCGGAGATCTGCCTCTTCTAACCAAACGAACAGGAGGAAAACTGGTTATAGTTAAC
CTTCAGCCTACCAAACATGACAAACACGCTCACCTGCGTATATACGGTTACGTCGATGAT
GTCATGGGTCAGCTCATGAAGCTGCTGGGATTGGACGTTCCGGAGTGGGCCGGGCCAACC
CTCTGCGAAGACTCGGGTGGAGATCTGGACATCCTGCCATACGGAGCCTGGAAGAAGGAA
GTGAAGATCGAGCTGAAGATCGAGGAGAGTAATCACACCGTCTCAAAGAAACGCAAGAGG
AAAGAGCAGCACGCTGAGGAGGGCTACAAGAATGGTGTTAAAGTAGAAGAAGAGATGAAA
GAAGAGGGAAAGGAAAGTGACTCTCACGTACACACACACACT
>Great_tit_FOXO3a
ATGGTCCGCTGCGTGCCCTACTTCAAGGACAAGGGCGACAGCAACAGCTCGGCCGGGTGG
AAGAATTCGATCCGGCACAACTTGTCGCTCCACAGCCGATTTGTCAGGGTGCAGAATGAA
GGCACCGGGAAAAGCTCTTGGTGGATGATCAATCCAGATGGTGGAAAAGGCGGCAAGGCG
CCCCGGAGACGCGCCGTGTCAATGGACAACAGCAACAAGTACACCAAGAGCAGAGGGCGA
GCGGCAAAGAAAAAGGCAGCCCTGCAGACAGCCCAGGAGACGAGTGAGGATAGCCCTTCC
CAGCTCTCCAAGTGGCCAGGGAGTCCCACCTCCCGCAGCAGCGATGAGCTGGATGCGTGG
ACAGATTTTCGCTCCCGTACAAATTCAAACGCCAGTACGATCAGTGGCCGCTTGTCACCA

ATTTTGGCAAGCACCGAGCTCGATGATGTTCAAGATGACGATGCTCCACTTTCTCCCATG
TTGTACAGTAGTCCATCAAGCTTGTCCCCATCGGTAAACAAACCATGTACTGTGGAGTTG
CCTAGGTTGACTGATATGGCTGGGACAATGAACTTGAATGATGGTCTGACTGATAACCTC
ATGGATGATCTCTTGGACAATATAACACTCCCTTCCTCCCAGCAGTCACCCACAGGAGGG
ATAATGCAGAGAAGCTCCAGTTTTCCTTATGGTTCCAAAGGTTCAGGGCTGGGCTCCCCG
TCAAGTAGTTTCAACAACGCTGTGTTTGGGCCGTCATCCCTGAATTCCCTCCGCCAGTCA
CCCATGCAGACAATTCAGGAGAACAAGCAGGCCACCTTCTCTTCCATTTCTCATTACAAC
AACCAGACGCTGCAGGATCTCCTCACCTCTGATGCGCTTAGTCACAGCGACGTCATGATG
ACACAGTCTGACCCACTCATGTCACAAGCCAGCACAGCTGTGTCCGCCCAGAATTCCCGC
AGGAATATAATGCTCCGCAGCGACCCCATGATGTCGTTTGCCGCGCAGTCCAGCCAGGGC
GGTCTGGTCAACCAGAACCTGTCCCATCACCAGCACCAGTCCCACAACTCCTCTCTTAGT
GGCAGCCGTGCCTTGTCCAATTCCATCAGTAACATAGGCTTGAGTGACTCCAGCAGCTTG
GGATCCACCAAACATCAGCAGTCACCTGTCAATCAGTCTATGCAAACACTTTCTGACCCG
CTGTCAGGCTCCTCTTTGTACTCCTCTAGCGTGAGCCTCCCGGTCATGGGACATGAGAAA
TTCCCGAGTGACTTGGACCTGGATATTTTCAATGGGAGCCTGGAGTGTGACATGGAGTCC
ATTATCCGCAGTGAACTCATGGATGCCGATGGGCTGGATTTTAACTTTGATTCCCTCATC
TCAGCTCAGAACGTTGTCAGTCTGAATGTGGGGAACTTCACTGGTGCTAAACAGGCTTCA
TCACAGAGTTGGGTACCAGGC
>African_ostrich_FOXO3a
ATGCGAGGTGAAATAAATTTTCTGCGCTCCTTAATTCAACACAAATGCAGCAGCAGTGGC
CGTACAAATTCAATCCGGCACAACTTGTCACTTCACAGCCGATTCATCAGGGTGCAGAAC
GAAGGCACTGGGAAAAGCTCTTGGTGGATGATCAATCCAGATGGTGGAAAAGGTGGGAAG
GCACCGCGAAGACGTGCTGTGTCAATGGACAATAGCAACAAGTACACGAAGAGCAGAGGG
CGGGCAGCTAAGAAAAAGGCAGCTCTGCAAACTGCCCAAGAGGCAAGTGAGGACAGCCCT
TCTCAGCTCTCCAAGTGGCCCGGGAGTCCAACTTCTCGCAGCAGCGATGAGCTGGATGCA
TGGACAGATTTCCGCTCCCGTACAAATTCAAACGCCAGTACGATAAGTGGCCGCTTGTCA
CCAATTTTGGCAAGCACCGAACTAGATGACGTTCAAGATGATGACGCTCCACTTTCTCCC
ATGCTGTACAGTAGTCCATCGAGCTTGTCCCCATCAGTAAACAAACCATGTACTGTGGAG
TTACCTAGGTTGACTGATATGGCTGGGACAATGAATTTGAATGACGGACTGGCAGACAAC
CTCATGGATGATCTCTTGGACAACATAACACTCCCTTCCTCCCAGCAGTCACCCACAGGA
GGGATAATGCAGAGAAGCTCCAGTTTTCCATATGGTTCCAAAGGTTCAGGGCTGGGTTCT
CCGTCAAGTAGTTTCAATAACGCCGTATTCGGGCCGTCGTCCCTGAACTCCCTTCGCCAG
TCGCCCATGCAAACCATTCAAGAGAACAAGCAGGCCACCTTCTCTTCCATTTCTCATTAT
AACAACCAGACGCTGCAGGATCTGCTCGCCTCTGATGCACTTAGTCACAGCGATGTCATG
ATGACACAGTCTGACCCGCTCATGTCGCAAGCCAGCACAGCTGTGTCCGCCCAGAATTCC
CGCAGGAATATCATGCTCCGCAATGACCCCATGATGTCGTTTGCTGCGCAGTCCAACCAG
GGCGGTCTGGTCAATCAGAGCCTGCCCCATCACCAGCACCAGTCTCACAATTCCTCTCTT
AGTGGCAGCCGTGCCTTGTCGAATTCCATCAGTAACATAGGCTTAAATGACTCAAACAGC
TTGGGATCCACCAAACATCAGCAGTCACCTGTCAATCAGTCTATGCAAACACTTTCTGAC
CCACTCTCAGGCTCCTCTTTGTATTCCTCTAGCGTGAACCTCCCGGTCATGGGACATGAG
AAATTCCCAAGTGACTTGGACCTGGATATTTTCAATGGGAGCTTGGAGTGTGACATGGAG
TCCATTATCCGCAGTGAACTCATGGATGCAGATGGGTTGGATTTTAACTTTGATTCCCTC
ATCTCAGCCCAGAACGTTGTCAGTCTGAATGTGGGGAACTTCACTGGTGCTAAACAGGCT
TCATCACAGAGTTGGGTACCAGGC

>Adelie_penguin_FOXO3a
ATGACCCTGTCTTCAGGCTTCGGTCCTAATCCTGCAAACTTTTTCTCCCGTGAAGAGTCC
TGCAGAGGTCAGCCAGACTTCTTGCACGAACGGCGATTACTTGCTATTCAGCATCATGCT
CGGATTACGCTTAAAGTGGCCCAGTTCCCTGCGTGCTGGTATGTTGTGCGCTCCCGTGTA
TGGCACTGCTATGAAATGAAGAGGCAGCGTTTCGAACCGCCCCGTACTCCCGTTGCATTT
CTGATTACATTGTTGGCTCTTGCAGCGGGAACCATTTGTTGGGGCCTGGAGCTGCAGAGC
AGCTCGCCCGGCTGGAAGAATTCGATCCGGCACAACTTGTCACTCCACAGCCGATTCGTC
AGGGTGCAGAATGAAGGCACCGGGAAAAGCTCTTGGTGGATGATCAATCCAGATGGTGGA
AAAGGCGGCAAGGCGCCCCGGAGACGTGCTGTGTCGATGGACAACAGCAACAAGTACACG
AAGAGCAGAGGGCGGGCGGCTAAGAAAAAGGCAGCCCTGCAGACCGCCCAGGAGACGAGC
GAGGACAGCCCTTCTCAGCTCTCCAAGTGGCCAGGGAGTCCCACCTCCCGCAGCAGCGAT
GAGCTGGATGCATGGACAGATTTTCGCTCCCGTACAAATTCAAACGCCAGTACGATCAGT
GGCCGCTTGTCACCCATTTTGGCAAGCACGGAACTAGATGATGTTCAAGATGACGACGCT
CCACTTTCTCCCATGCTATACAGTAGTCCATCGAGCTTGTCCCCATCGGTAAACAAACCG
TGTACTGTGGAGTTGCCTAGGTTGACTGATATGGCTGGGACAATGAATTTGAACGATGGA
CTGACAGATAACCTCATGGATGATCTCTTGGACAATATAACACTCCCTCCCTCCCAGCAG
TCGCCCACAGGAGGGATAATGCAGAGAAGCTCCAGTTTTCCGTATGGTTCCAAAGGTTCA
GGGCTGGGTTCCCCATCAAGTAGTTTCAACAACGCCGTGTTTGGGCCGTCATCCCTGAAT
TCCCTCCGCCAGTCACCCATGCAGACCATTCAGGAGAACAAGCAGGCCACCTTCTCTTCC
ATTTCTCATTACAACAACCAGACGCTGCAGGATCTCCTTGCCTCTGATGCACTTAGTCAC
AGCGATGTCATGATGACACAGTCTGACCCACTCATGTCACAAGCCAGCACAGCTGTGTCC
GCCCAGAATTCCCGCAGGAATATAATGCTCCGCAACGACCCCATGATGTCGTTTGCCGCG
CAGTCCAGCCAGGGCGGTCTGGTCAATCAGAGCCTGCCTCATCACCAGCACCAGTCCCAC
AACTCCTCTCTCAGTGGCAGCCGTGCCTTGTCCAATTCCATCAGTAACATAGGCTTGAGT
GACTCGAACAGCTTGGGATCCACCAAACATCAGCAGTCACCTGTCAATCAGTCTATGCAA
ACACTTTCTGACCCGCTCTCAGGCTCCTCTTTGTACTCTTCTAGCGTGAACCTCCCGGTC
ATGGGACACGAGAAATTCCCAAGTGACTTGGACCTGGATATTTTCAATGGGAGCTTGGAG
TGTGACATGGAGTCCATTATCCGCAGTGAACTCATGGATGCAGATGGGCTGGATTTTAAC
TTTGATTCCCTCATCTCAGCTCAGAACGTTGTCAGTCTGAATGTGGGGAACTTCACTGGT
GCTAAACAGGCTTCATCACAGAGTTGGGTACCAGGC
>common_starling_FOXO3a
ATGGCAGAGGCGTCGCCCCCCGCCCCGCTTTCGCCCCTGGACGTGGAGCTGGACCCCGAG
TTCGAGCCGCAGAGCCGCCCCCGCTCCTGCACCTGGCCCCTGCAGAGGCCCGAGCTGCAG
GCCAGCCCAGCCAAGCCTGCCGGCGAGTCGACCGCCGACGCCGCCTCCATGATCCCCGAG
GAGGAGGACGACGAGGAGGAGGGGGCCGGCTCGGCCATGACCGTCGGCAGCGCGGCCCCC
GCGGGCGGAGAAGCGGCGGCGGCGGCGACCACCGCCGTGCCGGAGGAGGCGGCGCGG
CTGCTGGCCCCNCCCCGCGGCCTCTCGCCGGGGGGAGCGGCGGCGGCGGCAGGCGGC
GGGGGGCTGAGCGGGGGCCCGGCGGCGGCGCCGAGGAAGTGCTCGTCGCGGCGCAACGCG
TGGGGCAACCTCTCCTACGCCGACCTCATCACCCGCGCCATCGAGAGCTCCCCGGAGAAG
CGCCTCACTCTCTCCCAGATCTACGACTGGATGGTCCGCTGCGTGCCCTACTTCAAGGAC
AAGGGCGACAGCAACAGCTCGGCCGGGTGGAAGAATTCCATCCGGCACAACTTGTCACTC
CACAGCCGATTCGTCAGGGTGCAGAATGAAGGCACCGGGAAAAGCTCTTGGTGGATGATC
AATCCAGATGGTGGAAAAGGCGGCAAGGCGCCCCGGAGACGCGCTGTGTCAATGGACAAC
AGCAACAAGTACACCAAGAGCAGGGGGCGGGCAGCAAAGAAAAAGGCAGCCCTGCAGACA

GCCCAGGAGACGAGTGAGGACAGCCCTTCCCAGCTCTCCAAGTGGCCAGGGAGTCCCACC
TCCCGCAGCAGTGACGAGCTGGATGCATGGACAGATTTTCGCTCCCGTACAAATTCAAAT
GCCAGTACAATCAGTGGCCGCTTGTCACCAATTTTGGCAAGCACAGAGCTCGATGATGTT
CAAGATGACGATGCTCCACTCTCTCCCATGCTGTACAGTAGTCCATCGAGCTTGTCCCCG
TCGGTAAACAAACCATGTACTGTGGAGTTGCCTAGGTTGACTGATATGGCTGGGACAATG
AACTTGAATGATGGACTGACTGATAACCTCATGGATGATCTCTTGGACAATATAACACTC
CCTCCCTCCCAGCAGTCACCCACAGGAGGGATGATGCAGAGAAGCTCCAGTTTTCCTTAT
GGTTCCAAAGGTTCAGGGCTGGGCTCCCCATCAAGCAGTTTCAACAACGCTGTGTTTGGG
CCGTCGTCCCTGAATTCCCTCCGCCAGTCACCCATGCAGACAATTCAGGAGAACAAGCAG
GCCACCTTCTCTTCCATTTCTCATTACAACAACCAGACGCTGCAGGATCTCCTCACCTCT
GACGCACTTAGTCACAGCGATGTCATGATGACACAGTCTGACCCACTCATGTCACAAGCC
AGCACAGCTGTGTCCGCCCAGAATTCCCGCAGGAATATAATGCTCCGCAACGACCCCATG
ATGTCGTTTGCTGCTCAGTCCAGCCAGGGCGGTCTGGTCAATCAGAACCTGTCCCATCAC
CAGCACCAGTCCCACAACTCCTCTCTCAGTGGCAGCCGTGCCTTGTCCAGTTCCATCAGT
AACATAGGCTTGAGTGACTCCAACAGCTTGGGATCCACCAAACATCAGCAGTCACCTGTC
AATCAGTCTATGCAAACACTTTCTGACCCGCTGTCAGGCTCCTCTTTGTACTCTAGCGTG
AACCTCCCTGTCATGGGACATGAGAAATTCCCGAGTGACTTGGACCTGGACATTTTCAAC
GGGAGCCTGGAGTGTGACATGGAGTCCATTATCCGCAGTGAACTCATGGATGCCGATGGG
CTGGATTTTAACTTTGATTCCCTCATCTCAGCTCAGAACGTTGTCAGTCTGAATGTGGGG
AACTTCACTGGTGCTAAACAGGCTTCATCACAGAGTTGGGTACCAGGC
>central_bearded_dragon_FOXO3a
ATGGCAGAGGCGTCTCCCCCCGCCCCCTCGTCCCCCCTCGAGGTGGAACTGGACCCCGAA
TTTGAGCCCCAGAGCCGCCCGCGCTCCTGTACGTGGCCCCTGCAGAGGCCCGAGCTGCAG
GCAAGCCCGGCCAAGCCCGCCGGGGAAGCGCCTGCCGACGCCACCTCCATGATCCCGGAG
GAGGACGACGACGCGGACGACGAGGAGCAAGGGGGCAGCTCGGCCATGACCGTCGCCGTC
CGGAGTGGCGCGCCCGGCGGCGGCGACGCCGAGGCTTTGGCCCCGGTGCTGGCTCCTTTC
TCCGGGCTTGCAGGCGAGGGAGCCGGCCAGGGCGCCAGGGGGGCGCTGGGCGGCGGGCAG
CAGCAACCACAACAACAACCAGGGGCGACGGGCACGGGGACCGCCCCGCGCAAATGCTCG
TCCCGGAGGAACGCGTGGGGGAACTTGTCTTACGCGGACTTGATCACTCGCGCCATCGAG
AGCTCGCCGGAGAAGCGCCTCACCCTGTCCCAGATCTACGAGTGGATGGTGCGCTGCGTG
CCCTACTTCAAGGATAAGGGCGACAGCAACAGCTCCGCAGGCTGGAAGAATTCGATCCGG
CACAACTTGTCCCTTCATAGTCGATTCATCAGGGTACAGAATGAAGGAACTGGGAAAAGC
TCATGGTGGATGATCAATCCAGACGGTGGGAAAGGTGGGAAGGCACCACGGAGGCGGGCA
GTTTCAATGGACAACAGCAACAAGTACACAAAGAGCCGAGGGCGAGCGGCTAAGAAGAAA
GCGGCCCTGCAAGCAGCACAAGACACAAGCGAAGACAGCCCCACCCAACTCTCCAAGTGG
CCTGGCAGCCCAACATCACGAAGCAGCGATGAGCTCGAAGCGTGGACAGATTTCCGGTCC
CGGACAAACTCCAACGCCAGTACAATAAGTGGCCGCTTGTCGCCCATTTTGGCAACCACG
GAGCTCGATGAGGCTCAGGATGATGACGCTCCCCTTTCCCCAATGCTGTACAGTAGCCCA
CCTAGCATGTCCCCATCAGTAAACAAACCCTGTAATGTAGAGCTGCCGAGACTGACTGAT
ATGGCAGGCACCATGAACTTGAACGATGGCCTGACAGATAACCTAATGGATGATCTTCTG
GACAATATAACTCTCCCCGCTTCCCAGCAGTCTCCCACGGGGGGACTCATGCAAAGAAGC
TCGAGTTTTCCCTACAGTTCCAAAGGGTCAGGCCTTGGTTCCCCGTCGAGTAGTTTCAAT
AACGCCGTGTTCGGGCCGTCGTCTCTGAATTCCCTCCGACAGTCTCCCATGCAAACCATC
CAAGAGAACAAGCAAGCGACGTTTTCTTCCATGTCTCACTATAATAACCAAACCTTGCAG

GATCTGCTGGCATCGGATTCGCACAGCCACAGTGACGTCATGATGACTCAGTCCGATCCG
CTTATGTCGCAAGCCAGCACGGCGGTGTCTGCCCAAAACGTACGCAGGAATATTATGCTA
CGGAATGATCCAATGATGTCCTTTGCTGCCCAGCCAAGCCAGGGCAGTTTGGCCAACCAG
AATCTGCTCCACCACCAGCATCCACCCCAGAACTCCTCCCTCAGCGGCAGCCGTGCCTTG
TCCAATTCCGTCGGTACTATGGGCTTAAGTGACTCAAACAACGTTGGGTCGGCAAAACAC
CAGCAACAGTCACCTGCCAACCAGTCTATGCAATCACTCTCTGACTCGCTCTCAGGCTCT
TCTTTGTATTCCAACAGCGTGAGCCTCCCGATGATGGGGCAAGATAAATTCCCCACTGAT
TTGGACCTGGATATTTTCAACGGGAGCTTGGAATGCGATATGGAGTCCATTATCCGCAGT
GAACTCATGGATGCAGATGGGTTGGATTTTAACTTTGATTCCCTCATCTCAGCCCAGAAT
GTGGTCACTCTGAATGTGGGAAACTTCGCTGGTGCTAAGCAAGCGTCATCACAGAGTTGG
GTTCCAGGC
>Gecko_FOXO3a
ATGATCAACCCAGACGGTGGGAAAGGTGGGAAAGCACCACGGAGGCGTGCTGTTTCAATG
GACAACAGCAACAAGTACACGAAAAGCAGAGGACGAGCGGCTAAGAAAAAGGCTGCTCTT
CAAGCTGCCCAGGAAACTACTGAAGACAGCCCCTCCCAGCTCTCCAAATGGCCAGGCAGC
CCAACCTCACGTAGCAGTGATGAGCTGGATGCATGGACGGATTTCCGGTCTCGTACAAAC
TCAAATGCCAGCACAATAAGTGGCCGCTTGTCACCAATATTGGCAAGTACTGAGTTAGAT
GAGGTCCAGGATGATGATGCTCCACTATCACCCATGCTGTACGGTAGCCCACCCAGCATG
TCCCCATCGGTAAATAAACCCTGTAATGTTGAGCTGCCTAGGTTGACTGATATGGCTGGC
ACCATGAACTTGAATGATGGCCTGACAGATAATCTTATGGATGACCTTCTGGATAATATC
ACACTCCCCTCTTCCCAGCAATCACCCACAGGAGGACTCATGCAAAGAAGCTCAAGTTTT
CCTTATGGATCCAAAGGTTCAGGTCTTGGTTCTCCATCAAGCAGTTTCAATAATGCTGTG
TTTGGACCTTCATCTCTGAATTCCCTCCGTCAGTCTCCCATGCAAACCATCCAAGAGAAC
AAGCAAGCTACATTTTCTTCCATGTCTCACTACAACAACCAAACGCTGCAGGATCTGCTA
GCATCGGATTCGCTGAGCCACAGCGATGTCATGATGACTCAGTCTGATCCACTCATGTCA
CAAGCTAGCACAGCTGTGTCTGCCCAAAATGTACGCAGGAGCATTATGCTCCGGAACGAC
CCAATGATGTCATTTGCTGCTCAGCCAAGCCAGGGCAGTTTGGTCAATCAGAACCTGCTC
CACCACCAGCATCAGTCCCAGAATTCTTCTCTCAGTGGCAGCTGTGCCTTGTCAAATTCC
ATTGGTACTATAGGCTTAAGTGACACAAACAACGTAGGGTCGGCCAAACACCAGCAACAG
TCACCTGTCAGCCAGTCTATGCAATCACTTTCTGACTCACTCTCAGGCTCTTTATATTCC
AACAGTGTGAGCCTTCCAGTCATGGGGCATGATAAATTCCCCAGTGATTTGGACCTAGAT
ATTTTCAATGGGAGCCTGGAGTGTGACATGGAGTCCATTATCCGCAGCGAACTCATGGAT
GCAGATGGTTTGGATTTTAACTTTGATTCCCTCATCTCTGCCCAGAATGTTGTCACTCTG
AATGTGGGAAACTTCACTGGTGCTAAGCAAGCTTCATCACAGAGTTGGGTTCCAGGC
>Taiwan_habu_FOXO3a
ATGGGTGGTCAGGCCATTGGCAAGAGCAATGCATCTGAGCGGAAAGCGGCCTCGGAACAA
GTGGGGCAGAACTCAATCAGACACAACTTGTCCCTTCACAGTCGATTCATCAGGGTACAG
AATGAAGGAACTGGGAAAAGCTCGTGGTGGATGATCAACCCCGATGGTGGGAAAGGAGGG
AAAGCACCCCGCAGACGTGCGGTATCAATGGACAACAGCAACAAATACACAAAGAGCCGA
GGACGAGCAGCTAAGAAAAAAGCAGCCCTCCAAGTTGCACAGGAGACAAGTGATGACAGC
CCCGCCCAGCTATCCAAGTGGCCTGGTAGCCCAACCTCCCGTAGCAGTGACGAACTGGAT
GCATGGACAGATTTCCGGTCTCGTACCAACTCGAATGCCAGTACAATAAGTGGCCGCTTG
TCCCCAATATTGGCAAGTACTGAGCTAGATGAGGTGCAGGACGACGATGCTCCACTTTCT
CCCATGTTGTACAGTAGCTCACCCAGCATGTCCCCGTCAGTGAGCAAGCCCTGTAATGTT

GAGCTGCCTAGGTTGACTGACATGGCAGGCACGATGAACTTGAATGATGGCTTGACAGAT
AACCTTATGGATGACCTCCTGGACAATATAACCCTTCCCTCTTCCCAGCAGTCACCCCCA
GGAGGACTCATGCAGAGAAGCTCAAGCTTTCCATACGGCTCCAAAGGTTCAGGGCTTGGT
TCTCCATCCAGTAGTTTCAACAATGCTGTGTTTGGACCATCATCGCTGAATTGTCTCCGT
CAGTCTCCCATGCAAACCATCCAAGAGAACAAGCAAGCTACATTTTCTTCCATTTCGCAC
TACAATAACCAAACTCTGCAGGATCTGCTGGCATCAGATTCACATAGCCACAGTGATGTC
ATGATGACTCAGTCTGATCCGCTCATGTCACAAGCTAGCACTGCTGTGTCTGCCCAAAAC
GCACGCAGGAATATTATGCTTCGGAACGATCCGATGATGTCATTTGCTGCTCAACCAAGT
CAGAGCAGTTTGACCAATCAGAATCTGCTCCACCACCAGCATCAATCCCAGAATTCCTCT
CTCAGTGGCAGCCGTGCCTTGTCAAATTCTGCCGGTACTATGGGCTTAAGTGACACCAAC
ATTGGATCAGCAAAACACCAGCAACAGCCACCTGCCAACCAGTCTATGCAATCACTTTCT
GACTCCCTATCGGGCTCTTCTTTGTATTCCAACAGTGTGAGCCTTCCAGTCATGGGGCAC
GATAAATTCCCCAGTGATTTGGATCTGGATATTTTCAACGGGAGCTTGGAATGCGACATG
GAGTCCATTATCCGCAGTGAACTCATGGATGCAGATGGATTGGATTTTAATTTTGATTCC
CTCATCTCAGCCCAGAACGTTGTTACACTGAATGTGGGAAACTTCACTGGTGCTAAGCAA
GCTTCATCACAGAGTTGGGTTCCAGGC
>green_sea_turtle_FOXO3a
ATGGGGTGGGAATGGGTCTCTTTCTTGCCTTGCATTTTTTCTTGCTCTGTGAAGATGTTT
ACTCTAATAGGCAGTGGCACCCTCTGGCTTTTGCTCTCTACACAACTTCATACGGTCCTG
CTGATACAGAGTCTTTGTAACTCAATCCGGCATAATTTGTCACTTCACAGTCGATTCATC
AGGGTACAGAATGAAGGAACTGGGAAAAGCTCTTGGTGGATGATTAATCCAGATGGTGGA
AAAGGTGGGAAGCCTCCCCGGAGACGTGCTGTTTCAATGGACAATAGCAACAAGTACACA
AAGAGCAGAGGCCGAGCAGCTAAGAAAAAGGCAGCCCTGCAAGCTGCACAAGAAGCTAGC
GAGGACAGCCCATCTCAGCTTTCCAAGTGGCCGGGGAGCCCAACTTCCCGCAGCAGTGAT
GAGCTGGATGCTTGGACAGATTTCCGCTCTCGTACAAATTCAAATGCCAGTACAATAAGT
GGCCGTTTGTCACCGATATTGGCGAGTACCGAACTCGATGACGTTCAGGATGATGATGCT
CCACTTTCTCCAATGCTGTACAATAGTCCATCAAGCATGTCCCCATCGGTAAATAAACCA
TGTACTGTTGAGTTGCCGAGGTTGACTGATATGGCAGGCACAATGAACTTGAATGATGGA
CTGACAGATAACCTCATCATGGATGATCTTTTGGACAATATAACGCTCCCCTCTCCCCAA
CAGTCACCATCAGGGGGGCTCATGCAAAGAAGCTCCAGTTTTCCGTATGGTTCCAAAGGT
TCCGGACTTGGTTCTCCATCAAGTAATTTCAACAGTGCTGTGTTTGGACCATCATCTCTG
AATTCTCTTCGTCAGTCTCCCATGCAAACCATTCAAGAGAACAAGCAAGCTACCTTTTCT
TCCATTTCTCATTATAACAACCAGACGCTGCAGGATCTGCTGGCATCTGACTCACTTAGT
CACAGTGATGTCATGATGACGCAGTCTGATCCACTCATGTCTCAAGCCAGCACAGCTGTG
TCTGCCCAGAATTCACGCAGGAGTATCATGCTTCGTAGTGATCCAATGATGTCATTTGCT
GCTCAGTCCAACCAGGGAAGTTTGGTAAATCAGAACCTGCTCCACCACCAGCATCAATCT
CAGAATTCTTCTCTTGGTGGCAGTCGTGCCTTGTCAAATTCCATCAGTAACATGGGCTTA
AATGATACGAACAACTTGGGGTCGGCCAAACACCAGCAGCAGTCACCTGTCAATCAGTCT
ATGCAAACACTTTCTGACTCGCTCTCAGGCTCTTCTTTGTATTCCACTAGTGTGAACCTT
CCAGTCATGGGGCATGATAAATTCCCAAGTGATTTGGACCTGGATATTTTCAATGGAAGC
TTGGAATGTGACATGGAGTCCATTATCCGCAGTGAACTCATGGATGCAGATGGGTTGGAT
TTTAACTTCGATTCCCTCATCTCAGCTCAGAACGTTGTCAGTCTGAATGTGGGGAGCTTC
ACTGGTGCTAAGCAGGCTTCGTCACAGAGTTGGGTGCCAGGC
>Chinese_alligator_FOXO3a

ATGGATGGGCTTCGCAAGAACTCAATCCGGCATAACCTATCACTTCATAGTCGATTCATC
AGGGTGCAGAATGAAGGAACCGGGAAAAGCTCTTGGTGGATGATCAACCCAGATGGTGGG
AAAGGAGGGAAGGCCCCAAGGAGACGTGCTGTTTCCATGGACAATAGCAACAAGTACACT
AAGAGCAGGGGGCGAGCAGCTAAGAAGAAGGCAACCCAACAAGCCGCACAAGAAGTGAGT
GATGACAGCCCTTCTCAGCTTTCCAAGTGGCCAGGGAGCCCAACATCCCGTAGCAGCGAT
GAGTTGGATGCCTGGACAGATTTTCGATCTCGTACAAATTCAAATGCCAGTACGATAAGT
GGCCGCTTGTCACCCATATTGGCAAGTACTGAACTGGATGATGTTCAGGATGATGATGCT
CCACTGTCCCCAATGCTGTACAATAGTCCATCAAGCTTGTCCCCATCAGTAAATAAGCCG
TGTACAGTTGAGTTGCCTCGGCTAACCGATATGGCAGGCACAATGAACTTAAACGATGGA
CTGACGGATAATCTCATGGATGATCTTTTGGATAATATAACGCTCCCCTCTTCCCAGCAG
TCACCTTCTGGGGGGCTGATGCAAAGAAGCTCAAGTTTTCCGTATGGCTCCAAAGGTTCG
GGTCTTGGTTCTCCATCCAGTAGTTTCAACAATGCTGTGTTTGGACCATCCTCTCTGAAT
TCCCTCCGCCAGTCTCCCATGCAAACTATACAAGAGAACAAGCAAGCTACCTTTTCCTCC
ATTTCTCATTACAACAACCAGACGCTTCAGGACCTGCTGGCATCTGATTCGCCTAGTCAC
AGTGATGTCATGATGACGCAGTCGGATCCGCTCATGTCACAAGCCAGCACTGCTGTGTCT
GCCCAAAACTCACGCAGGAATATAATGCTTCGTAATGACCCAATGATGTCATTTGCTGCT
CAGTCCAGCCAGGGAAGTTTGGTCAGTCAAAACCTGCTCCACCACCAGCATCCATCTCCG
AATTCTCCTCTTAGTGGCAGCCGTGCCTTGTCGAATTCAGTCAGTAACATGGGCTTAAAT
GACACGAACAGCTTGGGGTCAGCCAAACACCAGCAGCAGTCACCTGTCAATCAGTCTATG
CAAACACTTTCTGATTCACTCTCAGGCTCTTCTTTGTATTCTGCTGGTGTGAACCTTCCA
GTCATGGGACATGATAAATTCCCAAGTGATTTGGACCTGGATATTTTCAATGGGAGCTTG
GAATGTGACATGGAGTCCATTATCCGCAGTGAGCTCATGGATGCAGACGGGTTGGATTTT
AATTTTGATTCCCTCATCTCAGCTCAGAACGTTGTCACTCTAAATGTGGGGAACTTCACT
GGTGCTAAACAGGCTTCATCACAGAGTTGGGTGCCTGGC
>Gharial_FOXO3a
ATGGCAGAGGCGTCGCCGCCCGCGCCCCCGTCACCACTGGACGTGGAGCTGGACCCGGAG
TTCGAGCCCCAGAGCCGGCCGCGCTCCTGCACTTGGCCGCTGCAGAGGCCCGAGCTGCAA
GGCAGCCCGGCCAAGCCCGCGGGGGAGGCGGCCGCCGACGCCGCCTCAGGGCCGGGGGCC
GCCGCCGCCTCCTCCTCGGCCCCGCGCAAGTGCTCGTCGCGGCGCAACGCGTGGGGCAAC
CTGTCGTACGCCGACCTGATCACCCGCGCCATCGAGAGCGCCCCGGACAAGCGCCTCACG
CTCTCGCAGATCTACGACTGGATGGTGCGCTGCGTGCCCTACTTCAAGGATAAGGGCGAC
AGCAACAGCTCGGCCGGCTGGAAGAATTCAATCCGGCATAACCTATCACTTCATAGTCGA
TTCATCAGAGTGCAGAATGAAGGAACCGGGAAAAGCTCTTGGTGGATGATCAACCCAGAT
GGTGGGAAAGGAGGCAAGGCCCCAAGGAGACGTGCTGTTTCCATGGACAATAGCAACAAG
TACACTAAGAGCAGGGGGCGGGCCGCTAAGAAGAAGGCAACTCAACAAGCCGCACAAGAA
GTGAGTGATGACAGCCCTTCTCAGCTTTCCAAGTGGCCAGGGAGCCCAACATCCCGTAGC
AGTGATGAGTTGGATGCCTGGACAGATTTCCGATCTCGTACAAATTCAAATGCTAGTACG
ATAAGTGGCCGCTTGTCACCCATATTGGCAAGTACTGAACTGGATGATGTTCAGGATGAT
GACGCTCCACTTTCCCCAATGCTGTACAGTAGTCCATCGAGCTTGTCCCCATCAGTAAAT
AAGCCGTGTACTGTTGAGTTGCCTCGGCTAACCGATATGGCAGGCACAATGAACTTAAAC
GATGGACTGACAGATAATCTCATGGATGATCTTCTGGACAATATAACGCTCCCCTCTTCC
CAGCAGTCACCTTCTGGGGGGCTGATGCAAAGAAGCTCCAGTTTTCCATATGGCTCCAAA
GGTTCGGGTCTTGGTTCTCCATCCAGTAGTTTCAACAATGCTGTGTTTGGACCATCCTCT
CTGAATTCCCTCCGCCAGTCTCCCATGCAAACTATACAAGAGAACAAGCAAGCTACCTTT

TCCTCCATTTCTCATTACAACAACCAGACACTTCAGGACCTGCTGGCATCTGATTCACCT
AGTCACAGTGATGTCATGATGACGCAGTCAGATCCGCTCATGTCACAAGCCAGTACTGCT
GTGTCTGCCCAAAGCTCACGCAGGAATATAATGCTTCGTAATGACCCAATGATGTCATTT
GCTGCTCAGTCCAGCCAGGGAAGTTTGGTCAATCAGAACCTGCTCCACCACCAGCATCCA
TCTCCAAATTCTCCTCTTAGTGGCAGTCGTGCCTTGTCGAATTCAGTCAGTAACATGGGC
TTAAATGACACGAACAGCTTGGGGTCAGCCAAACACCAGCAGCAGTCACCTGTCAATCAG
TCTATGCAAACACTTTCTGATTCACTCTCAGGCTCTTCTTTGTATTCTGCTGGTGTGAAC
CTTCCAGTCATGGGACATGATAAATTCCCAAGTGATTTGGACCTGGATATTTTCAATGGG
AGCTTGGAATGTGACATGGACTCCATTATCCGCAGTGAGCTCATGGATGCAGACGGGTTG
GATTTTAATTTTGATTCCCTCATCTCAGCTCAGAACGTTGTCACTCTAAATGTGGGGAAC
TTCACTGGTGCTAAACAGGCTTCATCACAGAGTTGGGTGCCTGGC
>African_clawed_frog_FOXO3a
ATGGCAGAAGCACTGCCTCCCCGCTCCCCGCCTGACGACGTGGACATAGACCCGGACTTC
GGGCCGCAGAGTCGACCTCGGTCTTGCACGTGGCCCCTGCAGAGACTAGACTCCCAAGGC
AGCCCGGGCAAGCCGAATAGTGGCGCTGGGGAAGCCGCAGACACGTCCTCTATGATCCCG
GAGGAGGAGGATGATGACTATGAGGGAGCTGCTAGTACAGCCACTGTGCTGGGGACAGCG
GGGGACAAGGGCACGTTGGTGCTGCTGAGCGGCGGAGAATCGGGACAACTGGCCGTGCTG
GCGTCACCTGTTGGGGGGGTGGAAACGCTGCAGGTTTCGCTCGGGGGTGAAGGAGCAGGG
GGCGCCGTGAGCGGTGCAGGAGGACAGCAGCAGCAGAGGAAATGTTCATCCCGCAGAAAT
GCCTGGGGCAACATGTCCTACGCTGACCTTATCACTAGGGCCATCGAGAGCACCCAGGAT
AAACGGCTCACCCTGTCCCAGATCTATGATTGGATGGTCCGCTCCGTGCCCTACTTCAAG
GATAAGGGAGACAGCAACAGCTCTGCAGGCTGGAAGAATTCAATCCGGCATAATCTGTCA
CTACATAGCAGGTTTATTAGAGTTCAGAATGAGGGCTCTGGAAAAAGCTCTTGGTGGATG
ATCAACCCAGAGGGTGGTAAAGGTGGAAAGGCACCAAGAAGACGTGCTGTTTCAATGGAC
AATAGTAACAAGTACACCAAGAGTCGCGGGAGAGCAGCAAAGAAAAAAGCATCCTTGCAG
GCATCTTCTGATGCTACAGATGATAGCCCTTCACAGCTGTCAAAGTGGCCAGGTAGTCCC
ACCTCACGTAGTAGTGATAAACTTGATACCTGGACAGACTTTCGATCTCGTACAAACTCT
AATGCCAGTACTATAAGTGGTCGGTTGTCTCCAATTCCAGCTACCACTGAACTTGATGAT
GTTCAAGATGATGATTCTCCCCTGTCCCCTATGTTGTATAACAGCCCAGGTAGTTTGTCC
CCATCCATAAGTAAACCATGCACAGTGGAGATGCCAAGAATAACTGATATGGCTGAAACC
ATGAACTTAAATGACGGATTGCCGGAGAACCTCATGGATGACTTGCTGGATGACATTTCT
CTCACTTCTTCACAGCAATCATCTCCCGGTGTCCTCATGCAGAGAAGTTCCAGCTTTACA
TATGGCACTAAGGGCTCAGGCATTGGCTCTCCATCGAATAATTTTAACAACACTGGTAGC
TTCAACTTTCCCTTGACATCTCTACGCCAGTCTCCCATGCAAACGATCCAAGAGAACAAA
CAAGCTACATTTTCTTCCATGAATCATTATAGTAACCAGTCTTTGCAAGACCTGTTGAAT
ACAGACACCCCTCAGCCACAGCGATGTGTTAATGACCCAGTCTGATCCGCTCATGTCACAA
GCCAGTACTGCAGTAACTGCTCAGAATAGCAGAAGAAATATCATTCTGAGAAATGATCCT
ATGATGTCTTTTGCAGCCCAACCTAACCAAGGAGGAAACTTGGTTAATCAAAACTCGCTG
CACCAGCAGCAGTCTCTTAACTCTTTTCAAGGTGGCAGCCGTGCCTTGTCAAACAATTTA
AGCAACACTGGTTTAAATGACAGCAGCATCTTGGAATCAACCAAACACCAGCAGCAGTCC
TCAGTGAGTCATTCTATGCAAACCATCTCCGACACGCTCTCAGGATCCTTGTACTCCTCA
GGCGTGACCCTTCCAACACTGGGACATGAAAAGTTTCCCACTGATTTGGACCTGGATATT
TTCAATGGGAGTTTGGAATGTGATATGGAGACGATAATTCGCAACGATCTCATGGATGCA
GATGGGTTGGATTTTAATTTTGACACCCTCATCTCAGCTCAGAATGTCAGTCTATCTGTA

GGCAGTTTCACTGGTGCTAAGCAGACATCATCACAGAGCTGGGTGCCAGGC
>Tibetan_frog_FOXO3a
ATGGCAGAAGCTGACCCCCCGGTGTCCCCTGCCGGGAACGTGGAGATAGACCCGGACTTT
GAGCCGCAGAGCCGGCCCAGATCGTGCACATGGCCCCTGCAGAGACTGGACTCCCAAGCC
AGCCCGGCCAAGCCCGGTGTAGGCGGGGAGCCGGGGGACGCAGCCATTGAGAGCTCCCCG
GACAAGAGGCTCACCCTGTCCCAGATCTATGACTGGATGGTCCGCTCCGTGCCCTACTTC
AAGGACAAGGGGGACAGCAACAGCTCTGCGGGGTGGAAGAATTCAATTCGACATAATTTG
TCCCTGCACAGCAGATTTATTCGAGTACAAAACGAGGGCACTGGAAAAAGCTCTTGGTGG
ATGATTAATCCAGAAGGAGGGAAAGGTGGGAAGGCACCTAGAAGACGTGCCGTGTCAATG
GACAACAGCAACAAATACACTAAGAGCCGTGGAAGAGCAGCAAAGAAAAAAGCATCAATG
CAGGTGACCCAGGATGCTGCAGATGATAGCCCATCGCAACTCAAATGGCCAGGAAGCCCA
ACATCGCGTAGCAGTGATGAGTTGGATGCTTGGACAGATTTCCGCTCCCGTACGAACTCG
AACGCCAGCACGATAAGTGGTCGTTTATCTCCAATTCCAGCAACAACTGAGCTTGATGAT
GTTCAGGATGATGATTCACCTATTTCCCCAATGCTGTATAGCCCTGGCAGTATGTCTCCA
TCCATAACAAAACCATCCACAGTGGAGTTGCCTGGGATAACAGATATGACTGGAACCATG
AACTTAAATGATGGTCTAACAGAGAATCTTATTGATGATTTTCTGGAGGACATTTCTCTT
ACGCCATCTCAGCAGTCATCTCCTAGTGTTGGTGGCATGCAGAGAAGTTCCAGCTTTACC
TATGGCACTAAGGGCTCAAGCCTTGGCTCTCCATCCAGTAGCTTCAACAACACTAGTAGC
TTCAATTTCCCATTGACGTCTCTTCGTCAGTCTCCAATGCAAACCATTCAAGAGAACAAA
CAAGCAACATTTTCTTCCATGAACCATTATAGCAACCAGTCCATTCAGGATATGCTGAAT
ACTGATTCACTTAGCCATAGTGGCGTATTAATGACTCAGTCTGACCCCCTCATGTCCCAA
GCCAGTACTGCAGTCACTGCTCAGAATTCTCGTAGAAACATTATTCTGAGGAGTGACCCA
ATGATGTCGTTTGCAGCCCAGCCCAATCAAGGGGGAAGCCTGGTTAATCAGAACTTGCTC
CACCACCAGCACCAGTCCCATAACTCATTTCTTGGTGGCAGTCGTGCCTTGTCAAACAGC
ATAAATAACATTGGATTAAATGACAGCAACAACTTAGACTCGTCTAAACACCAGCAGCAG
TCCTCAGTCACTCATTCTATGCAAGCCCTCTCAGACACACTCTCAGGACCCTTGTACTCC
ACAGGCATGAACCTTCCAGTGCATGAAAAGTTCCCAACTGATTTGGACCTGGAAATTTTC
AGTGGGAGCTTGGAATGTGACATGGAGACTATCATTCGCAATGAACTAATGGATGCAGAT
GGGCTGGATTTTAACTTTGATTCTCTTATCTCTGCTCAGAATGTCAGTCTTGCAGTGGGA
AATTTCACAGGTGCTAAGCAGACATCGTCACAGAGTTGGGTACCGGGT
>southern_platyfish_FOXO3a
ATGGCCGAGGCTCCGCTCCCCGACACGCTCCCGGACTTGGACGTGGTCATTGACCCGGAC
TTTGAGCCCCAGAAGCGGCCCCGGTCTTGCACCTGGCCGCTGCCGCGACCGGACTCCAGC
GCGGTGAAACCGGAGAGCACCGAGGCGGATATCATACCCGAGGAGGAGGATGACGAGGAG
GACAGCGCAACCCCCACGGCAATCACTGTCAATGGCTCCGCTGCGGCGACGGAGGACCAG
AGCAGTAACAGCCCCATTACCGACGGTGCGTTCCCCTCCCCCGGCCATGACAGCGGAGGC
TCCCCGCTCTCCACGCACTCACCGACGGCCACCTCCGGCGCCCTGACTCCGAGCGGCTTG
CCTGCAGCGCAGACCCCGAGGAAGGCATCATCCCGCCGTAACGCCTGGGGCAACCTCTCC
TACGCCGACCTGATCACCAAAGCCATCGAGAGCTCACCAGAGAAGAGACTGACTCTGTCC
CAGATCTATGACTGGATGGTGAGATCCATCCCCTACTTTAAGGACAAAGGCGACAGCAAC
AGCTCTGCAGGATGGAAGAACTCCATCCGGCACAACCTATCTCTCCACAGTCGTTTCATC
CGTGTCCAGAATGAAGGGACAGGAAAGAGTTCGTGGTGGATGATCAACCCAGAGGGAGGA
AAAGGAGGCAAGGCTCCACGGCGCCGGGCCGTCTCCATGGATAACAGCAACAAGTACACC
AAGTCTGCTCGTGGCCGTGCTGCTGCTAAGAAGAAGGCGGCCCTGCAGGCTGTGGCTGCT

GCAGCTGGGGAAGGCGGTGGAGACAGTCCTACAGGTCCCTCTAAGTGGCCGGGAAGCCCG
ACGTCACGCAGCAGCGAGGAGCTGGATGCTTGGACAGACTTCCGCTCCCGAACAAACTCT
AATGCCAGCACGCTCAGCGGCCGCCTCTCGCCAATTTTAGCGAATCCTGAGCTAGACGAG
GTGGCTGACGATGAGCCTCCTCTCTCACCGATGATCTACTCCAGTCCTGGCAGGGCGTTG
TCTCCAGGAAACACCAATGGGAAGGCTGTGCCCACTGAGCTGCCTCGTCTGGCAGATCTG
GCAAATACAATGAATCTAAATGATGGAATCACACAAGATCTGATGGATGATTTTCTGGAT
AACATCAAGCTTGTACCAACCACCTGCAGCCAAGCCATGCAGAATGGTTCCTCAGGGTTT
AGTTTTGGGTCCAAACCCAACGGGATAGGGTCACCTTCTTCCACTTCATCTCCATCCTCT
AACAACTCCTCTAATGGAGGAGGTAATAGCTACAGTAACTCCATCTTTAGCCCTTACACA
GCAGGCTCCTCCCTACGTCCATCTCCCATGCAAACCATCCAGGAGAATAAGCAAACTTCA
TTTTCCACAACTGGCATGTCTCACTTTGGCAGCCATACACTACAAGATCTTCTTAACTCT
GACAGCCACAATCATAGTGATGTCATGATGACCCAGTCTGACCCCTTAATGTCACAGGCC
AGTGCTGTTGCCATCATTTCCCAGAACTCTCGTCGAAATGTTATGGTCCGTAATGATCCT
GTTATGACTTTTGGGACCACCGGTGGACTCCAGAGTAGCCATAGGGAGACGCTCCAAACT
AACAACCACAACCAGAGTACCGTGAGGTCTTTGAACGGAGATCTGAACCTTGCCAACGAG
GCAAACACTTTGGCTAATGTCAAACAGCAACTTCTACTGTCACCAATTGGTGGAAATGGA
ACATCCTCCATGCAAATTGACACCTCCATCTTTCTAAATGGTACAGCAAGCAGCAGCGGC
GGGTGTCAGGACCGCTTCCCAACAGACTTGGACCTGGACATGTTCAACAGTGGCAGCTTG
GATTGTGACATGGAGTCCATCATTAGGAATGATCTGATGGATGCTGATGGTCTGGATTTT
AATTTTGAGTCACTGGCCAACATGAATGGAGTCAGCAACTTCACAAGCACCAAGCACAGC
TGGGTGCCTGGT
>spotted_gar_FOXO3a
ATGGCAGAAGCACCTCCCCCTGACCCTCTTTCCCCGCTGGACGTTGAGATAGACCCTGAT
TTCGAGCCACAGAAGAGGCCAAGGTCCTGCACCTGGCCTTTACCCCGACCTGACCTGCAG
TCCAATGTGGAGAAGCCCGAAGCCACTGACACGGACATTATCCCGGAGGAAGAGGACGAT
GAGGACGACACTGCCAATTCTATGACCATCAATGCCAATGGCACCCTGAGCAACACCAGC
ACGACAAGGGAAGACCAAAACAGTCCAAGCGCCCTCTTAGTGGACGCGATAGGTGCAGCG
GCGACTGGCCAGGAGAACGCAGGGTCCCCGCTCTCGTCCCAGTCCCCGGGCACGGCTGGA
AGCGGCTCCAGCGGCGGCCTGGGGTCCCAGCAGCCGAGGAAGTCCTCCTCACGCCGGAAC
GCCTGGGGCAACCTGTCGTACGCAGACCTGATAACCAAAGCCATAGAGAGCACGCCGGAC
AAGAGGCTCACCTTGTCCCAGATCTACGACTGGATGGTCAGGTGTGTCCCGTACTTTAAG
GATAAAGGCGACAGCAACAGCTCGGCCGGGTGGAAGAATTCCATCCGACACAACTTGTCG
CTGCACAGTCGTTTTATTCGGGTGCAGAACGAGGGCACTGGGAAGAGCTCTTGGTGGATG
ATCAATCCGGATGGTGGGAAGGGTGGCAAGGCCCCGCGCCGACGGGCCGTTTCCATGGAC
AACAGCAACAAGTACAGCAAGAGCCGGGGGCGCGCAGCCAAGAAGAAGGCAGCTTTGCAA
GCAGGGCAAGAGGGGGGTGCGGAGAGTCCCTCCAGCCAGCTCAACAAGTGGCCGGGGAGC
CCTACCTCTCGCAGCAGCGATGAGATGGACCCCTGGACCGACTTCCGCTCGCGCACCAAC
TCCAATGCGAGCACCATCAGCGGACGCCTCTCGCCAATCTTAGCCAACCCTGAGCTGGAC
GAGGTGCCTGACGACGACGCACCGCTGTCCCCCATGCTGTACTCCAGCCCCAGCAGTCTG
TCCCCTTCGGTCAGCAAACCCTGCTCCACAGAGCTCCCCAGGCTGGCAGACTTGGCCGGT
ACTATGAATCTCAATGAAGGGCTCTCGGACAACCTGATGGATGACCTTCTTGACAACATC
AACCTGACCCCCTCCCAGCAACAGTCTCCAGGGAGTGTCAACGGCAGCAATGGGGGTGGC
GGGATCATGCAGAGGAGCTCCAGCTTCACCTACGGCTCCAAAGGCACGGGCCTGGGTTCC
CCTTCTGGCAGCTACCCCAACTCCATCTTCGGCACCCCGTCCCTCACTGGCCTACGCCAG

TCGCCCATGCAGACCATCCAGGAGAACAAGCAGGCCACTTTCTCTTGCATCTCCCACTTT
GGCAACCAAACCCTGCAGGATCTGCTCAGCTCAGACTCGCACAGCCACAGTGACGTCATG
ATGACTCAGTCGGATCCTCTCATGTCGCAGGCCAGCACCTCCGTCTCCTCGCAGAGCTCC
CGCCGGAACATCATGTTGCGCAGTGACCCGATGATGTCCTTCACCGCCTCCCAGTCGAAC
CAGGGAAGCCCTGTGAACCATGGGAGCCTGCTGCACCCTCATCCCCATTCTCAGAACTCG
CTGGGGGGAAGCCGGGCTGGGTTGTCCAGCCTGGTCAACGGGGGCATCGGCCTGGCCAAT
GAGACCAACAGCCTGGTTTCTGCCAAGCACCAGCTCCAGTCTCCCAGTGGCAGTAACCAA
TCTATGCAAATCTGTAGCTCTGACTCTTCCTTATACTCCAGTCTCAGCGAAAGTGGCATC
ACCCTTCCGTCCATGAATCAGGACAAGTTCCCTAGTGACCTTGACCTGGACATGTTTAAT
GGCAGCCTGGAGTGTGATATGGACTCCATCATCCGCAGTGAGCTCATGGATGCTGATGGT
TTGGACTTCAACTTTGATTCGCTCATCTCCGCTCAGAATGTTGTCAACCTTAATGTGGGG
AACTTCACTGGTGCTAAACAGACCTCCTCCCAGAGCTGGGTGCCTGGT
>guppy_FOXO3a
ATGGCCGAAGCTCCGCTCCCCGACACGCTCCCGGACTTGGACGTGGTCATTGACCCGGAC
TTTGAGCCCCAGAAGCGGCCCCGGTCTTGCACCTGGCCGCTGCCGCGACCGGACTCCAGC
GCGGTGAAACCGGAGAGCACCGAGGCGGATATCATACCCGAGGAGGAGGATGACGAGGAG
GACAGCGCAACCCCCACGGCAATCACTGTCAATGGCTCCGCTGCGGCGACGGAGGACCAG
AGCAGTAACAGCCCCGTTACCGACGGTGCGTTCCCCTCCCCCGGCCACGACAGCGGAGGC
TCCCCGCTCTCCACGCACTCACCGACGGCCACCTCCTGTGCCCTGACTCCGAGCGGCTTG
GCTGCAGCGCAGACCCCGAGGAAGGCATCATCCCGCCGCAACGCCTGGGGGAACCTCTCC
TACGCCGACCTGATCACCAAAGCCATCGAGAGCTCACCAGAGAAGAGACTGACCCTGTCC
CAGATCTATGACTGGATGGTGAGATCCATCCCTTACTTTAAGGACAAAGGCGACAGCAAC
AGCTCTGCCGGATGGAAGAACTCCATCCGGCACAACCTGTCTCTCCACAGTCGTTTCATC
CGTGTCCAGAATGAGGGAACAGGAAAGAGTTCGTGGTGGATGATCAACCCAGAGGGAGGA
AAGGGAGGCAAGGCTCCACGGCGCCGGGCCGTCTCCATGGACAACAGCAACAAGTACACC
AAGTCTGCTCGTGGCCGTGCTGCTGCTAAGAAGAAGGCCGCCCTGCAGGCTGTGGCTGCT
GCAGCTGGGGAAGGCGGCGGAGACAGTCCGTCAGGCCCCTCGAAGTGGCCGGGAAGCCCG
ACGTCACGCAGCAGCGAGGAGCTGGATGCTTGGACAGACTTCCGCTCCCGAACCAACTCT
AACGCCAGCACGCTCAGCGGACGCCTCTCGCCAATCTTAGCGAATCCCGAGCTGGACGAG
GTGCCTGACGACGAGCCTCCTCTCTCGCCAATGATCTACTCCAGTCCTGGCAGGGCGTTG
TCTCCTGGAAACACCAACGGGAAGGCCGTGCCCGCTGAGCTGCCTCGTCTGGCAGATCTG
GCAAACACAATGAATCTAAATGATGGGATCGCACAAGATCTGATGGATGATTTTCTGGAT
AACATCAAGCTCGTACCAACCACCTGCAGCCAAGCCATGCAGAATGGTTCCTCAGGGTTT
AGTTTTGGGTCCAAGCCCAACGGGATAGGGTCACCTTCTTCCACTTCATCTCCACCCTCC
AATAACTCCTCTAACGGAGGAGGCAATAGCTACAGTAACTCCATCTTTAGCCCTTACACA
GCAGGCTCCTCCTTACGTCCATCTCCCATGCAGACCATCCAGGAGAATAAGCAAACTTCA
TTTTCCACAACTGGCATGTCTCACTTTGGCAGCCAAACACTACAAGATCTTCTTAACTCT
GACAGCCACAATCACAGTGACGTCATGATGACCCAGTCTGACCCATTAATGTCACAGGCC
AGTGCTGTTGCCATCATTTCCCAGAACTCCCGTCGAAATGTTATGGTCCGTAACGATCCT
GTCATGACTTTTGGGACCACTGGTGGACTCCAGAGTAGCCATAGGGAGATGCTCCAAACT
AACAACCACAACCAGAGCACCATGAGGTCTTTGAACGGAGATCTGAACCTCGCCAACGAG
GCAAACACTCTGGCTAGCGTCAAACAACAACTTCTACTGTCACCAATTGGTGGAAATGGA
ACGTCCTCCATGCAGATTGACACCTCCATCTTTCTAAATGGCACGGCGAGCAGCAGCGGC
GGGTGTCAGGACCGCTTCCCGACAGACCTGGACCTGGATATGTTCAACAGCGGCAGCTTG

GAGTGTGACATGGAGTCCATCATTAGGAATGATCTGATGGATGCTGATGGTCTGGATTTT
AATTTTGAGTCACTGGCCAACATGAACGGAGTCAGCAACTTCACAACCACCAAGCAGAGC
TGGGTGCCTGGT
>American_alligator_FOXO3a
ATGGCAGAGGCGTCGCCGCCCGCGCCCCCGTCGCCGCTGGACGTGGAGCTGGACCCCGAG
TTCGAGCCCCAGAGCCGGCCGCGCTCCTGCACTTGGCCGCTGCAGAGGCCCGAGCTGCAA
GGCAGCCCGTCCAAGCCCGCGGGGGAGGCGGCCGCCGACGCCGCCTCCATGATCCCCGAG
GAGGACGACGACGACGAGGACGGGGGCGGCTCGGCCATGGCCATCGGCGGCGCCGGGCCC
AGCGGCGGGGACGCGGCGCTGGTGCNNNNNNNNNNGGGCGCGGGGGCCGCCGCCACCGCC
TCTTCGGCCCCGCGCAAGTGTTCGTCGCGGCGCAACGCGTGGGGCAACCTGTCGTACGCC
GACCTGATCACCCGCGCCATCGAGAGCGCCCCGGACAAGCGCCTCACGCTCTCGCAGATC
TACGACTGGATGGTGCGCTGCGTGCCCTACTTCAAGGATAAGGGCGACAGCAACAGCTCG
GCCGGCTGGAAGAACTCAATCCGGCATAACCTATCACTTCATAGTCGATTCATCAGGGTG
CAGAATGAAGGAACCGGGAAAAGCTCTTGGTGGATGATCAACCCAGATGGTGGGAAAGGA
GGGAAGGCCCCAAGGAGACGTGCTGTTTCCATGGACAATAGCAACAAGTACACTAAGAGC
AGGGGGCGAGCAGCTAAGAAGAAGGCAACCCAACAAGCCGCACAAGAATTGAGTGATGAC
AGCCCTTCTCAGCTTTCCAAGTGGCCAGGGAGCCCAACATCCCGTAGCAGCGATGAGTTG
GATGCCTGGACAGATTTTCGATCTCGTACAAATTCAAATGCCAGTACAATAAGTGGCCGC
TTGTCACCCATATTGGCAAGTACTGAACTGGATGATGTTCAGGATGATGATGCTCCACTT
TCCCCAATGCTGTACAATAGTCCATCAAGCTTGTCCCCATCAGTAAATAAGCCGTGTACA
GTTGAGTTGCCTCGGCTAACCGATATGGCAGGCACAATGAACTTAAACGATGGACTGACG
GATAATCTCATGGATGATCTTTTGGACAATATAACGCTCCCCTCTTCCCAGCAGTCACCT
TCTGGGGGGCTGATGCAAAGAAGCTCCAGTTTTCCATATGGCTCCAAAGGTTCGGGTCTT
GGTTCTCCATCCAGTAGTTTCAACAATGCTGTGTTTGGACCATCCTCTCTGAATTCCCTC
CGCCAGTCTCCCATGCAAACTATACAAGAGAACAAGCAAGCTACCTTTTCCTCCATTTCT
CATTACAACAACCAGACGCTTCAGGACCTGCTGGCATCTGATTCGCCTAGTCACAGTGAT
GTCATGATGACGCAGTCAGATCCGCTCATGTCACAAGCCAGCACTGCTGTGTCTGCCCAA
AACTCACGCAGGAATATAATGCTTCGTAATGACCCAATGATGTCATTTGCTGCTCAGTCC
AACCAGGGAAGTTTGGTCAGTCAAAACCTGCTCCACCACCAGCATCCATCTCCGAATTCT
CCTCTTAGTGGCAGCCGTGCCTTGTCGAATTCAGTCAGTAACATGGGCTTAAATGACACG
AACAGCTTGGGGTCAGCCAAACACCAGCAGCAGTCACCTGTCAATCAGTCTATGCAAACA
CTTTCTGATTCACTCTCAGGCTCTTCTTTGTATTCTGCTGGTGTGAACCTTCCAGTCATG
GGACATGATAAATTCCCAAGTGATTTGGACCTGGATATTTTCAATGGGAGCTTGGAATGT
GACATGGAGTCCATTATCCGCAGTGAGCTCATGGATGCAGATGGGTTGGATTTTAATTTT
GATTCCCTCATCTCAGCTCAGAACGTTGTCACTCTAAATGTGGGGAACTTCACTGGTGCT
AAACAGGCTTCATCACAGAGTTGGGTGCCTGGC
>Armadillo_FOXO3a
ATGGCAGAGGCACCGGCCTCCCCGGCCCCACTGTCTCCGCTCGAAGTGGAGCTGGACCCC
GAATTCGAGCCTCAGAGCCGGCCACGCTCTTGTACATGGCCCTTGCAGAGGCCGGAGCTC
CAGGCGAGCCCGGCCAAGCCCTCGGGGGAGGTGGCCGCCGACTCCATGATCCCCGAGGAG
GAGGACGATGAAGACGACGAGGACGGCGGCGGCGGGGCCGGCTCGGCCATGGCGATCGGC
GGCGTTGGGAGCGGCACGCTGGGCTCCGGGCTGCTCCTTGAGGACTCGGCCCGGCTCCTG
GTTCCCGGCGGGCAGGACCCCGGGTCCGGGCCAGCCCCAGCAGCGGGTGCGCTGAGCGGG
GGAACGCCGACGCCGCTGCAGCCTCAGCAGCCGCTGCCACTGCCGCAGCCGGGGGCGGCT

GGGGGCTCCGGGCAGCCGAGGAAATGCTCGTCGCGGCGGAACGCGTGGGGGAACATGTCC
TACGCCGACCTAATCACCCGCGCCATCGAGAGCTCCCCGGACAAACGGCTCACTCTGTCC
CAGATCTACGAGTGGATGGTTCGCTGCGTGCCCTACTTCAAGGATAAGGGTGACAGCAAC
AGCTCTGCGGGCTGGAAGAACTCCATCCGGCATAACCTGTCCCTGCACAGCCGGTTCATG
CGGGTTCAGAATGAAGGGACTGGCAAGAGTTCTTGGTGGATCATAAACCCTGATGGGGGG
AAGAGTGGAAAGGCACCCCGGCGGCGGGCGGTCTCCATGGACAACAGCAACAAGTACACC
AAGAGCCGTGGCCGTGCAGCCAAGAAGAAGGCAGCCCTGCAGACAGCCCCTGAGTCGGCA
GACGACAGTCCCTCCCAGCTCTCCAAATGGCCTGGCAGCCCCACCTCCCGCAGCAGCGAT
GAGTTGGATGCATGGACGGACTTCCGCTCGCGCACCAATTCCAACGCCAGCACGGTCAGC
GGCCGCCTGTCACCCATCCTGGCAAGCACAGAGTTGGATGACGTCCAAGACGATGACTCA
CCGCTCTCCCCCATGCTGTACAGCAGTTCAGCCAGCCTCTCACCCTCTGTAAGTAAGCCG
TGCACCGTGGAGCTGCCGCGGCTGACCGACATGGCAGGCACCATGAATCTGAACGATGGG
CTGGCTGACAACCTCATGGATGACCTGCTGGACAACATCGCCCTCCCATCGTCCCAGCCG
TCGCCCACTGGGGGGCTCATGCAGCGCAGCTCCAGCTTCCCTTACACCACCAAGGGCTCT
GGCCTGGGCTCTCCCACCAGCTCCTTTAACAGCACGGTGTTTGGACCCTCGTCTCTGAAT
TCCCTGCGTCAGTCTCCCATGCAGACCATCCAAGAGAACAAGCCAGCCACCTTCTCTTCC
ATGTCACACTATGGCAACCAGACACTCCAGGACCTGCTCACTTCCGACTCGCTCAGCCAC
AGCGATGTCATGATGACCCAGTCGGACCCCTTGATGTCTCAGGCCAGCACTGCTGTGTCT
GCCCAAAACTCGCGCCGGAACGTGATGCTTCGCAATGACCCGATGATGTCCTTTGCTGCC
CAACCCAACCAGGGGAGTTTGGTCAATCAGAACTTGCTCCACCACCAGCACCAAACCCAG
AACGCAATCGGTGGCAGCCGTGCCTTGTCGAATTCCGTCAGCAACATGGGCTTGAGCGAC
TCCAGCAGCCTTGGGTCCACCAAACACCAGCAGCAGTCTCCTGTCAGCCAGTCTATGCAA
ACCCTCTCGGACTCTCTCTCAGGCTCCTCCTTGTACTCAACTAGTGCAAACCTTCCTGTC
ATGGGCCATGAGAAGTTCCCCAGCGACTTGGACCTGGACATGTTCAATGGGAGCTTGGAA
TGTGACATGGAGTCCATTATCCGTAGTGAACTCATGGATGCTGATGGGTTGGATTTTAAC
TTTGATTCCCTCATCTCCACACAGAATGTTGTTGGTTTGAACGTGGGGAACTTCACTGGT
GCTAAGCAGGCCTCATCTCAGAGCTGGGTGCCAGGC
>Chicken_FOXO3a
ATGGCAGAGGCGTCGCCCCCCGGGCCGCTCTCGCCCCTGGACGTGGAGCTGGACCCCGAG
TTCGAGCCGCAGAGCCGCCCCCGCTCCTGCACCTGGCCGCTGCAGAGGCCCGAGCTGCAG
GCCAGCCCCGCCAAGCCCGCCGGCGAGCCGCCCGCCGACGCCGCCTCCATGATCCCCGAG
GAGGAGGACGACGACGAGGAGGGGGGCGGCGCGGCCATGGCCGTCGGCGGCGCGGCCCCC
GCGGGCGGCGAAGCGGCGGCCGCCGGCGGCCCCGAGGAGGCGGCGCGGCCGCCGGTCCCG
CTGCCCGGAGGCGGCCCGGAGGGGCCCGGGCCGGCGCCGGGAGGAGCGGCGGCGGCGGCG
GGCGGCGGGGGGTTGAGCGGCGGCGGCCCCGCGGCGGCGGCGCCGAGGAAGTGCTCGTCG
CGGCGGAACGCGTGGGGGAACCTCTCGTACGCCGACCTCATCACCAAAGCCATCGAGAGC
GCCCCCGAGAAGCGGCTCACCCTCTCGCAGATCTACGACTGGATGGTGCGGTGCGTGCCC
TACTTCAAGGACAAGGGCGACAACAACAGCTCGGCCGGATGGAAGAATTCAATCCGGCAT
AACTTGTCGCTCCACAGCCGATTCATCAGGGTGCAGAACGAAGGCACTGGGAAGAGCTCC
TGGTGGATGATCAATCCAGACGGTGGAAAAGTTGGCAAGGCGCCCCGGAGACGCGCCGTG
TCCATGGACAACAGCAACAAGTACACAAAGAGCAGGGGGCGGGCGGCGAAGAAAAAGGCA
GCCCTGCAGACTGCCCAGGAGGCGAGCGAGGACAGCCCTTCGCAGCTCTCCAAGTGGCCG
GGGAGCCCGACTTCCCGCAGCAGTGACGAGCTGGATGCCTGGACAGATTTTCGCTCCCGG
ACAAATTCGAACGCCAGTACAATAAGTGGCCGCTTGTCACCGATTTTGGCGAGCACCGAG

CTGGATGATGTTCAAGATGACGACGCTCCACTTTCTCCCATGCTGTACAGTAGTCCTTCG
AGCTTGTCCCCCTCGGTAAACAAACCGTGCACTGTGGAGTTGCCTAGGTTGACTGATATG
GCTGGGACAATGAATTTGAACGATGGACTGACAGATAACCTCATGGATGACCTCTTGGAC
AATATAACACTCCCTTCCTCCCAGCAGTCGCCCACAGGAGGGATGATGCAGAGAAGCTCC
AGTTTTCCGTATGGTTCCAAAGGTTCAGGGCTGGGCTCCCCCTCGAGTAGTTTCAACAAC
GCTGTGTTTGGGCCATCGTCCCTGAATTCCCTCCGCCAGTCGCCCATGCAGACTATTCAG
GAGAACAAGCAGGCTACCTTTTCTTCCATTTCTCATTACAACAACCAGACGCTGCAGGAT
CTGCTCGCCTCGGATGCACTTAGTCACAGCGATGTCATGATGACACAGTCTGACCCGCTT
ATGTCCCAGGCCAGCACAGCTGTGTCCGCCCAGAACTCCCGCAGGAATATAATGCTCCGC
AATGACCCCATGATGTCGTTTGCTGCACAGTCCAGTCAGGGCGGCCTGGTCAACCAGAGC
CTGCCCCATCACCAGCACCAGTCTCACAGCTCTCCTCTTAGCGGCAGCCGTGCCTTGTCC
AATTCCATCAGTAACATAGGCTTGAATGACTCCAACAGCTTGGGATCCAAACATCAGCAG
TCTCCTGTCAATCAGTCTATGCAAACACTTTCTGACCCTCTCTCAGGCTCCTCTTTGTAT
TCCTCTAGCATGAACCTTCCGGTCATGGGACACGAGAAATTCCCAAGTGACTTAGACCTG
GATATTTTCAATGGGAGCTTGGAGTGTGACATGGAGTCCATCATCCGCAGTGAACTCATG
GATGCAGATGGGCTGGATTTTAACTTTGATTCCCTCATCTCAGCACAGAACGTTGTCAGT
CTGAATGTGGGGAACTTCACTGGTGCTAAACAGGCTTCATCACAGAGTTGGGTACCAGGC

>Chinese_softshell_turtle_FOXO3a
ATGGCCATAGGCAGCGCCGTGCCCACCGGCGGCGGAGGGGAAGCGCTGGCCCCCGAGGAG
GCGGCCCGGCTGCTGGCCCCGCTCTCCGGGGTTGGGACGGAGGGCTCGGGCAAGGCTTCT
GGGCCAGCGGCTGCGGGGGGCAGCGGGCTGAGCGGGGGCCAGGCAGCTGCGGCGCCGCGG
AAATGCTCGTCGCGGCGAAACGCGTGGGGCAACCTGTCCTACGCCGACCTGATCACCCGC
GCCATCGAGAGCGCCCCGGACAAGCGGCTCACCCTGTCCCAGATCTACGACTGGATGGTG
CGCTGCGTGCCCTACTTCAAGGATAAGGGCGACAGCAACAGCTCGGCCGGCTGGAAGAAC
TCGATCCGGCATAACTTGTCACTGCACAGTCGATTCATCAGGGTACAGAACGAAGGAACT
GGGAAAAGCTCGTGGTGGATGATTAACCCAGATGGCGGGAAAGGTGGGAAGCCCCCACGG
AGACGCGCCGTTTCAATGGACAACAGCAACAAGTACACAAAGAGCAGAGGCCGAGCAGCG
AAGAAAAAGGCAGCCCTGCAAGCTGCACAAGAAGCCAGCGAGGATAGCCCCTCTCAGCTT
TCCAAGTGGCCGGGGAGCCCGACTTCCCGCAGCAGCGATGAGCTGGATGCATGGACCGAT
TTCCGCTCCCGTACAAATTCAAACGCTAGTACAATAAGTGGTCGTTTGTCACCTATCTTG
GCGAGTACCGAACTAGATGAAGTTCAGGATGATGACGCTCCACTTTCTCCAATGCTGTAC
AGTAGCCCATCAAGCATGTCCCCATCGGTAAATAAACCATGTACTGTTGAGTTGCCTAGG
TTGACTGATATGGCAGGCACAATGAACTTGAATGATGGACTGACAGATAACCTCATCATG
GATGATCTTTTGGACAATATGACGCTCCCCTCTTCCCAGCAGTCACCATCAGGGGGGCTC
ATGCAAAGAAGCTCCAGTTTTCCATATGGTTCCAAAGGTTCAGGCCTTGGTTCTCCGTCA
AGTAATTTCAACAGTGCTGTGTTTGGCCCATCATCTCTGAATTCCCTTCGTCAGTCTCCC
ATGCAAACCATTCAAGAGAACAAGCAAGCTACTTTTTCTTCCATTTCTCATTATAACAGC
CAGACGCTGCAGGATCTCCTGGCATCTGACTCACTTAGTCACAGTGATGTCATGATGACG
CAGTCTGATCCACTCATGTCTCAAGCCAGCACAGCTGTGTCTGCCCAGAACTCGCGCAGG
AGTATCATGCTTCGTAGTGATCCAATGATGTCATTTGCTGCTCAGTCCAACCAGGGAAGT
TTGGTCAATCAGAACCTGCTCCACCACCAGCATCAATCTCAGAATTCTTCTCTTGGTGGC
AGTCGTGCCTTGTCTAATTCCATCAGTAACATGGGCTTAAATGATACAAACAGCTTGGGG
TCGGCCAAACACCAGCAGCAGTCACCTGTCAATCAGTCTATGCAAACACTTTCTGACTCA

CTCTCAGGCTCTTCTTTGTATTCCACTAGTATGAACCTTCCAGTCATGGGACACGATAAA
TTCCCAAGCGATTTGGACCTGGATATTTTCAATGGAAGCTTGGAATGTGACATGGAGTCC
ATTATCCGCAGCGAACTCATGGATGCAGATGGGTTGGATTTTAACTTCGATTCCCTCATC
TCTGCTCAGAACGTTGTCAGTCTGAATGTGGGAAGTTTCACTGGTGCTAAGCAGGCTTCG
TCACAGAGTTGGGTGCCAGGC
>Cod_FOXO3a
ATGGCCGAGGCGTCGCAGGACGAACAAAACCTAAACGTTGAAATAGACCCAGACTTCGAG
CCCCAGAAAAGGCCCCGGTCGTGCACTTGGCCCCTGCCGCGTCCGGAGTCGGGCGCGAGC
AAACCGGGGACGAATGACACTGACGTCATCCCCGAAGAGGAGGACGACGAGAACGACGCG
GAGAATCGCGGCGTCGAGAAGAACTGCGCCGAGCTCGGGCCAAGGCCGCCGAATGGACCC
TCCTCCCTGCCCGTGGAGGTACGGAGGAGCACCCCGTCCAGCAGCAGGGAGGAAGGGGTG
GAGGACGGGTCCCCTTCCTCCTCCTCCATCCACCCCTCCGCTGCGGCGGCTCACGGCGGC
TCTGCCTCCCAGCAGCTGAGGAAGTCTTCCGCCCGGAGGAACGCCTGGGGGAACTACTCG
TACGCGGACCTCATCACGCAGGCCATCGAGAGCTCCCCGGAGAACCGGCTGACCCTTTCC
CAGATCTACGACTGGATGGTGAGGTCGGTGCCCTACTTCAAGGACAAAGGAGACAGCAAC
AGCTCCGCGGGCTGGAAGAACTCCATCCGCCACAACCTGTCGCTGCACAGCCGCTTCGTC
AAGGTGCAGAACGAGGGCACGGGCAAGAGCTCCTGGTGGATGGTCAACCCCGAGGGCGGC
AAGGGGGGCAAGGCGCCGCGCCGCCGCGCCGTCTCCATGGACAACAGCAAGTACATCAAG
GGCGCCCGGGGGCGGGCCACCAAGAAGAAGGCGTCCCTGATGGCCGCGCAGGACGGCAGC
TCGGAGAGCTCGTCCAGCCTGTCCAAGTGGACGGGCAGCCCCACGTCGCGCAGCAGCGAC
GAGCTGGACGCCTGGACCGACTTCCGCTCGCGCACCAACTCCAACGCCAGCACGCTGAGC
GGCCGCCTGTCGCCCATCCTCGCCAACCCGGAGCTGGACGAGGCTCAGGACGACGGCGGC
GGCGGCGGAGACTCGGCCCCCCTCTCCCCCATGCTGTACTCCAGCCCCAGCAGCATGTCC
TCCTCCACGGGCCCCATGGCGCTCCCCGACCTGGCGGGCACCATGAACCTCAACGACGGG
CTCTCGGACAACCTGATGGACGACCTCCTGGACAACATCAGCCTGACGGCCGCCCAGCAG
GCGCCCCCCGGCGAGGGGGACGACGACGACGACAACGTCGAGGACGAC
>Cow_FOXO3a
ATGGCAGAGGCGCCGGCCTCCCCGGCCCCGATCTCTCCGCTCGAAGTGGAGCTAGACCCG
GAGTTCGAGCCCCAGAGCCGGCCGCGCTCCTGTACGTGGCCCCTGCAGAGGCCGGAGCTC
CAGGGGAGCCCGGCCAAGCCCTCTGGGGAGGCGGCTGCTGACTCCATGATCCCCGAGGAG
GAGGACGATGAAGACGACGAGGACGGTGGCGGTAGGGCCGGCTCGGCCATGGCGATCGGC
GGCGGCGGGGGCGGCCCGCTGGGCTCTGGGTTGCTCCTGGAGGACTCGGCCCGGCTGCTG
GCTCCTGGAGGGCAGGACCCCGGGTCCGGGCCAGCCCCCGCGGCGGGCGCGCTGAGCGGG
GGGACGCAGACACCGCTGCAGCCTCAGCAGCCACTGCCACCGCCGCAGCCGGGGACGGCT
GGGGGCTCTGGGCAGCCGAGGAAATGCTCGTCGCGGAGGAACGCCTGGGGGAACCTGTCC
TACGCCGATCTGATCACTCGCGCGATCGAGAGCTCTCCAGACAAACGGCTCACTCTGTCC
CAGATCTATGAGTGGATGGTGCGCTGCGTGCCCTACTTCAAGGATAAGGGCGACAGCAAC
AGCTCTGCCGGCTGGAAGAACTCTATCCGGCACAACCTGTCACTGCACAGCCGGTTCATG
CGGGTCCAGAATGAGGGAACCGGCAAGAGCTCGTGGTGGATCATCAACCCTGACGGCGGG
AAGAGTGGCAAGGCGCCCCGGCGGCGGGCCGTCTCCATGGACAACAGCAACAAGTACACC
AAGAGCCGCGGCCGTGCAGCCAAGAAGAAGGCAGCCCTGCAGACCGCCCCCGAGTCAGCA
GATGACAGTCCCTCCCAGCTCTCCAAGTGGCCCGGCAGCCCCACATCCCGCAGCAGCGAT
GAGCTGGACGCGTGGACCGACTTCCGCTCGCGCACCAATTCCAACGCCAGCACAGTCAGC
GGCCGCCTGTCCCCCATCCTGGCGAGCACGGAGCTGGACGACGTCCAGGACGATGATGCA

CCACTGTCCCCCATGCTCTATAGCAGCTCGGCCAGCCTGTCGCCCTCCGTCAGCAAGCCG
TGCACTGTGGAGCTGCCCCGGCTGACCGACATGGCGGGCACCATGAATCTGAACGACGGG
CTGGCCGACAACCTCATGGACGACCTGCTGGACAATATCGCGCTCCCTGCATCCCAGCCA
TCGCCCCCGGGGGGGCTCATGCAGCGCAGCTCCAGCTTCCCGTACACCACCAAGGGCTCC
GGCCTGGGCTCCCCCACCAGCTCCTTCAGCAGCGCGGTATTTGGTCCCTCGTCTCTGAAC
TCCCTGCGCCAGTCTCCCATGCAGACCATCCAAGAGAACAAGCCAGCCACCTTCTCTTCC
ATGTCCCACTACGGCAACCAGACACTCCAGGACCTGCTCACATCGGACTCACTCAGCCAC
AGCGATGTCATGATGACCCAGTCGGACCCCTTGATGTCTCAGGCCAGCACCGCTGTGTCC
GCCCAGAACTCCCGCCGGAACGTGATGCTTCGCAGTGACCCAATGATGTCCTTTGCCGCC
CAGCCTAACCAGGGGAGTTTGGTCAATCAGAACTTGCTCCACCACCAGCACCAAACCCAG
GGCGCTCTCGGTGGCAGCCGTGCCTTGTCGAATTCCGTCAGCAACATGGGCTTGAGCGAC
TCCAGCAGCCTCGGGTCAGCCAAACACCAGCAACAGTCTCCCGTCAGCCAGTCTATGCAA
ACCCTCTCGGACTCTGTCTCAGGCTCCTCCTTGTACTCAACCAGTGCGAACCTTCCAGTC
ATGGGTCACGAGAAGTTCCCCAGCGACTTGGACCTGGACATGTTCAATGGGAGCTTGGAA
TGTGACATGGAGTCCATTATCCGTAGCGAACTCATGGATGCTGATGGGTTGGATTTTAAC
TTTGATTCCCTCATCTCCACACAGAACGTTGTTGGTTTGAACGTGGGGAGCTTCACTGGT
GCTAAGCAGGCCTCATCTCAGAGCTGGGTGCCAGGC
>Dog_FOXO3a
ATGGCAGAGGCGCCGGCCTCCCCGGGCCCGCTCTCCCCGCTCGAAGTGGAGCTGGACCCC
GAGTTCGAGCCCCAGAGCCGGCCGCGCTCCTGTACGTGGCCCCTGCAGAGGCCGGAGCTG
CAGGGGAGCCCGGCCAAGCCCTCGGGGGAGACGGCCGCCGACTCGATGATCCCCGAAGAG
GACGACGACGACGACGACGAGGACGGCGGTAGGGCCGGCTCGGCCATGGCGCTCGGCGGC
GGCGGGGGCGGCGCGCTGGGCTCCGGGCTGCTCCTCGAAGACGCGGCCCGGCTGCGGGCT
CCCGGGGGGCAGGACCCCGGGGCCGCGCCGGGCCCCGCGGCGGGCGCGCCGAGCGGGGGG
ACCCAGGCGCCGCCGCAGCCTCCGCAGGCGCTGGCCCCGCCGCAGCCGGGGGCGGCCGGG
GGCTCGGGGCAGCCGAGGAAGTGCTCGTCGCGGCGCAACGCCTGGGGCAACCTGTCCTAC
GCCGACCTCATCACTCGCGCCATCGAGAGCTCGCCCGACAAACGGCTCACTCTGTCCCAG
ATCTACGAGTGGATGGTGCGCTGCGTGCCCTACTTCAAGGATAAGGGCGACAGCAACAGC
TCGGCGGGGTGGAAGAACTCTATCCGGCACAACCTGTCACTGCACAGTCGGTTCATGCGA
GTCCAGAATGAGGGGACTGGCAAGAGCTCTTGGTGGATCATCAACCCTGATGGGGGAAAG
AGCGGGAAGGCACCCCGGCGGCGGGCTGTCTCCATGGACAACAGCAACAAGTATACCAAG
AGCCGTGGCCGTGCAGCCAAGAAGAAGGCAGCCCTGCAGACAGCCCCTGAGTCAGCAGAT
GACAGTCCCTCCCAGCTCTCCAAGTGGCCCGGCAGCCCCACGTCACGCAGCAGCGATGAG
CTGGATGCATGGACGGACTTCCGCTCGCGCACCAATTCTAATGCCAGCACTGTCAGTGGC
CGCCTGTCACCCATCTTGGCAAGCACGGAGTTGGATGACGTCCAGGATGATGACGCACCA
CTCTCCCCCATGCTCTACAGCAGCTCAGCTAGCCTCTCACCCTCTGTGAGTAAGCCATGC
ACTGTGGAGCTACCACGGCTGACCGACATGGCAGGCACCATGAATCTGAATGATGGGCTC
TCTGACAACCTCATGGATGACCTGCTGGATAACATCACACTCCCATCGTCCCAGCCATCG
CCCACTGGAGGGCTCATGCAGCGGAGCTCTAGCTTCCCATATACCACCAAGGGCTCCGGC
CTGGGTTCTCCAACTAGCTCCTTTAACAGCACGGTGTTCGGACCCTCGTCTCTGAATTCC
CTGCGCCAGTCTCCCATGCAGACCATCCAAGAGAACAAGCCAGCTACCTTCTCTTCCATG
TCACACTATGGCAACCAGACACTCCAGGACCTGCTCACTTCAGACTCACTCAGCCACAGC
GATGTCATGATGACCCAGTCGGACCCCTTGATGTCTCAGGCCAGCACCGCTGTGTCCGCC
CAGAACTCCCGCCGGAACGTGATGCTTCGAAATGACCCAATGATGTCTTTTGCCGCCCAG

CCTAACCAGGGGAGTTTGGTCAATCAGAACTTGCTCCACCACCAGCACCAAACCCAGGGT
GCTCTCGGTGGCAGCCGTGCCTTGTCGAATTCTGTCAGCAACATGGGCTTGAGCGACTCC
AGCAGCCTTGGGTCGGTTAAACACCAGCAACAGTCTCCTGTCAGCCAGTCTATGCAAACC
CTCTCGGACTCTCTCTCAGGCTCCTCCTTGTACTCAACTAGTGCAAACCTTCCCGTCATG
GGCCATGAGAAGTTCCCCAGCGACTTGGACCTGGACATGTTCAATGGGAGCTTGGAATGT
GACATGGAGTCCATTATCCGTAGCGAACTCATGGATGCTGATGGGTTGGATTTTAATTTT
GATTCCCTCATCTCCACACAGAATGTTGTTGGTTTGAACGTGGGGAACTTCACTGGTGCT
AAGCAGGCCTCATCTCAGAGCTGGGTGCCAGGC
>Dolphin_FOXO3a
ATGGCAGAGGCGCCGGCCTCCCCGGCCCCGCTCTCTCCGCTCGAAGTGGAGCTGGACCCG
GAGTTCGAGCCCCAAAGCCGGCCGCGCTCCTGTACGTGGCCCCTGCAGAGGCCGGAGCTC
CAGGGGAGCCCGGCCAAGCCCTCGGGGGAGGCGGCCGCTGACTCCATGATCCCCGAGGAG
GAGGACGATGAAGACGACGAGGACGGCGGCGGTAGGGCCGGCTCGGCCATGGCGATCGGC
GGCGGCGCGGGCGGCCCGCTGGGCTCAGGGCTGCTCCTGGAGGACTCGGCTCGGCTGCTA
GCTCCGGGAGGGCAGGACCCCGGGTCCGGGCCAGCCCCCGCGGCGGGCGCGCTGAGCGGG
GGGACGCAGACGCCGCTGCAGCCTCAGCAGACACTGCCACCGCCGCAGCCGGGGGCGGCT
GGGGGCTCCGGGCAGCCGAGGAAATGCTCGTCGCGACGGAACGCCTGGGGGAACCTGTCA
TACGCTGACCTGATCACTCGCGCCATCGAGAGCTCCCCGGACAAACGTCTCACTCTGTCC
CAGATCTACGAGTGGATGGTGCGCTGCGTGCCCTACTTCAAGGATAAGGGCGACAGCAAC
AGCTCTGCCGGCTGGAAGAACTCCATCCGGCACAACCTGTCGCTGCACAGCCGGTTCATG
CGGGTTCAGAATGAGGGGACCGGCAAGAGCTCATGGTGGATCATCAACCCCGACGGGGGG
AAGAGCGGGAAGGCGCCCCGGCGGCGGGCCGTCTCCATGGACAACAGCAACAAGTACACC
AAGAGCCGGGGCCGTGCAGCCAAGAAGAAGGCAGCCCTGCAGACCGCCACCGAGTCAAAC
GACGACAGTCCCTCCCAGCTCTCCAAGTGGCCCGGCAGCCCCACGTCTCGCAGCAGTGAT
GAGCTGGATGCGTGGACCGACTTCCGCTCACGCACCAATTCCAATGCCAGCACGGTCAGT
GGCCGCCTGTCCCCTATCCTGGCTAGCACGGAGTTGGACGACGTTCAGGATGATGACGCG
CCACTCTCCCCCATGCTCTACAGCAGCTCGGCCAGCCTGTCCCCCTCCGTCAGCAAGCCG
TGCACCGTGGAGCTGCCCCGGCTGACCGACATGGCGGGCACCATGAATCTGAACGACGGG
CTGGCCGACAACCTCATGGACGACCTGCTGGACAACATCGCGCTCCCTGCGTCCCAGCCG
TCGCCCCCCGGAGGGCTCATGCAGCGCAGCTCCAGCTTCCCATACACCACCAAGGGCTCC
GGCCTGGGCTCCCCCACCAGCTCCTTCAGCAGCACGGTGTTTGGCCCCTCGTCTCTGAAC
TCCCTGCGTCAGTCTCCCATGCAGACCATCCAAGAGAACAAGCCAGCCACCTTCTCTTCC
ATGTCGCACTACGGCAACCAGACACTCCAGGACCTGCTCACGTCGGACTCACTCAGCCAC
AGCGATGTCATGATGACCCAGTCGGACCCCTTGATGTCTCAGGCCAGCACCGCTGTGTCC
GCCCAGAACTCCCGCCGGAGCGTGATGCTTCGCAGCGATCCAATGATGTCCTTTGCCGCC
CAGCCTAACCAGGGGAGTTTGGTCAATCAGAACTTGCTCCACCACCAGCACCAAACCCAG
GGCGCTCTCGGTGGCAGCCGTGCCTTGTCAAGTTCCGTCAGCAACATGGGCTTGAGCGAT
TCCAGCAGCCTCGGGTCAGCCAAACACCAGCAGCAGTCTCCTGTCAGCCAGTCTATGCAA
ACCCTCTCGGACTCTCTCTCAGGCTCCTCCTTGTACTCAGCTAGTGCACACCTTCCCGTC
ATGGGCCACGAGAAGTTCCCCAGCGACTTGGACCTGGACATGTTCAATGGGAGCTTGGAA
TGTGACATGGAGTCCATTATCCGTAGCGAACTCATGGATGCTGATGGGTTGGATTTTAAC
TTTGATTCACTCATCTCCACACAGAACGTTGTTGGTTTGAATGTGGGGAGCTTCACTGGT
GCTAAGCAGGCCTCATCTCAGAGCTGGGTGCCAGGC
>Duck_FOXO3a

ATGTTCTGGGGGAGGCGAGATCAATTTTCTGCACTTCAACGCAGCTGCAGCAGCGCTGGC
CATACAAACTCCATCCGGCACAACTTGTCGCTCCACAGCCGCTTCATCAGGGTGCAGAAC
GAGGGCACCGGGAAGAGCTCCTGGTGGATGATCAATCCAGATGGCGGAAAAGTGGGCAAG
GCGCCACGCAGACGCGCCGTGTCCATGGACAACAGCAACAAGTACACCAAGAGCAGAGGG
CGGGCGGCTAAGAAAAAGGCGGCCCTGCAAACGGCCCAGGAGGCAAGCGAGGACAGCCCT
TCCCAGCTCTCCAAGTGGCCGGGGAGCCCAACGTCCCGCAGCGGCGATGAGCTGGACGCC
TGGACGGATTTTCGCTCCCGGACGAATTCCAACGCCAGTACGATAAGCGGCCGCTTGTCC
CCCATTTTGGCAAGCACCGAGCTAGATGATGTTCAAGACGACGACGCTCCGCTTTCTCCC
ATGCTGTACAGCAGTCCATCGAGCTTGTCCCCCTCGGTAAACAAACCGTGTACCGTGGAG
TTGCCTAGGTTGACTGATATGGCCGGGACAATGAATTTGAACGATGGACTGACAGATAAC
CTCATGGATGATCTCTTGGACAATATAACACTCCCTTCCTCCCAGCAGTCGCCCACGGGA
GGGATGATGCAGAGGAGCTCCAGCTTTCCGTATGGTTCCAAAGGTTCGGGGCTGGGTTCC
CCCTCAAGTAGTTTCAACAACGCCGTGTTCGGGCCCTCGTCCCTGAACTCCCTCCGCCAG
TCGCCCATGCAGACCATTCAGGAGAACAAGCAGGCCACCTTCTCTTCCATGTCCCATTAC
AACAACCAGACGCTGCAGGATCTGCTCGCCTCGGACGCGCTCAGTCACAGCGATGTCATG
ATGACGCAGTCCGACCCGCTCATGTCCCAAGCCAGCACAGCTGTGTCCGCCCAAAACTCC
CGCAGGAACATCATGCTCCGCAACGACCCCATGATGTCGTTTGCCGCGCAGTCCAGCCAG
GGCGGTCTGGTCAATCAGAGCCTGCCACATCACCAGCACCAGTCTCACAGCTCTCCTCTT
AGCGGCAGCCGTGCCTTGTCCAATTCCATCAGTAACATAGGCTTGAGTGACTCGAACAGC
TTGGGTTCCTCCAAACATCAGCAGTCACCCGTCAACCAGTCTATGCAAACACTTTCTGAC
CCGCTCTCAGGCTCCTCTTTGTACTCCTCTAGCGTGAACCTCCCGGTCATGGGACACGAG
AAATTCCCGAGTGACTTGGACCTGGATATTTTCAATGGGAGCTTGGAGTGTGACATGGAG
TCCATCATCCGCAGTGAACTCATGGATGCAGATGGGCTGGATTTTAACTTTGATTCCCTC
ATCTCAGCTCAGAACGTTGTCAGTCTGAATGTGGGGAACTTCACTGGTGCTAAACAGGCT
TCATCACAGAGTTGGGTACCAGGC
>Elephant_FOXO3a
ATGGCAGAGGCACCGGCCTCCCCGACCCTGCTTTCGCCGCTGGAAGTGGAGCTGGACCCT
GAGTTCGAGCCCCAGAGCCGGCCGCGCTCCTGTACGTGGCCCTTGCAGAGGCCGGAGCTC
CAGGCGAGCCCGGCCAAGCCCTCGGGGGAGGCGGCCGCCGATTCCATGATCCCCGAGGAG
GACGACGATGAAGACGAGGAGGACGGCGGCGGTGGGGCCGGCTCGGCTATGGCGATCGGC
GGCGGCGAGAGCGGCACGCTGGGCTCGGGGCTGCTTCTTGAGGACCCGGCTAGGCTGCTG
GCTCCCGGAGGGCAGGACCCTGGGTCCGGGCAAGCCCCCGCGGCGGGCGCGCTGAGCGGG
GGGATGCAGGGGGCGGCTGGGGGCTCCGGGCAACCGAGGAAATGCTCGTCGCGCCGGAAC
GCTTGGGGGAACCTGTCCTACGCTGATCTGATCACCCGCGCCATCGAGAGCTCTCCGGAC
AAGCGGCTCACCCTGTCCCAGATCTACGAGTGGATGGTGCGCTGCGTGCCCTACTTCAAG
GATAAGGGCGATAGCAACAGCTCGGCGGGTTGGAAGAACTCTATCCGGCACAACCTGTCG
CTGCATAGCAGGTTCATGCGGGTCCAGAATGAGGGGACCGGCAAGAGTTCTTGGTGGATC
ATCAACCCCGATGGGGGGAAGAGTGGAAAGGCACCCCGGCGGCGGGCAGTGTCCATGGAC
AACAGCAACAAGTATACCAAGAGCCGTGGCCGTGCAGCCAAGAAGAAGGCAGCCCTGCAG
ACGGCCCCTGAGTCAGCAGATGACAGTCCCTCCCAGCTTTCCAAGTGGCCTGGCAGCCCC
ACCTCACGCAGCAGTGATGAGCTGGATGCGTGGACGGACTTCCGCTCACGCACCAATTCT
AATGCCAGCACGGTCAGCGGCCGCCTGTCACCCATCCTGGCAAGCACAGAGTTGGACGAC
GTCCAGGACGATGACGCACCTCTCTCCCCCATGCTCTACAGCAGCTCAGCCAGCCTTTCA
CCCTCTGTAAACAAGCCATGCACTGTAGAGCTGCCACGGCTGACTGACATGGCAGGCACC

ATGAATCTGAACGATGGGCTGGCTGACAACCTCATGGATGACCTGCTGGACAACATCACG
CTCCCGTCGTCCCAGACATCGCCTCCTGGGGCGCTTATGCAGCGGAGCTCCAGCTTCCCG
TACACCACCAAGGGCTCCACCCTGGGTTCTCCAACCAGCTCCTTTAACAGCACGGTGTTT
GGACCTCCATCTCTGAATTCCCTACGCCAGTCTCCCATGCAAACCATCCAAGAGAACAAG
CCGGCCACTTTCTCTTCCCTTTCACACTATGGCAACCAGACACTCCAAGACCTGCTTACT
TCGGACTCGCTCAGCCACAGTGATGTCATGATGACCCAGTCGGACCCTTTGATGTCTCAG
GCCAGCACTGCTGTGTCTGCCCAGAACTCACACCGGAACGTGATGCTTCACAATGACCCG
ATGATGTCCTTTGCTGCCCAGCCCAACCAGGGGAGTTTGGTCAATCAGAACTTGCTCCAC
CACCAGCACCAAACCCAGGGCGCTCTTGGTGGCAGCCGTGCCTTGTCGAATTCTGTCAGC
AACATGGGCTTGAGTGACTCCAGCAGCCTTCGGTCAGCCAAACACCAGCAGCAGTCTCCT
GTCAGCCAGTCTATGCAAACCCTCTCAGACTCTCTCTCAGGCTCCTCCTTGTACTCAACT
AGTGCAAACCTTCCCGTTATGGGCCATGAGAAGTTCCCCAGCGATTTGGACCTGGACATG
TTCAACGGGAGCTTGGAATGTGACATGGAGTCCATTATCCGTAGTGAACTCATGGATGCT
GATGGGTTGGATTTTAACTTTGATTCCCTCATCTCCACACAGAACGTTGTTGGTTTGAAC
GTGGGGAACTTCACTGGTGCTAAGCAGGCCTCATCTCAGAGCTGGGTGCCAGGC
>Flycatcher_FOXO3a
ATGGTCCGCTGCGTGCCCTACTTCAAGGACAAGGGCGACAGCAACAGCTCGGCCGGGTGG
AAGAATTCGATCCGGCACAACTTGTCACTCCACAGCCGATTCGTCAGGGTGCAGAATGAA
GGCACTGGGAAAAGCTCTTGGTGGATGATCAATCCAGATGGTGGAAAAGGCGGCAAGGCG
CCCCGGAGACGCGCTGTGTCAATGGACAACAGCAACAAGTACACCAAGAGCAGAGGGCGG
GCAGCAAAGAAAAAGGCAGCCCTGCAGACAGCCCAGGAGACGAGCGAGGACAGCCCTTCC
CAGCTCTCCAAGTGGCCGGGGAGTCCCACCTCCCGCAGCAGCGACGAGCTGGATGCGTGG
ACGGATTTTCGCTCCCGTACAAATTCAAATGCCAGTACGATCAGTGGCCGCTTGTCACCC
ATTTTGGCGAGCACCGAGCTCGATGATGTTCAAGATGACGATGCTCCACTTTCTCCCATG
CTGTACAGTAGTCCATCAAGCTTGTCCCCATCAGTAAACAAACCATGTACTGTGGAGTTG
CCTAGGTTGACTGATATGGCTGGGACAATGAACTTGAATGATGGACTGACTGATAACCTG
ATGGATGATCTCTTGGACAATATAACACTCCCTCCCTCCCAGCAGTCACCCACAGGAGGG
ATGATGCAGAGAAGCTCCAGTTTTCCTTATGGTTCCAAAGGTTCAGGGCTGGGCTCCCCA
TCAAGCAGTTTCAACAATGCTGTGTTTGGGCCGTCGTCCCTGAATTCCCTCCGCCAGTCA
CCCATGCAGACGATTCAGGAGAACAAGCAGGCCACCTTCTCTTCCATTTCTCATTACAAC
AACCAGACGCTGCAGGATCTCCTCACCTCTGACGCGCTGAGTCACAGCGATGTCATGATG
ACACAGTCTGACCCACTCATGTCACAAGCCAGCACAGCTGTGTCCGCCCAGAATTCCCGC
AGGAATATAATGCTCCGCAACGACCCCATGATGTCGTTTGCCGCGCAGTCCAGCCAGGGC
GGTCTGGTCAATCAGAACCTGTCCCATCACCAGCACCAGTCCCACAGCTCCTCTCTCAGC
GGCAGCCGTGCCTTGTCCAATTCCATCAGTAACATAGGCTTGAGTGACTCCAACAGCTTG
GGATCCTCCAAACATCAGCAGTCACCTGTCAATCAGTCTATGCAAACACTTTCTGACCCG
CTGTCAGGCTCCTCTTTGTACTCTAGCGTGAACCTCCCGGTCATGGGGCATGAGAAATTC
CCGAGTGACTTGGACCTGGACATTTTCAACGGGAGCCTGGAGTGTGACATGGAGTCCATT
ATCCGCAGTGAACTCATGGATGCTGATGGGCTGGATTTTAATTTTGATTCCCTCATCTCA
GCTCAGAACGTTGTCAGTCTGAATGTGGGGAACTTCACTGGTGCTAAACAGGCTTCATCA
CAGAGTTGGGTACCAGGC
>tropical_clawed_frog_FOXO3a
ATGGCAGAAGCCGTGCCTTCCCTCTCCCCGCCTGGGGACGTGGACATAGACCCGGACTTC
GAGCCGCAGAGCCGACCCCGGTCCTGCACGTGGCCCCTGCAGAGACCGGACTCCCAAGGC

AGCCCGGGCAAGCCGAATAGTGGCGCTGGGGAAGCCGGAGACGCGTCCTCTATGATCCCG
GAGGAGGAGGATGATGACGATGAGGGATCTGGTACTACAGCCACTATGGTAGGGACAGCG
GGGGAGAAGGGGACTGTGGTGCTACTGAGCGGAGGAGACCTGACCGTGCTGGCGTCACCT
GTTGGTGGGGTGGAAACACTGCAGGCTTCGCTTGGGGGTGGGGGAGCAGGGGGCGCCCAG
AGTGGCGCAACCGGACAGCAACAGAGGAAATGTTCATCCCGCAGAAATGCCTGGGGCAAC
ATGTCCTACGCTGACCTTATCACTAGGGCCATCGAGAGCTCCCAGGATAAACGGCTCACC
CTGTCCCAGATATATGATTGGATGGTCCGCTCAGTGCCCTACTTCAAGGATAAGGGAGAC
AGCAACAGCTCTGCAGGATGGAAGAATTCAATCCGGCATAATCTGTCACTACACAGCAGG
TTTATTAGAGTTCAGAATGAGGGCTCTGGCAAAAGCTCTTGGTGGATGATCAACCCTGAA
GGAGGAAAAGGGGGGAAGGCACCAAGAAGACGGGCTGTTTCAATGGACAATAGCAACAAA
TACACCAAGAGCCGTGGGAGAGCAGCAAAGAAAAAAGCGTCTTTGCAGGCATCTACTGAT
GCTACAGATGATAGCCCTTCGCAGCTGTCAAAGTGGCCAGGTAGTCCCACCTCACGTAGT
AGTGATGAACTTGATGCCTGGACAGATTTTCGATCTCGTACAAACTCTAATGCCAGTACC
ATAAGTGGTCGTTTGTCTCCAATTCCAGCTACAACTGAACTTGATGATGTTCAAGATGAT
GACTCTCCCTTGTCCCCTATGTTGTATAGCAGCCCAGGTAGTTTGTCCCCATCTTTAAGT
AAACCATGCACAGTGGAGATGCCAAGAATAACAGATATGGCTGGAACCATGAACTTAAAT
GATGGATTGCCAGAGAACCTCATGGATGACTTGCTGGATGACATTTCTCTCACTTCTTCA
CAGCAGTCATCTCCCGGTATCCTCATGCAGAGAAGTTCCAGCTTTACATATGGCACTAAG
GGCTCAGGCATTGGCTCTCCATCTAATACTTTTAACAACACTAGTAGCTTCAACTTTCCC
TTGACATCGCTGCGCCAGTCTCCCATGCAAACTATCCAAGAGAACAAGCAAGCTACTTTT
TCTTCCATTAATCATTACAGTAACCAGTCTTTGCAAGACCTGTTGAATACAGACACCCTC
AGCCACAGTGATGTGTTAATGACCCAGTCTGATCCACTCATGTCACAAGCCAGTACTGCT
GTGACTGCTCAGAATACCAGAAGAAATATAATTCTGAGAAATGACCCTATGATGTCTTTT
GCAGCCCCATCTAACCAAGGAGGAAATTTGGTTAATCAGAACTCGCTGCACCAACAGCAG
TCTCTTAACTCTTTTCAAGGTGGCAGCCGTGCCTTGTCAAACAATTTAAGCAACACTGGT
TTAAATGATAGCAGCAACTTGGACTCAACCAAACACCAGCAGCAGTCCTCAGTTAGTCAT
TCTATGCAAACCATCTCAGACACGCTCTCAGGATCCTTGTACTCCTCAGCCGTGAACCTT
CCAACACTGGGGCATGAAAAGTTTCCCACTGATTTGGACCTGGATATTTTCAATGGGAGT
TTGGAATGTGACATGGAGACAATAATTCGCAACGATTTAATGGATGCAGATGGGTTGGAT
TTTAATTTTGACACCCTCATCTCAGCTCAGAATGTCAGTCTATCGGTGGGCAGTTTCACT
GGTGCTAAGCAGACATCGTCACAGAGCTGGGTGCCAGGC
>Fruitfly_FOXO3a
ATGATGGACGGCTACGCGCAGGAATGGCCCAGGCTGACCCACACAGATAACGGCCTGGCC
ATGGACCAGCTGGGCGGGGATCTGCCCCTGGACGTGGGCTTCGAGCCACAGACCCGGGCC
AGATCCAACACATGGCCATGTCCGCGTCCCGAAAACTTTGTGGAGCCCACCGACGAGTTG
GACAGTACAAAGGCCAGCAATCAGCAGTTGGCCCCAGGAGACTCACAGCAGGCTATACAG
AATGCGAATGCAGCCAAGAAGAACTCATCGCGTCGCAATGCATGGGGAAATCTATCCTAT
GCGGATCTCATCACGCATGCCATTGGATCGGCCACCGACAAACGATTGACACTGAGTCAG
ATTTACGAGTGGATGGTCCAGAATGTGCCATATTTCAAGGACAAGGGCGATTCGAATAGC
AGTGCCGGATGGAAGAACTCCATACGTCACAATCTGTCGCTGCACAACCGCTTTATGAGG
GTCCAAAACGAGGGCACCGGCAAGTCATCCTGGTGGATGCTCAACCCGGAGGCCAAGCCC
GGCAAGTCTGTGCGCCGCCGTGCCGCTTCCATGGAGACGTCCCGGTACGAGAAGCGGCGC
GGCAGGGCCAAGAAGCGGGTGGAGGCACTGCGTCAGGCGGGCGTGGTGGGCCTCAACGAT
GCCACGCCCTCGCCCAGCAGCAGCGTCAGCGAGGGGCTGGATCACTTTCCCGAGAGTCCG

CTCCACAGTGGCGGTGGCTTCCAATTATCGCCCGATTTCCGGCAACGCGCCTCATCCAAT
GCCAGTTCCTGCGGACGCCTGAGCCCCATTAGGGCGCAGGATCTTGAGCCCGACTGGGGA
TTCCCCGTTGACTACCAGAACACAACGATGACGCAGGCCCACGCCCAGGCGCTCGAGGAG
CTGACGGGCACAATGGCGGATGAGCTGACGCTGTGCAACCAGCAGCAGCAAGGGTTCAGT
GCCGCCTCGGGACTTCCCTCTCAGCCCCCGCCCCCGCCCTATCAGCCGCCGCAGCATCAA
CAGGCGCAGCAGCAGCAACAGCAGCAGTCGCCCTACGCCCTCAACGGCCCCGCCTCCGGC
TACAACACGCTGCAGCCGCAGTCGCAGTGCCTGCTGCACCGGTCCCTCAATTGCAGTTGC
ATGCACAATGCAAGAGATGGTCTCTCGCCGAACTCAGTAACCACAACAATGTCGCCCGCC
TATCCAAACAGCGAGCCCTCATCGGACTCCCTGAACACGTACAGCAACGTGGTGCTCGAT
GGTCCGGCGGACACTGCGGCACTGATGGTGCAGCAGCAGCAGCAGCAGCAGCAACAGCAA
CAGCTGTCCGCCAGCTTGGAAGGACAATGCCTGGAGGTGCTCAATAACGAGGCGCAGCCG
ATAGACGAATTTAATCTGGAGAACTTTCCCGTGGGCAATCTCGAGTGCAATGTCGAGGAG
CTGCTGCAGCAGGAGATGAGCTACGGCGGCCTGCTGGACATCAATATACCGCTGGCCACG
GTCAACACGAACCTGGTCAACAGCAGCAGTGGGCCCCTGAGCATCAGCAACATTAGCAAC
CTCAGCAACATAAGCAGCAATTCCGGCAGCAGTCTCAGTCTGAATCAGTTGCAGGCTCAG
CTGCAACAGCAGCAGCAGCAGCAGCAGGCGCAGCAACAGCAGCAGGCGCAGCAGCAACAA
CAGCAGCATCAGCAGCACCAGCAACAGTTGCTGCTAAATAATAACAACAACAGCAGCAGC
AGCCTGGAATTGGCAACACAAACGGCTACCACAAATCTGAATGCTCGGGTTCAGTACTCA
CAGCCCAGCGTGGTGACCTCGCCACCATCCTGGGTGCAC
>Fugu_FOXO3a
ATGGCTGAGGCGCCGTGCAACGAGCCTCCCTCCAACATCGAGATCGACCCGGATTTCGAG
CCCCAGAAGCGGCCACGCTCCTGTACTTGGCCCCTTCCCCGTCCGGAGTCTGGTGCGGGC
AAACCCGGGACTAATGACACTGACGTAATCCCCGAAGAGGAGGACGATGAGGGTGGCAGC
TCGGGCAGTGGCGATGCTCAGAAAACCAGCGGTGCCATTAACATTAAACCCCGGGAGTCG
GGCAGGAGCTCCGCTTCTCAGCCCGTGGAGCTCCAGCGCGGACCCTGCAAGGAGGAGGCC
GCCGATGGCTCGCCCTCCTCCGCACAGACCCCCGCCGCTGCCGCTCTCAGTACCTCTGCG
TCCCAGCAGCTGAGGAAGTCCTCTGCCCGTCGGAACGCATGGGGCAACTACTCCTATGCA
GACCTCATTACGCAAGCCATCGAGAGCTCCCCCGAGAAGAGGCTGACCTTGTCCCAAATC
TATGACTGGATGGTGAGGTCTGTGCCATATTTCAAGGACAAAGGCGACAGCAATAGCTCT
GCTGGCTGGAAGAATTCCATCCGACACAATCTGTCCCTTCACAGCCGATTTGTGAAAGTC
CAGAATGAAGGAACTGGAAAAAGCTCCTGGTGGATGGTCAACCCCGAGGGTGGGAAAGGA
GGCAAAGCTCCAAGACGAAGGGCCGTATCCATGGATAACAGCAAGTACATTAAAGGAGCC
CGAGGACGTGCCACCAAGAAGAAAGCCTTGCTGCAGGCTGCTCAGGACGGCAGCTCGGAA
AGCTCCTCCAGCCTCTCCAAATGGACAGGAAGTCCCACATCCCGCAGTAGCGACGAGCTG
GATGCCTGGACAGACTTCCGCTCCCGGACCAATTCTAACGCCAGCACGCTCAGTGGTCGT
CTGTCCCCGATCTTGGCCAACCTGGAGCTTGACGAGGTGCCCGATGATGACTCGCCCCTT
TCTCCCATGTTGTATTCCAGTCCCAGCAGCATGTCCCCGTCCACTGGGCCCACAGTGCTG
TCTGATTTAGCGGGCACGATGAACCTCAATGATGGGCTTTCCGACAACCTGATGGATGAC
CTTTTAGACAATATCAGCCTGACCGCAACCCAGCCGCTTCCTCCTGGAGAGGAAGAAAAT
GACAGTCAGGGGACCTCTGTTTTTACCTTTGGCTGCTCAGGAAGCGGTCTAGGTAGCCCT
TCGGGCAGCTATGGGCCTAACCCTCTCTTTAGCCCTCCGTCCATCACAAGCCTGCGGCAG
TCGCCCATGCAGACCATCCAGGAGAACAAGCAGACCACCTTCTCCTCCATCTCGCACTTC
AGTGACCACCAGACCCTCCAGGATCTGCTGAGTTTGGACTCCCACAGCCATAGCAATGTC
ATGCTCACCCAGTCGGACCCACTGATGTCACAGGCCAGCACTGCCATCGCCCTGCAGAAC

TCCCGCCGGAATGCTATGCTTCTCCGTAAAGACCCCGCCTCGGTGAATCACGCCGGTGCA
GGTCAGGCTCAAAGCGCTTCAGTGCCTGGTTGGCAAGCTGGCTTGTCAACCCCTGATGAA
AACGGTGGCCGCAACAATGCCAAGCAGCCACACCTGAAGTCTCCCAGCAAGAATGCCTCT
ATGCAGCTCGGCTCTGGTTTTCCCAGCCAGGATCGCTTTCCCACCGATCTGGACCTCGAT
GTGTTCAACAGCAGCCTCGAGTGTGACATGGACGCCATCATCCGGAATGAACTGATGGAC
GCGGATTGCCTGGACCTCAGCTTTGACTCTCGCCTCGCCTCCACCCAGAATGGTAACAAG
AATTCAGGAAGCTTCTCCGGCTCAAAACCAACGTCCCCTCACAGCTGGGTCCCAAGC
>Anole_lizard_FOXO3a
ATGGCAGAGCCGTCTCCGCCGTCGCCGCTGGACGTGGAGCTGGACCCGGACTTCGAGCCC
CAGAGCCGCCCGCGGTCCTGCACGTGGCCCCTGCAGAGGCCCGAGCTGCAGGCCAGCCCG
GAGGGGGGAGGCGGCGGCCCCGGCGGAGACGCGATGATCCCCGAGGAGAAGGACGACGAC
GAGGACGACGACGAGGAGCCCGCGGGCCCGGCGGCGGCGGAGGAGGGCCAGGAGGAGGCC
GCGGCCGCCCCCTTCCCCGCGGGCCCCAAGCAGCAGCTCCAGCAGCCCTCCTCGCCACAA
GCGGCCCCGCCACTGCCGCCCCCTCCGCCTCAGGCGGCTCCGCGGAAGTGCTCCTCCCGG
CGCAACGCCTGGGGGAACCTCTCTTACGCGGACCTCATCACCCGCGCCATCGAGAGCGCG
CCCGAGAAGCGCCTCACCCTGGCGCAGATCTACGAGTGGATGGTGCGCTGCGTGCCCTAC
TTCAAGGACAAGGGCGACAGCAACAGCTCCGCCGGGTGGAAGAACTCCATCAGACACAAC
TTGTCCCTTCACAGTCGATTCATCAGGGTACAGAATGAAGGAACCGGGAAAAGCTCGTGG
TGGGTGATCAACCCAGATGGAGGGAAAGGTGGGAAAGCGCCACGGAGACGTGCTGTCTCA
ATGGACAACAGTAACAAGTATACAAAGAGCCGAGGACGGGCAGCCAAGAAAAAAGCATCT
CTACAAGCTGCACAAGAAACCACGGAAGACAGCCCCACCCAGCTCTCCAAGTGGCCAGGT
AGCCCCACGTCACGCAGCAGTGATGAACTGGAAGCATGGACAGATTTCCGGTCCCGTACA
AACTCAAATGCTAGTACCATAAGTGGCCGCTTGTCTCCAATCTTGGCAAGTACTGAGCTA
GATGAAGTCCAGGACGATGATGCTCCGCTTTCCCCCATGCTGTATAGTAGCCCGCCCAGC
ATGTCTCCATCGGTAAACAAACCCTGTAACGTAGAGTTGCCTAGGTTGACGGATATGGCA
GGCACCATGAACTTGAATGATGGCCTGACAGATAACCTTATGGACGATCTTCTGGACAAT
ATAAGTCTCCCCCCTTCCCAGCAGTCTCCTACAGGGGGTCTCATGCAGAGAAGCTCAAGT
TTTCCATACAGCTCCAAAGGTTCAGGTCTTGGTTCTCCATCGAGTACTTTCAACAATGGT
GTGTTTGGGCCATCATCTCTGAATTCCCTCCGTCAGTCTCCCATGCAAACCATCCAAGAG
AACAAGCAAGCAACATTTTCTTCCATTTCTCACTACAATAACCAAACGCTGCAGGATCTG
CTGGCATCAGATTCGCACAGCCACAGTGATGTCATGATGACTCAGTCCGATCCACTCATG
TCGCAGGCTAGCACAGCTGTGGCTGCCCAGAACGTGCGCAGGAGTATTATGCTGCGGAAT
GATCCAATGATGTCATTTGCTGCGCAACCAAGCCAGGGTAGTTTGGTCAACCAGAACCTG
CTCCACCACCAGCATCAATCCCAGAATTCCTCTCTCAGTGGCAGTCGTGCCTTGTCGAAT
TCCGTCGGTACTATGGGCTTAAGTGACTCAAACAGCATTGGGCCGGCAAAACACCAGCAA
CAGTCGCCTGCCAACCACTCTATGCAATCCCTTTCTGACTCACTCTCAGGCTCTTCATTG
TATTCCAACAGCCTGAGCCTTCCAGTCATGGGGCATGATAAATTTCCCAGTGATTTGGAT
CTGGATATTTTCAATGGGAGCTTGGAATGCGATATGGAGTCCATTATCCGCAGTGAACTC
ATGGATGCAGATGGGTTGGATTTTAACTTTGATTCCCTGATCTCAGCCCAGAATGTTGTC
ACTCTGAATGTGGGAAACTTCACTGGTGCTAAGCAAGCTTCATCACAGAGTTGGGTTCCA
GGC
>Horse_FOXO3a
ATGCGGGTCCAGAACGAGGGGACGGGCAAGAGTTCTTGGTGGATCATCAACCCTGATGGG
GGCAAGAGCGGGAAGGCACCACGGCGGCGGGCTGTCTCCATGGACAACAGCAACAAGTAT

ACCAAGAGCCGTGGCCGCGCAGCCAAGAAGAAGGCAGCCCTGCAGACAGCCCCTGAGTCA
GCAGATGACAGTCCCTCCCAGCTCTCCAAGTGGCCCGGCAGCCCCACGTCACGCAGCAGC
GATGAGCTGGATGCATGGACGGACTTCCGCTCACGCACCAATTCCAATGCCAGCACTGTC
AGCGGCCGCCTGTCACCCATCCTGGCAAGCACAGAGTTGGATGACGTCCAGGATGATGAT
GCCCCGCTCTCCCCCATGCTCTATAGCAGCTCAGCCAGCCTGTCACCCTCTGTAAGCAAG
CCATGCACCGTGGAGCTGCCACGGCTGACCGACATGGCGGGCACCATGAATCTGAATGAT
GGGCTGGCTGACAACCTCATGGAGGACCTGCTGGATAACATCACGCTCCCATCCTCCCAG
CCATCGCCCCCTGGAGGGCTCATGCAGCGGAGCTCTAGCTTCCCATACACCACCAAGGGC
TCCGGCCTGGGCTCTCCCACCAGCTCCTTTAACAGCACGGTGTTTGGACCCTCGTCTCTG
AATTCTCTGCGTCAGTCGCCCATGCAGACCATCCAAGAGAACAAGCCAGCTACCTTCTCT
TCCATGTCACACTATGGCAACCAGACACTCCAGGACCTGCTCACTTCGGACTCACTCAGC
CACAGTGATGTCATGATGACCCAGTCGGACCCCTTGATGTCTCAGGCCAGCACCGCTGTG
TCCGCCCAGAACTCCCGCCGGAACGTGATGCTTCGCAACGACCCAATGATGTCCTTTGCT
GCCCAGCCTAACCAAGGGAGTTTGGTCAATCAGAACTTGCTCCACCACCAGCACCAAACC
CAGGGCGCTCTCGGTGGCAGCCGTGCCTTGTCGAATTCCGTCAGCAACATGGGCTTGAGC
GACTCCAGCAGCCTTGGGTCAGCCAAACACCAGCAGCAGTCTGTCAGCCAGTCTATGCAA
ACCCTCTCAGACTCTCTCTCAGGCTCCTCCTTGTACTCAACTAGTGCAAACCTTCCCGTC
ATGGGCCATGAGAAGTTCCCCAGTGACTTGGACCTGGACATGTTCAATGGGAGCTTGGAA
TGTGACATGGAGTCCATTATCCGTAGTGAACTCATGGATGCTGATGGGTTGGATTTTAAC
TTTGATTCCCTCATCTCCACACAGAACGTTGTTGGTTTGAACGTGGGGAACTTCACTGGT
GCTAAGCAGGCCTCATCTCAGAGCTGGGTGCCAGGC
>Human_FOXO3a
ATGGCAGAGGCACCGGCTTCCCCGGCCCCGCTCTCTCCGCTCGAAGTGGAGCTGGACCCG
GAGTTCGAGCCCCAGAGCCGTCCGCGATCCTGTACGTGGCCCCTGCAAAGGCCGGAGCTC
CAAGCGAGCCCTGCCAAGCCCTCGGGGGAGACGGCCGCCGACTCCATGATCCCCGAGGAG
GAGGACGATGAAGACGACGAGGACGGCGGGGGACGGGCCGGCTCGGCCATGGCGATCGGC
GGCGGCGGCGGGAGCGGCACGCTGGGCTCCGGGCTGCTCCTTGAGGACTCGGCCCGGGTG
CTGGCACCCGGAGGGCAAGACCCCGGGTCTGGGCCAGCCACCGCGGCGGGCGGGCTGAGC
GGGGGTACACAGGCGCTGCTGCAGCCTCAGCAACCGCTGCCACCGCCGCAGCCGGGGGCG
GCTGGGGGCTCCGGGCAGCCGAGGAAATGTTCGTCGCGGCGGAACGCCTGGGGAAACCTG
TCCTACGCGGACCTGATCACCCGCGCCATCGAGAGCTCCCCGGACAAACGGCTCACTCTG
TCCCAGATCTACGAGTGGATGGTGCGTTGCGTGCCCTACTTCAAGGATAAGGGCGACAGC
AACAGCTCTGCCGGCTGGAAGAACTCCATCCGGCACAACCTGTCACTGCATAGTCGATTC
ATGCGGGTCCAGAATGAGGGAACTGGCAAGAGCTCTTGGTGGATCATCAACCCTGATGGG
GGGAAGAGCGGAAAAGCCCCCGGCGGCGGGCTGTCTCCATGGACAATAGCAACAAGTAT
ACCAAGAGCCGTGGCCGCGCAGCCAAGAAGAAGGCAGCCCTGCAGACAGCCCCCGAATCA
GCTGACGACAGTCCCTCCCAGCTCTCCAAGTGGCCTGGCAGCCCCACGTCACGCAGCAGT
GATGAGCTGGATGCGTGGACGGACTTCCGTTCACGCACCAATTCTAACGCCAGCACAGTC
AGTGGCCGCCTGTCGCCCATCATGGCAAGCACAGAGTTGGATGAAGTCCAGGACGATGAT
GCGCCTCTCTCGCCCATGCTCTACAGCAGCTCAGCCAGCCTGTCACCTTCAGTAAGCAAG
CCGTGCACGGTGGAACTGCCACGGCTGACTGATATGGCAGGCACCATGAATCTGAATGAT
GGGCTGACTGAAAACCTCATGGACGACCTGCTGGATAACATCACGCTCCCGCCATCCCAG
CCATCGCCCACTGGGGGACTCATGCAGCGGAGCTCTAGCTTCCCGTATACCACCAAGGGC
TCGGGCCTGGGCTCCCCAACCAGCTCCTTTAACAGCACGGTGTTCGGACCTTCATCTCTG

AACTCCCTACGCCAGTCTCCCATGCAGACCATCCAAGAGAACAAGCCAGCTACCTTCTCT
TCCATGTCACACTATGGTAACCAGACACTCCAGGACCTGCTCACTTCGGACTCACTTAGC
CACAGCGATGTCATGATGACACAGTCGGACCCCTTGATGTCTCAGGCCAGCACCGCTGTG
TCTGCCCAGAATTCCCGCCGGAACGTGATGCTTCGCAATGATCCGATGATGTCCTTTGCT
GCCCAGCCTAACCAGGGAAGTTTGGTCAATCAGAACTTGCTCCACCACCAGCACCAAACC
CAGGGCGCTCTTGGTGGCAGCCGTGCCTTGTCGAATTCTGTCAGCAACATGGGCTTGAGT
GAGTCCAGCAGCCTTGGGTCAGCCAAACACCAGCAGCAGTCTCCTGTCAGCCAGTCTATG
CAAACCCTCTCGGACTCTCTCTCAGGCTCCTCCTTGTACTCAACTAGTGCAAACCTGCCC
GTCATGGGCCATGAGAAGTTCCCCAGCGACTTGGACCTGGACATGTTCAATGGGAGCTTG
GAATGTGACATGGAGTCCATTATCCGTAGTGAACTCATGGATGCTGATGGGTTGGATTTT
AACTTTGATTCCCTCATCTCCACACAGAATGTTGTTGGTTTGAACGTGGGGAACTTCACT
GGTGCTAAGCAGGCCTCATCTCAGAGCTGGGTGCCAGGC
>Macaque_FOXO3a
ATGGCAGAGGCACCGGCCTCCCCGGCCCCGCTCTCTCCGCTGGAAGTGGAGCTGGACCCG
GAGTTCGAGCCCCAGAGCCGTCCGCGCTCCTGTACGTGGCCCCTGCAAAGGCCGGAGCTC
CAAGCGAGCCCTGCTAAGCCCTCGGGGGAGACGGCCGCCGACTCCATGATCCCCGAGGAG
GAGGACGATGAAGACGACGAGGACGGCGGGGGACGGGCCGGCTCGGCCATGGCGATCGGC
GGCGGCGGGAGCGGCACGCTGGGCTCCGGGCTGCTCCTTGAGGACTCGGCCCGGGTGCTG
GCACCCGGAGGGCAAGACCCCGGGTCTGGGCCAGCCACCGCGGCGGGCGCTCTGAGCGGG
GGTACACAGGCGCTGCTGCAGCCTCAGCAGCCGCTGCCACCGCCGCAGCCGGGGGCGGCT
GGGGGCTCCGGGCAGCCGAGGAAATGCTCGTCGCGGCGGAACGCCTGGGGAAACCTGTCC
TACGCGGACCTGATCACCCGCGCCATCGAAAGCTCCCCGGACAAACGGCTCACTCTGTCC
CAGATCTACGAGTGGATGGTGCGTTGCGTGCCCTACTTCAAGGATAAGGGCGACAGCAAC
AGCTCTGCCGGCTGGAAGAACTCCATCCGGCACAACCTGTCGCTGCATAGTCGATTCATG
CGGGTCCAGAATGAGGGAACTGGCAAGAGCTCTTGGTGGATCATCAACCCGGATGGGGGG
AAGAGCGGAAAAGCCCCCCGGCGGCGGGCTGTCTCCATGGACAACAGCAACAAGTATACC
AAGAGCCGTGGCCGCGCAGCCAAGAAGAAGGCAGCCCTGCAGACAGCCCCTGAATCAGCT
GACGACAGTCCCTCCCAGCTCTCCAAGTGGCCTGGCAGCCCCACGTCACGCAGCAGTGAT
GAGCTGGATGCGTGGACGGACTTCCGTTCACGCACCAATTCTAACGCCAGCACAGTCAGT
GGCCGCCTGTCGCCCATCATGGCAAGCACAGAGTTGGATGAAGTCCAGGACGACGATGCA
CCTCTCTCGCCCATGCTCTACAGCAGCTCAGCCAGCCTGTCACCTTCAGTGAACAAGCCA
TGCACGGTGGAACTGCCACGGCTGACTGATATGGCAGGCACCATGAATCTGAATGATGGG
CTGACTGAGAACCTCATGGACGACCTGCTGGATAACATCACGCTCCCGCCATCCCAGCCA
TCACCCACTGGGGGACTTATGCAGCGGAGCTCTAGCTTCCCATACACCACCAAGGGCTCG
GGCCTGGGCTCCCCAACCAGCTCCTTCAACAGCACGGTGTTTGGACCTTCATCTCTGAAC
TCCCTACGCCAGTCTCCCATGCAGACCATCCAAGAGAACAAGCCAGCTACCTTCTCTTCC
ATGTCACACTATGGTAACCAGACACTCCAGGACCTGCTCACTTCGGACTCACTTAGCCAC
AGCGATGTCATGATGACACAGTCGGACCCCTTGATGTCTCAGGCCAGCACCGCTGTGTCT
GCCCAGAATTCCCGCCGGAACGTGATGCTTCGCAATGACCCAATGATGTCCTTTGCTGCC
CAGCCTAACCAGGGAAGTTTGGTCAATCAGAACTTGCTCCACCACCAGCACCAAACCCAG
GGCGCTCTTGGTGGCAGCCGTGCCTTGTCGAATTCTGTCAGCAACATGGGCTTGAGTGAG
TCCAGCAGCCTTGGGTCAGCCAAACACCAGCAGCAGTCTCCTGTCAGCCAGTCTATGCAA
ACCCTCTCGGACTCTCTCTCAGGCTCCTCCTTGTACTCAACTAGTGCAAACCTGCCCGTC
ATGGGCCATGAGAAGTTCCCCAGCGACTTGGACCTGGACATGTTCAATGGGAGCTTGGAA

TGTGACATGGAGTCCATTATCCGTAGCGAACTCATGGATGCTGATGGGTTGGATTTTAAC
TTTGATTCCCTCATCTCCACACAGAATGTTGTTGGTTTGAACGTGGGGAACTTCACTGGT
GCTAAGCAGGCTTCATCTCAGAGCTGGGTGCCAGGC
>Medaka_FOXO3a
ATGGCCGAGGCCCCGCTTCCCGACGCGCTACCGGACTTGGACGTGGCCATCGACCCGGAC
TTTGAGCCTCAAAAGCGGCCCAGATCCTGCACTTGGCCGCTTCCACGGCCGGACTCCGCT
GCGGTGAAGCCGGAGAGCAACGACACAGATATTATACCGGAGGAGGAAGACGATGAGGAG
GATGCTACCCCCACAGCTGCACGCGTCAACGGCTCCGTTGCAGCCGCGGAGGACCAGAGC
AGCAACAGCCCCGTGGGTGATGGAGCGCTGCCTTCCCCGGTCAAGAGAGCGGAGGCTCG
CCGCTCTCCTCGCACTCCCCGGCGGCCACCTCCGGCGCCTTGACTCCCAGCGGCTTGGCT
GCAGCGCAGACCCCGAGGAAGACCTCCTCCCGCCGCAACGCCTGGGGGAACCTCTCCTAC
GCCGACCTGATCACCCAGGCCATTGAGAGTTCGCCCGAGAAGAGGCTGACTCTGTCCCAG
ATTTATGACTGGATGGTGAGATCCATCCCCTACTTCAAAGACAAAGGCGACAGCAACAGC
TCTGCGGGATGGAAGAACTCCATCCGGCACAACCTCTCCCTCCACAGTCGGTTCATTCGC
GTCCAAAACGAAGGGACAGGAAAGAGTTCCTGGTGGATGATCAATCCGGAAGGAGGAAAA
GGCGGCAAGGCTCCAAGACGTCGGGCTGTTTCCATGGACAACAGCAACAAGTACACCAAG
TCCGCCCGTGGTCGTGCTGCTAAGAAAAAGGCTGCACTGCAGGCTGCTGCTGCCGCAGCA
GGTGAAGGTGGTGGAGACAGTCCTTCAGGTCTTTCCAGGTGGCCTGGGAGTCCAACATCC
CACAGCACAGAGGAGCTTGATGGTGGATGGACAGATTTCCGCTCTCGCACCAACTCCAAT
GCCAGCACTGTTAGTGGCCACCTCTCACCCATTATGGCCAACCCTGAACTTGACGAAGTA
CCCGATGATGAGGCTCCTCTCTCCCCCATGATCTACTCCAGTCCTGGCAGGGCGCTGTCT
CCTGGAAATGCCATATCCAACGGGAAAGCAGCCCCTGCTGAGCTGCCCCGCCTAGCGGAC
CTGACAGGTACGATGAACTTGAATGATGGAATCACAGATGAACTGATGGACGATTTGCTT
GACAACATCAAGTTGGTACCACCTTCCTCCAGCCAGAACCTCCAAAATGGCTCTTCAGGC
TTCACTTTTGGGTCCAAATCCAATGGAATAAGCTTAACTTCCTCCACATCCTCTCCATCG
TCCAACTCTTCATCTAATGGTGGAAGTAATGGTTACAGTAACTCCATCTTTGGCTCCCAA
GCAATAGGTTCCTCTCTGCGCTCCTCCCCAATGCAAACCATCCAGGAGAACAAGCAAACA
TCTTTCTCCAGCATCAGCATGTCTGGTTTTGGCAGCCAAACACTACAAGACCTGCTTAAC
TCTGGTAGTCACAACCATAGTGATGTGATGATGACCCAGTCTGACCCTTTGATGTCGCAA
GCCAGCTCAGCTGCTGTCAATTCCCAAAACTCCCGCCGTGGTCTGATCCTCCGTAACGAT
CCTGTGATGACCTTTGGGACCAGTGGAGGTATTCAAGCCAGCCAGAGGGACGTGCTACAA
ACTAACAACCAAAACCAGAGCTCCATGAGGTCAAGAAATGGAGGTCTGAACCTCGTCAAC
GAGGCTAATACTCTGGCTAATGCTAAACAGCAGCTCCTGCTCTCACCATTAGGAGGAAAT
GGTTCGTCCACCATGCAGATCGACCCCTCCATCTTTCTTAACGGCACCGTCAGCAGCAGC
GGAGGAGTTTCTCAGGATCGCTTCCCCACCGATCTGGACCTGGACATATTCAACGGCAGC
CTGGAGTGTGACATGGACTCCATCATCAGAAACGACCTGATGGATGCAGATGGCCTGGAT
TTTAACTTTGACTCTCTGGTAAACATGAACGGAGTAAGCAACTTCACGAGCACCAAGCAG
AGCTCTCAGAGCTGGGTACCTGGC
>Mouse_FOXO3a
ATGGCAGAGGCACCAGCCTCCCCGGTCCCGCTCTCTCCGCTCGAAGTGGAGCTGGACCCA
GAGTTCGAGCCACAGAGTCGGCCACGCTCCTGTACGTGGCCCCTGCAGAGGCCGGAGCTG
CAGGCGAGCCCGGCCAAGCCCTCGGGGGAGACGGCCGCAGACTCCATGATCCCCGAGGAG
GACGACGATGAAGACGACGAGGACGGCGGCGGCCGAGCCAGCTCGGCCATGGTGATCGGT
GGCGGCGTGAGCAGCACGCTGGGTTCCGGGCTGCTCCTCGAGGATTCGGCCATGCTGCTG

GCTCCAGGAGGGCAGGACCTCGGGTCGGGGCCAGCGTCCGCCGCAGGCGCTCTGAGTGGG
GGCACGCCGACGCAGCTGCAGCCTCAGCAGCCACTGCCACAGCCGCAGCCGGGGGCGGCT
GGGGGCTCTGGGCAACCAAGGAAATGCTCCTCGCGGCGGAATGCCTGGGGGAACCTGTCC
TATGCCGACCTGATCACCCGCGCCATCGAGAGCTCCCCGGACAAACGGCTCACTTTGTCC
CAGATCTACGAGTGGATGGTGCGCTGTGTGCCCTACTTCAAGGATAAGGGCGACAGCAAC
AGCTCTGCGGGCTGGAAGAACTCCATCCGGCACAACCTGTCCCTGCACAGCCGCTTCATG
CGCGTTCAGAATGAAGGCACGGGCAAGAGCTCTTGGTGGATCATCAACCCCGATGGGGGA
AAGAGCGGGAAGGCCCCCCGGCGGCGTGCGGTCTCCATGGACAACAGCAACAAGTACACC
AAGAGCCGAGGCCGGGCAGCCAAGAAGAAGGCGGCCCTGCAGGCTGCCCCAGAGTCGGCA
GACGACAGTCCTTCCCAGCTCTCCAAGTGGCCTGGCAGCCCCACGTCCCGCAGCAGCGAC
GAGCTGGATGCGTGGACCGACTTCCGCTCGCGCACCAATTCCAACGCCAGCACCGTGAGC
GGCCGCCTGTCGCCCATCCTGGCAAGCACGGAGCTGGATGACGTCCAGGATGATGATGGA
CCCCTGTCCCCCATGCTGTACAGCAGCTCTGCCAGCCTGTCGCCCTCCGTGAGCAAGCCG
TGTACTGTGGAGCTTCCGCGGCTGACGGACATGGCCGGCACCATGAATCTGAATGATGGG
CTGGCCGAGAACCTCATGGACGACCTGCTGGATAACATCGCGCTCCCGCCATCGCAGCCA
TCGCCTCCTGGCGGGCTTATGCAGCGGGGCTCCAGCTTCCCATATACCGCCAAGAGCTCC
GGCCTGGGCTCCCCAACCGGCTCCTTCAACAGTACCGTGTTTGGACCTTCGTCTCTGAAC
TCCTTGCGTCAGTCACCCATGCAGACTATCCAGGAGAACAGACCAGCCACCTTCTCTTCC
GTGTCACACTACGGCAACCAGACACTCCAAGACCTGCTTGCTTCAGACTCACTCAGCCAC
AGCGACGTCATGATGACCCAGTCGGACCCCTTGATGTCTCAGGCTAGCACCGCCGTGTCC
GCCCAGAATGCCCGCCGGAACGTGATGCTTCGCAACGATCCAATGATGTCCTTTGCTGCC
CAGCCTACCCAGGGGAGTTTGGTCAATCAGAACTTGCTCCACCACCAGCACCAAACCCAG
GGCGCTCTTGGTGGCAGCCGTGCCTTGTCAAATTCTGTCAGCAACATGGGCTTGAGTGAC
TCCAGCAGCCTTGGCTCAGCCAAACACCAGCAGCAGTCTCCCGCCAGCCAGTCTATGCAA
ACCCTCTCGGACTCTCTCTCAGGCTCCTCACTGTATTCAGCTAGTGCAAACCTTCCCGTC
ATGGGCCACGATAAGTTCCCCAGTGACTTGGACCTGGACATGTTCAATGGGAGCTTGGAA
TGTGACATGGAGTCCATCATCCGTAGTGAACTCATGGATGCTGACGGGTTGGATTTTAAC
TTTGACTCCCTCATCTCCACACAGAACGTTGTTGGTTTGAATGTGGGGAACTTCACTGGT
GCTAAGCAGGCCTCATCTCAAAGCTGGGTACCAGGC
>Naked_mole_rat_FOXO3a
ATGGCAGAGACATCGGCCTCCCCGATCCCGCTCTCTCCTCCCGAAGTGGAGGTGGACCCC
GAGTTTGAGCCCCAGAGCCGCCCGCGCTCTTGTACGTGGCCCCTGCAGAGGCCGGAGCTC
CAGGCCAGCCCGGCTAAGCCCTCGGGGGAGACGGCCGCCGACTCCATGATCCCGGAGGAG
GAGGACGATGAAGACGACGAGGACAGCGGCCGGACCAGCTCGGCCATGGCGATCGGCGGT
GGCGGGAGCGGAACCCTGGGCTCCGGGCTGCTTCTTGAAGACTCGGCCCGACTGCTGGCT
TCCGGAGGGCAGGACTTCGGGTCCGGGCCAGCTCCCGCGGTGGGCGCGCTGAGCGGGGGA
ACCCAGACGCCGCTGCAGCCTCAGCAGCCACTGCCAGCGCCGCAACCCGGGGCAGCTGGG
AGCTCCGGGCAGCCGAGGAAGTGCTCTTCGAGACGGAACGCCTGGGGAAACCTGTCCTAC
GCCGACTTGATCACCCGAGCCATCGAGAGCTCCCCGGACAAACGGCTCACTCTGTCCCAG
ATCTACGAATGGATGGTGCGATGTGTGCCCTACTTCAAAGACAAGGGCGACAGCAACAGC
TCTGCGGGCTGGAAGAACTCTATCCGGCATAACTTGTCTCTGCACAGTCGATTCATGAGG
GTCCAGAATGAGGGGACTGGCAAGAGCTCATGGTGGATCATCAATCCTGATGGGGGCAAG
AGTGGCAAGGCTCCCCGGCGGCGGGCCGTCTCCATGGACAACAGCAACAAGTATACCAAG
AGCCGTGGCCGAGCAGCCAAGAAGAAGGCGGCCTTGCAGACTGCCCCAGAGTCTGCAGAT

GACAGTCCTTCCCAGCTCTCTAAGTGGCCTGGCAGCCCCACCTCACGCAGCAGCGACGAG
CTTGATGCGTGGACGGACTTCCGTTCACGTACCAATTCCAATGCCAGCACAGTGAGCGGC
CGTCTGTCACCCATCCTGGCAACCACAGAGCTGGATGATGTCCAAGATGACGATGCACCG
CTCTCCCCAATGCTCTACAGCAGCTCAGCCAGCCTGTCACCCTCTGTAAGCAAGCCATGC
ACCGTGGAGCTGCCACGGCTGACTGACATGGCAGGCACCATGAATCTGAATGATGGGCTG
GCTGATAACCTCATGGATGACCTGTTGGATAACATCACGCTGCAGTCATCCCAGCCATCG
CCCACTGGGGCGCTCATGCAGCGCAGCTCCAGCTTCCCATACACCACCAAGGGCTCTGGC
CTAGGCTCTCCAACCAGCTCCTTTAACAGCACAGTGTTTGGACCCTCGTCTCTGAACTCC
TTGCGCCAGTCTCCCATGCAGACCATCCAAGAGAACAAGCCAGCCACCTTCTCTTCCATG
TCACACTATGGCAACCAGACACTCCAAGACCTGCTCACTTCTGACTCGCTCAGCCACAGC
GATGTCATGATGACTCAGTCAGATCCCTTGATGTCCCAGGCCAGCACTGCTGTGTCTGCC
CAGAACTCCCGCCGGAATGTGATGCTTCGCAATGACCCAATGATGTCCTTTGCTGCCCAG
CCTAATCAGGGGAGTTTGGTCAATCAGAACTTGCTCCACCACCAGCACCAAACCCAGGGC
GCTCTAGGTGGCAGCCGTGCCTTGTCGAATTCTGTCAGCAACATGGGCTTGAGTGACTCC
AGCAGCCTCGGGTCAGCCAAACACCAGCAGCAGTCTCTTGTCAGCCAGTCTATGCAAACC
CTCTCGGACTCCCTCTCAGGCTCTTCCTTGTACTCAGCTAGTGCAAACCTTCCCGTCATG
GGCCATGAGAAGTTCCCCAGCGACTTGGACCTGGACATGTTCAATGGGAGCTTGGAATGT
GACATGGAGTCCATTATCCGAAGTGAACTCATGGATGCAGATGGGTTGGATTTTAACTTT
GATTCCCTCATCTCCACACAGAATGTTGTTGGTTTGAATGTGGGGAACTTCACAGGTGCT
AAGCAGGCCTCATCTCAGAGCTGGGTGCCAGGC
>Opossum_FOXO3a
ATGGCAGAGGCGCCGTCCTCTCCGGCCCCGCTGTCGCCGCTGGAAGTGGAGCTGGACCCA
GAGTTCGAGCCCCAGAGCCGGCCCCGCTCCTGTACGTGGCCCCTGCAGAGGCCCGAGCTG
CAGGCGAGCCCCGCCAAGCCCTCGGGGGAAGCGTCCGCCGACTCCATGATCCCCGAGGAG
GAGGACGACGAGGAAGACGACGAAGGAGGCTGCGGAGTCCCTGGGGGCGGCGGAGGAGCT
GGCACGGCCATGGCCATGGGCGGTGCGAGCGGCGTTCTCGGCCCGGGACTGCGCCCCGAG
GAGGCGGCCCGGCTCTTGGCCCCTAGCGGGCAAGACTCGGGGGCCGGTGCGCCCCCCGGG
GCGGGCGTGTTGAGCGGGGGGCAGCAGGCGCCGCTGCAGCCTCCGCAGGCGCCGCTGCCG
CTGCCGCCGCCGCCGCCACCGCCGCCGCTGCAGCAGCAGCAGCCGCCGCAGCAACAGGGG
GCGAGCGGGGCCGCGGGGCAGCCGAGGAAATGCTCTTCCCGGAGGAACGCGTGGGGAAAC
CTGTCCTACGCCGACCTGATCACTCAGGCCATCGAAAGCTCTCCGGACAAGCGGCTCACC
CTGTCCCAGATCTATGACTGGATGGTGCGCTGCGTGCCCTACTTCAAGGATAAGGGCGAC
AGCAACAGCTCGGCCGGCTGGAAGAACTCTATCCGGCACAACTTGTCCCTACACAGTCGA
TTCAAAAGGGTACAAAACGAAGGGACCGGGAAAAGTTCTTGGTGGATGATCAATCCGGAC
GGGGGCAAGAGCGGAAAGGCCCCCGGCGCAGAGCCGTCTCCATGGACAACAGCAACAAG
TACACCAAGAGCAGGGGGCGGGCGGCTAAGAAAAAGGCGTCGCTGCAGGCCGCCCAGGAG
GCGCCCGAGGACAGCCCGTCGTCGTCGTCGCAGCTGGCCAAGTGGCCGGGCAGCCCCACC
TCCCGGAGCAGCGACGAGCTGGACACCTGGACGGACTTCCGCTCCCGGACCAACTCCAAC
GCGAGCACGATCAGCGGGCGCCTGTCGCCCATCCTGGCCACGTCGGAGCTGGATGACGTG
CAGGACGACGACCCCCCGCTCTCGCCGATGCTGTACAGCAGCTCGTCCAGCCTGTCCCCG
TCGGTCAGCAAACCGTGCACGGTGGAGCTCCCGCGGCTCACCGACATGGCGGGCACCATG
AACCTCAACGACGGCCTGACGGACAACCTCATGGACGACCTGCTGGACAACATCACGCTG
CCGCCCTCGCAGCCGTCGCCGTCGGCGGGCCTCATGCAGCGGAGCTCCAGCTTCCCGTAC
GCCGCCAAGGGCTCCGGCCTGGGCTCCCCGTCCGGCGGCTTCGGCAACGCCGTGTTCGGC

CCCTCGTCGCTGAACTCCCTGCGCCAGTCCCCCATGCAGACCATCCAGGAGAACAAGGCG
GCCACCTTCTCTTCCATGTCGCACTACGGCAACCAGACGCTGCAGGACCTGCTGACGTCG
GACTCCCTGAGCCACAGCGACGTCATGATGACCCAGTCGGACCCGCTCATGTCCCAGGCC
AGCACGGCCGTGTCGGCCCAGAACTCCCGCAGGAGCGTCATGCTCCGCAGCGACCCGATG
ATGTCCTTCGCGGCCCAGTCCGGCCAGGGGGGTCTGGTGGCTCAGAACCTCCTGCACCAG
CAGCACCCGGCCCAGAGTCCTCTCGGCGGCAACCGTGCCTTGTCTAGCGCCGGCGGCGGC
CTGGGCCTGAGCGACGCCGGCGGCCTGGGCTCGGCCAAGCACCAGCAGCCGTCGCCCGCC
GGCCAGTCTATGCAAACCCTTTCTGACTCACTCTCAGGGTCCTCTTTGTATTCCACTAGC
GTAAACCTCCCCGTCATGGGCCATGACAAATTCCCCAGCGACTTGGACTTGGATATTTTT
AATGGGAGCTTGGAATGTGACATGGAATCCATCATTCGCAGTGAACTCATGGATGCCGAT
GGGTTGGATTTTAACTTTGATTCCCTCATCTCCGCTCAGAACGTTGTCACTCTGAATGTG
GGGAACTTCACTGGTGCTAAGCAGGCCTCATCTCAGAGTTGGGTGCCGGGC
>Orangutan_FOXO3a
ATGGCAGAGGCACCGGCCTCCCCGGCCCCGCTCTCTCCGCTCGAAGTGGAGCTGGACCCG
GAGTTCGAGCCCCAGAGCCGTCCGCGATCCTGTACGTGGCCCCTGCAAAGGCCGGAGCTC
CAAGCGAGCCCTGCCAAGCCCTCGGGGGAGACGGCCGCCGACTCCATGATCCCCGAGGAG
GAGGACGATGAAGACGACGAGGACGGCGGGGGACGGGCCGGCTCGGCCATGGCGATCGGC
GGCGGCGGGAGCGGCACGCTGGGCTCCGGGCTGCTCCTTGAGGACTCGGCCCGGGTGCTG
GCACCCGGAGGGCAAGACCCGGGGTCTGGGCCAGCCACCGCGGCGGGCGCGCTGAGCGGG
GGTACACAGGCGCTGCTGCAGCCTCAGCAACCACTGCCACCGCCGCAGCCGGGGGCGGCT
GGGGGCTCCGGGCAGCCAAGGAAATGTTCGTCGCGGCGGAACGCCTGGGGAAACCTGTCC
TACGCGGACCTGATCACCCGCGCCATCGAGAGCTCCCCGGACAAACGACTCACTCTGTCC
CAGATCTACGAGTGGATGGTGCGTTGCGTGCCCTACTTCAAGGATAAGGGCGACAGCAAC
AGCTCTGCCGGCTGGAAGAACTCCATCCGGCACAACCTGTCACTGCATAGTCGATTCATG
CGGGTCCAGAATGAGGGAACTGGCAAGAGCTCTTGGTGGATCATCAACCCTGATGGGGGG
AAGAGCGGAAAAGCCCCCCGGCGGCGGGCTGTCTCCATGGACAACAGCAACAAGTATACC
AAGAGCCGTGGCCGCGCAGCCAAGAAGAAGGCAGCCCTGCAGACAGCCCCCGAATCAGCT
GACGACAGTCCCTCCCAGCTCTCCAAGTGGCCTGGCAGCCCCACGTCACGCAGCAGTGAT
GAGCTGGATGCGTGGACGGACTTCCGTTCACGCACCAATTCTAACGCCAGCACAGTCAGT
GGCCGCCTGTCACCCATCATGGCAAGCACAGAGTTGGATGAAGTCCAGGACGACGATGCG
CCTCTCTCGCCCATGCTCTACAGCAGCTCAGCCAGCCTGTCACCTTCAGTAAGCAAGCCG
TGCACGGTGGAACTGCCACGGCTGACTGATATGGCGGGCACCATGAATCTGAATGATGGG
CTGACTGAAAACCTCATGGACGACCTGCTGGATAACATCACGCTCCCGCCATCCCAGCCA
TCGCCCACTGGGGGACTCATGCAGCGGAGCTCTAGCTTCCCATATACCACCAAGGGCTCA
GGCCTGGGCTCCCCAACCAGCTCCTTTAACAGCACGGTGTTTGGACCTTCATCTCTGAAC
TCCCTACGCCAGTCTCCCATGCAGACCATTCAAGAGAACAAGCCAGCTACCTTCTCTTCC
ATGTCACACTATGGTAACCAGACACTCCAGGACCTGCTCACTTCGGACTCACTTAGCCAC
AGCGATGTCATGATGACACAGTCGGACCCCTTGATGTCTCAGGCCAGCACCGCTGTGTCT
GCCCAGAATTCCCGCCGGAACGTGATGCTTCGCAATGATCCGATGATGTCCTTTGCTGCC
CAGCCTAACCAGGGAAGTTTGGTCAATCAGAACTTGCTCCACCACCAGCACCAAACCCAG
GGCGCTCTTGGTGGCAGCCGTGCCTTGTCGAATTCTGTCAGCAACATGGGCTTGAGTGAG
TCCAGCAGCCTTGGGTCAGCCAAACACCAGCAGCAGTCTCCTGTCAGCCAGTCTATGCAA
ACCCTCTCGGACTCTCTCTCAGGCTCCTCCTTGTACTCAACTAGTGCAAACCTGCCCGTC
ATGGGCCATGAGAAGTTCCCCAGCGACTTGGACCTGGACATGTTCAATGGGAGCTTGGAA

TGTGACATGGAGTCCATTATCCGTAGTGAACTCATGGATGCTGATGGGTTGGATTTTAAC
TTTGATTCCCTCATCTCCACACAGAATGTTGTTGGTTTGAACGTGGGGAACTTCACTGGT
GCTAAGCAGGCCTCATCTCAGAGCTGGGTGCCAGGC
>Western_painted_turtle_FOXO3a
ATGGCAGAGGCGTCTCCCCCTGCCCCCCTCTCCCCTCTGGACGTGGAGCTGGACCCCGAG
TTCGAGCCCCAGAGCCGGCCCCGCTCCTGCACTTGGCCCCTACAAAGGCCTGAGTTGCAG
GCGAGCCCGGCCAAGCCCTCGGGGGAAGCGGCCGCGGACGCCGCCTCCATGATCCCCGAG
GAGGAGGACGACGAGGAGGAAGGGGGCAGCTCGCCCATGGCCATCGGCAGTGCCGTCCCC
GGCGGAGGGGAAGCGCTGGCTCCGGAGGAGGCGGCCCGGCTGCTGACCCCGCTGTCCGGG
GTCGGGACGGAGGGCTCGGGCCAGGCCCCCGGGGCAGCGGCGGCTGGGGGCAGCGGGCTG
AGTGGGGGGCAGCCGGCGGCGGCGCCGCGGAAATGCTCGTCCCGGCGGAACGCGTGGGGC
AACCTGTCCTACGCCGACCTGATCACCCGCGCCATCGAGAGCGCCCCGGACAAGCGGCTC
ACCCTGTCCCAGATCTACGACTGGATGGTGCGCTGCGTGCCCTACTTCAAGGATAAGGGC
GACAGCAACAGCTCGGCCGGCTGGAAGGTCTTTTTCTTGCCTTGCATCTTTTCTTGGTCT
GTGAAGATGTTTACTCTAAGAATAGGCAGTGGCACCCTCTGGCTTTTGCTGTCTACACAG
CTTCAATCGGTCCTGCTGATACAGAGTCCTTGTAACTCAATCCGGCATAATTTGTCACTT
CACAGTCGATTCATCAGGGTACAGAATGAAGGAACTGGGAAAAGCTCTTGGTGGATGATT
AATCCAGATGGTGGAAAAGGTGGGAAGCCCCCACGGAGACGCGCTGTTTCAATGGACAAT
AGCAACAAATACACAAAGAGCAGAGGCCGAGCAGCTAAGAAAAAGGCAGCCCTGCAAGCT
GCACAAGAAGCTACCGAGGACAGCCCGTCTCAACTTTCCAAGTGGCCAGGGAGCCCAACT
TCTCGCAGCAGTGATGAGATGGATGCTTGGACAGATTTCCGCTCTCGTACAAATTCAAAT
GCCAGTACAATAAGTGGCCGTTTGTCACCGATATTGGCGAGTACCGAACTAGATGACGTT
CAGGATGATGATGCTCCACTTTCTCCAATGCTGTACAATAGTCCATCAAGCATGTCCCCA
TCGGTAAATAAACCATGTACTGTTGAGTTGCCTAGGTTGACTGATATGGCAGGCACAATG
AACTTGAATGATGGACTGACAGATAACCTCATCATGGATGATCTTTTGGACAATATAACG
CTCCCCTCTCCCCAGCAGTCACCATCAGGGGGGCTCATGCAAAGAAGCTCCAGTTTTCCA
TATGGTTCCAAAGGTTCCGGACTTGGTTCTCCATCAAGTAATTTCAACAGTGCTGTGTTT
GGACCATCATCTCTGAATTCCCTTCGTCAGTCTCCCATGCAGACCATTCAAGAGAACAAG
CAAGCTACCTTTTCTTCCATTTCTCATTATAACAACCAGACGCTGCAGGATCTGCTGGCA
TCTGAGTCACTTAGTCACAGTGATGTCATGATGACGCAGTCTGATCCACTCATGTCTCAA
GCCAGCACTGCTGTGTCTGCCCAGAATTCACGCAGGAGTATCATGCTTCGTAGTGATCCA
ATGATGTCATTTGCTGCTCAGTCCAACCAGGGAAGTTTGGTCAATCAGAACCTGCTCCAC
CACCAGCATCAATCTCAGAATTCTTCTCTTGGTGGCAGTCGTGCCTTGTCAAATTCCATC
AGTAACATGGGCTTAAATGATACGAACAACTTAGGGTCAGCCAAACACCAGCAGCAGTCA
CCCATCAATCAGTCTATGCAAACCCTTTCTGACTCACTCTCAGGCTCTTCTTTGTATTCC
ACTAGTGTGAACCTTCCAGTCATGGGGCATGATAAATTCCCAAGCGATTTGGACCTGGAT
ATTTTCAATGGAAGCTTGGAATGTGACATGGAGTCCATTATCCGCAGTGAACTCATGGAT
GCAGATGGGTTGGATTTTAACTTCGATTCCCTCATCTCAGCTCAGAACGTTGTCAGTCTG
AATGTGGGGAGCTTCACTGGTGCTAAGCAGGCTTCGTCACAGAGTTGGGTGCCAGGC
>Panda_FOXO3a
ATGGCAGCAGTGGGAGCAAGGAGTGACAAGAGCCAGTCCGCGCTCGGACCCACGTTCGTC
CTCCTGGCCCGCCGGCGAAGAGGGGAGAAGGTGGCTGCCCGCGCCGTCCCCGGACTCGGG
CCCCTCAGCGGGGAGATGGGAGTACAAGGGGGCGCGGAGGCGGCAGGCTCCGGGCAGCCG
AGGAAATGCTCTTCGCGGCGGAACGCCTGGGGGAACCTGTCCTACGCCGACCTGATCACT

CGCGCCATCGAGAGCTCGCCGGACAAACGGCTCACTCTGTCCCAGATCTACGAGTGGATG
GTGCGCTGCGTGCCCTACTTCAAGGATAAGGGCGACAGCAACAGCTCTGCGGGGTGGAAG
AACTCTATCCGGCACAACCTGTCACTGCACAGCCGGTTCATGCGGGTCCAGAATGAGGGG
ACTGGCAAGAGCTCTTGGTGGATCATCAACCCTGATGGGGGAAAGAGTGGGAAGGCACCC
CGGCGGCGGGCTGTCTCCATGGACAACAGCAACAAGTATACCAAGAGCCGTGGCCGTGCA
GCCAAAAAGAAGGCAGCCCTGCAGACAGCCCCTGAGTCAGCAGATGATAGTCCCTCCCAG
CTCTCCAAGTGGCCTGGCAGCCCCACGTCACGCAGCAGCGATGAGCTGGATGCATGGACA
GACTTCCGCTCGCGCACCAATTCCAATGCCAGCACCGTCAGCGGCCGCCTGTCACCCATC
TTGGCAAGCACAGAGTTGGATGACGTCCAGGATGATGACGCGCCTCTCTCCCCCATGCTC
TACAGCAGCTCAGCTAGCCTCTCACCCTCTGTAAGTAAGCCGTGCACCGTGGAGCTACCA
CGGCTGACCGACATGGCAGGCACCATGAATCTGAATGATGGGCTGTCTGACAACCTCATG
GATGACCTGCTGGATAACATCACGCTCCCATCGTCCCAGCCATCGCCCACTGGAGGGCTC
ATGCAGCGGAGCTCTAGCTTCCCATATACCACCAAGGGCTCCGGCCTGGGTTCTCCAACT
GGCTCCTTTAACAGCACAGTGTTTGGACCCTCGTCTCTGAATTCCCTGCGCCAGTCTCCC
ATGCAGACCATCCAAGAGAATAAGCCAGCTACCTTCTCTTCCATGTCCCACTATGGCAAC
CAGACACTCCAGGACCTGCTCACTTCAGACTCACTCAGCCACAGTGATGTCATGATGACC
CAGTCAGACCCCTTGATGTCTCAGGCCAGCACCGCTGTGTCTGCCCAGAACTCCCGCCGG
AACGTGATGCTTCGAAATGACCCAATGATGTCCTTTGCCGCCCAGCCTAACCAGGGGAGT
TTGGTCAATCAGAACTTGCTCCACCACCAGCACCAAACCCAGGGCGCTCTCGGTGGCAGC
CGTGCCTTGTCGAATTCTGTCAGCAACATGGGCTTGAGCGACTCCAGCAGCCTTGGGTCA
GTTAAACACCAGCAACAGTCTCCTGTCAGCCAGTCTATGCAAACCCTCTCGGACTCTCTC
TCAGGCTCCTCCTTGTACTCAACTAGTGCAAACCTTCCCGTCATGGGCCATGAGAAGTTC
CCCAGCGACTTGGACCTGGACATGTTCAATGGGAGCTTGGAATGTGACATGGAGTCCATT
ATCCGTAGCGAACTCATGGATGCTGATGGGTTGGATTTTAATTTTGATTCCCTCATCTCC
ACACAGAATGTTGTTGGTTTGAACGTGGGGAACTTCACTGGTGCTAAGCAGGCCTCATCT
CAGAGCTGGGTCCCAGGC
>Rock_pigeon_FOXO3a
ATGTGGTGCACCTGGGCTCGCTCCCCCCTGCGGCTTCTCCCGCGGGCGCTTGCTCACAGG
TACCGCACTGATGCTGCAGCAGTGATGCTGCGGCTGTGGCGGACTAAGGATGCAGCTGAG
ATGAAGTTTGTGGACAAAGACGACTCTGTACTGCAGTGGAAGCACTTGCAGCTGGTGTCT
GAGTTAATGGACAGGCTCCACAAGAATTCGATCCGGCACAACTTGTCACTCCACAGCCGC
TTCGTCAGAGTGCAGAATGAAGGCACTGGGAAAAGCTCTTGGTGGATGATTAATCCAGAT
GGTGGAAAAGGTGGCAAGGCGCCCCGGAGACGTGCTGTGTCAATGGACAACAGCAACAAG
TACACGAAGAGCAGAGGGCGGGCGGCTAAGAAGAAGGCAGCCCTGCAGACCGCCCAGGAG
ACGAGCGAGGACAGCCCTTCTCAGCTCTCCAAATGGCCGGGGAGTCCCACCTCCCGCAGC
AGTGATGAGCTGGATGCATGGACAGATTTTCGCTCCCGTACAAATTCAAACGCCAGTACG
ATCAGTGGCCGCTTGTCCCCGATTTTGGCAAGCACCGAGCTAGATGATGTTCAAGATGAT
GACGCTCCACTTTCTCCCATGCTCTACAGTAGCCCATCGAGCTTGTCCCCATCAGTAAAC
AAACCATGTACTGTGGAGTTGCCTAGGTTGACTGATATGGCTGGGACAATGAATTTGAAC
GATGGACTGACAGATAACCTCATGGATGATCTCTTGGACAATATAACACTCCCTCCCTCC
CAGCAGTCGCCTACAGGAGGGATAATGCAGAGAAGCTCCAGTTTTCCATATGGTTCCAAA
GGTTCAGGGCTGGGTTCCCCATCAAGTAGTTTCAACAATGCTGTGTTCGGGCCATCGTCG
CTGAATTCCCTCCGCCAGTCACCCATGCAGACCATTCAGGAGAACAAGCAGGCCACCTTC
TCTTCCATGTCTCATTACAACAACCAGACGCTGCAGGATCTCCTTGCCTCTGATGCACTT

AGTCACAGCGATGTCATGATGACACAGTCTGACCCACTCATGTCACAAGCCAGCACAGCT
GTGTCCGCCCAGAATTCCCGCAGGAATATAATGCTCCGCAACGACCCCATGATGTCATTT
GCCGCGCAGTCCAGCCAGGGCGGTCTGGTCAATCAGAGCCTGCCTCATCACCAGCACCAG
TCCCACAACTCCTCTCTTAGTGGCAGCCGTGCCTTGTCCAATTCCATCAGTAACATAGGC
TTGAGTGACTCGAACAGCTTGGGATCCTCCAAACATCAGCAGTCATCTGTCAATCAGTCT
ATGCAAACACTTTCTGACCCTCTCTCAGGCTCCTCTTTGTACTCCTCTAGTGTGAACCTC
CCGGTCATGGGACACGAGAAATTCCCAAGTGACTTGGACCTGGATATTTTCAATGGGAGC
TTGGAGTGTGACATGGAGTCCATTATCCGCAGTGAACTCATGGATGCAGATGGGCTGGAT
TTTAACTTTGATTCCCTCATCTCAGCTCAGAACGTTGTCGGTCTGAATGTGGGGAACTTC
ACTGGTGCTAAACAGGCTTCATCACAGAGTTGGGTACCAGGC
>Pig_FOXO3a
ATGGCAGAGGCGCCGGCCTCCCCGGTCCCGCTCTCTCCGCTCGAAGTGGAGCTGGACCCG
GAGTTCGAGCCCCAGAGCCGTCCGCGCTCCTGTACGTGGCCCCTGCAGAGGCCGGAGCTC
CAGGGGAGCCCGGCCAAGCCCTCGGGGGAGGCGGCCGCTGACTCCATGATCCCCGAGGAG
GAGGACGATGAAGACGACGAGGACGGCGGCGGTAGGGCCGGCTCGGCCATGGCGATCGGC
GGCGGCGGGGGCGGCCCGCTGGGCTCTGGGCTGCTCCTGGAGGACTCGGCCAGGCTGCTG
GCTCCCGGAGGGCAGGAACCCGGGTCCGGCCCAGCCTCCGCGGCGGGCGCGCTGAGCGGA
GGGACGCAGACGCCGCTGCAGCCTCAGCAGCCACTGCCACCGCCGCAGCCGGGGGCGGCT
GGGGGCTCCGGGCAGCCGAGGAAATGCTCCTCCCGGCGGAACGCCTGGGGGAACCTGTCC
TACGCCGATCTGATCACTCGCGCCATCGAGAGTTCCCCGGACAAACGGCTCACTCTGTCC
CAGATCTATGAGTGGATGGTGCGCTGCGTGCCCTACTTCAAGGATAAGGGCGACAGCAAC
AGTTCTGCCGGCTGGAAGAACTCTATCCGGCACAACCTGTCACTGCACAGCCGGTTCATG
CGAGTCCAGAACGAGGGGACCGGCAAGAGCTCTTGGTGGATCATCAACCCTGATGGGGGA
AAGAGCGGGAAGGCGCCCCGGCGGCGGGCTGTCTCCATGGACAACAGCAACAAGTACACC
AAGAGCCGCGGCCGGGCAGCCAAGAAGAAGGCCGCCCTGCAGACAGCCCCTGAGTCGGCA
GACGACAGTCCCTCCCAGCTCTCCAAGTGGCCCGGCAGCCCCACGTCCCGCAGCAGTGAT
GAGCTGGACGCCTGGACTGACTTCCGCTCGCGCACCAATTCCAACGCCAGCACGGTCAGC
GGCCGCCTGTCCCCCATCCTGGCCAGCACAGAGTTGGATGACGTCCAGGATGACGACGCG
CCGCTCTCCCCCATGCTCTACAGCAGCTCGGCCAGCCTGTCCCCCTCGGTCAGTAAGCCC
TGCACCGTGGAGCTGCCCCGGCTGACCGACATGGCCGGAACCATGAATCTCAATGACGGG
CTGGCTGACAACCTCATGGACGACCTGCTGGACAACATCGCGCTGCCCTCGTCCCAGCCA
TCGCCCCCCGGGGGGCTCATGCAGCGCAGCTCTAGCTTCCCCTACACCACCAAGGGCTCC
GGCCTGGGCTCGCCCACCAGCTCCTTCAGCAGCACAGTGTTTGGACCCTCGTCTCTGAAC
TCGCTGCGCCAGGCGCCCATGCAGACCATCCAAGAGAACAAGCCAGCCACCTTCTCTTCC
ATGTCGCACTACGGCAACCAGACACTCCAGGACCTGCTCACGTCGGACTCACTCAGCCAC
AGCGATGTCATGATGACCCAGTCGGACCCCTTGATGTCTCAGGCCAGCACCGCTGTGTCC
GCCCAGAACTCCCGCCGGAACGTGATGCTTCGCAGTGACCCAATGATGTCCTTTGCCGCC
CAGCCTAACCAGGGGAGTTTGGTCAATCAGAACTTGCTCCACCACCAGCACCAAACCCAG
GGCGCTCTCGGTGGCAGCCGTGCCTTGTCGAATTCCGTCAGCAGCATGGGCTTGAGCGAC
TCAAGCAGCCTCGGGTCAGCCAAACACCAGCAGCAGTCTCCTGTCAGCCAGTCTATGCAA
ACCCTCTCGGACTCCCTCTCAGGCTCCTCCTTGTACTCAACGAGTGCGAACCTTCCCGTC
ATGGGCCACGAGAAGTTCCCCAGCGACTTGGACCTGGACATGTTCAACGGGAGCTTGGAA
TGTGACATGGAGTCCATTATCCGTAGCGAACTCATGGATGCTGACGGGTTGGATTTTAAC
TTTGATTCCCTCATCTCCACACAGAACGTTGTTGGTTTGAACGTGGGGAGCTTCACTGGT

GCTAAGCAGGCCTCATCTCAGAGCTGGGTGCCAGGC
>Platypus_FOXO3a
AACGCCTGGGGGAACCTGTCCTACGCCGACCTCATCACCCGCGCTATAGAGAGCTCCCCG
GACAAGCGCCTCACCCTCTCCCAGATCTACGACTGGATGGTGCGCAGCGTGCCTTACTTC
AAGGATAAAGGCGACAGCAACAGCTCCGCAGGCTGGAAGAACTCAATTCGGCACAACCTG
TCCCTGCACAATCGGTTCATCAGGGTCCAGAACGAGGGGACCGGGAAAAGCTCCTGGTGG
ATGATCGACCCGGACGGTGGCAAGGGCGGGAAGGCCCCCAGGCGCCGGGCTGTCTCGATG
GACAACAGCAACAAGTACAGCAAGAGCCGCGGGCGAGCGGCCAAGAAGAAGGCCACCCTG
CAGGCCGCCCAGGAGGGGGCGGAAGACAGCCCGGGGTCGCAGCTGGCCAAGTGGCCAGGC
AGCCCCACCTCCCGCAGCAGCGACGAGCTGGATGCGTGGACAGACTTCCGCTCGCGGACC
AACTCCAACGCCAGCACGGTGAGCGGGCGCCTGTCGCCCATCCTGGCGAGCGCGGAGCTG
GACGAGGCCCAGGATGACGCCGGCCCTCCCTCACCCATGCTGTACGGCAGCCCCCCAACC
ATGTCCCCGGCCGGGGGTGCCCCGTGCATGGTGGAGCTGCCCCGGCTGACTGACATGGCG
GGGACCATGAACCTGAATGACGGGCTGGCGGCGGACGACCTCATGGACGACCTCCTGGAC
GACATCTCCCTGCCCTCCACCCAGCCATCCCCCTCGGCGGGGCTCCTGCAGCGGAGCGCC
ACCTTCCCCTATGGCCCCAAGGGCTCGGGGCTTGGCTCCCCGCCGGGTGGCTTCGGCCCT
GCGGCCTTCGGTCCCTCCCCGCTCAACTCCCTCCGCCAGTCCCCCATGCAGACCATCCAG
GAGAACAAGCCGGCCACCTTCTCGTCCCTGTCTCATTACGGCACCCAGACGCTGCAGGAC
CTGCTGGCGTCCGACTCGCTGAGCCACAGCGACGTCATGATGACACAGTCGGACCCGTTG
ATGTCACAAGCCAGCACGGCCGTGTCCGCCCAGAACCCCCGCCGGAGCGTCATGCTCCGG
AGCGACCCGATGATGTCCTTCGCCGCCCCGCCCGGCCAGGGCGGCCTGGTCCACCAGAAC
CTGCTCCACCACCAGCACCCGGCCCAGGGCTCCCTGGGCGGTGGCCGGGCACTGGCCAGT
GGCGCCGGCGGCCTGGGGTCGGGCAAGCACCAGCAGCAGCAGCAGCAGACGCAGCCGCCG
CAACAGCAGCAGCAGCCGCCGCAGCCGCCGCAGTCCCCCGTCAGCCAATCTATGCAAACC
CTTTCCGACTCCCTCTCAGGCTCCTCTTTGTATTCTTCTAGCGTGTCCCTCCCCGTCCTG
GGCCACGAGAAATTCCCCAGCGATTTGGACCTGGATATTTTCAATGGGAGCTTGGAGTGT
GACATGGAGTCCATCATCCGCAGCGAACTCATGGATGCAGACGGCTTGGATTTTAACTTT
GACTCCCTCATCTCCGCTCAGAACGTTGTCAGTCTGAATGTGGGAAACTTCACTGGTGCT
GCGCAGGCCTCCTCCCAGAGCTGGGTGCCGGGC
>Rabbit_FOXO3a
ATGGCAGAGGCGCCGGCCTCCCCGGCCCCGCTCTCTCCGCTCGAAGTGGAGCTGGACCCG
GAGTTCGAGCCCCAGAGCCGACCGCGTTCCTGCACTTGGCCCCTGCAGAGGCCGGAGCTC
CAGGCGAGCCCGGTCAAGCCCTCGGGGGAGACGGCCGCCGACTCCATGATCCCCGAGGAG
GACGACGATGAAGACGACGAGGACGGCGGCGGCCGGGCTGGCTCGGCCATGGCGATCGGC
GGCGGCGGCGGCGGCGGGAGCGGCGCGCTGGGCTCCGGGCTGCTCCTGGAGGATTCGGCC
CGCCTGCTGGCTGCCGGAGGGCAGGACCTCGGGCCCGGGCCAGCGCCCGCGGCGGGCGCG
CTGAGCGGGGGGACGCAGACTCCGCTGCAGCCTCAGCAGCCGCTGCCACCGCTGCAGCCG
GGGGCGGCTGGGGGCTCAGGGCAGCCGAGGAAATGCTCCTCGCGCCGGAACGCCTGGGGG
AACCTGTCCTACGCCGACCTGATCACTCATGCCATCCAGAGCTCCCCGGACAAGCGGCTC
ACTCTGTCCCAGATCTATGAGTGGATGGTGCGCTGTGTGCCCTACTTCAAGGATAAGGGC
GACAGCAACAGCTCCGCCGGTTGGAAGAACTCCATCCGACACAACCTGTCGCTGCACAGT
CGGTTCATGCGGGTGCAGAACGAGGGGACGGGCAAGAGCTCATGGTGGATCATCAACCCC
GATGGGGGCAAGAGCGGGAAGGCGCCCCGGCGGCGGGCCGTTTCCATGGACAACAGCAAC
AAGTACACCAAGAGCCGTGGCCGCGCCGCCAAGAAGAAGGCGGCCCTGCAGACGGCACCC

GAGGCGGCCGACGACAGCCCCTCCCAACTGGCCAAGTGGCCCGGCAGCCCCACGTCGCGC
AGCAGCGACGAGCTGGACGCCTGGACGGACTTCCGCTCGCGCACCAACTCCAACGCCAGC
ACGGTTAGCGGCCGCCTGTCGCCCATCCTGGCGAGCACAGAGCTTAATGAGGTCCAGGAC
GACGATGCTCCGCTCTCCCCGATGCTGTACAGCAGCTCCGCCAGCCTGTCGCCTTCTGCG
AGCAAGCCGTGCGCCGTGGAGCTGCCGCGGCTCACCGACATGGCGGGCACCATGAACCTG
AACGACGGGCTGGCTGACAACCTCATGGACGACCTGCTGGACAACATCCCCCTGCCGTCC
TCCCAGCCGTCACCCCCCGGGACGCTCATGCAGCGGAGCTCCAGCTTCCCCTACGCCGCC
AAGGGCTCCGGCCTGGGCTCTCCGACCGGCTCCTTTAACAGCACGGTGTTCGGGCCGTCG
TCTCTGAACTCCCTGCGCCAGTCCCCCATGCAGACCATCCAGGAGAACAAGCCAGCCACT
TTCTCTTCCATGTCGCACTACGGCAGCCAGACACTCCAAGACCTGCTCACTTCAGACTCG
CTCAGCCACAGCGACGTCATGATGACCCAGTCGGACCCCTTGATGTCTCAGGCCAGCACC
GCTGTGTCTGCCCAGAACTCCCGCCGGCACGTGATGCTGCGCAATGACCCGATGATGTCC
TTTGCTGCCCAGCCTAACCAGGGGAGTTTGGTCAATCAGAACTTGCTCCACCACCAGCAC
CAGACCCAGGGCGCTCTCGGTGGCAGCCGTGCCTTGTCGAATTCCGTCAGCAGCATGGGC
TTGAGTGACTCCAGCAGCCTCGGGTCAGCCAAACACCAGCAGCCGTCTCCCGTCAGCCAG
TCTATGCAAACCCTCTCGGACTCGCTCTCAGGCTCCTCCTTGTACTCAGCTAGTGCAAAC
CTTCCCGTCATGGGCCACGAGAAGTTCCCCAGCGACTTGGACCTGGACATGTTCAATGGG
AGCTTGGAATGTGACATGGAGTCCATTATCCGTAGTGAACTCATGGACGCGGACGGGTTG
GATTTTAACTTTGACTCCCTCATCTCCACACAGAACGTTGTTGGTTTGAACGTGGGGAGC
TTCACTGGTGCTAAGCAGGCCTCATCTCAGAGCTGGGTGCCGGGC
>Tetraodon_FOXO3a
ATGGCCGAGGCGCCGCGCGACGAGCCGCCCTCGAACGTCGAGATCGACCCGGATTTCGAG
CCTCAGAAGCGGCCGCGCTCCTGCACTTGGCCCCTTCCCCGTCCGGAGTCCGGCGGCAAG
CCCGGGACCCATGACACTGACGTAATCCCCGAAGAGGAGGACGATGAAGGCGGCAGCTCG
GGCAACGCCGAGAAAGCCTGCGCCGCCGTCGGCGTCCAGCCCCGGGAGTCGGGCGGCGGC
GGCGGCTCCGGCTCCCAGCCCCTGGAGCTCCGGCGCGGGCCCTGCAAGGAGGAGGCCGCC
GATGGATCGCCCTCCTCGGCGCAGACCCCGGCGGCCGCCGCGCTGAGCGCCTCTGCGTCC
CAGCAGCTGCGGAAGTCCTCCGCCCGTCGGAACGCGTGGGGCAACTACTCCTATGCAGAC
CTCATCACGCAAGCCATCGAGAGCTCCCCCGAGAAGAGGCTGACCTTGTCCCAGATCTAC
GACTGGATGGTCCGGTCGGTGCCATATTTCAAGGACAAAGGCGACAGCAACAGCTCTGCT
GGCTGGAAGAATTCCATCCGGCACAATCTGTCCCTCCATAGCCGATTCGTGAAGGTTCAG
AATGAGGGGACGGGAAAAAGCTCCTGGTGGATGGTCAACCCAGAAGGCGGGAAAGGAGGC
AAAGCTCCGAGACGCAGGGCCGTTTCCATGGACAACAGCAAGTACATCAAAGGAGCCCGC
GGGCGCGCCACCAAGAAGAAGGCCCTGCTGCAGGCCGCCCAGGACGGCAGCTCGGAAAGC
TCCTCCAGCCTGTCCAAGTGGACGGGAAGTCCCACCTCCCGCAGCAGCGACGAGCTGGAC
GCCTGGACGGACTTCCGCTCGCGGACCAACTCCAACGCCAGCACCCTCAGCGGCCGCCTG
TCCCCCATCCTGGCCAACCTGGAGCTGGACGAGGTGCCCGACGACGACTCGCCCCTCTCC
CCCATGTTGTACTCCAGTCCCAGCAGCATGTCCCCGTCCACCGGGCCCACGGTGCTGTCG
GATTTGGCGGGCACCATGAACCTCAACGACGGGCTCTCGGACAACCTGATGGACGACCTT
TTGGACAACATCAGCCTGACCGCCACCCAGCAGCTTCCTCCTGGAGAGGAAGACAGCGGC
GGTCAGGCCACCTCGGTGTTTACCTTCAGCTGCTCGGGAAGCAGTCTCGGCAGCCCTCTC
TTCAGCCCTCCGTCCATCACCAGCCTGCGGCAGTCCCCCATGCAGACCATCCAGGAGAAC
AAGCAGACCACCTTCTCCTGCGTCTCCCACTTCGGCGACCACCAGAGTCTCCAGGACCTG
CTCAGTCTGGACTCCCACGGCCACAGCAATGTCATGCTCACCCAGTCGGACCCGCTGATG

TCCCAGGCCAGCACCGCCGTCGCCCTGCAGAACTCCCGCCGGAACGCCATGCTTCTCCAT
AAAGACCCCCCCTCGGTGAACCACACCGGCGCAGGTCAGGCCCAAAGCTCTTCAGTGCCT
GGTTGGCAAGCTGGCTTGTCGACCTCGGAGGACAACGGCGGGCGCAACAACGCCAAACAG
CCGCACCTGAAGTCTCCCAGCAAGAATGCCTCTATGCAGCTCGGCTCTGGTTTCCCCAGC
CAGGATCGCTTCCCCGCCGACCTGGACCTCGACGTGTTCAACAGCAGCCTGGAGTGCGAC
ATGGACGCCATCATCCGTAACGAGCTGATGGACGCCGACTGCCTGGACCTCAGTTTTGAC
TCCCGCCTCACTCCCACCCAGAACGGCAACAAGAATTCAGGAAGCTACTCCGGCTCCAAA
CCGGCGGCCCCTCGCAGCTGGGTGCCGAGC
>Tilapia_FOXO3a
ATGGCTGAGGCGTCGCGCAGCGGCAACGAGCCGCCTCTAAACGTTGAGATAGATCCGGAT
TTCGAGCCCCAGAAGCGGCCACGGTCCTGCACTTGGCCCCTGCCCCGTCCTGAATCTGGT
GCGGGCAAGCCCGGGGCAAATGACACTGACGTAATCCCCGAAGAGGAGGATGATGAGGGT
GGGAGCACGGGGAGCGGCGCTGCTCAGAAAGCCAGCGGCGGCGCTATTAAAACCCGAGAG
CCGAGCAGCAACAGCTCAATCTCCCAGCCGGTGGAGGTCCAGCGGGGCCCCTCCAAAGAG
GAGACCGCCGACGGCTCGCCGTCCTCGGCGCAAACCCCCGCAGCAGCTCTGGGAGGTTCT
GCCACCCAGCAGCTGAGGAAGTCCTCCGCCCGCAGGAATGCCTGGGGCAACTACTCCTAT
GCAGACCTCATTACCCAAGCCATCGAGAGCTCCCCTGAGAAAAGGTTGACCTTGTCCCAA
ATCTATGAGTGGATGGTGAGATCCGTGCCATATTTCAAGGACAAAGGCGATAGCAACAGC
TCTGCTGGCTGGAAGAATTCCATCCGACACAATCTTTCCCTCCATAGCCGTTTTGTGAAA
GTCCAGAATGAAGGAACTGGGAAAAGCTCCTGGTGGATGGTCAACCCAGAAGGTGGAAAA
GGAGGTAAAGCTCCCAGACGTAGGGCTGTGTCGATGGACAACAGCAAGTACATCAAAGGA
GCCCGAGGACGTGCCACAAAGAAAAAAGCCACATTGCAGGCTGCTCAAGATGGGAGCTCT
GAGAGCTCCTCAAGCCTCTCCAAATGGACAGGAAGTCCCACCTCCCGCAGCAGTGACGAG
CTCGATGCCTGGACAGACTTCCGCTCTCGGACAAATTCCAACGCCAGCACGCTCAGCGGT
CGTCTGTCCCCAATCCTGGCTAACCTGGAGCTGGATGAGGTACCTGATGACGACTCGCCC
CTGTCGCCAATGCTGTACTCCAGCCCCAGCAGCATGTCTCCATCCACTGGACCAACAGGA
CTGTCTGATCTAGCAGGCACAATGAACCTCAATGACGGGCTCTCTGACAACTTGATGGAT
GATCTTTTAGACAATATCAGCCTCACAGCATCCCAGCAGCCGCCTCCTGGAGAGGAAGAT
GGGGCCAACAGTCAGGGGAGTTCAGTATTTACCTTCAGCTGCTCAGGAAGCAGTTTAGGT
AGTCCCTCTAGCAGCTATGGGACAAACCCACTCTTCAGCCCTCCGTCCATCACGAGCCTG
CGACAGTCACCCATGCAGACCATCCAAGAGAACAAGCAGACCACTTTTTCCTGCATGTCT
CACTACAGTGAGCACCAGGCCCTGGATCTGCTTGGGATGGACTCCCACAGCCACAGCAAT
GTCATGATGACCCAGTCCGACCCTCTGATGTCGCAGGCCAGCACCGCCATCGCTCTGCAG
AACTCCCGCCGAAATGCCATGCTGCTCCGCAAAGACCACATTCTGGTTAACCACACCAGT
GCAGGCCAGGCCCAAAGCTCTTCAGTGCCTGGTTGGCAAGCGGGCTTGTCAACCTCTGAC
AGTGACGACGGCCACCCTGAAACCAAGCAGCTACACCTGAAATCTCCTAGTAAGAATGCC
TCTATGCAGCTCAGTTCCAGTTTAACCAGTCAGGACCGTTTCTCCGCTGATCTGGACCTC
GAGGTGTTCAGCAGCAATCTGGACTGCGACATGGACTCCATCATCCGCAATGAACTGATG
GATGCTGATTGCCTGGACCTCAGCTTTGACTCTCGTCTCGCCTCCACACAGAACGGCAAC
AAGAATTCAGGAAGCTTCTCCAGCTCGAAACAGAGCCCTCAGAGCTGGGTGCCGAGC
>Turkey_FOXO3a
ATGTCCAGGAGGGCCCAGTGTTTTGATTCATGTCATTTTCCTGTGATGTTGGTGTTGCGA
TTGAGATTTGGAGTAGCAAAAGCAGTGCTGGTCTTGACATGTTGTTTTCTTCCTTTCCTG
CAGAATTCAATCCGGCACAACTTGTCGCTCCACAGCCGATTCATCAGGGTGCAAAATGAA

GGCACTGGGAAGAGCTCCTGGTGGATGATCAATCCAGATGGTGGAAAAGTTGGCAAGGCG
CCCCGGAGACGCGCCGTGTCTATGGACAACAGCAACAAATACACAAAGAGTAGAGGGCGG
GCAGCGAAGAAAAAGGCAGCTCTGCAGACTGCCCAGGAGGCGAGTGAGGACAGTCCTTCA
CAGCTCTCCAAGTGGCCGGGGAGCCCGACTTCCCGCAGCAGTGACGAGCTGGATGCCTGG
ACAGATTTTCGCTCCCGGACCAATTCGAACGCCAGTACAATAAGTGGGCGCTTGTCGCCG
ATTTTGGCGAGCACCGAGCTGGATGATGTTCAAGATGATGACGCTCCACTTTCTCCCATG
CTGTACAGTAGCCCTTCGAGCTTGTCCCCCTCGGTAAACAAACCATGCACTGTGGAGTTG
CCTAGGTTGACTGATATGGCTGGGACAATGAATTTGAACGATGGACTGACGGATAACCTC
ATGGATGACCTCTTGGACAATATAACACTCCCTTCCTCCCAGCAGTCGCCCACAGGAGGA
ATAATGCAGAGAAGCTCCAGTTTTCCGTATGGTTCCAAAGGTTCAGGGCTGGGCTCCCCC
TCGAGTAGTTTCAACAACGCCGTGTTTGGGCCATCGTCCCTGAATTCCCTCCGCCAGTCG
CCCATGCAGACTATTCAGGAGAACAAGCAGGCTACCTTCTCTTCCATTTCTCATTACAAC
AACCAGACGCTGCAGGATCTGCTCGCCTCAGATGCACTTAGTCACAGCGATGTCATGATG
ACACAGTCTGACCCGCTCATGTCCCAGGCCAGCACAGCTGTGTCCGCCCAGAACTCCCGC
AGGAATATAATGCTCCGCAATGACCCCATGATGTCGTTTGCTGCGCAGTCCAGTCAGGGC
GGTCTGGTCAATCAGAGCCTGCCCCATCACCAGCACCAGTCTCACAGCTCTCCTCTTAGT
GGCAGCCGTGCCTTGTCCAATTCCATCAGTAACATAGGCTTGAATGACTCCAACAGCTTG
GGATCCAAACATCAGCAATCACCTGTCAATCAGTCTATGCAAACACTTTCTGACCCTCTC
TCAGGCTCCTCTTTGTATTCCTCTAGCATGAACCTTCCGGTCATGGGACACGAGAAATTC
CCAAGTGACTTAGACCTGGATATTTTCAATGGGAGCTTGGAGTGTGACATGGAGTCCATC
ATCCGCAGTGAACTCATGGATGCAGATGGGCTGGATTTTAACTTTGATTCCCTCATCTCA
GCACAGAACGTTGTCAGTCTGAATGTGGGGAACTTCACTGGTGCTAAACAGGCTTCATCA
CAGAGTTGGGTACCAGGC
>Worm_FOXO3a
ATGATGGAGATGCTGGTAGATCAGGGAACTGATGCATCGTCATCCGCCTCCACGTCCACC
TCATCTGTTTCGAGATTCGGAGCGGACACGTTCATGAATACACCGGATGATGTGATGATG
AATGATGATATGGAACCGATTCCTCGTGATCGGTGCAATACGTGGCCAATGCGTAGGCCG
CAACTCGAACCACCACTCAACTCGAGTCCCATTATTCATGAACAAATTCCTGAAGAAGAT
GCTGACCTATACGGGAGCAATGAGCAATGTGGACAGCTCGGCGGAGCATCTTCAAACGGG
TCGACAGCAATGCTTCATACTCCAGATGGAAGCAATTCTCATCAGACATCGTTTCCTTCG
GATTTCAGAATGTCCGAATCGCCAGACGATACCGTATCGGGAAAAAAGACAACGACCAGA
CGGAACGCTTGGGGAAATATGTCATATGCTGAACTTATCACTACAGCCATTATGGCTAGT
CCAGAGAAACGGTTAACTCTTGCACAAGTTTACGAATGGATGGTCCAGAATGTTCCATAC
TTCAGGGATAAGGGAGATTCGAACAGTTCAGCTGGATGGAAGAACTCGATCCGTCACAAT
CTGTCTCTTCATTCTCGTTTCATGCGAATTCAGAATGAAGGAGCCGGAAAGAGCTCGTGG
TGGGTTATTAATCCAGATGCAAAGCCAGGAAGGAATCCACGGCGTACACGTGAACGATCC
AATACTATTGAGACGACTACAAAGGCTCAACTCGAAAAATCTCGCCGCGGAGCCAAGAAG
AGGATAAAGGAGAGAGCATTGATGGGCTCCCTTCACTCGACACTTAATGGAAATTCGATT
GCCGGATCGATTCAAACGATTTCTCACGATTTGTATGATGATGATTCAATGCAAGGAGCA
TTTGATAACGTTCCATCATCTTTCCGTCCCCGAACTCAATCGAACCTCTCGATTCCTGGA
TCGTCGTCTCGTGTTTCTCCAGCTATTGGAAGTGATATCTATGATGATCTAGAATTCCCA
TCATGGGTTGGCGAATCGGTTCCAGCAATTCCAAGTGATATTGTTGATAGAACTGATCAA
ATGCGTATCGATGCAACTACTCATATTGGTGGAGTTCAGATTAAGCAGGAGTCGAAGCCG
ATTAAGACGGAACCAATTGCTCCACCACCATCATACCACGAGTTGAACAGTGTCCGTGGA

TCGTGTGCTCAGAATCCACTTCTTCGAAATCCAATTGTGCCAAGCACTAACTTCAAGCCA
ATGCCACTACCGGGTGCCTATGGAAACTATCAAAATGGTGGAATAACTCCAATCAATTGG
CTATCAACATCCAACTCATCTCCACTGCCTGGAATTCAATCGTGTGGAATTGTAGCTGCA
CAGCATACTGTCGCTTCTTCATCGGCTCTTCCAATTGATTTGGAAAATCTGACACTTCCC
GATCAGCCACTGATGGATACTATGGATGTTGATGCATTGATCAGACATGAGCTGAGTCAA
GCTGGAGGGCAGCATATTCATTTTGATTTG
>Zebra_finch_FOXO3a
ATGCCGCGGGCGGGCTGGCGGCTCGCCCCGACAGCAAGCCTCCTTCCCAGTCACGACGAG
TCGCTGCGTGCCTACTCAAGGACAGCGCAACGCGTGGGCAACTTCGAGCTCGGTACCCGG
GGATCCCACCGCCGACGCCGCCTCCTGATCCCCGAGGAGGAGGACGACGAGGAGGAGGGG
GGCAGCTCGGCCATGACCGTCGGCAGCGCGGCCCCCGCGGGCGGAGAAGCAGCGGCGGCG
GCGGCGACCACCGCCGTGCCGGAGGAGGCGGCGCGGCTGCTGGCCCCGCTGCCCGGCGGC
GGCCCGGAGGGGCCGAGCCTCTCGCCGGGGGGAGCGGCGGCGGCGGCAGGCGGCGGGGGG
CTGAGCGGGGGCCCGGCGGCGGCGCCGAGGAAGTGCTCGTCGCGGCGCAACGCGTGGGGC
AACCTCTCCTACGCCGACCTCATCACCCGCGCCATCGAGAGCTCCCCGGAGAAGCGCCTC
ACTCTCTCCCAGATCTACGACTGGATGGTCCGCTGCGTGCCCTACTTCAAGGACAAGGGC
GACAGCAACAGCTCGGCCGGGTGGAAGAATTCGATCCGGCACAACTTGTCACTCCATAGC
CGATTTGTCAGGGTGCAGAATGAAGGCACCGGGAAAAGCTCTTGGTGGATGATCAATCCA
GATGGTGGAAAAGGCGGCAAGGCGCCCCGGCGACGCGCTGTGTCAATGGACAACAGCAAC
AAGTACACCAAGAGCAGAGGGAGGGCAGCAAAGAAAAAGGCAGCCCTGCAGACAGCCCAG
GAGACGAGTGAGGACAGCCCTACCCAGCTCTCCAAGTGGCCAGGGAGTCCCACCTCCCGC
AGCAGCGATGAGCTGGATGCATGGACAGATTTTCGCTCCCGTACAAATTCAAATGCCAGT
ACGATCAGTGGCCGCTTGTCACCAATTTTGGCAAGCACCGAGCTCGATGATGTTCAAGAT
GATGATGCTCCACTTTCTCCCATGCTGTACAGTAGTCCATCGAGCTTGTCCCCATCGGTA
AACAAACCATGTACTGTGGAGTTGCCTAGGTTGACTGATATGGCTGGGACAATGAACTTG
AATGATGGACTGACTGATAACCTCATGGATGATCTCTTGGACAATATAACACTCCCTCCC
TCCCAGCAGTCACCCACAGGAGGGATAATGCAGAGAAGCTCCAGTTTTCCTTATGGTTCC
AAAGGTTCAGGGCTGGGCTCCCCATCAAGTAGTTTCAACAATGCTGTGTTTGGGCCGTCG
TCCCTGAATTCCCTCCGCCAGTCACCCATGCAGACAATTCAGGAGAACAAGCAGGCCACC
TTCTCTTCCATTTCTCATTACAACAACCAGACGCTGCAGGATCTCCTCACCTCTGATGCG
CTTAGTCACAGCGATGTCATGATGACACAGTCTGACCCACTCATGTCACAAGCCAGCACG
GCTGTGTCCGCCCAGAATTCCCGCAGGAATATAATGCTCCGCAACGACCCCATGATGTCG
TTTGCCGCACAGTCCAGCCAGGGCGGTCTGGTCAATCAGAACCTGTCCCATCACCAGCAC
CAGTCCCACAACTCCTCTCTTAGTGGCAGCCGTGCCTTGTCCAATTCCATCAGTAACATA
GGCTTGAGTGACTCCAGCAGCTTGGGATCCAGCAAACATCAGCAGTCATCTGTCAATCAG
TCTATGCAAACACTTTCTGACCCGCTGTCAGGCTCCTCTTTGTACTCCTCTAGCGTGAAC
CTCCCGGTCATGGGACATGAGAAATTCCCGAGTGACTTGGACCTGGATATTTTCAATGGC
AGCCTGGAGTGTGACATGGAGTCCATTATCCGCAGTGAACTCATGGATGCCGATGGGCTG
GATTTTAACTTTGATTCCCTCATCTCAGCTCAGAACGTTGTCAGTCTGAATGTGGGGAAC
TTCACTGGTGCTAAACAGGCTTCATCACAGAGTTGGGTACCAGGC
>Zebrafish_FOXO3a
ATGGCAGAGGAGCTAGATAAGCCTTTGGCTGTGGACGTCGACATAGACCCTGATTTTGAG
CCCCAAAAAAGGCCCAGGTCTTGCACATGGCCTCTGCCCAGACCCGAGTCCAATTCGGGC
AAAGCAGAACCTTCAGATGTGGGGATCATTCCTGAAGAAGAGGTAGATGAAAATGGCACT

GATGATGCTTGTGCATCTGGTGACATTACAGGCGCATCAAAGCCTGCCAGTGTCACAGAA
GGAGACCCGAGTTCTGCTGCTGCTCTTCCTGCCATAGAAACGAACGCTTCTGCCAATGAT
AAAGACATCTATGGCTCTCCTGGATCTTCCCAACACGCTCTAGCAGCATGCAGCGACTCC
AGCATCAATGGTCTGATTCCTCAGCAGCCCAGAAAATCCTCTGCCCGCAGGAACGCCTGG
GGAAACTATTCCTACGCCGACCTCATCACCCAAGCCATCGAGAGCTCGCCAGAGAAGAGG
CTGACACTGGCCCAGATTTATGATTGGATGGTCCGAAATGTGCCATACTTCAAGGACAAA
GGTGACAGCAACAGCTCTGCAGGATGGAAGAACTCAATACGACATAACCTGTCGCTCCAT
AGTCGCTTTGTCAGAGTCCAAAATGAAGGAACAGGAAAGAGTTCATGGTGGATGGTCAAC
CCTGATGGTGGAAAAGGGGGCAAAGCTCCACGCAGGCGTGCAGTATCAATGGACAACAGT
AATAAGCTCATCAAGAGCGCCCGTGGCCGTGCCGCAAAGAAGAAAGCCGCTCTGCAGGCA
TCCCAGGACGGAAGCTCTGAGAGTTCCTCCAGTCTGTCCAAATGGACCGGCAGCCCCACG
TCCCGTAGTAGTGACGAGCTAGATGCTTGGACAGATTTTCGTTCTCGCACTAATTCTAAT
GCCAGCACCCTCAGTGGACGTCTTTCCCCAATTCTAGCCAACCTGGAGGTGGATGAAGTT
CCCGATGATGACTCTCCCCTGTCACCCATGCTGTACTCCAGCCCCAGCAGTATGTCTCCG
TCCACTGGGCTCACCGAACTGCCACGTCTAGCTGACCTTGCAGGAACTATGAACCTCAAC
GATGGCCTCTCTGACAACCTAATGGATGACCTTCTGGACAACATCAGCTTGACGGCTTCA
CAGTCTCCGGGCCATGATGAGAGTGGAGCCAACCTACAGGGAAGCCCTGTGTTTACCTTC
AGCTGCTCTGGGAGCAGTCTGGCAATTCCCTCCGGCAGCTATGGCACCAACTCCCTGTTT
AGCCCTCCATCAGTCACTGGGCTGAGACAGTCTCCAATGCAAACCATCCAGGAAAACAAG
CAGGCAACATTCTCCTGCGGTTCCCTCTTTAGTGAGCAGAGTCTGCAGGATTTGCTCAGC
TCTGAGTCCAGTAGTCGCAGTGATGTCCTTCTTACCCAATCTGACCCGCTTATGTCTCAA
GCCAGTGCCTCCGTTTCCTCTCAGAATGCTCGTCGCATTTTGTTGCGTAATGACCCCATG
ATGTCCAACCAGGCTGGGCCTGCAGGACAAGCAGGGCTCAAAAAGGCATCGCCATCTGGA
TGGCGGATGAACTGCGTCCCGAGCAGCGAGTCAGACTACCAAAGCTTGATGAAGCAACTC
CAACAGTCTCCCTTCAGAAGCACATCTATGCAGCTCAACTCCTCCGATTCATTGTTGGCA
GGTCTCAACGGCAGCGTGACCTCTGCTCAGTCGGTGTCTCAGGACCGTTTCTCATCTGAC
TTGGATCTTGAGGCATTCAGTGGCAGTTTCGATTGCGATATGGACATCATAACCCGCAAC
GATCTGATGGATGCCGAAGGCCTGGAGCTCAGCTTTGATTCCCATCTCATCTCCTCTCAG
AATGCTAACCTGACTTCAGGGAGCTTCTCCAGAACCAAACGAACCTCCTCCCAAAGCTGG
GTGCCAGGC
>Adelie_penguin_MRPS5
ATGTACGCCAAGGTTTCTGGATCCAAAAACTTGATTAACATTACCAGAGCTCTCTTTAAA
GGCTTGACACAACAGGAGACCCACCAACAGTTAGCGAACCAAAAGAGTCTCTATGTAGTG
GAGTTCCGGGAGGAGCAGGGCCCTCTGCCCATCGTGGTGGCACTGCCTGAGGGGACTGTC
CGCGAGGATCCCGAGCTTGAAGATGAGGTTCCAGACACAAAGCTGGAGTGGAGTGAGGTG
AAAGAAGCTCAGGGAATGAAGAAATCTCCCTGGGCAAATGTCAGACGGACAGTATGG
>common_starling_MRPS5
ATGGCGGCGGCGGTGATGGCGGCCGGGTGGGCATGTGCCCGCGGCGCGCTCCGAGCGGCC
TGGCGAGGGTTTGCTTCTGTGCCAGTGAAAGGGGGTTGCAGTTCGTACTTCAGCCTCGCA
TGGACTTTCCAGACACGGTACTCCCTCTCTGTTCCCTGGACTGGGACAGTCAAGCAGTGC
AGGAAAAGCAGTTTCTTCAATACCTTGACAGCTGATCAGCTATGGAAAGGAGCTTTGGCA
GAGACTGGCGTGGGAGTAAAGAAAGGAAGAGGAAAGAAAAGGAAGAAAAAGCTAAAGAAG
AATCTTAATAGAGGCCAAGAGATTGGTGAAGGACGTTCTGGTTTCCTCTGGCCTGGTCTT
AACGCTCCTGTGTTGCAAACTGGGAAAGTTCAGGAACTTGCCCAACGAAAAAAAGAGGAA

CGAGAGAGAATTGAGACTGAAATTATTCAGCAGAGAGATACATGGGAGAAGAAAAAGAAA
TTAAAAATTAAGAGAGAGGGAGGATGGAGTGGAAAGTGCTGGGGAGGTATCATTCTGGAT
CCTCCTGACCCTGGCCCTAATGGAGAAACTTATGAAGATTTTGAAACAAGAGTCATCGAG
GTGAGAAATGTGTTTTGTATGAAGGCAAAGGAGGGCAGAAAAAAATCAGTGCGTGCCTTA
GTGGCTGTTGGGAATGGTAAAGGGGCTGCAGGTTTTGCAATAGGGAAGGCAGGTGACAGG
ACAAATGCTTTAAGGAAAGCAAAGAATAAAGCAATAAGCTCCTTACACTTCATAGAGCTA
TATCAGAACCACACAATTTACCACGACATTTCTGCGAAATTTAAAAGGACAAAAATCCGC
ATGAAGAAACAAAACAAAGGGTATGGTCTGCATTGCCACAGAGCTATTATCACCATTTGC
GGGCTAATTGGCATTAAGGACATGTATGCCAAGGTCACTGGATCCAAAAACTTGATTAAC
ATCACCAGAGCTCTCTTTAGAGGCTTGACCCTACAGGAGACTCACCAGCAGCTTGCAAAC
CAGAAGAACCTCTACGTGGTGGAGTTCCGGGAGGAGCAGGGCCCTCTGCCCATCGTGGTG
GCCCTGCCCGAGGGGACTGTCCGTGAGGAGCCTGAGCCAGAGGATGAAGTTCTAAACACA
AAGCTGGAGTGGAGGGAGGTGAAAGAAGCCCAGGGAATGCTGAAATCTCCCTGGGCAAGT
GCCAGACGGGCGGCGTGC
>central_bearded_dragon_MRPS5
ATGGCCGTATGTCCTGGGCGTTGCAAAACGGAGCGGGTGGCCTTAGTGACCTGGAAATCG
AGGGAAGGGAAGGGAAAGGGAGGGGGAAAGAAAGCGCTCACAGTACGGTGGCCACGAGGG
AACAAGAAGAAAATGGCGGCCGCCTTAGCATCGGCGGCCCAAAGAGTCTGCAGCCCCTGG
ATCTGGAGAACCGCGTGGAGAGGCTGTGCCTTCATTCCATCTCATGGAGCCCATTGCCAG
TACACGACATTGGCAAATGCTTTACAGAAACATTGCTATGTTTCTGCACCTCCCAGCGTC
ACAGTGCAACAGTACAGACAAAGCAGCTTTTTCGGCAAATTGACAGCTGATGAAATCTGG
AAAGCAGTTGTGGCACAGTCTAGTTCAAGTAAAAACAGAGCAAGAGGCAAAAGGACCAAG
AAGAAGATAAAACTGAATCTCAATAGAGGCCAGATGATCGGTGAAGGAAGATCTGGGATC
CTTTGGCCCGGCCTCAGCGCCCCCATCTTAAACCAGAGAAGCACGCTGGGGATTCAACGA
CGAGAAAAAGAAGAGCAAGGGAAACTAATGTCGGAAAGGCTGATAAAGCGGGAGGAATGG
GAGAAGAAAATGAGACGGAAGGTGAAGAAAGAGAGAGGCTTTACTGGAGCCTCCTGGAAC
GGTATCAGTCTGGGACACCCTGATCCAAGCCCTGATGGAGAAACATATGAAGATTTTGAC
TCCACGCTGATTGAGCTGAAAACGGTATCTACCATGACAGCCAAGGAAGGAAGAAGAAGG
TCCTTTAGTGCCCTGGTGGTGGTTGGGAATAAAAAAGGGGCTGCTGGTTTTGCACTGGGG
AAGGCCAATGTAAAGCAGAATGCATTGAGAAAGGCAAAGAACAAAGCCATTCATAATTTA
CACTATATTGAAAGATACAATAATCACACAATTTATCATGATATTTCAAGCACTTTTCAC
CAAACAACCATCCGGATGAAGAAGCAAAGCAAGGGATATGGCCTTCACTGTCACCGAGCT
ATCATTGCCATGTGCAAACTGATTGGTATTGAAGATATGTATGCCAAGCTTTATGGATCC
AACAATCCACTGAATCTTACTAAGGCTTTCTTCAAAGGCTTAGCAAATCAGAAAACTCAC
CAGAAGCTGGCAGATGAGAAGAGTCTCTACGTAGTAGAATTTCGGGACGAATGTGGCCCA
CTGCCCATCGTTGTTGCAAAGCCCCAAGGGCCGGTGAGGGAAGATCCTGAGCCTGAGGAT
GAAGTTTCTGATATCATACCAGAGTGGAGAGAAGTGAAGGCTGCCCAGGGAGCAAAGAGT
CGCTGGGCAAATCTCAAAAGAACAATTTGG
>Gecko_MRPS5
ATGTTGGCATGTGCTTTGCAGACACATTGCTGTATTTCTGCACCTCGTGATGTGACACTG
GAACAGCGCAGGCAAAGCAGCTTTTTAAACAAATTGACAGCTGACGAACTCTGGAAAGGA
GTTTTGGCAGAGACTACGAGTGGAAGGAAAGGAAGAGGGAAGAGGACCAAGAAAAAGCTG
AAAAGGGATCTTAACAGAGGCCAGACAATTGGCGAAGGACGTTCAGGTTTCCTTTGGCCT
GGCCTCAATGCTCCTGTGCTAAAAGGTGGAACAATCCAGACAATTGGACGGCGAGGTGAA

GAAGAGCGGGAGAAGATGCAGTTGGAAATCATCCGTCAGCGGGATGAGTGGGCGAAGAGG
AAAAAAACGAAGGTGAAGAAGGAGAGGGGATGGACTGGACGTTCCTGGGGAGGTGTCAGC
CTAGGGCCTCCTGACCCGGGTCCTAATGGAGAAACATATGAAGACTTTGACTCCAGAGTG
ATTGAGATGAAGAATGTATTTAATATGACAGCAAAGGAAGGAAGAAAAAAATCTGTTAGT
GCCCTGGTGGCTGTGGGAAATGGAAAAGGAGCTGCTGGTTTCGCAGTGGGAAAAGCAACT
GACAGGATGGCTGCATTACGAAAAGCAAAGAACAAAGCAATTCACTATTTACACTATATT
GAAAGATATGAAGACCACACAATCTATCACGATATTGCAGTTTCGTTTAAAAAAACAACC
ATCCGGATGAAAAAGAAAAACAAGGGACATGGACTTCACTGTCACCGAGCTATCATTACT
ATGTGCAAACTGATTGGGATTAAAGACATGTATGCCAAACTTTATGGATCTAATAATCTA
TTGAATCTCACCAAAGCGCTCTTCAAGGGATTATCAAGACAGGAAACTCACCAAGCCCTA
GCCAATAAGAAAAGCCTCTATGTGGTAGAATTTCGTGAGGAATGTGGCCCCCTGCCCATC
GTGATCGCTGCACCCCAAGGGGTCCCCAGGGAAGACCCTGAGCCTGAGGATGAAGTTCCT
GATGTGAAGCTGGAGTGGAACGAAGTGAAGGCAGCCCAAGGAATGAAGAAGTCTCCCTGG
GCAAACGCCAGAAGACCAGTGTTG
>Taiwan_habu_MRPS5
ATGGCGGCCGCCGTAGTTGTCGCGGCCCAGAGAGTCTGTAATGTCGGCATCCTCAGGACC
GCGTGCAGAGGTTATGCTTTGATTTCAAACCATGGAATTAGTTGCCAGCATGCTGCCTTG
GCATGGACTTTTCAGACACATTGCAACATTTCTGCATCATATAATGTGACAGTGCAACAG
AGAAGGCAGAGAAGCTTTTTCAATAAATTGACAGCCAGTGAACTCTGGAAAGGAGTTGCA
GCAGAGAATACTGCAGGTGGAAGGAAGGGGCGAGGCAAGCGAGGCAAGAAGAAAAAAACA
AGGGATCTCAATCGAGGCCAGTTCCTTGGCGAAGGAAAAATCGGTTTTCTTTGGCCTGGC
CTCAATGCTCCATTGATGGTATCTGGAAAAGTTCAAAGCATTGCTCAGAGAGATGAAGAA
GAGCAGAAGGCATTGCAGTTAGCAAAAGCTGAGGAAATAAAACAACTAGACAAAAGAAGG
AAAATACGGATTAAGAAGGAGAGAGGTTGGACTGGACGATCCTGGGGAGGTGTCAGTTTA
GGACCTCCTGATCCTGGTCCCAATGGAGAAACATTTGAAGATTTTGATAGCAGAATACTT
GAGCTAAGATCCGTATTTAACATGACTGCAAATGAAGGCCGAAAAAGATCTATGAGGGCC
CTCGTGGCTGTTGGCAATGGAAAAGGATCTGCTGGTTTTGCTCTAGGAAAAGCATCTCAC
ATGAAGAATGCACTAAGAAAAGCAAAAAACAAAGCTATTCACCATTTACAGTATGTTGAA
AGATATGACGATCATACAATTTACCATGATATTGCAGCTACTTTTAAGAAAACAACAATT
CGGATGAAGAAGCAAAACAAAGGGTATGGTCTTCGTTGTCACCGTGCTATCATCACACTC
TGCAAACTGATTGGGATTAAAGACATGTATGCCAGGGTTTATGGATCCAATAATATATTG
AATCTCACTACGTGTCTCTTTAAGGGATTAGCTAATCAGGAAACTCATCAAAATCTAGCA
AACAAGAAGAAGCTCTACGTGGTAGAATTCCGTGAAGAATGTGGCCCACTGCCCATTGTT
GTAGCAAAGCCTAATGGACCAGTCAGAGAAGATCCTGAATCAGAAGAAGCAGCTCTTGAT
GTCAAATTGGAATGGAAAGAAGTGAAGGCAGCACAAGGAATAAGGAGTAGATGGGCTGAT
GTCAAAAGAGCTGTTTGG
>green_sea_turtle_MRPS5
ATGGATTATGTGCTTACAGAAAAAGGGCCCATCATTGATCACCTCTTAGCAGAAGCTGGA
AATGTGTGCAATTTCATTACAGTGACAGCTAATGAGCTGTGGAAAGGGGTTTTGGCAGAG
ACTGGTGCTGGAGCAAGGAAGGGAAGAGGAAAGCGAACCAAGAAAAAGTTCAGGAGGGAT
CTCAATAGAGGCCAGATCATTGGTGAAGGACGTTCTGGTTTCCTTTGGCCTGGTCTCAAT
ACTCCTGTGCTGAAAAGTGGGGCACTCCAGACAATTGGTCAGCGAGACAAAGAGGAGCAG
GAGAAGGTGCAGTCTGAAATTATCCGGCAGAGGGATGAGTGGGAGAAGAGAAGAAAAACA
AAGGTGAAGCGAGAGAGAGGCTGGACTGGAAGTTCCTGGGGAGGCATCAGTCTGGGACCC

CCTGAGCCAGGTCCTAATGGAGAAACTTACGAAGACTTTGACTCCAGGGTAATTGAGGTG
AAAAATGTATTTAATATGACAGCAAAGGAAGGAAGAAGAAGATCAGTTAGTGCCCTAGTG
GTTGCTGGAAATGGAAATGGGGCTGCAGGTTTTGCCGTGGGAAAAGCAAGTGACAGGATG
ACAGCTTTAAGAAAAGCAAAGAACAGAGCAATTCACTATTTACACTATGTAGAGCGATAT
CAAAACCACACAATTTACCATGATATTACAACAACTTTTAAAAAAACAACCATCCGGATG
AAGAAGCAAAACAAAGGGCATGGTCTTCATTGCCATCGAGCCATCATTACCATCTGCAAG
CTAATTGGGATTACAGACATGTATGCCAAGCTTTCTGGATCCAACAATCTGCTTAACCTC
ACCAGAGCTCTCTTTAAGGGTTTAGCAAAACAGGAAACCCATCAAGAACTAGCAGATAAG
AAAAGCCTCTACGTGGTAGAATTCCGTGAGGAGTGTGGCCCCCTGCCGATCGTAGTTGCT
TCACCTAATGGAGATGTCAGGAAGGAACCAGAACCTATTGATGAGGTTCCCAATACCAAG
CTGGAATGGAGTGAAGTGAGAGTAGCTCAAGGAATGAAACGTTCTGCCTGGGCAAATGTC
AAAAGGACAGTGTGG
>Chinese_alligator_MRPS5
ATGGCGGTGGTGGGGCGGCTGTGCGCCTCGGTGGCCTGGAGGGCAGCGTGGAGAGGCTAC
ATCTCGCTACCAGTCAACGGGAGTGGCTGTCGATATACGAACTTAAGGTGGGCACTGCAG
ACACATTGTTACATTTCTGCTCCATGCGGTGTGACTATACAGCAAAGCAGGCAAAGCAGT
TTCTTCAACAAATTGACGGCTGATGAACTATGGAAAGGAGTTCTGGCAAACACTTCTTCG
AGAGCAAAGAGAGGAAGAGGAAAGCGGACAAAGAAAAAATTAAGGAAGGATCTCAATAAA
GGGCAGATGATTGGTGAAGGGCGTTCCGGAATTCTTTGGCCTGGTCTCAGTATTCCTGTG
ATGGTAGATGGAAAAGCCCAGGTAATTTCCCAACACAAAAAAGAGCAAAGGGAGATTCAA
GATGAAATTTTACGTCGGAGGGATGAATGGGAAAAAAAAAGAAAAGTAAAGGTGAAGAAA
GAGAGAGGATGGACTGGACGATCCTGGGGGGGCATCAGTCTAGGACCTCCTGACCCTGGT
CCTAATGGAGAAACATATGAAGGCTTTGACTGCCAAGTACTTGAGCTGAAAAGCGTAGCT
TGTATGACAGGAAGAGAAGGGCGGAAAAGATCTGTGAGTGCCCTTGTGGCTGTTGGAAAT
GGAAATGGGGCTGCAGGTTTTGCATTGGGGAAAGCAAAAGACAGAATGGTAGCTTTAAGA
AAGGCAAAGAACCAGGCAATTCACTATTTACACTATATAGAACGATACCAGGACCATACA
ATTTATCATGACATTACGACAACTTTTAAAAAAACAACCATCCAGATGAAAAAAAAACAC
AAAGGGTATGGTCTACGTTGTCACCGAGCTATTATCACTATCTGCAAACTCATCGGGATT
ACAGACATGTATGCCAAAGTGTGTGGATCCCCTCATATCCTTAACCTCACCAAAGCACTC
TTTAAGGGATTGGCAAACCAGAAAACCCACCAGGACTTGGCAAATGAGAAGAGCCTCCAT
GTGGTGGAATTTCGTGAGGAGCAAGGCCCATTGCCTATCCTTGTGGCTTCCCCTCAGGGG
ACAGTCAGAAAGGATCCCGAGACAATAGAGGAGGTTCCAGATGTCAAGCTGGAGTGGAAT
GAAGTAAGGGTGGCACACAGAAGGGGAAGGTCTGCCTGGTCCAGTGTCAAGAGAATGGCA
TGT
>Gharial_MRPS5
ATGGCGGCTGTGGGGCGGCTGTGCGCCTCGGTGGCCTGGAGGGCGGCGTGGAGAGGCTAC
ATCTCACTACCAATCAACAGGAGTGGATATACAAACTTAGCGTGGGCACTGCAGACACAT
TGTTACGTTTCTGCTCCATGCAGTGTGACTGTACAGCAAAGCAGGCAAAACAGTTTCTTC
AACAAATTGACGGCTGATGAACTGTGGAAAGGAGTTCTGGCAAACACTGCTTTGAGACCA
AAGAAAGGAAGAGGAAAGCGGACAAAGAAAAGAATAAGGAAGGATCTCAATAAAGGGCAG
GTTCTTGGTGAAGGGCTTTCCGGAATTCTTTGGCCTGGTCTCAATTCTCCTGTGATGGTA
GAAGGACGAATGCAGGCAATTTCCCAAAGCAACAGAGAGCAAAAGGAGATTCAAACTGAA
ATTTTTCGTCGGAGGGTTGAATGGGAAAAAAGAAGAAAAGTAAAGGTGAAGAAAGGGAGA
GGATGGACTGGACGATCCTGGGGGGGCATAAGTCTAGGACCTCCTGACCCTGGTCCTAAT

GGAGAAAAATATGAAGACTTTGACTGTCAAGTACTTGAGCTGAAACATGTAGCTAGTATG
ACAGGAAGAGATGGGCGGAAAAGAACTGTGAGTGCCCTTGTGGTTGCTGGAAATGGAAAT
GGGGCTGCAGGTTTTGCAGTGGGGAAAGCAAAAGACAGGATGACTGCTTTAAGAAAGGCA
AAGAACCAGGCAGTTAACTATTTACACTATATAGAACGATACCAGAACCATACAATTTAT
CATGACATTACGACAACTTTTAAAAAAACAACCATCCAGCTGAGAAGACGACGCAAAGGG
CATGGTCTACGTTGTCACCGGGCTATTATCAGTATCTGCAAACTCATCGGGATTACAGAC
TTGTATGCCAAACTATGTGGATCCCATAATATACTTAACCTCACCAAAGCACTCTTTAAG
GGATTGGCAAACCAGAAAACCCACCAGGACTTGGCAAATGAGAAGAGCCTCCACGTGGTG
GAATTCCGTGAGGAGCAAGGCCCGTTGCCTCTCCTTGTGGCTTCCCCTCAGGGGACAGTC
AGAGAGGATCCCGAGATAACAGAGAATGTTCCAGATGTCAAGCTGGAGTGGAATGAAGTA
AGGGTGGCACACGGATTGAGACGTTCTTCCTGGTCCAATGTCAAGAGAATGGGGTGG
>Tibetan_frog_MRPS5
ATGGCGGCTGTGAGATGGTGTAGCGGTGTCCTCCGCTCTGTTTATAGAGGTGGGATCTCT
GCACCAGTGATAAGGACCAGCTGCCAATTCCACAGCCTCAGACTTCCACAGCTTCCACAA
GAACCATGTTACTTATCGACTTATCACAATGTCTCTATTCAGCAAGCAAGGCTGGCAAGC
AGTTTCTTTGGCAAATTGACAGCAAATGAGCTGTGGAAAGGAGTGTTGGCAGATACGTCA
GGAGGATCTCGTAAGGGAAGAGGTAAAAGAACCAAGAAGAGGATTAAAAAGAATCTGAAC
GTTGGACAGCGTATTGGAGACGGAAAATCTGGATTTCTGTGGCCAGGCTTGAATTCACCT
ATAGTATTGAACAAAAAGGAGCAAAACATTGAACAGCGGAATAAAGAGCAGCAGGAAGAA
AGGCAAGCTCAGATATTTAAGGAGAGAGATCAGTGGGAGAAAAAGAAGAAGACCAGAGTT
AGAAGAGAAGAGAGAGGATGGACTGGAAGAAGCTGGGGTGGACTCAGCGTTGGAAAACCT
GATCCAGGTCCTAATGGAGAAAAATATGAAGATTTTGAATGCAGAGTGATTGAGCTGAAA
AGTGTATTCACCATGACAGCCAAAGAAGGAAGGAAGAGAAGTATGAGTGCCTTAGTGGTG
GTTGGCAATGGCAATGGAGCAGCTGGGTTTGCAGTTGGAAAATCCAACGACAGAGCGATA
GCACTAAGAAAAGCAAAGAACAGAGCTGTCCATTATCTGCATTACGTAGAACGATACAAC
AATCATACAATATATCATGATATCACAACAAAATTTAAGAGAACAACAATTAAAATGAAG
AAACAAAACCCAGGTTATGGTCTTCATTGCCATAGAGCAATCATCACTATCTGCAAACTG
ATTGGCATCAAGGACATGTATGCCAGGTTGTCAGGTTCTCACAATCTGCTTAACATTACA
AATGCACTCTTCCAGGGTCTGACCAAGCAGGAAACGCATCAAGAACTAGCCAGCAGAAAA
GGTCTACATGTTGTGGAGTTTAGAGACGAATGTGGTCCCCTTCCTAGAATTGTGGCTACA
CCTCATGGACCTCTTTCAACAGTTCCCGAACCTGAAGGGGAAATAGTTGACACACCACTC
GACTGGGATGATGTTAAGGTGACCCAGGGAATAAAGCGCTCTGTCTGGGCCAATGTGAAA
AGGACAATTTGG
>southern_platyfish_MRPS5
ATGGCGGCGTCCGTATGGGTGTGCAGTGCCCTCCGCATCACGCTCGGAGGCGCAGCATCA
CTCCGCACCACGGTGGGAGCTGTTCAGATGTCTCACTTTGTCTGTCGAACCTCAGCAGGC
TCTCTGCAGAGACGCCCAGAGTTTCTGTTGAACCCGTCAGCAACCTGGCAGCAGAGCAGA
CATGGCAGTTTCTTCAACAAGTTGACGGCTGAGGAACTGTGGAGGGGTGTGTTGGCTGAA
TCCGGAGCCGGAGCCAGAAAGGGCAGAGGGAAGCGAACCAAACGCAAACTGAGGAGAGAC
CTCAATCGAGGACAGCTGATCGGAGAAGGTCGAGGTGGTTTTTTGTGGCCCGGTCTGAAT
TCTCCTGTATTAAAAGACGGCGCGGTACAGAGCTTCAGCCGAAGAAGCGACGCCGAGCAA
CAGGAAGTTCACGCTGAACTAATGCGGCAGAGGGACGAGTGGGAGAAGAGGAGGAGGATG
AAGGTGAAGAGAGAGAGAGGCTGGACCGGAAGCTCCTGGGGGGGCATCAGCCTGGGGCAG
CCTGACCCCGGACCCAATGGAGAAACCTATGAAGACTTCGATTCTCGAGTCATTGAGGTG

AAGAGCGTGTTCAACATGACGGCCAGGGAGGGCAGGAAAAGGTCCATCAGCTGTTTGGTT
GCTGTAGGAAATGGCAACGGAGTGGCAGGGTTCGCTTTGGGTAAAGCAACAGACAGAAAT
GCAGCTCTGAGGAAGGCCAAGAACCGAGCCAAACGCCATTTGTACCACATTGAGCGATAC
AACAACCACACCATTTATCACGACATCAACTCCAGGTTCAAGAGGACGACTCTGCGCATG
AAGAAGCAAAACGAGGGTTACGGTCTGCGCTGCCACAGGGCCGTCATCACGCTGTGCAAG
CTGATCGGCATCAAGGACATGTACTGCAAAGTGGACGGCTCGGTCAATCTCCTCAACATC
ACGCGGGCGCTCTTCACTGGGTTAGCCAATCAGGAAACTCATCAGAACCTGGCCAACAAG
AAGCAGCTCCACGTGGTGGAGTTCCAGGCCCACCGGGGCCCGCTGCCCGTGGTGGTGGCC
AGCCCGAAAGACGGAGCGCGTCTCCAGCCGGAGACCGTGGACGAGGTCCCCAACACCAAG
CTGCACTGGGACGACGTCCGAGACGCGCAGGGAACCAAGCGCTCCATCTGGGCGGGGGTG
AAGCGCACCGTGTGG
>spotted_gar_MRPS5
ATGGCGGCGGCCAGATGGGTATGGTCTGCCCTCCGGATACCTGTGAGAGGGGGTATCCTG
GTCCCAACACAGGCTTGTACTAGTGCTGTACAAACCACCTGGGCCGTCTCTTCAACCACT
GCCAGCGTCTCCGTGCAGCAGTACAGATTCAGCAGTTTCTTCAACAAATTGACGGCTACT
GAGTTATGGAGGGGGGTGCTGGCAGAGAGCGGGCCAGGGGCACGGAAGGGAAGGGGGAAG
AGAACTAAGCGAAAGCTGAAGAAAGACCTCAACAGAGGGCAGAGTGTCGGAGAAGGACGA
GGAGGCTACCTCTGGCCCGGCCTGAACTCGCCTGTGCTGAGAAGTGGTACCATCCAGTCC
ATCTCCCAGAGGGACCAGGCCCAGCAGGAGGAGCTGCAGGCGGACATTGTCCGCCAGAGA
GACGAGTGGGACAGGAAGAGGAGGACGAAGGTGAAGAGGGATCGGGGATGGACCGGGAAT
TCCTGGGGGGGGATCAGCCTCGGATCGCCAGATCCTGGGCCGTACGGAGAAACATATGAA
GATTTCGATTCTCGGGTGATTGAGGTGAAAAGCGTTTTCAACATGACAGCGAACGAGGGA
AGGAAGAGATCGATCAGTGCTCTGGTCGCTGTCGGAAATGGGAATGGGGCAGCAGGTTTT
GCTTTGGGGAAGGCAGCCGACCGAGCAACTGCACTTCGAAAAGCGAAGAACAAAGCTGTC
CATTATTTGTACTACATAGAGCGATACAACAACCACACAATCTATCATGACATGACTTCC
ACCTTCAAGAGAACCACCCTCCGGATGAAGAAACAAAGCAGAGGTAAGTCTGGCACTGCC
AGGGCAGTACATGCCTCTCTCCTGTTTGTTTTGCCA
>guppy_MRPS5
ATGGCGGCGTCCATATGGGTGTGCAGTGCCCTCCGCATCACACTCGGAGGAGCAGCATCA
CTCCGTACCGCGGTGGGAGCTGTTCAGATCTCTCACTTTGCCTGTCGAACCTCAGCAGGC
TCACTGCAGAGACGCCCAGAGTTTCTGTTGAACCCATCAGCAGCCTGCCAGCAGAGCAGA
CATGGCAGTTTCTTCAACAAGTTGACGGCTGAGGAACTGTGGAAGGGTGTGTTGGCCGAA
ACCGGAGCCGGAGGCAGAAAGGGCCGAGGAAAGCGAACCAAACGCAAAATGAAGAAAGAT
CTCAATCGAGGACAGGTGATCGGAGAAGGTCGAGGTGGTTTTTTGTGGCCCGGTCTGAAT
TCTCCCGTATTTAAAGACGGCGCCGTGCAGAGCTTCAGCCGAAGAAGCGACGCCGAGCAA
CAGGAAGTTCAAGCTGAACTAATTCGGCAGAGGGACGAGTGGGAGAAGAGGAGGAGGATG
AAGGTGAAGAGAGAGAGAGGCTGGACCGGAAGCTCCTTGGGAGGCATCAGCCTGGGGCAG
CCTGACCCCGGGCCCTATGGAGAAACCTATGAAGGCTTTGATTCTCGAGTCATTGAGGTG
AAGAGCGTGTTCAACATGACAGCCAAGGAGGGCAGGAAGAGATCCATCAGCTGTTTGGTT
GCTGTAGGAAATGGCAACGGAGTGGCAGGCTTCGCTCTGGGTAAAGCAGCAGACAGAATT
GCAGCTCTGAGGAAGGCCAAGAACCGAGCCAAACGCCATTTGTACCACATTGAGCGATAC
AACAACCACACCATTTATCACGACATGGACTCCAGGTTCAAGAGGACGACTCTACGCATG
AAGAAGCAAAGCGAGGGTTACGGTCTGCGCTGCCACAGGGCCGTCATCACGCTGTGCAAG
CTGATCGGCATCAAGGACATGTACTGTAAAGTTGAAGGCTCGGTCAATCTCCTCAGCATC

ACTCGGGCGCTCTTCACTGGGTTGGTCAAGCAGGAAACTCATCAGAAGCTGGCCAACAAG
AAGCAGCTCCACGTCGTGGAGTTCCAGGCCCAGCGGGGCCCGCTGCCAATGGTGGTGGCC
AGCCCGAAAGACGGAGCGCGTCTCCAGCCGGAGTCCGTGGACGAGGTCCCCGACACCAAA
CTGCACTGGGACGACGTCCGAGACGCGCAGGGAACCAAACGCTCCATGTGGGTGGGGCTG
AAGCGCACCGTGTGG
>American_alligator_MRPS5
ATGGCGGTGGTGGGGCGGCTGTGCGTCTCGGTGGCCTGGAGGGCAGCGTGGAGAGGCTAC
ATCTCGCTACCAGTCAACGGGAGTGGCTGTCGATATACGAACTTAAGGTGGGCACTGCAG
ACACATTGTTACATTTCTGCTCCATGCGGTGTGACTGTACAGCAAAGCAGGCAAAGCAGT
TTCTTCAACAAATTGACAGCTGATGAACTATGGAAAGGAGTTCTGGCAAACACTTCTTCG
AGACAAAAGAAAGGAAGAGGAAAGCGGTCAAAGAAAAAATTAAGGAAGGATCTCAATAAA
GGGCAGATTATTGGTGAAGGGCGTTCCGGAATTCTTTGGCCTGGTCTCAGTATTCCTGTG
ATGGTGGATGGAAAAGCCCAGGTAATTTCCCAACACAAAAAAGAGCAAAGGGAGATTCAA
GATGAAATTTTACGTCGGAGGGATGAATGGGAAAAAAAAAGAAAAGTAAAGGTGAAGAAA
GAGAGAGGATGGACTGGACGATCCTGGGGGGGCATCAGTCTAGGACCTCCTGACCCTGGT
CCTAATGGAGAAACATATGAAGACTTTGACTGCCAAGTACTTGAGCTGAAAAGCGTAGCT
TGTATGACAGGAAGAGAAGGGCAGAAAAAATCTGCGAGTGCCCTTGTGGTTGTTGGAAAT
GGAAATGGGGCTGCAGGTTTTGCATTGGGGAAAGCAAAAGACAGACTGGTAGCTTTAAGA
AAGGCAAAGAACCAGGCAATTCACTATTTACACTATATAGAACGATACCAGGACCATACA
ATTTATCATGACATTACGACAACTTTTAAAAAAACAACCATCCAGATGAGAAAAAAACAC
AAAGGGTATGGTCTACATTGTCACCGAGCTATTATCACTGTCTGCAAACTCATCGGGATT
ACAGACATGTATGCCAAAGTGTGTGGATCCCCTCATATGCTTAACCTCACCAAAGCACTC
TTTAAGGGATTGGCAAACCAGAAAACCCACCAGGACTTGGCAAATGAGAAGAGCCTCCAC
GTGGTGGAATTTCGTGAGGAGCAAGGCCCATTGCCTATCCTTGTGGCTTCCCCTCAGGGT
GCAGTTAGAAAGGATCCTGAGACAATAGAGGAGGTTCCAGATGTCAAGCTGGAGTGGAAT
GAAGTAAGGGTGGCACACAGAAGGGGAAGTTCTGTCTGGCCCAGTGTCAAGAGAATGGCA
TGT
>tropical_clawed_frog_MRPS5
ATGGCGGCGCTGAGGTGGTGCAGTAGTGTCCTCCGTACAGCATGTAGAGGAGGAATCTTC
TTGCCAACAGTACGAAGCAGCTGCCAGAACAGTCACTTGGCCAACATACTTCAAGTGCAC
CCATGTGTTTCTGTACTTCACAGTGTTACTATTCAACAAAACAGGCAAAGCAGCTTCTTT
AACAAGTTGACTGCAAATGAACTATGGAGAGGAGTTTTGGCAGAAAGTGGGACAGGTGCA
CGGAAGGGAAGAGGCAAAAGAACAAAGAAAAAGCTTAAGAAAGATCTCAACCGCGGACAG
ATCCTTGGAGAAGGGAGATCTGGATATCTATGGCCTGGGTTGAATGCACCTATTGTAAAG
AGTGGCTCAGTACAGACCATTGCAAAGAGGGATCAGGCACAGCAGGAGGAAATGCAAGCA
GAAATCATAAAGCAACGAGACGAATGGGACAAAAAGAGGAAAATGAGAATCAAACGAGAG
AGAGGCTGGACTGGGAACAGTTGGGGTGGTGTTAGCATTGGAGTCCCAGATCCAGGGCCT
AATGGAGAAACCTATGAAGATTTTGACTGCAGAGTAATTGAGGTTAAAAATGTTTTCAAC
ATGACTGCTAAAGAAGGGAGAAAGAAATCTGTGAGTGCCTTGGTTGTTGTTGGGAATGGA
AATGGAGTGGCAGGATTTGCTCTTGGTAAGGCCAGTGACAGAATGATAGCTTTAAGAAAA
GCAAAAAACAGAGCTGTTCACTATCTGCACTATATAGAGCGATATAACGATCATACTATA
TACCATGACGTAACTTCTTCTTTTAAGAGGACATCAATAAGAATGAAGAAACAAAACTTA
GACTTATGGTGTGAAGATCGAAATCCAGAAAACGCCAGATCCCAAGCATTCTACTTAACA
GGATATGGTCTTCATTGCCATAGGGCAATAATCACTATCTGCAAATTAATTGGCATCACA

GACATGTATGCAAAGGTTTCTGGATCGATCAATCTGCTGAATCTTACAAGGGCACTTTTC
CTTGGTTTTGCAAAGCAGGAGACACACCAGGAACTTGCAGACAATAAGGGTCTTCACGTG
GTTGAATTTAGAGAGGAGCGGGGCCCCTTGCCTGTCATCGTAGCCTCTCCCAAGGGAGCC
CTCCGAAAAGAACCAGAGCTTGAAGGAGAGGTTCCTGACACCAAACTGGACTGGGACGAT
GTGAAAGCAGCTCAAGGAATGAAGCGTTCTATTTGGGCAAATGTCAAGAGGACATCTTGG

>Flycatcher_MRPS5
GCCGGCCGGGCGTGTGCCCGCGGCGCGCTGCGAGCGGCCTGGCGAGGATTTTCTTCTGTG
CCAGTGAAAGGGGGTTGCAGTTCCTACTCCAGCCTGGCATGGGCTTTCCAGACACAGTGC
TCCCTCTCTGCTCCCTGGACTGGGACAGTCCAGCAGTGCAGGAAAAGCAGTTTCTTCAAT
ACCTTGACAGCTGATCAGCTGTGGAAAGGAGCTTTGGCAGAGACTGGCGTGGGAGTAAAG
AAAGGAAGAGGAAAGAAAAGGAAGAAAAAGCTAAGGAAGAATCTCAATAGAGGCCAGGAG
ATTGGTGAAGGACGTTCTGGTTTCCTCTGGCCTGGTCTTAATGCTCCAGTGTTGCAAACT
GGGAAAGTGCAGCAACTTGCCCAACGAAAAAAAGAGGAACGAGAGAGAATTCAGACTGAA
ATTATTCAGCAGAGAGATACATGGGAGAAGAGAAAGAAAATAAAAATTAAGAGAGAGGGA
GGATGGAGTGGAAAGTGCTGGGGAGGGATCATTTTGGATCCTCCTGACCCTGGTCCTAAT
GGAGAAACTTATGAAGATTTTGAAACAAGAGTCATCGAGGTGAGAAATGTATTTTGTATG
AAGGCAAAGGAGGGCAGAAAAAAGTCAGTACGTGCCTTAGTGGCTGTTGGCAATGGTAAA
GGGGCTGCAGGTTTTGCAATAGGGAAGGCAGGTGACAGGACGAATGCTTTAAGGAAAGCA
AAGAATAAGGCAATAAGCTCCTTACACTTCATAGAGCTGTATCAGAACCACACAATTTAC
CACGACATTTCTGTGAAATTTAAAAGGACAAAAATTCGCATGAAGAAACAAAACAGAGGG
TATGGTCTGCATTGCCACCGAGCCATTATCACCATCTGCAGGCTAATTGGCATTAAGGAC
ATGTATGCCAAGGTCACTGGATCCAAAAACTTGATTAACATCACCAGAGCTCTCTTTAGA
GGCTTGACCCTTCAGGAGACTCACCAGCAGCTGGCAAACCAGAAGAGCCTCTACGTGGTG
GAGTTCCGGGAGGAGCAGGGCCCCCTGCCCATCGTGGTGGCCCTGCCCGAGGGCACTGTC
CGAGAGGAGCCTGAGCCAGAGGACGAGGTTCCGAACACAAAGCTGGAGTGGAGGGAGGTG
AAGGAAGCCCAGGGAATGCTGAAATCTCCCTGGGCAAGTGTCAGACGGGCGGCGTGC
>Armadillo_MRPS5
ATGGCGGCGGCGGTGCGCGCTGCGGGCTGCCTCCGCGCGCTGAGCGGCGCGCGGGCGGGT
CATTTCTGGTCGAGGCAGCTTTATCTAAACACCTTCTCAGCAGCTTCCATTTTAGCTCTG
AAGACAGGCCTCAGCATTGGCCCTTTGTCGCCCCCAGGACCCAGAGATCACCGACCTTTT
GCCAGCTTGAGCCATGCGCTCCAGACACAGTGCTGTATTTCTTCTCCCAGGAGCTTGGTG
GGCCAACAGCATAGACCCTACAGCTTCTTCACTAAGTTGACAGCAGATGAGCTCTGGAAA
GGAGTTCTGGCTGAGACTGGCGCTGGAGCAAGAAAAGGAAGAGGCAAAAGGAGCAAGAAA
AAGAGAAAAAAGGATTTGAACAGGGGTCAGATCATTGGTGAAGGGCGTCACGGCTTCCTG
TGGCCGGGTCTGAACGCCCCTCTCATGAGGAATGGGGCCGTGCAGACCATTGCCCAGAGG
AGCCGGGAGGAGCAGGAGAAGGTGGAGGCCGACATGGCGCAGCAGAGAGAAGAGTGGGAG
CGGAAGAGGACGGCGAAGGTCAAGCACGCGCGAGGATGGAGCGGGAACTCGTGGGGCGGC
CTCAGCCTCGGCCCCCCCGACCCTGGCCCTCACGGAGAAACGTATGAGGATTTTGATACG
CGGATACTCGAGGTTAGAAATGTGTTCAACATGACCGCAAAAGAGGGAAGAAAGAGATCA
GTCCGCGTCCTGGTGGCTGTGGGGAATGGAAGAGGAGCTGCAGGTTTTGCCATTGGGAAA
GCTGCTGAACGGGTGGATGCATTCAGAAAAGCAAAGAACAGAGCAATTCACTATTTGCAC
TACATAGAACGCTATGAAGACCATACAATCTTCCATGACATTTCTTTAACATTTAAAAGG
ACACATATCAAGATGAAGAAACAGCCCAGAGGTCACGGCCTCCGCTGCCACCGCGCCGTC

ACCACCATCTGCCGGCTCATCGGCATCAAGGACATGTACGCCAAGGTCTCGGGCTCCATG
AACATGCTCAGCCTCACGCGGGGCCTCTTCCGCGGGCTCTCGCGCCAGCCTGATTCAAGC
AAATTGTGTAAAAACCCAATCTTTTTTTATCTGAAAAGTGAGGCCATCCGAACAATTCCT
GGATATTTGAGGATAGTGAGTCACTGGTATGGTGGTATTGTGCGACCTTTTAAAGTGCCT
GTTTTAGAGATAGATGCCGAAATACCTACGGATGATGTGGAACGGTATCTGGGGCTTAGG
CGCACTGTGTGGTCGGGCTTGAAGAGGGCCGCCACG
>Chicken_MRPS5
ATGGCGGCGGCGGTGGTGGCGGCGGCCGGCCGGGTGTGCGCCTTTGGTGTCGTGCGAGCG
GCCTGGCGAGGGTTTGCTTCTGTACCAGTGAAAGGGAGCTGCTGTTCCTATTCTAGCTTG
GCGTGGTCTCTCCAGGTGCGGTGTTCCATCTCTGTCTCTCAGAACGTGATTGTACAGCAA
TGCAGGCAAAGCAGCTTCTTCAATACATCGACAGCTGAGGAGCTATGGAAAGGAGCTTTG
GCAGAGACTGGTGTGGGGGTAAGGAAAGGAAGAGGAAAGAGAAGGAAGAAAAAGCTGAAA
AAGAATCTCAACAGAGGCCAGGAGATTGGGGAAGGACGCTCTGGTTTCCTCTGGCCTGGC
CTTAATGCTCCTCTGATAAAAAGTGGGAGAGTACAGACACTTACTCAAAGGAAAAAAGAA
GAACGAGACAAAATCCAGTCTGAGATTATTCAGCAGAGAGATACGTGGGAGAAGAAAAGA
AAAATAAAAATCAAGAGAGAGGGAGGCTGGAGTGGAAAGTGTTGGGGAGGTGTCCTTTTG
GATCCTCCTGACCCAGGTCCTAATGGAGAAACTTACGAGGATTTTGAAACAAGAGTCATT
GAGGTGAAAAATGTGTTTTGTATGAAGGCGAAGGAAGGCAGGAAAAAATCAATACGCGCC
TTAGTGGCTGTTGGAAATGGTAAAGGGGCTGCAGGTTTTGCACTGGGGAAAGCAGGTGAC
AGGATGAACGCTTTACGGAAAGCAAAAAACAAAGCAATACGCAGCTTACATTTTATAGAG
CTATACCAGAACCACACAATTTACCATGATGTTACAGTGAAATTTAAACGCACACACATC
CGCATGAAGAAGCAAAACAAAGGGTATGGTCTTCGTTGCCACCGAGCTATTATTACCATC
TGCAAACTAATTGGCATTAAAGACATGTATGCCAAGGTTACTGGATCCAAAAACTTGATT
AATATTACCAGAGCTCTCTTTAAAGGATTGACACAACAGGAGACTCACCAGCAGTTGGCA
AACCAGAAAAACCTCTACGTGGTGGAATTCCGTGAGGAGCAAGGCCCACTGCCCATTGTC
GTGGCGCTGCCCGAGGGGACTGTCCGCGAGGATCCTGAGCCTGAGGATGAGGTTCCAGAC
ACTAAACTGGAGTGGTGTGAGGTGAAAGAAGCTCAGGGAATGAAGAAATCACCCTGGGCA
AATGTTAGACGGACAGTATGG
>Chinese_softshell_turtle_MRPS5
ATGAACCTGGCATGGACTGTAGAGACGCGCTGTTACATTTCCGCTCCATGTAGTGTGGCA
ATACAACAATGCAGGCAAAGCAGTTTTTTCAACAAATTGACAGCTGATGAGCTGTGGAAA
GGAGTTTTGGCAGAGTCTGGTGCTGGAGCAAGGAAGGGAAGAGGAAAGCGAACCAAGAAA
AAGTTTAAAAGGGATCTCAATAGAGGCCAGATCATTGGTGAAGGACGTTCTGGTTTCCTT
TGGCCTGGCCTCAATGCGCCTCTGCTGAAAAGTGGAGCAGTCCAGACAATAAGTCAGCGC
GACAAAGAGGAGCAGGAGAAGGTACAGTCTGAAATTATCCGGAAGAGGGATGAGTGGGAG
AAGAGAAGAAAAACAAAGGTGAAGAGAGAGAGAGGCTGGACTGGAAATTCTTGGGGAGGC
ATCAGTCTGGGACCACCTGACCCAGGTCCCAATGGAGAAACTTACGAAGACTTCGACTCC
CGGGTAATTGAGGTGAAAAATGTATTTAATATGACAGCAAAGGAAGGGAGAAAAAAATCT
GTTAGTGCTCTAGTGGCTGTTGGAAATGGAAATGGAGCAGCAGGTTTTGCAGTGGGGAAA
GCAAGTGACAGGATGACAGCTTTAAGAAAAGCAAAGAACAGAGCAATTCACTATTTACAC
TATGTAGAGCGATACCAAAATCACACAATTTATCATGATATTACAGCGACTTTTAAAAAA
ACAACCATCCGGATGAAAAAGCAAAACAAAGGCTATGGTCTTCATTGCCATCGAGCCATC
ATTACCATCTGCAAGCTAATTGGGATTACAGACATGTATGCCAAGCTTTCTGGATCCAAC
AATTTACTTAACCTCACCCGAGCTCTCTTTAAGGGACTAGCACGCCAGGAGACCCACCAG

GAGCTGGCAGAGAAGAGGAGCCTCTACGTGGTCGAATTCCGGGAGGAATGTGGCCCCCTG
CCCATCGTCGTTGCTGCTCCAAGTGGCGCCGTCAGGAAGGAACCGGAGCCTGTGGAAGAG
GTTCCCGACATCAAGCTGGAGTGGAGCGAAGTGAGAGCTGCCCAGGGAATGAAGCGTTCT
CCCTGGGCACATGTCCGAAGGACCGTGTGG
>Cod_MRPS5
atgatgatgatggcggtggcggtggcgtgtgttccagggagggcgggcttcctgtggcct
gggctgaacgccccggtgttccgggccggagcgccgcaggccgtctccagacggggggac
gcagagcagctagagatgcaggcagaactagtgcgttccagagatgagtgggagaagagg
aggaggatgaaggtgaagagaggaagaggatggaccggaaactcctgggggggcatcagt
ctgggggccccggaccctgggccaaatgggg
>Cow_MRPS5
ATGGCGGCGGCGGTACGCGCTGCGGGCTTCCTCCCTGCTCTGTGTGGCGCTTCGGCGGGT
CGTTTATGGTCCAGGCAGCTTTACCTAAACACCTTTCCAACAGCTTCCATTTGGGCGTTG
AAAGCTGTTCCCAGCAATGGCCCTTCGTCATCTGCAGGAGCCAGAGGCCGCTGCCGTTCT
ACCCACTTGGGCCCCGCGCTGCAGACACAGTGCTGCACTCCTGCTCCCGGGAACGTGACG
GCCCAGCAGTACAGATCCTATAGTTTCTTCACTAAACTGACAGCAGATGAGCTATGGAAA
GGTGCTTTAGCAGAAACCGGAGCTGGAGCAAGAAAAGGAAGAGGCAAAAGAACCAAGAGA
AAAAGAAGGAAGGATTTGAACAGGGGTCAGATCATCGGCGAAGGGCGTCGTGGCTTCCTA
TGGCCTGGTCTGAATGCCCCACTTATGAAAAGTGGAGCTATACAGACCATCACTCAAAGA
AGCAAGGAAGAGCAAGAAAAGGTGGAGGCCGATATGGTCCAACAGAGAGAAGAGTGGGAC
CGGAAGAGGAAGATGAAGGTTAAACGGGAGCGAGGATGGAGCGGAAACTCGTGGGGCGGC
ATCAGTCTTGGCCCCCCTGACCCCGGTCCCAATGGAGAAACGTATGATGATTTCGATACC
AGGATACTTGAGGTGAGAAATGTTTTCAACATGACGGCCAAAGAGGGAAGGAAGAGATCC
GTCCGTGTCCTGGTCGCTGTGGGGAACGGCAGAGGAGCCGCAGGTTTTGCCATTGGGAAA
GCCACTGAACGGGCAGATGCTTTCAGAAAAGCAAAGAATAGAGCCGTTCACTATTTGCAT
TATATAGAACGATATGAAGACCATACCATATACCACGATATTTCCTTAACATTTAAAAGG
ACACATATCAAGATGAAGAAACAACCCAGAGGCTATGGCCTCCGCTGCCACCGAGCCATC
ACCACCATCTGCCGGCTCATCGGCATCAAAGACATGTATGCCAAGGTCTCTGGCTCCGTC
AACATGCTCAGCCTCACCCGGGGCCTCTTCCAGGGGCTCTCTCGCCAGGAAACCCACCAA
CAGCTGGCTGATAAGAAGAGTCTCCATGTTGTGGAATTCCGGGAGGAATGTGGCCCTCTG
CCCATCGTGGTTGCCTCACCACAGGGGGCCTTGAGGAAGGATCCGGAGCCAGAAGATGAA
GTTCCGGATATCAAATTGGACTGGGACGATGTCAAGGCTGTGCAGGGAATGAAGCGCTCT
GTGTGGTCAGGTTTAAAGAGAGCTGCCACG
>Dog_MRPS5
ATGGCGGCGGCGGTGCGCGCTGCGGGCCGCCTCCCTGCGCTGTGCGGCGTGCCGGCGGGT
CACATATGGTCCAGGCAGCTTTACCTAAACACCTATCCAACAGCTTCCGTTTTGGCATTG
AAGACTGTTCTCAACAATGGCCCTTTGTCATATCCAGGAACCAGAAACAACCATCATTTC
ATCAGCTTGACCCGTGCACTACAGACACAATGCTGTATTTCTTCTTCCAGTAATTTGATG
GGTCAACAGTACAGATCCTATAGTTTCTTCACTAAATTGACGGCAGATGAGCTTTGGAAA
GGCGCTTTAGCAGAGACTGGAGCAGGAGCAAGAAAAGGAAGAGGCAAAAGATCTAAGAAA
AAGAGAAAAAAGGATTTGAATAGGGGTCAGATCATTGGTGAAGGACGTCATGGCTTCCTA
TGGCCTGGTCTGAATGTCCCTCTTATGAAAGAGGGAGCTATGCAGACCATTGCTCAAAGA
AGCAAGGAAGAGCAGGAAAAGGTGGAGGCTGATATGATCCAGCAGAGGGAAGAGTGGGAC
CGGAAGAGGAAGATGAAGATTAAACGGGAGCGAGGATGGAGTGGAAACACATGGGGAGGC

GTCAGTCTTGGCCCCCCTGACCCTGGTCCCAGTGGAGAAACATATGATGATTTTGATACC
AGGATACTTGAGGTAAGAAATGTATTCAATATGACAGCAAAAGAGGGAAGAAAGAAATCA
GTCCGTGTCCTGGTTGCTGTGGGGAATGGTAGAGGAGCTGCAGGTTTTGCCGTTGGGAAA
GCCACTGACCGGATGGATGCTTTCAGAAAAGCAAAGAACAGAGCAGTTCACTATTTGCAT
TATATAGAACGATACGAAGACCATACAATATTCCACGATATTTCTTTAAGATTTAAAAGG
ACGCATATCAAGATGAAGAAACAACCCAGAGGCTACGGCCTCCGCTGCCATCGGGCCATC
ATCACCATCTGCCGGCTCATTGGCATCAAAGACATGTATGCCAAGGTTTCTGGGTCTGTC
AACATGCTCAACCTCACACGGGGCCTCTTCCATGGGCTCTCCCACCAGGAAACCCATCAA
CAACTGGCTGATAAGAAGAGTCTACATGTCGTAGAATTCCGGGAAGAATGTGGCCCTCTG
CCCATCGTGGTTGCCTCCCCCCAGGGAGCCTTGAGAAAGGACCCAGAGCTAGAGGATGAG
GTTCCAGACATCAAACTTGACTGGGAAGAAGTGAGGGCTGCGCAGGGAATGAAGCGCTCA
GTGTGGTCAAATTTGAAGAGAGCTGCCACC
>Dolphin_MRPS5
ATGGCGGCGACGGTGCGCGCTGCGGGAATTCTCCCTGTGCTGTGTGGCGCGCCGGCGGGT
CATCTATGGTCCAGGCAGCTTTACCTAAACAGCTTTCCAGCAGCTTCCATTTTGGCATTG
AAGACTGTTCCCAGCAATGGCTGTTTGTCATCTCCAGGAACCAGAGACAACCGTCATTTT
ATCAGCTTGACCCGTGCGCTACAGACACAGTGCTGTATTTCTTCTCCCAGTAACTTGATG
GGCCAACAGTATAGATTCTATAGTTTCTTCACTAAATTGACAGCAGATGAGCTGTGGAAA
GGTGCCTTAGCAGAGTCTGGTGCCGGAACAAGAAAAGGAAGAGGCAAAAGAACTAAGAAA
AAGAAAAGAAAAGATTTGAACAGGGGTCAGATCATCGGTGAAGGGCGTTATGGCTTCCTA
TGGCCTGGTCTGAATGTCCCTCTTATGAGAAATGGAGCTGTGCAGACCATTGCCCAAAGA
AGCAAGGAAGAGCAGGAGAAGGTAGAGGCGGATATGGTCCGGCAGAGAGAAGAGTGGGAC
TGGAAGAGGAAGATGAAGGTTAAACGGGAGCGAGGATGGAGCGGAAACACATGGGGAGGC
GTCAGTCTTGGCCCCCCTGACCCTGGTCCCAACGGAGAAACATATGATGATTTCGATACC
AGAATACTTGAGGTGAGGAATGTTTTCAACATGACAGCAAAAGAGGGAAGAAAGAGATCA
GTCCGTGTCCTGGTCGCTGTGGGGAATGGCAGAGGAGCTGCAGGTTTTGCCATTGGGAAA
GCCAGTGAACGGGCAGATGCTTTCAGAAAAGCAAAGAACAAGGCAGTTCACTATTTGCAT
TATATAGAACGATATGAAGACCATACAATATACCATGATATTTCTTTAAGATTTAAAAGG
ACGCATATCAAGATGAAGAAACAAACCAGAGGTTATGGCCTCCGCTGCCACCGGGCCATC
ATCACCATCTGCCGGCTCATTGGCATCAAAGACATGTATGCCAAGGTCTCTGGCTCCGTC
AACATGCTTAACCTCACCCGGGGCCTCTTCCACGGGCTGTCCCGCCAGGAAACCCATCAA
CAGCTGGCTGATAAGAAGAGTCTCCATGTTGTGGAATTCCGGGAGGAATGTGGCCCTCTG
CCCATCGTGGTTGCCTCCCCCAAGGGGGCCTTGAGAAAGGATCCGGAGCCGGAAGATGAG
GTTCCAAACATCAAACTGGACTGGGAAGATGTGAAGGCTGCCCAGGGTATGAAGCGCTCT
GTGTGGTCAAGTTTAAAGAGAGGCGCCACC
>Duck_MRPS5
ATGCCTGGCTTTCAGCTTGAGAAATTGTTCTTCACATCAACGTTTTGCCAAGGGTTTGCT
TCTGTGCCAGTGAAAGGGAGTTGCTGTTCCTATTCCAGCCTGGCATGGGCTCTCCAGGTG
CAATGTTCCATCTCTGCCCCCCGGAATGTCATGGTACAGCAATGCAGACAAAGCAGTTTC
TTCAATATGTTGACAGCTGATGAGCTCTGGAAAGGAGCTTTGGCAGAGACTGGTGTGGGA
GTAAAGAAAGCAAGAGGGAAGAGAAGGAAGAAGAAGCTAAGAAAGAATCTCAATAGAGGC
CAGGAGATCGGAGAAGGACGTTCTGGTTTCCTCTGGCCTGGCCTTAATGCTCCTATAATA
CAAAGTGGGATGGTACAGACAGTTTCCCAACGAAAAAAAGAAGAACGAGAGAGAATTCAG
TCTGAAATTCTTCAGCAGAGAGACACATGGGAGAAGAGAAGAAAAATAAAAGTTAAGAGA

GAGGGAGGATGGAGTGGAAAGTGTTGGGGAGGTGTCATTCTGGATCCTCCTGACCCAGGT
CCTAACGGAGAAACTTACGAAGATTTTGAAACAAGAGTCATTGAGGTGAAAAATGTGTTC
TGTATGAAGGCAAAGGAAGGCAGAAAAAAATCAATACGTGCCTTAGTGGCTATTGGAAAT
GGTAAAGGGGCTGCAGGTTTTGCATTGGGGAAAGCAGGTGACAGAATGAATGCTTTACGA
AAAGCAAAGAACAAAGCAATACGCAGCTTACATTATATAGAGCAGTATCAGAACCACACA
ATTTACCATGACATTACTGTGAAATTTAAAAGAACAACCATCCGCATGAAGAAGCAAAAC
AAAGGTTATGGTCTTCATTGCCACCGAGCTATTATCACCATCTGCAAACTAATTGGCATT
AAAGACATGTATGCTAAGGTTACTGGATCCAAAAACTTGATTAACATTACCAGAGCTCTC
TTCAAAGGACTGACACAACAGGAAACTCACCAACAGTTAGCAAACGAGAAGAACCTCTAT
GTTGTGGAGTTCCGGGAGGAGCAGGGCCCACTGCCCATCGTTGTAGCACTGCCTGAGGGG
ACCGTCCGTGAGGATCCTGAGCCCGAAGATGATGTTCCAGACACTAAGTTGGAGTGGAGC
GAGGTGAAAGAAGCTCAGGGAATGAAGAAATCACCCTGGACAAATGTCAGGCAGAGAGTA
TGG
>Elephant_MRPS5
ATGCAGCATCGCTTGACCACGGAACTCACTTTGCAGCAGACGAAGTGTGGCAGTGAGCTC
ACGCTTGTGGAATTCACTGGTCTTACCACATTCCCCATCATCCTGAAAGAGGATTTTCAC
TGCACTTCGTCCCCTTCCGTGCTCGTCTGTACCTTGCACTTCGGTGAGAAGCGCCTGCCT
GGCCCGGCTCCCAGCCCAGGCCCCGCCCACGCCCGGCCCCCAGCCCAGGCCCCGCCCACG
CCCGGCCCCGAGCCCCGAAGCCCCGTCCTCATCCCGGCCCCCAGCCCAGGCCCCGCCCAC
GCCCGGCCACCACGCCGCCCGCTGTGTGACCTTGCGCGCCTCCTCGCCCTCTCTGGCCGC
GGAAGCCTGGCTGCAGGCGAGGCCTCCTGGACGGCGTCCGCGGCCGCTCGTGTGCTCCGG
GGAGGAGCGGGCGGCCGGGCGGGCGGCCCTCGTCGGCTTCCCGGGCAGCGTGCCCCGCGG
GCTCCCGCCGCCATGTGGGGCCGTGGGGGGTCCGGGGGTCCGGGGGGCGAGCCGCCGTCA
GGAGCCGCCGTGAGGAAGGGGGTGCCGGCGTCGCTGCTCGCTGTGGCCGCCCTTCCCCTC
CCGCCCCCGCTCCCGCCTTCGCGGCCGGGCAGCTCGGTCTCCCCGCGGCCCAGGCCTTGC
CTCCGGGAACCGCTGACAGCAGAGGAGCTCTGGAAAGGAGTTTTAGCAGAGACTGGTGCT
GGAGCAAGAAAGGGAAGAGGCAAAAGAACCAAGAAAAAGAGAAGAAAGGATTTGAACAGG
GGTCAGATCATTGGTGAAGGGCGTTCTGGTTTCCTCTGGCCTGGTCTGAACGTTCCCCTT
ATGAGAAATGGAGTTGTGCAGACCATTGCCCAAAGAAGCAAGGAAGAGCAGAAGAAGGTA
GAGGCCAACATGGTCCGGCGGAGAGAAGAGCAGGAACAGAAGAAGAAAATTAAGGTTAAA
CAGGAGCGAGGATGGAGCGGAAACACGTGGGGAGGTGTCAGTCTTGGCCCTCCTGACCCT
GGTCCCAATGGAGAAACATACGAGGATTTTGATACCAGAGTACTTGAGGTAAAAAACGTT
TTCAATATGACAGCAAAAGAGGGAAGAAAGAAATCGGCCCGTGTCCTGGTAGCTGTGGGG
AATGGCAGAGGTGCTGCAGGTTTTGCGGTTGCGAAAGCTGCTGAACGGACGGACGCTTTC
AGAAAAGCCAAGAACAAAGCAGTTAACGTTTTGCATTACATAGAACGCTATGAAGACCAC
ACAATCTTCCATGATGTTTCTTTAACGTTTAGAAAGACGCATATCAGGATGAAGAGACAA
CACAGAGGTTACGGCCTCCGCTGCCACCGGGCCATCATTACCCTGTGCCGGCTCGTTGGC
ATCAAAGACATGTATGCAAAGGTGTCGGGACCCGTCAATATGCTCAGCCTCACCCGGGCC
TTCTTCCATGGACTGTCCCACCAGGAGACTCACCAGCAGCTGGCTGACAGGAAGAGGCTG
CATGTGGTGGAATTCCGGGAGGAGTGTGGCCCTCTGCCCATCGTGGTTGCCTCCCCCCAG
AGCACCTTGAGAAAGAATCCAGAGCCAGAAGAGGAGGTTCCTAATATCAGACTGGACTGG
AACGATGTGAAGACCATGCAGGGAATAAAGCGCTCTGTGTGGTCAGGGTTGAAGAGAGGC
GCCACC
>Fruitfly_MRPS5

ATGAGTCAAACATTGATCCGTCGTCTCTTCAATCAAGCTTGTAGCAGAAATATTTTTATT
TTGAACCAAAGCTTGTTATCTTCACATGAACCCATTTTACCAGGTTACATTGGTTTACGT
AATACCAGCTTCTTTAACAAATTACCTGCTGAAGACATTTGGCGCGGAGTAACTGCGGTT
AGCAATGCTGGAAAAAAAAGAGGCCGTGGGAAAGGAAGTGGAAAAAAAGTTGCAAAGGAT
CTTAACAAAGGGCAATCTATCGGATTCGGAAAATGCGGTCGCATATGGCCTGGATTGAAT
TCACCTCTTATACGAGGAAACGAGTTAATTAATCAGCAGAAACTCAATGAGAATTTAGAT
AGAGAAAATGGTATTTTAAAATTACGCGATTCAATGGGAAGTTTTAAATTGATGAAACTA
AATCCAATAGACCGAGGTTGGTCTGGAAGTAAAATGCCCGGCCGAAGCATTGGACCTCCT
GATCCTGTTGGCGACGAAGAGTTTGTGTCATTTGATACTAGAGTTTTAGAAAACAAAATT
GTTTTTATTATGAAAGGAAATATGGGAAGAAAACGAAGATATTCCGTTCTTTCAGTCACG
GGAAATGGTAATGGTTTGGCAGGATTCGCTACCGCTAAAGCGCCAGAAGTTCGTACGGCC
CTTCGAAAATCAAAGAACCGAGCAGGACAAAAACTAATCAATATAAGCCTTTGTGAAAAT
AGAACAATTTTTCATGACTTTCGTACAGATTTCGGAAAAACAAAAATTTTTTGTTTTCAG
AAACCTGATGGATACGGCTTAGTCTGTCACCGGGCAATTCAGACAATTTGTAAAGTTATA
GGTATCAAGGATTTATATGCCAAAATTGAGGGGTCTACGAATATCCAGAATATTGCAAAG
GCATTTTTTATTGGTCTGATGACGCAAAGGTCCTATCAGTATATTGCTAACGAAACAAAT
TCAAACATTGTTCAACTGCAAAAGACAAGAAATAGATTCATTGATGTAAAAGGAATGCCT
TTTCCTAATTCAACTCTAACGCAGAACACCCAAGAAATTGACTATATGCATTTCACATTA
GGAAATCAAATAGTTTTACAGCGAAAAGGCACAGTACCATTTTATGTTTATAGCAAAGGA
CATGACTTGCATCAATTTAAAAAGGAGCGATTTCGGAACCAAGTTAATGTTCGGCTACAC
AAGCTAGTTAATGCTTATGAT
>Fugu_MRPS5
ATGGCGGCGTCCATACGGGTGTGCTTTACCCTCCGCTTTGTACTTCGAGGTGCATCTTCA
CTCCATACCCTTGCAGGGCCAGTTCAGTTGTCCCAGTTTGGGTGTGGAGCCTTCCAAAGA
AACCCCACACTTCCATTGTTACCAGTGGCAGCAGGCCAACAGGCTAGACATAGCAGCTTC
TTCAACAAATTGACAGCAGAAGAATTATGGAGAGGCGTTTTGGCAGAAACTGGTGCCGGA
AGCAAAAAGGGCCGAGGAAAAAGAACAAAACGCAAATTGAAAAAGGATCTGAACCGTGGA
CAGAGGATTGGAGAAGGTCGTGGAGGATTCCTCTGGCCTGGTTTGAATGCTCCTGTTCTG
AAGGCTGGGGTTGTTGGGAGCATTGGGCGCAGGAGCGAGTCGGAGCAGCAGGATATAAAA
GCTGAACAAGTGCGGCAAAGGGAGGAATGGGAAAAGAGGAGGAAGACGAAGGTGAAGAGG
GAAAGAGGTTGGACCGGTCACTCATGGGGGGGCATCAGCCTTGGACCCCCTGATCCTGGG
CCTAATGGAGAGACTTATGAAGACTTTGATACTCGTGTCATTGAGGTGAAGAATGTGTTC
AACATGACAGCCAAGGAAGGTCGGAAGAGGTCCACCAGCTGTCTGGTTGCTGTTGGAAAC
GGCAACGGAGCTGCAGGCTTTGCGCTGGCTAAAGCAGCAGACAGAAACACGGCTCTGAGA
AAGGCTAAAAACCGAGCGATCCACTATTTATATTACATAGAGCGACACAACGACCACACC
ATTTATCACGACATTGAGTCCAAGTTCAAGAGGACAACACTTCGCATGAAGAAGCAAAAC
GAAGGTTACGGTCTGCACTGCCACCGAGCTGTCATCACTCTGTGTAAGCTGATCGGCATC
AAGGATATGTACTGCAAATTAGAAGGATCTGCCAATCTCCTCAATATCACCAGGGCTCTC
TTCACTGGGCTGGCCAATCAGGAAACCCACCAGAAGTTGGCTGACAAGAAGCAGCTCCAC
GTGGTCGAGTTTCAGGATCACCAAGGCCCGCTGCCTCAGGTGGTGGCGAGCCCCAAAAAT
GGCGCGCGACCTGACGCGGAGCCCGAGGATGAGATCCCCAACACACGACTGCACTGGGAC
GACGTACGAGCGTCACAGGGACTCAAACGCTCCATCTGGGCGGGCGTCAAGCGCACCATC
TGG
>Anole_lizard_MRPS5

ATGGCGGCCGCCGTAGCCGCTCTGGTGCAGAAAGTCTGCAGTTCGGGCGTCTTGAGGGCG
GCGCGGAGTGGCTATACCCTGATTTCATGGAATGGAACCAAGTGTCAATATACTACCTTG
GCATGTGCTTCACAGACACTTTGCTATATTTCTACACCTTGTAACATCGCAATACAGCCC
TGCAGGCAAAGCAGCTTTTTCAACAAATTGACAGCTGATGAACTCTGGAAAGGAGTTTTG
GCAGAGAGTGGCGCAGGGGGAAGGAAAGGAAGAGGGAAGAGGACCAAGAAAAAGCTAAAG
AAGGATCTCAACAGAGGCCAGGTCATTGGGGAAGGACGTTCAGGTTTCCTTTGGCCTGGC
CTCAATGCTCCAGTGATGAAACAAGGAACAATGCAGGCAGTTAGCCAGCGCAATAAAGAA
GAGCAGGAGAAGCTGCAGGCTGAGAGACTCCAGAAGCGAGACGAGTGGGAGAAGAGGAGA
AAAACGAAGGTGAAGCGGGAGAGAGGCTGGACAGGAAGCTCATGGGGAGGAATCAGTTTG
GGACCTCCTGATCCAGGTCCCAATGGAGAAACATATGAAGATTTCGACTCCAGAGTGATT
GAGTTGAAAAGTGTATTTAACATGACAGCCAAGGAAGGAAGAAAAAGATCTGTTAGTGCT
CTGGTGGTTGTTGGGAATGGAAAAGGGGCTGCTGGTTTTGCACTTGGAAAAGCCTCTGAC
AGGATGGTGGCACTAAGAAAAGCAAAGAACAGAGCAGTTCATTATTTGCACTATATTGAA
AGATACGAAGATCATACAATTTATCATGACATTGCAACCTCATTTAAGAAAACAACTATT
CGGATGAAGAAGCAAAACAAAGGATATGGCCTTCACTGTCACCGAGCTATTATTACCATC
TGCAAGCTGATCGGAATTAAAGACATGTACGCTAAGGTTTATGGATCCGGGAATTTACTG
AATCTCACCCGAGGACTCTTCAGGGGATTAGCAACTCAGGAAACTCACCAAAATCTAGCA
AATAAGAAGGGCCTCTATGTCGTAGAATATCGCGATGAGTGTGGCCCACTGCCCATCATT
GTGGCAAAGCCTGATGGGGAGGTGAGACAAGATCCCGAACCTCTGGATGAAGTTCCTGAT
GTCAAATTAGAATGGGATGAAGTGAAAGCAGCCCAAGGAATGAAGAAAAGTATCTGGGCC
AATGTCAGAAGAACAGTTTGG
>Horse_MRPS5
ATGGCGGCTGTGGTGCGCGCTGCTGCGGGCTGCCTGCCTGCGGTGTGCTGCGCGCCAGCG
GGTCATTTATGGTCTAGGCAGCTTTACCTAAACACCTTTCCAACAGCTTCCGTTTGGGCA
TTGACATCTGTTCTCAGCAATGGCCGTTTGTCATCTCTTGGACCCAGAGACAACCGTCAT
TTCACCAGCTTGACCCGTGCACTGCAGACACAATGCTGTATTTCTTCTTGCAGTAACTTG
ATGGGCCAACAGTATAGATCCTATAGTTTCTTCACTAAATTGACAGCAGATGAGCTGTGG
AAAGGTGCTTTAGCAGAGACTGGTGCTGGAGCAAGGAAAGGAAGAGGCAAGAGAACAAAG
AAAAAGAGAAGGAAGGATTTGAACCGGGGTCAAATCATTGGTGAAGGGCGTCAAGGCTTC
CTATGGCCTGGTCTGAATGTCCCTCTTCTGAGAAATGGAGCTGTGCAGACCATTGCCCAA
AGAAGCAAGGAAGAGCAGGAGAAGGTGGAGGCTGAGATGGTCCAGCAGAGAGAGGAGTGG
GACCGGAAGAGGAAGATGAAGGTTAAACGGGAGCGAGGATGGAGCGGAAACACGTGGGGA
GGTGTCAGTCTCGGCGCCCCAGACCCTGGTCCCTATGGAGAAACATATGACGACTTTGAT
ACCAGGATACTTGAGGTCAGAAATGTCTTCAATATGACGGCAAAAGAGGGAAGAAAGAAA
TCAATCCGCGTCCTGGTCGCTGTGGGGAACGGCAGAGGAGCTGCAGGTTTTGCCATTGGG
AAAGCCACTGAAAGGGCGGACGCTTTCAGAAAAGCAAAGAACAAAGCAGTTCACTATCTG
CATTATATAGAACGATACGAAGACCATACAATATTCCACGATATTTCTTTGACATTTAAA
AGGACGCATATCAAGATGAAGAAACAACCCAGAGGCTACGGCCTCCGCTGCCACCGGGCC
ATCATCACCATCTGCCGGCTCATTGGCATCAAAGACATGTACGCCAAGGTCTCGGGATCC
ATGAACATGCTCAACCTCACCCGGGGCCTCTTCCGTGGACTCTCCCGCCAGGAAACCCAC
CAACAGCTGGCTGATAAGAAGAGCCTCCACGTGGTGGAATTCCGGGAGGAATGCGGCCCT
CTGCCCATCGTGGTCGCCTCCCCCCAGGGGCCCTTGAGAAAGGATCCGGAGCCAGAAGAT
GAGGTTCCAGACGTCAGACTGGACTGGGAAGAAGTGAAGGCCGCACAGGGAATGAAGCGC
TCAGCGTGGTTGGGTTTAAAGAGAGCCGCCACC

>Human_MRPS5
ATGGCGACCGCGGTGCGCGCTGTGGGCTGCCTCCCCGTGCTGTGTAGCGGGACGGCAGGT
CATTTATTGGGGAGGCAGTGTTCCCTAAACACCTTACCAGCAGCTTCCATTTTGGCATGG
AAGAGTGTTCTCGGCAATGGCCATTTGTCATCACTGGGAACCAGAGACACCCATCCCTAC
GCCAGCTTGAGCCGTGCACTGCAGACACAATGCTGTATTTCTTCTCCCAGTCACCTGATG
AGCCAGCAGTATAGACCATATAGTTTCTTCACTAAATTGACTGCAGATGAGCTGTGGAAA
GGCGCTTTAGCAGAGACTGGTGCTGGAGCAAAAAAAGGAAGAGGCAAAAGAACTAAAAAG
AAGAAAAGAAAGGATCTGAACAGGGGTCAGATCATTGGTGAAGGGCGTTATGGTTTTCTA
TGGCCCGGACTGAATGTCCCTCTTATGAAAAATGGAGCAGTGCAGACCATTGCCCAAAGA
AGCAAGGAAGAGCAGGAGAAGGTGGAGGCAGACATGATCCAGCAGAGAGAAGAGTGGGAC
CGAAAGAAGAAGATGAAGGTTAAACGGGAGCGAGGATGGAGTGGAAACTCATGGGGAGGC
ATCAGTCTTGGCCCCCCTGACCCTGGTCCCTGTGGAGAAACATATGAGGATTTTGATACC
AGGATACTTGAGGTAAGAAACGTTTTCACTATGACTGCGAAAGAGGGAAGAAAGAAATCG
ATCCGTGTCTTGGTGGCTGTGGGGAACGGAAAAGGAGCTGCAGGTTTTTCTATTGGGAAA
GCTACTGATCGGATGGATGCTTTCAGGAAAGCAAAGAACAGAGCAGTTCACCATTTGCAT
TATATAGAACGATATGAAGACCATACAATATTCCATGATATTTCATTAAGATTTAAAAGG
ACGCATATCAAGATGAAGAAACAACCCAAAGGTTACGGCCTCCGCTGCCACAGGGCCATC
ATCACCATCTGCCGGCTCATTGGCATCAAAGACATGTATGCCAAGGTCTCTGGGTCCATT
AATATGCTCAGCCTCACCCAGGGCCTCTTCCGTGGGCTCTCCAGACAGGAAACCCATCAA
CAGCTGGCTGATAAGAAGGGCCTCCATGTTGTGGAAATCCGGGAGGAATGTGGCCCTCTG
CCCATTGTGGTTGCGTCCCCCCGGGGGCCCTTGAGGAAGGATCCAGAGCCAGAAGATGAG
GTTCCAGACGTCAAACTGGACTGGGAAGATGTGAAGACTGCACAGGGAATGAAGCGCTCT
GTGTGGTCTAATTTGAAGAGAGCCGCCACG
>Macaque_MRPS5
ATGGCGACCGCGGCGCGTGTTGTGGGCCGGCTGCCCGCGCTGTGTAGCGGGACGGCAGGT
CATTTATTGGGGAGGCAGCGTTCCCTAAGCACCTTACCAGCAGCTTCCATTTTGGCGTGG
AAGAGTGCTCTGAGCAATGGCTGTTTGTCATCACTGGGAACCAGAGACACCCATCCCTAC
GCCAGCTTGAGCCACGCGCTGCAGACACAATGCTGTGTTTCTTCTCCCAGTCACCTGATG
GGCCAGCAGTATAGACCATATAGTTTCTTCACTAAATTGACTGCAGATGAGCTGTGGAAA
GGCGCTTTAGCGGAGAGTGGTGCCGGATCAAGAAAAGGAAGAGGCAAAAGAACTAAAAAG
AGGAAAAGAAAGGATCTGAACAGGGGTCAGATCATTGGTGAAGGGCATTACGGTTTTCTG
TGGCCTGGTCTGAATGTCCCTCTTATGAAAAATGGAGCAGTGCAGACCATCGCCCAAAGA
AGCAAGGAAGAGCAGAAGAAGATGGAGGCCGACATGATCCAGCAGAGAGAAGAGTGGTAC
CGAAAGAGGAAGATAAAGGTTAAACACGAGCGAGGATGGACTGGAAACTCCTGGGGAGGC
GTCAGTCTTGGCCCCCCTGGCCCTGGCCCCAATGGAGAAACATACGAGGATTTTGATACC
AGGGTACTTGAGGTAAGAAATGTTTTCAATATGACAGCGAAAGAGGGAAGAAAGAAATCG
GTCCGTGTCCTGGTGGCTGTGGGGAACGGAAAAGGAGCTGCAGGTTTTGCTATTGGGAAA
GCCACTGATCGGATGGACGCTTTCAGGAAAGCAAAGAACAGAGCACTTTACTGTTTGCAT
TATATAGAACGATATGAAGACCATACAATATTCCATGATATTTCATTAAGATTTAAAAGG
ACGCGTATCAGGATGAAGAAACAACCCAAAGGTTATGGCCTCCGCTGCCACAGAGCCATC
ATCACCATCTGCCGGCTCATTGGCATCAAAGACATGTACGCCAAGGTCTCTGGGTCTGTT
AATATGATCAACCTCACCCGGGGCCTCTTCTATGGGCTCTCCAGACAGGTAACC
>Medaka_MRPS5
ATGGCGGCGTCCGTAAGGGTGTGCAGTGCCCTCCGTATCACAATCGGAGGTGTCGCCTCA

ATCCGCTCTCAGGCTGCGGTGGGCGCTGTTCACTTCTCCAATTTGGTGTGTAGAGCCTCA
TCATCCTCTTTTTCCCAACGACAGTCTTCGATTCCACTCAACCCACCAACATGGCAGCAG
ACCAGACATGGCAGCTTCTTTAACAAGTTGACGGCAGAGGAGCTGTGGAGAGGCGTTCTG
GCAGAGTCCGGAGCCGGGGCCAGGAAGGGCAGAGGGAAGCGGACAAAACGGAAATTAAGG
AGAGACCTGAATCGAGGACAGATCATTGGAGAAGGTCGTGGGGGGTTTCTGTGGCCCGGC
CTGAATGCTCCGGTGTTGAAGGACGGTTCTCTGCAGAGCATGAGCAGAAGAGGCGAGACT
GAACAGCAGGAGGTCCAAGCTGAACTGGTGCGTCAAAGGGATGAGTGGGAAAAGAAGAGG
AAGATGAAGGTGAAGAGGGAGAGAGGGTGGACCGGGAACTCCTGGGGGGGCACCAGTTTG
GGTCCCCCTGATCCTGGACCTAATGGAGAAACCTACGAAGACTTTGATTCACGGGTTATT
GAGGTGAAGAGTGTTTTTAACATGACGGCTAAAGAAGGCAGAAAAAGGTCCATCAGCTGT
TTGGTCGCCGTTGGAAACGGCAATGGAGCTGCAGGTTTTGCGCTGGGCAAAGCAGCAGAT
AGAAACACAGCTCTGAGGAAGGCTAAGAACCGTGCCATCCACTACCTGTACTATATAGAG
CGATACAACAACCACACAATTTATCATGACATTGAGTCCAAGTTCAAGAGGACCACGCTC
CGCATGAAGAAACAAAACGAGGGTTACGGTCTGCACTGCCACAGAGCTGTCATCACCCTC
TGCAAGCTGATCGGCATCAAGGATTTGTACTGCAAAGTAGAAGGATCGGTCAACCTCCTC
AACATCACCAGGGCTCTGTTCACTGGATTAGCCCAGCAGGAAACTCATCAGGTGCTGGCT
GACAAGAAGAAGCTCCATGTGGTGGAGTTTCAGTCAAATCAAGGCCCGCTGCCCATTGTG
GTGGCGAGCCCCAAAGATGAGGTTCGCTCCACCCCCGAGTGTGAGGATGAAATCCCCAAC
ACCAAGCTGCACTGGGGCGATGTCCGCGTAGCGCAGGGGATTAAACGCTCGGTCTGGACC
GGCGTCAAGCGCACAGTCTGG
>Mouse_MRPS5
ATGGCGGCGGCCGTACGCGCTGCAGGCTGTCTCCCTGCATTGTGTAGCTTGCAGGCAGGT
CATTTCCTGTCTAGGCAACTCTCTTTAAACGCCTTTCCAGTAGCAGCCACTTCCTTTTTG
GCAGTGAAGACAGCTCTTAGCCACGGCTCATTGTCATCCAGGGAAACAAGGCGCAACCAC
TGTCTGACCAGCTTGAGCCATGTGCTACAGACACAGTGCTGCGTTTCCTCTCCCGGCAAC
TGGACGGGCCAGCAGTGCCGGCCCTACAGCTTCTTCACCAAACTGACTGCAGAAGAGCTC
TGGAAAGGCGCATTAGCAGAGACTGGAGCTGGAGCAAGAAAAGGCAGAGGCAAAAGAACA
AAGAAAAAGAAGAGGAAGGATTTGAACAGGGGCCAGATCATTGGTGAAGGGCGCTCTGGC
TTCCTGTGGCCTGGTTTGAACGTTCCTCTGATAAAAAGTGGGGTCGTCCAGAACATCGGC
CAGAGAAGCAAAGAGGAGCAGCAGAAGGTGGAGGCCACCATGGTCGAGCAGCGAGAGGAG
TGGGACCGGAAGAGGAAGATCAAAGTTAAAAGGGAGCGCGGCTGGAGTGGGAACACGTGG
GGGGGTGTCAGTATTGGCCCCCCAGACCCGGGACCCAATGGAGAAACATATGAAGACTTT
GATACCAGGATTCTTGAGGTAAGAAATGTCTTCAATATGACAGCAAAAGAGGGAAGAAAG
AAGTCGGTCCGAGTCCTGGTTGCTGTTGGGAATGGAAATGGAGCTGCAGGTTTTGCTATT
GGGAAAGCTGCTGACCGGGGAGACGCTTTCAGAAAGGCAAAGAACCGAGCGATTCATTAT
TTGCATTACATAGAACGATACGAAGGACATACAATATTCCATGACATTTCCCTAAGATTC
AAAAGGACACAGATCCGGATGAAGAAACAACCCAGAGGTTACGGCCTCCGCTGCCACAGG
GCCATTATCACCATCTGCCGCCTCATCGGCATCAAGGACATGTATGCGAGGGTCACCGGG
TCCATGAACATGCTCAACCTCACCCGGGGCCTCTTCCATGGGCTTGCCCGCCAGGAAACC
CACCAACATCTGGCGGATAAGAAGGGTCTCCACGTGGTGGAATTCCGGGAGGAATGTGGG
CCTCTGCCTATCGTGGTGGCCTCCCCACATGGGGCCTTGAGTAAGGAGCCGGAGCCGGAA
CCTGAGGTTCCTGATACCAAGCTGGACTGGCAGGACGTGAAGGCCATGCAGGGACTGAAG
CGCTCTGTGTGGTTTAATTTAAAGAGGCCTGCCACC
>Naked_mole_rat_MRPS5

ATGGCGGTTTCCGTGCGCGCTGTTCGCTGCCTGCCTGCTCTGTGCGGAGTCCCGGCGGGC
CATTTCTGGTCCAGGCAGCTTTCTGTAAGTGCCTTTCCAACAACTTCTGTTTTGGCATTA
AAGGCTACGACTCTCAGCAATGGCACTTTGTCACCACGGGGCTCCAGGAATGACCATCAT
TTCACCAGCTTGAGCTGCGCGCTGCAGACACAGTGCTGTGTTTCTTCTTCCAGAAACCTG
ATGGGCCAGCAGTGTAGACCCTATAGTTTCTTCACTAAATTGACGGCAGATGAGCTGTGG
AAAGGCGCTTTAGCAGAGACTGGTGCTGGAGCAAGAAAAGGAAGAGGCAAAAGAACTAAG
AAAAAGAGAAGAAAAGATTTGAACAGGGGTCAGATTATTGGTGAAGGGCATTCTGGTTTC
CTGTGGCCTGGCCTGAATGCCCCTCTCATGAAAAATGGGGATGTGCAGACGATTGCCCAA
AGGAGAAAGGAGGAGCAGGAGAAGGTGGAGGCTGACATGCTCCAGCAGCGGGAAGAGTGG
GACCGGAAGAGGAAGATTAAAGTGAAGCGGGAGCGTGGGTGGAGCGGCCACACATGGGGC
GGCGTCAGCCTTGGGCCCCCTGACCCTGGGCCCAATGGAGAAACCTATGAGGATTTTGAT
ACCAGGATACTAGAGGTAAGAAATGTTTTCAATATGACAGCAAAAGACGGAAGGAAGAAA
TCGATCCGCGTCCTGGTCGTTGTGGGGAATGGGAAAGGAGTCGCAGGTTTTGCCATTGGG
AAAGCCACTGAACGGGTAGACGCTTTCAGGAAAGCAAAGAACAGAGCAGTTCACTATTTG
CATTACATAGAACGATATGAAGACCATACAATATTCCATGACATTTCATTAAGATTTAAA
AGGACACATATCAAGATGAAGAAACAACCCAGAGGTTATGGCCTCTGCTGCCACCGAGCC
ATCATCACCATCTGCCGGCTCATTGGCATCAAGGACATGTATGCCAAGGTCTCCGGGTCC
ATCAACATGCTCAACCTCACCCAAGGCCTCTTCCAGGGCCTTGCCCGCCAGGAAACCCAT
CAACACCTGGCTGATAAGAAGGGGCTGCATGTGGTGGAATTCCGGGAGGAATGTGGCCCT
CTGCCCATCGTGGTCGCCTCCCCCCAGGGGGCCTTGAGGAAGGACCCGGAGCCAGAAGAT
GAGGTTCCAGACATCAAACTGGACTGGCAGGAGGTGAAGGCTGCACAGGGCATGAAGCGC
TCGGTGTGGTCAGGTCTGAAGCGAGCCGCCACC
>Opossum_MRPS5
ATGGCGGCTGCTGTGGCTTTGCGGGCCGGCACCCGTCTTCCCATATCTTGGGCTGCTGGG
CCAGGGTATTTATGGACAAGGCAGTTTTACCCAACTACATTACCTGTGATTTCCAGTTTT
TCTTCGAGGGTCTCCCCTTGTAATGGTAGTTTTGCATTCCAGGGAGCCAGGAGTCATCAT
TTTATCAACTTGGCCCATGCCCTCCAGACTCAATGTTGTATTACTTCTCCTGGCACATTG
ATAGGACAACAGAGACACTATAGTTTCTTCACTAAATTGACGGCAGAAGAGCTATGGAAA
GGGGTCTTAGCTGAGTCTGGTGCTGGAGCCAGGAAAGGAAGAGGGAAAAGGAGCAAGAAA
AAGAGGAAAAAGGACCTCAACAGGGGCCAGATTATTGGTGAAGGACGTTCTGGTTTCCTC
TGGCCTGGTTTGAATGCCCCTGTTTTAAAACAGGGCATGATTCAGCCAGTTGGCCAAAGA
GGCAAAGAAGAACAGGAAAAGATGCAGACTGAAATTCTCCAACAGAGAGATGAATGGGAG
AAAAAGAGAAAAAGGAAGATTAAACGGGAGAGAGGTTGGACTGGAAGTTCTTGGGGGGGA
ATCAGTTTAGGACCCCCAGATCCTGGTCCTAATGGAGAAACTTATGAAGATTTTGATACC
AGAATAATTGAGGTAAGAAGTGTTTTCAATATGACGGCCAAAGAAGGAAGAAAGAAATCT
GTTCGGGTTCTGGTGGCAGTTGGAAATGGAAAAGGAGCTGCAGGTTTTGCCGTGGGAAAA
GCCCCTGACCGGATCAATGCATTTAGAAAAGCAAAGAATAAAGCGATTAATTATTTGCAT
TACATAGAAAGATATGAAGACCATACAATTTACCATGATATTTCTTTAACATTTAAGAAA
ACACATATCAAGATGAAGAAGCAGCCCAGAGGTCATGGTCTCCATTGTCATCGAGCCATT
ATTACAATTTGCAAATTAATTGGAATTAAAGATATGTATGCAAAGGTTACTGGGTCATGT
AATTTAATTAATATAACCAGAGGACTCTTTCATGGATTATCAAAACAGGAAACTCATCAG
CAGTTGGCAGATAAGAAAAGCCTTCATGTCGTTGAGTTCCGAGATGAATGTGGTCCCCTG
CCCATAGTGGTCGCCTCTCCTCAAGGAGCAATTAGAAAAGAGCCTGAGCCCGAAGATGAG
GTTCCAGATACCAAATTAGACTGGGATGAAGTTAGGATGAGTCAGGGAATGAAAAAATCA

GTTTGGGCAAACTTGAAGAGAGCAGCAGCC
>Orangutan_MRPS5
ATGGCGACCGCGGTGCGCGCTGTGGGCTGCCTCCCCGTGCTGTGTAGCGGGACGGCAGGT
CATTTATTGAGGAGGCAGCATTCCCTAAACACCTTACCAGCAGCTTCCATTTTGGCATGG
AAGAGTGTTCTCAGCAATGGTCATTTGTCATCACTGGGAACCAGAGATACCCATCCCTAC
GCCAGCTTGAGCCGTGCACTGCAGACACAATGCTGTATTTCTTCTCCCAGTCACCTGATG
AGCCAGCAGTATAGACCATATAGTTTCTTCACTAAATTGACTGCAGATGAGCTGTGGAAA
GGCGCTTTAGCAGAGACTGGTGCTGGAGCAAAAAAAGGAAGAGGCAAAAGAACTAAAAAG
AAGAAAAGAAAGGATCTGAACAGGGGTCAGATCATTGGTGAAGGGCGTTATGGTTTTCTA
TGGCCTGGGCTGAATGTCCCTGTTATGAAAAATGGAACAGTGCAGACCATTGCCCAAAGA
AGCAAGGAAGAGCAGGAGAAGGTGGAGGCAGACGTTATCCAGCAGAGAGAAGAGTGGGAA
CGAAAGAAGAAGATGAAGGTTAAACGGGAACGAGGATGGAGTGGAAACTCATGGGGAGGC
CTCAGTCTTGGCCCCCCTGACCCTGGTCCCAGTGGAGAAACATATGAGGATTTTGATACC
AGGATACTTGAGGTAAGAAATGTTTTCACTATGACTGCGAAAGAGGGAAGAAGGAAATCG
ATCCGTGTCCTGGTGGCTGTGGGGAACGGAAAAGGAGCTGCAGGTTTTGCTATTGGGAAA
GCCACTGATCGGATGGATGCTTTCAGGAAAGCAAAGAACAGAGCAGTTCACCATTTGTAT
TATATAGAACGATATGAAGACCATACAATATTCCATGATATTTCATTAAGATTTAAAAGG
ACGCATATCAAGATGAAGAAACAACCCAAAGGTTACGGCCTCCGCTGCCACAGGGCCATC
ATCACCATCTGCCGGCTCATTGGCATCAAAGACATGTATGCCAAGGTCTCTGGGTCCACT
AATATGCTCAGCCTCACCCAGGGCCTCTTCCATGGGCTCTCCAGACAGGAAACCCATCAA
CAGCTGGCTGATAAGAAGGGCCTCCATGTTGTGGAAATCCGAGAGGAATGTGGCCCTCTG
CCCATTGTGGTTGCCTCCCCCCGGGGGGCCTTGAGGAAGGATCCAGAGCCAGATGAGGTT
CCAGACATCAAACTGGACTGGGAAGACGTGAAGACTGCACAGGGAATGAAGCGCTGTGTG
TGGTCTAATTTAAAGAGAGCCGCCACG
>Western_painted_turtle_MRPS5
ATGTGGACAGCTGATGGCATCGACCTTGAGAAAGCAACTCAGTTCTCAGGCTGTCTCTCG
TTACCAGCGAATAGGAGTGGCTGTCGCTATACGAACCTGGCATGGGCTCTACAGACTCAT
TGTTACATTTCTGCTCCATGTAGCGTGACAATACAACAATGCAGGCAAGGCAGCTTTTTC
AACAAATTGACAGCTGATGAGCTGTGGAAAGGGGTTTTGGCAGAGACCGGTGCTGGAGCA
AGGAAGGGAAGAGGAAAGCGAACCAAGAAAAAGATTAGGAGGGATCTCAATAGAGGCCAG
GTCATTGGTGAAGGACGTTCTGGTTTCCTTTGGCCTGGTCTCAATACTCCTATAATGAAA
AGTGGGGTAGTCCAGACAATTGGTCAACGAGACAAAGAGGAGCAGGAGAAGGTGCAGTCT
GAAATTATCCGGAAGAGGGATGAGTGGGAGAAGAGAAGAAAAACAAAGGTGAAGAGAGAG
AGAGGCTGGACTGGAAATTCCTGGGGAGGCATCAGTCTGGGACCCCCTGAGCCAGGTCCT
AATGGAGAAACTTATGAAGACTTTGACTCCAGGGTAATTGAGGTGAAACATGTATTTAAT
ATGACAGCAAAGGAAGGCAGAAGAAGATCAGTTAGTGCCCTAGTGGTTGTTGGAAATGGA
AATGGGGCTGCAGGTTTTGCAGTGGGGAAAGCAAGTGACAGGATGACAGCTTTAAGAAAA
GCAAAGAACAGAGCAATTCACTATTTACACTATGTAGAGCGATATCAGAACTACACAATT
TACCATGATATTACAACAACTTTTAAAAGAACAACCATCCGGATGAAAAAGCAAAACAAA
GGGCATGGTCTTCATTGCCATCGAGCCATCATTACCATCTGCAAGCTGATTGGGATTACA
GACCTGTATGCCAAACTTTCTGGATCCAACAATCTACTTAACCTCACCAGAGCTCTCTTT
AAGGGTTTAGCAAAGCAGGAAACCCATCAAGAGCTAGCAGATAAGAAAAGCCTCTACGTG
GTAGAATTCCGTGAGGAGTGTGGCCCCCTGCCGACCGTAGTTGCTTCACCTAATGGAGAT
GTCAGGAAGGAACCAGAGCCTATAGATGAGGTTCCCAATACCAAGCTGGAATGGAGCGAA

GTGAGAGTAGCTCAAGGAATGAAACGTTCTTCCTGGGCACATGTCAAAAGGACAGTGTGG

>Panda_MRPS5
ATGGCGGTGGCGGTGGCGGTGGCGGTGCGCGCCGCGGGCTACCTCCCTGCGCTGTGTGGC
GTGCCGGCGGGCCACGTATGGTCCAGACAGCTTTACCGAAACACCTTTCCGACAGCTTCC
ATTTTGGCATTGAAGACTGTTCGTAGCAACGGTCCTTCGTCGTCTCTAGGAACCAGAGAC
AACCATCATTTCACCAGCTTGACCCATGCGCTACAGACACAATGCTGTATTTCATCTCCC
AGTAATTCGATGGGTCAACAGTATAGATCCTATAGTTTCTTCACTAAATTGACAGCAGAT
GAGCTTTGGAAAGGTGCTTTGGCAGAGACTGGAGCAGGAGCAAGAAAAGGAAGGGGCAAA
AGAACTAAGAAAAAGAGAAAAAAAGATTTGAACAGGGGTCAGATCATTGGTGAAGGGCGT
CGTGGCTTCCTCTGGCCTGGTCTGAATGTCCCTCTTATGAGAGAGGGAGCTGTGCAGACC
ATTGCGCAAAGAAGCAAGGAAGATCAGGAGAAGGTAGAGGCCGATCTGGTCCAGCAGAGG
GAAGAGTGGGACCGCAAGAGGAAGAGGAAGGTTAAACGGGAGCGAGGATGGAGTGGAAAC
ACATGGGGAGGCGTCAGTCTTGGCCCTCCTGACCCTGGTCCCAGTGGAGAAACATATGAT
GATTTTGATACCAGGGTACTTGAGGTAAGAAATGTTTTCAACATGACAGCAAAAGAGGGA
AGAAAGAAATCAGTCCGTGTCCTGGTCGCGGTGGGGAACGGCAGAGGAGCTGCAGGTTTT
GCCGTTGGGAAAGCCGCTGATCGGATGGATGCTTTCAGGAAAGCGAAGAACAGAGCAGTT
CACTATTTACATTATATAGAACGATATGAAGACCATACGATATTCCACGATATTTCTTTA
AGATTTAAAAAGACGCATATCAAGATGAAGAAACAACCCAGAGGCTATGGCCTCCGCTGC
CATCGGGCCATCATCACCATCTGCCGGCTCATTGGCATTAAAGACATGTATGCCAAGGTC
TCTGGGTCCCTCAACATGCTCAACCTCACACGGGGCCTCTTCCATGGGCTCTCCCGCCAG
GAAACCCATCAACAACTGGCCGATAAGAAGAGTCTCCATGTTATAGAATTCCGGGAGGAG
TGTGGCCCTCTGCCCATCGTGGTGGCCTCCCCGCAGGGAGCCCTGAGAAAGGATCCTGAG
CCAGAAGATGAAGTTCCAGACATCAAACTGGACTGGGAAGAAGTGAGGGCCGCACAGGGA
ATGAAGCGCTCAGTGTGGTCGAATTTAAAGAGAGCCGCTACC
>Rock_pigeon_MRPS5
ATGACCCCTGCTGGGGAGCCTATTCCAGTGATCAGTCACCCTCTCAGCGAGGAGCTTCTT
CCTGGGTTTGCTTCTGTGCCTGTGAATGGGAGTTGCTGCTCATATTCTAGCCTGGCATGG
GCTTTGCAGACACGGTGTTTCATCTCCGCACCCTGGAATGTGACAGTTGAGCAACGCAGA
CAAAGCAGTTTCTTCAATACGTTGACAGCTGATGAGCTTTGGAAAGGAGCTTTGGCAGAG
ACTGGTGTGGGAGTAAAGAAAGGAAGAGGAAAGAGAAGGAAGAAAAAGCTAAGGAAGAAT
CTCAATAAAGGCCAGGAGATTGGTGAAGGCCGATCTGGTTTCCTCTGGCCTGGTCTTAAT
GCTCCTGTGATACAAAATGGGAAAGTCCAGGCAGTTACTCAACGAAAAAAAGAAGAACGA
GAGAGAATTCAGTCGGAAATTGTTCAGCAGAGAGATACATGGGAGAAGAAAAGAAAAATA
AAAATTAAGAGAGAGAGGGGGGATGGAGTGGAAAGTGTTGGGGAGGTGTCCTTTTGGATCCT
CCTGACCCAGGTCCTAATGGAGAAACTTATGAAGAATTTGAAACAAGAGTCATTGAGGTA
AAAAATGTGTTTTGTATGAAGGCAAAGGAAGGCAGAAAAAAATCAGTACGTGCCTTAGTG
GCTGTTGGAAATGGTAAAGGGGCTGCAGGTTTTGCAATGGGGAAAGCAGGCGACAGGATG
AATGCTTTACGGAAAGCGAAGAATAGAGCAATACACTGCTTACATTTTATAGAGCTGTAT
CAGAACCACACAATTTATCATGACATTACAGTGAAATTTAAAAGCACAAACATCCGCATG
AAGAAGCAAAACAAAGGGTATGGTCTTCATTGCCACCGAGCTATCATCACCATCTGCAAA
CTAATTGGCATTAAAGACATGTATGCCAAGGTTTCTGGATCCAAAAACTTGATTAACATT
ACCAGAGCTCTCTTTAAAGGCTTAACACAACAGGAGACTCACCAGCAGTTAGCAAACCAG
AAGAGTCTCTACGTAGTGGAGTTCCGAGAGGAGCAGGGCCCTCTGCCTATTGTCGTGGCA

CTGCCTGAGGGACCTATTCGCAAGGATCCTGAGCCTGAAGATGAGGTTCCAGACACAAAG
TTGGAGTGGAGTGAGGTGAAAGAAGCTCAGGGAATGAAGAAATCTCCCTGGGTGAATGTC
AGACGGACAATATGG
>Pig_MRPS5
ATGGCGGCGGCGGTGCGCGCCGCGGGGTTCTTCCCCGGGCTGTTTGGCGCGTCGGCAGGT
AATTTATGGTCCAGGCAGCTTTACTTAAACACCTTTCCAGCGGCTTCCATTTTGACATCG
AAGACTTTTCCCAGCAATGGCCCTTTGTCACCTCCAGGAACCAGAGACAACCATCATTTT
ACCAGCTTGGCTTGTGCACTTCAGACACAATGCAGTATTTCTTCTCCCAGTAACTGGATG
GGCCAACAGTACAGATCCTATAGTTTCTTCACTAAATTGACAGCAGATGAGCTATGGAAA
GGTGCTTTAGCAGAGAGTGGTGCTGGAGCAAGAAAAGGAAGAGGCAAAAGAACTAAGAAA
AAGAGAAGAAAGGATTTGAACAGGGGTCAGATCATTGGTGAAGGACGTCATGGCTTCCTC
TGGCCTGGTCTGAATATCCCTCTTATGAGAAATGGTGCTGTACAGACCATTGCCCAGAGG
AGCAAGGAAGACCAGGAAAAGGTGGAGGCCGATATGGTCCAGCAGAGAGAAGAGTGGGAC
CGGAGGAGGAAGATGAAGGTTAAACGGGAGCGAGGATGGAGTGGAAACACATGGGGAGGC
GTCAGTCTTGGTCCCCCGGATCCTGGTCCCAATGGAGAAACATATGATGATTTTGATACC
AGGATACTTGAGGTGAGAAATGTTTTCAATATGACAGCAAAAGAGGGAAGAAAGAGATCA
GTCCGTGTCCTGGTCGCTGTGGGGAATGGCAAAGGAGCTGCAGGTTTTGCCATTGGGAAA
GCCACTGAACGGGCAGATGCTTTCAGAAAAGCAAAGAACAGAGCAGTTCACTATCTGCAT
TACATAGAGCGATATGAAGACCATACAATATACCATGATATTTCTTTAAAATTTAAAAGG
ACGCATATCAAGATGAAGAAACAACCCAGAGGCTACGGTCTCCACTGCCACCGGGCCATC
ATGACCATCTGCCGGCTCATTGGCATCAAAGACTTGTACGCCAAGGTGTCTGGCTCTGTC
AACATGCTCAACCTCACCCGGGGCCTCTTCCTTGGGCTCTCCCGCCAGGAAACCCATCAA
CAACTGGCTGATAAGAAGAGTCTCCATGTTGTGGAATTCCGGGAGGAATGTGGCCCTCTG
CCCATCGTGGTTGCCTCCCCCCAGGGAGCCTTGAGAAAGGATCCAGAGCCAGAAGATGAG
GTTCCAGACATCACACTGGACTGGGAAGATGTGAAGGCCGCTCAGGGAATGAAGCGCTCT
GTGTGGTCGGGTTTAAAGAGAGCCGCCACC
>Platypus_MRPS5
ATGACAGCGAAAGAGGGAAGAAAGAAATCCATCCGTGTCCTAGTGGTTGCCGGGAATGGA
CAAGGAGCAGCGGGTTTTGCTGTGGGGAAAGCCAGCGACCGGATGAGCGCTTTTAGAAAA
GCCAAGAATAAAGCAATTCACTACTTGCACTACATAGAAAGATACCAAGACCATACAATC
TACCAGGATATTTCATTAACTTTCAAAAGGACAACAATCAAGATGAAGAAACAACCCCGC
GGCTATGGTCTGCGTTGCCACCGGGCCATCATCACAATCTGCAGGCTCATCGGAATCCGG
GACATGTATGCCAAGGTCACCGGGTCGGTCAACTTACTGAACCTCACCAGAGGAGTCTTT
AAGGGCTTATCCAGCCAGGAGACGCACCAGCAACTGGCCGACAAGAAAGGCCTCCACGTG
GTGGAGTTCCGGGAGGAGTCTGGCCCCCTGCCCCTGCTCGTCGCCTCCCCACTAGGGGTG
CTCAGCAAGGAGCCCGAGCCCGAGGACCAGATTCCTGACACCAAGCTGGACTGGAGCGAT
GTCAAGGCCAGCCAAGGCTTCAAACGCTCCATCTGGTCCAATTTGAAGAGGCCGGCATCC

>Rabbit_MRPS5
ATGGCGGCGGCGGTGCTAGCCGCGCGCTGCGTCCCTGGACTGTGTGGGGTGCAGGCGGGT
CTTCTGTGGTCCAGGCAGCTTTCCCTAAACACCTTTCCAGCAGCTTCTGTTTTGGCGTGG
AAGACTGCTCTCAGCAATGGCCCGGTGTCGTCGCGGGGAAGCAGAGACAGCCGTGGAGAC
GCCAGCCTGAGCCACGCGCTGCAGACACAGTGCTGGATTTCTTCTCCCAGTGCCTGGACG
GGCCAGCAGCACAGAGCCTACAGCTTCTTCACCAAATTGACAGCAGACGAGCTATGGAAA

GGTGCTTTAGCAGAGACTGGCGCTGGAGCAAGAAAAGGAAGAGGCAAAAGAACAAAGAGA
AAGAGAAGAAAGGATTTGAACAGGGGTCAGATCATTGGTGAAGGACGTTCTGGTTTCCTG
TGGCCTGGTCTGAATGTGCCTCTTATGAAAAATGGAGCAGTGCAGACCATTACCCAGAGA
AGCAAGGAAGAGCAGGCGAAGGTGGAGGCCGACATGATCCAGCAGAGAGAAGAGTGGGAC
CGGAAGAGGAAGACGAAGGTTAAACGGGAGCGAGGATGGAGCGGCAACACGTGGGGAGGC
GTCAGTCTTGGGCCCCCTGACCCTGGTCCCAACGGAGAAACGTATGAAGATTTTGATACC
CGGGTACTGGAGGTAAGGAACGTTTTTAATATGACGGCAAAGGAAGGAAGGAGGAAGTCC
GTCCGGGTGCTGGTCGCTGTGGGGAATGGAAAGGGGGCTGCAGGTTTTGCTGTTGGGAAA
GCCACTGAACGAATAGATGCTTTCAGAAAAGCTAAAAACAAAGCAATTCACTATTTGCAT
TACATAGAACGATATGAAGACCATACAATATTCCATGACATTTCTTTAAGATTTAAAAGG
ACACATATCAGGATGAAGAAACAACCCAGAGGGTACGGCCTCCGCTGCCACAGGGCCATC
ATCACCATCTGCCGGCTCATTGGCATTAAAGACATGTACGCCAAGGTCTCCGGGTCCGTC
AACATGCTCAACCTCACCCGGGGCCTCTTCCAGGGGCTCTCCCGCCAGGAAACCCATCAA
CAGCTGGCTGATAAGAAGGGTCTGCACGTTGTGGAGTTCCGGGAAGAATGTGGCCCTCTG
CCCATTGTGGTCGCCTCCCCACAGGGGGCCTTGAGAAAGGATCCAGAGCCCGAGGATGAG
GTTCCAGACATCAAACTGGACTGGCAAGAAGTGAAGGCCGCACAGGGACTGAAGCGCTCT
GTGTGGGCGAATTTAAAGCGAGCTGCCACC
>Tetraodon_MRPS5
ATGAAAGTGAAGAGAGAAAGAGGCTGGACCGGTCACTCGTGGGGCGGCATCAGCCTGGGC
CCCCCTGATCCTGGGCCAAATGGAGAGACATACGAGGACTTTGATTCACGTATCATTGAG
GTGAAGAGTGTGTTCAACATGACAGCCAAGGAAGGTCGCAAGAGGTCCATCAGCTGTCTG
GTCGCTGTTGGGAACGGCAACGGAGCCGCAGGCTTTGCTCTGGGTAAAGCAGCAGACAGA
AACACAGCTCTGAGGAAGGCTAAAAACAGAGCCATCCATTATTTGTATTACATAGAGCGA
TACAACGACCACACC
>Tilapia_MRPS5
ATGGCGGCATCCATCGGGGTGTGCTGTGCCCTCCGCATCACACTTGGAGGTGCAACATCC
CTCCGTGCTGTAGGAGGAGTTGTACAGGCATCTCTCTTGGCAAGTCGAGCCTCAGCTGCG
TCTCTGCAAAGACACTCTGCTCTTTCACCGCTCCCTCCAACAACATGGCAGCAAACAAGA
CACGGCAGCTTCTTCAACAAGTTAACGGCTGAAGAGCTATGGAGAGGTGTGTTGGCTGAG
TCCGGTGCTGGAGCAAGGAAGGGTCGAGGGAAGCGTACCAAACGCAAACTAAGGAGAGAT
CTGAACCGAGGACAGACCATTGGAGAGGGTCGTGGCGGTTTTCTGTGGCCTGGACTCAAC
AGTCCAGTGTTGAAGGATGGCGCTTTGCAGAGCATGAGTCGAAGAGGCGAAGCTGAGCAG
CAGGAGGTTCAAGCTGAACTAGTGCGTCAGAGGGACGAGTGGGAGAGAAGGAGGAAGATG
AAGGTGAAGAGGGAGAGAGGTTGGACAGGAAACTCCTGGGGGGGAATCAGCCTGGGTCCT
CCTGACCCTGGACCCAATGGAGAAACCTACGGGGATTTCGATTCACGTGTCATCGAGGTG
AAGAGTGTGTTCAACATGACAGCCAAAGAAGGCAGAAAGAGGTCCATCAGCTGTTTGGTC
GCAGTGGGAAATGGCAACGGAGCCGCAGGCTTTGCTTTGGGGAAAGCAGCAGACAGGAAC
ACGGCTCTGAGGAAGGCCAAGAACAGAGCCATCCACTACTTGTACTATATAGAACGATAC
AATGACCACACCATTTACCATGACATTGATTCCAAGTTTAAGAGGACGACACTCCGCATG
AAAAAACAAAACAAAGGTCACGGTCTGCACTGCCACAGGGCGGTCATCACTCTTTGCAAG
CTGATCGGCATAAAAGACATGTATTGCAAAGTAGAGGGATCAGTCAATCTCCTGAACATC
ACCCGGGCCCTCTTCACCGGATTAGCCAGTCAGGAAACCCACCAGACTCTGGCTGACAAG
AAGCAACTCCATGTCGTAGAGTTTGAGTCGCAGCGTGGCCTTCTGCCCATGGTGGTGGCG
AGTCCTAAAGACGGCGCACGACCCAACCCAGAGTCAGAGGATGAGATTCCCAACACCCGG

CTGCACTGGGATGACGTACGAGCTGCACAGGGGTTGAAACGCTCGATCTGGGCAGGTGTC
AAACGCACCATCTGG
>Turkey_MRPS5
ATGGAGTGGAAAAGCTTTCCTATTTCAGCGACAGCTGAAGAGCTATGGAAAGGAGCTTTG
GCGGAGACTGGTGTGGGAGTAAAGAAAGGAAGAGGAAAGAGAAGGAAGAAAAAGCTGAGA
AAGAATCTCAACAGAGGCCAGGAGATTGGAGAAGGACGTTCCGGTTTCCTCTGGCCAGGT
CTTAATGCTCCTCTGATACAAAGTGGGAAAGTACAGGCACTTAGTCAACGGAAAAAAGAA
GAACGAGACAGAATTCAGTCTGAGATTATTCAGCAGAGAGATACATGGGAGAAGAAAAGA
AAAATAAAAATGAAGAGAGAGGGAGGATGGAGTGGAAAGTGTTGGGGAGGTGTCCTCATG
GATCCTCCTGATCCAGGTCCTAATGGAGAAACTTACGAGGATTTTGAAACAAGAGTCATT
GAGGTGAAAAATGTGTTTTGTATGAAGGCGAAGGAAGGCAGAAAAAAATCAATACGTGCT
TTAGTGGCTATTGGAAATGGTAAAGGGGCTGCAGGTTTTGCACTGGGGAAAGCAGGTGAC
AGGATGACTGCTTTACGGAAAGCAAAGAACAAAGCAATACGCAGCTTGCATTTTATAGAG
CTGTATCAGAACCACACAATTTATCATGACATTACAGTGAAATTTAAATGCACAAACATC
CGCATGAAGAAGCAAAACAAAGGGTATGGTCTTCATTGCCACCGAGCTATTATTACCATC
TGCAAACTAATTGGCATTAAAGACATGTATGCCAAGGTTACTGGATCTAAAAACTTGATT
AATATTACCAGAGCTCTCTTTAAAGGATTGACACAACAGGAGACTCACCAGCAGTTAGCA
AACCAGAAAAACCTCTACGTGGTGGAGTTCCGGGAGGAGCAAGGCCCACTGCCCATCGTC
GTGGCACTGCCCGAGGGGACTGTCCGTGAGGATCCTGAGCCTGAGGATGAGGTTCCAGAC
ACTAAACTGGAGTGGTGTGAGGTGAAAGAAGCTCAGGGAATGAAGAAATCACCCTGGGCG
AATGTTAGGCGGACAGTATGG
>Worm_MRPS5
ATGGCATCACTTTTGCCATTTGTCCAGACCCGTAGCAATACGGTGAACTTCTTTATGAGA
AGATCTGGTCCAGAACTGTGGAAAACATTAACCTCCGTTTCAAAATCTGGTCAGAAGAAA
GGACGTCGTAACACAAGACAACCAGTTAGACCTCTCAATCGATTTTATCGAATTGGATCC
AGTCCAATGAAAATCGAATTTGCTGGCCTGAATGCTCCAATCAGAATGAGAGAGACAGAA
AATCAAAATTTGATGTCAATTGCTGAACAGACGGAAGATGAAATTCGAGATTCAATGGGT
GGAACCAAGAAAATTCTCGAAGAACGAGATACTGGAAAAAAGAAGCGCAACCGTGAAAAA
CTGCATCCGATGGAGCGTGGATTCTCTGGAACACAACTTGTCGGACAGAAACTTGGTGCA
CCACCACCACTCGATGGTGTGAATTTCGATGATTTTGAAACTTATTGTTTAGAGGTTAAG
CGTACTTCTAACATGACAAATGTATTTGGAAGAGTTCACACAATGTCAGCACTAGTTGTT
ACTGGAAATGGACGAGGACTTGCTGGATATGCTGTTGGAAAAGCACCAATTCATCGTACC
ACTACAGCTATCATTAATGGAATGGGAATGGCATCCAGAAAGCTATTCCACGTTGAACTT
CATGAAGGACGTACGATTTATCAGGACTTTTATGCTGAATGCCGTAACACTCGAGTATTT
GCACAACGTCGCCCACGTGGATTCGGTCTTACATGCCATCCCAGACTGATCAAAATTTGT
GAGGCTATCGGTATCAAGGATATTTACGTGAAAGTTGAAGGATCCACGAAAAACTATTTG
GCATTGACTCATGCATTTGTCACTGGACTCCTCAACCAAGAGACTCATCAACAACTGGCC
GAACGAAAAGGTCTTCATGTCGTTGAAATGTCCCCATCTCGTCATTTCTTACCCCAAATT
GTGGCGTCACCGATTTCCACTGAACTCAAGACCGAGGAAACTTTAGAAGCACTTGATCGC
CTGAATCTTGATGACTTCTATGGTGAAGGACGGTATCCATTAAGAAAGCCGAAATCTCTT
CCATTCTTTTCCAACCTCGAAGGTCATTTGGATGCCAGATGGAGGAAACATCCTTTCAGA
AATCAGGAATCGACTATGATTCGCCTGATTGCCGATAACATGGTTCCACGTTGGACTCGT
GATGCTCGTGCTGCGTGGGCGGATCAAAGAAATGAACGAATGACAACGGGTGTCGAACCA
ATGCCACTCGGTATCGGACTCTCGCATGTGGTTCCCAAAAAGGATGAC

>Yeast_MRPS5
ATGTTCAAGAGGCAATTATCGACTAGTGTTCGTTATCTACAGCATTATGATGAGTCGCTA
TTATCAAGGTACTATCCTGAAAGTCTTTTAAAATCCATAAAACTTGCTCAACAAACGATA
CCAGAAGATACTAAATTCAGAGTATCTCGTAACGTGGAATTCGCACCACCATATTTGGAT
GATTTTACCAAAATACATCCTTTTTGGGATTACAAACCAGGCATGCCCCATCTCCACGCT
CAAGAGGAGAATAATAACTTCAGCATCTTTAGATGGGACCAGGTACAACAACCATTACCA
GGCGAAGGTAACATTCTGCCTCCAGGAGTCAGCTTACCAAACGATGGTGGTCGGAAATCG
AAAAGCGCCGATGTAGCTGCAGGGCTACACAAGCAAACCGGTGTAGATCCGGATTATATC
ACTAGAAAGTTGACTATGAAGCCGCTGGTGATGAAAAGAGTGTCAAATCAGACTGGGAAG
GGTAAAATTGCGTCTTTCTATGCCTTGGTTGTCGTTGGTGACAAAAACGGTATGGTAGGT
TTGGGAGAAGGTAAATCTCGTGAAGAAATGTCCAAAGCGATCTTTAAAGCTCATTGGGAT
GCGGTAAGGAACCTGAAGGAAATACCTAGGTATGAAAACAGAACTATTTATGGTGATATA
GATTTTAGGTATCATGGTGTGAAACTACATTTAAGAAGTGCAAAACCAGGGTTTGGATTA
CGTGTAAACCATGTAATCTTTGAGATTTGTGAATGTGCAGGTATCAAAGATCTGAGTGGG
AAAGTATATAAATCCAGAAACGATATGAATATAGCTAAAGGTACCATCGAGGCTTTCACG
AAAGCTCAAAAGACATTGGATGAGGTTGCCCTGGGTAGAGGCAAAAAGCTTGTTGATGTC
AGGAAAGTTTACTATTCAAGC
>Zebra_finch_MRPS5
ATGGCGGGCGCTGGGGCCTGCCGGGGGCTTTCCCTCAGCTGTGCCCGCCTGCCTGCCGGC
TTTGCTTCTGTGCCAGTGAAAGGGAGCTGCAGTCCCTACTCCAGCCTGGCATGGGCTTTC
CAGACACAGTGCTCCCTCTCTGCCCCCTGGACTGGGACAGTGGAGCAGTGCAGGAAAAGC
AGTTTCTTCAATACCTTGACAGCTGATCAGCTATGGAAAGGAGCTTTGGCAGAGACTGGT
GTAGGAGTAAAGAAAGGAAGAGGAAAGAAAAAGAAGAAGAAGCTAAGGAAGAATCTCAAT
AGAGGCCAGGAGATTGGTGAAGGACGTTCTGGTTTCCTCTGGCCTGGTCTTAATGCTCCT
GTGTTGCAAACTGGGAGAGTGCAAGAAGTTGCCCAGCGGAAAAAAGAAGAACGAGAGAGA
ATTCAGTCTGAAATCATTCAGCAGAGAGATACATTTGAGAAGAAAAAGAAAATAAAAATT
AAGAGAGAGGGAGGATGG
>Zebrafish_MRPS5
ATGGCGGCGGTCACAAGGCTGTGCTGTGTCCTACGGTTGTCATTTGAAGGTGTTGGAGCA
CTGCGAACTCTAGGGGGCGCTGTTTACTTCTCACACTTGGCAAGACCCAGTCCTACTCAT
TATGCTCCAGTCACTGTGGCCCTACAGCAGATCAGACATGGAAGCTTCTTCAACAAATTG
ACAGCTGATGAGCTCTGGCGAGGTGTCTCAGCTGAGACTGGAGCTGGTGCCAGAAAAGGA
AGAGGAAAGAGAGCCAAACGAAAAGTGAAGAAAGATCTCAACAAAGGCCAGAGTCTTGGA
GAAGGTCGTGCTGGATACCTGTGGCCCGGTCTAAACACTCCAATATTTAAGGATGGCTCC
ATTCAGAAACCCATGCAGCGTGGAGAGGCAGAGCAGAAGGAGATGACAGCAGCCCTGGAA
CGCCAGAGAGATGAATGGGAAAAGCGCAGAAAGGCCAAGATAAAGAAGGAAAGGGGATGG
ACTGGAGGCTCCTGGGGTGGGGTCAGCCTGGGCCCGTTGGATCCCGGGCCCAATGGAGAA
ACCTATGAAGATTTTGATTCGCGTGTTATTGAGCTCAAGAGTGTGTTTACTGTTACACCC
AAAGAGAGCAGGAAGAGATCCATCAGTGCTTTGGTGGCAGTGGGCAATGGAAATGGAGTC
GCAGGGTTTGCACTGGGGAAAGCAGCAGACCGGACCGCCGCTCTCAGAAAAGCAAAGAAC
AGAGCCATGAATTATCTGTACTACATCGAGAGATACAATGACTATACAATATATCATGAC
ATTGAGTCCAAGTATAAAAAGACCACACTTCGAATGAAGAAACAAAACAAAGGTTATGGT
CTGCGCTGTCATCGGGCTGTGATCACTCTCTGCAAGCTGATTGGGATAAAGGATATGTAT
GCAAAAGTAGATGGCTCGGTAAACCTCCTTAACATCACCAGGGCTCTTTTCCAAGGCCTG

GCCAGTCAGGAGACTCATCAGACTCTAGCTGATAAAAAGCAGCTGAATGTGGTTGAGTTC
AGGGCTGAACAGGGCCCTCTGCCCATCGTGGTGGCACGACCGCACCTCGGCGCCCGTCAG
GATCCTGAAGGGGAGGATGAAGTGCCCAACACGCGTCTGCACTGGGCTGATGTGAAGGCC
TTGCAGGGTGTTAAGAAATCCATATGGGCAGGGGTTAAGAGGACAATCTCT
>Great_tit_MRPS5
ATGGCGGCGGCGGTGGTGGCGGCCGGCCGGGCGTGTGCCCGCGGCGCGCTGCGAGCGGCC
TGGAGAGGGTTTGTTTCTGTGCCAGTGAAAGGGAGCTGCAGTTCCTACTCCAGCCTGGCA
TGGGCATTCCAGACACGGTGCTCCCTCTCTGCCCCCTGGACTGGGAGACTCGAGCAGTGC
AGGAAGAGCAGTTTCTTCAATACAGTGACTGCTGATCAGCTATGGAAAGGAGCTTTGGCA
GAGACTGGCGTGGGAGTAAAGAAAGGAAGAGGAAAGAAAAGGAAGAAAAAGCTAAGGAAG
AATCTGAATAGAGGCCAGGAGATTGGTGAAGGACGTTCTGGTTTCCTCTGGCCTGGTCTT
AATGCTCCTCTGTTGCAAACTGGGAGAGTGCAGGAAGTTGCCCAACGAAAAAAAGAAGAA
CGGGAGAGAATTCAGACTGAAATTATTCAGCAGAGAGATACTTGGGAGAAGAAAAAGAAA
ATAAAAATTAAGAGAGAGGGAGGATGGAGTGGAAAGTGTTGGGGAGGTATCATTCTGGAT
CCTCCTGACCCTGGTCCTAATGGAGAAACTTATGAAGATTTTGAAGCAAGAGTCATCGAG
GTGAGAAATGTGTTTTGTATGAAGGCAAAGGAAGGCCGGAAAAAATCAGTACGTGCCTTA
GTGGCTGTTGGGAATGGTAAAGGGGCTGCAGGTTTTGCAATAGGGAAGGCAGGTGACAGG
ACGAATGCTTTAAGGAAAGCAAAGAATAAAGCAATAAGCTCCTTACACTTCGTAGAGCTG
TATCAGAACCACACAATTTACCACGACATTTCTGTGAAATTTAAAAGGACAAAAATCCGC
ATGAAGAAACAAAACAAAGGGTATGGGCTGCATTGCCACCGAGCCATTATCACCATCTGC
AGGCTAATTGGCATTAAGGACATGTATGCCAAGGTCACTGGATCCAAAAACTTGATTAAC
ATCACCAGAGCTCTCTTTAGAGGCTTGACCTTACAGGAGACTCACCAGCAGCTGGCAAAC
AAGAAGAGCCTCTACGTGGTGGAGTTCCGGGAGGAGCAGGGCCCTCTGCCCATCGTGGTG
GCCCTGCCCGAGGGGACTGTCCGTGAGGAGCCTGAGCCTGAGGATGAGGTTCCAAACACA
AAGCTGGAGTGGAGGGAGGTGAAAGAAGCCCAGGGAATGTTGAAATCTCCCTGGGCAAGC
GTCAGACGGGCGGCGTGC
>African_ostrich_MRPS5
ATGTTGACAGCTGATGAGCTATGGAAAGGAGCTTTGGCAGAGACTGGTGTGGGAGTAAAG
AAAGGAAGAGGAAAGAGAAGGAAGAAGAAGCTAAGGAAGAATCTCAATAGAGGCCAGGAG
ATCGGTGAAGGACGTTCCGGTTTCCTCTGGCCTGGTCTTAATACTCCTTTAGTACAAAGT
GGGAGAGTCCAGGCAGTTACCCAACGAAAAAAAGAAGAACGAGAGCGAATTCAGTCTGAA
ATTGTTCAGCAGAGAGATACATGGGAGAAAAAAAGGAAAATAAAAGTGAAGAGAGAGGGA
GGTTGGAGTGGAAAGTGTTGGGGAGGTGTCATTCTGGACCCCCCTGACCCAGGTCCAAAT
GGAGAGACTTATGAAGATTTTGAAACAAGAGTCATTGAGGTGAAAAATGTGTTTTGTATG
AAAGCGAAGGAAGGCAGAAAAAAATCAATTCGTGCCGTAGTTGCTATTGGAAATGGAAAA
GGGGCTGCAGGTTTTGCAGTGGGAAAAGCAGGTGACAGGATGAATGCTTTACGGAAAGCA
AAGAACAAAGCGATACGCTGTTTACATTTTATCGAGCTGTATCAAAACCACACAATTTAC
CATGACATTAGTGTGAAATTTAAAAGGACAAAAATCCGCATGAAGAAGCAAAACAAAGGC
TATGGCCTTCGTTGCCACCGAGCTATTATCACCATCTGCAAACTAATTGGCATTAAAGAC
ATGTACGCCAAGGTTTCTGGTTCCAAAAACTTGATTAACATCACCAGAGCTCTCTTTAAA
GGCTTAACAGAACAGGAGACTCACCAGCAATTAGCAAACCAGAAGAGCCTCTACGTTGTA
GAGTTTCGGGAGGAGCAGGGCCCGCTGCCCATTGTTGTGGCATCGCCCGAGGGAGCCATC
CGCGAAGATCCTGAGCCTGAAGATGAGGTTCCAGACACCAAGCTGGAGTGGAGTGAGGTG
AAAGAAGCTCAAGGAATGAAGAAATCTCCCTGGGCAAATGTCAGACGGACAGCATGG

>common_starling_TP53
TTTGTTTCTGGGGCGGCTCCCCGAGCCCTCCCAGTGACTCCCAGTCCCTCCCAGTACTCT
CCAGTGCTGAACAAGCTCTATTGCCGCCTGGCCAAGCCGTGCCCGGTGCAGGTGCGGGTG
GGGGTCCCGCCGCCCCCCGGGGCCCTGGTCCGGGCCGTGGCCGTCTACAAAAAATCCGAG
CACGTGGCCGAGGTGGTCCGGAGGTGTCCCCACCACGAGCGCTGCGGGGGAGGGGCAGAC
GTGGTTTTTAGGTTTTTTTTTCCACCCCAGGTGGGCTCAGAGTGCACCACGGTCCTGTAC
AACTTCATGTGCAACAGCTCCTGCATGGGGGGCATGAACCGCAGGCCCATCCTCACCATC
CTCACCCTGGAGGGGCCTGGG
>central_bearded_dragon_TP53
ATGGAGCGTGATTTGGAGTCCGACCTGGACCGCACCCTGAGTCCGCTGCTGAGCCAGGAG
ACATTTCAGGACCTGTGGGGTTCCTTGGAGGCGACAGACTCAACAGGTTTCCTGGACCCG
GGCAACATGCCAAGACCGATGGGCTTCAGCGACCTGGGTTTTTGTCCTTTGTACGTGCAG
GCGGAGAACATCCCGGAGGTGGGCGGAGACCGTGCTGACCTCCCCCAGCCAGGTCACCCG
GCCAGCGACCCTCTCCCGGATCCAGCAGCCCCCGCGGCCGCTCCCGCCGTCCCCTGCACC
GAGGACTACGTCGGCGAGCACGGGTTTGAGCTGGCTTTCGAGACGTCCGGCATGGCGAAA
TCCGTCACGTGCACATACTCACCAGATCTGAATAAGCTGTTCTGCCAACTTGCTAAGACA
TGCCCGGTCCATATTAAAGTGTCGGCCTCGCCGCCGGCCGACGCCGTCATCCGCACCATG
GCCGTGTACAAGAAGTCTGAGCACATTGCCGATGTGGTGAAGCGCTGCCCGCACCACGAG
CGATCCCCCGACTTCAAGGATGGTACAGCGCCTGCTGAACATCTGATCCGAGTGGAAGCC
AATCCGCAGGCTAAGTACATCTCGGACACCGCGAGCAAGCGCCACAGCGTCACCGTTCCT
TACGAGCAGCCTCAGCTGGGAACGGACAGCACCACCATCTTGTACAACTTCATGTGCAAC
AGCTCCTGCATGGGAGGCATGAACCGGCGCCCCATCTTGACCATCGTCACTTTGGAGACC
CCTCTGGGTATGATTCTCGGACGCCGCTCCTTTGAAGTCCGGGTTTGCGCCTGCCCCGGA
CGGGACCGCCGGACCGAAGAGGAAAACCTGAAGAAAACAACAAATTCCAGAAGGGAATCC
AAAAAGGCTGTTGCACAGAGCAGCAGTTCTGAAAGCACCAAGCGGTCCTCCGCAGAAACG
TCTGGCAATAACAACGAGAGCGGGCCGTACACCCTCCAGGTTCGCAACTTGAAGCACTAC
AAGATTTTGGAGTACCTGCTCAAAGCCATGGAGTCATTTGACTCGAAGCAGCAGCAGCAA
CCGCCACAGCCGCAGGGAGAATCGGAACAGCAGGGGGAGCCGGAGAGCCGGTCTAGTCTT
CTGAAGGCCCGGAAACAAGACGGTGGTCAAAGTTCGTCCAGCGAAGGGCCGTCCAGGGTG
AAGAAGCGAAAGGTGAAAGAGGAGACGCTGGATTCCGAC
>Gecko_TP53
ATGGAGCAATCTGCAGATTCAGATGTGGACCCACTGCTGGTCACTGATTCGTTTCGTGCG
CATTGGAGCCAGATTAATGAAGATAGCTTGTTGAGTTCAAACTTGCTGCTGGATTTCAAC
TTAGACTTCCAGCTCCCTGAGGAAAATCCCGAGCTGCCCTTTGCCCAGGAAGGGGGCAGC
AGAGGGCCCGGGGAAATCAGAGAGGATGGCCCCGCCCTGGATATGACGGCTTGCACCGCC
CCCTCCACCGAAGACTACACCGGGAACCAGTGTTACCAGCTGGCATTTGATCAACTTTCC
GAGACGCCCTTCGTACAGGAAGGGGGCAGCAGCGGGCCGGGGGAAGGCAGAGAGTGGGGC
CCCGCCCCGTATACAACGGTTTGCACCACCTCCTCCACCGTCCCCTCCACCGAAGACTAC
GCCGGGGGACACTGTTTCGAGCTGGTGTTTGAGCAGTCGGGAACGGCCAAATCCGTCACC
TGCACATATTCAAAACAATTGAACAAGCTGTTCTGTCAGCTCGGAAAGACTTGCCCCGTT
CTGGTGAAGCTGTCTACCTCGCCACCCCCCGGCTCAGTCATCCGGGCCACGGCCGTCTAC
AAGAAGTCGGAGCATGTGGCCGAGGTGGTGAAGCGCTGCCCGCACCACGAGCGTGCGCCG
GAGTACAGCGAAGACGGAGTCCCTGCTGAGCACCTGATCCGCGTCGAAGGGAACCAAAAC
GCCCAGTACTTCTCTGACAGGATCACCAAGCGCCACAGCGTTGTGGTTCCTTATGAGATG

CCTCAGGTGGGATCGGAGTGCAGCACCGTCCTCTACAGCTACATGTGCAACAGTTCCTGC
ATGGGTGGCATGAACCGGCGCCCCATCCTCACCATCGTTACGCTGGAATCTCAAGAAGGG
CAGCTGCTCGGCCGCCGCTGCTTCGAAGTACGGGTCTGCGCCTGCCCTGGCCGGGACCTC
AAGTCGGAGGAAGAGAACTTCCGTAAAGCTGCTCGTGGCGAACGGTCCAAAAAGGGAGGG
GCTCCGGCGGAGAACGGACCCGAGAAGAAGCACAGTCCCGGGACCTCCAACAACAGAGAA
GACGTTTACACCCTCAAGATCAGGGGACGGGATCGGTATCTCATGTTGAAGAAGATCAAC
GATGCGCTGGAGGTCACAGATGTGCTGGAGGTCACAGACGCACAGGAGGTGCTCAAACTG
AGGAATGGCCCACAGAAGACCCGCAAGCGGAGTGCGGCGCCCCCGCCGGGCAGTGGGAAG
AAGCTGCTGCTGAAGGACGAGGCGGGGGGCTCCAGT
>Chinese_alligator_TP53
ATGGAGTCGATCATGGACCCCGACCTGGACCCCCCCCTGAGCCAGCCCTTCCTCGACTTC
TGGAATGTTCTGGACAACAATGTGAGATCCATCCCCAAGGAGCAGGCAGAGCTGTGGGAT
CCACAGGACCTGGTCCTAGGGCTGCCAGATTTGGGTGACTTGCCCCTGCTGGAGGAGCTG
GAAGGGGCCCCAGTGGCGGGGCTGGGAAGGGAGGCCCCGCCCCCGGGTGCCCTGCCCACA
TCCTCTATCGTGCCTTCCACTGAGGACTACCCCGGCGCCCATGGCTTTGAGGTGGCCTTC
CAGCCCTCGGGCACCGCCAAGTCCGTCACCTGCACATACTCGCCGGTGCTGAACAAGCTG
TTCTGCCAGCTGGCACAGTCATGCCCGGTGCAGGTGCGGGTGGCACAGGCTCCGCCCCCT
GGTGCCATGATCCGCGCTGGTGCTGTCTACAAGAAGGCTGAGCACGTAGCTGAGGTCGTG
CGGCGCTGCCCCCACCATGAGCGCAGTGCCGAGCACAGTGACGGGGTGGCTCCAGCCCAG
CACCTGATCCGAGTGGAGGGGAACCCACAGGCCCAGTACTGCCACGATGAGACCACCAAG
CGCCACAGCGTGACTGTGCCCTACACCCCCCCAGAGGTGGGCTCGGACTCCACGACTGTC
CTCTACAACTTCATGTGCAACAGCTCATGCATGGGGGGCATGAACCGGCGCCCAATCCTG
GCTATCCTCACCCTGGAGACGAAGAGTGGGCAGCTTCTTGGCCGGCGCTGCTTTGAAGTC
CGGATCTGTGCCTGCCCTGGCCGCGATCGCAAGACAGAAGAGGAGAATTTGCGCAACAAG
GCGGCAACCACTGGGGGTGGGGCCAAGCGGGCTCTCAAGGTCCCTACAGATGACCTCCCA
AACCCAAAGAAGCGAGTCCCGAATCCCAGCACTGAGATTTTCACCCTCCAGATCCGGGGC
CATGAGCGCTATGAGATGTTCAAGAAGCTCAACGAGGGGCTGGAGGCCTTGGACGGGCAG
GAGGCCCGCGCCGAGGACCCAGGCATCCGGTCCCCCAAACCGCTGCTGAAGGCACGCCGT
GCCAAGGGGCTGGCCCTGGTTTCATGCAAGAAGCTGCTGGTGAAGGACGAGAGCCAGGAC
TCAGAC
>African_clawed_frog_TP53
ATGGAACCTTCCTCTGAGACCGGCATGGACCCCCCCTCAGCCAGGAGACATTCGAGGAT
CTGTGGAGTCTGTTGCCTGACCCCCTGCAGACTGTCACGTGTCGGCTGGACAACCTATCA
GAGTTTCCAGACTATCCCCTGGCAGCAGACATGACGGTCCTACAGGAGGGGCTTATGGGT
AATGCTGTTCCCACCGTCACTTCATGTGCTGTCCCCTCAACTGACGATTATGCTGGGAAG
TATGGGCTCCAACTGGACTTCCAACAGAACGGCACCGCAAAGTCTGTTACCTGCACGTAT
TCCCCAGAGCTCAACAAACTCTTCTGCCAGTTGGCCAAGACTTGCCCTTTGCTGGTGCGT
GTGGAGAGCCCCCCGCCTCGCGGCTCCATTCTCCGGGCTACGGCCGTCTACAAGAAATCT
GAGCATGTGGCCGAGGTGGTGAAGAGATGCCCCCACCATGAGCGCAGTGTGGAGCCAGGG
GAGGATGCTGCGCCTCCCAGTCACCTGATGCGAGTGGAGGGAAATCTCCAGGCTTATTAT
ATGGAGGATGTAAATAGCGGGCGCCATAGTGTCTGTGTTCCCTATGAGGGGCCTCAGGTT
GGAACGGAATGTACAACAGTTCTGTACAATTATATGTGTAACAGCTCCTGTATGGGAGGG
ATGAACCGCCGGCCCATTCTCACCATCATCACCCTCGAGACCCCACAGGGGCTACTTCTT
GGCCGCCGCTGTTTTGAGGTTCGAGTGTGTGCCTGCCCAGGGAGGGATCGTCGCACAGAG

GAAGACAATTACACAAAAAAGAGGGGCCTGAAACCCAGCGGCAAGAGAGAACTTGCTCAC
CCACCATCCAGTGAACCACCACTTCCTAAGAAGCGTCTTGTTGTTGTTGATGATGATGAA
GAAATCTTCACTTTGCGGATTAAGGGGCGCAGCCGCTATGAGATGATTAAGAAACTGAAT
GACGCACTTGAATTACAAGAAAGCCTCGATCAGCAGAAAGTGACCATTAAGTGCCGCAAG
TGCCGGGATGAAATCAAACCCAAGAAAGGAAAGAAGCTGCTGGTTAAAGATGAACAGCCC
GACTCGGAA
>Tibetan_frog_TP53
GTCATGTGACTCCCTCACCCCCATGTTTCTGTTCTCTCTCAGACTTTGCCCCTCGAAGTC
ACCTGATCCGCGTGGAAGGGAATTTAATGGCTCAATACTCGGAGGACGGGAACGGGCGTC
ACAGCGTGTGCGTGCCTTACGAGGAGCCGCAGGTGGGGTCCCAGTGTACGGCCGTCCTCC
TGAACTACATGTGTAACAGTTCCTGCATGGGGGGTATGAACCGCAGACCCATCATGACCA
TCATCACCCTGGAGTCCAAGGAGGGCATCCTCCTGGGCCGGCGCTGCTTCGAGGTCCGCG
TGTGCGCCTGTCCTGGTCGGGACCGCCGGACTGAGGAGGAGAACTTCACCAAGAAAAAGG
AGCTGAAGGGCAGCGGGAAGAGAGGTGAGACTCTGCGGAGGTGGGACCAG
>southern_platyfish_TP53
ATGGAGGAGGCGGATCTCACACTGCCTTTGAGTCAGGACACCTTCCATGACTTATGGAAC
AATGTGTTTTTGTCGACTGAGAATGAGTCTTTGCCCCCCCCAGAAGGATTGTTGAGTCAA
AATATGGATTTTTGGGAGGATCCGGAAACTATGCAGGAAACTAAAAATGTGCCCACTGCA
CCAACTGTCCCAGCGATTTCTAATTATGCTGGGGAGCATGGGTTCAATCTCGAATTTAAT
GACTCGGGCACAGCTAAAAGTGTCACCTCCACATACTCAGTTAAACTTGGGAAGCTGTTC
TGCCAGCTGGCAAAAACGACTCCAATTGGAGTTTTGGTTAAGGAGGAGCCTCCGCAGGGT
GCCGTCATTAGAGCAACTGCAGTATACAAGAAGACTGAACATGTGGGAGAAGTCGTTAAG
AGGTGCCCACACCACCAAAGTGAAGACTTGTCTGACAACAAGAGTCACCTGATCAGAGTG
GAGGGCAGCCAGCTGGCGCAATACTTTGAGGACCCAAACACCAGGAGGCACAGCGTGACC
GTGCCCTACGAACGCCCACAGCTGGGATCCGAGATGACCACAATCCTTCTGAGCTTTATG
TGCAACAGCTCCTGCATGGGAGGCATGAACCGGAGGCCCATCCTCACCATCCTGACCCTG
GAGACCACAGAGGGGGAGGTGTTGGGCAGGCGGTGCTTCGAGGTCCGTGTCTGTGCCTGT
CCAGGCAGGGACCGCAAGACAGAGGAAGGAAACCTGGAAAAAAGTGGGACTAAGCAGACG
AAGAAAAGAAAGAGCGCTCCTGCTCCAGATACCTCCACCGCAAAAAAGTCCAAGTCTGCC
TCTAGTGGAGAGGATGAGGACAAGGAGATTTACACTCTCTCTATCCGGGGCCGTAATCGT
TATCTGTGGTTCAAGAGCCTCAACGACGGTCTGGAACTGATGGATAAAACGGGGCCCAAG
ATAAAACAGGAGATTCCTGCGCCCTCCAGTGGGAAGAGGCTGCTGAAGGGGGGAAGCGAC
AGCGAC
>spotted_gar_TP53
ATGGATCCAGACGCTGTCCCCTCGACCTCGAAGTTCTCCCCCGATTTGGACTCTTACAGT
TTTCCGGAGGACTCTGGTGAAGCTCCTGAGCTCAGCTGCAGCAGCCTTGCTGCCTGGCCT
GCCTGTCGCCTTCTGAGCTGCTGCGAGTGCGAGGACAGCATGGCGGATCCCGAGCCTGAG
GGGCTGCCCCTGAGCCAGGAGTCCTTCCAGGAGCTCTGGAACATGGTGGCTGTGCCTCCT
GAAAATGCTCAGTGGCCAGACCTGCAGAGCATGCTGTATACCAATGAATTGGTTCCCAAC
GCCATCGACAGCCTGTACGAGGAACTGGAGCCTCACGTCTCCATGCCCCCCCAGCCCATG
GTCTCGAACCCGGATGGCTTGGCGCCCTCTTCCTCCACTGTGCCATCCACGTCCGACTAC
CCCGGAGAGCTGGGGTTCCAGCTGCGCTTCCAGCAGTCTGGCACTGCCAAGTCGGTCACC
TGCACTTACTCCCCAGATCTGAACAAGCTGTACTGCCAGCTGGCTAAGACCTGCCCTGTC
CAGATCTTTGTGTCTAACCCACCACCCATGGGTGCCCTGATCCGAGCCATGGCTGTCTAC

AAGAAGTCGGAGCACGTAGCCGACGTGGTCCGCCGCTGCCCGCATCACGAGAGGGCCCCC
GAAAACAACGAAGGTCCTGCACCCCCAGGGCACCTGATCCGCGTGGAGGGTAGCCAGATG
GCACAGTACATGGAAGACGGTAACACCCGCCGGCAGAGTGTGCTGGTGCCCTACGAGTCT
CCTCAGCTGGGGTCAGAGTGCACCACTGTGCTCTACAACTTCATGTGCAACAGCAGCTGT
ATGGGTGGCATGAACCGGCGCCCCATCCTCACTATCATCACCTTGGAGACCAAGGAGGGT
CAGCTCCTGGGGCGCCGGTGTTTTGAGGTACGCGTGTGCGCCTGCCCCGGCCGAGACCGC
AAGACGGAGGAGGAGAACTTCCACAAGCAGCAGGAGAAAGGGAGCAGCAAGTCGACGGGG
AGTAACAAGCGCAATATTAAAGAAGTGAACCAGTCCACTCCCCCGTCTGACAACAACAAG
AAGACCAAGTCCAGTTCGAGCGCTGAGGAGGAGATATTCACCCTGCAGGTCCGGGGCCGG
GAGCGATTCGAGATGCTGAAGAAGATCAACGAGAGCCTGGAGCTGAAGGATCTGGTCCCA
GTGGCGGACCTGGAGAAATACCGCCAGAAGCTGCACACCAGGAGCAGCAGCCGGAGGGAG
AAGGACAAGGAAAAAGAGCCCAAGAAAGGGAAGAAGCTGTTGGTGAAGGAGGAGAAGACT
GACTCCGAC
>guppy_TP53
ATGGACCCCGACTTCAACCTCCCTTTGAGTCAGGACACCTTCCATGATTTATGGAACACT
GTTGTTTTGTCGACTGAGAATGAGTCTTTGGCTACAGGTGACGGATTGCTGGATCTAAAT
ATACATTTTTGGGAGAATGGAGAACTTCCACAGCAGGAAACTAAAAATGTGCCTGCAGCC
CCAATGGTCCCTGCAATTTCCAATTACGCGGGAGAATTAGATTTTGCCCTCCGATTTAGC
GAGTCTGGCACCGCAAAAAGCGTGACTTCCACGTTTTCAGAAAAATTGACCAAACTATTC
TGCCAGCTGGCTAAGACCACTCCGATTGGGATTTTGGTTAAGGAGGAGCCTCCGCAGGGC
GCCGTCATCAGAGCAACGGCTGTGTATAAAAAGACTGAGCATGTGGCAGAAGTCGTCAAG
AGGTGCCCACACCACCAAAGTGAAGACGCGTCCGACAACAAGAGCCATCTGATCCGAGTG
GAGGGCAGCCAGCTGGCCCAGTACTTTGAGGATCCGAACACCAAAAGGCAGAGCGTGACC
GTACCTTACGAACGCCCACAGCGGGGATCTGAGATGACGACCATCCTGCTGAGTTTTATG
TGCAACAGCTCCTGTATGGGAGGCATGAACCGGAGGCCCATCCTTGCCATCCTGACCCTG
GAGACCACAGAGGGGGGAGGTTTTGGGCAGGCGGTGCTTCGAGGTCCGTGTCTGTGCCTGT
CCAGGCAGGGACCGCAAGACAGAGGAAGAAAACCTGGAGAAAAACGGCACTAAGCAGACA
AAGAAAAGAAAGAGCGCTCCTGCTCCAGACACATCCACTGCTAAAAAGTCCAAGTCTGCC
TCTAGTGGAGAAGATGAGGACAAGGAGTTGTACACTCTGCAGATACGGGGCCGTGACAGA
TTCAATATGTTTAAAAACCTGAATGATGGTCTTGAACTGCTGGACAAAACAGGGTCCAAG
AAAAAACAGGAGCTTCCTGCGCCCTCCAGCGGGAAGAGGCTGCTGAAGGGGGCGAGCGAC
AGCGAC
>American_alligator_TP53
ATGGAGTCGATCATGGACCCCGACCTGGACCCCCCCCTGAGCCAGCCCTTCCTCGACTTC
TGGAATGTTCTGGACAACAATGTGAGATCCATCCCCAAGGAGCAGGCAGAGCTGTGGGAT
CCACAGGACCTGGTCCTAGGGCTGCCAGATTTGGGTGACTTGCCCCTGCTGGAGGAGCTG
GAAGGGGCCCCAGTGGCGGGGCTGGGAAGGGAGGCCCCACCCCCGGACGCCCTGCCCACA
TCCTCTATCGTGCCTTCCACTGAGGACTACCCCGGCGCCCATGGCTTTGAGGTGGCCTTC
CAGCCCTCAGGCACCGCCAAGTCCGTCACCTGCACATACTCGCCGGTGCTGAACAAGCTG
TTCTGCCAGCTGGCACGGTCATGCCCGGTGCAGGTGCGGGTGACACAGGCTCCGCCCCCT
GGTGCTCTGATCCGCGCTGGTGCTGTCTACAAGAAGGCTGAGCACGTAGCTGAGGTCGTG
CGGCGCTGCCCCCACCATGAGCGCAGTGCCGAGCACAGTGACGGGGTGGCTCCAGCCCAG
CACCTGATCCGAGTGGAGGGAAACCCACAGGCGCAGTACTGCCACGACGAGACCACCAAG
CGTCACAGCGTGACTGTGCCCTACACCCCCCCAGAGGTGGGCTCGGACTCCACAACTGTC

CTCTACAACTTCATGTGCAACAGCTCATGCATGGGGGGCATGAACCGGCGCCCAATCCTG
GCTATCCTCACCCTGGAGACGAAGAGTGGGCAGCTTCTTGGCCGGTGCTGCTTTGAAGTC
CGGATCTGTGCCTGCCCTGGCCGCGATCGCAGGACAGAAGAGGAGAATTTGCGCAACAAG
GCGGCAACCGCTGGGGGCGGGGCCAAGCGGGGTGAGTGCTCTGGATGCCTGGGTTCTCTG
GAGGTGGATAATGAGAGTAGCGGGGCTGGGAGACCAGGTGCCTGGGTTCTCTTCAGCTCT
ATACCCCCCTCCCCCCAGCTCTCAAGGTCCCTGCAGATGACCTCCCAAACCCAAAGAAGC
GAGTCCCGAATTCCAGCACTGAGATTTTCACCCTCCAGATCCGGGGCCGTGAGCGCTATG
AGATGTTCAAGAAGCTCAACGAGGGGCTGGAGGCCTTGGATGGGCAGGAGGCCCGCGCCG
AGGACCCAGGCATCCGGTCCCCCAAACCGCTGC
>Armadillo_TP53
ATGGAGGAGCCCCCGTCAGATCTGAGCATCGAGGCGCCTCTGAGTCAGGAGACATTTTCA
GACTTATGGAAACTACTTCCCCAGAACAACGTTTTGTCCCCATCGCTGTCTACCATGGAA
GATCTGCTCCTCTCAGATGATGTTACCAGCTGGTTCGAAGGCCAAGATGAGCCTCTCAGA
ACACCAGAGGCTCCTGCGCCCACTACCCCTGCACCAGCCGCCCCCACGCCAGCCACCCCC
TGGCCCCTGTCATCCTCTGTCCCTTCCCAAAAGGAATACCCTGGCGACTATAATTTCCGT
CTAGGCTTCCTGCGTTCTGGAACAGCCAAGTCTGTCACCTGCACGTACTCACCTATCCTC
AACAAGCTGTTTTGCCAGCTGGCAAAGACCTGCCCAGTGCAGCTTTGGGTCAACTCACCA
CCCCCTCCTGGGGCCCGCGTCCGGGCCATGGCTGTCTACAAGAAGTCCGAGCACATGACG
GAGGTCGTGAGGCGCTGCCCCCACCACGAGCGCTGCTCAGAACATGGGGATGGTCTGGCC
CCCCCTCAGCACCTCATCCGGGTGGAAGGAAACCTGCATGCCGAGTATTTGGACGACATC
AACACCTTCCGACATAGTGTGGTGGTGCCCTACGAGATGCCCGAGGTCGGCGCCGACTGC
ACCACCATCCACTACAACTACATGTGTAACAGCTCCTGCATGGGAGGCATGAATCGGCGG
CCCATCCTCACCATCATCACCCTGGAAGACTCCAATGGTAATCTGCTGGGACGGCGCAGC
TTTGAGGTGCGTGTTTGTGCCTGTCCTGGGAGGGACCGAAGAAATGAGGAAGACAATTTC
CGCAAGAAGGGGGAGCCTTGCCCTGAGCCACCGGTTGGGACCACTAAGCGAACACTGCCC
ACGACCACCAGCTCGTCTCCTCCGCCAAAGAAGAAGCCAATGGATGGGGAATATTTCACC
CTTCAGATCAGAGGGCGTGAACGCTTTGAGATGTTCCGAGAGCTAAATGAAGCTTTGGAG
CTGAAGGATGCCCAGACTGGGAAGGAGCCAGAGGGGAGCAGGGCTCACACCAGCTATCTG
AAGTCTAAGAAGGGGCAATCTACCTCCCGCCATAAAAAATTACTGTTTAAGAGAGAGGCG
CCTGACTCCGAC
>Chicken_TP53
ATGGCGGAGGAGATGGAACCATTGCTGGAACCCACTGAGGTCTTCATGGACCTCTGGAGC
ATGCTCCCCTATAGCATGCAACAGCTGCCCCTCCCTGAGGATCACAGCAACTGGCAGGAG
CTGAGCCCCCTGGAACCCAGCGACCCCCCCCCACCACCGCCACCACCACCTCTGCCATTG
GCCGCCGCCGCCCCCCCCCCATTAAACCCCCCCACCCCCCCCCGCGCTGCCCCCTCCCCG
GTGGTCCCATCCACGGAGGATTATGGGGGGGACTTCGACTTCCGGGTGGGGTTCGTGGAG
GCGGGCACAGCCAAATCGGTCACCTGCACTTACTCCCCGGTGCTGAATAAGGTCTATTGC
CGCCTGGCCAAGCCGTGCCCGGTGCAGGTGAGGGTGGGGGTGGCGCCCCCCCCCGGTTCC
TCCCTCCGCGCCGTGGCCGTCTATAAGAAATCAGAGCACGTGGCCGAAGTGGTGCGGCGC
TGCCCCCACCACGAGCGCTGCGGGGGGGGCACCGACGGCCTGGCCCCCGCACAGCACCTC
ATCCGGGTGGAGGGGAACCCCCAGGCGCGTTACCACGACGACGAGACCACCAAACGGCAC
AGCGTCGTCGTCCCCTATGAGCCCCCCGAGGTGGGCTCTGACTGTACCACGGTGCTGTAC
AACTTCATGTGCAACAGTTCCTGCATGGGGGGGATGAACCGCCGCCCCATCCTCACCATC
CTTACACTGGAGGGGCCGGGGGGGCAGCTGTTGGGGCGGCGCTGCTTCGAGGTGCGCGTG

TGCGCATGTCCGGGGAGGGACCGCAAGATCGAGGAGGAGAACTTCCGCAAGAGGGGCGGG
GCCGGGGGCGTGGCTAAGCGAGCCATGTCGCCCCCAACCGAAGCCCCCGAGCCCCCCAAG
AAGCGCGTGCTGAACCCCGACAATGAGATATTCTACCTGCAGGTGCGCGGGCGCCGCCGC
TATGAGATGCTGAAGGAGATCAATGAGGCGCTGCAGCTCGCCGAGGGGGGGTCCGCACCG
CGGCCTTCCAAAGGCCGCCGTGTGAAGGTGGAGGGACCCCAACCCAGCTGCGGGAAGAAA
CTGCTGCAAAAAGGCTCGGAC
>Chinese_softshell_turtle_TP53
ATGGAGCCGATCTTGGACACCGGACTGGATCTTCCCCTGAGTCAGGAGAGCTTCTCCGAT
TTATGGAAAACACTTAGCCCCCTGGCAGAAGAGTACCTGGTGAAGCCTGCGGAACCGGTG
AGCCAGGAGCTGTTTACCCTACCTGACATGAGCCTGGGCCTGTCTGGCTCTGCAGACTCC
TCCCTCCTACTTCCGCAGGCTGGGCACAGCTATGAAAGTTGGGATCTCCCTTGCCTGGCC
CTGGAGCCACCGCCCACCTCCTCCACCGTCCCCTCCACTGAAGATTACGCCGGGGAGCAC
GACTTTCAACTGGCCTTCCAGCAGTCAGGGACTGCCAAGTCCGTCACATGCACCTATTCC
CCAGATCTGAACAAGCTCTTCTGTCAGCTGGCGAAGACCTGCCCGGTGCAGATCAAAGTG
TCCAGCCAGCCCCCGCCCGGCTCTGTCATCCGGGCCACGGCTGTCTACAAAAAATCAGAG
CATGTGGCTGAAGTGGTGAGACGCTGCCCGCACCATGAGCGCTCCGCAGACTACAGTGAC
GGGGTCGCCCCAGCCCAGCACCTGATCCGCATAGAAGGGAACCATCAGGCCTATTACCGC
GATGACGAGAACACCAAGCGCCAGAGCGTTACGGTGCCCTATGAGACACCCCAGGTGGGG
TCTGACTGCATCACTGTGCTTTACAACTTCATGTGTAACAGCTCTTGCATGGGGGGAATG
AACCGCCGCCCCATCCTGGCCATCATCACTCTGGAGAGCAAGAACGGGCAGCTCCTGGGG
CGCCGATGCTTTGAGGTTCGAGTGTGCGCCTGCCCCGGGCGGGACCGCAGGACGGAGGAG
GAGAATCACCGCAAAAAATTGTCTTGCCGGGCCCTGAGCGGAAGCGTGGTCCTCAAAGAG
AGCAAAGCCAAGAGGACTCTCCAAGCCACCATGGAGGCATCTGAGAACCCTAAGAAGCGG
ACCGTGTCCTCCGACAAAGAGGTCTTCTGCCTCGAAGTTCATGGGCGCGAAAACTACGAG
ATGCTGAAGAAAATCAACAATGCCCTGGAGGTGGCGGCTGCGAGGCCGCAGGGGGAGCTG
GAGACTCAGAGGAACCCCAAGGCGTGGTTGAAGACCCGGAAGGAGCGGGGGGATGGGCCG
CTGCCGCAAAGTGGGAAGAAACTGCTGGTGAAGGAAGAGGATTCAGAA
>Cod_TP53
CCCCCCACGCACGCGGCCCCCACCGTGCCCACCACCTCAGATTATGCCGGAGAGTACGGC
TTTCACATCCGCTTCCAGAACTCTGGAACGGCGAAATCTGTTACCTCCACATACTCGGTC
TCGCTGAACAAGCTGTTCTGCCAGCTCGCCAAGACGTGCCCGGTGGAGGTCCTGGTGGAC
GGGGCCCCTCCCCCGGGGGCCATCCTGAGGGCCACGGCCGTCTACAAAAAGACGGAGCAT
GTGGCGGACGTGGTGCGGCGGTGCCCCCACCACCTGAAGGAGGACGCGGTGGAACATCGC
AGTCACCTGATCCGCGTGGAGGGGGAGCCAGCGGGCCCAGTACATGGAAGACCCCAACACC
AAGCGGCAGAGTGTGACCCTGCCCTACGAGGCCCCCCAGCTGGGCTCTGGGCACACGACG
CTGCTGCTGAACTTCATGTGCAACAGCTCCTGCATGGGAGGGATGAACCGGAGAGCCATC
CTGACCATCCTGACCCTGGAGTCCTCTGAGGGGCATGTTCTCGGGCGGCGTTGCTTCGAG
GTGCGCGTCTGCGCCTGTCCCGGGCGCGACCGCAAGACGGAGGAGGGCAGCCCCCCCACT
CCGGCCACCGCGGCCCCGCCCAAAAGGGTCGTGTCCGCCTCCAGCGCTGAAGAGGAGGAT
AAGGAGGTGTTTGTGCTTCAG
>Cow_TP53
ATGGAAGAATCACAGGCAGAACTCAATGTGGAGCCCCCTCTGAGTCAGGAGACATTTTCC
GACTTGTGGAACCTACTTCCTGAAAATAACCTTCTGTCCTCCGAGCTCTCCGCACCCGTG
GATGACCTGCTCCCGTACACAGATGTTGCCACCTGGCTGGATGAATGTCCGAATGAAGCG

CCCCAAATGCCAGAGCCTTCTGCCCCAGCTGCCCCGCCACCAGCCACCCCAGCACCAGCC
ACCTCCTGGCCCCTGTCGTCCTTTGTCCCCTCCCAGAAGACCTACCCTGGCAATTACGGT
TTCCGTCTAGGGTTCCTGCAATCCGGAACAGCCAAGTCCGTGACCTGCACGTATTCCCCT
TCCCTTAACAAGCTGTTCTGCCAGCTGGCCAAGACCTGCCCAGTGCAGCTGTGGGTCGAC
TCGCCACCCCCGCCCGGCACCCGCGTCCGCGCCATGGCCATCTACAAGAAGTTGGAGCAC
ATGACGGAGGTTGTGAGGCGTTGTCCCCACCATGAGCGCTCCTCTGACTATAGCGATGGT
CTGGCCCCTCCTCAGCACCTTATCCGGGTGGAAGGGAATTTACGCGCGGAGTATTTGGAC
GACCGGAACACCTTTAGACACAGTGTGGTGGTGCCCTATGAGTCCCCCGAGATCGACTCT
GAGTGCACCACCATCCACTACAATTTCATGTGTAACAGCTCCTGCATGGGGGGCATGAAC
CGGCGGCCCATCCTCACCATCATCACACTGGAAGACTCTTGTGGTAACCTGCTGGGACGG
AACAGCTTTGAGGTGCGTGTTTGTGCCTGTCCTGGGAGAGACCGCCGCACTGAGGAAGAA
AATCTTCGCAAGAAGGGGCAGTCTTGCCCCGAGCCACCCCCTAGGAGCACTAAGCGAGCA
CTGCCTACCAACACCAGCTCCTCTCCACAGCCAAAGAAGAAACCACTGGATGGAGAGTAC
TTCACTCTTCAGATCCGTGGGTTTAAACGCTATGAGATGTTCCGAGAGCTGAATGATGCC
TTGGAGCTGAAGGATGCTCTGGATGGAAGGGAACCAGGGGAAAGCAGGGCTCACTCTAGC
CACCTGAAGTCTAAGAAGAGGCCTTCTCCCTCCTGCCATAAAAAACCAATGCTCAAGAGA
GAGGGGCCTGACTCAGAC
>Dog_TP53
ATGGAGGAGTCGCAGTCAGAGCTCAATATCGACCCCCCTCTGAGCCAGGAGACATTTTCA
GAATTGTGGAACCTGCTTCCTGAAAACAATGTTCTGTCTTCGGAGCTGTGCCCAGCAGTG
GATGAGCTGCTGCTCCCAGAGAGCGTCGTGAACTGGCTAGACGAAGACTCAGATGATGCT
CCCAGGATGCCAGCCACTTCTGCCCCCACAGCCCCTGGACCGGCCCCCTCGTGGCCCCTA
TCATCCTCTGTCCCTTCCCCGAAGACCTACCCTGGCACCTATGGGTTCCGTTTGGGGTTC
CTGCATTCCGGGACAGCCAAGTCTGTTACTTGGACGTACTCCCCTCTCCTCAACAAGTTG
TTTTGCCAGCTGGCGAAGACCTGCCCCGTGCAGCTGTGGGTCAGCTCCCCACCCCCACCC
AATACCTGCGTCCGCGCTATGGCCATCTATAAGAAGTCGGAGTTCGTGACCGAGGTTGTG
CGGCGCTGCCCCCACCATGAACGCTGCTCTGACAGTAGTGACGGTCTTGCCCCTCCTCAG
CATCTCATCCGAGTGGAAGGAAATTTGCGGGCCAAGTACCTGGACGACAGAAACACTTTT
CGACACAGTGTGGTGGTGCCTTATGAGCCACCCGAGGTTGGCTCTGACTATACCACCATC
CACTACAACTACATGTGTAACAGTTCCTGCATGGGAGGCATGAACCGGCGGCCCATCCTC
ACTATCATCACCCTGGAAGACTCCAGTGGAAACGTGCTGGGACGCAACAGCTTTGAGGTA
CGCGTTTGTGCCTGTCCCGGGAGAGACCGCCGGACTGAGGAGGAGAATTTCCACAAGAAG
GGGGAGCCTTGTCCTGAGCCACCCCCCGGGAGTACCAAGCGAGCACTGCCTCCCAGCACC
AGCTCCTCTCCCCCGCAAAAGAAGAAGCCACTAGATGGAGAATATTTCACCCTTCAGATC
CGTGGGCGTGAACGCTATGAGATGTTCAGGAATCTGAATGAAGCCTTGGAGCTGAAGGAT
GCCCAGAGTGGAAAGGAGCCAGGGGGAAGCAGGGCTCACTCCAGCCACCTGAAGGCAAAG
AAGGGGCAATCTACCTCTCGCCATAAAAAACTGATGTTCAAGAGAGAAGGGCTTGACTCA
GAC
>Dolphin_TP53
ATGGAGGAGTTGCAGGCAGAACTCGGCGTGGAGCCCCCTCTGAGTCAGGAGACGTTTTCA
GACTTGTGGAAACTGCTTCCTGAAAACAACCTTCTGTCCTCTGAGCTCTCCCCAGCCGTG
GATGACCTGCTGCTGTCCCCAGAAGACGTCGCAAACTGGCTGGATGAACGTCCAGATGAA
GCCCCCCAAATGCCGGAGCCCCCTGCGCCAGCTGCCCCCACCCCAGCCGCCCCAGCACCA
GCCACCTCCTGGCCCCTGTCGTCCTTTGTCCCTTCCCAGAAGACCTACCCTGGCAGCTAC

GGGTTCCGTCTAGGTTTCCTGCATTCCGGAACAGCCAAGTCTGTAACCTGCACGTATTCC
CCTCCCCTCAACAAGCTGTTTTGCCAGCTGGCCAAGACCTGCCCGGTGCAGCTGTTGGTC
AGCTCACCACCCCCGCCCGGCACCCGGGTCCGCGCCATGGCCATCTATAAGAAGTCAGAG
TACATGACGGAGGTTGTGAGGCGCTGTCCCCACCATGAGCGCTGCTCTGACTATAGCGAC
GGTCTGGCCCCTCCTCAGCATCTCATCCGGGTGGAAGGGAATTTACGTGCTGAGTATTTG
GACGACAGACACACTTTTCGACACAGCGTGGTGGTGCCCTACGAGCCACCCGAGGTTGGC
TCTGACTGTACCACCATCCACTACAACTTCATGTGTAACAGCTCCTGCATGGGAGGCATG
AACCGGCGGCCCATCCTCACCATCATCACACTGGAAGACTCGAATGGTAATCTGCTGGGA
CGGAACAGCTTTGAGGTGCGTGTTTGTGCCTGTCCTGGGAGAGACCGCCGCACAGAGGAA
GAAAATTTCCGCAAGAAGGGGCAGTCTTGCCCTGAGCTGCCCACTGGGAGCGCTAAGCGA
GCACTGCCTACCAGCACCAGCTCCTCTCCACCACAAAAGAAGAAACCACTGGATGGAGAA
TATTTCACTCTTCAGATCCGTGGGCGTGAACGCTTTGAGATGTTCCGAGAGCTGAATGAG
GCCTTGGAGCTGAAGGATGCTCAGGCTGGAAAGGAGCCAGGGGAAAGCAGGGCTCACTCT
AGCCACCTGAAGTCTAAGAAGGGGCCGTCTCCCTCCCGCCATAAAAAACTGATGTTCAAG
AGAGAAGGGCCTGATTCAGAC
>Elephant_TP53
ATGGAGGAGCCCCAGTCAGATCTCAGCACCGAGCTCCCTCTGAGTCAAGAGACGTTTTCA
TACTTATGGGAACTCCTTCCTGAGAATCCGGTTCTGTCCCCCACACTACCCCCGGCAGTG
GAGGTCATGGACGATCTGCTACTCTCAGAAGACACTGCAAACTGGCTAGAAAGCCAAGTG
GAGGCTCAGGGAATGTCCACAACCCCTGCACCAGCCACCCCTACACCGGTGGCCCCCGCA
CCAGCCACCTCCTGGACCCTGTCATCTTCCGTCCCTTCCCAAAAGACCTACCCTGGCACC
TATGGTTTCCGTCTGGGCTTCCTACATTCTGGGACAGCCAAGTCCGTCACCTGCACGTAC
TCCCCTGACCTTAACAAGCTGTTTTGCCAGCTGGCAAAAACCTGCCCAGTGCAGCTGTGG
GTCGCCTCACCACCCCCGCCCGGCACCCGTGTTCGCACCATGGCCATCTACAAGAAGTCA
GAGCATATGACGGAGGTCGTCAAGCGCTGCCCCCACCATGAGCGCTGCTCTGACTCTAGC
GATGGCCTGGCCCCTCCTCAGCACCTCATCCGGGTGGAAGGAAACCTGCGTGCTGAGTAT
CTGGAGGACAGCATCACTCTCCGACACAGTGTGGTGGTGCCCTACGAGCCGCCCGAGGTC
GGGTCTGACTGTACCACCATCCACTTCAACTTCATGTGTAACAGCTCCTGCATGGGGGGC
ATGAACCGGCGGCCCATCCTCACCATCATCACACTGGAAGACTCCAGTGGTAATCTGCTG
GGACGTAACAGCTTTGAGGTGCGCATTTGTGCCTGTCCTGGAAGAGACAGACGTACAGAA
GAAGAAAATTTCCACAAGAAGGGAGAGCCTTGCCCAGAGCCGCCACCCCCTGGGAGGAGC
ACTAAGCGAGCACTGCCCACCAACACCAGCTCCTCTACCCAGCCAAAGAAGAAGCCACTG
GATGAAGAATATTTCACCCTTCAGATCCGTGGGCGTGAACGCTTCAAGATGTTCCTAGAG
CTAAATGAGGCCTTGGAGCTGAAGGATGCCCAGGCTGGGAAGGAGCCAGAGGGGAGCCGG
GCTCACTCCAGCCCTTCGAAGTCTAAGAAGGGACAGTCTACCTCCCGCCATAAAAAACCA
ATGTTCAAGAGAGAGGGACCTGACTCAGAC
>tropical_clawed_frog_TP53
ATGGAACCTTCTTCTGAGACCGGCATGGAGCCGCCCCTCAGCCAGGAGACCTTCGAGGAT
TTGTGGAGCCTGTTGCCGGACCCCCTACAGACCGGGACAGGTCAGATGGAAAACTTTGCG
GAGTTTTCAGAGTACCCCCTGGCGCCAGACATGACGGTTCTGCAGGAAGGGCTTATGGGT
AATACCGTGCCCACCGTGACTTCGTCTGCTGTCCCCTCAACTGAGGATTACGCAGGGAGC
TACGGGCTCAAATTGGAGTTCCAACAGAATGGAACCGCAAAGTCTGTTACCTGCACATAC
TCCACCGACCTCAACAAGCTCTTCTGCCAATTGGCCAAGACCTGCCCTTTGCTGGTCCGT
GTGGAGAGACCCCCGCCGCTCGGCTCCATTCTCCGGGCTACGGCCGTGTACAAGAAATCC

GAGCACGTGGCCGAGGTGGTCAAGAGGTGCCCCCACCATGAGCGCAGCGTGGAGCCAGGG
GATGACCCTGCCCCTCCCAGTCACCTGATGCGCGTGGAGGGAAATTCAAAGGCTTATTAT
ATGGAGGATGTTGGTACCGGGCGCCATAGTGTCTGTGTGCCCTATGAGGGCCCTCAGGTC
GGAACCGAATGTACCACCGTTCTGTACAACTATATGTGTAACAGCTCGTGTATGGGTGGG
ATGAACCGCCGGCCCATTCTCACTATTATCACCCTGGAGTCCCCAGAGGGGCTGCTGCTT
GGCCGCCGCTGCTTTGAGGTTCGTGTGTGCGCCTGCCCCGGGAGGGATCGTCGCACAGAA
GAAGATAATTGCACAAAGAAACGGGGCCTGAAACCCAACGGGAAGAGAGAGCTTTCTCAC
CCACCATCCAGTGACCCTCCACTTCCCAAGAAGCGCCTTGTTGAGGAGGATGATGAAGAG
ACCTTCACCTTGCTGATTAAGGGGCGGAGCCGCTATGAGATGATAAAGAAACTGAATGAC
GCACTTGAATTACAAGAGAGCCTCGACCAGCAGAAACTGTCCATAAAGTGCCGCAAGTGC
CGGGACGAGATCAAACCCAAGAAAGGGAAGAAGCTGCTGGTGAAAGATGAGCTGCAGGAC
TCCGAG
>Fruitfly_TP53
ATGTATATATCACAGCCAATGTCGTGGCACAAAGAAAGCACTGATTCCGAGGATGACTCC
ACGGAGGTCGATATCAAGGAGGATATTCCGAAAACGGTGGAGGTATCGGGATCGGAATTG
ACCACGGAACCCATGGCCTTCTTGCAGGGATTAAACTCCGGGAATCTGATGCAGTTCAGC
CAGCAATCCGTGCTGCGCGAAATGATGCTGCAGGACATTCAGATCCAGGCGAACACGCTG
CCCAAGCTAGAGAATCACAACATCGGTGGTTATTGCTTCAGCATGGTTCTGGATGAGCCG
CCCAAGTCTCTTTGGATGTACTCGATTCCGCTGAACAAGCTCTACATCCGGATGAACAAG
GCCTTCAACGTGGACGTTCAGTTCAAGTCTAAAATGCCCATCCAACCACTTAATTTGCGT
GTGTTCCTTTGCTTCTCCAATGATGTGAGTGCTCCCGTGGTCCGCTGTCAAAATCACCTT
AGCGTTGAGCCTTTGACGGCCAATAACGCAAAAATGCGCGAGAGCTTGCTGCGCAGCGAG
AATCCCAACAGTGTATATTGTGGAAATGCTCAGGGCAAGGGAATTTCCGAGCGTTTTTCC
GTTGTAGTCCCCCTGAACATGAGCCGGTCTGTAACCCGCAGTGGGCTCACGCGCCAGACC
CTGGCCTTCAAGTTCGTCTGCCAAAACTCGTGTATCGGGCGAAAAGAAACTTCCTTAGTC
TTCTGCCTGGAGAAAGCATGCGGCGATATCGTGGGACAGCATGTTATACATGTTAAAATA
TGTACGTGCCCCAAGCGGGATCGCATCCAAGACGAACGCCAGCTCAATAGCAAGAAGCGC
AAGTCCGTGCCGGAAGCCGCCGAAGAAGATGAGCCGTCCAAGGTGCGTCGGTGCATTGCT
ATAAAGACGGAGGACACGGAGAGCAATGATAGCCGAGACTGCGACGACTCCGCCGCAGAG
TGGAACGTGTCGCGGACACCGGATGGCGATTACCGTCTGGCTATTACGTGCCCCAATAAG
GAATGGCTGCTGCAGAGCATCGAGGGCATGATTAAGGAGGCGGCGGCTGAAGTCCTGCGC
AATCCCAACCAAGAGAATCTACGTCGCCATGCCAACAAATTGCTGAGCCTTAAGAAACGT
GCCTACGAGCTGCCA
>Fugu_TP53
ATGGAAGATGAAGGCTTCAGTTTGCCTCTGAGCCAGGACACCTTTCAGGATCTGTGGGAG
AATGTGGCGGCACCCTCCATAGTTACGATCCCAACAACGAGCTTGGAAAACGAGCAATGG
CACCAAGAACTTCTCATGAACTACCCAGAGTTGCCTTTCAATGAAGAACTCTTCAATCTG
CCCTCGGAGATGGCGAGCAAAGACAGCGCGAACCTTTCCACCCCAACCGTCCCGGTTACC
ACCGATTACCCAGGAGAATATGGCTTTGAGCTGCGCTTCCAAAAGTCCGGCACTGCAAAG
TCCGTCACCTCCACCTACTCCGAGATCCTCAATAAGTTGTACTGCCAGCTTGCCAAGACC
AGCCTGGTTGAGGTGCTTCTCATCAAGAAGCCTCCTGCGGGCGCCGTCCTGAGGGCTACA
GCCATTTACAAGAAGATCGAACACGTGGCAGATGTGGTCCGCCGGTGCCCCCACCACCAA
AACGAGGACTCCGCGGCACACCGGAGCCATCTCATCAGAATGGAGGGCAGCCAGCGGGCT
CAGTACTTTGAGGATCCGCACACAAAAAGACAGAGCGTGACGGTGCCGTACGAGCCCCCC

CAGCTCGGCTCTGAATTCACCACCATCCTCCTCAGTTTTATGTGCAATAGTTCCTGCATG
GGGGGCATGAACCGCCGGCCCATCCTCACCATCCTGACCCTGGAGACTCAGGAGGGCGTG
GTCTTGGGCCGCAGGTGCTTTGAGGTCCGTGTCTGCGCATGTCCCGGCAGGGACCGCAAA
ACGGAGGAGGCCAACAGCACCAATATGCAGAACGGAACCAAAGAAACTAAGAAGCGAAAG
AGCGTCCCACCTCCTGCCGCCGCCGCCGCCGCCAAGAAGTCCAAGACGGCCTCCAGTGCC
GAGGAGGACGACAAGGAGCTTTTCACTCTGCAGATCCGCGGTCGCAAACGCTACGAAATG
CTGAAGAAGATCAACGACGGTCTGGAATTGCTTGAAAATAAACCCAAGTGCAAGGCCGCT
GCCAAACCGGAGTGTCCCGTCCCTCCGAGAGGCAAGAGGCTGCTGCACCGGGGAGAGAAG
AGCGACAGCGAC
>Anole_lizard_TP53
ATGGCCTCCGCGGCTTCCTCTCGCGGTAACGGGCGAGACTTGGAGCCCGGGGATTCCCCG
GCACATGGGTGCTGTGCTGTGCCTTGCTTGGCGCCCGGGGATTTTCATTTGGAAGCGATG
CAGAGGCGAGGAGGGATGGAAGACCCTTCGCTCCTTGATTCCGATTTCGGGATGATGGAG
ATGGACCCAAACGAAGTCTTGGACCTGGTGCCCATGCCAAGCTCCCCTCTGAGCCAGGAC
ACCTTCCAAACCCTGTGGAGCTCCTTACTGGACGGAAAAGAGGATCAGTCCCTTTTGAAT
CCGGACCTGCCGTATCCCATGTCCTATGAGGAAGGGACCAGCCCGAAGGCAGCGGAAGGG
GACTACAGCACCCTCGAACCCGTTTCATCGGTGTTCGTCTCGACACCCATCATCCCCTCC
ACCGAGGACTATGTTGGTGACCACGGCTTCGAGCTGGCCTTTGAGCAGTCGGGCACCGCC
AAGTCCGTCACCTGCACCTATTCTCCAAAGCTCAACAAACTTTTTTGCCAGCTGGCCAAG
ACCTGCCCCGTCCACATCAAAGTGGCCAACATGCCCCCTTTGGGTGCCGTCATCCGCACC
ATGGCCGTCTACAAGAAGTCCGAACACGTGGCCGAAGTGGTGAAGCGCTGCCCACACCAC
GAGCGTTCTCAGGAGTTCAGCGACAGCAGTGCGCCAGCTGAGCACCTCATAAGGGTGGAG
GCCAATCAGCAGGCCAGATACATTGCCGACCCAAACAGGCGCCACAGTGTCATCGTCCCA
TATGAACCGCCACAGGTCGGGACCGATAATACTACTTTATTGCTCAATTTTATGTGCAAT
AGCTCTTGCATGGGAGGCATGAACCGGCGTGCCATTTTGGCCATCATCACTTTGGAGACT
CTGCAAGGTGACCTCCTCGGACGCCGCTGCTTCGAGGTCCGGGTTTGCGCTTGTCCCGGG
AGGGACCGGAAGTCGGAGGAGGAGAATGCCCTGAAAGCAGCCGTCCCTAAAGGGAAATCC
AAAAAAGTTGTTCTTCCAGCTGCTCCTCGGAGCAACTCTTCTGAAAACGCTAAGAGATCA
GCTGCGGGGTCTTCCAACCACGAGACCGAAAGCGGACCCTATATCCTCCAGGTCCGCAAT
CGGAAACACTACCGGATGCTGAAGATGATCCTGGAAGGGCTTGAACTTCGCGAGAAGCAG
CAAGGAGAGGAGGAAGAGGAGGCGGAGCCAGAAACCCGCTGCTTGCCAAAGGCTCCAAAA
CGGAAGAGGCTCAGAGTGAAGGACGAGAACCCAGATTCGGAC
>Horse_TP53
ATGGAGGAGACGCAGACAGAACTCGGCATCGAGCCCCCTCTGAGTCAGGAGACGTTTTCA
GATTTGTGGAAGCTACTTCCTGAAAACAATGTTCTGTCCCCTGACCTGTCCCCAGCAGTG
AATAATCTGCTGCTGTCCCCAGATGTTGTGAACTGGCTGGATGAAGGCCCAGATGAAGCC
CCCAGAATGCCAGCAGCTCCTGCACCACTAGCCCCTGCACCAGCCACCTCCTGGCCCCTG
TCATCCTTTGTCCCTTCCCAAAAGACCTACCCTGGCTGCTATGGTTTCCGTTTAGGGTTC
CTGAATTCTGGGACAGCCAAGTCTGTTACTTGCACGTACTCCCCTACCCTCAACAAACTG
TTTTGCCAGCTGGCAAAGACCTGCCCCGTGCAGCTCCTGGTCAGCTCACCACCCCCACCC
GGCACCCGTGTCCGGGCCATGGCCATCTACAAGAAGTCAGAGTTCATGACGGAGGTCGTG
AGGCGCTGCCCCCACCATGAACGCTGCTCTGACAGTAGCGATGGTCTGGCCCCTCCTCAG
CATCTCATCCGGGTGGAAGGGAATCTGCGTGCTGAGTATTTGGAAGACAGAAACACTTTT
CGACATAGCGTGGTGGTGCCCTACGAGCCACCTGAGGTTGGCTCTGACTGTACCACCATC

CACTACAACTTCATGTGTAACAGCTCCTGCATGGGCGGCATGAACCGGCGGCCCATCCTC
ACTATCATCACCCTGGAAGACTCCAGTGGTAATCTGCTGGGACGGAACAGCTTTGAGGTG
CGTGTTTGTGCCTGTCCTGGGAGAGACCGGCGCACAGAGGAAGAAAACTTCCGCAAGAAG
GAGGAACCTTGCCCTGAGCCGCCTCCTAGGAGCACTAAGCGAGTATTGTCCAGTAACACC
AGCTCCTCTCCCCCACAAAAGAAGAAGCCACTGGATGGAGAATATTTCACCCTTCAGATC
CGTGGGCGTGAACGTTTTGAGATGTTCCGAGAGCTGAATGAGGCCTTGGAGCTGAAAGAT
GCCCAGACTGGAAAGGAGCCAGGGGGAAGCAAGGCTCACTCCAGCCACCTGAAGTCTAAG
AAGGGGCAGTCTACCTCCTCCCATAAAAAGCTGATATTCAAGAGAGAAGGGCCTGACTCA
GAC
>Human_TP53
ATGGAGGAGCCGCAGTCAGATCCTAGCGTCGAGCCCCCTCTGAGTCAGGAAACATTTTCA
GACCTATGGAAACTACTTCCTGAAAACAACGTTCTGTCCCCCTTGCCGTCCCAAGCAATG
GATGATTTGATGCTGTCCCCGGACGATATTGAACAATGGTTCACTGAAGACCCAGGTCCA
GATGAAGCTCCCAGAATGCCAGAGGCTGCTCCCCGCGTGGCCCCTGCACCAGCAGCTCCT
ACACCGGCGGCCCCTGCACCAGCCCCCTCCTGGCCCCTGTCATCTTCTGTCCCTTCCCAG
AAAACCTACCAGGGCAGCTACGGTTTCCGTCTGGGCTTCTTGCATTCTGGGACAGCCAAG
TCTGTGACTTGCACGTACTCCCCTGCCCTCAACAAGATGTTTTGCCAACTGGCCAAGACC
TGCCCTGTGCAGCTGTGGGTTGATTCCACACCCCCGCCCGGCACCCGCGTCCGCGCCATG
GCCATCTACAAGCAGTCACAGCACATGACGGAGGTTGTGAGGCGCTGCCCCCACCATGAG
CGCTGCTCAGATAGCGATGGTCTGGCCCCTCCTCAGCATCTTATCCGAGTGGAAGGAAAT
TTGCGTGTGGAGTATTTGGATGACAGAAACACTTTTCGACATAGTGTGGTGGTGCCCTAT
GAGCCGCCTGAGGTTGGCTCTGACTGTACCACCATCCACTACAACTACATGTGTAACAGT
TCCTGCATGGGCGGCATGAACCGGAGGCCCATCCTCACCATCATCACACTGGAAGACTCC
AGTGGTAATCTACTGGGACGGAACAGCTTTGAGGTGCGTGTTTGTGCCTGTCCTGGGAGA
GACCGGCGCACAGAGGAAGAGAATCTCCGCAAGAAAGGGGAGCCTCACCACGAGCTGCCC
CCAGGGAGCACTAAGCGAGCACTGCCCAACAACACCAGCTCCTCTCCCCAGCCAAAGAAG
AAACCACTGGATGGAGAATATTTCACCCTTCAGATCCGTGGGCGTGAGCGCTTCGAGATG
TTCCGAGAGCTGAATGAGGCCTTGGAACTCAAGGATGCCCAGGCTGGGAAGGAGCCAGGG
GGGAGCAGGGCTCACTCCAGCCACCTGAAGTCCAAAAAGGGTCAGTCTACCTCCCGCCAT
AAAAAACTCATGTTCAAGACAGAAGGGCCTGACTCAGAC
>Macaque_TP53
ATGGAGGAGCCGCAGTCAGATCCTAGCATCGAGCCCCCTCTGAGTCAGGAAACATTTTCA
GACCTATGGAAACTACTTCCTGAAAACAACGTTCTGTCCCCCTTGCCGTCCCAAGCAGTG
GATGATTTGATGCTGTCTCCAGACGATCTTGCACAATGGTTAACTGAAGACCCAGGTCCA
GATGAAGCTCCGAGAATGTCAGAGGCTGCTCCCCCCATGGCCCCCACACCAGCAGCTCCT
ACACCGGCGGCCCCTGCACCAGCCCCCTCCTGGCCCCTGTCATCCTCTGTCCCTTCCCAG
AAAACCTACCATGGCAGCTACGGTTTCCGTCTGGGCTTCCTGCATTCTGGAACAGCCAAG
TCTGTGACTTGCACGTACTCCCCTGACCTCAACAAGATGTTTTGCCAGCTGGCCAAGACC
TGCCCCGTGCAGCTATGGGTTGATTCCACACCCCCACCCGGCAGCCGCGTCCGCGCCATG
GCCATCTACAAGCAGTCACAGCACATGACGGAGGTCGTGAGGCGCTGCCCCCACCATGAG
CGCTGCTCAGACAGCGATGGACTGGCCCCTCCTCAGCACCTTATCCGAGTGGAAGGAAAT
TTGCGTGTGGAGTATTCGGATGACAGAAACACTTTTCGACATAGTGTGGTGGTGCCCTAT
GAGCCGCCTGAGGTTGGCTCTGACTGTACCACCATCCACTACAACTACATGTGTAACAGT
TCCTGCATGGGCGGCATGAACCGGAGGCCCATCCTTACCATTATCACACTGGAAGACTCC

AGTGGTAATCTACTGGGACGGAACAGCTTTGAGGTGCGAGTTTGTGCCTGTCCTGGGAGA
GACCGGCGCACAGAGGAAGAGAATTTCCGCAAGAAAGGGGAGCCTTGCCACCAGCTGCCC
CCTGGGAGCACTAAGCGAGCACTGCCCAACAACACCAGCTCCTCTCCCCAGCCAAAGAAG
AAACCACTGGATGGAGAATATTTCACCCTTCAGATCCGTGGGCGTGAGCGCTTCGAGATG
TTCCGAGAGCTGAATGAGGCCTTGGAACTCAAGGATGCCCAGGCTGGGAAAGAGCCAGCG
GGGAGCAGGGCTCACTCCAGCCACCTGAAGTCCAAGAAGGGGCAATCTACCTCCCGCCAT
AAAAAATTCATGTTCAAGACAGAGGGGCCTGACTCAGAC
>Medaka_TP53
ATGGATCCTGTACCCGACCTGCCCGAGAGCCAAGGTTCTTTTCAAGAACTCTGGGAGACT
GTTAGTTATCCTCCGTTGGAAACTTTATCACTTCCAACTGTAAATGAGCCCACCGGGTCA
TGGGTCGCAACGGGGGATATGTTTCTCTTGGATCAAGACCTCAGTGGGACCTTCGATGAC
AAGATCTTYGACATACCGATTGAGCCAGTTCCCACCAACGAAGTAAACCCGCCACCYACC
ACCGTCCCGGTGACCACCGACTACCCGGGAAGCTACGAACTGGAACTTCGTTTTCAAAAA
TCTGGCACTGCAAAGTCTGTGACGTCCACATACTCTGAAACTTTAAATAAGCTGTACTGC
CAGCTTGCAAAAACCAGCCCCATAGAAGTCCGGGTGAGCAAGGAGCCTCCAAAGGGCGCC
ATTCTCAGGGCCACTGCGGTCTACAAGAAGACGGAGCACGTGGCGGACGTGGTGAGGAGG
TGTCCCCACCACCAAAACGAGGACTCTGTGGAGCACCGAAGCCATCTGATTCGGGTGGAG
GGCAGCCAGCTGGCCCAGTATTTTGAAGACCCTTATACCAAAAGGCAGAGTGTTACAGTT
CCTTATGAGCCCCCGCAGCCGGGCTCTGAGATGACCACCATCYTGCTYAGCTACATGTGT
AACAGCTCGTGCATGGGGGGGATGAACCGCAGACCCATCCTCACCATCCTCACTCTGGAA
ACCGAGGGTCTGGTTCTTGGCCGGAGGTGTTTYGAGGTCAGAATCTGTGCTTGCCCGGGG
CGGGACCGCAAAACGGAGGAGGAAAGCCGGCAGAAAACGCAGCCCAAAAAAAGGAAGGTG
ACACCCAACACCTCCTCCTCTAAGAGGAAGAAGTCCCACTCAAGTGGAGAGGAGGAGGAC
AACAGAGAAGTCTTTCATTTTGAGGTTTATGGGAGGGAGCGCTACGAGTTCCTTAAGAAG
ATAAACGATGGCCTTGAACTGCTGGAAAAGGAGAGCAAAAGTAAAAACAAAGATTCTGGG
ATGGTCCCCTCCAGTGGGAAGAAGCTGAAGAGCAAT
>Mouse_TP53
ATGACTGCCATGGAGGAGTCACAGTCGGATATCAGCCTCGAGCTCCCTCTGAGCCAGGAG
ACATTTTCAGGCTTATGGAAACTACTTCCTCCAGAAGATATCCTGCCATCACCTCACTGC
ATGGACGATCTGTTGCTGCCCCAGGATGTTGAGGAGTTTTTTGAAGGCCCAAGTGAAGCC
CTCCGAGTGTCAGGAGCTCCTGCAGCACAGGACCCTGTCACCGAGACCCCTGGGCCAGTG
GCCCCTGCCCCAGCCACTCCATGGCCCCTGTCATCTTTTGTCCCTTCTCAAAAAACTTAC
CAGGGCAACTATGGCTTCCACCTGGGCTTCCTGCAGTCTGGGACAGCCAAGTCTGTTATG
TGCACGTACTCTCCTCCCCTCAATAAGCTATTCTGCCAGCTGGCGAAGACGTGCCCTGTG
CAGTTGTGGGTCAGCGCCACACCTCCAGCTGGGAGCCGTGTCCGCGCCATGGCCATCTAC
AAGAAGTCACAGCACATGACGGAGGTCGTGAGACGCTGCCCCCACCATGAGCGCTGCTCC
GATGGTGATGGCCTGGCTCCTCCCCAGCATCTTATCCGGGTGGAAGGAAATTTGTATCCC
GAGTATCTGGAAGACAGGCAGACTTTTCGCCACAGCGTGGTGGTACCTTATGAGCCACCC
GAGGCCGGCTCTGAGTATACCACCATCCACTACAAGTACATGTGTAATAGCTCCTGCATG
GGGGGCATGAACCGCCGACCTATCCTTACCATCATCACACTGGAAGACTCCAGTGGGAAC
CTTCTGGGACGGGACAGCTTTGAGGTTCGTGTTTGTGCCTGCCCTGGGAGAGACCGCCGT
ACAGAAGAAGAAAATTTCCGCAAAAAGGAAGTCCTTTGCCCTGAACTGCCCCCAGGGAGC
GCAAAGAGAGCGCTGCCCACCTGCACAAGCGCCTCTCCCCCGCAAAAGAAAAAACCACTT
GATGGAGAGTATTTCACCCTCAAGATCCGCGGGCGTAAACGCTTCGAGATGTTCCGGGAG

CTGAATGAGGCCTTAGAGTTAAAGGATGCCCATGCTACAGAGGAGTCTGGAGACAGCAGG
GCTCACTCCAGCTACCTGAAGACCAAGAAGGGCCAGTCTACTTCCCGCCATAAAAAAACA
ATGGTCAAGAAAGTGGGGCCTGACTCAGAC
>Naked_mole_rat_TP53
ATGGAAGAGCCACAGTCGGATCTCAGCATCGAGCCTCCACTGAGTCAGGAGACATTTTCA
GACTTATGGAAACTACTTCCTGAAAACAACGTTCTGTCCAGCTCACTGTCCTCTCCCATG
GATGATCTGCTGCTGTCCCCAGAAGATGTTGTAAACTGGCTGGGAGGAAACCCAGATGAA
GATGTCCAAGTGTCAGCAGCTCCTGTACCAGAGCCCCCAACACCAGTGGCCCCTGCCCCG
GCAGCTCCCGCACCAGCCACTTCCTGGCCTCTGTCATCCTCCGTCCCTTCCCATAAGACC
TACCAAGGCAACTATGGTTTCCATCTGGGCTTCCTTCAGTCTGGGACGGCCAAATCTGTC
ACATGCACGTACTCCCCTGTTCTCAACAAGTTATTCTGCCAACTGGCAAAGACCTGCCCT
GTGCAAGTGTGGGTCGAATCACCACCCCCACCTGGCACCCGAGTCCGTGCCATGGCCATC
TACAAGAAGTCACAGCACATGACAGAAGTTGTGAGGCGCTGCCCCCACCATGAGCGCTGC
TCCGATAGTGATGGCCTGGCCCCTCCTCAGCATCTTATCCGGGTGGAAGGAAATCTGCGT
GCAGAATATTTGGATGACAGAACCACTTTTCGCCATAGCGTGGTGGTACCCTATGATCTG
CCTGAGGTTGGCTCTGACTGTACCACCATCCACTACAACTATATGTGCAACAGTTCTTGC
ATGGGGGGCATGAACCGTAGGCCCATCCTCACCATTATCACACTGGAAGACTCCAGTGGG
AACCTGCTGGGGCGGAACAGCTTTGAGGTGCGTGTTTGTGCCTGTCCTGGGAGAGACCGG
CGCACAGAGGAAGAAAATTTCCACAAGAAAGGGGGGTCATGCCCAGAGCCAACACCAGGA
AGCATTAAGCGAGCACTGCCCACTGGCACCAACTCTTCTCCTCAGCCAAAGAAGAAACCA
CTGGATGGGGAATATTTCACCCTTAAGATCCGTGGGCGTGAACGCTTTGAGATGTTCCGA
GAGCTAAATGAGGCCTTGGAACTCAAGGATGCCCAAACTGAGAAGGAGCCAGGGGAGAGC
AGGCCTCACTCAAGCTACCTGAAGTCTAAGAAGGGGCAGTCTACCTCCTGTCATAAAAAA
CTAATGTTCAAGAAAGAAGGACCTGATTCAGAC
>Opossum_TP53
ATGAGTCAGTCTTTAATCATTCACCATAAGATTTATCCCAAGCAAGATTCTAGGGGTCAA
CTTCTTGGACGTCGAAGCTTTGAAGTTCGTATATGTGCCTGCCCTGGAAGAGACCGGCGG
ACTGAGGAGGAAAATTTCCACAAGAAGGGAGGTCCCAGCCCACAGCCATCATCTGAGAGC
AATAAGCGTGCATTACCCACAACGCCTGGCAGCACTCCCAAGGCTAAAAAGAAGCTAGTG
GAAGGAGAATATTTCACTCTTCAGATCCGAGGTCGACAGCGCTATGAGCTGCTCCGGGAG
ATAAATGAAGCCTTGGAGCTGAAAGAAGCTCACAGCAGGAAAGAACCTGAGGGGAGCCGC
CCCCACCGCAGCCAATTGAAGTCCAAGAGAGGAGATTCCACCCCCTGTCAAGGCAAAAGA
CTTTTGGTGAAAAATGAAGTTCCTGACTCAGAC
>Orangutan_TP53
ATGGAGGAGCCACAGTCAGATCCTAGCGTCGAGCCCCCACTGAGCCAGGAAACATTTTCA
GACCTATGGAAACTACTTCCTGAAAACAACGTTCTGTCCCCCTTGCCGTCCCAAGCGGTG
GATGATTTGCTGCTGTCCCCGGACGATATTGCACAATGGTTCATTGAAGACCCAGGTCCA
GATGAAGCTCCCAGAATGTCAGAGGCTGCTTCCCCTGTGGCCCCCGCACCAGCAGCTCCT
ATACCGGCGGCCCCTGCACCAGCCCCCTCCTGGCCCCTGTCATCCTCTGTCCCTTCCCAG
AAAACCTACCAGGGCAGCTACGGTTTCCGTCTGGGCTTCTTGCATTCTGGGACAGCCAAG
TCTGTGACTTGCACGTACTCCCCTGCCCTCAACAAGATGTTTTGCCAGCTGGCCAAGACC
TGCCCTGTGCAGCTGTGGGTTGATTCCACACCCCCACCTGGCACCCGCGTCCGCGCCATG
GCCATCTACAAGCAGTCACAGCACATGACGGAGGTCGTGAGGCGCTGTCCCCACCATGAG
CGCTGCTCAGATAGTGATGGTCTGGCCCCTCCTCAGCATCTTATCCGAGTGGAAGGAAAT

TTGCGTGTGGAGTATTTGGATGACAGAAACACTTTTCGACATAGTGTGGTGGTGCCCTAT
GAGCCGCCTGAGGTTGGCTCTGACTGTACCACCATCCACTACAACTACATGTGTAACAGT
TCCTGCATGGGCGGCATGAACCGGAGGCCCATCCTCACCATCATCACACTGGAAGACTCC
AGTGGTAATCTACTGGGACGGAACAGCTTTGAGGTGCGCGTTTGTGCCTGTCCTGGGAGA
GACCGGCGCACAGAGGAAGAGAATTTCCGCAAGAAAGGGGAGCCTCACCATGAGCTGCCC
CCTGGGAGCACTAAGCGAGCACTGCCCAACAACACCAGCTCCTCTCCCCAGCCAAAGAAG
AAACCACTGGATGGAGAATATTTCACCCTTCAGATCCGTGGGCGTGAGCGCTTCGAGATG
TTCCGAGAGCTGAATGAGGCCTTGGAACTCAAGGATGCCCAGGCTGGGAAGGAGCCAGGG
GGGAGCAGGGCTCACTCCAGCCACCTGAAGTCCAAGAAGGGTCAGTCTACCTCCCGCCAT
AAAAAACTCATGTTCAAGACAGAAGGGCCTGACTCAGAC
>Western_painted_turtle_TP53
ATGGAGCCGATGTTGGACCCCGGGCTAGAGCCGCCCCTGAGCCAGGAGAGCTTCTCTGAT
TTCTGGAGCAATATCTGGTATCCCACGAACGCGGATTCCACCGCAACGGAGAGCCAGCGG
CTGTCTAGCCTGCCGGACCCAGACCCGGACCTGGCCCTGGGCCTGGCCCTGGGCCTGTCT
GGCTCGGGGGACCCCTCCCTCCTGCTCTCACAGGCTGGGGGCAGCGACGGGGGCTGGGAG
CTCCCCGGCCCGGCCCCAGAGCCGCCCCCACCTCTTCCACTGTCCCCTCCACCGAGGAC
TACGCTGGGGAGCACGGATTCGAGCTGGTCTTCCAGCAGTCGGGGACCGCCAAGTCCGTC
ACCTGCACCTACTCCTTGGAGCTGAACAAGCTCTACTGCCAGCTGGCAAAGACCTGCCCT
GTGCAGATCAAGACGGCCAGCCAGCCCCCGCCCGGCTCCGTCGTCCGGGCCACAGCCGTC
TACAAGAAATCGGAGCACGTGGCCGAGGTGGTGAGACGCTGCCCCCACCACGAACGCTGC
GAGGAGTACCGCGACGGGGTCGCCCCGGCCCGCCACCTGATCCGGATCGAGGGGAACCAG
CAGGCGCATTACTACGATGACGAAAACACCAAGCGCCAGAGTGTCACGGTGCCCTATGAG
ACGCCCCAGGTGGGGTCAGACTGCACCACCGTGCTGTATAACTTCATGTGTAACAGTTCG
TGCATGGGGGGCATGAACCGGCGCCCCATCCTGGCCATCATCACTCTGGAGGGCAGGCAC
GGGCAGCTCCTGGGCCGCCGATGCTTCGAGGTTCGAGTCTGCGCCTGCCCCGGACGGGAC
CGCAGGACAGAGGAGGATAATTTCCGCAAGAAATTGGCCGGCAGGGTCCTGAGCGGGGCC
GGGGCCCTCAAAGGGGGCAGAGCCAAGAGGGCTCTCCAGGCTACCATGGAAACAGCTGAG
AACCCCAAGAAGCGGGTGGTGTCCGCCGAGAAAGAGGTCTTCCTACTCGAGGTTCATGGG
CGCAAGCGATACATGATGCTGAAGGAAATCAACGACGCCCTGGAGATGGTGGCCGCCAAG
CAGCAGGGGGAGCCGGAGAGTCACCGGAACCCCACGCCCTCGAGGTTGCTGAAGACCCGG
AAGGAGTCAGGGGACGAGCTGCTGCCTCAGAGTGGAAAGAAGCTGCTGGTGAAGGAGGAG
GATTCGGAA
>Panda_TP53
ATGCAGGACCCACAGTCGGAGCTCACCATCGACCCCCCTCTGAGCCAGGAGACATTTTCC
GAATTGTGGAACCTACTTCCTGAAAACAATGTTCTGTCTTCCGAGCTGTCCCCAGGAGTG
GATGAACTGCTGCTCTCAGAAGGCGTCGTGAACTGGATGGACGAAGGTTCAGATGACACT
CCCAGGATGCCAGTGGCTCCTGCCCCTGCGGCCCCTGGACCAGCCATCTCCTGGCCCCTG
TCATCCTCTGTCCCTTCCCCGAAGACCTACCCTGGCACCTATGGGTTCCGTCTGGGGTTC
CTGCATTCCGGGACAGCCAAGTCTGTTACTTGCACGTACTCCCCCTCCCTCAACAAGCTG
TTTTGCCAGCTGGCAAAGACCTGCCCCGTGCAGCTGTGGGTCAACTCCCCGCCCCCACCC
GACACCTGCGTCCGCGCCATGGCCATTTATAAGAAGTCGGAGTTCGTGACAGAGGTTGTG
CGGCGCTGTCCCCACCATGAGCGCTGCTCTGACAGTAGTGATGGGCTGGCCCCGCCTCAG
CATCTCATCCGGGTGGAAGGAAACTTGCGTGCCAAGTACTTGGACGACAGAAACACTTTC
CGACATAGCGTGGTGGTGCCCTATGAGCCGCCCGAGGTCGGCTCTGACTGTACCACCGTC

CACTATAACTACATGTGTAACAGTTCCTGCATGGGAGGCATGAACCGGCGGCCCATCCTC
ACCATCATCACCCTGGAAGATTCCAGTGGTAATGTGCTGGGACGGAGCAGCTTTGAGGTA
CGTGTCTGTGCCTGTCCTGGGAGAGACCGGCGCACAGAGGAGGAGAATTTCCGTAAGAAG
GGGGAGCCTTCCCCCGAGCTGCCCCCCGGAAGCACTAAGCGAGCACTGCCTCCCAGCACC
AGCTGCTCTCCCCCACAAAAGAAGAAGCCACTGGATGGGGAATATTTCACCCTTCAGATC
CGTGGGCGTGAACGTTTCAATATGTTCAGGGAGCTGAATGAGGCCTTGGAGCTGAAGGAT
GCCCTGAGTGGCAAGGAGCCGGGGGGGAGCAGGGCTCACTCCAGCCACCTGAAGGCTAAG
AAGGGGCAGTCTACCTCCCGCCATAAAAAGCTGATGTTCAAGAGAGAGGGACCCGACTCA
GAC
>Pig_TP53
ATGGAGGAGTCGCAGTCCGAGCTGGGCGTGGAGCCCCCTCTGAGTCAGGAGACATTTTCA
GACTTGTGGAAACTGCTTCCTGAAAACAACCTGCTGTCCTCTGAGCTCTCCCTGGCAGCA
GTGAACGATCTGCTGCTGTCCCCAGTCACGAACTGGCTGGATGAAAATCCAGATGACGCC
TCCAGAGTGCCAGCGCCTCCTGCAGCAACAGCGCCCGCACCAGCTGCCCCCGCACCAGCC
ACCTCCTGGCCCCTGTCGTCCTTTGTCCCTTCTCAGAAGACCTACCCTGGCAGCTATGAT
TTCCGTCTAGGGTTCCTGCATTCTGGAACAGCCAAGTCTGTAACCTGCACGTACTCCCCT
GCCCTCAATAAGCTGTTTTGCCAGCTGGCCAAGACCTGCCCGGTGCAGCTGTGGGTCAGC
TCGCCACCCCCGCCTGGCACCCGTGTCCGCGCCATGGCCATCTACAAGAAGTCAGAGTAC
ATGACCGAGGTGGTGAGGCGCTGTCCCCACCATGAGCGCAGCTCTGACTATAGCGATGGT
CTGGCCCCTCCCCAGCATCTCATCCGGGTGGAAGGGAATTTACGGGCCGAGTACTTGGAT
GACAGAAACACTTTTCGACACAGCGTTGTGGTGCCCTACGAGCCGCCCGAGGTCGGCTCT
GACTGTACCACCATCCACTACAACTTCATGTGTAACAGCTCCTGCATGGGGGGCATGAAC
CGGCGGCCCATCCTCACCATCATCACACTGGAAGATGCCAGTGGCAACTTGCTGGGACGG
AACAGCTTTGAGGTGCGTGTTTGTGCCTGTCCTGGGAGAGACCGCCGCACAGAGGAAGAA
AATTTCCTCAAGAAGGGCCAATCTTGCCCCGAGCCTCCCCCTGGCAGCACTAAGCGAGCA
CTGCCCACCAGCACCAGCTCCTCGCCAGTGCAAAAGAAGAAGCCACTGGATGGCGAGTAT
TTCACCCTCCAGATCCGTGGGCGTGAACGCTTCGAGATGTTCCGAGAGCTGAATGACGCC
TTGGAGCTGAAGGATGCCCAGACTGCGCGGGAGTCGGGGGAAAACAGGGCCCACTCCAGC
CACCTGAAGTCTAAGAAGGGGCAGTCTCCCTCCCGCCATAAAAAACCGATGTTCAAGAGA
GAAGGACCTGACTCAGAC
>Platypus_TP53
ATGATTTTGATGGTCGCCCTCCCCTGCGTCGGCTTTTCACCCCGTATTCCTGGCTCTGTC
TCCCCCCAGTACTCCCCATTGCTCAACAAGCTGTTCTGCCAGTTGGCCCGGACCTGCCCC
GTCCAGCTGTGGGTCGACTCCCCGCCCCCGCCGGGGGCCCGGGTCCGGGCCATGGCCGTC
TACAAGAAGACCGACCACAGGGCCGAGGTGGTGAAGAGGTGCCCCCACCACGAGCGCTCT
TCCGACGGTGACGGAGCGGCGCCCGCCCAGCATCTGATCCGCGTGGAGGGGAACCCCCAG
GCCACATACTTGAATGACGAGAAGACAACCCGTCAGAGCGTCGTCGTCCCCTACGAGCCA
CCTCAGATCCGTGGCCGGGAGCGCTACGAAATGTTTCGCACAATGAACGAAGCTTTCGAG
TTCAAAGATGCCAAAGGCGGACAGGAACCTGAGGGGACACGCACATGCCGACAGTCCCTG
AAATCGAAGAGGGAGGCTCCAGGCCCCAGGAAGGGCAAACGGCTGCTTGTCAAAGAAAGA
GACTCCCGAGTCCAACTGACATTTCCCCCTGAACTGCCATCCCCCACGCCGGTCCCCACC
TCC
>Rabbit_TP53
ATGGAGGAGTCGCAGTCGGATCTCAGCCTCGAGCCTCCCCTGAGTCAGGAGACGTTTTCA

GACCTGTGGAAACTGCTTCCTGAGAACAACCTGCTGACCACCTCGCTGAACCCTCCCGTG
GATGATCTGCTGTCTGCAGAAGACGTTGCAAACTGGCTCAACGAAGACCCAGAAGAAGGG
CTCCGAGTACCAGCAGCTCCTGCACCAGAGGCCCCCGCCCCAGCTGCCCCTGCGCTGGCG
GCCCCTGCACCAGCCACCTCCTGGCCCCTGTCATCCTCTGTCCCTTCCCAAAAGACCTAC
CATGGCAACTACGGTTTCCGGCTGGGCTTCCTGCACTCTGGGACGGCCAAGTCTGTCACC
TGCACATACTCCCCCTGCCTCAACAAGCTGTTCTGCCAGCTAGCAAAGACCTGCCCTGTG
CAGCTGTGGGTCGACTCGACGCCCCCGCCTGGCACCCGCGTCCGGGCCATGGCCATCTAC
AAGAAGTCTCAGCACATGACGGAAGTTGTCAGACGCTGCCCCCACCACGAGCGCTGCTCC
GACAGCGATGGTCTGGCCCCCCCTCAGCATCTCATCCGGGTGGAGGGAAATCTCCGTGCG
GAGTATTTGGATGACAGAAACACCTTCCGACACAGCGTGGTGGTGCCCTACGAGCCGCCC
GAGGTTGGCTCTGACTGTACCACCATCCACTATAATTACATGTGTAACAGCTCCTGCATG
GGGGGCATGAACCGGCGGCCCATCCTCACCATCATCACACTGGAAGACTCCAGTGGGAAC
CTGCTGGGACGGAACAGCTTTGAGGTGCGAGTGTGTGCCTGTCCTGGGAGGGACCGCCGC
ACGGAGGAAGAAAATTTCCGCAAGAAAGGGGAGCCCTGCCCTGAACTGCCCCCTGGGAGC
TCTAAGCGAGCACTGCCGACCACCACCACCGACTCCTCTCCCCAGACAAAGAAGAAGCCG
CTGGATGGAGAATATTTCATCCTTAAGATCCGCGGGCGTGAACGGTTCGAGATGTTCCGA
GAGCTGAACGAGGCCCTGGAGCTAAAGGATGCCCAGGCCGAGAAGGAGCCCGGGGGGAGC
AGGGCTCACTCCAGCTACCTGAAGGCCAAGAAGGGGCAGTCTACCTCCCGCCATAAAAAA
CCAATGTTCAAGAGAGAGGGGCCTGACTCAGAC
>Tetraodon_TP53
ATGGAAGAGGAAACTTTCAGTCTGCCCTTAAGCCAGGACACCTTCCAAGATCTTTGGGAA
AATGTAGCGGCTCCCTCCATCTCCACCATCCAAACAACAGTTTCAGGAAATGAGTGCTGG
CAGGACGGATCGTTGACTATGGCTCTCATGGACATGCCCTACGATGAGGATCTGTTCAAT
CTGCCTTCTGAGCTGCCAAACAAAGATGGCGCTAACTCTTCCTGCCCCACCGTCCCGGTC
ACCACCGATCACCCCGGAGAGTATGACTTCAAGCTGCGTTTTCAGAAGTCTGGCACGGCA
AAGTCCGTCACCTCCACCTACTCCGAGAGCCTCAACAAGCTCTACTGCCAGCTTGCGAAG
ACCAGTCCGCTGGAAGTGCTCCTGAGCAGGGAGCCTCCGCTGGGGGCCATGCTGAGGGCC
ACTGCCATTTACAAGAAGACGGAGCACGTGGCAGAAGTGGTGCGCCGGTGCCCCCACCAC
CAGAACGAGGACTCCACGGAGAACCGGAGCCACCTGATCAGGATGGAGGGCAGTCAGCGA
GCGCAGTACTTCGAAGATCCGCACACTAAACGGCAGAGCGTCACCGTCCCCTACGAGCCC
CCTCAGCTCGGGTCCGAATTCACCACCATCCTGCTCAGTTTTATGTGCAATAGTTCATGC
ATGGGGGGCATGAACCGCCGGCCCATCCTCGCCATCTTGACCCTGGAAACTCAAGAGGGT
GTCGTGTTGGGACGGAGGTGCTTTGAAGTCCGCGTCTGCGCATGTCCGGGCAGAGACCGC
AAAACCGAGGAGGCCAACAGCACCAAGATGCAGACCGAGACCAAAGATGCCAAAAAGCGC
AAAAGCGCCCCAACGTCTGACAGCACCACCGTGAAGAAGTCCAGGACGGCCTCCAGCGCG
GAGGAGGATGACAAGGAGGTCTTCACTCTGCAGATTCGTGGTCGCAAACGCTATGAAATG
ATAAAGAGGATTAATGACGGTCTGGATTTACTTGAAAATAAAACCAAGTCCAAGACTACT
TATAAACCCGAGGGTCCCGTCCTGCCAAGTGGAAAGAGGCTGATGCACAGAGGAGAGAAG
AGCGACAGCGAC
>Tilapia_TP53
ATGGAAGAACAGGGCGTGGAAAACGTGAGCCTGCCGCTGAGCCAGGAAAGCTTTCCGGAT
CTGTGGGCGAACGTGGTGATGCCGATTAGCACCATTCAGACCGCGGCGCTGAACGAACCG
ACCGGCAGCTGGGTGGCGAGCCTGACCATGGCGCTGATGGATATGCCGGATCTGAACTGC
CTGTTTGAACTGCAGCCGAGCATTAGCACCCTGGATACCGGCAGCCCGCCGACCAGCACC

GTGCCGGTGACCACCGATCATCCGGGCGAATATGATTTTAAACTGCGCTTTCAGAAAAGC
GGCACCGCGAAAAGCGTGACCAGCACCTATAGCGAACTGCTGAACAAACTGTATTGCCAG
CTGGCGAAAACCAGCCCGGTGGAAGTGCTGGTGAGCAAAGAACCGCCGAAAGGCGCGATT
CTGCGCGCGACCGCGGTGTATAAAAAAAGCGAACATGTGGCGGAAGCGGTGCGCCGCTGC
CCGCATCATCAGAACGAAGATAGCGTGGAACATCGCAGCCATCTGATTCGCGTGGAAGGC
AGCAGCCAGCGCGCGCAGTATTTTGAAGATCTGCATACCAAACGCCAGAGCGTGACCGTG
CCGTATGAACCGCCGCAGCTGGGCAGCGAATTTACCACCATTCTGCTGAGCTTTATGTGC
AACAGCAGCTGCATGGGCGGCATGAACCGCCGCCCGATTCTGACCATTCTGACCCTGGAA
ACCCCGGAAGGCCTGGTGCTGGGCCGCCGCTGCTTTGAAGTGCGCGTGTGCGCGTGCCCG
GGCCGCGATCGCAAAACCGAAGAACAGGCGAACAAAAAAGAAAGCGGCCCGAAACAGACC
AAAAAACGCAAAGTGACCCCGAACACCAGCAGCCTGACCACCCCGGCGAAAAAAATGAAA
AGCAGCAGCAGCGGCGAAGATGAAGATAAAGAAGTGTTTCATTTTGAAGTGTATGGCCGC
GAACGCTATGGCCGCTATGAAATGTTTAAAAAAATTAACGAAGGCCTGGATCTGGTGGAA
AGCGATGCGGAAAAATATCGCCAGAAAGGCAAAAAAAAAGATGGCCAGACCCCGGAAGGC
CCGAAAAAAGGCAAAAAACTGCTGGTGAAAGAAGAAAAAAGCGATAGCGAT
>Zebrafish_TP53
ATGGCGCAAAACGACAGCCAAGAGTTCGCGGAGCTCTGGGAGAAGAATTTGATAAGTATT
CAGCCCCCAGGTGGTGGCTCTTGCTGGGACATCATTAATGATGAGGAGTACTTGCCGGGA
TCGTTTGACCCCAATTTTTTTGAAAATGTGCTTGAAGAACAGCCTCAGCCATCCACTCTC
CCACCAACATCCACTGTTCCGGAGACAAGCGACTATCCCGGCGATCATGGATTTAGGCTC
AGGTTCCCGCAGTCTGGCACAGCAAAATCTGTAACTTGCACTTATTCACCGGACCTGAAT
AAACTCTTCTGTCAGCTGGCAAAAACTTGCCCCGTTCAAATGGTGGTGGACGTTGCCCCT
CCACAGGGCTCCGTGGTTCGAGCCACTGCCATCTATAAGAAGTCCGAGCATGTGGCTGAA
GTGGTCCGCAGATGCCCCCATCATGAGCGAACCCCGGATGGAGATAACTTGGCGCCTGCT
GGTCATTTGATAAGAGTGGAGGGCAATCAGCGAGCAAATTACAGGGAAGATAACATCACT
TTAAGGCATAGTGTTTTTGTCCCATATGAAGCACCACAGCTTGGTGCTGAATGGACAACT
GTGCTACTAAACTACATGTGCAATAGCAGCTGCATGGGGGGGATGAACCGCAGGCCCATC
CTCACAATCATCACTCTGGAGACTCAGGAAGGTCAGTTGCTGGGCCGGAGGTCTTTTGAG
GTGCGTGTGTGTGCATGTCCAGGCAGAGACAGGAAAACTGAGGAGAGCAACTTCAAGAAA
GACCAAGAGACCAAAACCATGGCCAAAACCACCACTGGGACCAAACGTAGTTTGGTGAAA
GAATCTTCTTCAGCTACATTACGACCTGAGGGGAGCAAAAAGGCCAAGGGCTCCAGCAGC
GATGAGGAGATCTTTACCCTGCAGGTGAGGGGCAGGGAGCGTTATGAAATTTTAAAGAAA
TTGAACGACAGTCTGGAGTTAAGTGATGTGGTGCCTGCCTCAGATGCTGAAAAGTATCGT
CAGAAATTCATGACAAAAAACAAAAAAGAGAATCGTGAATCATCTGAGCCCAAACAGGGA
AAGAAGCTGATGGTGAAGGACGAAGGAAGAAGCGACTCTGAT
>Chinese_softshell_turtle_PPARGC1A
ATGGCGTGGGACATGTGCAACCAGGACTCTGTATGGAGTGATATAGAGTGTGCTGCTCTG
GTTGGTGAAGACCAGCCTCTTTGCCCAGATCTCCCAGAACTTGACCTCTCTGAACTAGAT
GTGAATGATTTGGATGCAGACGGTTTTCTGGGAGGACTCAAGTGGTACAGCGACCAATCA
GAAATCATTACTAATCAGTACAGCAATGAATCATCAAATATATTTGAGAAGATAGATGAA
GAGAATGAAGCAAACTTGCTAGCAGTTCTCACAGAGACACTGGACAGTATCCCTGTGGAT
GAGGATGGATTGCCTTCATTTGATACACTGACAGATGGAGATGTGACCAATGAAAATGAT
GCTAGCCCTTTGCCAATGCCCGACGGCACCCCTCCGACTCAGGAGGCAGAAGAGCCGTCT
CTACTTAAGAAGCTCTTGCTGGCTCCAGCCAACATTCAGCTAAATTACAATGAATGCAGT

GGTCTCAGCACACAAAACCATGCAAACACTAATCACAGGATCAGAACAAGCCCTGTGGTT
GTTAAGACCGAGAATTCATGGAGCAATAAACCGAAGAGCATTTGTCAACAGCAAAAGCCA
CAAAGACGTCCCTGCTCTGAGCTTCTCAAGTATCTGACTACAAATGATGACCCTCCTCAG
ACCAAACCCACAGAGAACAGGAACAGCAGTAAAGACAAATGCATCTCCAAAAAGAAGCCC
CATCTGCAGTCTCAGGCACATCATTTGCAAGCCAAACCAACAAGTTTATCACTTCCTTTG
ACACCTGAGTCACCAAATGATCCCAAGGGTTCCCCATTTGAGAACAAGACTATTGAACAA
ACCTTAAGTGTGGAACTCTCTGGAACTGCAGGCCTAACTCCACCTACAACCCCTCCTCAC
AAAGCCAACCAAGATAATCCTTTCAGAACTTCACCCAAACTGAAGTCATCATGCAAGACT
GTTGTACCACCCTTAAAAAAGCCCCGCTACAGTGAATCTTCCAGTTCTCAAGGAAATAAT
ACAATCAAGAAGAGTCCAGAACAATCTGAGCTGTATGCACAGCTTAGCAAGACAACAGTC
CTGTCCAGTGGCCATGAGGAGAGAAAGACAAAACGGCCAAATTTGCGGCTTTTTGGTGAC
CATGACTACTGTCAATCTGTGAATTCGAAAACTGAAATACATATTAAAATATCGCAGGAA
CTTCAGGACTCTGGACAACAAGAATTTAAAGATTCTTCATCTGTGTGGCAGTGTCAGATT
TGTTCGTCTTTAGAACAAGACCAGTATTACAAGAGAGACACTTTACAGGCAAGTAAGCAG
GGTTCCCTGTCTAACAGCCGAAAACAGCTCCAAGATCAGGAGATCCGGGCTGAACTGAAT
AAGCACTTTGGTCACCCCAGCCAAGCTGTTTTTGATGAAGAGGCAGATAAGGCCAGTGAA
TTGAGGGACAGTGATTATAGCAATGAACAATTCTCCAAACTACCTATGTTTATAACGTCG
GGACTAGCTATGGATGGCCTCTTTGATGACAGTGAAGATGAAAGTGATAAACTATGCTAC
CCTTGGGATGGGACACAAGCCTATTCATTGTTTGATGTATCGCCTTCTTGCTCTTCTTTT
AATTCTCCATGCAGAGATTCATTGTCTCCACCCAAATCCTTATTTTCTCAAAGATCCCAA
AGGATGCGCTCTAGATCAAGGTCCTTTCCTCAACACAGGTCTTGTTCCCGTTCTCCATAT
TCCCGATCGAGATCAAGGTCGCCATGTAGCAGATCCTCTTCCAGATCTTGTTACTATTAT
GAGTCCAGCCACTGCAGACACCAAGCATACAGAAGTTCTCCTTTATATGCAAGATCACGA
TCCAGATCACCATATAGTTGTAGACCCAGATATGACAACTATGAAGAATATCAGCATGAA
AGACTGAAGAGGGAAGAATACCGCAAAGAGTATGAAAAACGGGAATCTGAAAGGGCCAAA
CAAAGGGAGAGGCAGAGACAAAAAGCAATTGAGGAACGTCGTGTGATTTTTGTGGGTAAA
ATCAGAGCTGATACCACCCGAACAGAACTGAGGGATCGGTTTGAAGTTTTTGGTGAAATT
GAAGAGTGCACAGTCAATCTGCGGGATGATGGAGACAGCTATGGTTTCATCACCTACCGC
TATACTTGTGATGCGTTTGCTGCTCTCGAAAATGGATACACTTTACGCAGGTCAAATGAA
CCTGACTTTGAGTTGTACTTTTGTGGACGCAAGCAATTTTGCAAGTCTAACTATGCAGAC
CTAGACTCAAACTCAGATGATTTTGATCCTGCTTCCACCAAGAGCAAATATGACTCCATG
GATTTTGATAGTTTACTTAAAGAGGCACAGCGAAGCCTGCGTAGG
>Cod_PPARGC1A
TTCTGCTTCCGTTTCGTCGTCACCGACGACCGATGGACGGACTTGCAGTGTGCTGCCTTG
GTGGGCGAGGACCAGCCCCTCTGTCCCGACCTCCCCGAACTCGACCTATCAGAGCTGGAC
GTCAGCGACCTCGACGCTGACAGCTTCCTGGGCGGGCTCAAGTGGTACAGCGACCAATCG
GATATCATTTCGACGCAGTACGGCAACGAAGCGTCCAATCTGTTTGAGAAGATAGATGAG
GAGAATGAGGCCAACCTGCTGGCGGTGCTCACAGAGACTCTGGACAGCATCCCGGTGGAC
GAGGACGGCCTGCCGTCCTTCGAGGCCCTGGCAGATGGGGAGGTGACCAATGCCAGCGAC
CGGAGCTGCCCCTCGTCACCGGAGGGCTCGCCGCGCAGCCCCGAGCCCGAGGAGCCGTCT
CTGCTGAAGAAGCTTCTTCTGGCTCCAGCAAACTCCCAGCTCAGCTATAATCAATACACA
GCTGTGAAGGCCCAGGACCATGCAGCCGGCACCAACCACCGCATCCGAACACCACCTGCT
GTCGTCAAGGTAGGAAATGACACAATTGCCATCGTACATATACTTATAAATATTTACGAG
CAGGTCCGTATGAGATGGCGCTTCTTTTTGTCTTTTCTTACTCCTAACTCTGATTCTCTA

ATTTCCATAATTGTTCCCTATTCTCTTCCCCCTACCCCCACTTACAAGCTCTCTCTCTCT
CCCTTTCTCTCTCCTCATCTGTCTCATCATCTCTCTCTCTCTCTCTCTCTCCCAGCCAAA
CCAACCACCTTGCCTCTTCCTTTGACCCCAGAGTCTCCAAATGACCACAAGGGATCACCG
TTTGAGAACAAAACCATTGAATGCACATTGAGTGTGGCGATCGCTGGAACCCCAGCATGG
CTCCGCCCACTTACACAATCACCCAATCACAGAACATCTCGAGATTATTTTCTCTCTCCC
GATGCTTTTCCCCCGTTTTCTTCTCTCTGGCCCAGTGATTCTTTTCAAAAGCATTCTGAG
CAGAGCAGCGGAGCGCGCACGAATAACCATCAGAGGTCGAAGGTCAGACCCAGCGAGGCG
CACGTCCAAACGGGCCGCCCAGGCTCAGCGGGAGATTACAAACGAGTGCATCGTGCCGGT
TCTCTCAATGTCAAACCTTTTTTTCACGAGATTCGTCGACAAATTAAAAAAAAGCTGAAG
CCCTTCCGAAAAATGACGGGGCACTCAGACACCTTTTTAGCTAGGAGCGATTCGGTCTCC
TGTACCACGGCCATGAAACAGATGGATCCCAGTAAAGGCCGGGATGAAAGCTTTATGGTC
CGAGGTGAGGCTTCCCTGAGCAGTCCCAGAGTAGCATTCCACTACAGAAGACATTATGAG
ACCCCCGCGACGCTAATAACCTGTGAGCTGTGCGTCCCTCACACCCCGTTGAAACGTGGG
TCCAGCGCCCGGGTACAGACGTTTTTATTTTGGGTCAACCGCAGCCTGTTTGGTCGGTGT
TCCCGATCAGAGGGGGGGGGGACGCGATCCCACCAACCACAACCACGACAGCAGCAGACCG
CCTCCCTCGGGGGATTTACCCGTTTATTTCCTTTTTTTTCGGGTGGGGGGCTTGTCGGCT
TTCCATTCAGGCGGCGCTGTCAATCCTCGGCCGCTGAAAAGCAGGAGCGATGAAAGCTAT
CGGAGCGGTTGTGCGGTCCCTCTACACTATCAACAACACACAGTCTGCTGTGTATATATA
TATATTGTGACGAAGCCACGCCCCGTCCCTGGCCAGTCGCTCACCGGTTCCTCCTCTTCT
TCTCTCCTTCTCCTCAGGTCCCGTCACCACGGCGACCAGTCCCGCTCCAGGTACCACCAG
GGCCCCCACGCCGGCTCCCGCTCCCCGTTGAGCCGCAGGCCCAGCAGGTACGACAGCTAT
GAGGACTACCAGCACGAGAGGCTGAAGCGGGAGGAGTATCGCCGTGACTACGAGAAGCGG
GAGTTTGAGCGCGCAGAACAGCGGGAGAAACAGAGACTCCCCCGGCGTTCCTCTGTGATG
AAGGCCCGCATACTTGCAGTGTTAGAGGGAGAAGGAAGGGGGGGGGGGGGGAGGAAGAGA
GAGAGAGAGAGAGGAGGGAGGGAGAGAGAGGAGGAGGAGAACGTTCTCGTTGTTCTATTC
CTGACCAGGGACAACTTTGGCTTCATCACCTACCGCTACACGTGTGACGCCTTCGCCGCC
CTGGAGAACGGACACACCCTGCGCCGCTCCGACGAACCGCAGTTCGAGCTGTGCTTCGGC
GGGCAGAAGCAGTTCTGCAAATCGGATTACACCGACTTGGACTCCCATTCAGACGACTTT
GACCCAGCCTCCACCAAGAGTAAGTACGACTCCCTGGACTTTGACAGCTTGCTGAGGGAG
GCCCAGTACAGCCTCAGAAGG
>Cow_PPARGC1A
ATGGCGTGGGACATGTGCAACCAGGACTCTGTATGGAGTGACATCGAGTGTGCTGCTCTG
GTTGGTGAAGACCAGCCTCTTTGCCCAGATCTTCCTGAACTTGACCTTTCTGAACTAGAC
GTGAACGACTTGGATACAGACAGCTTTCTGGGTGGACTCAAGTGGTGCAGTGACCAATCA
GAAATAATATCTAATCAGTACAACAACGAGCCTTCAAACATATTTGAGAAGATAGATGAA
GAGAATGAGGCAAACTTGCTAGCAGTCCTCACAGAGACACTGGACAGTCTCCCTGTGGAT
GAAGACGGATTGCCCTCATTTGATGCACTGACAGATGGAGATGTGACCACCGAGAATGAG
GCTAGTCCTTCCTCCATGCCTGACGGCACCCCTCCGCCTCAGGAGGCAGAAGAGCCGTCT
CTACTTAAGAAGCTCTTACTGGCACCAGCCAACACTCAGCTAAGTTATAATGAATGCAGT
GGCCTCAGTACCCAGAACCATGCAAACCATAATCACAGGATCAGAACAAACCCTGCAGTT
GTTAAGACCGAGAATTCATGGAGCAATAAAGCGAAGAGCATTTGTCAACAGCAAAAGCCA
CAAAGACGTCCGTGCTCAGAGCTTCTCAAGTATCTGACCACAAATGATGACCCTCCTCAC
ACCAAACCCACAGAGAACCGGAACAGCAGCAGAGACAAATGCACCTCCAAAAAGAAGGCC
CACACACAATCGCAGACACAACATCTACAAGCCAAACCAACAACTTTATCTCTTCCTCTG

ACCCCAGAGTCACCAAATGACCCCAAGGGTTCCCCATTTGAGAACAAGACTATTGAACGA
ACCTTAAGTGTGGAACTCTCTGGAACTGCAGGCCTAACTCCACCCACAACTCCTCCTCAT
AAAGCCAACCAAGATAACCCTTTCAGGGCTTCTCCAAAGCTGAAGCCCTCTTGCAAGACT
GTGGTACCTCCACCATCCAAGAAGGCCCGGTACAGTGAGTCTTCTTGTACCCAAGGCAGT
AATTCCACCAAGAAGGGGCCCGAGCAGTCTGAGTTGTACGCACAGCTCAGCAAGACCTCT
GTGCTCACCAGTGGACACGAGGAAAGGAAGGCCAAACGGCCCAGTCTCCGGCTGTTTGGT
GACCATGACTATTGTCAGTCAATTAATTCCAAAACGGAAATACTCGTTAGTACATCACAG
GAGCTCCATGACTCCAGACAACTAGAAAATAAAGATGCCCCCTCCTCCAACGGGCCGGGG
CAAATACACTCTTCCACAGATTCCGACCCGTGCTACCTGAGAGAGACCGCGGAGGTGAGC
AGGCAGGTCTCTCCCGGCAGCACCAGAAAACAGCTCCAAGACCAGGAAATCCGAGCTGAG
CTGAATAAGCACTTCGGTCATCCCAGTCAAGCTGTTTTTGACGACAAAGCAGACAAGACC
AGTGAACTGAGGGACAGTGATTTCAGTAATGAACAATTCTCCAAACTACCTATGTTTATA
AATTCAGGACTAGCCATGGATGGCCTGTTTGATGACAGCGAAGATGAAAGTGATAAACTG
AACTCCCCTTGGGATGGCACGCAGTCCTATTCATTGTTCGATGTGTCGCCTTCTTGTTCT
TCTTTTAACTCTCCGTGTAGAGATTCTGTGTCACCACCCAAATCTTTATTTTCTCAAAGA
CCCCAAAGGATGCGCTCTCGTTCAAGGTCCTTTTCTCGACACAGGTCGTGTTCTCGATCA
CCATATTCCAGGTCAAGATCAAGGTCCCCAGGCAGTAGATCTTCTTCAAGATCTTGCTAC
TACTATGAGTCAGGCCACTGCAGACACCGCACACACCGAAATTCGCCCCTGTGCGCGTCA
CGTTCAAGATCGCCCCATAGCCGGCGGCCCAGGTATGACAGCTACGAGGAGTACCAGCAC
GAAAGGCTCAAGAGGGAAGAATACCGCAGAGAGTATGAGAAGCGGGAATCCGAAAGGGCC
AAGCAGAGGGAGAGGCAGAGGCAGAAGGCAATTGAAGAGCGCCGTGTGATTTATGTTGGG
AAAATCAGGCCTGACACAACACGGACAGAACTGAGGGACCGTTTTGAAGTTTTTGGTGAA
ATTGAGGAGTGCACAGTAAATCTGCGGGATGATGGAGACAGCTATGGTTTCATTACCTAC
CGTTATACCTGTGATGCTTTTGCTGCTCTTGAAAATGGATACACTTTGCGCAGGTCGAAT
GAAACTGACTTCGAGCTGTACTTTTGTGGACGCAAGCAATTTTTCAAGTCTAACTATGCA
GACCTAGATTCAAATTCAGATGACTTTGACCCTGCTTGCATCAAGAGCAAGTATGACTCT
CTGGATTTCGATAGTTTACTGAAAGAAGCCCAGAGAAGCTTACGCAGGAATCTGGGTGGG
AGAGGATGCTGCAGGCACCGAATGCTCAACTTTCCTAACATTTTGAAGTTTCTG
>Dog_PPARGC1A
ATGGCGTGGGACATGTGCAACCAGGACTCTGTATGGAGTGACATCGAGTGTGCTGCTCTG
GTTGGTGAAGACCAGCCTCTTTGCCCAGATCTTCCTGAACTTGACCTTTCTGAACTAGAC
GTGAACGACTTGGATACAGACAGCTTTCTGGGTGGACTCAAGTGGTGCAGTGACCAATCA
GAAATAATATCCAATCAGTACAACAATGAGCCTTCAAACATATTTGAGAAGATAGATGAA
GAGAACGAGGCGAACTTGCTAGCCGTCCTCACAGAGACACTGGACAGTCTCCCCGTGGAT
GAAGACGGATTGCCCTCATTCGATGCACTGACAGATGGAGATGTGACCACTGAGAATGAG
GCCAGTCCTTCCTCCGTGCCTGACGGCACCCCTCCACCGCAGGAGGCCGAAGAGCCGTCT
CTACTTAAGAAGCTCTTACTGGCACCAGCCAACACTCAGCTAAGTTATAATGAATGCAGT
GGTCTCAGTACCCAGAACCATGCAAACCATAATCACAGGATCAGAACAAACCCTGCAGTT
GTTAAGACCGAGAATTCATGGAGCAATAAAGCGAAGAGCATTTGTCAACAGCAAAAGCCA
CAAAGACGTCCCTGCTCGGAGCTTCTCAAGTATCTGACCACAAATGATGACCCGCCTCAC
ACCAAACCCACAGAGAACAGGAACAGTAGCAGAGACAAATGCACCTCCAAAAAGAAGTCC
CACACACAATCGCAGTCTCAACATTTGCAAGCCAAACCAACAACTTTATCTCTTCCTCTG
ACCCCAGAGTCACCAAATGACCCCAAGGGTTCCCCATTTGAGAACAAGACTATTGAACGA
ACCTTAAGTGTGGAACTCTCTGGAACTGCAGGCCTAACTCCACCCACAACTCCTCCTCAT

AAAGCCAACCAAGATAACCCTTTCAGGACTTCTCCGAAGCTGAAGTCCTCTTGCAAGACT
GTGGTACCTCCGCCATCGAAGAAGCCCCGGTACAGTGAGCCTTCTGGCACCCAAGGCAAT
ACCTCCACCAAGAAAGGGCCCGAGCAGTCTGAGTTGTACGCACAACTTAGCAAGACCTCA
GTGCTCACCAGTGGACACGAGGAAAGGAAGGCCAAGCGGCCCAGCCTACGGCTGTTTGGT
GACCATGACTACTGTCAGTCAATTAATTCCAAAACGGAAATACTCATTAATATATCACAG
GAGCTCCAAGACTCTAGACAACTAGAATATAAAGATGCCTCCTCCGATTGGCAGGGGCAG
ATTTGTTCTTCCACAGATTCAGACCAGTGCTACCTGAGAGAGACTTCGGGGGCGGGCAAG
CAGGTCTCTCCTTGCAGCACCAGAAAACAGCTCCAAGACCAGGAAATCCGAGCCGAGCTG
AACAAGCACTTCGGTCATCCCAGTCAAGCTGTTTTTGACGACGAAGCAGACAAGACCAGT
GAACTGAGGAACAGTGATTTCAGTAATGAACAATTCTCCAAACTACCTATGTTTATCAAT
TCAGGACTAGCCATGGATGGCCTGTTTGATGACAGCGAAGATGAAAGTGATAAACTGAAC
TACCCTTGGGATGGCACGCAATCCTATTCATTGTTCGATGTGTCGCCTTCTTGCTCTTCC
TTTAACTCTCCATGTAGAGATTCCGTGTCACCACCCAAATCCTTATTTGCTCAAAGACCC
CAAAGGATGCGCTCGCGTTCAAGATCCTTTTCTCGACACAGGTCGTGTTCCCGATCACCA
TATTCCAGGTCAAGATCAAGGTCCCCAGGCAGTAGATCGTCTTCAAGATCTTGCTACCAC
TACGAGTCGAGCCACTGCAGACACCGCGCGCACCGAAATTCTCCCCTGTGTGCAAGATCA
CGTTCCAGGTCGCCCTACAGCCGTCGGCCCAGGTATGACAGCTACGAGGAGTATCAGCAC
GAGAGGCTGAAGAGGGAAGAGTACCGCAGAGAGTATGAGAAGCGGGAGTCTGAGAGGGCC
AAGCAAAGGGAGAGGCAGAGGCAGAAGGCAATTGAAGAACGCCGTGTGATTTATGTTGGT
AAAATCAGACCTGACACAACACGGACGGAACTGAGGGACCGTTTTGAAGTTTTTGGTGAA
ATTGAGGAGTGCACAGTAAATCTGCGGGATGATGGAGACAGCTATGGTTTCATTACCTAC
CGTTATACCTGTGATGCTTTTGCTGCTCTTGAAAATGGATACACTTTGCGCAGGTCGAAT
GAAACTGACTTCGAGCTGTACTTTTGTGGACGCAAGCAATTTTTCAAGTCTAACTATGCA
GACCTAGATTCAAACTCAGATGACTTTGACCCTGCTTCCACCAAGAGCAAGTATGACTCT
CTGGATTTCGATAGTTTACTGAAAGAAGCTCAGAGAAGCTTGCGCAGG
>Dolphin_PPARGC1A
ATGGCGTGGGACATGTGCAACCAGGACTCTGTATGGAGTGACATCGAGTGTGCTGCTCTG
GTTGGTGAAGACCAGCCTCTTTGCCCAGATCTTCCTGAACTTGACCTTTCTGAACTAGAC
GTGAACGACTTGGATACAGACAGCTTTCTGGGTGGACTCAAGTGGTGCAGTGACCAGTCA
GAAATAATATCCAATCAGTACAACAATGAGCCTTCAAACATATTTGAGAAGATAGATGAA
GAGAATGAGGCAAACTTGCTAGCAGTCCTCACAGAGACACTGGACAGTCTCCCTGTGGAT
GAAGACGGATTACCCTCATTTGATGCACTGACAGATGGAGATGTGACCACCGAGAATGAG
GCTAGTCCTTCCTCCATGCCTGATGGCACCCCTCCGCCTCAGGAGACAGAAGAGCCGTCT
CTGCTTAAGAAGCTCTTACTGGCACCAGCCAACACTCAGCTAAGTTATAATGAATGCAGT
GGCCTCAGTACCCACAACCATGCAAACCATAATCACAGGATCAGAACAAACCCTGCAGTT
GTTAAGACCGAGAATTCATGGAGCAATAAAGCGAAGAGCATTTGTCAACAGCAAAAGCCA
CAAAGGCGTCCCTGCTCGGAGCTTCTCAAGTATCTGACCACAAATGATGACCCTCCTCAC
ACCAAACCCACAGAGACCCGGAACAGCAGCAGAGACAAATGCACCTCCAAAAAGAAGGTC
CACACACAATCTCAGTCACAACATTTACAAGCCAAACCAACAACTTTATCTCTTCCTCTG
ACCCCAGAGTCACCAAATGACCCCAAGGGTTCCCCATTTGAGAACAAGACTATTGAACGA
ACCTTAAGTGTGGAACTCTCTGGAACTGCAGGCCTAACTCCACCCACAACTCCTCCTCAT
AAAGGCAACCAAGATAACCCTTTTAGGGCTTCTCCAAAGCTGAAGCCCTCTTGCAAGACT
GTGGTACCTCCGCCATCAAAGAAGGCCCGGTACAGTGAGTCTTCCGGTATCCAAGACAAT
AACTCCACCAGGAAAGGGCCCGAGCAGTCTGAGTTGTACGCACAGCTCAGCAAGACCTCC

GTGCTCACCAGTGGACACGAGGAAAGGAAGGCCAAGCGGCCCAGTCTGCGGCTGTTTGGT
GACCATGACTATTGTCAGTCAATTAATTCCAAAACGGAAATACTCATTAATATATCACAG
GAGCTCCACGACTCCAGACAACTAGAATATAAAGATGCCTCCTCCAACTGGCAGGGGCAG
ATGCGCTCTTCCACAGATTCAGACCAGTGCTACCCGAGAGAGGCCTCGGGGGTGAGCAGG
CAGGTCTCTCCCGGCAGCACCAGAAAACAGCTCCAAGACCAGGAAATCCGAGCTGAGCTG
AACAAGCACTTCGGTCATCCCAGTCAAGCTGTTTTTGACGACGAAGCAGACAAGACCAGT
GAACTGAGGGACAGTGATTTCAGTAATGAACAATTCTCCAAACTACCTATGTTTATAAAT
TCAGGACTAGCCATGGATGGCCTGTTTGATGACAGCGAGGATGAAAGTGATAAACTGAAG
TCCCCTTGGGATGGCACGCAGTCCTATTCATTGTTCCATGTGTCGCCTTCTTGTTCTTCT
TTTAACTCTCCATGTAGAGATTCCGTGTCACCACCCAAATCCTTATTTTCTCAAAGACCC
CAAAGGATGCGCTCTCGTTCACGGTCCTTTTCTCGACATAGGTCGTGTTCTCGATCACCA
TATTCCAGGTCAAGATCAAGGTCCCCAGGCAGTAGATCCTCTTCAAGATCTTGCTACTAC
TATGAGTCAGGCCACTGCAGACACCGCGCGCACCGAAATTCTCCCCTGTGCGCGAGATCA
CGTTCAAGNNNNNNNNNNNNNNNNNNNNNNNNNGTATGACAGCTACGAAGAGTATCAGCAC
GAGAGGCTGAAGAGGGAAGAATACCGCAGAGAGTATGAGAAACGGGAGTCTGAAAGGGCC
AAACAGAGAGAGAGGCAAAGGCAGAAGGCAATTGAAGAACGCCGTGTGATTTATGTTGGT
AAAATCAGACCTGACACAACACGGACAGAACTGAGGGACCGTTTTGAAGTTTTTGGTGAA
ATTGAGGAGTGCACAGTAAATCTGCGGGATGATGGAGACAGCTATGGTTTCATTACCTAC
CGTTATACCTGTGATGCTTTTGCTGCTCTTGAAAATGGATATACTTTGCGCAGGTCGAAT
GAAACTGACTTCGAGCTGTACTTTTGTGGACGCAAGCAGTTTTTCAAGTCTAACTATGCA
GACCTAGATTCAAACTCAGATGACTTTGACCCTGCTTCCACCAAGAGCAAGTATGACTCT
CTGGATTTCGATAGTTTACTGAAAGAAGCCCAGAGAAGCTTGCGCAGG
>Duck_PPARGC1A
ATGGCGTGGGACATGTGCAGCCAGGACTCTGCATGGAGTGACATCGAGTGTGCTGCTCTG
GTTGGCGAAGACCAGCCTCTTTGCCCAGATCTCCCAGAACTTGACCTCTCCGAACTAGAC
GTGAACGACCTGGACACGGACAGCTTTCTGGGGGGGCTCAAGTGGTACAGCGACCAGTCA
GAGATCATCTCCAACCAGTACAGCAATGAACCCGCCAATATATTCGAGAAAATAGATGAA
GAGAATGAGGCGAACTTGCTAGCAGTTCTCACTGAGACACTGGACAGCATCCCCGTGGAT
GAGGATGGATTGCCTTCATTTGATGCACTGACAGATGGAGATGTGACCAATGAAAATGAC
GCTAGCCCTTCCCCAATGCCCGACGGCACCCCTCCAACCCAGGAGGCAGAAGAGCCGTCT
CTACTTAAGAAGCTCTTGCTGGCTCCAGCCAACACTCAGCTAAATTACAATGAATGCAGT
GGTCTCAGCACACAAAACCATGCAAACACAAATCACAGGATCAGAACAAGCCCTGTGGTT
GTTAAGACTGAGAATTCATGGAGCAATAAAGCAAAGAGCATTTGTCAACAGCAAAAGCCA
CAAAGACGTCCCTGCTCTGAACTTCTCAAATATCTGACTACAAATGATGACCCTCCTCAG
ACCAAACCAGCAGAGAACAGGAACAGCAGCAAAGAGAAATGCACCTCCAAAAGGAAGCCC
CATCTGCAGTCTCAGACAAATCACCTGCAAGCCAAACCAACAAGTTTATCACTTCCGTTG
ACGCCCGAGTCACCAAATGATCCCAAGGGTTCCCCATTTGAGAACAAGACTATTGAACAA
ACCTTAAGTGTGGAACTCTCTGGAACTGCAGGCCTAACTCCACCTACAACCCCTCCTCAT
AAAGCCAACCAGGATAATCCTTTTAGGACTTCACCTAAGCCGAAGTCATCATGCAAGACT
GTTGTACCACCTTCAAAAAAGCCCCGCTATAGTGAGTCTTCTGGTTCTCAAGGAAACAAC
CCGATCAAGAAGGGTCCAGAACAGTCTGAGCTGTATGCACAGCTTAGCAAGACTACAGTA
CCGTCCAGTGGACATGAGGAGAGAAAGACAAAACGGCCCAGTTTGCGGCTGTTTGGTGAC
CATGACTACTGTCAATCTGTGAATTCAAAATCGGAAATACACATTAAAATATCCCAGGAA
CTTCAGGACTCCAGACAACTAGAATTTAAGGATTCTTCACCTGGGTGGCAGTGTCAGATT

TGTTCTTCTTTAGAACAAGACCAGTATTTCAAGAAAGAGACTTTACAGACAAGTCAGCAG
GGATCCCTCAGTAATAACAGAAAACAGCTCCAAGACCAGGAAATTCGGGCTGAACTGAAT
AAGCATTTTGGTCACCCCAGCCAAGCTGTTTTTGATGAAGAAGCAGATAAGACCAGTGAA
CTAAGGGACAGTGATTACAGTAATGAACAATTTTCCAAACTACCTATGTTTATAAATTCA
GGACTAGCAATGGATGGTCTCTTTGATGACAGTGAAGATGAAAGTGATAAACTATGCTAC
CCTTGGGATGGGACACAAGCCTATTCATTATTTGATGTATCGCCTTCTTGCTCTTCTTTT
AACTCTCCATGCAGAGATTCAGTGTCTCCACCCAAATCCTTATTTTCTCAAAGATCCCAA
AGGACACGCTCTAGATCAAGGTCCTTTCCTCAACGCAGGTCTTGTTCCCGTTCTCCATAT
TCCCGATCGAGATCAAGGTCGCCCTGTAGTAGATCCTCTTCAAGATCTTGTCACTGTTAT
GAGTCCAGCCACTGCAGACACCGAGCATACAGAAGTTCTCCCTTACGTGCAAGATCGCGA
TCCAGATCACCGTACAGTCGCAGACCCAGATATGACAGCTATGAGGAATATCAGCATGAA
AGGCTGAAGAGGGAAGAATACCGCAAAGAGTATGAAAAACGGGAATCTGAAAGGGCCAAA
CAAAGAGAGAGACAGAGGCAAAAAGCAATTGAAGAACGTCGTGTGATTTATGTGAGTAAA
ATCAGACCTGACACAACCCGAACAGAACTGAGGGACCGGTTTGAAGTTTTTGGTGAAATC
GAGGAGTGCACAGTAAATTTGCAGGATGATGGAGACAGCTATGGTTTCATCACCTACCGC
TACACTTGTGATGCCTTTGCTGCTCTTGAGAATGGATACACTTTACGCAGGTCAAATGAA
CCTGACTTTGAGCTGTACTTTTGTGGACGCAAGCAATTTTGCAAGTCTAACTATGCAGAC
CTAGATTCAAACTCAGATGATTTTGATCCTGCTTCCACTAAAAGCAAGTATGACTCCATG
GATTTTGATAGTTTACTTAAAGAGGCACAGCGGAGCCTGCGCAGG
>tropical_clawed_frog_PPARGC1A
ATGGCGTGGGACATGTGCAACCAGGACTCTGTATGGAGTGACATAGAGTGTGCTGCTCTG
GTCGGTGAAGACCAGCCTCTCTGCCCCGATCTTCCTGAACTTGATCTCTCTGAACTTGAT
GTCAATGACTTAGATGCAGACAGCTTTTTGGGTGGATTAAAGTGGTACAGTGACCAATCA
GAAAACATTTCCAATCAGTACAGCAGCGAATCGTCCAACATATTTGAGAAGATAGACGAG
GAGAATGAAGAGAATTTGCTAGCAGTTCTTACAGAGACATTGGACAGTCTCCCTGTGGAT
GAGGATGGATTGCCTTCATTTGATGCACTGACAGATGGAGATGTGACCAATGAACATGAT
CCTAGCCTTTCATCTATGCCTGACGGCACCCCTCCAACTCAGGAGGCAGAAGAGCCGTCA
CTACTTAAGAAGCTGTTGCTGGCCCCAGCTAACGCTCAGCTGATTTACAATGAATGCATT
GGGTTCACTACACAGAACCATGCCAGCCCCAGTCAGAGGATCAGAACTAATTCTGCGGTT
GTTAAGACCGAGAATTCATGGAGCAATAAACCGAAGACCATTTGTCACTCGCAAAAGCCA
CCGCGGCGTCCTTGCTCAGAGCTCCTTAAGTACCTGACTTCAAATGACGACCCTCCTCAG
ACCAAATCGACAGAGAGCAGGAACAGCAGGCTTGACAAATGCAGCAGCAAAAAGAAGCCG
TACTTACAGCCTCAGCCACATTATCAAGCCAGGGCAACGAGTTTGTCGCTTCCCTTGACG
CCCGAGTCACCAAATGATCCAAAAGAATGTCCTTTTATAAGGAAATTTTCTCAAGACCTT
CTGTTTGTGGAATTTTTTTTTGTTATAGGATTAACTCCACCTACAACACCTCCTCACAAA
GCCAATCAAGAGAACCCCTTCAGGACCTCACCTAAGCTGAAATCTTGCAAATCTTCTGTG
CCACCTGCTAAAAAATCTCGCTACATTGAGTCTTCCAGTATCCAAGTTCTCTATCCAGCT
AAGAAAGGTCCAGAGCAATCAGAACTGTACGCACAGCTCAGTAAAGCGACTGTGGTAATT
GGACAAGAGGAGAAAAAAGCTAAGCGACCTAGTTTACGACTTTTTGGAGACCACGACTAC
TGTCAGTTTATGAATTCAAAATCTGAAATACATATTAGCTTATCACAAGAATTACAGGCC
TCAAGACATCTTGAATGTAAGGATCCTTTGCCTGGCAAAGAACTGAAAGTCTGTTCATAC
ACTGATCAAGAGCAATGCCAGAAAGACAGCTTACCAGTCACAATGCCAAGTTCTCAGAAC
AGCCATAGGAAACAGCTCCAGGACCAGGAAATACGCGCTGAACTCAACAAGCACTTTGGG
CACCCAACGCAGGCAGTCTACAACGATGAAACTAAAATCAGTGAACTGGTGGACAATAAA

TATAGCGATGAACAGTTGTCAAGACTACCTATGTTTCTAACTGCTGGGTTGGAAATAGAT
AGTTTGTTTGATGACAGCGAGGACGAAAATGATAAACTGTGCTATACTTGGGATGAAACA
CAGTCATATTCATTATTTGATGAATCGCCGTCTTGCTCTACTTTTAATTCTCCGAGTAGA
TATTCAGTATCCCCACCAACATCCCTGTTTTCACAAAGGATATGCTCTAGATCAAGATCT
AGGTCCTTTTCACAGTTTAGATCGTCTTCTCTTTCCCCATATTCTCGTTCAAGATCCAGA
TCTCCATCAAGCAGATCATCATCAGGGTCTTGCTGCTGCTATGAGACTGATCATTGCAGA
GAAAAAACTTCTCCAATGTATGCACGATCACGGTCAAGGTCGCCATATGGTCGTAGGCCC
AGATATGACAGCTATGAGGAATACCAACATGAACGGCTGAAGAGGGAGGAGTACCGCAAG
GAGTATGAAAAGCGGGAATCAGAGAGAGCAAAACAAAGAGAGAGACAAAGGCAAAAAGCC
ATTGAAGAACATAGAGTGATATTCGTGGGTAAAATGAGACCTAACATGAGCCGCGCAGAG
CTGCGTGCCCGGTTTGAAGTTTTTGGAGAAATTGAGGAATGCACAGTAAATCTGCGGGAC
GATGGCAATTGTTACGGATTCATCACCTATCGCTACACCTGTGACGCTTTTGCTGCTCTT
GAAAATGGATACACGCTGCGCAGGTCAAATGAGCCAGACTTCGAGGTTTGCTTTTGTGAA
AGAAAGCAGGTTTGCAAGTCCAACTATGCAGACTTAGATTCAAATTCAGATGACTTTGAT
CCTGCTTCCACCAAAAGCAAATATGACTCCATGGATTTTGACAGTTTGCTCAAGGAGGCA
CAGAGAAGCCTGCGTAGG
>Fruitfly_PPARGC1A
ATGGATTCACGAATGCTAAATGTGTTCCAAGGAGATACCTTTGAGGCAAATTATACCAAA
TACGAGTCTGAGCCGATATTCTGGTCATCTAATGATGAACTGCAACTGACAGATACACTG
AGCGGCATCCTACCCATCGCGCCCACAGACGAATCACTGCTCAACCATAACCGGGAGGAA
GATCTGCAAAACAGAAAGAATCTGCTGATTTACAACGACTTTCTGAAACAGGAGCTCAGC
AACCATGATGCCAATGAGCTAAATGCCCAGGTCTATCGGTCCATCGCCGACAGCGGGGGC
GAGATTGCCTATGAGCAGCAGCGCCAGATCAGCGCCATTGTGGAGTACGACAAAGAAGAT
ATTAAAATCGACGGCCTTACGGAGGAGGAGGACATACAAAGCCAGTCCACATTTCGCACC
CGCACAGACAGCTCTTCGATTGGTGACTATGAATCGCACTCTAGCGACTATGATGATGAA
TTTGACATTGTCAACGAAGAAAAAAGTCCAAGGCTGCAGGTTGTCCATAACAATATCGGC
AACTTGCAGAGCTCCTGTGAGCGCAAGGGACGGACGCTCAGTACTAACACTATTATCTCC
GAAGTAGACGCATTGGGGCTAAGTATTGATGTTAATAACTTTGACCTAGCTGATTTTATT
ACCAAAGACGATTTTGCTGAGAATTTAAACGCCTGTCGCAAAAAGGAATCTCTGCAAGCA
CAGGCGCAAATTAAATCTAATACTTTAGCAGATGTTCCCATACTTCTTAATCCACCGGTT
GCCACAATTGTGGCAAAACCCGGGGTCCCATCCTCTAAACCTGGCGATTCTGATTATGAC
TCTGACTCGATTATCGACGTTGAAACTGTCGATGTACTCGATATGAATATGGCCAATATC
TGGTCAATAGATAAGCCGGCAAAAATGGGCCAAACTCCCGATGAATTACGCTATGTGGAC
AATGTAAAGGCCGATCCCTCTTGGAGTCCGAGATCCGCAAAAAAGACCACACCTACTATC
AAAGAGAATAAGCCGGAACAACTGGGGAACCATCAGCTCGCGGTCCCAGCATCAAACAAA
CCGAAGCATAAGAATATTTGCCTGGTGCCTAATAAAAAACAGTGCGATTTTCTTAAACGT
AAGGTTGGTAGTCCCTCCTTGTCCAAGCTAACTAAGGCGACAACAAAAAAAGCGACTGAG
TCTATGAGCAACAAGCAGCCGTCAGTAAAAAATACTCAATGCACGGGTCTCGGATTGGGT
GGAAAAAACCTTTCGCTTCTGAAGCAGGAGAGGGACGCCGCATTATCGGTGTGTGCTGCA
AAACCTATAGCACCTCCAACCCTTTCGACAACAACCCCGACTTTGACACCGCCAGCAAAG
CGAAAGCTAAATCTAGAGGAATATAAACAGCGTCGTTGTGGCGGTGTGGGGGCTGTGTAT
CCTAAACCAACTCCGCCCAAGCAGGCGAAAATAGAGGTTGCCGCCAAACGCATTGCTCCT
ACCCCGGTTTCAAAACCAACACCTTCACCAAAAAAAGCGGAAATAGTCAATATTGTAAAC
AGCCTTAAATGTCCTCGCACAGAACAATCTTCTTTGCCCATGGATCCCATTACCATGGCC

AAGAACAAGGTGCTGCGTATGCTAGAGATGAAACGTGCCCAACAGCTAAAAATAATCGAT
TCCCGCGTATCAGCCAAGGTGCCACGCGTAACCAAGCTGCCGCCACTTAAGGATATTGTA
AAGGACACCTACTGCATGGAAACTGAGGATCCTGGCACAGAGATAGCGCCACTCAGCAAC
AAACTCCACCCGGACTATGAGGAGATTATAATAGTTTCAGCTAGTTGCAATACAGACATA
ACAATACCTCCCAATCAATTAAGCAAGGCCTCCCCTCGCTCTCTCTTAAAGTCATCAGTT
TTGCTGTACAACATCTCAAACGGCCAGGATGCCAATAAGAACATGAGCAACTCCCTAATA
GCCAGCATCCAAAGCGAGGTCGCCAGGCAAACGAGTAACACTACTCTCTCGACGATACAG
TCCAACGCAACAAAAGTTATGTCTTCCGCTGATAAGAATTGTCAGCATGGCGAGGACATG
GTCATCATGCACTTACCCAAGGATCGGGTGCGAAAAACTCTTGTGAGCATTGCAACCCAA
ACTGATCTGCAACCCGAATTTCCCCTATTGACGCTGCCCCCAAAGCGACAGTCTCGAGAG
CGTACGCGTCGAAATTATCGCAGGCGACGGACACAAGGCTCGGGCTCAAATATGTCCACT
TCTTCGAGTGACTTTTCCTCCGACTGCAGCAGCTTGGTGTCACACAGATCTCGCTCACAA
TACGATTCGATTGATCAGCTGCGTAACTTAGATGTAGCAGCCACGTGTGGGGGCAGTATT
GGAAACGGCGGTTATTCTTCTCGTAGCACTCAACGCCATCGCAGCTCGGTGAGTTCTTCA
TCTTACTCAGAGAATGGACAGTACAGGCGCAGGCAGCGCCGCACGAGTTACAACAAGCGT
AGATCTAGAAACCAAAAGCGGGGCAGTACTTCCTCGTCATGCTCGGGCTCCGAAAAAAGC
GATCGCGAACGTAGCCGCAGCCCACACAGAAAATTGCGCCACTCGCGGTCCCGGTCGCGC
TCAAAATCTGATACACGCTACCCTAACAACAACTCCTCCTCCAATAACAACAATCGGCGA
GGATTTTTTGATCGTAACGTGTCGCAACCGGCCGTTGAAGAGCGGCGAATCGTCTACGTC
GGTCGCATTGAGCAAGAAACCACCAAGGAAATTCTGCGTCGTAAATTTCTGCCCTATGGA
AGCATTAAACAAATAACAATACACTACAAGGAAAATGGAATGAAGTATGGGTTCGTTACG
TACGAAAGAGCGCAGGACGCATTCACGGCCATTGACACGAGTCATAGAGACTCGCAAATA
AGCATGTATGACATAAGTTTTGGAGGAAGACGTGCCTTCTGTCGTTCATCTTACGCTGAC
TTGGATAATGCTGGCATTAATAATTACAACTCGTATGTGTTTCCCAAGGAAGCACCAGCA
CCGAATGTAGTCGAGGACTCTTTCGAAGCTTTGTTGCTTCAGGTAAAAGCAAAGTTAAAC
GCGGGCAAGTCACCGGTAGGTTCATCTACGCTGGAGGCACCATCAGCTTCCGGTACTGTA
CTTCAGGGCCACAGTCAGATG
>Fugu_PPARGC1A
AAGAAGATAGATGAAGAAAATGAGGCCAACTTGCTGGCAGTGCTTACAGAGACCCTGGAC
AGCATCCCGGTGGATGAGGACGGATTGCCTTCGTTTGAGGCCCTGGCAGACGGGGACGTG
ACCAATGCCAGTGACAGGAGCTGTCCCTCGTCCCCCGACGGCTCGCCGCGCACCCCCGAG
CCCGAGGAGCCGTCCCTGCTGAAGAAGCTCCTTCTGGCACCCGCAAACTCCCAGCTCAGC
TATAATCAATACACAGGTGGCCAAGCACAGAACCATGCAGCCAGCAGCAACCACCGAATC
AGACCACCACCTGCCGTCGTCAAGATGGAGAGCACCTGGAATGGCAAAGCAAGAGGAGGT
TCCAGCCAGCAGAACCGCCCAGTGAGGCGACATTGCACCGAGTTGCTGAAATACCTGACA
GCCACCGATGACATCCTGCTGCACGCCAAAAATAACTCCTCCACCTCATCTTCCTCCTCC
TCCAGCGCCTCCAAGAAGAAGTCGGCTGCATCGTCTCAGCAGCAGCAGCAGCCGCCCAAA
CCAACCACCTTGCCACTTCCTTTGACCCCAGAGTCTCCAAATGACCACAAGGGATCACCG
TTTGAGAACAAAACCATTGAACGCACATTGAGTGTGGAGATAGCTGGAACCCCTGGTTTG
ACACCACCTACCACGCCCCCACACAAAGCCAGTCAAGAGAATCCTTTCAAAGCATCGCTC
AAAACCAAGTTGTCTTCATGTTCCTCCTCAGCCTCTGTGTGCAAAAGAGCCAGGCTGAGC
GAGTCCGGCCCCGGCGCTCTGGCCCCGGCCCCGGGTGCCTCGGGCGGGGGCCCCATTAGG
AAGGGTCCAGAACAGACTGAGCTCTATGCCCAGCTGAGCAAAGCTTCCACCGCCCTACCT
TATTGCATCACCCTTCGCGCGGCCGGGGGCGGCCCCGAGGAGCGTCGTAGCACTGGCAAC

AATAGGCGGGCGGCGCCCCGCGGCTATAGTGACCACGACTATTGCCAGGCATCGGCCAAA
AAGGACAGCAGCGCCGACACTGTTACCATGACTACAGCCGAGCAGCAGGATCTGTCCTCT
GTGGATGGAAAAGCAGCACCTGCGCGGGGGTTCGGCTCCGCCCGGACCCCCTCAGGAGAT
GACGAGGACCAACTGCAGACTTGTGCCATCAGCCGGAAGCTCCTGCGTGACAACCAAATC
AGAGCAGAACTCAATAAGCACTTTGGCCACCCTTTGCAAGCCCTCTACAGCCAGGCCAGC
CAGGGCAGAGAGCCCCCCAGCCATGCAAATCAGGCCGCGGCCGCTCCGTCCCTGGAGGGG
GGCTTCCTGCCCTTCCATGAAGAGCTGGAGCTGACCCAGGGCCGCGTGAGCCGCTTCCTG
TACCCGTGGGAGGGCACCCCGCTGGACCTCCTCTTTGACTGCCCTCCATGCTCCCCCTCC
TGCTCTCCACCCTCCAGCTGCTCCCCCTCGCGAGGCTCCGTCTCCCCACCTTCCTCCCTG
CTCCTCTCCCCCAGCAGGCCTCTCTGCTGGACCAGCGCCGGCTCCCGCTCCCGCTCTCGT
TCCCGCTCCGGCTCCCGCAGCTCCTCCTCGCACTACCGGAGACGCTCCCTCTCTTCTTCG
CCCGACCGACGCCCCTCCTCTTGGTCTCGTCACAACCAAGACTCCAGCACTTTTCGTTCC
CGGACCCAGAAGAGCCCCCGCCCTCAGCCTCGCTCCCCCCTCAGCCGCAGGCCAAGGTAT
GACAGTTATGAGGAGTACCAGCACGAGAGGCTGAAGAGGGAGGAGTACCGCCGGGACTAC
GAGAAGCGGGAGTCTGAGAGGGCCGAGCAGAGGGAGAAGCAGCGGCAAAAAGCAATAGAG
GAGAGACGGGTCGTGTACGTGGGGCGTCTGAGGTCCGACTGCACGCGCACGGAGCTGAAG
CGCCGCTTTGAAGTCTTTGGCGAAATTGAAGAATGTGCGGTGAACCTGAGGGACGACGGG
GACAATTTTGGCTTCATCACGTACCGCTACACCTGCGACGCCTTCGCCGCCCTGGAGAAC
GGACACACCTTACGCCGGTCAAACGAGCCTCAGTTCGAGCTGTGCTTCGGCGGACAAAAG
CAGTTCTGCAAATCCCATTACACAGACTTGGACTCCCACTCGGACGACTTTGATCCGGCC
TCCACCAAGAGCAAGTACGACTCCCTGGACTTTGACAGCTTGCTGCGGGAGGCCCAGCGC
AGCCTCAGAAGG
>Horse_PPARGC1A
ATGGCGTGGGACATGTGCAACCAGGACTCTGTATGGAGTGACATCGAGTGTGCTGCTCTG
GTTGGTGAAGACCAGCCTCTTTGCCCAGATCTTCCCGAACTTGACCTTTCCGAACTAGAC
GTGAATGACTTGGATACAGACGGCTTTCTGGGTGGACTCAAGTGGTGCAGTGACCAATCA
GAAATCATATCCAATCAGTACAACAATGAGCCTTCTAACATATTTGAGAAGATAGATGAA
GAGAATGAGGCAAACTTGCTAGCAGTCCTCACAGAGACACTGGACAGTCTCCCTGTGGAT
GAAGACGGATTGCCCTCATTTGATGCGCTGACAGATGGAGATGTGACCACTGAGAATGAG
GCTAGTCCTTCCTCCATGCCTGACGGCACCCCTCCGCCTCAGGAGGCAGAAGAGCCGTCT
CTACTTAAGAAGCTCTTACTGGCACCGGCCAACACTCAGCTAAGTTATAATGAATGCAGT
GGTCTCAGTACCCAGAACCATGCAAACCACAATCACAGGATCAGAACAAACCCTGCAGTT
GTTAAGACCGAGAATTCTTGGAGCAATAAAGCGAAGAGCATTTGTCAACAGCAAAAGCCA
CAGAGACGTCCCTGCTCGGAGCTTCTCAAGTATCTGACCACAAATGATGACCCTCCTCAC
ACCAAACCCACAGAGAACAGGAACAGCAGCAGAGACAAATGCACCTCCAAAAAGAAGTCC
CACACACAATCGCAGTCACAACATTTACAAGCCAAACCAACAACTTTGTCTCTTCCTCTG
ACCCCAGAGTCACCAAATGACCCCAAGGGTTCCCCATTTGAGAACAAGACTATTGAACGA
ACCTTAAGTGTGGAACTCTCTGGAACTGCAGGCTTAACTCCACCCACAACTCCTCCTCAC
AAAGCCAACCAAGATAACCCTTTCAGGGCTTCTCCAAAGCTGAAGTCCTCTTGCAAGACT
GTGGTCCCTCCGCCATCAAAGAAGCCCCGGTACAGTGAGCCTTCTGGTACCCAAGGCAAT
AACTCCACCAAGAAAGGGCCCGAGCAGTCTGAGTTGTACGCACAACTCAGCAAGTCCTCA
GTGCTCACCAGTGGACACGAGGAAAGGAAGGCCAAGCGGCCCAGTCTACGGCTGTTTGGT
GACCATGACTATTGTCAGTCAATTAATTCCAAAACGGAAATACTCATTAATATATCACAG
GAGCTCCAAGACTCTAGACAACTAGAATATAAAGATGCCTCCTCCAATTGGCAGGGGCAG

ATTTGTTCTTCTACAGATTCAGACCAGTGCTACCTGAGAGAGACTTTGGAGGGGAGCAAA
CAGGTTTCTCCTTGCAGCACCAGAAAACAGCTCCAAGACCAGGAAATCCGAGCTGAGCTG
AACAAGCACTTCGGTCATCCCAGTCGAGCTGTTTTTGACGACGAAGCAGACAAGACCAGT
GAACTGAGGGACAGTGATTTCAGTAATGAACAATTCTCCAAACTACCTATGTTTATAAAT
TCAGGACTAGCCATGGATGGCCTGTTCGATGACAGCGAAGATGAAAGTGATAAACTGAAC
TACCCTTGGGATGGCACGCAATCCTATTCATTGTTCGATGTGTCGCCTTCTTGTTCGTCT
TTTAACTCTCCATGTAGAGATTCCGTGTCACCACCCAAATCCTTATTTTCTCAAAGACCC
CAAAGGATGCGCTCTCGTTCAAGGTCCTTTTCTCGACACAGGTCGTGTTCCCGATCACCA
TATTCCAGGTCAAGATCAAGGTCCCCAGGCAGTAGATCCTCTTCAAGATCTTGCTACTAC
CATGAGTCAAGCCACTGCAGGCACCGCACACACCGAAATTCTCCCCTGTGCGCGAGATCA
CGTTCAAGATCGCCCTACAGCCGTAGACCCAGGTATGACAGCTACGAGGAATATCAACAT
GAGAGGCTGAAGAGGGAAGAGTACCGCAAAGAGTATGAGAAGCGAGAGTCTGAAAGGGCC
AAACAAAGGGAGAGGCAGAGGCAGAAGGCAATTGAAGAACGTCGTGTGATTTATGTTGGT
AAAATCAGACCTGACACGACACGGACAGAACTGAGGGACCGTTTTGAAGTTTTTGGTGAA
ATTGAAGAGTGCACAGTAAATCTGCGGGATGATGGAGACAGCTATGGTTTCATTACCTAC
CGTTACACCTGTGATGCTTTTGCTGCCCTTGAAAATGGATACACTTTGCGCAGGTCGAAT
GAAACTGACTTCGAGCTGTACTTTTGTGGACGCAAGCAATTTTTCAAGTCTAACTATGCA
GACCTAGATTCAAACTCAGATGACTTTGACCCTGCTTCCACCAAGAGCAAGTATGACTCT
CTGGATTTCGATAGTTTACTGAAAGAAGCTCAGAGAAGCTTGCGCAGG
>Human_PPARGC1A
ATGGCGTGGGACATGTGCAACCAGGACTCTGAGTCTGTATGGAGTGACATCGAGTGTGCT
GCTCTGGTTGGTGAAGACCAGCCTCTTTGCCCAGATCTTCCTGAACTTGATCTTTCTGAA
CTAGATGTGAACGACTTGGATACAGACAGCTTTCTGGGTGGACTCAAGTGGTGCAGTGAC
CAATCAGAAATAATATCCAATCAGTACAACAATGAGCCTTCAAACATATTTGAGAAGATA
GATGAAGAGAATGAGGCAAACTTGCTAGCAGTCCTCACAGAGACACTAGACAGTCTCCCT
GTGGATGAAGACGGATTGCCCTCATTTGATGCGCTGACAGATGGAGACGTGACCACTGAC
AATGAGGCTAGTCCTTCCTCCATGCCTGACGGCACCCCTCCACCCCAGGAGGCAGAAGAG
CCGTCTCTACTTAAGAAGCTCTTACTGGCACCAGCCAACACTCAGCTAAGTTATAATGAA
TGCAGTGGTCTCAGTACCCAGAACCATGCAAATCACAATCACAGGATCAGAACAAACCCT
GCAATTGTTAAGACTGAGAATTCATGGAGCAATAAAGCGAAGAGTATTTGTCAACAGCAA
AAGCCACAAAGACGTCCCTGCTCGGAGCTTCTCAAATATCTGACCACAAACGATGACCCT
CCTCACACCAAACCCACAGAGAACAGAAACAGCAGCAGAGACAAATGCACCTCCAAAAAG
AAGTCCCACACACAGTCGCAGTCACAACACTTACAAGCCAAACCAACAACTTTATCTCTT
CCTCTGACCCCAGAGTCACCAAATGACCCCAAGGGTTCCCCATTTGAGAACAAGACTATT
GAACGCACCTTAAGTGTGGAACTCTCTGGAACTGCAGGCCTAACTCCACCCACCACTCCT
CCTCATAAAGCCAACCAAGATAACCCTTTTAGGGCTTCTCCAAAGCTGAAGTCCTCTTGC
AAGACTGTGGTGCCACCACCATCAAAGAAGCCCAGGTACAGTGAGTCTTCTGGTACACAA
GGCAATAACTCCACCAAGAAAGGGCCGGAGCAATCCGAGTTGTATGCACAACTCAGCAAG
TCCTCAGTCCTCACTGGTGGACACGAGGAAAGGAAGACCAAGCGGCCCAGTCTGCGGCTG
TTTGGTGACCATGACTATTGCCAGTCAATTAATTCCAAAACAGAAATACTCATTAATATA
TCACAGGAGCTCCAAGACTCTAGACAACTAGAAAATAAAGATGTCTCCTCTGATTGGCAG
GGGCAGATTTGTTCTTCCACAGATTCAGACCAGTGCTACCTGAGAGAGACTTTGGAGGCA
AGCAAGCAGGTCTCTCCTTGCAGCACAAGAAAACAGCTCCAAGACCAGGAAATCCGAGCC
GAGCTGAACAAGCACTTCGGTCATCCCAGTCAAGCTGTTTTTGACGACGAAGCAGACAAG

ACCGGTGAACTGAGGGACAGTGATTTCAGTAATGAACAATTCTCCAAACTACCTATGTTT
ATAAATTCAGGACTAGCCATGGATGGCCTGTTTGATGACAGCGAAGATGAAAGTGATAAA
CTGAGCTACCCTTGGGATGGCACGCAATCCTATTCATTGTTCAATGTGTCTCCTTCTTGT
TCTTCTTTTAACTCTCCATGTAGAGATTCTGTGTCACCACCCAAATCCTTATTTTCTCAA
AGACCCCAAAGGATGCGCTCTCGTTCAAGGTCCTTTTCTCGACACAGGTCGTGTTCCCGA
TCACCATATTCCAGGTCAAGATCAAGGTCTCCAGGCAGTAGATCCTCTTCAAGATCCTGC
TATTACTATGAGTCAAGCCACTACAGACACCGCACGCACCGAAATTCTCCCTTGTATGTG
AGATCACGTTCAAGATCGCCCTACAGCCGTCGGCCCAGGTATGACAGCTACGAGGAATAT
CAGCACGAGAGGCTGAAGAGGGAAGAATATCGCAGAGAGTATGAGAAGCGAGAGTCTGAG
AGGGCCAAGCAAAGGGAGAGGCAGAGGCAGAAGGCAATTGAAGAGCGCCGTGTGATTTAT
GTCGGTAAAATCAGACCTGACACAACACGGACAGAACTGAGGGACCGTTTTGAAGTTTTT
GGTGAAATTGAGGAGTGCACAGTAAATCTGCGGGATGATGGAGACAGCTATGGTTTCATT
ACCTACCGTTATACCTGTGATGCTTTTGCTGCTCTTGAAAATGGATACACTTTGCGCAGG
TCAAACGAAACTGACTTTGAGCTGTACTTTTGTGGACGCAAGCAATTTTTCAAGTCTAAC
TATGCAGACCTAGATTCAAACTCAGATGACTTTGACCCTGCTTCCACCAAGAGCAAGTAT
GACTCTCTGGATTTTGATAGTTTACTGAAAGAAGCTCAGAGAAGCTTGCGCAGG
>Macaque_PPARGC1A
ATGGCGTGGGACATGTGCAACCAGGACTCTGAGTCTGTATGGAGTGACATCGAGTGTGCT
GCTCTGGTTGGTGAAGACCAGCCTCTTTGTCCAGATCTTCCTGAACTTGATCTTTCTGAA
CTAGATGTGAACGACTTGGATACAGACAGCTTTTTGGGTGGACTCAAGTGGTGCAGTGAC
CAATCAGAAATAATATCCAATCAGTACAACAATGAGCCTTCAAACATATTTGAGAAGATA
GATGAAGAGAATGAGGCAAACTTGCTAGCAGTCCTCACAGAGACACTGGACAGTCTCCCT
GTGGATGAAGACGGATTGCCCTCATTTGATGCGCTGACAGATGGAGACGTGACCACTGAC
AATGAGGCTAGTCCTTCCTCCATGCCTGACGGCACCCCTCCACCCCAGGAGGCAGAAGAG
CCGTCTCTACTTAAGAAGCTCTTACTGGCACCGGCCAACACTCAGCTAAGTTATAATGAA
TGCAGTGGTCTCAGTACCCAGAACCATGCAAATCACAATCACAGGATCAGAACAAACCCT
GCAATTGTTAAGACTGAGAATTCATGGAGCAATAAAGCGAAGAGTATTTGTCAACAGCAA
AAGCCACAAAGACGTCCCTGCTCGGAGCTTCTCAAATATCTGACCACAAACGATGACCCT
CCTCACACCAAACCCACAGAGAACAGAAACAGCAGCAGAGACAAATGCACCTCCAAAAAG
AAGTCCCACACACAATCGCAGTCTCAACATTTACAAGCCAAACCAACAACTTTATCTCTT
CCTCTGACCCCAGAGTCACCAAATGACCCCAAGGGTTCCCCATTTGAGAACAAGACTATT
GAACGCACCTTAAGTGTGGAACTCTCTGGAACTGCAGGCCTAACTCCACCCACCACTCCT
CCTCATAAAGCCAACCAAGATAACCCTTTTAGGGCTTCTCCAAAGCCGAAGTCCTCTTGC
AAGACTGTGGTGCCACCACCATCAAAGAAGCCCCGGTACAGCGAGTCTTCTGGTACACAA
GGCAATAACTCCACCAAGAAAGGGCCGGAGCAATCCGAGTTGTATGCACAACTCAGCAAG
TCCACAGTGCTCACTGGTGGACACGAGGAAAGGAAGACCAAGCGGCACAGTCTACGGCTG
TTTGGTGACCATGACTATTGCCAGTCAATTAATTCCAAAACGGAAATACTCATTCATATA
TCACAGGAGCTCCAAGACTCTAGACAACTAGAAAATAAAGATGTCTCCTCTGATTGGCAG
GGGCAGATTTGTTCTTCCACAGATTCAGACCAGTGCTACCTGAGAGAGACTTCGGAGGCA
AGCAAGCAGGTCTCTCCTTGCAGCACAAGAAAACAGCTCCAAGACCAGGAAATCCGAGCC
GAGCTGAACAAGCACTTCGGTCATCCCAGTCAAGCTGTTTTTGACGATGAAGCAGACAAG
ACCAGTGAACTGAGGGACAGTGATTTCAGTAATGAACAATTCTCCAAACTACCTATGTTT
ATAAATTCAGGACTAGCCATGGATGGCCTGTTTGATGACAGCGAAGATGAAAGTGATAAA
CTGAGCTACCCTTGGGATGGCACGCAATCCTATTCATTGTTCAATGTGTCTCCTTCTTGC

TCTTCTTTTAACTCTCCATGTAGAGATTCCGTGTCACCACCCAAATCCTTATTTTCTCAA
AGACCCCAAAGGATGCGCTCTCGTTCAAGGTCCTTTTCTCGACACAGGTCGTGTTCCCGA
TCACCATATTCCAGGTCAAGATCAAGGTCCCCAGGCAGTAGATCCTCTTCAAGATCCTGC
TATTACTATGAGTCAAGCCACTACAGACACCGCACGCACCGAAATTCTCCCTTGTATGTG
AGATCACGTTCAAGATCGCCCTACAGCCGTCGGCCCAGGTATGACAGCTACGAGGAGTAT
CAGCACGAGAGGCTGAAGAGGGAAGAATATCGCAGAGAGTATGAGAAGCGAGAGTCTGAG
AGGGCCAAACAAAGGGAGAGGCAGAGGCAGAAGGCAATTGAAGAGCGCCGTGTGATTTAT
GTTGGTAAAATCAGACCTGACACAACACGGACAGAACTGAGGGACCGTTTTGAAGTTTTT
GGTGAAATTGAGGAGTGCACAGTAAATCTGCGGGATGATGGAGACAGCTATGGTTTCATT
ACCTACCGTTATACCTGTGATGCTTTTGCTGCTCTTGAAAATGGATACACTTTGCGCAGG
TCGAACGAAACTGACTTCGAGCTGTACTTTTGTGGACGCAAGCAATTTTTCAAGTCTAAC
TATGCAGACCTAGATTCAAACTCAGATGACTTTGACCCTGCTTCCACCAAGAGCAAGTAT
GACTCTCTGGATTTTGATAGTTTACTGAAAGAAGCTCAGAGAAGCTTGCGCAGG
>Medaka_PPARGC1A
ATGGCGTGGGACAGGTGTAACCAAGACTCGGTGTGGACAGAATTAGAGTGTGCTGCCTTG
GTTGGTGAAGATCAGCCCCTCTGCCCGGACCTCCCTGAACTTGACCTCTCAGAGCTGGAC
GTCAGTGATTTAGATGCAGACAGCTTCCTGGGCGGCCTCAAATGGTACAGTGACCAATCA
GAGATCGTTTCCACTCAGTATGGGAATGAAGCATCGAATCTTTTTGAGAAGATAGATGAA
GAAAATGAGGCCAACTTGCTGGCAGTGCTCACAGAGACCCTGGACAGCATCCCGGTGGAT
GAGGACGGATTGCCTTCATTTGAGGCCCTGGCAGATGGGGACGTGACCAATGCCAGTGAT
CGGAGCTGTCCCTCCTCTCCCGACGGCTCGCCACGCACCCCGGAGCCCGAGGAGCCTTCC
TTGCTGAAGAAGCTCCTTCTGGCACCTGCAAACTCCCAGCTCAGCTATAATCAATACACA
GGTGGCAAGGCACAGAACCATGCAGCCAGCAGCAACCATCGGATCAGACCACCACCTGCC
GTCGTCAAGACGGAGAGCCCCTGGAATGGCAAGGCAAGAGGGGGCTCCAGCCAACAGAAC
CGCCCGGTGAGGCGGCCTTGCACTGAGCTGCTAAAATACCTAACAGCCACTGATGACATC
CTACTCCACACCAAAGGCAGTGAACCTAAGAGCGCCTGGGGAGGTGCTAGTAGCAAAGAC
AAGAGCGGATTGGCTCTGCCATCTCAGCAGCCACTGCAGCAGCATCACCAGCGAGCCAAA
CCAACCACCTTGCCACTTCCTTTGACCCCAGAGTCTCCAAATGACTACAAGGGATCACCT
TTTGAGAACAAAACCATTGAACGCACATTAAGTGTGGAGATTGCTGGAACCCCAGGTCTG
ACACCACCAACCACGCCCCCACACAAAGCCAGTCAAGAGAATCCTTTCAAAGCATCTCTC
AAAACCAAGTTGTCCTCATGTTCCTCCTCGGCCTTGACATGCAAAAGAAGCAGGTTGAGT
GAGTTAGGCGCCTGCGTTGTGGCCCTGGCCCCGGGTGCCTCAGCTGGGGGCCCCGCCAGG
AAGGGTCCCGAACAGACTGAGCTTTACGCGCAGCTGAGCAAAGCGTCCACCGCCCTCCCT
TACACTATTGCTCCATATGCAGTAGGGGGCGGCCTTGAGGAGCACCGCAGCCCTGGCAAC
AACAAGCGGGCCTGTCCCCGCGGCCACAGTGACCATGATTATTGCCAGGCATCAGCTCGT
TCCAAGGCGGCAGGGGGCACAGCCACTGTGACCACAGTGGAAATGACATTTACCCCACGT
GCTTCTGATGCCTCAATGCCCAGTTCTGGCAAACAAACCCTCACACAAGCCAGTCCTATC
CCCGCATTCGGGGCCGCCTCCATGAGGGACCAACCACAACTCTGTGCCACGAGCCGGAAG
CCCCTGTGCGACCAGGAAATCAGAGCAGAGCTCAACAAGCACTTTGGCCACCACCCACAA
GCCGTCCACAGCCAGGCCCCTCAGCCTGCTGTGGCGGGAGAAAATGACTATTACTCCCAG
AAGCTGCTTGGCTCCAGCTACCTGCACACAGGGTTTCTGCCTTTCCATGATGAGATAGAG
CTGGGCGAGGGCCGTGACAGTCGCTTTGTCTACCCTTGGGAGGGAACCCCTCTGGACCTA
CTCTTTGACTGCTCTCCCAGCTCTCCCTCCTGTTCCCCACCATCCAGCTGCTCCCCCTCC
CGAGGCTCCCTCTCCCCGCCTTCCTCCCTTCTCCTGTCACCCAATAGACCTTTCTGCTGG

ACCACCAGCGGATCCCGCTCCCGCTCCCGTTCCCACTCCGGCTCCCGCAGCTCGTCATCA
CGGTATCGCCGGCGCTCCCTGTCCAGCTCCCCTGATAGACGCCCCCCCTCCTGGTCGCGT
CACAGCACAGATTTGAATGTTTCTCGCTCCAGAACCCACAAGAGTCCCCGCCCCCAGTCA
CGCTCTCCTCTCAGCCGCAGGCCAAGGTATGACAGCTATGAGGAGTACCAGCATGAGAGG
CTGAAGAGGGAGGAGTACCGCCTGGATTACGAGAAGCGGGAGTTTGAAAGGGCTGAGCAG
AGAGAAAAGCAACGGCAAAAAGCCATAGAGGAGAGGAGGGTGGTGTATGTGGGGCGACTG
AGGTCCGACTGCACCCGGACCGAGTTGAAGCGCCGCTTTGAAGTCTTCGGAGAAATTGAA
GAATGTGCAGTGAACTTGAGGGATGATGGGGACAATTTTGGCTTCATCACCTACCGCTAT
ACTTGTGACGCCTTTGCCGCCCTTGAGAACGGACACACCTTACGCAGGCCAAACGATCCT
CAGTTCGAGCTGTGCTTCGGTGGACAAAAGCAGTTCTGCAAATCACATTACACAGACTTG
>Mouse_PPARGC1A
ATGGCTTGGGACATGTGCAGCCAAGACTCTGTATGGAGTGACATAGAGTGTGCTGCTCTG
GTTGGTGAGGACCAGCCTCTTTGCCCAGATCTTCCTGAACTTGACCTTTCTGAACTTGAT
GTGAATGACTTGGATACAGACAGCTTTCTGGGTGGATTGAAGTGGTGTAGCGACCAATCG
GAAATCATATCCAACCAGTACAACAATGAGCCTGCGAACATATTTGAGAAGATAGATGAA
GAGAATGAGGCAAACTTGCTAGCGGTCCTCACAGAGACACTGGACAGTCTCCCCGTGGAT
GAAGACGGATTGCCCTCATTTGATGCACTGACAGATGGAGCCGTGACCACTGACAACGAG
GCCAGTCCTTCCTCCATGCCTGACGGCACCCCTCCCCCTCAGGAGGCAGAAGAGCCGTCT
CTACTTAAGAAGCTCTTACTGGCACCAGCCAACACTCAGCTCAGCTACAATGAATGCAGC
GGTCTTAGCACTCAGAACCATGCAGCAAACCACACCCACAGGATCAGAACAAACCCTGCC
ATTGTTAAGACCGAGAATTCATGGAGCAATAAAGCGAAGAGCATTTGTCAACAGCAAAAG
CCACAAAGACGTCCCTGCTCAGAGCTTCTCAAGTATCTGACCACAAACGATGACCCTCCT
CACACCAAACCCACAGAAAACAGGAACAGCAGCAGAGACAAATGTGCTTCCAAAAAGAAG
TCCCATACACAACCGCAGTCGCAACATGCTCAAGCCAAACCAACAACTTTATCTCTTCCT
CTGACCCCAGAGTCACCAAATGACCCCAAGGGTTCCCCATTTGAGAACAAGACTATTGAG
CGAACCTTAAGTGTGGAACTCTCTGGAACTGCAGGCCTAACTCCTCCCACAACTCCTCCT
CATAAAGCCAACCAAGATAACCCTTTCAAGGCTTCGCCAAAGCTGAAGCCCTCTTGCAAG
ACCGTGGTGCCACCGCCAACCAAGAGGGCCCGGTACAGCGAGTGTTCTGGTACCCAAGGC
AGCCACTCCACCAAGAAAGGGCCCGAGCAATCTGAGTTGTACGCACAACTCAGCAAGTCC
TCAGGGCTCAGCCGAGGACACGAGGAAAGGAAGACTAAACGGCCCAGTCTTCGGCTGTTT
GGTGACCATGACTATTGTCAGTCACTCAATTCCAAAACGGATATACTCATTAACATATCA
CAGGAGCTCCAAGACTCTAGACAACTAGACTTCAAAGATGCCTCCTGTGACTGGCAGGGG
CACATCTGTTCTTCCACAGATTCAGGCCAGTGCTACCTGAGAGAGACTTTGGAGGCCAGC
AAGCAGGTCTCTCCTTGCAGCACCAGAAAACAGCTCCAAGACCAGGAAATCCGAGCGGAG
CTGAACAAGCACTTCGGTCATCCCTGTCAAGCTGTGTTTGACGACAAATCAGACAAGACC
AGTGAACTAAGGGATGGCGACTTCAGTAATGAACAATTCTCCAAACTACCTGTGTTTATA
AATTCAGGACTAGCCATGGATGGCCTATTTGATGACAGTGAAGATGAAAGTGATAAACTG
AGCTACCCTTGGGATGGCACGCAGCCCTATTCATTGTTCGATGTGTCGCCTTCTTGCTCT
TCCTTTAACTCTCCGTGTCGAGACTCAGTGTCACCACCGAAATCCTTATTTTCTCAAAGA
CCCCAAAGGATGCGCTCTCGTTCAAGATCCTTTTCTCGACACAGGTCGTGTTCCCGATCA
CCATATTCCAGGTCAAGATTAAGGTCCCCAGGCAGTAGATCCTCTTCAAGATCCTGTTAC
TACTATGAATCAAGCCACTACAGACACCGCACACACCGCAATTCTCCCTTGTATGTGAGA
TCACGTTCAAGGTCACCCTACAGCTGTAGGCCCAGGTACGACAGCTATGAAGCCTATGAG
CACGAAAGGCTCAAGAGGGATGAATACCGCAAAGAGCACGAGAAGCGGGAGTCTGAAAGG

GCCAAGCAGAGAGAGAGGCAGAAGCAGAAAGCAATTGAAGAGCGCCGTGTGATTTACGTT
GGTAAAATCAGACCTGACACAACGCGGACAGAATTGAGAGACCGCTTTGAAGTTTTTGGT
GAAATTGAGGAATGCACCGTAAATCTGCGGGATGATGGAGACAGCTATGGTTTCATCACC
TACCGTTACACCTGTGACGCTTTCGCTGCTCTTGAGAATGGATATACTTTACGCAGGTCG
AACGAAACTGACTTCGAGCTGTACTTTTGTGGACGGAAGCAATTTTTCAAGTCTAACTAT
GCAGACCTAGATACAAACTCAGACGATTTTGACCCTGCTTCCACCAAGAGCAAGTATGAC
TCTCTGGATTTTGATAGTTTACTGAAGGAAGCTCAGAGAAGCTTGCGCAGG
>Naked_mole_rat_PPARGC1A
ATGGATGAGACCTCTCCCAGGCTGGAAGAAGACTGGAAAAAAGAACTTCAGCAAGAAGCA
GGCTGGCAGTGTGCTGCTTTGGTTGGTGAAGACCAGCCCCTTTGCCCAGATCTTCCTGAA
CTTGATCTTTCTGAACTAGATGTGAACGACTTGGATACAGACAGCTTTCTGGGTGGACTG
AAGTGGTGCAGTGACCAATCAGAAATAATATCCAACCAGTACAACAATGAGCCTTCAAAC
ATATTTGAGAAGATAGATGAAGAAAATGAGGCAAACTTGCTAGCTGTCCTCACAGAGACA
CTGGACAGTCTCCCTGTGGACGAAGACGGATTGCCCTCATTTGACGCACTGACAGATGGA
GACGTGACTACTGACAACGAGGCCAGTCCTTCCTCCATGCCTGACGGCACCCCTCCACCT
CAGGAGGCAGAAGAGCCATCTCTACTTAAGAAGCTCTTACTGGCACCGGCAAATACTCAG
CTGAGTTATAATGAATGCAGTGGTCTCAGTACCCAGAACCATGCAAACCACAATCACAGG
ATCAGAACAAACCCTGCAGTTGTTAAGACCGAGAATTCATGGAGCAATAAAACAAAGAGC
ATTTGTCAACAGCAAAAGCCACAAAGACGTCCCTGCTCGGAGCTTCTCAAGTATCTGACC
ACAAATGATGACCCTCCTCACACCAAACCCACAGAGAACAGGAACAGCAGCAGAGACAAA
TGCACTTCCAAAAAGAAGTCCCACACACAATCGCAGTCCCAACATGTACAAGCCAAACCA
ACAACTTTATCTCTTCCTCTGACCCCAGAGTCACCAAATGACCCCAAGGGTTCCCCATTT
GAGAACAAGACTATTGAACGAACCTTAAGTGTGGAACTCTCTGGAACTGCAGGCCTAACT
CCACCTACAACTCCTCCTCATAAAGCCAACCAAGATAACCCTTTCAGGGCTTCTCCAAAG
CTGAAGTCCTCTTGCAAGACAGTGGTGCCACCGCCATCAAAGAAGCCCCGGCACAGTGAG
TCTTCTGGTACCCAAGTCAATAACTCCACCAAGAAGGGGCCCGAGCAATCTGAGTTGTAT
GCACAACTCAGCAAGTCCTCAGTGCTCGGAAGTGGACAAGAGGAGAGGAAGACTAAGCGG
CCCAGCCTACGGCTGTTTGGTGACCATGACTATTGTCAGTCCATTAATTCCAAAACAGAA
ATAATTATTAATATATCACAAGAGCTCCAAGACTCTAGACAACTAGACTATAAAGATGCC
TCCTCTGACTGGCAGGGGCAGGTTTGTTCTTCCACAGATTCAGACCAGTGCTACCTGAGA
GAGACTTTGGAGGCCAGCAAGCAGCTCTCTCCTTGCAGCACCAGAAAACAGCTCCAGGAC
CAGGAAATCCGAGCCGAGCTGAACAAGCATTTCGGTCATCCCAGTCAAGCTGTTTTTGAC
GACGAAGCAGACAAGACCAGTGAACTGAGGGACAGTGATTTCAGTAATGAACAATTCTCC
AAACTACCTATGTTTATAAATTCAGGACTAGCCATGGATGGCCTGTTTGATGACAGCGAA
GATGAAAGTGATAAACTGAGCTACCCTTGGGATGGCACGCAATCCTATTCATTGTTTGAT
GTGTCGCCTTCCTGCTCTTCTTTTAACTCTCCATGTAGAGATTCCGTGTCGCCACCCAAA
TCCTTATTTTCTCAAAGACCCCAAAGGATGCGCTCTCGTTCAAGGTCCTTTTCTCGACAC
AGGTCATGTTCCCGATCACCTTATTCCAGATCAAGATCAAGGTCCCCAGGCAGTAGATCT
TCAAGATCCTGCTACTACTATGAATCAAGCCACTACAGACACCACACGCACCGAAACTCT
CCCTTGTATGTGAGATCACGTTCAAGATCACCCTACAGCCGTAGGCCCAGGTATGACAGC
TACGAGGCATATCAGCATGAAAGGCTGAAGAGGGAAGAATACCGGAAAGAGTACGACAAG
CGGGAATCTGAAAGGGCCAAACAGAGGGAGAGGCAGAGGCAGAAGGCAATTGAAGAGCGC
CGTGTGATTTACATCGATAAAATCAGACCTGACACAACACGGACAGAACTGAGGGACCGC
TTTGAAGTTTTTGGTGAAATTGAGGAGTGCACAGTAAATCTGCGGGATGATGGAGACAGC

TATGGTTTCATCACCTATCGTTATACCTGTGACGCTTTTGCTGCTCTTGAAAATGGATAT
ACTTTGCGCAGGTCGAACGAAATGGACTTCGAGCTGTACTTTTGTGGACGCAAGCAATTT
TTCAAGTCTAACTATGCAGACTTAGATTCAAACTCAGATGACTTTGACCCTGCTTCCACC
AAGAGCAAGTATGACTCTCTGGATTTTGATAGTTTACTGAAAGAAGCTCAGAGAAGCTTG
CGCAGG
>Opossum_PPARGC1A
ATGGCGTGGGACATGTGCAACCAGGACTCTGTATGGAGTGACATAGAGTGTGCTGCTCTC
GTTGGTGAAGACCAGCCTCTTTGCCCAGATCTCCCAGAACTTGACCTCTCTGAACTAGAC
GTGAATGACTTGGATGCCGACGGCTTCCTAGGTGGACTCAAGTGGTGTAGCGACCAATCA
GAAATCATTTCTAATCAGTACAGCAATGAATCTTCAAATATATTTGAGAAGATAGATGAA
GAGAATGAGGCAAACTTGCTAGCAGTTCTCACAGAAACATTGGACAGTCTCCCTGTGGAT
GAGGATGGATTGCCCTCATTTGATGCACTGACAGATGGAGATGTGACCAATGACAATGAT
GCTAGCCCTTCGCCTATGCCCGACGGCACCCCTCCAACTCAGGAGGCAGAAGAGCCGTCT
CTACTTAAGAAGCTCTTACTGGCTCCAGCCAACACTCAGCTAAGTTATAATGAATGCAGT
GGTCTCAGTACTCAGAACCATGCAAATACTAATCATAGGATCAGAACAAACCCTGCGGTT
GTTAAGACCGAGAATTCATGGAGCAATAAAGCAAAGAGCATTTGTCAACAGCAAAAGCCA
CAAAGGCGTCCCTGCTCGGAGCTCCTCAAGTATCTGACTACAAATGATGACCCTCCTCAG
ACCAAACCAACAGAGAGCAGGAACGGCAGCAAAGACAAATGCCCTTCCAAAAAGAAGCCC
CATCTCCAGTCTCAGCCGCACCATTTGCAAGCCAAACCAACAAGTTTATCACTTCCTTTG
ACACCCGAGTCACCAAATGATCCCAAGGGTTCCCCATTTGAGAACAAGACTATTGAAAGA
ACCTTAAGTGTGGAACTCTCTGGAACTGCAGGCCTAACTCCACCTACGACTCCTCCTCAT
AAAGCTAATCAAGATAATCCTTTTAGGACTTCTCCAAAGCCGAAGTCATCATGTAAAAGC
AGTGTGCCACCTTCCAAGAAACCCCGCTATAGCGAATCTTCTGGTACCCAAGTAAATACT
CCCACTAAGAAAGGTCCAGAACAATCGGAGCTGTACGCCCAGCTCAGCAAGACTTCAGGG
CTATCCAGCGGACAGGAGGAGAGAAAGACAAAACGGCCTAGTTTACGGCTATTTGGAGAC
CATGACTACTGTCAGTCGATTAATGCAAAAACTGAAATCCGCATTAATTTATCACAGGAG
CTCCAGGTCTCTAGACAACTAGAATATAAAGATGCCTCCCCTGGATGTCAGGGGCCGACT
CGTTCTTCCACAGACCCAGACCAGTGCTACACAAGAGAGACTTTTCAGGCAAGCAAGCAG
GGTTCCCCATGCGGCCACAGAAAACAGCTCCAAGACCAGGAAATCCGGGCTGAGCTGAAC
AAGCACTTTGGTCATCCCAGTCAAGCTGTTTTTGATGACGAAGCAGACAAGACCAGTGAA
CTGAGGAACAGTGATTTTAGTAACGAACAATTCTCCAAACTACCTATGTTTATAAATTCA
GGACTAGCCATGGATGGTCTGTTTGATGACAGCGAAGATGAAAGTGATAAACTGAGCTAC
CCTTGGGATGGGACACAATCCTATTCATTGTTCGACGTGTCACCTTCTTGCTCTTCTTTT
AATTCTCCATGTCGAGATTCAGTGTCTCCACCCAAATCCTTATTTTCTCAAAGATCCCAA
AGGATACACTCTAGATCACGGTCCTTTTCTCGACACAGGTCATGTTCCCGATCTCCATAT
TCCAGGTCAAGATCAAGGTCACCAGGCAGCAGATCTTCCTCAAGATCTTGTTACTACTAT
GAGTCCAGCCATTGCAGACATCGGGGACACCACAGTTCTCCCTTATATGCAAGGTCACGC
TCCAGATCGCCCTACAGCCGAAGGCCCAGGTATGACAGCTACGAAGAATATCAGCATGAA
AAGCTGAAGAGGGAAGAATACCGCAAAGAATATGAGAAGCGAGAGTCTGAAAGGGCCAAG
CAAAGGGAGAGACAAAGGCAGAAGGCAATTGAAGAGCGTCGTGTGATTTATGTTGGTAAA
ATCAGACCTGACATCACCCGGACAGAACTGAGGGACCGATTTGAAGTTTTTGGTGAAATT
GAGGAGTGCACAGTAAATCTGCGGGATGATGGAGATAGCTATGGTTTCATCACCTACCGC
TATACTTGTGATGCTTTTGCTGCTCTTGAAAATGGATACACTTTGCGCAGGTCGAATGAA
CCTGACTTTGAGCTGTACTTTTGTGGACGCAAGCAATATTTCAAGTCTAACTATGCAGAC

CTAGATTCACACTCAGATGATTTTGATCCTGCTTCTACCAAGAGCAAGTATGACTCTCTG
GATTTTGATAGTTTACTGAAAGAGGCCCAGAGAAGCCTACGCAGG
>Orangutan_PPARGC1A
ATGGCGTGGGACATGTGCAACCAGGACTCTGAGTCTGTATGGAGTGACATCGAGTGTGCT
GCTCTGGTTGGTGAAGACCAGCCTCTTTGCCCAGATCTTCCTGAACTTGATCTTTCTGAA
CTAGATGTGAACGACTTGGATACAGACAGCTTTCTGGGTGGACTCAAGTGGTGCAGTGAC
CAATCAGAAATAATATCCAATCAGTACAACAATGAGCCTTCAAACATATTTGAGAAGATA
GATGAAGAGAATGAGGCAAACTTGCTAGCAGTCCTCACAGAGACACTGGACAGTCTCCCT
GTGGATGAAGACGGATTGCCCTCATTTGATGCGCTGACAGATGGAGACGTGACCACTGAC
AATGAGGCTAGTCCTTCCTCCATGCCTGACGGCACCCCTCCACCCCAGGAGGCAGAAGAG
CCGTCTCTACTTAAGAAGCTCTTACTGGCACCAGCCAACACTCAGCTAAGTTACAATGAA
TGCAGTGGTCTCAGTACCCAGAACCATGCAAATCACAATCACAGGATCAGAACAAACCCT
GCAATTGTTAAGACTGAGAATTCATGGAGCAATAAAGCGAAGAGTATTTGTCAACAGCAA
AAGCCACAAAGACGTCCCTGCTCAGAGCTTCTCAAATATCTGACCACAAACGATGACCCT
CCTCACACCAAACCCACAGAAAACAGAAACAGCAGCAGAGACAAATGCACCTCCAAAAAG
AAGTCCCACACACAATCGCAGTCACAACATTTACAAGCCAAACCAACAACTTTATCTCTT
CCTCTGACCCCAGAGTCACCAAATGACCCCAAGGGTTCCCCATTTGAGAACAAGACTATT
GAACGCACCTTAAGTGTGGAACTCTCTGGAACTGCAGGCCTAACTCCACCCACCACTCCT
CCTCATAAAGCCAACCAAGATAACCCTTTTAGGGCTTCTCCAAAGCTGAAGTCCTCTTGC
AAGACTGTGGTGCCACCACCATCAAAGAAGCCCAGGTACAGTGAGTCTTCTGGTACACAA
GGCAATAACTCCACCAAGAAAGGGCCGGAGCAATCCGAGTTGTATGCACAACTCAGCAAG
TCCTCAGTGCTCACTGGTGGACACGAGGAAAGGAAGACCAAGCGGCCCAGTCTACGGCTG
TTTGGTGACCATGACTATTGCCAGTCAATTAATTCCAAAACGGAAATACTCATTAATATA
TCACAGGAGCTCCAAGACTCTAGACAACTAGAAAATAAAGATGTCTCCTCTGATTGGCAG
GGGCAGATTTGTTCTTCCACAGATTCAGACCAGTGCTACCTGAGAGAGACTTTGGAGGCA
AGCAAGCAGGTCTCTCCTTGCAGCACAAGAAAACAGCTCCAAGACCAGGAAATCCGAGCT
GAGCTGAACAAGCACTTCGGTCATCCCAGTCAAGCTGTTTTTGACGACGAAGCAGACAAG
ACCAGTGAACTGAGGGACAGTGATTTCAGTAATGAACAATTCTCCAAACTACCTATGTTT
ATAAATTCAGGACTAGCCATGGATGGCCTGTTTGATGACAGCGAAGATGAAAGTGATAAA
CTGAGCTACCCTTGGGATGGCACGCAATCCTATTCATTGTTCAATGTGTCTCCTTCTTGT
TCTTCTTTTAACTCTCCATGTAGAGATTCCGTGTCACCACCCAAATCCTTATTTTCTCAA
AGACCCCAAAGGATGCGCTCTCGTTCAAGGTCCTTTTCTCGACACAGGTCGTGTTCCCGA
TCACCATATTCCAGGTCAAGATCAAGGTCCCCAGGCAGTAGATCCTCTTCAAGATCCTGC
TATTACTATGAGTCAAGCCACTACAGACACCGCACGCACCGAAATTCTCCCTTGTATGTG
AGATCACGTTCAAGATCGCCCTATAGCCGTCGGCCCAGGTATGACAGCTACGAGGAATAT
CAGCACGAGAGGCTGAAGAGGGAAGAATATCGCAGAGAGTATGAGAAGCGAGAGTCTGAG
AGGGCCAAGCAAAGGGAGAGGCAGAGGCAGAAGGCAATTGAAGAGCGCCGTGTGATTTAT
GTCGGTAAAATCAGACCTGACACAACACGGACAGAACTGAGGGACCGTTTTGAAGTTTTT
GGTGAAATTGAGGAGTGCACAGTAAATCTGCGGGATGATGGAGACAGCTATGGTTTCATT
ACCTACCGTTATACCTGTGATGCTTTTGCTGCTCTTGAAAATGGATACACTTTGCGCAGG
TCGAACGAAACTGACTTCGAGCTGTACTTTTGTGGACGCAAGCAATTTTTCAAGTCTAAC
TATGCAGACCTAGATTCAAACTCAGATGACTTTGACCCTGCTTCCACCAAGAGCAAGTAT
GACTCTCTGGATTTTGATAGTTTACTGAAAGAAGCTCAGAGAAGCTTGCGCAGG
>Western_painted_turtle_PPARGC1A

ATGCCTTCTTCTCTTCCTTCTTCTACAACTATACCTCCTCCTGGGAAAACTCCTACAACC
AGGAGCTGGATGGCGTGGGACATGTGCAACCAGGACTCTGTATGGAGTGATATAGAGTGT
GCTGCTCTGGTTGGTGAAGACCAGCCTCTTTGCCCAGATCTCCCAGAACTTGACCTCTCT
GAACTAGATGTGAATGACTTGGATGCAGACGGTTTTCTGGGTGGACTCAAGTGGTACAGC
GACCAATCAGAAATCATTACCAATCAGTACAGCAATGAATCATCAAATATATTTGAGAAG
ATAGATGAAGAGAATGAAGCAAACTTGCTAGCAGTTCTCACTGAGACACTGGACAGTATC
CCTGTGGATGAGGATGGATTGCCTTCATTTGATGCACTGACAGATGGAGATGTGACCAAT
GAAAATGATGCTAGCCCTTCACCAATGCCCGACGGCACCCCTCCGACTCAGGAGGCAGAA
GAGCCGTCTCTACTTAAGAAGCTCTTGCTGGCTCCAGCCAACATTCAGCTAAATTACAAT
GAATGCAGTGGTCTCAGCACACAAAACCATGCAAACACTAATCACAGGATCAGAACAAGC
CCTGTGGTTGTTAAGACCGAGAATTCATGGAGCAATAAACCGAAGAGCATTTGTCAACAG
CAAAAGCCACAAAGACGTCCCTGCTCTGAGCTTCTCAAGTATCTGACTACAAATGATGAC
CCTCCTCAGACCAAACCAACAGAGACCAGGAACAGCAGCAAAGACAAATGCATCTCCAAA
AAGAAGCCCCATCTGCAGTCTCAGGCACATCATTTGCAAGCCAAACCAACAAGTTTATCA
CTTCCTTTGACACCTGAGTCACCAAATGATCCCAAGGGTTCCCCATTTGAGAACAAGACT
ATTGAACAAACCTTAAGTGTGGAACTCTCTGGAACTGCAGGCCTAACTCCACCTACAACC
CCTCCTCACAAAGCCAACCAAGATAATCCTTTCAGGACTTCACCCAAGCTGAAGTCATCA
TGCAAGACTATTGTACCACCCTCAAAAAAGCCCCGCTACAGTGAGTCTTCCAGTTCTCAA
GGACATAACACAATCAAGAAGAGTCCAGAACAGTCTGAGCTGTATGCACAGCTTAGCAAG
ACAACTGTACTGTCCAGTGGACATGAGGAGAGAAAGACAAAACGGCCTAGTTTGCGGCTG
TTTGGTGACCATGACTACTGTCAATCTGTGAATTCAAAAACCGAAATACACATTAAAATA
TCCCAGGAACTTCAGGACTCCNNACAACAAGAATTTAAAGATTCTGCACCTGGGTGGCAG
TGTCAGATTTGTTCTTCTTTAGAACAAGACCAGTATTACAAGAGAGAGAGACTTTACAGGCA
AGTAAGCAGGGTTCCCACTCTAGCAGCCGAAAACAGCTCCAAGACCAGGATATCCGGGCC
GAACTGAATAAGCACTTTGGTCACCCCAGCCAAGCTGTTTTTGATGAAGAGGCAGATAAG
GCCAGTGAACTGAGGGACAGTGATTATAGTAATGAACAATTCTCCAAACTACCTATGTTT
ATAACTTCAGGACTAGCTATGGATGGCCTCTTTGATGACAGTGAAGATGAAAGTGATAAA
CTATGCTACCCTTGGGATGGGACAGAAGCCTATTCATTATTTGATGTATCGCCTTCTTGC
TCTTCTTTTAACTCTCCATGCAGAGATTCAGTGTCTCCACCCAAATCCTTATTTTCTCAA
AGATCCCAAAGGATACGCTCTAGATCAATGTCCTTTCCTCAGCACAGGTCTTGTTCCCGT
TCTCCATATTCCCGATCGAGATCAAGGTCGCCATGTAACAGATCCTCTTCCAGATCTTGT
TACTATTATGAGTCCAGCCACTGTAGACATCGAGCATACAGAAGTTCTCCTTTATATGCA
AGATCACGATCCAGATCACCGTATAGTTGTAGACCCAGATATGACAGCTATGAGGAATAT
CAGCATGAAAGACTCAAAAGGGAAGAATACCGCAAAGAGTATGAAAAACGGGAATCTGAA
AGGGCCAAACAAAGGGAGAGGCAGAGACAAAAAGCAATTGAAGAACGTCGTGTGATGTAT
GTGGGTAAAATCAGAGCTGATACCACCCGAACAGAACTGAGGGACCGGTTTGAAGTTTTT
GGTGAAATTGAGGAGTGTACAGTACATCTGCGGGATGATGGAGACAGCTATGGTTTCATC
ACCTACCGCTATACTTGTGATGCGTTTGCTGCTCTCGAAAATGGATACACTTTACGCAGG
TCAAATGAACCTGACTTTGAGTTGTACTTTTGTGGACGCAAGCAATTTTGCAAGTCTAAC
TATGCAGACCTAGACTCAAACTCAGATGATTTTGATCCTGCTTCCACTAAGAGCAAATAT
GACTCCCTGGATTTTGATAGTTTACTTAAAGAGGCACAGCGAAGCCTGCGTAGG
>Panda_PPARGC1A
ATGGCGTGGGACATGTGCAACCAGGACTCTGTATGGAGTGACATCGAGTGTGCTGCTCTG
GTTGGTGAAGACCAGCCTCTTTGCCCAGATCTTCCTGAACTTGACCTTTCTGAACTAGAC

GTGAACGACTTGGATACAGACAGCTTTCTGGGCGGACTCAAGTGGTGCAGTGACCAATCA
GAAATAATATCCAATCAGTACAACAATGAGCCTTCAAACATATTTGAGAAGATAGATGAA
GAGAACGAGGCGAACTTGCTAGCAGTCCTCACAGAGACACTGGACAGTCTCCCTGTGGAT
GAAGACGGATTGCCCTCATTTGACGCGTTGACAGATGGAGATGTGACCACGGAGAACGAG
GCTAGTCCTTCCTCCATGCCTGACGGCACCCCTCCGCCTCAGGAGGCAGAAGAGCCGTCT
CTACTTAAGAAGCTCTTACTGGCACCAGCCAACACTCAGCTAAGTTATAATGAATGCAGT
GGTCTCAGTACCCAGAACCATGCAAACCATAATCACAGGATCAGAACAACCCCTGCAGTT
GTTAAGACTGAGAATTCATGGAGCAATAAAGCGAAGAGCATTTGTCAACAGCAAAAGCCA
CAAAGACGTCCCTGCTCGGAGCTTCTCAAGTATCTGACCACAAACGATGACCCTCCTCAC
ACCAAACCCACAGAGAACAGGAACAGTAGCAGAGACAAATGCACCTCCAAAAAGAAGTCC
CACACACAATCGCAGTCTCAACATTTACAAGCCAAACCAACAACTTTATCTCTTCCTCTG
ACCCCAGAGTCACCAAATGACCCCAAGGGTTCCCCATTTGAGAACAAGACTATTGAACGA
ACCTTAAGTGTGGAACTCTCTGGAACTGCAGGCCTAACTCCACCCACAACTCCTCCTCAT
AAAGCCAACCAAGATAGCCCTTTCAGGACTTCTCCGAAGCTGAAGTCCTCTTGCAAGACT
GTGGTACCTCCGCCGTCCAAGAAGCCCCGGAACAGTGAGCCTTCGGGCGCCCAAGGCAAT
AACTCCACCAAGAAAGGGCCCGAGCAGTCTGAGCTGTACGCGCAGCTCAGCAAGACCTCC
GTGCTCACCAGTGGACACGAGGAAAGGAAGGCCAAGCGGCCCAGTCTACGGCTGTTTGGT
GACCATGACTATTGTCAGTCAATTAATTCCAAAACAGAAATACTCATTAATCTATCACAG
GAGCTCCAAGACTCTAGACCACTAGAATATAAAGATGTCTCCTCCGATCGGCAGGGGCAA
ATTTGCTCTTCCACAGATTCAGACCAGTGCTACCTGAGAGAGACTTCGGAGGCGGGCAAG
CAGGTCTCTCCTTGCAGCACCAGAAAACAGCTCCAAGACCAGGAAATCCGAGCCGAGCTG
AACAAGCACTTCGGTCATCCCAGTCAAGCTGTTTTTGACGACGAAGCAGACAAGACCAGT
GAACTGAGGGACAGTGATTTCAGTAATGAACAATTCTCCAAACTACCTATGTTTATAAAT
TCAGGACTAGCCATGGATGGCCTGTTTGATGACAGCGAAGATGAAAATGATAAACTGAAC
TACCCTTGGGACGGCACGCAGTCCTATTCATTGTTCGGTGTGTCGCCTTCTTGCTCTTCT
TTTAACTCTCCATGTAGGGATTCCGTGTCACCACCCAAATCCTTATTTGCTCAAAGACCC
CAAAGGATGCGCTCTCGTTCAAGGTCCTTTTCTCGACACAGGTCGTGTTCCCGATCACCA
TATTCCAGGTCAAGATCAAGGTCCCCAGGCAGTAGATCCTCTTCAAGATCTTGCTACTAC
TATGAGTCGAGCCACTGCAGACACCGCGCGCACCGAAATTCCCCTCTGTGCGCAAGATCA
CGTTCCAGGTCGCCCTACAGCCGTAGGCCCAGGTATGACAGCTACGAGGAATATCAGCAC
GAAAGGCTGAAGAGGGAAGAATACCGCAAAGAGTATGAGAAGCGGGAGTCTGAGAGGGCC
AAGCAAAGGGAGAGGCAGAGGCAGAAGGCAATTGAAGAACGCCGTGTGATTTACGTTGGT
AAAATCAGACCTGACACGACACGGACGGAACTGAGGGACCGTTTTGAAGTTTTTGGTGAA
ATTGAGGAGTGCACAGTAAATCTACGGGATGATGGAGACAGCTATGGTTTCATTACCTAC
CGTTATACCTGTGATGCTTTTGCTGCTCTTGAAAATGGATATACTTTGCGCAGGTCGAAT
GAAACTGACTTCGAGCTGTACTTTTGTGGACGCAAGCAATTTTTCAAGTCTAACTATGCA
GACCTAGATTCAAACTCAGATGACTTTGACCCTGCTTCCACCAAGAGCAAGTATGACTCT
CTGGATTTCGATAGTTTACTGAAAGAAGCTCAGAGAAGCTTGCGCAGG
>Platypus_PPARGC1A
ATGGCGTGGGACATGTGTAACCAGGATTCTGTATGGAGTGACATAGAGTGTGCTGCTCTG
GTTGGTGAAGACCAGCCTCTCTGTCCGGACCTCCCAGAACTTGACCTCTCCGAACTAGAC
GTGAATGACTTGGATGCCGACAGCTTCCTGGGTGGACTCAAGTGGTACAGTGACCAATCG
GAAATCATTTCCAATCAGTACAGCAATGAACCTTCAAATATATTTGAGAAGATAGATGAA
GAGAATGAGGCAAACTTGCTAGCAGTCCTCACAGAGACACTGGACAGTATCCCCGTGGAC

GAGGATGGATTGCCTTCATTTGATGCACTGACAGATGGAGATGTGACCAACGAAAATGAT
TCTAGCCCTTCATCCATGCCTGACGGCACCCCTCCGACTCAGGAGGCAGAAGAGCCGTCT
CTACTTAAGAAGCTCTTGCTGGCTCCAGCCAATACTCAGCTAAGTTATAATGAATGCTGT
GGTCTCAGCACACAAAACCATGCAAACCCTAATCACAGGATCAGAACAATCCCTGCAGTG
GTTAAGACTGAGAATTCGTGGAGCAATAAAGCGAAGAGCATTTGTCAACAGCAAAAGCCG
CAAAGGCGCCCCTGCTCAGAGCTCCTCAAGTATCTGACCACGAATGACGACCCGCCTCAG
ACCAAAGCAGCAGAGAGCAGGAGCAGCAGCCAAGACAAATGCACCTCCAAAAAGAAGCCC
CACTTGCAGCCTCAGACGCATCATTTGCAAGCCAAACCAACAAGTTTATCACTTCCTCTG
ACCCCCGAGTCACCAAATGATGCCAAGGGTTCCCCATTTGAGAACAAGAATATTGAACGA
ACCTTAAGTGTGGAACTCTCTGGAACTGCAGGCCTAACTCCACCTACAACTCCTCCTCAC
AAAGTCAACCAAGATAGTCCTTTTAGGACTTCTCCCAAGCCGAAGTCCTCATGCAAGACT
GTTGTGCCACCGTCCAAAAAGCCCCGTTTCAGTGAGTCTTCCAGGACCCAAGGAAATAAT
CCCAATAAGAAAAGCCCGGAGCAATCGGAGCTGTACGCCCAGCTCAGCAAGAACTCCGTA
CTGTCCAGCGGACATGAGGAGAGGAAATCAAAACGACCGAGTTTGCGGCTGTTTGGTGAC
CACGACTACTGTCAGTCGCTCAATGCTAAAGCCGAAATCCGCATCAACGTACCGCAGGAG
CTCCAGGACTCTAGACAACTCGAATATAAAGATTCCTCATCTGGATGGCAGTGGCAGATT
TGTACTTCTGCAGATCAAGACCAGTGCTCCACGAGAGAGACTTCACAGGAGAGCAAGCAG
GGAGCCCCACGCAACAACCGCAAGCAGCTTCAAGACCAGGAAATCCGCGCTGAGCTGAAC
AAGCACTTTGGTCATCCCAGCCAGGCTGTTTTTGATGACGAAGCAGACAAGACCAGTGAA
CTGAGGGACAGTGATTTTAGTAATGAACAATTCTCCAAACTACCTATGTTTATAAATTCA
GGACTAGCTATGGATGGTCTGTTTGATGACAGCGAAGATGAAAATGATAAACTGTGCTAC
CCTTGGGATGGGACACAATTCTATTCATTGTTTGATGTATCGCCTTCCTGCTCTTCTTTT
AACTCTCCATGTAGAGATTCAGTGTCTCCACCCAAATCCTTATTTTCTCAAAGATCCCAA
AGGATACTCTCGAGATCAAGGTCCTTTTCTCGACACAGGTCATGTTCCCGATCTCCATAT
TCCCGGTCAAGATCAAGGTCTCCCAGCAGCAGATCCTCTTCAAGATCTTGTTACTACTAC
GAGCCAAGCCACTGCAGACCCCGAGCTCCGCGGAGATCTCCTTTACACGCAAGATCGCGC
TCCAGGTCGCCCTTCAGTCGTAGGCCCAGGTATGACAGCTACGAGGAATATCAGCACGAA
AGGCTGAAGAGGGAGGAATATCGCAAAGAGTACGAAAAACGGGAGTCTGAGAGGGCCAAG
CAAAGGGACAGACAAAGGCAGAAGGCAATTGAAGAACGTCGTGTGATTTATGTGGGTAAA
ATCAGACCTGACACAACCCGGACAGAACTGAGGGACCGTTTTGAAGTTTTTGGTGAAATT
GAGGAGTGTACCGTAAATCTGCGGGATGATGGAGACAGCTATGGTTTCATCACCTACCGC
TACACCTGCGATGCTTTTGCTGCTCTTGAAAACGGATACACTTTGCGCAGGTCGAATGAA
CCTGACTTTGAGCTGTACTTTTGTGGACGCAAGCAATTTTTCAAGTCTAACTATGCAGAC
CTAGATTCAAACTCAGATGATTTTGACCCCGCTTCCACCAAGAGCAAGTATGACTCCATG
GATTTTGATAGTTTACTTAGAGAAGCCCAGCGGAGCCTGCGCAGG
>Rabbit_PPARGC1A
ATGGCGTGGGACATGTGCAGCCAGGACTCTGTATGGAGTGACATCGAGTGTGCTGCTCTG
GTTGGTGAAGACCAGCCTCTTTGCCCAGATCTTCCTGAACTTGATCTTTCTGAACTAGAT
GTGAATGACTTGGATACAGACAGCTTTCTGGGTGGACTCAAGTGGTGCAGTGACCAATCA
GAAATAATATCCAACCAATACAACAATGAGCCTTCAAACATATTCGAGAAGATAGATGAA
GAGAATGAGGCAAACTTGCTAGCAGTCCTCACTGAGACCCTGGACAGTCTCCCTGTGGAT
GAAGACGGATTGCCTTCATTCGATGCGCTGACAGATGCTGACGTGACCACTGACAACGAG
GCCAGCCCTTCCTCCATGCCGCACGGCACCCCTCCCCCTCAGGAGGCAGAAGAGCCGTCT
CTACTTAAGAAGCTCTTACTAGCACCGGCCAACACTCAGTTAAGTTATAATGAATGCAGC

GGTGTCAGTACCCACAACCATGCAAACCATAATCACAGGATCAGAACAAACCCCGCAGTT
GTTAAGACTGAGAATTCATGGAGCAATAAAGCGAAGAGCATTTGTCAACAGCAAAAGCCA
CAAAGACGTCCCTGCTCGGAGCTTCTCAAGTATCTGACCACAAACAATGACCCTCCGCAC
ACCAAACCCGCAGAGAACAGGAACAGCAGCAGAGACAAATGCAACTCCAAAAAGAAGTTC
CACTCACAGTCGCAGTCGCAACATGTGCAAGCCAAACCAACAACTTTATCTCTTCCTCTG
ACCCCAGAGTCACCAAATGACCCCAAGGGTTCCCCATTTGAGAACAAGACTATTGAACGA
ACCTTAAGTGTGGAACTCTCTGGAACTGCAGGCCTAACTCCACCCACGACTCCTCCTCAT
AAAACCAACCAAGATAACCCTTTCAGGGCTTCTCCAAAGCCGAAGCCCTCTTGTAAGACC
GTGGTGCCACCACCATCAAAGAAGGCCCGGTGCAGTGAGACTTCTTGTACCCAAAGCAGT
AACTCCGCCAAGAAGGGGCCCGAGCAATCCGAGTTGTACGCACAACTCAGCAAGTCCTCG
GTGCTCGGCAGTGGGCCCGAGGAGAAGAAGCCTAAGAGGCCCAGCCTGCGGCTGTTTGGT
GATCATGACTATTGTCAGTCAATTAATTCCAAAACAGAAATACTCGTTAATATATCACAG
GAGCTTCAAGACTCCAGGCAACTAGACTATAAAGATGCCTCCTCTGATTGGCAGGGGCAG
ATCTGCTCTTCCCCAGATTCAGACCAGTGCTCCCTGAGAGAGACGTTGGAGGCCAGCAAG
CAGGTCTCTCCTTGCAGCACCAGGAAACAGCTCCAAGACCAGGAAATCCGAGCCGAGCTG
AACAAGCACTTCGGTCATCCCAGTCAAGCTGTTTTTGACGACGAAGCAGACAAGACCAGT
GAACTGAGGGACAGTGATTTCAGTAATGAACAATTCTCCAAACTACCTATGTTTATAAAT
TCAGGACTAGCCATGGATGGCCTGTTTGATGACAGCGAAGATGAAAGTGACAAACTGAGC
TACCCTTGGGATGGCACGCAATCCTATTCATTGTTTGATGTGTCTCCGTCTTGCTCTTCT
TTTAACTCTCCGTGTAGAGATTCCGTGTCACCACCCAAATCCTTATTTTCTCAAAGACCC
CAAAGGATGCGTTCTCGTTCAAGGTCCTTTTCTCGGCACAGGTCGTGTTCCCGATCACCA
TATTCCAGGTCAAGATCAAGGTCCCCAGGCAGTAGATCCTCTTCAAGATCCTGCTACTAC
TATGAGTCAAGCCACTACAGACACCGCACACACCGAACTTCCCCCTTGTATGTGAGATCA
CGTTCAAGATCACCCTACAGCCGTAGGCCCAGGTATGACAGCTACGAGGAATATCAGAAC
GAAAGGCTGAAGAGAGAAGAATACCGCAAAGAGTACGAGAAGCGGGAATCGGAAAGGGCC
AAGCAAAGGGAGAGGCAGAGGCAGAAGGCGATTGAAGAGCGCCGCGTGATTTATGTGGGT
AAAATCAGACCTGACACAACACGGACAGAACTGAGGGACCGTTTTGAAGTTTTTGGTGAA
ATTGAGGAGTGCACAGTAAATCTGCGGGATGATGGAGACAGCTATGGTTTCATTACCTAC
CGATACACCTGTGATGCTTTTGCTGCTCTTGAAAATGGATATACTTTGCGCAGGTCGAAC
GAAACAGACTTCGAGCTGTACTTTTGTGGACGCAAGCAATTTTTCAAGTCTAACTATGCA
GACCTAGATTCAAACTCAGATGACTTTGACCCTGCTTCCACCAAGAGCAAGTACGACTCT
CTGGATTTTGATAGTTTACTGAAAGAAGCTCAAAGAAGCTTGCGCAGG
>Tetraodon_PPARGC1A
AGGTATGACAGTTATGAGGAGTACCAGCACGAGAGGCTGAAGAGGGAGGAGTACCGCCGG
GACTACGAGAAGCGGGAGTCCGAGAGGGCCGAGCAGAGGGAGAAGCAACGGCAAAAAGCA
ATAGAGGAGAGACGGGTCGTGTACGTGGGCCGACTGAGGTCCGACTGCACGCGCACGGAG
CTGAAGCGCCGCTTTGAAGTCTTTGGCGAAATTGAAGAATGTGCGGTGAACCTGAGGGAC
GACGGGGACAACTTTGGCTTCATCACGTACCGCTACACCTGCGACGCCTTCGCTGCCCTG
GAGAACGGACACACCTTACGCCGCTCGGATGAGCCTCAGTTCGAGCTGTGCTTCGGCGGA
CAAAAGCAGTTCTGCAAATCGCATTACGCAGACTTGGACTCCCACTCGGACGACTTTGAT
CCGGCCTCCACCAAGAGCAAGTACGGCTCCCTGGACTTCGACAGCTTGCTGCGGGAGGCC
CAGCGCAGCCTCCGGAGG
>Tilapia_PPARGC1A
ATGGCGTGGGACAGGTGTAACCAGGACTCGGTGTGGAGAGAATTAGAGTGTGCTGCCTTG

GTTGGTGAAGACCAGCCCCTCTGCCCAGATTTACCTGAACTTGACCTCTCAGAGCTGGAT
GTCAGTGACTTAGATGCAGACAGTTTCCTGGGCGGCCTCAAATGGTACAGTGACCAATCA
GAGATCATTTCTGCTCAGTATGGGAACGAAGCGGCCAATCTTTTTGAGATAGATGAAGAA
AATGAGGCCAACTTGCTGGCAGTGCTTACAGAGACCCTGGACAGCATCCCGGTGGATGAG
GACGGATTGCCTTCGTTTGAGGCCCTGGCAGATGGGGACGTGACCAATGCCAGTGACCAG
AGCTGTCCCTCCTCTCCCGACGGCTCACCGCGCACCCCAGAGCCCGAGGAGCCTTCCCTG
CTGAAGAAACTCCTTCTGGCACCCGCAAACTCCCAGCTCAGCTATAATCAATACACAGGT
GGCAAGGCACAGAACCATGCAGCCAGCAGCAACCACCGGATCAGACCACCACCTGCCGTC
GTCAAGACGGAGAGCCCCTGGAACGGCAAAGCAAGAGGGGGCTCCAGCCAACAGAACCGC
CCGGTGAGGCGGCCCTGCACCGAGCTGCTGAAATACCTAACCGCCACCGATGACATTCTC
CTCCACGCCAAAGCCAGCGAAGCCAAGAGCGCCTGGGGGGGTGCCAGTAGCAGGGAGAAG
AGTGGCATGGGTCTCGGTGCCTCTTCTTCCTCCTCTTCACCGTCCTCGTCATCCACCTCC
TCGTTCTCCTCCCTCTCCTCCACCTCTTCCTCCTCTTCCTCTACCACCTCCAAGAAGAAG
CCGGCTGTGCCGTCTCATCAACAGCAGCAGCAGCAGCCGCCACAGCAGCATCACCAGCGA
GCCAAACCAACCACCTTGCCACTTCCTTTGACCCCAGAGTCTCCAAATGACTACAAGGGA
TCACCGTTTGAGAACAAAACCACTGAACGCACATTAAGTGTGGAGATTGCTGGAACTCCA
GGTTTGACACCACCTACCACGCCCCCACACAAAGCCAGTCAAGAGAATCCTTTCAAAGCA
TCTCTCAAAACCAAGTTGTCTTCATGTTCCTCCTCGGCCTTGGCATGCAAAAGAGCCAGG
CTGAGTGAGTTGGGCCCCGGCGCTCTGGCCCCGGCCCCAGGTGCCTCAGGCAGGGGCCCC
ACCAGGAAGGGTCCTGAACAGACTGAGCTTTACGCCCAGCTGAGCAAAGCAACCACCACC
CTCCCTTACTCCGTCACTCAACACGCAGTGGGGGGCGGCCTTGAGGAGTATCGCAGCACC
GGCAACACTAAGCGGGCAACGCCCCGTAACTACAGCGACCATGACTATTGCCAGGCAACA
GCTGGTAATAAGAAGGATGGGGGCTCAGCCACTGTTACCACAACCGCAGCTGTGAAAATG
ACATTCACCTCAGGTGCCACTGATGCTCCAGTGCCTGCTGAAGGTAAAGTGGAGAGCAGG
CATGTGGAATGTAAGGATTCAGCCATGCCACCGTCATCTTCATCATTTCCTCCATCATCA
GCTTCACCTGGTTTTTTGGCTAAACAGCAGAATTTTGGGTCTGTGGATGGAGAGGCGGGC
CAGGTCCGGGGGTTAGGGGAGCACGCCCTCACACAAACCGCTCAGATCCCCTCACAAGAG
GCCACTATTGACAGGGACCAACACAATCTTTGCGCCACCAGCCGAAAGCTCCTGTGCGAC
CAGGAAATCCGAGCAGAACTTAACAAGCACTTTGGCTACCCTTTGCAAGCCCTCTACACC
CCGGGTAGCCAGGAGAAAGAATCAAGCAGCAAAACGAACAATGCTACAGCTCCTCAGTCC
CTCGAGGGGGGAGAGAATGACTGCTACCCCCAGAGGCTGCCTGCCTCCAGCTACCTTCAC
CCGGGGTTTCTGGCCTTCCACGACGAACTAGAGCTGGACGAGAGCCGTGAAAGTCGCTTC
CTCTTTCCATGGGAGGGCACCCCTCTGGATCTACTCTTTGACTGCGCCGCCTGCTCTCCC
TCTTCTTCCCCACCATCCAGCTGCTCCCCTTCACGAGGCTCTGTCTCTCCACCTTCCTCC
CTGCTCCTGTCACCCAGCAGACCTTCCTGCTGGGCCGGCAGCGGGTCCCGCTCCCGTTCC
CGTTCCCAGTCTGGGTCCCGCAGCTCCTCTTCACGATACCGCAGGCGCTCTCTCTCCAGC
TCACCCGATAGACGCCCGTCCTCCTGG
>Turkey_PPARGC1A
ATGGCGTGGGACATGTGCAACCAGGACTCTGTATGGAGTGACATCGAGTGTGCTGCTCTG
GTTGGTGAAGACCAGCCTCTCTGCCCAGACCTCCCAGAACTTGACCTCTCCGAACTAGAT
GTGAACGACCTGGATGCAGACAGCTTCCTGGGGGGGCTCAAGTGGTACAGCGACCAGTCT
GAGGTCATCTCCAACCAGTACAGCAATGAGCCTGCCAACATCTTTGAGAAAATAGATGAA
GAGAATGAGGCAAACTTGCTAGCAGTCCTCACTGAGACACTGGACAGCATCCCTGTGGAT
GAGGATGGATTGCCTTCATTTGATGCACTGACAGATGGAGATGTGACCAATGAACATGAC

GCCAGCCCTTCCCCGATGCCCGACGGCACCCCTCCGCCCCAGGAGGCAGAAGAGCCGTCT
CTACTCAAGAAGCTCTTGCTGGCTCCAGCCAACACTCAGCTAAATTACAATGAATGCAGT
GGTCTCAGCACACAAAACCATGCAAATACAAATCACAGGATCAGAACAAGCCCTGTGGTT
GTTAAGACTGAGAATTCGTGGAGCAATAAAGCGAAGAGCATTTGTCAACAACAAAAGCCA
CAAAGACGTCCCTGCTCTGAACTTCTCAAATATCTGACTACGAATGATGACCCTCCTCAG
AACAAACCAGCAGAGAACAGGAACAGCAGCAAAGAGAAATGCACCTCCAAAAGGAAGCCC
CATCTGCAGTCTCAGACAAATCACCTGCAAGCCAAACCAACAAGTTTATCACTTCCGTTG
ACGCCTGAGTCTCCAAATGATCCCAAGGGTTCCCCATTTGAGAACAAGACTATTGAACAA
ACCTTAAGTGTGGAACTCTCTGGAACTGCAGGCCTAACTCCACCTACGACCCCTCCTCAT
AAAGCCAACCAGGATAATCCTTTCAGGACTTCACCTAAGCCGAAGTCATCATGCAAGACT
GTTGCACCACCTTCAAAAAAGCCCCGTTATAGTGAGTCTTCCAGTTCTCAAGGAAACAAC
CCAGTCAAGAAGGGTCCAGAACAGACTGAGCTGTATGCACAGCTTAGCAAGACTACAGCA
CTGTCCAGTGGACATGAGGAGAGAAAGACAAAACGGCCCAGTTTGCGGCTGTTTGGTGAC
CATGACTACTGTCAATCTGTGAATTCAAAGTCGGAAATACACATTAAAATATCCCAGGAA
CTTCAGGACTCCAGACAACTAGAATTTAAGGATTCTTCACCTGGGTGGCAGTGTCAGATT
TGTTCTTCTTTAGAACAAGACCAGTATTTCAAGAAAGAGACTTTACAGACAAGTAAGCAG
GGATCCCAAGGTAATAACAGAAAACAGCTCCAAGACCAAGAAATTCGGGCTGAACTGAAT
AAGCATTTTGGTCACCCCAGCCAAGCTGTTTTTGATGAAGAAGCAGATAAGACCGGTGAA
CTAAGGGACAGTGATTACAGTAATGAACAATTTTCCAAACTACCTATGTTTATAAATTCA
GGACTAGCAATGGATGGTCTCTTTGATGACAGTGAAGATGAAAGTGATAAACTATGCTAC
CCTTGGGATGGGACACAAGCCTATTCATTATTTGACGTATCGCCTTCTTGCTCTTCTTTT
AACTCTCCATGCAGAGATTCAGTATCTCCACCCAAATCCTTATTTTCTCAAAGATCCCAA
AGGACACGCTCTAGATCAAGGTCCTTTCCTCAACGCAGGTCTTGTTCCCGTTCTCCATAT
TCCCGATCGAGATCAAGGTCACCCTGTAGTAGATCCTCTTCAAGATCTTGCCACTGTTAT
GAGTCCAGCCACTGTAGACACCAAGCACACCGAAGTTCTCCCTCACGTGCAAGGTCGCGA
TCCAGATCACCGTACAGTCGCAGACCCAGATATGACAGCTATGAGGAATATCAGCATGAA
AGGCTGAAGAGGGAAGAATACCGCAAAGAGTATGAAAAACGGGAATCTGAAAGGGCCAAA
CAAAGGGAGAGACAGAGGCAGAAAGCAATTGAAGAGCGTCGTGTGATTTACGTGGGTAAA
ATCAGACCTGACACAACCCGAAAAGAACTGAGGGACCGGTTTGAAGTTTTTGGTGAAATA
GAGGAGTGCACAGTAAATTTGCGGGATGATGGAGACAGCTATGGTTTCATCACCTACCGC
TATACTTGTGATGCCTTTGCTGCTCTTGAAAATGGATACACTTTACGCAGGTCAAATGAG
CCTGACTTTGAGCTGTACTTTTGTGGACGCAAGCAGTTTTGCAAGTCTAACTATGCAGAC
CTAGATTCAAACTCAGATGATTTTGATCCTGCTTCCACTAAAAGCAAGTATGACTCCATG
GATTTTGATAGTTTACTTAAAGAGGCACAACGGAGCCTGCGCAGG
>Zebra_finch_PPARGC1A
ATGGCGTGGGACATGTGCAACCAGGACTCTGTATGGAGTGATCTCGAGTGTGCTGCTCTG
GTTGGTGAAGACCAGCCTCTTTGCCCAGATCTCCCAGAACTTGACCTCTCTGAACTAGAT
GTGAACGACCTGGATGCGGACAGCTTTCTGGGGGGACTCAAGTGGTACAGCGACCAGTCA
GAGATCATCTCCAATCAGTACAGCAATGAACCCGCCAATATATTCGAGAAGCTAGATGAA
GAGAGCGAGGCAAACTTGCTAGCTGTTCTCACTGAAACATTGGACAGCATCCCTGTGGAT
GAGGATGGATTGCCTTCATTTGATGCACTGACAGATGGAGATGTGACCAACGAAAATGCC
GCTAGCCCCTCCCCAGTGCCCGACGGCGCCCCTCCAACTCAGGAGGCAGAAGAGCCGTCT
CTACTTAAGAAGCTCTTGCTGGCTCCAGCCAACACTCAGCTAAATTACAATGAATGCAGT
GGTCTCGGCACACAAAACCATGCAAACACTAATCACAGGATCAGAACAAGCCCTGTGGTT

GTTAAGACTGAGAATTCATGGAGCAATAAAGCGAAGAGCATTTGTCAACAGCAAAAGCCT
CAAAGACGTCCCTGCTCTGAACTTCTCAAATATCTGACTACAAATGATGATCCTCCTCAC
ACTAAACCAGCAGAGAACAGGAACAGCAGCAAAGAGAAATGCACCTCCAAAAGGAAGCCC
CATCTGCAGTCTCAGACAAATCATCTGCAGGCCAAACCAACAAGTTTATCACTTCCATTG
ACACCCGAGTCACCAAATGATCCCAAGGGTTCCCCATTTGAGAACAAGACTATTGAACAA
ACCTTAAGTGTGGAACTCTCTGGAACTGCAGGCCTAACTCCACCGACGACCCCTCCTCAT
AAAGCCAACCAAGATAATCCTTTTAGGACTTCACCTAAACTGAAGTCATCATGCAAGACT
GTTGTACCACCTTCAAAAAAGCCCCGCTATAGTGAGTCTTCCAGTTCTCCAGGAAATAAC
CCAATCAAGAAGGGTCCAGACGAGTCTGAGCTGTACCCACAGCTTAGCGAGACTACAGTA
CTGTCCAGTGGACATGAGGAGAGAAAGACAAAACGGCCCAGTTTGCGGCTGTTTGGTGAC
CATGACTACTGTCAATCTGTGAATTCAAAATCGGAAATACACATTAAAATATCCCAGGAA
CTTCAGGACTCCAGACAACCAGAATTTAAGGATTCTTCACCTGGGTGGCAGTGTCAGATT
TGTTCTTCTTTAGAACAAGACCAGAATTTCAAGAAAGAGACTTTACAGACAAATAAGCAG
GGATCCCATGGTAATAACAGAAAACAGCTCCAAGACCAGGAAATTCGGGCTGAACTGAAT
AAGCATTTTGGTCACCCCAGCCAAGCTGTTTTTGATGAAGAAGCAGATAAGAGCAGAGAA
CTAAGGGACAGTGATTACAGTAATGAACAATTCTCCAAACTACCTATGTTTATAAATTCA
GGACTAGCAATGGATGGTCTGTTTGATGACAGTGAAGATGAAAGTGATAAACTATGCTAC
CCTTGGGATGGGACACAAGCCTATTCATTATTTGATGTATCACCTTCTTGCTCTTCTTTT
AACTCTCCATGCAGAGATTCAGTGTCTCCACCCAAATCCTTATTTTCTCAAAGATCCCAA
AGGACACACTCTAGATCAAGGTCCTTTCCTCAACGCAGGTCTTGTTCCCGTTCTCCATAT
TCCCGATCGAGATCAAGGTCGCCCTGTAGTAGATCCTCTTCAAGATCTTGTTGCTATTGT
GAGTCCAGCCACTGTAGACACCGAGCATACAGAAGTTCTCCCTTAGGTGGAAGATCGCGG
TCCAGATCACCGTACAGTCGCAGACCCAGATATGACAGCTATGAGGAATATCAGCATGAA
AGGCTGAAGAGGGAAGAATACCGCAAAGAGTATGAAAAACGGGAATCTGAAAGGGCCAAG
CAAAGGGAGAGACAGAGGCAGAAAGCAATTGAAGAACGTCGTGTGATTTATCTGGGTAAA
ATCAGACCTGACACAACCCGAACAGAACTGAGGGACCGGTTTGAAGTTTTTGGTGAAATT
GAGGAGTGCACAGTAAATTTGCAGGATGATGGAGACAGCTATGGTTTCATCACCTACCGC
TATACTTGTGACGCCTTCGCTGCTCTTGAAAATGGATACACTTTACGCAGGTCAAATGAA
CCTGATTTTGAGCTGTACTTCTGTGGACGCAAGCAGTTTTGCAAGTCTAACTATGCAGAC
CTAGATTCAAACTCAGATGATTTTGATCCTGCTTCTACTAAAAGCAAGTATGACTCCATG
GATTTTGATAGTTTACTCAAAGAGGCCCAGCGGAGCCTGCGTAGG
>Zebrafish_PPARGC1A
ATGGCGTGGGACAGGTGTAATCAGGATTCGGTGTGGAGAGAACTAGAGTGCGCTGCCTTG
GTTGGTGAAGACCAGCCCCTTTGCCCTGACCTGCCTGAGCTTGACCTTTCTGAGCTGGAT
GTCAGCGACCTCGACGCGGATAGCTTTCTGGGAGGACTCAAGTGGTACAGCGACCAATCA
GAAATCATTTCCAGTCAGTATGGCAATGAAGCATCCAACCTGTTTGAGAAGATAGATGAG
GAAAATGAGGCCAACTTGCTGGCAGTGCTCACAGAAACCCTGGACAGTATCCCAGTGGAT
GAAGACGGGTTGCCTTCGTTCGAAGCCCTGGCAGATGGGGACGTGACCAATGCCAGTGAT
CAGAGCTGTCCTTCTACCCCTGACGGCTCGCCACGCACCCCAGAGCCAGAGGAGCCTTCC
CTGCTGAAGAAGCTCCTCCTGGCCCCTGCTAACTCCCAGCTCAGCTATAATCAATACCCA
GGTGGCAAGGCACAGAACCATGCAGCCAGCAACCAACGGATCAGACCAGCACCTGCTGTT
GCCAAGGTAGCTGCTCACCGCACTTCTTGCATCAAAGGCCTGACAGAAAACCCCTGGAAC
AGCAAACCACGAGGGGCCTGTCCCAACCGGTCCATGAGACGTCCCTGCACTGAGCTGCTC
AAGTACCTCACCTCTAGCGACGAGGCCTTCCAGACCAAAGCCGGTGAAGCCAAGAGCACC

TGGACAGGTTGCGGCAAGGACAGGGGAGGTGCTTGCATCTCATCCTGCTCGTCTTCTTCC
TCTCCATCGTCCTCGTCCACCTCCTCGTTCTCCTCCCTGTCGTCCTGCTCGTCTTCCACC
GCCTCCAAAAAGAAGACGTCCTCTGCCTCCCCATCATCACAGCAGCAGCAGCTGGCAGTG
CAGGCCCAGCGAGCCAAACCAACCATCTTGCCACTTCCTTTGACCCCAGAGTCTCCAAAT
GACCACAAGGGATCTCCGTTTGAGAACAAAACCATTGAACGCACACTGAGTGTGGAGATC
TGTGGAACCCCAGGTCTGACACCACCTACCACGCCTCCTCACAAAGCCAGTCAAGAGAAC
CCTTTCAAAGTATCACTCAAAAACAAGCTGTCTTCATGCTCTCCCTCGGCCCTGACAAGC
AAAAGGCCCAGGCTGAGTAATGGGGGCTCTTGCCCTCAGCCAACCAGCGGCTCTATTCGG
AAGGGCCCAGAGCAGACTGAGCTCTATGCCCAGCTGAGCAAGGCGTCCTCCACTATGCCC
CAAGGGGGCTTGGAGGATCGTCGGGGCAAGCGGCCCATGCCCCGTGTCTTTGGCGATCAT
GACTATTGCCAATCTACAAGCACAAAACGAGACAGCACCACCCCAGCTGCAGTGGTACCC
GGGCCAACAGAGGGCCGGCATGTGGAATGTAAAGACTTAAACATGCCAACCTCCACTACT
ACGACATCATCGTTGTCTTCCACCCCCCCTTCGTCTTCCTCACTGGCCAGGCAGCTTCAA
GGCCTTTCCCCAACACCTCAGGAGGCTTGTCCGGACACATATGCTCACGTGCAGCACCAC
GACTCAAGCTCCAAAATGACAATGGACTGCAGTTCTGGTGGCAGGAAACTTCTTAGGGAC
CAGGAGATCCGGGACGAGCTCAACAAGCACTTTGGAAAGCCTCAGCAAGCCTTCTATAGC
GGGGTAGTGGGAGAGCCGAGGGGCAAACAGCCAATTGAGGACAGTGACTCTGGGGATGAG
TACCCGGGTCTACTCGGCGACTACATCCACCCAGGTCTGCCTGATTTCGAGGACCTGGAG
GTAGGCCGGGAGCGCCTGTTCTACTTGGGGGAAGGTTCTCCACTCGAGCTGCTCCTCGAA
GGGTCACCCTCCAGCTCCCCTTCCAGCAGTTCCTTTTCATGGTGCTCTGTCTCGCCTCCT
TCCTCTCAGCTCTCCCCACAGCACCTCCGCTGGCCACGCTCCATCTCCCGCTCCCGTTCT
CGTTCTTCATCTCACCACAGGCGCAGATCCCTCTCCAGGTCTCCCTACTCCCGCTCCGGG
TCTCCCAGCAGCCGTTCTCCCTCTTGGTCTCCTCGCAACATGGACGAAAGCACTTTCACT
CCCAGGATTTGTGGAAACCCTCAGTCCCAGTCGCATTCTCTTTTTGGTCGGAGACCCAGG
TATGACAGCTATGAGGAATACCAGCACGAGCGTCTGAAGCGAGAGGAGTACCGACGCGAC
TATGAGAAACGGGAATGTGAGAGGGCCGAGCAGAGGGAGAGACAACGGCAAAAAGCAATA
GAGGAGAGGCGAGTGGTGTATGTGGGACGTCTTCGCGCCGACAGCACACGCACCGAGCTC
AAACGCCGCTTTGAAGTCTTCGGCGAGATTGAGGAGAGCACAGTCAACCTGAGACATGAC
GGGGATAACTTTGGCTTCATCACCTACCGCTACACTTGTGATGCTCTCGCTGCCCTTGAG
AATGGACACACTTTGCGCAGGTCGAACGAACCTCACTTTGAGCTCTGCCTTGGTGGACAA
AAGCAGTACAGCAAATCCAATTACACAGACTTGGATTCCCACTCTGACGACTTTGATCCA
GCCTCCACTAAAAGCAAGTACGACTCCATGGATTTCGACAGCTTGTTGCGAGAAGCCCAG
TACAGCCTGAGAAGG
>Pig_PPARGC1A
ATGGCGTGGGACATGTGCAACCAGGACTCTGTATGGACTGACATCGAGTGTGCTGCTCTG
GTTGGTGAAGACCAGCCTCTTTGCCCAGATCTTCCTGAACTTGACCTTTCTGAACTAGAC
GTGAACGACTTGGATACAGACAGCTTTCTGGGTGGACTCAAGTGGTGCAGTGACCAATCA
GAAATAATATCCAATCAGTACAACAATGAGCCTTCAAACATATTTGAGAAGATAGATGAA
GAGAATGAGGCAAACTTGCTAGCAGTTCTCACAGAGACGCTGGACAGTCTCCCTGTGGAT
GAAGACGGATTGCCCTCATTTGATGCACTGACAGATGGAGATGTGACCACTGAGAATGAG
GCTAGTCCTTCCTCCATGCCTGACGGCACCCCTCCGCCTCAGGAGGCAGAAGAGCCGTCT
CTACTTAAGAAGCTCTTACTGGCACCAGCCAACACTCAGCTAAGTTATAATGAATGCAGT
GGTCTCAGTACCCAGAACCATGCAAACCACAATCACAGGATCAGAACAAACCCTGCAGTT
GTTAAGACCGAGAATTCATGGAGCAATAAAGCGAAGAGCATTTGTCAACAGCAAAAGCCA

CAAAGACGTCCATGCTCCGAGCTTCTCAAGTATCTGACCACAAATGATGACCCTCCTCAC
ACCAAACCCACAGAGACCCGAAACAGTAGCAGAGACAAGTGCACCTCCAAAAAGAAGGCC
CACACACAATCGCAGTCGCAACATTTACAAGCCAAACCAACATCTTTATCTCTTCCTCTG
ACCCCAGAGTCACCAAATGACCCCAAGGGTTCCCCATTTGAGAACAAGACTATTGAACGA
ACCTTAAGTGTGGAACTCTCTGGAACTGCAGGCCTAACTCCACCCACAACTCCTCCTCAT
AAAGCCAACCAAGATAACCCTTTTAGGGCTTCTCCAAAGCTGAAGCCCCCTTGCAAGACT
GTGGTACCTCCGCCATCGAAGAAGACCCGGTACAGTGAGTCTTCGGGGACCCACGGCAAC
AACTCCACCAAGAAAGGGCCCGAGCAGTCCGAGCTGTACGCGCAGCTCAGCAAGACGTCC
GCGCTCGGCGGCGGACACGAGGAACGGAAGGCCAGGCGGCCCAGTCTGCGGCTATTTGGT
GACCATGACTATTGTCAGTCGATTAATTCCAAAGCGGAAATCCTCATCAATATATCGCAG
GAGCTCCACGACTCCAGACAACTAGACTCTAAAGATGCCGCCTCTGACTGGCAGAGGCAG
ATGTGTTCTTCCACAGACTCAGACCAGTGCTACCTGACCGAGACGTCGGAGGCGAGCAGG
CAGGTCTCTCCGGGCAGCGCCCGAAAACAGCTCCAAGACCAGGAAATCCGAGCCGAGCTG
AACAAGCACTTCGGTCATCCCAGTCAAGCTGTTTTTGACGACGAAGCAGACAAGACCAGT
GAACTGAGGGACAGTGATTTCAGTAACGAACAATTCTCCAAACTACCTATGTTTATAAAT
TCAGGACTAGCCATGGATGGCCTGTTTGATGACAGCGAAGATGAAAGTGATAAACTGAAC
TCCCCTTGGGATGGCACGCAGTCCTATTCATTGTTCGATGTGTCGCCTTCTTGTTCTTCT
TTTAACTCTCCGTGTAGAGATTCCGTATCACCACCCAAATCCTTATTTTCTCAAAGACCC
CAAAGGATGCGCTCTCGTTCAAGGTCCTTTTCTCAACACAGGTCGTGTTCTCGATCACCA
TATTCCAGGTCAAGATCAAGGTCCCCAGGCAGTAGATCCTCTTCAAGATCTTGCTACTAC
TCTGAGTCAGGCCACTGCAGACACCGCACGCACCGAAATTCTCCCCTGTGCGCCAGATCA
CGTTCAAGATCTCCCTACAGCCGGCGGCCCAGGTATGACAGCTACGAGGAATATCAGCAC
GAGAGGCTGAAGAGGGAAGAATACCGCAGAGAGTATGAGAAGCGGGAGTCTGAAAGGGCC
AAGCAGAGGGAGAGGCAGAGGCAGAAGGCAATTGAAGAACGTCGTGTGATTTATGTTGGT
AAAATCAGACCTGACACAACACGGACAGAACTGAGGGACCGTTTTGAAGTTTTTGGTGAA
ATTGAGGAGTGCACAGTAAATCTGCGGGATGATGGAGACAGCTATGGTTTCATTACCTAC
CGTTATACCTGTGATGCTTTTGCTGCTCTTGAAAATGGATACACTTTGCGCAGGTCGAAT
GAAACTGACTTCGAGCTGTACTTTTGTGGACGCAAGCAATTTTTCAAGTCTAACTATGCA
GACCTAGATTCAAATTCAGATGACTTTGATCCTGCTTCCACCAAGAGCAAGTATGACTCT
CTGGATTTCGATAGTTTACTGAAAGAAGCTCAGAGAAGCTTGCGCAGG
>Elephant_PPARGC1A
ATGGATGAGACCTCTCCCAGGCTGGATGAAGACTGGAAAAAAGGACTTCAGCGAGAAGCA
AGCTGGCCGTGTGCTGCTCTGGTTGGTGAAGACCAGCCTCTTTGCCCAGATCTTCCTGAA
CTTGACCTTTCTGAACTAGACGTGAACGACTTGGATACAGACAGCTTTCTGGGTGGACTC
AAGTGGTGCAGTGACCAATCAGAAATAATTTCCAACCAGTACAACAATGAGCCTTCAAAT
ATATTTGAGAAGATAGATGAAGAGAGTGAGGCAAACTTGCTAGCAGTCCTCACAGAGACA
CTGGACAGTCTCCCTGTGGATGAAGACGGATTGCCCTCGTTTGATGCGCTGACAGATGGA
GATGTGACCACTGACAATGACGCTAGTCCTTCCTCTATGCCCGACGGCACCCCTCCGCCT
CAGGAGGCAGAAGAGCCATCTCTACTTAAGAAGCTCTTACTGGCACCAGCCAATACTCAG
CTAAGTTATAGTGAATGCAGTGGTCTCAGTACCCAGAACCATGCAAACCATAATCACAGG
ATCAGAACAAACCCTGCAGTTGTTAAGACCGAGACTTCATGGAGCAATAAAGCAAAGAGC
ATTTGTCAACAGCAAAAGCCACAAAGACGTCCCTGCTCCGAGCTTCTCAAGTATCTGACC
ACAAACGATGACCCTCCTCACACCAAACCCACAGAGAACAGAAACAGCCACAGAGACAAA
TGCACCTCGAAAAAGAAGCCCCACACACAGTCGCAGGTGTCCCATTTACAAGCCAAACCA

ACAACTTTATCTCTTCCTCTGACCCCAGAGTCACCAAATGACCCCAAGGGTTCCCCATTT
GAGAACAAGACTATTGAACGAACCTTAAGTGTGGAACTCTCTGGAACTGCAGGCCTAACT
CCACCCACAACTCCTCCTCATAAAGCCAACCAAGATAACCCTTTTAGGACTTCTCCAAAA
TTGAAGTCCTCTTGCAAGACTGTGGTACCACTACCATCAAAGAAGCCCCGGCATAGCGAG
TCTTCTGGTACCCAAGGAAACAACTCCACCAAGAAAGGTCCCAAGCAATCTGAGTTGTAC
GCACAACTCAGCAAGACCTCGGTACTCACCAGTGGACACGAGGAAAGGAAGACCAAGCGG
CCCAGTCTGCGGTTGTTTGGTGACCATGACTATTGTCAGTCAATTAATTCCAAATCGGAA
ATACTCATTAATATATCACAGGAGCTCCAAGACTCTAGAAAACTAGAATATAAAGATGCC
TCCAGTGATTGGCAGGGGCAGATTTGTTCTTCCACAGACTCAGACCAGTGCTACCTGAGA
GAGACTTTGGAGGCCAGCAAGCAAGTCTCTCCTGGCAGCACCAGAAAACAGCTCCAAGAC
CAGGAAATCCGAGCCGAGCTGAACAAGCACTTTGGTCATCCCAGTCAAGCTGTTTTTGAC
GACGAAGCAAACAAGACCAGTGAACTGAGGGACAGTGATTTTAGTAATGAACAATTCTCC
AAACTACCTATGTTTATAAATTCAGGACTAGCCATGGATGGCATGTTTGAGGACAGCGAA
GATGAAAGTGATAAACTGAGCTACCCTTGGGATGGCACGCAATCCTATTCATTGTTCGAT
GTGTCGCCTTCTTGCTCTTCATTTAACTCTCCGTGTAGAGATTCCGTGTCGCCACCCAAA
TCCTTATTTTCTCAAAGACCCCAAAGGATGTGCTCTCGTTCAAGGTCCTTTTCTCGACAC
AGGTCCTGTTCCCGGTCACCGTATTCCAGGTCAAGATCTAGGTCCCCAGGCAGTAGATCC
TCTTCAAGATCTTGCCACTACTGTGAGTCAAGCCACTGCAGACACCGCACGCACCGAAAT
CCTCCCTTGTGCGGGAGATCACGCTCCAGATCACCCTGCAGCCGTAGGCCCAGGTATGAC
AGCTACGAGGAGTATCAGCACGAAAGGCTGAAGAGGGAAGAATACCGCAAAGAGTATGAG
AAGCGGGAGTCTGAAAGGGCCAAACAAAGGGAGAGACAGAGGCAGAAGGCAATTGAAGAA
CGCCGTGTGATATATGTTGGTAAAATCGGATCTGACACAACACGGACAGAACTGAGGGAC
CGTTTTGAAGTTTTTGGTGAAATTGAGGAGTGCACAGTAAATCTGCGGGATGATGGAGAC
AGCTATGGTTTCATTACCTACCGTTATACCTGCGATGCTTTTGCTGCTCTTGAAAATGGA
TACACTTTGCGCAGGTCGAATGAAACTGACTTCGAGCTGTACTTTTGTGGACGCAAGCAA
TTTTTCAAGTCTAACTATGCAGACCTAGATTCAAACTCTGATGACTTTGACCCTGCTTCC
GCCAAGAGCAAGTATGACTCTCTGGATTTCGATAGTTTACTGAAAGAGGCTCAGAGAAGC
TTACGCAGG
>Chicken_PPARGC1A
ATGGCGTGGGACATGTGCAACCAGGACTCTGTATGGAGTGACATCGAGTGTGCTGCTCTG
GTTGGTGAAGACCAGCCTCTTTGCCCAGATCTCCCAGAACTTGACCTCTCCGAACTAGAC
GTGAACGACCTGGATGCAGACAGCTTCCTGGGGGGGCTCAAGTGGTACAGCGACCAGTCT
GAGGTCATCTCCAGCCAGTACAGCAATGAGCCTGCCAACATCTTTGAGAAAATAGATGAA
GAGAATGAGGCAAACTTGCTAGCAGTTCTCACTGAGACACTGGACAGCATCCCTGTGGAT
GAGGATGGATTGCCTTCATTTGATGCACTGACAGATGGAGATGTGACCAATGAACATGAC
GCCAGCCCTTCCCCGATGCCCGACGGCACCCCTCCGCCCCAGGAGGCAGAAGAGCCGTCT
CTACTCAAGAAGCTCTTGCTGGCTCCAGCCAACACTCAGCTAAATTACAATGAATGCAGT
GGTCTCAGCACACAAAACCATGCGAACACAAATCACAGGATCAGAACAAGCCCTGTGGTT
GTTAAGACTGAGAATTCGTGGAGCAATAAAGCGAAGAGCATTTGTCAACAACAAAAGCCA
CAAAGACGTCCCTGCTCTGAACTTCTCAAATATCTGACTACGAACGATGACCCTCCTCAG
ACCAAACCAGCAGAGAACAGGAACAGCAGCAAAGAGAAATGCACCTCCAAAAGGAAGCCC
CATCTGCAGTCTCAGACAAACCACCTGCAAGCCAAACCAACAAGTTTATCACTTCCATTG
ACACCTGAGTCTCCAAATGATCCCAAGGGTTCCCCATTTGAGAACAAGACTATTGAACAA
ACCTTAAGTGTGGAACTCTCTGGAACTGCAGGCCTAACTCCACCTACGACCCCTCCTCAT

AAAGCCAACCAAGATAATCCTTTCAGGACTTCACCTAAGCCGAAGTCATCATGCAAGACT
GTTGCACCACCTTCAAAAAAGCCCCGTTATAGTGAGTCTTCCGGTTCTCAAGGAAACAAC
CCTGTCAAGAAGGGTCCAGAACAGACTGAGCTGTATGCACAGCTTAGCAAGACTACAGCA
CTGTCCAGTGGACATGAGGAGAGAAAGACAAAACGGCCCAGTTTGCGGCTGTTTGGTGAC
CATGACTATTGTCAATCTGTGAATTCAAAGTCGGAAATCCACATTAAAATATCCCAGGAA
CTTCAGGACTCCAGACAACTAGAATTTAAGGATTCTTCACCTGGGTGGCAGTGTCAGATT
TGTTCTTCTCTAGAACAAGACCAGTATTTCAAGAAAGAGACTTTACAGACAAGTAAGCAG
GGATCCCAAGGTAATAACAGAAAACAGCTCCAAGACCAGGAAATTCGGGCTGAACTGAAT
AAGCATTTTGGTCACCCCAGCCAAGCTGTTTTTGATGAAGAAGCAGATAAGACCGGTGAA
CTAAGGGACAGTGATTACAGTAATGAACAATTTTCCAAACTACCTATGTTTATAAATTCA
GGACTAGCAATGGATGGTCTCTTTGATGACAGTGAAGATGAAAGTGATAAACTATGCTAC
CCTTGGGATGGCACACAATCCTATTCATTATTTGACGTATCGCCTTCTTGCTCTTCTTTT
AACTCTCCATGCAGAGATTCAGTATCTCCACCCAAATCCTTATTTTCTCAAAGATCCCAA
AGGACACGCTCTAGATCAAGGTCCTTTCCTCAACGCAGGTCTTGTTCCCGTTCTCCATAT
TCCCGATCGAGATCAAGGTCACCCTGTAGTAGATCCTCTTCAAGATCTTGTCACTGTTAT
GAGTCCAGCCACTGTAGACACCGAGCACACCGAAGTTCTCCCTCACGTGCAAGATCGCGA
TCCAGATCACCGTACAGTCGCAGACCCAGATATGACAGCTATGAGGAATATCAGCATGAA
AGGCTGAAGAGGGAAGAATACCGCAAAGAGTATGAAAAACGGGAATCTGAAAGGGCCAAA
CAAAGGGAGAGACAGAGGCAGAAAGCAATTGAAGAGCGTCGTGTGATTTACGTGGGTAAA
ATCAGACCTGACACAACCCGAAAAGATCTGAGGGACCGGTTTGAAGTTTTTGGTGAAATC
GAGGAGTGCACAGTAAATTTGCGGGATGATGGAGACAGCTATGGTTTCATCACCTACCGC
TATACTTGTGATGCCTTTGCTGCTCTTGAGAATGGATACACTTTACGCAGGTCAAACGAG
CCTGACTTTGAGCTGTACTTTTGTGGACGCAAGCAGTTTTGCAAGTCTAACTATGCAGAC
CTAGATTCAAACTCAGATGATTTTGATCCTGCTTCCACTAAAAGCAAGTATGACTCCATG
GATTTTGATAGTTTACTTAAAGAGGCACAGCGGAGCCTGCGCAGG
>Rock_pigeon_PPARGC1A
ATGGCGTGGGACATGTGCAACCAGGACTCTGTATGGAGTGATCTGGAGTGTGCTGCTCTG
GTTGGCGAAGACCAGCCTCTTTGCCCGGATCTCCCAGAACTTGACCTCTCCGAATTAGAT
GTGAACGACCTGGATGCAGACAGCTTTCTGGGAGGACTCAAGTGGTACAGCGACCAGTCA
GAGATCATCTCCAATCAATACAGCAATGAACCTGCCAACATATTCGAGAAGATAGATGAA
GAGAGTGAGGCAAACTTGCTAGCCGTTCTCACTGAAACACTGGACAGCATCCCTGTGGAT
GAGGATGGGTTGCCTTCATTTGATGCACTGACAGATGGAGATGTGACCAACGAAAATGCC
GCTAGCCCTTCCCCAATGCCCGACGGCACCCCTCCAACTCAGGAGGCAGAAGAGCCGTCT
CTACTTAAGAAGCTCTTGCTGGCTCCAGCCAACACTCAACTAAGTTACAATGAATGCAGT
GGTCTCAGCACACAAAACCATGCAAACACTAATCACAGGATCAGAACAAGCCCTGTGGTT
GTTAAGACTGAGAATTCATGGAGCAATAAGGCGAAGAGCATTTGTCAACAGCAAAAGCCT
CAAAGACGTCCCTGCTCTGAACTTCTCAAATATCTGACTACAAATGATGACCCTCCTCAG
ACCAAACCAGCAGAGAACAGGAACAGCAGCAAAGAGAAATGCACCTCCAAAAGGAAGCCT
CATCTGCAGACTCAGACAAATCATCTGCAAGCCAAACCAACAAGTTTATCACTTCCATTG
ACACCCGAGTCACCAAATGATCCCAAGGGTTCCCCATTTGAGAACAAGACTATTGAACAA
ACCTTAAGTGTGGAACTCTCTGGAACTGCAGGCCTAACTCCACCTACGACCCCTCCTCAT
AAAGCCAACCAGGATAATCCTTTTAGGACTTCACCTAAGCCGAAGTCATCATGCAAGACT
GTTGTACCACCTTCCAAAAAGCCCCGCTATAGTGAGTCTTCTGGTTCTCAAGGAAATAAC
CCAACCAAGAAGGGTCCAGAACAGTCTGAGCTGTATGTACAGCTTAGCAAGACTACAGGA

CTGTCCAGTGGACATGAGGAGAGAAAGACAAAACGGCCCAGTTTGCGGCTGTTTGGTGAC
CATGACTACTGTCAATCTGTGAATTCAAAATCGGAAATACACATTAAAATATCCCAGGAA
CTTCAGGACTCCAGACAACTAGAATGTAAGGATTCTTCACCTGGGTGGCAGTGTCAGATT
TGTTCTTCTTTAGAACAAGACCAGTATTTCAAGAAAGAGACTTTACAGACAAGTAAGCAG
GGATCCCATGGTAATAACAGAAAACAGCTCCAAGACCAGGAAATTCGGGCTGAACTGAAT
AAGCATTTTGGTCACCCCAGCCAAGCTGTTTTTGATGAAGAAGCAGATAAGACAAGAGAA
CTAAGGGACAGTGATTACAGTAATGAACAATTCTCCAAACTACCTATGTTTATAAATTCA
GGACTAGCAATGGATGGTCTCTTTGATGACAGCGAAGATGAAAGTGATAAACTATGCTAC
CCTTGGGATGGGACACAAGCCTATTCATTATTTGATGTATCGCCTTCTTGCTCTTCTTTT
AACTCTCCATGCAGAGATTCAGTGTCTCCACCCAAATCCTTATTTTCTCAAAGATCCCAA
AGGACACTCTCTAGATCAAGGTCCTTTCCTCAACGCAGGTCTTGTTCCCGTTCTCCATAT
TCCCGATCGAGATCAAGGTCACCCTGTAGCAGATCCTCTTCAAGATCTTGTTGCTATTAT
GAGTCCAGCCACTGTAGACACCGAGCACACAGAAGTTCTCCCTCACATGCAAGATCGCGA
TCCAGATCACCGTGCAGTCGCAGACCCAGATATGACAGCTATGAGGAATATCAGCATGAA
AGGCTGAAGAGGGAAGAATACCGCAAAGAGTATGAAAAACGGGAATCTGAAAGGGCCAAA
CAAAGGGAGAGACAGAGGCAGAAAGCAATTGAGGAACGTCGTGTGATTTATCTGGGTAAA
ATCAGACCTGACACAACCCGAACAGAACTGAGGGACCGGTTCGAAGTTTTTGGTGAAATT
GAGGAGTGCACAGTAAATTTGCAGGATGATGGAGACAGCTATGGTTTCATCACCTACCGC
TATACTTGTGACGCCTTTGCTGCTCTTGAAAATGGATACACTTTACGCAGGTCAAATGAA
CCTGACTTTGAGCTGTACTTTTGTGGACGCAAGCAATTTTGCAAGTCTAACTATGCAGAC
CTAGATTCAAACTCAGATGATTTTGATCCTGCTTCCACTAAAAGCAAGTATGACTCCATG
GATTTTGATAGTTTACTTAAAGAGGCACAGCGGAGCCTGCGTAGG
>Flycatcher_PPARGC1A
ATGGCTGGCTGCGGGCTGTCGGAAGACGCTTGCTTCTCCTCCTTCTTTTACAACTACACC
TCCTCCTGGGAGACCTCCTACAACCAGTGTGCTGCTCTGGTTGGCGAAGACCAGCCTCTT
TGCCCAGATCTCCCAGAACTTGACCTCTCTGAACTAGATGTGAACGACCTGGATGCAGAC
AGCTTTCTGGGGGGGCTCAAGTGGTACAGTGACCAGTCAGAGATCATCTCCAACCAGTAC
AGCAATGAACCCGCCAATATATTCGAGAAGATAGATGAGGAGAGCGAGGCAAACTTGCTA
GCTGTTCTCACTGAAACACTGGACAGCATCCCTGTGGATGAGGATGGATTGCCTTCCTTT
GATGCACTGACAGATGGAGATGTGACCAACGAGAATGCGGCCAGCCCCTCCCCGCTGCCC
GACGGCGCCCCGCCCGCTCCGGAGGCAGAAGAGCCGTCTCTACTTAAGAAGCTCTTGCTG
GCTCCAGCCAACACTCAGCTAAATTACAATGAATGCAGTGGTCTCAGCACACAAAACCAT
GCAAACACTAATCACAGGATCAGAACAAGCCCTGTGGTTGTTAAGACTGAGAATTCATGG
AGCAATAAAGCGAAGAGCATTTGTCAACAGCAAAAGCCTCAAAGACGTCCCTGCTCTGAA
CTTCTCAAATATCTGACTACGAATGATGATCCTCCTCAGACCAAACCAGCAGAGAACAGG
AACAGCAGCAAAGAGAAATGCACCTCCAAAAGGAAGCCCCATCTGCAGTCTCAGACAAAT
CATCTGCAGGCCAAACCAACAAGTTTATCACTTCCATTGACACCCGAGTCACCAAATGAT
CCCAAGGGTTCCCCATTTGAGAACAAGACTATTGAACAAACCTTAAGTGTGGAACTCTCT
GGAACTGCAGGCCTAACTCCACCTACGACCCCTCCTCATAAAGCCAACCAAGATAACCCT
TTTAGGACTTCACCTAAACTGAAGTCATCATGCAAGACTGTTGTACCACCTTCAAAAAAG
CCCCGCTATAGTGAGTCTTCCGGTTCTCAAGGAAATAACCCAACCAAGAAGGGTCCAGAA
CAGTCTGAGCTGTACACACAGCTTAGCAACACTACAGTACTGTCCAGTGGACATGAGGAG
AGAAAGACAAAACGGCCCAGTTTGCGGCTGTTTGGTGACCATGACTACTGTCAATCTGTG
AATTCAAAATCGGAAATACACATTAAAATATCCCAGGAACTTCAGGACTCCAGACAACCA

GAATTTAAGGATTCTTCACCTGGGTGGCAGTGTCAGATTTGTTCTTCTTTAGAACAAGAC
CAGTATTTCAAGAAAGAGACTTTACAGACAAATAAGCAGGGATCCCATGGTAATAACAGA
AAACAGCTCCAAGACCAGGAAATTCGGGCTGAACTGAATAAGCATTTTGGTCACCCCAGC
CAAGCTGTTTTTGATGAAGAAGCAGATAAGACCAGAGAACTGAGGGACAGTGATTACAGT
AATGAACAATTCTCCAAACTACCTATGTTTATAAATTCAGGACTAGCAATGGATGGTCTG
TTTGATGACAGTGAAGATGAAAGTGATAAACTATGCTACCCTTGGGATGGGACACAAGCC
TATTCATTATTTGATGTATCACCTTCTTGCTCTTCTTTTAACTCTCCGTGCAGAGATTCA
GTGTCTCCACCCAAATCCTTATTTTCTCAAAGATCCCAAAGGACAGGCTCTAGATCAAGG
TCCTTACCTCAACGCAGGTCTTGTTCCCGTTCTCCATATTCCCGATCGAGATCAAGGTCG
CCCTGTAGTAGATCCTCTTCAAGATCTTGTTGCTATTGTGAGTCCAGCCACTGTAGACAC
CGAGCAAACAGAAGCTCTCCCCTAGGTGGAAGATCACGGTCCAGATCCCCGTGCAGTCGC
AGACCCAGATATGACAGCTATGAGGAATATCAGCATGAAAGGCTGAAGAGGGAAGAATAC
CGCAAAGAGTATGAAAAACGGGAATCTGAAAGGGCCAAGCAAAGGGAGAGACAGAGGCAG
AAAGCAATTGAAGAACGTCGTGTGATTTATCTGGGTAAAATCAGACCTGACACAACCCGA
ACAGAACTGAGGGACCGGTTTGAAGTTTTTGGTGAAATTGAGGAGTGCACAGTAAATTTG
CAGGATGATGGAGACAGCTATGGTTTCATCACCTACCGCTATACTTGTGACGCCTTCGCT
GCTCTTGAAAATGGATACACTTTACGCAGGTCAAGCGAGCCTGATTTTGAGCTGTACTTC
TGTGGACGCAAGCAGTTTTGCAAGTCTAACTATGCAGACCTAGATTCAAACTCAGATGAT
TTTGATCCTGCTTCCACTAAAAGCAAGTATGACTCCATGGATTTTGATAGTTTACTTAGA
GAGGCCCAGCGGAGCCTGCGTAGG
>Anole_lizard_PPARGC1A
ATGGATGAAACACGCACGAGGGAGAATAAGGACTTGGAGGAAGACTGGATAAAAGGGCTG
CGGCGGGAACCTAGCTGGCAGTGTGCTGCTCTGGTTGGTGAAGACCAGCCTCTTTGCCCA
GATCTTCCAGAACTTGACCTGTCAGAGTTAGACGTGAACGACTTGGATGCTGACAGTTTT
TTGGGTGGACTCAAATGGTACAGTGACCAATCAGAAATAATTTCCAATCAGTACGCCAAT
GAATCATCAAACATCTTCGAGAAGATAGATGAAGAGAATGAAGCAAACTTGCTAGCAGTT
CTCACAGAGACATTGGACAGTATCCCTGTGGATGAAGATGGATTGCCTTCATTTGATGCA
CTGACAGATGGAGATGTGACCAACGAAAATGATGCTAGCCCTTCACCAATGCCTGACGGC
ACCCCTCCAGCACAGGAGGCAGAAGAGCCGTCTCTACTTAAGAAGCTCTTGTTGGCACCA
GCTAACATCCAGCTAAATTACAATGAATGCAGTGGCCTCAGCACGCACAACCATGCAAAT
GCCAATCATAGGATCAGAACAAGCCCTGTGGTGGTTAAGACTGAGAATTCATGGAGCAAT
AAGACAAAGAGCATTTGTCAACCACAAAAACCACAAAGACGTCAATGCTCAGAGCTTCTC
AAGTATCTGACCACCAACGATGACCCTCCACAGACCAAACCAACAGAGAACAGGAACAGC
AGCAGAGACAAATGCACCTCTAAAAAGAAGGTGCTCTTGCACTCTCAGATGCATCATTTG
CAAGCCAAACCAATGAGTTTATCGCTTCCTTTGACGCCCGAGTCACCAAATGATCCCAAG
GGTTCCCCATTTGAGAACAAGACTATTGAACAAACTTTAAGTGTGGAACTCTCTGGAACT
GCAGGCCTAACTCCGCCTACAACCCCTCCTCACAAAGCGAACCAAGACAACCCTTTTAGG
ACTTCACCAAAGCCAAAGTCGTCATGCAAGACTGTTATGCCACCTTCCAAAAAACCCCGC
TGCAATGAGTCTTCCAGCTCTCAAGGACATAATTTAGGTCGGAAGGGTCCAGAGCAGTCA
GAGCTGTATGCACAGCTTAGTAAGACGACCGTGCTGTCTGTTAGTCATGAGGAGAGGAAG
ACAAAGCGGCCTGGTTTGCGGCTGTTTGGTGACCATGACTACTGTCAATCTGCTAATTCA
AAAACAGGAATACGCATCAACAGATCCCAGGAACCTCAGGATTCCAGACAAGTAGATTTA
AATGGTGGGTTGCCTGAGCAGTGGTGTCATATTTGTTCTTCTTTTGAACAATACAATCAA
TATGACAAGAGAGAGACTTCACAGGCAAGCAAGCAGGATTTGCAATTCAACAACCGCAAA

CTGCTCCAAGACCAAGAAATCCGAGCTGAACTCAACAAGCACTTTGGTCACCTTAGCCAA
GCTGTTTTTGAGGAAGAGACCACCAAGATCAGTGAACTGAGGGACAACAGTAATTTTAGT
GATGAACAATTCTCCAAACTACCTATGTTTATAAATTCTGGACTAACAATGGATGGTCTT
TTTGATGATAGCGAAGATGAAAATGACAAACTATGTTGCTCGTGGGATGGAACACAGGCC
TACTCACTGTTTGACCTGTCACCATCTTGCTCATCCTTTAATTCTCCATGCGGAGATTCA
GTGTCTCCACCTAAATCCCTTCTTTCTCAAAGATTCCAAAGGATACGCTCTCGATCACGG
TCCTTCCCTCAGCACAGGTCTTGTTCCCATTCTCCATATTCCCGATCAAGATCAAGGTCA
CCATGCAGTAGATTCTCCTCAAGATCTTGTTACTATTATGAGTCCAGTCATTGTAGACCG
CAGGCATATAGAAGTTCTCCCTTGTATTCAAGATCACGATCCAGATCACCATATAGCCAT
AGACCCAGATATGACAGCTATGAGGAATATCAGCATGAAAGGCTGAAGAGGGAAGAATAC
CGCAAAGAATATGAAAAACGGGAATCTGAAAGGGCCAAACAAAGAGAGCGACAGAAGCAG
AAAGCAATTGAAGAACAGCGTGTAATTTATGTTGGCAAAATTGGGCCTGACACAACCCGA
GCAGAACTAAGGGACCGGTTTGAAGTTTTTGGTGAAATTGAGGAGTGCTCTGTGAATCTA
CAGGACAATGGAGATAACTATGGTTTCATCACCTACCGCTACACTTGTGATGCTTTTGCT
GCTCTGGAAAATGGATACACTCTGCGCAGAACCAATGAGCCTGACTTTGAGCTGTATTTT
TGTGGACAAAAGCAATTTTGCAAGTCTAACTATGCAGACCTAGATTCAAATTCAGACGAT
TTTGATCCTGCTTCTACTAAGAGCAAGTATGACTCCATGGATTTCGACAGTTTACTTAAA
GAGGCACAGCGAAGCTTGCGGAGG
>Great_tit_PPARGC1A
ATGGCTGGCTGCGGGCTGTCGGAAGACACTTGTTTTTCCTCCTTCTTTTACAACTACACC
TCCTCCTGGGAGACCTCCTACAACCAGTGTGCTGCTCTGGTTGGCGAAGACCAGCCTCTT
TGCCCAGATCTCCCAGAACTTGACCTCTCTGAACTAGATGTGAACGACCTGGATGCAGAC
AGCTTTCTGGGGGGACTCAAGTGGTACAGCGACCAGTCAGAGATCATCTCCAATCAGTAC
AGCAATGAGCCCGCCAATATATTTGAGAAGATAGATGAAGAGAACGAGGCAAACTTGCTA
GCTGTTCTCACTGAAACACTGGACAGCATCCCTGTGGATGAGGATGGATTGCCTTCATTT
GATGCACTGACAGATGGAGATGTGACCAACGAAAATGCCGCTAGTCCCTCCCCAATGCCC
GACGGCACCCCTCCAACTCAGGAGGCAGAAGAGCCGTCTCTACTTAAGAAGCTCTTGCTG
GCTCCAGCCAACACTCAGCTAAATTACAATGAATGCAGTGGTCTCAGCACACAAAACCAT
GCAAACACTAATCACAGGATCAGAACAAGCCCTGTGGTTGTTAAGACTGAGAATTCATGG
AGCAATAAAGCGAAGAGCATTTGTCAACAGCAAAAGCCTCAAAGACGTCCCTGCTCTGAA
CTTCTCAAATATCTGACTACGAATGATGATCCTCCTCAGACCAAACCAGCAGAGAACAGG
AACAGCAGCAAAGAGAAATGCACCTCCAAAAGGAAGCCCCATTTGCAGTCTCAGACAAAT
CATCTGCAGGCCAAACCAACAAGTTTATCACTTCCATTGACACCCGAGTCACCAAATGAT
CCCAAGGGTTCCCCATTTGAGAACAAGACTATTGAACAAACCTTAAGTGTGGAACTCTCT
GGAACTGCAGGCCTAACTCCACCTACGACCCCTCCTCATAAAGCCAACCAAGATAACCCT
TTTAGGACTTCACCTAAATTGAAGTCATCATGCAAGACTGTTGTACCACCTTCAAAAAAG
CCCCGCTATTGTGAGTCTTCCAGTTCTCAAGGAAATAACCCAATCAAGAAGGGTTCAGTA
CCGTCTGAGCTGTACACACAGCTTAACAAGACTACAGTACTGTCCAGTGGACATGAGGAG
AGAAAGACAAAACGGCCCAGTTTGCGGCTGTTTGGTGACCATGACTACTGTCAATCTGTG
AATTCGAAATCGGAAATACACATTAAAATATCCCAGGAACTTCAGGACTCCAGACAACCA
GAATTTAAGGATTCTTCACCTGGGTGGCAGTGTCAGATTTGTTCTTCTTTAGAACAAGAC
CAGTATTTCAAGAAAGAGACTTTACAGACAAATAACCAGGGATCCCATGGTAATAACAGA
AAACAGCTCCAAGACCAGGAAATTCGGGCTGAACTGAATAAGCATTTTGGTCACCCCAGC
CAAGCTGTTTTTGATGAAGAAGCAGATAAGACCAGAGAACTAAGGGACAGTGATTACAGT

AATGAACAATTCTCCAAACTACCTATGTTTATAAATTCAGGACTAGCAATGGATGGTCTG
TTTGATGACAGTGAAGATGAAAGTGATAAACTATGCTACCCTTGGGATGGGACACAAGCC
TATTCATTATTTGATGTATCACCTTCTTGCTCTTCTTTTAACTCTCCATGCAGAGATTCA
GTGTCTCCACCCAAATCCTTATTTTCTCAAAGATCCCAAAGGACACGCTCTAGATCAAGG
TCCTTTCCTCAACGCAGGTCTTGTTCCCGTTCTCCATATTCCCGATCGAGATCAAGGTCG
CCCTGTAGTAGATCCTCTTCAAGATCTTGTTGCTATTGTGAGTCCAGCCACTGTAGACAC
CGAGCATACAGAAGTTCTCCCTTAGGTGGAAGATCGCGGTCCAGATCACCGTACAGTCGC
AGACCCAGATATGACAGCTATGAGGAATATCAGCATGAAAGGCTGAAGAGGGAAGAATAC
CGCAAAGAGTATGAAAAACGGGAATCTGAAAGGGCCAAGCAAAGGGAGAGACAGAGGCAG
AAAGCAATTGAAGAACGTCGTGTGATTTATCTGGGTAAAATCAGACCTGACACAACCCGA
ACAGAACTGAGGGACCGGTTTGAAGTTTTTGGTGAAATTGAGGAGTGCACTGTAAATTTG
CGGGATGATGGAGACAGCTATGGTTTCATCACCTACCGCTATACTTGTGACGCCTTCGCT
GCTCTTGAAAATGGATACACTTTACGCAGGTCAAATGAGCCTGATTTTGAGCTGTACTTC
TGTGGACGCAAGCAGTTTTGCAAGTCTAACTATGCAGACCTAGATTCAAACTCAGATGAT
TTTGATCCTGCTTCCACTAAAAGCAAGTATGACTCCATGGATTTTGATAGTTTACTTAAA
GAGGCACAGCGGAGCCTGCGTAGG
>African_ostrich_PPARGC1A
ATGGCGTGGGACATGTGCAACCAGGACTCTGTCTGGAGTGACATAGAGTGTGCTGCTCTG
GTTGGTGAAGACCAGCCTCTTTGCCCAGATCTCCCAGAACTTGACCTCTCTGAACTAGAC
GTGAACGACTTGGATGCAGACAGCTTTCTAGGGGGACTGAAGTGGTACAGCGACCAATCA
GAAATTATTTCCAATCAGTACAGCAATGAATCCTCCAATATATTTGAGAAGATAGATGAA
GAGAATGAGGCAAACTTGCTAGCAGTTCTCACAGAAACACTGGACAGTATCCCTGTGGAT
GAGGATGGATTGCCTTCATTTGATGCGCTGACAGATGGAGATGTGACCAATGAAAATGAT
GCTAGCCCTTCCCCAATGCCCGACGGCACCCCTCCAACTCAGGAGGCAGAAGAGCCGTCT
CTACTTAAGAAGCTCTTGCTGGCTCCAGCCAACACTCAGCTAAATTACAATGAATGCAGT
GGTCTCAGCACACAAAACCATGCAAACACTAATCACAGGATCAGAACAAGCCCTGTGGTT
GTTAAGACTGAGAATTCATGGAGCAATAAAGCGAAGAGCATTTGTCAACAGCAAAAGCCA
CAAAGACGTCCCTGCTCTGAACTTCTCAAATATCTGACTACAAATGATGACCCTCCTCAG
ACCAAACCAGCAGAGAACAGGAACAGCAGCAAAGAGAAATGCACCTCCAAAAGGAAGCCC
CATCTGCAGTCTCAGACAAATCATCTGCAAGCCAAACCAACAAGTTTATCACTCCCGTTG
ACACCCGAGTCACCAAATGATCCCAAGGGTTCCCCATTTGAGAACAAGACTATTGAACAA
ACCTTAAGTGTGGAACTCTCTGGAACTGCAGGCCTAACTCCACCGACGACCCCTCCTCAT
AAAGCCAACCAGGATAATCCTTTTAGGACTTCACCTAAGCCGAAGTCATCATGCAAGACT
GTTGTACCACCTTCAAAAAAGCCCCGCTATAGTGAGTCTTCCAGTTCTCAAGGAAATAAC
CCAACCAAGAAGGGTCCAGAACAATCTGAGCTGTATGCGCAGCTTAGCAAGACTACAGTA
CTGTCCAGTGGACATGAGGAGAGAAAGACAAAACGGCCCAGTTTGCGGCTGTTTGGTGAC
CATGACTACTGTCAATCTGTGAATTCAAAATCGGAAATACACATAAAAATATCCCAGGAA
CTTCAGGACTCCAGACAACTAGAATTTAAGGATTCTTCACCTGGGTGGCAATGTCAGATT
TGTTCTTCTTTAGAACAAGACCAGTATTTCAAGAAAGAGACTTTACAGACAAGTAAGCAG
GGTTCCCACTGTAATAACAGAAAACAGCTCCAAGACCAGGAAATTCGGGCTGAACTGAAT
AAGCATTTTGGTCACCCCAGCCAAGCTGTTTTTGATGAAGAGGCAGATAAGACCAGTGAG
CTAAGGGACAATGATTATAGTAATGAACAATTCTCCAAACTACCTATGTTTATAAATTCA
GGACTAGCAATGGATGGTCTCTTTGATGATAGCGAAGACGAAAGTGATAAACTATGCTAC
CCTTGGGATGGGACACAAGCCTATTCATTATTTGATGTATCGCCTTCTTGCTCTTCTTTT

AACTCTCCATGCAGAGATTCAGTGTCTCCACCCAAATCCTTATTTTCTCAAAGATCCCAA
AGGATACGCTCTAGATCAAGGTCCTTTCCTCAACGCAGGTCTTGTTCCCGTTCTCCATAT
TCCCGATCGAGATCAAGGTCGCCCTGTAGTAGATCCTCTTCAAGATCTTGTTACTATTAT
GAGTCCAACCACTGTAGACAGCGAGCATACAGAAGTTCTCCCTTATATGCAAGATCGCGA
TCCAGATCACCGTACAGTCGAAGACCCAGATATGACAGCTATGAGGAATATCAGCATGAA
AGGCTGAAGAGGGAAGAATACCGCAAAGAGTATGAAAAACGGGAATCTGAAAGGGCCAAA
CAAAGGGAGAGACAGAGGCAGAAAGCAATTGAAGAACGTCGTGTGATTTATGTGGGTAAA
ATCAGACCTGACACAACCCGAACAGAACTGAGGGACCGGTTTGAAGTTTTTGGTGAAATT
GAGGAGTGCACAGTAAATTTGCGGGATGATGGAGACAGCTATGGTTTCATCACCTACCGC
TACACATGTGATGCCTTTGCTGCTCTTGAAAATGGATACACTTTGCGCAGGTCAAATGAA
CCTGACTTTGAACTGTACTTTTGTGGACGCAAGCAATTTTACAAGTCTAACTATGCAGAC
CTAGATTCAAACTCAGATGATTTTGATCCTGCTTCCACTAAAAGCAAGTATGACTCCATG
GATTTTGATAGTTTACTTAAAGAGGCACAGCGGAGCCTGCGTAGG
>Adelie_penguin_PPARGC1A
ATGGCGTGGGACATGTGCAACCACGACTCTGTATGGAGTGATCTCGAGTGTGCTGCTCTG
GTTGGCGAAGACCAGCCTCTTTGCCCAGATCTCCCAGAACTTGACCTCTCCGAACTAGAT
GTGAACGACCTAGACGCAGACAGCTTTCTGGGGGGACTGAAGTGGTACAGCGACCAGTCT
GAGATCATCTCCAATCAGTACAGCAATGAACCCGCCAATATATTCGAGAAGATAGATGAA
GAGAGCGAGGCAAACTTGCTAGCTGTTCTCACTGAAACACTGGACAGCATCCCTGTGGAT
GAGGATGGGTTGCCTTCATTTGATGCACTGACAGATGGAGATGTGACCAACGAAAATGCC
GCTAGCCCTTCCCCAATGCCCGACGGCACCCCTCCAACTCAGGAGGCAGAAGAGCCGTCT
CTACTTAAGAAGCTCTTGCTGGCTCCAGCCAACACTCAGCTAAATTACAATGAATGCAGT
GGTCTCAGCACACAAAACCATGCAAACACTAATCACAGGATCAGAACAAGCCCTGTGGTT
GTTAAGACTGAGAATTCATGGAGCAATAAAGCAAAGAGCATTTGTCAACAGCAAAAGCCT
CAAAGACGTCCCTGCTCTGAACTTCTCAAATATCTGACTACGAATGATGACCCTCCGCAG
ACCAAACCAGTAGAGAACAGGAACAGCAGCAAAGAGAAATGCACCTCCAAAAGGAAGCCC
CATCTGCAGTCTCAGACAAATCATCTGCAAGCCAAACCAACAAGTTTATCACTTCCATTG
ACGCCCGAGTCACCAAATGATCCCAAGGGTTCCCCATTTGAGAACAAGAATATTGAACAA
ACCTTAAGTGTGGAACTCTCTGGAACTGCAGGCCTCACTCCACCTACGACCCCTCCTCAT
AAAGCCAACCAGGATAATCCTTTTAGGACTTCACCTAAGCCGAAGTCATCATGCAAGACT
GTTGTACCACCTTCAAAAAAGCCCCGCTATAGTGAGTCTTCCGGTTCTCAAGGAAGTAAC
CCAATCAAGAAGGGTCCAGAACAGTCTGAGCTGTACGCACAGCTTAGCAAGACTACAGTA
CTGTCCAGTGGACATGAGGAGAGAAAGACAAAACGGCCCAGTTTGCGGCTGTTTGGTGAT
CATGACTACTGTCAATCTGTGAATTCAAAATCGGAAATACACATTAAAATATCCCAGGAA
CTTCAGGACTCCAGACAACTAGAATTTAAGGATTCTTCACCTGGGTGGCAGTGTCAGATT
TGTTCTTCTTTAGAACAAGACCAGTATTTCAAGAAAGAGACTTTACAGACAAGTAAGCAG
GGATCCCACGGTAATAACAGAAAACAGCTCCAAGACCAGGAAATTCGGGCTGAACTGAAT
AAGCATTTTGGTCACCCCAGCCAAGCTGTTTTTGATGAAGAAGCAGATAAGACCAGAGAA
CTAAGGGACAGTGATTACAGTAATGAACAATTCTCCAAACTACCTATGTTTATAAATTCA
GGACTAGCAATGGATGGTCTCTTTGATGACAGTGAAGATGAAAGTGATAAACTATGCTAC
CCTTGGGATGGGACACAAGCCTATTCATTATTTGATGTATCGCCTTCTTGCTCTTCTTTT
AACTCTCCATGCAGAGATTCAGTGTCTCCACCCAAATCCTTATCTTCTCAAAGATCCCAA
AGGACACGCTCTAGATCAAGGTCCTTTCCTCAACGCAGGTCTTGTTCCCGTTCTCCATAT
TCCCGATCGAGATCAAGGTCGCCCTGTAGTAGATCCTCTTCAAGATCTTGTTGCTATTAC

GAGTCCAGCCACTGTAGACACCGAGCATACAGAAGTTCTCCCTTAGGTGCAAGATCGCGA
TCCAGATCACCGTACAGTCGCAGACCCAGATATGACAGCTATGAAGAATATCAGCATGAA
AGGCTGAAGAGGGAAGAATACCGCAAAGAGTATGAAAAACGGGAATCTGAAAGGGCCAAA
CAAAGGGAGAGACAAAGGCAGAAAGCAATTGAAGAACGTCGTGTGATTTATCTGGGTAAA
ATCAGACCTGACACAACCCGAACAGAACTGAGGGACCGGTTTGAAGTTTTTGGTGAAATT
GAGGAGTGCACAGTAAATTTGCGGGATGATGGAGACAGCTATGGTTTCATCACCTACCGC
TATACTTGTGACGCCTTTGCTGCTCTTGAAAATGGATGTACTTTACGCAGGTCAAATGAA
CCTGACTTTGAGCTGTACTTCTGTGGATGCAAGCAATTTTGCAAGTCTAACTATGCAGAC
CTAGATTCAAACTCAGATGATTTTGATCCTGCTTCCACTAAAAGCAAGTATGACTCCATG
GATTTTGATAGTTTACTTAAAGAGGCACAGCGGAGCCTGCGTAGG
>common_starling_PPARGC1A
ATGGCGTGGGACATGTGCAACCAGGACTCTGTATGGAGTGATCTCGAGTGTGCTGCTCTG
GTTGGTGAAGACCAGCCTCTTTGCCCGGATCTCCCAGAACTTGACCTCTCTGAACTAGAT
GTAAACGACCTGGATGCAGACAGCTTTCTGGGGGGACTCAAGTGGTACAGCGACCAGTCT
GAGATCATCTCCAACCAGTACAGCAATGAACCCGCCAATATATTCGAGAAGATAGATGAA
GAGAGCGAGGCAAACTTGCTAGCTGTTCTCACAGAAACACTGGACAGCATCCCTGTGGAT
GAGGATGGATTGCCTTCCTTTGATGCACTGACAGATGGAGATGTGACCAACGAAAATGCC
GCTAGCCCCTCCCCAATGCCCGACGGCACCCCTCCCACTCAGGAGGCAGAAGAGCCGTCT
CTACTTAAGAAGCTCTTGCTGGCTCCAGCCAACACTCAGCTAAATTACAATGAATGCAGT
GGTGTCAGCACACAAAACCATGCAAACACTAATCACAGGATCAGAACAAGCCCTGTGGTT
GTTAAGACTGAGAATTCATGGAGCAATAAAGCGAAGAGCATTTGTCAACAGCAAAAGCCT
CAAAGACGTCCCTGCTCTGAACTTCTCAAATATCTGACTACGAATGATGATCCTCCTCAG
ACCAAACCAGCAGAGAACAGGAACAGCAGCAAAGAGAAATGCACCTCCAAAAGGAAGCCC
CATCTGCATTCTCAGACAAATCATCTGCAGGCCAAACCAACAAGTTTATCACTTCCATTG
ACACCCGAGTCACCAAATGATCCCAAGGGTTCCCCATTTGAGAACAAGACTATTGAACAA
ACCTTAAGTGTGGAACTCTCTGGAACTGCAGGCCTAACTCCACCTACGACCCCTCCTCAT
AAAGCCAACCAAGATAATCCTTTTAGGACTTCACCTAAACTGAAGTCATCATGCAAGACT
GTTGTACCACCTTCAAAAAAGCCCCGCTATAGTGAGTCTTCCGGTTCTCAAGGAAATAAC
CCAATCAAGAAGGGTCCAGATCAGTCTGAGCTGTACACACAGCTTGGCAACACTACAGTA
CTGTCCAGTGGACATGAGGAGAGAAAGACAAAACGGCCCAGTTTGCGGCTGTTTGGTGAC
CATGACTACTGTCAATCTGTGAATTCAAAATCGGAAATACACATTAAAATATCCCAGGAA
CTTCAGGACTCCAGACAACCAGAATTTAAGGATTCTTCACCTGGGTGGCAGTGTCAGATT
TGTTCTTCTTTAGAACAAGACCAGTATTTCAAGAAAGAGACTTTACAGACAAATAAGCAG
GGATCCCATGGTAATAACAGAAAACAGCTCCAAGACCAGGAAATTCGGGCTGAACTGAAT
AAGCATTTTGGTCACCCCAGCCAAGCTGTTTTTGATGAAGAAGCAGATAAGACCAGAGAA
TTAAGGGACAGTGATTTCAGTAATGAACAATTCTCCAAACTACCTATGTTTATAAATTCA
GGACTAGCAATGGATGGTCTGTTTGATGACAGTGAAGATGAAAGTGATAAACTATGCTAC
CCTTGGGATGGGACACAAGCCTATTCATTATTTGATGTATCACCTTCTTGCTCTTCTTTT
AACTCTCCGTGCAGAGATTCAGTGTCTCCACCCAAATCCTTATTTTCTCAAAGATCCCAA
AGGACACGCTCTAGATCAAGGTCCTTTCCTCAACGCAGGTCTTGTTCCCGTTCTCCATAT
TCCCGATCGAGATCAAGGTCGCCCTGTAGTAGATCCTCTTCAAGATCTTGTTGCTATTGT
GAGTCCAGCCACTGTAGACACCGAGCAAACAGAAGTTCTCCCCTAGGTGGAAGATCGCGG
TCCAGATCACCGTACAGTCGCAGACCCAGATATGACAGCTATGAGGAATATCAGCATGAA
AGGCTGAAGAGGGAAGAATACCGCAAAGAGTATGAAAAACGGGAATCTGAAAGGGCCAAG

CAAAGGGAGAGACAGAGGCAGAAAGCAATTGAAGAACGCCGTGTGATTTATCTGGGTAAA
ATCAGACCTGACACAACCCGAACAGAACTGAGGGACCGGTTTGAAGTTTTTGGTGAAATT
GAGGAGTGCACAGTAAATTTGCGGGATGATGGAGACAGCTATGGTTTCATCACCTACCGC
TATACTTGTGACGCCTTCGCTGCTCTTGAAAATGGATACACTTTACGCAGGTCAAATGAA
CCTGATTTTGAGCTGTACTTCTGTGGACGCAAGCAGTTTTGCAAGTCTAACTATGCAGAC
CTAGATTCAAACTCAGATGATTTTGATCCTGCTTCCACTAAAAGCAAGTATGACTCCATG
GATTTTGATAGTTTACTTAAAGAGGCACAGCGGAGCCTGCGTAGG
>central_bearded_dragon_PPARGC1A
ATGGCTGGTTGCGGGCTCTCCGAAGATGCCGCCTTCTCCATCGCCACCGCCGCCACCTCC
TCGTCCTGCTCCTCCTTCTGCAACCTCGCCGCCTCCTGGGAAGCCCCTTTCAGCCATCAG
CCTTTTTCCAGCGCTGATCTTCCTGCAAAGGAATGCAACATTCTTTTCAGCAACACACAG
TGCGCTGCTCTGGTTGGTGAAGACCAGCCTCTTTGCCCAGATCTTCCAGAACTTGACCTT
TCTGAATTAGATGTGAATGATTTGGATGCAGACAGCTTCTTGGGTGGACTCAAGTGGTAT
AGCGACCAATCAGAAATAATTTCCAATCAGTATGCCAATGAATCGACAAACATATTCGAG
AAGATAGATGAAGAGAATGAAGCAAACTTGCTAGCAGTTCTCACAGAGACGTTGGACAGC
ATCCCTGTGGATGAAGATGGATTGCCTTCATTTGATGCATTGACAGATGGCGATGTGACC
AACGAAAATGATGCTAGCTCTTTGCCAATGCCTAATGGCACCCCTCCACCTCAGGAGGCA
GAAGAGCCATCTCTACTTAAGAAGCTCTTGTTGGCTCCAGCTAACACCCAGCTAAATTAC
AATGAATGCAGTGGCCTCAGCACACACAATCATGCAAATGCCAGTCACAGGATCAGAACA
AGCCCTGTGGTGGTTAAGACTGAGAATTCATGGAGCAATAAGGCAAAAAGCATTTGTCAA
CCACAAAAGCCACAAAGACGTCCCTGTTCAGAACTTCTCAAATATCTGACTACAAGCAAT
GACTCTCCTCAGACCAAACCAACAGAGAACAGAAACAGTAGCAGAGACAAATGCACTACT
AAAAAGAAAACACTTTTGCACTCTCAGACACATCATTTGCAAGCCAAACCAATGAGTTTA
TCGCTTCCTTTGACGCCTGAGTCACCAAATGATCCCAAGGGTTCCCCATTTGAGAACAAG
ACTATTGAACAAACTTTAAGTGTGGAACTCTCTGGAACTGCAGGCCTAACTCCGCCTACA
ACCCCTCCTCACAAAGCCAATCAAGACAACCCTTTTAGGACATCACCAAAGCCAAAGTCA
TCATGCAAGACTGTTATGCCACCTTCCCAAAACCCCTGCTGCAGTGAGTCTTCCAGCTCT
CAAGGACATATTTTCGTTCGGAAGGGTCCAGAGCAGTCAGAACTGTATGCACAGCTTTGC
GAGACAACTGTGCTGTCTGTCAGTCACGAGGAGAGGAAGACAAAGCGGCCTGGTTTACAG
CTGTTTGGTGATCATGACTACTGTCAGTCTGCTAATTCAAAAACAGAAATACACATTAAT
CTGTCCCAGGAACTTCAGGATTCCAGACAAGTAGAGCTGAAAGGCTGCTTGCCTGGGCGA
TGGTGTCAAATTTGTTCTTCTTTTGAAAAACATGACCAGTATGCCAAGAGAGAGACTTCA
CAGGCAAGCAAGCAGAATTTGCAGTGCAACAACCGGAAACCGCTCCAAGACCAAGAAATC
CGGGCTGAACTGAATAAGCACTTTGGTCACCTTAGTCAAGCTTTTTTTGAGGAAGAGACC
ACTAAGACCAGTGAACTGAGGAACAGCAGTAATTATAGTGGTGAACAATTCTCCAAGCTA
CCTATTTTTATAAATTCTGGACTAGCAATGGATGGTCTTTTTTATGACAGTGAAGATGAA
AATGACAAATTGTGCTGCTCATGGCATGGGACACAAGCCTACTCATTGTTTGACCTGTCG
CCTTCTTGCTCATCCCTTAATTCTCCATGCAGAGATTCAGTGTCTCCACCCAAATCCTTG
CTTTCAAGATTCCAAAGGATACGTTCTCGATCAAAGTCCTTTCCTCAGCGCAGGTCTTGT
CCCCGTTCTCCATATTCCCAATCACGGTCAAGGTCACCGTGGAGTAGATCCTCTTCCAGA
TCATCTTACTGTTATGAGTCCAGTCATTGTAGATCCTGGGAATATAGAAGTTCTCCCTTA
TATTCAAGATCACGATCCAGATCACCACATAGTCATAGAGCCAGATATGACAGCTACGAG
GAATATCAGCATGAAAGACTGAAGAGGGAAGAATACCGCAAAGAATATGAAAAACGGGAA
TCTGAAAGGGCCAAACAAAGAGAGAGACAGAGGCAGAAGGCAATTGAAGAACAACGTGTA

ATTTATATTGGTAAAATTGGCCCTGACACAAGCCGAACAGAACTGAGGGACCGGTTTGAA
ATATTTGGTGAAATTGAGGAGTGCACAGTAAATCTACAAGATAATGGAGATAGCTATGGT
TTCATCACTTACCGCTACACTTGTGATGCTTTTGCTGCTCTAGAAAATGGATACACTCTA
CGCAGGTCCAATGAGCCTGACTTTGAGCTGTATTTTTGTGGACAAAAGCAGTTTTACAAG
TCCAACTATGCAGACTTAGATTCAAATTCAGACGATTTTGATCCTACTTCTACTAAGAGC
AAGTATGACTCCATGGATTTTGACAGTTTACTTAAAGAGGCGCAACGCAGCTTGCGGAGG
>Gecko_PPARGC1A
ATGGCGTGGGACATGTGCAGCCCGGACGCCGCCTGGACTGACATCGAGTGTGCTGCTCTG
GTTGGTGAAGACCAGCCCCTTTGCCCGGATCTTCCCGAACTCGACCTCTCTGAACTAGAC
GTGAATGACTTGGATGCGGACAGCTTCCTGGGCGGACTCAAGTGGTACAGCGACCAACCA
GAGATCATTTCCAATCAGTACAACAATGAGTCATCAAATATCTTTGAGAAGATCGATGAA
GAAAACGAAGCCAACTTGCTAGCAGTCCTCACAGAGACGCTGGACAATATCCCGGTGGAC
GAGGATGGATTACCTTCATTCGACGCCCTGGCAGATGGTGATGTGACCAATGAAAACGAT
GCTAGTTCTTCACCAATGCCCGACAGCGCTCCTCCATCTCAGGAGGCAGAAGAGCCGTCT
CTACTTAAGAAGCTCTTGTTGGCTCCAGCTAACACCCAGCTAAATTACGATGAATGCAGT
GGCCTCAGCACACACAATCATGCCAGCACCAATCACAGGATCAGAGCAAGCCCTATGGCC
ATTAAGACTGAGAATTCATGGAGCAATAAAGCGAAGAGCGTTTGTCAACCGCCAAAGCCA
CAGAGACGCCCTTGCTCGGAGCTCCTCAAGTATCTGACTACAAACGATGACCCTCCACAG
ACCAAACCAACAGAGAACAAGAACAGCAACAGAGACAAGTGCACCTCGAAAAAGAAGGCC
CTTTTGCAGTCTCAGACACATCACTTGCAAGCCAAACAAATGAGTTTATCGCTTCCTTTG
ACGCCCGAGTCACCAAATGATCCCAAGGGTTCCCCATTTGAGAACAAGACTATTGAACAA
ACTTTAAGTGTGGAACTCTCTGGAACTGCAGGCCTAACTCCACCTACAACCCCTCCTCAC
AAAGCCAACCAAGACAACCCTTTTAGGACTTCGCCAAAGCCGAAGTCATCATGCAAGACT
GTCGTGCCATCTTCCAAAAAACCCCGCTGTAGTGAGTCTTCCAGCTCTCAAGGACCTAAC
TCGCTTCGGAAGGGTCCAGCACAGTCAGAGCTGTATGCGCAGCTTAGCAAGACAACCGTT
CTGTCTGCCGGCCACGAGGAGAGGAAGACAAAGCGGCCTGGTTTGCGGCTGTTTGGCGAC
CATGACTACTGTCAGTCTGTTAACTCAAAAGCAGACGTGCGTATCAACCTACCCCAGGAA
CTCCAGTATTCTGGACAAGTAGAATCACAAGGCTGCTTGCCTGGGTGGCAGTGTCATATT
TGTTCTCCCTTTGACCCACTCAGCCAGTATGGCAAGAGAGAGACTTCACAGGTGAGCAAG
CAGGATCCGCAGCACAACAACCGGAAACAGCTCCAAGACCAGGAAATCCGGGCCGAACTC
AATAAGCACTTTGGTCACCCGAGCCAAGCGGTTTTTGAGGAAGAGACCACTAAGAGCAGT
GGGCTGAGGGACAGCCATTATAGCGACGAACAATTCTCCAGACTACCTATGTTTATAAAT
TCAGGGCTGGTAATGGATGGCCTTTTGGATGACAGTGAAGACGAATGTGACAAACTGTGC
TGCTCGTGGGATGGAACGCAAACCTACTCCTTGTTTGATCTGTCGCCTTCTTGCTCTTCC
CTTAACTCTCCGTGCAGAGATTTGGTATCTCCACCCAAATCCTTCCTTTCTCAAAGATTC
CAAAGGATACGATCTCGATCAAGGTCCTTTCCTCAGCACAGGTCTTGTTCCCGTTCTCCA
TATTCCCGATCGAGATCAAGGTCACCGTGCAGTAGATCCTCTTCAAGATCTTCCTATTAT
CAAGAGTCTGGTCACTGTAGACCCTGGGCATATAGAAGTTCTCCCTTGTATTCAAGATCA
CGTTCCAGATCACCATATAGCCGTAGACCCAGGTATGACAGCTACGAGGAATATCAGCAT
GAAAGGCTGAAGAGGGAAGAATACCGCAAAGAGTATGAAAAACGGGAATCCGAAAGGGCC
AAACAAAGGGAGAGGCAGAGGCAGAAGGCAATTGAAGAGCGGCGCGTAATTTATGTCGGA
AGAATTGGACCTGACATCACCCGAGCAGAACTGAGCGACCGGTTTGAAGTTTTCGGCGAA
ATTGAGGAGTGCACAGTAAATCTACAGGATGATGGGGATAGCTACGGTTTCATCACTTAC
CGCTACACCTGCGATGCTTTTGCTGCTCTGGAAAATGGATACACCTTGCGCAGGTCCAGT

GAGCCTGACTTAGAGTTGTATTTTTGCGGACACAAGCAGTTTTGCAAGTCTAACTATGCA
GACCTAGATTCAAACTCGGATGATTTTGATCCTGCTTCTACCAAGAGCAAGTATGATTCG
ATGGATTTTGACAGCTTGCTTAAAGAGGCTCAGCGAAGCTTGCGAAGG
>Taiwan_habu_PPARGC1A
TGTGCTGCTTTAGTTGGGGAAGACCAGCCTCTCTGCCCAGACCTTCCGGAACTTGATCTC
TCCGAATTAGATGTCAACGATCTGGATGACAGCGGTTTCCTGGGAGGGCTTAAGTGGTAC
AACGACCAATCGGAAATCATCTCCCATCCGTATCCTAACGAGACATCCAACATATTTGAG
AAGATAGATGAAGAGAACGAAGCAAACTTGCTAGCGGTCCTCACCGAAACGTTGGACAGT
ATCCCAGTGGACGAAGATGGATTGCCTTCCTTCGACGCCCTGACAGATGGAGATGTGACC
AGCCAAAATGAGGCCAGCCCTTCATCAGTGGCTGACAGCACCCCCCCACCTCAGGAGGCC
GAAGAGACGTCTCTACTTAAGAAGCTCTTGCTGGCTCCAGCCAACATCCAGCTAAACTAC
AATGAATGCAGTGGCCTGGGCACGCACAATCATGCAAACTCCAATCACAGGATCAAAACA
AGCCCTGTGGTGGTCAAGACCGAGAATCCATGGAGCAATAAAGTGAAGAGCATCTGCCAG
GCGCAAAAGCCGCAGAGACGCCCCTGCTCAGAACTTCTCAAGTACCTGACCACCGGTGAT
GACCCCGGTCAGGCCAAACAGACGGAAAACAGGAACAGCAACAGGGACAAATACACTTCT
AAAAAGAAGGCACTGTTGCACTCTCAGACGCACCACTTGCAAGAGAACGAATGTTATGGG
GCACCTGCTAAGCAGACCAAATCAATGAATTTATCGCTTCCGTTGACGCCTGAATCGCCA
AACGATCCGAAGGGTTCCCCATTTGAGAGCAAGGCCATCGGACAAAGCTTAAGTGTGGAA
CTCTGTGGAACTGCAGGCCTAACTCCACCTACCACCCCTCCTCATAAGAGCAGCCAAGAC
AATCCTTTTGGGACTTTGCCAAAGCCCACGGTGTCATGCCCGACTGTTATGCCACCTTCC
CCGACACCTCGCTGCAGTGACTCTTCCATCTCTCAAGGACGCAATTTGTTCTGGAAGGGT
CCGGAGCAGTCGGAGCTATATGCCCAGCTGAGCAAGACCACCGTGCTGCCCGTGGGCCGT
GAGGAGAGGAAGGCGAGGCGGGCTGGCTTGACACTGTTCGGCGACCACGATTACTGTCAG
TCGGCCATTTCTAAGACAGAACGATGCGTCAACCGCTCCCTGGAACTTCAGAATCCCCGT
CAGGAATTTGAATTCTCAGGCTGCCTATCCGGCTATGAGTGGTGCCACATTTGTTCCTCC
TTTGAGCAGAACCGCCAGGAGGATGAGCGAGAAGCTTCGCAGGCGAGGAAGCAGGACTTA
GCGTGCAACAGCCGGAAACAGCTGCAAGACCAGGAAATCCGAGCTGAACTCAACAAGCAT
TTTGGTTACCCTAGCCAAGCGGTTCTCGAGGAAGAGATGAATAACACCGGCCATCTGAGG
GAGTACAGTCACTACAGCGATGAACAGTTCTCTAAGCTATCCATGTTTATCAATTCGGGA
CTAGCGATGGAGGGCTTTTGGGACGACGGCGAAGCGGAAAACGAAAAGCTGTGCTGCTCG
TGGTACGGGACGCAAGGCTGCACCTTATTTGATACGTCACCTTCTTCCTCATCCCTGAAT
TCTCCATGCAGAGACTCGGTGTCTCCACCCAACTCATTTGCTCAACGGTTCCAGAGGCTA
TGCGCTCAGTCGAGGTCCTTCCCACAGTGCAGGCATTGCGCCCACTCTCCGTATTCCCGT
TCAAGATCACGGTCGCCCGGCAAACGCTGCTCGTCGAGATATGACAGCTACGAGGAATAT
CAGCACGAGAGGCTGAAGAGGGAAGAATATCGCAAAGAATACGAGAAACGGGAATCCGAA
AGGGCGAAGCAGAGAGAAAGAGAGAGGCAGAAAGCAGTGGAAGAACGTCGCGTCATTTAT
ATTGGTAGAATTGGACCTGGCTTTACCCAAAGGCAACTGAGGGACCGGTTTGAAGTTTTT
GGTGAAATTGAGGAGTGTACAGTGAATCTACAGGATGATGGAGATAGCTATGGATTCATC
ACTTACCGCTATACTTGTGATGCCTTTGCTGCACTAGAAAATGGAAATACTCTACGCAGA
TCCAATGAACCTGCCTTTCAGCTGTATTTTTGTGGACAGAGGCAACGCTGCAAGGCTAAC
TATGCAGATCTTGGATCACATTCGGACGACTTTGATCCTGCTTCTACTAAGAGCAAGTAC
GACTCCATGGATTTCGATAGTTTACTTCGAGAGGCGCAAGGCAGCTTCCGAAGG
>green_sea_turtle_PPARGC1A
ATGGCGTGGGACATGTGCAACCAGGACTCTGTATGGAGTGATATAGAGTGTGCTGCTCTT

GCTGGTGAAGCCCAGCCTCTTTGCCCAGATCTCCCAGAACTTGACCTCTCTGAACTAGAT
GTGAATGACTTGGATGCAGACGGTTTTCTGGGTGGACTCAAGTGGTACAGCGACCAATCA
GAAATCATTACCAATCAGTACAGCAATGAATCATCAAATATATTCGAGAAGATAGATGAA
GAGAATGAAGCAAACTTGCTAGCAGTTCTCACTGAGACACTGGACAGTATCCCTGTGGAT
GAGGATGGATTGCCTTCATTTGATGCACTGACAGATGGAGATGTGACGAACGAAAATGAT
GCTAGCCCTTCACCAATGCCAGACGGCACCCCTCCGACTCAGGAGGCAGAAGAGCCGTCT
CTACTTAAGAAGCTCTTGCTGGCTCCAGCCAACATTCAGCTAAATTACAATGAATGCAGT
GGTCTCAGCACACAAAACCATGCAAACACTAATCACAGGATCAGAACAAGCCCTGTGGTT
GTTAAGACCGAGAATTCATGGAGCAATAAACCGAAGAGCATTTGTCAACAGCAAAAGCCA
CAAAGACGTCCCTGCTCTGAGCTTCTCAAGTATCTGACTACAAATGATGACCCTCCTCAG
ACCAAGCCAACAGAGAACAGAAACAGCAGCAAAGACAAATGCATCTCCAAAAAGAAGCCC
CATCTTCAGTCTCAGGCACATCATTTGCAAGCCAAACCAACAAGTTTATCACTTCCTTTG
ACACCTGAGTCACCAAATGATCCCAAGGGTTCCCCATTTGAGAACAAGACTATTGAACAA
ACCTTAAGTGTGGAACTCTCTGGAACTGCAGGCCTAACTCCACCTACGACCCCTCCTCAC
AAAGCCAACCAAGATAATCCTTTCAGGACTTCACCCAAGCTGAAGTCATCATGCAAGACT
ATTGTACCACCCTCAAAAAAGCCCCGCTACAGTGAGTCTTCCAGTTCTCAAGGACATAAC
ACAATCAAGAAGAGTCCAGAACAGTCTGAGCTGTATGCACAGCTTAGCAAGACAACGATA
CTGTCCAGTGGACATGAGGAGAGAAAGACAAAACGGCCTAGTTTGCGGCTGTTTGGTGAC
CATGACTACTGTCAATCTGTGAATTCAAAAACCGAAATACACATTAAAATATCCCAGGAA
CTTCAGGACTCCAGACAACAAGAATTTAAAGATTCTGTACCTGGGTGGCAGTGTCAGATT
TGTTCTTCTTTAGAACAAGACCAGTACTACAAGAGAGAGACTTTACAGGCAAGTAAGCAG
GGTTCCCACTCTAGCAGCCGAAAACAGCTCCAAGACCAGGATATCCGGGCTGAACTGAAT
AAGCACTTTGGTCACCCCAGCCAAGCTGTTTTTGATGAAGAGGCAGATAAGGCCAGTGAA
CTGAGGGAAAGTGATTATAGTAATGAACAATTCTCCAAACTACCTATGTTTATAACTTCA
GGACTAGCTATGGATGGCCTCTTTGATGACAGTGAAGATGAAAGTGATAAACTATGCTAC
CCTTGGGATGCGACAGAAGCCTATTCATTGTTTGATGTATCGCCTTCTTGCTCTTCTTTT
AACTCCCCAAGCAGAGATTCAGTGTCTCCACCCAAATCCTTATTTTCTCAAAGATCCCAA
AGGATACGCTCTAGATCAAGGTCCTTTCCTCAACACAGGTCTTGTTCCCGTTCTCCATAT
TCCCGATCGAGATCAAGGTCGCCATGTAGCAGATCCTCTTCCAGATCGTGTTACTATTAT
GAGTCCAGCCACTGTAGACATCGAGCATACAGAAGTTCTCCTTTATATGCAAGATCACGA
TCCAGATCACCATATAGTTGTAGACCAAGATATGACAGCTATGAGGAATACCAGCATGAA
AGACTGAAGAGGGAAGAATACCGCAAAGAGTATGAAAAACGGGAATCTGAAAGGGCCAAA
CAAAGGGAGAGGCAGAGACAGAAAGCAATTGAAGAACGTCGTGTGATGTATGTGGGTAAA
ATCAGAGCTGATACGACCCGAACAGAATTGAGGGACCGGTTTGAAGTTTTTGGTGAAATT
GAGGAGTGTACAGTACATCTGCGGGATGATGGAGACAGCTATGGTTTCATCACCTACCGC
TACACTTGTGATGCGTTTGCTGCTCTCGAAAATGGATACACTTTGCGGAGGTCAAATGAA
CCTGACTTTGAGTTGTACTTTTGTGGACGCAAGCAATTTTGCAAGTCTAACTATGCAGAC
CTAGACTCAAACTCAGATGATTTTGATCCTGCTTCCACTAAGAGCAAATATGACTCCATG
GATTTTGATAGTTTACTTAAAGAGGCACAGCGAAGCCTACGTAGG
>Chinese_alligator_PPARGC1A
ATGGCGTGGGACATGTGCAACCAGGACTCTGTATGGAGTGATATAGAGTGTGCTGCTCTG
GTTGGTGAAGACCAGCCTCTTTGCCCAGATCTCCCAGAACTTGACCTCTCCGAACTAGAT
GTGAATGATTTGGATGCAGACAGCTTTCTGGGTGGACTCAAGTGGTACAGCGACCAATCA
GAAGTTATTTCCAATCAGTACAACAATGAATCATCAAATATATTTGAGAAGATAGATGAA

GAGAATGAGGCAAACTTGCTGGCAGTTCTCACAGAGACACTGGACAGTATCCCTGTGGAT
GAGGATGGATTGCCTTCATTTGATGCACTGACAGATGGAGAAGTGACCAATGAAAATGAT
ACTAGCCCTTCACCAATGCCTGACGGCACCCCTCCAGCTCAGGAGGCAGAAGAGCCGTCT
CTACTTAAGAAGCTCTTGCTGGCTCCAGCCAACACTCAGCTAAATTACAATGAATGCAGT
CGCCTCAGCACACAAAACCATGCAAACACTAATCACAGGATCAGAACAAGCCCTGTGGTT
GTTAAGACCGAGAATTCATGGAGCAATAAATCAAAGAGCATTTGTCAACAGCAGAAGCCA
CAAAGACGTCCCTGCTCTGAGCTTCTCAAGTATCTGACTACAAATGATGACCCTCCTCAG
ACCAAACTGACAGAGAACAGGAACAGCAGCAAAGACAAATGCAACTCCAAAAAGAAGCCC
CAACTGCACTCTCAGGCACATCATTTGCAAGCCAAACCAACAAGTTTATCACTTCCTTTG
ACACCTGAGTCACCAAATGATCCCAAGGGTTCCCCATTTGAGAACAAGACTATTGAACAA
ACCTTAAGTGTGGAACTCTCTGGAACTGCAGGCCTAACTCCACCTACGACCCCTCCTCAC
AAAGCCAACCAAGATAATCCTTTTAGGACTTCACCTAAGCCGAAGTCATCATGCAAGACT
ATTGTACCACCTCCAAAAAAGCCTCGCTATAGTGAGTCTTCCAGTTCTCAAGGAAATAAC
CCAATCAAGAAGGGTGCAGAACAGTCTGAGCTGTATGCACAGCTCAGCAAGACAACAGTA
CTGTCCAGTGGACATGAGGAGAGAAAGACAAAACGGCCTAGTTTGCGGCTGTTTGGTGAC
CACGACTATTGTCAGTCTGTGAATTCAAAATCAGAAATACATATTAAAATATCCCAGGAA
CTTCAGGAGTCCAGACAACTAGAATTTAAGGATTCTTCACCTGCATGGCAGTGTCAGATT
TGTTCTTCTTTAGAACAAGACCAGTATTACAAGAAAGAGACTTTACAGACAAATAAGCAG
GGTTCCCACTGCAATAATAGAAAGCAGCTCCAAGACCAGGAAATCCGGGCTGAACTGAAT
AAGCACTTTGGTCACCCCAGCCAAGCTGTTTTTGAAGAAGAGGCAGATAAGACCAGTGAA
CTGAGGGACAGTGATTATAGTAATGAACAGTTCTCCAAACTACCTATGTTTATAAATTCA
GGACTAGCAATGGATGGTCTCTTTGATGATAGTGAAGATGAAAGTGATAAACTATGCTAC
CCTTGGGATGGGACACAAGCCTATTCTTTGTTTGATGTATCGCCTTCTTGCTCTTCTTTT
AACTCTCCATGCAGAGATTCAGTGTCTCCATCCAAATCTTTATTTTCTCAAAGATCCCAA
AGGATACGCTCTAGATCAAGGTCCTTTCCTCAACGCAGGTCTTGTTCCCGTTCTCCATAT
TCCCGATCGAGATCAAGATCGCCATGTAGCAGATCCTCTTCAAGATCTTGTTACTATTAT
GAGTCCAGCCACTGTAGACACCGAGCATACAAAAGTTCTCCCTTACATGCAAGGTCACGA
TCCAGATCACCCGGTAATCGTAGACCCAGATATGACAGCTATGAGGAATATCAGCATGAA
AGGCTGAAGAGGGAAGAATACCGCAAAGAGTATGAAAAACGGGAATCGGAAAGGGCCAAA
CAAAGGGAGAGGCAGAGGCAGAAAGCTATTGAAGAACGTCGTGTGATATATGTGGGTAAA
ATCAGACCTGACACAACTCGAACAGAACTGAGGGACCGCTTTGAAGTTTTTGGTGAAATT
GAGGAGTGCACAGTAAATCTACGGGATGATGGAGACAGCTATGGTTTCATCACCTACCGC
TATACTTGTGATGCTTTTGCTGCTCTTGAAAATGGATACACTTTACGCAGGTCAAATGAA
CCTGACTTTGAGCTGTACTTTTGTGGACGCAAGCAATTTTGCAAGTGTAACTATGCAGAC
CTAGATTCAAACTCAGATGATTTTGATCCTGCTTCCACTAAAAGCAAGTATGACTCCATG
GATTTTGATAGTTTACTGAAGGAGGCACAGCGAAGCCTGCGTAGG
>Gharial_PPARGC1A
ATGGCGTGGGACATGTGCAACCAGGACTCTGTATGGAGTGATATAGAGTGTGCTGCTCTG
GTTGGTGAAGACCAGCCTCTTTGCCCAGATCTCCCAGAACTTGACCTCTCCGAACTAGAT
GTGAATGATTTGGATGCAGACAGCTTTCTGGGTGGACTCAAGTGGTACAGCGACCAATCA
GAAGTTATTTCCAATCAGTACAACAATGAATCATCAAATATATTTGAGAAGATAGATGAA
GAGAATGAGGCAAACTTGCTGGCAGTTCTCACAGAGACGCTGGACAGTATCCCTGTGGAT
GAGGATGGATTGCCTTCATTTGATGCACTGACAGATGGAGAAGTGACCAATGAAAATGAT
ACTAGCCCTTCACCAATCCCTGACGGCACCCCTCCAGCTCAGGAGGCAGAAGAGCCGTCT

CTACTTAAGAAGCTCTTGCTGGCTCCAGCCAACACTCAGCTAAATTACAATGAATGCAGT
GGTCTCAGCACACAAAACCATGCAAACACTAATCACAGGATCAGAACAAGCCCTGTGGTT
GTTAAGACCGAGAATTCATGGAGCAATAAAGCAAAGAGCATTTGTCAACAGCAGAAGCCA
CAAAGACGTCCCTGCTCTGAGCTTCTCAAGTATCTGACTACAAATGATGACCCTCCTCAG
ACCAAACCAACGGAGAACAGGAACAGCAGCAAAGACAAATGCAACTCCAAAAAGAGGCCC
CAACTGCACTCTCAGGCACATCATTTGCAAGCCAAACCAACAAGTTTATCACTTCCTTTG
ACACCTGAGTCACCAAATGATCCCAAGGGTTCCCCATTTGAGAACAAGACTATTGAACAA
ACCTTAAGTGTGGAACTCTCTGGAACTGCAGGCCTAACTCCACCTACAACCCCTCCTCAC
AAAGCCAACCAAGATAATCCTTTTAGGACTTCACCTAAGCTGAAGTCATCATGCAAGACT
GTTGTACCACCTCCAAAAAAGCCTCGCTATAGTGAGTCTTCCAGTTCTCAAGGAAATAAC
CCAATCAAGAAGGGTCCAGAACAGTCTGAACTGTATGCACAGCTCAGCAAGACAACAGTA
CTGTCCAGTGGACATGAGGAGAGAAAGACAAAACGGCCTAGTTTGCGGCTGTTTGGTGAC
CACGACTATTGTCAGTCTGTGAATTCAAAATCAGAAATACATATTAAAATATCCCAGGAA
CTTCAGGAGTCCAGACAACTAGAATTTAAGGATTCTTCACCTGCATGGCAGTGTCAGATT
TGTTCTTCTTTAGAACAAGACCAGTATTACAAGAAAGAGACTTTACAGACAAATAAGCAG
GGTTCCCACTGCAATAATAGAAAGCAGCTCCAAGACCAGGAAATCCGGGCTGAACTGAAT
AAGCACTTTGGTCACCCTAGCCAAGCTGTTTTTGAAGAAGAGGCAGATAAGACCAGTGAA
CTGAGGGACAGTGATTATAGTAATGAACAGTTCTCCAAACTACCTATGTTTATAAATTCA
GGACTAGCAATGGACGGTCTCTTTGATGATAGTGAAGATGAAAGTGATAAACTATGCTAC
CCTTGGGATGGGACACAAGCCTATTCTTTGTTTGATGTATCGCCTTCTTGCTCTTCTTTT
AACTCTCCATGCAGAGATTCAGTGTCTCCATCCAAATCTTTATTTTCTCAAAGATCCCAA
AGGATACGCTCTAGATCAAGGTCCTTTCCTCAACGCAGGTCTTGTTCCCGTTCTCCATAT
TCCCGATCGAGATCAAGATCGCCATGTAGCAGATCCTCTTCAAGATCTTGTTACTATTAT
GAGTCCAGCCACTGTAGACACCGAGCATACAAAAGTTCTCCCTTGCATGCAAGGTCACGA
TCCAGATCACCCGGTTATCGTAGACCCAGATATGACAGCTATGAGGAATATCAGCATGAA
AGGCTGAAGAGGGAAGAATACCGCAAAGAGTATGAAAAACGGGAATCGGAAAGGGCCAAA
CAAAGGGAGAGGCAGAGGCAGAAAGCTATTGAAGAACGTCGTGTGATTTATGTGGGTAAA
ATCAGACCTGACACAACTCGAACAGAACTGAGGGACCGCTTTGAAGTTTTTGGTGAAATT
GAGGAGTGCACAGTAAATCTACGGGATGATGGAGACAGCTATGGTTTCATCACCTATCGC
TATACTTGTGATGCTTTTGCTGCTCTTGAAAATGGATACACTTTACGCAGGTCAAATGAA
CCTGACTTTGAGCTGTACTTTTGTGGACGCAAGCAATTTTGCAAGTCTAACTATGCAGAC
CTAGATTCAAACTCAGATGATTTTGATCCTGCTTCCACTAAAAGCAAGTATGACTCCATG
GATTTTGATAGTTTACTGAAGGAGGCACAGCGAAGCCTGCGTAGG
>African_clawed_frog_PPARGC1A
TATGGAGTGACATAGAGTGTGCTGCTCTGGTCGGTGAAGACCAGCCTCTGTGCCCCGATC
TTCCTGAACTTGGTCTCTCTGAACTTGATGTTAATGACTTAGATGCCGACAGCTTCTTGG
GTGGATTAAAGTGGTACAGTGACCAATCAGAAAACATTTCCAATCAATACAGCAGCGAAT
CATCCAACATATTTGAGAAGATAGACGAGGAGAATGAAGAGAATTTGCTAGCAGTTCTTA
CAGAGACATTGGACAGTCTCCCTGTGGATGAGGATGGATTGCCTTCATTTGATGCACTGA
CAGATGGAGATGTGACCAATGAACATGAGCCTAGCCTTTCACCTATGCCTGACGGCACCC
CTCCAATTCAGGAGGCAGAAGAGCCGTCACTACTTAAGAAGCTGTTACTGGCTCCAGCTA
ACGCTCAGCTGATTTACAATGAGTGCGTTGGTTTCACTACGCAGAACCATGGCAGCTCCA
GTCAGAGGATCAGAACCAATTCTTCGGTTATTAAGACCGAGAATCCATGGAGCAGTAAAC
CGAGGGCCATTTGTCACTCCCAAAAGCCACCACGGCGTCCTTGCTCAGAGCTCCTTAAGT

ACCTGACTTCAAATGACGACCCTCCTCAGACCAAATCGACAGAGAGCAGAAACAACAGGC
TTGACAAATGCAGCAGCAAAAAGAAGCCGTACTTACAGCCCCAGCAACATTATCAAGCCA
GGTCAATGAATTTGTCGCTCCCCTTGACGCCCGAGTCACCAAATGACCCCAAGAGTTCCC
CATTTGAGAGCAAGACTATTGAACGAGGCTTATGTATGGAGCTCTCTGGAACTGCAGGAT
TAACTCCACCTACAACACCTCCTCACAAAGCCAACCAAGAGAACCCCTTCAGGACCTCTC
CTAAGTTGAAATCTTGCAAATCTTCTGTGCCACCTGCTAAAAAATCACGCTACATTGGGT
CTTCCAGTATCCAAGTTCTATATCCAGCTAAGAAAGGTCCAGAGCAATCAGAACTTTATG
CACAGCTCAGTAAAGCGACTGTGGTAATTGGACAAGAGGAGAAAAAATCAAAGCGACCTA
GTTTACGACTTTTTGGAGACCACGACTACTGTCAGTTTATGACTTCAAAATCTGAAAGAC
ATATTAGTTTATCACAACAATTACAGGCCTCAAGACATCTTGAATGTAAGGATCCTTTGC
CTGGCAAGGAACTGCAAGTCTGTTCAAACACTGACCAAGAGCAATGCCAGAAAGACAGCT
TACCAGTCACAATGCCAAGTTCTCAGAACAGCCATAGGAAACCGCTCCAGGACCAGGAAA
TACGAGCCGAACTCAACAAGCACTTTGGGCACCCAACGCAGGCAGTCTACAACAATGAAA
CTAAAATAAGTGAACTGGTGGACAGTAAATACAGTGATGAACAGTTGTCAAGACTACCTT
TGTTTCTAACTGCCGGGTTGGGAATAGATAGTTTGTTTGATGACAGCGAGGATGAAAATG
ATAAACTGTGCTATACTTGGGATGAAACACAGTCATATTCATTATTTGATGAATCACCGT
CTCGCTCTACTTTTAATTCTCCAAGTAGATATTCAGTATCCCCACCAGCATCCCTGTTTT
CACAAAGGATATGCTCTAGATCAAGATCTAGGTCCTTTTCACAGTTCAGATCATCTTCTC
GTTCCCCATATTCTCGTTCAAGATCCAGATCTCCATCAAGCAGATCATCATCAGGGTCTT
GCTGCTACTCTGAGACTGATCATTGCAGAGAAAAAAGTTCTCCAATGTATGCACGGTCAA
GGTCACCGCGTGGTCGTAGGCCCAGATATGACAGCTATGAGGAATACCAACATGAACGGC
TGAAGAGGGAGGAGTACCGCAAGGAGTATAAAAAGCGTGAATCCGAGAGAGCAAAACAAA
GAGAGAGACAAAGGCAAAAAGCCATTGAAGAACATAGAGTGATTTTCGTGGGTAAAATGA
GATCTAGCATGAGCCGCACGGAGCTGCGAGCACGGTTTGAGGTTTTTGGAGAAATTGAGG
AATGCACAGTAAATCTGCGGGACGATGGCAATTGTTACGGATTCATCACCTATCGCTACA
CCTGTGACGCTTTCGCTGCTCTTGAAAATGGATACACGCTGCGCAGGTCAAATGGGCCAG
ACTTTGAGGTTTGCTTTTGTGAAAGAAAGCAGGTGTGCAAGTCCAACTATGCAGACTTAG
ATTCAAATTCAGATGACTTTGATCCTGCTTCCACCGAAAGCAAGTACGACTCCATGGATT
TTGACAGTTTGCTCAAGGAGGCG
>Tibetan_frog_PPARGC1A
ATGGCGTGGGACATGTGCAACCAGGACTCTGTATGGAGTGACATAGAGTGTGCTGCTCTG
GTTGGTGAAGACCAGCCCCTTTGCCCAGATCTTCCAGAGCTGGATCTTTCTGAACTCGAT
GTGAACGATTTGGATGCAGACAGCTTTTTGGGTGGATTAAAGTGGTACAGTGACCAATCA
GAAAACATTTCCAATCAATACAGCAACGAGTCATCAAACATATTTGAGAAAATTGAGGAG
GAGAATGAAGAGAACCTGCTAGCAGTTCTTACAGAAACACTGGACAGTCTCCCTGTGGAC
GAGGATGGATTGCCTTCCTTCGATGCACTGACAGATGGAGATGTGACCAGTGAACATGAT
CCCAGACTTTCATCCCTGCCTGACGGCACCCCTCCAACACAGGAGGCAGAAGAGCCGTCA
CTACTTAAGAAGCTCTTACTGGCTCCAGCGAACACTCAGCTGATTTATAATGAATGTGTC
GGACTCAGTGCACACAACCATGCAAGCCCTAATCAGAGGATCAGAACAAGCTCTGCACCT
GTTAAGAGCGAGAACTGTTGGAGCAATAAACCCAGAAACATTTGTCAACCGCAGAAGCCA
CAGAGGCGCCCCTGCTCGGAGCTCCTTAAGTACCTGACTGCAAATGATGACTCTCCTCAG
ACCAAATCAACAGAGAGCAGGACGAATAACAGACTTGACAAATGCACCAAAAAGAAGCCC
TGCTTGCATCCTCTGCCACATTTTCAAGCCAAGTCAACGAATCTGTCCCTTCCTCTGACA
CCCGAGTCACCAAATGACCCCAAGGGTTCCCCATTTGAGAGCAAGACTATTGAACGAACC

TTAAGTGTGGAGCTCTCTGGAACTGCAGGACTAACTCCACCAACAACACCACCTCACAAA
GCCAACCAAGATAACCCATTCAGGACTTCTACCAAGCCGAAGTCTTCTTGTAAGCCTGTT
GTGCCCCCCGCTAAGAAATCTCGCTACATTGAGTCTTCCACTATTCAAGTTATTAACTCT
AAAAAAGGTCCCGAACAATCTGAACTGTATGCACAGCTGAGCAAAACCTCCGTGGTCATT
GGACAAGAGGAGAAAAAAGTGAAGCGACCTAGTTTACGGCTTTTTGGTGACCATGATTAC
TGTCAGTTTATGAATTCAAAATCAGAAATACGTATAAGCTTAACACAAGAATTACAGGCC
TCAAGACAACTTGAATGTAAGGATCCTCTGCCTGGGAAAAAAGTACAGATCTATTCAACT
AAAAAACAGGAGAACCTGAAAGACAGTTTTGAGTTGGCGATGCCAAGTCCTCAGAATGGC
AATAGGAAGCCACTGCAAGACCAAGAGATCCGTGCAGAACTTAATAAGCACTTTGGTGAC
CCAACACAAGCAGTCTATGAGGAAGAGAACAAAAATGGTGATGCAAGGAGCAATGATTAC
AGTGTTGATCAATTCTCTAGACTACCTATGTTTTTAACCTCTGGACTGGGTATGGATAAT
TTGTTTGATGAAAGCGAAGATGAAAATGACAAACTGTGCTATTCCTGGGATGGAATACAA
TCCTATTCATTGTTTGACGTATCACCTTCCTGCTCAACTTTCAATTCTCCAAGCAGATAT
TCTGTTTCCCCACCAACATCCCTTTTATCGCAAAGGATATGTTCTCGGTCGAGATCTAGA
TCTTATTCTCAAAACAGATCATGTTCTCAGTCTCCATATTCACGTTCAAGATCAAGATCT
CCATCCAGCAGATCTTCTTCAGGGTCATGCTGCTGCTATGATACTGATCATTGTAGACCC
AACAATTCTGCAGTCTGTGCACGATCACGGTCAAGATCCCCATACAATCGTAGGCCCAGA
TACGACAGTTATGAGGAATACCAACATGAGCGGCTGAAGAGGGAGGAATACCGCAAAGAG
TATGAGAAACGTGAATCAGAGCGAGCCAAACAAAGGGAACGGCAAAGGCAAAAAGCAGTT
GAGGAACATAGAGTTCTCTACGTCGGTAAGATGAGAACGAACATGACCCGTAGTGAACTG
CGAGCCCGTTTTGAAGTTTTTGGTGAAATTGAAGAATCTACAGTCAATCTGCGGGATGAC
GGAGACTGTTACGGTTTCATCACCTACCGCTACACCTGTGACGCATTTGCTGCTCTTGAA
AATGGATATACTCTGCGCAGGTCAAATGAACACGACTTTGAGCTATGCTTTTGCGAACGA
AAGCAGTTTTGCAAGTCCAACTATGCAGATTTAGATTCAAATTCAGATGACTTCGATCCT
GCTTCCACCAAAAGCAAATATGACTCCATGGATTTTGACAGTTTGCTTAAAGAGGCACAA
AGGAGCCTGCGTAGG
>southern_platyfish_PPARGC1A
ATGGCGTGGGACAGGTGTAACCAGGACTCGGTGTGGACAGAATTAGAGTGTGCTGCCTTG
GTTGGTGAAGACCAGCCCCTCTGCCCAGACCTTCCTGAACTTGACCTCTCAGAGCTGGAT
GTCAGCGACTTGGATGCTGACAGCTTTCTGGGTGGCCTTAAATGGTACAGCGACCAATCA
GAGATCATTTCCTCCCAGTATGGGAATGAAGCGTCTAATCTTTTTGAGAAGATAGATGAA
GAAAATGAGGCCAACTTACTGGCAGTGCTTACAGAGACCCTGGACAGCATCCCGGTGGAT
GAGGACGGATTGCCTTCGTTTGAGGCCCTGGCAGATGGGGACGTGACCAATGCCAGTGAC
CGGAGCTGTCCCTCCTCCCCAGACGCCTCACCTCACACCCCAGAGCCCGAGGAGCCTTCT
TTGCTGAAGAAGCTCCTTCTGGCGCCCGCAAACTCCCAGCTCAGCTATAATCAATACACA
GGTGGCAAGGCACAGAACCATGCAGCCAGCAGCAACCACCGGATCAGACCACCACCTGCC
GTCGTCAAGACGGAGAACCCCTGGAGCGGCAAAGCAAGAGGGGGCTCCGGCCAACAGAAC
CGCCCGGTGAGGCGACCTTGCACTGAGCTGCTCAAATACCTAACAGCCACTGATGACATC
CTGCTACACACCAAAGCCAGCGATGCCAAGAGCGCCTGGGGGGGTCCCAGTAGCAGGGAC
AAGAGTAGCCTGGGTCTCAGTGCCTCATCCTCGTCCTCATCGCCATCCTCGTCATCCACC
TCTTCGTTCTCCTCCCTGTCTTCCACTTCTTCCTCCTCTTCGTCCACCACCTCCAAGAAG
AAGTCGGCTGTGTCATCACAACAACAGCAGCAGCAGTCGCCGCAGCAGCATCACCAGCGA
GCCAAACCAACCACCTTGCCACTTCCTTTGACCCCAGAGTCTCCAAATGACCACAAAGGA
TCACCCTTTGAGAACAAAACCATTGAACGCACATTAAGTGTGGAGATTGCTGGAACCCCA

GGTCTGACACCACCTACCACGCCCCCACACAAAGCCAGTCAAGAGAATCCTTTCAAAGCA
TCTCTGAAAACCAAGTTGTCCTCATGTTGCTCCTCTGCCTTGACGTGCAAAAGAGCCAGG
CTGAGTGAGTTGGGCGCCGGCGCTTTGGCCCCGGCCCCAGGTGCCTCAGCCAGGGGCCCT
GCCAGGAAGGGTCCCGAACAGACTGAGCTCTATGCGCAGCTAAGCAAAGCATCCACCGCC
CTCCCTTACGCCATCGCTCGACGTGCACTGGGGGGCGGCCTTGAGGGGCACTGCAGCACT
GGCAACAACAAGCGGGCCACTGGGCAGGATGGCCACAGCGACCATGACTATTGCCAGGCA
CTGGCTGCTTGTAAGATGGATGGGGGCATATCCGCTGTTTCCATGGCTACATCTTTGGAA
ATGACATTCACCCCAGGTGCCACCTCCACTGGCAAGGTGGAGAACAGGCATGTCGAATGT
ATGGACTTAGTCATGTCAACATCGTTTCTATCATCTTCTTGTTCTACATCATCATCCTCT
CTTAGTCATTTGACTAAAGAGCAGAAATGTGTCCCCGTGGATGGAGATGCTTGCCGTGTC
AGGGGCTTAGGGGAGCGTACCCTTCCACAAACCACTCAGATACCCACACAGGGGGGCACT
GCTGTTAGGGACCAACTGCAACTTTCTGCCACCAGCCGGAAGCCCCTGTGCGACCAGGAA
ATCAGAGCAGAACTCAACAAGCACTTTGGCCCTCCCCTGCAAGCCCTTTATACTCAGGGT
GAGCAGGAGAGAGACGATAGCAGTAAACCAAACAAGCCTACAACACCCCTGTCCCCTGAG
GGGGGAGAGATACACTTTTACTCCCAGAGACTGTCTGGATCCAGCTACCTGCACCCAGGG
TTTCTGTCCTTTCATAATGAGCTGGAGCTGGATGATGGCCGTGAGAGTCGCTTCATCTTT
CCATGGGAGGGCACCCCTCTGGACCTACTCTTTGACTGCTCTCCCCATTCTCCCTCCTGT
TCCCCACCATCCACTTGCTCCCCTTCACGAGGCTCCATCTCTCCACCGTCCTCCCTTCTC
CTGTCACCTAGTCGACCGTTCTGCTGGACCAGCAGCGGGTCCCGTTCCCGTTCACGGTCC
CACTCTGGCTCCCGCAGCTCCTCATCACGATACCGCAGGCGCTCTCTGTCCAGCTCACCA
GACAGACTCCAAAGGACTCCAAATCACTTTACACTATGGTCTCATCATAGCTCAGATCTG
AACGCTGTCCGCTCCAGACTCCATAAGAGCCCTCACCCTCAGTCCCGGTCTCCGCTCAGC
CACAGGCCAAGGTACGACAGCTACGAGGAGTACCAGCACGAGAGGCTTAAGAGGGAGGAG
TACCGACTGGATTATGAGAAGCGGGAGTTTGAAAGGGCCGAGCAGAGGGAGAAGCAAAGG
CAAAAAGCCATAGAGGAGAGACGGGTGGTGTACGTGGGGCGACTGAGGTCCGACTGCAGC
CGATCAGAGCTGAAGCGCCGCTTTGAAGTCTTTGGGGAAATTGAAGAATGTGCAGTGAAC
CTGAGGGACGATGGGGACAATTTCGGCTTCATCATGTTCCGCTACACCTGTGACGCCTTT
GCCGCCCTTGAGAACGGACACACCCTACGCAGGTCGAACGAGCCTCAGTTCGAGTTGTGT
TTTGACGGACAAAAGCAGCTCTGCAAATCACGATACACAGACTTGGACTCCCATTCGGAC
GACTTTGATCCGACTTCCACGAAGAGCAAGTATGACTCCATGGATTTTGATAGCTTGCTG
AAAGAGGCCCAGTGCAGCCTGAGAAGG
>spotted_gar_PPARGC1A
ATGATGGAAGGTTATGCGCTTTCTGAAGATGCATTATTTTCCTCCTGCCTTTACAACTAC
ACCTGGGAAAATCCATACGACCAGTGTGCTGCCTTGGTTGGTGAAGACCAGCCTCTCTGC
TCCGACCTCCCAGAGCTTGACCTTTCAGAGCTAGATGTCAGTGACCTTGATGCTGATAGT
TTTCTGGGAGGACTCAAATGGTACAGTGACCAATCAGAAATCATTTCCAGTCAGTATGGC
AGTGAACCTTCAAATCTGTTTGAGAAGATAGATGAAGAAAATGAGGCCAACTTGCTGGCA
GTTCTCACCGAGACCCTGGACAGCCTCCCGGTGGATGAGGACGGATTGCCTTCATTCGAT
GCTCTGGCAGAAGGCGACGTGACCAACACCAGTGATCAGAGCTGCCCATCCTCACCTGAT
GGCTCTCCTCCCACACCAGAAGCAGAGGAGCCATCTCTACTAAAGAAGCTCTTGCTGGCA
CCAGCTAACTCCCAGCTCAGTTATAATCAATACACAGGTGGCAAGGCACAAAATGAGGCT
CCTAGTAATCAAAGGATCAGACCAACACCTGCTGTTGTCAAGACAGAAAACCCATCCTGG
AATAATAAGCAGAGGGTCCCCTGTCAGCAGCAGCAGAAGCCCACACGACGCCCTTGTGCC
GAGCTTCTCAAATACCTAACAGCCAGTGACGAGACCATCCAGACCAAAGCCTGCGAGCAG

AAAAGCGGTGGGGGTAGCAGCAGCAAAGTAAAAAGCGGAGGTGCCTCTTCATCCTCATCC
TCTGTTGCCTCCTCCTCCTCATCCTCTTCTAAGAAGAAGCCAGTTCATCCCTCTCAGCAG
CAGCTGCATCACCAGCGAGCCCTTAGGAACGGCCATGGCAATTCCCGCGTCAAAAGGGAA
CGCACGCAATCCAGTGAAGAAGGAAGGCCGCCAGGTCAGCAACCTGCCCTGGATGCTGGG
GTCCTGGCAGCCGCTAGATTCATTAGGTACATGCATTCTTATTCTCTCCGTCCTAGAGAG
AAAGATCCGTCAGGCAGGTGCGAGCAGTGTTATGAAGAGTACAGAAACCAAAGTACTTTA
GGTAGCACCCATCGGAGAAGGGATGAATTTGACAGGCCCATTCCAGGGCATGTAAAAGTG
ACCATTAAGAAAAGAGAAGATCTCGGAGTTCGATTGCATGGGGAGATGCAGAACTCTAAA
CAACAACAGTATAACTGTCGGGCCCAGAAACGTAGTCCACCGTTACCGACCGAGACAGAG
AGGGAAGAGATTAGGAGTGCTCGCCAAGTAGGTCTTCGCAGCGTCCCCATGAAGAAGAAA
CGCCCAAGCAAGAGCGGTGAGAGAACGGAGAGGCGAGCATGCAGCCCAGACAGAGCTGTC
AGGAAATGGGCTGCTCAACCCAGTCTCCAGTCCCTGCTTCTGGAAGGGGTAGGCGTTGAT
CGCTGGGGTAGTGAGACAGATGAAGAGATGGCGCGGGAAGCGAAAGGCAGGGCGGAGCAG
GTCTGCTGTGGTGGGAATGGTGGCCACAATCATGTCATGGGCAGCCCCATGCAGCTCTCA
CTGGGCCCCCCAAGCTCCCTCTTCCCGGACTCTATTATTCCAATAGGAGACCTCACACCC
TCCGTCACACAAGAGCACCACCTTTACAGGCAGGCATTCTGCAAGTACCAAGATGACCAG
GTCCTCCCCCTGTTAGCCAAACCAACCACCTTGCCACTTCCTTTGACCCCAGAGTCTCCA
AATGACCAAAAAGGATCTCCATTTGAGAACAAAAGCATTGAACGGACATTGAGTGTGGAG
CTCTCTGGAACAGCAGGTCTGACACCACCTACCACTCCTCCACACAAGGCCACTCAAGAA
AATCCTTTCAAACCTTCAGTCAAGCCCAAGTTGTCTTCATGCAGCTCCTCAGCTCAAGCA
AGTAAAAGACCTCACTACAGTGATTCTGCTGTTTGCCCCTCTATCAAAAAGGGTCCAGAA
CAGACTGAGTTGTATGCCCAGCTTAGCAAAGTCTCTGCATTACCTGCAGGGCAGGAGGAC
CGCAAAAGCAAGCGCTCCACTCTGCGTCTGTTTGGTGATCACGATTATTGCCAGTTTGTA
AGCACAAAAAGAGACATTTCCCTGGCCTACTCAGTGGAATCTCAGGATGGGAGGCAACTG
GAATGTTGGGACTTGGTCTTGTCTCCCTCCTCTTTGGCTGTGCAGCAGCAGAATTACTCT
TCGCCTGACCAGGACCTCTGGTGCAACAGGGAAGGCAAACAGCTGCAGACATATCAAAAC
AGGCAGCAGGCTAACAGCAGGAAGCCGCTCCGAGACCAAGAGATCAGGGCGGAACTCAAC
AAGCACTTTGGTAACCCAAAACAAGCCATTTACAATGAGGATGAAAAGGCTGGCCCCCAG
CAGAGAGATGAAAGTGACTCTGAATATTACTACAAACTGACTGACTACGTGCACCCAGGC
CTGCCCATGGAAGGGGTTTTCGACGACAGCGAGGATGAGAACGAGAAGTTTCTCTACCCC
TGGGAAGGGACCCACCTGGACTTGTTGTTTGGGGGTTCTCCATCCTGTTCGCCTTCCAGT
TGCTCCCCTTCAAGGAGCTCTGTCTCCCCTCCCAAATCCCTCTTCTCTTCCCAGCGCTGT
CACTGGTCCAGGTCAGCGTCTCACTCCAGGTCCCGGTCGCGATCTTCGTCTTGCCACCGG
CGGCGGTCTTACTCCCGATCTCCTTACTCACGCTCCAGATCAAGGTCCCCCTACAGCCGG
TCCTCCTCACGGTCTCATCACTGTGTGGATTCCAGCAACACTAGACCAAGGTCTCGCAGA
AGCCCACCCATACATTCAAGATCACAGTCTAGATCCCCCTTCAGTCGGAGGCCAAGGTAC
GACAGCTACGAGGAGTACCAACATGAGCGACTTAAGAGGGAGGAGTATCGACGAGATTAT
GAAAAACGCGAGTTTGAAAGAGCCGAACAGAGAGAGAGGCAGAGGCAAAAGGCAATTGAG
GAGAGACGCGTGGTGTATGTGGGAAGACTAAGGCCTGACATTACCCGGACGGAACTGAAG
CGCCGCTTTGAAGTGTTTGGTGAAATCGAAGAGTGTGCAGTCAATTTGAGAGATGACGGG
GATAATTTTGGCTTCATCACCTACCGCTACACTTGTGATGCTTTTGCTGCTCTGGAAAAT
GGACACACCTTACGCAGATCTAATGAACCTCAGTTTGAGCTGTGCTTCGGTGGACGAAAG
CAGTTCTGCAAATCAAATTATACAGACTTGGATTCCCATTCTGATGACTTTGATCCATCA
TCCACAAAGAGCAAGTATGACTCCATGGATTTCGATAGTTTGCTGAAAGAAGCACAGCGC

AGCCTGCGAAGG
>guppy_PPARGC1A
ATGGACGGTTATGGTCGGACCGAAGATGAGCTGTTCTCCTCCTCCTGCCTTCTGAACCTC
ACCTGGGAGACTTGCTACGAGCAGTGTGCTGCCTTGGTTGGTGAAGACCAGCCCCTCTGC
CCAGACCTTCCTGAACTTGACCTCTCAGAGCTGGATGTCAGCGACTTGGATGCCGACAGC
TTTCTGGGTGGCCTTAAATGGTACAGCGACCAATCGGAGATCATTTCCTCTCAGTATGGG
AATGAAGCGTCTAATCTTTTTGAGAAGATAGATGAAGAAAATGAGGCCAACTTACTGGCA
GTGCTTACAGAGACCCTGGACAGCATCCCGGTGGATGAGGACGGATTGCCTTCGTTTGAG
GCCCTGGCAGATGGGGACGTGACCAATGCCAGTGACCGGAGCTGTCCCTCCTCCCCAGAC
GCCTCACCTCACACCCCAGAGCCTGAGGAGCCTTCTTTGCTGAAGAAGCTCCTTCTGGCG
CCTGCAAACTCCCAGCTCAGCTATAATCAATACACAGGTGGCAAGGCACAGAACCATGCA
GCCAGCAGCAACCACCGGATCAGACCACCACCTGCCGTCGTCAAGACGGAGAGCCCCTGG
AACGGCAAAGCAAGAGGGGGCTCCGGCCAACAGAACCGCCCGGTGAGGCGACCTTGCACT
GAGCTGCTCAAATACCTAACAGCCACTGATGACATCCTGCTACACACCAAAGCCAGCGAT
GCCAAGAGCGCCTGGGGGGGTCCCAGTAGCAGGGACAAGAGTAGCCTGGGTCTCAGTGCC
TCATCCTCGTCCTCGTCGCCATCCTCGTCATCCACCTCCTCGTTCTCCTCCCTGTCTTCC
ACTTCTTCCTCCTCTTCGTCCACCGCCTCCAAGAAGAAGTCGGCTGTGCCATCACAACAG
CAGCAGCAACAGCAGCAGCAACAGCAGCAGCAACAGCAGCAGCATCAGCATCAGCAGTCG
CCGCAGCAGCATCACCAGCGAGGTGAGAGCCGGGCTGCAGGCGAGTGTATTGTGGCTGGT
CATGGGGCTGGGAAGTGGCAGCGTTGCTCTCACGATGACGGGGTTGAGGATTCGGAGGGT
GCCTCTGTCCCTGTTGGTCGCAGAACCTACACCTGCGGCCATGCTCGCCCCAAACAGGAG
CACGGGCCACCCAATGAAGAAGGAAGGCCGCCAGGCGATGTTGGCCGCCTGGCCGCCGCT
AGGTTCATTAGTGACCACAAAGGATCACCCTTTGAGAACAAAACCATTGAACGCACATTA
AGTGTGGAGATTGCTGGAACCCCAGGTCTGACACCACCTACCACGCCCCCACACAAAGCC
AGTCAAGAGAATCCTTTCAAAGCATCTCTGAAAACCAAGTTGTCCTCGTGTTGCTCCTCG
GCCTTGACGTGCAAAAGAGCCAGGCTGAGTGAGTTGGGCGCCGGCGCTTTGGCCCCGGCC
CCAGGTGCCTCAGCCGGGGGCCCTGCCAGGAAGGGTCCCGAACAGACTGAGCTCTATGCG
CAGCTAAGCAAAGCATCCACCGCCCTTCCTTACGCCATCGCTCAACGTGCACTGGGGGGC
GGCCTTGAGGGGCACTGCAGCACTGGCAACAACAAGAGGGCCGCTGGGCAGGATGGCCAC
AGCGACCATGACTATTGCCAGGCACTGGCTGCTTGTAAGATGGATGGGGGCATATCCGTT
GTTTCCATGGCTACATCTTTGGAAATGACATTCACCCCGGGTGCCACTTCCACTGGCAAG
GTGGAGAACAGGCACGTCGTCGAATGTATGGACTCAGTCATGTCAACATCGTTTCTGTCA
TCTTCTTGTTCTACATCATCATCCTCACTTAGTCATTTGACTAAAGAGCAGAAATTTATC
CCCATGGATGGAGATGCTTGCCGTGTCAGGGGCTTAGGGGAGCGTACCCTTCCACAAACC
ACTCAGATACCCACACAGGGGGGCAGTGCTGTTAGGGACCAACTGCAACTTTCTGCCACC
AGCCGGAAGCCCCTGTGCGACCAGGAAATCAGAGCAGAACTCAACAAGCACTTTGGCCCT
CCCCTACAAGCCCTCTATACTCAGGGTGAGCAGGAGAGAGAAGATAGCAGTAAACCAAAC
AAGCCTACAACCCCCCAGTCCCCTGAGGGGGGAGGGACTGACTTTTACTCCCAGAGACTG
TCCGGATCCAGCTACCTGCACCCAGGGTTTCTGTCCTTTCAAGACGAGCTGGAGCTGGAT
GATGGCCGTGAGAGTCGCTTCATCTTTCCATGGGAGGGCACCCCTCTGGACCTACTCTTT
GACTGCTCTCCTCGTTCTCCCTCCTGTTCCCCACCATCCACTTGCTCCCCTTCACGAGGC
TCCGTCTCCCCACCGTCCTCCCTTCTCCTGTCACCTAGTCGACCGTTCTGCTGGACCAGC
AGCGGGTCCCGTTCCCGTTCACGGTCCCACTCCGGCTCCCGCAGCTCCTCATCACGATAC
CGCAGGCGCTCTCTGTCCAGCTCACCAGACAGACGTCCCTCCTCTTGGTCTCATCACAGC

TCCGATCTGAACGCCGTCCGCTCCAGACTCCATAAGAGCCCTCACCCTCAGTCTCGATCT
CCGCTCAGCCGCAGGCCAAGGTACGACAGCTACGAGGAGTACCAGCACGAGAGGCTTAAG
AGGGAGGAGTACCGACTGGATTATGAGAAGCGGGAGTTCGAAAGGGCCGAGCAGAGGGAG
AAGCAAAGGCAAAAAGCCATAGAGGAGAGACGGGTGGTGTACGTGGGGCGACTGAGGTCC
GACTGCAGCCGTTCAGAGCTGAAGCGCCGCTTTGAAGTCTTTGGGGAAATTGAAGAATGT
GCAGTGAACCTGAGGGACGATGGGGACAATTTTGGCTTCATCATGTTCCGATACACCTGT
GACGCCTTTGCTGCCCTTGAGAACGGACACACCCTACGCAGGTCGAACGAGCCTCAGTTT
GAGTTGTGTTTTGACGGACAGAAGCAGCTCTGCAAATCACGATACACAGACTTGGACTCC
CATTCGGACGACTTTGATCCGACTTCCACGAAGAGCAAGTATGACTCCATGGATTTTGAT
AGCTTGCTGAAAGAGGCCCAGTGCAGCCTGAGAAGG
>American_alligator_PPARGC1A
ATGGCGTGGGACATGTGCAACCAGGACTCTGTATGGAGTGATATAGAGTGTGCTGCTCTG
GTTGGTGAAGACCAGCCTCTTTGCCCAGATCTCCCAGAACTTGACCTCTCCGAACTAGAT
GTGAATGATTTGGATGCAGACAGCTTTCTGGGTGGACTCAAGTGGTACAGCGACCAATCA
GAAGTTATTTCCAATCAGTACAACAATGAATCATCAAATATATTTGAGAAGATAGATGAA
GAGAATGAGGCAAATTTGCTGGCAGTTCTCACAGAGACACTGGACAGTATCCCTGTGGAT
GAGGATGGATTGCCTTCATTTGATGCACTGACAGATGGAGAAGTGACCAATGAAAATGAT
ACTAGCCCTTCACCAATGCCTGACGGCACCCCTCCAGCTCAGGAGGCAGAAGAGCCGTCT
CTACTTAAGAAGCTCTTGCTGGCTCCAGCCAACACTCAGCTAAATTACAATGAATGCAGT
CGCCTCAGCACACAAAACCATGCAAACACTAATCACAGGATCAGAACAAGCCCTGTGGTT
GTTAAGACCGAGAATTCATGGAGCAATAAAGCAAAGAGCATTTGTCAACAGCAGAAGCCA
CAAAGACGTCCCTGCTCTGAGCTTCTCAAGTATCTGACTACAAATGATGACCCTCCTCAG
ACCAAACTGACAGAGAACAGGAACAGCAGCAAAGACAAATGCAACTCCAAAAAGAAGCCC
CAACTGCACTCTCAGGCACATCATTTGCAAGCCAAACCAACAAGTTTATCACTTCCTTTG
ACACCTGAGTCACCAAATGATCCCAAGGGTTCCCCATTTGAGAACAAGACTATTGAACAA
ACCTTAAGTGTGGAACTCTCTGGAACTGCAGGCCTAACTCCACCTACGACCCCTCCTCAC
AAAGCCAACCAAGATAATCCTTTTAGGACTTCACCTAAGCCGAAGTCATCATGCAAGACT
ATTGTACCACCTCCAAAAAAGCCTCGCTATAGTGAGTCTTCCAGTTCTCAAGGAAATAAC
CCAATCAAGAAGGGTGCAGATCAGTCTGAGCTGTATGCACAGCTCAGCAAGACAACAGTA
CTGTCCAGTGGACATGAGGAGAGAAAGACAAAACGGCCTAGTTTGCGGCTGTTTGGTGAC
CACGACTATTGTCAGTCTGTGAATTCAAAATCAGAAATACATATTAAAATATCCCAGGAA
CTTCAGGAGTCCAGACAACTAGAATTTAAGGATTCTTCACCTGCATGGCAGTGTCAGATT
TGTTCTTCTTTAGAACAAGACCAGTATTACAAGAAAGAGACTTTACAGACAAATAAGCAG
GGTTCCCACTGCAATAATAGAAAGCAGCTCCAAGACCAGGAAATCCGGGCTGAACTGAAT
AAGCACTTTGGTCACCCCAGCCAAGCTGTTTTTGAAGAAGAGGCAGATAAGACCAGTGAA
CTGAGGGACAGTGATTATAGTAATGAACAGTTCTCCAAACTACCTATGTTTATAAATTCA
GGACTAGCAATGGATGGTCTCTTTGATGATAGTGAAGATGAAAGTGATAAACTATGCTAC
CCTTGGGATGGGACTCAAACCTATTCTTTGTTTGATGTATCGCCTTCTTGCTCTTCTTTT
AACTCTCCATGCAGAGATTCAGTGTCTCCATCCAAATCTTTATTTTCTCAAAGATCCCAA
AGGATACGCTCTAGATCAAGGTCCTTTCCTCAACGCAGGTCTTGTTCCCGTTCTCCATAT
TCCCGATCGAGATCAAGATCGCCATGTAGCAGATCCTCTTCAAGATCTTGTTACTATTAT
GAGTCCAGCCACTGTAGACACCGAGCATACAAAAGTTCTCCCTTACATGCAAGGTCACGA
TCCAGATCACCCGGTAATCGTAGACCCAGATATGACAGCTATGAGGAATATCAGCATGAA
AGGCTGAAGAGGGAAGAATACCGCAAAGAGTATGAAAAACGGGAATCGGAAAGGGCCAAA

CAAAGGGAGAGGCAGAGGCAGAAAGCTATTGAAGAACGTCGTGTGATATATGTGGGTAAA
ATCAGACCTGACACAACTCGAACAGAACTGAGGGACCGCTTTGAAGTTTTTGGTGAAATT
GAGGAGTGCACAGTAAATCTACGGGATGATGGAGACAGCTATGGTTTCATCACCTACCGC
TATACTTGTGATGCTTTTGCTGCTCTTGAAAATGGATACACTTTACGCAGGTCAAATGAA
CCTGACTTTGAGCTGTACTTTTGTGGACGCAAGCAATTTTGCAAGTGTAACTATGCAGAC
CTAGATTCAAACTCAGATGATTTTGATCCTGCTTCCACTAAAAGCAAGTATGACTCCATG
GATTTTGATAGTTTACTGAAGGAGGCACAGCGAAGCCTGCGTAGG
>Armadillo_PPARGC1A
ATGATTATGACCATCCAAAATAGCCTTGGTGATAAGCAGCCAGCTGGGGTAGAGTGTGCT
GCTCTGGTTGGTGAAGACCAGCCTCTTTGCCCAGATCTTCCTGAACTTGACCTTTCTGAA
CTAGACGTGAATGACTTGGATACAGACAGCTTTCTGGGTGGACTAAAGTGGTGCAGTGAC
CAATCAGAAATAATTTCCAATCAGTACAACAATGAGCCATCAAATATATTTGAGAAGATA
GATGAAGAGAACGAGGCCAACTTGCTAGCAGTCCTCACTGAGACACTGGACAGTCTCCCT
GTGGATGAAGACGGATTGCCCTCATTTGATGCGCTGACAGATGGAGATGTGACCACTGAC
AATGATGCTAGTCCTTCCTCCATGCCTGACGGCACCCCTCCGCCTCAGGAGGCAGAAGAG
CCATCTCTACTTAAGAAGCTCTTACTGGCACCGGCCAACACTCAGCTAAGTTATAATGAA
TGCAGTGGTCTCAGTATCCAGAACCATGCAAATCATAATCACAGGATCAGAACAAGCCCT
GCAGTTGTTAAGACTGAGAATTCATGGAGCAATAAAGCGAAGAGCATTTGTCAACAGCAA
AAGCCACAAAGACGTCCCTGCTCAGAGCTTCTCAAGTATCTGACCACAAACGATGACCCT
CCTCACACCAAACCCACAGAGAACAGGAACAGCAGCAGGGATAAATGCACCTCCAAAAAG
AAACCCCCCACACAATCTCAGTCACAGCATTTGCAAGCCAAACCAACAACTTTATCTCTT
CCTCTGACCCCAGAGTCACCAAATGACCCCAAGGGTTCCCCATTTGAGAACAAGACTATT
GAACGAACCTTAAGTGTGGAACTCTCTGGAACTGCAGGCCTAACTCCACCCACAACTCCT
CCTCACAAAGCCAACCAAGATAACCCTTTTAGGGCTTCTCCAAAGCTGAAGTCCTCTTGC
AAGACTGTGGTACCACCACCATCAAAGAAGCCCCGGTACAGTGAGCCTTCTGGTACCCAC
GGAAATAACTCCACCAAGAAAGGGCCAGAGCAATCTGAGTTGTATGCACAACTCAGCAAG
ACCTCAGGACTCATCAGTGGACATGAGGAAAGGAAGACTAAGCGGCCCACTCTGCGGCTG
TTTGGTGACCATGACTATTGTCAGTCAATTAATTCCAAAACAGAGATACTCATTAATATA
TCACAGGAGCTCCAAGACTCTAGACAACTAGAATATAAAGATGCCTCCTCCGATTGTCAG
GGGCAGATTTGTTCTTCCACAGATTCAGACCAGTGCTACCTGAGAGAGACTTCGGAGGCA
AGCAAGCAGGTCTCTCCTTGCAGCACCAGGAAACAGCTCCAAGACCAGGAAATTCGAGCC
GAGCTGAACAAGCACTTTGGTCATCCCAGCCAAGCTGTTTTTGACGACGAAGCAGACAAG
ACCAGTGAACTGAGGGACAGTGATTTCAGTAATGAACAGTTCTCCAAACTACCTATGTTT
ATAAATTCAGGACTAGCCATGGATGGCCTGTTTGATGACAGCGAAGATGAAGGTGATAAA
CTGAGTTACCCTTGGGATGGCACGCAGTCCTATTCATTGTTCGATGTGTCGCCTTCTTGC
TCTTCTTTTAACTCTCCATGTAGAGATTCCGTGTCGCCACCCAAATCCTTATTTTCTCAG
AGACCCCAAAGGATGCGCTCTCGTTCAAGGTCCTTTTCTCGACACAGGTCATGTTCCCGA
TCATCGTATTCCAGGTCAAGATCAAGGTCTCCAGGCAGTAGATCCTCTTCAAGATCTTGC
TACTACTATGAGGCTAGCCACTGCAGGCACCGCATGCACCGAAATTCACCCTTGTGTGCG
AGATCAGGCTCCAGGTCACCCTATAGCCGTAGACCCAGGTATGACAGCTACGAGGAATAT
CAGCACGAAAGACTGAAGAGGGAAGAATACCGCAAGGAGTATGAGAAGCGGGAGTCTGAG
AGGGCCAAGCAAAGGGAGAGGCAGAGGCAGAAGGCGATTGAAGAACGCCGTGTGATTTAT
GTTGGTAAAATCAGACCTGACACAACACGGACAGAACTGAGGGACCGTTTTGAAGTTTTT
GGAGAAATTGAGGAGTGCACAGTAAATCTGCGGGATGATGGAGACAGCTATGGTTTCATC

ACCTACCGTTATACCTGCGATGCTTTTGCTGCTCTTGAAAATGGATACACTCTGCGCAGG
TCGAATGAAACTGACTTCGAGCTGTACTTTTGTGGACGCAAGCAATTTTTCAAGTCTAAC
TATGCAGACCTAGATTCAAACTCAGATGACTTTGACCCTGCTTCCACCAAGAGCAAGTAT
GACTCTCTGGATTTCGATAGTTTACTGAAAGAAGCTCAGAGAAGCTTGCGCAGG
>American_alligator_SOD3
ATGTTTCTATCCCTTTGTCTGGTATCCGTGGGGCTGTTCCTATCCTTACCTGGTGTTGTA
AGCGGAGAATTGGAGCCTGTTCCTGAAACAGAAGGCCTGTTTCAAGAATTACGGAAAAAA
GTGAATGACTTGTGGCAGAATTTGCTCTACCCACAATTTATCAGAGACAGTGACTGGACG
GCTTACGCTACTTGTGAAATGAAGCCCAGCTCCAAATTAGATGCTGACAAACCACAAGTG
ACAGGACAAATCTTGTTCAAACAGTCCTACCCAATTGGAAAGCTAGAAGCTTTCTTTGAC
CTGGATGGGTTTCCATCAGAGAACAATCAGTTGGGTAGGGCTATTCATATCCATAAGTTT
GGAGACCTCAGCGACGGCTGTAACACTACTGGAGGACACTATAACCCCTTCAATGTGAAC
CATCCTCACCATCCAGGGGATTTTGGCAACTTCTACTCTAAAGAAGGCAAAATTAGAAAA
TACAAGTCAAACCTGTCTGCTACTCTGTTTGGCCCATATACCATTATGGGCAGATCTGTC
GTTATTCATGAGCAGGAAGATGACATGGGCAAAGGTAACAATAAGGCCAGTTTGGAGAAT
GGAAATGCGGGCAGACGTCTGGCTTGCTGTGTCATTGGGATATGCGACAAGAACATGTGG
GAGAATAAATTCTCCAAGTTTGTAGAAAGGAAGAAAAGAGGCTCACAAAATGAGCACAGA
AGCAGGCCCAAC
>Armadillo_SOD3
ATGCTGGCACTGCTGTGTTCCTGCCTGCTCCTGGTGGCCCGCGCTTCCTGCGCCTCCACC
GGCTGGGACCCGGAGGAGCCCAGCTCCAGCACGGAGGAGCAGATCCGCGACATGCACGCG
AAGGTGAGGGAGATCTGGCAGGAGCTGCTGCAGCGGCAGGCGGCGGACGGCGGGGCGGAC
TCGGCGCTCTACGCCGCCTGCCAGGTGCAGCCGTCGGCCGCGCTGGACGCCGCGCAGCCC
CGGGTCTCCGGCCTCGTCCTCTTCCGGCAGCCGGCGCCGGGGGCCCAGCTCGAGGCCTTC
TTCGACCTGGAGGGCTTCCCAGCCGAGGCGAACGGCTCCAGCCGCGCGGTCCACGTGCAC
CGCTTCGGGGACCTGAGCCAGGGCTGCGAGTCCACCGGGCCGCACTACAACCCGCTGGGC
GTGCCGCACCCGCAGCACCCGGGCGACTTCGGCAACTTCGCCGTCCGCGCCGGCCGCGTC
TGGAAGTACCGCGCTGGCCTGGCCGCCTCGCTCGCGGGCCCGCACTCCATCGTGGGCCGC
GCCGTGGTGGTCCACGCGGGCGAGGACGAC
>Chinese_softshell_turtle_SOD3
ATGCTTCTGTTCCTTTACCTGGCCATTATCCTGGGCCTCTCTGCATCTGGTATTGTGAGA
GGAGACGAGGAGGCTGATTCCCACAGAGACAGCCCACTTCAAGACATACGGAAAAAAGTG
AATGATCTGTGGCAGAATTTACTCTACCCACAGTTAATCAGTAAGGAGACTGAGTGGACA
GGTTATGCCACTTGTGAAGTGAAGCCCAGCTCCAACCTAGATGCTAACAAGCCACAGGTG
ACAGGCCAAGTTTTATTCAGGCAGTCCTATCCAAATGGAAAACTAGAGGTTATCTTTGAC
TTGGATGGGTTTCCATTACACAGCAACCAGTCTGGCAGAGCTATTCACATCCACGAGCTT
GGAGACCTCAGCAAAGGCTGTGACTCTGCTGCAGGGCATTATAATCCTTTCAAGGTGAAC
CACCCTCACCACCCAGGGGATTTTGGCAACTTCTTGTCTAAAGGCGGCAAAATTAGAAAA
TACAAGGGAAATCTGCTTGCCACACTGTTTGGCCCATATTCTATCATTGGGAGATCGATT
GTGATCCACGAGCAGGAAGATGATATGGGCAAGGGTGGTAATAAAGCCAGTTTGGAGAAT
GGGAATGCTGGCAGACGTCTAGCTTGCTGTGTCATTGGGATATGCAACAAGAACTCATGG
GAGAAAAATCTTCCTGACATTACAGAGAGGAGGAAGAGGAGGATAACAAATGAGCACAAA
ACAACCAGGCCTAAC
>Cow_SOD3

ATGCTGGCGCTGCTCTGTGCCTCTGTGGTCCTGGTGGCCTATGCCTCGGCCGACCAGATC
CAGCAGCAGATGGGCTCCAACACGGAGGAGCAGATCCGCGACATGCACGCCAAGGTGACG
GAGATCTGGCAGGAGATGATGCAGCGGCAGGCGGCGGCCATCGACCCGGACGCGGCGCTC
CATGCCGTCTGCCGGGTGCTGCCGTCGGCCACGCTGGAGGCGGAGCAGCCCCGGGTCAGC
GGCCTCGTGCTCTTCCGGCAGCTCCGGCCTGGCGCCCTGCTGGAGGCCTTCTTCCACCTT
GAGGGCTTCCCGAACGAGCCCAACGGCACAAACCGTGCCATCCACGTGCACCAGTTTGGG
GACCTGAGCCAGGGCTGCGACTCCACCGGGCCGCACTACAACCCGATGTCCGTGCCGCAC
CCGCAGCACCCGGGCGACTTCGGCAACTTCGCCGTGCGCGATGGCCAGGTCTGGAAGTAC
CGCTCCGGCCTGGCTGCCTCGCTCACCGGCCCGCATTCGATCGCGGGCCGTGCCGTGGTG
GTCCACGCGGGCGAGGACGACATGGGCCGCGGCGGCAATCAGGCCAGTCTGGAGAACGGT
AACGCCGGCCGCCGGCTTGCCTGCTGCGTGGTGGGTCTGTGTGGCCCCGGGCCCTGGGCA
CGCCAAACGCAGGAGCACGCGGAGCGCAAGAAGCGGCGGCGCGAGAGCGAGTGTAAAGCC
GTC
>Dog_SOD3
ATGGCCTGCCTTCCCTGCAGTGACAGTGACCGTGACCCGCTCCCGGGGGCTGGCTTGCCC
GCACGCCCACAAACAGCCCAGCCTAAGGAGTCAGCCCGCATTCCTGGCTGCTGCTCCCTA
CTGCTCAGAAGGAAAGCTCTCTCGGAAGAGCTGGGAAGGTGCCCGCCCCCGGCCATGCTG
GCGCCCGCGCTGCTCTGCGCCCACCTGCTGCTGGCGGCCCCCGCCTCGCGCGCCTGGCCC
GGCGCCGCCCCGGACGAGCCCGAGCCCCAGCCCCAGCCCCAGCCCGAGCCCGGCCCCGGC
GCGGTGGCGGCGCAGCTCAGCGACATGCACGACAAAGTGACGGCCATCTGGCAGCAGCTG
ACGCAGCGCGCGGCCGAGCCGGGGGCCCCGGGCTCCGCGCTGCACGCCGCCTGCCGGGTG
CAGCCGTCCGCCTCGCTGGACCCCGCGCAGCCGCGCGTGAGCGGCCTCGTGCTGTTCCGC
CAGCCCGCGCCCGGCGCCCCGCTCGAGGCCTTCTTCCACCTGGACGGCTTCCCGGCCGAG
CGCAACGGCTCCCGCCGCGCCATCCACGTGCACCGCTTCGGCGACCTGAGCCCGGGCTGC
GAGGCCGCGGGGCCGCACTACAACCCGCGCGCCGCGCCGCACCCGCACCACCCCGGCGAC
TTCGGCAACTTCGCGGTGCGCGACGGCCGCGTCCGGAAGCACCGCGGCGGCCTGGCCGCC
TCCCTCGCCGGCCCGCACTCCATCGTGGGCCGCGCCGTCGTGGTGCACGCGGGCGAGGAC
GACCTGGGCCGCGGCGGCGACCCCGCCAGCCTGGACCACGGCAACGCGGGCCGCCGCCTG
GCCTGCTGCGTCGTCGGCGTGTGCGGGCCGCTGCCCTGGGCGCGCGCGGCGCGGGAGCAC
GCGGAGCGCAGGAAGCGGCGGCGGGACAGCGACTGCCAGGCGGCC
>Elephant_SOD3
ATGCTGGCGCTGCTGTGTTCCTTCCTGCTCCTGGCGGCCCGCGCCTCTACCGATATGGAT
ACCGACACGGCGGAGCCCGGTTCCAACACGGCAGAGCAAATCCGCGACATGCACGTCAAG
GTGACGGAGATTTGGCAGGCGCTCACGCACCGGGGGGCGAGGGACGGCGGGCCGGGCGCC
GACCTATACACCGCATGCAGCGTGCAGCCATCCGCCTCGATAGGCGCTGCGCAGCCGCGG
GTCACTGGCCTCGTCCTCTTCCGGCAGCTGGCGCCCGGCGCCAAGCTCGAGGCCTTCCTG
GACCTGGAAGGCTTCACCGCAGAGCACAACGGCTCCGCCCGCGCCATCCACGTGCACCAG
TTCGGGGACGTGAGCCAGGGCTGCGAGGCCACTGGGCCCCACTACAACCCGCTGGGCGTG
CCGCACCCGCAGCACCCGGGCGACTTCGGCAACTTCTGGGTCCGCGACGGTAGCCTCTGG
AAGTACCGCACGGGCCTCAACGCCTCGCTCGTCGGCCCCCATTCGATCGTGGGCCGCGCA
GTCGTGGTGCACGCGGGCCAGGATGACCTGGGCCGCGGCGGCGACCAGGCCAGCGTGGAG
AACGGCAACGCGGGCCCCCGGCTGGCCTGCTGCGTGGTGGGCGTGTGCGGGCCTGAGCCG
TGGGCGCGCCAGGCTCAGAAACATGCTGAGCGCAAGAAGCGGTGGCGCGAGAACGAGTGC
AAGGCTGCC

>tropical_clawed_frog_SOD3
ATGAACAATCTGTTGTACTTGGCCGTGGCTCTCACCGTTTGTGAACTGCTCAGCGCTGGA
GCAGAAGTGGTGAAACCGGTGGAAGAAGAACTTCTGACAGACACAAACAAAAAAGTCAAC
GAGCTGTGGATAAACCTTCTCAATATGAAGCCGACGGATAATGACGGGATTGCCTACGCA
ACCTGTAGTCTAAGTCCTAGTTCCAAACTGGAACCATCAGAGGTAAAAGTGACAGGACTT
GTGCTGTTTAAGCAGGTTTTTCCTAGCGGCACGCTCGAAGCCATTTTTGATTTAGAAGGG
TTTCCAACTGATGCCAATCAGTCAGCAAGAGCTATTCACATCCACACATACGGCGATCTA
ACCAATGGATGCGACTCTGCCGGTGGCCACTATAACCCTATGTCTGTAGACCACCCACAG
CATCCTGGTGATTTTGGCAATTTCCGTGTCAGGGATGGAAAGATCCAAAAATTTTTTGCA
AATCTTGATGCCACTCTCTTTGGTCCATTCTCTGTCATCGGCAGATCAGTTGTGGTCCAC
AAACAAGCAGATGATCTTGGCAAAGGGAACAATCAGGCCAGTTTGGAGAATGGAAATGCA
GGGAAACGCCTGGCTTGTTGCATTATTGGATCCAGCAGTAAAAATAACTGGGAAAAGTAT
GCCCAAGACAGTGCAGCACCAAGGAACCTCAGGTTTTCCAGACGGGTAAAAAATGGT

>Fruitfly_SOD3
ATGATGCAATATCTTGTTGTTAGCCTGGCACTCTGTGCCACAATTTGCTCTGCTGCGCAG
ACGCGCAATATGCCCATTCAAGCCATTGCCTATCTGATTGGACCCGTGCAATCGGATAAT
ACCCAGGTCAAGGGCAACGTGACCTTTACGCAGAACGACTGTGGCCAGAATGTCCATGTG
CGCGTCCAGCTGGAGGGATTGAAGGAGGGCAAGCACGGCTTCCACATTCACGAGAAGGGA
GATCTGACCAATGGATGCATCAGCATGGGTGCTCACTATAACCCCGATAAGGTTGATCAC
GGTGGCCCCGATCACGAGGTGCGTCATGTTGGCGATCTGGGCAACCTGGAGGCCAACTCC
ACGGGCATTATCGACGTTACATACACGGATCAGGTGATCACCTTAACTGGCAAGCTGGGG
ATCATTGGCAGGGGAGTTGTTGTCCACGAATTGGAGGATGATCTCGGTCTGGGCAACCAC
ACGGATTCCAAGAAGACCGGCAATGCAGGCGGCCGCATTGCCTGTGGTGTTATTGGCATC
AATGGCCCATCTGTACCTGCACCTGCACCACCAACACCACGCCATCAGCCACTGTACTAT
GTGCCCCATGGGGAGCCACAAGATCATGTTGTCTATCCACTCCAGCACTATCCTTTGCCA
TACCCACACCCATATCCATATCCGCACCCATACCCCTATCCGTATCCGTTGTCGCACCAA
TTGTATCTG
>Anole_lizard_SOD3
ATGTTTCTGCTGCTGTTTCTCATCCCTGGCTTGATTCTTTGTGAATGCAACGTCTCCACA
GGAGAAGATGTCCAGCCTACCGCAGTGAGCCCACTTCAAGACATTCAGAAAAAAGTCAAC
GAGCTTTGGTCGAGTTTGCTCTACCCGCACCTCTATGAGGAGGTACTTGCATCAAACGGG
ACTGCTTATGCTGTTTGTACAGTGAAGCCCAGCACCAAACTGGATGCAGGAATGCCGCAA
GTGACAGGGCAAGTTCTGTTCAGGCAAACCTACCCCTATGGAAAGCTGGAGAGTATATTC
TATTTGGATGGGTTTCCTACAGGTGCCAACTTGTCTGGGAGAGCAATTCACATCCACCAG
TATGGAGACCTCAGCGACAGCTGCGACTCAGCCGGGGGACACTACAATCCTTTTAAAGTC
AATCACCCTTCTCATCCTGGGGACTTTGGCAACTTCTACTCTAAAGAAGGCAAAATTATA
AAATATAAGGCCAACCTCGTGGCCACTCTTTTCGGTCCGTACACCATTATGGGAAGGTCT
GTTGTGGTCCATGTACAGGAAGATGACCTGGGGAAAGGGAACAACAAAGCCAGCTTGGAG
AACGGAAATGCCGGCAAACGCTTAGCTTGCTGTGTTATTGGAACATGCAACAAAAACCAG
TGGGTGAAGCGGTTTTCTGAGATTATGGAGAAGAGGAAGAAAAGGCTCACGAGACGTGCA
CAGAACAGCCCAGCC
>Human_SOD3
ATGCTGGCGCTACTGTGTTCCTGCCTGCTCCTGGCAGCCGGTGCCTCGGACGCCTGGACG

GGCGAGGACTCGGCGGAGCCCAACTCTGACTCGGCGGAGTGGATCCGAGACATGTACGCC
AAGGTCACGGAGATCTGGCAGGAGGTCATGCAGCGGCGGGACGACGACGGCGCGCTCCAC
GCCGCCTGCCAGGTGCAGCCGTCGGCCACGCTGGACGCCGCGCAGCCCCGGGTGACCGGC
GTCGTCCTCTTCCGGCAGCTTGCGCCCCGCGCCAAGCTCGACGCCTTCTTCGCCCTGGAG
GGCTTCCCGACCGAGCCGAACAGCTCCAGCCGCGCCATCCACGTGCACCAGTTCGGGGAC
CTGAGCCAGGGCTGCGAGTCCACCGGGCCCCACTACAACCCGCTGGCCGTGCCGCACCCG
CAGCACCCGGGCGACTTCGGCAACTTCGCGGTCCGCGACGGCAGCCTCTGGAGGTACCGC
GCCGGCCTGGCCGCCTCGCTCGCGGGCCCGCACTCCATCGTGGGCCGGGCCGTGGTCGTC
CACGCTGGCGAGGACGACCTGGGCCGCGGCGGCAACCAGGCCAGCGTGGAGAACGGGAAC
GCGGGCCGGCGGCTGGCCTGCTGCGTGGTGGGCGTGTGCGGGCCCGGGCTCTGGGAGCGC
CAGGCGCGGGAGCACTCAGAGCGCAAGAAGCGGCGGCGCGAGAGCGAGTGCAAGGCCGCC
>Macaque_SOD3
ATGCTGGCGCTGCTGTGTTCCTGCCTGCTTCTGGCAGCCGGTGCGTCGGACGCCTGGACG
GGCAAGGACTCAGCGGAGCCCAACTCTGACTTGGCAGAGTCGATCCGAGACATGCACGCC
AAGATCACGGAGATCTGGCAGGAGCTCACGCAGCGGCGGGACGGCGACGGCGCACTCCAC
GCCGCCTGCCAGGTGCAGCCGTCGGCCACGCTGGACGCCGCGCAGCCCCGGGTGACCGGA
GTCGTCCTCTTCCGGCAGCTCGCGCCCCGCGCCAAGCTCGAGGCCTTCTTCGCCCTGGAG
GGCTTCCCGACTCAGCCGAACAACTCCAGCCGCGCCATCCACGTGCACCAGTTCGGGGAC
CTGAGCCAGGGCTGCGAGTCCACCGGGCCCCACTACAACCCGCTGGCCGTGCCGCACCCG
CGGCACCCGGGCGACTTCGGCAACTTCGCGGTGCGCGACGGCAGCCTCTGGAAGTACCGC
GCCGGCCTGGCCGCCTCACTCGCGGGCCCGCACTCAATCGTGGGCCGGGCCGTGGTCGTC
CACGCTGGCGAGGACGACCTGGGCCGCGGCGGCAACCAGGCCAGCGTGGAGAACGGGAAC
GCGGGCCGGCGGCTGGCCTGCTGCGTGGTGGGCGTGTGCGGGCCCGGGCTCTGGGAGCGC
CAGGCGCGGGAGCACTCAGAGCGCAAGAAGCGGCGGCGCGAAAGCGAGTGCAAGGCCGTC
>Mouse_SOD3
ATGTTGGCCTTCTTGTTCTACGGCTTGCTACTGGCGGCCTGTGGCTCTGTCACCATGTCA
AATCCAGGGGAGTCCAGCTTCGACCTAGCAGACAGGCTTGACCCGGTTGAGAAGATAGAC
AGGCTTGACCTGGTTGAGAAGATAGGCGACACGCATGCCAAAGTGCTGGAGATCTGGATG
GAGCTAGGACGACGAAGGGAGGTGGATGCTGCCGAGATGCATGCAATCTGCAGGGTACAA
CCATCAGCCACGCTGCCACCGGATCAGCCGCAGATCACCGGCTTGGTTCTCTTCCGGCAG
CTGGGGCCGGGCTCCAGGCTTGAGGCCTATTTCAGTCTGGAGGGCTTCCCAGCTGAGCAG
AACGCCTCCAACCGTGCCATCCACGTGCATGAGTTCGGGGACCTGAGCCAGGGCTGCGAT
TCCACCGGGCCGCACTACAACCCGATGGAGGTGCCGCACCCTCAGCACCCGGGCGACTTT
GGCAACTTCGTGGTGCGCAACGGCCAGCTCTGGAGGCATCGCGTCGGCCTGACCGCGTCG
CTGGCCGGACCGCACGCCATCTTGGGCCGCTCTGTGGTGGTCCACGCCGGCGAGGACGAC
CTGGGTAAAGGTGGCAACCAGGCCAGCCTGCAGAACGGCAATGCAGGTCGCCGGCTCGCC
TGCTGCGTGGTAGGCACCAGCAGCTCCGCCGCCTGGGAGAGCCAGACAAAGGAGCGCAAG
AAGCGGCGGCGGGAGAGCGAGTGCAAGACCACT
>Naked_mole_rat_SOD3
ATGCTGGCTTTGCTGTGTGGCTGCCTGCTTCTGGCAGCCCCAGCCGTGCACACCCTGAGC
GTCACGGACCTGGCGAAGCCCAGCCCCGAGCTGATGGAGCAGATCCACGACATGCACACC
AAGGTGACAGACATCTGGCAGGAGCTGACGCAGTGGCGGGAGGCCGCCGGCGGGCCAGAC
GTGGGGCTCCACGCAGTGTGCCACATGCAGCCATCCGCCACTCTGGACGCCGAGCAGCCC
CGCGTGAGCGGCCTGGTCCTCTTCCGGCAGCCCGGGCCCGGGGCCAGGCTCGAAGCCTTC

TTCGACCTGGAGGGCTTCCCCGAGGAGGCCAACAGCTCCAAACGCGCCATCCACGTGCAC
CGGTTCGGCGACCTGAGCCAGGGCTGCGACTCCACCGGGCCCCACTACAACCCGCTGTCC
GTGCCGCACCCGCAGCACCCTGGCGACTTCGGCAACTTCGTCGTGCGCGACGGCCGCCTC
TGGAAGTACCAGGCCGGCCTCCCCGCCTCGCTGGCCGGCCCTCACTCCATCGTGGGCCGC
GCCGTGGTGGTCCACGCCGGCGAGGACGACCTCGGCCGCGGCGACACCCCGGCCAGCGTG
GAGAACGGCAACGCGGGGCCCAGGCTCGCCTGCTGCGTGGTGGGCGCGGGGGGCCCCGAG
GCCTGGGCGCGTCAGGCGCGGGAGCACGCGGAGCGCAAGAAGAGGCGGCGCGACAGCGGG
CGCAAGACAGCA
>Orangutan_SOD3
ATGCTGGCGCTACTGTGTTCCTGCCTGCTCCTGGCAGCCGGTGCCTCGGACGCCTGGACG
GGCGAGGACTCGGTGGAGCCCAACTCTGACTCGGCGGAGTGGATCCAAGACATGTACGCC
AAGGTCACGGAGATCTGGCAGGAGGTCATGCAGCGGCGGGACGACGACGGCGCGCTCCAC
GCCGCCTGCCAGGTACAGCCGTCGGCCACGCTGGACGCCACGCAGCCCCGGGTGACCGGC
GTCGTCCTCTTCCGGCAGCTCGCGCCCCGCGCCAAGCTCGACGCCTTCTTCGCCCTGGAG
GGCTTCCCGACCGAGCCGAACAGCTCCAGCCGCGCCATCCACGTGCACCAGTTCGGGGAC
CTGAGCCAGGGCTGCGAGTCCACCGGGCCCCACTACAACCCGCTGGCCGTGCCGCACCCG
CGGCACCCGGGAGACTTCGGCAACTTCGCGGTGCGCGACGGCAGCCTCTGGAAGTACCGC
GCCGGCCTGGCCGCCTCGCTCGCGGGCCCGCACTCCATCGTGGGCCGGGCCGTGGTCGTC
CACGCTGGCGAGGACGACCTGGGCCGCGGCGGCAACCAGGCCAGCGTGGAGAACGGGAAC
GCGGGCCGGCGGCTGGCCTGCTGCGTGGTGGGCGTGTGCGGGCCCGGGCTCTGGGAGCGC
CAGGCGCGGGAGCACTCAGAGCGCAAGAAGCGGCGGCGCGAGAGCGAGTGCAAGGCCGCC
>Western_painted_turtle_SOD3
ATGTTTCTGTTCCTTTACCTGGTCATCAACCTGGGCCTCTCTGCATCTGGTGTTGTGAGG
GGAGAAGGTGAGGCTGATTCCAATGGAGACAGCCCACTTCAAGACATACAGAAAAAAGTG
AATGACCTGTGGCAGAATTTACTCTACCCACAGTTCATCAATGAGACGACTAACCGGATC
GGTTATGCCACTTGTGAAGTGAAGCCCAGCTCCAATCTAGATGCTGACAAGCCACAGGTG
ACAGGCCAAGTCTTGTTCAGACAGTCCTACCCAAATGGAAAACTAGAGGCTCTCTTTGAC
TTGAATGGGTTTCCATCAGGCAGCAATCAGTCTGGCAGAGCTATTCACATCCATAAGCTC
GGAGACCTCAGCAACAGCTGTGACTCTACTGGGGGGCACTATAACCCTTTCAACGTGAAC
CACCCTCGCCATCCAGGGGATTTTGGCAACTTCTACTCTAAAGAAGGCAAAATTAGAAAA
TACAAGCCAAATCTGCTCGCCACTCTGTTTGGCCCCTACTCCATCATGGGGAGATCCATT
GTGATCCATGAGCAGGAAGATGATATGGGCAAGGGTAACAATAAAGCCAGTTTGGAGAAT
GGAAATGCTGGCAGACGTCTAGCTTGCTGTGTCATTGGGACATGCAACAAGAACTTATGG
GAGCAAAAGTTTCCTGACATTACAGAGAGGAGGAAGAAGAGGATCACAAATGAGCACAAA
ACAACCAGGCCTAAC
>Panda_SOD3
ATGCTGGCCCCGGCGCTGCTGTGTGTCTACGTGCTGCTGGTGGCCCCCGCCTCGCGCGCCGGGCCCGGCC
CCAGCCCGGAGGAGCCCGGCTCCAGCACGGAGGCGCAGATCCGCGACATGCACGCGAAAGTGACGGCCA
T
CTGGCAGGAGCTGACGCAGCGACAGGCGGCGGCCGACGGTNNNNNNNNNNNNNNNNNNNNNNNNN
NNNNG
TCGGCCACGCTGGACACCGCGCAGCCCCGGGTCAGCGGCCTCGTGCTCTTCCGGCAGCTGGCGCCCGGCG
CCCGGCTCGAGGCCTTCTTCGACCTGGAGGGCTTCCCGGCCGAGGCCAACAGCTCCAGCCGCGCCATCCA
CGTGCACCAGTTCGGGGACCTGAGCCAGGGCTGCGAGTCCACCGGCGCGCACTACAACCCGCTGGCCGTG

CCGCACCCGCAGCAC
>Pig_SOD3
ATGCTGACGCTGCTCTGTGCTTACCTGCTCCTGGCGCCCGGCGCCTCCGACGCCTTGACC
CACGTGGACGTGGAGCGGCCAGGCTCCCACATGGAGGAGCAGATCCGGGACATGCAGGCC
AAAGTGACGGAGATCTGGCAGGAGTTGACGCAGCAGCGGGCGGCGGGCGGCGGCCAGGAG
GCCGCGCTCCACGCCACCTGCTCGATGCAGCCTTCGGTCTTGCTGGACGCGGCGCAGCCC
CAGGTGACCGGCCTTGTGCTCTTCCGGCAGCTCCGGCCCGGCGCCCTGCTCGAGGCCTTC
TTCCACCTGAAGGGCTTCCCGGCCGAGCCCAACAGCACCAGCCGCGCCATCCACGTGCAC
CAGTTCGGGGACCTGAGCCAGGGCTGCGACTCCACAGGGCCGCACTACAACCCGCTGGAT
GTGCCACATCCGCAGCACCCGGGAGACTTCGGCAACTTCGCCGTGCGTGACGGCCAGATC
TGGAGGTACCGCTCCAGTCCGGGCGCCTCGCTGTTCGGCCCGCACTCGATCGCGGACCGC
GCGGTGGTTGTCCACGTGGGCGAGGACGACATGGGCCGCGGGAACCAGGCCAGCCTGGAG
AACGGCAACGCCGGCCGCCGCCTGGCATGCTGCGTGGTGGCCCTGTGCGGCCCCGGGCCC
TGGGCGCCCCAGGCGCAGGAGCACGCCGATCACATGAAGCGGCGGCGCGCAAGCGAGTGC
AAGGCCAGC
>Platypus_SOD3
ATGTCCCCCACCCTGGGCCCCGCCCTCATCCTGGCCACCTGTGCCTTCTGGGCCGCCGCA
GGGCAGGGCGCGAACGGGGAGGACCCGGAAAACCAGGTGAACGCCATCTACCAGAAGGTG
ACGGACCTGTGGAACAAGCTGTACATGGTGAAGCCGGTGACGGCCAACGGCAGCGACGAG
GATCCGGTGTACGCGGCTTGCGAGGTCAAACCGTCCTCCATTCTGGAGGCCGACCAGCCG
CGGGTCACCGGCCGGGTGCTCTTCAGGCAACAGTACCCGGACGGAAGGCTGGAGGCCATC
TTCGACCTGAACGGCTTCCCCACCGTGGGCAACGGGACCGGCCGGGCCATCCACGTCCAC
AAGTTTGGGGATATGAGCAACGGTTGTGATGGGGCTTCTAGTCACTACAACCCCTTCTCC
AAACCACACCCTCAGCACCCGGGGGACTTCGGGAACTTCCTGCCTAGGGAAGGCAAGATC
AAGCGTTACCGGACCAACCTGCTGGCCTCGCTCTTCGGCCCATACTCCATCCTGGGCCGC
TCCATCGTGGTGCACGAGCGGGATGATGACCTCGGCAAGGGTGGGAACAAGGGCAGCCAG
GAGCACGGGAACGCCGGGAAGCGGCTGGCCTGTTGCGTCATCGGGATCTGCGGCCGGGAG
TCGTGGGATAAAAAGGCCCTCGAGATCAACGAGCGGAGGAAGAAGAGGCGGGAGAGCGAG
TGCAAGACGGCC
>Rabbit_SOD3
ATGCTGGCGTTGGTGTGCTCCTGCCTGCTCCTGGCGGCCCTCCCAGCGGACACCTGGAGC
GGCCCGGCCGCAGTGGAGCTCGGCTCAGACACGGTGGAGCAGATCCGTGACACGCACGCC
AAAGTCACGGAGATCTGGCAGGCGCTCACGCAGCAGCGGGCAGCGCAAGGCGAGCCGGCC
GGGGCGCTCCACGCCGTCTGCCGGGTGCAGCCGTCGGCCACGCTTGACGCCGCGCAGCCG
CGAGTCAGCGGCTTGGTAGTCTTCCGGCAGCTTGGGCCCGGTGCCCAGCTCGAAGCCTTC
TTCGACTTGGAGGGCTTCCCGGTCGAGGCCAACCTCTCCAGCCGCGCCATCCACGTGCAC
CAGTTCGGGGACCTGAGCCAGGGCTGCGACTCCACCGGCGCCCACTACAACCCGCTGGCC
GTGCAGCACCCGCAGCACCCGGGAGACTTCGGGAACTTCGCCGTGCGCGACGGCCGCCTC
TGGAAGTACCGCTCGGGCCTGGCCGCCTCGCTCGCAGGGCCGCACTCGATCGTGGGCCGC
GCCGTGGTGGTGCACGCCGGCGAGGACGACCTGGGCCGCGGCGGCAACGCGGCCAGCGTG
GAGAACGGCAACGCGGGCCCCAGGCTGGCCTGTTGCGTGGTGGGCGCCAGCGGCCCGCG
CCCTGGGCGCGCCAGGCGCAGGAGCACGCAGAGCGCAAGAAGCGACGGCGCGAAAGCGAG
TGCAAGGCCGCC
>Tetraodon_SOD3

ATGCGTCTGCACGGGTGGGTGATCGCGTCGGCAGTGCTGCTGCTTCTGCTGGCCGGTTGT
CAAGATTGCGGCTCAGCTCACGGTGACCCTGCAGCTCCGCCGGAGGCCTCTCAGAACAAT
GGCAGCCTGTATGCGGCCTGCAACATGAGACCCAGCGCCTTGCTGCCAGAGGACCTGCCC
AAAGTGCACGGTCACGTGCTGTTCAAGCAGGACCACCCTCAGGGAGGACTCTCGGCCCTC
CTTCAGCTTGGCGGCTTTCTCAGCGACGGCGAGCCCACGGCCGTCCACATCCATCAGTAC
GGGGACCTGAGCCAGGGGTGCGGCTCCACCGGTGGGCACTACAACCCACACGGCAAAAAC
CACCCCAACCACCCCGGAGACTTTGGTAACTTTGAGCCTCAGGAGGGGAAGGTCGACGCC
GCGGTAGAGTCAAACGCCACGCTCTTTGGAGCGACGTCTGTGATCGGAAGGGCAGTGGTG
GTCCACGAGAAGAGAGATGACCTGGGCCAGGGTGGAGACGCCGGGAGCCTCCTGCACGGA
AACGCAGGACGGAGGCTTGCCTGCTGCGTTATTGGAATTTCCTCTTCCGATCTGTGGAAC
ACCTCCAAGGAGTTTACAGAAAGGGGG
>Tilapia_SOD3
ATGCATCGATACGCGACAGTGGGTATACTGGAAGCTGCTTTGTTGGTTTTGCTGGCAGGC
TGTCAACAATGTGTCTTAGCATCCAGCGACACATTGGCTCCAGAGGTCTTGCAGAACAAC
GGCACCCTGTATGCAGTCTGCAAGGTGAGACCCAGCACCTCTCTCCCGGAGGACCTGCCC
AAAGTGTATGGCCATGTGCTGTTCAAGCAGGATCATCCTCAGGAGAAAGTCAAGGTCCTC
CTCCGGTTTAGTGGCTTCCCCAGGGATGGCAGTCCGGAGCCCAGAGCAGTGCACATCCAT
CAATATGGAGACCTGAGCCGGGGATGTGACTCCACCGGTGGCCACTACAATCCATATGGT
GTAAATCACCCTAACCACCCTGGAGACTTTGGAAACTTTGAGCCTCAGCAAGGGAAGATT
AATACAATGCTAGAATCTGATGCAACACTGTTTGGAAGCCTGTCTGTGTTTGGGAGAGCA
GTGGTAATTCATGAAAAGATAGACGACTTAGGGCGCGGTGGAGACGCTGGGAGCCTGCTG
CATGGAAACGCAGGCCGAAGGCTCGCATGCTGCATTATTGGGGTTTCGTCCCCCAATCTC
TGGAATATGAACTTAAAGCAGTACTCCAGGCAGCTGAGGAGG
>Worm_SOD3
ATGCTGCAATCTACTGCTCGCACTGCTTCAAAGCTTGTTCAACCGGTTGCGGGAGTTCTC
GCCGTCCGCTCCAAGCACACTCTCCCAGATCTCCCATTCGACTATGCAGATTTGGAACCT
GTAATCAGCCATGAAATCATGCAGCTTCATCATCAAAAGCATCATGCCACCTACGTGAAC
AATCTCAATCAGATCGAGGAGAAACTTCACGAGGCTGTTTCGAAAGGGAATCTAAAAGAA
GCAATTGCTCTCCAACCAGCGCTGAAATTCAATGGTGGTGGACACATCAATCATTCTATC
TTCTGGACCAACTTGGCTAAGGATGGTGGAGAACCTTCAAAGGAGCTGATGGACACTATT
AAGCGCGACTTCGGTTCCCTGGATAACTTGCAAAAACGTCTTTCTGACATCACTATTGCG
GTTCAAGGCTCTGGCTGGGGATGGTTGGGATATTGCAAGAAAGACAAAATCTTGAAGATC
GCCACCTGTGCAAACCAGGATCCTTTGGAAGGAATGGTCCCACTTTTTGGAATTGACGTT
TGGGAGCACGCCTACTACTTGCAGTACAAAAATGTCCGCCCAGACTATGTCCATGCTATT
TGGAAGATTGCCAACTGGAAGAATATCAGCGAGAGATTTGCCAATGCTCGACAA
>Zebrafish_SOD3
ATGATACGCTTCAACATTTTACCCCTTCTGGCATTCCTGCTCAGTTGCCATGTGCTCTTC
TGTGGATCAGGCTCAGGTCTGGCATATGCTTCAAACAGTGATTCCCTTCAGGCCGTATGC
AGAATGCAGCCTAACACTCGACTAGAGCCTGGCATGCCTCGTGTGTATGGTCACATTTTA
TTCAGACAGTCTGGCCCAAAGGAAAAGCTGAGCGTGACTTTCAGGCTCTATGGTCTCCCT
GCGGACAGTCAGCAGCCCAGAGCCATGCACATTCATGAGTATGGAGACTTAAGCAGGGGC
TGCGACTCTACAGGTGGGCATTACAATCCCCTCAACGTCAACCATCCACAACATCCAGGG
GACTTTGGCAACTTTGTGCCCGTTAACAAGAAGATTCGGCAGTCACTGGAATCCCCTGCA
ACTCTCTTTGGGAAACTTTCAATAGTCGGCCGGTCTGTTGTCATTCATGAGGGAAAGGAT

GATTTGGGCAGAGGTGGGAATGTGGGAAGCTTGCTGAATGGCAACGCTGGAGGGCGACTG
GCATGCTGTGTTATTGGACTGAGAAACCCCCAAAATTAA
>Horse_SOD3
ATGACCTTCCTCGCTCACAGTGACCACTCCTCTGGGCTGGCTTCCTGCAGGCCCACAAAC
AGCCCAGTCTCAGGAGCCAGCCTGCGTTCCTGCCTCCAGCTTGGGCGCTGCTCGCACTTC
CCAGGAAGGAAAGCTCTCTTGAAAGGGCTGGAAAGGTGCCTGACTCCCGCCATGTTGGCG
CTGCTGTGTGCCTTCCTGCTCCTGGCGGCCGGCGCCTCGGACCCTCGGGCGGAGCCCAGC
TCTAATACGACGGAGCAGATCCGCGACATGCACGCCAAAGTGACGGAGATCTGGCAGGAG
CTGCTGCAGCGGCGGCAGGGGGGCAGCGGCCGGGATGCTGCGCTCCACGCTGCCTGCCAC
GTGCAGCCGTCGGCCACGCTGGACGCCGCGCAGCCCCGAGTGACCGGCCTCGTGTCTTCC
GGCAGCTCGTGCCCGGCGCCAAGCTGGAGGCCTTCTTTGACCTGGAGGGCTTCCCGGCCG
AGCCAACGCTCCAGCCGCGCCATCCACGTGCAC
>Dolphin_SOD3
ATGCTGGCGCTGCTCTGTGCCTACCTGCTCCTGACATCCCGCACCTCGGAAGTCTCCGCC
GACCCGGACATGGAGCAGCCCGGCTCCCGCATTCAGGAGCAGATCCGCGACATACACGCC
AAAGTGACGGAGATCTGGCGGCAGCTGACGCAGTGGTGGGCGGCGGGCGGAAGCCAGGAC
GCCGCGCTCCACGCCACCTGCCCCGTGCTGCCGTCGGCCACGCTGGACGCGGCGCAGCCA
CGGGTGAGCGGCGTCGTGCTCTTCCGGCAGCTGCGGCCCGGCGCCTTGCTCGAGGCCTTC
TTCCACCTGGAGGGGTTCCCGGCCGAGCCCAACGGCACCAGCCGCGCCATCCACGTGCAC
CAGTTCGGGGACCTGAGCCAGGGCTGCGACTCCACCGGCACGCACTACAACCCGCTGGGC
GTGCCGCATCCGCAGCACCCGGGCGACTTCGGCAACTTCGTGGTGCGCGACGGCCAAATC
TGGAAGTACCGCTCCGGCCTGGCCGCCTCGCTCTTCGGCCCGCACTCGATCGCGGGCCGC
GCCGTAGTGATCCACGAGGGCGAGGATGACCTGGGCCGCGGTGGTAACCAGGCCAGCCTG
GAGAACGGCAACGCGGGCCGCCGGCTCGCCTGCTGCGTGGTGGGCCTGTGCGGGCCGGGG
CCCTGGGCGCGCCAGGCACAGGAGCACGCGGAGCGCAAGAAGCGGCGGCGAGAGAGCGAG
TGCAAGGCCAGC
>Opossum_SOD3
ATGCTTCCAGTCCTGAGTGCCACCATCATCCTGTCTGCCTTTGTGTCCTGTGCCACAGTG
GAACTAAATGGGGAGCAGATGAATGACATCCAGAAAAAAGTCAGTGACCTCTGGCATAGG
CTCATCTACCTGAAGCCAGTCACTTCAGGCAGTGATGATCATATTGTCTATGCCACCTGC
CAGGTACAGCCTTCGTCTACTTTGGAGGCCAATAAACCCCAGGTGACAGGGCAAGTTCTA
TTTAAACAGCTCTACCCAGATGGGAAGATGGAAGTCTTCTTTGATCTGCAAGGGTTTCCA
GCAGATACCAGGAATGCCACTGGCCGAGCTATCCACATTCACAACTTTGGAGATCTCAGC
AATGGCTGTGATAGCACCTCCAGCCACTATAACCCTCATGGGGTAGCCCACCCCCTCCAC
CCAGGGGACTTCGGGAACTTCCTTGTGAAGGATGGAAAGATCAGGAAGTCTCGATCCAAC
GTTTCTGCTTCCCTATTTGGGCCCCACTCTGTTCTTGGAAGGGCCATTGTAGTTCATGAG
CAAGAAGATGACCTTGGCAAAGGTGGAAACAAAGGCAGCTTGGAACATGGCAATGCTGGG
AAGCGCCTGGCCTGCTGTGTCATTGGCATCTGTGGACCAGAGCTCTGGAATAAGGCAGCA
ATGGACAATGCATCGAGGAAAAAGAAGAGGAGAGAAAGTGAATGCAAGACTGCC
>Chicken_SOD3
ATGCTTCTGCTTCTTTCTCTGGTCACTGGGCTGGCCCTGTCTGCCTCTGATGTCGTGACA
GAAACAGGAGCCGATCCAAGCAGCGCGTTACTTCATGACATACAGAGAAAAGTGAATGAC
CTCTGGCAGAGCTTGCTCTATCCAGTGATGGCTGATAATGAGACTGATGGGCTGACCTAC
GCCACCTGTGAAGTGAAGCCCAGCTCCAAAATAGATGCTGACAAGCCACAAGTGACTGGA

CAAGTCTTATTCAGACAGCATTACTCACAAGGAAAATTAGAAGCCATTTTTCACTTGGAT
GGGTTTCCTCTGGATAACAACCAGTCTGGCAGGGCCATACACATCCATGAGCTCGGAGAT
CTCAGCGACGGCTGCAACTCCGCGGGAGGACACTACAACCCCTTTGGAGTGAACCACCCC
CGCCACCCAGGGGACTTCGGCAACTTTTCTCCTAAAGATGGCAAGATCAGAAAATACAAA
CCCAACCTCTTCGCCACAATGTTTGGTCCGTACTCCATCCTGGGCAGATCCGTTGTGATC
CATGAGCAGGAAGATGACATGGGCAAGGGCAATAACAAGGCCAGTCTGGAAAATGGAAAT
GCTGGCAAACGTCTGGCGTGCTGCGTGATTGGGATCTGCAACAAGAACTTGTGGGAGGAG
AAACAGTCTGAGGCTATGGACAGGAAGAAAAGATGGCGCCGAACCAGGCTCAGC
>Turkey_SOD3
ATGCTTCTGCTTCTTTCTCTGGTCACTGGACTGGCCCTGTCTGCCGCTGGTGTCGTGACA
GAAACAGGAGCCGATCCAAGCTGTGCATCACTTCATGATGTACAGAGAAAAGTGAATGAC
CTCTGGCAGAGCTTGCTCTATCCAGTGATGGCTGATAATGAGACTGATGGGCTGACCTAC
GCCACCTGTGAAATGAAGCCCAGCTCCAAAATAGGTGCTGACAAGCCACAAGTGACTGGA
CAAGTCTTATTCAGACAGCATTACTCACAAGGAAGATTAGAAGCCATTTTTCACTTGGAT
GGGTTTCCACTGGATAACAACCAGTCTGGCAGAGCTATACACATCCACGAGCTCGGAGAT
CTCAGCAACGGCTGTGACTCCACGGGAGGACACTACAACCCCTTCAGAGTGAACCACCCC
CGCCACCCGGGGGACTTTGGCAACTTTTCTCCTAAAGATGGCAAAATCAGAAAATACAAA
TCCAATCTCTTCGCCACAATGTTTGGTCCGTACTCCATCCTGGGCAGAGCCATTGTGATC
CACGAGCAGGAAGATGACATGGGCAAGGGCAATAACAAGGCCAGTCTGGAAAATGGAAAT
GCTGGCAAACGTCTGGCGTGCTGCGTGATTGGGATCTGCAACAAGAACTTGTGGGAGGAG
AAACAGTTTGAGGCTACGGACAAGAAGAAGAGATGGCTCCGAACCAGGCTCAGC
>Duck_SOD3
ATGAAAACTTTTCCCTGTCCCTGCTGCCTCCAGCCCTCCCCTGTGCTGCGTTTCCAAGCT
CTTACACAAGCACACCCCAGCCTCCAGCAGCTCGGCGCTGAGGGCTGGCCGCACTCTGCT
TGGTGGGAGCAGAGGCGAGGAGCTGCCACCGAGGCATCCTGCGGTGATTGGGAGAGGCTC
AGCGTGGCCAGGATGCTTCTGCTGCTTTCTCTGCTCGCGGGACTCGCCCTGCCCGCCCCT
GGCATCGCGGCGGAAGAAGAAACCGAGCCAAGCAGAGAGACCCTTCAAGACATACAGAAA
AAAGTGAACGACCTCTGGCAGAATTTGCTCTACCCGCTAACGGCCGGTAACGAGACCGGC
GGGATGACTTACGCCGCTTGTGAGATGCAGCCCAGCCCCAAACTAGACGCTGAGAAGCCG
CAGGTGACCGGCCAGGTCCTGTTCCGGCAGCATTACCCGCAGGGAAGGCTGGAAGCCATT
TTTCACTTGGAGGGCTTTCCGCTGAACGACGACCAGCCCGGCAGAGCGATCCACATCCAC
GAGCTGGGGGACCTCAGCAGCGGCTGCGATTCCACCGGAGGCCACTACAACCCCTTCCGC
GTCAACCACCCCCGCCACCCGGGGGATTTCGGGAACTTCTCTCCCAAAGATGGCAAAATC
AGGAAATTCAAACCGAATCTCTTCGCCACTTTGTTCGGCCCGTACTCCATCCTGGGCAGA
TCCGTCGTGATCCACGAGCAGGAAGACGACATGGGCAAGGGCAACAACAAGGCCAGCCTG
GAGAACGGCAACGCGGGCAAGCGCCTGGCTTGCTGCGTGATCGGGGTGTGCAGCGGGAGC
TTGTGGGAAGAGAAACTGCCTGAGGTGGCGGACAAGAAGAAGAGATTGCTCAGAACAAGG
CTT
>Zebra_finch_SOD3
ATGTTTCTGATTCTTTCTCTGGTCATTGGGCTTACCCTGTCTGCCTCTGGTGTCATGACA
GACAAGGAAACTGACCCATGCCAGGAGTCACTGAATGAAATACAGAAACAAGTGAACGAC
CTCTGGCAGAATTTGCTCTACCCAGTAACAAATGGTAACGACACTGATGGAATGATTTAC
GCCACTTGTGAAATGAAGCCCAGCTCCAAAATAGATGCTGACAAGCCACAAGTGACTGGA
CAAGTCTTATTCAGGCAGAGTTACTCATATGGAAGACTGGAAGCCTTATTTTACTTGGAT

GGGTTTCCACTGGATAACAATCAGTCCAGCAGAGCTATTCACATCCATGAGCTTGGAGAC
CTCAGCAATGGCTGTGATTCTACAGGGGGACACTATAACCCTTTCAGAGTGAATCATCCT
CGTCACCCAGGAGATTTTGGCAACTTTCTTCCTAAAGAAGGCAAAATCAGAAAATACAAA
ACAAACCTCTTTGCCACAATCTTTGGTCCATATTCCATCATGGGCAGATCTGTTGTAATC
CACGAGCAGGAAGATGACATGGGCAAGGGCAATAATAAGGCCAGTTTGGAAAATGGAAAT
GCTGGGAAACGTCTGGCTTGCTGTGTGATTGGGATAAGCAACAAGAACCTGTGGGAAGAG
AAACTGCCTGAGGTTACGGACAAGAAGAAGAGAGGGCTCAACAGAAGAACATACAGCCAG
GCT
>Rock_pigeon_SOD3
ATGCTTCTGCTTCTTTCTCTGGTCGCTGGGCTTGCCCTGTCTGCCTCTGGTGGCGTGACA
GACAAAGAAAGTGATTCAAGCCAAGAGCCATTTCAAGACATAGAGACAAAAGTGAACGAC
CTCTGGCAGAGTTTGCTCTATCCGGTAGCGCCTGGTAATGAGAGCGACAGGATGATTTAC
GCCACTTGTGAAATGAAGCCCAGCTCCAAAATAGATGCTGACAAGCCACAAGTGACTGGA
CAAGTCTTATTCAGGCAGTATTACTCATATGGAAGACTAGAAGCCATTTTCTACTTGGAT
GGGTTTCCGCTGGATAATAATCAGTCTGGTAGAGCTATCCACATCCACGAGCTTGGGGAC
CTCAGCAACGGCTGCGATGCTACGGGAGGACACTATAACCCCTTCAGAGTGAACCACCCC
CGGCACCCAGGGGATTTTGGCAACTTTTTTCCTAAAGAAGGCAAAATCAGAAAATACAAA
ACTAATCTCTTTGCCACTATGTTTGGTCCATATTCCATCATGGGCAGATCTGTTGTGATC
CACGAACAGGAAGACGACATGGGCAAGGGCAACAATAAGGCCAGTTTGGAAAATGGAAAC
GCTGGGAAACGTCTGGCTTGTTGCGTGATTGGGATCTGCAACAAGAACTTGTGGGAAGAG
AAACTGTCTGAAGTTACAGACAAGAAGAAGAGAGGGCTCAACAAACGAACACAGAACCAG
GTT
>Flycatcher_SOD3
ATGTTTCTGATTCTTTCTCTGGTCACTGGGCTCGTCCTGTCTGCCTCTGGTGTCACCACA
GACAAGGAAACTGACCCACTCCAGGAATCATTGCATGAAATACAGAAACAAGTGAATGAC
CTCTGGCAGAATTTGCTCTACCCAGTAGCACGTGGTAACGAGACTGATGGGATGATTTAT
GCCACTTGTGAAATGAAGCCCAGCTCCAAAATAGATGCTGACAAGCCACAAGTGACTGGA
CAAGTCTTATTCAGGCAGCTTTACTCATATGGAAAACTGGAAGCCATTTTTTACTTGGAT
GGGTTTCCATTGGATAGCAATCAATCTAGCAGAGCTATACACATCCACGAGCTTGGAGAC
CTCAGCAATGGCTGTGATTCTACAGGGGGACACTACAACCCTTTCAGAGTGAATCACCCT
CGTCACCCAGGAGATTTTGGCAACTTTCTTCCCAAAGAAGGCAAAATCAGAAAATACAAA
ACAAACCTCTTTGCTACAGTCTTTGGCCCATATTCCATCATGGGCAGATCTGTTGTGATC
CACGAGCAGGAAGATGACATGGGCAAGGGCAATAATAAGGCCAGTTTGGAAAATGGAAAT
GCTGGGAAACGTCTGGCTTGCTGTGTGATTGGGATAAGCAACAAGAACTTGTGGGAAGAG
AAACTGCCTGAGGTTATGGACAAGAAGAAGAGAGGGCTCAACAAAAGAATGTACAGCCAG
GCT
>Medaka_SOD3
ATGAGTTTGCACAGGTCAGTAAGAGTCTTGGAAACGGCTTTCCTGGTCTGGCTGGCATGC
AGTCAACAATGCTTCTCAACGCACACCGATCATTTGCTCCCACCAGAGTTCTCACAGTAC
AATGGCACTCTGTATGCCGGCTGCAAAGTGAGCCCCAGCACTTCCCTTCCAGATGACCTG
CCTAAAGTGTACGGCCAAGCATTGTTTAAGCAAGATCACGGACAGGGAAAACTCCAAGTC
CTTCTGCAGCTCGCTGGCTTTCCTGAAGATGAGTCGCCACAGTCCAGAGCCATTCACATC
CACCAGTATGGGGATCTGAGCCAAGGATGCATTTCCACTGGTGGACATTACAACCCATAT
GGGGTGGATCACCCTAATCATCCGGGAGACTTTGGTAACTTTGTGGCCCACGAGGGGAGA

ATCAGTGAGCAGATAGAATCTGAAGCAACGCTGTTTGGTGGGCTGTCTGTGCTTGGAAGA
GCTGTGGTGGTTCACGAAACGATCGACGATTTAGGCCAAGGTGGAGATGTAGGGAGTCTG
CTGCATGGGAATGCAGGAAGAAGGCTTGCCTGCTGTGTTATTGGAATGTCTTCTTCCGAT
TTATGGGAAGAGCAGCAGAAACTGCAAAGCAGC
>Fugu_SOD3
ATGCGTCCACACGGGTCCGCGAGTGCATTGGCAGCGCTGATGCTTCTGCTGGCTGATTGT
CAGCGCTGTGTCTTAGCTGAAGGGAACGGCTCAGCTCTTCCAGAGGCCTCCCAGAACAAT
GGCAGCCTGTACGCGTCCTGCAACATGAGACGCAGCAGCGCGCTGCCAGAGGATGTGCCT
CAACTCTACGGTCACGTGCTGTTCAAGCAGGACCGGCCTCAGGGAATTCTCAGGGTCCTC
TTTCACGTTGGTGGCTTTCTGACTGATGCCGAGCCCAAAGCCATCCACATCCATCAGTAC
GGAGACCTGAGCCAGGGGTGCGGCTCCACCCGGGGCCACTACAACCCATATGGCGAAAAC
CACCCTAACCACCCGGGAGACTTCGGGAACTTCGTGCCTCAGGAGGGAATTATCTCAGCT
GTGGTTGAGTCGAAGGCGACGCTGTTCGGAGGATGTCTGCCATTGAGAGCAGTGNGTGTC
CACGAAAAGAACGACGACNCTGGCCAGGGCGGCGACGCCGGGAGCCTCCTGCATGGAAAC
GCGGGGCGGAGGCTGGCCTGCTGCGTTATTGGGATTTCCNTTCCCGATCTGTGGAACCTC
AACTATCCGAAGTTTGCAGAAAGGATGAAAAAGAAT
>Great_tit_SOD3
ATGTTTCTGATTCTTTCCCTGGTCACTGGGCTCGCCCTGTCTGCCACTGCTGTCACAACA
GACAAGGAAACTGACCCACGCCAGGAGTCACTGCATGAAATACAGAAACAAGTGAACGAC
CTCTGGCAGAATTTGCTCTACCCTGTAACACGTGGTAACGAGACTGATGGGATGATTTAC
ACCACTTGTGAAATGAAGCCCAGCTCCAAAATAGATGCTGACAAGCCACAAGTGACTGGA
CAAGTCTTATTCAGGCAGTATTACCCATATGGAAGACTGGAAGCCCTTTTTTACTTGGAT
GGGTTTCCACTGGATAACAATCAATCTAGCAGAGCTATACACATCCACGAGCTTGGAGAC
CTCAGCAACGGCTGTGATTCTACAGGGGGACACTATAACCCTTTCAGAGTGAATCACCCT
CGTCACCCAGGAGATTTTGGCAACTTTCTTCCTAAAGAAGGCAAAATCAGAAGATACAAA
ACAAACATCTTTGCCACAGTCTTTGGTCCGTATTCCATAATGGGCAGATCTGTTGTGATC
CACGAGCAGGAAGATGACATGGGCAAGGGCAATAATAAGGCCAGTTTAGAAAATGGAAAT
GCTGGGAAACGTCTGGCTTGCTGTGTGATTGGGATAAGCAACAAGAACTTGTGGGAAGAG
AAACTGCCTGAGGTTATGGACAAGAAGAAGAGAGGTCTCAAAAGAACA
>African_ostrich_SOD3
ATGCTTCTGCTTCTTTCTCTGGTCACTGGACTTGCCCTTTCCGCCTCTGGTGCCGTGACA
GAAACCGATCCAAATTCAGAGCCATTTCATGATATACAGAAAAAAGTGAATGACCTCTGG
CAGAATTTGCTCTATCCAGTAATGGTTAATAATGAGACTGATGGGATACTTTATGCCACC
TGTGAAATGCTGCCCAACTCCAAAATACATGCTAACAAGCCAAAAGTGACTGGACGAGTT
TTGTTCAAACAGTGTTACTCATATGGAAGATTAGAAGCAATTTTTGACTTGGATGGGTTT
CCATTGCATAACAATCAGTCTGGGAGAGCTATACACATCCACAAGCTCGGAGATCTCAGC
GACGGCTGTGATTCTACAGCAGGACACTATAACCCTTTCGGCGTGAATCACCCTCATCAC
CCAGGGGATTTTGGCAACTTTTTCCCTAAAGAAGGCAAAATCAGAAAATACAAACCAAAC
CTCTCTGCCACTATGTTTGGTCCATATTCCATCCTGGGCAGATCCGTTGTGATTCATGAG
CAGGAAGATGACATGGGCAAAGGCAACAACAAGGCCAGTTTGGAAAATGGAAATGCTGGC
AAACGTCTAGCTTGCTGTGTGATTGGGATATGCAACAAGAACTTGTGGGAAGAGAAACTG
TCTGAGGTTGCAGACAAGAAGAAGAGATGGCTCAACAAGCGAACACACAGCCAGGCT
>Adelie_penguin_SOD3
ATGCTTCTGCTTCTTTCTCTGGTCGCTGGGCTTGCCCTGTCTGCCTCTGGCATCATGACA

GACAAAGAAACTGATCCAAGCCAAGAGTCATTTCATGACATACAGAAAAAAGTGAACGAC
CTCTGGCAGAAATTGCTCTATCCGGTAATGCCTGGTAATGAGACTGATGGGATGATTTAC
GCCACTTGTGAAATGAGGCCGAGCTCCAGAATAGATACTGACAAGCCACAAGTGACTGGA
CAAGTCTTATTCAGACAGTATTACTCATATGGAAGATTAGAAGCCATTTTTTACTTGGAT
GGGTTTCCGTTGGATAATAATCAATCTGGTAGAGCTGTACACATCCATGAGCTTGGGGAT
CTCAGCAACGGCTGTGATTCTACGGGAGGACACTATAACCCTTTCAGCGTGAATCACCCC
CGTCACCCAGGGGATTTTGGCAACTTTTTTCCTAAAGAAGGCAAAATCAGAAAATACAAA
CCAAATCTCTCTGCCACTATGTTTGGTCCATATTCCATCATGGGCAGATCTGTTGTGATC
CATGAGCAGGAAGACGACATGGGCAAGGGCAACAATAAAGCCAGTTTGGAAAATGGAAAT
GCTGGGAAACGTCTGGCTTGCTGCGTGATTGGGATATGCAACAAGAACTTGTGGGAAGAG
AAACTGCCCGAGGCTACAGACAAGAAGAAGAGAGGGCTCAACAAACGAACA
>common_starling_SOD3
ATGTTTCTGATTCTTTCTCTGGTCACTGGGCTCGCCCTGTCTGCCTCTGGTGTCATGACA
GACAAGGAAACTGACCCACTCCAGGAGTCATTGCATGAAATACAGAAACAAGTGAACGAC
CTGTGGCAGAATTTGCTCTACCCAGTAGCACGTGGTAACGAGACTGATGGGATGATGTAC
GCCACTTGTGAAATGAAGCCCAGCTCCAAAATAGATGCTGACAAGCCACAAGTGACTGGA
CAAGTCTTATTCAGGCAGCTTTACTCATATGGAAGACTGGAAGCCATTTTTTACTTGGAT
GGGTTTCCACTGGATAACAATCAATCTAGCAGAGCTATACACATCCACGAGCTTGGAGAC
CTCAGCAATGGCTGTGATTCTACAGGGGGACACTATAACCCTTTCAGAGTGAATCACCCT
CGTCACCCAGGAGATTTTGGCAACTTTCTTCCTAAAGAAGGCAAAATCAGAAAATACAAA
ACAAACCTCTCTGCCACAGTCTTTGGTCCATATTCCATCATGGGCAGATCTGTTGTGATC
CACGAACAGGAAGATGATATGGGCAAGGGCAATAATAAGGCCAGTTTGGAAAATGGAAAT
GCTGGGAAACGTCTGGCTTGCTGTGTGATTGGGATAAGCAACAAGAACTTGTGGGAAGAG
AAACTGCCTGAGGTGATGGACAAGAAGAAGAGAGGGCTCAACAAAAGAACATACAGCCAG
GCT
>central_bearded_dragon_SOD3
ATGTTCCTGCTTCTGTTTCTTGCACCAGGCTTGATTCTTTGTGATTCTGATGGCATACCA
GCAGATGTCCAGCCTCTTGACCAAAGCCCACTTGAAGACATTCAGAAAAAAGTTAATGAC
TTATGGCTGAGTTTGCTTTACCCCCAGTTCTATGAGGAGCTGCTGGCAACCAACCAGACT
GCTTATGCTGTGTGTGTAGTGAAACCCAACACCAAATTGGATGCAGACAAGCCACAGGTG
ACTGGGCATGTTCTTTTCAAGCAAGCCTATCCATATGGAAAATTGGAGGCTGTTTTCTAT
TTGGATGGGTTTCCTGCAGGCACCAACTTGTCTGGCAGAGCTATTCATGTTCACCAGCAT
GGAGACCTCAGTGACAGCTGTGACTCTGCTGGGGGACACTACAATCCTTTCAAAGTAAAT
CACCCTAAACATCCTGGGGACTTTGGGAACTTCTACTCTAAAGGAGGCAAAATTATAAAA
TACAAATCCAATCTCACGGCAACTCTTTTTGGTCCACACACCATTATAGGAAGATCCATT
GTGATCCATGAACAGGAAGATGACCTAGGGAAAGGGAGCAACAAAGCCAGCCTGGAGAAT
GGAAATGCTGGTAAACGTCTGGCTTGTTGTGTCATTGGAACATCCAACAAAAACCAGTGG
GCAAATCAGTTTGCTGAGATTCCAGCAAAGAGGAGGAAAAGGATCACAAAA
>Gecko_SOD3
ATGTCCCTGCTGCGGTTTCTAGTCACGGGCTTGATCCTTTGTGTATCTGATGAAACAAAA
CAAGAAGAAAGCTCCAAGCTTGCCATTGAGGAGACTCAGGGAAAGCTGAATTTACTGTGG
CTGGCTATCCTCTACCCAAATATCTACGGGCACTTGTACGAAGGCAACTGGACCTATGCC
GCTTGTGAATTGAAGCCCAGTACCAACCTAGAAGCTGGCAAACCACAGGTGACAGGGAGA
GTTCTGTTCAAGCAAATTCACCCACATGGAAAACTGAAGGCCATATTCTACCTGGATGGG

TTTCCCGAGGACGCCAACCAAACTGGTAGAGCTATCCACATCCATGAACTGGGAGACCTC
AGCAACGGCTGTGATTCTGTCAGCGGCCACTACAATCCTTTCAAAGTCCGTCACCCCAAC
CATCCGGGGGACTTTGGAAACTTCTATCCTAAAGATGGCAAGATCAGAACATTGATGGTC
AACCTCCAACCGTCTCTTTTTGGCCCCCAAGCTGTTCTAGGAAGATCTGTCGTGATCCAC
GAGAATGCAGACGATATGGGGAAAGGAAAAAACACAGGCAGTTTAGAACATGGAAATGCT
GGCAAACGCTTGGCTTGCTGCGTCATTGGATACTGCACAAAAGAATTGTGGCTGAAGGCC
TACGAGGAGCTTCTTAAGCTCAGAGAC
>Taiwan_habu_SOD3
ATGAAGGGTCTGACTTGGTCCAACATGCTGCTTCTGCTGCTGCTCACTCCCAGCTTGGTC
CTTTGTGACACTAACACCACCGTGGTATGGGAAGACGCGCCGTCTACCGTCAGCCCCCTT
GTAGACATGCAGAAGAAAATTAACGATCTGTGGCTGAGTTTGCTGTACCCCCAGCTATAC
GAGGACATCATCAAAGCCAACCGGACCGCCTACGCTTGCTGCGCAGTCAAGCCCAGTAGC
AAATTAGAGGCGGAGAAACCTCAGGTGAAGGGTTCGGTTTTGTTCAAGCAGGGCTACCCC
CATGGAAAGCTGGAGGTCATCATCTACCTGGCCGGCTTTCCCATGAACACATCGGCCAGG
GCCATTCACGTCCACCAGTACGGTGACCTCAGCGACGGCTGCGATTCGGTCGGAGGTCAC
TACAACCCTTGGAAAGTCAACCACCCTCACCATCCTGGGGATTTTGGGAACTTTGAGCCT
CAAGAGGGCAAGGTTCAAAGATTCAAATCGAACCTCAAAGCCACCCTCTTTGGACCCCAG
ACCATTCTAGGAAGATCGGTTGTGATCCACGAGCAGAAAGATGACCTGGGAAAGGGCGGC
AACAAAGGCAGCCTGGAGCACGGGAACGCTGGCCTGCGTCTAGCGTGTTGCGTCATCGGC
ACCTGTAAGGAGAACCTGTGGGAGCAGCATTTGCCTAAAGTCCCCACCAAAAGGAAGAGA
AGGGTTATAAAATTGGGGGTCCCT
>green_sea_turtle_SOD3
ATGTTTCTGTTCCTTTACCTGGTCATCAGCCTGGGCCTCTCTGCATCTGGTGTTGTGAGG
GGAGAAGGTGAGGTTGATTCCAATGGAGACAGCCCACTTCAAGACATACAGAAAAAAGTG
AATGACCTGTGGCAGAATTTACTCTACCCACAGTTCATCAATGAGGAGACTGACCGGGCA
GGTTATGCCACCTGTGAAGTGAAGCCCAGCTCCAACCTAGATGCTGACAAGCCACAGGTG
ACAGGCCAAGTCTTGTTCAGACAGTCCTACCCAAATGGAAAACTAGAGGCTCTCTTTGAC
TTGAATGGGTTTCCGTCAGGCAGCAATCTGTCTGGCAGAGCTATTCACATCCATAAGCTC
GGAGACCTCAGCAACAGCTGTGACTCTACTGGGGGGCACTATAACCCTTTCAATGTGAAC
CACCCTCGCCATCCAGGGGATTTTGGCAACTTCTACTCTAAAGAAGGCAAAATTAGAAAA
TACAAGCCAAATCTGCTCGCCACTCTGTTTGGCCCCTACTCCGTCATGGGGAGATCCATT
GTGATCCATGAACAGGAAGATGACATGGGCAAGGGTAACAATAAAGCCAGTTTGGAGAAT
GGGAATGCTGGCAGACGTCTAGCTTGCTGTGTCATTGGGATATGCAACAAGAACTTATGG
GAGAAAAAGCTTCCTGACATTACAGACAGGAGGAAGAAGAGGATCACAAATGAGCACAAA
ACAACCAGGCCTAAC
>Chinese_alligator_SOD3
ATGTTTCTATCCCTTTGTCTGGTATCCGTGGGGCTGTTCCTATCCTTACCTGGTGTTGTA
AGCGGAGAATTGGAGCCTGTTCCTGAAACGGAAGGCCTGTTTCAAGAATTACGGAAAAAA
GTGAATGACTTGTGGCAGAATTTGCTCTACCCACAATTTATCAGAGACAGTGACTGGACG
GCTTACGCTACTTGTGAAATGAAGCCCAGCTCCAAATTAGATGCTGACAAACCACAAGTG
ACAGGACAAATCTTGTTCAAACAGTCCTACCCAATTGGAAAGCTAGAAGCTTTCTTTGAC
CTGGATGGGTTTCCATCAGAGAACAATCAGTTGGGTAGGGCTATTCATATCCATAAGTTT
GGAGACCTCAGCGACGGCTGTAACACTACTGGAGGACACTATAACCCCTTCAATGTGAAC
CATCCTCACCATCCAGGGGATTTTGGCAACTTCTACTCTAAAGAAGGCAAAATTAGAAAA

TACAAGTCAAACCTGTCTGCTACTCTGTTTGGCCCATATACCATTATGGGCAGATCTGTT
GTTATTCATGAGCAGGAAGATGACATGGGCAAAGGTAACAATAAGGCCAGTTTGGAGAAT
GGAAATGCGGGCAGACGTCTGGCTTGCTGTGTCATTGGGATATGCGACAAGAACATGTGG
GAGAATAAATTCTCCAAGTTTGTAGAAAGGAGGAAAAGAGGCTCACAAAATGAGCACAGA
AGCAGGCCCAGC
>Gharial_SOD3
ATGTTTCTGGCCTTTTGTCTGGTATCCGTGGGGCTGCTCCTCTCCTTACCTGGTGTTGCA
AGGGGAGAATTGGAGCCTGTTCCTGAAATGGAAGGCCTGTTTCAAGAATTACAGAAAAAA
GTGAATGACTTGTGGCAGAATTTGCTCTACCCACAATTTCTCAGACAGAGTGAGTGGACG
GCTTACGCTACTTGTGAAATGAAGCCTAGCTCCAAATTAGATGCTGACAAACCACAAGTG
ACAGGACAAATCTTGTTCAAACAGTCCTACCCAATTGGAAAGCTAGAAGCTTTCTTTGAC
CTGGATGGGCTTCCATCAGAGAACAATCAGTTGGGTAGGGCTATTCACATCCACAAGTTT
GGAGACCTCAGTGATGGCTGTAACACTACTGGAGGACACTATAACCCCTTCAATGTGAAC
CATCCTCACCATCCAGGGGATTTTGGCAACTTCTACTCTAAAGATGGCAAAATTAGAAAA
TACAAGTCAAACCTGTCTGCTACTCTGTTTGGCCCATATACCATTATGGGCAGATCTGTT
GTTATTCATGAGCAGGAAGATGACATGGGCAAAGGTAACAATAAGGCCAGTTTGGAGAAT
GGAAATGCGGGCAGACGTCTGGCTTGTTGTGTCATTGGGATATGTGACAAGAACACGTGG
GAGAATAAATTCTCCAAGTTTGCAGAAAGAAGGAAAAGAGGCTCACAAAATGAGCACAGG
AGCAGGCCCAAC
>southern_platyfish_SOD3
ATGCGCATTTACAGGTCATCGAGTGGACTCCAAACTGCTCTGCTGGTACTGCTGGCCAGT
TGTCAATGCATCTCAGCAAACACCGATACTCTCCTTCCTCCAGAGGTCTCTCAGTACAAT
GGCACTCTGTATGCAGCCTGCAAAGTGAGACCCAGCACTTCACTTCCTGAGGGGCTTCCC
AAGGTATACGGGCAGGTGCTGTTTAAACAAGAGCAGTCTGAGGGAAAGCTTCAAGTCCTC
CTTCGGCTCAATGGCTTCCCAACAGAGGATGCTCCAGAACCCAGAGCCGTGCACATCCAT
CAGTACGGAGACCTGAGCCGGGGATGCGACTCAACCGGTGGTCACTACAACCCGTTTGAT
GTGAACCATCCTAAACACCCAGGAGACTTTGGTAACTTCATCTCACAGGAAGGACAAATC
AAAGTGCTGATTGAATCAGAGGCCACCCTGTTTGGAAACCTGTCTGTGCTCGGAAGAGCG
GTGGTGATCCATGAGAAGAGAGACGACTTGGGGCTCGGCGGAGACATCGGGAGCCTGCTC
CATGGGAACGCAGGCCGCAGGCTTGCCTGCTGCGTTATTGGGATGTCCTCCTCCAGCCCC
TGGAATATGCAACATGAGCGTTTCGCAAGGCAGGTCAGGAGGAAC
>spotted_gar_SOD3
ATGGCGAAAACTATTTTCTGGCTAAAATGTCTGCTCATGCTGGTCTTCTTGGGAATCCAC
GTGTTTTGCTGTGAAGATGGCACAGTCAGCGAAGAGCAGGTGCAGAAATACATCGAGGAG
AGGATGAAAGCCGTTTGGCCTCTTCCTGCTACCCCAGAAGCGGGTAACGACGACCAAAAC
GTCTACGCAGCATGTGTCATGAAGCCCAGCAGTAAACTGGAAGAAGGCAAGCCACAAGTC
ACAGGAGAAGTGCTTTTCAAGCAGCTCTACCCGGATGGAAAACTTGACGTGATATTTAGA
CTGGCGGGCTTCAACACCACCGATAACACATCAAAAGCCATCCACGTTCACACGTACGGA
GACCTCAGCAACGGGTGCGATTCTACCGGCGGCCATTTCAACCCCCAAAATGTTGACCAC
CCCATGCACCCAGGCGACTTTGGCAACTTTACGCCAAAGAAGGGGAAGATACATAAAATG
AAGAGCAACAGCAAAGCCACCATGTTTGGGCCAGAGTCGGTACTCAGCCGGGCAGTGGTG
GTTCACGAGAAGGAAGACGACATGGGAAAAGGAGGCAATGCTGGAAGCCTGCTGCACGGA
AACGCAGGCAAGCGGCTGGCCTGCTGTGTCATTGGGATCACCAAGCAGAAGCTCTGGAGC
GACACAATCGAAAGCACCCCCTTCAAGGACTGGCATCCTGACCAGGGGGTTCCCTGCCTT

GTGCCCATTGCTTGCCAGGAAAATCTCCAAATCCCCCTCAACTCTGAATTGGAAGAAACG
GTTGGAAGAATGGAAGAG
>guppy_SOD3
ATGCGCATTAACAGGTCATCGAGTGGACTCCAAACTGCTCTGCTGGTACTGCTGGCCAGT
TGTCAATGCATCTCAGCAAACACCGATACTCTCCTTCCTCCAGAGGTCTCTCAGTACAAT
GGCACTCTGTATGCAGCCTGCAAAGTGAGACCCAGCACTTCACTTCCTGAGGGGCTTCCC
AAGGTATACGGGCAGGTGCTGTTCAAACAGGAGCAGTCTGAGGGAAAGCTTCAAGTCCTC
CTTCGGCTCAATGGCTTCCCAGCAGAGGACGCTCCAGAACCCAGAGCCGTGCACATCCAT
CAGTACGGAGACCTGAGCCGGGGATGCGATTCAACCGGTGGCCACTACAACCCGTTTAGT
GTGAACCATCCTAAACACCCGGGAGACTTTGGCAACTTCGTCTCACAGGAAGGACAAATC
ATTGAGCTGATTGAATCAGAGGCCACCCTGTTTGGAGGCCTGTCTGCGCTCGGAAGAGCG
GTGGTGATCCATGAGAAGAGAGACGACCTGGGGCTCGGCGGAGACATCGGGAGCCTGCTC
CATGGGAACGCAGGCCGCAGGCTTGCCTGCTGCGTTATCGGGATGTCCTCCTCCAGCGTC
TGGAATACGCAGCATGAGCGTTTCGCAAGGCAGCTTAGGAGGAAT
