# Supplementary material for: Molecular evolutionary patterns of NAD+/Sirtuin aging signaling pathway across taxa
Source: PLoS One. 2017 Aug 2;12(8):e0182306. doi: 10.1371/journal.pone.0182306 (PMC5540417; doi:10.1371/journal.pone.0182306)
Supplement: S2 Table — (PDF) [file pone.0182306.s002.pdf]

Taxon		Species	PARP1	PARP2	SIRT1	SIRT2	SIRT6	MRPS5	FOXO3a	TP53	PPARGC1A	SOD3
	Fruitfly	*Drosophila melanogaster*	NCBI protein id: NP_001104452.1	×	NCBI protein id: NP_477351.1	NCBI protein id: NP_650880.2	NCBI protein id: NP_649990.2	NCBI protein id: NP_001036653.1	NCBI protein id: NP_996205.1	NCBI protein id: NP_996268.1	NCBI protein id: NP_649469.2	NCBI protein id: NP_725046.2
	Worm	*Caenorhabditis elegans*	NCBI protein id: NP_491072.1	NCBI protein id: NP_001022057.1	×	NCBI protein id: NP_001255484.1	×	NCBI protein id: NP_506145.2	NCBI protein id: NP_001021595.1	×	×	NCBI protein id: NP_510764.1
	Yeast	*Saccharomyces cerevisiae*	×	×	×	NCBI protein id: NP_010242.1	×	NCBI protein id: NP_009810.3	×	×	×	×
	Mouse	*Mus musculus*	NCBI protein id: NP_031441.2	NCBI protein id: NP_033762.1	NCBI protein id: NP_062786.1	NCBI protein id: NP_001116237.1	NCBI protein id: NP_853617.1	NCBI protein id: NP_084239.1	NCBI protein id: NP_062714.1	NCBI protein id: NCBI protein id: BAA82343.1	NCBI protein id: NP_032930.1	NCBI protein id: NP_035565.1
Model species	Zebrafish	*Danio rerio*	NCBI protein id: NP_001038407.1	NCBI protein id: NP_001191199.1	NCBI protein id: XP_001334440.4	NCBI protein id: NP_955890.1	NCBI protein id: NP_001002071.1	NCBI protein id: NP_001070064.1	NCBI protein id: AAH88386.1	NCBI protein id: AAH95597.1	NCBI protein id: XP_017212627.1	NCBI protein id: XP_001332758.1
	Human	*Homo sapiens*	NCBI protein id: NP_001609.2	NCBI protein id: NP_005475.2	NCBI protein id: NP_036370.2	NCBI protein id: AAK51133.1	NCBI protein id: NP_057623.2	NCBI protein id: NP_114108.1	NCBI protein id: NP_963853.1	NCBI protein id: BAC16799.1	NCBI protein id: NP_001317680.1	NCBI protein id: CAG46651.1
	Macaque	*Macaca mulatta*	NCBI protein id: XP_005541069.1	NCBI protein id: NP_001244367.1	NCBI protein id: XP_015003002.1	NCBI protein id: XP_014979357.1	NCBI protein id: XP_001101773.1	NCBI protein id: XP_014968320.1	NCBI protein id: XP_014993103.1	NCBI protein id: AAB91534.1	NCBI protein id: XP_014993594.1	NCBI protein id: NP_001181261.1
	Orangutan	*Pongo abelii*	NCBI protein id: XP_002809428.2	NCBI protein id: XP_002824550.1	NCBI protein id: XP_002820941.1	NCBI protein id: NP_001125519.1	NCBI protein id: XP_003779077.1	NCBI protein id: NP_001124839.1	NCBI protein id: XP_009240402.1	NCBI protein id: XP_002827020.1	NCBI protein id: NP_001125407.1	NCBI protein id: XP_009238123.1
	Naked mole rat	*Heterocephalus glaber*	NCBI protein id: NP_001297155.1	NCBI protein id: XP_012921120.1	NCBI protein id: XP_012928418.1	NCBI protein id: XP_021095315.1	NCBI protein id: NP_001297193.1	NCBI protein id: XP_004844395.1	NCBI protein id: XP_004855478.1	NCBI protein id: NP_001297199.1	NCBI protein id: XP_021115878.1	NCBI protein id: XP_004847262.1
	Rabbit	*Oryctolagus cuniculus*	NCBI protein id: XP_008266574.1	NCBI protein id: XP_008267516.1	NCBI protein id: XP_002718506.1	NCBI protein id: XP_008255379.1	×	NCBI protein id: XP_002709627.1	NCBI protein id: XP_008261561.1	NCBI protein id: CAA62216.1	NCBI protein id: XP_002709423.1	NCBI protein id: NP_001076101.1
	Panda	*Ailuropoda melanoleuca*	NCBI protein id: XP_011232467.2	NCBI protein id: XP_002929561.1	NCBI protein id: XP_019663508.1	NCBI protein id: XP_002927438.2	NCBI protein id: XP_002928505.1	NCBI protein id: XP_002927098.1	NCBI protein id: XP_019653183.1	NCBI protein id: XP_002924483.1	NCBI protein id: XP_019656088.1	NCBI protein id: XP_011235621.2
	Dog	*Canis lupus familiaris*	NCBI protein id: XP_547506.3	NCBI protein id: XP_532616.3	NCBI protein id: XP_546130.2	NCBI protein id: XP_013976051.1	NCBI protein id: XP_542163.2	NCBI protein id: XP_532953.3	NCBI protein id: XP_003639448.1	NCBI protein id: BAA78379.1	NCBI protein id: XP_005618712.1	NCBI protein id: XP_545973.3
	Horse	*Equus caballus*	NCBI protein id: XP_014593504.1	NCBI protein id: XP_005603232.1	NCBI protein id: XP_014588584.1	NCBI protein id: XP_014590298.1	NCBI protein id: XP_005614857.1	NCBI protein id: XP_001494506.3	NCBI protein id: XP_014590758.1	NCBI protein id: NP_001189334.1	NCBI protein id: XP_014594249.1	NCBI protein id: XP_005615141.2
	Cow	*Bos taurus*	NCBI protein id: NP_777176.1	NCBI protein id: XP_010808738.1	NCBI protein id: NP_001179909.1	NCBI protein id: NP_001107003.1	NCBI protein id: NP_001091553.1	NCBI protein id: NP_001039588.1	NCBI protein id: NP_001193012.1	NCBI protein id: CAA57348.1	NCBI protein id: NP_808814.1	NCBI protein id: NP_777040.1
	Pig	*Sus scrofa*	NCBI protein id: XP_003357689.2	NCBI protein id: XP_005656371.1	NCBI protein id: NP_001139222.1	NCBI protein id: NP_001107743.1	NCBI protein id: NP_001107745.1	NCBI protein id: XP_003124867.3	NCBI protein id: NP_001129431.1	NCBI protein id: NP_998989.3	NCBI protein id: NP_999128.2	NCBI protein id: XP_020956182.1
	Dolphin	*Tursiops truncatus*	NCBI protein id: XP_019776083.1	NCBI protein id: XP_019790777.1	NCBI protein id: XP_004320193.2	NCBI protein id: XP_019796593.1	NCBI protein id: XP_019802690.1	NCBI protein id: XP_004312773.1	NCBI protein id: XP_004327408.2	NCBI protein id: XP_019799782.1	NCBI protein id: XP_019783967.1	NCBI protein id: XP_019783965.1
	Elephant	*Loxodonta africana*	NCBI protein id: XP_010589425.1	NCBI protein id: XP_010599303.1	NCBI protein id: XP_010587464.1	NCBI protein id: XP_003420816.1	NCBI protein id: XP_003421943.1	NCBI protein id: XP_010599670.1	NCBI protein id: XP_003404353.1	NCBI protein id: XP_010594888.1	NCBI protein id: XP_010589697.1	NCBI protein id: XP_003411340.2
	Armadillo	*Dasypus novemcinctus*	NCBI protein id: XP_004472547.1	NCBI protein id: XP_004482743.1	NCBI protein id: XP_004470564.1	NCBI protein id: XP_004481514.2	NCBI protein id: XP_012382537.1	NCBI protein id: XP_012374946.1	NCBI protein id: XP_004468469.1	NCBI protein id: XP_012384548.1	NCBI protein id: XP_004461876.1	NCBI protein id: XP_012381927.1
	Opossum	*Monodelphis domestica*	NCBI protein id: XP_007481563.1	NCBI protein id: XP_007479798.1	NCBI protein id: XP_001369605.3	NCBI protein id: XP_007491852.1	NCBI protein id: XP_001374380.1	NCBI protein id: XP_00138207	NCBI protein id: XP_001368493.1	NCBI protein id: XP_007483418.1	NCBI protein id: XP_007496717.1	NCBI protein id: XP_016278786.1
Mammal	Platypus	*Ornithorhynchus anatinus*	NCBI protein id: XP_016083918.1	NCBI protein id: XP_007654968.1	NCBI protein id: XP_001508324.2	×	NCBI protein id: XP_016080987.1	NCBI protein id: XP_001506236	NCBI protein id: XP_001511165.2	NCBI protein id: XP_001521340.4	NCBI protein id: XP_007671417.1	NCBI protein id: XP_003428606.2
	Chicken	*Gallus gallus*	NCBI protein id: NP_990594.2	×	NCBI protein id: NP_001004767.1	NCBI protein id: NP_001017414.1	NCBI protein id: NP_001034409.1	NCBI protein id: NP_001186319.1	NCBI protein id: XP_001234496.4	NCBI protein id: NP_990595.1	NCBI protein id: NP_001006457.1	NCBI protein id: XP_015141186.1
	Turkey	*Meleagris gallopavo*	NCBI protein id: XP_010704958.1	×	NCBI protein id: XP_010712566.1	×	NCBI protein id: XP_003213275.1	NCBI protein id: XP_010704918	NCBI protein id: XP_010705770.1	×	NCBI protein id: XP_003205943.1	NCBI protein id: XP_019470472.1
	Duck	*Anas platyrhynchos*	NCBI protein id: XP_005014800.3	×	NCBI protein id: XP_005009174.1	×	NCBI protein id: XP_021127464.1	NCBI protein id: XP_005022109	NCBI protein id: XP_012952028.1	×	NCBI protein id: XP_005031720.1	NCBI protein id: XP_005019322.1
	Flycatcher	*Ficedula albicollis*	NCBI protein id: XP_005043071.2	×	NCBI protein id: XP_005047970.1	×	NCBI protein id: XP_005060274.1	NCBI protein id: XP_016152426	NCBI protein id: XP_005044018.2	×	NCBI protein id: XP_005045466.1	NCBI protein id: XP_005045465.1
	Common starling	*Sturnus vulgaris*	NCBI protein id: XP_014732939.1	×	NCBI protein id: XP_014738778.1	NCBI protein id: XP_014749139.1	NCBI protein id: XP_014742716.1	NCBI protein id: XP_014748112	NCBI protein id: XP_014743370.1	NCBI protein id: XP_014748868.1	NCBI protein id: XP_014740637.1	NCBI protein id: XP_014746986.1
	Great tit	*Parus major*	NCBI protein id: XP_015478265.1	×	NCBI protein id: XP_015488114.1	NCBI protein id: XP_015472185.1	NCBI protein id: XP_015507146.1	NCBI protein id: XP_015476521	NCBI protein id: XP_015476009.1	×	NCBI protein id: XP_015480610.1	NCBI protein id: XP_015480484.1
	Zebra finch	*Taeniopygia guttata*	NCBI protein id: XP_012427694.1	×	NCBI protein id: XP_012429832.1	×	NCBI protein id: XP_002195564.1	NCBI protein id: XP_012427623	NCBI protein id: XP_002192850.2	×	NCBI protein id: XP_002192636	NCBI protein id: XP_002192051.1
	Rock pigeon	*Columba livia*	NCBI protein id: XP_005507407.2	×	NCBI protein id: XP_005512829.1	×	NCBI protein id: XP_021155704.1	NCBI protein id: XP_021151992.1	NCBI protein id: XP_021152934.1	×	NCBI protein id: XP_005501343.1	NCBI protein id: XP_021141766.1
	Adelie penguin	*Pygoscelis adeliae*	NCBI protein id: XP_009331916.1	×	NCBI protein id: XP_009324269.1	×	NCBI protein id: XP_009321769.1	*NCBI protein id: XP_009322493*	NCBI protein id: XP_009324696.1	×	NCBI protein id: XP_005501343.1	NCBI protein id: XP_009318163.1
Aves	African ostrich	*Struthio camelus*	NCBI protein id: XP_009684683.1	×	NCBI protein id: XP_009674395.1	×	NCBI protein id: XP_009675807.1	NCBI protein id: XP_009685136	NCBI protein id: XP_009675233.1	×	NCBI protein id: XP_009671609.1	NCBI protein id: XP_009671612.1
	Anole lizard	*Anolis carolinensis*	NCBI protein id: XP_003216114.1	NCBI protein id: XP_008113575.1	NCBI protein id: XP_003223739.1	NCBI protein id: XP_003229391.2	NCBI protein id: XP_008123970.2	NCBI protein id: XP_003216145	NCBI protein id: XP_003215683.1	NCBI protein id: XP_008123458.1	NCBI protein id: XP_008109489.1	NCBI protein id: XP_008109486.1
	central bearded dragon	*Pogona vitticeps*	NCBI protein id: XP_020642204.1	NCBI protein id: XP_020668376.1	NCBI protein id: XP_020665072.1	NCBI protein id: XP_020642803.1	NCBI protein id: XP_020636527.1	NCBI protein id: XP_020650441	NCBI protein id: XP_020656989.1	NCBI protein id: XP_020665272.1	NCBI protein id: XP_020636089.1	NCBI protein id: XP_020636087.1
	Taiwan habu	*Protobothrops mucrosquamatus*	NCBI protein id: XP_015675666.1	NCBI protein id: XP_015680516.1	NCBI protein id: XP_015672141.1	NCBI protein id: XP_015682155.1	NCBI protein id: XP_015680119.1	NCBI protein id: XP_015673444.1	NCBI protein id: XP_015676797.1	×	NCBI protein id: XP_015683012	NCBI protein id: XP_015684068.1
	Gecko	*Gekko japonicus*	NCBI protein id: XP_015284457.1	NCBI protein id: XP_015273533.1	NCBI protein id: XP_015278674.1	NCBI protein id: XP_015280504.1	NCBI protein id: XP_015276013.1	NCBI protein id: XP_015271625.1	NCBI protein id: XP_015260889.1	NCBI protein id: XP_015279412.1	NCBI protein id: XP_015272246.1	NCBI protein id: XP_015272245.1
	painted turtle	*Chrysemys picta bellii*	NCBI protein id: XP_005289622.1	NCBI protein id: XP_008172910.1	NCBI protein id: XP_005297342.1	NCBI protein id: XP_005293248.2	NCBI protein id: XP_005281259.1	NCBI protein id: XP_008165301.1	NCBI protein id: XP_005287710.1	NCBI protein id: XP_005279396.1	NCBI protein id: XP_005303505.1	NCBI protein id: XP_005303507.1
	Chinese softshell turtle	*Pelodiscus sinensis*	NCBI protein id: XP_006118141.1	×	NCBI protein id: XP_006125338.1	NCBI protein id: XP_014431529.1	NCBI protein id: XP_006135667.1	NCBI protein id: XP_014435291	NCBI protein id: XP_006123241.1	NCBI protein id: XP_006112198.1	NCBI protein id: XP_006138221.1	NCBI protein id: XP_006138225.1
	American alligator	*Alligator mississippiensis*	NCBI protein id: XP_019338553.1	NCBI protein id: XP_006266632.2	NCBI protein id: XP_019339945.1	NCBI protein id: XP_019333189.1	NCBI protein id: XP_014464318.1	NCBI protein id: XP_014453699.1	NCBI protein id: KYO24694.1	NCBI protein id: KYO28679.1	NCBI protein id: XP_019335753.1	NCBI protein id: KYO45825.1
	Chinese alligator	*Alligator sinensis*	NCBI protein id: XP_014380389.1	NCBI protein id: XP_006037762.1	NCBI protein id: XP_006024871.1	NCBI protein id: XP_014382971.1	NCBI protein id: XP_006017656.1	NCBI protein id: XP_006028019	NCBI protein id: XP_014378344.1	NCBI protein id: XP_006038716.1	NCBI protein id: XP_006024875.1	NCBI protein id: XP_006024876.1
	Gharial	*Gavialis gangeticus*	NCBI protein id: XP_019372092.1	NCBI protein id: XP_019368768.1	NCBI protein id: XP_019363424.1	×	NCBI protein id: XP_019368938.1	NCBI protein id: XP_019371719	NCBI protein id: XP_019375775.1	×	NCBI protein id: XP_019381909.1	NCBI protein id: XP_019381807.1
Reptile	Green sea turtle	*Chelonia mydas*	NCBI protein id: XP_007071821.1	NCBI protein id: XP_007072052.1	NCBI protein id: XP_007054699.1	NCBI protein id: XP_007068343.1	NCBI protein id: XP_007072169.1	NCBI protein id: XP_007059047.1	NCBI protein id: XP_007057509.1	×	NCBI protein id: XP_007058167.1	NCBI protein id: XP_007058166.1
	tropical clawed frog	*Xenopus tropicalis*	NCBI protein id: XP_002941174.2	NCBI protein id: XP_002934323.2	NCBI protein id: NP_001136381.1	NCBI protein id: XP_002935827.3	NCBI protein id: NP_989353.1	NCBI protein id: XP_017949732	NCBI protein id: NP_001096419.1	NCBI protein id: NP_001001903.1	NCBI protein id: XP_004911281.1	NCBI protein id: XP_012827249.1
	African clawed frog	*Xenopus laevis*	NCBI protein id: NP_001081571.1	NCBI protein id: NP_001086535.1	NCBI protein id: NP_001091195.1	NCBI protein id: NP_001088636.1	NCBI protein id: NP_001085592.1	×	NCBI protein id: NP_001086418.1	NCBI protein id: CAA54672.1	NCBI protein id: ACY24354.1	×
Amphibian	Tibetan frog	*Nanorana parkeri*	NCBI protein id: XP_018423552.1	NCBI protein id: XP_018425531.1	NCBI protein id: XP_018429020.1	NCBI protein id: XP_018412794.1	NCBI protein id: XP_018429083.1	NCBI protein id: XP_018414321.1	NCBI protein id: XP_018419253.1	NCBI protein id: XP_018431451.1	NCBI protein id: XP_018418337.1	×
	Cod	*Gadus morhua*	Ensembl: ENSGMOG00000014929.1	Ensembl: ENSGMOG00000010852.1	BLASTN in Ensembl	Ensembl: ENSGMOG00000014240.1	Ensembl: ENSGMOG00000012243.1	Ensembl: ENSGMOG00000008049	Ensembl: ENSGMOG00000012748	BLASTN in Ensembl	NCBI protein id: Ensembl: ENSGMOG00000014173.1	×
	Tilapia	*Oreochromis niloticus*	NCBI protein id: XP_003449904.1	NCBI protein id: XP_005476770.1	NCBI protein id: XP_005473903.1	NCBI protein id: XP_003449312.1	NCBI protein id: XP_003438026.1	NCBI protein id: XP_003438366.2	NCBI protein id: XP_005454675.1	NCBI protein id: XP_005463895.1	NCBI protein id: XP_019208071.1	NCBI protein id: XP_003454237.1
	spotted gar	*Lepisosteus oculatus*	NCBI protein id: XP_015201493.1	NCBI protein id: XP_015195173.1	NCBI protein id: XP_006630852.1	NCBI protein id: XP_015196298.1	×	NCBI protein id: XP_015217885.1	NCBI protein id: XP_006626029.1	NCBI protein id: XP_015196219.1	NCBI protein id: XP_015200728.1	NCBI protein id: XP_006629932.1
	Medaka	*Oryzias latipes*	NCBI protein id: XP_004083528.1	NCBI protein id: XP_011484327.1	NCBI protein id: XP_004077552.1	NCBI protein id: XP_011481038.1	NCBI protein id: XP_004079352.1	NCBI protein id: XP_004077078.1	NCBI protein id: NP_001153937.1	NCBI protein id: NP_001098212.1	NCBI protein id: XP_020567248.1	NCBI protein id: XP_011490815.1
	southern platyfish	*Xiphophorus maculatus*	NCBI protein id: XP_005809620.1	NCBI protein id: XP_005796607.1	NCBI protein id: XP_005795318.1	NCBI protein id: XP_005804579.1	NCBI protein id: XP_005797215.1	NCBI protein id: XP_005814190.1	NCBI protein id: NP_001273216.1	NCBI protein id: NP_001273218.1	NCBI protein id: XP_014325970.1	NCBI protein id: XP_005807205.1
	guppy	*Poecilia reticulata*	NCBI protein id: XP_008396025.1	NCBI protein id: XP_008432237.1	NCBI protein id: XP_008428228.1	NCBI protein id: XP_008424350.1	×	NCBI protein id: XP_008427037.1	NCBI protein id: XP_008395877.1	NCBI protein id: XP_008433635.1	NCBI protein id: XP_008434034.1	NCBI protein id: XP_008409654.1
	Fugu	*Takifugu rubripes*	NCBI protein id: XP_003971725.1	NCBI protein id: XP_003978290.2	NCBI protein id: XP_003963777.1	NCBI protein id: XP_003968814.1	NCBI protein id: XP_003975688.1	Ensembl: ENSTRUG00000000770.1	NCBI protein id: XP_003971932.1	NCBI protein id: XP_011604971.1	NCBI protein id: XP_011605089.1	NCBI protein id: XP_011610687.1
Fish	Tetraodon	*Tetraodon nigroviridis*	Ensembl: ENSTNIG00000016384.1	Ensembl: ENSTNIG00000004223.1	Ensembl: ENSTNIG00000013095.1	BLASTN in Ensembl	Ensembl: ENSTNIG00000015330.1	BLASTN in Ensembl	Ensembl: ENSTNIG00000019314	Ensembl: ENSTNIG00000010137.1	BLASTN in Ensembl	Ensembl: ENSTNIG00000012367
