# Supplementary material for: Molecular evolutionary patterns of NAD+/Sirtuin aging signaling pathway across taxa
Source: PLoS One. 2017 Aug 2;12(8):e0182306. doi: 10.1371/journal.pone.0182306 (PMC5540417; doi:10.1371/journal.pone.0182306)
Supplement: S3 Table — (PDF) [file pone.0182306.s003.pdf]

S3 Table. Lineages test for postive selection by branch-site model using PAML

| Gene | Models | P | LnL | Parameters | | | | |
|---|---|---|---|---|---|---|---|---|
| ***MRPS5*** | Branch-site #1 (Mammals) | 102 | -30920.221289 | site class | 0 | 1 | 2a | 2b |
| | | | | proportion | 0.62268 | 0.34477 | 0.02095 | 0.01160 |
| | | | | background w | 0.11432 | 1.00000 | 0.11432 | 1.00000 |
| | | | | foreground w | 0.11432 | 1.00000 | 998.98326 | 998.98326 |
| | Branch-site null #1 (Mammals) | 101 | -30927.633289 | site class | 0 | 1 | 2a | 2b |
| | | | | proportion | 0.62279 | 0.34476 | 0.02089 | 0.01156 |
| | | | | background w | 0.11431 | 1.00000 | 0.11431 | 1.00000 |
| | | | | foreground w | 0.11431 | 1.00000 | 1.00000 | 1.00000 |
| | Branch-site #2 (Birds) | 102 | -30923.441079 | site class | 0 | 1 | 2a | 2b |
| | | | | proportion | 0.57814 | 0.32814 | 0.05978 | 0.03393 |
| | | | | background w | 0.11287 | 1.00000 | 0.11287 | 1.00000 |
| | | | | foreground w | 0.11287 | 1.00000 | 2.57000 | 2.57000 |
| | Branch-site null #2 (Birds) | 101 | -30927.932329 | site class | 0 | 1 | 2a | 2b |
| | | | | proportion | 0.57806 | 0.32812 | 0.05986 | 0.03395 |
| | | | | background w | 0.11307 | 1.00000 | 0.11307 | 1.00000 |
| | | | | foreground w | 0.11307 | 1.00000 | 1.00000 | 1.00000 |
| | Branch-site #3 (Birds/Reptiles) | 102 | -30925.394879 | site class | 0 | 1 | 2a | 2b |
| | | | | proportion | 0.63803 | 0.36197 | 0.00000 | 0.00000 |
| | | | | background w | 0.11418 | 1.00000 | 0.11418 | 1.00000 |
| | | | | foreground w | 0.11418 | 1.00000 | 3.54000 | 3.54000 |
| | Branch-site null #3 (Birds/Reptiles) | 101 | -30927.365679 | site class | 0 | 1 | 2a | 2b |
| | | | | proportion | 0.63605 | 0.36395 | 0.00000 | 0.00000 |
| | | | | background w | 0.11422 | 1.00000 | 0.11422 | 1.00000 |
| | | | | foreground w | 0.11422 | 1.00000 | 1.00000 | 1.00000 |

| | | | | | | | | |
|---|---|---|---|---|---|---|---|---|
| | Branch-site #4 (Amphibian) | 102 | -30925.394879 | site class | 0 | 1 | 2a | 2b |
| | | | | proportion | 0.63803 | 0.36197 | 0.00000 | 0.00000 |
| | | | | background w | 0.11418 | 1.00000 | 0.11418 | 1.00000 |
| | | | | foreground w | 0.11418 | 1.00000 | 1.00000 | 1.00000 |
| | Branch-site null #4 (Amphibian) | 101 | -30931.762979 | site class | 0 | 1 | 2a | 2b |
| | | | | proportion | 0.63816 | 0.36184 | 0.00000 | 0.00000 |
| | | | | background w | 0.11412 | 1.00000 | 0.11412 | 1.00000 |
| | | | | foreground w | 0.11412 | 1.00000 | 1.00000 | 1.00000 |
| | Branch-site #5 (Fish) | 102 | -30925.394879 | site class | 0 | 1 | 2a | 2b |
| | | | | proportion | 0.63803 | 0.36197 | 0.00000 | 0.00000 |
| | | | | background w | 0.11418 | 1.00000 | 0.11418 | 1.00000 |
| | | | | foreground w | 0.11418 | 1.00000 | 1.00000 | 1.00000 |
| | Branch-site null #5 (Fish) | 101 | -30930.444079 | site class | 0 | 1 | 2a | 2b |
| | | | | proportion | 0.63812 | 0.36188 | 0.00000 | 0.00000 |
| | | | | background w | 0.11420 | 1.00000 | 0.11420 | 1.00000 |
| | | | | foreground w | 0.11420 | 1.00000 | 1.00000 | 1.00000 |
| ***FOXO3a*** | Branch-site #1 (Mammals) | 102 | -38935.573507 | site class | 0 | 1 | 2a | 2b |
| | | | | proportion | 0.81509 | 0.18491 | 0.00000 | 0.00000 |
| | | | | background w | 0.09022 | 1.00000 | 0.09022 | 1.00000 |
| | | | | foreground w | 0.09022 | 1.00000 | 2.69405 | 2.69405 |
| | Branch-site null #1 (Mammals) | 101 | -38939.122607 | site class | 0 | 1 | 2a | 2b |
| | | | | proportion | 0.81501 | 0.18499 | 0.00000 | 0.00000 |
| | | | | background w | 0.09023 | 1.00000 | 0.09023 | 1.00000 |
| | | | | foreground w | 0.09023 | 1.00000 | 1.00000 | 1.00000 |

| | | | | | | | |
|---|---|---|---|---|---|---|---|
| Branch-site #2 (Birds) | 102 | -38935.573518 | site class | 0 | 1 | 2a | 2b |
| | | | proportion | 0.81510 | 0.18490 | 0.00000 | 0.00000 |
| | | | background w | 0.09022 | 1.00000 | 0.09022 | 1.00000 |
| | | | foreground w | 0.09022 | 1.00000 | 1.00000 | 1.00000 |
| Branch-site null #2 (Birds) | 101 | -38938.600618 | site class | 0 | 1 | 2a | 2b |
| | | | proportion | 0.81502 | 0.18498 | 0.00000 | 0.00000 |
| | | | background w | 0.09029 | 1.00000 | 0.09029 | 1.00000 |
| | | | foreground w | 0.09029 | 1.00000 | 1.00000 | 1.00000 |
| Branch-site #3 (Birds/Reptiles) | 102 | -38933.906557 | site class | 0 | 1 | 2a | 2b |
| | | | proportion | 0.81266 | 0.18401 | 0.00272 | 0.00062 |
| | | | background w | 0.09004 | 1.00000 | 0.09004 | 1.00000 |
| | | | foreground w | 0.09004 | 1.00000 | 27.74363 | 27.74363 |
| Branch-site null #3 (Birds/Reptiles) | 101 | -38937.013557 | site class | 0 | 1 | 2a | 2b |
| | | | proportion | 0.81252 | 0.18382 | 0.00285 | 0.00082 |
| | | | background w | 0.09003 | 1.00000 | 0.09003 | 1.00000 |
| | | | foreground w | 0.09003 | 1.00000 | 1.00000 | 1.00000 |
| Branch-site #4 (Amphibian) | 102 | -38922.535789 | site class | 0 | 1 | 2a | 2b |
| | | | proportion | 0.78923 | 0.17906 | 0.02584 | 0.00586 |
| | | | background w | 0.08918 | 1.00000 | 0.08918 | 1.00000 |
| | | | foreground w | 0.08918 | 1.00000 | 998.94735 | 998.94735 |
| Branch-site null #4 (Amphibian) | 101 | -38933.498589 | site class | 0 | 1 | 2a | 2b |
| | | | proportion | 0.78925 | 0.17912 | 0.02579 | 0.00583 |
| | | | background w | 0.08924 | 1.00000 | 0.08924 | 1.00000 |
| | | | foreground w | 0.08924 | 1.00000 | 1.00000 | 1.00000 |

| | | | | | | | | |
|---|---|---|---|---|---|---|---|---|
| | Branch-site #5 (Fish) | 102 | -38927.422004 | site class | 0 | 1 | 2a | 2b |
| | | | | proportion | 0.78869 | 0.17774 | 0.02740 | 0.00617 |
| | | | | background w | 0.08951 | 1.00000 | 0.08951 | 1.00000 |
| | | | | foreground w | 0.08951 | 1.00000 | 20.50811 | 20.50811 |
| | Branch-site null #5 (Fish) | 101 | -38935.042454 | site class | 0 | 1 | 2a | 2b |
| | | | | proportion | 0.77654 | 0.17634 | 0.03062 | 0.01650 |
| | | | | background w | 0.08952 | 1.00000 | 0.08952 | 1.00000 |
| | | | | foreground w | 0.08952 | 1.00000 | 1.00000 | 1.00000 |
| ***PARP1*** | Branch-site #1 (Mammals) | 102 | -61077.131990 | site class | 0 | 1 | 2a | 2b |
| | | | | proportion | 0.81034 | 0.18460 | 0.00412 | 0.00094 |
| | | | | background w | 0.07227 | 1.00000 | 0.07227 | 1.00000 |
| | | | | foreground w | 0.07227 | 1.00000 | 251.03911 | 251.03911 |
| | Branch-site null #1 (Mammals) | 101 | -61080.756293 | site class | 0 | 1 | 2a | 2b |
| | | | | proportion | 0.80447 | 0.18345 | 0.00984 | 0.00224 |
| | | | | background w | 0.07228 | 1.00000 | 0.07228 | 1.00000 |
| | | | | foreground w | 0.07228 | 1.00000 | 1.00000 | 1.00000 |
| | Branch-site #2 (Birds) | 102 | -61076.797603 | site class | 0 | 1 | 2a | 2b |
| | | | | proportion | 0.80779 | 0.18392 | 0.00675 | 0.00154 |
| | | | | background w | 0.07210 | 1.00000 | 0.07210 | 1.00000 |
| | | | | foreground w | 0.07210 | 1.00000 | 6.47247 | 6.47247 |
| | Branch-site null #2 (Birds) | 101 | -61079.205043 | site class | 0 | 1 | 2a | 2b |
| | | | | proportion | 0.79750 | 0.18187 | 0.01680 | 0.00383 |
| | | | | background w | 0.07209 | 1.00000 | 0.07209 | 1.00000 |
| | | | | foreground w | 0.07209 | 1.00000 | 1.00000 | 1.00000 |

| | | | | | | | |
|---|---|---|---|---|---|---|---|
| Branch-site #3 | 102 | -61079.954352 | site class | 0 | 1 | 2a | 2b |
| (Birds/Reptiles) | | | proportion | 0.81157 | 0.18514 | 0.00268 | 0.00061 |
| | | | background w | 0.07229 | 1.00000 | 0.07229 | 1.00000 |
| | | | foreground w | 0.07229 | 1.00000 | 12.82835 | 12.82835 |
| Branch-site null #3 | 101 | -61082.867902 | site class | 0 | 1 | 2a | 2b |
| (Birds/Reptiles) | | | proportion | 0.82763 | 0.19009 | 0.00159 | 0.01931 |
| | | | background w | 0.07216 | 1.00000 | 0.07216 | 1.00000 |
| | | | foreground w | 0.07216 | 1.00000 | 1.00000 | 1.00000 |
| Branch-site #4 | 102 | -61059.880116 | site class | 0 | 1 | 2a | 2b |
| (Amphibian) | | | proportion | 0.79593 | 0.17831 | 0.02105 | 0.00471 |
| | | | background w | 0.07199 | 1.00000 | 0.07199 | 1.00000 |
| | | | foreground w | 0.07199 | 1.00000 | 25.21602 | 25.21602 |
| Branch-site null #4 | 101 | -61070.660566 | site class | 0 | 1 | 2a | 2b |
| (Amphibian) | | | proportion | 0.75145 | 0.17070 | 0.06344 | 0.01441 |
| | | | background w | 0.07148 | 1.00000 | 0.07148 | 1.00000 |
| | | | foreground w | 0.07148 | 1.00000 | 1.00000 | 1.00000 |
| Branch-site #5 | 102 | -61054.508022 | site class | 0 | 1 | 2a | 2b |
| (Fish) | | | proportion | 0.78332 | 0.17601 | 0.03321 | 0.00746 |
| | | | background w | 0.07175 | 1.00000 | 0.07175 | 1.00000 |
| | | | foreground w | 0.07175 | 1.00000 | 16.89611 | 16.89611 |
| Branch-site null #5 | 101 | -61066.746233 | site class | 0 | 1 | 2a | 2b |
| (Fish) | | | proportion | 0.73822 | 0.16845 | 0.07599 | 0.01734 |
| | | | background w | 0.07115 | 1.00000 | 0.07115 | 1.00000 |
| | | | foreground w | 0.07115 | 1.00000 | 1.00000 | 1.00000 |

| | | | | | | | | |
|---|---|---|---|---|---|---|---|---|
| ***PARP2*** | Branch-site #1 (Mammals) | 78 | -38883.371059 | site class | 0 | 1 | 2a | 2b |
| | | | | proportion | 0.64832 | 0.33328 | 0.01215 | 0.00625 |
| | | | | background w | 0.10409 | 1.00000 | 0.10409 | 1.00000 |
| | | | | foreground w | 0.10409 | 1.00000 | 251.57836 | 251.57836 |
| | Branch-site null #1 (Mammals) | 77 | -38889.059433 | site class | 0 | 1 | 2a | 2b |
| | | | | proportion | 0.65075 | 0.34041 | 0.00580 | 0.00303 |
| | | | | background w | 0.10375 | 1.00000 | 0.10375 | 1.00000 |
| | | | | foreground w | 0.10375 | 1.00000 | 1.00000 | 1.00000 |
| | Branch-site (Reptiles) | 78 | -38884.369940 | site class | 0 | 1 | 2a | 2b |
| | | | | proportion | 0.64401 | 0.33559 | 0.01341 | 0.00699 |
| | | | | background w | 0.10347 | 1.00000 | 0.10347 | 1.00000 |
| | | | | foreground w | 0.10347 | 1.00000 | 171.74271 | 171.74271 |
| | Branch-site null (Reptiles) | 77 | -38888.771709 | site class | 0 | 1 | 2a | 2b |
| | | | | proportion | 0.64378 | 0.33662 | 0.01287 | 0.00673 |
| | | | | background w | 0.10353 | 1.00000 | 0.10353 | 1.00000 |
| | | | | foreground w | 0.10353 | 1.00000 | 1.00000 | 1.00000 |
| | Branch-site #4 (Amphibian) | 78 | -38873.897205 | site class | 0 | 1 | 2a | 2b |
| | | | | proportion | 0.62476 | 0.30747 | 0.04542 | 0.02235 |
| | | | | background w | 0.10445 | 1.00000 | 0.10445 | 1.00000 |
| | | | | foreground w | 0.10445 | 1.00000 | 42.33460 | 42.33460 |
| | Branch-site null #4 (Amphibian) | 77 | -38884.883940 | site class | 0 | 1 | 2a | 2b |
| | | | | proportion | 0.61315 | 0.31396 | 0.04821 | 0.02468 |
| | | | | background w | 0.10301 | 1.00000 | 0.10301 | 1.00000 |
| | | | | foreground w | 0.10301 | 1.00000 | 1.00000 | 1.00000 |

| | | | | | | | | |
|---|---|---|---|---|---|---|---|---|
| | Branch-site #5 (Fish) | 78 | -38861.798644 | site class | 0 | 1 | 2a | 2b |
| | | | | proportion | 0.60523 | 0.31870 | 0.04983 | 0.02624 |
| | | | | background w | 0.10163 | 1.00000 | 0.10163 | 1.00000 |
| | | | | foreground w | 0.10163 | 1.00000 | 188.08457 | 188.08457 |
| | Branch-site null #5 (Fish) | 77 | -38880.530188 | site class | 0 | 1 | 2a | 2b |
| | | | | proportion | 0.56678 | 0.29712 | 0.08929 | 0.04681 |
| | | | | background w | 0.10113 | 1.00000 | 0.10113 | 1.00000 |
| | | | | foreground w | 0.10113 | 1.00000 | 1.00000 | 1.00000 |
| ***PPARGC1A*** | Branch-site #1 (Mammals) | 100 | -43108.340734 | site class | 0 | 1 | 2a | 2b |
| | | | | proportion | 0.66562 | 0.33438 | 0.00000 | 0.00000 |
| | | | | background w | 0.10326 | 1.00000 | 0.10326 | 1.00000 |
| | | | | foreground w | 0.10326 | 1.00000 | 1.00000 | 1.00000 |
| | Branch-site null #1 (Mammals) | 99 | -43111.422284 | site class | 0 | 1 | 2a | 2b |
| | | | | proportion | 0.66551 | 0.33449 | 0.00000 | 0.00000 |
| | | | | background w | 0.10327 | 1.00000 | 0.10327 | 1.00000 |
| | | | | foreground w | 0.10327 | 1.00000 | 1.00000 | 1.00000 |
| | Branch-site #2 (Birds) | 100 | -43108.340734 | site class | 0 | 1 | 2a | 2b |
| | | | | proportion | 0.66562 | 0.33438 | 0.00000 | 0.00000 |
| | | | | background w | 0.10326 | 1.00000 | 0.10326 | 1.00000 |
| | | | | foreground w | 0.10326 | 1.00000 | 1.00000 | 1.00000 |
| | Branch-site null #2 (Birds) | 99 | -43110.045834 | site class | 0 | 1 | 2a | 2b |
| | | | | proportion | 0.66430 | 0.33570 | 0.00000 | 0.00000 |
| | | | | background w | 0.10128 | 1.00000 | 0.10128 | 1.00000 |
| | | | | foreground w | 0.10128 | 1.00000 | 1.00000 | 1.00000 |

| | | | | | | | |
|---|---|---|---|---|---|---|---|
| Branch-site #3 | 100 | -43108.340736 | site class | 0 | 1 | 2a | 2b |
| (Birds/Reptiles) | | | proportion | 0.66562 | 0.33438 | 0.00000 | 0.00000 |
| | | | background w | 0.10326 | 1.00000 | 0.10326 | 1.00000 |
| | | | foreground w | 0.10326 | 1.00000 | 1.00000 | 1.00000 |
| Branch-site null #3 | 99 | -43111.731236 | site class | 0 | 1 | 2a | 2b |
| (Birds/Reptiles) | | | proportion | 0.65541 | 0.34459 | 0.00000 | 0.00000 |
| | | | background w | 0.10208 | 1.00000 | 0.10208 | 1.00000 |
| | | | foreground w | 0.10208 | 1.00000 | 1.00000 | 1.00000 |
| Branch-site #4 | 100 | -43108.340734 | site class | 0 | 1 | 2a | 2b |
| (Amphibian) | | | proportion | 0.66562 | 0.33438 | 0.00000 | 0.00000 |
| | | | background w | 0.10326 | 1.00000 | 0.10326 | 1.00000 |
| | | | foreground w | 0.10326 | 1.00000 | 1.00000 | 1.00000 |
| Branch-site null #4 | 99 | -43111.662184 | site class | 0 | 1 | 2a | 2b |
| (Amphibian) | | | proportion | 0.64202 | 0.35798 | 0.00000 | 0.00000 |
| | | | background w | 0.10329 | 1.00000 | 0.10329 | 1.00000 |
| | | | foreground w | 0.10329 | 1.00000 | 1.00000 | 1.00000 |
| Branch-site #5 | 100 | -43107.047250 | site class | 0 | 1 | 2a | 2b |
| (Fish) | | | proportion | 0.65594 | 0.32580 | 0.01220 | 0.00606 |
| | | | background w | 0.10269 | 1.00000 | 0.10269 | 1.00000 |
| | | | foreground w | 0.10269 | 1.00000 | 4.18203 | 4.18203 |
| Branch-site null #5 | 99 | -43109.5773 | site class | 0 | 1 | 2a | 2b |
| (Fish) | | | proportion | 0.65472 | 0.32572 | 0.01295 | 0.00661 |
| | | | background w | 0.10254 | 1.00000 | 0.10254 | 1.00000 |
| | | | foreground w | 0.10254 | 1.00000 | 1.00000 | 1.00000 |

| | | | | | | | | |
|---|---|---|---|---|---|---|---|---|
| ***SIRT1*** | Branch-site #1 | 100 | -43627.353497 | site class | 0 | 1 | 2a | 2b |
| | (Mammals) | | | proportion | 0.54289 | 0.45711 | 0.00000 | 0.00000 |
| | | | | background w | 0.05190 | 1.00000 | 0.05190 | 1.00000 |
| | | | | foreground w | 0.05190 | 1.00000 | 1.00000 | 1.00000 |
| | Branch-site null #1 | 99 | -43629.399897 | site class | 0 | 1 | 2a | 2b |
| | (Mammals) | | | proportion | 0.54291 | 0.45709 | 0.00000 | 0.00000 |
| | | | | background w | 0.05187 | 1.00000 | 0.05187 | 1.00000 |
| | | | | foreground w | 0.05187 | 1.00000 | 1.00000 | 1.00000 |
| | Branch-site #2 | 100 | -43627.353505 | site class | 0 | 1 | 2a | 2b |
| | (Birds) | | | proportion | 0.54289 | 0.45711 | 0.00000 | 0.00000 |
| | | | | background w | 0.05190 | 1.00000 | 0.05190 | 1.00000 |
| | | | | foreground w | 0.05190 | 1.00000 | 1.00000 | 1.00000 |
| | Branch-site null #2 | 99 | -43629.745105 | site class | 0 | 1 | 2a | 2b |
| | (Birds) | | | proportion | 0.54186 | 0.45814 | 0.00000 | 0.00000 |
| | | | | background w | 0.05078 | 1.00000 | 0.05078 | 1.00000 |
| | | | | foreground w | 0.05078 | 1.00000 | 1.00000 | 1.00000 |
| | Branch-site #3 | 100 | -43627.353499 | site class | 0 | 1 | 2a | 2b |
| | (Birds/Reptiles) | | | proportion | 0.54289 | 0.45711 | 0.00000 | 0.00000 |
| | | | | background w | 0.05190 | 1.00000 | 0.05190 | 1.00000 |
| | | | | foreground w | 0.05190 | 1.00000 | 1.00000 | 1.00000 |
| | Branch-site null #3 | 99 | -43629.854099 | site class | 0 | 1 | 2a | 2b |
| | (Birds/Reptiles) | | | proportion | 0.53158 | 0.46842 | 0.00000 | 0.00000 |
| | | | | background w | 0.05191 | 1.00000 | 0.05191 | 1.00000 |
| | | | | foreground w | 0.05191 | 1.00000 | 1.00000 | 1.00000 |

| | | | | | | | | |
|---|---|---|---|---|---|---|---|---|
| | Branch-site #4 (Amphibian) | 100 | -43625.175152 | site class | 0 | 1 | 2a | 2b |
| | | | | proportion | 0.52422 | 0.44249 | 0.01805 | 0.01524 |
| | | | | background w | 0.05098 | 1.00000 | 0.05098 | 1.00000 |
| | | | | foreground w | 0.05098 | 1.00000 | 1.00000 | 1.00000 |
| | Branch-site null #4 (Amphibian) | 99 | -43629.721902 | site class | 0 | 1 | 2a | 2b |
| | | | | proportion | 0.52834 | 0.44250 | 0.01702 | 0.01214 |
| | | | | background w | 0.05099 | 1.00000 | 0.05099 | 1.00000 |
| | | | | foreground w | 0.05099 | 1.00000 | 1.00000 | 1.00000 |
| | Branch-site #5 (Fish) | 100 | -43621.181594 | site class | 0 | 1 | 2a | 2b |
| | | | | proportion | 0.52954 | 0.44190 | 0.01557 | 0.01299 |
| | | | | background w | 0.05148 | 1.00000 | 0.05148 | 1.00000 |
| | | | | foreground w | 0.05148 | 1.00000 | 998.99613 | 998.99613 |
| | Branch-site null #5 (Fish) | 99 | -43623.749044 | site class | 0 | 1 | 2a | 2b |
| | | | | proportion | 0.52962 | 0.44198 | 0.01543 | 0.01297 |
| | | | | background w | 0.05140 | 1.00000 | 0.05140 | 1.00000 |
| | | | | foreground w | 0.05140 | 1.00000 | 1.00000 | 1.00000 |
| ***SIRT2*** | Branch-site #1 (Mammals) | 86 | -26945.165813 | site class | 0 | 1 | 2a | 2b |
| | | | | proportion | 0.68166 | 0.28773 | 0.02152 | 0.00908 |
| | | | | background w | 0.08111 | 1.00000 | 0.08111 | 1.00000 |
| | | | | foreground w | 0.08111 | 1.00000 | 28.52968 | 28.52968 |
| | Branch-site null #1 (Mammals) | 85 | -26948.711963 | ite class | 0 | 1 | 2a | 2b |
| | | | | proportion | 0.68178 | 0.28775 | 0.02140 | 0.00906 |
| | | | | background w | 0.08129 | 1.00000 | 0.08129 | 1.00000 |
| | | | | foreground w | 0.08129 | 1.00000 | 1.00000 | 1.00000 |

| | | | | | | | |
|---|---|---|---|---|---|---|---|
| Branch-site #2 (Birds) | 86 | -26950.612331 | site class | 0 | 1 | 2a | 2b |
| | | | proportion | 0.69067 | 0.29731 | 0.00840 | 0.00362 |
| | | | background w | 0.08122 | 1.00000 | 0.08122 | 1.00000 |
| | | | foreground w | 0.08122 | 1.00000 | 157.14276 | 157.14276 |
| Branch-site null #2 (Birds) | 85 | -26954.110481 | site class | 0 | 1 | 2a | 2b |
| | | | proportion | 0.69265 | 0.29850 | 0.00638 | 0.00247 |
| | | | background w | 0.08069 | 1.00000 | 0.08069 | 1.00000 |
| | | | foreground w | 0.08069 | 1.00000 | 1.00000 | 1.00000 |
| Branch-site #3 (Birds/Reptiles) | 86 | -26942.816770 | site class | 0 | 1 | 2a | 2b |
| | | | proportion | 0.68439 | 0.29303 | 0.01581 | 0.00677 |
| | | | background w | 0.08074 | 1.00000 | 0.08074 | 1.00000 |
| | | | foreground w | 0.08074 | 1.00000 | 172.82907 | 172.82907 |
| Branch-site null #3 (Birds/Reptiles) | 85 | -26950.41352 | site class | 0 | 1 | 2a | 2b |
| | | | proportion | 0.68226 | 0.29210 | 0.01789 | 0.00685 |
| | | | background w | 0.08075 | 1.00000 | 0.08075 | 1.00000 |
| | | | foreground w | 0.08075 | 1.00000 | 1.00000 | 1.00000 |
| Branch-site #4 (Amphibian) | 86 | -26951.693147 | site class | 0 | 1 | 2a | 2b |
| | | | proportion | 0.69126 | 0.29805 | 0.00747 | 0.00322 |
| | | | background w | 0.08114 | 1.00000 | 0.08114 | 1.00000 |
| | | | foreground w | 0.08114 | 1.00000 | 4.88201 | 4.88201 |
| Branch-site null #4 (Amphibian) | 85 | -26953.701797 | site class | 0 | 1 | 2a | 2b |
| | | | proportion | 0.69114 | 0.29710 | 0.00762 | 0.00332 |
| | | | background w | 0.08109 | 1.00000 | 0.08109 | 1.00000 |
| | | | foreground w | 0.08109 | 1.00000 | 1.00000 | 1.00000 |

| | | | | | | | | |
|---|---|---|---|---|---|---|---|---|
| | Branch-site #5 (Fish) | 86 | -26934.971329 | site class | 0 | 1 | 2a | 2b |
| | | | | proportion | 0.65809 | 0.27556 | 0.04677 | 0.01958 |
| | | | | background w | 0.08100 | 1.00000 | 0.08100 | 1.00000 |
| | | | | foreground w | 0.08100 | 1.00000 | 999.00000 | 999.00000 |
| | Branch-site null #5 (Fish) | 85 | -26949.019929 | site class | 0 | 1 | 2a | 2b |
| | | | | proportion | 0.65817 | 0.27565 | 0.04668 | 0.01950 |
| | | | | background w | 0.08114 | 1.00000 | 0.08114 | 1.00000 |
| | | | | foreground w | 0.08114 | 1.00000 | 1.00000 | 1.00000 |
| ***SOD3*** | Branch-site #1 (Mammals) | 96 | -19858.187943 | site class | 0 | 1 | 2a | 2b |
| | | | | proportion | 0.43924 | 0.49242 | 0.03222 | 0.03612 |
| | | | | background w | 0.12728 | 1.00000 | 0.12728 | 1.00000 |
| | | | | foreground w | 0.12728 | 1.00000 | 362.12813 | 362.12813 |
| | Branch-site null #1 (Mammals) | 95 | -19861.204193 | site class | 0 | 1 | 2a | 2b |
| | | | | proportion | 0.43812 | 0.49234 | 0.03229 | 0.03625 |
| | | | | background w | 0.12908 | 1.00000 | 0.12908 | 1.00000 |
| | | | | foreground w | 0.12908 | 1.00000 | 1.00000 | 1.00000 |
| | Branch-site #2 (Birds) | 96 | -19867.546063 | site class | 0 | 1 | 2a | 2b |
| | | | | proportion | 0.46905 | 0.53095 | 0.00000 | 0.00000 |
| | | | | background w | 0.12957 | 1.00000 | 0.12957 | 1.00000 |
| | | | | foreground w | 0.12957 | 1.00000 | 1.00000 | 1.00000 |
| | Branch-site null #2 (Birds) | 95 | -19870.780313 | site class | 0 | 1 | 2a | 2b |
| | | | | proportion | 0.46903 | 0.53097 | 0.00000 | 0.00000 |
| | | | | background w | 0.12893 | 1.00000 | 0.12893 | 1.00000 |
| | | | | foreground w | 0.12893 | 1.00000 | 1.00000 | 1.00000 |

| | | | | | | | |
|---|---|---|---|---|---|---|---|
| Branch-site #3 (Birds/Reptiles) | 96 | -19866.642871 | site class | 0 | 1 | 2a | 2b |
| | | | proportion | 0.46640 | 0.51719 | 0.00778 | 0.00863 |
| | | | background w | 0.12998 | 1.00000 | 0.12998 | 1.00000 |
| | | | foreground w | 0.12998 | 1.00000 | 9.06156 | 9.06156 |
| Branch-site null #3 (Birds/Reptiles) | 95 | -19870.692621 | site class | 0 | 1 | 2a | 2b |
| | | | proportion | 0.46622 | 0.51717 | 0.00789 | 0.00872 |
| | | | background w | 0.12876 | 1.00000 | 0.12876 | 1.00000 |
| | | | foreground w | 0.12876 | 1.00000 | 1.00000 | 1.00000 |
| Branch-site #4 (Amphibian) | 96 | -19866.748346 | site class | 0 | 1 | 2a | 2b |
| | | | proportion | 0.45924 | 0.51347 | 0.01289 | 0.01441 |
| | | | background w | 0.13002 | 1.00000 | 0.13002 | 1.00000 |
| | | | foreground w | 0.13002 | 1.00000 | 10.35377 | 10.35377 |
| Branch-site null #4 (Amphibian) | 95 | -19871.298796 | site class | 0 | 1 | 2a | 2b |
| | | | proportion | 0.45012 | 0.51107 | 0.01391 | 0.02491 |
| | | | background w | 0.13312 | 1.00000 | 0.13312 | 1.00000 |
| | | | foreground w | 0.13312 | 1.00000 | 1.00000 | 1.00000 |
| Branch-site #5 (Fish) | 96 | -19862.561954 | site class | 0 | 1 | 2a | 2b |
| | | | proportion | 0.44467 | 0.46020 | 0.04675 | 0.04838 |
| | | | background w | 0.13422 | 1.00000 | 0.13422 | 1.00000 |
| | | | foreground w | 0.13422 | 1.00000 | 999.00000 | 999.00000 |
| Branch-site null #5 (Fish) | 95 | -19868.661404 | site class | 0 | 1 | 2a | 2b |
| | | | proportion | 0.44677 | 0.46119 | 0.04375 | 0.04829 |
| | | | background w | 0.13421 | 1.00000 | 0.13421 | 1.00000 |
| | | | foreground w | 0.13421 | 1.00000 | 1.00000 | 1.00000 |

| | | | | | | | | |
|---|---|---|---|---|---|---|---|---|
| ***TP53*** | Branch-site #1 (Mammals) | 78 | -27706.106253 | site class | 0 | 1 | 2a | 2b |
| | | | | proportion | 0.50565 | 0.45732 | 0.01944 | 0.01758 |
| | | | | background w | 0.09401 | 1.00000 | 0.09401 | 1.00000 |
| | | | | foreground w | 0.09401 | 1.00000 | 834.09156 | 834.09156 |
| | Branch-site null #1 (Mammals) | 77 | -27710.564903 | site class | 0 | 1 | 2a | 2b |
| | | | | proportion | 0.50697 | 0.45835 | 0.01909 | 0.01558 |
| | | | | background w | 0.09381 | 1.00000 | 0.09381 | 1.00000 |
| | | | | foreground w | 0.09381 | 1.00000 | 1.00000 | 1.00000 |
| | Branch-site #2 (Birds) | 78 | -27701.790550 | site class | 0 | 1 | 2a | 2b |
| | | | | proportion | 0.49185 | 0.45047 | 0.03011 | 0.02757 |
| | | | | background w | 0.09304 | 1.00000 | 0.09304 | 1.00000 |
| | | | | foreground w | 0.09304 | 1.00000 | 92.55009 | 92.55009 |
| | Branch-site null #2 (Birds) | 77 | -27703.735200 | site class | 0 | 1 | 2a | 2b |
| | | | | proportion | 0.50287 | 0.45050 | 0.02006 | 0.02657 |
| | | | | background w | 0.09321 | 1.00000 | 0.09321 | 1.00000 |
| | | | | foreground w | 0.09321 | 1.00000 | 1.00000 | 1.00000 |
| | Branch-site #3 (Birds/Reptiles) | 78 | -27708.436755 | site class | 0 | 1 | 2a | 2b |
| | | | | proportion | 0.52037 | 0.47963 | 0.00000 | 0.00000 |
| | | | | background w | 0.09401 | 1.00000 | 0.09401 | 1.00000 |
| | | | | foreground w | 0.09401 | 1.00000 | 1.00000 | 1.00000 |
| | Branch-site null #3 (Birds/Reptiles) | 77 | -27710.982555 | site class | 0 | 1 | 2a | 2b |
| | | | | proportion | 0.52139 | 0.47861 | 0.00000 | 0.00000 |
| | | | | background w | 0.09403 | 1.00000 | 0.09403 | 1.00000 |
| | | | | foreground w | 0.09403 | 1.00000 | 1.00000 | 1.00000 |

| | | | | | | | | |
|---|---|---|---|---|---|---|---|---|
| | Branch-site #4 (Amphibian) | 78 | -27708.436768 | site class | 0 | 1 | 2a | 2b |
| | | | | proportion | 0.49375 | 0.45509 | 0.02663 | 0.02454 |
| | | | | background w | 0.09401 | 1.00000 | 0.09401 | 1.00000 |
| | | | | foreground w | 0.09401 | 1.00000 | 1.17904 | 1.17904 |
| | Branch-site null #4 (Amphibian) | 77 | -27713.482968 | site class | 0 | 1 | 2a | 2b |
| | | | | proportion | 0.49380 | 0.45604 | 0.02588 | 0.02429 |
| | | | | background w | 0.09398 | 1.00000 | 0.09398 | 1.00000 |
| | | | | foreground w | 0.09398 | 1.00000 | 1.00000 | 1.00000 |
| | Branch-site #5 (Fish) | 78 | -27700.442422 | site class | 0 | 1 | 2a | 2b |
| | | | | proportion | 0.48957 | 0.45474 | 0.02887 | 0.02681 |
| | | | | background w | 0.09196 | 1.00000 | 0.09196 | 1.00000 |
| | | | | foreground w | 0.09196 | 1.00000 | 35.63319 | 35.63319 |
| | Branch-site null #5 (Fish) | 77 | -27710.534172 | site class | 0 | 1 | 2a | 2b |
| | | | | proportion | 0.48962 | 0.45489 | 0.02879 | 0.02669 |
| | | | | background w | 0.09290 | 1.00000 | 0.09290 | 1.00000 |
| | | | | foreground w | 0.09290 | 1.00000 | 1.00000 | 1.00000 |
| ***SIRT6*** | Branch-site #1 (Mammals) | 94 | -26980.481368 | site class | 0 | 1 | 2a | 2b |
| | | | | proportion | 0.65686 | 0.31285 | 0.02052 | 0.00977 |
| | | | | background w | 0.09346 | 1.00000 | 0.09346 | 1.00000 |
| | | | | foreground w | 0.09346 | 1.00000 | 8.79210 | 8.79210 |
| | Branch-site null #1 (Mammals) | 93 | -26985.218468 | site class | 0 | 1 | 2a | 2b |
| | | | | proportion | 0.65083 | 0.31282 | 0.02560 | 0.01075 |
| | | | | background w | 0.09347 | 1.00000 | 0.09347 | 1.00000 |
| | | | | foreground w | 0.09347 | 1.00000 | 1.00000 | 1.00000 |

| | | | | | | | |
|---|---|---|---|---|---|---|---|
| Branch-site #2 | 94 | -26984.548942 | site class | 0 | 1 | 2a | 2b |
| (Birds) | | | proportion | 0.67547 | 0.32453 | 0.00000 | 0.00000 |
| | | | background w | 0.09370 | 1.00000 | 0.09370 | 1.00000 |
| | | | foreground w | 0.09370 | 1.00000 | 1.00000 | 1.00000 |
| Branch-site null #2 | 93 | -26989.034142 | site class | 0 | 1 | 2a | 2b |
| (Birds) | | | proportion | 0.66520 | 0.33480 | 0.00000 | 0.00000 |
| | | | background w | 0.09378 | 1.00000 | 0.09378 | 1.00000 |
| | | | foreground w | 0.09378 | 1.00000 | 1.00000 | 1.00000 |
| Branch-site #3 | 94 | -26984.548942 | site class | 0 | 1 | 2a | 2b |
| (Birds/Reptiles) | | | proportion | 0.67547 | 0.32453 | 0.00000 | 0.00000 |
| | | | background w | 0.09370 | 1.00000 | 0.09370 | 1.00000 |
| | | | foreground w | 0.09370 | 1.00000 | 1.00000 | 1.00000 |
| Branch-site null #3 | 93 | -26988.060742 | site class | 0 | 1 | 2a | 2b |
| (Birds/Reptiles) | | | proportion | 0.67425 | 0.32575 | 0.00000 | 0.00000 |
| | | | background w | 0.09371 | 1.00000 | 0.09371 | 1.00000 |
| | | | foreground w | 0.09371 | 1.00000 | 1.00000 | 1.00000 |
| Branch-site #4 | 94 | -26976.180244 | site class | 0 | 1 | 2a | 2b |
| (Amphibian) | | | proportion | 0.65047 | 0.30576 | 0.02978 | 0.01400 |
| | | | background w | 0.09333 | 1.00000 | 0.09333 | 1.00000 |
| | | | foreground w | 0.09333 | 1.00000 | 500.19230 | 500.19230 |
| Branch-site null #4 | 93 | -26980.499194 | site class | 0 | 1 | 2a | 2b |
| (Amphibian) | | | proportion | 0.53027 | 0.28948 | 0.03775 | 0.01593 |
| | | | background w | 0.09332 | 1.00000 | 0.09332 | 1.00000 |
| | | | foreground w | 0.09332 | 1.00000 | 1.00000 | 1.00000 |

| | | | | | | | |
|---|---|---|---|---|---|---|---|
| Branch-site #5 (Fish) | 94 | -26957.679678 | site class | 0 | 1 | 2a | 2b |
| | | | proportion | 0.60519 | 0.29354 | 0.06820 | 0.03308 |
| | | | background w | 0.09230 | 1.00000 | 0.09230 | 1.00000 |
| | | | foreground w | 0.09230 | 1.00000 | 999.00000 | 999.00000 |
| Branch-site null #5 (Fish) | 93 | -26967.210578 | site class | 0 | 1 | 2a | 2b |
| | | | proportion | 0.61550 | 0.30323 | 0.06016 | 0.02112 |
| | | | background w | 0.09121 | 1.00000 | 0.09121 | 1.00000 |
| | | | foreground w | 0.09121 | 1.00000 | 1.00000 | 1.00000 |
