# Supplementary material for: Molecular evolutionary patterns of NAD+/Sirtuin aging signaling pathway across taxa
Source: PLoS One. 2017 Aug 2;12(8):e0182306. doi: 10.1371/journal.pone.0182306 (PMC5540417; doi:10.1371/journal.pone.0182306)
Supplement: S4 Table — (PDF) [file pone.0182306.s004.pdf]

S4 Table. Likelihood ratio tests for positive selection using branch-site models

Gene	Models Compared	2ΔlnL	df	*p* value
MRPS5	Branch-site #1 (Mammals)	14.8240	1	$p<0.001$
	Branch-site #2 (Birds)	8.9825	1	$p=0.0027$
	Branch-site #3 (Birds/Reptiles)	3.9416	1	$p=0.047$
	Branch-site #4 (Amphibian)	12.7362	1	$p<0.001$
	Branch-site #5 (Fish)	10.0984	1	$p=0.0015$
FOXO3a	Branch-site #1 (Mammals)	7.0982	1	$p=0.008$
	Branch-site #2 (Birds)	6.0542	1	$p=0.014$
	Branch-site #3 (Birds/Reptiles)	6.2140	1	$p= 0.013$
	Branch-site #4 (Amphibian)	21.9256	1	$p<0.001$
	Branch-site #5 (Fish)	15.2409	1	$p<0.001$
PARP1	Branch-site #1 (Mammals)	7.2486	1	$p=0.007$
	Branch-site #2 (Birds)	4.8149	1	$p=0.03$
	Branch-site #3 (Birds/Reptiles)	5.8271	1	$p= 0.016$
	Branch-site #4 (Amphibian)	21.5609	1	$p<0.001$
	Branch-site #5 (Fish)	24.4764	1	$p<0.001$
PARP2	Branch-site #1 (Mammals)	11.3767	1	$p<0.001$
	Branch-site (Reptiles)	8.8035	1	$p=0.003$
	Branch-site #4 (Amphibian)	21.9734	1	$p<0.001$
	Branch-site #5 (Fish)	37.4631	1	$p<0.001$
PPARGC1A	Branch-site #1 (Mammals)	6.1631	1	*p=0.013*
	Branch-site #2 (Birds)	3.4102	1	*p=0.06*
	Branch-site #3 (Birds/Reptiles)	6.7810	1	*p=0.009*
	Branch-site #4 (Amphibian)	6.6429	1	*p=0.01*
	Branch-site #5 (Fish)	5.0601	1	*p=0.024*
SIRT1	Branch-site #1 (Mammals)	4.0928	1	$p=0.04$
	Branch-site #2 (Birds)	4.7832	1	$p=0.03$
	Branch-site #3 (Birds/Reptiles)	5.0012	1	$p=0.025$
	Branch-site #4 (Amphibian)	9.0935	1	$p=0.003$
	Branch-site #5 (Fish)	5.1349	1	$p=0.023$
SIRT2	Branch-site #1 (Mammals)	7.0923	1	$p=0.008$
	Branch-site #2 (Birds)	6.9963	1	$p=0.008$
	Branch-site #3 (Birds/Reptiles)	15.1935	1	$p<0.001$
	Branch-site #4 (Amphibian)	4.0173	1	$p=0.045$
	Branch-site #5 (Fish)	28.0972	1	$p<0.001$
SOD3	Branch-site #1 (Mammals)	6.0325	1	$p=0.014$
	Branch-site #2 (Birds)	6.4685	1	$p=0.011$
	Branch-site #3 (Birds/Reptiles)	8.0995	1	$p=0.004$
	Branch-site #4 (Amphibian)	9.1009	1	$p=0.002$
	Branch-site #5 (Fish)	12.1989	1	$p<0.001$
TP53	Branch-site #1 (Mammals)	8.9173	1	$p=0.003$
	Branch-site #2 (Birds)	3.8893	1	$p=0.048$
	Branch-site #3 (Birds/Reptiles)	5.0916	1	$p=0.024$

	Branch-site #4 (Amphibian)	10.0924	1	$p=0.001$
	Branch-site #5 (Fish)	20.1835	1	$p<0.001$
SIRT6	Branch-site #1 (Mammals)	9.4742	1	$p=0.002$
	Branch-site #2 (Birds)	8.9704	1	$p=0.003$
	Branch-site #3 (Birds/Reptiles)	7.0236	1	$p=0.008$
	Branch-site #4 (Amphibian)	8.6379	1	$p=0.003$
	Branch-site #5 (Fish)	19.0618	1	$p<0.001$

Note: The p value adjusted by Bonferroni correction for multiple testing is 0.001.
